# Genome survey sequencing and characterization of simple sequence repeat (SSR) markers in *Platostoma palustre* (Blume) A.J.Paton (Chinese mesona)

**DOI:** 10.1038/s41598-021-04264-x

**Published:** 2022-01-10

**Authors:** Zhao Zheng, Nannan Zhang, Zhenghui Huang, Qiaoying Zeng, Yonghong Huang, Yongwen Qi

**Affiliations:** 1 Guangdong Sugarcane Genetic Improvement Engineering Center, Institute of Bioengineering, Guangdong Academy of Sciences, Guangzhou, 510316 China; 2 Zhongkai University of Agriculture and Engineering, Guangzhou, 510225 China

**Keywords:** Genetic markers, Natural variation in plants, Plant genetics

## Abstract

*Platostoma palustre* (Blume) A.J.Paton is an annual herbaceous persistent plant of the Labiatae family. However, there is a lack of genomic data for this plant, which severely restricts its genetic improvement. In this study, we performed genome survey sequencing of *P. palustre* and developed simple sequence repeat (SSR) markers based on the resulting sequence. K-mer analysis revealed that the assembled genome size was approximately 1.21 Gb. A total of 15,498 SSR motifs were identified and characterized in this study; among them, dinucleotide, and hexanucleotide repeats had the highest and lowest, respectively. Among the dinucleotide repeat motifs, AT/TA repeat motifs were the most abundant, and GC/CG repeat motifs were rather rare, accounting for 44.28% and 0.63%, respectively. Genetic similarity coefficient analysis by the UPMGA methods clustered 12 clones, of *P. palustre* and related species into two subgroups. These results provide helpful information for further research on *P. palustre* resources and variety improvements.

## Introduction

*Platostoma palustre* (Blume) A.J.Paton, also known as Chinese mesona, is an annual herbaceous persistent plant of the Labiatae (Lamiaceae) family^1^. In China, *P. palustre* is mainly distributed in Taiwan, Zhejiang, Jiangxi, Guangdong, Fujian, and Guangxi provinces^2^. As a traditional Chinese edible and medicinal plant, it contains polysaccharides^3^, triterpenoid acids^4,5^, flavonoids^6^, phenolic compounds (such as epicatechins^7^ and caffeic acid^8^), and trace elements^8^. Wang et al.^9^ isolated five new caffeic acid oligomers, as well as four known analogues, and one compound showed significant in vitro antiviral activity against respiratory syncytial virus. A study by Song et al*.*^10^ showed that an extract of *P. palustre* had antioxidant and α-glucosidase inhibitory activities.*P. palustre* is widely used as a raw material for herbal tea, Guiling paste, and Chinese medicine. The caffeic acid extracted from *P. palustre* was proven to have antioxidative activity^11^. Moreover, it was also reported that *P. palustre* polysaccharide (MP) treatment can increase the immunomodulatory activity of mice^12^. Water and alcohol extracts of *P. palustre* were reported to be effective in ameliorating hypertension^13^ and hyperglycaemia^14^ in rats and can inhibit the growth of *Escherichia coli* and *Salmonella*^15,16^.

To date, research on *P. palustre* has mainly focused on component extraction, activity, and development for food, and few studies on the genetic diversity of germplasm resources have been reported because of the limited genetic and genomic resources for this species. The concentrations of polysaccharides, triterpenoid acid, flavonoids, and other compounds of different *P. palustre* varieties vary widely, which directly affects their palatability and use in production^17^. Hence, variety identification is very important for *P. palustre*.

For the identification of *P. palustre,* morphological features such as leaf colour, tillering number, and flowering time have been employed, but this method relies on the accumulated experience of the appraiser, which is vulnerable to environmental and subjective factors and is time-consuming, laborious, and inaccurate. Therefore, it is very important to establish a set of rapid, accurate, and economical identification technologies to promote the utilization of *P. palustre*. Simple sequence repeat (SSR) marker are a powerful and cost-effective molecular method for quantifying genetic variation in plants due to their abundance in genome, polymorphism, co-dominance and high reproducibility^18^, and have been developed for many plant species^19^, especially those used in traditional Chinese herbal medicine, such as watermelon^20^, *Psidium*^21^, *Bupleurum falcatum*^22^, and *Ligusticum chuanxiong*^23^. These SSR markers have been broadly applied for genetic purity detection (for identifying off-types and selfed females in many hybrid seeds)^20^, species identification^21^, haplotype determination, quantitative trait locus (QTL) discovery, marker-assisted selection (MAS) for desired traits and breeding, cultivar DNA fingerprinting, genome-wide association studies (GWASs), and harnessing heterosis^19,24^. It is almost certain that, when developed, SSR markers can be used for *P. palustre* identification and accelerating breeding. There have been a few studies on SSR marker development for Chinese herbal medicines; however, few studies on *P. palustre* molecular markers have been reported. Thus, it is urgent to develop SSR markers for *P. palustre*.

With advances in next-generation sequencing (NGS) technology, genome survey sequencing has proven to be an important and cost-effective strategy for exploring genomic information and developing molecular markers for plants^25^, especially for non-model plants which no genetic information is known^19^. In this study, genome survey sequencing was employed to investigate the genome of *P. palustre*. We first mined SSRs from genome survey sequences of *P. palustre* and validated 90 SSRs to understand the genetic relationships among six *P. palustre* varieties and six other Labiatae species. We aimed to provide a reference for the genotyping, breeding, germplasm collection, and management of *P. palustre*.

## Results

### Genome sequencing and estimation of genome size

**Figure 1:**
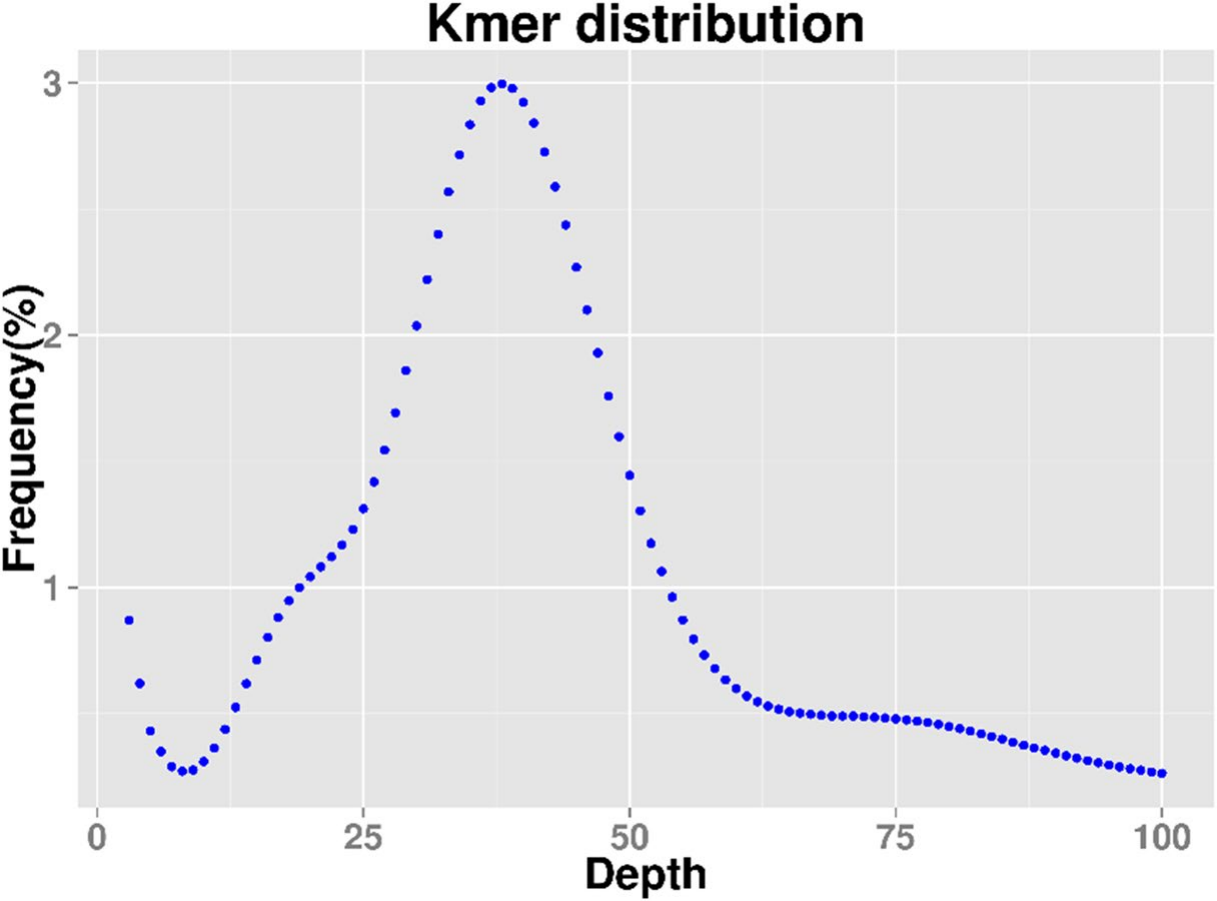
Estimation of *P. palustre* genome size using k-mer (k = 19) analysis.

Paired-end sequencing with 270-bp short inserts of *P. palustre* was conducted using genomic DNA from sample MX 1. A total of 54.99 Gb of raw data was generated by the Illumina HiSeq sequencing platform, which was approximately 45.37-fold the estimated genome size. All reads were used for k-mer analysis, and abnormal k-mers were removed to calculate genome size, the repeat rate, and heterozygosity. We used 270-bp library data to construct a k-mer distribution map with k = 19 (Fig. 1). For the 19-mer frequency distribution, the peak of the depth distribution was approximately 38. The sequence at the k-mer depth was more than twice the depth at the main peak, which can be attributed to the repeated k-mer sequence with a depth of more than 76. Moreover, a k-mer depth at half the main peak (near 19) represents heterozygosity.

The sequencing data yielded total k-mer values of 48,380,469 and 234. When k-mers with depth abnormalities were removed, the remaining k-mer values were found to be 46,608,868 and 033. These values were further used for the estimation of gene leaders. The genome size was estimated to be 1.21 Gbp, using the following formula: genome size = k-mer count/peak of the k-mer distribution. To ensure the accuracy of the genome size prediction, GenomeScope2 and findGSE software with different k-mer sizes (k = 21, 23, 25, and 27) as well as MGSE were used for genome size prediction. The genome sizes predicted by the different tools with different parameters were in the range of 1.3 Gb to 1.4 Gb (Supplementary Table 1). Based on the k-mer distribution, almost 70.62% of the sequence was repeated. The peak heterozygosity was as low as 0.33%; thus, there was no obvious heterozygosity. The results suggest that the genome of *P. palustre* is highly complex and has a high degree of repetition.

The resequencing data was de novo assembled by SOAP denovo software. A total of 6,968,859 raw contigs were observed. Unique contigs for scaffold generation were obtained after blasting reads and contigs. Gaps resulting from sequence repetition were filled with paired-end reads. Consequently, the genome was assembled in the form of a total of 5,822,179 scaffolds with a length of 1,374,372,218 bp. We obtain totally 401,762,775 raw reads. Among them, 393,971,228 (98.06%) reads were properly mapped against the assembled sequence by Bwa mem software. Among the scaffolds, scaffold N50 was found to be 191 bp in length and L50 was 1,359,845 from the SOAP denovo software, as shown in Table 1. The raw sequencing data have been submitted to the NCBI database (accession number: PRJNA706453). As the N50 value was very low in contigs as well as scaffolds, we also performed assembly with another software programme, SPAdes. The results showed that contig N50 was 193 bp, which was consistent with results from SOAP. The low quality of the assembled sequence might have been due to the complexity of the *P. palustre* genome.

**Table 1:** Contigs and scaffolds of *P. palustre*.

ID	Features	Sequencing reads	Value (SOAP)	Value (SPADes)
1	Total number of bases (Gb)	63.19	–	–
2	Clean reads (Gb)	54.99	–	–
3	Clean reads proportion (%)	87.02%	–	–
4	Q20%	99.43	–	–
5	Q30%	95.31	–	–
6	GC content (%)	40.19%	–	–
7	Total number of contigs	–	6,968,859	6,783,951
8	Assembly length (bp)	–	1,374,372,218	1,335,937,505
9	Largest contig size (bp)	–	61,900	62,170
10	L50	–	1,359,845	1,310,596
11	N50 (bp)	–	191	193
12	Number of SSR identified through MISA	–	15,498	–
13	Number of SSRs tested	–	90	–
14	Number of polymorphic SSR markers	–	37	–

### Identification and characterisation of SSR motifs

**Figure 2:**
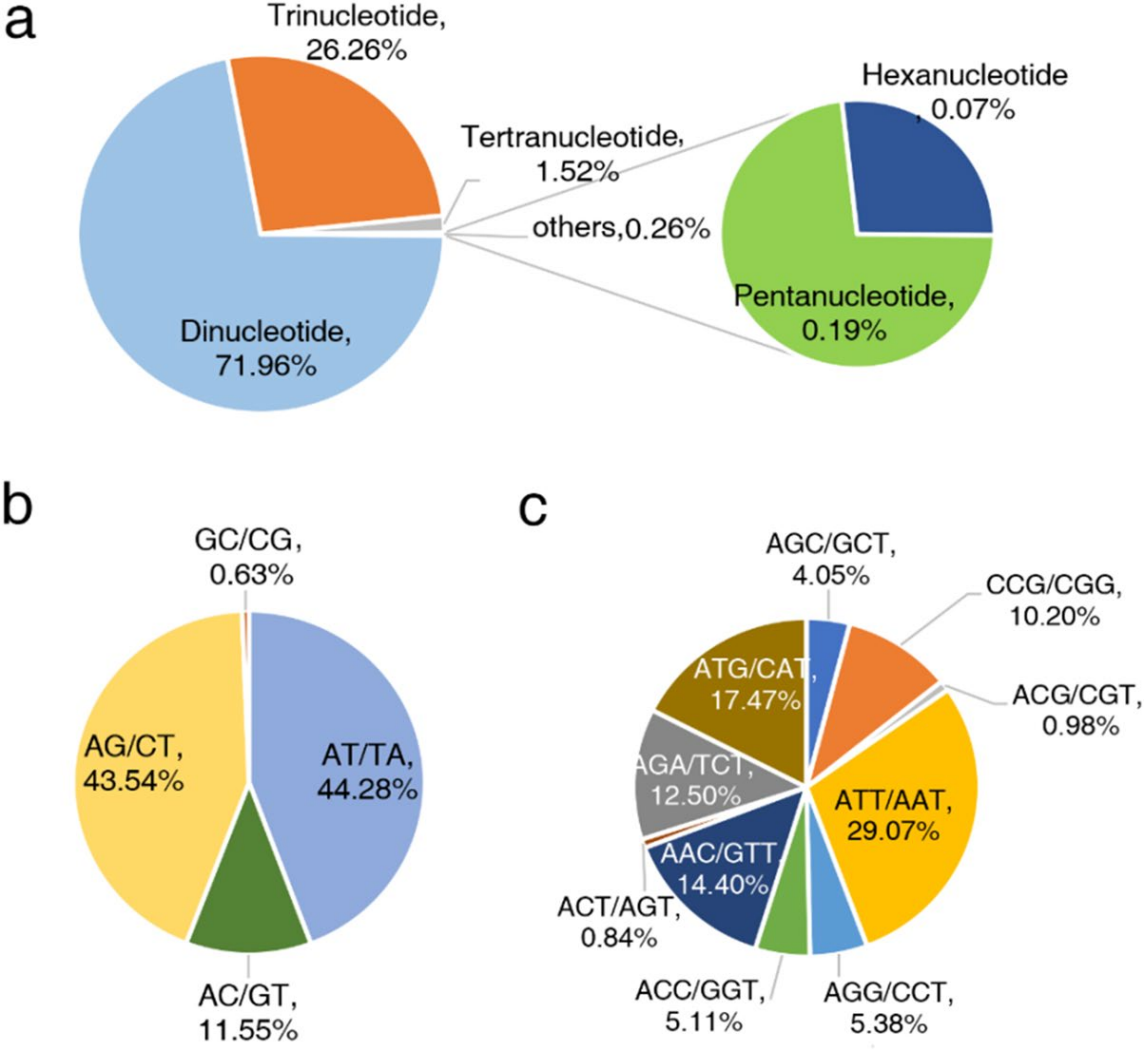
Identification and characteristics of SSR motifs. (**a**) Frequency of different SSR motif types. (**b**) Frequency of different dinucleotide SSRs motifs. (**c**) Frequency of different trinucleotide SSR motifs.

In total, 15,498 SSRs were identified from the *P. palustre* genome survey results (Supplementary Table 2). The identified SSR motifs included dinucleotide (71.96%), trinucleotide (26.26%), tetranucleotide (1.52%), pentanucleotide (0.19%), and hexanucleotide (0.07%) repeats, as shown in Fig. 2a. AT/TA was the most abundant type of dinucleotide repeat, with a content of 44.28% (4939 of all dinucleotide repeats). The AG/CT content was 43.54% (4856 repeats), and the very rare type GC/CG accounted for only 0.63% (70 repeats) (Fig. 2b). In the case of trinucleotides, the most abundant type was ATT/AAT (1183, 29.07% of all trinucleotide repeats), followed by ATG/CAT (17.47%, 711 repeats) and AAC/GTT (14.40%, 586 repeats) (Fig. 2c). Among the tetra-, penta-, and hexanucleotide repeats, the most abundant type was TTTA/TAAA (48.94%, 115 repeats). Furthermore, 99.99% di-, 99.92% tri-, 100% tetra-, 79.31% penta- and 90.00% hexanucleotide repeats were shorter than 30 bp.

**Figure 3:**
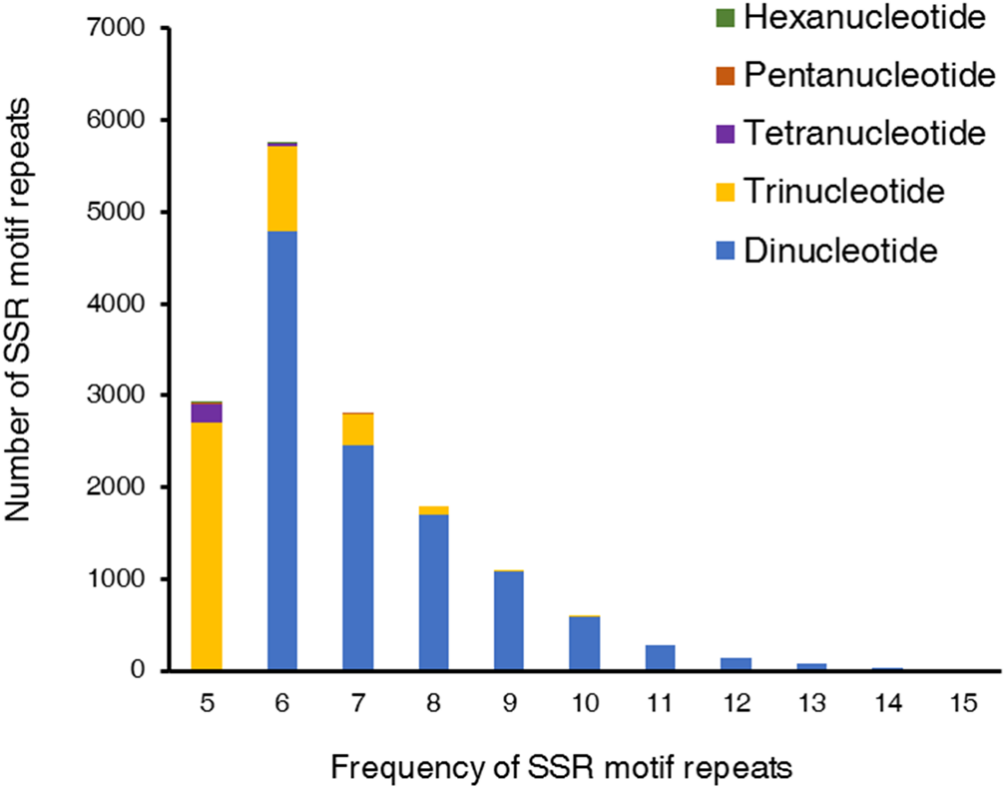
Distribution and frequency of SSR motif repeat numbers. The X-axis shows the frequencies of SSR types, while the Y-axis shows SSR repeat numbers.

SSR motif analysis of *P. palustre* revealed repeat frequencies of 6–15, 5–10, and 5–6 for dinucleotide, trinucleotide, and hexanucleotide repeats, respectively. The repeat frequencies for both tetra- and pentanucleotides were in the range of 5–7, as shown in Fig. 3. The results further revealed the highest frequency for motifs with 6 tandem repeats (37.11%, 5751), followed by motifs with 5 tandem repeats (18.93%, 2934), 7 tandem repeats (18.07%, 2801), and 8 tandem repeats (11.60%, 1789).

### SSR marker verification

A subset of 90 SSR markers representing each repeat class were randomly selected for their validation through PCR amplification (Supplementary Table 3). Among the selected markers, the di-, tri-, tetra-, penta-, and hexanucleotide repeat classes, were represented by 8, 6, 37, 29, and 10 SSR primer pairs, respectively. The results showed that 79 SSRs (87.78%) were successfully amplified, and 37 of these (46.83%) demonstrated polymorphic banding pattern.

**Figure 4:**
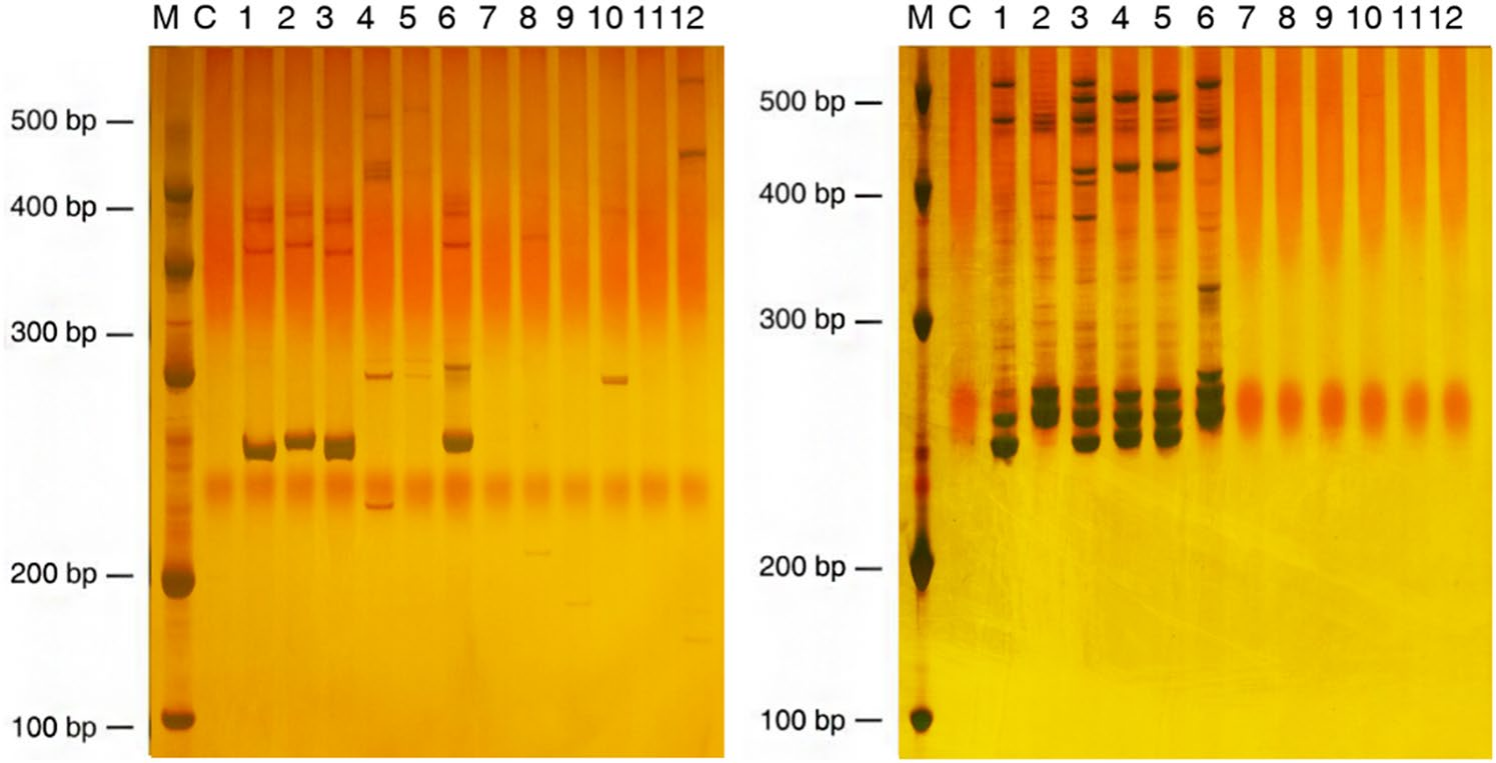
PCR-amplified products of markers McSSR_67 (left) and McSSR_78 (right) for six accessions of *P. palustre* and related species resolved on 7% PAGE. Lanes: M, 500 bp DNA marker; C, control; 1, *P. palustre* MX 1; 2, *P. palustre* TW 1; 3, *P. palustre* ZC 1; 4, *P. palustre* ZC 2; 5, *P. palustre* XU 1; 6, *P. palustre* XU 2; 7, *M. haplocalyx*; 8, *M. spicata*; 9, *P. vulgaris*; 10, *S. miltiorrhiza*; 11, *S. indica*; 12, *S. barbata*.

**Figure 5:**
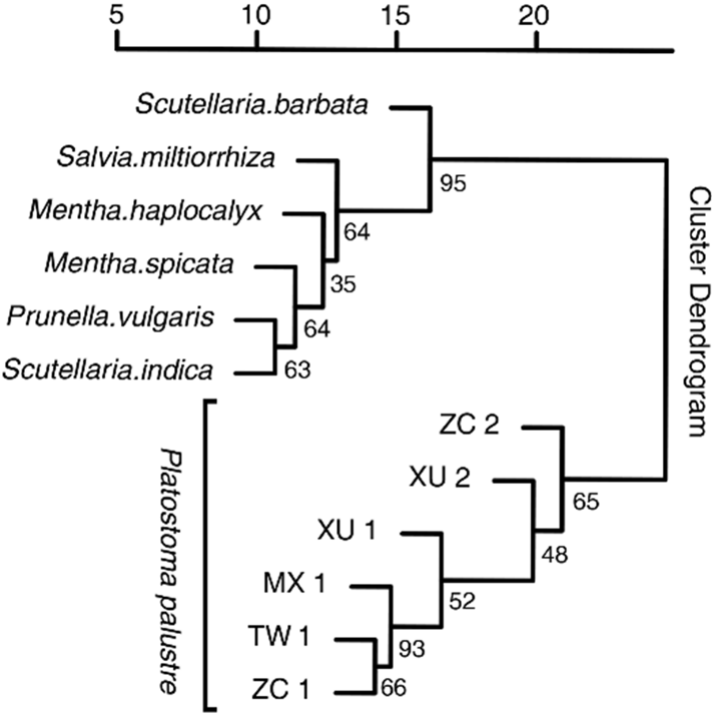
Genetic similarity coefficient analysis of six accessions of *P. palustre* and six related species performed based on the neighbor-joining method using the pvclust R package. Numbers at the nodes are bootstrap values from 1000 replicates.

The thirty-seven SSR markers were further investigated among *P. palustre* and related Labiatae genera including *Mentha haplocalyx, M. spicata, Prunella vulgaris, Salvia miltiorrhiza, Scutellaria indica*, and *S. barbata* (Supplementary Table 4). A total of 685 fragments were generated through the PCR amplification of the 12 accessions with a mean of 18.5 alleles per marker loci (Fig. 4 and Supplementary Table 4; the full-length gels are presented in Supplementary Fig. 1). Among the tested SSRs, 10 were specifically amplified in *P. palustre* while the remaining 27 showed a varied level of cross transferability in other related taxa. According to the clustering analysis performed with 27 SSR markers (Fig. 5), 12 accessions were divided into two groups. The 6 *P. palustre* accessions were clustered into one group, and the 6 accessions of related Labiatae genera were clustered into another group.

## Discussion

*P. palustre* is an important traditional Chinese medicine and edible plant resource with heat-clearing and detoxifying functions. The leaves, roots, and stems of *P. palustre* have been widely found to contain gel mainly consisting of cortex phellodendri, benzoic acid, ursolic acid, organic acids, flavones, and catechins^3–5^. Because food and medicinal products of *P. palustre* have different requirements in terms of quality, it is necessary to breed varieties with different characteristics through genetic improvement. In addition, adulterant plants are common in *P. palustre* collections. Thus, establishing an accurate and rapid method by molecular markers to identify *P. palustre* and related species is important for the genetic identification and improvement of *P. palustre*. Shi et al*.*^2^ analysed *P. palustre* and its adulterants using the internal transcribed spacer 2 (ITS2) region and found that the ITS2 region, as a DNA barcode, could accurately and effectively distinguish *P. palustre* from its adulterants, including *Isodon serra* Maxim. However, the study showed that there was no difference in the ITS2 region among the 26 *P. palustre* accessions from Guangxi province, Guangdong province, Jiangxi province, Fujian province, and Hainan province in China. The results showed that the ITS2 region is not suitable for identifying *P. palustre* cultivars. Therefore, it is necessary to develop alternativemolecular markers for genetic resource evaluation and improvement.

A genome survey of *P. palustre* was applied for the first time in this study, with the aim of identifying markers for *P. palustre* and understanding the genetic diversity and relationships among cultivars and related species. According to the k-mer analysis of the genome survey sequences, the genome of *P. palustre* is approximately 1.21 Gbp and is complex with a low level of heterozygosity (0.33%). The genome of *P. palustre* is smaller than that of its related species; for instance, the genome of *S. miltiorrhiza* is 8.19 Gbp^26^. However, it is much larger than that of other dicotyledons, such as buckwheat (497 Mb)^27^, shantung maple (529 Mb)^28^ and jute (338 Mb)^29^. In plants, there is a positive correlation between genome size and repetitive elements^30^. For example, the repetitive element content of *P. palustre* is 70.62%, which is higher than that of shantung maple (529 Mb, 48.8%) and lower than that of *Radix bupleuri* (2.11 Gb, 83.89%)^31^.

A draft reference de novo assembly with sequencing data was used to explore SSRs. A total of 54.99 Gb of clean reads were generated and de novo assembled into 6,968,859 contigs. Due to the complex genome of *P. palustre*, the contig N50 value was lower. SSRs with high polymorphism and codominance have been used to evaluate genetic resources and in a variety of improvement programs^32,33^. In this study, a total of 15,498 SSRs were identified in *P. palustre* using genome survey sequencing. Morgante et al.claimed that there was a negative correlation between genome size and SSR distribution frequency^34^. However, the SSR distribution frequency in this genome survey was estimated to be 12.80 SSRs per Mb, which is lower than that in *R. bupleuri* (43.11 SSR per Mb)^31^ and buckwheat (49.30 SSR per Mb)^27^. Obviously, *P. palustre* did not follow this rule. The di- and trinucleotide repeats accounted for the majority of the SSRs, while tetra-, penta-, and hexanucleotide repeats accounted for a very small proportion. Similarly, among the five tandem repeat types of SSRs in *P. palustre*, di- and trinucleotide repeats accounted for 98.22% of the total SSRs, while tetra-, penta-, and hexanucleotide repeat SSRs accounted for only 1.52%. In *P. palustre*, we found that AT/TA (44.28%) and ATT/AAT (29.07%) were frequent among the di- and trinucleotide repeat SSRs; these percentages are different not only from those in sorghum^35^ (AT/AT, 54.4% and CCG/CGG, 18.1%), rice^33^ (AG/CT, 41.9% and CCG/CGG, 47.5%), and buckwheat^27^ (AT/AT, 78.60% and AAT/TTA, 31.83%) but also from those in the majority of grasses (GA/TC dimers, A/T monomers, and GCG/CGC trimers were the most abundant SSR types), with some exceptions^34^. Interestingly, in a study of 16 tree species, a similar trend was observed, where AT/TA base pairs were found to be the most prevalent dimers, followed by AG/TC. AAT/TTA were the most frequent trimers^36^. In summary, SSR types have different distribution patterns among species at a large evolutionary scale^37,38^, but the distribution patterns of closely related species and even different parts of the same species differ^39,40^. The reason for the high polymorphism at these loci needs much more exploration.

As high variability in repeat unit number is observed, SSRs are highly polymorphic and are suitable for use as specific markers for different species/genera and germplasm characterization. In this study, we identified 64 SSRs with polymorphisms among the *P. palustre* accessions. By using 37 of the 64 SSRs, 395 specific fragments of *P. palustre*, accounting for 58.96% of all fragments, were detected. The results showed that there was significant genetic differentiation between *P. palustre* and related Labiatae species. The high polymorphism and specificity of the SSR markers developed in this research suggest that these SSRs could be further used in genetic linkage mapping, MAS, and the identification of genuine hybrids between cultivated *P. palustre* varieties and the other 6 related Labiatae genera.

This study revealed genomic information for *P. palustre* and unique SSR loci, providing valuable information for follow-up studies on cultivar identification, improvement and genetic resource management. However, because of the current absence of a reference genome sequence for this species, the genome location/genome coverage of these SSRs makers is unknown. In future, with more genome information for *P. palustre* is revealed, more molecular makers could be developed and accelerate genetic improvement of *P. palustre*.

## Methods

### Plant materials

The plant materials comprised six *P. palustre* accessions (MX 1, TW 1, ZC 1, ZC 2, XU 1, and XU 2) and six accessions of related Labiatae species, including *M. haplocalyx*, *M. spicata*, *P. vulgaris*, *S. miltiorrhiza*, *S. indica*, and *S. barbata*. Of the six *P. palustre* accessions, M X 1 and TW 1 was from Fujian province, China. While ZC 1, ZC 2, XU 1 and XU 2 were from Guangdong province, China.

### Library construction, genome sequencing and genome character estimation

Total genomic DNA was isolated from young leaf tissue of all plants following a modified CTAB procedure^41^, and the quality was evaluated by 1% agarose gel electrophoresis. The concentrations of DNA were checked by a BioPhotometer (Eppendorf, Germany). The most widely planted *P. palustre* MX 1, was selected for the genome survey.

The genomic DNA was broken into fragments of approximately 270 bp by the ultrasonic vibration. The small-insert fragment library was constructed from fragmented random genomic DNA following the manufacturer’s instructions (NEBNext® Ultra DNA Library Prep Kit for Illumina). Adapter ligation and DNA cluster preparation were performed, followed by sequencing using an Illumina Genome Analyzer (Illumina HiSeq 2000, USA) according to the manufacturer’s standard protocol.

In total, four paired-end sequencing libraries with insert sizes of approximately 270 bp were constructed, and paired-ends of 150 bp were sequenced using the Illumina HiSeq 2100 platform. The quality control and pre-processing of sequencing raw reads were carried out using the fastp software^42^. 284, Raw reads were filtered by Trimmomatic software (v0.39; http://www.usadellab.org/cms/?page=trimmomatic) to remove low quality reads and adaptor sequences. GC distribution analysis was performed by in-house perl code After filtering, clean reads were obtained and used for the following analyses. K-mer (k = 19) analysis was performed, and the abnormal k-mers were filtered out for subsequent analysis. The rate of heterozygosity and the repeat rate were estimated according to k-mer analysis^43^. GenomeScope2^44^ and findGSE^45^ with different k-mer sizes (k = 21, 23, 25, and 27) as well as MGSE software^46^ were employed to predict genome size. The genome size was estimated with the formula: Genome_Size = K-mer coverage/Mean k-mer depth^47^.

### Genome assembly and SSR marker development

After removing the adapters, raw sequencing data were further cleaned for downstream analysis by filtering out reads containing low-quality bases, reads < 100 bp in length, and duplicated reads. The clean reads of all the libraries were assembled into scaffolds and contigs using SOAPdenovo v2 (http://soap.genomics.org.cn/soapdenovo.html) software. SSRs in the DNA sequences were identified using MIcro-SAtellite (MISA) software (version 1.0)^48^. SSR identification was based on two parameters. First, SSR minimum numbers of 6, 5, 5, 5, and 5 were adopted for the identification of di-, tri-, tetra-, penta-, and hexanucleotides, respectively. Second, an interruption of less than 100 bp between two SSRs was defined as a compound repeat of SSR. Primer Premier V5.0 software (Premier Biosoft International, Palo Alto, CA) was used for primer design with the following parameters: 100–300 bp for final product length, 18–25 bp for primer size (with an optimum size of 20 nucleotides), 35–70% for GC content, and 55–65 °C for annealing temperature.

### Verification of SSR markers and genetic similarity analysis

A total of six accessions of *P. palustre* and six related species, including *M. haplocalyx*, *M. spicata*, *P. vulgaris*, *S. miltiorrhiza*, *S. indica*, and *S. barbata*, were used for the verification of SSR markers developed by genome survey sequencing. In total, 90 SSR markers were selected to verify the quality of SSR markers and polymorphisms in the six accessions of *P. palustre*. Thirty-seven SSR markers were used to analyse the genetic similarity among the 12 accessions of *P. palustre* and related species. PCR was performed using *EasyTaq*® DNA Polymerase (TransGen Biotech, China) with the following programme: 94 °C for 5 min (initial denaturation) followed by 35 cycles of 94 °C for 30 s, 58–61 °C for 30 s, and 72 °C for 1 min, with an extension of 72 °C for 10 min and hold at 4 °C. The products obtained from the PCR were analyzed with 7% polyacrylamide gel electrophoresis (PAGE) and detected by staining with AgNO_3_ solution. Clear and strong allelic fragments in the same horizontal position were scored manually as 0 (absent) or 1 (present), and the number of alleles (Na), effective number of alleles (Ne), percentage of polymorphic loci (PIC) and expected heterozygosity were calculated using GenAlEx 6.5^49,50^. The genetic similarity coefficients of these clones were calculated and cluster analysis was performed based the neighbor-joining method using the pvclust R package^51^.

## Supplementary Information

Supplementary Information.
