# Supplementary material for: Genome survey sequencing and characterization of simple sequence repeat (SSR) markers in Platostoma palustre (Blume) A.J.Paton (Chinese mesona)
Source: Sci Rep. 2022 Jan 10;12:355. doi: 10.1038/s41598-021-04264-x (PMC8748427; doi:10.1038/s41598-021-04264-x)
Supplement: Supplementary file 1 — Supplementary Information. [file 41598_2021_4264_MOESM1_ESM.pdf]

Genome Survey Sequence and Characterization of Simple Sequence Repeat (SSR) Markers in *Mesona chinensis* Benth (Chinese Mesona)

Zhao Zheng [1,2,#], Nannan Zhang [1,#], Zhenghui Huang [1,2], Qiaoying Zeng [1], Yonghong Huang [1] and Yongwen Qi [1,2,*]

[1] Guangdong sugarcane Genetic Improvement Engineering Center，Guangdong Provincial Bioengineering Institute (Guangzhou Sugarcane Industry Research Institute), Guangzhou 510316, China；

[2] Zhongkai University of Agriculture and Engineering, Guangzhou 510225, China

* Correspondence: yongwen2001@163.com; Tel.: +86 20 84168434 (F.L.)

[#] These authors contributed equally to this work

Authors list

Zhao Zheng

Email: 13710053962@163.com
Affiliation:
Guangdong Sugarcane Genetic Improvement Engineering Center，Guangdong Provincial Bioengineering Institute (Guangzhou Sugarcane Industry Research Institute), Guangzhou 510316, China；
Zhongkai University of Agriculture and Engineering, Guangzhou 510225, China

Nannan Zhang

Email: zhangnn23@mail2.sysu.edu.cn
Affiliation:
Guangdong Sugarcane Genetic Improvement Engineering Center，Guangdong Provincial Bioengineering Institute (Guangzhou Sugarcane Industry Research Institute), Guangzhou 510316, China；

Zhenghui Huang

Email: hzh85501@foxmail.com
Affiliation:
Guangdong Sugarcane Genetic Improvement Engineering Center，Guangdong Provincial Bioengineering Institute (Guangzhou Sugarcane Industry Research Institute), Guangzhou 510316, China；
Zhongkai University of Agriculture and Engineering, Guangzhou 510225, China

Qiaoying Zeng

Email: zengqiaoying123@163.com
Affiliation:
Guangdong Sugarcane Genetic Improvement Engineering Center，Guangdong Provincial Bioengineering Institute (Guangzhou Sugarcane Industry Research Institute), Guangzhou 510316, China；

Yonghong Huang

Email: bear20081013@126.com

Affiliation:
Guangdong Sugarcane Genetic Improvement Engineering Center, Guangdong Provincial Bioengineering Institute (Guangzhou Sugarcane Industry Research Institute), Guangzhou 510316, China；

Yongwen Qi

Email: yongwen2001@163.com
Affiliation:
Guangdong Sugarcane Genetic Improvement Engineering Center, Guangdong Provincial Bioengineering Institute (Guangzhou Sugarcane Industry Research Institute), Guangzhou 510316, China；
Zhongkai University of Agriculture and Engineering, Guangzhou 510225, China

Supplementary Table 1. The predict genome size of *Mesona chinensis* Benth with different tools.

Software	Kmer size	Genome size (bp)
GenomeScope2	21	1258798638
GenomeScope2	23	1258743939
GenomeScope2	25	1258153982
GenomeScope2	27	1257443508
FindGSE	21	1387232711
FindGSE	23	1406061078
FindGSE	25	1384082434
FindGSE	27	1403375561
MGSE	-	1346321495
Gce	21	2196260000
Gce	23	2180150000
Gce	25	2128670000
Gce	27	2219320000

Supplementary Table 2. Details of all the SSR markers that identified from *Mesona chinensis* Benth

ID	SSR nr.	SSR type	SSR	size	start	end	FORWARD PRIMER1 (5'-3')
scaffold61_15.9	1	p2	(AT)9	18	1483	1500	AACGAATTGAAATGAATTCTACG
scaffold84_14.4	1	p2	(TA)9	18	50	67	CGAACGCTCCAAATTAGAATG
scaffold124_12.6	1	p2	(AT)7	14	277	290	GGCTATAGGTCAACGGACCA
scaffold127_13.5	1	p2	(TA)9	18	132	149	CTGAGCTTCAGAGCCCAGTT
scaffold219_12.9	1	p2	(TA)8	16	644	659	TGAATTCATAACCTTCCCATTCA
scaffold343_12.1	1	p2	(CT)11	22	423	444	GCTCCACCTCCAAACAAGAG
scaffold367_15.0	1	p2	(AT)6	12	15141	15152	CTGGAGCAGCATCCAAAGTT
scaffold407_11.5	1	p2	(AT)9	18	47	64	TGTGTTGTGTGGAATAAAAGTTTG
scaffold441_16.0	1	p2	(CT)6	12	694	705	TCTTCGTTTCTGGTCTGCCT
scaffold452_15.0	1	p2	(AT)10	20	48	67	TGCAGCAATGGTTGTGATTT
scaffold509_17.8	1	p2	(AT)6	12	183	194	CGAGACACTTGGCAACTTCA
scaffold549_11.1	1	p2	(AC)6	12	65	76	CCCTCGACACACACTCACAT
scaffold642_16.5	1	p2	(TC)6	12	301	312	TAAATCATCAGCCCACGTCA
scaffold820_12.7	1	p2	(AT)9	18	375	392	CAGTAATCATGCGCGAAAAA
scaffold851_10.9	1	p2	(AG)6	12	302	313	TGACCGGAAGGTATGAACAA
scaffold900_11.1	1	p2	(TA)7	14	523	536	TCGCTGACATCATCTTATGGA
scaffold909_11.1	1	p2	(GA)6	12	532	543	CAAGGGTTCAAGCATTGGAG
scaffold914_14.0	1	p2	(GA)7	14	586	599	AAGCATTTGAGGGGTTTTCC
scaffold992_13.1	1	p2	(GC)6	12	61	72	CGGGGACACATAAACCTCAC
scaffold1174_17.5	1	p2	(CT)6	12	54	65	TTCCCCTTTGCCCTCTATTT
scaffold1268_20.1	1	p2	(GA)7	14	75	88	CCCACTGATTTGTGTGAGGA
scaffold1316_13.6	1	p2	(AG)8	16	52	67	AGTTTTCATGGATGGATGCC
scaffold1359_13.2	1	p2	(AT)7	14	694	707	TCCAAGTTTTTACCCCACCA
scaffold1398_10.7	1	p2	(TA)6	12	54	65	TGACCATATCTCGGTTTAGCA
scaffold1420_11.1	1	p2	(TC)6	12	695	706	TTTTAATGGGCGGTTATGGA
scaffold1450_12.4	1	p2	(AG)7	14	263	276	GACATATCGTCGATCGGCTT
scaffold1683_20.2	1	p2	(AT)6	12	107	118	GGGTGGATGACTTTGGTGTT
scaffold1739_11.1	1	p2	(CT)6	12	44	55	TCTAACCCAAACCCAAACCC
scaffold2019_12.7	1	p2	(TA)6	12	94	105	TTTCGTCCCGAAATTTTGTT
scaffold2112_11.2	1	p2	(TA)9	18	209	226	GACAACCTTCGGCTTGAAAA
scaffold2138_14.4	1	p2	(CT)6	12	185	196	AACTTGGCCCCTAAGCTTGT
scaffold2197_15.4	1	p2	(TA)7	14	52	65	CCATCTATAGCGGTTAGCGTG
scaffold2272_15.8	1	p2	(TA)6	12	54	65	CCCACTTAGGAACATTTAGGGA
scaffold2610_13.8	1	p2	(AT)10	20	401	420	TTGATACCTCCTCCCACGAC
scaffold2693_15.1	1	p2	(AT)8	16	375	390	GTTAGCGGAGTGAATGAGGG
scaffold2696_12.8	1	p2	(CT)7	14	104	117	AATTGATCATGGCTTCTGGC
scaffold2789_10.8	1	p2	(TC)6	12	208	219	GGGAGTTTGACACCTAAAGCC
scaffold2800_12.0	1	p2	(AT)6	12	388	399	ACAGGCTCTTTGGAAGACGA
scaffold2816_18.4	1	p2	(TA)9	18	100	117	TGGTTATTACTGGAACGCGA
scaffold2829_15.3	1	p2	(CT)7	14	2222	2235	ACAGGTCCCTGAAACAAACG
scaffold2857_14.5	1	p2	(TC)6	12	2399	2410	ACGTGAAGGCTGGGACATAC
scaffold2889_11.2	1	p2	(AT)6	12	54	65	ATTTGACATCTAATACCCAACTATTTT
scaffold3055_12.9	1	p2	(CT)10	20	266	285	TTTATTGCAGGCGCTCTCTT
scaffold3064_11.0	1	p2	(TG)6	12	54	65	GGATACCCTCTCAAACCCTAGT
scaffold3164_15.8	1	p2	(TC)7	14	209	222	GGGGCGTGACAAGTTATGTT
scaffold3415_18.4	1	p2	(GA)7	14	1148	1161	ATCAAACAGCTGAGGCTCGT
scaffold3600_10.3	1	p2	(GA)6	12	106	117	TGATGAAGGTCCAAATGCAA
scaffold3641_11.1	1	p2	(AT)6	12	54	65	AATTCTAACCATCTTCTTTTGCC
scaffold3739_12.5	1	p2	(TA)7	14	238	251	ATCCTCCCACCCCACTTAAC
scaffold3746_15.6	1	p2	(GA)6	12	490	501	AGAAAAGGGGAGAGAGCAGG
scaffold3844_17.5	1	p2	(TC)6	12	139	150	GACTGTTCGAGCTTATCCGC
scaffold3968_15.2	1	p2	(AT)6	12	417	428	CACGTACTGGCCTAAATAATGTCA
scaffold4045_13.5	1	p2	(AT)10	20	818	837	GAGAGCATGAAGAATGAACCC
scaffold4135_23.5	1	p2	(AG)6	12	54	65	TGGTGAGATTTGTGGTTTGG
scaffold4211_12.1	1	p2	(AT)6	12	176	187	TCGGTCGAAACCGAATAAAC

scaffold4517_11.1	1	p2	(TA)8	16	485	500	ATTGGCAAAATATCACCGGA
scaffold4547_12.1	1	p2	(AT)6	12	55	66	CAATAATTTTGTTTCCCACCAA
scaffold4626_12.8	1	p2	(AC)7	14	236	249	AAGTCCACCACGATGTCCTC
scaffold4628_13.4	1	p2	(TA)9	18	1079	1096	TTTCACAAACCACGTTGACA
scaffold4661_18.0	1	p2	(CT)7	14	341	354	CCCTAACTCGACCCAGATCA
scaffold4751_14.3	1	p2	(TC)8	16	1454	1469	GCTGAGTTTTGAGGATTGGG
scaffold4790_16.1	1	p2	(AG)6	12	3258	3269	CGCTAAGATGGAGTTTTCGG
scaffold4808_11.4	1	p2	(TC)6	12	34	45	GGGATGCCATTCGTTAGTCC
scaffold5156_12.2	1	p2	(AT)7	14	1019	1032	TGGAAGATTAAGAGGCCCAA
scaffold5238_13.0	1	p2	(TC)6	12	48	59	CCCCTTTGTCAACTTAGGCA
scaffold5329_11.0	1	p2	(AT)6	12	587	598	ATTCCTAGGCGGCATTCTGT
scaffold5553_14.7	1	p2	(TG)10	20	302	321	CGGTGGAGTGGTTTCACAGT
scaffold5614_15.9	1	p2	(TA)8	16	1933	1948	CAGAGGATCCAAATCCGAAG
scaffold5636_13.9	1	p2	(AT)6	12	54	65	GCTACCTTATTGATCCATTCCTC
scaffold5978_13.4	1	p2	(AC)8	16	42	57	GCATGAAATTGTGTATTTTGGTG
scaffold6020_16.6	1	p2	(GA)8	16	2628	2643	TGCCATGCACAGTTTTTCTC
scaffold6052_11.1	1	p2	(CT)8	16	52	67	TCTCTCTTTTTGGTTGGTGGA
scaffold6064_14.9	1	p2	(TA)8	16	1004	1019	TGTGAATTGGGTATGTGAATCAT
scaffold6255_13.3	1	p2	(AT)7	14	756	769	TGCAATCTCAGCCACTCATT
scaffold6310_14.4	1	p2	(TG)7	14	4263	4276	TGGTCGTTGACACGACAGAT
scaffold6349_13.7	1	p2	(AT)7	14	466	479	TGCAAGAATGGGTTTTTGAAT
scaffold6381_11.9	1	p2	(AT)7	14	250	263	TTTCGTGTTGCCATTGATCT
scaffold6512_15.5	1	p2	(AG)9	18	231	248	GAGCACTCGAAAAGTAGCGG
scaffold6602_12.8	1	p2	(TA)6	12	95	106	CGAGGAGAACCTTGGAGTGA
scaffold6618_10.5	1	p2	(AG)6	12	54	65	TGAGTTTCAATTGCGTAGTCAAA
scaffold6619_15.4	1	p2	(AG)6	12	415	426	TCACAGACGGAGAGAGACACA
scaffold6738_16.2	1	p2	(AT)7	14	1345	1358	TCTCATTCTCCATCAGCCAA
scaffold6920_13.8	1	p2	(TA)6	12	156	167	TATTTTGGACGATTTTGCCC
scaffold6952_14.0	1	p2	(CT)6	12	493	504	TCGTCCGCTGCAGTAATATG
scaffold7013_13.2	1	p2	(TA)7	14	550	563	TGTCTTGCAGGCAATTCATAA
scaffold7196_16.8	1	p2	(TA)6	12	627	638	ACGGAAATGACCCTCATTCA
scaffold7245_14.4	1	p2	(TA)6	12	239	250	CCGATCCCACCTTGTTTTTA
scaffold7348_15.5	1	p2	(TA)8	16	380	395	TTCATTTGCCTAAAATGCCC
scaffold7671_15.8	1	p2	(CT)6	12	1325	1336	AAGGTGAACTCAAACGGCTC
scaffold7698_16.2	1	p2	(GA)7	14	1656	1669	ACAACTGGTTTCATCCGTGG
scaffold7781_16.5	1	p2	(TA)6	12	1023	1034	CTTGTTCAGCGTCACAAGGA
scaffold7789_12.7	1	p2	(TA)6	12	151	162	CAGATATTACGGTTGGCGCT
scaffold7795_12.6	1	p2	(TG)6	12	54	65	TGGAAATTAGACGATTTACACAAAA
scaffold7872_13.4	1	p2	(TA)8	16	1620	1635	ATTGGACACAACCCACACCT
scaffold7881_14.8	1	p2	(AT)6	12	1056	1067	CCTCTCTTTCCCCTCCATTC
scaffold7900_13.9	1	p2	(AG)9	18	71	88	AAAAGGTGGTGGTGGTGTTC
scaffold7903_15.7	1	p2	(AG)7	14	316	329	GTTCTGCCCTCCTTTTCCTC
scaffold7944_15.0	1	p2	(AG)6	12	466	477	ATTCCCCCAAAACCGAATAA
scaffold7956_13.3	1	p2	(TA)9	18	266	283	ACTAGATCACTCGGCCAACC
scaffold8351_16.2	1	p2	(AG)6	12	55	66	TGGAAATTCATCCACGTTCA
scaffold8352_11.8	1	p2	(TA)7	14	76	89	GCATACATGAATGGATGTGATTT
scaffold8401_11.5	1	p2	(AG)6	12	157	168	TTGGGCTCGAGTGAAAAGAT
scaffold8559_13.9	1	p2	(GA)6	12	265	276	TCCCAAACTTCGTGTGTGTT
scaffold8644_10.7	1	p2	(AC)8	16	47	62	GAAAAGCTTGGCCATTCAAC
scaffold8647_13.5	1	p2	(TA)6	12	831	842	CCCCCTTAATTACTTTGTCACG
scaffold9000_15.0	1	p2	(TC)6	12	1431	1442	GAAACCCGCTGTTTGGTTTA
scaffold9015_16.4	1	p2	(AG)6	12	78	89	TTTTGTTTGTTCCCAGGTTG
scaffold9063_17.2	1	p2	(TC)8	16	5059	5074	GCCCATTATGGAATCTCACG
scaffold9386_16.2	1	p2	(TA)6	12	398	409	ACTTTGAATCACAGCCGTCC
scaffold9656_15.1	1	p2	(CT)7	14	53	66	AAACAACAATTCATCAAATATCCG
scaffold9734_14.4	1	p2	(CT)6	12	273	284	ACTTTGCATCCCAGTCAGGA
scaffold9750_14.4	1	p2	(AG)9	18	2311	2328	TCCTGCATATTTGAGGTCCC
scaffold9825_15.2	1	p2	(AG)7	14	639	652	TCACCGGAGAAGCTTAGGAA

scaffold9930_12.2	1	p2	(TC)6	12	154	165	GGCTGCGATTTGTACTCTCC
scaffold9955_16.7	1	p2	(GT)8	16	2157	2172	GCAAATGTAGCACGCAGAAA
scaffold10006_19.5	1	p2	(TA)6	12	725	736	TTGTGCACTTCATATCGCGT
scaffold10021_17.7	1	p2	(TC)7	14	53	66	AAGTGAGAATGATGAGGGCG
scaffold10026_12.8	1	p2	(GA)7	14	674	687	TTCCTTTAGGCCATCCACAG
scaffold10202_18.3	1	p2	(TA)9	18	102	119	CACTGACATCCATCCATTGC
scaffold10504_12.8	1	p2	(AT)6	12	745	756	GATAGTATGCCAATTTTTCGTGA
scaffold10505_13.1	1	p2	(AT)9	18	391	408	TGGCACATTATTGGTCTCTCA
scaffold10509_11.7	1	p2	(AT)7	14	209	222	CGTACGTGTGTGTGTGCATT
scaffold10556_13.4	1	p2	(AG)6	12	1050	1061	AGAAGAGCAAATGGTGGTGG
scaffold10610_13.9	1	p2	(GA)6	12	103	114	CGAATTTGGTGTTGGAATTG
scaffold10725_10.5	1	p2	(TA)9	18	442	459	CAACGGTTGTCATTGTTTCCT
scaffold10927_11.0	1	p2	(GT)6	12	950	961	AACATTTCGTGGCCTTGATT
scaffold11109_15.2	1	p2	(AT)8	16	205	220	CGGCCGGAATATCTAAAGTG
scaffold11245_15.3	1	p2	(AT)6	12	100	111	AATATTGGATGTCTTGCGGC
scaffold11632_13.4	1	p2	(GT)6	12	147	158	TTCCCAAATTTTGATTCCGA
scaffold12076_16.0	1	p2	(TC)7	14	212	225	TTGCTTTCCAAAAAGTTGTACG
scaffold12338_19.0	1	p2	(TA)7	14	185	198	CGTGGTTCGGGTATTCACTT
scaffold12523_15.6	1	p2	(TC)7	14	1289	1302	CAATTTCCATGTTCCGCTCT
scaffold12579_14.6	1	p2	(TC)9	18	54	71	CATCCCAAACTCTCTCGCTC
scaffold12584_13.1	1	p2	(GA)9	18	194	211	CAACATTCCCCTCAACACCT
scaffold12609_15.7	1	p2	(TC)7	14	53	66	CGTCGATCCTTCCTCATCTC
scaffold12818_12.8	1	p2	(TC)6	12	406	417	TGCACTTGAGTTGCTTTTGC
scaffold12829_13.2	1	p2	(CT)6	12	190	201	CAATAACCCTTGGCTCGAAA
scaffold13262_12.2	1	p2	(CT)9	18	641	658	TCTTTATTTCCACCCCCTCC
scaffold13418_14.1	1	p2	(TC)6	12	461	472	TCTTCCCCAAATTGGTTTCC
scaffold13611_11.1	1	p2	(CT)6	12	250	261	CGTTGTTTGCTTCCAATTCA
scaffold13755_15.4	1	p2	(AT)7	14	1110	1123	AACGGACGTGTTTTGGCTAC
scaffold13867_12.0	1	p2	(GT)7	14	53	66	TCTGTGCTCTCTTTCTCAGTCG
scaffold14014_13.2	1	p2	(CT)6	12	257	268	TTCACCACCTAAAGAAGGGC
scaffold14047_13.9	1	p2	(TC)7	14	53	66	TTGGGAGGATTTTCTCTTGC
scaffold14058_10.3	1	p2	(CA)6	12	339	350	TTCCTTCATCTGTCACGTCG
scaffold14070_14.4	1	p2	(TA)9	18	542	559	TTATGAAATTCCGACGAGCA
scaffold14173_11.4	1	p2	(GA)9	18	641	658	TGACGAGAGCAGAGCAGAGA
scaffold14271_15.7	1	p2	(GA)6	12	63	74	TGTCCTGTTGAGGTGGAACA
scaffold14290_16.7	1	p2	(TA)6	12	1980	1991	TCCCACGTAACGAATTTTCC
scaffold14393_18.3	1	p2	(GA)7	14	1373	1386	ACAAAAAGCAGCACAACGTG
scaffold14571_15.9	1	p2	(TA)8	16	328	343	ACTGTCCTCTTAGACCGCCA
scaffold14576_13.7	1	p2	(AT)6	12	737	748	GATCGGCACGATAAGGACAC
scaffold14583_17.9	1	p2	(AT)7	14	696	709	TGGTAGGCGAACTCGTAACC
scaffold14676_12.1	1	p2	(AT)7	14	320	333	GTTGCCACCAATGTGTTTCA
scaffold14735_14.1	1	p2	(CT)6	12	1641	1652	TCAATGTTTTGCGTTTCCAA
scaffold14770_13.8	1	p2	(AT)6	12	138	149	AACAATGCAAGTGATATTCATCG
scaffold14976_15.1	1	p2	(CT)6	12	167	178	GAGCTTGCACTCAGAGAGCTT
scaffold15213_14.6	1	p2	(AG)7	14	53	66	GTGACCCTATGACGCCAAGT
scaffold15319_15.4	1	p2	(TA)6	12	942	953	TCCCGCTTCATAGCAAATTC
scaffold15396_15.5	1	p2	(AG)8	16	1941	1956	ATAAGGGTTTGTGCTCCGTG
scaffold15494_19.2	1	p2	(CT)8	16	580	595	AGTGCGTGCTTGTTTCAATG
scaffold15586_14.3	1	p2	(TA)6	12	533	544	TGGCGTTTGATATTTGAGAAGA
scaffold15592_15.7	1	p2	(TG)7	14	1084	1097	TTTCCACACCACGTCAGCTA
scaffold15604_13.1	1	p2	(TC)7	14	636	649	CGGTTGTGATAACGATGACG
scaffold15653_13.1	1	p2	(TG)6	12	251	262	CGTGTTCTTCACTCATCCCA
scaffold15658_16.5	1	p2	(AT)6	12	1223	1234	GCACTGAAACGCCAAAAGAT
scaffold15744_15.0	1	p2	(TC)6	12	108	119	CTCCCCAAACCCACTATCAC
scaffold15796_16.2	1	p2	(CA)6	12	2254	2265	GGCATGAAGGAAACCAAAGA
scaffold16102_19.0	1	p2	(AG)7	14	1097	1110	GTGACTGTGAGGGAGTGGGT
scaffold16183_16.3	1	p2	(AG)6	12	42	53	GATGAACTCTCTTGGGTGGG
scaffold16380_14.5	1	p2	(AG)8	16	2267	2282	AGAATGGCACAGACTCCAGAA

scaffold16415_18.1	1	p2	(GA)6	12	298	309	TCCGACTTACCACCACACAA
scaffold16429_15.3	1	p2	(GA)7	14	54	67	AACCCAGAAGAAAGACAGCAA
scaffold16490_10.1	1	p2	(AT)9	18	714	731	AGACTCATGCGGCTCTTGAT
scaffold16617_19.7	1	p2	(TA)6	12	510	521	TCTGTCGATCAACACCACAAA
scaffold16628_13.0	1	p2	(AT)8	16	712	727	TCGAGTGAGCATTTTGGATG
scaffold16695_15.3	1	p2	(CT)9	18	460	477	CCTCCAAATCTAACCCCTCC
scaffold16976_13.7	1	p2	(GA)7	14	665	678	AAACACACACACCGTCTCCA
scaffold16993_17.1	1	p2	(TA)6	12	145	156	CGTGCATTCCTACCCTATTTG
scaffold17025_17.7	1	p2	(TC)6	12	106	117	TCTCATTCACGCTTTTCAGC
scaffold17035_13.6	1	p2	(CT)6	12	2315	2326	AAGGAGTCCTCTCAGTTTCATCA
scaffold17725_11.1	1	p2	(AG)6	12	277	288	CCTTGAAGACAATGAAATTTTGG
scaffold17815_12.7	1	p2	(AG)6	12	146	157	TGTTATCCTTTGTGAGCCCC
scaffold17887_14.0	1	p2	(GC)6	12	210	221	GTTCAAGTCCGGCTCTTCAG
scaffold17896_15.4	1	p2	(TC)6	12	128	139	GATTTGCACCTTCCACGAAT
scaffold17935_10.1	1	p2	(AT)7	14	185	198	GTCGGAACACTTCGTGTTGA
scaffold18182_11.5	1	p2	(TA)7	14	184	197	ACACATTTTTCTTTTGGGCG
scaffold18195_18.3	1	p2	(GA)7	14	54	67	CATGAGAGAGAGGGCTGGAA
scaffold18226_11.3	1	p2	(CT)6	12	75	86	CCCCCAAAGTTATGGTGAAA
scaffold18386_13.8	1	p2	(GT)6	12	109	120	AGTGCACGTCAGAGTTGGC
scaffold18474_15.9	1	p2	(TA)7	14	400	413	CAATCAATCTCAGCCGTTTG
scaffold18527_15.1	1	p2	(AT)8	16	105	120	AAACATTTTACCACATGATTAAGACC
scaffold18576_15.8	1	p2	(TA)6	12	477	488	ACCAATTGCCCATCTGCTAC
scaffold18624_12.6	1	p2	(AT)8	16	2083	2098	CACCAAACTGATTAAAATCCCAA
scaffold18640_12.5	1	p2	(TA)7	14	54	67	GGTTTCACGTCGGAATATTGA
scaffold18659_17.0	1	p2	(AT)6	12	1468	1479	TCCGAATATGAAATCGGGTT
scaffold18668_12.8	1	p2	(TG)7	14	1222	1235	TCGAGAAGTTGTTTGTCTCAACA
scaffold18787_12.1	1	p2	(CA)6	12	208	219	GGTCCCCTCCTAACTTTGCT
scaffold18794_15.1	1	p2	(TG)6	12	461	472	CCCCGAGGAAGCTAGAAAAA
scaffold19150_15.0	1	p2	(TC)6	12	109	120	TAATCCACTCCCACTTTCCG
scaffold19158_15.9	1	p2	(GA)6	12	56	67	CGGGCAGAGAGAGAGAACC
scaffold19324_12.0	1	p2	(AG)11	22	374	395	GGGTGGGTTTGGAGAAGAGT
scaffold19347_14.4	1	p2	(AT)7	14	416	429	GGACATTCCGTGGAGTTTGA
scaffold19477_14.8	1	p2	(CT)6	12	314	325	GTCACGTCAGCAAACCATTG
scaffold19491_14.8	1	p2	(AT)6	12	1301	1312	GAGATAGGGGTTGGGGTGTT
scaffold19757_16.2	1	p2	(TC)10	20	102	121	ACGTTGGACCTGAAAGAGCA
scaffold19937_15.1	1	p2	(TA)6	12	195	206	AACCTTAACAAAATGAATCATCAAA
scaffold19974_18.0	1	p2	(AT)6	12	81	92	TTCCAACTCCATTTGCAATACT
scaffold20002_17.4	1	p2	(TA)7	14	152	165	AAAGCCTACCTTGCTTCCGT
scaffold20074_13.5	1	p2	(GA)8	16	172	187	GGAGAAACTTGCTTCTTGGC
scaffold20104_13.2	1	p2	(TA)10	20	1093	1112	GATCCACATGCATGAAATCG
scaffold20185_15.1	1	p2	(AG)6	12	570	581	AATAACAAATGGCCACGGAA
scaffold20444_16.2	1	p2	(AC)6	12	322	333	CAAAGGAAGGGCAGAAACAA
scaffold20565_11.9	1	p2	(GA)9	18	486	503	TGCATTCCTTTCCAGTCTCA
scaffold20626_15.3	1	p2	(AT)6	12	1201	1212	CCGTTCCGTTCCATAAATGA
scaffold20660_14.1	1	p2	(AG)7	14	900	913	CAGGCCAACTCCTATCTGGA
scaffold20704_12.8	1	p2	(TC)6	12	182	193	TAAAAATGGGAGATGAGGCG
scaffold20825_11.7	1	p2	(TA)7	14	729	742	TTTTAATTCATTACTCATATTTGCATC
scaffold20936_12.9	1	p2	(CT)6	12	46	57	CTCTCTCCTGCGTCTCTCTCA
scaffold21030_12.2	1	p2	(AT)9	18	831	848	TGCTATTGAAAATGATCCCACA
scaffold21236_14.6	1	p2	(AG)7	14	1914	1927	CAAGACCTACCGAGCTTCTGA
scaffold21289_15.3	1	p2	(TA)6	12	3727	3738	TCGAAGGGGATTTTATGTGG
scaffold21314_13.4	1	p2	(TA)8	16	151	166	TAGAAATGGGATACGGACGG
scaffold21318_16.3	1	p2	(CT)6	12	2654	2665	TTTGACAGACAAATCGCTCG
scaffold21423_10.9	1	p2	(TA)10	20	111	130	CGCAGATCTAATGGTCCAGA
scaffold21430_13.6	1	p2	(TA)8	16	105	120	GCACCACAAACATGTCACAA
scaffold21500_15.1	1	p2	(TA)7	14	326	339	CGGATTGTAAATCGGGAGAA
scaffold21518_14.4	1	p2	(TA)7	14	313	326	TCTTCGTCGTACAACTCGTCA
scaffold21688_15.8	1	p2	(GA)9	18	536	553	ATGCCCATTCCAAAACACAC

scaffold21713_11.3	1	p2	(AG)7	14	54	67	CGCCTTTTCACCTCAAATTC
scaffold21799_14.1	1	p2	(AT)7	14	143	156	GCTACTTCCGACTCTCTCGC
scaffold21832_12.9	1	p2	(TA)8	16	1049	1064	TCTTCCACGATCTCCCATACA
scaffold21974_19.3	1	p2	(TC)6	12	55	66	TGCTAATCAGAAACTTGGAGCA
scaffold22095_13.0	1	p2	(AT)9	18	6948	6965	CATGGTTTGAGACGGATTGA
scaffold22126_16.2	1	p2	(GA)6	12	267	278	GATATTGACGGTTGGTTGGC
scaffold22303_17.6	1	p2	(AG)7	14	510	523	ACGCAAGCGATATGGAAAAG
scaffold22306_11.5	1	p2	(CG)6	12	52	63	CATGGGTGGTATGGTGTGG
scaffold22391_13.0	1	p2	(GA)6	12	86	97	GATCGGTGAGGAGATGAAGG
scaffold22631_14.2	1	p2	(AT)8	16	1517	1532	AAGTTGGCTAGGTGGCCTCT
scaffold22751_13.1	1	p2	(AT)6	12	54	65	TTTACGTGTTAAGCCTTGGGA
scaffold22868_14.3	1	p2	(AG)11	22	168	189	ATGCGTGTGTGTCTGCATGT
scaffold23028_10.7	1	p2	(CT)6	12	24	35	CTTCTCTTCCACATCCGGTA
scaffold23082_15.0	1	p2	(CT)6	12	100	111	TTCGATGTTGTAACGGAGACA
scaffold23170_14.8	1	p2	(TC)7	14	52	65	TGGCTCTCACACTCACTCTCA
scaffold23350_12.1	1	p2	(TC)6	12	737	748	TCGATCGATAATAACGCTTTCA
scaffold23417_14.4	1	p2	(AG)9	18	240	257	AGATTCCGTCCGATTTAGCA
scaffold23575_15.8	1	p2	(GA)6	12	3568	3579	CTTCCCACATGTCGTTCCTT
scaffold23652_15.3	1	p2	(TC)6	12	664	675	GTGGGTGCAGTTGGATTACC
scaffold23713_17.6	1	p2	(AG)6	12	1626	1637	GCTGCGAAGAACACAATCAA
scaffold23732_15.9	1	p2	(TC)8	16	315	330	TGTGGAAACAAGTGCAGAGG
scaffold23750_12.1	1	p2	(AG)6	12	151	162	TCGAAACAGAAAATCCGAGG
scaffold23881_16.2	1	p2	(TA)6	12	56	67	GGGGCGTGACAATATCCTAA
scaffold23894_10.3	1	p2	(TA)7	14	107	120	GTCGCTTGAATTGCGACATA
scaffold23961_11.5	1	p2	(TC)6	12	271	282	ATGTGGTCCCAAACTCTCCA
scaffold23963_13.3	1	p2	(TA)6	12	875	886	CCGGCTCTGGGTACAGATTA
scaffold24071_10.1	1	p2	(CT)7	14	47	60	CACTCACTCTCACTAGCCGC
scaffold24205_16.1	1	p2	(TC)7	14	54	67	ACGCATTCAAAGCTGAAGAT
scaffold24355_15.5	1	p2	(TC)8	16	3453	3468	AGACTGGGGAATGGCTTCTT
scaffold24430_13.8	1	p2	(TA)9	18	325	342	TTGGGAATTCAAAATGATTCG
scaffold24613_13.4	1	p2	(AG)6	12	162	173	ACTCGCATTAATGAGCCGTC
scaffold24667_14.6	1	p2	(AC)7	14	3151	3164	TTTGTCAACTTCCCTCACCC
scaffold24685_16.0	1	p2	(AC)8	16	1605	1620	AGCACTGTGAGACTGAGGCA
scaffold24712_11.9	1	p2	(TA)7	14	84	97	AAGGCATCAAAGTTGTCGAT
scaffold24832_10.0	1	p2	(TC)6	12	77	88	CGAGGGCATTATGGGAAGT
scaffold24883_15.3	1	p2	(CT)10	20	194	213	AATGATAATCTTCGCCGTGG
scaffold24945_16.0	1	p2	(TA)7	14	90	103	GCAATAAATCCACGTACAGGAAA
scaffold24992_12.5	1	p2	(TA)9	18	276	293	AAAACACCTCCTTAGGTGCAAA
scaffold25112_16.3	1	p2	(TC)7	14	54	67	GGAAATGAAAGGCCAAGTCA
scaffold25265_10.5	1	p2	(TA)6	12	334	345	CGAAGAACACACACTTTTGGG
scaffold25281_11.2	1	p2	(AT)7	14	54	67	ATCACCAACTGGCGAGAAAT
scaffold25398_17.5	1	p2	(CT)6	12	499	510	ACCCTATCTGCCCCAATACC
scaffold25401_17.5	1	p2	(CT)6	12	71	82	CAAATGGCCAAGAAAGTTTACA
scaffold25421_14.5	1	p2	(CT)6	12	56	67	GGGGACTTTTCACTTTTGGC
scaffold25507_12.4	1	p2	(GA)6	12	107	118	TGAAGAATTCCCTTGGGTTG
scaffold25519_19.2	1	p2	(AG)7	14	192	205	TTTCTAACGATGATGGAAAAACAA
scaffold25718_12.1	1	p2	(AT)7	14	564	577	TCAATGGTTTTGGTTTGTTCC
scaffold25729_16.1	1	p2	(CA)6	12	2978	2989	ATTTCACCTGCAGAAACGCT
scaffold25792_13.9	1	p2	(GA)10	20	1178	1197	TTGGCCTTTCTTGATTGTCA
scaffold25870_21.8	1	p2	(TC)7	14	496	509	TCCACTTAGGTTGGCTTTGC
scaffold25893_16.0	1	p2	(AT)6	12	6106	6117	TTTCTCTTCACACGTTTGGATT
scaffold26018_11.9	1	p2	(GA)6	12	297	308	AGACAAGAGCCGAAAAGTCG
scaffold26053_16.5	1	p2	(CT)6	12	38	49	ACTCTATAGCAAGATCTCACGCA
scaffold26077_14.0	1	p2	(AT)8	16	3308	3323	ACATGATTCGTCGGGTCTTC
scaffold26197_13.9	1	p2	(TA)8	16	464	479	CACATTAAGAGGAGGTATCATTTGAA
scaffold26225_17.2	1	p2	(TC)6	12	270	281	AAGTTGGGCTAAGTGGAGGG
scaffold26343_18.6	1	p2	(GA)6	12	914	925	AGTAGTTCCGGTCGGGTGAT
scaffold26943_13.7	1	p2	(TC)7	14	538	551	CTGTTAGGCCCATGTCGTTT

scaffold27238_15.1	1	p2	(TA)8	16	779	794	TATGTTGGACCAAAAAGCCC
scaffold27243_17.9	1	p2	(CT)8	16	507	522	CGTTGTTTCCTCACACCCTT
scaffold27244_13.3	1	p2	(AT)6	12	50	61	CATTTCATTTCAGGAGCTTCG
scaffold27248_10.3	1	p2	(AT)7	14	309	322	TCCATTTTTATCTTCTCTATCTTCCC
scaffold27280_11.3	1	p2	(AC)6	12	328	339	GGCAGCAATCAATTGGAAAT
scaffold27421_16.2	1	p2	(TA)9	18	290	307	AGATCATCACGTAAACGGGG
scaffold27545_16.5	1	p2	(AT)7	14	355	368	ACAGCTTACACCGTTGAGCA
scaffold27709_16.3	1	p2	(GA)6	12	517	528	GAGCAAGGAGAAGTTCGTGG
scaffold27756_17.8	1	p2	(AT)12	24	1058	1081	GGTGAACATTGGGAGCAAAT
scaffold28165_11.9	1	p2	(TA)7	14	340	353	ACCGATTGTTGCATTTTAAGTT
scaffold28175_14.2	1	p2	(AT)6	12	824	835	AAGTTGTGTCCCCCAACACT
scaffold28277_17.2	1	p2	(AG)6	12	55	66	TGTATTTAATTGAATGCTAGAGAGAG
scaffold28322_13.6	1	p2	(GT)6	12	55	66	GGGTAACTTGGTTCTGTAACCG
scaffold28370_12.0	1	p2	(TC)6	12	55	66	CCCACTAACCCTTCATCACC
scaffold28421_10.0	1	p2	(TC)6	12	285	296	TGTGGAATATTGTCCCGGAT
scaffold28565_10.2	1	p2	(TA)9	18	102	119	CTTAGCCGAGAAGAACCGTG
scaffold28616_10.2	1	p2	(TA)6	12	55	66	CTCCAAATGATAAAATCGTGG
scaffold28682_12.6	1	p2	(TA)6	12	408	419	AGTCTTCACAAGCGGGTGTC
scaffold28700_12.6	1	p2	(TA)8	16	1029	1044	TCAATTTGTGTCTTATAAATTAAATGC
scaffold28762_11.4	1	p2	(TA)6	12	185	196	TTCCCTAGGATGAAAACTTGC
scaffold28851_15.9	1	p2	(AT)6	12	55	66	CGAACGGTTGGAATATCTAAAG
scaffold28911_16.2	1	p2	(CT)7	14	781	794	CAAAACCCAAATCCCCTTTT
scaffold29014_12.6	1	p2	(TA)6	12	55	66	AGTTATGCGTATTGTTACCTTCATT
scaffold29070_10.9	1	p2	(GA)7	14	147	160	CTTTGCGACGTAATTCCCAT
scaffold29112_15.1	1	p2	(CT)6	12	461	472	TGTATCAGTCACCACCTCCACT
scaffold29162_11.8	1	p2	(AT)8	16	106	121	TTGTGCCTTTTATTTGAGTAAGACA
scaffold29163_13.4	1	p2	(AT)8	16	488	503	TTCAGCCTTTTTCATGTGGA
scaffold29164_10.9	1	p2	(TA)6	12	126	137	GCTTGTAATTTTTGGCTGGC
scaffold29178_12.8	1	p2	(TA)10	20	74	93	CGGGTTATTACCTGATTGCG
scaffold29216_14.0	1	p2	(CT)6	12	280	291	ACCAACCCATCAAGAATCCA
scaffold29242_16.6	1	p2	(CA)7	14	149	162	AAACGAAAAAGCCGAAACTG
scaffold29377_19.5	1	p2	(GA)6	12	55	66	TCAAGATGAGGAATGAAAAAGAGA
scaffold29487_13.1	1	p2	(TA)7	14	54	67	GGAAATAAGGAGGTGTTATGAAAGG
scaffold29563_10.8	1	p2	(GA)6	12	55	66	GGTGATGGTTGGTGTTGATG
scaffold29678_12.7	1	p2	(AG)6	12	55	66	AGGGGGAAGAGGTTGAAGAC
scaffold29824_16.7	1	p2	(AG)6	12	213	224	TTTGATAGAGCCGGATTTGG
scaffold29880_16.2	1	p2	(AT)9	18	1913	1930	CGAAGTTTGCATTTTAAGATGAGTT
scaffold30087_15.1	1	p2	(AT)8	16	626	641	TGTTTTGTCTTTGTGGTGCG
scaffold30637_10.1	1	p2	(AT)10	20	50	69	TGGTTACTGTTACGTATGCCAA
scaffold30666_11.8	1	p2	(CT)9	18	569	586	TAACCGTTGTTTTCCCCTCC
scaffold30669_16.5	1	p2	(CT)7	14	1482	1495	ACCATCAAGGTCAAACCTGC
scaffold30692_17.7	1	p2	(AT)9	18	1571	1588	GCTGTTGTGTTGGCATTAGC
scaffold30807_14.9	1	p2	(AT)9	18	349	366	AATGGGGACATCTGGTGTGT
scaffold30864_10.4	1	p2	(AT)6	12	254	265	AGGGAGAAGATGAGGAGGGA
scaffold30982_16.9	1	p2	(AT)6	12	3530	3541	CGCCGTTTCACCATTTTATT
scaffold30986_16.9	1	p2	(TA)7	14	3593	3606	GGGCCCACATTAGCTGTCT
scaffold31055_16.3	1	p2	(CT)9	18	5870	5887	AATTTAGTCGGCCGGATTTT
scaffold31144_12.9	1	p2	(AT)9	18	103	120	TTGAACCGTGTTACCCTTCC
scaffold31172_13.2	1	p2	(TA)7	14	489	502	GATTCTCCAAATCTGACGGC
scaffold31482_14.2	1	p2	(TA)8	16	2153	2168	TTTTCCCCAAAACACCTCTG
scaffold31497_16.4	1	p2	(TG)7	14	843	856	TCTTCTCCAACCCACAAACC
scaffold31576_15.2	1	p2	(AT)6	12	55	66	TCCTAACAACAAAAGATAAAGTCAA
scaffold31729_17.7	1	p2	(CT)9	18	196	213	ACCACCACCACCTCAACATT
scaffold31755_12.2	1	p2	(GT)9	18	103	120	TCTGCGGTACATCCAAATGA
scaffold31756_16.4	1	p2	(CT)7	14	202	215	TCACACTCACTCTCACTAGCTGC
scaffold31788_15.6	1	p2	(TC)7	14	492	505	AGATGTTCCCAGAGTGGTGG
scaffold31824_14.6	1	p2	(TA)8	16	485	500	ATTACCTCCACGCCGAGAG
scaffold31917_14.4	1	p2	(TC)8	16	1963	1978	GTTGGAAAATGGTGGAATGG

scaffold32086_13.9	1	p2	(TC)9	18	493	510	TGCACCCCAAGATGGTAAAC
scaffold32184_12.1	1	p2	(TA)7	14	488	501	GCTTGCTGAGTTGGCATGTA
scaffold32321_10.7	1	p2	(TA)12	24	482	505	ATTTCGAAGATCAACGGACG
scaffold32385_13.2	1	p2	(TA)10	20	770	789	AAAAATGTGCATGCAACTGA
scaffold32440_15.7	1	p2	(CT)7	14	63	76	TCTCTCTTTTTGGTTGGTGGA
scaffold32447_13.2	1	p2	(AT)6	12	203	214	GAGGTTTTGGGAGAAGGAGG
scaffold32610_11.9	1	p2	(TG)8	16	821	836	TCAGTGCTGAACGTGTCAAA
scaffold32640_17.1	1	p2	(AT)7	14	302	315	CGGTCTACCAGTCCGTGAAT
scaffold32686_10.9	1	p2	(AT)10	20	48	67	AGTGTTTTGACATGATTACTCGTC
scaffold32799_13.2	1	p2	(TA)12	24	1740	1763	AAATATCGGCCGTCAGATTG
scaffold32886_15.2	1	p2	(TC)9	18	95	112	ACTGCAGAAAAATTCACGCC
scaffold33031_10.2	1	p2	(CT)6	12	119	130	ACACATGGCCACTAACACCA
scaffold33109_14.7	1	p2	(TA)6	12	1223	1234	TTTGTTCTCGTTCAAACCCC
scaffold33135_11.2	1	p2	(CA)6	12	55	66	CTCTCACACACGCAGTTAGACA
scaffold33204_15.2	1	p2	(AT)9	18	3309	3326	ATTTAATGTCCCACCCCGAG
scaffold33339_13.1	1	p2	(TA)8	16	2780	2795	CTCGCCGCTTAAAATTGTGT
scaffold33458_10.0	1	p2	(AT)7	14	230	243	TTCTTGGCATCCAGTTCCTT
scaffold33501_18.8	1	p2	(TA)7	14	52	65	AAGGGGGTTATTAGTGGATCA
scaffold33588_14.5	1	p2	(AT)6	12	995	1006	TTGGTGTTATGGATTGTGTGA
scaffold33613_14.8	1	p2	(TA)6	12	55	66	ACTATGTGCCCTCGCCAATA
scaffold34095_15.5	1	p2	(AG)7	14	215	228	CTGAACATTGCCTGCTTGAA
scaffold34230_15.7	1	p2	(TC)13	26	510	535	AGCCTCACTGCAGCTGTCTA
scaffold34316_18.1	1	p2	(TA)6	12	55	66	TCGTCATTTATACAAAAACAATTCC
scaffold34388_14.5	1	p2	(TA)9	18	163	180	CCCCTCGTATGCACTCAACT
scaffold34491_19.2	1	p2	(GA)8	16	216	231	CACCATCATCCTCAGAGCAA
scaffold34494_17.0	1	p2	(TA)8	16	623	638	CGTGAATGAAAGTGATTGCG
scaffold34497_12.4	1	p2	(TA)6	12	91	102	AGATTTGGGCAGTGTGGTGT
scaffold34548_16.8	1	p2	(CA)7	14	38	51	GCGAATCGAACGAATCTGA
scaffold34600_12.6	1	p2	(AT)8	16	2999	3014	AACACCTCCTTATTTCCTAACTTCA
scaffold34616_11.1	1	p2	(CT)6	12	284	295	CTCTCAGCTCTTTTGCCTGG
scaffold34701_10.7	1	p2	(AG)6	12	219	230	TGTTCGTTGGTGGAGTTGAG
scaffold34734_14.6	1	p2	(GA)6	12	55	66	GTCGGAAAAGCTTGGATGAA
scaffold34894_11.4	1	p2	(TC)6	12	47	58	TCTTTTGGTAGGTGAATGTATTGAA
scaffold35174_11.2	1	p2	(AC)6	12	219	230	TCGTGTCAGAAATCGTCAGC
scaffold35218_15.9	1	p2	(AT)8	16	540	555	TCGCCTTGTTTAGGAGAGGA
scaffold35323_14.5	1	p2	(CT)6	12	215	226	ATATGGCAATGGGACCAAGA
scaffold35350_13.8	1	p2	(GA)7	14	460	473	TTCATCAACATGCAGCCATT
scaffold35374_12.7	1	p2	(AT)6	12	75	86	GGTGCTTGGTGAAGGAAGTG
scaffold35399_14.9	1	p2	(AT)10	20	283	302	TGCAGTTGAGATGTCCTTGC
scaffold35412_13.8	1	p2	(AT)6	12	2772	2783	ACATGGGCCAAATGAGAGAC
scaffold35419_14.1	1	p2	(CT)8	16	3146	3161	TTTTCAAGGAGGGGTGAATG
scaffold35439_10.6	1	p2	(AT)7	14	271	284	TATGGATTATCACCGGATCG
scaffold35482_20.2	1	p2	(GA)6	12	191	202	TAAATGGCAGTGTGGGTTGA
scaffold35966_10.2	1	p2	(AT)6	12	181	192	TCAACAAAATTCTAAAATTGCAGG
scaffold36081_12.6	1	p2	(TA)8	16	40	55	CGGATTTAAGGGTTATTACCTGAT
scaffold36127_11.0	1	p2	(TC)7	14	252	265	GAACACGAGAGGAAGAACGC
scaffold36213_15.1	1	p2	(TA)9	18	341	358	GGCGAATGATGTTTCTAGGG
scaffold36244_16.4	1	p2	(AG)9	18	293	310	CGCATTATGGTTATGCGAAA
scaffold36295_13.7	1	p2	(AT)7	14	1698	1711	AACGGTTTGAGCCTATTGAGT
scaffold36339_12.6	1	p2	(GT)6	12	445	456	CTTGGAAGAAAAACCCCACA
scaffold36392_21.6	1	p2	(AT)8	16	622	637	CTATTCGAATGCGAGGGAAA
scaffold36445_15.4	1	p2	(TA)6	12	1273	1284	CATTCCAACCTCCTAATCAAGAA
scaffold36447_15.7	1	p2	(AC)9	18	1076	1093	AATGTCGCCATCATATTTTGC
scaffold36452_14.7	1	p2	(TA)7	14	684	697	GCTCATTCTGCTTCTTCCGT
scaffold36489_15.8	1	p2	(TA)6	12	1083	1094	GAGTTCGATCTGGAACCTCG
scaffold36504_15.6	1	p2	(AG)6	12	1477	1488	CTTGACCGCAAGAAAGGTTC
scaffold36545_14.3	1	p2	(TA)8	16	1789	1804	TTCGGGCTAAACGAGTCAAT
scaffold36548_14.1	1	p2	(TA)9	18	654	671	GGGCACTCAACCCTTCAATA

scaffold36648_10.7	1	p2	(GT)8	16	50	65	AGGATGTAAATACGGTCTGTGC
scaffold36658_13.4	1	p2	(GA)6	12	569	580	CGTTCATTCGTATTGGGCTT
scaffold36735_11.1	1	p2	(AT)6	12	218	229	AAGTGACGACTCACGACCCT
scaffold36905_16.0	1	p2	(TA)7	14	1210	1223	TGTATGGTCTCCTTCCCTTTG
scaffold37028_13.0	1	p2	(TC)6	12	376	387	CTCCAACACCTCCAACTTGC
scaffold37093_16.9	1	p2	(AT)6	12	143	154	TCTTTAGCCCAAGTTGTATCCAT
scaffold37106_10.5	1	p2	(CT)10	20	112	131	AAAAATGGCGATGAAACCAG
scaffold37279_16.1	1	p2	(TA)6	12	54	65	TGAGCTGTTATGCGTTTTGA
scaffold37396_11.8	1	p2	(CA)6	12	137	148	GAACACCGGATTTACGTGGT
scaffold37402_11.1	1	p2	(CT)7	14	351	364	TAAGCAATGAGCGAAAAGCA
scaffold37499_11.9	1	p2	(GA)6	12	56	67	GTTAAGTAACCGGAGAGGCG
scaffold37527_15.9	1	p2	(GA)8	16	539	554	TCAGAAATTGTAGTCGCCCA
scaffold37548_13.2	1	p2	(AT)6	12	93	104	TTGGAACTTGAGCAACTGGA
scaffold37569_11.9	1	p2	(GA)11	22	969	990	TCTGGGGTTTCAGGTAGTCG
scaffold37624_15.6	1	p2	(AT)7	14	1029	1042	ATAGGGGCATATTCAGGTGC
scaffold37664_12.5	1	p2	(AC)6	12	56	67	AGAGGTAGAAGCACTTGTGTGG
scaffold37683_12.8	1	p2	(TA)6	12	226	237	AGGGCAACATTCGTCAAAAA
scaffold37761_10.4	1	p2	(AT)6	12	220	231	CGCACGTCGAGAATTGAATA
scaffold37815_13.5	1	p2	(GA)7	14	160	173	CGGGTTTTAGTGGAGAACGA
scaffold37878_10.9	1	p2	(AT)6	12	145	156	GCCACCCTTAAGTGATTTCG
scaffold37889_13.4	1	p2	(AT)6	12	574	585	GCTGCGAAAAAGGTCATCAT
scaffold37905_14.1	1	p2	(AG)8	16	1601	1616	GCTTACTCCCACCGATGTTC
scaffold37935_15.4	1	p2	(TA)9	18	371	388	CACATGCATGCTCGTACTCA
scaffold38074_13.9	1	p2	(AT)8	16	576	591	TCCTTTTTGTCTTTCATTCGG
scaffold38219_16.5	1	p2	(CT)6	12	626	637	GAAGGGAAACCGGTTAGGAC
scaffold38234_12.2	1	p2	(TC)9	18	635	652	TCAGCCGGAGAAACTCATCT
scaffold38307_15.4	1	p2	(GA)7	14	2060	2073	CAGCGCAGATAACAATTGGA
scaffold38377_17.6	1	p2	(AT)6	12	56	67	ACACTCGACAATTTGGGAGG
scaffold38415_16.6	1	p2	(TG)6	12	82	93	TGCTTTTCAACTTAGAATTCAATCA
scaffold38622_10.0	1	p2	(GT)7	14	300	313	GTGAGCTGAAGCGAGCTGAT
scaffold38717_15.0	1	p2	(AT)6	12	56	67	AAAACTATAGGTGTTTGGTAAACGTC
scaffold38766_16.9	1	p2	(AG)7	14	39	52	CAATTACAATGTTTATCCAATCCC
scaffold38991_10.0	1	p2	(CA)6	12	56	67	CAGGATCTCATCGGAACCAT
scaffold39087_13.1	1	p2	(AC)7	14	816	829	TTTCCGATTCTCAATTCCCA
scaffold39139_16.8	1	p2	(TA)8	16	857	872	TTGTGCATGCGGACTATGTT
scaffold39253_17.1	1	p2	(AG)7	14	612	625	GCAGCGTCGTTCTTCAGAG
scaffold39351_20.9	1	p2	(CT)6	12	56	67	AATCTCACAAACCCACACCC
scaffold39404_10.6	1	p2	(TC)6	12	56	67	TGGTCACTTACACAGCAATCAA
scaffold39519_18.2	1	p2	(CT)6	12	281	292	CTCGTCAACTGCTCGGTACA
scaffold39522_18.4	1	p2	(TA)6	12	142	153	TGCAGTAGGTTGTAGGTACTAAAATA
scaffold39545_12.3	1	p2	(AT)6	12	125	136	TGAATTTGGGTCATGAATTTTG
scaffold39578_13.5	1	p2	(TA)6	12	1046	1057	GGATTTTCTCGATTGCCAGA
scaffold39580_10.9	1	p2	(TA)8	16	130	145	GTGGCTAGATGCAAGCCTTT
scaffold39635_12.6	1	p2	(TA)7	14	406	419	GGGCAGTAAGTTGAGTCCGA
scaffold39779_14.2	1	p2	(AT)7	14	341	354	TTGATTGGTTGTTGGGCATA
scaffold39902_11.9	1	p2	(TA)8	16	1373	1388	GGCTTACCCCTGCACTTATG
scaffold40050_12.7	1	p2	(AG)6	12	56	67	TCGCACTAAAGCAGAACGAA
scaffold40145_11.0	1	p2	(GC)7	14	869	882	TCAGCCCAGCCCAATATTAC
scaffold40299_10.4	1	p2	(TA)9	18	104	121	CCCCTATTTTTGGAGGGAAA
scaffold40302_14.9	1	p2	(AT)7	14	3753	3766	GCACCACCATCTTCTTCGAT
scaffold40385_16.2	1	p2	(AC)6	12	1606	1617	CAGCAACCTGAAAGCATGTG
scaffold40409_17.2	1	p2	(TA)6	12	204	215	TTAAGGGGCGGTATCATTTG
scaffold40443_15.1	1	p2	(AT)6	12	967	978	AATTTGGTGGATGGGACAAA
scaffold40508_17.2	1	p2	(AG)8	16	1050	1065	CATGCAATGCGATGTGTTTT
scaffold40511_12.5	1	p2	(TC)8	16	1435	1450	GCCACCTCAGAAGATGGAAG
scaffold40583_10.0	1	p2	(CA)8	16	54	69	CAATTCATAAAATACATAGAAAGGTG
scaffold40611_13.6	1	p2	(AT)6	12	83	94	CCCCATCAACACACATACACA
scaffold40637_16.6	1	p2	(TC)6	12	39	50	CAAACCCCATCACCAACTCT

scaffold40681_13.1	1	p2	(TA)6	12	81	92	GAAGTTTTCGGCCTTTTGAA
scaffold40726_11.4	1	p2	(CA)7	14	913	926	TTCAGGCAATACCAGTTCCC
scaffold40747_16.6	1	p2	(AG)6	12	37	48	GAAGAACAATTACAATGTTTATCCAA
scaffold40752_20.5	1	p2	(AT)8	16	655	670	TGTTTGCAATTATTTCGTGG
scaffold40921_19.5	1	p2	(AT)6	12	56	67	TGCATCAAGTTCTCTTCTCTAATTTT
scaffold40926_10.3	1	p2	(AT)6	12	129	140	TTGAACCGATGAAACTAAAATCAA
scaffold40985_14.7	1	p2	(TC)6	12	308	319	GTTGGAAGTGCGAATGTTCA
scaffold41039_18.8	1	p2	(AG)8	16	2884	2899	AGAATGGGGAAGCTCCAACT
scaffold41047_10.5	1	p2	(AT)6	12	176	187	AGTTGAACCTCGAACCGTTG
scaffold41081_15.8	1	p2	(TA)6	12	690	701	AATCATTGTGCGCATGTGTT
scaffold41215_13.5	1	p2	(TA)6	12	2325	2336	CTAGGCCCAAGGTAGTGGTG
scaffold41519_16.8	1	p2	(AC)6	12	2182	2193	ACGTGTTCAAGGTTGAAGGG
scaffold41908_11.3	1	p2	(CA)6	12	462	473	AAAGGCATCTGAGCCGTTAC
scaffold41963_12.2	1	p2	(AG)6	12	151	162	TCGGCTGGGGTAGTAATTTG
scaffold41965_17.8	1	p2	(GA)7	14	195	208	CCGAGATAGGAGCAAGCACT
scaffold41971_15.8	1	p2	(GA)8	16	234	249	GGGAAGAAAAACGACGAGAG
scaffold41989_15.1	1	p2	(TC)8	16	1837	1852	GTTTCCCAAAGCCACAAAAA
scaffold42065_17.4	1	p2	(CT)8	16	971	986	AAAATGAGGAAATCCCTCGG
scaffold42288_20.7	1	p2	(TC)6	12	156	167	CTCCAACACCTCCAACTTGC
scaffold42290_16.0	1	p2	(CT)8	16	2188	2203	ATTTCCTGCCCCAAATTACC
scaffold42291_10.7	1	p2	(AT)6	12	210	221	TGAAACTCCATCAAAACCTCTTT
scaffold42364_11.5	1	p2	(AC)7	14	74	87	GGCAGCAATCAATTGGAAAT
scaffold42625_17.2	1	p2	(GA)6	12	1056	1067	AGATTGGAGCGAAGAGTGGA
scaffold42762_12.4	1	p2	(AG)6	12	67	78	TGTTTTGTAATTTCCCATGTTTG
scaffold42948_16.5	1	p2	(CT)8	16	54	69	TCAATTTTCTCAAGAAACCTCC
scaffold43012_12.3	1	p2	(AT)6	12	259	270	AGAAACGCATCGTTTTCTGG
scaffold43292_10.5	1	p2	(AT)6	12	56	67	CCGATACGGTATAATTTCCAAAA
scaffold43398_15.6	1	p2	(TC)6	12	235	246	ATAACCCCACCATCCTAGCC
scaffold43419_13.8	1	p2	(AG)6	12	521	532	AGGCAACTTGGTGGAGAAGA
scaffold43445_16.4	1	p2	(TC)6	12	2766	2777	TTTCAGTTTCGCCATTTTCC
scaffold43964_12.6	1	p2	(TC)8	16	188	203	AAACAAGCCAATAAAGGCCA
scaffold43975_15.8	1	p2	(CT)6	12	1805	1816	GGCTAGATGCAGTGGACCAT
scaffold44077_15.7	1	p2	(TA)6	12	591	602	AGGATGGTGATGTTGAAGCC
scaffold44096_12.8	1	p2	(TA)10	20	474	493	GTATACACGGTCCGGTTTGG
scaffold44104_11.2	1	p2	(AT)10	20	463	482	GTGCGTGTGTATGCTGAGGT
scaffold44276_13.6	1	p2	(TA)6	12	1524	1535	CCTATATCAGCGGCCAAAAA
scaffold44489_16.4	1	p2	(TG)6	12	240	251	TGCATTCATGTACTCAGCCC
scaffold44540_13.3	1	p2	(TA)6	12	393	404	CCATGGAGTCCAATCTACCG
scaffold44589_12.4	1	p2	(AT)9	18	313	330	TGCAACAAATATCTGCACACG
scaffold44611_10.8	1	p2	(AG)6	12	94	105	TTCTGTTCATCATCTTCGTCCT
scaffold44613_13.9	1	p2	(GA)8	16	724	739	TCAGCTTGCAAAAGAGAGGA
scaffold44759_10.9	1	p2	(CT)6	12	38	49	CACTCTCCCTCTTTCTCTCTCG
scaffold44771_13.8	1	p2	(CT)6	12	82	93	TCGAAATCGTAATAATCGGACA
scaffold44778_13.4	1	p2	(CT)7	14	169	182	CTTCATTTCCCTTCCCCTTC
scaffold45060_11.2	1	p2	(TC)8	16	54	69	TGTTCTCATTCGTTTGCTCTAA
scaffold45274_10.4	1	p2	(GT)6	12	56	67	TTGTCTGCGGTAATGCTTTG
scaffold45337_10.2	1	p2	(AG)6	12	512	523	GTTAAAATGTGTGCCGGCTT
scaffold45611_10.7	1	p2	(GA)8	16	54	69	TGGAGTTGGAGTTGAGAGGAG
scaffold45757_15.2	1	p2	(AT)6	12	208	219	GCCTTCAATTTTATACCCGAGA
scaffold45982_15.6	1	p2	(TC)6	12	444	455	CTTGGTAAGCGGAGAGAACG
scaffold46113_15.5	1	p2	(GT)6	12	220	231	GCCTAACGTGGCTCTGATGT
scaffold46130_12.1	1	p2	(CT)6	12	56	67	AGCCTTCTAGCATCTCTTGCTC
scaffold46265_13.7	1	p2	(TC)6	12	936	947	CATTCTCTGCCTGTGTCTGC
scaffold46398_13.1	1	p2	(CA)9	18	1121	1138	ACTGGTTTCGACAAAATGGC
scaffold46882_17.0	1	p2	(TG)6	12	1177	1188	TTCGCAAGAACAACGACAAC
scaffold47031_12.8	1	p2	(AT)10	20	306	325	TTGATAAATAGTGCAATCCCAAC
scaffold47072_15.7	1	p2	(AT)6	12	56	67	GATTGTCTAAAACGTAAAGCTTGATT
scaffold47105_15.4	1	p2	(TA)6	12	94	105	GGGACAAAAACCGAAGGAAT

scaffold47160_11.4	1	p2	(TC)6	12	1030	1041	CGAAATTCCACCGACTCAAC
scaffold47168_16.1	1	p2	(AG)6	12	232	243	ATCTTGGGGAATGTGTGCAT
scaffold47242_15.0	1	p2	(TA)7	14	3783	3796	TCCGTAGACCTTCCAACCAC
scaffold47262_12.7	1	p2	(TA)8	16	353	368	CATGGATATGTGGAAGCACG
scaffold47301_13.3	1	p2	(TC)7	14	2153	2166	GGTGCCCATTCTGTTCACTT
scaffold47469_14.1	1	p2	(TA)6	12	267	278	TGAAAGAAAGAGAAAAGGAATTTTT
scaffold47593_14.0	1	p2	(TG)7	14	364	377	CAGTGGATCGTTCTGCTTCA
scaffold47614_13.4	1	p2	(AT)11	22	766	787	TTGATTGCAATAAGCAAATTTTTA
scaffold47664_15.2	1	p2	(AT)6	12	603	614	TCGCGGATATCAATTATCAACA
scaffold47763_14.3	1	p2	(TA)8	16	824	839	TTCTGTAATCGAGTTCAAGAGAGAGA
scaffold47947_14.6	1	p2	(AT)6	12	145	156	GCAAGGTGAGGAAGGAAGAA
scaffold48158_13.7	1	p2	(GA)6	12	449	460	CGAGGAATGAAATCGATGGT
scaffold48249_13.1	1	p2	(TA)8	16	157	172	GCAAACCCGTGTTCTTCAAT
scaffold48356_14.3	1	p2	(GA)10	20	371	390	GCCTGCAGACAGACAAACAA
scaffold48387_14.9	1	p2	(TC)7	14	272	285	TGCCATGGATATGATTGTTGA
scaffold48402_20.5	1	p2	(AT)7	14	713	726	GCATAAATTCATCTGAACAAGGG
scaffold48491_13.6	1	p2	(TA)10	20	331	350	GGATGAACGTCGGTCGTATT
scaffold48501_12.6	1	p2	(AT)10	20	1202	1221	GAATGTCATTGTTCGGACCC
scaffold48671_14.0	1	p2	(GA)8	16	723	738	CCGGTTTTGTTGACGAACTT
scaffold48765_14.5	1	p2	(AG)7	14	286	299	AAGCAAAAGAGTCAGCCGAA
scaffold48824_12.3	1	p2	(CT)6	12	463	474	CCGCATGTCATGTTATTGGA
scaffold48828_15.0	1	p2	(TA)6	12	1142	1153	CAAAAATACCCCGAAAACCC
scaffold48859_10.0	1	p2	(AT)8	16	74	89	AATCTCCCACTTGGGCAAC
scaffold48975_17.7	1	p2	(AG)6	12	318	329	CCGGGTTCAAGTTCCTATCA
scaffold48987_14.8	1	p2	(TA)6	12	919	930	ATGCCAGTCTTGAGCCAGAT
scaffold49021_16.1	1	p2	(TA)11	22	1350	1371	GGTCTTTTACTTTTCAGACAACGA
scaffold49032_10.2	1	p2	(CT)7	14	1189	1202	GAGTCGAGCCGAGTAGTTGC
scaffold49325_14.3	1	p2	(AT)9	18	953	970	AAGTTCGAGCGGAGTTTCAA
scaffold49339_10.5	1	p2	(AT)10	20	334	353	GCATTCATGCAGCATTCAAC
scaffold49375_12.4	1	p2	(GA)7	14	874	887	GGGAGAGATGCGTCAGAAGA
scaffold49397_15.6	1	p2	(AG)6	12	1245	1256	CTTCCCAAAGTCATTTCCCA
scaffold49410_16.5	1	p2	(AG)6	12	2347	2358	CGAGGATCCGAGGATACAGA
scaffold49414_13.9	1	p2	(AT)7	14	164	177	GCCATATGTGGAAACCTTCCT
scaffold49582_14.7	1	p2	(AT)7	14	767	780	GTCACGCCCCGACTATCTTA
scaffold49596_10.9	1	p2	(TA)7	14	224	237	CCCAATTTGCCCTTATATGC
scaffold49789_17.1	1	p2	(CT)7	14	446	459	TCTGTTGTTGGCAGTTCTCG
scaffold49842_17.5	1	p2	(AT)6	12	664	675	TGGGGGTCTGTCAAAAATTC
scaffold49899_14.7	1	p2	(AT)6	12	821	832	TCGATTTCGTGGAGATTGAA
scaffold49910_11.0	1	p2	(CA)6	12	131	142	GTGAAACCCCCAATTGTGTC
scaffold49957_14.6	1	p2	(GT)7	14	2299	2312	GCGTTTGACATCCTCAACCT
scaffold50057_16.6	1	p2	(TC)6	12	589	600	ATGGTTCGCCATAAAAATGC
scaffold50117_10.2	1	p2	(AT)6	12	317	328	TGATAGTGGGTTGGGTGTCA
scaffold50215_14.7	1	p2	(TA)7	14	51	64	CCCACTTTTGGGTTATTACCTG
scaffold50337_17.1	1	p2	(TC)7	14	2396	2409	TACGTGGATTTGACAGTGGC
scaffold50406_14.4	1	p2	(GA)6	12	489	500	GAGAGAGGAAGAGGGATGGG
scaffold50603_15.1	1	p2	(CT)10	20	221	240	TGTCCAAGCAAAATTGACGA
scaffold50898_10.7	1	p2	(AG)7	14	53	66	AAAGCTCAAAATGAAGAGGGC
scaffold50982_12.0	1	p2	(CT)6	12	157	168	CGCGACACCTCTAGAACACA
scaffold51119_15.4	1	p2	(TG)6	12	1145	1156	GGGAGGAAATTTTGAGGCTT
scaffold51332_14.1	1	p2	(TA)10	20	1017	1036	TCATAGTTTTCATTTGGACACCTC
scaffold51539_13.8	1	p2	(CT)8	16	380	395	CATAAGCCTACGAGCCCAAA
scaffold51591_12.0	1	p2	(AT)7	14	178	191	CCGAAAACCAAACCGAATTA
scaffold51800_11.1	1	p2	(CA)6	12	49	60	TTTTGGAGAATTTTCTCTTGCTC
scaffold51817_15.4	1	p2	(AG)6	12	378	389	TGTTTGAGTTAGCAGCCAGG
scaffold51942_11.2	1	p2	(TA)9	18	655	672	ACAAATTTGCCCTCGTATGC
scaffold52146_11.1	1	p2	(CT)6	12	295	306	CTGCATGGAGACTTGCATCA
scaffold52335_14.7	1	p2	(AT)6	12	1456	1467	TGCATAAACCTGCATACCCA
scaffold52435_13.4	1	p2	(AC)7	14	382	395	AAAAATCGTGCATTTTTGGC

scaffold52523_10.0	1	p2	(AT)10	20	288	307	CAATATCCCAATGTTTGAATCG
scaffold52705_12.4	1	p2	(TA)8	16	260	275	TCACCAAAAGGAGGGGTTATC
scaffold52807_13.4	1	p2	(AT)7	14	814	827	TTGGGCCTGAATGGACTTTA
scaffold52845_19.2	1	p2	(TC)6	12	1114	1125	GGAGGAGATGGAGACAGTCG
scaffold52868_16.5	1	p2	(AG)6	12	131	142	ACGTCGTCTCAAAGGGTGTC
scaffold52894_16.0	1	p2	(AT)6	12	263	274	GCTGGATATTTGGGCTTGAA
scaffold53005_15.6	1	p2	(AG)7	14	110	123	AAATGCTCGCTCTCACACCT
scaffold53126_14.0	1	p2	(TA)9	18	845	862	CGCGTCCCTTTTTACATGAT
scaffold53272_15.5	1	p2	(CT)6	12	577	588	TCCAGACTCAACCGGGTACT
scaffold53274_11.5	1	p2	(TA)10	20	100	119	TCAGATCTGCGCTGAAAATAAA
scaffold53345_15.8	1	p2	(CT)6	12	1388	1399	CACATGCACACACGTGATTC
scaffold53462_12.0	1	p2	(AT)8	16	704	719	ATGGAAACAATCACATCGCA
scaffold53484_14.4	1	p2	(TC)8	16	2474	2489	CTTGGCCAGCTCTAGGAATG
scaffold53634_15.9	1	p2	(TC)8	16	432	447	TGCATGGTAGTTGGGTGGTA
scaffold53859_10.3	1	p2	(TA)9	18	181	198	ACACATTTTTCTTTTGGGCG
scaffold54193_11.0	1	p2	(AT)10	20	513	532	GACTTCGTGGCGGTATTCTC
scaffold54246_20.6	1	p2	(AC)6	12	99	110	TGTCTCCATTTGTGTGTGTGT
scaffold54270_17.0	1	p2	(AG)8	16	125	140	ACCGACCTCCACTTCAACAC
scaffold54349_10.7	1	p2	(AT)7	14	184	197	TGGCCTATGAAGGAAAATGC
scaffold54599_14.6	1	p2	(AT)6	12	514	525	TTGCTAACCAACTACGTTGAGA
scaffold54716_14.5	1	p2	(AG)7	14	3513	3526	AAATGGGCAGACATACTCGG
scaffold54783_15.3	1	p2	(AG)6	12	131	142	ACACGCCTTTATCCGATGAG
scaffold54940_10.3	1	p2	(AG)6	12	53	64	AACAATTACAATGTTTGTGAAATCAA
scaffold54960_10.7	1	p2	(AG)6	12	492	503	TGTACCTCTCGCTGCTCTGA
scaffold55381_17.5	1	p2	(GA)9	18	6557	6574	GCAATGGCAAACCATTACCT
scaffold55384_10.4	1	p2	(CG)6	12	86	97	GGGTCCAGTCAATGAAACGA
scaffold55500_14.1	1	p2	(AT)6	12	1384	1395	CGGTGAAGATAAATTGCACGTA
scaffold55533_15.9	1	p2	(GA)6	12	918	929	AACACAATGGCGGGAATAAG
scaffold55771_11.4	1	p2	(GA)6	12	67	78	GAAACACGCAACGCAAGTAG
scaffold55892_15.1	1	p2	(TA)9	18	312	329	CCATCCAGCGCTATAAAATCA
scaffold55951_15.6	1	p2	(TC)6	12	200	211	TGCCATTTTGAGACCAGACA
scaffold56133_17.7	1	p2	(AT)7	14	194	207	GGTTGTAATGAGGCTTTCGG
scaffold56254_10.5	1	p2	(TA)8	16	50	65	AAGGGGAGTATCACTTGAATGC
scaffold56284_15.4	1	p2	(GA)6	12	2560	2571	AGCTGGAAAAGGCTATGCAA
scaffold56303_13.8	1	p2	(GA)6	12	2106	2117	GGGCTACTTCCCAACACAAA
scaffold56673_16.3	1	p2	(AT)9	18	271	288	CAACACGAATGTGCTGATGA
scaffold56746_16.8	1	p2	(TG)7	14	869	882	CACGGGTCTTCATCCATTTT
scaffold56794_14.0	1	p2	(GA)6	12	576	587	GAGCAAGAGAAAATTCTCCCAA
scaffold56796_16.6	1	p2	(GT)8	16	1219	1234	TGAAAGTGCCAACTTACGGA
scaffold56807_12.9	1	p2	(GA)6	12	3585	3596	TTTTTGCCCCAATCCATTTA
scaffold56831_16.5	1	p2	(TC)8	16	986	1001	CCAACCTTGTCGGAACAGTC
scaffold56849_16.0	1	p2	(TG)8	16	513	528	TGTCCAGCACATCAAAGCTC
scaffold57139_14.2	1	p2	(TG)6	12	482	493	ATGCAGGTTAGCGACGTTTT
scaffold57167_13.7	1	p2	(AG)6	12	95	106	TTCTGTTCATCATCTTCGTCCT
scaffold57169_11.1	1	p2	(CT)6	12	253	264	TCTTTTCCAGCCTGCAACTT
scaffold57170_11.3	1	p2	(GA)7	14	109	122	CATCAACTCTGATGCGGAGA
scaffold57258_12.2	1	p2	(AT)7	14	109	122	TCTTATCTCATCAACCGCCA
scaffold57325_15.2	1	p2	(TC)6	12	152	163	CAATCCCAATTGTAGCCTGC
scaffold57488_11.2	1	p2	(GA)9	18	291	308	TGCTGAGACATAGCGGACAC
scaffold57547_16.3	1	p2	(AG)6	12	57	68	AAAGAAGAACTAAAGCTTCGGAAA
scaffold57611_11.2	1	p2	(CT)6	12	63	74	TGTGAACACAGCCTCACCTT
scaffold57798_17.3	1	p2	(AG)9	18	1909	1926	CAGCCATGGATCAGCAGTAA
scaffold57902_19.1	1	p2	(AT)6	12	109	120	TCAGAAAAGTTCGAGCGTGA
scaffold58112_16.5	1	p2	(AT)6	12	521	532	ATCACCAACTGGCGAGAAAT
scaffold58242_13.0	1	p2	(AT)7	14	55	68	TGGACGTGGAACAATTACGA
scaffold58399_10.7	1	p2	(GA)7	14	252	265	AAGGTGTAGATCTGGCGGTG
scaffold58401_15.6	1	p2	(CT)6	12	949	960	GGCTCAGATTTTCCCAATCA
scaffold58491_13.2	1	p2	(TA)8	16	108	123	TGGTGTAAAAAGGGGTTGATG

scaffold58561_10.2	1	p2	(AT)6	12	66	77	TAACGACACGAACGGTTTTG
scaffold58654_13.7	1	p2	(CT)8	16	95	110	TTTGCACAAATTCGTTTAGGC
scaffold58657_16.1	1	p2	(AT)6	12	3245	3256	CTTGCGATCGTTTCTGTCAC
scaffold58720_12.9	1	p2	(TA)6	12	241	252	TTTCTTTTGTCAATGGAGCATCT
scaffold58853_15.1	1	p2	(GA)6	12	418	429	TGCAATAACAAATTTGGGAAA
scaffold58861_15.5	1	p2	(TG)7	14	2513	2526	ATGAGGCTGCTTTGCAAGTT
scaffold58925_16.1	1	p2	(GA)8	16	361	376	AAATGCGAGAGTGGTACGCT
scaffold59114_16.1	1	p2	(AC)6	12	4337	4348	GATTTCCCAACCCCAGTTCT
scaffold59129_14.6	1	p2	(TC)10	20	3160	3179	CAACTTTCTGCCCATCCATT
scaffold59205_10.3	1	p2	(AG)6	12	111	122	TGACTAAGCACAAGAGACGAAGA
scaffold59218_16.9	1	p2	(TC)6	12	611	622	ACATACCCTGTTCCTCGTGC
scaffold59358_11.6	1	p2	(TA)7	14	271	284	ATACCCTTGCATGCACAACA
scaffold59589_13.1	1	p2	(CT)8	16	405	420	GATGCATCAAGTCGACAATCA
scaffold59673_16.0	1	p2	(AG)6	12	1258	1269	GATGTCCCCATTGAGACTCG
scaffold59807_17.8	1	p2	(TC)6	12	231	242	TAGAATCCGATGTATGGCCC
scaffold60005_12.4	1	p2	(TA)6	12	523	534	CACCTGAAGGCGAAAAAGAG
scaffold60017_15.9	1	p2	(GA)7	14	326	339	ACATACTCCACCAACTCGGC
scaffold60137_10.7	1	p2	(TA)7	14	45	58	TTTCCAAGTCACCTTCCATT
scaffold60211_16.2	1	p2	(CT)6	12	297	308	CCAAAGGGGCTGCAATACTA
scaffold60282_10.4	1	p2	(TA)8	16	48	63	ACCATATTTAAGGGTTATCACCTG
scaffold60545_10.7	1	p2	(AT)6	12	119	130	AGCACGAATCACACAGCAAC
scaffold60673_17.7	1	p2	(AG)6	12	195	206	TCCCCATGTATATTGCCGTT
scaffold61001_14.0	1	p2	(TC)10	20	51	70	ACTGAGCCCTGATTTGGAAG
scaffold61042_16.3	1	p2	(AG)7	14	1474	1487	ATTTGGTATCAGAGCCACGG
scaffold61066_14.9	1	p2	(AG)6	12	2747	2758	TCTTTCAACCTGAATTGCCC
scaffold61119_11.3	1	p2	(AT)6	12	811	822	AGCTATCAAGCACCGGAAAA
scaffold61168_16.1	1	p2	(AT)7	14	272	285	CGGGTTCCCACATTTAACAA
scaffold61186_14.6	1	p2	(GA)7	14	87	100	TTTTATCAAGCGCAACCTCC
scaffold61355_10.3	1	p2	(TA)8	16	157	172	TTGTCGTATGCTGTCCGAAA
scaffold61357_10.3	1	p2	(TG)9	18	808	825	GGGATAGTACCTGATCATCTCCA
scaffold61549_14.2	1	p2	(TA)11	22	1125	1146	GCAGCCTTCTTGTCTCCTGT
scaffold61642_11.3	1	p2	(AT)8	16	648	663	CCGCCTGATTTGTTTCTCAT
scaffold61826_13.1	1	p2	(TA)6	12	807	818	CAAAGCATAAACATCCCAGC
scaffold61843_13.7	1	p2	(GA)9	18	441	458	TGATGGCATTCATCGTCAGT
scaffold61977_12.5	1	p2	(TA)9	18	82	99	ACGCCCCAGATTGTTTCTTA
scaffold62095_18.0	1	p2	(AT)9	18	245	262	GGGGAGATGTTCCTTTAGTCG
scaffold62187_11.9	1	p2	(AT)8	16	315	330	ACTTATTTGCATTGGGCCAC
scaffold62244_12.1	1	p2	(TG)7	14	143	156	CATTCGTACCCCAAACTTGC
scaffold62265_14.2	1	p2	(TA)7	14	936	949	AAACTCGACTGCCCCTTTTT
scaffold62278_16.9	1	p2	(TC)6	12	432	443	CATCACTCTGAGCCTCTCCC
scaffold62317_10.1	1	p2	(CA)7	14	183	196	TCGTGCGGTCAATGATTTTA
scaffold62360_14.3	1	p2	(TC)6	12	153	164	GGATTCAGTTGCTTCAAGAGC
scaffold62374_17.7	1	p2	(CT)7	14	265	278	CAATATTACAATGGGCAAGAGAG
scaffold62410_15.1	1	p2	(TG)6	12	3061	3072	TGGAATGCTTCTTTCTTGGG
scaffold62431_14.7	1	p2	(TA)6	12	443	454	GATCGACGTCCCAGATTTGT
scaffold62474_12.3	1	p2	(TC)6	12	732	743	CTCGTCTCCTCCACTTCCTG
scaffold62639_13.4	1	p2	(AC)6	12	83	94	AAAGAACAAAATGACACGAAGAA
scaffold63015_14.5	1	p2	(AT)10	20	638	657	AATGGTTCGGTTCCAGTTTC
scaffold63163_14.9	1	p2	(AG)7	14	65	78	TTTTGTGATTTCCCATGTTTG
scaffold63191_13.9	1	p2	(GT)6	12	2778	2789	CTCTCAACACTTCAACGGGAG
scaffold63399_14.7	1	p2	(AT)9	18	405	422	CCCGGCAACTTCTCAAATAA
scaffold63424_15.7	1	p2	(TA)8	16	3194	3209	CTGCAACAGCATAGTGGCAT
scaffold63688_13.4	1	p2	(TA)6	12	504	515	AGGAAATTGGGGGAAAATTG
scaffold63740_11.3	1	p2	(AG)7	14	272	285	CTCTTCCCATTTTCCCAACA
scaffold63772_16.0	1	p2	(GA)12	24	7241	7264	TCTCTCTGCAGGGGAGTCAC
scaffold63886_10.5	1	p2	(TA)6	12	371	382	CGAAGGAGACTGGAACCGTA
scaffold63970_14.3	1	p2	(AT)8	16	442	457	GGGCCCCTAGTCTGTCTCTT
scaffold64133_13.7	1	p2	(TC)6	12	254	265	ACCTAAACCCAAAACCCCAC

scaffold64202_15.3	1	p2	(AT)8	16	161	176	TGTCAACATTCAATCACAGCC
scaffold64267_10.4	1	p2	(AT)9	18	204	221	TCCCACCACTGGTTTCTTTC
scaffold64348_14.7	1	p2	(TA)7	14	391	404	TGAATCCCATCGATCCATTT
scaffold64461_13.7	1	p2	(AG)7	14	1082	1095	GGCTCTTCTCTGTCCGCTAA
scaffold64467_17.4	1	p2	(AG)6	12	2396	2407	GGTCACAGAGGTCGTCCCTA
scaffold64917_14.6	1	p2	(TA)6	12	1852	1863	GCTTGGTTTGCAGTCTCCTC
scaffold65164_13.0	1	p2	(AT)6	12	192	203	TTCTGAACGTGCAACTCGTC
scaffold65248_18.0	1	p2	(AG)6	12	806	817	TCCCTTACCAACATTCACGA
scaffold65405_10.6	1	p2	(AT)7	14	65	78	TCAAAAGCAAGAAAATGTGATTTC
scaffold65470_13.6	1	p2	(AG)6	12	1578	1589	CCACAAGCATTGCAGAGAGA
scaffold65495_10.5	1	p2	(AG)6	12	207	218	GGGGTTAATGATTGGTGTCG
scaffold65564_11.2	1	p2	(TC)6	12	23	34	ATACCACTCGTTAATCCGGC
scaffold65574_16.3	1	p2	(AG)6	12	3495	3506	AGAGGGAGTTTCCGGAGAGA
scaffold65640_17.7	1	p2	(TA)6	12	425	436	TGGTTCAATGGGTTATTCGAC
scaffold65643_19.0	1	p2	(TA)9	18	107	124	GTGCATCAACTTATGCCAAA
scaffold65647_12.9	1	p2	(AT)10	20	105	124	TGAAGTTAGGAAATAAGGAGGTGTT
scaffold65648_11.0	1	p2	(AT)9	18	564	581	ACGCGATTTCACTCACAACA
scaffold65649_15.9	1	p2	(AT)7	14	111	124	ATGATGGGCACATGGATTTT
scaffold65650_13.3	1	p2	(AT)7	14	174	187	GGTGTTGGATGGAAAATGCT
scaffold65651_15.9	1	p2	(AT)6	12	113	124	CGCTTTGGGACAGCAATTAG
scaffold65760_10.7	1	p2	(CA)7	14	379	392	ACTCCATCGATATTCACCGC
scaffold65783_10.2	1	p2	(AG)6	12	1582	1593	ATCCGCCATTGTTGAAACTC
scaffold65975_15.9	1	p2	(TC)12	24	43	66	CTTAGGCCACCACAAAGGAA
scaffold65986_16.2	1	p2	(TC)6	12	590	601	TTTCCCTCTCCTCCTCCATT
scaffold66008_15.3	1	p2	(GA)8	16	1590	1605	GACCAATTCGGTTTGGTGAC
scaffold66195_16.6	1	p2	(TA)6	12	2846	2857	CACGTGTCATCTTCTTCAGCA
scaffold66342_17.4	1	p2	(GA)6	12	837	848	GGTGGTGCAATGGGTAAAAC
scaffold66411_11.7	1	p2	(AT)7	14	139	152	ATTTTCAATCTCCGGGCTCT
scaffold66442_15.5	1	p2	(TA)6	12	1324	1335	CTTGGACCAGATTGTCCCTC
scaffold66569_11.9	1	p2	(GA)7	14	470	483	GCTGGAGCAGGTCTCTTGTC
scaffold66707_14.1	1	p2	(TA)6	12	181	192	GGGGAAGTGTGATGTTTGGT
scaffold66711_14.1	1	p2	(AT)9	18	467	484	AAGTAGGGTTGGGGTGCATA
scaffold66715_13.9	1	p2	(TA)9	18	1017	1034	GGGTACGGGTTGAGACAAAA
scaffold66757_11.4	1	p2	(TA)13	26	420	445	TGAAAATAAAAATGGAGTGATGTGA
scaffold66776_11.3	1	p2	(AG)7	14	147	160	TTTTACGAGATGGAAGAAGCTG
scaffold66851_10.1	1	p2	(TA)10	20	63	82	GCGAAATTCATGTTGACCCT
scaffold66911_10.3	1	p2	(AT)8	16	447	462	GAAATGCAAGGATTTCCCAA
scaffold66914_12.1	1	p2	(TA)7	14	93	106	GGCATTGCTAGAGCCATTGA
scaffold66961_21.4	1	p2	(AT)10	20	164	183	CCCTCGGATGCTAGTGTTTC
scaffold67058_10.3	1	p2	(TA)8	16	390	405	ATGCTAACTCCCCTTCCCAT
scaffold67350_12.3	1	p2	(AT)8	16	127	142	CCTCCGAACTCATGGTGTTT
scaffold67562_10.8	1	p2	(TA)10	20	693	712	GATGGGAATGGGAGGTATGA
scaffold67614_11.4	1	p2	(AG)7	14	113	126	AAAAGAGAGGGATGGGGAGA
scaffold67678_17.9	1	p2	(AG)6	12	1919	1930	TTGTGGCAGTCAGTGAGGAG
scaffold67698_13.4	1	p2	(GA)8	16	523	538	AGGATTCCAGCCACATGAAC
scaffold67727_11.1	1	p2	(AT)8	16	341	356	TGGGCTAATCATTCTCAGCA
scaffold67751_15.6	1	p2	(AG)7	14	2186	2199	GTCCTCTTTCTTCGCCTCCT
scaffold68014_16.6	1	p2	(GA)6	12	791	802	GTTCCGAATGCGAGTTTGTT
scaffold68050_11.4	1	p2	(TA)9	18	84	101	ATCAACCGCCTGATTTGTTC
scaffold68147_12.1	1	p2	(AG)6	12	141	152	GAATGAGTTTGCCGTGTTGA
scaffold68232_10.3	1	p2	(TA)6	12	86	97	TTGGTGCGATATTTTGTTCG
scaffold68284_15.2	1	p2	(AG)7	14	283	296	CAATCTCAACCCAAACCCAT
scaffold68324_10.7	1	p2	(AC)7	14	38	51	GAAAAGCTTGGCCATTCAAC
scaffold68365_16.8	1	p2	(TC)7	14	1529	1542	AGCTATGATGCTGATGCGTG
scaffold68439_13.4	1	p2	(GA)8	16	374	389	GTTTTTCCACCGAATCAAGC
scaffold68509_16.6	1	p2	(CT)6	12	529	540	TGCTGCTAATTATCCCTCCAA
scaffold68725_11.5	1	p2	(CT)8	16	178	193	ATGATTGGCTAGACATCGCC
scaffold68785_14.4	1	p2	(AT)6	12	58	69	TTCATCCATTTTGCACCTGA

scaffold68837_16.2	1	p2	(GA)6	12	206	217	ATTTTGATCCTTGTGGCCTG
scaffold68883_15.6	1	p2	(AG)7	14	675	688	GATGGTAGAAGAAGCGCAGC
scaffold68889_13.6	1	p2	(AT)9	18	697	714	TGGATGGCTAAGACAACAACA
scaffold68919_15.6	1	p2	(AG)6	12	221	232	CACGTCAAATCCTCCCAAAT
scaffold68965_13.2	1	p2	(TA)6	12	1542	1553	CTACAGCTCTATCCCGTCGC
scaffold69138_13.4	1	p2	(TC)6	12	67	78	ATTTGCAAGTTCCAGCGATT
scaffold69210_18.3	1	p2	(GA)6	12	1063	1074	GTCGAGGCGTAAGTTTGAGC
scaffold69241_13.5	1	p2	(AG)8	16	109	124	AGCCAGGGCTTTTATCATGT
scaffold69326_15.6	1	p2	(TC)6	12	2664	2675	ATCAGGAGACAACGGAGTGG
scaffold69338_14.5	1	p2	(CT)6	12	112	123	GCTTCATAGCATCTCTTGCTCA
scaffold69450_10.7	1	p2	(GA)6	12	134	145	GGTAAGCAGACTGCCAGGAC
scaffold69456_16.5	1	p2	(TA)7	14	2708	2721	TGTGGCTGAATTTGACGATG
scaffold69613_14.8	1	p2	(TA)7	14	49	62	GCACACAAGTTATTGGTTTCTATTTG
scaffold69649_14.2	1	p2	(GA)7	14	873	886	ACGGGCGATAGTAAGAGGGT
scaffold69713_10.2	1	p2	(TA)9	18	194	211	ATTTCGAAGATCAACGGACG
scaffold69879_10.8	1	p2	(TA)8	16	109	124	AATTGAGCCTTTACAAACTCTCTACA
scaffold70086_15.6	1	p2	(TC)6	12	2737	2748	GAGTTACCGCTTGCTCCTTG
scaffold70201_13.9	1	p2	(AT)10	20	246	265	TGCGATGTCTATGTGACGGT
scaffold70478_13.3	1	p2	(GA)6	12	208	219	AAGTCGGACTCTTGTTCGGA
scaffold70516_10.1	1	p2	(GA)6	12	199	210	TTCATCCTCCCTCATTTTCG
scaffold70547_16.7	1	p2	(CT)6	12	1487	1498	GGTGTCTACACGGTCCTCCT
scaffold70648_17.1	1	p2	(GA)8	16	698	713	AGAAAGGTGGGCATGAATTG
scaffold70700_15.4	1	p2	(AT)10	20	1874	1893	ACATCCATCTCAAAACAATATCAA
scaffold70754_15.3	1	p2	(AG)7	14	3082	3095	GCAGTTTGGCATCAGAGTCA
scaffold70765_14.9	1	p2	(GA)7	14	1269	1282	TGTGACAGAGCTGAAATGGG
scaffold70908_12.0	1	p2	(TC)6	12	326	337	GTTTCGTTTTGTTGCGGATT
scaffold71009_11.3	1	p2	(CT)6	12	833	844	CAACTGCTTCCGCATCTGTA
scaffold71097_11.0	1	p2	(TC)7	14	126	139	TTGCTTCAATGCAAGCTGTC
scaffold71188_15.8	1	p2	(AT)7	14	2060	2073	CCCCCTAACATTTTCCCACT
scaffold71421_10.6	1	p2	(TC)8	16	238	253	CAGCCCGGTACAGAAGGTAA
scaffold71441_17.3	1	p2	(AG)6	12	390	401	TGCAAAGGCTGAAGATCTGA
scaffold71536_14.5	1	p2	(TA)6	12	837	848	TTGTTTTCTCTTTTGCATAATCAAT
scaffold71577_13.5	1	p2	(GA)7	14	293	306	CGTGCTTGGCTTGGAGTAGT
scaffold71803_14.6	1	p2	(GT)6	12	474	485	TATGAGCCCGCACATTATCA
scaffold71837_15.2	1	p2	(TA)6	12	1309	1320	TTTTGTGCCTTCATTATGCC
scaffold71942_17.3	1	p2	(TA)8	16	989	1004	TTGGAAGGTATGTGAAATTTGG
scaffold71983_11.7	1	p2	(AT)6	12	172	183	ATCTTGGCAAGGCTCTGAAA
scaffold72090_10.4	1	p2	(TA)7	14	111	124	TCTGTTACTCTATCTGCATGCTCTG
scaffold72103_12.0	1	p2	(AT)6	12	301	312	AAAGGGAGTTGCAGTCAGGA
scaffold72139_15.3	1	p2	(CT)6	12	240	251	ACTTCTCCCCCTCTCTCTCG
scaffold72250_15.6	1	p2	(CT)6	12	65	76	CATACTCACGCATTCCCAGA
scaffold72400_10.0	1	p2	(TA)9	18	150	167	AAAGCCTACCTTGCTTCCGT
scaffold72443_13.1	1	p2	(CT)6	12	222	233	AGGATATCCCACGCCTCTTT
scaffold72497_12.9	1	p2	(TA)9	18	292	309	GACCGAACCGAAACTGGTAA
scaffold72610_14.2	1	p2	(TA)10	20	79	98	ATTTTGCAAGATCCAAGGGA
scaffold72933_12.5	1	p2	(AT)8	16	504	519	CAAACATTTATCTTGCACTTGG
scaffold72943_20.0	1	p2	(GA)6	12	113	124	ACAGCAGGGTGTTTGTGTGA
scaffold73058_16.7	1	p2	(AT)9	18	949	966	ACTCGCGTCTCTCATCCATT
scaffold73241_12.4	1	p2	(TA)8	16	786	801	GGGAGTATCACTGGAATGCG
scaffold73261_15.6	1	p2	(GA)7	14	407	420	TAACGACGCATAAAGAGGCA
scaffold73330_13.1	1	p2	(TA)7	14	314	327	CAGTCGCTGTTTCACCATTG
scaffold73359_14.6	1	p2	(AG)8	16	537	552	CCGCCATTAACTGCCATAGT
scaffold73547_18.4	1	p2	(TC)6	12	178	189	AAGGTTCACCAAACTACGCAA
scaffold73577_15.8	1	p2	(AG)9	18	433	450	CGTGGTTCAGCCATTTTGAT
scaffold73627_15.8	1	p2	(CT)7	14	1062	1075	AAAGAAGCGCGCTAAATCAG
scaffold73645_10.6	1	p2	(GA)6	12	82	93	GAACCAGAGGAGAAATAGGGAGA
scaffold73653_13.7	1	p2	(TA)11	22	182	203	TGACCACTTTTTACGGATTTGA
scaffold73752_15.2	1	p2	(CT)7	14	629	642	TTCTTTTGAACCAGGCAACC

scaffold73766_15.0	1	p2	(CT)7	14	3631	3644	CCAATCATGCTGGTTTTTCA
scaffold73768_16.2	1	p2	(GT)9	18	694	711	ATGCAACCAGATTCAAAGGG
scaffold73777_13.6	1	p2	(AT)9	18	556	573	AAATTGCAAAACCCAACTAAGC
scaffold74002_13.1	1	p2	(AT)6	12	185	196	AAGGTTTGGCTTGACCTTGA
scaffold74026_10.3	1	p2	(TC)8	16	182	197	GGAAATGACACCCAAACCAC
scaffold74468_12.1	1	p2	(TA)7	14	179	192	TCTCCACTCTCTTCTCTCTCCC
scaffold74505_17.1	1	p2	(AT)7	14	52	65	ACCATATTTAAGGGTTATCACCTG
scaffold74655_15.5	1	p2	(TA)7	14	223	236	TAACTCAAAATCTTGCCCCG
scaffold74691_15.7	1	p2	(AT)8	16	362	377	ATGCACGCCCTTAATATTGC
scaffold74821_12.4	1	p2	(TA)10	20	418	437	CAAGCCATGAGCCTTTGATT
scaffold74999_11.6	1	p2	(TA)8	16	174	189	AAAAATGGGGGAAATTTGATT
scaffold75004_11.1	1	p2	(AT)7	14	1663	1676	AAACCGTCCAAAATCGTATCC
scaffold75110_14.4	1	p2	(GA)6	12	1538	1549	TAGTGGCCATTCTTCAGGCT
scaffold75161_14.5	1	p2	(AT)8	16	1743	1758	CGTTTCGCAAATTAATCGGT
scaffold75322_12.4	1	p2	(GA)6	12	99	110	GTTAATGTGGATTGTGGGGG
scaffold75331_13.8	1	p2	(AT)6	12	1185	1196	CAATGAAACCAAACCATCCA
scaffold75332_16.5	1	p2	(TC)9	18	5652	5669	ATATGCCAATTTGGTTCCGA
scaffold75351_10.9	1	p2	(TA)9	18	349	366	TGCACTTTAAGAGGTGGTTGC
scaffold75530_10.9	1	p2	(TA)10	20	212	231	TCGAAAATCAAAGGCACACA
scaffold75602_15.4	1	p2	(AG)11	22	4802	4823	ATCGGAACGCTCATCAAATC
scaffold75683_15.9	1	p2	(AG)6	12	835	846	GCATTTGTCCCGTATGGCTA
scaffold75870_16.1	1	p2	(AC)6	12	1135	1146	CCCTCTTGATGGATTTTTGA
scaffold75920_13.8	1	p2	(CT)9	18	1794	1811	TTGGCAGACTACTTTGTGGG
scaffold75965_12.1	1	p2	(AT)9	18	619	636	CATTTATTTCTGCCCCTCCA
scaffold76005_15.8	1	p2	(TA)8	16	383	398	TGACATTAGACCTACCAGCACG
scaffold76059_16.4	1	p2	(TA)8	16	729	744	TCGCTGGTTTTGTAGTCGTG
scaffold76061_12.2	1	p2	(TC)6	12	31	42	ATTCTGTCGGATAGGTGCGT
scaffold76151_14.4	1	p2	(AG)6	12	1746	1757	TCATTTGGTTTGCAGTCAGC
scaffold76276_18.4	1	p2	(TA)11	22	42	63	GCGTTTTGACGATATATAGTGTTTTG
scaffold76979_11.3	1	p2	(TA)11	22	316	337	AATCAATCTTTGAAACCGGC
scaffold77023_14.2	1	p2	(TA)9	18	380	397	CTCTAACAAATGCCAGCCGT
scaffold77310_12.3	1	p2	(CT)6	12	297	308	TTTAACTAGCGTCGTTGCCC
scaffold77357_12.1	1	p2	(CA)7	14	197	210	GCTTCACTCACTCACGCAGA
scaffold77360_14.8	1	p2	(CT)6	12	2075	2086	CACTGCTCTCTTTTCCTCGG
scaffold77455_15.9	1	p2	(AG)6	12	312	323	TGATTTCGATGGTTTTGGGT
scaffold77486_15.2	1	p2	(CT)8	16	856	871	GGCACACCTGTATACCCCAC
scaffold77561_15.4	1	p2	(TG)6	12	1481	1492	TTGATGCACCCTCTTTTTCC
scaffold77651_14.8	1	p2	(TA)8	16	241	256	AAAATCGACCGCACATTTTA
scaffold77708_13.1	1	p2	(TC)6	12	708	719	CCCTAACTCGACCCAGATCA
scaffold77732_13.0	1	p2	(TA)6	12	170	181	CGTTGTCCTGAGCACGTTAG
scaffold77796_12.0	1	p2	(AT)6	12	635	646	ATCGTGGGAGAGAGAGATCG
scaffold78146_15.2	1	p2	(TA)6	12	805	816	TTCAATCATCTCAAGGCGAC
scaffold78188_14.5	1	p2	(GA)6	12	52	63	TGATGAAGTTGGGTGTTGGA
scaffold78227_10.1	1	p2	(AT)7	14	136	149	CCTAAAATTGAACGAAACTCGAA
scaffold78392_10.0	1	p2	(AC)8	16	276	291	TAGCCCCTTTGAGACCATTG
scaffold78396_13.6	1	p2	(AG)6	12	661	672	TCCAGTGTTGCTCTTGCATC
scaffold78453_13.0	1	p2	(TG)6	12	349	360	TTCCGATGGAATGCATTTTT
scaffold78461_10.9	1	p2	(TA)7	14	211	224	AGGCACATGCCAAACTTTTC
scaffold78530_17.2	1	p2	(TC)6	12	139	150	TCCCAGTTCAACTCTTTCTCG
scaffold78563_12.0	1	p2	(AG)6	12	345	356	GTGTGGGTTCCTCTCGTGTT
scaffold78622_17.0	1	p2	(TC)6	12	357	368	TTCCACTAGGGGTACCAAAATG
scaffold78859_10.5	1	p2	(AG)6	12	102	113	AAAGCCAATCGAGTCTTGGA
scaffold78947_15.0	1	p2	(GA)8	16	85	100	GGATGGGGCTGTGATTGTAG
scaffold79075_14.2	1	p2	(AT)6	12	298	309	CAATTCCGAACCGAATAAGC
scaffold79112_10.0	1	p2	(AT)10	20	45	64	CGAATAGAGTGATGAGCCAAC
scaffold79127_20.0	1	p2	(TC)6	12	96	107	GCAACGGAATACCACTCGTT
scaffold79379_15.8	1	p2	(CT)6	12	2695	2706	ACACCATACCCAAAAGCCTG
scaffold79413_19.3	1	p2	(TC)6	12	556	567	TCTCAATCATGCGATCCAAA

scaffold79543_13.0	1	p2	(AT)9	18	323	340	GGAAGCGCAATTAATACGGA
scaffold79547_20.3	1	p2	(TA)8	16	158	173	ACGATTTTGCCCTTGTATGC
scaffold79728_13.6	1	p2	(TA)6	12	185	196	CACGATAAGGGATGGATTCAC
scaffold79739_13.7	1	p2	(AG)6	12	1235	1246	ACGAGGGAGTGATCAAGTGG
scaffold80293_13.2	1	p2	(GT)8	16	166	181	TGTTCAAATGATTTTGGGGG
scaffold80295_13.1	1	p2	(AC)6	12	143	154	ACTCTCAGCCACGCACTCTC
scaffold80667_14.1	1	p2	(TA)7	14	1762	1775	CGCGCCTTGCCTAATAATAA
scaffold80688_13.0	1	p2	(AT)6	12	244	255	TAATCGTTGCTGGAGCGTC
scaffold80703_16.5	1	p2	(AT)6	12	153	164	ACAGCCATCGCTCAACCTAC
scaffold80711_14.1	1	p2	(TA)9	18	284	301	TCATGGGATCAGTTTCAATCC
scaffold80726_11.0	1	p2	(AG)7	14	247	260	ACTGTGCTGCTGGAAAATTG
scaffold80753_12.9	1	p2	(CT)7	14	80	93	ACAAATGACCAAGGAGGAGTT
scaffold80774_12.7	1	p2	(TA)6	12	201	212	TGGATTGTATGTGTCCCCAG
scaffold80812_13.9	1	p2	(TA)9	18	190	207	GTTATGTGGCCCGAATGACT
scaffold81113_22.3	1	p2	(AG)7	14	50	63	TCCCATGTTTGTAATTATTGTAGGG
scaffold81146_18.6	1	p2	(CT)7	14	3436	3449	CAGGGACGGGCATGTTACTA
scaffold81182_18.9	1	p2	(TA)6	12	593	604	TTCACGACCCTGTATGTCCA
scaffold81228_11.2	1	p2	(TA)6	12	494	505	CCTTATTTGATGCACGGACC
scaffold81275_18.3	1	p2	(GA)6	12	476	487	AGAAGTCTGCGAAGGTCAGC
scaffold81313_15.2	1	p2	(AG)8	16	272	287	GCCAACTATGAGACATGCGA
scaffold81406_13.5	1	p2	(TA)7	14	484	497	GCGTGACGCACAGTTTCTTA
scaffold81510_16.9	1	p2	(TA)7	14	844	857	CTGATGTCAATTTCCATATCCA
scaffold81530_18.7	1	p2	(TC)6	12	964	975	ATTCAGGCATATGCTTTGGC
scaffold81719_15.3	1	p2	(GA)6	12	4130	4141	GGGTTTGGTTGGAGACGTAA
scaffold81773_10.9	1	p2	(TC)6	12	215	226	GAGTTCGTCAAACCTCCCAA
scaffold81873_14.1	1	p2	(TC)6	12	39	50	GGCAACGGAATACCATTTGT
scaffold81933_15.9	1	p2	(AC)6	12	1686	1697	AGATGCTCCCTCTCCCATCT
scaffold82011_15.6	1	p2	(AG)7	14	2392	2405	TCCAAGGGAAAGTGTTCAAGA
scaffold82102_14.1	1	p2	(TC)6	12	1070	1081	AAGGAGGGGCAAAGAAAAGA
scaffold82158_17.4	1	p2	(TC)6	12	113	124	TCAAACACTAGCCTTTGCCTC
scaffold82302_16.3	1	p2	(CT)6	12	89	100	AACAAACACCTGGGACCACT
scaffold82381_15.2	1	p2	(AT)6	12	343	354	TGAAGAGTACAACTTACACCCCA
scaffold82405_14.2	1	p2	(AC)10	20	472	491	CGAAAGCTGAAGAGCGAGAT
scaffold82466_10.0	1	p2	(AT)6	12	560	571	GACTACTGGTCGGGGAACAA
scaffold82718_17.8	1	p2	(CT)6	12	56	67	CATGTGGCCTAACTTGACACA
scaffold82736_14.0	1	p2	(CT)7	14	381	394	ATCAAACGCTTTGTTTTCCG
scaffold82741_10.0	1	p2	(AT)6	12	422	433	AATTCCATGCACAGTGACCA
scaffold82925_14.9	1	p2	(AG)8	16	284	299	CAATCTCAACCCAAACCCAT
scaffold83013_17.1	1	p2	(CT)7	14	80	93	GTTCTCACCGGAGTTGGTTC
scaffold83070_11.6	1	p2	(TA)7	14	185	198	TCCCACTAAAATTTTCCCGTT
scaffold83254_16.4	1	p2	(AC)6	12	1418	1429	TTGATGCTGCAAGAAGCCTA
scaffold83389_10.0	1	p2	(AG)6	12	139	150	GCATGCTAGTGCCTGTTGTG
scaffold83423_13.2	1	p2	(AG)6	12	931	942	TCAAAAGAAGAGGCTGGCAT
scaffold83586_14.3	1	p2	(AG)8	16	380	395	AGGAAGGAGGCAGAGAGGAC
scaffold83612_16.1	1	p2	(TA)6	12	308	319	TGAAATGGTGATTTGAGGGG
scaffold83641_13.7	1	p2	(GT)9	18	695	712	AATGGATCTATAGAGCGCCG
scaffold83653_16.9	1	p2	(TA)6	12	219	230	GCCTAGTGAGGAGTTCACGG
scaffold83790_14.6	1	p2	(AT)10	20	2162	2181	TCTCAAAACCACAACAAACCC
scaffold84019_13.5	1	p2	(AT)7	14	544	557	CGACTACTCAATCATCCAACCA
scaffold84188_14.7	1	p2	(GA)6	12	680	691	TCCAAGATTGAAGTCGCAGA
scaffold84201_13.9	1	p2	(TA)10	20	191	210	CTCCCTCAATCAAACCTTCG
scaffold84225_13.9	1	p2	(AC)11	22	2421	2442	CCGTGGTTGATCCAGAGAAT
scaffold84249_17.6	1	p2	(TA)6	12	80	91	GGGGAAAATTTGCAAGATGA
scaffold84367_16.1	1	p2	(AG)10	20	4252	4271	CGTCCGTGTTCTATTGCAGA
scaffold84561_12.1	1	p2	(AT)7	14	443	456	TCATCTGCTGAAAGGAGGGT
scaffold84577_10.1	1	p2	(TC)6	12	100	111	ATCGGCGAATTTATCTGCAA
scaffold84595_11.8	1	p2	(AT)11	22	699	720	CAGGTGCAGGGAAGAAATGT
scaffold84671_16.8	1	p2	(GA)7	14	1871	1884	CAACCAGACCCCTACCAAGA

scaffold84675_10.7	1	p2	(TA)8	16	72	87	ATGGGAAGAGGGGGTATGAG
scaffold85082_13.5	1	p2	(AC)6	12	401	412	TAGAGCCAACCAAATGGTCC
scaffold85248_14.4	1	p2	(TA)9	18	237	254	TTAGGGTTTAAAATCGCGCA
scaffold85249_10.0	1	p2	(TA)8	16	131	146	CGTTGAGCCGAATGTGTTTA
scaffold85335_13.8	1	p2	(AG)6	12	1079	1090	ATTCACGTCCACGTTTCTCC
scaffold85607_14.8	1	p2	(AG)6	12	45	56	CCTTTCCTAACAGTTTGGCATC
scaffold85613_14.5	1	p2	(AG)7	14	112	125	CGCCATTCTCTCTCCACTCT
scaffold85756_13.2	1	p2	(AT)6	12	286	297	GCGTGTTCTTGGTGTTCTTG
scaffold86034_10.8	1	p2	(GA)10	20	238	257	AATCGCAAATCACACCATGA
scaffold86089_11.0	1	p2	(CT)9	18	129	146	TTGCTTCAATGCAAGCTGTC
scaffold86147_14.0	1	p2	(GA)8	16	1072	1087	TGACTCGCCGTAGAATCCTT
scaffold86450_17.3	1	p2	(TG)7	14	2655	2668	AGGCCTCTCACATCTTCACC
scaffold86573_11.2	1	p2	(AG)7	14	190	203	GATATTGACGGTTGGTTGGC
scaffold86741_16.1	1	p2	(TA)6	12	94	105	AATGAACCAAGTCATTGCAGC
scaffold86750_10.6	1	p2	(AT)6	12	169	180	AGTGAAGCCAAGCCAAAAGA
scaffold86829_14.3	1	p2	(TA)7	14	553	566	AGGTTGGAGGGAATGGAGAG
scaffold86867_10.4	1	p2	(AT)11	22	55	76	AAATACCATTTCATTTTGTAAACTCTT
scaffold86965_15.3	1	p2	(CT)8	16	3590	3605	AAAAACATGGAGCAACGACA
scaffold87030_13.0	1	p2	(GA)6	12	35	46	CACGAGTGAAGAAAGGGAGAG
scaffold87093_14.4	1	p2	(TC)6	12	913	924	TTGATTGATCGACAGCATCC
scaffold87127_17.6	1	p2	(AC)6	12	1051	1062	TGGGATTAATTTCCACCATGA
scaffold87146_10.6	1	p2	(TC)6	12	60	71	TCAAATCATAGTCTTTCCTCGTTTC
scaffold87264_15.1	1	p2	(TA)9	18	47	64	TGAAGTTAGGAAATAAGGAGGTGTT
scaffold87358_13.9	1	p2	(TC)6	12	866	877	TCCCACAAATCGCATAAACA
scaffold87503_11.0	1	p2	(GT)9	18	214	231	GAAATTTCGCTAAGTCGAGAATAA
scaffold87510_15.6	1	p2	(CT)9	18	2258	2275	AGGGACAGCTGTGGACTCTG
scaffold87547_12.3	1	p2	(TA)6	12	365	376	TTGTCGTGGTTCGGTATTCA
scaffold87549_19.0	1	p2	(TG)7	14	181	194	GCATTTATGTGTCAGCCCCT
scaffold87635_17.8	1	p2	(CT)6	12	365	376	TGGTGAACGTGTTTTGTTGC
scaffold87819_17.1	1	p2	(CT)7	14	148	161	TTTGTTGGCTAACAGGTCCC
scaffold87836_15.3	1	p2	(AG)6	12	232	243	TGTTCGTTGGTGGAGTTGAG
scaffold87992_12.1	1	p2	(AT)6	12	138	149	CATCCTAAAGATAAGAATGGTAATCA
scaffold88024_14.1	1	p2	(TC)7	14	294	307	GGCAAACATGAATTCGTTCC
scaffold88025_14.0	1	p2	(GA)6	12	114	125	TGGGAGAAGTTGGTGAATTTG
scaffold88033_10.2	1	p2	(TA)6	12	250	261	CTTTCTCCCCCGTAAATTCC
scaffold88052_12.9	1	p2	(TA)10	20	99	118	GCCCTTGATTTCCAAGATCC
scaffold88060_10.7	1	p2	(TC)6	12	111	122	CCTCCATCCTTACTCCACCA
scaffold88373_19.1	1	p2	(TA)6	12	334	345	TGAGTTTTGAATTCGGACCC
scaffold88663_15.0	1	p2	(AG)6	12	619	630	AGACAGCAATACCAGGGGTG
scaffold88725_13.9	1	p2	(GA)7	14	2448	2461	GAAGGTAACGTCGCTGAAGG
scaffold88735_15.0	1	p2	(AG)6	12	449	460	ACCGACTTCTGCACCATTTC
scaffold88775_12.0	1	p2	(TA)8	16	158	173	TCGGTTCTTGCTCCATTACC
scaffold88848_10.1	1	p2	(TC)8	16	119	134	TTTTTCTTTCCACAAACCCC
scaffold88855_15.6	1	p2	(TC)6	12	114	125	CAGGAAGGGAAAAGCATCTG
scaffold88921_11.0	1	p2	(TG)8	16	647	662	GGTAGGAAATTTTTGGCTAAGTGA
scaffold88926_15.0	1	p2	(AC)6	12	2077	2088	GGGTTTGAAATTTTGAATGCTT
scaffold88990_16.1	1	p2	(TA)8	16	574	589	CCTTGAAAATCTGAGCGTCC
scaffold89043_10.5	1	p2	(TA)7	14	329	342	GATTTGGCTAGGAATGGCAA
scaffold89126_17.5	1	p2	(GA)8	16	308	323	TCGTCCGAACACATTGAAGA
scaffold89226_16.9	1	p2	(CT)7	14	109	122	GTACTCGGACGGGATCTTGA
scaffold89596_15.0	1	p2	(TC)9	18	2078	2095	TAGGGCAGATTTATGCCAGG
scaffold89612_12.1	1	p2	(TC)6	12	190	201	CACGTCTCGTGTTTCTTCCA
scaffold89630_13.7	1	p2	(GA)6	12	67	78	CGAGTGGTTGTGAGGTGAGT
scaffold89631_11.9	1	p2	(TC)6	12	529	540	TCGCACAAATGATAGCGAAT
scaffold89673_14.1	1	p2	(TA)6	12	1348	1359	TGAACCACCTCTGCAAACAA
scaffold89677_12.5	1	p2	(TC)9	18	249	266	TGCATAAGACACCTAGCCCA
scaffold89747_16.3	1	p2	(GT)6	12	368	379	AGCAATATTGGTGGGACGAG
scaffold89787_12.3	1	p2	(TC)6	12	770	781	TCATCTCCATCCCCCAATAA

scaffold89854_12.9	1	p2	(AT)7	14	564	577	CCTGGGTTTAAAAATGCGAA
scaffold90114_10.9	1	p2	(TA)7	14	461	474	TGCAAATCTGGATCCTTCGT
scaffold90191_11.0	1	p2	(TA)9	18	258	275	AAACTTGAGCAGTCAGTTCGATT
scaffold90198_11.7	1	p2	(GA)6	12	205	216	TCTCCACCAGATCATCACCA
scaffold90221_15.6	1	p2	(AC)9	18	3195	3212	CTCCAACCTCCCTGTACTGC
scaffold90289_17.0	1	p2	(GA)6	12	1907	1918	TAGTTGGAGGGAATGCTTCG
scaffold90295_19.0	1	p2	(AT)8	16	380	395	TGGCTCCTGAATCTCACAGA
scaffold90477_14.5	1	p2	(TA)7	14	145	158	TCACACAGAATCCCTGTTGC
scaffold90480_20.7	1	p2	(AG)7	14	56	69	ATAAATATCAGGTGTTTTCTGAGTTG
scaffold90540_16.9	1	p2	(AT)6	12	53	64	CCCTTTTAGGCTCAAATCTCTTT
scaffold90609_12.5	1	p2	(AT)9	18	355	372	TTTTCGCCTCCCACTAAAAA
scaffold90611_10.6	1	p2	(TA)8	16	329	344	TTTTTGCCACCTATGCACAA
scaffold90630_11.6	1	p2	(AT)9	18	65	82	TCGGCCAGAGAGAAAAAGAA
scaffold90656_22.4	1	p2	(TC)7	14	144	157	AGTGCGTTTGCAGAGCTTTT
scaffold90675_16.7	1	p2	(AT)8	16	131	146	TCATGATATTTTTCCTAAATTTTCCTT
scaffold90762_10.9	1	p2	(AT)7	14	142	155	CTCACCGTGGACGTAGATCA
scaffold90841_16.8	1	p2	(AG)9	18	1248	1265	AGCGGCAGACGTTTCTAGTC
scaffold90872_11.4	1	p2	(TA)8	16	531	546	CGGAGATGAGGATGCTTAGG
scaffold91053_15.4	1	p2	(CT)6	12	24	35	CCACAAACCGAGCTCACC
scaffold91226_20.8	1	p2	(AT)9	18	225	242	CATGGATAGAGCGTGCAATG
scaffold91237_10.8	1	p2	(AT)10	20	218	237	TCCGTCGGTGATTCAAGAGT
scaffold91306_10.8	1	p2	(CA)7	14	155	168	ATTCCCGGGCTGAGATAGTT
scaffold91539_12.7	1	p2	(AT)9	18	46	63	AAGGAACAAATGAGAATGTTAGTGC
scaffold91625_11.1	1	p2	(CT)6	12	93	104	GGGATTTGGGACGATTTTCT
scaffold92324_14.2	1	p2	(AT)8	16	567	582	GTAACGCGCGAATGCTACTT
scaffold92325_16.1	1	p2	(TC)9	18	30	47	GGTGTTCTTGGCAACAGGTC
scaffold92365_10.7	1	p2	(TA)6	12	83	94	TCAGTTTCGAGTTATGAAGTTAGGAA
scaffold92386_15.5	1	p2	(TG)9	18	1971	1988	GTGGATTGAAGCATGAGCCT
scaffold92435_14.6	1	p2	(GA)6	12	238	249	ATGGGGCTGTGGTTGTAGAG
scaffold92521_11.7	1	p2	(TA)6	12	474	485	AGTGACTCACCTGAAACGCC
scaffold92650_14.7	1	p2	(AG)8	16	581	596	CACCTGCAACAGAGGAGTCA
scaffold92855_10.4	1	p2	(TA)6	12	186	197	TCACATCAATTTTCACACCCA
scaffold92942_14.4	1	p2	(AT)8	16	297	312	CCTGTTGCATCAATCAATGG
scaffold92968_11.7	1	p2	(GA)6	12	530	541	TGCAAGCATCAAACAACACA
scaffold93104_17.3	1	p2	(AT)8	16	247	262	ATGAGACAGTCCGGGATTTG
scaffold93263_13.9	1	p2	(TC)6	12	429	440	ACCTTACCCAACCCACTTCC
scaffold93321_15.8	1	p2	(AG)7	14	688	701	ATTCATTCCCACCAGAATGC
scaffold93480_10.8	1	p2	(TG)6	12	142	153	ACGTCTTGATGTCGAAACCC
scaffold93632_13.3	1	p2	(AG)6	12	934	945	CTAGGGTTAGCATGCTTGGG
scaffold93749_11.5	1	p2	(AT)7	14	312	325	TCAACCGCCAGGATTATTTC
scaffold93774_19.0	1	p2	(GA)6	12	302	313	CGTGGTTCGATCGTAAATGA
scaffold93780_11.7	1	p2	(TA)7	14	210	223	ACCCCACCTTTCTTGGATTC
scaffold93792_20.9	1	p2	(AG)6	12	516	527	GGTGGAGTGTTGGAAAAGGA
scaffold93947_13.8	1	p2	(TG)6	12	620	631	GCGTACATTTGCCCCTAAGA
scaffold93967_16.1	1	p2	(AT)7	14	250	263	ATCCAGGTTCGGTTTCAATG
scaffold94001_16.5	1	p2	(AT)7	14	242	255	TTGACTTGGGCTTTGCATTA
scaffold94218_15.7	1	p2	(TA)8	16	286	301	CTCTTGTAACAAAACCCCATGT
scaffold94308_10.5	1	p2	(CT)7	14	178	191	TATGGGGTGAGTGGGCTAAG
scaffold94373_15.2	1	p2	(CT)7	14	442	455	CCAATGGAGAACCCTTTGTC
scaffold94724_15.3	1	p2	(CA)6	12	3862	3873	CCAAGTCAATTCCTAGTGCG
scaffold94884_10.2	1	p2	(CT)8	16	415	430	TTGACTTGTGACCCTCTCCC
scaffold95089_18.1	1	p2	(CA)6	12	213	224	CATACCAAGTCCCAGGGAGA
scaffold95093_14.8	1	p2	(TC)6	12	400	411	TGAGACTGCCTTCGTCACAC
scaffold95133_15.2	1	p2	(GA)6	12	651	662	TGCTTCACTGCAAAACAAGG
scaffold95134_12.5	1	p2	(CT)6	12	78	89	CAAATGGCCGAGAGAGAGTT
scaffold95302_15.1	1	p2	(TC)6	12	1143	1154	CCCCACAGATATCCCACCTA
scaffold95343_12.1	1	p2	(TA)6	12	698	709	TCATCATTCGACTTCCATGC
scaffold95449_16.8	1	p2	(AT)7	14	79	92	AATCTCCATGTTCAGGACTTCA

scaffold95502_16.0	1	p2	(AT)6	12	3670	3681	TGTGACACCAATGTGATGGA
scaffold95592_14.0	1	p2	(TA)6	12	518	529	GCAAAGTGTGCGGTCAAAT
scaffold95628_14.2	1	p2	(TA)7	14	112	125	GAAGGAGCAATGAGAGGTGC
scaffold95630_14.4	1	p2	(AT)8	16	111	126	AATCTTCATAAGTGGAGGGGC
scaffold95669_14.9	1	p2	(AT)8	16	1027	1042	CAAAACGTCCGTATCGACAA
scaffold95717_10.8	1	p2	(AG)6	12	231	242	TGAAGATTTTCATGGTGGATTC
scaffold95781_13.6	1	p2	(CT)7	14	616	629	ATGTTTCAATGACACGTGGG
scaffold95826_18.3	1	p2	(TG)7	14	266	279	CTGGGTCTGGTGTTGTTGTG
scaffold95907_12.2	1	p2	(AG)6	12	1333	1344	TTACATTTTTCTGGCTCCCAA
scaffold96034_14.8	1	p2	(TA)9	18	71	88	TCAGTTTCGAGTTATGAAGTTAGGAA
scaffold96357_15.2	1	p2	(TC)10	20	4238	4257	TCATCATCGTGCTTTCCTTG
scaffold96416_14.0	1	p2	(AG)6	12	232	243	ACCATCATCGTCATCCACCT
scaffold96508_17.0	1	p2	(CT)7	14	2597	2610	CAACAAAAAGCTCGCAATCA
scaffold96755_16.6	1	p2	(AT)8	16	1512	1527	TTCATGCTGAGGTGTTGTCG
scaffold96796_15.4	1	p2	(CT)6	12	1670	1681	GGCTGAACCATTCACCTGAT
scaffold96934_17.3	1	p2	(AT)7	14	1280	1293	TTATATTTGGTCGGTGGGGA
scaffold97005_14.6	1	p2	(CT)7	14	441	454	CCTTCATTTTATCGGCTCCA
scaffold97045_10.2	1	p2	(AT)9	18	680	697	ATTGGTGACGAGAGAGTGGG
scaffold97071_12.4	1	p2	(AT)6	12	58	69	TTGCAATTAGTGCATACAATGATAA
scaffold97146_10.5	1	p2	(TA)10	20	176	195	ATCGCAAATAAAACAACCGC
scaffold97172_17.7	1	p2	(AT)7	14	1995	2008	ATTGCCCCCATCATAACAAA
scaffold97502_13.9	1	p2	(TC)6	12	114	125	CTGATTCGCACAAATGATCG
scaffold97673_12.0	1	p2	(AT)12	24	236	259	GACGTGTGTGTGATTGAGGG
scaffold97704_13.0	1	p2	(TA)13	26	417	442	TGGTTATTACTCGAACGCGA
scaffold97943_12.0	1	p2	(AG)6	12	275	286	GACCGTGTAGGAAGGCTGAG
scaffold98089_12.9	1	p2	(AC)8	16	316	331	TTCTAGGCACAAAACCCACC
scaffold98248_13.8	1	p2	(CT)6	12	204	215	AATGGCAGATTTGAAGACGG
scaffold98287_15.2	1	p2	(AT)8	16	400	415	GCCAACATCAACTGCGAGTA
scaffold98322_16.2	1	p2	(AT)6	12	928	939	GTTCAAGAAAGCCGTGGAAG
scaffold98487_12.0	1	p2	(AT)6	12	309	320	CATCCTCATAACCCCCACAC
scaffold98557_14.8	1	p2	(AT)6	12	2293	2304	CTTAAGACCCGATGCCTTTG
scaffold98577_13.9	1	p2	(AG)6	12	1016	1027	TTCTCTATCAATCGGCTGGG
scaffold98867_15.7	1	p2	(GA)7	14	488	501	AAACCACCCCGTCTTTTCTT
scaffold98871_13.8	1	p2	(AG)7	14	270	283	TCCTTTCCATCTTCCCTCCT
scaffold99059_14.3	1	p2	(CA)6	12	1933	1944	AAATGAGACAAATGTGGGGG
scaffold99064_15.8	1	p2	(TC)6	12	1078	1089	AATCTTGTGCACTGTGTGCC
scaffold99164_15.5	1	p2	(CA)6	12	1410	1421	GCGGCATCATCTTCTCTCTC
scaffold99432_16.3	1	p2	(CT)8	16	550	565	CGTCCGTCCCATAAACAACT
scaffold99434_15.6	1	p2	(AT)6	12	4425	4436	TCCGTACAACTAACATCCTCCC
scaffold99474_16.4	1	p2	(GA)8	16	176	191	GGCCTGATTTGCAATACTGTG
scaffold99531_15.1	1	p2	(CT)6	12	698	709	ACCCTCAAGCATCAGCATTC
scaffold99667_14.1	1	p2	(TC)9	18	2024	2041	CTACCTTAGGCCAACCCACA
scaffold99668_12.1	1	p2	(TG)6	12	341	352	GATTTTGCACGAAGCAATGA
scaffold99774_13.8	1	p2	(AG)6	12	394	405	TGAAAATGGGTACCACACACA
scaffold99932_15.6	1	p2	(CT)6	12	1455	1466	GCGCTACATGCTTTGTGAAT
scaffold100046_12.[illegible]	1	p2	(TA)10	20	328	347	GCAGGTTCGAAGTAGGGTGT
scaffold100151_15.[illegible]	1	p2	(AT)9	18	3923	3940	ATCTTTTGCGATGCCTCAAG
scaffold100153_13.[illegible]	1	p2	(TA)6	12	399	410	CCCCTTTGAGCTTTTTAGCC
scaffold100162_13.[illegible]	1	p2	(TA)7	14	171	184	CGATTGGGCCTATCCTATCA
scaffold100272_11.[illegible]	1	p2	(AG)6	12	79	90	GGATTTTCGAAATTTTCTCGC
scaffold100288_16.[illegible]	1	p2	(AT)8	16	457	472	GCGAAACAATGATCGCATTA
scaffold100746_10.[illegible]	1	p2	(AT)8	16	463	478	CTTTTTCGGGTTTGGACGTA
scaffold100776_13.[illegible]	1	p2	(AG)6	12	65	76	ACAATACAACTGTCGAGCTGAA
scaffold100999_13.[illegible]	1	p2	(CA)7	14	295	308	GGCCCACGATGGTTATTAAA
scaffold101406_11.[illegible]	1	p2	(AT)7	14	450	463	TGGAATTCAATGGCAGATGA
scaffold101547_12.[illegible]	1	p2	(TA)6	12	378	389	AACCGTGTGTACAACAATAGCAA
scaffold101660_12.[illegible]	1	p2	(GA)10	20	95	114	CCTCTCCTCTCAACTTGCTTG
scaffold101684_13.[illegible]	1	p2	(TG)9	18	99	116	TAATTGGCCCTTGATCTCCA

scaffold101705_13.[illegible]	1	p2	(CT)7	14	126	139 GCATCTCTTGCTCTCTCGTTG
scaffold101726_19.[illegible]	1	p2	(GA)9	18	1618	1635 GCAGAAGGAACTGATGGCTC
scaffold101865_18.[illegible]	1	p2	(TC)6	12	668	679 CCCTCAAACCTACTGTCGGA
scaffold101888_19.[illegible]	1	p2	(GA)7	14	559	572 CCCAATTCTAGCAGCAGAGC
scaffold102077_17.[illegible]	1	p2	(AT)8	16	234	249 CTCTTGACTTCTGATCCGGC
scaffold102318_10.[illegible]	1	p2	(CA)7	14	337	350 CCTTATGAAAAACGTTGGGG
scaffold102497_12.[illegible]	1	p2	(AT)7	14	64	77 GAGCCTCGGATTTCCTGTTT
scaffold102773_14.[illegible]	1	p2	(AG)6	12	300	311 CTAGGGTTAGCATGCTTGGG
scaffold102849_12.[illegible]	1	p2	(AT)7	14	258	271 ACGGATGCATTCGACATTTA
scaffold102891_15.[illegible]	1	p2	(AG)6	12	109	120 ACTGAATTTGGGGTTGTTGG
scaffold103040_11.[illegible]	1	p2	(AT)7	14	241	254 GGATTGGGCTTTGAGAATGA
scaffold103208_12.[illegible]	1	p2	(TA)6	12	326	337 CTTTTTGCCCTCGTATGCTC
scaffold103262_10.[illegible]	1	p2	(TC)7	14	43	56 CAGAAATTGCCTTGGTTGTTC
scaffold103433_12.[illegible]	1	p2	(TA)7	14	365	378 TTGACGCTGAAGTTAATGCAA
scaffold103583_13.[illegible]	1	p2	(CT)6	12	162	173 GGTCTTCTAGCCTCTCTCGCT
scaffold103610_17.[illegible]	1	p2	(TA)9	18	349	366 CGAATCGCATTGCTATCATC
scaffold103622_10.[illegible]	1	p2	(TC)6	12	183	194 TTACTTTCCGTTTTGGGACG
scaffold103869_16.[illegible]	1	p2	(CT)6	12	357	368 GAAACACTTTCACCTCCCCA
scaffold104571_10.[illegible]	1	p2	(CT)7	14	174	187 GGCAAACAGGTCCCTAAACA
scaffold104583_13.[illegible]	1	p2	(TC)6	12	1378	1389 ATGGGATAGCACTTTTGCCA
scaffold104709_11.[illegible]	1	p2	(TA)9	18	43	60 CGGATTTAAGGGTTATTACCTGAT
scaffold104722_11.[illegible]	1	p2	(AT)6	12	612	623 TTCTCCTCCCCTTAGCTTCC
scaffold104808_15.[illegible]	1	p2	(CT)6	12	226	237 CGTCGAAAGGGAAAGAAACA
scaffold104827_14.[illegible]	1	p2	(AT)6	12	254	265 TTTTGGGGTACATGCCACTT
scaffold104838_12.[illegible]	1	p2	(CT)8	16	675	690 CACAAAATGGATCGAAACGA
scaffold105003_13.[illegible]	1	p2	(GT)6	12	760	771 AAAAGCGTTATGCGTCGAGT
scaffold105045_11.[illegible]	1	p2	(GT)6	12	202	213 TTCCACAACTGCAATTTACTGTG
scaffold105151_16.[illegible]	1	p2	(CT)6	12	355	366 AGGATTGGAAGCAGTGCCTA
scaffold105170_17.[illegible]	1	p2	(AG)8	16	3103	3118 GAGACACGTTGGGTCTGGTT
scaffold105173_15.[illegible]	1	p2	(GA)6	12	2248	2259 CACAACAGGAGGAGGCACTT
scaffold105290_16.[illegible]	1	p2	(CT)9	18	1011	1028 CCAAGATTGAGGTCCGTTTC
scaffold105314_16.[illegible]	1	p2	(AG)6	12	4149	4160 TATTCCCAATCTGGGCACTC
scaffold105325_13.[illegible]	1	p2	(AT)6	12	287	298 GGACGCACCAGATTGTTTTT
scaffold105496_12.[illegible]	1	p2	(TA)7	14	176	189 TTTGACATCAACGGATGGAA
scaffold105518_16.[illegible]	1	p2	(AT)6	12	105	116 GAAAGTGGCTTTGATGGAGA
scaffold105585_11.[illegible]	1	p2	(TA)6	12	218	229 TGCAATATGTTTATTTCGGGA
scaffold105617_13.[illegible]	1	p2	(AT)6	12	139	150 CCACATTTTTCCCGACTGTT
scaffold105757_15.[illegible]	1	p2	(TG)7	14	174	187 GCCTGAATGTAATGCCGAAA
scaffold105937_13.[illegible]	1	p2	(TC)7	14	97	110 CCCCTTTGTCAACTTAGGCA
scaffold106235_14.[illegible]	1	p2	(CT)7	14	806	819 CAGCTTTCGGCTTCTTCTTG
scaffold106473_13.[illegible]	1	p2	(AG)10	20	179	198 GGAAATCCCGTACGCTATCA
scaffold106474_15.[illegible]	1	p2	(AG)6	12	2320	2331 TAAGCAATACACGCACAGGG
scaffold106499_16.[illegible]	1	p2	(TA)8	16	1052	1067 TGATATGCAAGAATTGTCACCC
scaffold106528_20.[illegible]	1	p2	(TC)7	14	764	777 GGCCTCAGCAGCTTCTCTAC
scaffold106593_10.[illegible]	1	p2	(TA)6	12	327	338 CGTGCGACCAAATGTGACT
scaffold106603_15.[illegible]	1	p2	(AT)6	12	4496	4507 TGTAGGCTTTGGTTTCCATTG
scaffold106633_16.[illegible]	1	p2	(AG)8	16	746	761 TGGTGTCTTGATTGAGCGAG
scaffold106666_13.[illegible]	1	p2	(GA)6	12	31	42 GAAGATGGAGTAGAGAGAGAGATTT[illegible]
scaffold106678_12.[illegible]	1	p2	(TA)6	12	466	477 CATTTCAACCCACCATCTCC
scaffold106707_13.[illegible]	1	p2	(GT)7	14	257	270 GAACCGGATTTAGAGCAATCA
scaffold106769_13.[illegible]	1	p2	(TA)9	18	639	656 GAACAAATCTGGGCCATCAG
scaffold106778_18.[illegible]	1	p2	(TA)6	12	351	362 GGTGTGGTGGGGACATTTTA
scaffold106974_15.[illegible]	1	p2	(CT)7	14	1596	1609 ACACCCATTGCTGTGTGAGA
scaffold107088_13.[illegible]	1	p2	(AT)6	12	1588	1599 ATCCAACACCGCATCTTCTC
scaffold107115_12.[illegible]	1	p2	(TG)6	12	90	101 AGACTAGAACGTAGACCTGGCA
scaffold107221_12.[illegible]	1	p2	(AT)6	12	188	199 CAATTTAGCCCGAAATGAGC
scaffold107252_16.[illegible]	1	p2	(TA)6	12	4607	4618 GAGGTGGCGCTCTGCTATAC
scaffold107257_14.[illegible]	1	p2	(TA)7	14	750	763 TCAACAATCTCAAACTGCAACC

scaffold107337_15.[illegible]	1	p2	(TA)6	12	310	321	ACAACCTGGGTATCCCAAAA
scaffold107459_15.[illegible]	1	p2	(AT)8	16	285	300	ACGGAATTGAATTTGGGACA
scaffold107504_10.[illegible]	1	p2	(AT)12	24	458	481	CATGCCAAGTGGTGTGATCT
scaffold107603_10.[illegible]	1	p2	(AT)6	12	102	113	GCTTGGTGAAGGAAGTGAGG
scaffold107689_12.[illegible]	1	p2	(AG)6	12	204	215	GAAACGACGGGAAGATGAAA
scaffold107806_11.[illegible]	1	p2	(AC)6	12	155	166	GCGCAGATCTAATGGTCCAG
scaffold107931_15.[illegible]	1	p2	(GA)11	22	2966	2987	ACCGGAGAGTGGTGGTGTAG
scaffold108279_15.[illegible]	1	p2	(TA)6	12	222	233	CCGAATGATGTGCATTTTTG
scaffold108368_11.[illegible]	1	p2	(TA)6	12	92	103	ACATTCAGGCCTTTTGAACA
scaffold108577_20.[illegible]	1	p2	(AT)6	12	108	119	TTCTAACTCCATTTGTAATAGTAAGG[illegible]
scaffold108710_15.[illegible]	1	p2	(AT)8	16	141	156	AGAAAGGAGATGGAGCGTGA
scaffold108812_10.[illegible]	1	p2	(GA)6	12	160	171	GGGTGATGAGGCTTGAAGAA
scaffold108860_14.[illegible]	1	p2	(AT)11	22	2438	2459	CATGCATGCAAAACTGAGGT
scaffold109050_14.[illegible]	1	p2	(AC)7	14	1232	1245	TGGGTGGAACTTCTTGCTTT
scaffold109061_17.[illegible]	1	p2	(AT)8	16	1609	1624	TTTCAAACGAATTGCAGCAG
scaffold109161_15.[illegible]	1	p2	(AG)7	14	2428	2441	AGCTGGTAGCCATTCTCCTG
scaffold109174_17.[illegible]	1	p2	(AG)8	16	327	342	TGCCTGAAAATGTCTGCAAG
scaffold109379_14.[illegible]	1	p2	(AT)7	14	233	246	CTCGTTCCCAAGCCTCCTAT
scaffold109456_15.[illegible]	1	p2	(CG)6	12	2690	2701	CACTGGGTTATTCCAATCCG
scaffold109463_16.[illegible]	1	p2	(TC)10	20	753	772	ATTAAATCCCCCGAAGAAGC
scaffold109545_14.[illegible]	1	p2	(TA)13	26	412	437	TGGGATATCGTCTAACCAGCA
scaffold109557_15.[illegible]	1	p2	(GC)6	12	125	136	GCAAATCCCAAAACCGAATA
scaffold109834_20.[illegible]	1	p2	(CT)6	12	459	470	ATCGAAAAGAGGTGAGCGAA
scaffold110045_10.[illegible]	1	p2	(AG)7	14	365	378	GACCGCGAAGTTTACATGGT
scaffold110087_15.[illegible]	1	p2	(CT)7	14	299	312	ACCAACCCAACCCATTATCA
scaffold110177_14.[illegible]	1	p2	(AC)7	14	1182	1195	GGCAGGTCATGTGTATGTGG
scaffold110178_10.[illegible]	1	p2	(TC)7	14	86	99	TGAAATGCCTCCGGTTAGTC
scaffold110196_15.[illegible]	1	p2	(AG)6	12	272	283	TTGGTTTGTTGCTTGATTGC
scaffold110232_13.[illegible]	1	p2	(AT)6	12	175	186	CCTTCTCCACCACCACTTTG
scaffold110245_15.[illegible]	1	p2	(AT)7	14	114	127	CCAGGTGAGCTAGCCCATAA
scaffold110366_15.[illegible]	1	p2	(GA)6	12	652	663	GGTGTGTGGAAGGAAGGTGT
scaffold110486_12.[illegible]	1	p2	(AC)7	14	277	290	AAATTCCATGGCCTTTCACA
scaffold110519_10.[illegible]	1	p2	(GT)9	18	389	406	GAGTGTGTGTCGTGGGTGTC
scaffold110535_14.[illegible]	1	p2	(CT)6	12	2021	2032	GGGATTTCAGCGACGATTTA
scaffold110711_12.[illegible]	1	p2	(AT)6	12	375	386	AGAGGAGGGCTTCAACATGA
scaffold110847_12.[illegible]	1	p2	(CT)6	12	163	174	CAAAGGATCGAAAAACGAGG
scaffold110891_10.[illegible]	1	p2	(TC)8	16	371	386	TAATGTGGAACCATAGGGGC
scaffold111070_16.[illegible]	1	p2	(AG)6	12	113	124	ACCTATCGTCTCCTCCCGAT
scaffold111248_15.[illegible]	1	p2	(CT)7	14	35	48	GCCTCTTCTCTGCTAAAAGCC
scaffold111277_14.[illegible]	1	p2	(TG)7	14	226	239	TGAAAAGATTTGAAAAACATTCAAC
scaffold111400_11.[illegible]	1	p2	(CA)6	12	260	271	CTCTCGCCATCTTCGACACT
scaffold111409_20.[illegible]	1	p2	(CT)6	12	306	317	TAGGCATGGCCTCAACTACC
scaffold111442_16.[illegible]	1	p2	(GA)6	12	114	125	GATGGAACGGGGATTTGTTA
scaffold111456_15.[illegible]	1	p2	(GA)6	12	458	469	TCGAGCATAAACACATCCCC
scaffold111468_21.[illegible]	1	p2	(TC)6	12	61	72	AGCTACACTCACCGTCTGAGAA
scaffold111671_15.[illegible]	1	p2	(CT)6	12	895	906	CGATTGCTCTCCGATCTCTC
scaffold111690_14.[illegible]	1	p2	(CT)8	16	327	342	GCAAGACATGCAAGGCAGT
scaffold111946_19.[illegible]	1	p2	(AG)7	14	360	373	TTGGGTTGTCCCTTGATCTC
scaffold111984_16.[illegible]	1	p2	(TA)8	16	366	381	CAGCAAAGTGCTAAATGGTTCA
scaffold112119_15.[illegible]	1	p2	(TA)9	18	200	217	TTGGTGGTTTGTATGCTATCG
scaffold112224_14.[illegible]	1	p2	(AT)10	20	4280	4299	GCCCCTCCTAGCAATTTGA
scaffold112265_10.[illegible]	1	p2	(AT)9	18	110	127	ACTCCGCATGTGTGTTGAAA
scaffold112267_10.[illegible]	1	p2	(AT)8	16	63	78	TTGGGATTAAGGCTTTGGTG
scaffold112270_13.[illegible]	1	p2	(AT)6	12	116	127	GAAACAACGAATCCATTAAGTGAAG
scaffold112290_15.[illegible]	1	p2	(AT)7	14	481	494	TTTCGGGACACGTTCTCTCT
scaffold112382_16.[illegible]	1	p2	(CT)6	12	309	320	CCCCTCATTTTCATTCCAAA
scaffold112447_13.[illegible]	1	p2	(CA)6	12	2553	2564	TGTCATTGGTTGTGGCTGTT
scaffold112464_15.[illegible]	1	p2	(TA)7	14	1726	1739	AATGCCCCTATTTTTGGAGG

scaffold112500_11.[illegible]	1	p2	(AT)7	14	204	217 ATGGACGTCGTGACCACATA
scaffold112660_13.[illegible]	1	p2	(GA)6	12	116	127 CACAATGGGGGTATTTGCTT
scaffold112791_17.[illegible]	1	p2	(CT)8	16	953	968 CCCTAACTCGACCCAGATCA
scaffold112990_15.[illegible]	1	p2	(TA)9	18	1315	1332 TCGAGTTTGTTTGCGAGATG
scaffold113105_11.[illegible]	1	p2	(TC)6	12	175	186 TCCCAATCAAGACAAGTCCC
scaffold113114_15.[illegible]	1	p2	(AG)6	12	644	655 TAATTTGGTTGGCCCATTGT
scaffold113121_15.[illegible]	1	p2	(TA)7	14	111	124 CCATTTGCAATACTAAGGCTTTT
scaffold113201_16.[illegible]	1	p2	(CT)6	12	861	872 TTTCAAGAAAAGGGTGCCAT
scaffold113205_12.[illegible]	1	p2	(TA)7	14	186	199 ACGTCGGTTTGGTTTAAAGG
scaffold113231_10.[illegible]	1	p2	(TA)7	14	99	112 GACCCATTTAGAACCCACGTT
scaffold113488_17.[illegible]	1	p2	(TA)8	16	210	225 TGGAACAATTTTTGCCACAT
scaffold113630_13.[illegible]	1	p2	(GA)6	12	126	137 GAAAAACGACGTGAGGGAGA
scaffold113781_17.[illegible]	1	p2	(AT)9	18	163	180 TTTTGGAGTATTCCGTGCCT
scaffold113924_12.[illegible]	1	p2	(AT)9	18	730	747 CACTTGACGAGGTCACTGGA
scaffold114052_13.[illegible]	1	p2	(AG)7	14	386	399 AATATTTCATTCATGCCACGC
scaffold114239_12.[illegible]	1	p2	(TC)6	12	1733	1744 GTCGAGGAGTATACGGAGCG
scaffold114339_10.[illegible]	1	p2	(TA)7	14	162	175 ACGTAAAAACCCTTCCACCC
scaffold114404_16.[illegible]	1	p2	(TA)6	12	195	206 ATGCATGCACGTGAATATGG
scaffold114476_10.[illegible]	1	p2	(TA)7	14	218	231 ACGGATATGCCCTTGTATGC
scaffold114555_13.[illegible]	1	p2	(GA)8	16	50	65 GATGCTTACCTGAACAGCACA
scaffold114904_11.[illegible]	1	p2	(TC)6	12	115	126 ACAAGGATTGCGAACTCAGC
scaffold114905_10.[illegible]	1	p2	(TC)7	14	113	126 CCCCTCTCAACAACCACAAT
scaffold115033_17.[illegible]	1	p2	(CT)12	24	4753	4776 AGAAATTGCCTGCTTCCAAA
scaffold115143_15.[illegible]	1	p2	(GA)7	14	258	271 CCGGGTTTAAAGCAATCAAA
scaffold115376_15.[illegible]	1	p2	(CT)9	18	3613	3630 CCACTTCCTAGGCAAACACC
scaffold115419_14.[illegible]	1	p2	(CT)8	16	296	311 GGGACTGGACCAAGAATTGA
scaffold115517_18.[illegible]	1	p2	(TA)8	16	706	721 GGTGCAGTGGTAGAGCATCA
scaffold115672_15.[illegible]	1	p2	(GA)6	12	388	399 TTGACCACAATCAAACACCAA
scaffold115696_14.[illegible]	1	p2	(CT)6	12	583	594 TTGATTTCCCACCACCATTT
scaffold116113_10.[illegible]	1	p2	(AG)6	12	52	63 GGTGGTGTGGAGGAGAGAAG
scaffold116128_11.[illegible]	1	p2	(AT)7	14	762	775 TGATTTTCCATTATTTTCGTTGG
scaffold116210_14.[illegible]	1	p2	(GT)7	14	2457	2470 TTAATGCTTTGAAGGGGTGC
scaffold116266_12.[illegible]	1	p2	(AT)6	12	659	670 ACAAATCCCACCTGCGTAAG
scaffold116286_18.[illegible]	1	p2	(CT)6	12	115	126 TAATTCGTATTTCGGTCCGC
scaffold116335_13.[illegible]	1	p2	(TA)6	12	226	237 CCCAAAAAATTTAACGACCCA
scaffold116391_14.[illegible]	1	p2	(AT)7	14	327	340 TGTTACATGGTTGTCGGTTCA
scaffold116554_10.[illegible]	1	p2	(AG)7	14	54	67 CCATGTTGTTTGTAATTATTGTAGGG
scaffold116716_16.[illegible]	1	p2	(AT)7	14	736	749 ATTTGGAAACCTCCCACTCC
scaffold117227_10.[illegible]	1	p2	(TC)6	12	335	346 CTCTCGCTAGGCGACTTTGT
scaffold117620_17.[illegible]	1	p2	(TC)8	16	190	205 GCAGCTAGGCTCCCTCCTAT
scaffold117645_10.[illegible]	1	p2	(GA)6	12	75	86 GGGGTCGGTGGAAAAATAGA
scaffold117924_15.[illegible]	1	p2	(AT)7	14	2134	2147 CAAAGAACCAACATCCACACA
scaffold118160_16.[illegible]	1	p2	(CT)14	28	396	423 ATGTCACGTCAAGCATCAGC
scaffold118164_10.[illegible]	1	p2	(GC)6	12	359	370 CGCTATGAGTCACGGGTACA
scaffold118256_12.[illegible]	1	p2	(AT)8	16	274	289 TGAAACAATGCCTGGAAACA
scaffold118351_14.[illegible]	1	p2	(TA)7	14	160	173 GCAACTGTACACGTGTGGCT
scaffold118603_16.[illegible]	1	p2	(AG)7	14	316	329 CGAAGAATTTTGTGCCATGA
scaffold118763_12.[illegible]	1	p2	(AT)9	18	566	583 TTGTTGAACGCATAATTCGATA
scaffold118777_15.[illegible]	1	p2	(AT)6	12	148	159 CGTTCCTCAGAGTTTCCAGC
scaffold118901_12.[illegible]	1	p2	(AG)6	12	66	77 CACAAACTTTCTGGATTGACTTTG
scaffold118931_18.[illegible]	1	p2	(AC)7	14	62	75 TCAACACTCTGTTTTTCTCTCACA
scaffold119077_11.[illegible]	1	p2	(GA)6	12	537	548 CGTGTCCTCCTCAAACTGGT
scaffold119101_14.[illegible]	1	p2	(AT)9	18	45	62 TCTATTATATGTCATGTTTTGCATTTT
scaffold119124_17.[illegible]	1	p2	(TC)6	12	103	114 CCCATCCCTTAATCAACCCT
scaffold119131_10.[illegible]	1	p2	(GA)6	12	68	79 GACGCGAGATTTACGTGGTT
scaffold119299_16.[illegible]	1	p2	(GA)6	12	381	392 TTTCATGTTTAGCATTTGCG
scaffold119359_14.[illegible]	1	p2	(TC)7	14	226	239 CAATCCGGTGATGTGTCAAG
scaffold119453_13.[illegible]	1	p2	(GA)7	14	543	556 CTTTGCGACGTAATTCCCAT

scaffold119511_16.2	1	p2	(CA)8	16	1252	1267	GCAGGAAGTCATGGTTGCTT
scaffold119593_14.2	1	p2	(TA)11	22	1250	1271	ATTTAATCACGTGCAACGCA
scaffold120058_15.3	1	p2	(CA)10	20	1031	1050	CATCCAATAGCCGAACAAAAA
scaffold120216_16.8	1	p2	(TC)6	12	1531	1542	AACTGCGATGCTCCAAAAGT
scaffold120263_12.8	1	p2	(AG)10	20	613	632	AGAAAGATCCCTGCGGTTTT
scaffold120352_15.1	1	p2	(TA)10	20	39	58	AGCACAATACAAACATGTCCAC
scaffold120409_11.9	1	p2	(GT)6	12	105	116	TTGTGTGTTATACGTGTGGGTG
scaffold120583_11.1	1	p2	(AT)7	14	194	207	TCAAGGTCTTCGCTGGTTCT
scaffold120643_14.5	1	p2	(TA)9	18	356	373	TATATGATGTAGGCCGCGTG
scaffold120882_14.8	1	p2	(TG)7	14	1075	1088	TGGACATGGCAATTATTCTTGA
scaffold120953_15.0	1	p2	(AG)7	14	564	577	ATGGGTTCCTTACCCCTTTG
scaffold121065_18.7	1	p2	(GA)6	12	290	301	GACAGAGGTCCCCTGGTTTT
scaffold121079_20.0	1	p2	(AT)9	18	330	347	CCAAGTCTCACCTACAAATCCA
scaffold121135_12.0	1	p2	(AG)6	12	56	67	GCAAGGGAAACTGATGGTGT
scaffold121210_15.3	1	p2	(TA)9	18	45	62	CGGATTTAGCGGTTATTACCTGA
scaffold121646_16.7	1	p2	(AT)7	14	1132	1145	GTTTAGATTCCGCATCAGGC
scaffold121793_12.6	1	p2	(GT)7	14	138	151	CAGTTGTGTTGTGGCAGCTT
scaffold121822_11.9	1	p2	(TC)6	12	82	93	TTTGAGATCGGAGTGGCTCT
scaffold121845_16.0	1	p2	(TA)7	14	616	629	ATCCGACGGTTGTAGTGGAG
scaffold121887_16.9	1	p2	(AG)6	12	3958	3969	TTCAACCGTGCGTGTATGTT
scaffold122450_16.6	1	p2	(GA)6	12	165	176	AAGATGATGGTGAAAGGGGA
scaffold122524_10.6	1	p2	(AT)6	12	91	102	ACTCACTTAGGGATATATTTAGGGAA
scaffold122594_10.8	1	p2	(TA)7	14	286	299	GACATGCATCGTTTGTACTCTTC
scaffold122648_10.4	1	p2	(AT)11	22	852	873	GGCTCAATTTTAATGGGCCT
scaffold122699_12.7	1	p2	(AC)6	12	324	335	GATCAACGCCCCATATTGTT
scaffold122819_16.1	1	p2	(TC)7	14	2446	2459	GATATTTCAGGGTCCCCGTT
scaffold122877_13.1	1	p2	(AC)6	12	389	400	TCTCCTTCTCAGGATCTCCG
scaffold123109_10.7	1	p2	(TC)7	14	63	76	GAACGGGCAGTACTCCAAAA
scaffold123324_13.3	1	p2	(CT)6	12	216	227	AAATGAAGGGTTGAGCATCG
scaffold123673_11.1	1	p2	(GA)7	14	232	245	AAGAACGTTGGGAGGGAAGT
scaffold123681_15.6	1	p2	(TA)7	14	807	820	CTGAACCTGAACCACCAACC
scaffold123762_14.3	1	p2	(AT)9	18	379	396	GTTGATAAATTACAAAAATTTAAGCG
scaffold123928_14.5	1	p2	(AG)6	12	556	567	ACACCAACCCATTTGCTCTC
scaffold123946_16.3	1	p2	(GA)6	12	70	81	TGTGTATATCTGATAGCCGTAGCA
scaffold124340_10.0	1	p2	(TA)8	16	47	62	AGGGGAGTTATCACTGGATCA
scaffold124770_14.3	1	p2	(AG)6	12	1431	1442	TAGAGATGCGAAGATGGCCT
scaffold124784_10.9	1	p2	(GA)8	16	756	771	GTCCCCAGCCTCAGATACAA
scaffold124800_15.2	1	p2	(TA)7	14	1157	1170	CAGATGATCACACGCTGCTT
scaffold124869_16.5	1	p2	(AT)8	16	6182	6197	TCCTCTGCCTTATTGATGGG
scaffold124892_10.0	1	p2	(AT)13	26	215	240	CGTGTTACCCTCTCAATTTATCA
scaffold124952_14.7	1	p2	(GT)6	12	705	716	TTTAGGCATTCCCACCTTTG
scaffold125082_19.0	1	p2	(CT)6	12	171	182	CCCAACTCTTCGGCTCAATA
scaffold125401_11.6	1	p2	(AT)9	18	49	66	GTCTGCAAGAAAATGGGTGG
scaffold125498_15.4	1	p2	(TC)6	12	206	217	AGAGATTGTGGGGCTTGATG
scaffold125582_10.7	1	p2	(TC)6	12	383	394	ACCAACCCAAACCCACTTTC
scaffold125628_17.8	1	p2	(CT)6	12	101	112	AGCATCTCTCTCTTGCTGTCA
scaffold125686_10.8	1	p2	(TA)9	18	297	314	CATTTTGACATGATCGACGG
scaffold125739_20.2	1	p2	(AT)8	16	802	817	CGTGTCTACAAAACTTGCTGC
scaffold125758_11.3	1	p2	(AG)8	16	424	439	TTCAAGGCATGGGAGGATAG
scaffold125767_14.8	1	p2	(AT)6	12	957	968	CACACGATAGAGGCAGACGA
scaffold125781_15.7	1	p2	(TA)11	22	293	314	TCAACTTTCGGAAGCGTACA
scaffold125784_10.8	1	p2	(AT)6	12	581	592	CGTCCTATTGGCCCATTTAT
scaffold125793_13.6	1	p2	(AT)8	16	1538	1553	TGCGCTTGGTTTGTAGACTTT
scaffold125849_13.7	1	p2	(TA)6	12	560	571	CTGTCTCCGGTCCATGAAAT
scaffold125852_15.0	1	p2	(TC)9	18	750	767	GCGAAAACAACAGACAGCAA
scaffold126248_15.2	1	p2	(AG)7	14	5697	5710	GGCTTGTGTGCATCTTCTTTC
scaffold126303_13.4	1	p2	(AT)6	12	267	278	TGTCGTGGTTCGGATATTCA
scaffold126342_15.7	1	p2	(AT)6	12	2128	2139	ACATATTTTTCCCCTTTCCACA

scaffold126470_15.2	1	p2	(AG)6	12	2681	2692	CAAAATTGCGTTTTATGGGG
scaffold126743_11.8	1	p2	(CT)8	16	423	438	TCGCCATTTCCCTGTCTTAC
scaffold126855_12.7	1	p2	(TA)7	14	40	53	TCGTGATATTGGGAAACAATG
scaffold126879_14.3	1	p2	(TA)10	20	419	438	GCATTAAAATTGTTGGTCCCT
scaffold126943_10.6	1	p2	(GT)6	12	89	100	TAGGATACCCTCCCAAACCC
scaffold127066_15.0	1	p2	(AT)8	16	685	700	GCGGATTTTATTCTCGTTCA
scaffold127079_10.0	1	p2	(AT)6	12	390	401	TGGAAAAGGTAGGGACAAAA
scaffold127175_16.1	1	p2	(AT)6	12	427	438	CAAGCAACAAGCCAATAGCA
scaffold127388_15.0	1	p2	(GT)7	14	263	276	AGTTATCAAGATGGCCACGG
scaffold127434_12.7	1	p2	(TA)8	16	130	145	TTTACCCAAAACACCCCTGA
scaffold127499_18.1	1	p2	(TG)8	16	282	297	TTTTTGGGAAATCCTTGCAC
scaffold127512_11.3	1	p2	(AT)6	12	602	613	TATCGATTCACCACACGAGG
scaffold127733_15.0	1	p2	(TC)6	12	388	399	ACGAAAGTGAACTCAACCCG
scaffold127784_15.9	1	p2	(TA)8	16	48	63	TGCTCCTAGTTAGCACACAACTT
scaffold127801_12.5	1	p2	(TA)8	16	45	60	TGAAGTTAGGAAATAAGGAGGTGTT
scaffold128053_15.2	1	p2	(TA)7	14	2149	2162	GCCCCATTTTCTTAGGGTTT
scaffold128141_16.3	1	p2	(AG)6	12	2128	2139	CTTGGAGGCTCAGAGGTTTG
scaffold128227_15.0	1	p2	(TC)7	14	2564	2577	GCATCACCTCTTCTACGGGT
scaffold128276_13.4	1	p2	(TG)6	12	317	328	GTGCTTTTAGCTTGGGCAAA
scaffold128364_10.3	1	p2	(AT)7	14	219	232	CACGAACGGCTGGAATATCT
scaffold128431_14.4	1	p2	(CT)6	12	163	174	TAATAGGTGCGGGTTTGAGG
scaffold128731_12.2	1	p2	(GA)7	14	52	65	TGGAGGAATGAAGGTGAAGG
scaffold128748_16.0	1	p2	(GA)9	18	589	606	ATGGAGAGAGGGAGGGAGAA
scaffold128752_13.3	1	p2	(TA)7	14	106	119	CCCTTGGTATTCTCCCTGTG
scaffold128901_13.3	1	p2	(AT)6	12	547	558	TGCTGTGGCAATATGATGCT
scaffold128937_14.9	1	p2	(AT)10	20	5618	5637	TGACATACATGGTTTGGGGA
scaffold129128_14.2	1	p2	(CT)7	14	610	623	GCTAATGCTTCAGCACCACA
scaffold129149_14.0	1	p2	(AG)6	12	459	470	ACACACCCACACCATTGAAA
scaffold129229_14.5	1	p2	(TA)7	14	415	428	TAAGGAGATGTGTGCATGGG
scaffold129814_13.0	1	p2	(AT)10	20	684	703	CACCTCACTTTCAATGCTCG
scaffold129952_14.3	1	p2	(TA)6	12	116	127	CCAAAACTCCACCTTCAACTG
scaffold130133_11.8	1	p2	(CT)7	14	246	259	GTTGGCAAACAGGTCCCTTA
scaffold130197_10.2	1	p2	(TA)8	16	491	506	TCATGGGGAAAAGAGTTATCA
scaffold130364_10.0	1	p2	(CT)11	22	54	75	TTGATTCATACAAGTCATCTTTATGTT
scaffold130409_13.5	1	p2	(TA)12	24	37	60	GGAAATAAGGAGGTGTTATGATATG
scaffold130514_15.2	1	p2	(AG)6	12	1189	1200	TGTTGTGACTGGATTGGCAT
scaffold130608_15.7	1	p2	(TA)8	16	270	285	AAAAATGAACCGTAACCGGC
scaffold130800_14.4	1	p2	(TA)6	12	1214	1225	GTGAGAGGAAGACAGGCGAC
scaffold130880_13.6	1	p2	(TA)7	14	219	232	ATGCAACTCAAAGGGAGGAA
scaffold130998_12.5	1	p2	(TA)10	20	108	127	ATCAATGGCCACAAATGGTT
scaffold131001_11.5	1	p2	(TA)7	14	114	127	TGATTTTGGTGTGTTGGCAT
scaffold131003_14.4	1	p2	(AT)6	12	513	524	CATAAAACATACCTCGGCATCA
scaffold131096_14.3	1	p2	(AT)10	20	485	504	AATCACGTCAATTCCGAACC
scaffold131183_12.9	1	p2	(TA)6	12	670	681	TGCCACTATAATTGTATGGTCTCG
scaffold131323_10.2	1	p2	(CT)6	12	235	246	CACCTTACCCACCCAACCTA
scaffold131332_14.7	1	p2	(TC)6	12	98	109	CACAAACACACTTGGGAGGA
scaffold131342_15.6	1	p2	(AT)7	14	849	862	AGCTCTTGAGCATATTGCGA
scaffold131424_17.5	1	p2	(TC)6	12	495	506	CTGTGTGCTGGTGCTGATTT
scaffold131576_14.0	1	p2	(AT)7	14	165	178	ACGTTTTTGTAAACGTTGTCCT
scaffold131710_12.3	1	p2	(AT)9	18	396	413	GTGGACGCTCCAGATTGTTT
scaffold131968_17.8	1	p2	(GA)6	12	105	116	ATGGTGCATGCAGTCAAATC
scaffold132012_12.9	1	p2	(CT)7	14	447	460	TGGCACCTAAACCATAAGGC
scaffold132029_11.7	1	p2	(TA)6	12	50	61	CATTTAGGATATTTTCTATTCTTGCAT
scaffold132146_13.8	1	p2	(AG)6	12	467	478	AGGATACCGGAGTGTTCACG
scaffold132174_12.9	1	p2	(TA)7	14	739	752	CTCCGCCCAGTTGGATACTA
scaffold132394_14.6	1	p2	(AT)10	20	275	294	CACCACCTGTCCACTCAAAT
scaffold132412_16.4	1	p2	(AT)11	22	33	54	TTACTCACACGCACACCCAC
scaffold132432_14.3	1	p2	(AG)7	14	802	815	CTAGGGCCAAGGAGTTAGGG

scaffold132695_11.6	1	p2	(AT)10	20	417	436	AATCACGTCCTTATTAACCAAGTT
scaffold132735_14.4	1	p2	(AT)9	18	580	597	GTGATGCATGATGGGTATCG
scaffold132854_10.0	1	p2	(TA)9	18	236	253	GGTCTCCGGTCCATGAAATA
scaffold132924_13.0	1	p2	(TA)6	12	209	220	GTGAATAGCCGTTGAGGCAT
scaffold132936_13.1	1	p2	(CT)8	16	998	1013	AAGCAAAAATCAGACCTAACCG
scaffold133005_13.9	1	p2	(AT)9	18	331	348	ACAGAAACATTGACTCCGGG
scaffold133012_14.6	1	p2	(AT)11	22	249	270	TTGTGCGAACCATGAAAAAC
scaffold133044_15.7	1	p2	(AT)7	14	250	263	GCAAAGGTCGCGAAAGATAC
scaffold133325_13.4	1	p2	(TA)6	12	113	124	TATTGGTTATCACCGGATCG
scaffold133413_10.6	1	p2	(CT)8	16	234	249	CGACAGAAAGTAACCCCCAA
scaffold133602_11.8	1	p2	(AT)7	14	928	941	TGTTACCCCCACTTCATTCA
scaffold133928_18.3	1	p2	(AG)6	12	164	175	CGTTTCCATTGTTTAGCCGT
scaffold134022_15.2	1	p2	(AT)6	12	205	216	ATTGGTCGAGAGAAAGGGCT
scaffold134090_14.0	1	p2	(GT)6	12	37	48	AAGTGATCTACGTCCACGGG
scaffold134151_16.4	1	p2	(GT)7	14	721	734	GTATGTCCGGTGCAAGGAGT
scaffold134369_15.7	1	p2	(AT)6	12	6372	6383	ATTGCACAAGTTTGGCCTTC
scaffold134409_14.3	1	p2	(CT)8	16	239	254	GTTGGCAAACAGGTCCCTTA
scaffold134698_15.2	1	p2	(AT)6	12	1643	1654	ATTCAACACACGACATGGGA
scaffold134717_14.1	1	p2	(AT)6	12	1177	1188	TAAATAATGATTGGGGGCCA
scaffold134784_11.6	1	p2	(CT)7	14	62	75	AGCATCTATTTTCGACCACTGA
scaffold134872_10.1	1	p2	(TA)7	14	291	304	CTGCTTAAAGCCCAGTCCAG
scaffold135133_12.8	1	p2	(AC)6	12	571	582	CCGCGGTTGAGGATAGATAG
scaffold135138_10.7	1	p2	(TA)8	16	172	187	TTTGCTGAGAAATCTAATGCG
scaffold135165_10.0	1	p2	(TC)6	12	248	259	CCAACCCATTATCAAAACCC
scaffold135207_10.5	1	p2	(TA)6	12	72	83	CCACCTTGAGTTACATGCTCC
scaffold135210_11.1	1	p2	(AC)8	16	519	534	TGACGAATTCTCAAGTTTGTCAC
scaffold135252_17.0	1	p2	(AG)6	12	662	673	CACTCCCGACCATCTCACTT
scaffold135415_14.4	1	p2	(TG)7	14	2382	2395	GAGAGAGAGTCGGCAATTCG
scaffold135523_12.1	1	p2	(GA)6	12	241	252	TTCATTCCTCTCCAGCTCGT
scaffold135589_15.2	1	p2	(AT)9	18	252	269	TACGATGGGCTTTAAGCAGG
scaffold135600_14.7	1	p2	(AT)8	16	112	127	TTTCTGCATTGTTGCATAGC
scaffold135755_10.4	1	p2	(TC)6	12	320	331	CCTCAAATCGTTGACACGAA
scaffold135790_14.0	1	p2	(TG)6	12	2369	2380	GTGCGCATTGATGTTCCTAA
scaffold135819_14.6	1	p2	(TA)7	14	1507	1520	AATGGTCCGGGGTGATTT
scaffold135911_14.9	1	p2	(GC)6	12	58	69	TCACAGGCATTGTCAGTGTGT
scaffold136059_11.1	1	p2	(CT)6	12	212	223	TGTGCCAAGAACAACTCCTCT
scaffold136111_16.2	1	p2	(AT)11	22	274	295	TAGGGATTGAGGGTGATTGG
scaffold136205_15.8	1	p2	(AG)6	12	161	172	TGGATAGACTCATAGCCCGTG
scaffold136448_13.8	1	p2	(AT)10	20	152	171	TGACATTGCCTAGAAAGTGGG
scaffold136763_15.1	1	p2	(AT)9	18	58	75	AGGGCTGAGTACACGAATGC
scaffold137230_16.2	1	p2	(TA)9	18	455	472	AAGAACACACAATTCATGTGGAA
scaffold137346_10.7	1	p2	(AT)6	12	410	421	TGGATGTCTTTGATTTGCGA
scaffold137390_11.5	1	p2	(AG)7	14	251	264	CTATAGCCCCCGATTCACAA
scaffold137438_12.1	1	p2	(AG)6	12	607	618	ATTGGGAGAAAAGAGGGGAA
scaffold137780_11.8	1	p2	(AT)7	14	219	232	AAGAGGGTTGATGGGTGATT
scaffold137789_10.6	1	p2	(CA)10	20	130	149	TCCATTCCGAAAATCAAAGG
scaffold137813_16.1	1	p2	(AC)6	12	706	717	AAATGAAACATCAACAATCCAAA
scaffold137897_14.8	1	p2	(AG)9	18	566	583	TTAATTTCAGTCCGGGAACG
scaffold137920_18.0	1	p2	(TA)6	12	72	83	AAAATCAAAGGCCCACAATG
scaffold138057_13.0	1	p2	(GA)8	16	488	503	TGTGTATGTGTGAGTGTTCGTGA
scaffold138194_18.8	1	p2	(TC)6	12	948	959	AATCCATGATCCGAAAATGC
scaffold138213_13.0	1	p2	(AG)6	12	157	168	CCAATGGAACAGTAGCCGAT
scaffold138330_14.0	1	p2	(GA)8	16	346	361	AACACAGAGACAAGCACCCC
scaffold138477_14.6	1	p2	(GT)7	14	32	45	TTGTGAGTGACTGCGTGTGA
scaffold138478_16.1	1	p2	(AT)7	14	401	414	ACGGTCGAGAAAGAGTCGTG
scaffold138523_14.7	1	p2	(AG)7	14	313	326	GGTCCTTGAACAGGACAGGA
scaffold138569_11.3	1	p2	(AT)6	12	1608	1619	CCCATCCTCATTTTTCTTTCA
scaffold138639_10.4	1	p2	(TA)6	12	194	205	CACGTGTCAGCCGAAAGATA

scaffold138708_10.	1	p2	(AG)6	12	212	223	GAATTTTTCGGGTTTTGGGT
scaffold138744_14.	1	p2	(AG)6	12	51	62	CCCATGTTGTAATTAATGTAGGGA
scaffold139049_13.	1	p2	(TA)7	14	115	128	CCTTAAATCAATCCCAGCCA
scaffold139129_15.	1	p2	(TA)8	16	872	887	CTCCCACGCACACATACATC
scaffold139144_10.	1	p2	(AG)6	12	222	233	TGGTTACTGTTCCTCAGCCC
scaffold139226_15.	1	p2	(CT)8	16	515	530	TTCTCAGTTCTCCCCTCAAA
scaffold139487_12.	1	p2	(TC)6	12	172	183	TTCACGTGGACTGCTTTCAG
scaffold139578_12.	1	p2	(TA)7	14	234	247	ACTCCGTTGAATCTTGCCAT
scaffold139640_10.	1	p2	(TA)6	12	317	328	TCAGGGGCATATGTGTCAAA
scaffold139797_11.	1	p2	(GA)8	16	72	87	GTCCACGGTGGTTTGAGAGT
scaffold140267_12.	1	p2	(CT)8	16	590	605	CCAAGTTTGGGACTTAAGCG
scaffold140311_14.	1	p2	(AG)7	14	281	294	TATATTGTGGGGTGGATGGG
scaffold140332_13.	1	p2	(AT)6	12	754	765	CCAGCATTGTTTCCCATTTC
scaffold140346_15.	1	p2	(CA)6	12	124	135	AATATCCGATCCCTTGGAGC
scaffold140372_11.	1	p2	(AT)9	18	429	446	CCACATTTGAAGGTGATCTTTC
scaffold140654_10.	1	p2	(AT)6	12	116	127	CCAATCTAGGGTTCATCACCA
scaffold140657_12.	1	p2	(AT)6	12	395	406	CGAACGAATATGTTGCATCC
scaffold140788_19.	1	p2	(AT)6	12	112	123	AACTACGAGCATCATCATGTGTTT
scaffold140801_14.	1	p2	(AG)7	14	100	113	TCCACTGCCAATGAAATGAA
scaffold140806_10.	1	p2	(AT)11	22	757	778	GATTGGATGAGAAGAACCGC
scaffold140823_20.	1	p2	(TC)6	12	245	256	TTTGTCGGGTCATTACTATTACGA
scaffold140863_16.	1	p2	(CT)10	20	186	205	AGATCATCTCGGGTCCAAGG
scaffold140891_16.	1	p2	(AT)8	16	2916	2931	ACGGTCCGAGTTCATAGGTG
scaffold140953_14.	1	p2	(TA)10	20	109	128	ACCATGTTTCCATCGGTTGT
scaffold141180_14.	1	p2	(GA)6	12	117	128	TCTCTCTCAAACGCATGTGG
scaffold141436_14.	1	p2	(AG)8	16	93	108	TCTCTCTCTGACGCCATTCA
scaffold141497_14.	1	p2	(GA)6	12	32	43	TGAGTAAAACGTGTTTGTCAGAA
scaffold141554_18.	1	p2	(CT)7	14	138	151	GGATCGAAAAGAGAGGGAGG
scaffold141808_16.	1	p2	(CT)6	12	113	124	TCTGCTACACCCAAAATCCC
scaffold141860_12.	1	p2	(TC)6	12	190	201	AATGGATGAAAAGCTGCCAC
scaffold141889_12.	1	p2	(AT)7	14	610	623	AGCCTTCAGATCCCCTGTTT
scaffold142090_15.	1	p2	(TG)6	12	498	509	TGCAAAATACCTTTTGTCGG
scaffold142252_15.	1	p2	(AT)6	12	3169	3180	TGGAGTCCTAAACTAAACCTCTAAAA
scaffold142265_10.	1	p2	(AC)6	12	173	184	GCTGCAATTCCTATTCAGCA
scaffold142291_17.	1	p2	(CT)10	20	6690	6709	ACCCTATTTTGGCGTAGCCT
scaffold142434_16.	1	p2	(AT)9	18	46	63	TCTTGATAGTACTTTTCATGGAACTTT
scaffold142840_16.	1	p2	(AT)7	14	4138	4151	ATTGTTGCTTGAATGAGGGC
scaffold142854_11.	1	p2	(AT)7	14	64	77	TTTTATTTTTGTGTGGTTTTGATTT
scaffold142899_16.	1	p2	(GA)6	12	316	327	AGAGATCGACGCCTTGACAT
scaffold142945_12.	1	p2	(TA)7	14	1263	1276	CATATCAGCCAAAGGGAGGA
scaffold142970_11.	1	p2	(GA)7	14	50	63	AAATGCAGGGAAGATTGTGG
scaffold143391_14.	1	p2	(GA)6	12	848	859	GGAACATGTGTGTGCTCCAG
scaffold143536_11.	1	p2	(GA)6	12	107	118	CCGAGATGGCTGCATTAAA
scaffold143599_16.	1	p2	(TA)6	12	1213	1224	GATTCGTGTCGGATTAGCGT
scaffold143600_13.	1	p2	(AT)7	14	49	62	TTGCACAGTACATGCGTTCA
scaffold143614_14.	1	p2	(AT)10	20	1100	1119	ATTCTGAGCTGGCGTGAAAG
scaffold143806_15.	1	p2	(AG)8	16	172	187	GGTGGAGTTGGTTCGAAGTT
scaffold143875_12.	1	p2	(AG)7	14	170	183	TGAAAAACAGAAACCGAGATCA
scaffold143997_13.	1	p2	(TC)6	12	26	37	GGGATGCCATTCGTAAGTCC
scaffold144094_17.	1	p2	(CT)8	16	112	127	CGCTAAATTTGATCGTCTTCC
scaffold144329_10.	1	p2	(CT)12	24	46	69	CACGTTGGACTTTGGATTGA
scaffold144450_12.	1	p2	(AT)6	12	204	215	GAAGGCAGATGGACGGTAGA
scaffold144549_12.	1	p2	(TA)8	16	488	503	CCTTCTTTGAATAGCGTGCC
scaffold144565_10.	1	p2	(AT)10	20	328	347	CCCCCTCTTCGAACTTCTCT
scaffold144585_15.	1	p2	(AT)6	12	1568	1579	CCCGAAAGGTTTGTTTCTGA
scaffold144594_14.	1	p2	(TC)7	14	1108	1121	AACTCCACTGCCATCTCACC
scaffold144598_18.	1	p2	(AG)6	12	1117	1128	GCATCAGCATGCTCAACAAA
scaffold144600_16.	1	p2	(AG)9	18	852	869	ACGGTTGTGATCATTCGTCA

scaffold144618_12.5	1	p2	(CA)9	18	67	84	GCACTCGCCACAATGTCA
scaffold144634_13.4	1	p2	(AT)10	20	849	868	CCCTTCTTCAAAAAGTCCGA
scaffold144635_14.1	1	p2	(GT)7	14	122	135	TTTTTGAGATTAGGCTATAAGGAATTA
scaffold144817_14.1	1	p2	(AG)6	12	303	314	AGTTTTCTCCCCTTCGTTGC
scaffold144838_16.8	1	p2	(TA)6	12	635	646	ACATGATGGACAAGTCGCAA
scaffold144868_15.9	1	p2	(CT)6	12	277	288	AGGGACCTAATATGCCCACC
scaffold144915_13.5	1	p2	(GA)6	12	345	356	GGATTCGAAAAGACATCCCA
scaffold144924_13.8	1	p2	(AG)9	18	375	392	GGCCTATCGATGTCGGTAAA
scaffold145058_16.0	1	p2	(CT)7	14	821	834	TCTGTACGTGTGTGAATGGCT
scaffold145073_13.4	1	p2	(TA)8	16	281	296	GGTGGGGTTACATTGGAGAA
scaffold145227_13.6	1	p2	(AG)6	12	955	966	TATTACAGAATGCACCGCCA
scaffold145254_13.8	1	p2	(CT)6	12	1778	1789	GATTGAAGGGGTGCTTGAAA
scaffold145382_16.8	1	p2	(GA)7	14	110	123	AAACACGCAACACAAAGCAG
scaffold145391_11.1	1	p2	(TA)9	18	47	64	ACCATCCCACCAGCATTTAG
scaffold145398_14.5	1	p2	(TA)7	14	409	422	GAGCCTCTGCTATTGGCATC
scaffold145462_17.8	1	p2	(AT)6	12	2672	2683	CCCAGCCAATGGAGATTACA
scaffold145663_14.7	1	p2	(CT)7	14	366	379	GCAGCTGAGGTGCATAACAA
scaffold145674_15.3	1	p2	(TA)7	14	115	128	TGAACATGCTCCTATTTTAATACACC
scaffold145867_14.4	1	p2	(AT)7	14	609	622	AAAATGCCCTTGTATGCTGC
scaffold145894_16.7	1	p2	(AG)6	12	391	402	ACAGCAAAATTTGGCAGAGG
scaffold146060_13.7	1	p2	(TA)6	12	398	409	CCAACGCTACTTGGCCTTAC
scaffold146115_16.0	1	p2	(AT)7	14	218	231	TGCATTCTTGAAAGCTGGTG
scaffold146234_17.3	1	p2	(CA)7	14	1019	1032	GTAAGAACGAAAGCAGGCCA
scaffold146402_14.1	1	p2	(CT)7	14	376	389	AGTCACTCACCAGTCCCCAC
scaffold146485_16.1	1	p2	(TC)6	12	346	357	TGACATCTTTTGATTTGTTTTGC
scaffold146534_10.4	1	p2	(CG)6	12	236	247	ATAGTGGTATGGTGTGGGGG
scaffold146662_14.0	1	p2	(CT)6	12	3679	3690	AGGGCAATACGTTTTTCGTG
scaffold146665_14.5	1	p2	(TC)9	18	286	303	GCATGCTTCTGACCAATTCA
scaffold146887_15.8	1	p2	(CT)7	14	76	89	ATTACAAATGGCCAAGAGGG
scaffold146904_10.2	1	p2	(CT)11	22	599	620	AATAATCCACGCCAAACTGC
scaffold146937_12.2	1	p2	(TA)6	12	557	568	GAGAAAGCATGTTCTGCACG
scaffold146994_13.5	1	p2	(TC)6	12	173	184	TAGTTGGGCTTAGTGGGGGT
scaffold147222_17.4	1	p2	(TA)6	12	175	186	ATGCCATCTGCAATCATCAA
scaffold147274_14.8	1	p2	(GA)8	16	1269	1284	AGGGCTGAGAGCCTGAAACT
scaffold147313_14.5	1	p2	(TC)6	12	53	64	ATGAGAATGGCCGAGAGTTT
scaffold147451_15.9	1	p2	(TC)7	14	386	399	CCTCTCAACGCTTGGCTAAC
scaffold147458_13.8	1	p2	(TA)9	18	171	188	TCTTTGTCCGTTTTTCGTGA
scaffold147622_14.2	1	p2	(TC)6	12	117	128	TGAGGGGTGCCAAGACTTTA
scaffold147638_13.9	1	p2	(AT)8	16	67	82	GTGCCGTAATAAATTGGATCA
scaffold147689_10.7	1	p2	(AT)6	12	65	76	CGTTAATGCACACAGCTTCAA
scaffold148474_12.5	1	p2	(GA)7	14	335	348	TATTGATGGTGCAGCAATGG
scaffold148539_11.0	1	p2	(AT)8	16	78	93	CACCAAAAACACACATTGGA
scaffold148550_13.8	1	p2	(TC)6	12	114	125	GTCAACTTTGGCAACGGAAT
scaffold148606_18.3	1	p2	(CT)7	14	171	184	TCCCATAGCATCTTTTGCTCT
scaffold148639_11.0	1	p2	(TA)7	14	566	579	TTTTGGGCAAAAATACCCCT
scaffold148722_16.4	1	p2	(TA)8	16	1658	1673	ACCGCCATATTTGGTTGACT
scaffold148784_15.7	1	p2	(AG)6	12	1567	1578	GGTGTGACTGAAGTGATTTGCT
scaffold148963_18.1	1	p2	(CT)6	12	71	82	TGGTACAAAATTTAGGGTTATTACAGA
scaffold149080_10.5	1	p2	(AT)10	20	543	562	GAGATTGACGGCTGGAAAGT
scaffold149123_11.9	1	p2	(CT)6	12	1026	1037	CGCATACCACATATCATCGC
scaffold149304_15.1	1	p2	(AT)6	12	429	440	TGGCTCCAACTTGTGTTTTG
scaffold149368_10.7	1	p2	(TC)6	12	67	78	TACCAACCCAACCCACTCAT
scaffold149466_14.5	1	p2	(AG)6	12	1110	1121	CGATTCGAAGAAATGCATCA
scaffold149488_13.1	1	p2	(CT)6	12	114	125	CAAATTCCGAAATTCTCCCA
scaffold150017_14.1	1	p2	(GA)6	12	410	421	CATTCTCAAGCCACTCCCTC
scaffold150168_11.7	1	p2	(AT)9	18	46	63	TGGGTGAGAAAGAGAGATAGAGA
scaffold150331_14.3	1	p2	(AT)6	12	241	252	GCAAAAGTAATAAAGAGCGACACA
scaffold150397_17.3	1	p2	(CT)7	14	49	62	GGAAATTTTCTTTCTTCCTCACG

scaffold150477_18.	1	p2	(AG)7	14	1599	1612	CCATTACTCACCCCCACAAA
scaffold150496_15.	1	p2	(AT)9	18	909	926	GCTCAGGACCGATGAATGAT
scaffold150509_10.	1	p2	(AT)7	14	116	129	TGTAGCCGGAATATGAGCCT
scaffold150651_15.	1	p2	(AC)7	14	2638	2651	TGGACTGTGGGAATACTGGA
scaffold150785_12.	1	p2	(TA)8	16	67	82	TTCGATGATAACTATGGGTTTTG
scaffold150787_11.	1	p2	(TA)9	18	79	96	TCGAAATTTGGCAAACAATCT
scaffold150796_12.	1	p2	(AT)9	18	673	690	GCCTATCCGTTACCCTCCAC
scaffold150813_15.	1	p2	(TA)8	16	5265	5280	CGGTTCGGATTACGGTTTTA
scaffold150856_15.	1	p2	(GA)8	16	1127	1142	CATTGTTTGTGTGTCGGAGG
scaffold151144_17.	1	p2	(TA)8	16	43	58	ATCGGTTATTACCGGATCGC
scaffold151270_13.	1	p2	(TG)6	12	59	70	TTGAATTAATGATGTCAGATATTGTTG
scaffold151488_14.	1	p2	(AT)6	12	199	210	AATTTTCTACAAAACAAGACCAATC
scaffold151535_14.	1	p2	(AC)6	12	333	344	TTGCTCTAAGCTTCATCCGC
scaffold151586_16.	1	p2	(GA)6	12	357	368	CCTCCGATTTCGTCTTCAAA
scaffold151648_15.	1	p2	(AT)6	12	72	83	TCCGAAAATCGGTCAAAAAC
scaffold151895_11.	1	p2	(TA)7	14	335	348	TGCTCTTATGCTTTGTTAAATTAGG
scaffold151909_15.	1	p2	(TA)6	12	1180	1191	CAAGCTATTTCGAGGCAACA
scaffold151962_15.	1	p2	(CT)7	14	41	54	TGAAAGTAGCCGAGAGTTTACAA
scaffold152054_11.	1	p2	(TA)6	12	374	385	CCCCATCCAAGAAAAGATCA
scaffold152099_15.	1	p2	(GA)7	14	214	227	TCGAGAAATGCCACATGCTA
scaffold152389_17.	1	p2	(GA)9	18	804	821	AAAATCACCGCGAAAAATTG
scaffold152474_14.	1	p2	(TA)12	24	41	64	ACCATATTTAAGGGTTATCACCTG
scaffold152541_12.	1	p2	(AT)8	16	384	399	GGATTAATAGGCTGCCCCTC
scaffold152609_16.	1	p2	(TA)6	12	207	218	TGCCAAGTCTAAAGCCACTG
scaffold152773_11.	1	p2	(CT)8	16	370	385	TCCAACTTGTTCCCTCATTTG
scaffold152781_15.	1	p2	(TA)6	12	82	93	ACAATATTTAACGCAGCTAAACG
scaffold152796_15.	1	p2	(TA)10	20	2427	2446	AGGCCTACCGATCCAAACTT
scaffold153004_19.	1	p2	(AT)7	14	560	573	TCCCACTCGCCTGATACCTA
scaffold153193_18.	1	p2	(TC)6	12	168	179	TCATTCTCTCCCTCATTTTTGAA
scaffold153246_16.	1	p2	(AT)8	16	241	256	CCCTAAATTTTGTATCCGAATG
scaffold153417_13.	1	p2	(TA)9	18	1455	1472	TTTTAGAAGTAAGTGGTGTTCGG
scaffold153569_16.	1	p2	(CA)6	12	1386	1397	CATGCAGGAATTTTCACCAA
scaffold153614_17.	1	p2	(CT)6	12	68	79	AGCATCTCTTGCTCTCTCATTG
scaffold153636_11.	1	p2	(AG)9	18	324	341	ATTTCCTTTCAATGGGGGAC
scaffold153643_15.	1	p2	(TA)6	12	523	534	GGCGGGGGAGATATAGAGAG
scaffold153665_12.	1	p2	(AT)6	12	185	196	GCATGCTCTATCTGTTACAGGG
scaffold153666_12.	1	p2	(TA)8	16	79	94	GATTGCCCAAGATCTACGGTT
scaffold153685_13.	1	p2	(CA)6	12	832	843	TGCAGATGTCCTCCTTTCCT
scaffold153692_13.	1	p2	(AG)10	20	405	424	TGCCATCTCGAAAAACAATG
scaffold154134_15.	1	p2	(AG)7	14	99	112	TGGAATCCGAGAAATTCAGG
scaffold154251_12.	1	p2	(GA)6	12	37	48	GACTTAAATTGCTGCAGACGC
scaffold154489_14.	1	p2	(TA)8	16	232	247	TGCACTAACCACCCTTGATTT
scaffold154687_12.	1	p2	(TA)8	16	1103	1118	ATTAGGGTTGAGGTTGACCG
scaffold154694_14.	1	p2	(GA)6	12	153	164	CACTAGGCCTACGTCGCATT
scaffold154719_15.	1	p2	(AG)10	20	360	379	GAATGCTGGGCTGATAAGGA
scaffold154724_12.	1	p2	(TA)9	18	257	274	TTCTCTCTGGTTGGCTGCTT
scaffold154962_15.	1	p2	(AG)6	12	1627	1638	TGGCAATGGAAAGAAACACA
scaffold155233_15.	1	p2	(TC)6	12	39	50	GGCAACGGAATACCATTTGT
scaffold155274_14.	1	p2	(AT)8	16	3561	3576	CAACCATCCAACCACAACAA
scaffold155476_14.	1	p2	(GA)9	18	517	534	TTGCTCTTGTCGACATGGTT
scaffold155617_12.	1	p2	(AT)8	16	151	166	TGCAACCTCAATGCTTCTCA
scaffold155618_12.	1	p2	(TA)8	16	846	861	TGAATCCGTATCCTTATTGTTGC
scaffold155625_11.	1	p2	(TA)7	14	140	153	TGTCAACATTTTGTGCATTGG
scaffold155659_11.	1	p2	(TA)7	14	52	65	TGATTGGTCAGTTTGTCCGA
scaffold155763_13.	1	p2	(CT)6	12	180	191	CCCAGCTCTCTCACTCTGCT
scaffold155795_11.	1	p2	(AT)7	14	1960	1973	TATGGAGTGGAGCAAAACGG
scaffold156140_10.	1	p2	(TA)12	24	311	334	TTTAAACCCTAAAACGCGCA
scaffold156367_15.	1	p2	(AT)6	12	2418	2429	TCACCTAATTGCATCCCCAT

scaffold156398_14.5	1	p2	(AG)6	12	1417	1428	AGAGAGAGGCGATGCAAAAA
scaffold156563_14.9	1	p2	(AT)9	18	727	744	CAAAACTAATTGGGGAGCAAA
scaffold156607_14.7	1	p2	(GA)6	12	3519	3530	CGGATAATATAGCTCCGGCA
scaffold156718_14.0	1	p2	(TA)6	12	1098	1109	CATTCATCATCATCAATATCACAGTC
scaffold156806_15.9	1	p2	(AT)8	16	85	100	AATCTGACGGCCGATATTTG
scaffold156819_12.0	1	p2	(TA)7	14	1141	1154	TCAGATCTGCGCTGAAAATG
scaffold157076_15.9	1	p2	(CT)7	14	151	164	GTGTGCATGCATGGTTTAGG
scaffold157090_11.7	1	p2	(AT)9	18	132	149	TTAGCCCTTAATGGCCTGCT
scaffold157091_16.0	1	p2	(AG)6	12	841	852	TGGTGACTTTGAGGAGGGAG
scaffold157159_12.9	1	p2	(AT)11	22	894	915	AGTGAGGCCCCTTTTACGTT
scaffold157268_13.6	1	p2	(TA)6	12	111	122	AATTGAAATAAAGGCGCCAG
scaffold157292_14.8	1	p2	(AT)6	12	1028	1039	AATCGTGGTTCAAAAATCGG
scaffold157305_13.1	1	p2	(AT)9	18	308	325	TTCGGTTGCAATTTTAGTTGTTT
scaffold157428_13.4	1	p2	(TA)8	16	1065	1080	CAGGGGCATATGTGTCAAAA
scaffold157502_11.4	1	p2	(AG)8	16	284	299	AAGTTGGAGTGGAGGTGTCG
scaffold157515_10.5	1	p2	(AT)8	16	319	334	ATTTGAAACCGGACCGAACT
scaffold157516_13.0	1	p2	(AT)8	16	146	161	GGTCCATTGGTCTTTTGTATTGA
scaffold157555_12.7	1	p2	(CA)6	12	824	835	GATACGACCTGTAATGCCCC
scaffold157729_16.2	1	p2	(AT)6	12	129	140	TCATGGGGCAATGGGATA
scaffold157754_13.8	1	p2	(AT)8	16	2216	2231	GTGGTGGAAGAGGCAATGTT
scaffold157829_12.0	1	p2	(AT)7	14	555	568	AATAGGTCCATCCCTGCCTT
scaffold157849_14.7	1	p2	(AT)7	14	376	389	AAAAGGAGTTTTGACGGGATT
scaffold157932_13.6	1	p2	(TA)9	18	289	306	GTAAATAAGGCGGTGGGTGA
scaffold157942_10.0	1	p2	(CT)6	12	63	74	AAAAGTAATGCATATGTTTTCCTAAGT
scaffold157980_16.9	1	p2	(TA)9	18	47	64	CATGCACCTGAACATGCC
scaffold157989_11.5	1	p2	(AT)6	12	486	497	TGGTTGAATTTCGTACCCGT
scaffold158113_11.5	1	p2	(TC)6	12	54	65	CCTACCCAAACCCACTACCA
scaffold158303_14.8	1	p2	(CT)6	12	275	286	AAGCACTCAACCAAACACCC
scaffold158405_10.3	1	p2	(AG)6	12	605	616	TGATGGTTTGATGGGGTTTT
scaffold158455_19.1	1	p2	(TA)9	18	301	318	ATAAATGTTCAAACCGGCCA
scaffold158511_16.6	1	p2	(TC)6	12	479	490	GCACCGAGGGAATCAAATAA
scaffold158537_12.7	1	p2	(GA)6	12	220	231	CAAAGAAGAGTGGGCTTTGG
scaffold158730_13.6	1	p2	(AT)10	20	2214	2233	GTCAATCCAACGTCCCAAAT
scaffold159213_16.3	1	p2	(AG)7	14	272	285	CCTCTCGCATTTCTCTTGCT
scaffold159314_13.3	1	p2	(TA)6	12	246	257	AATCACATCAACACCTCAGCC
scaffold159372_12.6	1	p2	(AG)9	18	311	328	TCCCCTACTACACTCGCCAC
scaffold159433_12.7	1	p2	(TC)6	12	628	639	AGCAAATCCTCCCCAAATCT
scaffold159750_14.9	1	p2	(AG)10	20	2049	2068	CATCGCCCTTGATCTCTCTC
scaffold159899_13.6	1	p2	(AT)7	14	2248	2261	ACTAGAGGGGTCAGCCCATT
scaffold160005_12.4	1	p2	(AC)7	14	425	438	AACCACATTAGGCCATGCAC
scaffold160181_16.2	1	p2	(AG)10	20	490	509	CAACGTCGCTTTTAGGGTTG
scaffold160267_12.2	1	p2	(AT)7	14	115	128	TATGCATTTGCACCTGAGGA
scaffold160303_13.7	1	p2	(AG)6	12	664	675	AGGGGTGTAGGCAAAAGACC
scaffold160488_13.9	1	p2	(AT)7	14	2704	2717	TAGGGCAATTGGTAGGTTCG
scaffold160560_15.9	1	p2	(CA)6	12	581	592	CTTCCAGAGTCAATCCAGGC
scaffold160605_15.0	1	p2	(AG)6	12	139	150	GCTTCCAAAAGAAAGAGCCC
scaffold160831_16.1	1	p2	(TA)10	20	430	449	TGTTTCCCGTTTTATCAGCA
scaffold160961_10.7	1	p2	(TA)6	12	75	86	TCATTTATGAAGCAAGGGGG
scaffold161261_14.1	1	p2	(AG)7	14	290	303	TGCAAATTGATGAGGCCATA
scaffold161421_14.9	1	p2	(CT)8	16	67	82	CAACCCTACCTCCTTCTCCC
scaffold161559_13.7	1	p2	(TC)6	12	2167	2178	GGTGGCAAAAAGTGAGGAAA
scaffold161750_14.2	1	p2	(AG)7	14	2721	2734	TATAGGGCGGTGGTAGTTGC
scaffold161818_15.6	1	p2	(GA)6	12	797	808	TGGTGAAGATGTGGGTTGAG
scaffold161932_14.0	1	p2	(TC)7	14	1459	1472	GCACGTAACATACATTGCCC
scaffold161946_15.0	1	p2	(GA)10	20	748	767	GGCTCACTGCTTCTTTCTCG
scaffold161969_19.0	1	p2	(TA)7	14	117	130	CAATTGGTAGCAAACCATGAA
scaffold161992_15.1	1	p2	(CT)6	12	56	67	TTTGAAAACCCCATTTCGTC
scaffold162156_13.8	1	p2	(TA)10	20	43	62	CCCAAAAATGGGTTATTATTTGA

scaffold162356_10.3	1	p2	(TA)15	30	447	476	CACCGTTGTCTTTAAGCCGT
scaffold162372_16.6	1	p2	(TC)6	12	852	863	GCTGATCTCCATCTCCAAGC
scaffold162517_16.0	1	p2	(AT)6	12	286	297	TTGGGAAAAATAAAGAGGAAACC
scaffold162570_12.8	1	p2	(TC)6	12	219	230	AAATCCCACCTACCCAAACC
scaffold162670_12.7	1	p2	(TA)6	12	828	839	GCAATCCCCACTGCATCTTA
scaffold162709_12.6	1	p2	(TA)7	14	497	510	CCCTCCCTCTCTCTTTTCGT
scaffold162802_15.3	1	p2	(TA)7	14	1752	1765	TCCTAATTTGGAAAGTACTGGGTT
scaffold163064_12.9	1	p2	(TC)6	12	132	143	GCTCGAAATCGGAAAATCTG
scaffold163117_13.5	1	p2	(CT)6	12	46	57	GTTGGCTGGATCTCCTGAAA
scaffold163260_13.6	1	p2	(AT)11	22	213	234	TCATTCCGAAAATCAAAGGC
scaffold163280_13.8	1	p2	(TG)6	12	226	237	GCATTTAAGTTTACGGGGGA
scaffold163287_15.0	1	p2	(TA)10	20	456	475	TGCACGTTTCAAAGCATAGG
scaffold163360_10.4	1	p2	(TA)7	14	566	579	TCTCAGAATCCCATTAGAAGGGT
scaffold163625_11.4	1	p2	(AT)8	16	310	325	GAAATTGAAGGGATAAGAATTTCAA
scaffold163693_10.5	1	p2	(TA)6	12	371	382	ACCTGATTCCAGTTGGATGG
scaffold163908_15.9	1	p2	(TA)7	14	3076	3089	ACCGATCTTTTGGCATTCAA
scaffold163927_15.7	1	p2	(TC)7	14	581	594	AAATCCTATCCGGTCAACCC
scaffold163959_13.0	1	p2	(AG)6	12	227	238	GGGTGAAAGGAGAGGGAGAG
scaffold164119_16.3	1	p2	(AT)8	16	3020	3035	TTGTCGGGCTCGAATTTATC
scaffold164141_13.6	1	p2	(AT)9	18	873	890	CCCATACACAATTTGGGGTC
scaffold164177_13.0	1	p2	(TG)8	16	181	196	ATCTCGGCTACGAAGTGCAT
scaffold164195_13.6	1	p2	(AT)6	12	342	353	GCTTGGTGAAGGAAGTGAGG
scaffold164259_16.8	1	p2	(AT)6	12	53	64	GTCCTCCGAACTCATGGTGT
scaffold164403_16.5	1	p2	(AT)6	12	508	519	TTACCCGGACAATAAATCCG
scaffold164476_14.6	1	p2	(AT)12	24	1265	1288	GCTTCTTCTTCTCCGCATTG
scaffold164500_13.4	1	p2	(AG)9	18	997	1014	TCCCATTCAACCATTTTCAA
scaffold164622_15.2	1	p2	(AT)6	12	196	207	CAACGCACGGGTAAAAGACT
scaffold164636_15.2	1	p2	(AT)7	14	763	776	GAGCATTTTCATGCCATTTTT
scaffold164668_12.2	1	p2	(TA)7	14	51	64	GCACACAAGTTATTGGTTTCTATTTG
scaffold164671_10.2	1	p2	(AT)10	20	198	217	TTTTACTGCCTTGTGTTGCG
scaffold164825_12.9	1	p2	(TA)9	18	292	309	AATTGTGGAGTTGCCGTTTT
scaffold165037_11.4	1	p2	(TA)10	20	95	114	ACCAATTTGATGAGGGTCCA
scaffold165231_15.1	1	p2	(AT)9	18	167	184	TTCTGGATTATCCTGCGGTC
scaffold165239_11.1	1	p2	(AG)6	12	271	282	GTGTCGGGGGTACAAAAGTG
scaffold165276_15.8	1	p2	(GC)7	14	75	88	AAAATTATATTCTCGCTTCTTCGC
scaffold165283_17.5	1	p2	(TA)8	16	181	196	AAAGCCTACCTTGCTTCCGT
scaffold165428_13.1	1	p2	(TA)7	14	285	298	TGTCGGAAGCTCAACACTTG
scaffold165437_13.5	1	p2	(GA)7	14	145	158	CGCGTTTTTGGGGTTAAATA
scaffold165564_13.8	1	p2	(TA)8	16	286	301	AAGGTGGGAAAGAATTTGGC
scaffold165753_12.8	1	p2	(TA)7	14	376	389	CGAAATTGAATGGCAAGGAT
scaffold165755_13.2	1	p2	(TA)10	20	385	404	ATCGAGGCGTGACATCTACC
scaffold165819_10.4	1	p2	(AT)10	20	196	215	CGGAAGAATAAGAAACGGCA
scaffold165857_11.3	1	p2	(TA)6	12	262	273	TCAAAAGTGCAACAAATCTGC
scaffold165998_15.5	1	p2	(AT)7	14	682	695	GGAATGTAGTGTGCGCAAGA
scaffold166171_14.9	1	p2	(TC)6	12	184	195	TGAGCTCGGACCTCCAGTAT
scaffold166249_15.4	1	p2	(AT)7	14	167	180	TGCAACGAACAAATCTCATTG
scaffold166484_14.3	1	p2	(AT)8	16	726	741	CGTCAATCAATGATGCGATTA
scaffold166540_16.0	1	p2	(TC)12	24	2259	2282	AGTTTTGGACGAATTCCACG
scaffold166703_15.0	1	p2	(AT)6	12	925	936	ATCCAACACCCACCACATTT
scaffold166787_10.6	1	p2	(AG)6	12	66	77	ACTCGGAACAACGAAAATCG
scaffold166871_11.3	1	p2	(TA)6	12	213	224	GGCCATGATTCATTTTCTGC
scaffold166895_12.3	1	p2	(AT)6	12	324	335	TTCCATTTCAGACCCAAAAGA
scaffold166935_15.8	1	p2	(GA)6	12	2212	2223	CGAAAATTGAAAATTTATTCCCC
scaffold166938_15.1	1	p2	(AT)13	26	1314	1339	TCATGGACCTCATATGTCATAACC
scaffold167156_13.0	1	p2	(AT)6	12	212	223	CGTATACTTGCGGGGTTGTT
scaffold167161_18.7	1	p2	(AC)8	16	115	130	AAGTCTCTCACCCTTCGCAA
scaffold167215_16.3	1	p2	(CT)6	12	157	168	TCGATGTTGTAATTCATCCGAG
scaffold167506_10.5	1	p2	(CG)8	16	1164	1179	TATGCCCATGGAGGTATGGT

scaffold167528_16.9	1	p2	(TC)7	14	1237	1250	GCATCCATTCTGAATCACTCAA
scaffold167619_17.1	1	p2	(GA)6	12	229	240	GAGTCAGCTGCTCTTGGCTT
scaffold167624_15.8	1	p2	(CT)6	12	302	313	CATGGATTCGGGTAAACACC
scaffold167675_10.0	1	p2	(TA)10	20	45	64	AATTTACTAATTTATCCACCTCGATTT
scaffold167759_10.9	1	p2	(CT)6	12	151	162	ATGAGGTGGCATGTAGACCC
scaffold168086_10.7	1	p2	(TC)6	12	317	328	TCTCCTTCTCTCCCCCATTT
scaffold168186_16.5	1	p2	(TA)6	12	1098	1109	TTGCCGACCTTATCTCCATC
scaffold168314_11.2	1	p2	(AG)6	12	217	228	CGACATTGAGTAGCCGTCTG
scaffold168373_17.1	1	p2	(TC)10	20	67	86	GCTCTTCGAATTCTCCTCCC
scaffold168587_14.2	1	p2	(TA)8	16	51	66	AAAACCGTTCTCACCCCTTC
scaffold168626_17.6	1	p2	(TA)11	22	196	217	ACGGTTAAGATTCAGCACGC
scaffold168975_11.6	1	p2	(CT)7	14	170	183	ACTTCAATTTTCGAGCCCAA
scaffold169133_10.0	1	p2	(TA)10	20	66	85	CATGTTAAATAATCGTCAAGTTGAGT
scaffold169243_10.2	1	p2	(TA)8	16	411	426	TGTCGAATGCTAATGGGAGA
scaffold169520_15.0	1	p2	(AG)12	24	216	239	TTTTCTTTTTCCCGGTGATG
scaffold169548_11.4	1	p2	(GA)6	12	209	220	TTCGTGTCTATCCTCACCCC
scaffold169595_17.6	1	p2	(TC)6	12	733	744	TGATCGTTCTTCACTCTCTTGC
scaffold169652_12.3	1	p2	(AT)8	16	717	732	CCAGATCGCGACCCTATAGAT
scaffold169687_10.2	1	p2	(AT)8	16	198	213	AAAATCAAAGGCCCACAATG
scaffold169790_13.8	1	p2	(TA)7	14	277	290	TCATTAGCAGCTGGGGATTT
scaffold169889_11.3	1	p2	(CT)6	12	176	187	CATAGTTGACGCCCTTCCTC
scaffold170158_10.7	1	p2	(TG)6	12	224	235	ATTTGGGTCAAACCCCTTTT
scaffold170328_14.4	1	p2	(TA)8	16	414	429	TTTTTACCAATTTGCCCTCG
scaffold170418_16.8	1	p2	(TA)7	14	320	333	GAGCTGTTACCTCCGTGGAC
scaffold170502_16.8	1	p2	(AG)7	14	286	299	AGACATGGCGATACGACGTT
scaffold170506_11.0	1	p2	(CT)6	12	118	129	GTGTCCAACATCCCATTTCC
scaffold170509_16.0	1	p2	(CT)6	12	84	95	AGCATCTCTTGCTCTCTCATTG
scaffold170605_13.8	1	p2	(AT)8	16	189	204	TATTTTGGACGATTTTGCCC
scaffold170672_13.6	1	p2	(AG)9	18	85	102	TTTTGTTGTTTCCCAAGTTGC
scaffold170974_10.9	1	p2	(TA)10	20	64	83	TTTGCTTGATCCAATGGTGT
scaffold170977_18.1	1	p2	(AT)7	14	117	130	TGCGTGATGCAGACATATTG
scaffold170979_17.2	1	p2	(AT)6	12	1684	1695	TGTCCCCATCTCGTGTTGTA
scaffold171007_15.2	1	p2	(TA)6	12	484	495	CATGCAGTCATGCACAATCA
scaffold171067_13.1	1	p2	(AT)7	14	702	715	TTTGACCTTGCATTGCTGTC
scaffold171092_13.0	1	p2	(TA)7	14	181	194	AAGGAGGTGTTATGAAGGGATT
scaffold171093_17.5	1	p2	(GA)6	12	224	235	AATTGATGGAAGAAGGCACG
scaffold171246_13.5	1	p2	(AC)6	12	180	191	CTCCATCGTCGAAGTCATCA
scaffold171255_16.3	1	p2	(TA)9	18	211	228	GTGCGTGTGTATGCTGAGGT
scaffold171486_16.1	1	p2	(TA)9	18	407	424	TGGCGCATTTTACTGATATTTG
scaffold171628_16.7	1	p2	(AC)6	12	208	219	CGCAACGTTCTGAAACTGAA
scaffold172005_12.5	1	p2	(CT)6	12	52	63	TCTTGCACTCGTCTCTGTCG
scaffold172073_15.1	1	p2	(AT)6	12	657	668	GGAAGGAATATTTCATCACACCTT
scaffold172121_12.8	1	p2	(AT)8	16	104	119	TCGATAATGCACTTTATCAGAGTT
scaffold172144_13.1	1	p2	(TA)8	16	281	296	TGGATGTGGTTGGTGAGTTG
scaffold172398_16.6	1	p2	(AT)6	12	638	649	GATGATCACGATGAAGCCAA
scaffold172559_11.6	1	p2	(AT)7	14	750	763	CATGCACGGAAAACTTGAAA
scaffold172844_12.7	1	p2	(AG)6	12	918	929	TTGGTTCAAGCCATTGTGAA
scaffold172854_13.6	1	p2	(GT)6	12	1914	1925	TGCGTTTTACAGCCAAAAGA
scaffold172948_15.9	1	p2	(TA)6	12	510	521	CGAATAGTAGAAGCTGCGGG
scaffold172974_20.1	1	p2	(AT)7	14	76	89	TTGGTGTATACTTTTGTAGTGATCAAA
scaffold173074_11.2	1	p2	(CT)6	12	161	172	ACCCGAATTTTTCGGATACC
scaffold173396_16.5	1	p2	(GA)7	14	32	45	GAGAATAGATAGTTAGAGAGAGACC
scaffold173428_12.4	1	p2	(AG)6	12	282	293	CTTCTCACCGTCGACCAAAT
scaffold173472_12.3	1	p2	(TA)9	18	247	264	TTCTCAGCATATGGGAAGGG
scaffold173508_18.3	1	p2	(CA)6	12	1523	1534	TGACTGGACTGCCTTTTTCC
scaffold173509_14.6	1	p2	(TA)8	16	126	141	TGAGCCTATCTTGGAGCACA
scaffold173530_13.2	1	p2	(AC)7	14	140	153	TTCTTTGAATCATCTTCCACCTT
scaffold173658_11.0	1	p2	(AT)6	12	267	278	ACTCGTGCTGGCAATCTTCT

scaffold173740_18.5	1	p2	(CT)6	12	106	117	AACAAAAATGGCCAAAATCG
scaffold173862_10.6	1	p2	(TA)6	12	479	490	CAACTCCACATGTTGCCATC
scaffold173933_14.6	1	p2	(AG)7	14	49	62	TTTTTAGCATGCGAGAGGAA
scaffold173949_11.1	1	p2	(GA)7	14	171	184	TCAACAACATGCAAGCATCA
scaffold173981_18.0	1	p2	(GA)9	18	471	488	GGATGAGAGACTCGGGTTCA
scaffold174269_13.1	1	p2	(GA)7	14	1289	1302	CCATTTCTTTGGCAAGTTGA
scaffold174392_10.2	1	p2	(GC)6	12	576	587	TCAGCCCAGCCCAATATTAC
scaffold174455_14.3	1	p2	(CT)11	22	771	792	GACATTTGGTTCAGCGCTTT
scaffold174523_17.0	1	p2	(AG)8	16	2585	2600	TGATCATCGAGGCAGAGAAA
scaffold174754_10.0	1	p2	(TC)7	14	169	182	GGGCAAAACTTGAACAGCTC
scaffold174974_14.0	1	p2	(CT)6	12	916	927	CCCTGTCTTAGGTACTGGCG
scaffold175116_11.3	1	p2	(TC)7	14	40	53	GCACAACCACTCCACAAAGA
scaffold175173_12.5	1	p2	(TC)6	12	741	752	TTCCTACTCCCCTAGGCCAT
scaffold175292_14.9	1	p2	(AT)6	12	334	345	ACCGTTCATTTTCGGTTCAG
scaffold175294_15.7	1	p2	(GA)6	12	309	320	TCGAAGGTGAACTCGGTCTT
scaffold175297_16.7	1	p2	(CT)10	20	578	597	CCCTAACTCGACCCAGATCA
scaffold175387_13.0	1	p2	(AT)8	16	49	64	AGAGTTCACCAAGGTACTTTAATATGT
scaffold175434_14.4	1	p2	(TA)8	16	1479	1494	TGGGCACATTTGAATCTTGA
scaffold175442_13.5	1	p2	(GA)7	14	925	938	CCAAACGAAGTGACTTGCAG
scaffold175544_11.0	1	p2	(TC)7	14	354	367	TAGCCGATAAAAATGGCACC
scaffold175572_14.2	1	p2	(AT)10	20	554	573	ACACACACTCACTCACTCCCA
scaffold175836_13.3	1	p2	(GA)6	12	188	199	GTTCCGGGAATCACACCATA
scaffold176052_12.0	1	p2	(TA)12	24	40	63	AAGGGTTATCACCTGATTTTCTC
scaffold176180_14.4	1	p2	(GA)10	20	663	682	AAGGATCTTGTTACGGGCCT
scaffold176280_14.6	1	p2	(GA)7	14	1884	1897	TTGACTGATTGGAGCAGTGG
scaffold176320_10.8	1	p2	(GA)7	14	177	190	TCAAATCTTCAACATGCAAGC
scaffold176511_10.8	1	p2	(TA)10	20	457	476	TTTTCTTGGACCAGATTGCC
scaffold176747_14.1	1	p2	(GC)6	12	425	436	CACGTGCGTCAACTCCTTTA
scaffold176848_11.8	1	p2	(GA)7	14	116	129	AAACACGCAACACAAAGCAG
scaffold177379_14.8	1	p2	(CA)7	14	806	819	AGCCCTGTAGAGGAGCATCA
scaffold177501_18.9	1	p2	(AT)8	16	337	352	TGGGGTTATCACTGGATCGT
scaffold177572_13.9	1	p2	(TA)6	12	590	601	TAAACCGTTGTTTTGGCTCC
scaffold177580_10.5	1	p2	(AT)8	16	212	227	CACCAAATCTGTTAGTTTACCAAA
scaffold178175_10.5	1	p2	(AG)6	12	368	379	AGATGCCCATTTTGATCAGG
scaffold178465_15.2	1	p2	(TC)6	12	586	597	CCCGATTATCGCAAGAACAT
scaffold178502_11.1	1	p2	(AT)6	12	311	322	TTTTTCACGCACAAGCTGAG
scaffold178523_13.4	1	p2	(AT)10	20	204	223	TTTGTGCTTTGCTGCTGTTT
scaffold178540_14.0	1	p2	(TC)6	12	119	130	CGATCTCCTCTCTCTCGTCG
scaffold178544_15.5	1	p2	(GA)6	12	52	63	CACGAGTGAAGAAAGGGAGAG
scaffold178585_14.3	1	p2	(TA)8	16	162	177	AATACTTTCCGTCACCGTCG
scaffold178813_14.9	1	p2	(CT)8	16	1249	1264	CCCATTCACAAGACCTCCTC
scaffold179106_11.0	1	p2	(AT)8	16	491	506	GAGGAAGATAAATGTGGGACG
scaffold179397_16.2	1	p2	(TA)8	16	258	273	TTTGGCCGAATTGTCTTTTC
scaffold179684_12.9	1	p2	(AT)6	12	856	867	AAGGGGAAGTTCTGCATGTG
scaffold179886_14.9	1	p2	(AT)7	14	67	80	CGTCAGAAACCTCTCGCATT
scaffold179887_17.7	1	p2	(AT)6	12	385	396	AAACATCCCAATCCCATCAA
scaffold179890_14.2	1	p2	(CT)8	16	147	162	GAACACGAGGGATATTGGGA
scaffold179959_12.1	1	p2	(TA)8	16	67	82	TGTGTTGTCTTTTAGATCAATGTTTG
scaffold180081_16.4	1	p2	(AT)6	12	4881	4892	TCAAGGGTAATTTTTCAGGCA
scaffold180421_12.7	1	p2	(AT)7	14	300	313	TAGTTAGCATGGGGGAATGC
scaffold180599_12.1	1	p2	(TA)6	12	115	126	TAATTTGGCCGGATTGTCTC
scaffold180618_14.2	1	p2	(TA)8	16	1888	1903	TGCGCGTTGATTAATCTCAA
scaffold180702_19.0	1	p2	(AT)8	16	1094	1109	CCTCCAAGCACAGGTACAGG
scaffold180784_12.8	1	p2	(TA)6	12	305	316	CCACTTAGGAATTTCATCAAATGATAG
scaffold180893_14.7	1	p2	(AC)6	12	432	443	TGGACACAAGTATGGCTCTCA
scaffold180916_14.7	1	p2	(GA)9	18	1009	1026	TCAATCAAAACTGCACCCAA
scaffold180957_15.5	1	p2	(GA)7	14	1155	1168	ACAGATGAGCGAAAATTGGG
scaffold181000_15.4	1	p2	(AG)6	12	704	715	AGTGAACAACAATCCCCACC

scaffold181082_16.1	1	p2	(GA)9	18	187	204	CTGAAATTGGGATTTGTGGC
scaffold181160_12.3	1	p2	(AT)6	12	116	127	TGTAATGCTACAGTTCTTAATTTGGT
scaffold181185_13.8	1	p2	(TA)8	16	2272	2287	TAGTGGAAAACCCAACGACC
scaffold181192_15.7	1	p2	(GA)6	12	326	337	TCCCTAATCGACCTGACACC
scaffold181228_19.2	1	p2	(AC)10	20	641	660	GCAAACTGATTGACCCGAAT
scaffold181299_11.0	1	p2	(TA)9	18	69	86	GCTTAAACAAAATGGCATTAAAAA
scaffold181301_10.9	1	p2	(TA)6	12	36	47	ACGGTTAAGATTCAGCACGC
scaffold181377_11.3	1	p2	(AC)6	12	53	64	TCACGAAAACACACGCACTC
scaffold181378_16.9	1	p2	(TC)6	12	774	785	ATTGGGCATTTTGACGGATA
scaffold181525_14.7	1	p2	(TA)7	14	93	106	CAGTTATCACCGGATCACGA
scaffold181809_17.8	1	p2	(AT)9	18	152	169	CTCAACGGTATGCTCGGATT
scaffold181864_14.4	1	p2	(TA)6	12	344	355	GCACCCCGTATTTTAGGACC
scaffold182113_12.4	1	p2	(TA)7	14	1314	1327	AATCACAACTTCACCCGGAA
scaffold182478_14.8	1	p2	(AG)6	12	182	193	GCCCTTGCAGAGAAAAGAGT
scaffold182751_16.4	1	p2	(AT)6	12	176	187	CAACCTATAAATAAGATCTCAACCTG
scaffold182813_14.6	1	p2	(TA)6	12	810	821	CCTGCTGGTTCTTGCTTCTC
scaffold182903_13.3	1	p2	(TA)6	12	413	424	CATTTCGCACACAGAGATGC
scaffold182924_10.6	1	p2	(TA)10	20	571	590	CAAATTACCCCCGTATGCAG
scaffold182933_10.1	1	p2	(AG)8	16	303	318	TCAATGGTGACATTTGGGTG
scaffold182986_14.4	1	p2	(TA)7	14	691	704	CGGTTAGAAACCAGACCGAA
scaffold183028_15.2	1	p2	(TC)7	14	1284	1297	CATGCATTCAACCCTCTTGA
scaffold183053_13.7	1	p2	(AT)9	18	304	321	GGGCATAAATGCCAATTTTTA
scaffold183085_13.3	1	p2	(AT)7	14	207	220	TTAAACCTTTGTGCGGTTGC
scaffold183253_15.6	1	p2	(TA)9	18	520	537	TGTTCTGGTCCAAACCCAAT
scaffold183308_13.6	1	p2	(TA)7	14	782	795	CTTCGACCGGATGAATCCTA
scaffold183318_12.7	1	p2	(CT)11	22	812	833	TATGATGGAAAAAGGCCGAG
scaffold183512_14.9	1	p2	(AC)6	12	1002	1013	CCAGTGTAAAAAGATCGAGCAA
scaffold183586_14.1	1	p2	(TA)10	20	1084	1103	CGAAGATGGTTTCGTGTAAGC
scaffold183606_13.7	1	p2	(AT)8	16	749	764	CATGTAGTACGCCTCGCTGA
scaffold183608_16.2	1	p2	(CT)6	12	2152	2163	CTGTATCCCTGGATGCCTGT
scaffold183616_14.5	1	p2	(TA)9	18	325	342	AGCACGTGTTCTTGCTCGTA
scaffold183807_14.0	1	p2	(TC)6	12	187	198	CAACATCACCAAAAACCCCT
scaffold183817_16.9	1	p2	(TA)8	16	145	160	GCACACTCAGATCACACAGGA
scaffold183868_13.9	1	p2	(TA)7	14	131	144	ACCAAGATTTGATCATGGGC
scaffold184487_13.8	1	p2	(TC)6	12	278	289	ATGGAGGCAAGCTACTCGAT
scaffold184538_12.2	1	p2	(AT)8	16	2855	2870	CGGGAAACATGATCCGAATA
scaffold184721_11.2	1	p2	(CT)6	12	107	118	ATATTGCAAATGGCCGAGAG
scaffold184867_17.3	1	p2	(GA)7	14	285	298	GCCGAGTGATCCTCTCTCAT
scaffold184874_16.9	1	p2	(AG)8	16	3399	3414	AGGATCATGGCCACGTAGAG
scaffold185187_10.7	1	p2	(TA)12	24	475	498	GAGAAGGAGGTTGGGAGGAA
scaffold185243_14.8	1	p2	(AT)6	12	1205	1216	TCCACCTCACTTTTCCCTTG
scaffold185247_12.8	1	p2	(AT)6	12	399	410	CAGCTCTTCAAACTGGACACA
scaffold185293_15.8	1	p2	(TA)7	14	1179	1192	AACCGGTTAAAAATCGGACC
scaffold185421_16.5	1	p2	(TC)6	12	114	125	CAAACACCTGGGACCTCAAT
scaffold185738_16.2	1	p2	(CT)7	14	75	88	CAACCCTACCTCCTTCTCCC
scaffold185757_10.0	1	p2	(GA)7	14	411	424	CAACTGTTCTGAATGCGAGC
scaffold185915_15.6	1	p2	(TC)6	12	1907	1918	CTGGCAAAGATTCTTGGGAT
scaffold186032_16.7	1	p2	(TA)7	14	172	185	TGACATTGCCTAGAAAGTGGG
scaffold186114_11.0	1	p2	(AT)8	16	241	256	TGTTGCCAATTAAGATTGCG
scaffold186192_13.5	1	p2	(TC)6	12	82	93	TCATTCACATCGTCTCTGCAA
scaffold186385_14.5	1	p2	(AT)7	14	774	787	CCCTCGAATGCACTCAAACT
scaffold186705_14.3	1	p2	(TC)6	12	1246	1257	GCCGCATTGTATGAAAATTG
scaffold186741_10.2	1	p2	(TA)7	14	553	566	GGAGAACCGTCGACATGATT
scaffold187029_15.6	1	p2	(AT)7	14	293	306	TCGAATCTATGCATCCACCA
scaffold187070_12.3	1	p2	(AT)10	20	69	88	TCCATAGAAAATGTTGTTAGCAGG
scaffold187138_13.9	1	p2	(AT)6	12	158	169	CAGATTTTGAAACCTGTAAGTGAAA
scaffold187175_11.8	1	p2	(AT)7	14	349	362	TCAATTCATAAATATGTCGAAGAACA
scaffold187195_14.3	1	p2	(CT)9	18	802	819	CCCTCATCTTTTGCGAATTG

scaffold187238_16.4	1	p2	(AT)6	12	1679	1690	TGCTGCAAGCATGTATTTGTC
scaffold187445_14.6	1	p2	(TA)8	16	1129	1144	AACTCAAGTTCCGATCGTGC
scaffold187508_10.1	1	p2	(TA)8	16	380	395	CGTCGATGTTTGTCCATTTG
scaffold187813_20.6	1	p2	(AT)9	18	226	243	TTCTGACGAAATTGCCCTTC
scaffold187863_12.5	1	p2	(TC)6	12	493	504	CCTCTCCTTGCCCTCCTTAC
scaffold187951_18.3	1	p2	(AT)7	14	3323	3336	CGCCATATCGGACAAACTCT
scaffold187966_16.0	1	p2	(TA)7	14	1005	1018	CGTCCAACGAATCAAGACAA
scaffold188087_14.2	1	p2	(TA)7	14	39	52	GGAGCCTCCGAACTTTGAA
scaffold188126_15.3	1	p2	(AC)6	12	1740	1751	TTGTGTACCAAGACATTTGTGTG
scaffold188252_16.9	1	p2	(AT)6	12	197	208	CGACTATCTGCAGGAGGCAT
scaffold188270_14.3	1	p2	(AC)9	18	430	447	ATTTCATCTCCCCACAGACG
scaffold188698_15.2	1	p2	(AT)10	20	47	66	TGGATAAGTCTATCAAGATTTAATTGC
scaffold188824_14.7	1	p2	(AC)6	12	103	114	CACCGTTTATTCCCCATGTC
scaffold188826_14.2	1	p2	(AG)6	12	613	624	ACTTTTGTGGAAAAGTGGCG
scaffold188877_17.8	1	p2	(TG)6	12	56	67	TGATCATAACTCTCTCTCGCGT
scaffold188966_11.9	1	p2	(AG)6	12	173	184	TTGTGAGTCGTTTGTTCTTGG
scaffold189204_11.0	1	p2	(AT)7	14	578	591	TGACCACCCAACCACACTAA
scaffold189325_10.2	1	p2	(AT)6	12	179	190	TTAATCTCCCCGGAATAATTG
scaffold189349_14.7	1	p2	(AT)8	16	379	394	GAGGAATGAGGGGAGGGTAG
scaffold189580_15.4	1	p2	(TA)6	12	185	196	TGTTCATTGAGGAAATTATAAAGCA
scaffold189611_14.3	1	p2	(TA)6	12	505	516	TGTTGAGGGTTGTTTATTATGGG
scaffold189697_11.7	1	p2	(AT)9	18	1258	1275	TTAATGGTGGGGACATGCTT
scaffold189862_16.4	1	p2	(TC)6	12	532	543	AAACCCACTAACCCAAACCC
scaffold190021_10.6	1	p2	(AT)7	14	652	665	AAAAACCCTTCCACCCATTT
scaffold190744_10.2	1	p2	(TA)6	12	971	982	TGTGATGTGTGTGTGTCGTGT
scaffold190970_13.4	1	p2	(TA)10	20	224	243	TTTTCCGAAGGTAATTTTAGTGTT
scaffold191180_13.6	1	p2	(CT)12	24	2887	2910	TCACCCACCAAAATCAAACA
scaffold191238_13.3	1	p2	(TA)7	14	271	284	CGGACGGTCGAATCAATTA
scaffold191276_14.4	1	p2	(AG)7	14	273	286	TTCTCGTGTTTATCCTCGCC
scaffold191618_15.0	1	p2	(TC)6	12	49	60	GGGAAACAATGGATACTCCTTC
scaffold191636_13.1	1	p2	(AT)8	16	106	121	CATAAATGCGGTGGAGATGT
scaffold191774_13.7	1	p2	(AG)7	14	135	148	TTGCGTACCAAGTTTTTGGC
scaffold191936_10.6	1	p2	(CT)6	12	769	780	TTGGTAGCCTGGATAGCACC
scaffold192015_10.4	1	p2	(TG)6	12	297	308	GAGACTTGCAAACTCTCGGC
scaffold192053_11.9	1	p2	(GA)6	12	333	344	CAAACCCTAGCCCAAGAACA
scaffold192135_12.5	1	p2	(TA)7	14	195	208	ATCTTTCCCATTTTGCCCTT
scaffold192188_14.8	1	p2	(TA)9	18	360	377	CGCCCAAAAATCTAACATTCC
scaffold192220_15.2	1	p2	(AT)9	18	2423	2440	CTCTTCTTCGTCCCAAGCAC
scaffold192262_17.7	1	p2	(TA)7	14	445	458	AAAGGGCGGTATCATTTGAA
scaffold192834_15.8	1	p2	(TC)8	16	135	150	CCTTCGGCTTGAAAAACAAG
scaffold193013_15.2	1	p2	(AC)6	12	269	280	CTAGCTTCGGTACAAACCGC
scaffold193231_10.2	1	p2	(AG)6	12	356	367	ATGGGATATGATTCCGGTCA
scaffold193337_10.9	1	p2	(GA)6	12	438	449	TGCAGCAGCAGAAGAGAGAG
scaffold193450_10.9	1	p2	(TA)6	12	444	455	TAGCAGGCTGACCGTAGTGC
scaffold193729_11.2	1	p2	(AT)7	14	434	447	TGTGTGGTCTGAAAACTCGC
scaffold193864_10.5	1	p2	(GC)6	12	223	234	ATATTCTCGCTTCTTCGCCA
scaffold194094_11.8	1	p2	(AT)7	14	476	489	ACCCTCCTTCAGCCAAAAAT
scaffold194259_13.7	1	p2	(GC)8	16	317	332	CGTTGCCGGATAATTCAAAC
scaffold194383_17.4	1	p2	(TC)6	12	235	246	ATGTTTTGGCGTTGGCTATC
scaffold194565_16.7	1	p2	(AG)10	20	298	317	ACCAAGAACATTGAGGCTGC
scaffold194603_10.7	1	p2	(TA)8	16	170	185	TGGTAGCTTTGAGACACGATT
scaffold194669_15.6	1	p2	(TA)6	12	151	162	TGGTTCGGACCATGATTTATT
scaffold194676_15.9	1	p2	(GA)6	12	207	218	TTTTTCGATTGTGCTTGCTG
scaffold194855_12.0	1	p2	(TA)7	14	425	438	TTGATGGTGAACAAACGGAA
scaffold195046_13.0	1	p2	(AG)6	12	185	196	GCGTGGAAGAAAAGATCCCT
scaffold195180_11.2	1	p2	(TC)6	12	193	204	CCCATTTCTAACTACCCAAATCC
scaffold195208_11.0	1	p2	(AT)8	16	1121	1136	TGAAACATGGATTGCCACAT
scaffold195262_11.5	1	p2	(AT)7	14	819	832	CGAGTATGCTGACGGGAAAT

scaffold195448_15.3	1	p2	(AT)9	18	387	404	TGTTAACCAAAAAGAAGAATCGT
scaffold195575_14.7	1	p2	(AT)7	14	264	277	ATGCCCTTCTATGCTGAGGA
scaffold195687_15.7	1	p2	(TA)6	12	2344	2355	CGGGCTGTATTTTAACGGTC
scaffold195723_16.5	1	p2	(TA)6	12	117	128	CCATTTGCAATACTATGGCTTTC
scaffold195724_17.5	1	p2	(CT)6	12	47	58	TGAAAATGGGCGAGAGTTTT
scaffold195884_13.9	1	p2	(TA)7	14	53	66	GGTGTCCGAACTTTATATGGC
scaffold195954_12.8	1	p2	(TC)6	12	159	170	AGATGACAACCTTCGGCTTG
scaffold195995_13.7	1	p2	(AT)7	14	307	320	TGGTATAGGATCCGTCTGGC
scaffold196067_11.3	1	p2	(CT)9	18	261	278	AGAAGGAAAAACCACGGGAC
scaffold196121_17.0	1	p2	(AT)6	12	1457	1468	GCAAATAAAAATTATGGTTTTGGG
scaffold196268_14.1	1	p2	(AT)7	14	69	82	AAACTCGTAAAGTGTCGCGG
scaffold196353_13.8	1	p2	(GT)6	12	55	66	GGAAAATATCAAGTCAACACAAAA
scaffold196496_13.6	1	p2	(TA)7	14	738	751	TTTTTACCATTTTGCCCTGG
scaffold196632_17.1	1	p2	(TA)6	12	482	493	GGGACAATGGAGTTCACGTT
scaffold196753_14.6	1	p2	(TA)8	16	1687	1702	ATGAATTTGGTGTGTTGGCA
scaffold196787_11.4	1	p2	(AT)6	12	183	194	TGCCAACAGTCAAAGTGACC
scaffold196893_13.4	1	p2	(TA)8	16	896	911	TGTCGCCATATACGATCACTTC
scaffold196924_14.4	1	p2	(AG)6	12	93	104	TCTGCTTCAGCTCAGTGTGG
scaffold197312_13.3	1	p2	(TA)11	22	235	256	TAGGGGTTATCACCGGATCA
scaffold197542_13.2	1	p2	(AT)6	12	105	116	CCGAAAATTCAATCCTCGAT
scaffold197602_12.5	1	p2	(AT)7	14	40	53	TCCCAACTAAAATATGCTGCAA
scaffold197665_10.2	1	p2	(AG)7	14	274	287	TGACAGTTTGGCATCAGAGC
scaffold197686_12.0	1	p2	(GA)7	14	105	118	TGCAGAAAATGGAGAGGAGAA
scaffold197886_13.3	1	p2	(TA)7	14	1369	1382	CGAACGGTATAGGATCGGAA
scaffold197903_11.4	1	p2	(CT)7	14	357	370	TAATAGGCTGCCCTCCTCCT
scaffold198177_15.0	1	p2	(TA)8	16	887	902	AGGGCTGAGCAAAAATCAAA
scaffold198463_15.5	1	p2	(TA)8	16	92	107	CAACACAACAATACCACACAGG
scaffold198470_11.4	1	p2	(AT)6	12	829	840	TGTAGATGGCCTAAGACGGTG
scaffold198516_10.3	1	p2	(AG)6	12	363	374	GCTCCAAATCAAAATCCCCT
scaffold198531_10.5	1	p2	(AT)7	14	51	64	TTTGTTCCTACTCACTCACATTAAAA
scaffold198630_14.9	1	p2	(TA)7	14	152	165	TGAATTAACTTCCCAAAACGTG
scaffold198695_10.4	1	p2	(TA)7	14	255	268	CAGACATTGAGGTTTGGATGG
scaffold198826_14.6	1	p2	(AG)7	14	255	268	GTGGGTCGAATTTCGTGAAC
scaffold198946_10.6	1	p2	(TA)6	12	189	200	GGAACCTATGTGACATCCAGC
scaffold198950_13.7	1	p2	(TA)9	18	77	94	ATACCCTACCCGTTTACCCG
scaffold198951_16.1	1	p2	(AT)11	22	1156	1177	ATGCACATGTTTATCGCACG
scaffold199179_10.4	1	p2	(TA)11	22	320	341	TCGCTCATCCAAGTAAGTCG
scaffold199218_16.6	1	p2	(AC)7	14	389	402	GAAGACGCATGCCAGCTAAT
scaffold199316_10.3	1	p2	(TA)11	22	849	870	CTCTTTCCTTGCTCCCCTCT
scaffold199527_10.8	1	p2	(TA)12	24	191	214	TTATCAGCATTTGGGAAGGG
scaffold199716_13.5	1	p2	(AT)9	18	121	138	ATGTGCGCCTTTTAACCTTG
scaffold199793_10.4	1	p2	(GT)6	12	95	106	AATACGGTCCGTGCATGTTT
scaffold200082_11.5	1	p2	(TC)6	12	119	130	AGAACAAGCTAAGGCACCCA
scaffold200146_13.7	1	p2	(TA)7	14	571	584	CCTCCAAGTCTAAGCAGCCT
scaffold200343_10.1	1	p2	(TA)6	12	188	199	CCTCAGGTTCCGATCTCAAG
scaffold200431_12.4	1	p2	(CA)6	12	219	230	TCGTTGATTGCATAGTGGGA
scaffold200667_16.0	1	p2	(AC)7	14	1168	1181	TGTTCTTTGGGATCTGCCTC
scaffold200983_13.9	1	p2	(AG)6	12	286	297	TCCTCCCCAATTTTGTGTGT
scaffold201012_13.1	1	p2	(TC)6	12	605	616	ATGTAGCAGGTTTTGTCGCC
scaffold201538_15.3	1	p2	(TA)7	14	708	721	TTGGTGAGTGTATGGAGCCC
scaffold201747_15.0	1	p2	(TA)8	16	458	473	AAAATCAAAGGCCCACAATG
scaffold202245_13.2	1	p2	(TA)6	12	183	194	GAGTCACACAGTTTGGCACTT
scaffold202365_10.9	1	p2	(AT)6	12	183	194	GCCTTAAAAGGTGGTGTGGA
scaffold202459_16.0	1	p2	(TA)6	12	996	1007	AATGCAACACAACCAAAGCA
scaffold202841_12.1	1	p2	(GA)6	12	50	61	TTGCTGTGTTGGATGGAAAA
scaffold202996_15.0	1	p2	(TC)6	12	371	382	ATGAGCCACAGTTCCAATCC
scaffold203037_12.7	1	p2	(TC)6	12	305	316	CCTGTCTCTTCGGCGTAGTC
scaffold203116_16.8	1	p2	(GA)7	14	326	339	TCGTAAGTCTTCCGCTGGTT

scaffold203349_14.	1	p2	(TA)7	14	290	303	AATGAATGCACCGGATTAGC
scaffold203460_12.	1	p2	(AT)6	12	471	482	GCAATGTGGGCTGAAATCTT
scaffold203469_14.	1	p2	(TC)6	12	1195	1206	ATTCGTCATCAAATTAAAGCCT
scaffold203483_10.	1	p2	(TA)6	12	546	557	GGCGAGAATGGAGGAGAAA
scaffold203486_18.	1	p2	(AG)8	16	816	831	AGCCAGTCTCCACCTCAAGA
scaffold203983_10.	1	p2	(TA)8	16	235	250	TCGAATTCGGGATACCAGAG
scaffold204082_17.	1	p2	(AT)6	12	251	262	GCCTAATAGGGTTGTGTGCC
scaffold204378_12.	1	p2	(GA)7	14	162	175	GAGACGAGAGAAAAGAGTGCGT
scaffold204428_12.	1	p2	(AT)7	14	773	786	GGCAAAAAGCTAATATTTTCAGG
scaffold204647_13.	1	p2	(AT)8	16	193	208	CACCGTTGTCTTTAAGCCGT
scaffold204685_11.	1	p2	(TC)6	12	159	170	GTCGCAGTGATGTCCTTCCT
scaffold204743_10.	1	p2	(AT)6	12	179	190	TCCTCGTATCCGTTCTCCAC
scaffold204769_14.	1	p2	(AT)7	14	2054	2067	CTAACCAGTGGCATGGGAGT
scaffold205077_13.	1	p2	(AT)8	16	1531	1546	ATACATGTCATGCCCGTTTG
scaffold205310_15.	1	p2	(GA)7	14	581	594	AAGCTATGGTGGTTTTGTAAACG
scaffold205493_14.	1	p2	(TG)7	14	108	121	AACACCTGTGAAAGGGATCG
scaffold205539_12.	1	p2	(CT)6	12	419	430	GTTGGCAAACAGGTCCCTAA
scaffold205559_15.	1	p2	(TA)8	16	83	98	TTCGGAGTTATGAAGTTAGCACC
scaffold205565_10.	1	p2	(CT)6	12	257	268	GATTAGCAAATTTCGGGACG
scaffold205620_13.	1	p2	(CT)6	12	1527	1538	CGACCATAACAAAGAAAACACG
scaffold205673_11.	1	p2	(TA)7	14	425	438	CGCAGATCTGATGGTCCAG
scaffold205706_12.	1	p2	(TA)8	16	124	139	GGAGGAGAGGAAATTGAGGG
scaffold205947_10.	1	p2	(AG)7	14	508	521	GGTGCTCAAAAATCCGAAAA
scaffold206176_11.	1	p2	(TA)8	16	253	268	TGAGATAAGGGGGTGTTTGC
scaffold206359_11.	1	p2	(TA)7	14	175	188	ACCCTCCTTCAGCCAAAAAT
scaffold206493_10.	1	p2	(AT)9	18	333	350	TCCCTATTTTTCGTCTCCCA
scaffold206497_17.	1	p2	(AT)6	12	385	396	CGAAGAGTGTGGAATCAAACC
scaffold206978_13.	1	p2	(AT)7	14	623	636	CATCGAAAGAATGTAAGATGCG
scaffold207222_15.	1	p2	(TA)6	12	548	559	TGATTTAAGGGGGTGTTTGC
scaffold207600_14.	1	p2	(AT)8	16	185	200	TGATTAGTACCGATGAATCAACCA
scaffold207843_14.	1	p2	(GA)6	12	91	102	ACCTATGGAAGCCCCAAACT
scaffold208012_15.	1	p2	(GA)7	14	3830	3843	TTGGGAGATTTCAAGAACGG
scaffold208063_15.	1	p2	(GA)6	12	384	395	TCAACATGCAACCAACAAAAA
scaffold208075_22.	1	p2	(CT)6	12	282	293	CAGGAACCCTTTCTCTCATCC
scaffold208112_13.	1	p2	(TA)6	12	1032	1043	ATTTTTGGATGCCAATGCTT
scaffold208204_13.	1	p2	(TA)7	14	359	372	ATTGACATGGCATCGTCTCA
scaffold208354_15.	1	p2	(CT)8	16	2025	2040	TGGCTCTTGTCTTCCTGCTT
scaffold208400_12.	1	p2	(AG)6	12	193	204	CGTGTTCTTCATCCTTTCCC
scaffold208469_14.	1	p2	(AG)10	20	69	88	GCATGGTTTGTTCAATGCAG
scaffold208631_11.	1	p2	(AC)6	12	88	99	CCTCGGCTCTGATACCAAAC
scaffold208674_17.	1	p2	(TC)7	14	344	357	CCTTCCCATTTCCTCTCTCC
scaffold208726_15.	1	p2	(TA)6	12	154	165	CATTGGCATTGTAAGCTTCG
scaffold208882_16.	1	p2	(AT)12	24	46	69	TATCCTCCTCCCAATCGAAA
scaffold209343_18.	1	p2	(TA)8	16	119	134	TGATGGAGGACGACAATGAA
scaffold209354_21.	1	p2	(TA)6	12	241	252	TCACTAACGGCCGGAATATC
scaffold209357_16.	1	p2	(TC)6	12	667	678	AAGGGGTGTTGTGTTGTGCT
scaffold209468_15.	1	p2	(TA)7	14	73	86	GCGAATGTAGGCTGGATCAT
scaffold209533_15.	1	p2	(GA)6	12	951	962	GCATGCATATCGCAGAGAGA
scaffold209593_11.	1	p2	(GA)6	12	168	179	GTTGGAGCTGCTAGGATTCG
scaffold209717_14.	1	p2	(TA)7	14	353	366	TCAACATCGTTTATCAGTTTTGTT
scaffold209718_14.	1	p2	(TA)13	26	1584	1609	TTAATACCCAAAATGCCCCA
scaffold209748_11.	1	p2	(TA)14	28	42	69	TTTTGACAATATTTAGGTGTTTTGA
scaffold209817_13.	1	p2	(AG)7	14	344	357	CGACTCCCAATCAGCAAAAC
scaffold209969_14.	1	p2	(AT)10	20	385	404	TCTCTCTCGACGGTGAAGGT
scaffold210204_17.	1	p2	(AT)6	12	70	81	GTAAGGCTTTTCTAACTTATTGGAAC
scaffold210915_18.	1	p2	(TA)6	12	154	165	CGGCCGGAATATCTAAAGTG
scaffold211083_15.	1	p2	(AT)7	14	962	975	GGTTCAATTTTGCCAACCAC
scaffold211301_10.	1	p2	(TA)8	16	166	181	TGGTGGCCATATATACGCAAT

scaffold211408_10.8	1	p2	(TC)6	12	38	49	CTTTGGCAACGGAATACCAC
scaffold211562_14.6	1	p2	(CT)6	12	964	975	TCCAAATACCCAGTCCCAAA
scaffold211597_14.3	1	p2	(GA)6	12	97	108	CTCTATCTTCGGCCCCTTTC
scaffold212436_12.0	1	p2	(AG)9	18	580	597	GCGAAGCTTTTCCAACTCAC
scaffold212590_18.2	1	p2	(CT)6	12	716	727	TACACGAATAGCGGTTGCTG
scaffold212657_14.4	1	p2	(CT)8	16	428	443	CCTTGGAGAAAAGGACGACA
scaffold212700_13.8	1	p2	(GA)6	12	871	882	CCCACAAAACCTAAAGCCAA
scaffold212778_12.7	1	p2	(TA)7	14	237	250	ATGGGTTATTACCGGATCGC
scaffold213144_15.4	1	p2	(AT)8	16	2022	2037	AAAACTTCAAACCACGGGAT
scaffold213406_16.2	1	p2	(TC)7	14	1293	1306	TCACAAGCATGCAAGATAAGG
scaffold213409_10.6	1	p2	(AT)6	12	106	117	GGATGGACCGGATGTTAGAA
scaffold213424_18.1	1	p2	(TA)6	12	310	321	GGAAAATTTGACGTCGGAAA
scaffold213511_14.0	1	p2	(TA)6	12	23	34	ACAAGCACGAACACACACAC
scaffold213638_15.2	1	p2	(CT)10	20	3974	3993	AAGCTAAGGATTGAAGCGCA
scaffold213684_15.0	1	p2	(CT)8	16	993	1008	ACCATCGATTTTCTGCAACC
scaffold213772_12.0	1	p2	(TA)6	12	162	173	CCCATGCATGTAACAAACCA
scaffold213806_14.5	1	p2	(CT)9	18	851	868	GCAGTTGGGAAGGCACTAAA
scaffold214000_16.0	1	p2	(AC)6	12	141	152	AAAATGCCCTTGTATGCTGC
scaffold214055_10.8	1	p2	(AG)6	12	202	213	TTGGATTCAGATCGTGGAAA
scaffold214073_18.4	1	p2	(CT)7	14	42	55	TTAAATTTTCGAACGCCTGC
scaffold214079_16.0	1	p2	(TG)8	16	138	153	TATCACCATAACGCACCCCT
scaffold214314_11.5	1	p2	(CA)8	16	979	994	GTTCGGGGTTGCACTGTATT
scaffold214464_13.1	1	p2	(TA)6	12	756	767	TCTAGTTTCGCCCCTGCTTA
scaffold214612_13.4	1	p2	(TA)6	12	895	906	TGAAGGGGATAGCATTTTGG
scaffold214689_10.8	1	p2	(TA)8	16	536	551	CCTTGGATCACATCAGAACG
scaffold214727_17.5	1	p2	(AT)6	12	238	249	AAGAAAGCCCACCTGATTTG
scaffold215004_16.7	1	p2	(TC)6	12	1340	1351	TAAATTGCTTGCCTCTGCCT
scaffold215094_13.9	1	p2	(CT)6	12	159	170	AGCAAGGAGAATCGAACCTG
scaffold215230_10.5	1	p2	(TC)8	16	166	181	GGGGACCACAAAATATTCCC
scaffold215238_15.4	1	p2	(CA)6	12	163	174	CGTCCACGGAGAGGTTAGAG
scaffold215266_12.9	1	p2	(TC)6	12	658	669	ATGCCATACGGACGTTGAAT
scaffold215457_18.0	1	p2	(TA)7	14	52	65	TCGAGTTATGAAGTTAAGAAATAAGG
scaffold215555_10.6	1	p2	(GA)7	14	78	91	CGGTACCATCGTCTCTCGTT
scaffold215666_15.8	1	p2	(GA)13	26	1384	1409	CAAGTTCCACCCAGGTGTCT
scaffold215887_10.4	1	p2	(TA)7	14	328	341	TGCATAGGCTGTCTCTTGGA
scaffold215951_11.1	1	p2	(TA)9	18	382	399	CCCTTGTATGCAGCCATTTT
scaffold215970_15.9	1	p2	(TA)9	18	560	577	TTCGAGTCATACGGAGGAGG
scaffold216094_11.0	1	p2	(AT)7	14	175	188	TGGGCATCACAAATAGACGA
scaffold216128_19.3	1	p2	(TA)8	16	754	769	TAAAAAGTTGAGCCCACCCA
scaffold216133_17.3	1	p2	(AT)8	16	171	186	TCCTTTCGTTTAACTCCTCCA
scaffold216170_13.1	1	p2	(TC)6	12	143	154	TACCGTTGGTTAATCCTGCC
scaffold216222_15.3	1	p2	(CT)6	12	1057	1068	TATGCGGTGTTTTGGTTTGA
scaffold216402_11.2	1	p2	(AT)10	20	681	700	ATCTTGACCGCTGATTTTGG
scaffold216411_14.4	1	p2	(AT)6	12	491	502	GGATCCCACTGAATTGTGCT
scaffold216421_16.0	1	p2	(TA)6	12	459	470	AAGAGGAATTGAACCCAGGA
scaffold216795_12.2	1	p2	(TA)9	18	162	179	AGGCAATTAAGATTCGCCCT
scaffold216990_12.1	1	p2	(TC)6	12	46	57	AGGGGGTGAATAGCCTCACT
scaffold217146_12.8	1	p2	(AG)6	12	455	466	GCCCATTTGAGCCAATCTAA
scaffold217414_12.0	1	p2	(TA)8	16	138	153	ATTGCCCCTCATATGCAGTC
scaffold217620_13.2	1	p2	(TA)8	16	809	824	GGCTAATTTGATGCACGGAT
scaffold217795_11.9	1	p2	(AT)10	20	196	215	AAAAATCACGCCACCATTAG
scaffold217964_18.0	1	p2	(TA)7	14	178	191	TGGATCAAATGTTTGGTTGC
scaffold218120_13.4	1	p2	(AG)6	12	139	150	AAGCAGCCATTAAGAGTCGG
scaffold218440_13.0	1	p2	(GA)6	12	149	160	GACGCCTGAATATCGGTCAT
scaffold218514_17.8	1	p2	(TC)6	12	226	237	AACCCATGCCAGGAATACAA
scaffold218545_11.9	1	p2	(TA)6	12	352	363	TGCCCTCAATCTCGAAAAAT
scaffold218840_15.7	1	p2	(TC)6	12	1787	1798	GCGGTTAGGCTTCCACCTAT
scaffold218862_10.5	1	p2	(AG)7	14	65	78	AAAACTCCTCCAAACTTCCTCC

scaffold218920_11.6	1	p2	(TA)6	12	117	128	TCTCCTTACATAATTTGCCCTCA
scaffold219033_11.6	1	p2	(CT)6	12	110	121	TCCTCACTCTCACTAGCCGC
scaffold219072_13.1	1	p2	(AT)11	22	1453	1474	TACCTGCCACAACACCAGAG
scaffold219080_14.2	1	p2	(CT)6	12	73	84	CAGCCCTCTACCCTTCTTCC
scaffold219363_11.0	1	p2	(GA)6	12	289	300	TTGATGTCCCTTCTCCTTGC
scaffold219370_15.4	1	p2	(CT)6	12	595	606	CTGTTCACGGGAGCTTTGAT
scaffold219783_16.3	1	p2	(TA)7	14	507	520	CGAGTGCATGAGCTTGAGAG
scaffold219866_16.7	1	p2	(AT)8	16	5050	5065	TGGTGATGGTGATGAATGTTTT
scaffold220072_17.7	1	p2	(CT)6	12	170	181	CCATGCATGCAAAATTGAAC
scaffold220088_17.5	1	p2	(TA)6	12	924	935	AACTCTCAATGCCCAAATCC
scaffold220119_14.8	1	p2	(TC)12	24	203	226	AAGGGGTCAACACAGTGGAA
scaffold220264_18.6	1	p2	(AT)10	20	1341	1360	GAACTGATCAAACCGTCCGT
scaffold220473_14.6	1	p2	(TA)6	12	274	285	TGATGCAGTATATAATCCTTAATCAAA
scaffold220553_12.1	1	p2	(AT)10	20	1101	1120	ATCCAAAGGGACAAAATCCC
scaffold220638_12.2	1	p2	(AC)6	12	215	226	TTCAAGCTGAGATCGACGAA
scaffold220806_13.0	1	p2	(TA)7	14	207	220	TGCATGCACCTGATTACAAA
scaffold220855_11.0	1	p2	(AT)6	12	97	108	TCCATATCATTATCAAATGTTGACTT
scaffold220938_10.2	1	p2	(TG)8	16	280	295	CAGAATCACCATCAACACCG
scaffold220955_10.6	1	p2	(GA)7	14	384	397	GTAGGAGATGGAGTTGGGCA
scaffold220974_13.2	1	p2	(TA)9	18	255	272	TCATGCAATGAAATCCGAAA
scaffold220995_10.4	1	p2	(TC)6	12	169	180	ATATCTGGAGGCGGGAACTT
scaffold221261_13.7	1	p2	(TC)7	14	114	127	TACCCAAACCCACCAACCTA
scaffold221589_16.0	1	p2	(CT)6	12	232	243	GCACCATCAGAGCAACAGTC
scaffold221668_17.4	1	p2	(TC)6	12	223	234	AGGTGGGGTGTTAGGAGGAT
scaffold221848_11.9	1	p2	(AT)10	20	441	460	CGACTCATGTTGTTCCATGC
scaffold221883_16.5	1	p2	(TA)8	16	3520	3535	TCCTCTAATGGCCGAAAATG
scaffold222102_10.4	1	p2	(CT)7	14	158	171	TAGATGGGCTAAGTGGGGTG
scaffold222236_13.0	1	p2	(TG)6	12	232	243	GGAAAGTCTCACTCGCAAGG
scaffold222290_12.0	1	p2	(AT)10	20	1258	1277	ATCATGCGGTATCATGGTGA
scaffold222358_10.5	1	p2	(AT)7	14	772	785	TGATCCAGTCACGTTTCACAA
scaffold222455_13.1	1	p2	(AG)6	12	55	66	GATCTCGCCTCAACGAGAAG
scaffold222496_16.0	1	p2	(TA)6	12	264	275	CATTCAAGGGGCATTTTTGT
scaffold222589_15.6	1	p2	(TA)7	14	369	382	CTCATTGAGTTGACCCAGCA
scaffold222790_16.2	1	p2	(CT)6	12	835	846	TCACACCCTCTCCCACTCTC
scaffold222824_12.7	1	p2	(TA)7	14	227	240	ATGCTGTCAATTAGTGGGGG
scaffold222857_10.3	1	p2	(GT)8	16	52	67	AATTTTCTGGCAGCCTTTGA
scaffold222863_10.1	1	p2	(AT)6	12	180	191	ATGCAAGCACAATTCGATCA
scaffold223163_11.6	1	p2	(TG)7	14	610	623	CAACCATTTTAAGCCCTTCG
scaffold223175_16.4	1	p2	(TA)7	14	157	170	AACGGTATGCTCGGATTTTG
scaffold223291_10.1	1	p2	(AT)11	22	416	437	TAACCGTTGTCTATGCACGC
scaffold223441_10.9	1	p2	(TC)6	12	233	244	ATTGCTTTTGGCTTGTTGCT
scaffold223497_12.1	1	p2	(AT)8	16	271	286	AAATATACCCCCGCATGCTA
scaffold223658_12.7	1	p2	(GA)7	14	226	239	AGGGAAGAACGAATTGAGGC
scaffold223795_11.9	1	p2	(TC)7	14	145	158	ATTCGCACAAATGATCGTGA
scaffold223923_23.0	1	p2	(TA)6	12	54	65	GGCTTTCTTAACTTGTTGGAACC
scaffold224010_10.2	1	p2	(AT)9	18	378	395	TTGAGCACAGGTTGATCACG
scaffold224042_11.2	1	p2	(CT)6	12	509	520	ATCTTGCTAGATGTGCGCCT
scaffold224085_11.0	1	p2	(AT)6	12	181	192	ACAAGGGGCAGAAGGATTTT
scaffold224507_16.8	1	p2	(TC)7	14	454	467	TAAGAGATCCGAAGCCAAGG
scaffold224830_11.3	1	p2	(AT)11	22	183	204	GCTACTTCCGACTCTCTCGC
scaffold225025_13.8	1	p2	(AT)7	14	391	404	GGAAGCAAGGTAGGCTTTCG
scaffold225086_17.5	1	p2	(TC)9	18	1286	1303	CTGGAATCGAAATGATGAAGC
scaffold225152_12.4	1	p2	(AT)9	18	237	254	GGGGAGGGTGGTTAGGTTAG
scaffold225279_11.6	1	p2	(AT)6	12	377	388	GTGTGCGGTCAAGGGTACTT
scaffold225452_16.1	1	p2	(TC)6	12	587	598	TTTTTGGTGGGTTTCGTCAT
scaffold225489_12.9	1	p2	(AT)7	14	242	255	AAGGTGGGAAAGAATTTGGC
scaffold225752_15.1	1	p2	(CT)9	18	1613	1630	GATGCCCAAACTTCCTACCA
scaffold225753_12.5	1	p2	(AG)7	14	87	100	TTTTGTTGTTTCCCAAGTTGC

scaffold225882_14.5	1	p2	(TA)8	16	887	902	TCATCCCAAAAATACCCTCG
scaffold226074_16.1	1	p2	(TC)6	12	651	662	TCAAGGACTTACCGCTGGAC
scaffold226076_15.4	1	p2	(TA)6	12	1474	1485	CAAACTCCCCCTTCCTCAAT
scaffold226167_14.9	1	p2	(AT)8	16	1146	1161	CTCGAGCTGGATGAGAATGG
scaffold226282_13.1	1	p2	(CG)6	12	239	250	GTTGCCGGATAATTCAAACG
scaffold226317_11.6	1	p2	(TA)11	22	109	130	GTCGCTTGAATTGCGACATA
scaffold226548_14.4	1	p2	(TA)11	22	180	201	TATGGGTTAGCGTGTGATCG
scaffold226561_15.9	1	p2	(AG)9	18	4215	4232	AAGAACCCCAGCATGAATTG
scaffold226568_15.5	1	p2	(TC)6	12	711	722	GGAAAATGTCTTGTGGTCCC
scaffold226587_12.2	1	p2	(AT)11	22	266	287	TTCGGACTTATGAAGTTAGCACC
scaffold226796_16.5	1	p2	(GT)6	12	57	68	GGCTGTGAGATCTAGCGTGA
scaffold227468_11.8	1	p2	(GA)6	12	109	120	TGATCTATGGAAGCCCCAAA
scaffold227758_16.8	1	p2	(AG)6	12	77	88	CCCATGTTGTAATTAATGTAGGGA
scaffold228015_11.5	1	p2	(AT)9	18	637	654	CGAATTTGCAAAACCGAAAT
scaffold228042_10.7	1	p2	(AG)6	12	147	158	TCCATGAAAGCTCACCCTCT
scaffold228253_10.5	1	p2	(GA)6	12	717	728	ATGGAATTGGGTGTTGGATG
scaffold228272_15.6	1	p2	(GA)6	12	583	594	AAGTTCACCGCCTGCTCTTA
scaffold228654_10.0	1	p2	(TC)8	16	181	196	CGCTCAAATCACAAACCCTT
scaffold228698_15.6	1	p2	(AT)8	16	3097	3112	TTGCCTTGTTTAACTTCGGG
scaffold228785_14.9	1	p2	(AT)6	12	443	454	TCAGTTAATATCCCCCGCAG
scaffold228983_12.7	1	p2	(AG)8	16	488	503	TTGTTTGTACCGAATGCGAG
scaffold228985_16.3	1	p2	(AT)6	12	760	771	GAGCTGAATTGGTTCGCTTC
scaffold229230_16.0	1	p2	(TC)6	12	143	154	TGGAGGATTTTCTTTTCCCA
scaffold229298_14.9	1	p2	(TG)7	14	1331	1344	GCAAGTTTCCGTAGCTGGAG
scaffold229592_10.8	1	p2	(CT)7	14	319	332	ACACAGATCATCTCGGGTCC
scaffold229625_15.7	1	p2	(AG)6	12	394	405	ATCGATCGGCTTATGAAACG
scaffold229749_12.4	1	p2	(AG)6	12	1193	1204	AAGAGATGGTTCCGCTCTGA
scaffold230403_11.8	1	p2	(TA)8	16	211	226	GGAGGTTCGACGTGGTTTC
scaffold230637_16.8	1	p2	(TA)6	12	106	117	TCACACCACATCTCAAATGACTC
scaffold230656_16.5	1	p2	(TA)6	12	355	366	GGCTTCAACCTTGACGAAAC
scaffold230670_14.1	1	p2	(TA)6	12	239	250	GGCCTCACTCCCTTTTTCTC
scaffold230710_12.0	1	p2	(AT)6	12	220	231	TTTTCAAGCCTTGTTGAGCA
scaffold230888_10.4	1	p2	(TC)6	12	87	98	AGATGATCCACCGACTCCAC
scaffold230967_17.9	1	p2	(AG)6	12	44	55	GCGATGAGAGAAGGAAATGG
scaffold231010_17.6	1	p2	(TA)8	16	867	882	GCACTGCTTTTTGGTGATGA
scaffold231468_10.4	1	p2	(AT)6	12	208	219	ACCCTAGAAACCCGATCGTT
scaffold232828_10.0	1	p2	(TA)6	12	169	180	AAACATGCAGGTTAGGTGCC
scaffold233077_10.7	1	p2	(TA)9	18	48	65	ATTACCATATTTAAGGGTTATCATCTG
scaffold233385_12.7	1	p2	(TC)10	20	483	502	ATTTTTCCTCTTTCCCCACG
scaffold233696_14.6	1	p2	(AG)7	14	2881	2894	AAGGCGTCTCGAGTGAAAAA
scaffold234041_10.0	1	p2	(TA)6	12	240	251	CAAATACGACACCGAAAGGG
scaffold234359_11.6	1	p2	(AG)7	14	1025	1038	AGAGTCGGTGGAAATGGATG
scaffold234371_11.5	1	p2	(TA)8	16	762	777	TGGCCGGCAGTTTATATAGG
scaffold234988_17.3	1	p2	(TC)6	12	237	248	TGACACCTAGCCCAAACCTC
scaffold235022_11.2	1	p2	(TC)6	12	71	82	TCTGAGACGACGGTGATACAA
scaffold235045_15.3	1	p2	(TC)6	12	1128	1139	CCTCGCACATTCAATTTCCT
scaffold235562_15.3	1	p2	(AT)10	20	36	55	GAAAAAGTCAGCCGAGAGAAAA
scaffold235653_12.6	1	p2	(TA)7	14	419	432	TGATGTTGATTTGGGTTCAAG
scaffold235891_15.3	1	p2	(CT)8	16	49	64	CACTCACTCTCACTAGCCGC
scaffold236163_21.5	1	p2	(GA)7	14	76	89	TGATTTTCTGAAATCGCACG
scaffold236226_15.2	1	p2	(AC)6	12	1292	1303	GGCTCAAATCCGATGATTCT
scaffold236548_17.8	1	p2	(TA)9	18	821	838	ACCACGGAAATTACGGAAAA
scaffold236592_15.0	1	p2	(CT)6	12	566	577	TCTGCTAGGGTTGGGAGAGA
scaffold236645_13.1	1	p2	(AT)9	18	144	161	TTTGGACGATTTTTCCCTTTT
scaffold236858_13.9	1	p2	(AT)6	12	534	545	GCAAACATCCAACTACGAGC
scaffold236993_11.9	1	p2	(AG)6	12	306	317	CTTCGGAACTCTATCCACCG
scaffold237031_16.5	1	p2	(AT)8	16	393	408	CCCCAATTTTGTACCCGAA
scaffold237067_12.7	1	p2	(AC)7	14	632	645	AATTGTGGAGGTTTGAAGCG

scaffold237148_16.4	1	p2	(TA)9	18	397	414	CGGCCTCAACCTATATTTGG
scaffold237226_12.4	1	p2	(TA)8	16	438	453	TGAAAACTGAAACTAGGAACGAAA
scaffold237280_13.9	1	p2	(TA)9	18	2950	2967	ACCATGCTCGAATATCTGGC
scaffold237837_12.5	1	p2	(AT)7	14	322	335	CGTCCGTATGAAATTCCAGG
scaffold238266_16.3	1	p2	(TC)7	14	171	184	GTCAACTTTGGCAATGGGAT
scaffold238425_12.2	1	p2	(TA)10	20	367	386	CGCCTTTAAATCAGGTGCAT
scaffold238583_16.1	1	p2	(AT)11	22	1228	1249	CGCTACAGTTCACACGTTCG
scaffold238737_10.6	1	p2	(TG)6	12	72	83	TCAAAATTTCCAAAAATCAAAGC
scaffold238853_11.0	1	p2	(TA)6	12	150	161	CTCAGGTTCCGACCTCAAAC
scaffold238983_12.0	1	p2	(TC)6	12	139	150	TTCAAGGCATTTATCTGGGC
scaffold239219_11.3	1	p2	(TC)7	14	273	286	GCCCCTTGAAGTTTCGTAGA
scaffold239249_17.9	1	p2	(TA)8	16	2303	2318	TTCCTTGGGACACAATCCAT
scaffold239432_17.0	1	p2	(AG)7	14	1157	1170	GGGCTAGTCCTGCAACACAT
scaffold239459_11.4	1	p2	(AT)8	16	537	552	CCCGATTTGTAAGGTTCCATT
scaffold239647_10.0	1	p2	(CT)6	12	44	55	GCTCTACAATATTACAATGGGCAA
scaffold239769_10.3	1	p2	(TC)6	12	182	193	TGTGATAGGATAGGGCCAGC
scaffold239846_14.8	1	p2	(TC)6	12	91	102	AGGACACGTTGGACCTGAAG
scaffold239960_16.5	1	p2	(TA)6	12	1006	1017	GCTTGAAAAGCCATGCAAAT
scaffold239998_18.8	1	p2	(AT)9	18	555	572	AACGTGAATCTCATCCGTCC
scaffold240085_14.8	1	p2	(TA)9	18	1599	1616	TGATTTCCAAGTGAAGGCTG
scaffold240099_11.5	1	p2	(TA)11	22	523	544	TTGTCATTGTTAATCCCAAAACA
scaffold240190_12.4	1	p2	(AG)6	12	334	345	TTATTGAAGCAGCAGCAACG
scaffold240355_16.0	1	p2	(AT)6	12	177	188	AGCACGGCTTGTGCTATCTT
scaffold240369_13.8	1	p2	(CT)8	16	2539	2554	CCCCAGAAACACCAACTTGT
scaffold240425_11.3	1	p2	(TA)7	14	323	336	TATAACGACACGGCCATTGA
scaffold240746_14.8	1	p2	(TA)6	12	83	94	CAAGTGGTAGCAAACCATGAA
scaffold240836_14.3	1	p2	(TA)7	14	846	859	AGAATTCGTGCCCCTCATTA
scaffold241004_14.7	1	p2	(AT)8	16	313	328	GAACGTTAGTGAGGACGGGA
scaffold241339_14.8	1	p2	(TA)6	12	813	824	CCCGGTATGCTGACGATAAC
scaffold241379_13.2	1	p2	(AG)6	12	248	259	ACATGTTCACCAAGCCACTG
scaffold241664_12.1	1	p2	(GA)6	12	28	39	GGAGGGAGGGAGAGATAGGA
scaffold241735_16.0	1	p2	(GA)8	16	37	52	GGAAGAATGATGGAATTGGG
scaffold241806_12.9	1	p2	(TC)7	14	1053	1066	TGAACAATTGAACACCGGAA
scaffold241938_16.6	1	p2	(TA)8	16	263	278	ATGACACGCATAAAGGGACC
scaffold241944_15.7	1	p2	(GA)7	14	349	362	TCACAATAGGGCATGGGACT
scaffold242054_13.1	1	p2	(CA)7	14	69	82	CACTCACACTTTGCACACTGAA
scaffold242072_13.4	1	p2	(CT)6	12	389	400	GGCAGATGCCTTTGAAGAAC
scaffold242170_16.3	1	p2	(CT)6	12	427	438	TTAAAACAAGAAAGCCCACCA
scaffold242351_16.5	1	p2	(CA)6	12	469	480	TCAAATCTCTAATTGCACCCTTC
scaffold242363_16.8	1	p2	(TA)6	12	220	231	AGTTGAAAGAGCCATCAGCC
scaffold242477_15.3	1	p2	(AT)7	14	1046	1059	AAATGGGGAGATGATGAACG
scaffold242486_11.0	1	p2	(AT)6	12	292	303	CCATTCAAGTCGTGGTCCTT
scaffold242623_10.1	1	p2	(AT)8	16	267	282	TGGACGTAAATACCCTTTTACCA
scaffold242685_15.0	1	p2	(AT)6	12	116	127	ACACAACAATACCACGCAGG
scaffold242959_13.8	1	p2	(GT)6	12	208	219	TTGTTGCCGTTAAAGTGTGG
scaffold242962_12.5	1	p2	(AG)7	14	205	218	GGGAGAGAAGGGAGGAGAGA
scaffold243021_11.3	1	p2	(GA)6	12	98	109	GGATTTGTCAACCGGAAAAC
scaffold243145_14.8	1	p2	(TC)7	14	901	914	AAAGCAACACCATCAGACCC
scaffold243199_10.3	1	p2	(GA)6	12	171	182	TCAACAACATGCAAACACCA
scaffold243221_17.5	1	p2	(CT)6	12	212	223	CACCCCTCCACTCTCTCATT
scaffold243316_12.0	1	p2	(AT)9	18	166	183	ACCACGCATGGTTGAGTACA
scaffold243762_14.9	1	p2	(TC)6	12	412	423	CACCAACATCATCGAACAGG
scaffold243931_16.1	1	p2	(AG)11	22	1698	1719	TTGTCACATACACGTTGGGAA
scaffold244306_15.3	1	p2	(AT)6	12	81	92	CACCACAATGAAGTGGATGC
scaffold244372_15.9	1	p2	(TA)10	20	219	238	ACAACAATGTCGCGAAACAA
scaffold244393_16.1	1	p2	(CA)6	12	139	150	TCAATCTCTCGTGCTCACTGAT
scaffold244762_11.7	1	p2	(CA)8	16	569	584	TCAAAAGCAATCAAATATCCCA
scaffold244828_10.3	1	p2	(TA)10	20	479	498	CAAAAGATCGGCTATTTGGTG

scaffold244940_16.[illegible]	1	p2	(GA)6	12	410	421 TGGATGCCTTTCATTTAGGTG
scaffold245015_12.[illegible]	1	p2	(GT)10	20	661	680 CGGAAGCGCACATTAAGATT
scaffold245367_12.[illegible]	1	p2	(AC)7	14	157	170 CACTTGGCAAAAAGCACAAA
scaffold245653_13.[illegible]	1	p2	(GA)7	14	600	613 CATTCTCACCGGAACCCTAA
scaffold246200_12.[illegible]	1	p2	(TG)6	12	399	410 TCAATAGAAAATGAGTTTGAGTTTCG
scaffold246523_14.[illegible]	1	p2	(AT)7	14	291	304 ATGCATCCTGGCCTTGTTTA
scaffold246897_13.[illegible]	1	p2	(AG)7	14	2093	2106 AGAATGATGAACCCCTTCCC
scaffold246965_12.[illegible]	1	p2	(CT)8	16	611	626 GTTCTTGCTTGCTGTCCCTC
scaffold247028_13.[illegible]	1	p2	(GT)6	12	402	413 CAAGCTGTCAACCCAAAACA
scaffold247219_13.[illegible]	1	p2	(CT)10	20	398	417 GTCGGCAAACCAGAGCTAAC
scaffold247256_15.[illegible]	1	p2	(TA)7	14	176	189 TTTTACCCTCCCATGCTTTG
scaffold247353_16.[illegible]	1	p2	(TA)6	12	314	325 CCAGGTTCAACTCTCTTCCG
scaffold247459_20.[illegible]	1	p2	(TA)8	16	78	93 CCATTTGCAATACTAAGGCTTTC
scaffold247565_14.[illegible]	1	p2	(TA)10	20	1126	1145 AGGAATAGGGCCGAGAAAAA
scaffold247692_14.[illegible]	1	p2	(TA)8	16	2165	2180 ATCAACTAAATTTGGCGCGT
scaffold247711_16.[illegible]	1	p2	(TG)7	14	2528	2541 GACATTGGCATTTTCTGCAA
scaffold248051_11.[illegible]	1	p2	(GA)6	12	73	84 TCTTGGATGATGAGGCTTGA
scaffold248103_13.[illegible]	1	p2	(GA)6	12	1558	1569 TTTTCTCCTTGTAGCGTGGG
scaffold248110_14.[illegible]	1	p2	(AT)8	16	240	255 TCGTCAAAAACTAATTAAGGCCA
scaffold248264_14.[illegible]	1	p2	(GA)6	12	55	66 TGAATTACTTGAAGAATGATGAGAGA
scaffold248329_10.[illegible]	1	p2	(CT)7	14	100	113 TAACCAAACCCCCAATTTCA
scaffold248478_15.[illegible]	1	p2	(AT)7	14	924	937 CAGGAAGCTTCAACTCCAGG
scaffold248621_12.[illegible]	1	p2	(AG)6	12	54	65 GTGAAATTGCTTTTTGGGGA
scaffold248634_16.[illegible]	1	p2	(CT)13	26	2274	2299 GCCAGCTAACACATCAAGCA
scaffold248651_15.[illegible]	1	p2	(AC)7	14	525	538 TCAATGCAACTCAAATCCCA
scaffold248670_13.[illegible]	1	p2	(TG)6	12	495	506 TGTGTGCAATGTGTGTGTGTT
scaffold248820_17.[illegible]	1	p2	(CT)6	12	221	232 ACACATGCTCGGCATACAAA
scaffold248988_15.[illegible]	1	p2	(AG)8	16	3697	3712 AACAACCACTGGCAGAATCC
scaffold249102_16.[illegible]	1	p2	(AT)7	14	2543	2556 CACTTCTGCGGCTGAAAAAT
scaffold249107_14.[illegible]	1	p2	(TG)6	12	1211	1222 TTTGTTTCCTCCCTTTGTGC
scaffold249134_10.[illegible]	1	p2	(AC)6	12	158	169 CAGATACGCGGGTTCATTCT
scaffold249452_10.[illegible]	1	p2	(TG)6	12	67	78 TCTGTGCTCTCTTTCTCAGTCG
scaffold249548_17.[illegible]	1	p2	(TA)7	14	50	63 ATGAAGTTAGAAAATAAGGAGGTGTT
scaffold249652_11.[illegible]	1	p2	(TG)10	20	289	308 AAACTGCCCTCCAATGCTAA
scaffold249770_12.[illegible]	1	p2	(TA)9	18	255	272 CACAGAAATCACGTTGCAAAA
scaffold249883_11.[illegible]	1	p2	(TA)7	14	167	180 TGTGGCATCCTATCAACCCT
scaffold249933_12.[illegible]	1	p2	(AG)6	12	137	148 ACCTCCCCTGCTCCATACTT
scaffold250351_12.[illegible]	1	p2	(AT)9	18	874	891 GGCCATTCACTTCAATGCTT
scaffold250610_11.[illegible]	1	p2	(GA)6	12	31	42 TTGCCGATTAGGATTTCGTT
scaffold250906_11.[illegible]	1	p2	(TA)6	12	41	52 CAAGCACTATGAAAGCTAAATACTAC
scaffold251053_11.[illegible]	1	p2	(GT)6	12	207	218 ACGCTTGTCCCCTTAATCCT
scaffold251127_15.[illegible]	1	p2	(TA)10	20	381	400 TCAATTCCGAAAATCAAGGC
scaffold251245_12.[illegible]	1	p2	(GA)6	12	805	816 TCGTGTGTGTGAGTGAACCA
scaffold251435_13.[illegible]	1	p2	(TA)10	20	329	348 AGGGAGGAATAGAGCCGAAA
scaffold251609_15.[illegible]	1	p2	(TA)6	12	158	169 AACCGTAGGCTTGCTTCTGA
scaffold251710_15.[illegible]	1	p2	(TA)6	12	1319	1330 TCCAGTGACAAAAACCGACA
scaffold251777_15.[illegible]	1	p2	(GA)6	12	1904	1915 CAGACAGAGAGATGCCACCA
scaffold252123_12.[illegible]	1	p2	(AT)7	14	300	313 CCAATTGATCGGGAATTGAT
scaffold252167_13.[illegible]	1	p2	(AT)6	12	627	638 TTGGAGCTTGCTGCAATTTT
scaffold252283_11.[illegible]	1	p2	(TA)8	16	267	282 AGAGTTTTGCGTGCATGTTG
scaffold252301_13.[illegible]	1	p2	(AT)6	12	108	119 ATCCATCCTGTTTCTCCCCT
scaffold252337_13.[illegible]	1	p2	(AT)11	22	919	940 CAAAATCCGTTACATATCTTGGG
scaffold252587_14.[illegible]	1	p2	(AT)9	18	633	650 AACCCAACCCACAATAAACG
scaffold252630_13.[illegible]	1	p2	(TA)6	12	109	120 CCACCGAGCACCAAGTAAAT
scaffold252825_11.[illegible]	1	p2	(TC)6	12	162	173 AGTTGGGCTTAATGGTGTGG
scaffold253319_13.[illegible]	1	p2	(AG)6	12	103	114 GGAGATGGAGAGAAAGTGTCG
scaffold253770_11.[illegible]	1	p2	(GT)6	12	218	229 GAATCAACTCCAATCCGGTG
scaffold253906_16.[illegible]	1	p2	(TA)6	12	517	528 AAGGGGTTTTCTCTGGATCG

scaffold254087_16.(	1	p2	(CA)6	12	635	646	AATGGGACCTCCTTTACGCT
scaffold254272_14.8	1	p2	(AG)6	12	62	73	GTGGATGATGAGGAGGAGGA
scaffold254351_14.4	1	p2	(CT)10	20	604	623	TACTGCTCTTGAACTCGCCA
scaffold254451_12.1	1	p2	(CT)6	12	38	49	TTACAATGGCCAAGAGGATT
scaffold254628_13.9	1	p2	(AG)8	16	195	210	AGTTGGTCATTTGAAGCGAGA
scaffold255067_13.7	1	p2	(AG)6	12	105	116	TTTTGTTTGTTCCCAGGTTG
scaffold255334_11.9	1	p2	(AG)6	12	234	245	CTTCCTCTCCCTTCCGTTTC
scaffold255358_14.1	1	p2	(GA)7	14	409	422	AGATTTGAACTTGGAGGGCA
scaffold255458_13.8	1	p2	(CT)6	12	106	117	TGCATGGTTAATGTTGTAAGGG
scaffold255463_13.7	1	p2	(TC)6	12	201	212	TGCGTCGTGTTCTGATTTTC
scaffold255507_13.5	1	p2	(AT)7	14	52	65	AGGGCTGAGTACACGAATGC
scaffold255617_18.9	1	p2	(TA)6	12	171	182	GCTTTCAAGGAGTGAGGCAC
scaffold255638_14.2	1	p2	(TA)9	18	262	279	TTGGGATCTAGGCAATCCAG
scaffold255727_14.(	1	p2	(TA)6	12	3411	3422	TCCAGTTTTCAATGGTGCAA
scaffold256399_11.2	1	p2	(AC)6	12	181	192	TTCTCACATCCTTCTCGCCT
scaffold256411_11.9	1	p2	(AT)7	14	601	614	TTGCATGCCACCTTTATTTT
scaffold256973_16.1	1	p2	(AG)8	16	5940	5955	ACTGGCCGGCTAAGTTGATA
scaffold257036_13.9	1	p2	(TC)6	12	37	48	GAATACCGCTCGTAAGTCCG
scaffold257089_10.9	1	p2	(TA)8	16	331	346	TGATTTTGGTGTGTTGGCAT
scaffold257472_14.1	1	p2	(CT)6	12	1522	1533	TGATCATACCAAGGCGATCA
scaffold257487_14.(	1	p2	(CT)7	14	485	498	CATATGGCGCTCCACCTACT
scaffold257844_15.7	1	p2	(AT)6	12	100	111	TGAAATCTCCCGTACTTCCG
scaffold257958_11.2	1	p2	(TA)6	12	177	188	TGCCCATACGGTTGTATGAA
scaffold258004_11.4	1	p2	(TA)8	16	52	67	TTTTATCTGAATGCTAGATGTTTGG
scaffold258131_16.7	1	p2	(TC)7	14	281	294	GAAAAATAGCATGCCTTCGC
scaffold258209_14.5	1	p2	(AT)7	14	358	371	ACGGAAATACGAATGCGAAA
scaffold258229_12.1	1	p2	(AC)6	12	53	64	TGTGTACTTCCCTACTCATAAGCAA
scaffold258504_11.6	1	p2	(CT)6	12	272	283	GTACAGCCATTCGTCCACCT
scaffold258753_15.6	1	p2	(AT)9	18	1033	1050	TTAGCATGTGGGAAGGGTTC
scaffold259181_16.(	1	p2	(AC)6	12	3523	3534	ACCGGCGTATAAAGACATGC
scaffold259281_14.(	1	p2	(TA)9	18	184	201	TATGATGGGAAGGGTGGGTA
scaffold259290_15.8	1	p2	(TA)8	16	205	220	TCACGAAAACACGACACACA
scaffold259505_15.6	1	p2	(AG)7	14	425	438	GGTGGGAGCAGACAGAAAAG
scaffold259609_13.8	1	p2	(TC)7	14	461	474	GAAGCGCAGTCATTTAAGGC
scaffold260213_12.8	1	p2	(CT)6	12	51	62	CTCATTGTAATCTCTCTCCAAAGTT
scaffold260377_12.4	1	p2	(GA)6	12	55	66	CACCTTCATCTTCACCACCA
scaffold260493_16.1	1	p2	(CT)7	14	362	375	ACACTAAACCCGCGTATTGC
scaffold260799_15.7	1	p2	(GT)6	12	268	279	TAGGATACCCTCCCAAACCC
scaffold260844_12.1	1	p2	(GC)6	12	223	234	ACGAGCCTCGAGGTAGCTG
scaffold260952_17.7	1	p2	(AG)6	12	74	85	GTCCACGGTGGTTTGAGAGT
scaffold261186_13.7	1	p2	(AG)6	12	615	626	CGAATGGGAGAGAGTGGATG
scaffold261262_12.9	1	p2	(AG)7	14	49	62	CAATTACAATGTTTATGAAAATCCA
scaffold261323_13.8	1	p2	(TA)8	16	387	402	CTCAAACTTCAGCCCGTACC
scaffold262190_17.(	1	p2	(CT)7	14	52	65	TCCTCACTCTCACTAGCCGC
scaffold262347_14.6	1	p2	(AG)6	12	236	247	ATATAGCCGTTGGGCTTGTG
scaffold262529_14.(	1	p2	(TC)6	12	36	47	CATCAACTCACCCTCACATTTC
scaffold262560_12.5	1	p2	(AT)6	12	694	705	TCCAAGAAACTACGCCAACA
scaffold262870_11.9	1	p2	(AT)10	20	144	163	TGGAGTTTAAATGTAGAGGCGA
scaffold262937_14.4	1	p2	(TA)7	14	48	61	TTTAGTGTTTTGACATGATTACTCG
scaffold262944_19.4	1	p2	(CT)9	18	327	344	TTCGAACGAAAACGTGTCAG
scaffold263142_14.2	1	p2	(AC)6	12	1693	1704	GCATTAGCAAATCCCGAAAA
scaffold263490_13.1	1	p2	(TC)6	12	168	179	ATGTTCGAAGCAGCCAAACT
scaffold263539_11.1	1	p2	(AG)7	14	208	221	TGTTCCGGTCCGAGTTTATT
scaffold263559_17.1	1	p2	(CT)6	12	90	101	TCCTTCTTAAAGGTCTTAATCGGA
scaffold263571_16.5	1	p2	(GA)7	14	464	477	TTGGGGGTTTAGGGTTCTCT
scaffold263572_15.9	1	p2	(AT)6	12	2900	2911	CCGTCGAACTACCGGTTCTA
scaffold263702_11.(	1	p2	(TC)7	14	262	275	ATTGAAACTACGTCCACGGG
scaffold263874_14.8	1	p2	(TC)6	12	232	243	AGACCCAACTACCAAACCCA

scaffold263950_16.8	1	p2	(AT)7	14	2151	2164	ACGATCCTACACAATGGCG
scaffold263964_17.0	1	p2	(TG)7	14	150	163	CATGATTGGGGACTGACCTT
scaffold264109_16.9	1	p2	(AC)6	12	240	251	TGGCAACAAACAAAGCTGAG
scaffold264305_16.9	1	p2	(AT)8	16	242	257	GGTGATGATGGAAAACCTGG
scaffold264356_11.2	1	p2	(GA)8	16	284	299	CCAAGGATCACAAGTGGATG
scaffold264494_12.5	1	p2	(TA)8	16	337	352	TTTTGTCGGCCATTTTCTTT
scaffold264729_11.8	1	p2	(AT)7	14	159	172	TGATTGATCTTAACTGCAAAAGAGA
scaffold265133_18.5	1	p2	(AT)6	12	69	80	TTCACGAATAAACATGTTCCAGA
scaffold265371_13.4	1	p2	(AT)6	12	199	210	CGCCCACAATCCTGATATTT
scaffold265627_13.3	1	p2	(CT)9	18	416	433	CGCACCTATTAGGGTCGGAT
scaffold266076_17.3	1	p2	(CT)7	14	631	644	TAACGAGAAAATTCGGCCAC
scaffold266140_11.5	1	p2	(TC)6	12	142	153	CCGAGGTGGAGTAGTATCCG
scaffold266357_14.2	1	p2	(AG)9	18	2040	2057	CTAGAACCCCCTCATCCGA
scaffold266693_12.8	1	p2	(AT)10	20	448	467	GGATCGTAGGAGCAGATGGA
scaffold266745_10.8	1	p2	(AT)7	14	257	270	TTTTCGCAAGCCACTAAAAA
scaffold266766_15.4	1	p2	(TA)9	18	2350	2367	CAGGAAAGAAATGGGCTTCA
scaffold266830_13.4	1	p2	(TA)8	16	115	130	AAGCGAAACTGTTGTATGCTG
scaffold266838_11.7	1	p2	(TA)6	12	119	130	TCGAGCTGTTATGCGTTTTG
scaffold266841_15.7	1	p2	(AT)6	12	120	131	CCCGAGTTGAGGTCCTGATA
scaffold266875_13.5	1	p2	(AT)8	16	249	264	ACTCAACCGATTGACGATCC
scaffold266923_15.7	1	p2	(CT)6	12	204	215	TTGGAAGATGGAGAAATAAGGC
scaffold266956_16.8	1	p2	(GA)7	14	2016	2029	TGGTTTGGTTGTCACTTTCTTG
scaffold267035_12.1	1	p2	(TA)6	12	169	180	TTAGTTAGCAAGGGGGAGGG
scaffold267046_17.2	1	p2	(CT)7	14	469	482	AACCCATACTTAATTCATAACCCAA
scaffold267279_10.0	1	p2	(GA)6	12	198	209	CTGCAGACATTTCTCGGTGA
scaffold267310_14.0	1	p2	(TA)6	12	70	81	TTCGATGATAACTATGGGTTTTG
scaffold267411_18.0	1	p2	(TA)7	14	354	367	CTCTCAATGGACCAGCACCT
scaffold267639_13.8	1	p2	(TA)11	22	504	525	GATGGAGAAGTTACGATGGCA
scaffold267677_15.4	1	p2	(AT)7	14	51	64	GGTTTTGACATAACTTAGGTGTTTTG
scaffold267732_13.1	1	p2	(AG)7	14	503	516	CTGACACAACCCTCCATGTG
scaffold267791_15.3	1	p2	(GA)6	12	632	643	AGGGAAAAAGACATCAGCCA
scaffold267839_15.1	1	p2	(TA)8	16	1839	1854	TGCTCCGAGACCTTAGACTCA
scaffold267868_11.8	1	p2	(AT)8	16	254	269	AGAATCCCGAGATTTCATGC
scaffold267874_10.9	1	p2	(TA)6	12	167	178	GCTGGAAGATAAGATGTGAACG
scaffold267986_13.7	1	p2	(AC)7	14	429	442	GACAAAGCTAACGGAACCCA
scaffold268090_11.2	1	p2	(TA)7	14	1187	1200	GTCCAAAATTGTGTTACGGG
scaffold268098_13.3	1	p2	(CT)6	12	928	939	GAAGAGAAAGCGCGATCATT
scaffold268137_14.6	1	p2	(AT)6	12	68	79	GTGGGTTAGCACGTGATCG
scaffold268230_18.3	1	p2	(GA)8	16	3843	3858	GGGATTTAGGCCAAACGATTA
scaffold268422_16.8	1	p2	(GA)8	16	1713	1728	AATTAGTCGTGGCCGTGAAC
scaffold268616_12.3	1	p2	(TA)9	18	487	504	GCCATGATCTCATGATTCAAAA
scaffold268741_11.6	1	p2	(TA)8	16	260	275	TTCGGTTAATCCAAAGGTCAA
scaffold268915_12.1	1	p2	(TA)10	20	85	104	AATTGAATGGCAAGGATTGG
scaffold268949_10.8	1	p2	(TA)11	22	716	737	TGTAATAGTACATACAATGCAGGGA
scaffold269277_14.0	1	p2	(GA)6	12	76	87	AGCCCCAAACTTTGCTGAAT
scaffold269502_18.6	1	p2	(TG)6	12	105	116	GATGTGGAGATGGGCTGACT
scaffold269522_15.8	1	p2	(TC)6	12	117	128	CGCGCACAAAGAACTTTACA
scaffold269583_17.1	1	p2	(CT)7	14	72	85	CACTTGGACCTGAAAAGGGA
scaffold269682_13.6	1	p2	(CA)8	16	3628	3643	GCCTTTTGGCATGCAGTATT
scaffold269694_15.4	1	p2	(TC)7	14	404	417	CGTGCAAGCATGAATCACTC
scaffold269737_19.7	1	p2	(GT)6	12	565	576	GGAATGCGAGATGGAAGAAA
scaffold270090_16.1	1	p2	(TA)7	14	493	506	TGTCTCGGTGTTCATGGATT
scaffold270168_13.6	1	p2	(TA)8	16	508	523	GAAAATTGGTTGTTATCCACAAG
scaffold270307_11.6	1	p2	(CT)6	12	141	152	CAATACCGTGACATGCCTTG
scaffold270452_15.7	1	p2	(AG)8	16	4383	4398	CAAGAAAATCCCACCTCCAA
scaffold270525_11.6	1	p2	(TA)6	12	178	189	TCATGAGAAGCGTGGATTTG
scaffold270569_12.5	1	p2	(AG)7	14	53	66	GGCCACCACACGAGTATATCA
scaffold270585_14.9	1	p2	(AC)8	16	2844	2859	TCCTCTTTCTTTTTGCGAGC

scaffold271008_12.3	1	p2	(TA)9	18	330	347	TGTAGGCGCCTCTGATCTTT
scaffold271098_10.0	1	p2	(AT)6	12	117	128	TCGCGTTCGACTCCTTAACT
scaffold271239_15.6	1	p2	(CT)6	12	113	124	TGCTCTCTTTGTAATCTCTCCCA
scaffold271381_14.6	1	p2	(TG)6	12	674	685	GTCAGCAGAGGAGGAGTTGC
scaffold271462_12.4	1	p2	(CT)7	14	223	236	GCGAAATCAAAGAATCCGAA
scaffold272471_14.9	1	p2	(AT)8	16	683	698	GGAAATCAATCACAGCCGTT
scaffold272573_13.1	1	p2	(TA)9	18	586	603	TAAAAAGATGAGCCCACCCA
scaffold272606_16.1	1	p2	(TA)8	16	390	405	TATGATGGGAAGGGTGGGTA
scaffold272617_14.7	1	p2	(CT)6	12	293	304	GAAATGAAATCCTGGCTTCG
scaffold272685_18.7	1	p2	(TC)8	16	76	91	TCAGAAACTTGGAGCAGAAGAA
scaffold272917_12.5	1	p2	(TC)6	12	117	128	CAAGCCCAAAAGCACAAAGT
scaffold273254_11.4	1	p2	(CA)9	18	881	898	TCTTTTCAATATTTATACCAACCGA
scaffold273522_15.0	1	p2	(TC)7	14	1869	1882	CCCCAAACATCACACACAAG
scaffold274081_14.8	1	p2	(TC)6	12	195	206	AAATGGTTGTTTGTCCCCAA
scaffold274090_18.8	1	p2	(TA)11	22	523	544	TACTTGCTCCCGAGCTCAAT
scaffold274243_12.2	1	p2	(TC)7	14	603	616	GATTCCCCATCACCATCATC
scaffold274368_13.6	1	p2	(GT)7	14	412	425	TTGACATGATTTGTTAACGGG
scaffold274576_12.1	1	p2	(TA)6	12	133	144	TTTTTCATCCCGTTTCATCG
scaffold274662_10.1	1	p2	(TC)7	14	104	117	GAGGACACGTTGGACCTGAT
scaffold275039_13.5	1	p2	(CT)6	12	48	59	CTCACTCTCACTAGCCGCAG
scaffold275042_18.0	1	p2	(CT)6	12	105	116	CAATCATCTAGCCCTCTAACATCTC
scaffold275207_15.8	1	p2	(GA)7	14	805	818	TTCTCTGTCGCCAGATCCTT
scaffold275653_18.0	1	p2	(GA)6	12	119	130	AGAGCCACGTTAGGCTCAAG
scaffold275672_13.0	1	p2	(AG)8	16	350	365	TGCAAACCCATTTAAAACCC
scaffold275848_11.7	1	p2	(TA)7	14	905	918	GATATGGAAGAGTGGCACCG
scaffold275876_14.4	1	p2	(CT)6	12	384	395	CCGGCTACTTGTGAATTCCT
scaffold276066_11.0	1	p2	(AT)8	16	744	759	ATGTCGATGAACACGGATGA
scaffold276335_17.6	1	p2	(AT)6	12	1367	1378	ATTTTAGAACGGGGGTGGAC
scaffold276343_16.7	1	p2	(TG)7	14	263	276	TGGTTGGAGGAAAATCTTGTG
scaffold276448_12.7	1	p2	(CA)6	12	104	115	CGTGTAATCACCATCATCGG
scaffold276908_19.2	1	p2	(CT)8	16	65	80	GGAGTCTCTCTCGCTGATGC
scaffold277841_10.5	1	p2	(TA)8	16	588	603	GGGACAAAGCAAAACGAAAA
scaffold278081_13.6	1	p2	(GA)6	12	225	236	CGGCTCCTTTGTGTAGTTCC
scaffold278301_13.6	1	p2	(AT)7	14	374	387	GCCACGTGTGTTCATTGAGA
scaffold278354_13.2	1	p2	(AT)6	12	1212	1223	CGAAAACCTTGAGAGCTCGTT
scaffold278390_13.7	1	p2	(AT)6	12	660	671	CGGAGTTTCGTATCTTGGGA
scaffold278514_14.1	1	p2	(AT)9	18	2291	2308	AGGGGAAAAGAAGAGTGGGA
scaffold278538_12.9	1	p2	(AT)7	14	787	800	TCCATTTGCATATTATTTTTGCAC
scaffold278679_13.5	1	p2	(TA)6	12	1295	1306	TCAAGAGCCTCCGACTTTTC
scaffold278700_12.7	1	p2	(AG)14	28	326	353	CGAGCGAGAGGAGAGAGAAA
scaffold278911_10.1	1	p2	(CT)7	14	30	43	CAAATGGCCAAGAGAGTTTACA
scaffold279147_13.9	1	p2	(AT)6	12	532	543	GCCTGAATCGACGACAAACT
scaffold279349_10.5	1	p2	(TA)10	20	215	234	CGATTTGGATTCGAATTCTGT
scaffold279401_17.1	1	p2	(TC)6	12	772	783	CAAAGAACGTGCACACACAA
scaffold279605_16.0	1	p2	(AG)10	20	515	534	ATGTGAGATGGTGGTGGTCA
scaffold279810_16.4	1	p2	(GA)7	14	2147	2160	AAGGTAAGGGGGTGGGTATG
scaffold279907_12.7	1	p2	(TA)8	16	272	287	TTTGAAATGGATGGTGGTGA
scaffold280012_10.9	1	p2	(GA)7	14	293	306	AGGGGCTTAAGATCCCAGAA
scaffold280044_10.2	1	p2	(TA)8	16	168	183	AAAAACCCTTCCGCCCTT
scaffold280052_18.0	1	p2	(TA)6	12	408	419	CTACTCTCAATTCCCGTCGC
scaffold280205_13.0	1	p2	(CT)6	12	575	586	CTGCGCACCGATATGATAGA
scaffold280254_14.9	1	p2	(CT)6	12	145	156	CCCTAAGATAATGCCACCCA
scaffold280275_13.3	1	p2	(TC)6	12	322	333	GGCCCTAACTTGACCCAGAT
scaffold280276_14.5	1	p2	(AC)6	12	42	53	GAACAAAGCTTGTCACACGG
scaffold280280_15.4	1	p2	(GC)6	12	732	743	CCCAGACTCGATAACTGGCT
scaffold280296_14.8	1	p2	(GT)7	14	433	446	AGGATTTGAGTTGGCTGTGG
scaffold280734_17.2	1	p2	(GA)6	12	166	177	CCAAAAGACGATCTGGGATG
scaffold280804_11.4	1	p2	(TA)8	16	307	322	ACGCTGCCTTGTCTTGATTT

scaffold281078_16.6	1	p2	(TA)8	16	294	309	TGCGAGCCATCAACATGTAA
scaffold281148_16.1	1	p2	(AT)9	18	3364	3381	TCATGCACGAAACTCTTGGA
scaffold281530_17.8	1	p2	(TA)8	16	343	358	TGTATTCCTTGAATATGAGAACTTGT
scaffold282023_13.7	1	p2	(TC)6	12	440	451	CCAAACATGGGGATATGAAA
scaffold282127_12.9	1	p2	(TC)6	12	411	422	TATTTTCCACCAACCCAACC
scaffold282163_14.0	1	p2	(GA)6	12	183	194	CTTACGATCAGGGTTGTCCG
scaffold282572_17.3	1	p2	(CT)8	16	4521	4536	TTTTCTTGGCCATAGGCATC
scaffold282679_14.7	1	p2	(TG)6	12	1231	1242	GCATGCATCCACCACTCTAC
scaffold282734_11.7	1	p2	(TA)8	16	169	184	AAATCTGAATTTCGATGCCG
scaffold282744_15.1	1	p2	(TC)9	18	1097	1114	CCCAAATTCACATGCTTTCC
scaffold282970_14.8	1	p2	(TA)9	18	3304	3321	CCATTAGCACAACACATCGC
scaffold283163_10.4	1	p2	(AT)11	22	77	98	TTTAAATGCACACCCCTTTCT
scaffold283293_12.0	1	p2	(TA)7	14	176	189	TAAAATTCACCCAAGACGGC
scaffold283313_16.2	1	p2	(TC)7	14	233	246	GAGAGAGTTGATTGTGGGGG
scaffold283358_10.3	1	p2	(GA)6	12	162	173	GTTGGAATCAGTAATGGCGG
scaffold283626_12.0	1	p2	(GA)6	12	89	100	ATACAGTGCAGATGCGCGTT
scaffold283810_15.0	1	p2	(TC)9	18	189	206	ATTTCGCTGCACTCCAACAC
scaffold283878_16.2	1	p2	(TA)6	12	241	252	AAATGTTTCAGCCCATAGAATTT
scaffold283992_12.0	1	p2	(AG)6	12	99	110	AGCTCACGGTGGAAGAAGAA
scaffold284077_13.0	1	p2	(TA)7	14	197	210	CTTCTTCCCAAATTGCCCTT
scaffold284078_18.1	1	p2	(TC)6	12	1450	1461	CATAATGAGAGGCGGTTGGT
scaffold284128_11.9	1	p2	(AT)8	16	192	207	TGTATGCACCTGAACATGCC
scaffold284468_13.7	1	p2	(TC)6	12	319	330	GAGTCCCCTTGGTCTGATGA
scaffold284492_12.5	1	p2	(TA)11	22	845	866	TTACCCCCATATGCAGTGGA
scaffold284545_14.4	1	p2	(TA)7	14	142	155	TTTGGCTTGTTTTTGCACCT
scaffold284750_11.2	1	p2	(TA)8	16	449	464	CCCAGCTATATTGCATTCACAA
scaffold284883_10.4	1	p2	(CT)6	12	446	457	AGGGGGCTTTTAGACACGTT
scaffold284909_13.9	1	p2	(AT)9	18	224	241	GAGGAACCTCTTTAAATGCAGTG
scaffold284953_17.4	1	p2	(TG)8	16	370	385	CCGACGGTTGTAGTGGAGTT
scaffold285210_12.9	1	p2	(TA)12	24	482	505	TGTAATGCGCACGTTGAAAT
scaffold285289_12.3	1	p2	(GA)7	14	49	62	TCGGCTGTCAAAGACAAAAA
scaffold285332_15.1	1	p2	(CT)9	18	791	808	GATGAAGGGGCCTATGTGAA
scaffold285381_17.6	1	p2	(TC)6	12	708	719	GCCCCACCCTTCTATCTCTC
scaffold285628_10.7	1	p2	(AT)6	12	67	78	GGCTTTCTTAACTTGTTGGAACC
scaffold285629_14.8	1	p2	(GA)6	12	546	557	ATGATGGAATTTGGGTGCTG
scaffold285644_16.4	1	p2	(AC)6	12	1851	1862	AGGGGTTGATTGCTACGATG
scaffold285829_14.4	1	p2	(GT)6	12	453	464	CTGGGGGAGATTTGTTGAGA
scaffold285921_12.2	1	p2	(TA)7	14	103	116	TCTCACGCACCTTCACTCAC
scaffold286109_21.6	1	p2	(GA)6	12	39	50	CAGATTTAAACAGTGGCTGCG
scaffold286375_10.6	1	p2	(AT)7	14	246	259	GTTCGAAAGCCTACCTCGCT
scaffold286603_11.3	1	p2	(GA)6	12	236	247	GGTGTCTGGAACAAGGGAAA
scaffold286753_12.9	1	p2	(TA)6	12	1348	1359	TGGTTAGTATTGGAACGCGA
scaffold286759_12.6	1	p2	(AG)8	16	342	357	GTTATGCGATCTGCTCGTGA
scaffold286835_11.6	1	p2	(AT)8	16	1204	1219	TCTGATTCGGGAAAACATGA
scaffold287109_14.2	1	p2	(TC)11	22	1469	1490	ATTGGTTTGCAGGTACGAGG
scaffold287184_14.4	1	p2	(AC)6	12	2263	2274	TAAAACTGGCCCCAAAATGA
scaffold287248_17.9	1	p2	(AT)7	14	202	215	AGGAAGCGAGGTAGGCTTTC
scaffold287508_15.2	1	p2	(TC)6	12	4524	4535	CGAGAGAGGTTCGGACGTAG
scaffold287640_11.2	1	p2	(AT)6	12	197	208	CAATTTATTTACCGAGGGTTCG
scaffold287841_11.5	1	p2	(TA)7	14	436	449	AATTATCGTCCGTCCCCAAG
scaffold287872_11.2	1	p2	(AT)11	22	352	373	GAAATATCCACCGAACGGAA
scaffold287896_15.0	1	p2	(AG)7	14	681	694	GGGAAGAGGGGACTGAAAGT
scaffold288123_10.7	1	p2	(AT)7	14	70	83	TCATTTAGAATTGGTGGAATGAT
scaffold288599_11.4	1	p2	(AT)10	20	294	313	TTCGAGTTATGAAGTTTGCACC
scaffold288721_10.3	1	p2	(AT)8	16	222	237	CCTCCGTGGACGTAGATCAT
scaffold289165_10.4	1	p2	(AT)8	16	220	235	TAACGTCCCAGATTTGCTCC
scaffold289410_15.6	1	p2	(AT)10	20	5550	5569	TCTCGTAAACTCCCCCTCAA
scaffold289501_12.1	1	p2	(TA)7	14	355	368	CAATAGTGACGTTCGCCTCA

scaffold289506_16.4	1	p2	(AT)8	16	5484	5499	TGCAGGTTTGTTGAATTTCG
scaffold289566_17.0	1	p2	(GA)6	12	41	52	CACGAGAGTTGAAAGGGAGC
scaffold289905_15.1	1	p2	(TA)7	14	239	252	CAAATGGAGGTGAGAGGTTTG
scaffold290213_14.3	1	p2	(CA)8	16	190	205	TTCCTCTCTTTTTCCAGCCA
scaffold290369_11.4	1	p2	(TC)7	14	753	766	GGCCAGTAACATTACCCACCT
scaffold290517_10.9	1	p2	(GA)6	12	112	123	AATAGAGCAGCAGACGCGAT
scaffold290802_17.9	1	p2	(CT)7	14	89	102	GAGGCGGTTAAAGAAACGTG
scaffold290803_12.4	1	p2	(CT)10	20	159	178	AGATGAAGCTGAACCGGCTA
scaffold291072_10.9	1	p2	(AT)6	12	276	287	GTCGTATACTGCCGCCACTT
scaffold291225_11.7	1	p2	(CT)6	12	107	118	GGAGAGGAATCAAGTCGACAA
scaffold291416_16.2	1	p2	(AT)6	12	166	177	TGATGAAGAAATGGAAGGGG
scaffold291781_14.9	1	p2	(GA)6	12	1592	1603	CGTGTTTATCCTCACCCGTT
scaffold291813_14.4	1	p2	(AT)6	12	941	952	AAGGCTAGCACAAGAATGGC
scaffold291860_10.2	1	p2	(CT)6	12	67	78	CAAATGGCCAAGAGAGTTTACA
scaffold291947_16.6	1	p2	(AT)7	14	183	196	CAAACATAGAAACATGGACGC
scaffold291962_14.6	1	p2	(TA)6	12	407	418	ATTCATACCTTTCTGCCCCC
scaffold292006_17.5	1	p2	(AT)10	20	939	958	TGTCATGTATAGGCGCATCTG
scaffold292035_12.0	1	p2	(TA)11	22	286	307	TCTCAAGCTTGTCAACCCCT
scaffold292338_14.2	1	p2	(AG)6	12	1922	1933	AGCGAGTTCCGAAATGAAGA
scaffold292617_10.7	1	p2	(GA)6	12	88	99	TTCTGTCTTGGATGATGAGGC
scaffold292629_17.6	1	p2	(TG)7	14	205	218	GGCAATTATGTTGGAGGTGG
scaffold292631_10.9	1	p2	(CT)6	12	72	83	CACATACCTCACCTTCTCTCCA
scaffold293334_15.3	1	p2	(AC)7	14	178	191	AAGTCCACCACGATGTCCTC
scaffold293353_13.7	1	p2	(AG)7	14	363	376	TTTGTCCCATAAAACTCGCC
scaffold293385_18.3	1	p2	(GA)6	12	282	293	TAGGGGAGACAGGGGGTAAC
scaffold293435_20.6	1	p2	(TC)7	14	208	221	GGGGTTGAAGTAAATGAGGTG
scaffold293581_10.2	1	p2	(GA)7	14	256	269	TGTCAAGATTGGTCTCGTTTT
scaffold293743_14.3	1	p2	(TC)6	12	763	774	TACCCTGGTTTTGGAAGGTG
scaffold293793_12.0	1	p2	(TC)6	12	167	178	CCTAAGTGCCTACGTGTTGGA
scaffold294082_13.3	1	p2	(AC)10	20	273	292	CCAGATGCCTTCATTTTTCA
scaffold294170_16.3	1	p2	(AT)9	18	1194	1211	CGACGAGGCTTGGATTTCTA
scaffold294223_16.3	1	p2	(TA)6	12	915	926	TCTGGCTCTTTTCCAGAGCA
scaffold294327_14.3	1	p2	(TG)8	16	211	226	GAAGTTTTGATCAATTTTCACTCG
scaffold294351_11.8	1	p2	(TA)6	12	176	187	CAACTCCACATGTTGCCATC
scaffold294353_15.5	1	p2	(TA)8	16	4448	4463	CCATGAGTCCATGACCCTTC
scaffold294394_18.6	1	p2	(CT)8	16	534	549	GGAGTCTCTCTCGCTGATGC
scaffold294559_15.3	1	p2	(TA)6	12	750	761	TCTTCTATAAGGGCGGCTGA
scaffold294566_17.2	1	p2	(GA)6	12	47	58	GGAGAAAAAGAGATTGAGAGGGA
scaffold294622_11.7	1	p2	(TA)6	12	166	177	TTCAAACGTGAGGTGATGGT
scaffold294677_15.6	1	p2	(GA)6	12	529	540	TAGTTTTCAGTTCGGGCGAT
scaffold294795_14.8	1	p2	(TC)6	12	87	98	TACGTTGGACCTGAAAAGGG
scaffold294813_17.3	1	p2	(TC)6	12	353	364	GGCAAAGGAATACCTCCGAT
scaffold295048_15.0	1	p2	(CT)6	12	112	123	CCTTGAACCGAGAAAATGGA
scaffold295139_16.9	1	p2	(TC)7	14	302	315	CCTTGGCGGATCATATCACT
scaffold295140_11.3	1	p2	(AT)6	12	1100	1111	ATGGACGTCGTGACTGCATA
scaffold295210_13.4	1	p2	(TA)8	16	105	120	ATGAGTTATCACCGGATCGC
scaffold295275_10.8	1	p2	(TA)8	16	1018	1033	TATGTGATAGGCTACGGCCC
scaffold295510_14.5	1	p2	(AG)6	12	52	63	AGCTGTTAGGGTTGTATAGTGAGTAG
scaffold295532_18.7	1	p2	(AT)6	12	209	220	CCCCAGTAGTTCCCATCAAA
scaffold295539_17.5	1	p2	(CT)7	14	85	98	TACCCAACCCACTAACCCAA
scaffold295704_19.0	1	p2	(AT)6	12	1225	1236	GAAGCAAAGAGGATCCCACA
scaffold295897_10.6	1	p2	(TC)6	12	47	58	CAATGTTACAAAATGGGCGA
scaffold296494_13.0	1	p2	(AT)6	12	902	913	CATTTGATAGTTGTTTTTGTGGTGA
scaffold296635_14.6	1	p2	(GA)6	12	93	104	TGACTTATGGGGGCTCAAAC
scaffold296661_10.6	1	p2	(AT)12	24	267	290	GGGGCGTGACATTATGAAAT
scaffold296714_16.5	1	p2	(TA)10	20	1249	1268	TTGGATTGATTATGCTTTGCC
scaffold296880_13.2	1	p2	(AT)6	12	573	584	CCCAGACACCGTAAGGAAAA
scaffold297488_11.4	1	p2	(TC)6	12	79	90	ATTCCGTTTTTAAGTCCGGC

scaffold297588_16.4	1	p2	(AT)7	14	196	209	ACCCTTTTGTAACATAAAAATTCTAAA
scaffold298109_13.5	1	p2	(AT)7	14	155	168	AAGTCCCAACCACTTTCACTG
scaffold298158_13.6	1	p2	(TA)6	12	502	513	TTTTCTGCAAATTTGAGGGC
scaffold298289_11.4	1	p2	(TA)8	16	93	108	ATTGCCCACGAGAGAGATTG
scaffold298920_13.1	1	p2	(GA)6	12	38	49	TCAACAGAGAATGCAAGCAAA
scaffold298997_13.2	1	p2	(AG)9	18	151	168	TATGCGGGCTGGAAGACTAC
scaffold299095_17.6	1	p2	(AC)8	16	2119	2134	GAAAAGCACCACACTGCAAA
scaffold299249_14.1	1	p2	(CT)7	14	177	190	CCTAGCAAGGCTTCCATCTG
scaffold299354_10.4	1	p2	(TA)7	14	216	229	ACGCACACACTCTCACGAAC
scaffold299435_17.7	1	p2	(CT)6	12	4254	4265	CCATTTCAGTTGCAGAAGCA
scaffold299742_13.7	1	p2	(GA)7	14	192	205	CCTTGAAGACAATGAAATTTTGG
scaffold299756_12.5	1	p2	(GA)6	12	162	173	AAACACGCAACACAAAGCAG
scaffold299799_18.6	1	p2	(AG)7	14	243	256	TCGGGAGTCTGTATTGAGCC
scaffold299860_16.9	1	p2	(GA)6	12	1470	1481	GGAGAAATCAAGAGGAGGGG
scaffold299876_11.7	1	p2	(GA)8	16	122	137	TTGTGGTTCGATCGTAATTGA
scaffold300019_16.8	1	p2	(CT)6	12	115	126	GGTTCTCACAGGAGGGTTCA
scaffold300023_13.5	1	p2	(TG)6	12	473	484	GCAATTGAGAGTGAGTGCGA
scaffold300209_13.4	1	p2	(AC)6	12	441	452	ACCCTCTCTGGTTCACGAAA
scaffold301074_15.6	1	p2	(TG)7	14	278	291	TTTTTACGATTTTGCCCTCG
scaffold301347_16.5	1	p2	(CA)6	12	1387	1398	GGACACGGTTTCTAATGTTTTGA
scaffold301432_11.1	1	p2	(TA)8	16	250	265	CCTTTCATGTTATTCTTTTCTCACC
scaffold301487_10.9	1	p2	(TA)7	14	165	178	AAGTTATCACCGGATCGCTA
scaffold301501_22.1	1	p2	(CT)7	14	580	593	GAACCAAAGTGAGCCCGATA
scaffold301583_19.6	1	p2	(TA)10	20	349	368	GCTGACGAGGTGATGAGTGA
scaffold301959_22.4	1	p2	(TC)6	12	103	114	ATGGATCGTATCACAAGCCC
scaffold301988_10.6	1	p2	(CT)7	14	142	155	TAGCCGATAAAAATGGCACC
scaffold302108_10.9	1	p2	(TC)6	12	239	250	CGATTCCCATACCATTCGAC
scaffold302449_10.5	1	p2	(TA)7	14	360	373	ATCCTTGACGACTGTTTGGC
scaffold303182_17.0	1	p2	(CA)6	12	893	904	TGCCAATTTGATAGAAGCCC
scaffold303184_16.1	1	p2	(TA)9	18	465	482	TCGTGGAAATTCCTTTAATGTG
scaffold303431_11.2	1	p2	(AT)7	14	457	470	TTAGCACCCGAACCAATTTC
scaffold303827_10.0	1	p2	(TC)7	14	155	168	CGGAGTGTATTGCGAACTCA
scaffold303941_20.0	1	p2	(CT)6	12	108	119	TTCTCTCTCTCGCTGCACAC
scaffold304124_20.8	1	p2	(CA)8	16	316	331	GTTTCCATGTTTCACCCACC
scaffold304672_16.1	1	p2	(TA)6	12	1403	1414	TTTGACCTCGAAGGATTTGG
scaffold304939_13.0	1	p2	(AT)12	24	467	490	GCAGGTGAGGGACAAGAATG
scaffold305168_16.0	1	p2	(TC)6	12	2194	2205	ACACCGTCAATCACAACACA
scaffold305277_12.2	1	p2	(TC)6	12	296	307	ACTGCGGATGCCCTAATATG
scaffold305452_13.8	1	p2	(TA)9	18	183	200	TGAATCCTTTTTAAACGCGG
scaffold305630_11.6	1	p2	(TA)7	14	75	88	ACCCACACCAGACCAACATA
scaffold305688_14.9	1	p2	(AG)12	24	1929	1952	CCATCTCCTCGAGCTCAATC
scaffold305885_14.2	1	p2	(TG)9	18	185	202	TCCTTTAGGCCGACAACATT
scaffold306069_12.1	1	p2	(CG)8	16	215	230	GCATCGCGTTTCAATACAGA
scaffold306095_11.6	1	p2	(AT)8	16	244	259	CCGCCATAACCTGTTCAACT
scaffold306100_19.0	1	p2	(AT)9	18	215	232	ATTGGTCGAGAGAAAGGGCT
scaffold306172_14.7	1	p2	(AT)11	22	780	801	CCATGGGAAATGGTTAGTTGA
scaffold306363_10.2	1	p2	(TA)6	12	114	125	TTGAATGGAAATACCCTCTTAGG
scaffold306451_11.8	1	p2	(TC)9	18	356	373	GTGGTATGGAAAATGGGCAC
scaffold306597_13.8	1	p2	(TC)6	12	1771	1782	TGTTTTGTAGGCCTGCCTTT
scaffold306633_15.2	1	p2	(TA)6	12	92	103	ATGCTTTTCCCCTTTTTCGT
scaffold306843_11.7	1	p2	(AT)6	12	587	598	TGACCAAAATTTAGCGAGGG
scaffold307006_15.8	1	p2	(TA)7	14	252	265	TACCGGCTTCAACAGCTTCT
scaffold307273_12.3	1	p2	(TA)6	12	31	42	GAATCTTGTCACATTTACCAATGTATC
scaffold307296_15.0	1	p2	(TA)8	16	3336	3351	GGTTTTGGGAATTCCATCCT
scaffold307602_12.2	1	p2	(AT)7	14	101	114	TTTGCACAAGTGCCAAAGAG
scaffold307896_14.5	1	p2	(AG)9	18	1772	1789	AGGTCAAGGGTACCATCCTG
scaffold308095_15.8	1	p2	(GA)7	14	1706	1719	TCTCAAGCTCCTCCAACGAT
scaffold308408_13.1	1	p2	(AT)6	12	367	378	TGGCTCATTCCCTAACTTGC

scaffold308593_15.(	1	p2	(TG)8	16	289	304	TGTAAGGGGAGCAATCGAAG
scaffold308613_16.8	1	p2	(AT)9	18	1329	1346	TCACATTCTGCGATTGCTAA
scaffold308852_12.(	1	p2	(AT)6	12	184	195	ACTCATGACCCTACCAACGG
scaffold308922_10.5	1	p2	(AT)6	12	53	64	GACCGGTTAGACCGGATTTT
scaffold308965_17.8	1	p2	(CT)6	12	230	241	AAGTCCGGCCATCTCTTTCT
scaffold309030_11.9	1	p2	(AC)8	16	309	324	TGACGAATTCTCAAGTTCCTCAC
scaffold309241_12.2	1	p2	(GA)6	12	98	109	AAGCTGCTCCTTTCCACAGA
scaffold309624_15.1	1	p2	(AT)11	22	2110	2131	ACAAGTTGAAGGATGGCCTG
scaffold309717_15.6	1	p2	(TC)6	12	313	324	AGCCAAGGTATGGTAGCCCT
scaffold309783_17.(	1	p2	(GA)8	16	143	158	GAGAGAAAAGGTGGCAGGTG
scaffold309788_11.8	1	p2	(TC)6	12	173	184	CATAGGCCAAAGCCCAAATA
scaffold309906_13.8	1	p2	(AG)6	12	708	719	TGGCCTGGCTTAGTTGTATTG
scaffold310416_10.1	1	p2	(AG)6	12	43	54	GTCTTCTCCGCCTTTGTGTG
scaffold312002_10.7	1	p2	(AT)8	16	442	457	ACGTCAGCATCTACCCCAAC
scaffold312074_10.1	1	p2	(TC)6	12	53	64	CAACACATTCAATACATTCTCCAA
scaffold312178_12.8	1	p2	(AG)8	16	517	532	AATGTTTGATGCATTTCGCA
scaffold312298_14.4	1	p2	(TC)7	14	1914	1927	CTCTCTCGCCTCGCTTTCTA
scaffold312378_13.2	1	p2	(AC)6	12	336	347	GACGAGATTGTCGAGTGCAA
scaffold312843_15.8	1	p2	(GA)6	12	1232	1243	CGACGAGAAAGTCGTGTCAA
scaffold312914_10.(	1	p2	(GT)6	12	136	147	TCATGTAAATTCTTGGTCATTCTCA
scaffold313011_13.8	1	p2	(TG)7	14	77	90	CCCAAGCTATCACTCATCACC
scaffold313094_14.2	1	p2	(TC)7	14	445	458	TGTTTTCGCGTTTCCTCTCT
scaffold313419_16.2	1	p2	(TC)6	12	101	112	GCAACGGAATACCACTCGTT
scaffold313480_13.8	1	p2	(TA)6	12	144	155	AATACGCGAGTTCATGGAGG
scaffold313665_13.2	1	p2	(AT)6	12	320	331	TGCACGTAGGCATGTTCAAT
scaffold313673_12.9	1	p2	(TA)6	12	244	255	CATTCAAGGGGCATTTTTGT
scaffold313763_11.9	1	p2	(CT)14	28	512	539	GGGCTCGAATTCAAAACTGA
scaffold313781_14.(	1	p2	(TC)8	16	51	66	GAGGGTTTTAGCGTGATAGGG
scaffold313828_16.1	1	p2	(TC)6	12	104	115	CACCCAAACCCATTATCACC
scaffold314026_10.8	1	p2	(GA)10	20	209	228	GCATTTGGAGCAAGGCTAAG
scaffold314032_15.(	1	p2	(AG)7	14	99	112	CCCATGTTGTCATTAATGTAGGG
scaffold314073_10.7	1	p2	(TA)8	16	233	248	TTCTCAGCATATGGGAAGGG
scaffold314135_14.7	1	p2	(TA)9	18	490	507	TCTGGACCATCAGATTTGGA
scaffold314230_12.(	1	p2	(GA)6	12	107	118	AGAAAATGCATGCGAAGAGC
scaffold314251_15.9	1	p2	(TA)7	14	176	189	AAAGGGAAAACAGTTGGAAGC
scaffold314688_13.(	1	p2	(CT)6	12	45	56	CAATATTACAATGGCCAAGAGAG
scaffold314815_10.4	1	p2	(GA)7	14	202	215	CATTCGGAGCAAGGAGAAGT
scaffold314816_14.9	1	p2	(AT)8	16	159	174	TTGCCATATTTAAGCCGAGG
scaffold314983_18.6	1	p2	(AG)6	12	76	87	TGTCTTTTCGTGTTGAGTCGTT
scaffold315188_14.4	1	p2	(TA)6	12	875	886	CATGATTTTGCAACTTACCATTTC
scaffold315253_13.5	1	p2	(AG)6	12	160	171	CCCTCAACAATGCAAAAAGC
scaffold315266_17.1	1	p2	(AG)6	12	603	614	TTCGGCCAAAAGAAAAGAAA
scaffold315297_15.(	1	p2	(AT)10	20	1283	1302	AGTCGTCACCCCGAACATAG
scaffold315303_15.1	1	p2	(TA)7	14	409	422	GAAATGAGAAGCCCAATGGA
scaffold315351_15.9	1	p2	(CT)6	12	441	452	CCGTTGCCTCTTTTGAACTC
scaffold315354_10.5	1	p2	(GA)6	12	83	94	GCCGGAGGTTTGATATGGTT
scaffold315615_14.2	1	p2	(AG)12	24	1613	1636	CCCTGTCTTCCAAAAACCAA
scaffold315657_12.(	1	p2	(TG)6	12	161	172	GTGGCATGGGGAAGCTACTA
scaffold315702_17.4	1	p2	(GA)8	16	73	88	CACTGTGTAGAAACATAAACTGCG
scaffold315755_15.7	1	p2	(CA)7	14	1106	1119	AGCCGTGGGACATAAGAGTG
scaffold315912_11.5	1	p2	(TA)6	12	270	281	GGGTTTTGCAGGATTGTGAT
scaffold316021_14.3	1	p2	(AT)6	12	843	854	TTGTGTTACAATGATGGGGG
scaffold316027_16.9	1	p2	(CT)8	16	442	457	GTTGGAGGTGCCAAAACTGT
scaffold316120_16.8	1	p2	(AT)8	16	113	128	GGGTGTTTTGACCTAATCGC
scaffold316285_15.2	1	p2	(CA)10	20	3770	3789	CGAAATAGGGTTGGTGAAGC
scaffold316422_10.(	1	p2	(GA)6	12	523	534	CGGAACCCTCTCTCTTACCC
scaffold316630_15.2	1	p2	(TA)6	12	412	423	GAAAACGACGAAGAAATGGC
scaffold316689_15.6	1	p2	(GA)6	12	515	526	TCAAGTGAAAATGGCAACCA

scaffold316940_11.(	1	p2	(AG)6	12	195	206	AGCTGGAATAGTCCGACACG
scaffold317113_17.:	1	p2	(TC)11	22	2104	2125	ACCGAAGTTCCTGCTCACAG
scaffold317119_14.9	1	p2	(AG)6	12	154	165	CCAAACGCATCAAATTCTCA
scaffold317153_18.5	1	p2	(AT)6	12	1544	1555	TCCATTTCACATCCGATGAA
scaffold317265_10.6	1	p2	(TA)7	14	150	163	AAACAGTCTTCGGGACACCA
scaffold317410_10.3	1	p2	(CA)7	14	896	909	TCTACGTCCACGGAGAGGTT
scaffold317520_10.(	1	p2	(AT)9	18	705	722	CCGAACTTTTGGCCTTCATA
scaffold317598_13.6	1	p2	(TC)6	12	2974	2985	AACTTCTCCTCCCAATCGCT
scaffold317609_11.6	1	p2	(GT)7	14	89	102	TACGATACCCTCCCAAACCC
scaffold317645_14.4	1	p2	(AT)6	12	2246	2257	TCTGCATGCAAGATAGTACGG
scaffold317699_16.5	1	p2	(AG)6	12	535	546	ATTGCCATCACCTTCTCCAC
scaffold317857_14.:	1	p2	(TA)10	20	772	791	TCAAAATCTTTGAAACGGGTG
scaffold317870_10.4	1	p2	(CT)9	18	422	439	ACTAGAATTCCCTCTGCCCC
scaffold317926_19.5	1	p2	(TA)9	18	447	464	GTCTGTGAGGTGGGCTGAGT
scaffold318201_11.3	1	p2	(AT)6	12	667	678	GGGGTCCCAGAAAACTGATT
scaffold318303_14.:	1	p2	(GC)6	12	148	159	ATATTCTCGCTTCTTCGCCA
scaffold318340_15.:	1	p2	(AT)6	12	554	565	TTTTGCCTCTCGCAAAAAGT
scaffold318363_14.8	1	p2	(AT)6	12	356	367	TCACCACGAAGTCTACAAAAACA
scaffold318533_14.7	1	p2	(TC)8	16	49	64	CGCGCCTACAATTCAAAGTA
scaffold318607_18.:	1	p2	(AG)6	12	449	460	ATCCCCACTTTTTCGCTTTT
scaffold318858_12.4	1	p2	(TA)9	18	587	604	CATAATGCCCTCGAATGCTAA
scaffold319053_15.:	1	p2	(AG)12	24	262	285	GGTATATGGAGGAATGCCCA
scaffold319991_15.9	1	p2	(AT)8	16	2774	2789	GAGAATCTGGTTCTGGTGGC
scaffold320117_15.:	1	p2	(AT)6	12	90	101	CACATCAACACACCCACACA
scaffold320125_14.8	1	p2	(AT)6	12	1553	1564	GACCGATCGAAACACACTGA
scaffold320396_15.9	1	p2	(AT)8	16	1511	1526	CGGTTTCCACAACAATAGCC
scaffold320407_12.4	1	p2	(TC)6	12	92	103	ATGAGTCGTAGTTGACGCCC
scaffold320614_12.8	1	p2	(AG)7	14	230	243	GGAAATTGATGGATTTGGGA
scaffold320646_14.:	1	p2	(GT)7	14	262	275	GGAATGCGTCGAGTACCACT
scaffold320698_14.3	1	p2	(AG)6	12	71	82	CCCATGTTGTAATTAATGTAGGGA
scaffold320978_15.5	1	p2	(AG)6	12	983	994	TAGTTGGAGCAGGGGAAGAA
scaffold321005_15.4	1	p2	(AC)6	12	125	136	CCCCTCCTAACTTTGCTGC
scaffold321194_11.6	1	p2	(CA)7	14	183	196	TGACCCACCATTGTCAAGAA
scaffold321259_15.5	1	p2	(GA)6	12	697	708	GTCCCACAAAAGCTCAGAGG
scaffold321417_13.4	1	p2	(TA)10	20	158	177	CTCAGCATTTGGGAAGGGTA
scaffold321803_12.6	1	p2	(TA)10	20	744	763	AACAATACGGAAGCAATTTTATCA
scaffold321960_10.(	1	p2	(GA)8	16	153	168	TTGATGTCCCTTCTCCTTGC
scaffold321999_15.7	1	p2	(GA)7	14	1051	1064	TGCCTTCCATGTTTGTTTCA
scaffold322026_17.(	1	p2	(CA)6	12	242	253	TCTGTGCCGCATTTATGATT
scaffold322075_10.5	1	p2	(TA)8	16	82	97	TTTTCGAGTTATGAAGTTAGCACC
scaffold322082_13.6	1	p2	(GA)6	12	151	162	ACCCAAGGTCAGTGGAACAA
scaffold322106_15.4	1	p2	(TA)8	16	517	532	ACATAGCAATTTTCGGGGTG
scaffold322170_17.9	1	p2	(TA)8	16	1366	1381	TGGTTTTCTCCTTCTCAACGA
scaffold322395_14.8	1	p2	(AT)6	12	610	621	GCTACCCTTTTTGACCTACGG
scaffold322408_15.6	1	p2	(TA)10	20	1470	1489	CTTCAATGGACCAGCACCTT
scaffold322936_17.3	1	p2	(AT)8	16	71	86	CCTGCACGAAGAAAGGATTC
scaffold323072_15.2	1	p2	(TC)6	12	48	59	TTGGTTCACACAACCACTCC
scaffold323272_16.:	1	p2	(AT)9	18	151	168	TGAATGATTTAATAACCTATGGTGC
scaffold323277_14.4	1	p2	(AT)6	12	437	448	CGTCGTGCTCAAACTTCAAA
scaffold323464_14.9	1	p2	(CT)6	12	80	91	CAATGTGAAATGGGCAAGAG
scaffold323820_15.3	1	p2	(CT)6	12	83	94	GTTCGCACAACCTCTCCACT
scaffold323925_15.6	1	p2	(GA)7	14	803	816	TGCAAGAAAATCCAGCTTCA
scaffold324006_11.5	1	p2	(CT)7	14	355	368	CAATTCCAAGCTACAACGCA
scaffold324010_16.2	1	p2	(AC)8	16	1231	1246	AACAAAACCACGAATCCAGC
scaffold324194_16.7	1	p2	(TA)6	12	468	479	TAGCGTGATTTGTGGACGAG
scaffold324215_17.9	1	p2	(CT)6	12	53	64	CACTCACTCTCACTAGCCGC
scaffold324218_16.(	1	p2	(CT)6	12	285	296	TGAGCAAAGCATTTTGGATG
scaffold324418_16.(	1	p2	(TA)7	14	86	99	CCATTTGCAATACTAAGGCTTTC

scaffold324446_16.(	1	p2	(GT)9	18	855	872	GAGCAATAGCAGGTTCGGAG
scaffold324788_14.7	1	p2	(TA)8	16	232	247	CAAGCATATTGGCAAGAAACA
scaffold324951_10.5	1	p2	(GA)6	12	116	127	AGTTTGGGGCTTGATTGATG
scaffold325007_17.6	1	p2	(TC)6	12	291	302	CAAGCAAACCACTCACTCTCC
scaffold325142_12.8	1	p2	(TA)11	22	163	184	CTGAGATTTCACATGGCAACA
scaffold325269_11.5	1	p2	(CT)6	12	194	205	GAAGCAAACACGTCACTCCA
scaffold325313_16.8	1	p2	(CT)6	12	1948	1959	AATTCATTTCCCCTCGACCT
scaffold325338_11.2	1	p2	(AT)9	18	377	394	GGTCCATTGGTCTTTTGTATTGA
scaffold325517_14.3	1	p2	(AT)6	12	133	144	TGCATCACGTCTCCTCAATC
scaffold325695_13.2	1	p2	(CA)6	12	432	443	CTCACGCTCTCTCACTCACG
scaffold325869_12.8	1	p2	(AT)7	14	372	385	GACCACCCAACCATCAAATC
scaffold326122_14.5	1	p2	(TA)10	20	1059	1078	CAATCCCAGCCATTCATTTT
scaffold326194_10.7	1	p2	(TC)6	12	33	44	TCTCATTCTCTTCTCTATCTCTCTCG
scaffold326712_11.(	1	p2	(GA)6	12	944	955	TAACTGCCATTTCCCGATTC
scaffold326718_12.2	1	p2	(TA)10	20	349	368	TACCTTTTTGCCCTCGAATG
scaffold326912_13.2	1	p2	(TG)7	14	340	353	AATTAATGTGGCATGGGGAA
scaffold326949_14.6	1	p2	(TA)6	12	76	87	ACAGTAGGGTGGAAAACAAAAA
scaffold327064_11.(	1	p2	(AT)6	12	77	88	TGCATTTTCTTCTTTCTTTTTCA
scaffold327099_10.7	1	p2	(GA)8	16	68	83	TGGAGAAGAAAGAATGAGGAATG
scaffold327145_12.8	1	p2	(AT)6	12	62	73	CAGATGGAGGGTGTGTGAAA
scaffold327246_14.6	1	p2	(AT)6	12	643	654	CGTTTAGCCGCAAGTTTCTC
scaffold327419_17.8	1	p2	(TA)7	14	263	276	CGAATTTTGTGTGGACCGTA
scaffold327547_12.(	1	p2	(AG)7	14	158	171	AAGGCCAAGCAAGGAAAAAT
scaffold328349_13.9	1	p2	(CT)6	12	119	130	TGGCTTCTCTTGCTCTCTCA
scaffold328452_12.2	1	p2	(CT)6	12	687	698	GCCGAGTCTTGTTGATGATG
scaffold328697_15.7	1	p2	(TA)6	12	776	787	GAGTCAAGGGCGGATACAAG
scaffold328755_11.(	1	p2	(TA)6	12	289	300	TTGTAATGTGGCATGCGTTT
scaffold328967_18.5	1	p2	(TA)9	18	358	375	AGGTGGCACGCATCTGTAAC
scaffold329265_11.7	1	p2	(TA)6	12	344	355	TTGATGTTTAAAGTCGTCGGC
scaffold329603_16.5	1	p2	(AG)6	12	867	878	TGTTCATGCTGGCTGATAGG
scaffold329862_16.2	1	p2	(GA)6	12	901	912	TGCAAGCCTATGGTGGGTAT
scaffold330369_12.6	1	p2	(AT)9	18	136	153	GCCGAGAGAGATGAGGAGTG
scaffold330708_16.5	1	p2	(GA)7	14	3123	3136	GGCCCTGATCAGAATCAAAA
scaffold330732_10.1	1	p2	(AT)6	12	162	173	GGAAAAGGGAAGAACAAGGC
scaffold330861_17.5	1	p2	(GA)7	14	2640	2653	CAGAGGGGACCTTCCTTTTC
scaffold331413_16.(	1	p2	(GA)7	14	2199	2212	TGCAAAAGCTTAGGTGGACA
scaffold331432_14.7	1	p2	(GA)8	16	1168	1183	TGATTGCTTTGGCCCTACTC
scaffold331777_12.3	1	p2	(TA)6	12	73	84	CTGACGTCTTGTGGGAGTGA
scaffold332162_11.9	1	p2	(AT)8	16	470	485	TTTCGTCGCTCTCAACTTCA
scaffold332332_11.3	1	p2	(GA)6	12	404	415	ACCCCAATCTCCGTTCTTCT
scaffold332545_16.5	1	p2	(CT)7	14	9125	9138	TCACCTTTGCCTTGGGTATC
scaffold333233_11.9	1	p2	(TA)9	18	207	224	TCGGAATTCACTTCTTTGCTG
scaffold333356_16.3	1	p2	(GA)6	12	499	510	TGTTGGACTGTGTTGGGCTA
scaffold333858_10.5	1	p2	(AG)7	14	69	82	TTGTGGGACTTTGAAAGAGGA
scaffold333865_16.8	1	p2	(TA)9	18	1027	1044	CAACGGTATGCTCGGATTTT
scaffold333989_16.(	1	p2	(GA)6	12	79	90	GGCTCAAGCTTGCTGTTTTC
scaffold334347_15.8	1	p2	(AT)6	12	216	227	GGTTGTAATGAGGCTTTCGG
scaffold334580_16.4	1	p2	(GA)6	12	615	626	ATCATCCAATTCCAATCCCA
scaffold334757_15.5	1	p2	(CA)7	14	279	292	GGTGTGGCTCTGTGGATTCT
scaffold334891_14.9	1	p2	(TC)6	12	969	980	TGTGAGATTGTGTGTGGCAA
scaffold334969_13.9	1	p2	(TA)7	14	469	482	ATCGGCCGAGATTTGTTATG
scaffold335149_11.9	1	p2	(GA)7	14	525	538	TGAACATCCATGGTCCAAAA
scaffold335722_16.5	1	p2	(TC)7	14	1205	1218	CCCACACCTCTTTGCTTTGT
scaffold335902_13.9	1	p2	(AT)7	14	505	518	AACCCGTTTGCTGCATAGTT
scaffold336129_13.(	1	p2	(AT)9	18	300	317	TCGAAGAGGAAAATAAAATAATCCC
scaffold336159_15.1	1	p2	(TG)9	18	2694	2711	TTTTGGAGAGGTTGGAGGTG
scaffold336169_10.1	1	p2	(TA)8	16	206	221	CGTCTAATCCACCGCCTAAA
scaffold336306_11.2	1	p2	(AT)6	12	329	340	TCTTCGATGTTGCTGGATTG

scaffold336327_17.3	1	p2	(AT)6	12	477	488	AATGGCTGCAATTATCCCTG
scaffold336438_11.2	1	p2	(AT)7	14	223	236	CATGATCAAATCTTGGTGCG
scaffold336460_12.4	1	p2	(CT)7	14	68	81	CAAATGCCCAAGAGGGTTTA
scaffold336476_16.6	1	p2	(AG)6	12	300	311	GGCTGTACCATAAAAGCCCA
scaffold336568_13.4	1	p2	(TC)6	12	253	264	CCGAATTTTCCACTCCAGAA
scaffold336742_16.0	1	p2	(AT)7	14	2427	2440	TTGCCCAAACAAATGAAAAA
scaffold336786_19.9	1	p2	(AT)6	12	159	170	CGATCAATAAAGCTGCACACA
scaffold336870_13.0	1	p2	(CT)6	12	296	307	CGCACTACTAGGGTTTGGGA
scaffold337112_19.2	1	p2	(GA)6	12	150	161	CATTCTCAAGCCACTCCCTC
scaffold337344_17.7	1	p2	(TC)8	16	104	119	TGAAAAAGGACTCTTGGGCA
scaffold337506_16.5	1	p2	(TC)7	14	673	686	AGAGGTAGCCATTTACGCGA
scaffold337681_11.8	1	p2	(TC)6	12	89	100	TAGGCAATGGAATACCTCCG
scaffold337783_11.3	1	p2	(TA)8	16	320	335	CACCACCACCTCACTGTCAC
scaffold337813_10.9	1	p2	(TA)10	20	563	582	CAAATTCTTGATTCGCCACC
scaffold338083_14.2	1	p2	(CA)6	12	1134	1145	GGTTGCTCTCCTTTGAGCAC
scaffold338132_16.6	1	p2	(AG)8	16	2073	2088	CCTGGCTCGTTAGCTGATCT
scaffold338234_15.0	1	p2	(AC)6	12	1390	1401	TTCCAAAGCAACGTAACATGA
scaffold338395_11.5	1	p2	(AG)6	12	615	626	AGGTCGTGGTGAAAAAGGGT
scaffold338474_11.3	1	p2	(TA)6	12	109	120	TCTGCATGCTCTATGCAAGAAT
scaffold338497_14.3	1	p2	(CT)6	12	47	58	CAATGATTGTCAAAATTAGAGTTCAA
scaffold338688_16.7	1	p2	(GT)7	14	100	113	TAGGATACCCTCCCAAACCC
scaffold338716_14.2	1	p2	(CT)6	12	418	429	AAAAGGGGCATTTTGCACTA
scaffold338858_13.7	1	p2	(TA)8	16	255	270	TGCACAGCATATGCCTTCAT
scaffold338959_16.7	1	p2	(CT)11	22	436	457	GCTCCTCCTTGGTACTCACAA
scaffold339016_18.1	1	p2	(TC)6	12	218	229	CGAGATGTCTTCAGGGCATT
scaffold339153_13.8	1	p2	(CA)6	12	208	219	CTCCTTCTATCTCCCCTGCC
scaffold339183_15.3	1	p2	(TA)6	12	397	408	ACGCTCTTGAAATATGCGCT
scaffold339911_18.9	1	p2	(CT)7	14	83	96	CTCTCTCACCTCCCCAAACC
scaffold339963_15.9	1	p2	(TC)6	12	202	213	AAAGGTTAGTGGGCTGAGGAA
scaffold340103_13.5	1	p2	(TA)7	14	319	332	TGAGCTAGCTTTTGTGGTGG
scaffold340139_10.6	1	p2	(TC)6	12	448	459	CACGCACAAGTACATCTCGG
scaffold340231_19.1	1	p2	(TA)7	14	689	702	GCCTTGGTCATGCCATTTAT
scaffold340387_11.0	1	p2	(TA)6	12	211	222	CGACGGTTGAGGAAGAAACA
scaffold340409_12.7	1	p2	(TA)7	14	145	158	CACCGTCCAATAAACCGAAG
scaffold340809_10.4	1	p2	(TA)7	14	254	267	GAAAATCAAAGGCCCACAAA
scaffold340813_14.2	1	p2	(CT)6	12	345	356	AGAAGGCAATCTATCCCGTG
scaffold340821_17.1	1	p2	(TC)9	18	507	524	TCGTCGTTGTTACTGGCCTA
scaffold340852_12.5	1	p2	(TA)9	18	861	878	TCTGATAAGGTTCGAATTCACG
scaffold340956_16.9	1	p2	(TA)14	28	1108	1135	ATGGGCTTCGAGTTTGGATA
scaffold341328_13.9	1	p2	(AG)6	12	424	435	GTAGCGGTGGTTGACTGGTT
scaffold341543_13.2	1	p2	(AG)8	16	649	664	TGGTAGTGTTGAGTTGGCGA
scaffold341822_16.2	1	p2	(AT)10	20	584	603	TGCAGATTCAGGATGAGACG
scaffold342015_13.6	1	p2	(TA)7	14	193	206	CAAGATTCGAAATCACATTCAAA
scaffold342495_16.0	1	p2	(AG)8	16	110	125	GTCCACGGTGGTGCTAGAGT
scaffold343175_14.6	1	p2	(TC)7	14	1140	1153	ATTCTTCTTCAACCTCCGCC
scaffold343896_16.3	1	p2	(CT)8	16	379	394	TGCCCTTAAGTTAGGACACTAAA
scaffold344024_11.5	1	p2	(TA)6	12	298	309	TTTTAGGGGTGCGAAAGTTG
scaffold344062_14.5	1	p2	(CT)6	12	46	57	TCATCTTGGCATGGTCAAAG
scaffold344160_15.0	1	p2	(CT)6	12	73	84	CCTAGTTCCTACCGCGTGTC
scaffold344442_13.9	1	p2	(GA)7	14	120	133	ACTTGAGCGTAACCACGTCC
scaffold344486_12.2	1	p2	(TA)6	12	139	150	CTGCTTAAAGCCCAGTCCAG
scaffold345111_16.4	1	p2	(TA)6	12	265	276	AGGGCATTATTTTAACCCCG
scaffold345129_10.6	1	p2	(TC)6	12	409	420	ATTGAATCTACGTCCACGGG
scaffold345147_18.3	1	p2	(GA)6	12	38	49	GAGTTTAGAGAGAGGAAATGGTATTC
scaffold345172_16.0	1	p2	(TC)6	12	2068	2079	TCGAAATCCCATCTACCCAC
scaffold345277_15.4	1	p2	(CT)6	12	2308	2319	TGACAACCATCACCTCGAAA
scaffold345489_12.7	1	p2	(TC)6	12	53	64	TCCTGCCATCTCTCAATGTG
scaffold345774_15.4	1	p2	(GT)6	12	653	664	GACACCAAAACGGAGGAAAA

scaffold345781_15.	1	p2	(GA)7	14	351	364	GCCAACACTCCAAAGAAATGA
scaffold345835_17.	1	p2	(CT)7	14	76	89	TTCCTCCTCAATATTTTCAACACA
scaffold345880_14.	1	p2	(GA)6	12	224	235	CAAGCCATGAAACAACACGA
scaffold346132_10.	1	p2	(CT)8	16	308	323	GCGTGTGGAAGAGGACAAA
scaffold346164_16.	1	p2	(GA)6	12	2946	2957	AGCTTCAGTGTGTGTGCGAG
scaffold346510_10.	1	p2	(TA)7	14	326	339	TGCAATGCATGGAGGAAATA
scaffold346752_16.	1	p2	(TA)6	12	98	109	TCCATTCCGAAAATCAAAGG
scaffold346987_10.	1	p2	(AG)6	12	312	323	TCTGGAAATGGGGAAAACAG
scaffold347086_12.	1	p2	(TA)6	12	227	238	ATCTTGACCGCTGATTTTGG
scaffold347321_15.	1	p2	(AT)6	12	108	119	TCTGAAACCCTTCCCATCTG
scaffold347380_18.	1	p2	(CT)7	14	443	456	TCGGAAGACACCTATTTGCC
scaffold347437_14.	1	p2	(AC)6	12	663	674	CCACCATCTCTCATCCCTCT
scaffold347530_13.	1	p2	(AT)11	22	1631	1652	GAACGCAAATGCGATATGAA
scaffold347858_15.	1	p2	(AT)6	12	1407	1418	AAACCTTACTGTCACGCGCT
scaffold348045_17.	1	p2	(CT)6	12	635	646	TATCCCCGCCAACTTAACTG
scaffold348283_11.	1	p2	(AT)9	18	29	46	TCTATATACAACTCGCATACCCAAA
scaffold348289_16.	1	p2	(TA)6	12	349	360	CATGATTTCAGAATCGTGCG
scaffold348322_12.	1	p2	(CT)9	18	528	545	CTGAGCAGACCAGTCCAACA
scaffold348369_10.	1	p2	(AG)7	14	272	285	CTCGTCCCTTTGTGCTTGTT
scaffold348410_13.	1	p2	(AT)7	14	195	208	AAAGGTATGGGGAAAATGCC
scaffold348498_12.	1	p2	(AG)6	12	238	249	GCCCCACGTATGATTGTCTT
scaffold348513_12.	1	p2	(AG)7	14	51	64	TTTCTCATGTTGTAATTAATGTAGGGA
scaffold348556_17.	1	p2	(GA)6	12	119	130	GCCACTTTCCTTAAACCAAAA
scaffold348569_16.	1	p2	(CT)6	12	57	68	TGTCCTCACTCGTTTCCTCT
scaffold348571_13.	1	p2	(CT)6	12	743	754	AGATGTGAACCAAGAACCCG
scaffold348599_10.	1	p2	(CA)7	14	51	64	CTAACCTTGGGCTCTGATGC
scaffold348650_14.	1	p2	(GA)6	12	147	158	GCATCAGAGCCCAAGGTTAG
scaffold348911_14.	1	p2	(TA)8	16	407	422	TGAGGAATCGAACAAGGACA
scaffold349088_16.	1	p2	(CA)6	12	4609	4620	GGAATTTGATCCCCACACAC
scaffold349526_18.	1	p2	(TC)6	12	252	263	CATCACCTCCATTTTCCCAC
scaffold349549_14.	1	p2	(TG)7	14	448	461	ATGCCAAAGGGGAAACAAAT
scaffold349946_10.	1	p2	(AT)11	22	173	194	ATAAACTGTTACTCCTGGGTGC
scaffold350008_15.	1	p2	(GA)6	12	1507	1518	TCGAACCATTCACATCCTGA
scaffold350079_11.	1	p2	(AT)6	12	279	290	TGTGCCATAAAAATGGTTCAT
scaffold350124_10.	1	p2	(TC)6	12	274	285	GGGGTGGTAACCGATGTATG
scaffold350195_12.	1	p2	(TA)11	22	376	397	AGAGTTTTGCGTGCATGTTG
scaffold350315_10.	1	p2	(TA)10	20	87	106	TGTTGCGCGTGCTTTAAATA
scaffold350387_14.	1	p2	(AT)6	12	1540	1551	TATTATGATTCCGGGTTCCG
scaffold350624_16.	1	p2	(GA)7	14	283	296	TGTGCTTCCACCAAATCAGA
scaffold351156_12.	1	p2	(TC)6	12	72	83	GCTCTTTTGATCGATCTGCC
scaffold351651_13.	1	p2	(TA)13	26	471	496	CACGCATGAATTCTGTTGGA
scaffold351933_13.	1	p2	(CT)6	12	1097	1108	CCCACCCCAGATCATAGAAA
scaffold351935_15.	1	p2	(TC)7	14	591	604	TTGAGCATTGAGGTTGCTTG
scaffold352010_16.	1	p2	(TA)7	14	133	146	GAAGGAGGAGAAGTTTGGGG
scaffold352266_11.	1	p2	(AG)7	14	27	40	AGAATGAGGGAGGGAGGAGA
scaffold352290_13.	1	p2	(AT)6	12	598	609	GCCCCTGATCATAACTCGAA
scaffold352456_14.	1	p2	(AG)6	12	401	412	GGCATGTGTTGTGTGGACTC
scaffold352567_10.	1	p2	(TA)8	16	240	255	TCACAAATCGATGGTCTGGA
scaffold352700_10.	1	p2	(AG)6	12	199	210	CGAGTGCCATTCTGACAGTG
scaffold352946_10.	1	p2	(GA)6	12	41	52	GAACCAACTCTGTGAGAACCAA
scaffold353094_11.	1	p2	(AG)6	12	104	115	TCATCGTGTTCTTCGCTCAC
scaffold353137_18.	1	p2	(CT)6	12	406	417	CTTCCAACTTCGACAGCTCC
scaffold353197_15.	1	p2	(AG)6	12	462	473	CGGAACGCTTAATGCAAAAC
scaffold353403_12.	1	p2	(CT)6	12	278	289	GCACACCTCGTCTCTCACAA
scaffold353862_14.	1	p2	(CT)6	12	231	242	TCCACGGAGAGGTAAAAAGC
scaffold354188_15.	1	p2	(CT)9	18	218	235	GGGCTTGTCGCATTCATATT
scaffold354245_17.	1	p2	(AC)9	18	967	984	GCATTTGCCTTTAGGAGCTG
scaffold354354_15.	1	p2	(TA)6	12	161	172	AAATGCGCAATTTTAAACCC

scaffold354359_14.7	1	p2	(AT)9	18	61	78	CCGAAATCTGTAAATCCTAGCC
scaffold354581_13.3	1	p2	(AG)7	14	93	106	CATTAAATGCGCCTACACTTT
scaffold354707_15.6	1	p2	(CA)6	12	2912	2923	GATAACCGTGTGGCATTTGA
scaffold355276_12.9	1	p2	(AT)8	16	1433	1448	TGCCAATGATCACCAAACC
scaffold355557_12.8	1	p2	(TA)6	12	73	84	TTCAAATGCACGTGTTGGAT
scaffold355899_11.8	1	p2	(TC)6	12	67	78	GCTGATGCACCGAGCTAGTA
scaffold356064_15.8	1	p2	(AC)6	12	431	442	GTGCTGAAAGCCCCAATTTA
scaffold356309_17.1	1	p2	(CA)6	12	4376	4387	GAGTTCTAGCCTCAAGCAACG
scaffold356606_14.4	1	p2	(CA)7	14	332	345	ATTCATTCTGCCACCTCTGG
scaffold356760_15.3	1	p2	(AT)9	18	277	294	CATTCAAGGGGCATTTTTGT
scaffold356797_18.0	1	p2	(CA)6	12	297	308	CTGTCACCATCACCAACCAC
scaffold356813_11.5	1	p2	(AC)6	12	126	137	TCTGTTGGCAAATAGGCACA
scaffold356981_10.1	1	p2	(TC)6	12	305	316	TTTCTCTCGATGCACTCTCG
scaffold357004_14.6	1	p2	(TA)8	16	48	63	TTCGATGATAACTATGGGTTTGT
scaffold357095_14.7	1	p2	(GA)7	14	442	455	TAGGCAAAAACTTCCGTCCA
scaffold357116_11.3	1	p2	(AT)8	16	272	287	CAATGAAAATGCCACGTCTG
scaffold357152_13.5	1	p2	(AG)6	12	164	175	TAGTTGACGGTGGTGGTTCA
scaffold357274_13.7	1	p2	(AT)8	16	423	438	CCACATTTTTCCCGACTGTT
scaffold357307_13.2	1	p2	(CT)7	14	110	123	CCCACCAACTCTCGTCACTT
scaffold357980_10.2	1	p2	(TC)7	14	245	258	TTTTAGGGCATTCAACTGGG
scaffold358049_11.1	1	p2	(TC)6	12	155	166	AGGGCTGAGGAAAACCTGAT
scaffold358318_12.0	1	p2	(TG)6	12	193	204	CGAGTGAGAGAGTGAGTGCG
scaffold358450_10.2	1	p2	(AT)6	12	264	275	TCACACAGCACCTGATCACA
scaffold358681_12.5	1	p2	(AT)11	22	865	886	TACGATCCGAGTGCCTCTTT
scaffold358892_13.7	1	p2	(AT)8	16	659	674	TCGAATACCCTAATAACCCGAA
scaffold358974_13.5	1	p2	(AT)8	16	1740	1755	AGTTTGATGAGGGCATCCAC
scaffold359031_12.8	1	p2	(CT)6	12	67	78	CTCCACAAAGATCTCGCACA
scaffold359042_15.2	1	p2	(TA)8	16	422	437	TTGTTTTGTCCACACTTGGC
scaffold359242_16.1	1	p2	(AT)6	12	165	176	CATGCAGACATGGGAGTTTC
scaffold359290_14.1	1	p2	(TA)13	26	621	646	GTGGACGTCCCAGATTGTTT
scaffold359358_10.9	1	p2	(GA)8	16	180	195	CGCGAACTTAGATGGGCTAC
scaffold359487_13.6	1	p2	(TA)6	12	1727	1738	CGAAAACTCAATTTAAAAGGCTTG
scaffold359575_13.6	1	p2	(TA)10	20	553	572	CACTTGTCAAAATCTCCTTAATTTG
scaffold359726_13.8	1	p2	(CT)6	12	536	547	GCTCATCGTCATTCTCCACC
scaffold359728_16.3	1	p2	(CT)8	16	1060	1075	GAGGAGAGGGGGATCGATAG
scaffold359752_15.4	1	p2	(AT)7	14	685	698	CCTGCACCAAATTTAGCCAT
scaffold359771_12.8	1	p2	(TC)6	12	56	67	CACGCTATCTCGTTGCTCTCT
scaffold359834_14.3	1	p2	(TC)8	16	3864	3879	CCTTTTGCTTCAACCACCAT
scaffold360089_13.8	1	p2	(AT)9	18	1929	1946	TTTTTGCGGTGTGCATTTAG
scaffold360400_13.6	1	p2	(CT)11	22	1047	1068	TAGTTGCAGGCCCTCTTGAT
scaffold360547_12.3	1	p2	(TA)8	16	786	801	TCATGCACAAAATAAAAAGGGA
scaffold360592_12.0	1	p2	(TA)9	18	42	59	TCTCACACACGTTTACACCCA
scaffold360611_14.7	1	p2	(TA)7	14	1392	1405	GCCATCTTCTTTCCCCATTT
scaffold360654_16.2	1	p2	(TC)6	12	489	500	CCCTCTGTTCTCTCTCACCG
scaffold360688_14.8	1	p2	(AG)6	12	1589	1600	ATGTAGGTGCCTGTGCCTCT
scaffold360789_10.5	1	p2	(TC)6	12	385	396	TAGCCGTCCTCTCCATCACT
scaffold360894_16.0	1	p2	(TA)8	16	479	494	GCGATTGACACGAAATGAAA
scaffold361048_14.8	1	p2	(CA)8	16	447	462	TCGGATCGGATACCCAAATA
scaffold361190_11.8	1	p2	(AT)10	20	350	369	CCACGGAAGGAACTGACAAT
scaffold361588_12.1	1	p2	(AT)7	14	218	231	GGTCTGACAGGTTCAGAGGC
scaffold361716_11.7	1	p2	(GT)7	14	98	111	TCTTTCAGGTCCAGGTGTCC
scaffold361740_14.0	1	p2	(AT)6	12	202	213	CCTTCTCCATCACCACCTTG
scaffold361995_15.5	1	p2	(AC)6	12	944	955	CTCTTGTCCACCCACCACTT
scaffold362057_17.3	1	p2	(TA)8	16	59	74	CCGGTCTAAACCCGGAATA
scaffold362379_16.5	1	p2	(TC)9	18	791	808	AACACAAACGCTAAACCCGT
scaffold362560_14.1	1	p2	(TG)6	12	1280	1291	TGTAGTCATCCATGGGGGTT
scaffold362651_17.9	1	p2	(CT)6	12	313	324	TTGAGATCCATTCCCTCAAA
scaffold363042_12.0	1	p2	(TG)6	12	126	137	GGTTATCACCTGATTTTCTCCC

scaffold363404_15.1	1	p2	(TA)11	22	969	990	GCCGAAGATTTACTCCTAATTAACAC
scaffold363441_11.7	1	p2	(TA)9	18	177	194	GGCTAGATGCAAGCCTTTGA
scaffold363486_13.8	1	p2	(AT)6	12	65	76	TCAAATCTTCGAGTACAGAAATGG
scaffold363619_12.9	1	p2	(TG)7	14	854	867	GCAGCTGATGCACATATTCC
scaffold363720_13.2	1	p2	(TA)7	14	212	225	CTTCACTTGAATTGAGGGGG
scaffold363724_10.8	1	p2	(CT)7	14	238	251	TTATTTTCTTGGGTCGCTGG
scaffold363943_15.8	1	p2	(TA)7	14	234	247	ATGCGCGTTTTTAGGGTTTA
scaffold364125_17.9	1	p2	(TA)6	12	148	159	TCCATTCCGAAAATCAAAGG
scaffold364236_13.4	1	p2	(AT)6	12	56	67	TTTTGTGAAACAATAATGACTTTGA
scaffold364374_17.8	1	p2	(TA)6	12	774	785	TGGGCGTTTATTATTCCAGC
scaffold364596_15.1	1	p2	(GA)7	14	452	465	CCACTCTCTCACACGCAGAA
scaffold364664_12.1	1	p2	(TA)10	20	555	574	GCAAAAGGCAGCCTAAAGAG
scaffold364797_14.6	1	p2	(TG)6	12	2113	2124	TTATGGGAAGAGGGGGAGAC
scaffold364819_12.6	1	p2	(TA)8	16	897	912	CAATGCACCAGATTTGTTCCT
scaffold364875_19.8	1	p2	(TC)7	14	993	1006	AAAGGTCACGCCCTACAAAA
scaffold365151_15.4	1	p2	(TC)6	12	270	281	TTGGTGCATGCCTTTTGTTA
scaffold365250_13.7	1	p2	(TA)6	12	2312	2323	TGAAAAACAAGCGAGCAACA
scaffold365268_12.7	1	p2	(TA)8	16	502	517	GGCGAATGCTATGCATTTTT
scaffold365275_17.3	1	p2	(TG)6	12	238	249	CACAACACACATCCTTCATGC
scaffold365457_14.8	1	p2	(TA)10	20	1896	1915	AGTTGATGGTGTTCCCCTTG
scaffold365480_16.0	1	p2	(CT)6	12	2090	2101	TGTGCATTCCACATTTCCAC
scaffold365654_13.4	1	p2	(TA)7	14	44	57	TCCCAGGTGTGAATAGCATTT
scaffold365696_13.6	1	p2	(TA)11	22	497	518	GGAAATGATCACAGGCCATC
scaffold365997_14.0	1	p2	(TA)6	12	26	37	AGCGGTTATTACCGGATCG
scaffold366151_13.5	1	p2	(AT)10	20	859	878	AGTGGTTAGCACATGATCGC
scaffold366341_10.6	1	p2	(AG)6	12	102	113	GTCGGCAGTGAGCTTCTAGC
scaffold366548_13.9	1	p2	(AG)6	12	678	689	TGGGCAAACCAAGATACACA
scaffold366746_13.6	1	p2	(AT)6	12	740	751	TCCTTAAACATAACGAGATCAAAAA
scaffold367089_10.4	1	p2	(TA)7	14	300	313	TTTTTACCAATTTGCCCTCG
scaffold367132_16.9	1	p2	(CT)6	12	80	91	CTCTTTCTCTCTCCGCCCTT
scaffold367201_15.1	1	p2	(TA)9	18	502	519	CAAATCCACCAGAAACCCAC
scaffold367394_14.3	1	p2	(CT)8	16	222	237	ATCTCTTTTCCCCCACGAGT
scaffold367424_11.8	1	p2	(AT)9	18	422	439	ATTACGGCCACGCTAAGTTG
scaffold367446_13.1	1	p2	(TC)7	14	453	466	ATCCCCTCTCATTCAACACC
scaffold367502_14.4	1	p2	(TA)6	12	137	148	ACCTACTGCCGTGTTCGATT
scaffold367612_12.1	1	p2	(TA)9	18	73	90	TTCATGAATGGATGTGTTTTGTT
scaffold367760_13.0	1	p2	(TA)11	22	38	59	TTTAGTGTTTTGACATGATTACTCG
scaffold367767_17.0	1	p2	(TA)7	14	118	131	ATTTGCTTGATCCAACGGTC
scaffold367849_10.8	1	p2	(TA)7	14	706	719	TCTACCCTTCATTGTCATTCTCA
scaffold367932_16.8	1	p2	(CT)6	12	1914	1925	GTGAGATTCGATGCCCATTC
scaffold367983_11.6	1	p2	(TA)10	20	84	103	TGGCCGTGAATGTATGAGAG
scaffold368078_13.5	1	p2	(TC)6	12	145	156	TGAAGAAAATAGGACTAATGAGGTG
scaffold368148_13.8	1	p2	(TA)11	22	299	320	ATCGTGAGAAGCGTCGATCT
scaffold368239_13.1	1	p2	(CA)11	22	599	620	GCTTCTCGTTTTCTTGTCGG
scaffold368277_10.3	1	p2	(AT)9	18	207	224	TTTTCGGATCGGTAGAAGGA
scaffold368315_11.9	1	p2	(AG)9	18	619	636	CCAAATGCATGCAGCTTCTA
scaffold368360_14.4	1	p2	(AG)7	14	258	271	CCTGTAGACGGCCAAATGTT
scaffold368405_10.8	1	p2	(TC)6	12	462	473	TGGAAGCCATGCAAAATAAA
scaffold368503_17.4	1	p2	(CT)7	14	1436	1449	CTCCATCGAACATCTCACGA
scaffold368639_13.6	1	p2	(TG)7	14	2276	2289	TTTCTTTTGTGACGCAATCG
scaffold368710_17.3	1	p2	(TA)11	22	4860	4881	TGGCGAAGCAGAAAGAAACT
scaffold368855_16.7	1	p2	(CT)6	12	31	42	CAAATGGCCAAGAGAGTTTACA
scaffold368911_10.0	1	p2	(TA)9	18	515	532	TCAAAGATGGAGGTTGGAGG
scaffold368992_16.2	1	p2	(TA)6	12	6490	6501	GTCTCACGCGGGTAAGTGAT
scaffold369003_13.6	1	p2	(AT)6	12	62	73	CGTTCGATTTGCCGTTTTAT
scaffold369209_16.0	1	p2	(TC)8	16	1173	1188	GCAAGAAAAATAAAGGGCAGC
scaffold369467_14.8	1	p2	(GA)7	14	271	284	AGAAATCAGACGGGCAGTTG
scaffold369547_13.6	1	p2	(TA)7	14	1280	1293	TTAGGGTTTAAAATCGCGCA

scaffold369554_12.5	1	p2	(TC)7	14	74	87	GAGGACACTTGGACCTGAAAA
scaffold369608_10.9	1	p2	(GA)8	16	618	633	AAGTCACCGCCGTTCATATC
scaffold369711_17.7	1	p2	(AT)8	16	511	526	CCGTGATTTAAGGAGTCGGT
scaffold370064_14.9	1	p2	(AG)6	12	48	59	AGCTGTTAGGGTTGTATAGTGAGTAG
scaffold370280_12.2	1	p2	(TC)6	12	193	204	AGTGATGTGGTGTTGGAACATT
scaffold370431_14.4	1	p2	(TA)8	16	2578	2593	CACCAAGTCCAGCCTTCTTC
scaffold370466_18.7	1	p2	(CT)6	12	483	494	CACGTGGAGGATCGTTTTCT
scaffold370601_15.9	1	p2	(TA)10	20	164	183	GGAGTGTCGGTCTGAAGGTC
scaffold370749_17.2	1	p2	(AG)6	12	1436	1447	AGACGGGAGTGCGAGAAGTA
scaffold370930_16.5	1	p2	(AG)11	22	261	282	GCAAGCTTCTCTAACACGGC
scaffold370934_17.1	1	p2	(AT)6	12	401	412	TGCATGTTGCGATTTCATTT
scaffold370940_19.4	1	p2	(TA)8	16	4025	4040	TCTACCTGTTGCTGTGTGCC
scaffold371293_12.3	1	p2	(TA)6	12	117	128	TCAAATGAACATAATCCTGTAATGC
scaffold371622_13.2	1	p2	(TA)11	22	706	727	CCAAATCATTGTATTTCATGTTTCA
scaffold371784_12.1	1	p2	(AT)10	20	640	659	CACCAAATTTCATCATTTTCAA
scaffold371909_17.7	1	p2	(CA)10	20	1795	1814	AGCTTTATGCGTTTGCAGGT
scaffold372201_10.1	1	p2	(GA)6	12	372	383	TCATCAACATGCAACCAACA
scaffold372353_14.5	1	p2	(TA)9	18	1176	1193	ATTATCAACGGCTGCAAAAG
scaffold372390_15.7	1	p2	(GA)7	14	3003	3016	TGCCAGTTGCCATATTTTCA
scaffold372437_15.5	1	p2	(GA)10	20	50	69	TTGAGAGGAAGAAAGAAGATGGA
scaffold372474_14.3	1	p2	(AT)8	16	738	753	TCTAAAGAAAACAAAAGAAGTTCAAA
scaffold372585_14.9	1	p2	(AT)6	12	362	373	TGGAGCAACAAAGATGGAAA
scaffold372698_14.4	1	p2	(TA)7	14	468	481	TCCTAACAAAGAAAGGCGGA
scaffold372885_16.9	1	p2	(TA)6	12	559	570	ATCCCTTTTGCTTTTGCTGA
scaffold372897_16.1	1	p2	(GA)6	12	1642	1653	TGATCCTTGCTCTCTTGCCT
scaffold373129_13.6	1	p2	(CA)6	12	1115	1126	AACGCAAGATGGGAAAAATG
scaffold373236_15.0	1	p2	(AT)7	14	277	290	CAAGCAGGTAAATATCAGTTTTCG
scaffold373293_14.0	1	p2	(TC)6	12	557	568	AGGATCCCAAATCACCAACA
scaffold373316_14.3	1	p2	(AG)8	16	848	863	CCACCAACTCCGGTAAAGAA
scaffold373380_10.0	1	p2	(TA)8	16	293	308	ATAACCGACCACGACACCAT
scaffold373479_10.1	1	p2	(TA)8	16	50	65	GGTTATCACCGGATCACGAC
scaffold373565_10.4	1	p2	(AG)6	12	151	162	GCGGTAGGAGTTGATGATGG
scaffold373581_15.0	1	p2	(AT)6	12	848	859	CGGAATGTGATCGAATAGCA
scaffold373636_13.2	1	p2	(TA)8	16	2765	2780	GCTGCTGTGCGTTGATTTT
scaffold373789_16.9	1	p2	(CT)10	20	1103	1122	TGTCGAATGCAGGGTTGATA
scaffold373818_10.1	1	p2	(AT)8	16	303	318	CAAGAAACGCGCCATGTAT
scaffold373830_13.8	1	p2	(AG)6	12	2335	2346	TCAGGAAGATCATCTGCGG
scaffold373870_15.4	1	p2	(TC)6	12	752	763	AGCAGCAATTTCCGAAAAGA
scaffold373874_11.4	1	p2	(CA)6	12	153	164	AACACTGCACGCATTTTGAG
scaffold374115_11.2	1	p2	(GA)6	12	234	245	GACGGAGAAATCAGACGAGC
scaffold374390_14.9	1	p2	(GA)6	12	369	380	TCCAGTGTCTCCATCTCGTG
scaffold374392_16.8	1	p2	(AT)9	18	1724	1741	CACATTCTTGCTTCTGCTGC
scaffold374575_11.2	1	p2	(TA)9	18	67	84	TTCAACAAAGCAACAAAACATTG
scaffold374684_11.8	1	p2	(AG)6	12	229	240	CTTGAGCATGTGGAGAGCAA
scaffold374704_13.7	1	p2	(AT)9	18	66	83	TTTTGAATGGCACAGATTGG
scaffold374974_16.9	1	p2	(AT)6	12	483	494	AGAGATGCTGGTAGAGGCGA
scaffold375227_12.1	1	p2	(AG)6	12	236	247	CCATAAACACCGTGTCCAAA
scaffold375266_12.0	1	p2	(TG)7	14	570	583	TTTGGCATCAATGCATCCTA
scaffold375467_16.3	1	p2	(CT)6	12	373	384	AAAATGGTGCACAAACCCAT
scaffold375768_17.0	1	p2	(TG)6	12	3495	3506	ACACATCGGCACAGGTACAA
scaffold375824_15.4	1	p2	(AC)6	12	1262	1273	TTTTATTGCAGCTGTCACGC
scaffold376233_17.0	1	p2	(TA)9	18	242	259	GCAGACGAACCAGTAGAGGC
scaffold376275_16.1	1	p2	(AT)7	14	3186	3199	TCCGTTTTGCTAAGCCTTGT
scaffold376691_10.1	1	p2	(AT)6	12	194	205	GTCCTCCGAACTCATGGTGT
scaffold377186_13.6	1	p2	(TA)6	12	65	76	CCTCTCGCATTTCTCTTGCT
scaffold377467_18.8	1	p2	(TA)6	12	318	329	CCAAAGTGCCCTTTTATGCT
scaffold377571_15.5	1	p2	(TC)10	20	151	170	GGTGACCACATGTCGAAAAA
scaffold377819_14.8	1	p2	(TA)8	16	1080	1095	GGATCTACGAGATTTGCTCCC

scaffold377922_15.	1	p2	(AT)9	18	539	556	TGAAGCGTGAAAGTCAATCG
scaffold377960_12.	1	p2	(CT)6	12	1206	1217	TGAAATTATACGAAGCCGGAA
scaffold378185_12.	1	p2	(TC)6	12	154	165	GATGCAGTGGGGTTTTGTTC
scaffold378623_13.	1	p2	(TA)6	12	1138	1149	CAAAATGGGCGGCATAAATA
scaffold378659_12.	1	p2	(TA)8	16	310	325	CACCGAGTACCGAAGGGATA
scaffold378687_14.	1	p2	(AC)6	12	357	368	TTCCCTTCTCATTTCCATGC
scaffold378818_11.	1	p2	(CT)6	12	183	194	AAGGCAACATCTATCGAGGG
scaffold378868_10.	1	p2	(GA)6	12	370	381	AGTACTGCAATTCTGCCCGT
scaffold379478_16.	1	p2	(CT)7	14	315	328	TGGTTGGAGATCTTTGGAGG
scaffold379905_16.	1	p2	(AT)9	18	389	406	TGGGTTTTAGGTGTCATTGCT
scaffold380203_10.	1	p2	(GA)6	12	254	265	CGGTTGGATTGCTGAAAACT
scaffold380377_16.	1	p2	(TC)6	12	121	132	AGCAGCGAAGGAAAAACTTG
scaffold380904_13.	1	p2	(CT)8	16	448	463	TTTCTCTCCGACCACATTCC
scaffold380999_18.	1	p2	(AG)7	14	642	655	GACAGGGAGATGGGGAATTT
scaffold381047_13.	1	p2	(AG)7	14	295	308	TGGAACCATGTTCAATCCCT
scaffold381070_11.	1	p2	(AT)6	12	751	762	GGATAGTGGGCAGAGATGGA
scaffold381136_12.	1	p2	(AT)7	14	634	647	GCCCACCTGATTTTCTTCAA
scaffold381164_11.	1	p2	(TC)9	18	243	260	TATGACTCGCCACCTCTTCC
scaffold381240_11.	1	p2	(TA)6	12	337	348	AAGGGTTGGTATCATTTGAACA
scaffold381274_14.	1	p2	(TC)6	12	160	171	CCGGGTATCGGAAATTAGGT
scaffold381365_16.	1	p2	(TA)10	20	181	200	GTGAAACCCCCAATTGTGTC
scaffold381517_17.	1	p2	(AT)6	12	197	208	TCTGCGTTTAAACAACCCAA
scaffold381663_13.	1	p2	(TA)6	12	167	178	TTTATTTTTCTCAAATGCTATGCAA
scaffold381873_10.	1	p2	(TC)6	12	185	196	AGCACCAATTCTTCCAAACG
scaffold381903_15.	1	p2	(GA)8	16	163	178	TTGGGAGAATTTGTGGGAGA
scaffold381921_12.	1	p2	(TA)9	18	770	787	TAACCCCCGGAGTGAAGAAT
scaffold382176_16.	1	p2	(CT)6	12	215	226	AGCATAACAGCTGGCAAACA
scaffold382189_15.	1	p2	(AT)8	16	329	344	TTAGCTCTGGTTTGGTTCCG
scaffold382261_13.	1	p2	(TA)7	14	486	499	CAGATTACTGTGTGCGGCTG
scaffold382322_18.	1	p2	(AT)6	12	1781	1792	AACTTCCGAATTCCAATTACCA
scaffold382684_11.	1	p2	(AG)6	12	251	262	TGAGCTCATTCCGCATTAAA
scaffold382792_17.	1	p2	(CT)6	12	55	66	TTGCCCCTTCACTCTCATCT
scaffold383021_15.	1	p2	(AT)7	14	542	555	TGGTTGGAGTTGTCAGTTCCT
scaffold383144_12.	1	p2	(GA)6	12	168	179	TTGACCAAGGTGAGAATTTGAA
scaffold383190_12.	1	p2	(TA)8	16	135	150	CTTTTTCCTATGTGTGACAAAGC
scaffold383281_15.	1	p2	(CT)6	12	395	406	AGCATTTACCTGGGCATTTG
scaffold383354_13.	1	p2	(TG)7	14	239	252	CATGCTCCGAAACATGAAAA
scaffold383463_15.	1	p2	(GT)6	12	343	354	TCATGCTGGAAGCACGTAAC
scaffold384039_13.	1	p2	(TA)7	14	1456	1469	CGCACTGATCCAACTTTTCA
scaffold384261_13.	1	p2	(TC)7	14	79	92	CCCTAAGTTTGCATCTCCCA
scaffold384332_10.	1	p2	(CT)8	16	239	254	CCACCCTCTCACTTCCACTC
scaffold384389_14.	1	p2	(TA)8	16	251	266	CTTGGCCGATGAAGAAAGAG
scaffold384639_16.	1	p2	(CT)6	12	3513	3524	TAGTTCCTTTGCCAACGGTC
scaffold384660_10.	1	p2	(TA)7	14	145	158	TGTCCCGTTTTCTTCGTTTC
scaffold384887_18.	1	p2	(AT)6	12	717	728	ATGTGGAGAAAGCTCTGCGT
scaffold385095_12.	1	p2	(TA)8	16	248	263	CGATGGCCAAGATTGCTCTA
scaffold385414_13.	1	p2	(AT)7	14	101	114	TCCTTTTCTTTTTCTGAGCACC
scaffold385530_18.	1	p2	(TC)8	16	368	383	CATGCTGTGCAGCAGTAGGT
scaffold385563_20.	1	p2	(TA)8	16	229	244	TACCGGACCTTGGTCTGAAC
scaffold385653_14.	1	p2	(TA)12	24	571	594	TTTCCTACCAAATCTCCCCA
scaffold385747_14.	1	p2	(GT)9	18	3487	3504	TGGATCTCGGAATATCCAGC
scaffold385874_17.	1	p2	(GA)7	14	728	741	CATCCCTACCTGTTGCCACT
scaffold385922_15.	1	p2	(CT)6	12	602	613	CCAAGAAAACTATCGGCAGC
scaffold386012_10.	1	p2	(AT)6	12	340	351	TCACTCACGCAGACAACACA
scaffold386094_13.	1	p2	(TA)7	14	344	357	GCGTGGAAAAGAAGATGCTC
scaffold386210_10.	1	p2	(GT)6	12	191	202	CCGTGAATGAAGGGATTTCA
scaffold386375_13.	1	p2	(GA)6	12	51	62	GCGAAGCACCGAGACTACTG
scaffold386471_15.	1	p2	(TA)6	12	401	412	CCGTAAGGCTCGTATGCTGT

scaffold386689_13.2	1	p2	(AT)11	22	342	363	AATGCACTGACTTGGCATGA
scaffold386807_12.4	1	p2	(TA)7	14	257	270	TGATCGATTTTCAGCCATGA
scaffold386919_10.8	1	p2	(TG)10	20	163	182	CTTGGCCTAAAATCCCAACA
scaffold387006_12.6	1	p2	(AG)7	14	53	66	CGAACTCAATTTGATCCGAAA
scaffold387041_13.0	1	p2	(AT)6	12	204	215	CCACTTTGGTGACTAACGCA
scaffold387052_17.7	1	p2	(CT)8	16	390	405	TATTTCCGGTTCAAACTCCG
scaffold387091_11.8	1	p2	(TC)6	12	92	103	TACCCAACCCACTAACCCAA
scaffold387146_13.0	1	p2	(TC)7	14	287	300	TTTTCGTCAAATCGACAACG
scaffold387158_12.3	1	p2	(AG)9	18	507	524	GGTGAAGACGGATCGTCAAT
scaffold387251_20.6	1	p2	(TC)6	12	474	485	CGAATCCGTTTTCCAACAGT
scaffold387422_11.1	1	p2	(TA)7	14	71	84	TTGATTCAAAGGCTGATAATGG
scaffold387594_12.0	1	p2	(TA)9	18	203	220	TGTGATCGACGATTGGAAAG
scaffold387840_13.6	1	p2	(AT)9	18	210	227	GGAAATTATTGGGAGGGAATG
scaffold387854_12.6	1	p2	(CT)6	12	83	94	CACGCACAAAGAAGATCTCTACA
scaffold387911_14.7	1	p2	(TA)8	16	172	187	CAATTTGCCCTTTCATGCTT
scaffold387941_15.3	1	p2	(GA)10	20	1506	1525	CCTGTATGTTGGGGTGTGTG
scaffold388098_13.3	1	p2	(TA)10	20	148	167	TTTCCTCCCAGCTTCTACCA
scaffold388245_10.5	1	p2	(CT)6	12	452	463	TCCCACTTTGTCTCCCAATC
scaffold388298_16.3	1	p2	(GA)6	12	283	294	CAGGAGAACTCGGCTGAATC
scaffold389022_18.0	1	p2	(CT)6	12	3086	3097	CATTCAGTGAAAACATGGCG
scaffold389075_10.0	1	p2	(GA)6	12	192	203	TCTCACTAATGGGCCAAAGG
scaffold389132_14.7	1	p2	(TC)7	14	774	787	AAGTTCGAATTCTCCCCACC
scaffold389181_10.8	1	p2	(TA)7	14	669	682	TTGTTGTTTCACAAAATTTAAGCTG
scaffold389191_13.3	1	p2	(TA)7	14	252	265	TGGACGAAATTGGTGTGTTG
scaffold389243_15.1	1	p2	(GC)7	14	170	183	CACGTGCGTCAACTCCTTTA
scaffold389600_15.8	1	p2	(AG)6	12	67	78	CAATTACAATGTTTATCCAATCTCA
scaffold389897_20.3	1	p2	(TA)8	16	592	607	TTCACGTACCCCTCATGTCA
scaffold389990_14.7	1	p2	(TA)6	12	381	392	GATCCAGTTCAAGTGCCCAT
scaffold390145_11.5	1	p2	(TA)8	16	65	80	AAATGCATGCACCTGATTACA
scaffold390368_12.3	1	p2	(TA)13	26	581	606	ACGCAACAGCTCATGACAAG
scaffold390395_14.2	1	p2	(TC)6	12	517	528	ACGAACCACTGGCTGAAATC
scaffold390458_15.9	1	p2	(TA)9	18	67	84	TGAAGTGTTATGCGTTTTGACA
scaffold390624_13.1	1	p2	(TC)6	12	439	450	TTCAACTGCATTGTTTCCCA
scaffold390643_17.3	1	p2	(TA)10	20	132	151	TGATCTTACGCGCACATCTT
scaffold390808_11.5	1	p2	(CT)6	12	403	414	GAAGAGACCAGAAGTCCCCC
scaffold391188_10.6	1	p2	(TA)8	16	265	280	GGTGGGCTTTTCTGACTCAA
scaffold391745_14.1	1	p2	(AT)8	16	231	246	CCCCAAATGAGGAACTTTGA
scaffold391772_16.0	1	p2	(CT)6	12	201	212	CACACATGCGTTCATTCACA
scaffold391820_15.5	1	p2	(AT)6	12	179	190	TAAAACCATGCACGTAGGCA
scaffold392025_13.6	1	p2	(AG)8	16	640	655	TTCGAGTTTGATTCCATCCA
scaffold392142_11.8	1	p2	(TA)8	16	567	582	GATTTTCCTGTCTCCTTTCTCAA
scaffold392160_11.2	1	p2	(TA)7	14	156	169	TACCTTTTTGCCCTCGAATG
scaffold392489_16.4	1	p2	(TG)7	14	103	116	AACACCTGTGAAAGGGATCG
scaffold392571_15.0	1	p2	(TC)6	12	375	386	TCGGAATTTTACGTGGTTTGA
scaffold393129_13.5	1	p2	(TA)6	12	164	175	AAACGAAGGAGACGATCGAA
scaffold393488_19.1	1	p2	(GT)6	12	85	96	AGTGGGCTTTGGCACAAGTA
scaffold393678_14.6	1	p2	(GA)6	12	423	434	GCTGGTTTGAGGGAGATGAG
scaffold393822_10.1	1	p2	(GA)6	12	433	444	TCAAAAATCCAAGGGTGGAG
scaffold393882_14.0	1	p2	(TC)8	16	216	231	CAAACCGTTGTGGATCTGAA
scaffold394585_13.5	1	p2	(CG)8	16	303	318	ATGGTGGTATGGTGTGGAGG
scaffold394634_18.8	1	p2	(TA)8	16	177	192	CGGAAATGACCCCATAATGA
scaffold394761_11.4	1	p2	(TC)7	14	80	93	CAGAAATGGCATTGGTTGTTC
scaffold394922_14.8	1	p2	(AT)7	14	611	624	CGCATGATGTAACGTGAAGG
scaffold394982_16.4	1	p2	(TG)7	14	401	414	TTGATTATTCCGCTTACGTTTTT
scaffold395246_13.4	1	p2	(AC)6	12	73	84	TCACATCCTCAGCACAAACC
scaffold395910_15.4	1	p2	(CT)6	12	613	624	TGTTCACGGGTGCTTTGATA
scaffold396150_14.2	1	p2	(AT)8	16	79	94	CAGAATTAGCCTTACCAAGATGG
scaffold396192_15.5	1	p2	(CT)6	12	175	186	AGATCAACGCTCCTCGTGAT

scaffold396458_11.(	1	p2	(TA)6	12	374	385	AAGAGTTATCACCGGATCGC
scaffold396597_14.2	1	p2	(GA)6	12	163	174	GGATGCAAGGTGAACTTGGT
scaffold396652_11.(	1	p2	(GA)7	14	814	827	AGAAGAAGCAGAGACCGACG
scaffold396874_10.(	1	p2	(TG)9	18	449	466	TGGGGTTGGAGGTTTTTGTA
scaffold397028_12.8	1	p2	(TA)11	22	841	862	ACACTAACGCTCGACCTCGT
scaffold397140_15.7	1	p2	(AT)6	12	2264	2275	TGCGGATTATCACATTGAGG
scaffold397338_10.3	1	p2	(TA)8	16	199	214	AATACCCTTCGCATCCACTG
scaffold397502_15.6	1	p2	(GT)7	14	268	281	CGCCGGACTATTCTCTGAAG
scaffold397641_18.(	1	p2	(AG)7	14	755	768	CGGGCTTGCATGAGTTTAAT
scaffold398006_14.6	1	p2	(TG)6	12	659	670	TCAATCAAATTTTATCAAACAAGAA
scaffold398020_15.(	1	p2	(AT)12	24	493	516	ACAACCCCGGACTATCGAAT
scaffold398025_12.5	1	p2	(AT)6	12	298	309	AAAAGATTACTCATGTGATATTGCG
scaffold398093_12.7	1	p2	(GC)8	16	170	185	TCCGGTCCAATTCCAATAAG
scaffold398360_15.7	1	p2	(AT)8	16	1856	1871	GCTGACGTAGTAGTTCCGGC
scaffold398756_16.7	1	p2	(AT)11	22	3005	3026	TGGTTTTCGTACACAATTCGAG
scaffold399077_15.5	1	p2	(AC)7	14	5432	5445	TGGAACCAAGACATATGGGAA
scaffold399165_15.9	1	p2	(CT)8	16	126	141	TACGTGATCGGACACGAGAA
scaffold399410_14.1	1	p2	(TG)10	20	4683	4702	ATCCTCCTTGCTTCCTCCAT
scaffold399472_10.5	1	p2	(AG)6	12	1001	1012	CGAAGGTTCTGCGATTTGTT
scaffold399622_13.7	1	p2	(TA)6	12	250	261	CGTCAAAATGCAAGACGTGA
scaffold399990_11.3	1	p2	(AT)8	16	230	245	AAAAATCAAACCTCAACCCG
scaffold400138_14.2	1	p2	(GA)6	12	4229	4240	GGTAGAGGAGCTGATGTGGC
scaffold400146_10.(	1	p2	(AT)8	16	479	494	CCCCTTACTGCTAACACCGA
scaffold400149_15.1	1	p2	(GC)7	14	1056	1069	TCAGTTTTGCAAGGATTGAGAG
scaffold400153_10.2	1	p2	(GA)6	12	382	393	GTGGTGGGAAACAAGAACCT
scaffold400154_15.8	1	p2	(CT)7	14	677	690	CGCTAAGGAGAGGCAAAATG
scaffold400157_15.3	1	p2	(TA)9	18	695	712	CCCTACAGATGGCAGGTTGT
scaffold400164_14.6	1	p2	(AG)6	12	2764	2775	AAATGCTCCTGCACTTCTCG
scaffold400185_13.6	1	p2	(TA)9	18	483	500	ATGTGTCAATTCCTGGGCTT
scaffold400186_15.5	1	p2	(GA)9	18	1932	1949	CACCAATTCCATCAGCCTCT
scaffold400208_13.1	1	p2	(TA)8	16	1435	1450	CGGTTGCAAGTTTGGAAAA
scaffold400220_13.4	1	p2	(TC)6	12	205	216	TACTAAAATACCCCCGCGAA
scaffold400238_11.6	1	p2	(CT)6	12	340	351	GGCTTAATGGGGTGGAAAAT
scaffold400281_10.6	1	p2	(TA)8	16	198	213	ACGAAAAACGAAGGAGACGA
scaffold400647_15.8	1	p2	(AT)6	12	604	615	TTGATTTTAAGAGGTGATATGATACG/
scaffold400735_14.4	1	p2	(AT)10	20	235	254	GCAGAAATTCTTTCTTCTGTTTTAGG
scaffold400851_10.6	1	p2	(AT)6	12	387	398	GTTTTTGTACATCGGCGGAG
scaffold400989_10.3	1	p2	(CT)7	14	69	82	ACAAATGGCCAAGAGAGAGTT
scaffold401005_16.6	1	p2	(GA)6	12	601	612	GGAGGTGCGGTGTTAGAGAG
scaffold401012_14.7	1	p2	(AT)7	14	1707	1720	TTATCAAGCCCACCGTAAGTG
scaffold401018_11.1	1	p2	(AT)6	12	416	427	CAACGAAAAGAGAAAGAGGGC
scaffold401057_13.2	1	p2	(AT)11	22	45	66	TGAATTCATGTACACATTTGGAA
scaffold401142_10.5	1	p2	(TA)7	14	260	273	GAGATATACGCAGGGGACGA
scaffold401234_14.1	1	p2	(TA)8	16	290	305	TGCGTTTTGACAAGATTTGC
scaffold401311_19.9	1	p2	(TA)8	16	68	83	CGGAAGAAACAAACTAGAAAGAACA
scaffold401380_11.2	1	p2	(TC)7	14	151	164	AGCGATGAAATGTACGTTAGAGA
scaffold401429_12.3	1	p2	(GA)7	14	314	327	GCTACTAACTTGATGCGGGG
scaffold401436_10.9	1	p2	(GA)6	12	46	57	GGAGGGATGATGAAGATGGA
scaffold401490_12.9	1	p2	(TC)6	12	313	324	GCACCAGATGTCGTGGATTT
scaffold401514_15.4	1	p2	(GT)6	12	1214	1225	TGAATGGATTTTTGGCATGT
scaffold401696_15.(	1	p2	(CT)6	12	176	187	TGTTTTATTAACCTCAATTGTGAAA
scaffold401812_13.8	1	p2	(AC)6	12	1354	1365	GAAGATGGGAAACCCCTTGT
scaffold401915_13.9	1	p2	(TC)7	14	1094	1107	CCGAAATTTTACGTGGTTCG
scaffold402069_12.6	1	p2	(AT)9	18	195	212	GATGCATGCACGTGAATAAGA
scaffold402088_16.3	1	p2	(CT)6	12	279	290	TAGGGTTTCAAAACATCGCC
scaffold402250_16.1	1	p2	(AC)6	12	121	132	TCTTTCGGGTTCTCAACCAC
scaffold402276_16.2	1	p2	(TA)6	12	442	453	AGCGATTCTTCCGTATGCTG
scaffold402344_16.2	1	p2	(AG)6	12	110	121	ATGGTGGTTTGGTGGAAGAA

scaffold402366_15.2	1	p2	(CG)6	12	185	196	TTTGCATACTGCATGGGCTA
scaffold402473_10.6	1	p2	(TC)6	12	37	48	GGCAACGGAATACCATTTGT
scaffold402522_16.1	1	p2	(CA)9	18	362	379	TACCAAAGCCGCTCCTAATG
scaffold402653_19.4	1	p2	(AT)8	16	77	92	GCTTAAAACAATTATTGAACCCG
scaffold402863_16.5	1	p2	(TC)6	12	285	296	CCAGTTCGGCGATATATGGTA
scaffold402887_17.2	1	p2	(GA)6	12	1664	1675	GATGGGAAAAGAAGGGAAGG
scaffold403408_11.1	1	p2	(TC)7	14	96	109	TAATGGCTGCCTATCAACCC
scaffold403506_14.9	1	p2	(AT)7	14	1526	1539	CATGTGAGAAACGTCCCCTT
scaffold403665_18.9	1	p2	(TA)10	20	68	87	CAAAATTTAAGGGTTATTATTGGATCA
scaffold403865_16.4	1	p2	(CT)6	12	2656	2667	CCCCTCATTTTTCGTTTTCA
scaffold403875_19.8	1	p2	(GT)6	12	333	344	TCAGTTTCCACACGCCAATA
scaffold403911_10.0	1	p2	(CT)7	14	80	93	TCATCATGAAACACCCTAGAAAA
scaffold404001_14.7	1	p2	(TA)6	12	264	275	TTGACCCACCTTTGACCAAT
scaffold404029_10.0	1	p2	(TG)6	12	340	351	ATCGTAGTGAAGTAGCCGCC
scaffold404095_13.1	1	p2	(TA)7	14	145	158	TGTGAGTTGGTGAGGCAAAG
scaffold404858_14.7	1	p2	(CT)6	12	71	82	TGTTGCTGATGCAGACAGGT
scaffold404867_16.0	1	p2	(AG)6	12	167	178	TGGAGTAGGGAGCCTCGTAA
scaffold404901_13.6	1	p2	(AT)10	20	46	65	TGGTTATTACCTGATCACCATCC
scaffold404903_12.7	1	p2	(AT)8	16	181	196	GTGGACCGGTGGAAAGAATA
scaffold404905_15.3	1	p2	(TC)6	12	374	385	TTTAATGGACACCTCTGCGG
scaffold405055_12.6	1	p2	(TA)6	12	228	239	GTTTTGTTTCCTGGGTTTGG
scaffold405121_10.3	1	p2	(AT)6	12	387	398	AATACATCCAGTCATCCCGC
scaffold405149_13.2	1	p2	(GT)6	12	1046	1057	ACCACTCGTGCATCACTCAA
scaffold405486_15.3	1	p2	(CT)8	16	986	1001	AGTCAAATTGGCATTACGGG
scaffold405603_16.7	1	p2	(TA)6	12	3021	3032	CCCTTCCATTTTGTTATATGGG
scaffold405673_17.1	1	p2	(CA)6	12	2666	2677	GGGGCTTTGAATCTACCAAA
scaffold405790_10.5	1	p2	(AT)6	12	198	209	ATCCATCTCATGTTGGGCAC
scaffold405888_15.5	1	p2	(GA)6	12	2307	2318	TAGAGGGAGGGGAGGGTTTA
scaffold405920_21.2	1	p2	(TA)7	14	77	90	TGAACTAAGGAGGTGTTATGACAGA
scaffold406006_11.9	1	p2	(AT)10	20	207	226	AAACAAACCGTGTTAACATCCC
scaffold406051_15.9	1	p2	(CT)9	18	89	106	TCTCATCTCTCGCTCTCCCT
scaffold406089_20.1	1	p2	(GA)7	14	118	131	CCCGTTCCATCACACTTCTT
scaffold406177_16.3	1	p2	(AT)8	16	946	961	TGCTTGGAAAGACAATTGAATG
scaffold406516_10.7	1	p2	(AG)11	22	546	567	CGAGCCAAGCCAGAAGTATT
scaffold406583_17.8	1	p2	(GA)6	12	1118	1129	CGAAGGTTGGGTGGTAGAGA
scaffold406618_12.1	1	p2	(AG)6	12	51	62	CAATACAACTGCCGAGCTGA
scaffold406732_12.3	1	p2	(TA)6	12	121	132	TGCTTGATCCAACGGGTTAT
scaffold406850_22.9	1	p2	(AT)14	28	1180	1207	CCCAAATCCGTGGATAAAAA
scaffold406994_11.9	1	p2	(CT)6	12	256	267	CTTTCTCAACCGAGCTCACC
scaffold407041_13.7	1	p2	(AG)8	16	793	808	TAATGGCCACCAAGCTTTTC
scaffold407116_15.4	1	p2	(AG)6	12	1514	1525	GAACGACGGAAATATGGAGC
scaffold407164_21.5	1	p2	(CT)6	12	474	485	ACACTTGCCAAGGGCATAAC
scaffold407591_16.3	1	p2	(TA)10	20	64	83	TGATGGTTGTGATTTGTTAGAGG
scaffold407660_12.4	1	p2	(TA)10	20	784	803	CGGCTGAGGACGAGAATTAG
scaffold407672_16.0	1	p2	(TG)6	12	516	527	CGGGTTTGAACCCTCAATAA
scaffold407690_15.3	1	p2	(AG)8	16	571	586	ACTGCCCAACAATGGCTTAC
scaffold407708_13.8	1	p2	(AT)9	18	68	85	TAAGGGCGGTATCACCTGAA
scaffold407715_16.7	1	p2	(CT)7	14	984	997	CCAACACTCTGCTCTCTCCC
scaffold407854_13.7	1	p2	(AT)8	16	964	979	ACGTAAACCCCTGGTCAAAA
scaffold407950_10.5	1	p2	(CT)9	18	328	345	TCCCAAATTATATCCTCTCCCA
scaffold407995_14.4	1	p2	(AT)6	12	156	167	AAAAATCAAGTTTAGGTGCGTCA
scaffold408020_15.3	1	p2	(TA)7	14	77	90	TCCATTCCGAAAATCGAAAG
scaffold408070_16.2	1	p2	(AG)6	12	214	225	ATGTCCGTCGTTGGTTCTTC
scaffold408124_14.4	1	p2	(AT)6	12	575	586	CCACTGATGTGAGCCAACTG
scaffold408141_17.5	1	p2	(AT)8	16	493	508	CTTGCCGATGTTTACCGTTT
scaffold408275_13.8	1	p2	(AT)9	18	211	228	GACTCTCAATGCCTTCTACCC
scaffold408388_14.0	1	p2	(CT)13	26	1926	1951	TCAAAGAATCCACCATGCAA
scaffold408495_15.4	1	p2	(AG)6	12	52	63	AGCTGTTAGGGTTGTATAGTGAGTAG

scaffold408515_16.4	1	p2	(GA)6	12	282	293	GCATTAGGTTGAGGGGGAAT
scaffold408630_16.4	1	p2	(GA)7	14	51	64	GCGGTGGTAGGAAGAAACTG
scaffold408683_10.1	1	p2	(TA)8	16	203	218	TGCATGTGGGCATGTTTAAT
scaffold408704_14.7	1	p2	(TG)6	12	245	256	ATTTTATGGGACGGAGGGAG
scaffold408751_13.8	1	p2	(TA)7	14	1372	1385	GGAATTGAATGTGGGAGTGC
scaffold408798_15.5	1	p2	(AG)6	12	2838	2849	CAATGTGGCCTGCCTATCTT
scaffold408805_16.8	1	p2	(TA)10	20	186	205	CCCAGTACCCCTCTTTGGAC
scaffold408850_15.9	1	p2	(TA)7	14	120	133	AAAAATGCAGATTATGCTCCAC
scaffold408928_14.6	1	p2	(CA)8	16	679	694	TCAATATCATCGCCATTTTGAA
scaffold408939_12.4	1	p2	(AT)11	22	853	874	GGCCGGTTTAAGATTCATCA
scaffold408949_13.6	1	p2	(TA)9	18	461	478	GGCGACAAAATGGATGACTT
scaffold409011_14.8	1	p2	(GA)6	12	772	783	AGGTGCATATTGAACCCGAG
scaffold409174_17.0	1	p2	(CA)6	12	189	200	TGCACAATATGTTGGGAGGA
scaffold409209_14.2	1	p2	(TA)7	14	256	269	GTGTCAAACTGTTCGCCTCA
scaffold409317_13.6	1	p2	(AT)6	12	260	271	GCATCGTTAGAAGCGTCGAT
scaffold409322_15.5	1	p2	(GA)6	12	121	132	CAAACGAGGAGCAAAAATCA
scaffold409342_11.3	1	p2	(TA)7	14	514	527	TTTGTCCAAACAAATGAAAACA
scaffold409368_10.0	1	p2	(GA)7	14	53	66	GTGAGGGATGGACGAGAGAG
scaffold409407_16.2	1	p2	(AT)9	18	2608	2625	CCTCACTGAAGTTCGAAAACG
scaffold409485_15.0	1	p2	(TA)8	16	269	284	AGCCACGATTCCTGATTTTG
scaffold409598_14.9	1	p2	(GA)6	12	89	100	AAACACGCAACACAAAGCAG
scaffold409613_20.3	1	p2	(GT)7	14	748	761	GTTCCTCATGCACCTTGGAT
scaffold409660_12.5	1	p2	(TA)6	12	162	173	ATCATCGGGGTGTAACGAGA
scaffold409697_14.6	1	p2	(TC)7	14	925	938	CACTGCCAGCAGTCGATAAA
scaffold409751_13.7	1	p2	(AT)8	16	66	81	AATGGATGGTTGTGATTGAGTT
scaffold409755_15.5	1	p2	(CT)7	14	526	539	GTCCACGTGCTTTCCTCTGT
scaffold409765_11.3	1	p2	(TA)11	22	220	241	AGTCGAAGGTCGCGTGTAAT
scaffold409969_15.9	1	p2	(AG)6	12	844	855	GCTGGCAATCCTTTCTTCCT
scaffold410086_11.5	1	p2	(AT)7	14	154	167	TTTTGCCCTCGAATGCTATC
scaffold410087_14.1	1	p2	(GA)7	14	75	88	TCCCACATTGCATGAGAGAG
scaffold410118_18.3	1	p2	(TG)7	14	42	55	CATTGTGTTTTCTCCATATTTTCA
scaffold410162_14.4	1	p2	(AG)7	14	822	835	ACACAATGGTGATGGCAGAA
scaffold410219_12.1	1	p2	(AG)7	14	53	66	TGTTTGAGTGGTTTTTCCTTGA
scaffold410233_13.7	1	p2	(CT)7	14	29	42	CCTCTCACTCCCACTCCATC
scaffold410279_15.9	1	p2	(GT)7	14	56	69	CCTGAAACTCCAAATATTCTTCTTG
scaffold410312_12.4	1	p2	(GA)9	18	742	759	CTTGCCTTACATCAGCCCTC
scaffold410595_14.0	1	p2	(CA)6	12	470	481	CCGGTGAACTTCTCCAACTC
scaffold410606_15.7	1	p2	(AG)6	12	136	147	AGAAAAAGTTGGGACGGAGG
scaffold410696_14.0	1	p2	(TA)6	12	268	279	TTGTTCGAAACCTAATGCCA
scaffold410788_10.4	1	p2	(TA)8	16	371	386	AAAAAGGAAGGAGGGAGCTAA
scaffold411136_20.8	1	p2	(AT)6	12	121	132	TGTAAAACATGTATGGGATTGAAGA
scaffold411195_17.6	1	p2	(AT)7	14	3901	3914	TGTGGTTGCGTCCTTATGAA
scaffold411312_16.1	1	p2	(TA)8	16	1885	1900	CATGGAGGGGTCAAACATTC
scaffold411410_14.5	1	p2	(AT)7	14	637	650	GGGGTGAAATTGGGAGAGTT
scaffold411492_12.1	1	p2	(GT)7	14	35	48	GGATACCCTCCCAAACCCTA
scaffold411532_21.2	1	p2	(CT)6	12	97	108	TCTCATTCACGCTTTTCAGC
scaffold411615_15.0	1	p2	(TA)9	18	1060	1077	TTTTGAGCTTCCGCGAGTAT
scaffold411763_16.4	1	p2	(TA)10	20	173	192	TGGACTTAAGTACCCTTTTACCAA
scaffold411790_15.0	1	p2	(GA)7	14	1563	1576	GGGGTGGTTAAGTTGTGGTG
scaffold411810_13.4	1	p2	(AT)8	16	336	351	TTTGCTAGGGACCGAAACC
scaffold412001_14.7	1	p2	(TA)12	24	471	494	AGTATGCTGACGCTGGGTTT
scaffold412042_14.4	1	p2	(CT)8	16	117	132	CTTGGGCTAAAATCCCAACA
scaffold412086_16.6	1	p2	(GA)6	12	1819	1830	GGTTGATTGGTTTTCATGGC
scaffold412159_10.5	1	p2	(AG)7	14	69	82	CAAATATTTTGTAGTTTCCCATGTTG
scaffold412247_17.0	1	p2	(GA)12	24	4389	4412	TTCGAGCTCTCCTTTTTGGA
scaffold412258_10.4	1	p2	(AG)6	12	360	371	CCCCACCAGAAGTCCAACTA
scaffold412459_11.1	1	p2	(AG)6	12	86	97	GGATTTTCGAAATTTTCTCGC
scaffold412476_14.9	1	p2	(AT)6	12	1414	1425	CTGCATCGAGTATCGCTCAA

scaffold412534_10.6	1	p2	(GA)6	12	55	66	CGTGGTTCGATCGTAATTGA
scaffold412841_15.7	1	p2	(AT)6	12	1033	1044	TTTCTTTAATCACCGCACCA
scaffold412863_13.8	1	p2	(GA)6	12	463	474	TCTGGTCGAGCTGGAAGATT
scaffold412869_12.2	1	p2	(CA)9	18	55	72	TGTGATTTGTTCCCACAAGAA
scaffold413066_20.3	1	p2	(CG)6	12	60	71	GCACTGTGTTACGCCGATTA
scaffold413130_16.9	1	p2	(CT)7	14	295	308	AAAAGAAAAAGTGAAGGGCTCA
scaffold413146_13.4	1	p2	(CA)6	12	743	754	TTCCTAAAAACGTTCCGCTG
scaffold413147_11.1	1	p2	(AG)7	14	35	48	GAAGAACAATTACAATGTTTATCCAA
scaffold413223_17.4	1	p2	(TC)13	26	2490	2515	CTGCAGAGCTCCATGACAGA
scaffold413315_18.0	1	p2	(GA)6	12	53	64	TCAACAGAGAATGCAAGCAAA
scaffold413360_13.9	1	p2	(TA)8	16	181	196	AACATGATTTGATGGTGCCTC
scaffold413363_11.9	1	p2	(TA)9	18	45	62	TGCGTTTTGACAATATATAGGTGTTT
scaffold413392_16.6	1	p2	(TC)6	12	122	133	CTTCCCAGGATCGATTTGAA
scaffold413411_16.0	1	p2	(AT)9	18	609	626	AATGCAAAAATATGAGGCCG
scaffold413460_16.4	1	p2	(GA)6	12	72	83	TTGGTGGAAAAAGGTTAACGA
scaffold413678_11.7	1	p2	(TA)7	14	51	64	GGTTTCTGGTGAAGATGACGA
scaffold413693_15.4	1	p2	(TA)9	18	45	62	AAGGGGGTTATTAGTGGATCA
scaffold413719_12.0	1	p2	(AT)7	14	169	182	TGTGCGTGTATGCAATGTGT
scaffold413983_23.9	1	p2	(AT)9	18	1048	1065	ATGGAACGTTGATTGCCATT
scaffold414075_11.6	1	p2	(GA)6	12	217	228	TCCTGCATCCTATCACATGG
scaffold414101_14.1	1	p2	(CA)6	12	65	76	TCTCGACTAATTATTATGCCTACCA
scaffold414271_10.6	1	p2	(AT)11	22	67	88	AATTAAAATTTGGGGAGGGG
scaffold414329_13.4	1	p2	(GA)12	24	1026	1049	CAGACCGTCGAATTTAGACCA
scaffold414359_12.9	1	p2	(CT)6	12	81	92	TTTGGGACGATTTTCTCTCTATG
scaffold414787_12.6	1	p2	(AG)6	12	952	963	TGCTGCAGAGAAGGTAGGGT
scaffold414830_10.8	1	p2	(TG)6	12	108	119	CTCGTTTGTGTGTTCTTCGTG
scaffold414890_15.2	1	p2	(GA)6	12	306	317	TGGTTGTTTTAATTTGCTCCC
scaffold414923_15.6	1	p2	(CT)6	12	577	588	CCTTTCCTTTACACCACAAGGA
scaffold414974_17.3	1	p2	(TA)8	16	70	85	TCAATGGCTCAAAAAGTTTCA
scaffold414982_14.8	1	p2	(TA)8	16	235	250	AAATGAAATAGAATTCGCGAGG
scaffold415123_11.7	1	p2	(AT)6	12	122	133	CACACACATTACATTAAACCGATG
scaffold415129_14.1	1	p2	(AT)6	12	532	543	GATCGCATGTTCGATCTTCA
scaffold415154_16.9	1	p2	(TC)7	14	62	75	CACATCCCACCCAACTCTCT
scaffold415294_14.4	1	p2	(AT)6	12	592	603	TGGACGTAAATACCCTTTTACCA
scaffold415324_15.6	1	p2	(CT)7	14	270	283	GAGCCCAAACCTCAATCAAC
scaffold415374_14.3	1	p2	(AT)9	18	660	677	GGGCATGAATTCCAAAACTC
scaffold415578_17.2	1	p2	(TA)6	12	2523	2534	CTCGGAAAAGTCTACCGGC
scaffold415641_15.2	1	p2	(AG)6	12	881	892	AGTGAGCCTACACCACCACC
scaffold415744_13.1	1	p2	(AT)6	12	190	201	CCCCAATGCTATGGACAAAC
scaffold415894_10.3	1	p2	(GT)7	14	199	212	CGTCTGAGCTCGGTAGAAGC
scaffold416075_16.9	1	p2	(AT)7	14	325	338	AGATCCGATGTCCCAAACAC
scaffold416092_16.1	1	p2	(CT)6	12	3138	3149	ATGTGCTCTTCATGGGGAAG
scaffold416164_15.8	1	p2	(TG)9	18	116	133	CATCCCAACCACTCATTTCG
scaffold416549_13.8	1	p2	(AT)6	12	1990	2001	AACTGGTATGTGCGTGTGGA
scaffold416621_16.6	1	p2	(CA)7	14	48	61	GGTTAATCTCTCTATCTCTCTCACACA
scaffold416719_10.3	1	p2	(TA)7	14	120	133	GCAGTGTAGTTAGGCGGTTTG
scaffold416903_13.3	1	p2	(TA)7	14	867	880	TTGTCGGTCATTGAATTGTGA
scaffold417101_11.2	1	p2	(TG)7	14	79	92	TTTTCATTTGATCACCTCCC
scaffold417120_13.0	1	p2	(TA)9	18	264	281	GATCTGCGAAATGCTTAGCC
scaffold417421_16.7	1	p2	(CT)6	12	626	637	ACATCATCTTCCTTACGCGG
scaffold417455_11.3	1	p2	(AT)8	16	223	238	CTGGAACAACAGCTACGCTC
scaffold417477_11.2	1	p2	(TA)10	20	514	533	GCTCACAGATCCCACCTGAT
scaffold417608_11.0	1	p2	(TA)9	18	44	61	AAGGAGGTGTTATGAAGGGATT
scaffold417668_10.9	1	p2	(TA)10	20	77	96	CCCCTGTATGCACCTCATTT
scaffold417669_11.8	1	p2	(CT)6	12	623	634	TTCTGTCCTCAACCACCCTC
scaffold417751_16.6	1	p2	(TA)6	12	406	417	TTGGCACTGAGTACCCATTTT
scaffold417866_10.3	1	p2	(CT)6	12	38	49	CCCAGGTCTCAATCTTTTTGG
scaffold418001_16.9	1	p2	(GT)7	14	120	133	TGCTGCTATTGAAGCCATTG

scaffold418271_19.[illegible]	1	p2	(AT)6	12	1501	1512	GAAGGTGAACGTCGGTAGGA
scaffold418293_16.[illegible]	1	p2	(TA)7	14	486	499	TGATAGAAACACTTCGCGAGC
scaffold418387_14.[illegible]	1	p2	(CT)6	12	324	335	CACGCATCTCTCTCTTCCCT
scaffold418652_17.[illegible]	1	p2	(TA)7	14	823	836	TGAGGCTCCAGATTTGCTCT
scaffold418660_11.[illegible]	1	p2	(TA)6	12	158	169	GAGGCCTACTTCCTGGATCA
scaffold418727_12.[illegible]	1	p2	(CT)6	12	180	191	GGAGTTTCTATCCGCCCTTC
scaffold418728_17.[illegible]	1	p2	(AG)6	12	123	134	CGATGGTCTGAGTTATCCTCTG
scaffold418969_17.[illegible]	1	p2	(AT)7	14	120	133	TCGTGTGTAGGAAAAACATCG
scaffold418971_15.[illegible]	1	p2	(TA)7	14	46	59	CGATCACGTTTTCCAGTTCC
scaffold419114_17.[illegible]	1	p2	(TG)9	18	307	324	CGTTTTGATGATGAGCTGGA
scaffold419138_12.[illegible]	1	p2	(AT)8	16	165	180	GCGGTTTGAGCCTAATTTTG
scaffold419196_16.[illegible]	1	p2	(TA)6	12	159	170	TTCGTTTACAGCATACACCGA
scaffold419210_16.[illegible]	1	p2	(AG)7	14	385	398	GAGTGCTGCAGCGTATGTGT
scaffold419272_14.[illegible]	1	p2	(TA)12	24	534	557	CAATTGCTATAAATGAAACCGGA
scaffold419294_12.[illegible]	1	p2	(TA)7	14	197	210	AAACTTGCTTCCGTTGGATG
scaffold419457_12.[illegible]	1	p2	(AG)6	12	76	87	TCCTCAATTTCAGTGTGGAAAA
scaffold419514_16.[illegible]	1	p2	(TC)7	14	262	275	CACGGGGTTTCCAGACTTTA
scaffold419515_12.[illegible]	1	p2	(TA)6	12	97	108	AAATTAAGTGGGGGTGGAGG
scaffold419849_19.[illegible]	1	p2	(TC)6	12	236	247	CCCAAATCTAGAAGCCACGA
scaffold419894_15.[illegible]	1	p2	(AT)6	12	1423	1434	TTCGATACGAATTTTCATACCAT
scaffold420131_15.[illegible]	1	p2	(CT)6	12	84	95	GATTTTGGGACGATTTTCTCTC
scaffold420137_20.[illegible]	1	p2	(CT)6	12	86	97	GCGAGTGTCGCCTAGTGTAG
scaffold420231_16.[illegible]	1	p2	(AT)7	14	542	555	TGAGCACGTATGGACACGAT
scaffold420236_16.[illegible]	1	p2	(AG)7	14	2638	2651	GAGCCACGGCCTATTACAGA
scaffold420243_12.[illegible]	1	p2	(TA)6	12	563	574	TTGAATCCAGGATCTTGAGC
scaffold420246_12.[illegible]	1	p2	(AT)7	14	215	228	CCCAAGCCAGAGTGAGAGAG
scaffold420279_12.[illegible]	1	p2	(AT)7	14	54	67	TTTGCTAGAGGTTTTGACGAGA
scaffold420446_18.[illegible]	1	p2	(AG)6	12	2478	2489	TGGATTGAAGAATGGAAGCA
scaffold420448_14.[illegible]	1	p2	(GA)8	16	2338	2353	CATTCACTTGAGGAATGGGG
scaffold420479_15.[illegible]	1	p2	(GA)7	14	1799	1812	ATCCCGTCTGCGTTTATCAC
scaffold420492_15.[illegible]	1	p2	(AT)6	12	2491	2502	TTCAAACGTGGCACCAATTA
scaffold420502_14.[illegible]	1	p2	(TA)9	18	50	67	CACAAGATTTATGGGTTATCATTTG
scaffold420506_11.[illegible]	1	p2	(AT)10	20	369	388	AAAAGGTTAAGGGTGGGGTG
scaffold420516_15.[illegible]	1	p2	(TA)6	12	215	226	TTCCCATCAGTCAAGATCTACC
scaffold420555_16.[illegible]	1	p2	(TA)8	16	584	599	TCCTCGACTGTTTTTCCCAC
scaffold420617_13.[illegible]	1	p2	(AT)8	16	1637	1652	GCAATGGTGTTGCATATGTGT
scaffold420686_14.[illegible]	1	p2	(TC)6	12	2224	2235	TGCTACGAAGACATCCACCA
scaffold420693_15.[illegible]	1	p2	(AT)6	12	267	278	GCCTCCTTTTCTTTTTCTCG
scaffold420864_10.[illegible]	1	p2	(CA)6	12	61	72	TGGGAGAATTTTCTGCTCTTG
scaffold420921_10.[illegible]	1	p2	(AG)6	12	114	125	CCTCCCAACCCCAAATTTAT
scaffold420938_15.[illegible]	1	p2	(AT)10	20	43	62	ATCGGTTATTACCGGATCGC
scaffold420988_14.[illegible]	1	p2	(GA)6	12	895	906	CTTAAATCCACCGCCATTGT
scaffold421052_14.[illegible]	1	p2	(TG)6	12	1275	1286	TGAGGGACTTGGGTAGTTGC
scaffold421219_15.[illegible]	1	p2	(AG)6	12	1193	1204	ACAGAAGAACGGGCTTTGAA
scaffold421249_10.[illegible]	1	p2	(AC)6	12	691	702	TTTTTAATTCATCTTAACTGATGCAA
scaffold421276_17.[illegible]	1	p2	(TC)6	12	364	375	GCGCAAAAATACTCGCTTTC
scaffold421297_11.[illegible]	1	p2	(CT)6	12	209	220	ATCTACCCCTCATTCCCGAC
scaffold421350_12.[illegible]	1	p2	(CT)6	12	43	54	GATCTTTCCACAAACCGAGC
scaffold421401_10.[illegible]	1	p2	(AT)6	12	221	232	ACATTTTCGGCCATGACAAT
scaffold421501_17.[illegible]	1	p2	(TC)6	12	330	341	TCCACATTGGTTTGGCCTAT
scaffold421528_11.[illegible]	1	p2	(TA)9	18	1212	1229	CCAATTCGAAAACCGGTAAA
scaffold421589_16.[illegible]	1	p2	(TA)8	16	69	84	GGGCTAAGTGTTGAGATTGGA
scaffold421735_14.[illegible]	1	p2	(GA)6	12	731	742	TGCGTTTGATCGTTAGCTGT
scaffold421831_13.[illegible]	1	p2	(AT)7	14	189	202	TGCACGTAGGCATGTTCAAT
scaffold421857_14.[illegible]	1	p2	(GA)9	18	1035	1052	CCATCCATGCCTTGGTACTT
scaffold421859_11.[illegible]	1	p2	(TA)7	14	67	80	CGAGTGATCGTGAGACAGTGA
scaffold421881_18.[illegible]	1	p2	(GA)8	16	306	321	GCGAAGAAACATTCAGGGAA
scaffold421989_16.[illegible]	1	p2	(TC)7	14	692	705	ATCGAGTAAACTAGGCGCGA

scaffold422116_16.7	1	p2	(TA)8	16	119	134	ATGCATGTGTGCCAAGTGTC
scaffold422117_14.1	1	p2	(AT)8	16	1849	1864	CAAAATGTCGAAAAATTCGC
scaffold422118_15.8	1	p2	(TA)6	12	123	134	TATCCATGGAATGCCCAAAT
scaffold422119_17.0	1	p2	(AG)6	12	898	909	TTACATGGATGGATGGGTTG
scaffold422224_13.1	1	p2	(AT)6	12	63	74	GCAAACCAAACATGTATCAAAA
scaffold422267_10.2	1	p2	(TA)8	16	306	321	GAATCCATTTTGTGTTGGGC
scaffold422280_15.8	1	p2	(TA)6	12	4265	4276	TCTGGACCTTTCGGTTCTTG
scaffold422353_15.2	1	p2	(AT)9	18	241	258	AAGCAGGAGGTCCACAGTTG
scaffold422364_12.3	1	p2	(AT)7	14	920	933	GTGCTCTCAAGGTTCTTGCC
scaffold422520_16.5	1	p2	(TA)6	12	481	492	GCTGGTGCATCCTCTTCCTA
scaffold422748_15.0	1	p2	(TA)9	18	491	508	TGCGTTGGATCAATTTGTGT
scaffold422789_17.2	1	p2	(TG)8	16	663	678	ATCGCACGTGCTAGATCCTT
scaffold422798_14.3	1	p2	(AT)7	14	1356	1369	TTGCAATCCGGATCAAAAGT
scaffold422812_16.5	1	p2	(TG)6	12	1406	1417	TGCAACATGATTTTGTCGGT
scaffold423108_15.7	1	p2	(AT)6	12	71	82	TGAGCAAAAGTAATAAGAGCGACA
scaffold423140_15.1	1	p2	(AG)7	14	42	55	TTGTAATTATTGTAGGGAATAAAAGG
scaffold423167_10.7	1	p2	(TG)6	12	177	188	ATCTCAGTGCGTGTGAGTGC
scaffold423191_17.3	1	p2	(TA)6	12	503	514	TAGACGCCCCAGACTGTTTC
scaffold423248_13.9	1	p2	(TA)9	18	44	61	AAGGGTTATCACCTGATTTTCTC
scaffold423264_12.5	1	p2	(AC)7	14	447	460	CTCAACGGTATGCTCGGATT
scaffold423388_16.2	1	p2	(CT)9	18	105	122	CACTCACTCTCACTAGCCGC
scaffold423463_14.2	1	p2	(CT)8	16	341	356	ACGGCTATAGCAAGGGTCAA
scaffold423534_14.0	1	p2	(GA)6	12	152	163	CACGATGGACGATTGAACAC
scaffold423746_15.1	1	p2	(AT)6	12	764	775	TGGCTGTGAATCATTGTTCG
scaffold423864_15.9	1	p2	(TA)7	14	224	237	AAATGCGCAATTTTAAACCC
scaffold424034_10.2	1	p2	(TC)8	16	355	370	CGGCACCAATTTATTGTGAA
scaffold424097_15.8	1	p2	(AG)8	16	120	135	GGTTGGTCCACAAGATTTACAG
scaffold424539_13.3	1	p2	(CT)7	14	299	312	CATACCTCCCCTTACCATCG
scaffold424560_17.8	1	p2	(AT)7	14	612	625	TCCGAAATTTAAGGAGTCGG
scaffold424609_11.7	1	p2	(AC)6	12	234	245	AAGGAGATCATGGGATCGTG
scaffold424652_18.1	1	p2	(TC)7	14	122	135	TCTCAGGTGACAATAGGGGG
scaffold424791_15.3	1	p2	(CT)6	12	1294	1305	CTCATTTTGCACGTCTTCCA
scaffold424806_14.9	1	p2	(TA)6	12	199	210	AGAAACGTTTTCCCTGAGCA
scaffold424825_17.0	1	p2	(CT)6	12	846	857	AATGGTGGTGCACTTTTGGT
scaffold425061_10.0	1	p2	(AT)9	18	146	163	CCGGGGTATGCTATAAACGA
scaffold425067_13.3	1	p2	(TA)8	16	212	227	CCCTTCGCATCTTACTGCAT
scaffold425087_11.2	1	p2	(TA)8	16	267	282	TTCTCACAAGCAAAGACCCA
scaffold425112_15.9	1	p2	(TA)7	14	2022	2035	GAGCGTAATATCAAATTTTTGGG
scaffold425153_15.0	1	p2	(TA)6	12	123	134	TCGAAAATCAAAGAGCCTCAA
scaffold425160_14.8	1	p2	(AT)6	12	69	80	TTCTTAATTTGCATGTTCGTTATACA
scaffold425173_11.9	1	p2	(TA)8	16	48	63	GGTGCAAGTGTCAATAGAAGAGTT
scaffold425283_13.3	1	p2	(AG)7	14	48	61	AGAAACTTGACGTGTGCGTG
scaffold425316_13.5	1	p2	(CT)11	22	667	688	TCTGAAGTCCAGCAACAGGA
scaffold425420_11.1	1	p2	(GC)6	12	361	372	GTGGAGAGGCAAGAAACTGC
scaffold425461_15.6	1	p2	(GA)7	14	111	124	CATTCTCAAGCCACTCCCTC
scaffold425615_17.0	1	p2	(AC)6	12	307	318	TCCAGGTTTGCTGCTAGGTT
scaffold425741_13.0	1	p2	(GA)6	12	68	79	TGGATACGAGATTCGGGAAG
scaffold425790_15.8	1	p2	(CT)6	12	2595	2606	TTTTGGTAAAGTTTTGAGATTCCA
scaffold425805_15.4	1	p2	(GA)9	18	892	909	GATCCACGGGTCTCTTGAAA
scaffold425813_10.2	1	p2	(TC)7	14	412	425	TTCAACCTTTTCCTTGTCTCC
scaffold425837_19.7	1	p2	(TA)8	16	72	87	ACCAATTTGATGAGGGACCA
scaffold425850_11.8	1	p2	(GA)7	14	145	158	TGGTTGAAAATCATTGTGGG
scaffold425858_14.1	1	p2	(AT)7	14	560	573	AAAGGGAATTTTGTCAACTGGA
scaffold425875_16.6	1	p2	(CA)6	12	1829	1840	TGGAGCCTACCATGACCATT
scaffold425897_14.3	1	p2	(CA)7	14	1181	1194	AACGCGAAGAACACAAGAGG
scaffold426067_16.7	1	p2	(TA)6	12	382	393	ACACGTGCCTCGTTTGAAAT
scaffold426141_15.4	1	p2	(AC)7	14	5809	5822	GAAAAAGGCAGAAATGCAGC
scaffold426235_18.9	1	p2	(TG)6	12	824	835	GGCCAACATATGGGATTTTG

scaffold426255_20.5	1	p2	(AT)8	16	510	525	CAGAAGCCTCCAACACCAAT
scaffold426258_11.9	1	p2	(AT)6	12	56	67	CGACGTTGTCTTAACCTTGGA
scaffold426344_13.8	1	p2	(AT)7	14	2172	2185	TAGTTAAACTCACGCACGGC
scaffold426406_15.2	1	p2	(AT)6	12	407	418	TTTCCCTTCTTTTGGGAGGT
scaffold426448_15.0	1	p2	(GA)6	12	189	200	GTTGGAGGAGAGCTGTAGCG
scaffold426483_11.0	1	p2	(TA)10	20	46	65	CACCCAAAATGGGTTATTATTTG
scaffold426514_13.8	1	p2	(TA)6	12	164	175	GCACGTTTTTGGCCTGATA
scaffold426515_18.5	1	p2	(TA)9	18	112	129	GCGGGGTTGTTGAAGAATTA
scaffold426557_13.1	1	p2	(CA)11	22	291	312	TTACTGTTGCATGGACTGGG
scaffold426572_14.3	1	p2	(AT)11	22	72	93	GGTATTCGTGATCGTGATTCCT
scaffold426666_19.4	1	p2	(AT)6	12	34	45	GGGTTCGTAACCTAGGAGCTG
scaffold426740_15.9	1	p2	(CT)7	14	123	136	CAAGCGGAACAAACAAACAA
scaffold426812_14.7	1	p2	(AT)6	12	206	217	TGCTCAGCATACATGAAGGG
scaffold426859_13.0	1	p2	(TA)11	22	940	961	ATCAAGCCTGATATGTTTGACTC
scaffold426865_14.8	1	p2	(TC)6	12	3005	3016	GGCTGTTCAGCAATATGCAA
scaffold426905_12.4	1	p2	(TA)6	12	53	64	CGGATTTAAGGGTTATTACATGA
scaffold426947_13.2	1	p2	(CT)6	12	293	304	CATTTTTCCACCTTGTGCCT
scaffold426952_15.1	1	p2	(AT)8	16	255	270	GGAGGGCTGTGTGATTGATT
scaffold426959_15.7	1	p2	(CA)6	12	59	70	ATCGTCGCCTCTAGCCACTA
scaffold427000_11.0	1	p2	(AT)7	14	67	80	GGTTTTACGAACCACAATCCA
scaffold427267_14.2	1	p2	(TA)8	16	121	136	TTTGCACCTGAGGAGGTGTT
scaffold427325_16.5	1	p2	(TG)6	12	1077	1088	TCTTTGCAGCTTGCACATTC
scaffold427377_12.4	1	p2	(TC)6	12	607	618	TTCTCCTATCTTCCCCAGCC
scaffold427419_15.3	1	p2	(AT)7	14	389	402	AACTGGCATCCATACGAAGC
scaffold427532_13.2	1	p2	(CT)9	18	895	912	AATCTCTCAATCGGCACACC
scaffold427668_14.6	1	p2	(TA)8	16	151	166	AGGGTTTAAAATCGCGCATA
scaffold427679_16.3	1	p2	(CT)6	12	517	528	TCCCATTGGTTATGGGAAGA
scaffold427836_14.6	1	p2	(TA)8	16	627	642	ACACAGGAGTTTGCGGATTC
scaffold427838_10.8	1	p2	(TA)9	18	143	160	ACACCCACAAATCAACCACA
scaffold427860_15.3	1	p2	(AT)9	18	449	466	CAAACCAAGGTCGAGAGTTGA
scaffold427929_13.7	1	p2	(AG)7	14	89	102	CTTCACGTGACTTGCTTCCA
scaffold427936_13.9	1	p2	(CT)6	12	446	457	AGGTGATTTGGCATTAAGGG
scaffold427997_13.5	1	p2	(TA)10	20	365	384	AAAGCAATTTCAACATAGGTCGT
scaffold428103_17.4	1	p2	(CT)6	12	125	136	ACTCGCCCCTCAAGTTTCTT
scaffold428155_16.1	1	p2	(AG)7	14	431	444	CTGTAGTCGTGGTGGGCTTT
scaffold428381_15.7	1	p2	(TA)8	16	574	589	TTCACAAACCCAGATATCATAATCA
scaffold428453_13.9	1	p2	(AT)9	18	1317	1334	CTTCTAAAGAACCCTCCCCG
scaffold428478_11.7	1	p2	(AG)9	18	221	238	AAGCACCGGTACTCGACATC
scaffold428564_16.9	1	p2	(AT)6	12	53	64	TGTGATGTTGTATTTTGGTACATGA
scaffold428615_16.0	1	p2	(CT)6	12	200	211	ACAAACGGTAATTCCCCTCC
scaffold428663_17.3	1	p2	(TC)6	12	72	83	GCAACGGAATACCACTCGTT
scaffold428783_10.8	1	p2	(AT)11	22	291	312	TGCTATTGCATGCTAAGAAGTGA
scaffold428850_12.7	1	p2	(TA)9	18	526	543	TGTGGAATGGGAGATAACAGAA
scaffold428853_11.8	1	p2	(AG)6	12	73	84	GTGTTGGACGCATCAGGTAT
scaffold428904_12.2	1	p2	(CT)6	12	486	497	ACAACAATCCGGAGGTGAAG
scaffold429019_12.5	1	p2	(TA)6	12	205	216	ATCAAATCTTGGTGCGGAAC
scaffold429064_16.1	1	p2	(GA)9	18	115	132	TTACCTTTCTCGATCGGGTG
scaffold429075_15.9	1	p2	(TA)6	12	293	304	TCGCTTAATTGGTGATTGTGA
scaffold429223_15.6	1	p2	(AT)8	16	299	314	ATTTTTCGGCGAGATTGCTA
scaffold429241_11.4	1	p2	(TA)7	14	55	68	TCAATCAATTAGATTTTCGGCTC
scaffold429341_11.9	1	p2	(AG)7	14	123	136	AACAGCAGGTGAGTTGGAGG
scaffold429349_17.1	1	p2	(TA)6	12	1849	1860	TTTCACTGTTTCCACACATGC
scaffold429418_13.2	1	p2	(CT)7	14	410	423	AGAGCCATGTCTCCTTCACG
scaffold429574_18.0	1	p2	(CT)6	12	1150	1161	CTTTGAGTTGGATGGCTGGT
scaffold429637_10.4	1	p2	(AC)6	12	169	180	TCTTTTCTCTCCCTCCCCTG
scaffold429645_14.6	1	p2	(CT)6	12	877	888	GCTTCTATCGCGCTCTCTCT
scaffold429765_11.8	1	p2	(TG)6	12	39	50	TGTTCTTCGTGTTCTATATTTCTCG
scaffold429849_15.5	1	p2	(CT)8	16	10638	10653	TGAAATGTGTTGGCTGGGTA

scaffold429899_18.	1	p2	(TA)7	14	936	949	CATGTTCAGTTTTTGGGGCT
scaffold429909_14.	1	p2	(AT)7	14	72	85	TATCAAAACAACCCCCGGAC
scaffold429923_20.	1	p2	(TC)7	14	225	238	GAGGAGAATTCATGGGGTGA
scaffold430155_10.	1	p2	(CT)8	16	331	346	CAAGTCAAGCAAGGTGGACA
scaffold430183_11.	1	p2	(GA)6	12	53	64	GCGACAAGTCTGCGTGTTAT
scaffold430184_11.	1	p2	(TC)7	14	288	301	CACATTCTCCGGTTTTTCGT
scaffold430292_14.	1	p2	(TA)7	14	254	267	TAACATGGAGGACAGGGGTC
scaffold430432_16.	1	p2	(TC)7	14	556	569	TCTCAAGATGAGAACTCGGAGA
scaffold430448_16.	1	p2	(AC)6	12	587	598	CAATAAAGTGGGGCCAAAAC
scaffold430506_15.	1	p2	(GA)10	20	5349	5368	CCTCTGGTTGTTGGAATGGT
scaffold430547_16.	1	p2	(CT)6	12	536	547	CAAAGAAGGCAATCTATCCCA
scaffold430793_15.	1	p2	(TC)10	20	253	272	AATCCCAATTTCCCAACCTC
scaffold430851_13.	1	p2	(TA)6	12	2322	2333	ATGGACCCCACCTGTCATAA
scaffold430971_13.	1	p2	(CT)6	12	181	192	CTCACCCTTGTCATCACCCT
scaffold430995_10.	1	p2	(TA)6	12	349	360	CGCACTCACACACTCTGACA
scaffold431008_16.	1	p2	(AT)11	22	491	512	TGCACCGTTAACAAAAATGA
scaffold431020_14.	1	p2	(TA)7	14	741	754	ATTGCTAGGGTAGCCTCCTT
scaffold431075_14.	1	p2	(AC)6	12	1527	1538	AAACGCCATGCAAAAATACG
scaffold431142_14.	1	p2	(CT)6	12	120	131	AAGCTGCACGTGTTACACCTAA
scaffold431283_14.	1	p2	(TC)6	12	126	137	AAATAAAAGAGGAAGGCTGAGCTA
scaffold431315_14.	1	p2	(CA)6	12	2916	2927	TCTTTCCCACTTCCCACATC
scaffold431376_16.	1	p2	(CT)6	12	92	103	CAGTCACTCTCACTAGCCGC
scaffold431464_16.	1	p2	(TC)10	20	283	302	CTGAATGCTCGTTGTCTCCA
scaffold431640_14.	1	p2	(AT)6	12	628	639	CGCGCACACACTTACAAACT
scaffold431694_17.	1	p2	(CT)7	14	750	763	TCTTCTCCCTCTCTCCCTCC
scaffold431723_13.	1	p2	(CT)6	12	583	594	TGCCCACAAAGATTTCATCA
scaffold431801_15.	1	p2	(CT)6	12	52	63	GCATCTCTTGCTCTCTCGTTG
scaffold431913_16.	1	p2	(AT)6	12	126	137	AGGCATCCATCCTTTACTGC
scaffold432000_15.	1	p2	(AT)6	12	1369	1380	AAGGTAAACAAACAATCAAGGGA
scaffold432031_13.	1	p2	(TA)6	12	65	76	TCCACCGTTGTCTTTAAGTCG
scaffold432050_12.	1	p2	(GA)8	16	346	361	GAAAAACGACGTGAGGGAGA
scaffold432181_13.	1	p2	(CA)6	12	1152	1163	ATCGTTGGAAATATGCTGCC
scaffold432269_14.	1	p2	(CT)6	12	125	136	AGCATCTCTTGCTCTCTCATTG
scaffold432282_16.	1	p2	(AG)6	12	256	267	CCCTTTCTTCCCAAACAACA
scaffold432431_15.	1	p2	(TG)6	12	674	685	GCACCAAGACAATGCCCTAT
scaffold432486_13.	1	p2	(TC)6	12	114	125	CGTGTTTTACCGAAATGCGT
scaffold432512_15.	1	p2	(TA)6	12	73	84	CAATCCTTTGAGTATGGATTGATG
scaffold432518_16.	1	p2	(AT)8	16	1069	1084	CGATCCGTCGACTAAACCAT
scaffold432525_15.	1	p2	(AT)9	18	1201	1218	ATGCGAGAACGGAAAGAAAA
scaffold432570_11.	1	p2	(TA)12	24	399	422	CCTATGCCGCTTACCTTTTT
scaffold432878_15.	1	p2	(TA)8	16	606	621	CCAACTACTGATGGTCCAAGC
scaffold432885_11.	1	p2	(TC)6	12	347	358	CCCACTCCACCTTCTCCTAA
scaffold433106_15.	1	p2	(AG)7	14	1286	1299	TCTAACACGCGAATCTGACG
scaffold433413_12.	1	p2	(AT)6	12	98	109	TCGTGAATGGCATGACTGAT
scaffold433759_18.	1	p2	(TA)7	14	110	123	GAGGTACGGTGATATCATGCTTAGT
scaffold433773_16.	1	p2	(GA)8	16	1965	1980	ATGGGGAGTTCAATGTCGTC
scaffold433954_13.	1	p2	(TA)10	20	333	352	CCACGAAACATCAGCATACG
scaffold433958_17.	1	p2	(TA)8	16	250	265	CATATCTGACGGCCCAGATT
scaffold433975_11.	1	p2	(TA)8	16	533	548	TGGACGCCCAATATTGTTTC
scaffold434190_14.	1	p2	(AG)7	14	212	225	TGTAGAGGAAGTTGGGGGTG
scaffold434340_12.	1	p2	(AC)7	14	164	177	CGGTTAGTCCGTCCATCACT
scaffold434418_15.	1	p2	(AG)7	14	445	458	GGATCGCGTCGAATTAGTGT
scaffold434439_16.	1	p2	(GA)6	12	603	614	ATCAGCATCTTTACCGCCAC
scaffold434554_15.	1	p2	(GA)6	12	1177	1188	CTATGGCACTATGCCGGATT
scaffold434622_11.	1	p2	(TG)7	14	231	244	ACGCACACTTACGCATTCAG
scaffold434730_15.	1	p2	(GA)14	28	198	225	TGCAGCTGAATGAAAAATGTG
scaffold434785_10.	1	p2	(TA)9	18	284	301	AATCATCTCCTCACTAATTCCATC
scaffold434864_12.	1	p2	(TG)7	14	1316	1329	TACGTGTGTGCAGTTGCTGA

scaffold434867_13.4	1	p2	(TA)9	18	120	137	GACCGCAGGATAATCCAGAA
scaffold434869_14.9	1	p2	(TA)6	12	799	810	TTGGACTTTTCGAAGAGCAT
scaffold434870_12.4	1	p2	(TA)6	12	260	271	CCCGATTTCCATGCTTAAAA
scaffold434873_13.3	1	p2	(AT)6	12	561	572	GTCAAGGAAGGCATCCACAT
scaffold434908_14.4	1	p2	(AT)6	12	2259	2270	TTAGCTCCAAGGGCCCTAAT
scaffold435013_10.8	1	p2	(AT)7	14	226	239	CTTCTGTTGGGGCGAAATTA
scaffold435172_13.7	1	p2	(TA)7	14	139	152	CCAATTGACTTCGTGGAGGT
scaffold435260_16.9	1	p2	(TA)9	18	209	226	AGACCATGGTTCGGAGAGTG
scaffold435404_19.1	1	p2	(AC)7	14	2069	2082	TCGCTGATCCCCAATATAGC
scaffold435427_11.9	1	p2	(AT)12	24	410	433	TCACGTTATGGGGAGAATCC
scaffold435448_11.9	1	p2	(AC)7	14	1226	1239	CAACGATGGTTGTTGAAACG
scaffold435474_13.7	1	p2	(AT)9	18	48	65	TGCGTTTTGACAATATATAGGTGTTT
scaffold435490_13.9	1	p2	(AT)8	16	301	316	TTCAGTGGAACATGGTTTGC
scaffold435531_12.7	1	p2	(AT)7	14	346	359	GGTTGCCGCCTTCATTATTA
scaffold435561_14.6	1	p2	(GA)9	18	487	504	GCATGCATCAACTGCAAATC
scaffold435569_10.5	1	p2	(TA)7	14	104	117	CTTTAGGGCCCTTGATTTCC
scaffold435630_11.5	1	p2	(TA)7	14	402	415	GTTGCTTCCATCGTCACCTT
scaffold435741_15.1	1	p2	(TA)9	18	257	274	GCAGAATTTTGGCCTTTGAT
scaffold435941_15.6	1	p2	(TC)6	12	449	460	TCTCGTCTCCCTTGTTTGCT
scaffold435951_12.2	1	p2	(AT)8	16	73	88	GGGTGCTTGAAGATAAACATGA
scaffold436007_12.8	1	p2	(CT)6	12	540	551	CTAAATTCGCATGGGAAGGA
scaffold436135_18.6	1	p2	(TC)7	14	247	260	CGCGAAGAACACAAGAGGAT
scaffold436190_12.7	1	p2	(CT)6	12	343	354	CGCGAACTCAAATACCCTTC
scaffold436372_10.4	1	p2	(AG)6	12	307	318	GTAGCCGTCTATTCCGAACG
scaffold436384_18.6	1	p2	(GA)6	12	832	843	TCCAAACAAGGCTCTATGGC
scaffold436389_14.7	1	p2	(AG)7	14	582	595	ATTGTGTCACCGTCGTCTCA
scaffold436456_16.6	1	p2	(CA)6	12	1746	1757	TTCATGCCCGTTTTATTTGA
scaffold436479_14.3	1	p2	(AT)8	16	205	220	GGATATCCGATCCGGTTTTT
scaffold436550_15.1	1	p2	(CT)7	14	49	62	TTGCTCTCATTGTAATCTCTCTCAA
scaffold436577_14.6	1	p2	(CT)6	12	543	554	ATGCTTAGGTGGATTTCCCA
scaffold436602_14.4	1	p2	(TA)6	12	163	174	CACCATAATTTGACACACACACA
scaffold436607_15.4	1	p2	(TA)6	12	97	108	GCATATGATGTGCATATACGAAA
scaffold436609_11.3	1	p2	(CT)8	16	363	378	GCTTATCCCTTCACCGTTCA
scaffold436611_14.9	1	p2	(CT)6	12	377	388	CGAAATGTGAAGCGTTTGAA
scaffold436645_15.2	1	p2	(TC)7	14	335	348	TTGAACTCACCACGTGAAGG
scaffold436675_15.6	1	p2	(AT)8	16	2927	2942	GGACAAAGGGTTTGCGATAA
scaffold436740_13.1	1	p2	(GA)7	14	328	341	CAGGGGTAAAGTAGTCATTGTCG
scaffold436750_13.4	1	p2	(GA)9	18	391	408	AGCGTACATGGAATCCCAAC
scaffold436786_10.1	1	p2	(AT)10	20	227	246	TCTGTCACGTCATTGTGTCG
scaffold436888_10.5	1	p2	(GA)9	18	71	88	GGTGGTGCGAGAGTGATTGT
scaffold436936_12.3	1	p2	(TC)6	12	99	110	CACTAGCCGCAGCTCTCTCT
scaffold437346_13.0	1	p2	(GA)6	12	174	185	GAGAAATTGGCAATCGGAAA
scaffold437365_11.1	1	p2	(TA)12	24	48	71	CACGTTATATTTATAGTTTTGTGGGG
scaffold437427_17.6	1	p2	(TA)6	12	105	116	CGGTTGTATGAAACCGTTAGTTG
scaffold437595_13.8	1	p2	(AT)6	12	55	66	TCGGAGCTAAAACGGAGAAA
scaffold437597_16.6	1	p2	(AG)6	12	223	234	CTGCTCGAGAGATGCAAGAG
scaffold437761_14.0	1	p2	(AG)6	12	127	138	CCTTCCAACAGAACAAACTCG
scaffold438096_10.4	1	p2	(AT)8	16	332	347	ATGCGCGTTTTTAGGGTTTA
scaffold438109_15.1	1	p2	(GT)7	14	1951	1964	TGCTCTTTGACCAGCTGTTTT
scaffold438126_14.3	1	p2	(TC)7	14	124	137	TCTTACTTGCCTTGACCCTGA
scaffold438230_11.3	1	p2	(AT)6	12	216	227	AAGGGGGTTATCATGAGATCAA
scaffold438314_12.2	1	p2	(AT)8	16	54	69	AACGGTAGCCATCACAATTTTT
scaffold438372_10.8	1	p2	(AT)13	26	137	162	ACGACTGTCGATCCACACAA
scaffold438470_18.2	1	p2	(TG)6	12	2418	2429	TCGAAACAGATCAAAACTGCC
scaffold438640_13.4	1	p2	(TA)7	14	160	173	AGTCCCAACTTGCTCATTTCA
scaffold438733_15.5	1	p2	(CT)12	24	1832	1855	TTCGATCTTCATCGCCTCTT
scaffold438849_12.5	1	p2	(TG)8	16	49	64	AAACTCTGAAAGTTGTCGAGCAG
scaffold438850_14.7	1	p2	(AG)6	12	155	166	AGCGAGTTTCGAAATGGAGA

scaffold438895_12.3	1	p2	(TA)8	16	210	225	TGATAGCGTCATTGTTTGGC
scaffold439017_15.9	1	p2	(TC)6	12	1913	1924	GTTTCTTCTGCCAGCACCTC
scaffold439280_17.6	1	p2	(TG)6	12	2461	2472	TGTTTACGGCTGCAAATTCA
scaffold439316_10.0	1	p2	(GA)6	12	36	47	CGTGGTTCGATCGTAATTGA
scaffold439522_17.6	1	p2	(TA)9	18	441	458	TAAAACCATGCACGTAGGCA
scaffold439577_14.4	1	p2	(TA)10	20	453	472	TGCATCTTTGTACTCAGCCCT
scaffold439758_11.6	1	p2	(AT)8	16	276	291	GCCGAGTGTCACCCATATCT
scaffold439771_15.6	1	p2	(AT)9	18	120	137	TTAAAGCTCGGTTCAATTTCA
scaffold439773_17.7	1	p2	(AT)7	14	124	137	ATTGTTTGTGTGGCCCAGAT
scaffold439903_16.4	1	p2	(TA)8	16	74	89	TTGATCCAAGGGTGTATAATGG
scaffold440161_10.1	1	p2	(TA)9	18	596	613	TCATGACTCCATCCTTCACTTC
scaffold440214_13.3	1	p2	(TA)7	14	74	87	GTTGACAACTTGACATCAAATACAT
scaffold440217_13.5	1	p2	(TC)6	12	112	123	TGACACGTTGGACCTGAAAG
scaffold440247_10.7	1	p2	(AT)9	18	572	589	TGTAGTCAATGCTTTTGAGATTATG
scaffold440356_16.4	1	p2	(TA)6	12	543	554	CGGATTTAATCGTGGCTGTT
scaffold440453_14.8	1	p2	(AT)6	12	719	730	TTCAACCCGAACAACAATGA
scaffold440521_17.1	1	p2	(TC)6	12	127	138	TCTTTCAATTTTCAGCCCTCA
scaffold440572_15.6	1	p2	(CT)8	16	3210	3225	TGCTTGCATTTGAAGTAGCG
scaffold440690_11.1	1	p2	(GA)6	12	262	273	TGGTGTTCTTGGTGTTTTGG
scaffold440894_15.5	1	p2	(TA)7	14	260	273	TCACCTTATCAACGATCCGA
scaffold440943_15.8	1	p2	(AT)6	12	156	167	TGGAAATTTTCGGGGAATAA
scaffold440980_15.8	1	p2	(GA)8	16	480	495	GTGCCTATTGCCGACGTAGT
scaffold441052_18.3	1	p2	(GA)6	12	195	206	CGCTGTAGATCCAACAAGCA
scaffold441171_13.2	1	p2	(TA)8	16	340	355	TTCGATTCGACGCTTTTTG
scaffold441178_10.8	1	p2	(AG)7	14	77	90	CGCCCCATGGAGAAGTTTAT
scaffold441226_15.6	1	p2	(TC)6	12	740	751	CGTGTCATAGGAAAACGAGC
scaffold441269_15.7	1	p2	(AG)7	14	1965	1978	CAGCATGGCCATTACAACAG
scaffold441278_16.0	1	p2	(TA)10	20	531	550	ATAATCGTGGTGATAGGCGG
scaffold441329_20.3	1	p2	(CT)6	12	1044	1055	AAACGAGAAGAAAGCAGCCA
scaffold441540_14.4	1	p2	(TA)8	16	484	499	ATACAAAGTCCCACGACCGA
scaffold441568_14.0	1	p2	(CT)6	12	342	353	ACTTGCTTGACTTGCAACGA
scaffold441570_16.7	1	p2	(CA)8	16	429	444	ATCAACGCTTTGGATGTGCT
scaffold441612_10.5	1	p2	(CT)6	12	362	373	CGCCGAGTTGGTTCTCTCTA
scaffold441635_15.9	1	p2	(TC)7	14	3918	3931	CCAAGCTTGCCTCTTCATCT
scaffold441796_11.6	1	p2	(AT)9	18	737	754	TTCAGGATAAATATCAGTTTCATGG
scaffold441928_14.7	1	p2	(AT)6	12	127	138	TGCAATCACACAATAACAAGCA
scaffold441941_10.7	1	p2	(AT)9	18	345	362	CAACGCTAATATCAACCGCA
scaffold441967_15.9	1	p2	(AT)6	12	71	82	ATCCGGAGTAAGTTCGAGGA
scaffold441970_14.1	1	p2	(GA)6	12	341	352	TTCCAAACCTTTGTTCCGAG
scaffold441990_12.1	1	p2	(GA)6	12	605	616	CGATCCTTGAGTAAAAGCCG
scaffold441993_17.0	1	p2	(AT)7	14	2201	2214	CCGAAACCGTGTTCTTCAGT
scaffold442403_13.0	1	p2	(GC)6	12	432	443	CAACGGCTCTTTTCTCCAAC
scaffold442518_15.6	1	p2	(GA)7	14	411	424	GCATTGTTTTTGTCGGCTTT
scaffold442531_14.5	1	p2	(AT)6	12	503	514	CTGGGGGTTAGGTTGGTTTT
scaffold442532_10.2	1	p2	(TC)6	12	128	139	AGAGTTGGGCTAGGTGGGAT
scaffold442534_16.2	1	p2	(TC)6	12	1316	1327	CCTTTCTTGAGTTTCAACCCC
scaffold442614_18.0	1	p2	(CT)6	12	1502	1513	ATTCACAAGCGGCACTTCTT
scaffold442704_17.9	1	p2	(AG)9	18	2441	2458	CTGAGGCAGTTTTCAGAGCC
scaffold442736_14.7	1	p2	(CT)8	16	351	366	TGGCATAGTTTGGTTGTGGA
scaffold442746_16.0	1	p2	(TC)6	12	1371	1382	TGGCCGAAGTAATTGAAAAA
scaffold442753_14.8	1	p2	(GT)9	18	565	582	ACATACCACCGATTTTTCCG
scaffold442792_12.3	1	p2	(AC)6	12	1279	1290	TGCAAGGAAGAAAAACAAACAA
scaffold442903_13.4	1	p2	(CT)7	14	449	462	ATGGGACGGAGGGAGTATTT
scaffold443037_15.9	1	p2	(AG)8	16	73	88	TCCCATGTTGTAATTAATGTAAGGAA
scaffold443178_16.6	1	p2	(TC)7	14	71	84	TGAGAATGGCCGAGAGTTTT
scaffold443386_16.4	1	p2	(CA)6	12	4710	4721	TGCAAACATCAGCCATTTTC
scaffold443489_16.1	1	p2	(GA)7	14	4672	4685	TGGGAAGAGGAAATGGACAG
scaffold443506_14.0	1	p2	(AG)7	14	73	86	GAATCAGGGGAGGGAGAGAG

scaffold443552_15.8	1	p2	(AT)9	18	2185	2202	TTCATACACACCCTTGCAGC
scaffold443560_12.6	1	p2	(TA)7	14	415	428	TCTCCAGGAGTCGTGCTTGT
scaffold443562_16.8	1	p2	(AT)6	12	45	56	TTTGGTCCCTCAACAAATAATAAA
scaffold443611_12.9	1	p2	(AT)9	18	379	396	TTCCTGTGCTTTCAATTTGG
scaffold443731_12.1	1	p2	(TA)12	24	1033	1056	AGTCCCTGATTCTTCCCTCG
scaffold443754_12.2	1	p2	(AG)12	24	52	75	GCTTCAGCCATCGAGTTTTC
scaffold443838_13.9	1	p2	(AG)6	12	53	64	AGCTTCCGAACTTATGGGCT
scaffold443929_16.8	1	p2	(CT)6	12	1591	1602	CGAAAAATGGTGGGAAAGAA
scaffold443950_11.2	1	p2	(CA)6	12	226	237	AGTTTCATTTCCGGCTTCCT
scaffold444027_11.7	1	p2	(TA)10	20	158	177	TGTAGTCCGCTTTGCTGATG
scaffold444098_16.9	1	p2	(CT)11	22	442	463	AGCATAATAACCACGCAGCC
scaffold444228_12.2	1	p2	(TA)6	12	868	879	ACGAACACTCACGCACTCAC
scaffold444229_14.3	1	p2	(AC)6	12	9157	9168	TGGTGACCAGATCAGGGAAT
scaffold444285_19.0	1	p2	(CT)6	12	85	96	CGAGGAACTTGGGACGATT
scaffold444499_15.2	1	p2	(TA)6	12	5587	5598	CTCCTGCCTCGTAGCAGTTC
scaffold444506_13.9	1	p2	(AT)8	16	226	241	GACAACAGAAAAACCACGCA
scaffold444525_16.0	1	p2	(CT)7	14	777	790	TGGCAGGTTGGAGACACATA
scaffold444563_15.4	1	p2	(TA)7	14	1269	1282	TGCCTGCCCCTACTAGAAAA
scaffold444568_15.4	1	p2	(AG)6	12	54	65	TGTGCACTGTTTCTCTCTCTCA
scaffold444683_15.4	1	p2	(AT)10	20	4189	4208	GTCGGTGGCTGAAAGAAAAC
scaffold444818_16.8	1	p2	(GA)6	12	128	139	TGATTCAGATGTCAGCGAGC
scaffold444820_16.6	1	p2	(CA)6	12	57	68	TTTGATGTCCAAAGTGGCTG
scaffold444989_10.0	1	p2	(AT)7	14	84	97	AGTCGATTGTGTAGTTGTGGG
scaffold445019_13.6	1	p2	(TA)6	12	713	724	TGGTTCTAAATTCATGTTTCTTGG
scaffold445118_14.3	1	p2	(CT)8	16	1344	1359	TGTGAGAGGTGTGAAGCAGG
scaffold445135_16.3	1	p2	(CT)6	12	260	271	CTCTCTTGCCTTCCTCCTCC
scaffold445177_12.7	1	p2	(TC)6	12	590	601	ACCCTCATCCCTCAATTTCC
scaffold445375_16.1	1	p2	(TG)7	14	2343	2356	AAGAAACGGAGGGTGTTGTG
scaffold445519_14.4	1	p2	(CT)6	12	873	884	TCTCTTTGCAAAACCCTCGT
scaffold445535_13.6	1	p2	(TA)10	20	45	64	TCACGTTAAAGGAGGGTATCA
scaffold445544_14.3	1	p2	(TG)6	12	1289	1300	AATTTCACTCTAGCGGGGCT
scaffold445546_11.6	1	p2	(TA)10	20	407	426	GGCGCAGCATATATACACGA
scaffold445673_16.7	1	p2	(CT)6	12	220	231	AATTGCCATCCCTCACACTC
scaffold445695_12.1	1	p2	(TA)9	18	1985	2002	TCGATCGGCCTATTTTTCTG
scaffold445706_16.3	1	p2	(AT)6	12	439	450	TCGACTCCATAGTAGCCGAGT
scaffold445707_16.7	1	p2	(CT)7	14	479	492	TTCCATCCTTTCTTCCATGC
scaffold445864_13.5	1	p2	(AT)6	12	384	395	ATTTTGGTCGAAACCGAATG
scaffold445930_15.0	1	p2	(AG)9	18	521	538	CCCCTGTCCTCGACAGAAT
scaffold446002_16.0	1	p2	(TA)7	14	1054	1067	TTGGCAGGTTGATCATAAATTG
scaffold446039_16.1	1	p2	(AT)8	16	7721	7736	TGATTCTAGGATATCGCGGC
scaffold446141_14.1	1	p2	(AT)7	14	323	336	GCTGATCTTGGTCAGGCTCT
scaffold446386_15.6	1	p2	(AT)9	18	54	71	CCCTTTTATGCTCACATCTCTTTT
scaffold446522_14.0	1	p2	(TG)7	14	770	783	TTACGTGCCTGGCTTAGGTT
scaffold446803_18.5	1	p2	(AG)9	18	1155	1172	TTATTGCGTCACCTCATCCA
scaffold446921_14.0	1	p2	(AT)6	12	129	140	ATTTTAACATTGTTGGCGGC
scaffold446922_14.7	1	p2	(TA)9	18	1509	1526	AAACCGTGTTTTTACTGGCG
scaffold446976_17.2	1	p2	(CT)6	12	1075	1086	GGAAATGGCGTGAACTGTTT
scaffold447069_15.5	1	p2	(GA)6	12	122	133	CGATTTGCAAGTCTATGGCA
scaffold447084_13.9	1	p2	(TA)7	14	51	64	CATTGGTTAGCACTTGAATGAGA
scaffold447203_11.8	1	p2	(AT)6	12	143	154	TGCACCATAACAATATTCGACA
scaffold447313_15.5	1	p2	(CT)9	18	1421	1438	CGAAACGAGTTGCTGCTGTA
scaffold447412_14.6	1	p2	(AG)7	14	1253	1266	AACACACAGCCGCTACTCCT
scaffold447449_12.9	1	p2	(CG)7	14	48	61	ATAGTGGTATGGTGTGGGGG
scaffold447606_14.0	1	p2	(TC)8	16	790	805	GCCCTCCTTGATACCACAAA
scaffold447776_13.6	1	p2	(GA)8	16	444	459	AAAGGACCCGACCTTTTCAT
scaffold447938_15.0	1	p2	(CT)6	12	789	800	TCCTTGATGAACTGTGGGTG
scaffold448015_12.6	1	p2	(TA)9	18	587	604	ACGACGTGCATCATTTCTCA
scaffold448164_17.3	1	p2	(CA)7	14	1073	1086	CATTTCAGGCCAAATCCTGT

scaffold448181_12.9	1	p2	(CT)6	12	558	569	CCTTCTTCTTGGCGTCAACT
scaffold448215_16.8	1	p2	(AT)6	12	136	147	TCTACGGAGGACGAGCTGTT
scaffold448332_16.5	1	p2	(GA)6	12	353	364	CTCGACTCAGGGACCTTCAC
scaffold448399_14.5	1	p2	(AT)9	18	1370	1387	GCGCCACATCTTGTTGAATA
scaffold448405_10.1	1	p2	(AT)6	12	148	159	TGTGATCAGGTGCTGTGTGA
scaffold448413_13.6	1	p2	(TA)6	12	657	668	TTCTCAGCATATGGGAAGGG
scaffold448444_10.4	1	p2	(GT)7	14	409	422	ATGCATGTGTGCAGGTGTTT
scaffold448513_17.2	1	p2	(CT)9	18	545	562	TCGTTGACCGTATGGGATTT
scaffold448672_13.2	1	p2	(CT)10	20	220	239	CAAACAAAGTCTGCAACCCC
scaffold448718_11.8	1	p2	(AT)10	20	732	751	TGTTTTGCGAGCTTTTATTGG
scaffold448783_10.4	1	p2	(TA)8	16	48	63	CGGATTTAAGGGTTATTACCTGAT
scaffold448805_17.2	1	p2	(AT)6	12	1114	1125	CCTGCCCTCCAAGATCATAA
scaffold448840_10.5	1	p2	(TA)7	14	51	64	ATTTCTCACGCTGAAATGCC
scaffold449111_14.6	1	p2	(CT)6	12	54	65	CAATGGCCAAGAACGTAACT
scaffold449133_13.0	1	p2	(AG)7	14	1268	1281	TTTCCTTTTGCAGCTGGAGT
scaffold449178_14.0	1	p2	(TA)13	26	39	64	CCAATTTGAATGGTTATCACCTG
scaffold449266_18.2	1	p2	(AG)8	16	348	363	GCACAATCAAACCTTGCCTT
scaffold449471_10.3	1	p2	(TA)6	12	76	87	AACAATTGCACCTTTAATAGCAA
scaffold449525_10.9	1	p2	(CT)6	12	60	71	AATGTCTCTTCCTCCTCGCA
scaffold449528_15.1	1	p2	(CT)6	12	284	295	GCACCGGAGTATAACCCAGA
scaffold449560_13.0	1	p2	(AG)6	12	2777	2788	GCTTTTATCAATGCCCTCCA
scaffold449704_11.1	1	p2	(TG)9	18	47	64	GTTATCACCGGATCGCAAC
scaffold449710_15.4	1	p2	(TA)7	14	942	955	TGTTTATTTGAAGGGGCAGG
scaffold449770_14.9	1	p2	(AT)7	14	6969	6982	TGGCGGCATTCTATATGACA
scaffold449968_14.6	1	p2	(TA)7	14	3281	3294	TTGATGTTTTCCCCATTTTTG
scaffold450103_19.2	1	p2	(AG)9	18	414	431	CCCATCTTCTGCAAAAATCC
scaffold450162_14.1	1	p2	(CA)6	12	1085	1096	TATTTTTGGGTTTGGGGTCA
scaffold450194_16.9	1	p2	(GA)6	12	1190	1201	GCACCCTTTTGGTGGTAGAA
scaffold450199_14.2	1	p2	(TA)8	16	178	193	TGTGTCATTCCTTCCACTGC
scaffold450227_14.0	1	p2	(AT)13	26	555	580	TCATTTAATATGGTCTGTGCGAA
scaffold450238_10.1	1	p2	(TA)8	16	209	224	TTAGGGTTTAAAATCGCGCA
scaffold450239_15.9	1	p2	(AG)7	14	1417	1430	TGGGTTAGCCCAAAAATGAG
scaffold450311_15.7	1	p2	(TA)6	12	153	164	TTGTATTAGATTGTGGCTCCG
scaffold450336_10.0	1	p2	(CT)7	14	55	68	CACTCACTCTCACTAGCCGC
scaffold450354_14.8	1	p2	(TG)6	12	1014	1025	AAGCAATGGTGGTCTTCTCG
scaffold450522_14.9	1	p2	(CT)7	14	154	167	CACCATCGTTCCTCACACAC
scaffold450655_14.2	1	p2	(AT)9	18	521	538	TCTGCCTGTCGATCTCACAC
scaffold450701_14.2	1	p2	(AT)8	16	528	543	GCATTGGTAAAACCGGGATA
scaffold450732_16.7	1	p2	(TG)7	14	65	78	TGGTTATTACCTGAATACTCCCA
scaffold450777_10.3	1	p2	(AG)6	12	130	141	TGGTCGTCCAAATCTCCTTC
scaffold450909_12.7	1	p2	(AT)8	16	257	272	GGATATAACCACCTCACCCAAA
scaffold450970_10.2	1	p2	(TC)6	12	295	306	ATCGTAGTTGATGCCCTTCG
scaffold451112_15.4	1	p2	(TG)7	14	127	140	TGTTGTCGATCTGGCACTCT
scaffold451162_11.8	1	p2	(AT)8	16	50	65	ACCCAGATCGCACTCGAATA
scaffold451184_11.5	1	p2	(AT)8	16	161	176	AGTCATTCGGGCCACATAAT
scaffold451291_10.7	1	p2	(CT)6	12	331	342	TGAGCACGTACAGATAAAAGGG
scaffold451480_13.0	1	p2	(CT)6	12	994	1005	CGACACCACTCCACCTTTTT
scaffold451526_12.7	1	p2	(TA)9	18	123	140	GTGGCAGCATATCAACCCTT
scaffold451527_11.6	1	p2	(AT)8	16	126	141	CGTGGCAGGCTAATATCCAT
scaffold451531_12.9	1	p2	(TA)10	20	48	67	ATTGTTCGATGTCAGCTTCG
scaffold451545_11.3	1	p2	(CT)7	14	1271	1284	ATCGACATTCTTCTGCAGGC
scaffold451554_16.0	1	p2	(AG)8	16	126	141	CTCATTATTTCTCACTCACCAAGA
scaffold451758_11.3	1	p2	(GT)6	12	116	127	TTCTGCTCTCTCTCGGTGGT
scaffold451814_14.8	1	p2	(TA)9	18	1035	1052	ACTTAGGACCAAAGCCCGAT
scaffold451830_14.4	1	p2	(AG)6	12	859	870	TGGATTTTAGGGCTCATGCT
scaffold451861_14.9	1	p2	(TC)13	26	3204	3229	CCAGTAAAGGACCATTCGGA
scaffold451894_13.3	1	p2	(TA)9	18	1493	1510	CAGTTTCACCAAGTCACCCA
scaffold451914_11.3	1	p2	(TA)8	16	79	94	AAAATCAAAGGCCCACAATG

scaffold451938_13.8	1	p2	(CT)7	14	279	292	CCGTGTCCAGTCTGTGTTTG
scaffold451963_17.0	1	p2	(TG)6	12	476	487	AATTACACAATCCTCGTGGGA
scaffold451998_15.0	1	p2	(AT)11	22	1265	1286	GCCTAATGTCATTGCCTGCT
scaffold452414_11.9	1	p2	(TC)6	12	556	567	GGACCCACATGGAAGAGAGA
scaffold452548_17.2	1	p2	(GA)7	14	153	166	CTCCCTCCGTCCCATAAAAT
scaffold452601_11.5	1	p2	(AT)6	12	179	190	ATTGTTGGGTATTGTTGGCG
scaffold452701_12.2	1	p2	(TA)6	12	998	1009	GGGGAAGAGAAGAGTGAGGC
scaffold452897_19.5	1	p2	(AT)6	12	57	68	CGGATTTATTGGTTATTACATGATT
scaffold452900_14.9	1	p2	(AG)6	12	82	93	CTTCGGAACTCTATCCGCC
scaffold452909_15.2	1	p2	(TC)7	14	128	141	GCAGATCCAGATGCTCCAAT
scaffold452911_12.4	1	p2	(AG)7	14	267	280	GCAACGTGCCAAACTTCATA
scaffold452914_15.4	1	p2	(CT)6	12	1350	1361	TCTTGCCTTCGTTCCAAGTT
scaffold452934_10.7	1	p2	(TA)6	12	51	62	ATCCTAAGTTCGCCCCTGAT
scaffold452953_14.9	1	p2	(TA)9	18	108	125	CGAAATTGAATGGCAAGGAT
scaffold453079_14.3	1	p2	(AT)6	12	600	611	GCAGGTTAACTCTAATTAATCCGTACA
scaffold453153_14.5	1	p2	(TA)6	12	999	1010	CTTCCCCCACATTCAAAAGA
scaffold453157_16.6	1	p2	(GA)6	12	1128	1139	TGAAGAGAAATGGCAATGGA
scaffold453247_11.2	1	p2	(TA)6	12	461	472	CCCCGGTTTCATTTATAGCA
scaffold453268_15.3	1	p2	(CA)6	12	122	133	CCGGTGAACTTCTCCAACTC
scaffold453311_14.3	1	p2	(TA)6	12	524	535	ATGAGTTATCACCGGATCGC
scaffold453467_11.4	1	p2	(AG)8	16	75	90	CCCATGTTGTAATTAATGTAGGGA
scaffold453518_13.7	1	p2	(TA)7	14	173	186	CCGTGCATCCTATCCTGTTT
scaffold453528_13.0	1	p2	(AT)9	18	261	278	TCAATCGCAACCATCAATTT
scaffold453594_16.0	1	p2	(TA)7	14	304	317	GGCCGATATTTGCTCGTAGT
scaffold453666_12.7	1	p2	(AG)6	12	261	272	AATGGTGAGAGTGGAATGGG
scaffold453672_12.7	1	p2	(TA)8	16	220	235	AAGATCTACCTTTGGGTAACCG
scaffold453704_14.5	1	p2	(AT)7	14	276	289	TTTTCCCGCAATAACCACTC
scaffold453751_14.0	1	p2	(TC)6	12	273	284	ACAGAACAAGCATACGCACG
scaffold453822_15.6	1	p2	(TC)9	18	2818	2835	TCGTGCACTGGTGTTCTCTC
scaffold453921_15.4	1	p2	(TA)8	16	288	303	GTGACCCTTTGTGGGAAGAA
scaffold453926_15.3	1	p2	(TA)6	12	281	292	GGACTGGTTTGACACCTTTGA
scaffold453957_10.5	1	p2	(AT)6	12	275	286	CCCATTACTCCAAACCCAAA
scaffold454024_13.8	1	p2	(GA)6	12	2383	2394	ACGATCCATGAAAGCAAACA
scaffold454039_15.4	1	p2	(AT)6	12	77	88	TGCTCAAACTTGAACTTGAATAAA
scaffold454052_12.0	1	p2	(GT)8	16	256	271	ATGCCTTGGTCTTCCTCATC
scaffold454063_15.0	1	p2	(TC)6	12	170	181	TGATGAATGAAAGATGAAATGGA
scaffold454095_12.0	1	p2	(AT)7	14	383	396	TCTTTCACCCGCTTTAGGTG
scaffold454144_10.7	1	p2	(AT)9	18	257	274	GGAGAAGAGGGAGGATCGAG
scaffold454300_11.2	1	p2	(AT)8	16	169	184	ACCGATTGCTGAGCATTTCT
scaffold454347_15.2	1	p2	(TC)8	16	492	507	TGGAAAACCAAAGCAAAACA
scaffold454399_14.4	1	p2	(CT)8	16	354	369	CCAATGAAGCTCGTCAGGTT
scaffold454403_11.2	1	p2	(TA)7	14	491	504	CGAGCTGGCTTGAGCTACTT
scaffold454421_18.0	1	p2	(CT)6	12	502	513	AATACACCCCTGTCGCACTC
scaffold454491_12.9	1	p2	(AT)6	12	353	364	TAATGGGTGTTTGTGCGAAG
scaffold454620_15.8	1	p2	(GA)6	12	369	380	ACCGTTGCAGTAAACCTTGG
scaffold454627_14.7	1	p2	(AT)7	14	4420	4433	TGGGGTAAATATTTTGGGAGG
scaffold454637_16.8	1	p2	(TA)8	16	2708	2723	GCGGGACAACTACCAACAGT
scaffold454692_17.0	1	p2	(GA)8	16	2446	2461	GCTTCCCCCTAGTGAACACA
scaffold454816_18.6	1	p2	(TA)6	12	130	141	GGGCAATGTCTTATGGCCT
scaffold454869_10.8	1	p2	(TA)9	18	577	594	TTAGGGTTTAAAATCGCGCA
scaffold454955_14.5	1	p2	(TA)6	12	815	826	CCTCAATGGTATGCTCGGAT
scaffold455058_16.4	1	p2	(TC)6	12	33	44	GCAACGGAATACCCCTCTTT
scaffold455230_19.8	1	p2	(GA)8	16	1554	1569	GGTGCTCCATGTTTGTGAGA
scaffold455253_17.8	1	p2	(AT)6	12	418	429	GCTCAAAAGGTGATGGGTGT
scaffold455261_18.1	1	p2	(AT)8	16	332	347	CCAAAATTCAGTAAGGGTAACAAAA
scaffold455299_12.8	1	p2	(TA)8	16	68	83	TTATCACTTGATCATCCCCC
scaffold455343_10.9	1	p2	(AG)8	16	86	101	GAAGTTCGGCCATTCTTACG
scaffold455446_13.7	1	p2	(AT)6	12	775	786	TTGGTGGATTTTTGGAATGTC

scaffold455593_15.3	1 p2	(AT)7	14	427	440	CTGCAACCATAGCTCGATCA
scaffold455606_16.2	1 p2	(GA)6	12	165	176	TTGGGCTTTGATTCTTGCTT
scaffold455616_14.9	1 p2	(TC)6	12	52	63	CAGAAATTGCCTTGGTTGTTC
scaffold455678_15.0	1 p2	(AG)6	12	247	258	GCCGAGGTCACACCATACTT
scaffold455917_16.4	1 p2	(AG)7	14	129	142	CCTCGGATAAATTATGGGAAAA
scaffold455958_13.4	1 p2	(AG)7	14	78	91	TGTGCAAGGTTGAACCAAGA
scaffold455975_18.4	1 p2	(TA)6	12	130	141	TCTTGCCATTGATTTTGGTG
scaffold455976_15.4	1 p2	(AT)9	18	125	142	CGTTTTTGTGAATGTTGCCA
scaffold455977_14.3	1 p2	(GA)9	18	687	704	AAGGTGTAGATCTGGCGGTG
scaffold456219_13.9	1 p2	(GA)6	12	130	141	TTTGAGAATGGGGAGGTGAG
scaffold456238_14.9	1 p2	(TA)10	20	703	722	AACAGTTTCAATTCGCCACC
scaffold456257_10.1	1 p2	(TA)8	16	421	436	CACTCAAGGGGCATTTTTGT
scaffold456278_16.2	1 p2	(TC)8	16	347	362	GAGTTCGTCAAACCTCCCAA
scaffold456322_11.1	1 p2	(TA)8	16	52	67	TTTGTTCCTACTCACTCACATTAAGA
scaffold456326_10.1	1 p2	(GA)6	12	53	64	GAGGAGAGTTGATGGTGAGGA
scaffold456540_11.1	1 p2	(TA)11	22	225	246	GCAGGGGTTGCACAATATG
scaffold456619_11.4	1 p2	(TA)6	12	212	223	TTAGGTTGGACCAAAAAGCC
scaffold456821_18.0	1 p2	(CT)6	12	535	546	TACTCCGTGGTGTTCCCAGT
scaffold456822_19.7	1 p2	(AT)6	12	529	540	ACAATGGAGGCTGGAGGAG
scaffold456897_12.0	1 p2	(AG)6	12	130	141	TGAGAAATGCTCCAAAGCCT
scaffold456906_14.5	1 p2	(AT)8	16	370	385	CTTATTTGCATTTGGGCCAC
scaffold456934_10.3	1 p2	(TA)6	12	158	169	ACCGGATACCGAAGGGATAG
scaffold457006_16.5	1 p2	(TC)6	12	389	400	GTTGTCACGGCGTGTGATAG
scaffold457007_17.7	1 p2	(TA)6	12	490	501	TTGACCAAATTAGCCCCAAC
scaffold457127_13.2	1 p2	(AG)11	22	234	255	TTGAGTGGTTTGGTTGGAGA
scaffold457186_12.8	1 p2	(TA)9	18	76	93	TTCCTCAATTTTCGAATTCTCC
scaffold457194_17.1	1 p2	(TA)6	12	578	589	TGTGCCATTGATTTCTGAGG
scaffold457267_15.6	1 p2	(GA)6	12	2005	2016	AGGGAGAATCTATGGCAGCA
scaffold457577_20.2	1 p2	(AG)6	12	131	142	GAATGCTTAATGGGAAGCCA
scaffold457579_15.6	1 p2	(CT)8	16	256	271	TCAAGTTGAGCCTCCAAATCT
scaffold457838_19.3	1 p2	(TA)7	14	209	222	TATGGGAAGGACATTTTCGG
scaffold458004_10.4	1 p2	(AT)6	12	264	275	TCCCTTTATGATCATTCGGC
scaffold458129_11.8	1 p2	(TA)6	12	366	377	AATGTGATGTGGGTTGGGTT
scaffold458162_18.3	1 p2	(CT)8	16	323	338	GGAGGTGCAATGCGATATTT
scaffold458203_17.5	1 p2	(TC)6	12	2693	2704	CAAGCAAGCATTTCCTCCTC
scaffold458350_18.3	1 p2	(AG)6	12	524	535	TCACCTTTCTTCATGGCTCC
scaffold458514_12.4	1 p2	(TC)7	14	332	345	CGTTGTGCTTCTTTTCTCCC
scaffold458561_13.9	1 p2	(TC)7	14	460	473	TGGTTTAAACAAATCGTGTTCG
scaffold458611_17.8	1 p2	(GA)6	12	2835	2846	AAGAGGCTACGGTGAGGTGA
scaffold458616_13.8	1 p2	(AT)7	14	283	296	AGGTTGCTCTAAGGTGCCAA
scaffold458646_13.9	1 p2	(TA)8	16	48	63	TGGTTATTACTGGAACGCGA
scaffold458647_12.1	1 p2	(AT)11	22	47	68	GGGGTTATCACTGGATCACG
scaffold458667_15.4	1 p2	(CT)6	12	2297	2308	AAACAATCCTCCAATTCCCC
scaffold458714_12.7	1 p2	(AT)12	24	207	230	TGGGAAGAGTGGGTATGAGG
scaffold458731_11.3	1 p2	(AT)7	14	235	248	CGAACGACAATGGTTTGAGA
scaffold458741_16.4	1 p2	(AG)6	12	437	448	ACGAACATCACCATTCCCTC
scaffold458743_14.5	1 p2	(AT)7	14	499	512	GCAGATTCCCAAATCCAAAA
scaffold458771_17.9	1 p2	(CT)6	12	205	216	TCACGCATTCTCTCTTCGAC
scaffold458798_19.9	1 p2	(CT)9	18	804	821	AAGTTATTGCGAAACCCACG
scaffold458839_16.5	1 p2	(TG)7	14	246	259	TGATGCACGATGTATGTCGAT
scaffold458934_16.0	1 p2	(AG)6	12	3090	3101	GGAGAGAAAAGGGAAATCGG
scaffold458952_10.2	1 p2	(AT)6	12	239	250	AACACCCTGTGTATCCCAGC
scaffold459041_14.2	1 p2	(TA)12	24	493	516	TGGTTGGGCATTCCATTATT
scaffold459115_15.1	1 p2	(TA)6	12	1143	1154	AAACTCATATTCCATCCAAAGGTT
scaffold459175_17.2	1 p2	(CT)6	12	56	67	CAATATTACAAATGGCCAAGAGA
scaffold459181_12.8	1 p2	(AC)6	12	431	442	AGTGCTTTGGAAAACTCATGC
scaffold459406_16.3	1 p2	(CT)6	12	239	250	ATGGGAAGAAAACGCAACAC
scaffold459449_15.6	1 p2	(AG)6	12	2389	2400	CATTTGAGACGGACACATGC

scaffold459494_16.[illegible]	1	p2	(GA)10	20	192	211	CGTGGTTCGATCGTAATTGA
scaffold459505_14.[illegible]	1	p2	(AT)6	12	264	275	TCACATGAATGGACCCCTATC
scaffold459518_15.[illegible]	1	p2	(CT)7	14	107	120	GGGATTTGGGACGATTTTCT
scaffold459519_14.[illegible]	1	p2	(GA)6	12	2362	2373	GTGTGAAAGCCGAGAGAAGG
scaffold459565_12.[illegible]	1	p2	(AT)6	12	415	426	GCCTCAATAACATGAGGCGT
scaffold459568_13.[illegible]	1	p2	(TC)7	14	1468	1481	TTCGAGATGATGGTTGGTGA
scaffold459607_10.[illegible]	1	p2	(TA)11	22	498	519	TAACAATCGCAATGCCAAGA
scaffold459624_12.[illegible]	1	p2	(AT)7	14	196	209	CATCGATCAGTTATCATGGGA
scaffold459641_16.[illegible]	1	p2	(AT)6	12	79	90	CAACCGAGTACCTAACCACAA
scaffold459669_15.[illegible]	1	p2	(CT)6	12	744	755	AGGATAGGGAATAGCGTCCC
scaffold459741_11.[illegible]	1	p2	(TA)12	24	437	460	ATGAAACAGAGAGCCCCCTT
scaffold459753_10.[illegible]	1	p2	(GC)7	14	225	238	TATGGCAGGTGTCGGTATCA
scaffold459779_14.[illegible]	1	p2	(TA)6	12	667	678	TTGAACTTGAAAGCCCAACA
scaffold459885_16.[illegible]	1	p2	(TC)12	24	3463	3486	CCTTCTCCATCAACTGTGGG
scaffold460109_17.[illegible]	1	p2	(TC)6	12	123	134	TCCATTTTCAGGTCCTCACC
scaffold460190_14.[illegible]	1	p2	(TA)9	18	418	435	TAGCCGATAAAAATGGCACC
scaffold460257_13.[illegible]	1	p2	(CT)6	12	229	240	CTCACCCTTGTCATCACCCT
scaffold460381_13.[illegible]	1	p2	(GA)8	16	371	386	AGTATGCAACATGCCACCAA
scaffold460386_15.[illegible]	1	p2	(TA)10	20	123	142	TATGCAGTGGGTTTTTGGTG
scaffold460387_11.[illegible]	1	p2	(AT)8	16	128	143	TAGGACCACATTTTGTCGCC
scaffold460389_15.[illegible]	1	p2	(CT)13	26	1113	1138	CAGATGGTGGTGTACGGTTG
scaffold460542_15.[illegible]	1	p2	(CT)6	12	573	584	GCAGTGAGACCGAGTTGACA
scaffold460583_15.[illegible]	1	p2	(CT)9	18	947	964	ACAATGACCGTTGCAAATGA
scaffold460635_14.[illegible]	1	p2	(CT)6	12	562	573	TGCAAATCGTTACCAACCAA
scaffold460662_17.[illegible]	1	p2	(TA)9	18	408	425	GCCAACCTTCAATCAACACA
scaffold460663_14.[illegible]	1	p2	(TA)8	16	399	414	TCAGAACCGTTGTCTTTCAGC
scaffold460731_17.[illegible]	1	p2	(TA)7	14	173	186	TGATCCCTAAATTGCCCTTC
scaffold460752_10.[illegible]	1	p2	(CT)6	12	131	142	GCCTATCTCTCTTGCTGAAATTG
scaffold460764_12.[illegible]	1	p2	(TA)10	20	44	63	GGCTGATATCACCATAACGCA
scaffold460802_15.[illegible]	1	p2	(TA)6	12	54	65	TCACACACGTTTATTCGAGACA
scaffold460841_11.[illegible]	1	p2	(GC)7	14	113	126	ATATTCTCGCTTCTTCGCCA
scaffold460850_16.[illegible]	1	p2	(AG)7	14	3737	3750	ATCGGAAGGAGCCTTGTTCT
scaffold460927_13.[illegible]	1	p2	(TC)8	16	1248	1263	GCCATTATGCACTTCCCAAT
scaffold460934_19.[illegible]	1	p2	(AG)7	14	726	739	ATGTGGAATGAGGGTGAAGG
scaffold460950_13.[illegible]	1	p2	(TA)7	14	779	792	CATCGTATGTTGCTTGGGAA
scaffold460959_15.[illegible]	1	p2	(AG)7	14	897	910	TTCTGTGATCGCAAAAGGTG
scaffold460968_10.[illegible]	1	p2	(AT)11	22	47	68	TGACAAATAAATGAAATACAAGACA[illegible]
scaffold461007_15.[illegible]	1	p2	(CT)6	12	132	143	CTTTCATGGGTGATGGAAGC
scaffold461013_16.[illegible]	1	p2	(AG)6	12	2904	2915	GACGCTTCCAGAAGTATCGC
scaffold461019_13.[illegible]	1	p2	(TC)8	16	49	64	TCGGTTGACTCTTTTACTTTAGCC
scaffold461125_14.[illegible]	1	p2	(TA)6	12	363	374	CAATTGGGCCTTTCGTAAAT
scaffold461194_13.[illegible]	1	p2	(AT)7	14	51	64	GATCTAGGAGAAAAGTGACATTCTTG
scaffold461260_16.[illegible]	1	p2	(TG)7	14	945	958	CTGCACGTTACAACACCGAC
scaffold461366_14.[illegible]	1	p2	(TC)6	12	744	755	CTTGCCGAATGTTTGTCTCC
scaffold461445_14.[illegible]	1	p2	(AT)8	16	957	972	CATGGGGAATATGCATCCAC
scaffold461458_17.[illegible]	1	p2	(AT)6	12	370	381	GCTTGGTCCGAAAATTTCAA
scaffold461508_16.[illegible]	1	p2	(AT)6	12	252	263	CGTTTCGCGTGCTAATTTCT
scaffold461571_13.[illegible]	1	p2	(AT)7	14	136	149	CGTCGCGTTGGATTAATTTT
scaffold461601_16.[illegible]	1	p2	(AG)8	16	609	624	TGCTTGTGGGTTTTGTTTTG
scaffold461692_14.[illegible]	1	p2	(GA)6	12	248	259	TGCACTGATGAATGATGCTG
scaffold461694_14.[illegible]	1	p2	(GA)8	16	124	139	CAGAGGAGATGGGAATGGAA
scaffold461882_13.[illegible]	1	p2	(AT)8	16	504	519	GGACAAGCTGTCGGATAAGC
scaffold461924_15.[illegible]	1	p2	(AG)9	18	4523	4540	TACATGCCACATGTTGGGTC
scaffold461947_16.[illegible]	1	p2	(AG)7	14	851	864	TCCGATGATCAAGTCAGTGC
scaffold461953_11.[illegible]	1	p2	(CT)7	14	129	142	CCATCAAAAATTCCTCCCCT
scaffold461976_15.[illegible]	1	p2	(TA)7	14	130	143	CAACGGACGCTATCGGTTAT
scaffold462053_14.[illegible]	1	p2	(GA)6	12	94	105	CATCTCAATCCCGTCACCTT
scaffold462111_10.[illegible]	1	p2	(CT)7	14	277	290	GGGGGATGCCACTGTAGATA

scaffold462145_13.5	1	p2	(AT)8	16	438	453	GGAGAATTCGAGAGATTGCG
scaffold462246_18.1	1	p2	(AG)7	14	371	384	TTCGTACAGCCGTTTGATGA
scaffold462254_16.3	1	p2	(TG)7	14	187	200	TAGATGCACCGGTTAGGAGG
scaffold462301_17.9	1	p2	(AG)6	12	671	682	AGTTCTCCAACATCATCGCC
scaffold462328_16.0	1	p2	(CT)6	12	649	660	GCCATGCCATTTTCTTCACT
scaffold462532_14.4	1	p2	(TC)6	12	755	766	CTTTCTCACAGTCGCTGCAC
scaffold462626_16.1	1	p2	(AG)10	20	123	142	TTCAACCTGATTTTCCCCAC
scaffold462642_12.6	1	p2	(CA)11	22	75	96	TGTGCGTAAATTAGCGTTCG
scaffold462732_12.8	1	p2	(TA)10	20	219	238	AACGGCCGAGATTTGTTATG
scaffold462766_11.3	1	p2	(TA)6	12	391	402	GCATGTGCTCCTGCTCATAG
scaffold462954_15.0	1	p2	(AT)7	14	1165	1178	TATTGGTTGGGTACGGGTTG
scaffold462955_15.9	1	p2	(AG)6	12	53	64	TTTCTCATGTTGTAATTAATGTAGGGA
scaffold463052_12.6	1	p2	(CT)6	12	536	547	AAATCAACGAAATCGAACCG
scaffold463338_13.1	1	p2	(TA)7	14	837	850	GGAACAAATGAGACTGTTTCTGC
scaffold463342_18.0	1	p2	(TC)7	14	352	365	AACATTCCCTTTCTTCCACG
scaffold463353_14.3	1	p2	(AT)7	14	476	489	TTTTTCTTCTATCTTTTGATTACCG
scaffold463367_15.7	1	p2	(AG)7	14	320	333	ATTCCACATGGGGTGTCAGT
scaffold463370_15.9	1	p2	(GA)7	14	753	766	TGCAACAAATGATCCCAGAA
scaffold463424_15.0	1	p2	(AT)6	12	734	745	AAAGTTTGGTGAAGTCCGCA
scaffold463437_19.9	1	p2	(TA)6	12	330	341	GTGTTGTGCCTTAGCCCATT
scaffold463617_16.5	1	p2	(TC)10	20	610	629	TTGTGGAGGTGATGTGGAAA
scaffold463694_15.6	1	p2	(AG)7	14	3230	3243	TGCAAGAAATTGACATGTTGG
scaffold463773_13.2	1	p2	(TA)8	16	64	79	GGGAAATTCTTGTACCTCCTCC
scaffold463826_13.6	1	p2	(GA)6	12	171	182	AGCGTAAACACGTCCCCATA
scaffold463842_10.8	1	p2	(TA)10	20	155	174	CCGGAATCGCATCAATACTT
scaffold463892_12.6	1	p2	(GA)6	12	361	372	TCTTCTGAAGTTGCGACCCT
scaffold463919_20.9	1	p2	(AC)6	12	51	62	CTCCTATCTCCAATCCATGC
scaffold464095_15.8	1	p2	(TA)6	12	1300	1311	TTTGGGCCTCTTTGAATGAC
scaffold464137_14.1	1	p2	(TA)6	12	527	538	CACCGACGATCACTCCCTAT
scaffold464150_14.9	1	p2	(AT)9	18	963	980	AATGGGAAAGGGGCTGTAAT
scaffold464161_15.1	1	p2	(CT)7	14	3114	3127	TATTTGGTGGTGGGAGTGGT
scaffold464176_14.7	1	p2	(AT)7	14	221	234	GGGTCGTATCATTCAGCCAT
scaffold464200_12.4	1	p2	(AG)7	14	1097	1110	GAGTGGGGAGAAAGGAAAGG
scaffold464262_14.3	1	p2	(CT)9	18	538	555	CCTTGACTTCACGCCTTCTC
scaffold464366_14.6	1	p2	(GA)6	12	437	448	CGGACAAAATGTACCCCAAC
scaffold464381_13.2	1	p2	(TC)8	16	187	202	TATGGTGGACCGATACATGC
scaffold464389_12.8	1	p2	(TA)6	12	1961	1972	GTCGAAATTGCCCCCTGTAT
scaffold464524_14.0	1	p2	(TA)6	12	132	143	CGTGTTAAGCCTTGGGAAAA
scaffold464593_14.0	1	p2	(CT)8	16	208	223	AACCATTGCACATGAGACGA
scaffold464626_16.7	1	p2	(TA)6	12	133	144	AAAGGATTCGAACCCAGGAT
scaffold464628_12.7	1	p2	(TA)7	14	130	143	GCCATCCATTCTGAAGATCAA
scaffold464686_18.1	1	p2	(AG)7	14	272	285	TGGGACTGTTAAGTTTCCGC
scaffold464695_15.4	1	p2	(AT)7	14	81	94	AATATGAAACCCAACGTCGC
scaffold464715_13.0	1	p2	(GA)7	14	447	460	ATGGAACCACTTGTTGACCG
scaffold464730_10.3	1	p2	(AT)8	16	167	182	GAACTGCAGACGCGTGAAT
scaffold464764_12.4	1	p2	(AT)7	14	1507	1520	ACGTAAGTTCGCAACAACCA
scaffold464864_11.9	1	p2	(TA)6	12	68	79	AACCGAACTCGAGCTTCTCA
scaffold464917_11.7	1	p2	(TA)8	16	684	699	TTTTGAACCCTAAAAACTCGC
scaffold464940_13.3	1	p2	(AC)6	12	154	165	TTGTGTCTTAAAGTATAAAGGCATGA
scaffold465068_12.0	1	p2	(AT)9	18	995	1012	TCAAGGGTTTTCGCAGAGAC
scaffold465112_11.2	1	p2	(CT)6	12	106	117	AACCCCTACACCACCCCTAC
scaffold465175_17.6	1	p2	(TA)6	12	802	813	TACATGCTTTGTTGGGGACA
scaffold465255_14.1	1	p2	(AT)8	16	546	561	TCACCTTTCCTCCCAAATGA
scaffold465413_15.7	1	p2	(GA)6	12	132	143	CATAGGATTTGTGAAGGCCG
scaffold465504_14.1	1	p2	(AC)7	14	1188	1201	TCCATTCAACCAAATAGCCC
scaffold465569_10.0	1	p2	(CT)7	14	53	66	GCATCTCTCTCTTGCTCTTATTG
scaffold465579_14.8	1	p2	(AG)7	14	2076	2089	GCGTACGAATCCAAACCCTA
scaffold465809_13.9	1	p2	(AG)6	12	868	879	AGAGATCGGGTTTGCTGAAA

scaffold465823_17.2	1	p2	(CT)6	12	123	134	CTTTCCCCACCATTTTTCCT
scaffold465937_16.6	1	p2	(TA)7	14	67	80	TCAGTTTCGAGTTATGAAGTTAGGAA
scaffold466310_18.9	1	p2	(TA)7	14	858	871	GGTTATAGCTACGTGGGCGA
scaffold466337_13.2	1	p2	(AG)14	28	1138	1165	GAAACCGAGTGTGCATCTCA
scaffold466365_12.8	1	p2	(AC)8	16	73	88	CACCCACGCATATACACACA
scaffold466389_10.8	1	p2	(CT)8	16	374	389	TCATGCTGAAATCGTTCGAG
scaffold466441_19.2	1	p2	(TA)6	12	319	330	TAGGTCACGGGTTCGAATTT
scaffold466463_10.2	1	p2	(AG)8	16	76	91	TCTACGTCCACGGAGAAAGG
scaffold466570_14.2	1	p2	(AG)6	12	1962	1973	AAAACGTCATTTTTGGCCTG
scaffold466606_12.0	1	p2	(TA)6	12	350	361	AATTGAGGGAGTGGGTGTTG
scaffold466630_14.9	1	p2	(TA)8	16	128	143	GGAAGCTAGGCGGTTTTGAT
scaffold466647_10.4	1	p2	(TC)12	24	235	258	CTTCAGCCCCTCTAGGTTTG
scaffold466653_14.8	1	p2	(AT)9	18	2212	2229	TGTTGTATTGTAAAGCCGCAA
scaffold466656_13.4	1	p2	(AT)6	12	1568	1579	AATCCGTAAGAATAATCCATGC
scaffold466667_10.7	1	p2	(AT)8	16	164	179	ACGCGCGTTTTTGGATTTA
scaffold466686_17.0	1	p2	(AG)8	16	4973	4988	GGCGGATGAATAAACCTCTG
scaffold466706_15.0	1	p2	(CT)7	14	1474	1487	GTCAAAACCCTGCCTTCAAA
scaffold466737_14.2	1	p2	(TA)8	16	320	335	TACTTGGTTGAGCTTGGCG
scaffold466773_13.7	1	p2	(TG)7	14	415	428	CGATATGGACGCCTCAGATT
scaffold466826_16.2	1	p2	(AT)6	12	2855	2866	ACATTTAACTTCGCCCCTGA
scaffold466935_14.0	1	p2	(TA)7	14	145	158	ATTTGCTCCAACAAACCTCC
scaffold467017_11.0	1	p2	(TA)7	14	369	382	GCTTGCTGATTGTGGTTCAA
scaffold467115_17.0	1	p2	(AG)7	14	296	309	GCCAATTCATATTTATTTGTGATCTTT
scaffold467144_13.4	1	p2	(AG)9	18	225	242	AAAAATCTTGCTGCACCCAC
scaffold467178_15.0	1	p2	(AT)14	28	52	79	TTGACATTCGCATTTTAAGAGTAG
scaffold467184_15.0	1	p2	(TA)10	20	2210	2229	ACTTGTGGCTATGCCTTTGG
scaffold467263_13.0	1	p2	(TA)9	18	179	196	TGGCTATCATGGATGCAAAA
scaffold467355_21.8	1	p2	(TA)12	24	2221	2244	GCAACCAAAACACACACACA
scaffold467417_17.7	1	p2	(TC)6	12	1483	1494	TCTGTCTCGAATTTGCGATG
scaffold467443_14.2	1	p2	(TA)7	14	1820	1833	ACCGGGCACTAAGGGATTAT
scaffold467669_14.4	1	p2	(TA)7	14	267	280	ACCTTGACCTTCAGGCTTCA
scaffold467676_13.6	1	p2	(AT)10	20	577	596	TATGTGCGCGTTTCAATGAT
scaffold467753_15.7	1	p2	(CT)7	14	2133	2146	TGAAAATCTGACGAGCATGG
scaffold467817_17.6	1	p2	(GA)6	12	4116	4127	TGTGAGAGTGAGAGAGGGGG
scaffold467875_13.7	1	p2	(TC)8	16	121	136	CTGATTCGCACAAATGATCG
scaffold467887_11.3	1	p2	(TA)10	20	41	60	CGGATTTAAGGGTTATTACCTGATT
scaffold467902_13.9	1	p2	(TC)6	12	711	722	TTCCCCTCATCTTCAAGTGG
scaffold467922_14.6	1	p2	(AT)9	18	177	194	TTGCGTGTCAGTGTTTTTCC
scaffold467952_15.4	1	p2	(AT)8	16	327	342	CTTCATCCCTCAATCCCTCA
scaffold468007_10.8	1	p2	(GA)6	12	348	359	CGCGAAATGAGGAGCTTAAC
scaffold468115_11.8	1	p2	(TC)6	12	188	199	GGAAACCCACAATCATCACC
scaffold468146_15.7	1	p2	(AG)9	18	1724	1741	TTACAACCCGAAAGAAACCG
scaffold468164_12.6	1	p2	(AT)10	20	730	749	CCCCCTTATTTTATCCCAACA
scaffold468420_11.7	1	p2	(AT)6	12	401	412	CCGTTGCTTTCTCTCTCGTT
scaffold468591_11.4	1	p2	(CT)10	20	511	530	CTCAGAAATCAAGCCGAACC
scaffold468609_20.1	1	p2	(TC)6	12	307	318	CAGCGAGGAGGTTGAGGTAG
scaffold468615_14.5	1	p2	(AT)9	18	202	219	CAGTATTACAGCCGTGGCAA
scaffold468639_17.1	1	p2	(AT)6	12	1565	1576	TTCCCTTCCAGTTACACCGT
scaffold468658_16.5	1	p2	(AT)7	14	1998	2011	TCAAGTCCTCACACACTGATGA
scaffold468668_12.6	1	p2	(GT)7	14	747	760	CACGAGCTCTTATTTGGCCT
scaffold468669_12.1	1	p2	(AT)8	16	365	380	GGATGGAACGCACAAAACTT
scaffold468740_15.2	1	p2	(TA)6	12	1678	1689	TGGACAATTCAATAGAAAACCG
scaffold468785_14.6	1	p2	(TA)9	18	664	681	GGTTTGGTGAAAATGATGGC
scaffold468805_10.3	1	p2	(CT)6	12	299	310	GCTTCTTTCGCTTTCTCATTG
scaffold468828_11.4	1	p2	(TA)9	18	150	167	GTATTCCCGGGTTTCTCCAG
scaffold468909_13.8	1	p2	(AC)6	12	1271	1282	GGACGTTTCCTCGTTTTTCA
scaffold468921_18.1	1	p2	(AG)9	18	4646	4663	GGCTTTTTGCTAATTGGCTG
scaffold468936_13.7	1	p2	(CT)6	12	319	330	AACCAGCTCGGTGATATATGG

scaffold469003_19.2	1	p2	(TA)8	16	39	54	TGACGGGATTTAGGTGTTTTG
scaffold469083_15.2	1	p2	(CG)6	12	202	213	CACTCATTTGGGGGATTGTT
scaffold469108_17.0	1	p2	(CT)9	18	1112	1129	CACTGACATTTCCTCGCTCA
scaffold469290_12.0	1	p2	(GA)6	12	1248	1259	TGGCCACCTCCATTTCTAAC
scaffold469291_16.2	1	p2	(AT)8	16	338	353	TCCCCTTAGGAAAACCGTTC
scaffold469304_18.8	1	p2	(AG)8	16	130	145	ACACACGCCACCTTTTTAGG
scaffold469357_15.8	1	p2	(CA)7	14	3774	3787	AGTCACCAAAGCTCCCACTC
scaffold469366_11.3	1	p2	(CT)8	16	409	424	AAAGTATGCAAGGCCCACAC
scaffold469468_12.0	1	p2	(CT)6	12	122	133	GGATCGAAAAGAGAGGGAGG
scaffold469580_13.8	1	p2	(GA)7	14	572	585	AGTCACCTTGTTCAAACGGG
scaffold469620_20.1	1	p2	(GA)6	12	227	238	GCGGTGTTGTCTGAGTGTGT
scaffold469651_14.3	1	p2	(GA)6	12	426	437	GGTTGCTTCTTACAGGCTGG
scaffold469720_11.4	1	p2	(TA)7	14	609	622	GCATATCGGTTCGGTCTCAC
scaffold469818_14.1	1	p2	(GT)6	12	917	928	CATCAGTCCAGCCTTCTTGG
scaffold469847_11.2	1	p2	(TA)7	14	406	419	TGATGTTTGCATTTGAAGAGG
scaffold469952_10.7	1	p2	(TA)11	22	51	72	CGTTTTGACAATATATAGGTGTTTTGA
scaffold469953_13.7	1	p2	(TA)6	12	329	340	ATCAACGCCCATGATCAAAT
scaffold469956_14.1	1	p2	(CT)6	12	48	59	TTTCTCAGCCAAGCTCACCT
scaffold470011_15.1	1	p2	(AT)6	12	659	670	TTCCCCTTTATCTCCTCATTCA
scaffold470052_18.7	1	p2	(CT)7	14	311	324	TCTAAAACCCTCGCCTCTCA
scaffold470085_16.3	1	p2	(AT)6	12	1852	1863	TGATTCCATTCCATGCTTCA
scaffold470181_17.7	1	p2	(TA)6	12	719	730	GATTTTCGTAATCCCCTTTTCA
scaffold470248_11.5	1	p2	(AT)8	16	431	446	GTTTCCTGTTCGGCTGTTGT
scaffold470271_10.8	1	p2	(TA)11	22	305	326	AGACCACCGAAAAATGTTGG
scaffold470304_15.4	1	p2	(CT)6	12	2820	2831	AGGCTTGCAAGAAGCTGAAG
scaffold470381_14.5	1	p2	(AT)10	20	685	704	TCAGCGCCACGTATCCTAGT
scaffold470409_13.2	1	p2	(AG)6	12	415	426	TCATTTCGGGTTATTTTCGG
scaffold470459_13.9	1	p2	(CT)6	12	212	223	ACAGGTCCCTGAAACAAACG
scaffold470540_15.4	1	p2	(CT)7	14	109	122	TCCTTTCAGAGGCAGAAACC
scaffold470544_10.7	1	p2	(AT)9	18	154	171	GCATGCAAAATCAAGCTCAA
scaffold470585_14.4	1	p2	(TC)6	12	53	64	CCTGCCATTTCTCTCGTGTT
scaffold470587_14.3	1	p2	(CT)6	12	33	44	ACAAAATGGCCAAGAGAGTTT
scaffold470597_13.9	1	p2	(GA)6	12	429	440	TTGAACAACAAGGCCAGTGA
scaffold470660_16.4	1	p2	(TA)8	16	624	639	CAGAACCCGAAGTGAAGAGG
scaffold470702_14.6	1	p2	(TC)8	16	130	145	TCCACAGCAGAGTCTCCTCA
scaffold470708_16.3	1	p2	(AC)7	14	2283	2296	ACAGAAAACTTTCATGCCGC
scaffold470795_15.4	1	p2	(TA)6	12	2063	2074	CGAGCTCACCTTGTGTTGAA
scaffold470874_15.8	1	p2	(TG)6	12	2687	2698	CCAGCAAGTGCAGAGTGGTA
scaffold470877_14.4	1	p2	(TA)6	12	437	448	TTTCTCTCCCGTGGACGTAG
scaffold470992_10.4	1	p2	(CT)6	12	815	826	AGCAAGGTGATGCGAGACTT
scaffold471041_15.9	1	p2	(CA)6	12	1291	1302	CGGGTATACTTGGAGAAACACA
scaffold471121_17.5	1	p2	(TG)6	12	812	823	TGCACATGTAAAACGATCCA
scaffold471175_13.6	1	p2	(AT)8	16	86	101	GGACGGTTAAATTTTGTCCC
scaffold471176_14.8	1	p2	(GA)6	12	714	725	GGTGCAGAAGAGAAATTCCG
scaffold471351_11.0	1	p2	(AG)6	12	205	216	TCGAATCCACATCAGATCCA
scaffold471425_15.4	1	p2	(TC)6	12	393	404	CATGGGCCTTGCATGTATAA
scaffold471453_15.2	1	p2	(CT)6	12	133	144	TCTCGTTGCTATTCTTGCCA
scaffold471519_12.3	1	p2	(TC)7	14	77	90	GAAAGGATGAAAATCATTGGC
scaffold471536_14.4	1	p2	(AT)6	12	223	234	CCAATGGTTATCATCCCACC
scaffold471557_15.9	1	p2	(CT)11	22	470	491	ACTCTCTCTCACCGCCACAT
scaffold471578_16.3	1	p2	(TA)6	12	206	217	AATTAGCGTGTATGCTGGGG
scaffold471586_16.2	1	p2	(CA)6	12	272	283	TAAAGGCAGAAAACGGAGGA
scaffold471610_16.6	1	p2	(AC)6	12	584	595	CACTGGAAAAATGGCACCTT
scaffold471622_12.7	1	p2	(GA)6	12	687	698	GAACGAGGGCCTTCTTCATT
scaffold471656_15.1	1	p2	(TA)6	12	1664	1675	GGTAAATCAATGTGAAATGATGGA
scaffold471667_17.0	1	p2	(TA)6	12	2158	2169	CTATGGACCCTTGCAGGAAC
scaffold471777_10.7	1	p2	(CT)7	14	424	437	GAAGAATCACCGGACTGAGC
scaffold471800_17.7	1	p2	(TC)6	12	638	649	CTTGTGACCCTCACCACCTT

scaffold471822_10.6	1	p2	(AG)10	20	288	307	TCTTTGCGCCTAGATTTCTCA
scaffold471955_13.7	1	p2	(TA)6	12	134	145	CCCAAAGATTTATAGGTTTTATTTCAA
scaffold472039_14.8	1	p2	(TA)8	16	148	163	AACCACAGCCCTACATCTCG
scaffold472141_13.0	1	p2	(TA)6	12	52	63	CCAATTTGAATGGTTATCACCTG
scaffold472148_18.1	1	p2	(TC)6	12	68	79	GATAATATGGCCGAGAGTTTGC
scaffold472149_12.8	1	p2	(TA)9	18	294	311	AGCCACACATGTCACAAATGA
scaffold472183_11.7	1	p2	(TA)10	20	42	61	CGGATTTAAGGGTTATTACCTGAT
scaffold472273_16.3	1	p2	(CT)6	12	409	420	TGGAGAGCTCGAGAGAGAGG
scaffold472312_13.8	1	p2	(AT)6	12	217	228	GCAAAAGCTTTCTCGACCAC
scaffold472600_10.7	1	p2	(CT)6	12	351	362	CTTCAAGCGGTCTTGATTCC
scaffold472606_13.2	1	p2	(AG)6	12	307	318	TAGGGGGTGGTTGGTGTTTA
scaffold472691_16.7	1	p2	(GA)6	12	2033	2044	ATCGTCGTCGTCTTCTTCGT
scaffold472806_11.1	1	p2	(AT)8	16	605	620	TTCAATTAATGTTGTCATTATTCCAAG
scaffold472807_17.2	1	p2	(TA)7	14	545	558	GACTTGCCAACGGTCAATCT
scaffold472925_14.0	1	p2	(TC)6	12	208	219	GACTCATGCTCGGCATACAA
scaffold472941_12.9	1	p2	(AT)9	18	48	65	TCAAGGCCGATAAGATGGAT
scaffold473012_15.6	1	p2	(AT)9	18	323	340	TAACGATGGACTAGGGGCTG
scaffold473018_12.5	1	p2	(GA)6	12	87	98	TAAATATGCAGCAGACGCGA
scaffold473142_10.4	1	p2	(AT)8	16	257	272	TCGGAAAGGAAAACCGTAAA
scaffold473266_15.0	1	p2	(TC)6	12	134	145	ATCGGCTGAATAGCCGTATG
scaffold473401_17.0	1	p2	(AT)7	14	294	307	ACCTCGTCGTCCAACTCACT
scaffold473465_16.0	1	p2	(TC)9	18	180	197	CAGCTCCACTACTTCCTCCA
scaffold473475_11.4	1	p2	(AT)6	12	324	335	GAAAGGGCTGAGGTATGGTG
scaffold473491_12.6	1	p2	(TA)10	20	69	88	AAAAATGGTTATTATTGGATCGC
scaffold473547_14.7	1	p2	(TC)6	12	154	165	CGTAGAGAATGTTGCCACGA
scaffold473586_15.0	1	p2	(TA)6	12	525	536	GTTTTTGAACCCCCTCCTTC
scaffold473603_13.9	1	p2	(AT)7	14	1357	1370	ATGCGCGGGTGTAAAGTTAG
scaffold473664_10.0	1	p2	(AC)7	14	80	93	AGCCTCGGATTTCCTGTTCT
scaffold473793_12.0	1	p2	(TC)6	12	577	588	GCCTTCGGATCTGGGTATTT
scaffold473955_11.4	1	p2	(AG)8	16	1004	1019	GCTTCAGTCATGTCGTTCCA
scaffold474132_13.6	1	p2	(AG)6	12	795	806	GCAACGAAGATGGGAAATTG
scaffold474140_15.2	1	p2	(TA)6	12	62	73	CATCACACCCACAAATGACTCTCT
scaffold474259_15.9	1	p2	(AT)6	12	3913	3924	CGGCGTAACTACCATTCACC
scaffold474486_16.0	1	p2	(TA)8	16	152	167	TGTGGTTTGCTAGACATCGC
scaffold474566_18.7	1	p2	(CT)8	16	82	97	GGACCTGAAAAAGGACTTTGG
scaffold474623_15.4	1	p2	(AT)6	12	2506	2517	ATGGCTGCTCCAATATCACC
scaffold474648_15.9	1	p2	(GA)10	20	74	93	ATTCAGAAATGGCGATGACA
scaffold474851_16.1	1	p2	(AT)7	14	583	596	AAAAGCCGATCGAGTTCGTA
scaffold474929_13.2	1	p2	(CT)6	12	44	55	TCCACCCTCTCACTTCCACT
scaffold475149_10.7	1	p2	(TG)6	12	177	188	ACCACGAGATGGGGACATAA
scaffold475165_14.5	1	p2	(TC)7	14	282	295	ATTTGCGGAAACAATCCAGA
scaffold475200_23.6	1	p2	(AT)6	12	350	361	TCGATTCGACTGGATTCGTT
scaffold475308_12.0	1	p2	(CT)6	12	134	145	TATAGTCCACCCAACCCGAG
scaffold475369_19.5	1	p2	(CT)6	12	1230	1241	TTGGAGTTGGAGATGAAGGC
scaffold475474_14.4	1	p2	(TG)7	14	71	84	CTAGCGGATTTCTCTGGCTC
scaffold475525_11.0	1	p2	(TA)8	16	46	61	ATCGGTTATTACCGGATCGC
scaffold475712_12.0	1	p2	(AC)7	14	898	911	TCAATACCCGAGAATTGCAT
scaffold475714_17.4	1	p2	(AT)6	12	185	196	TCCAATTTCAGTTAAACACACCA
scaffold475720_12.3	1	p2	(CT)9	18	50	67	CACTCACTCTCTCTAGCCGC
scaffold475833_10.1	1	p2	(GA)6	12	127	138	AGATATGTCGGCAAGCGAAT
scaffold475932_12.1	1	p2	(TA)8	16	83	98	GCATCCAATACCAATATCTCAGC
scaffold475937_14.9	1	p2	(TA)9	18	478	495	TTCCACATTGATTCCTGGGT
scaffold476008_17.0	1	p2	(GA)6	12	721	732	AGTGCTTCTTGAAGCTGGGA
scaffold476039_11.3	1	p2	(TA)7	14	496	509	GTAATAGAACGTGCCCCCAA
scaffold476154_17.7	1	p2	(TC)6	12	594	605	GGAGGATTTTCCTTTCCCAA
scaffold476203_12.5	1	p2	(TA)6	12	129	140	GAATGCGGAGTTGGAATCAT
scaffold476209_16.3	1	p2	(TA)8	16	361	376	TTTTTCCTAACCGGACCACA
scaffold476319_11.4	1	p2	(CT)6	12	251	262	TCCCCATTGTTCTACCCAAA

scaffold476333_10.[illegible]	1	p2	(GA)6	12	42	53	TTTGTGTGAGGAATTGTTGAAGA
scaffold476452_16.[illegible]	1	p2	(TA)8	16	308	323	TTTTTGGGTTGAAAATAGCG
scaffold476505_16.[illegible]	1	p2	(TC)9	18	716	733	CCAACCATTGCAGAATCCTT
scaffold476601_14.[illegible]	1	p2	(TA)6	12	135	146	GCACACATTTTTCTCCCCAT
scaffold476603_15.[illegible]	1	p2	(AT)8	16	132	147	TTACTTTTCCGGCAACATCC
scaffold476649_15.[illegible]	1	p2	(TC)9	18	354	371	CCTAGCCAAGTTTCACGAGC
scaffold476674_14.[illegible]	1	p2	(TA)8	16	112	127	ATACCGGGATCGAATCTTCC
scaffold476922_17.[illegible]	1	p2	(AG)7	14	180	193	GAAAAGAAAAAGAAGGCACTCG
scaffold476982_12.[illegible]	1	p2	(AT)6	12	331	342	CCATCATGGGGTTTTCTTACA
scaffold477044_13.[illegible]	1	p2	(TC)6	12	136	147	CAAATCAACGCGAATTCAAC
scaffold477161_10.[illegible]	1	p2	(TC)12	24	483	506	CTTCTTCAAACACCCCCAGA
scaffold477283_10.[illegible]	1	p2	(GA)7	14	279	292	ATTTGGATGGTGATTGTGGC
scaffold477406_13.[illegible]	1	p2	(TC)6	12	552	563	GAGGAGCCCAAGATGTCAAG
scaffold477464_15.[illegible]	1	p2	(TA)10	20	803	822	ACAAGAATGCCCCTGTATGC
scaffold477485_14.[illegible]	1	p2	(TA)7	14	318	331	ATCCACTGCCAACCTCATTC
scaffold477491_13.[illegible]	1	p2	(TC)9	18	46	63	CAAGCCCAAAAGCACAAAGT
scaffold477517_18.[illegible]	1	p2	(GA)6	12	936	947	GAGGATTTGTCAACCGGAAA
scaffold477630_13.[illegible]	1	p2	(TA)6	12	542	553	GATGCTTTTCCCCTTCTTCC
scaffold477650_17.[illegible]	1	p2	(CT)6	12	1200	1211	CTCCCTTTACCTCCCCAAAG
scaffold477723_16.[illegible]	1	p2	(GA)6	12	399	410	ATATGGTTTGACGCCTTTGC
scaffold477735_15.[illegible]	1	p2	(TC)6	12	322	333	GAAGGAGGAGAAGTTTGGGG
scaffold477748_11.[illegible]	1	p2	(CT)6	12	55	66	CCCAATACTCTTTCCCAACC
scaffold477768_19.[illegible]	1	p2	(AG)7	14	90	103	TGAGGACGGTGAGGATTAGG
scaffold477770_16.[illegible]	1	p2	(AG)7	14	133	146	ATCCCGACTGATTTCGATTG
scaffold477802_15.[illegible]	1	p2	(GA)6	12	865	876	GCACAACTGCAGACCAAGAA
scaffold477993_12.[illegible]	1	p2	(CT)7	14	109	122	CACTTTCTCTCTTCCGACCG
scaffold478000_11.[illegible]	1	p2	(AG)6	12	149	160	GAATATTGTGCGTCCCCATC
scaffold478024_15.[illegible]	1	p2	(TA)10	20	1302	1321	GCGTGCGAATTTAATGATGA
scaffold478034_17.[illegible]	1	p2	(AT)8	16	672	687	AAGCCGCATTTTAATCGAGA
scaffold478044_10.[illegible]	1	p2	(TA)7	14	72	85	GATCGGATACGGATCGAAAAT
scaffold478075_14.[illegible]	1	p2	(AT)7	14	44	57	TGCTTTTCTAAATGGAGAGTTGG
scaffold478111_14.[illegible]	1	p2	(AT)7	14	83	96	TGCTGTAAATGAAACTGTTGTATGC
scaffold478135_12.[illegible]	1	p2	(AT)6	12	179	190	CCAACTTGAAACCGGAAAAA
scaffold478152_13.[illegible]	1	p2	(TC)6	12	1559	1570	GGCGGGATATACTAATGTCCAA
scaffold478305_12.[illegible]	1	p2	(TA)6	12	34	45	ACTTTTAAGGATCAAGGATATTCG
scaffold478362_15.[illegible]	1	p2	(GA)6	12	250	261	CTCGAGTGTAAACACGTCCCT
scaffold478378_11.[illegible]	1	p2	(TA)8	16	332	347	TCCGGATACCTACTACTGCGA
scaffold478448_14.[illegible]	1	p2	(CT)6	12	297	308	TTGGTATACAACTTTGATTCCCC
scaffold478586_18.[illegible]	1	p2	(TG)7	14	880	893	ATGCACACCGAGATGTGGTA
scaffold478666_14.[illegible]	1	p2	(AG)7	14	550	563	TTCAACGGAGTGGAAGATCC
scaffold478755_12.[illegible]	1	p2	(AT)10	20	442	461	CAGGTTCGAGCTTCCATAGG
scaffold478879_11.[illegible]	1	p2	(TA)9	18	361	378	AGCCTTTAATCAGCAATATAAGTCA
scaffold478890_16.[illegible]	1	p2	(AT)6	12	265	276	AGGGCTACAAATTGCAAGAA
scaffold478967_14.[illegible]	1	p2	(AT)7	14	355	368	TATCGGTTATCATCGGATCG
scaffold479173_10.[illegible]	1	p2	(TC)7	14	130	143	GCACAACCACTCCACAAAGA
scaffold479248_15.[illegible]	1	p2	(TA)9	18	1389	1406	CGATGTTTGTTGGCTGTCTG
scaffold479312_15.[illegible]	1	p2	(GA)7	14	1169	1182	TTGGAACCAACTAATTGCCA
scaffold479338_17.[illegible]	1	p2	(AT)6	12	109	120	ACGTTCATACCTTTGTGGCA
scaffold479341_16.[illegible]	1	p2	(CT)6	12	234	245	TCATTTCATCGATTTTTCCTCTC
scaffold479345_15.[illegible]	1	p2	(TA)6	12	214	225	TTCAATTTCTTGGCTCAGGG
scaffold479655_15.[illegible]	1	p2	(GA)6	12	6101	6112	AAAGCAAAATACCACCACGC
scaffold479701_17.[illegible]	1	p2	(GA)6	12	2931	2942	GAGCTAACTGCATTCCAGACG
scaffold479715_10.[illegible]	1	p2	(AG)12	24	667	690	GGGATCGGTTTATGTGTTGG
scaffold479734_17.[illegible]	1	p2	(AT)6	12	86	97	AGAATCACGGCCATTTTCAG
scaffold479803_15.[illegible]	1	p2	(TA)6	12	1047	1058	TCTTGTTGGCATGAGCTTGT
scaffold479806_12.[illegible]	1	p2	(GA)7	14	442	455	AAGGACGGAGAGAGAGAGCA
scaffold479996_16.[illegible]	1	p2	(AT)6	12	428	439	CGCATTCATTAGCTACACCC
scaffold480058_11.[illegible]	1	p2	(AT)6	12	331	342	AAATGAATGTTGTGTTTGTTTGTAA

scaffold480164_16.4	1	p2	(AG)8	16	528	543	AAACCCACCTGCTGCATAAT
scaffold480179_10.2	1	p2	(GA)6	12	108	119	ATATCGTCGTCTCCCGATGA
scaffold480225_14.0	1	p2	(TG)7	14	1041	1054	TCTCAAATCGTTTCTATCGTGC
scaffold480380_11.3	1	p2	(AG)10	20	78	97	TGTGCAAGGTTGAACCAAGA
scaffold480407_17.4	1	p2	(TA)6	12	786	797	GAAGAGGAGGAAAGAAAACGC
scaffold480425_14.4	1	p2	(AT)7	14	1389	1402	AATTGCGTTAGGGATTTGATT
scaffold480443_14.5	1	p2	(TA)7	14	564	577	GACTTGCCAACGGTCAATCT
scaffold480473_12.4	1	p2	(GA)9	18	80	97	ATGGAACCACTTGTTGACCG
scaffold480525_18.2	1	p2	(AG)9	18	52	69	CGAAGAACAATTACAATGTTTATCCA
scaffold480654_14.2	1	p2	(TA)6	12	55	66	TGCACCTGATCATGAACCTA
scaffold480660_13.8	1	p2	(CT)7	14	1434	1447	TGACTCTCCCCTTCCATCAC
scaffold480678_12.1	1	p2	(TA)10	20	1090	1109	TTTTTAGAAGGATACTCACTACTCCA
scaffold480754_15.0	1	p2	(AT)8	16	389	404	ATATTCCCCGGTATGCCAAC
scaffold480777_14.6	1	p2	(AG)7	14	487	500	ATCAAAGAAAACGGTGGTGG
scaffold480831_14.8	1	p2	(AT)6	12	1302	1313	ATCTCCCACCAATGCACTTC
scaffold480839_16.7	1	p2	(TA)6	12	1279	1290	AGCACATTTAATTCCAGGTCG
scaffold480889_12.1	1	p2	(CT)6	12	157	168	CGAGCTCTGAAAATTCTCCC
scaffold480928_16.0	1	p2	(CT)6	12	137	148	GGCGGATAGCAAATCAAAAA
scaffold480930_18.2	1	p2	(CT)6	12	165	176	CAGCCTCCATTTTGTTACCG
scaffold480954_16.6	1	p2	(GA)11	22	3796	3817	AAAGAGAAAACGAGGGGTGC
scaffold480979_16.3	1	p2	(GA)7	14	737	750	GGACAACCCATAACCCACAC
scaffold481059_17.1	1	p2	(AT)6	12	736	747	AGTTTGGAGCACACGAGCTT
scaffold481083_16.4	1	p2	(AC)7	14	237	250	TATTCAACTCCATCTCCCCG
scaffold481239_13.7	1	p2	(AT)11	22	2240	2261	TTCAACGGTCCCACAAATTA
scaffold481407_14.9	1	p2	(AT)9	18	175	192	CATCAGATCTGCGCTGAAAA
scaffold481541_10.6	1	p2	(AT)10	20	300	319	TCACTGGCAGCGAATATGAA
scaffold481564_12.6	1	p2	(TC)6	12	182	193	GTCCAATGTTTAGGCCCGTA
scaffold481807_16.5	1	p2	(GA)12	24	5944	5967	GCAATAAAGAGGCTGGTGCT
scaffold481870_15.6	1	p2	(CT)9	18	623	640	AGCCCTCATTCCTCCCTTTA
scaffold481939_13.6	1	p2	(TA)8	16	133	148	GGCAATATGCTGACAGACGA
scaffold482126_14.7	1	p2	(CA)7	14	54	67	GGAGTCTGGAAAAGAGAGAGTTG
scaffold482141_14.1	1	p2	(CT)8	16	515	530	CATTCTTTCTCCTCCCCACA
scaffold482147_16.3	1	p2	(AT)6	12	121	132	AAACGACATCATGACGCAAC
scaffold482179_12.6	1	p2	(TA)10	20	42	61	AAGGGTTATCACCTGATTTTCTC
scaffold482219_13.4	1	p2	(AT)7	14	60	73	TCTAAATTGCGAGAATATGAGAGTTT
scaffold482271_11.8	1	p2	(AT)9	18	610	627	TGCTTCAAGGTAGGTTTATTAGGC
scaffold482306_15.3	1	p2	(TG)9	18	236	253	TCGTATTGACGGGAGAAACC
scaffold482356_11.5	1	p2	(TA)10	20	734	753	TGGCTGCTCCTCATCCTTAT
scaffold482531_14.3	1	p2	(GA)6	12	4017	4028	ACTCGCGAAGTTCCTTCAGA
scaffold482608_12.4	1	p2	(GA)7	14	227	240	AGAACTCCACTTGCACAGCA
scaffold482617_16.5	1	p2	(GC)6	12	3215	3226	CACCACATAATGGACGTTGG
scaffold482656_14.2	1	p2	(GA)8	16	1414	1429	CGAGCTTTTATCGAACGGAG
scaffold482744_14.1	1	p2	(TC)8	16	3120	3135	GCTATGGTGGCCAATGTCTT
scaffold482749_15.0	1	p2	(AG)6	12	1196	1207	AACTCCCCTTTCCTTTGCAT
scaffold482821_12.7	1	p2	(TA)6	12	682	693	GGCAAATGATTGATTGCCTT
scaffold482961_11.5	1	p2	(AT)6	12	1054	1065	TGACTTTCATGTAGCGTGGC
scaffold483020_17.6	1	p2	(AG)6	12	8248	8259	TTTGCTGTGCGTCGTATTTC
scaffold483083_11.9	1	p2	(GA)6	12	112	123	TGCCACTTATTTCAGGAGGT
scaffold483092_17.9	1	p2	(GA)9	18	3339	3356	TTAGCACCTGATTACTCCCGA
scaffold483164_16.2	1	p2	(TG)6	12	91	102	CCGTGGACGTAGATCCTTGT
scaffold483239_15.1	1	p2	(TC)8	16	1021	1036	TCTGGTGTGCTTTCTGCAAC
scaffold483247_10.5	1	p2	(GA)6	12	30	41	GGGGGAGAATTGAATTGCAG
scaffold483367_12.1	1	p2	(TA)9	18	2452	2469	TGCAAATACGAATATCATTATTCAA
scaffold483414_13.2	1	p2	(CA)6	12	234	245	TTCAATCCAAAAAGACACATGC
scaffold483446_16.7	1	p2	(AT)6	12	307	318	ACATTGTATCTTGTCTTGCCCT
scaffold483487_13.7	1	p2	(AT)7	14	712	725	ATTACCTTGATCGACGACGC
scaffold483599_14.9	1	p2	(CT)6	12	1969	1980	CCTCTCAGCCCTAGCTTCCT
scaffold483707_11.3	1	p2	(CT)6	12	267	278	TTTAAATGGGCGGAAAATA

scaffold483770_15.7	1	p2	(CT)6	12	1193	1204	CATCCTCAATTTCAGCACCC
scaffold483772_16.2	1	p2	(TA)8	16	359	374	TGCTGACCCTCTGCTGTATG
scaffold483878_13.9	1	p2	(TA)8	16	4861	4876	CCTCTCCTCGTCTTGTGCTT
scaffold483924_11.6	1	p2	(TA)7	14	464	477	ATTTGTTTGCATACGGCCTC
scaffold484129_11.7	1	p2	(TA)7	14	136	149	GGCTTGTTTGAACCATTTTGA
scaffold484163_14.2	1	p2	(AT)8	16	251	266	CATGTCAATATCATCACGAGTTTGT
scaffold484191_13.9	1	p2	(TA)8	16	526	541	TTTTGGAGGGAAAACCACTG
scaffold484194_14.2	1	p2	(TA)8	16	133	148	AAAATCAAGGGCTGTGATGG
scaffold484195_15.1	1	p2	(AT)8	16	1784	1799	TAGGATCCCGGATAATCCAT
scaffold484295_13.4	1	p2	(AC)8	16	1006	1021	GCTGAGCATATTTTTCACCCA
scaffold484331_13.5	1	p2	(CT)6	12	106	117	TGCATTGTTCGACGATGTAAC
scaffold484469_15.7	1	p2	(AT)8	16	71	86	TTAACACCAAACCATCTAATTAGTACA
scaffold484521_12.9	1	p2	(GA)6	12	235	246	GGTTACCGTCGTGGTTATGG
scaffold484528_11.1	1	p2	(AT)10	20	43	62	TGGCAGAAAGATTTTAGATATAGACG
scaffold484720_17.0	1	p2	(AG)7	14	1162	1175	GCAGTCTGTAAGGCCTGGTC
scaffold484887_17.9	1	p2	(TA)8	16	147	162	ACCCAACCACCCTTTATTCC
scaffold484888_14.9	1	p2	(TA)10	20	1860	1879	TTTTACAATTTTGCCCCTCG
scaffold484957_15.2	1	p2	(TA)9	18	881	898	GGCAAGTTGAAGGAACAAGG
scaffold484995_11.6	1	p2	(AG)6	12	116	127	TTCCTCTCGTGTTTCATCCC
scaffold485004_10.6	1	p2	(AT)7	14	81	94	GGACAAAAGAAAAACCACGC
scaffold485103_13.6	1	p2	(AT)9	18	286	303	CCGAAAATCAAAGGCCTACA
scaffold485222_12.0	1	p2	(TA)7	14	222	235	TGGCACAGAGAGTACATAGTTTCC
scaffold485296_15.3	1	p2	(AT)6	12	1036	1047	TCATGCACAAGGTGGAATGT
scaffold485325_18.1	1	p2	(GA)9	18	259	276	AAAGGTGAGGGAGAGTGGGT
scaffold485502_15.9	1	p2	(TA)6	12	661	672	TTTGTTGCAAATATCAGGTCTTTT
scaffold485528_11.8	1	p2	(AT)6	12	725	736	TGCTGACCCTCTGCTGTATG
scaffold485542_12.9	1	p2	(TA)7	14	828	841	ATAACAGTGGTGGCCGAAAA
scaffold485569_14.8	1	p2	(GA)6	12	165	176	GGGTGCTTGAGGGAGTTGTA
scaffold485715_17.8	1	p2	(TG)6	12	638	649	TTGCTTGGGATTTTATTTTGAA
scaffold485769_10.8	1	p2	(AT)6	12	30	41	TTTGTATGGGTCATTGGACG
scaffold485863_14.2	1	p2	(GA)7	14	95	108	GGATTTGGGGGTGAGAAGAT
scaffold485868_10.4	1	p2	(AT)6	12	334	345	AGGAGCAAATTGAGGCTGTC
scaffold486007_10.8	1	p2	(AT)7	14	192	205	TTCGGGACCTCTTTGTATCG
scaffold486057_19.6	1	p2	(AC)7	14	319	332	GCGCATTTTCTGCTGTTAGA
scaffold486154_16.2	1	p2	(CT)6	12	106	117	TTCGATGTTGTAAGGGAGGA
scaffold486203_16.0	1	p2	(CT)7	14	270	283	TCCACCGCTAATGAATGACA
scaffold486204_11.5	1	p2	(GA)6	12	138	149	GGTGCAAGGGTTTCAAAGAG
scaffold486205_12.9	1	p2	(AT)9	18	226	243	CATATTTCAAATGCATGCCG
scaffold486206_21.5	1	p2	(GA)7	14	136	149	TAACACCAGCCTCTCTGCCT
scaffold486222_11.5	1	p2	(AT)7	14	400	413	TCGGCTATATTAGCGATCCG
scaffold486247_16.3	1	p2	(AT)11	22	51	72	ATGAAGTTAGGAAATAAAGAGGTGTT
scaffold486299_15.1	1	p2	(TA)8	16	333	348	TCTGATTCCAAACCCCAGTC
scaffold486692_16.8	1	p2	(TA)8	16	865	880	CTATTCCAACTTCGGGACCA
scaffold486706_10.7	1	p2	(TC)6	12	70	81	TTTCAGAAATTGCCTTGGTTG
scaffold486719_16.6	1	p2	(TA)6	12	2886	2897	GGGAACCATCATGGAAATTG
scaffold486816_17.7	1	p2	(AT)9	18	136	153	TTCGGAGCATGATTTAAGTGG
scaffold486903_14.8	1	p2	(CT)7	14	136	149	CCAATGCCAATCATCCTCTT
scaffold486948_16.5	1	p2	(GA)6	12	138	149	AAGGAAAAACATGAGTTTAGGGG
scaffold486991_12.4	1	p2	(AG)6	12	245	256	GCTGTTGCATTTCGATGTTG
scaffold487025_15.3	1	p2	(TC)7	14	1003	1016	ATCCAGATCCTTGTGGTTGG
scaffold487054_15.9	1	p2	(TC)6	12	168	179	TCCTATCTTCTGCGACGCTT
scaffold487173_18.7	1	p2	(AC)6	12	479	490	TTCCCTTCTCATTTCCATGC
scaffold487404_12.1	1	p2	(TA)7	14	234	247	TCAACGATTTTGGTTTGTTCC
scaffold487557_16.8	1	p2	(CT)8	16	5335	5350	ATTCTCCACCACCCCACATA
scaffold487593_16.9	1	p2	(AT)9	18	368	385	TGGAGAAGCTCTTACGGCAT
scaffold487797_11.1	1	p2	(GA)10	20	636	655	AATGGTTGTGGATCGCCTAA
scaffold487885_11.8	1	p2	(GC)7	14	261	274	TCCGGTCCAATTCCAATAAG
scaffold487974_14.8	1	p2	(AT)9	18	133	150	TTGCAGTAAATATCGGTTTTCG

scaffold487992_16.1	1	p2	(TA)6	12	352	363	AAAAATGAAACCGGCCAAA
scaffold488063_16.0	1	p2	(AT)8	16	2775	2790	GCCCTTCGAACAACAAAAAG
scaffold488176_15.2	1	p2	(TC)7	14	95	108	CCTCCACTGTTTTGTGCCAT
scaffold488196_16.4	1	p2	(GA)6	12	925	936	CTCGTGAAGCGACGTGAATA
scaffold488227_13.4	1	p2	(GA)6	12	736	747	ATGATGGAATTGGGTGTTGG
scaffold488246_18.7	1	p2	(AT)9	18	464	481	TGCTTTGATGTGTTGGCATT
scaffold488248_14.1	1	p2	(TC)6	12	242	253	ACAAGATCGAACGGAACGAG
scaffold488249_10.4	1	p2	(TC)7	14	46	59	GAAAATTCGAAAATTCCCCA
scaffold488306_11.3	1	p2	(GT)10	20	236	255	AGATAACGGCCTAGGCGAAG
scaffold488321_16.0	1	p2	(TC)6	12	295	306	TCACTCAATCTGATGCACACTC
scaffold488369_12.6	1	p2	(AT)6	12	307	318	TAATCTTGGCCGCTGATTTT
scaffold488429_14.0	1	p2	(GT)6	12	138	149	TAATTTTCGTCGGGATGGTT
scaffold488447_10.5	1	p2	(TC)8	16	84	99	TCTTTCTCGACGTTTCCTGC
scaffold488488_11.6	1	p2	(AT)6	12	1116	1127	TCAAATTTTGCGTAGCATCTC
scaffold488511_16.7	1	p2	(CT)6	12	60	71	TGAATCTGACTTCGATTATTTGTGA
scaffold488512_14.3	1	p2	(AG)6	12	917	928	CAATTGGTGCAAGAAGACGA
scaffold488592_16.4	1	p2	(CT)8	16	288	303	CTTGGGCTAAAATCCCAACA
scaffold488891_16.7	1	p2	(AT)10	20	486	505	CCAGGCAAAAATGCGCTA
scaffold488918_12.1	1	p2	(TA)6	12	1896	1907	CAAAAACCGGTGAAAAATCG
scaffold488989_16.6	1	p2	(CT)9	18	382	399	CACGCATTCTTCCAAACAAA
scaffold488994_19.3	1	p2	(CT)7	14	914	927	TTGGTAAATGTTTGGGCAGA
scaffold489067_16.2	1	p2	(TA)6	12	1332	1343	GGCGTGGTTTGCAGACTATT
scaffold489079_13.7	1	p2	(CT)11	22	155	176	TATGGTTTTTGGCCACTTCC
scaffold489104_14.6	1	p2	(CT)8	16	81	96	CATTCACATGGTCCCCTCTT
scaffold489143_16.1	1	p2	(CT)6	12	1362	1373	ATAGAGCCCCACGTTCACAG
scaffold489194_10.3	1	p2	(AG)6	12	245	256	AGGGTGCCAAGATTAACACG
scaffold489276_17.0	1	p2	(TA)6	12	132	143	ATATTCGAACGGCCTTGTTG
scaffold489281_15.1	1	p2	(AT)8	16	353	368	CACACTCAAGCACACACACG
scaffold489583_12.1	1	p2	(TG)7	14	1481	1494	AGCATTTGATTAATCCCCCA
scaffold489584_12.5	1	p2	(AC)6	12	422	433	AACAGCACGGACAGCTTCTT
scaffold489639_13.3	1	p2	(TC)6	12	482	493	CGTAGCGAAGAAGCGAGAAT
scaffold489656_14.7	1	p2	(GA)9	18	1137	1154	TGAGAGAGGAGACGCCATTT
scaffold489675_14.3	1	p2	(TA)9	18	772	789	TGATTGTAAATGAATTGAGTCGAA
scaffold489686_12.0	1	p2	(TA)8	16	535	550	AACAGGAACCGCTGATTTTG
scaffold489687_14.1	1	p2	(AT)6	12	138	149	GGTGCTTTGACTTATGTAAAGTTG
scaffold489894_12.0	1	p2	(GA)6	12	283	294	TGGAGATATGGTAGCGGTCC
scaffold489924_10.5	1	p2	(TC)6	12	65	76	TTGTTTTCTGTTCTGGCGTC
scaffold489950_15.5	1	p2	(CT)6	12	54	65	CGCGAGTTCATAACTCTCTCTC
scaffold490102_10.0	1	p2	(TG)8	16	176	191	AACTCAGCATACAGCAGTTTCA
scaffold490116_13.2	1	p2	(GA)8	16	1060	1075	GAATAATGGGGGTGATGTGC
scaffold490162_14.7	1	p2	(AG)10	20	460	479	TGGTTTCAATTTGGGTGGAT
scaffold490276_10.3	1	p2	(GA)10	20	184	203	ATTCGGAGCGAGGCTAAGTT
scaffold490293_10.1	1	p2	(GT)6	12	228	239	TCCCAACTCGTCAATCCTTC
scaffold490471_13.6	1	p2	(GA)6	12	93	104	GCTTCCATTTCACTTGAATGTTT
scaffold490493_15.5	1	p2	(TA)9	18	6282	6299	ATTGGGCCTATCAATGCAAG
scaffold490501_11.5	1	p2	(AT)6	12	832	843	AAGTGGTATAAACGGGCGTG
scaffold490513_12.5	1	p2	(GA)6	12	242	253	CGTTTGAGAGGAATCATGGC
scaffold490523_17.5	1	p2	(AG)10	20	48	67	GAATTAGCATACTGGCTCATTTATT
scaffold490557_15.1	1	p2	(AT)7	14	274	287	CGTTGAAGAGATTACGCAACA
scaffold490585_18.6	1	p2	(GA)6	12	87	98	AAAACGAATTTGGTGTTGGAA
scaffold490590_10.4	1	p2	(TA)10	20	685	704	CGGCCATCAGATATGGAGAT
scaffold490612_17.8	1	p2	(AT)7	14	3470	3483	TAACCACTCAAGGCCGGTAG
scaffold490622_10.5	1	p2	(TA)8	16	34	49	ACAAACTAATCAAGTTCCGCA
scaffold490635_15.9	1	p2	(AT)6	12	235	246	CTTTTCACACGAGGTCACGA
scaffold490652_17.4	1	p2	(AG)6	12	544	555	CACAGGATGATGCAGAGGAA
scaffold490705_15.2	1	p2	(TA)7	14	1036	1049	CGCGATTTTAAACCCTAAAGAA
scaffold490719_17.5	1	p2	(AT)6	12	87	98	GAAATTTCATTATTTCGTCGTTATT
scaffold490725_17.4	1	p2	(AT)7	14	138	151	CCAAAGAAAAGTGCATTACAAAA

scaffold490744_16.5	1	p2	(TA)7	14	964	977	TGGGAAAATTGAAGAACATTTG
scaffold490752_11.7	1	p2	(AT)7	14	1140	1153	CAAGCTGATTTCCCACCCTA
scaffold490840_12.5	1	p2	(GA)6	12	246	257	GATTTCTTGGTTCCGGATGA
scaffold490859_16.1	1	p2	(TC)6	12	1007	1018	ATTTGATCAACAGGCGATCC
scaffold491074_12.3	1	p2	(AG)6	12	752	763	CCAATGTGCACGCAAAATAC
scaffold491383_14.2	1	p2	(AT)6	12	361	372	AGCTGGGAGGCTTGGTAAGT
scaffold491520_13.1	1	p2	(TG)6	12	734	745	GTTCCCGCGTCATAAAAAGA
scaffold491538_15.4	1	p2	(CA)6	12	1702	1713	TGGAGAGGGCTAAACAGTCG
scaffold491562_12.3	1	p2	(GA)6	12	120	131	GAAGCGAGAAGGGAGGAACT
scaffold491578_14.6	1	p2	(TA)7	14	137	150	TATGCTCCTTTTGGTTTGGG
scaffold491579_17.3	1	p2	(AT)6	12	1773	1784	GGGCGCTTGTAGACTTTTCA
scaffold491600_13.0	1	p2	(TA)9	18	2131	2148	ACAACACATTGCCACACCAA
scaffold491613_11.3	1	p2	(TA)6	12	258	269	GCGTTGACGTACACAGCTTC
scaffold491696_15.3	1	p2	(TA)6	12	446	457	TGAAATATGTCTCCTCCGGC
scaffold491700_15.1	1	p2	(TC)10	20	1925	1944	ATCTCCACCTTCAATGTGCC
scaffold491790_20.4	1	p2	(CT)7	14	289	302	CAGCTGTCGAATCTGCTCTG
scaffold491806_10.3	1	p2	(CA)7	14	1066	1079	TGAGTGGTGTTGAGAAACGC
scaffold491910_15.8	1	p2	(AT)7	14	295	308	TTGAATTTTGTTGAAATTGGGA
scaffold492031_15.2	1	p2	(AT)8	16	3307	3322	AACGCGATATGGATGAAACC
scaffold492129_10.5	1	p2	(TA)7	14	121	134	GATATGAAGTGTCACAATATAAGCAT
scaffold492255_17.4	1	p2	(GA)6	12	153	164	CTCAGAGAGGTTCACGGTCC
scaffold492310_13.4	1	p2	(TA)6	12	140	151	ATTACATGAATGCCGAGCTG
scaffold492379_13.8	1	p2	(TC)7	14	174	187	AAGACCTTGTTGCCACGAAC
scaffold492455_16.5	1	p2	(AT)6	12	2148	2159	CGACGACTCAGTCAAACCAA
scaffold492474_13.4	1	p2	(CT)9	18	414	431	GGAACAAACCATCCTCAACC
scaffold492658_10.4	1	p2	(GA)7	14	54	67	GCGAAGCACCGAGACTACTG
scaffold492865_10.9	1	p2	(TC)6	12	309	320	TGGACTTGGCTAAGAATGGC
scaffold493039_17.1	1	p2	(TG)8	16	2472	2487	TCATTTCGGATGGAAGGAAC
scaffold493090_17.6	1	p2	(AT)6	12	54	65	CAGCACGAATCACACAGTAACA
scaffold493143_16.2	1	p2	(TC)7	14	4550	4563	AACTGACGGGTGATCAAAGG
scaffold493238_15.0	1	p2	(AT)9	18	133	150	TTATGAGTTTGCTCCTGGGG
scaffold493240_15.8	1	p2	(AT)8	16	135	150	TATATCACGGGGGACTCGAC
scaffold493258_13.7	1	p2	(AT)9	18	802	819	TGCCTCAATCTCCATAAATCAA
scaffold493345_10.7	1	p2	(GT)7	14	330	343	GCGTAGACGGAATAAAATTCG
scaffold493384_13.5	1	p2	(AT)6	12	222	233	CCAGGGAATCTTTTGAGCAC
scaffold493642_17.4	1	p2	(AT)7	14	585	598	CCAAATCGGGGACACATTAC
scaffold493686_13.5	1	p2	(TA)6	12	1557	1568	TGCATAATTGTAGATAATGGACCG
scaffold493704_14.8	1	p2	(CT)9	18	345	362	GGGAGTGAAGATTGGCAGAG
scaffold493801_19.9	1	p2	(TA)8	16	891	906	ACGGTGGGCACTACAAGAAC
scaffold493810_15.9	1	p2	(AG)6	12	538	549	GAAGCGGGATGAAGAATTGA
scaffold493822_11.4	1	p2	(TA)6	12	154	165	AAATATCGGCCGTCAGATTG
scaffold493907_10.7	1	p2	(TC)6	12	157	168	GCTTGGTTGGCTTGAGTTTC
scaffold493967_12.5	1	p2	(CT)7	14	431	444	CGGTCAACATGTCAGTGTCC
scaffold493993_14.6	1	p2	(CT)6	12	47	58	TCTCTCTTGGTTGGTGAAGAAA
scaffold494079_17.0	1	p2	(TC)9	18	497	514	CCTCTTGATTTTCTCGCTCC
scaffold494116_16.9	1	p2	(GA)8	16	301	316	TATGGTGATCGGGTTTGACA
scaffold494172_14.7	1	p2	(AT)6	12	72	83	CAAAACTTCACGAAGAAAACACA
scaffold494225_12.2	1	p2	(AG)11	22	31	52	AGAAACTTGACGTGTGCGTG
scaffold494246_10.7	1	p2	(TA)10	20	332	351	ATGCTTAAAAGAAAGCGGCA
scaffold494302_14.8	1	p2	(CT)8	16	2800	2815	CGGTGGATGTCTTGGTCTTT
scaffold494310_13.8	1	p2	(GA)6	12	114	125	AGATTTTCGGACATCGCATT
scaffold494403_10.0	1	p2	(GC)7	14	238	251	CGTTGCCGGATAATTCAAAC
scaffold494446_15.5	1	p2	(GA)6	12	69	80	TCCAGTATACAATTCCAACAGGAG
scaffold494453_10.9	1	p2	(CA)6	12	685	696	TAGTTTGTTTCCCGCCAAAA
scaffold494489_14.4	1	p2	(AG)6	12	93	104	TTTCCTTCTTGGATTATGCGA
scaffold494559_14.4	1	p2	(TC)6	12	70	81	CACCACACCCTCACTCTCCT
scaffold494580_11.5	1	p2	(AT)8	16	136	151	GGGGAATGTAACAGGGGATT
scaffold494593_10.7	1	p2	(AG)8	16	65	80	TGTTTTGTAATTTCCCATGTTTG

scaffold494604_10.3	1	p2	(TA)9	18	260	277	TGGTATCACATCACAACTTTTCA
scaffold494683_15.0	1	p2	(GT)7	14	1207	1220	CCGCTTTCTCATTTCTTCCA
scaffold494748_13.0	1	p2	(CT)6	12	557	568	CCCTTAGATGTCTTCATCCCA
scaffold494793_12.3	1	p2	(AT)8	16	534	549	AAAATGGTCCACATGTTGTAAAAA
scaffold494826_14.7	1	p2	(AG)7	14	197	210	TCAAAGTGGAGAAGGCATGA
scaffold494921_15.6	1	p2	(TA)9	18	172	189	TCGTATGCTGCCCCTAAAAC
scaffold495194_15.2	1	p2	(AT)9	18	649	666	TGTGCTATCCTAACCAAAAGC
scaffold495255_10.2	1	p2	(TA)10	20	91	110	GAAAAATCAAAGGCCCACAA
scaffold495354_18.1	1	p2	(TG)8	16	118	133	ATGAATTTTCTGCGAATGGG
scaffold495798_12.3	1	p2	(CT)7	14	237	250	GGGTGAATTTTTGGGGTCTT
scaffold495834_15.5	1	p2	(AC)6	12	67	78	ATTGCCCATGAATGTCACCT
scaffold496019_12.1	1	p2	(GA)6	12	471	482	AGAAATCAACCAATCGGCAC
scaffold496036_10.3	1	p2	(GA)6	12	70	81	CACAGAAGGAAAAGAGCGAGA
scaffold496548_15.0	1	p2	(TA)6	12	154	165	CTAGGCATGGAATCCGAGAA
scaffold496587_14.7	1	p2	(GA)7	14	79	92	CCACAAAGTTCCAATCTTTTAGC
scaffold496753_12.7	1	p2	(AT)10	20	86	105	TTACGGCATTAGAGATAATGATTAAA
scaffold496760_17.7	1	p2	(TA)6	12	164	175	TTCTAACAGAATAGAGCTTGGGG
scaffold496839_17.2	1	p2	(GA)6	12	583	594	ATATACGCCCACATGCGATT
scaffold496843_14.0	1	p2	(TA)8	16	137	152	TATGGCCAATTCCGAACTTT
scaffold496880_18.6	1	p2	(AT)6	12	91	102	TTTGAGCAGTTCCAATCTCTTG
scaffold496922_14.8	1	p2	(TA)8	16	694	709	TCTCGTACACGTGTCCAGTTTT
scaffold496962_14.8	1	p2	(AT)6	12	895	906	GAGGGTGGTATTTCGTGTCG
scaffold496983_14.4	1	p2	(TA)7	14	65	78	GGGTCATCATGGGATCAAGT
scaffold496994_15.3	1	p2	(GA)6	12	196	207	CTTAAGCCGAAAATTCCACG
scaffold497065_15.1	1	p2	(AT)6	12	787	798	TGGTGATTGAAATGATTAGCAAC
scaffold497090_14.1	1	p2	(AT)6	12	999	1010	ACATCCGGGAGAAGGGTTAG
scaffold497190_15.7	1	p2	(TC)7	14	1148	1161	GAAGCAACTTTCTTGGCTGC
scaffold497235_21.9	1	p2	(AG)8	16	193	208	GGCAAGCTTTCTCCTTCCTT
scaffold497267_12.9	1	p2	(TA)6	12	503	514	CAATTCCTCCCTTGGAGACA
scaffold497528_17.2	1	p2	(CT)6	12	1217	1228	ATCATCAGCCGCTTTATTGG
scaffold497539_11.6	1	p2	(AT)8	16	349	364	CATAAACCGTCGCACCTGTA
scaffold497540_16.0	1	p2	(GA)6	12	5527	5538	TGTTGCTTGTTTCTTGCACC
scaffold497601_13.1	1	p2	(TA)14	28	39	66	ATTAAAACCTCGCTCGGATG
scaffold497615_15.2	1	p2	(AC)6	12	2135	2146	CATAATAGTGCAGGTGGGGG
scaffold497649_15.6	1	p2	(AT)6	12	2529	2540	TAGGGGCCTGCTAAAATTCA
scaffold497663_10.2	1	p2	(TA)12	24	249	272	TTCGCTACATTTACGGATTGTG
scaffold497668_14.9	1	p2	(CT)6	12	342	353	GCGAGAACCACAAAGCTCTC
scaffold497695_16.0	1	p2	(AT)6	12	538	549	CAATTGTTGCAAACAACCAAA
scaffold497741_11.3	1	p2	(AT)6	12	411	422	TCGGTTGTTCCATCTCTAATCC
scaffold497742_12.3	1	p2	(AT)9	18	197	214	TGTTGGCGTTTTAATCTCCC
scaffold497882_16.0	1	p2	(AT)9	18	699	716	TCCCCATCTTCTTCAGTTGG
scaffold497895_11.3	1	p2	(TA)10	20	46	65	CCACATTTAAGGGTTATCACCTG
scaffold498040_20.9	1	p2	(TC)6	12	122	133	GGGATTCAATCTAGTCGACAATC
scaffold498167_14.7	1	p2	(GT)8	16	234	249	AGGGTGGTGAATGCTGAGAG
scaffold498519_12.2	1	p2	(AT)10	20	2042	2061	GAAATTAAGAATGTGGAGTGCAA
scaffold498665_11.6	1	p2	(TA)6	12	142	153	CCAAAGGTGAAAATCGCACT
scaffold498672_10.8	1	p2	(TA)6	12	141	152	TGAATTCAATTTATTTATTGTCGATGA
scaffold498673_15.0	1	p2	(AT)9	18	136	153	ATGTGTGATGTATCCCCGCT
scaffold498769_12.4	1	p2	(AT)6	12	126	137	GTTTTGCAGGATGGAACTCA
scaffold498841_13.0	1	p2	(TG)9	18	888	905	ATAACCCGGTCAACAAGCTG
scaffold498890_15.7	1	p2	(AT)6	12	1937	1948	CCACGTGGAAGGACTGAAAG
scaffold498933_16.1	1	p2	(AT)6	12	1153	1164	TTTGAGGGGTGGATTTGTTC
scaffold498995_17.1	1	p2	(AT)6	12	85	96	TCCGGAGTAAGTTCGAGGAT
scaffold499012_10.7	1	p2	(AT)6	12	89	100	TTGAGGCGGTACAAAGGAAC
scaffold499044_11.8	1	p2	(TA)8	16	281	296	CAAAGGATGAAAACGAGGGA
scaffold499085_10.3	1	p2	(AT)6	12	188	199	ATTGGCTGAGGCATTTGAAC
scaffold499133_13.0	1	p2	(TA)11	22	1641	1662	AGCCGTAGAGCCCTGTATGA
scaffold499136_15.4	1	p2	(AG)6	12	432	443	TCCTTTAGAGTCGTTGCACAAA

scaffold499167_14.2	1	p2	(TC)6	12	3263	3274	GAAAGATCTGGTCACGACAGC
scaffold499341_10.3	1	p2	(AT)9	18	324	341	AGAGGGGGTATGATGGGAAC
scaffold499418_12.4	1	p2	(CA)6	12	287	298	TGATTTGGCAGTTTGAGAGG
scaffold499517_10.1	1	p2	(AT)6	12	731	742	AACGTAAATGAGAAACATGCAA
scaffold499644_13.8	1	p2	(TC)6	12	2245	2256	ATTTCGAGCCAGACACTGCT
scaffold499710_13.7	1	p2	(TA)6	12	584	595	TTCCCTACAAAACGAGACCAA
scaffold499800_11.5	1	p2	(TA)10	20	169	188	TACGTAAAAACCCTTTCGCC
scaffold499852_15.0	1	p2	(AG)6	12	142	153	TTCCATGATCAGAACGGATTC
scaffold499993_16.3	1	p2	(TA)6	12	269	280	ATCACATCGGAAATTCGTCC
scaffold500074_17.1	1	p2	(AT)9	18	1224	1241	GGTGGGCAAATCAAATGTTC
scaffold500116_14.4	1	p2	(TG)9	18	3262	3279	GCAGAAGCGGGAACTAACTG
scaffold500121_13.4	1	p2	(TA)10	20	47	66	GGTGACAGGCAGCTGAATCTA
scaffold500173_16.8	1	p2	(AG)6	12	1296	1307	TTTGAACGAGGCGGATTTAG
scaffold500254_16.6	1	p2	(TG)8	16	476	491	TTTGACGGGGTAAAATAAAACAA
scaffold500268_11.4	1	p2	(TC)10	20	89	108	AAAAACATCGCTCTCTCCCC
scaffold500393_21.0	1	p2	(TA)6	12	113	124	TGGAACAGTTTGACACAACCA
scaffold500534_16.1	1	p2	(TC)6	12	290	301	TCGTAGTCCCCTTCAGATCG
scaffold500546_15.4	1	p2	(TC)11	22	587	608	ATGCTTGAAGCTATGGGTGC
scaffold500550_12.9	1	p2	(AT)11	22	573	594	TTCCCATCGCCCTACTTATG
scaffold500626_16.6	1	p2	(AT)7	14	3286	3299	ATCCCTCTACCGCCTTAGGA
scaffold500637_14.7	1	p2	(AG)9	18	4280	4297	CAAATTTCACCAAGAACGCA
scaffold500648_16.7	1	p2	(TA)8	16	3490	3505	AATCGAAAATAACGTTGGCG
scaffold500684_13.5	1	p2	(AT)9	18	807	824	CTTTTTGCTATGCAGCCTCC
scaffold500698_11.4	1	p2	(AT)9	18	608	625	AATCATAGCCACGCATGTCA
scaffold500701_14.9	1	p2	(AG)6	12	562	573	CCAATCGTCCTTGCTTCATT
scaffold500706_15.1	1	p2	(AT)6	12	2545	2556	TTTCGCTGTGCAATTCATTC
scaffold501002_10.8	1	p2	(TA)6	12	190	201	CTCACACACCCATATGTCCC
scaffold501014_15.0	1	p2	(CT)6	12	524	535	AAATTGATTTCCCTCCACCC
scaffold501038_11.4	1	p2	(AT)6	12	927	938	AACACATATGGGGACACGCT
scaffold501049_15.9	1	p2	(TC)7	14	3899	3912	TGCGACGTATTGAGAAGCAG
scaffold501077_18.4	1	p2	(AG)6	12	1563	1574	ATGTTCTCTCAGGCCACCAC
scaffold501119_15.7	1	p2	(AG)7	14	219	232	TGGGTGAAAAGAAAAGGCAC
scaffold501146_13.3	1	p2	(TC)7	14	1206	1219	AATATCGCCAAACACCTTGC
scaffold501159_11.4	1	p2	(AG)10	20	137	156	CAAGTCAACAAGCAAGCCAA
scaffold501161_16.1	1	p2	(AG)8	16	2477	2492	CCATCTCGAAAAGATGGGAA
scaffold501214_13.0	1	p2	(TA)7	14	453	466	TGGCTAAAGATCGATGTGACTT
scaffold501282_13.6	1	p2	(TA)9	18	662	679	CGTGAATATGCTGATGCTGG
scaffold501288_11.8	1	p2	(AT)6	12	56	67	TACAAGTGCAGCGGAAATGA
scaffold501475_16.0	1	p2	(CT)14	28	197	224	GGTTATGGTTCTTGATGGGG
scaffold501482_16.3	1	p2	(CT)9	18	202	219	GCGGTGGTGGAGAGAAAATA
scaffold501598_12.8	1	p2	(TA)8	16	772	787	ATGCATCGGGGCAATATTTA
scaffold501608_15.9	1	p2	(TC)8	16	659	674	CCTCCCTCAGATGCAAACTC
scaffold501615_14.7	1	p2	(CT)8	16	221	236	ATAAGGTGGCGATGAGTTGG
scaffold501624_11.7	1	p2	(TC)6	12	106	117	AGGACACGTTGGACCTGAAA
scaffold501678_16.0	1	p2	(TC)6	12	123	134	GTACCGCTGGATAATCCCCT
scaffold501687_14.7	1	p2	(TA)7	14	693	706	TTACGGGAGAATTCACGACC
scaffold501754_16.4	1	p2	(TG)7	14	140	153	TGGTTGTCACATCTTCATGC
scaffold501867_16.5	1	p2	(CT)8	16	1872	1887	GTCAATGTGGGTGCTGTGTC
scaffold501958_18.1	1	p2	(TA)9	18	1395	1412	TGCATGGTCTTCGAAGTAGG
scaffold502044_15.4	1	p2	(CT)7	14	271	284	TCGCATGCAACGATAGTAGG
scaffold502048_12.6	1	p2	(GA)8	16	232	247	TCAACGTCTTCAACGCAAAG
scaffold502063_18.6	1	p2	(AC)6	12	574	585	ACTTCACCGCCAAAACTCTG
scaffold502098_14.6	1	p2	(TA)9	18	326	343	TGATACATAGTGTTATTCTTTGGCG
scaffold502136_10.6	1	p2	(AT)8	16	86	101	GGATCTATTACTAACCGCCCC
scaffold502171_18.3	1	p2	(AT)6	12	809	820	TATCAGGCTGCCTACTCGGT
scaffold502221_14.0	1	p2	(AG)6	12	749	760	GCAGGACAACTACCATACGGA
scaffold502222_13.9	1	p2	(TC)8	16	1577	1592	TGAGGAAGAAGGGATGATGG
scaffold502297_14.1	1	p2	(AT)8	16	1679	1694	ATTGAGGCGTGTTTAGGCAT

scaffold502410_15.7	1	p2	(TA)8	16	2420	2435	TTTGAGGTGGGCCAAAATAG
scaffold502515_13.2	1	p2	(AT)9	18	533	550	AAAGCAAACCCATGTTCTTCA
scaffold502589_14.9	1	p2	(TC)8	16	4487	4502	GTGCTGTTAAGCATCCCCAT
scaffold502636_16.0	1	p2	(AT)7	14	141	154	GTCCTCCGAACTCATGGTGT
scaffold502669_15.4	1	p2	(GA)7	14	424	437	CGCAATCGATTTTCAAACCT
scaffold502792_12.6	1	p2	(AG)7	14	98	111	AAATGCGCCTATCACTTTAATTC
scaffold502861_18.6	1	p2	(TC)6	12	625	636	CCCAAACTTGGTTGCTCTTC
scaffold503032_17.9	1	p2	(TG)6	12	2089	2100	GCTATGAATGGCATCGAAGA
scaffold503122_17.7	1	p2	(TA)7	14	401	414	TGCAACTTTTGTACTCAGCCC
scaffold503129_17.0	1	p2	(TA)6	12	2315	2326	TTCCCAAAATAACCGTCAGC
scaffold503262_14.8	1	p2	(GT)8	16	32	47	ACTGACATTCACAGGCATTGT
scaffold503293_10.2	1	p2	(AT)6	12	265	276	TTGTAGCCCCTTTGAGCCTA
scaffold503315_10.3	1	p2	(TG)6	12	138	149	TCCTCAGAAACTTCAACGCTT
scaffold503338_19.9	1	p2	(TC)6	12	333	344	ACTATCGACGTCCGGAGCTA
scaffold503385_11.9	1	p2	(CT)6	12	88	99	GTCGTTTACTCTTTCCGGCT
scaffold503389_11.0	1	p2	(CT)7	14	581	594	TGAAATGGATCACATGCAAAA
scaffold503415_16.3	1	p2	(TA)8	16	204	219	TGACGAAAATTGCTTTGATCC
scaffold503417_14.9	1	p2	(GA)10	20	981	1000	ACCCAAAACCTCCCATATCC
scaffold503544_12.5	1	p2	(AT)7	14	53	66	AGATTGGGCTATTCGAGCCT
scaffold503698_16.1	1	p2	(TA)8	16	139	154	GGAAACAAGTGGATGACGGT
scaffold503721_15.7	1	p2	(TC)8	16	1647	1662	CAGTTTTGTGTGTCCATGTGC
scaffold503724_13.2	1	p2	(CT)6	12	247	258	GTTGGATAATACATGGCGGG
scaffold503840_14.2	1	p2	(CT)12	24	38	61	TTTCGAATTTTTCCTCTTTTGTG
scaffold503850_14.7	1	p2	(TA)8	16	1483	1498	TTGATCCTTCCTAGGATAACCG
scaffold503862_10.3	1	p2	(GT)6	12	25	36	GCGTGTGTGTAACCGTGTGT
scaffold503887_13.9	1	p2	(TA)10	20	875	894	AAATCGAGGAGCTGTCATCC
scaffold503971_10.0	1	p2	(AT)7	14	857	870	ACTACGTCGATCCAACGGTC
scaffold504093_15.2	1	p2	(CT)6	12	254	265	GGCTCGGTATAAAAATGGCA
scaffold504119_15.8	1	p2	(TA)7	14	1505	1518	CACAATCCATCTTCAACCCA
scaffold504136_17.5	1	p2	(GA)6	12	4755	4766	GACCCAAATGATTGTACGGG
scaffold504224_22.0	1	p2	(AT)6	12	358	369	GCTTAAAGTGCGGCCAAA
scaffold504275_17.0	1	p2	(TG)7	14	689	702	GTATGGCGTGGTTCCAGTTT
scaffold504299_17.7	1	p2	(AG)6	12	768	779	TAAGTTGGGCCTCTCGATCA
scaffold504329_10.6	1	p2	(AT)8	16	104	119	ACGAAACACGGAAAAAGCAT
scaffold504357_11.6	1	p2	(TA)12	24	477	500	CAATGATGATGCCAATCCAG
scaffold504370_11.1	1	p2	(TC)6	12	106	117	CAAATGGCCGAGAGGATTTA
scaffold504413_13.4	1	p2	(AG)7	14	210	223	CGCAGCAAAGTAGCATACGA
scaffold504424_10.9	1	p2	(TA)8	16	47	62	TTTAGTGTTTTGACATGATTACTCG
scaffold504517_10.7	1	p2	(AT)7	14	449	462	GCGTGTGGGAGTTGGTAAAT
scaffold504529_21.6	1	p2	(TA)7	14	64	77	ATGAACTAATCAAGTTCCGCA
scaffold504533_15.6	1	p2	(AT)8	16	3402	3417	TAGTAGGGTGCAGGGGATTG
scaffold504565_12.4	1	p2	(TA)7	14	206	219	CCCGATAGGCTTGAAGGATT
scaffold504687_11.6	1	p2	(AT)8	16	91	106	TTACCTACATGGCCCTGCTC
scaffold504752_15.2	1	p2	(TC)8	16	871	886	CGTGAAGTCCATCTCCCACT
scaffold504790_16.1	1	p2	(TC)6	12	308	319	ATTAATAAAATTTGGAATGATAGAGG
scaffold504791_12.5	1	p2	(TA)7	14	227	240	AAACTACCCTTGATCGCACG
scaffold504801_16.8	1	p2	(AT)7	14	405	418	CGACTCAGTCATGCACCATT
scaffold504806_18.0	1	p2	(AT)10	20	46	65	TGAAGTTAGGAATTAAGGAGGTGTT
scaffold504837_15.4	1	p2	(TA)6	12	5356	5367	TTCCTCACGAATAAACCAAGC
scaffold504907_16.7	1	p2	(TG)8	16	832	847	GAACGATTTGTTATCGAGGGA
scaffold504910_16.9	1	p2	(TA)6	12	143	154	TTTGACTATGTCTGCGTGCC
scaffold505032_13.0	1	p2	(AT)6	12	1345	1356	CCTACAAAGCCACTCTAAGCG
scaffold505057_15.0	1	p2	(CA)11	22	2040	2061	TCGAGTTGTACCGACACTGC
scaffold505394_15.7	1	p2	(AG)6	12	817	828	CTGAAAACTGCAGCACGAGA
scaffold505496_11.0	1	p2	(TA)10	20	547	566	TCACAAGTGCAAGCACAATTC
scaffold505505_10.8	1	p2	(TA)6	12	221	232	CTCGTCTCAAGAAGGTGCTTT
scaffold505643_16.0	1	p2	(TA)7	14	743	756	AGCGCTGAATGTCATTGGAT
scaffold505737_12.8	1	p2	(TA)10	20	382	401	TGGGAAGGGTTGTTACTGGA

scaffold505831_15.3	1	p2	(TA)6	12	1282	1293	TGATGGCTGTGAATTGGAGA
scaffold505873_23.0	1	p2	(TC)7	14	78	91	CCCTAGCTTTGCCCTCTTTC
scaffold505889_11.5	1	p2	(AT)8	16	1400	1415	TCAAGTAAGCGAGATTTTCTACATTG
scaffold506111_17.1	1	p2	(AG)8	16	50	65	TGATATCTTTGAAGAACAATTACAATC
scaffold506223_17.4	1	p2	(AC)7	14	5021	5034	TTTGGCCGAGTTCGGTATAG
scaffold506265_13.5	1	p2	(AT)12	24	1247	1270	AAAAAGGGAACAAAAACGGC
scaffold506456_16.8	1	p2	(AT)6	12	51	62	TGATGATGAGATTTTAGTCCAAGG
scaffold506478_16.1	1	p2	(TG)6	12	2987	2998	CCTTCTCCGTCTGCAAACTC
scaffold506694_14.1	1	p2	(GA)6	12	143	154	CACAGCCCTAATGCTACGTG
scaffold506824_16.2	1	p2	(TA)8	16	658	673	AGATCTGGGCAATGTGTGGT
scaffold506871_17.6	1	p2	(GA)8	16	55	70	GAGCTGGTTTGAGGGAGATG
scaffold506980_12.8	1	p2	(AT)6	12	55	66	CCAATTTACTAATTCTTGATGAATCC
scaffold507005_14.1	1	p2	(AT)6	12	1533	1544	GTGGTCTAGGATGGGAGCTG
scaffold507055_10.6	1	p2	(TA)6	12	186	197	GGGGTGGGAATTAAACACCT
scaffold507056_14.2	1	p2	(AT)10	20	81	100	CGCATCCGACTCCTCAATTA
scaffold507062_15.7	1	p2	(AT)12	24	1396	1419	TGAATGGGGATCAATTCCTT
scaffold507138_18.5	1	p2	(TC)6	12	195	206	CGCCCTCCACTGTTAATGTT
scaffold507150_15.8	1	p2	(CT)8	16	139	154	AGGGATCCCAAGGCACTACT
scaffold507161_13.9	1	p2	(TA)7	14	192	205	GGCATTTTGGGAAAGACAAA
scaffold507240_12.7	1	p2	(TC)6	12	237	248	ATGATCGCCACAAACATCAA
scaffold507312_19.1	1	p2	(AC)7	14	656	669	CACCATCAACCTCACCACCT
scaffold507569_10.8	1	p2	(TA)8	16	50	65	TGAAAGAAGTGGTGGTGGAG
scaffold507847_12.6	1	p2	(TA)9	18	170	187	GACATGCAGAGTTCAACAGCA
scaffold507850_14.1	1	p2	(AC)7	14	307	320	GGGGTAAGACCCACCTCATT
scaffold507862_18.3	1	p2	(CT)6	12	109	120	TAGCAGCCCACAATTCAACA
scaffold508070_14.7	1	p2	(GA)6	12	835	846	CCGGAGAAGTTTGTGGAGAG
scaffold508180_16.1	1	p2	(AT)7	14	1091	1104	GGACGCTGACCATCACTGTA
scaffold508197_17.7	1	p2	(TC)6	12	144	155	TCAACATTTTTCCCAGAATCG
scaffold508364_14.5	1	p2	(TA)7	14	660	673	ATCATCAGTAGCGCCACCTT
scaffold508417_10.5	1	p2	(AT)9	18	503	520	TCAAATTCGAAATTCGGACC
scaffold508434_12.2	1	p2	(TA)7	14	135	148	TCTGAAAATCAAAGGCCCAC
scaffold508517_14.3	1	p2	(CT)7	14	414	427	TACGTTCTTCCCCGTTTTTG
scaffold508523_10.0	1	p2	(TA)6	12	211	222	TGGTTGGAGTTGTCAGTTCCT
scaffold508668_17.2	1	p2	(GA)6	12	532	543	CGAAACCCCTTTCTCTCTCC
scaffold508676_20.9	1	p2	(CA)6	12	211	222	TCTCCCTCGGTAATTCCCTT
scaffold508826_16.8	1	p2	(AT)9	18	194	211	GGGGTGTATTAAAATAGGAGCA
scaffold508912_16.2	1	p2	(GA)8	16	2696	2711	CCTCCATCATGAAAACCGAG
scaffold508935_15.1	1	p2	(CT)6	12	457	468	CTCCTCTGCACCAACCTCTC
scaffold508982_16.9	1	p2	(AT)7	14	644	657	CACAAAACTCATACCCGTGC
scaffold509015_13.1	1	p2	(AG)6	12	100	111	CCAAAATAGATGGCATTCAAGA
scaffold509043_14.6	1	p2	(CT)6	12	72	83	TGGAAGTTCTCTCCTCTCGC
scaffold509125_16.4	1	p2	(AT)9	18	398	415	ATCGTGGGCGTAAATCATGT
scaffold509145_13.7	1	p2	(AT)7	14	71	84	GGTTATGACTTGATCATGACCCT
scaffold509161_10.2	1	p2	(TA)8	16	209	224	GCATATCAAATGGTGTTTGGG
scaffold509190_13.9	1	p2	(TA)10	20	218	237	GGTGGCGTATGCTGAGAGAT
scaffold509321_17.6	1	p2	(TC)6	12	365	376	TCTCTTTCGTCCTTTTTCCC
scaffold509346_15.3	1	p2	(TA)8	16	141	156	TTCCCGAGTAAGATGGATGG
scaffold509350_14.8	1	p2	(TC)6	12	269	280	TTCCCTCACTCCTCTTCTGC
scaffold509352_18.8	1	p2	(CA)6	12	73	84	GAACACACGAAGATTGGGAGA
scaffold509453_10.6	1	p2	(CT)7	14	661	674	CAAATACGACGGAAAATCGG
scaffold509514_16.5	1	p2	(AT)8	16	70	85	AATGAAATCTTACTACTCCAAAAATCA
scaffold509822_12.7	1	p2	(TA)8	16	301	316	TCTTTCGGCTGACTTTTTCTTT
scaffold509990_13.5	1	p2	(TA)6	12	309	320	AAGATCTACGTCCACGGGAG
scaffold510106_11.7	1	p2	(TA)8	16	482	497	TGACTGCCCAAGAAATCACA
scaffold510120_20.6	1	p2	(AG)6	12	993	1004	TGATTACTTCCGCACCATTG
scaffold510258_19.2	1	p2	(GA)7	14	1703	1716	AACAGAAAGCCACACATGTCA
scaffold510339_12.2	1	p2	(TA)9	18	44	61	CGGATTTAAGGGTTATTACCTGAT
scaffold510348_16.0	1	p2	(CT)6	12	88	99	CCTTCTTTTCACGCATTTCC

scaffold510359_17.	1	p2	(TA)8	16	576	591	TTTGCCGACCTTATCTCCAC
scaffold510432_13.	1	p2	(AC)9	18	559	576	TGGCTTCAGCAGACAATGAG
scaffold510436_10.	1	p2	(TA)7	14	143	156	ACGAGATCGAAGGGAGACAA
scaffold510443_15.	1	p2	(GC)6	12	516	527	TCACGATGCGTCACAAGTTT
scaffold510476_17.	1	p2	(AG)6	12	3397	3408	TGTTTGGTAGCCCACACAAT
scaffold510607_11.	1	p2	(TC)7	14	257	270	TAAGGCAAAGCACGTGAGTG
scaffold510664_13.	1	p2	(AG)7	14	1425	1438	TCAGATGAGTTGAATGGAACAGA
scaffold510665_12.	1	p2	(TA)8	16	711	726	CACTCGCATAAGTATGCATGAAA
scaffold510688_15.	1	p2	(AT)8	16	501	516	CACTCACACACACGCACTTG
scaffold510755_14.	1	p2	(TA)6	12	92	103	CAAGAGCTTGAGCTCGGAGT
scaffold510875_12.	1	p2	(AT)9	18	399	416	TTTCAAATTGCATCCTAGCAAA
scaffold511000_16.	1	p2	(CA)6	12	145	156	GAGAGTGGAGGCCATTTCTG
scaffold511062_19.	1	p2	(CT)9	18	519	536	TCAACAACCAGTCCACTCCA
scaffold511146_10.	1	p2	(TA)9	18	327	344	TGCGTCAAAAGCTCTTCAAA
scaffold511185_10.	1	p2	(TA)8	16	145	160	TCCAGATTTGTTCCTACGCA
scaffold511230_15.	1	p2	(GT)6	12	238	249	CCACAAATCCCTTCATTTGG
scaffold511392_14.	1	p2	(AT)9	18	699	716	CTCAAATTGGAATTGGGCTG
scaffold511413_16.	1	p2	(GA)9	18	47	64	GGGAGAAGAGAGAGTTAGGGTGA
scaffold511442_16.	1	p2	(GA)6	12	233	244	TGGAGCTTTGATCCTTGCTT
scaffold511512_13.	1	p2	(AG)6	12	55	66	AACCACCTTTACAAATGAGCTAGT
scaffold511567_17.	1	p2	(CT)7	14	56	69	TCACTTCATTACGAAAAACACCA
scaffold511640_14.	1	p2	(AC)7	14	2437	2450	TGTGTCATCTAGTCCGGTGG
scaffold511663_15.	1	p2	(TC)7	14	2401	2414	TCCATTTCCTCCACCGTTAG
scaffold511734_13.	1	p2	(AT)11	22	1312	1333	CACCGACCAAACATATGCAC
scaffold511842_15.	1	p2	(TA)9	18	241	258	AAAGTCATCCGCTATCTCGAA
scaffold511891_13.	1	p2	(TA)9	18	104	121	TTTTCATTCTTGACCCCTTCTC
scaffold511919_10.	1	p2	(TG)9	18	50	67	AAGCTGCATGCATTTCAGAG
scaffold512007_14.	1	p2	(AG)6	12	129	140	GCGCCGCCTATCACTTTAAT
scaffold512067_16.	1	p2	(AT)8	16	782	797	AGCGACGAACGGAAAAAGTA
scaffold512207_18.	1	p2	(AT)7	14	144	157	CCACATTTTTCCCGATTGTT
scaffold512216_15.	1	p2	(GA)9	18	1327	1344	AATGAGCAAAACGGTCCTTG
scaffold512392_13.	1	p2	(CT)7	14	62	75	ACAAAAAGGGATGAGCGACA
scaffold512399_21.	1	p2	(AG)9	18	140	157	TGTTTCCCATTTTCACCACA
scaffold512434_14.	1	p2	(TC)8	16	96	111	CATGAGGCCCCAAGATAATG
scaffold512565_18.	1	p2	(TA)6	12	252	263	TCTGCAAATGTCACACGTCA
scaffold512604_13.	1	p2	(AC)8	16	1184	1199	CCAAGAGGAGTTGAAGTCGC
scaffold512679_10.	1	p2	(TA)7	14	487	500	CACCGCCAAATATCACCTCT
scaffold512742_17.	1	p2	(CA)6	12	253	264	ACGTGGTCTCTCCCCTTTTT
scaffold512772_17.	1	p2	(CT)6	12	1280	1291	AAGAGGAGAGACGGAGAGGC
scaffold513022_10.	1	p2	(TA)10	20	172	191	TGCTGTGAATTGTCCAGCTC
scaffold513095_13.	1	p2	(CT)6	12	436	447	GAAGTAAACGGCCAAAGTGC
scaffold513106_11.	1	p2	(AT)7	14	66	79	CGAAATCTTACTTTGCGGCT
scaffold513134_13.	1	p2	(GA)6	12	1557	1568	CATCAAGTTTCCCAAGGCAT
scaffold513238_14.	1	p2	(TC)7	14	30	43	TGGTTGGTGGAAGAAATCAA
scaffold513266_15.	1	p2	(TA)6	12	704	715	TTAAATACGACCGGGCTCAC
scaffold513300_14.	1	p2	(CT)13	26	2032	2057	TGCCCAAAAACATTTCAACA
scaffold513337_12.	1	p2	(TA)12	24	87	110	AGGGGTTATCACCTGAATGC
scaffold513383_13.	1	p2	(AG)6	12	168	179	TGTTATCCTTTGTGAGCCCC
scaffold513562_12.	1	p2	(GA)6	12	830	841	ACCATCGCTGTTACCACCTC
scaffold513704_13.	1	p2	(TA)10	20	138	157	CTGACATTTGACCTGAGCGA
scaffold513706_18.	1	p2	(AG)6	12	94	105	TTGGAAAATAACATGCATAGAAAA
scaffold513797_13.	1	p2	(TA)9	18	278	295	CCGAAACCAGAATGAGAACC
scaffold513800_10.	1	p2	(TC)6	12	542	553	GCCGTAGTAACTCCAGCCAC
scaffold513804_12.	1	p2	(TA)6	12	547	558	CACTAGCAGGTCGACACGAA
scaffold513852_16.	1	p2	(AT)6	12	468	479	TTCATCTCAAATTTCATTGTGGA
scaffold513959_16.	1	p2	(GA)10	20	2736	2755	TAGCTTTCCCCCACATACCA
scaffold513983_14.	1	p2	(TA)7	14	348	361	CGAATGAAATTATTTGTTTTCCC
scaffold514008_11.	1	p2	(TA)8	16	198	213	CCCACATTAACACCCAATCC

scaffold514093_13.(	1	p2	(TA)7	14	572	585 GGCCAAGTTGGTGAAGTAGG
scaffold514102_15.:	1	p2	(TC)6	12	585	596 TAAAAACACAGCCTCCCTGC
scaffold514257_18.(	1	p2	(AT)6	12	237	248 CGTCGTTTCGTGTTCTTTCA
scaffold514268_13.2	1	p2	(TA)6	12	236	247 TCATGGTTGGGTGTTTTTATCA
scaffold514361_13.7	1	p2	(AT)9	18	93	110 TTGATCCAAGGGTGTATAATGG
scaffold514373_13.4	1	p2	(TA)9	18	406	423 CGCTCAGGTTTGAATCTGTTT
scaffold514392_15.7	1	p2	(CT)6	12	1253	1264 CATGGTTGCAAAGGTGATTG
scaffold514411_12.9	1	p2	(CT)6	12	2164	2175 GGAACAACTGCACCGAATTT
scaffold514424_16.5	1	p2	(TA)7	14	5081	5094 CTGGTGCAATGCTTGAGAAA
scaffold514512_17.8	1	p2	(AT)6	12	501	512 GAATGTCATGAGTTGAGGCG
scaffold514571_12.2	1	p2	(TA)7	14	569	582 AAACTCCCGCTCAAATAGCA
scaffold514639_15.5	1	p2	(AT)7	14	1623	1636 TCTGTTTGCGAAATTTGTTGA
scaffold514667_13.3	1	p2	(TA)6	12	168	179 TGTTTCGGACTAAACTGGCG
scaffold514668_16.(	1	p2	(AT)6	12	881	892 AGATTGGGCTATTCGAGCCT
scaffold514758_18.2	1	p2	(TC)6	12	302	313 ACTTTTGCGTCCTTTGTCGT
scaffold514788_13.2	1	p2	(TA)8	16	50	65 ATGAGTTATCACCGGATCGC
scaffold514794_15.2	1	p2	(AG)6	12	92	103 TGCTACGGTTTCTTCTTCCG
scaffold514822_15.(	1	p2	(GA)8	16	2999	3014 ACGATCGCCCACTTATTGAG
scaffold515106_12.9	1	p2	(AT)8	16	143	158 AGCTTGTAGAACGAGCCGAG
scaffold515216_16.7	1	p2	(GT)7	14	2903	2916 CAAAGCATGAACAAATGCGT
scaffold515220_17.8	1	p2	(TA)10	20	250	269 AATTGCTGGCAAATCGTAGC
scaffold515228_13.4	1	p2	(AG)6	12	418	429 TGAAGCAATCAAAAACGGTG
scaffold515241_18.3	1	p2	(AT)7	14	691	704 GGTCCAACTCCGATTCAAAA
scaffold515309_17.8	1	p2	(CT)8	16	52	67 TTGATTGTAATTCTCTGCACCA
scaffold515425_10.5	1	p2	(GA)6	12	55	66 AAAAACTGTAGTGGGTTAAAATTCA
scaffold515431_13.7	1	p2	(AT)7	14	145	158 TGTGACGAGCTTATGAAACTATACG
scaffold515448_11.4	1	p2	(AT)8	16	143	158 TATTAACCCTTCATTGTTTTTAAATG
scaffold515502_10.(	1	p2	(AG)8	16	52	67 CAAATGAGCATGAGGGAGGT
scaffold515512_16.2	1	p2	(TA)8	16	88	103 AACGGCCGAGATTTGTTATG
scaffold515658_21.4	1	p2	(CT)6	12	40	51 TTGATTCCTCCCCACAATTC
scaffold515689_15.7	1	p2	(TG)6	12	481	492 GTATGCTGACGTCCTCACGA
scaffold515852_18.:	1	p2	(TC)7	14	4205	4218 CAGAAGCAAGATTCGACCAA
scaffold515866_11.(	1	p2	(TA)7	14	266	279 TTTCTCCCCATCGATAACCA
scaffold515898_16.4	1	p2	(CT)8	16	1073	1088 CTTGGGATGGGGGAGTATTT
scaffold516004_13.(	1	p2	(AG)6	12	440	451 CCCAGTATCCCAAACTCGAA
scaffold516047_15.2	1	p2	(GA)8	16	1030	1045 TGCTTTTCCATGGTGACTGA
scaffold516215_17.2	1	p2	(AT)6	12	2072	2083 TGAGAACCAAACACAAATGTCA
scaffold516234_18.8	1	p2	(GA)8	16	898	913 CCGAGAATCAGGACTCGAAA
scaffold516363_11.(	1	p2	(AG)11	22	295	316 GGTATTGGCAAATGCAGGTT
scaffold516543_16.8	1	p2	(TC)7	14	246	259 AAACGCACCAATTTCCTTCA
scaffold516577_15.:	1	p2	(GT)6	12	318	329 TTTGACATGATCACACCCCT
scaffold516598_15.(	1	p2	(AG)6	12	147	158 CGACTGGGCCTATCCTATCA
scaffold516654_13.3	1	p2	(CT)6	12	102	113 GCCTTCTAGCTTCTCTTGCTTTC
scaffold516814_12.4	1	p2	(AG)8	16	690	705 TAGATGCGTGCATAATTGCC
scaffold516967_13.(	1	p2	(AT)9	18	221	238 TGTTTAAATGCACACCCCTTT
scaffold517060_15.9	1	p2	(AG)6	12	1873	1884 ATCGAACCGTTTTTCTGTCG
scaffold517061_14.8	1	p2	(CT)6	12	55	66 GCCTTCTAGCATCTCTTGCTC
scaffold517082_14.(	1	p2	(CT)7	14	50	63 CACTCAATTCATTTCACCTCCA
scaffold517277_10.7	1	p2	(AT)10	20	180	199 TCTCTCCACCAAATGTCCAA
scaffold517291_13.5	1	p2	(AG)6	12	109	120 GCGGCACACGAGGTATAAAA
scaffold517306_17.5	1	p2	(TA)6	12	436	447 TTTGTGGTATCAGAGCCACG
scaffold517487_12.2	1	p2	(CT)6	12	69	80 TCTTTTGCTATGTCCTCACTCG
scaffold517510_15.9	1	p2	(GA)6	12	98	109 CAGGGAGGCAAAAAGAGGTT
scaffold517547_16.(	1	p2	(AT)6	12	169	180 TGGGATATAGCGAGTTTGGTG
scaffold517585_10.2	1	p2	(TA)6	12	165	176 TGGGGTTTTACAAGAATGCC
scaffold517610_10.:	1	p2	(GT)7	14	1677	1690 GTGCGTGTGAGTGTGTGTCA
scaffold517667_14.:	1	p2	(CT)9	18	1349	1366 CCGTCTTCTTCTCCATTCCA
scaffold517741_14.4	1	p2	(TA)8	16	1571	1586 TGGGATGAAAATAGGGGAAA

scaffold517769_20.3	1	p2	(TA)6	12	51	62	TGACGGGATTTAGGTGTTTTG
scaffold517790_11.4	1	p2	(CT)6	12	76	87	AAGGGGGATCAAATCTAGTCTTTT
scaffold517856_12.5	1	p2	(TA)8	16	395	410	CATTCAAGGGGCATTTTTGT
scaffold517861_16.2	1	p2	(CA)9	18	2609	2626	GCATAGTGCTCCAAAAAGGC
scaffold517907_12.4	1	p2	(CT)6	12	93	104	GCCATCTCTCCCTAACCTCC
scaffold518056_15.6	1	p2	(TC)6	12	95	106	AGCATCTCTTGCTCTCTCGTT
scaffold518062_13.3	1	p2	(AG)6	12	503	514	TCGTCTTCGTTCTCCGTCTT
scaffold518123_14.3	1	p2	(TA)6	12	53	64	TTTCTCACTATAAGGTGGTGTTCC
scaffold518166_15.7	1	p2	(TG)8	16	1352	1367	GAATACTTGCCTCAGCTCCG
scaffold518253_11.3	1	p2	(TC)6	12	368	379	ATTGAATCTACGTCCACGGG
scaffold518271_14.3	1	p2	(AG)7	14	494	507	GCCAGCTTTTTCTTCTCCAA
scaffold518297_17.7	1	p2	(TA)6	12	147	158	CCCTTTACCTCACTGGTGGA
scaffold518305_13.8	1	p2	(CT)6	12	365	376	TCACCCCCATCTTCTCTCAC
scaffold518461_15.1	1	p2	(GA)6	12	1040	1051	CAGGTTCTCATCGCTCTGTG
scaffold518562_16.7	1	p2	(AG)6	12	889	900	CATGGTGATAGAGCCGGATT
scaffold518631_16.0	1	p2	(AT)6	12	48	59	CTTCTCGTGTCCGACTCCTC
scaffold518633_16.7	1	p2	(AT)7	14	146	159	AATCGAGGGCGAAGAGAAAT
scaffold518685_15.4	1	p2	(AT)6	12	682	693	AAAATATGGGGTTTTCCCAAA
scaffold518832_17.8	1	p2	(AT)6	12	4862	4873	CTTGGGCTTTCATGACGAAT
scaffold518849_13.4	1	p2	(TA)8	16	244	259	AACCCAAGACCTCAAGCTCA
scaffold518856_16.0	1	p2	(TG)7	14	1387	1400	TGTTGAAATCTTGAACGGGTC
scaffold518881_10.0	1	p2	(TA)8	16	181	196	AAAAACGCGAATTTTTAGGG
scaffold518886_14.6	1	p2	(AT)10	20	39	58	TATTTGTGCGTGCACCTGAT
scaffold519004_15.3	1	p2	(AT)7	14	1051	1064	AGCACTGAGCAGAAAAGGGA
scaffold519034_10.6	1	p2	(TA)7	14	49	62	ATCGGTTATTACCGGATCGC
scaffold519053_16.5	1	p2	(CT)7	14	146	159	GCGTTGAGAAGAAGGGAGAA
scaffold519056_13.8	1	p2	(CT)9	18	261	278	TCCCCTAATTCCCTCATTCA
scaffold519132_13.6	1	p2	(AG)6	12	756	767	GGTGAAACTTGAACAACCCC
scaffold519194_14.5	1	p2	(GA)6	12	1013	1024	TGGAACAAGGTGGTGGAAAT
scaffold519237_14.8	1	p2	(GA)7	14	358	371	GGAGATCAGCTACGGTCTGC
scaffold519306_15.2	1	p2	(CT)6	12	1524	1535	GCTCCCCTTGTGACACTGTT
scaffold519325_13.9	1	p2	(AG)12	24	460	483	TCCATCAGACAGTTTCTTGCAT
scaffold519551_17.7	1	p2	(CT)7	14	312	325	TCCCTCCTCAAATTTACCCC
scaffold519559_16.7	1	p2	(TA)8	16	517	532	CGGGATCTTCATCCAATTCA
scaffold519655_11.3	1	p2	(AT)9	18	423	440	TTGGAATAGGGTTTTCAGGG
scaffold519867_11.9	1	p2	(TA)9	18	347	364	GAAAGGCACAAAGCGATAGG
scaffold519926_14.1	1	p2	(AT)11	22	1367	1388	CGGCGTTTATTTTGTTTGCT
scaffold519946_15.6	1	p2	(AG)8	16	93	108	GCTCGTACGAAACCCTTGAG
scaffold520102_15.4	1	p2	(TC)6	12	606	617	AATTTCCCGGTGGACTCATT
scaffold520310_11.3	1	p2	(GT)6	12	319	330	GCATGAGAAACCTCAAAGGC
scaffold520362_14.9	1	p2	(TC)7	14	901	914	ATGCAACCCTCCCTTTCTCT
scaffold520396_10.5	1	p2	(AT)6	12	63	74	GACCTACAGACCGATCATGAAA
scaffold520468_20.0	1	p2	(AT)6	12	56	67	TGCTCACATCTCTTTTTCTCTTTTT
scaffold520495_13.2	1	p2	(AT)6	12	72	83	TGCCTTATAAGCTCGAGAAAAA
scaffold520516_17.2	1	p2	(TA)6	12	564	575	TGAGCCCAATTTAGTCAGCC
scaffold520533_12.2	1	p2	(AT)6	12	1344	1355	CATCGCATTGACACATAATCG
scaffold520538_15.7	1	p2	(AT)10	20	1323	1342	TAGCCGTTTATGACCGTTGA
scaffold520641_14.1	1	p2	(TC)8	16	1612	1627	TTTTGGGATGGACCAAGTTT
scaffold520747_17.0	1	p2	(TC)9	18	2108	2125	TCAAAGCCTTTTTGCCTCAT
scaffold520886_18.1	1	p2	(TG)7	14	2414	2427	CGATGCTTTAAAAGATGTCAACC
scaffold520938_11.5	1	p2	(AT)6	12	123	134	AAGAAAGAAATAGAGCTGCTGGAA
scaffold520954_10.2	1	p2	(AT)7	14	353	366	TCGTCCTGGTTGTTCCAAAT
scaffold520955_16.3	1	p2	(AT)7	14	1034	1047	CGACTTGGCTCCATGTTGTT
scaffold521129_17.7	1	p2	(AT)7	14	2201	2214	TGCATCCAGTGGCGTATCTA
scaffold521220_11.0	1	p2	(AT)9	18	117	134	GAATTCGGATTCGAGTCATATAAT
scaffold521286_12.4	1	p2	(AT)9	18	679	696	CAGGATGAAGACGATGCTGA
scaffold521408_12.5	1	p2	(GA)6	12	55	66	GAAGCTTCAATGGAGGTGATTC
scaffold521482_18.9	1	p2	(GA)6	12	889	900	TTCGGAATTGGATTATGTTGC

scaffold521593_18.[illegible]	1	p2	(TA)7	14	147	160	ACCCTCGAATGCTATGACCA
scaffold521658_13.[illegible]	1	p2	(CT)7	14	359	372	TCCCCTTATTTGTCCACACC
scaffold521670_16.[illegible]	1	p2	(TA)6	12	522	533	ACACATCTTGCAACCCACAG
scaffold521699_14.[illegible]	1	p2	(TA)6	12	53	64	CCAATTTGAATGGTTATCACCTG
scaffold521706_16.[illegible]	1	p2	(AT)6	12	809	820	GAGGCTTAAAGCTGTGCTGG
scaffold521806_11.[illegible]	1	p2	(AT)7	14	147	160	GGATAAATGTGGCGAATGCT
scaffold521816_15.[illegible]	1	p2	(CT)10	20	59	78	TCCATCATCATAGGCGTCAC
scaffold521843_10.[illegible]	1	p2	(CA)7	14	290	303	CCATCATCCTCTCTCCCAAA
scaffold521862_14.[illegible]	1	p2	(AT)9	18	1753	1770	AGGTGGCACTCTGAAATCAA
scaffold521863_14.[illegible]	1	p2	(AG)8	16	245	260	TTTGGTTCAAGGATTTGATGC
scaffold522013_17.[illegible]	1	p2	(AT)6	12	215	226	GCGTATGGGTAGAGCGAGAG
scaffold522123_13.[illegible]	1	p2	(AT)9	18	1207	1224	GAAGAAGCCGACAATTTGCT
scaffold522237_15.[illegible]	1	p2	(CA)8	16	673	688	TTCTACCGCAACACAAGGTG
scaffold522319_15.[illegible]	1	p2	(TA)6	12	1335	1346	GCTACGTGGGAGCTCAAGTC
scaffold522440_11.[illegible]	1	p2	(TA)6	12	921	932	CGACTCCATTTGGGTTTTGT
scaffold522484_13.[illegible]	1	p2	(CT)11	22	444	465	CAGATCTTGGCAACTCCCTC
scaffold522508_16.[illegible]	1	p2	(AT)9	18	2851	2868	ATGTCGAAGAATCCGAATGG
scaffold522528_18.[illegible]	1	p2	(AT)6	12	573	584	TGGACCGGTTAGATCGGATA
scaffold522531_14.[illegible]	1	p2	(CT)6	12	916	927	CATCCCCACCGTGGTATTTA
scaffold522566_16.[illegible]	1	p2	(AC)7	14	1664	1677	ACCGTTTGTGAAAAGAACGC
scaffold522588_13.[illegible]	1	p2	(TA)6	12	317	328	TCATTTTTCTTGTAGCGTATTACTCA
scaffold522605_16.[illegible]	1	p2	(AG)7	14	323	336	CGTTCATCCCCTTTGTGTCT
scaffold522698_14.[illegible]	1	p2	(TA)7	14	92	105	GTTGTTTTGTTAGCCACTGTGA
scaffold522715_18.[illegible]	1	p2	(AT)8	16	92	107	GACTGTAATGCATGGCTGGA
scaffold522847_12.[illegible]	1	p2	(TA)6	12	2470	2481	CCATCGATGCCGATAGATTT
scaffold522869_15.[illegible]	1	p2	(GA)9	18	147	164	AAATTGGATCGTTGCCAGAG
scaffold522989_14.[illegible]	1	p2	(AT)6	12	683	694	ATCTCGTTGAGGATGCTTGC
scaffold523075_14.[illegible]	1	p2	(CT)10	20	2281	2300	AAACACCACCTCCCTCACTG
scaffold523079_11.[illegible]	1	p2	(GA)6	12	144	155	CACGGTTAGGCTCGAGAGAG
scaffold523160_14.[illegible]	1	p2	(AG)6	12	352	363	TTTACGAAAATGCCACCACC
scaffold523224_19.[illegible]	1	p2	(CT)6	12	133	144	AGCATCTCTCCATCACTCTCA
scaffold523268_10.[illegible]	1	p2	(TA)7	14	215	228	TTCGAACCTGGTTTTCGTTC
scaffold523277_15.[illegible]	1	p2	(TA)6	12	99	110	TACCTTGATGTGGTGGTGCT
scaffold523306_15.[illegible]	1	p2	(TA)6	12	1410	1421	AAAACTCCCAATTTCCACCA
scaffold523356_15.[illegible]	1	p2	(GA)8	16	791	806	TCCCCTAGTCTAAGCCGAAA
scaffold523390_16.[illegible]	1	p2	(AT)6	12	313	324	GTGGGAGATGGTGGAGAGAG
scaffold523630_16.[illegible]	1	p2	(GA)7	14	4354	4367	GCTTTTGCTTTTGCTTTTGG
scaffold523684_16.[illegible]	1	p2	(GA)9	18	113	130	TTTGAAGGGTACTGCCATCA
scaffold523898_11.[illegible]	1	p2	(AG)6	12	961	972	TCACCTTTTCCCTCTTCCCT
scaffold523962_14.[illegible]	1	p2	(GA)7	14	740	753	ACTCCCATCCGAGGTCCTAA
scaffold524079_16.[illegible]	1	p2	(CA)7	14	2361	2374	TAGTCCGTGGGTTTACCGAG
scaffold524086_16.[illegible]	1	p2	(GA)6	12	1970	1981	TGGTGCAGATGGAAGTGAAG
scaffold524131_18.[illegible]	1	p2	(AT)6	12	56	67	AGCAAAGAAGGAATGCAACC
scaffold524160_13.[illegible]	1	p2	(TG)6	12	302	313	GCGCGATTATCGAAGAGAGA
scaffold524199_11.[illegible]	1	p2	(TA)10	20	249	268	GCATGAGAGCCAAGCAAAAT
scaffold524201_16.[illegible]	1	p2	(TA)12	24	1388	1411	CAGATCTGATGGCCCAGATT
scaffold524290_15.[illegible]	1	p2	(AG)6	12	1903	1914	CGTGTTCCTAATCCCCATGT
scaffold524333_12.[illegible]	1	p2	(AT)6	12	573	584	TTTTACGGGCAACGTACTCC
scaffold524368_13.[illegible]	1	p2	(TA)6	12	146	157	AGCTATAAGATTGACTAGATCAACGA
scaffold524582_11.[illegible]	1	p2	(AT)6	12	293	304	TCGCGTCAGACTCCTCACTA
scaffold524704_11.[illegible]	1	p2	(TA)9	18	530	547	CTCAATGATGGCCGAAAACT
scaffold524729_16.[illegible]	1	p2	(TC)6	12	1067	1078	GCCGTTGATCATTACCTCGT
scaffold524753_14.[illegible]	1	p2	(GT)7	14	294	307	TGCCCTTTCTTTTAGGAGCA
scaffold524816_15.[illegible]	1	p2	(CA)6	12	94	105	AACGAACACGAGAGGTTTGG
scaffold524854_13.[illegible]	1	p2	(AT)9	18	1393	1410	ACGGTGAAAATGGATCTTGG
scaffold524905_12.[illegible]	1	p2	(AT)7	14	270	283	AAATGACCAAAAAGGGACGA
scaffold524947_20.[illegible]	1	p2	(AT)6	12	356	367	AACGGCGCGACAGTTATAGA
scaffold524998_12.[illegible]	1	p2	(AG)7	14	546	559	TGCATGAGCACTAATGAGCC

scaffold525042_13.9	1	p2	(TA)10	20	837	856	GAATGGCCCGAGATTTGTTA
scaffold525077_13.9	1	p2	(TA)6	12	1353	1364	AGTGCCGTAGCCACAAAAAT
scaffold525332_14.3	1	p2	(TA)8	16	428	443	TTTTGCCCTCGAATGCTATC
scaffold525619_17.4	1	p2	(CT)7	14	269	282	AACCGTGATGGTTGGGATTA
scaffold525657_17.5	1	p2	(TA)9	18	761	778	TCCTCAACTCTGAGCCGAAT
scaffold525662_14.4	1	p2	(TA)7	14	354	367	TTTAATGATGCGTTTTGGCA
scaffold525811_11.6	1	p2	(TA)8	16	1351	1366	ATGGGATAGTACCGGAACGC
scaffold525881_14.8	1	p2	(CA)7	14	415	428	TTCACTCACACACACTCGCA
scaffold525897_13.4	1	p2	(TC)6	12	94	105	AATGAGGCTTTTGGATCGAC
scaffold525934_15.0	1	p2	(AT)7	14	649	662	AATGGCACACAGGTGAATGA
scaffold525956_12.2	1	p2	(AG)8	16	717	732	AATCATCCAATTTGCTTGCC
scaffold525990_14.9	1	p2	(AG)6	12	150	161	GCAACAACAACAACTGACGC
scaffold526002_16.5	1	p2	(AC)13	26	6219	6244	TGGAGATCATTTCAGGCACA
scaffold526101_14.7	1	p2	(TC)6	12	537	548	CACCATTTTGGTATTTGCCC
scaffold526115_16.8	1	p2	(TA)10	20	648	667	AGGGGGTCAAACTCCTTGAA
scaffold526144_15.7	1	p2	(TA)10	20	330	349	TTTTCCATCAACTAACCCGC
scaffold526272_12.3	1	p2	(TA)6	12	112	123	TCATTTCACCCATGAGCTTT
scaffold526273_13.6	1	p2	(CT)8	16	1073	1088	TCCAACTCTTCACACTCCCC
scaffold526306_15.0	1	p2	(CT)6	12	138	149	TCATACACGATGGGATCGAA
scaffold526347_16.4	1	p2	(TC)6	12	2828	2839	AACGTCAAGTATTCCGCCAC
scaffold526358_17.4	1	p2	(TC)6	12	376	387	TCCGCGAGAGAATTAAGGAA
scaffold526371_14.8	1	p2	(AC)6	12	1582	1593	TGACATGGAAGGAAAAAGGG
scaffold526395_12.5	1	p2	(TA)8	16	71	86	TCTCCAAATTTGAGGGTTATCA
scaffold526456_13.4	1	p2	(CT)6	12	41	52	CAAATGGCCAAGAGAGTTACAA
scaffold526473_20.6	1	p2	(GA)9	18	213	230	TCGTGTTTATCATCACCCCA
scaffold526572_12.6	1	p2	(TA)9	18	43	60	CGGTTATTACCGGATCGCT
scaffold526602_16.2	1	p2	(CT)6	12	69	80	TACAAATGGCCAAGGGAGTT
scaffold526623_16.9	1	p2	(GA)6	12	1261	1272	GGGTTTGAGTGAGTGGAGGA
scaffold526674_14.0	1	p2	(TA)10	20	377	396	AGAAATGACATCACCACGCA
scaffold526719_11.2	1	p2	(AT)7	14	54	67	TCAAACAACTAGATTAGTCCATATCC
scaffold526732_17.0	1	p2	(TC)6	12	3500	3511	AAACAGCAGGTTTTTGGTGG
scaffold526842_15.7	1	p2	(AG)8	16	3266	3281	AGAGAAGTGAAAGGGGCTCG
scaffold526844_14.3	1	p2	(CT)6	12	72	83	ACAATGGCAAGAGAGTTGCT
scaffold526854_14.2	1	p2	(GA)13	26	424	449	CGAATACCTACCCCGTGAGA
scaffold526855_13.2	1	p2	(CT)7	14	1425	1438	CACTCCGCTCTCATTCCTTC
scaffold526990_14.8	1	p2	(TC)6	12	150	161	TCTCTTCAATAAGGTAACAACCAAA
scaffold526998_17.9	1	p2	(TA)7	14	658	671	GGGTGTGGAAGCAAAGAAAA
scaffold526999_14.7	1	p2	(AT)6	12	866	877	TTCAACATGATGGTGCCAAT
scaffold527086_10.6	1	p2	(CT)7	14	96	109	CAAAATTAAAAGTGTTCCATCCC
scaffold527201_16.3	1	p2	(CT)13	26	4467	4492	GACCAGTTCAAGGGAGGTGA
scaffold527218_15.9	1	p2	(TA)8	16	888	903	ATGGCCACAAATGGTTCCTA
scaffold527220_18.5	1	p2	(TA)8	16	248	263	CATTCAAGGGGCATTTTTGT
scaffold527235_15.7	1	p2	(TC)6	12	445	456	CCACGGGAGAAAGGTGTTTA
scaffold527498_14.1	1	p2	(TA)8	16	1308	1323	TTGACACGCAAGATTTGCTC
scaffold527557_15.2	1	p2	(GA)6	12	834	845	CTACGCGACCCATTCTTGAT
scaffold527689_13.9	1	p2	(AT)7	14	2126	2139	CCAGTATTTGAGAGATGGGCA
scaffold527736_10.2	1	p2	(GA)6	12	96	107	TGTCTTTAGCTCTGGTGTGGC
scaffold527849_10.5	1	p2	(AC)6	12	295	306	ACTCCAGCCACCATCTTCAC
scaffold527888_14.9	1	p2	(TC)6	12	319	330	GGGACGGAGGGAGTAGTTTC
scaffold527929_14.7	1	p2	(TA)7	14	295	308	CGATGTTTTAACGCCGAGAT
scaffold528044_17.1	1	p2	(TC)8	16	147	162	GACCAAGCATGTGGTTCCTT
scaffold528063_16.4	1	p2	(TA)6	12	321	332	CCGTTAAATTACATGATTTTCCCT
scaffold528107_15.4	1	p2	(TA)7	14	1904	1917	AACCACGTTTTGGCTAGTTCA
scaffold528184_18.0	1	p2	(GA)6	12	1028	1039	CAAGAAACAACAGGGGGAAA
scaffold528219_12.4	1	p2	(TG)6	12	53	64	TGTTCCTCGTGTTCCTCCTC
scaffold528321_11.0	1	p2	(TA)8	16	194	209	GTGCGTGTGTATGCTGAGGT
scaffold528405_13.8	1	p2	(AT)6	12	284	295	GACAAGATCGTGCCAACAAA
scaffold528419_12.2	1	p2	(TA)8	16	1395	1410	TCGATTCGAATTATGAGGGC

scaffold528502_11.8	1	p2	(AC)9	18	465	482	ATGGCAAAAGGCATCAAAAG
scaffold528634_13.6	1	p2	(TC)7	14	1300	1313	CTTCACGCGCTCAATAAACA
scaffold528669_13.7	1	p2	(AT)6	12	54	65	CATGGTAAGCAGATTGGTTTTAAT
scaffold528677_10.1	1	p2	(TA)6	12	54	65	TCAGATTTGTTATGTGTTTTGACA
scaffold528684_15.3	1	p2	(AG)8	16	863	878	GACCAGGAGTTGAAGATGCC
scaffold528879_16.2	1	p2	(AT)6	12	94	105	CAACTTGGAACCCGATTCTC
scaffold528925_15.2	1	p2	(CT)8	16	1390	1405	TGGTCTCCATCTTCCGTCTC
scaffold528950_16.1	1	p2	(AT)6	12	426	437	TGAAAAATGCAACCAATGGA
scaffold529057_18.2	1	p2	(AG)7	14	360	373	AGGCCGTGTTCGTATTGTGT
scaffold529086_13.1	1	p2	(CT)6	12	486	497	ACTCATGTCACGGCACACAT
scaffold529088_15.2	1	p2	(AT)7	14	235	248	AAAAGCTCCTCCACAATTTGA
scaffold529093_12.5	1	p2	(TC)7	14	116	129	GATTCGCACAAATGAACACG
scaffold529096_17.8	1	p2	(TA)6	12	79	90	TTGTCGATCAGCGGTTACAG
scaffold529132_13.0	1	p2	(TA)11	22	600	621	TCAACCGCCAGGATTATTTC
scaffold529221_15.2	1	p2	(AC)7	14	184	197	CACGTGGCATGATAATAGCG
scaffold529264_12.6	1	p2	(TA)6	12	96	107	TACTCAAGCCCACGATCTCC
scaffold529327_14.4	1	p2	(TA)6	12	256	267	TTGTTCGTATTTGGAAGTCGTG
scaffold529373_21.4	1	p2	(TC)6	12	44	55	TGCCCTCTTTCTCTATCTCTCTTG
scaffold529422_10.3	1	p2	(AT)9	18	56	73	TCCACCGCTTTAAATTGCTT
scaffold529497_15.2	1	p2	(AT)6	12	759	770	CGACCAAACTCATAGCGTCA
scaffold529506_16.9	1	p2	(TA)10	20	2858	2877	AAAATGCAGTTCGGTTGGAG
scaffold529512_16.2	1	p2	(TA)7	14	266	279	CTTTTTCCATCAACCAACCC
scaffold529531_12.3	1	p2	(AC)7	14	365	378	TCACGGAATTACTAAGCCCC
scaffold529560_13.4	1	p2	(GA)10	20	54	73	TCTTCCGATCTCAGTTTTTCAA
scaffold529580_17.7	1	p2	(GT)7	14	653	666	AACAAGCTGGGTTTCTGCAC
scaffold529615_15.9	1	p2	(GA)8	16	475	490	GCCTGCAAGAAAGCAATCTC
scaffold529655_12.1	1	p2	(CT)8	16	41	56	CATACTCACTCTCACTAGCCGC
scaffold529681_12.3	1	p2	(AT)7	14	164	177	GTTGGAGGAATGGAGGGAAT
scaffold529705_17.5	1	p2	(CA)6	12	279	290	GATCTTTTCACTCGAGCCCA
scaffold529936_13.7	1	p2	(CT)11	22	106	127	TTGCATTGCAGCAGTAGAGG
scaffold529942_16.8	1	p2	(AT)6	12	749	760	CTTGTTTTGGAACCAGCCAT
scaffold529962_14.6	1	p2	(AG)9	18	1149	1166	GACTCCTGCAACTTGCCTTC
scaffold530007_12.9	1	p2	(CT)11	22	1743	1764	ATGTTCCCATTTCACCTTGC
scaffold530214_13.8	1	p2	(TG)7	14	184	197	GCTGCCTACAACAGCAACAA
scaffold530217_10.9	1	p2	(GT)6	12	479	490	TTACTATGTGGTTGCGGTGC
scaffold530231_15.0	1	p2	(AT)9	18	3411	3428	ATCAGGCCCATATTTCGATG
scaffold530431_13.3	1	p2	(TA)8	16	409	424	TTTTCGCCTCCCACTAAAAA
scaffold530576_13.9	1	p2	(AT)7	14	95	108	GCAATGCTCCATTTTCCAAC
scaffold530636_16.8	1	p2	(TC)6	12	2563	2574	TGGCTGTAGTGGAAATGTGG
scaffold530746_15.9	1	p2	(TG)7	14	1002	1015	GGTTAGCGTGTCGTGTCAGA
scaffold530850_12.6	1	p2	(AT)6	12	1265	1276	TCCCTTGCTAGATGGCAGTT
scaffold530856_14.5	1	p2	(AT)7	14	150	163	CGGCCATCAATATACACCAA
scaffold530894_11.8	1	p2	(TA)6	12	79	90	TCGGTTACCCTATAGCATTCG
scaffold530932_15.6	1	p2	(TC)9	18	4429	4446	TAACCACGGATGCCACTGTA
scaffold530963_11.4	1	p2	(GC)7	14	78	91	CTACGAGCCACGGCTACAC
scaffold531126_14.8	1	p2	(CT)6	12	334	345	TATCGCCGTTAAGCTGCTCT
scaffold531227_16.3	1	p2	(AG)7	14	1040	1053	AAAGATGGGAAAATGGGACC
scaffold531228_12.8	1	p2	(AG)9	18	32	49	AGAAACTTGACGTGTGCGTG
scaffold531273_15.0	1	p2	(AT)6	12	2102	2113	AATGAGGTAAATTTGAGAATGATGA
scaffold531296_10.0	1	p2	(TA)9	18	67	84	CAAAGGCCCACAATGGTTAT
scaffold531314_10.7	1	p2	(AG)9	18	146	163	GTGTTTGGTCTCCCCTCCTT
scaffold531380_15.3	1	p2	(GA)6	12	77	88	GTGGTTCGATCAAGGTGAGC
scaffold531386_12.5	1	p2	(TC)6	12	245	256	TGGGCATTTGTATACCCGTT
scaffold531528_11.7	1	p2	(AG)7	14	209	222	AATTTGCCCTCTCCCTCTTC
scaffold531532_14.3	1	p2	(TA)8	16	838	853	CAAAACAGTATACAACGGGCA
scaffold531535_11.7	1	p2	(AT)6	12	457	468	TGCTATCGGCTAACGGTTTT
scaffold531609_13.3	1	p2	(GA)6	12	331	342	CACAATCGATCAATAAAGCTGC
scaffold531706_13.8	1	p2	(TA)10	20	99	118	TGAGGCTTAGAGGGTGTTTTG

scaffold531730_15.(	1	p2	(GA)8	16	1202	1217	AGCCTTACCAAAGGGGAAAA
scaffold531751_10.4	1	p2	(CT)6	12	199	210	GGAAGAATTGGAAACTCGTGA
scaffold531765_18.8	1	p2	(TA)6	12	196	207	ATGATCTACGTCCACGGGAG
scaffold531768_18.(	1	p2	(AT)7	14	149	162	CCTTCCAATGGACTTCCTCA
scaffold531854_11.(	1	p2	(AT)7	14	209	222	GCAAAAGCTTTCTCGACCAC
scaffold531873_15.(	1	p2	(CT)6	12	140	151	GCATGATCGACATTGTAAAGGA
scaffold531924_13.6	1	p2	(AG)6	12	152	163	ATTGCTTCCCCTCTTCCATT
scaffold531925_17.4	1	p2	(CT)9	18	1740	1757	CATGCCATGGAGTCGTTCTA
scaffold531939_10.5	1	p2	(TC)6	12	85	96	GTTGATGCCCTTCGTCAACT
scaffold531952_16.1	1	p2	(GA)9	18	146	163	AACGCTTTCTGCTTCTGCAT
scaffold532051_18.4	1	p2	(GA)7	14	937	950	CTCGTTGGAGGCCAATAAAA
scaffold532075_15.(	1	p2	(TA)10	20	69	88	GGAAATAAGAAGGTGTTATGAAGGG
scaffold532213_14.4	1	p2	(AT)6	12	227	238	TCGCAGAAAATCAACTTCTCAA
scaffold532235_12.1	1	p2	(AT)8	16	3036	3051	CTTACACAGTGCCTGCTCCA
scaffold532243_12.4	1	p2	(AT)8	16	170	185	GGAGAAAATCAGGTGATAACCCTT
scaffold532336_14.4	1	p2	(AC)6	12	152	163	TGCGAATCAATTAGTGTGAGAA
scaffold532425_10.6	1	p2	(AG)7	14	184	197	TGGTGTTCTTGGGGTTTTGT
scaffold532428_10.1	1	p2	(TC)6	12	414	425	TTTGAGATCGGAGTGGCTCT
scaffold532453_14.(	1	p2	(GT)7	14	52	65	TCTTGGGAGTGAACATACATACA
scaffold532728_16.4	1	p2	(TA)6	12	2863	2874	ACAGCAAGCATCCAGAAAGC
scaffold532772_11.(	1	p2	(AC)8	16	225	240	AGAATATGCGTGATCGCCTT
scaffold532773_13.9	1	p2	(AC)6	12	225	236	AGAATATGCGTGATCGCCTT
scaffold532833_16.4	1	p2	(AG)13	26	80	105	ACCACGGCTGAATAAATTGC
scaffold532867_12.4	1	p2	(GC)6	12	275	286	GTTGACGGATAACCCAAACG
scaffold532896_13.2	1	p2	(CT)6	12	1921	1932	GGGTTTAGGGGACAGGTGAT
scaffold532957_20.6	1	p2	(AT)6	12	299	310	TGCGCCCTTTAAAACTAAGG
scaffold532958_13.2	1	p2	(AT)9	18	348	365	TTGTCCAAACAAATGAAACATTC
scaffold532975_14.(	1	p2	(AT)9	18	673	690	TGGATTTGGGATTCCTTCAA
scaffold532979_13.2	1	p2	(AT)10	20	734	753	ACAATTTTGCCCTTGAATGC
scaffold533009_21.4	1	p2	(GA)7	14	624	637	TGCGTTTACATTCAAGCAGTG
scaffold533036_17.1	1	p2	(TC)6	12	638	649	CCTCCCACTCCTCACTTCAA
scaffold533091_12.7	1	p2	(AG)6	12	145	156	GTGTGTGTGCATGAGTGTCG
scaffold533209_12.(	1	p2	(TG)8	16	98	113	TGATTTTTAATGCCTTTCAGCTT
scaffold533237_15.6	1	p2	(TA)10	20	199	218	TTCAAAAGGTTTGGGCTTTTT
scaffold533258_13.9	1	p2	(TA)7	14	749	762	TGCACCTCCTCCTAATTAATCC
scaffold533371_15.(	1	p2	(TC)6	12	40	51	TCTCTCTTTTGGTTGGTGAAGA
scaffold533416_21.(	1	p2	(AG)6	12	334	345	TGCAGCTGAAATCAATGAAAA
scaffold533428_14.4	1	p2	(GA)9	18	831	848	TCTACGCATGGAATCAGTCG
scaffold533512_15.6	1	p2	(GA)6	12	750	761	CTTGATTCATCGAACGCAAA
scaffold533568_17.7	1	p2	(AT)7	14	1762	1775	ATGCGATGTTTTGCATTTTT
scaffold533584_13.6	1	p2	(AT)7	14	28	41	CCAAAGTTTATTTAACACGGAAGAA
scaffold533624_10.1	1	p2	(AT)6	12	710	721	TTCTTTCAATCGACGCTCCT
scaffold533631_14.8	1	p2	(CT)6	12	136	147	TTGCTAAGCTCCAGCAACG
scaffold533796_15.9	1	p2	(TC)7	14	100	113	CAATCTGGACGATACTCAATCG
scaffold533971_16.7	1	p2	(CT)9	18	2810	2827	ATCTGTTTCTTCTGCGGCTC
scaffold533976_12.8	1	p2	(AG)6	12	440	451	AAATTGATGGCAAGAGCAGG
scaffold533977_16.1	1	p2	(AG)12	24	4211	4234	GGTTGGTTTGAGTTCCTCCA
scaffold534145_11.(	1	p2	(AG)8	16	50	65	TCCATACACACCAAATAAAAAGACA
scaffold534511_19.(	1	p2	(GA)9	18	156	173	TTGGGGAAATAATGTCGATCT
scaffold534587_14.9	1	p2	(CT)8	16	4379	4394	GCATAAAGCTCATCTTGCACA
scaffold534592_12.6	1	p2	(AG)6	12	241	252	GATCGGTGAAGATTGGAGGA
scaffold534624_12.2	1	p2	(GA)6	12	399	410	CCGCATTCTGTTTGTTGTTG
scaffold534675_16.4	1	p2	(GA)6	12	1520	1531	GTACAACGAGGATGTGGGCT
scaffold534749_20.6	1	p2	(GA)6	12	53	64	CCTTTGGTCTCGGTTGAAAT
scaffold534819_11.6	1	p2	(TA)6	12	112	123	CATGAAAGACTCTATAGACAAGAAA(
scaffold534878_18.7	1	p2	(GA)8	16	148	163	AGGGTGTGTGAGAGACCGAG
scaffold534921_19.1	1	p2	(TG)8	16	306	321	TGGCTTGTATGTTCTGATCACC
scaffold534955_12.8	1	p2	(AT)6	12	169	180	GTTCGAAAGCCACAAATGGT

scaffold534973_13.5	1	p2	(GA)10	20	58	77	GCTAGGGCGATTGTATTGATG
scaffold534988_14.9	1	p2	(TA)11	22	370	391	TGATGTTGAAGAATCAGCGG
scaffold535061_11.0	1	p2	(TC)7	14	65	78	TACCGTTGGTTAATCCTGCC
scaffold535282_14.0	1	p2	(TA)13	26	167	192	TTTCGAAAACATGCAATGGA
scaffold535311_16.2	1	p2	(AG)6	12	697	708	ATCACCCCTCACAAGAACCA
scaffold535348_18.9	1	p2	(GA)6	12	148	159	AGAAGGGCAATGAATTGGTG
scaffold535466_15.0	1	p2	(AT)7	14	225	238	AAATATCGGCCGTCAGATTG
scaffold535493_15.1	1	p2	(AG)7	14	796	809	AGCAAATGCCAATAAGGTGG
scaffold535580_13.7	1	p2	(AG)7	14	159	172	ATTGAGTCCCAATCTCGTGG
scaffold535615_14.8	1	p2	(CT)6	12	102	113	CACTCTGCTAGGGTTGGGAG
scaffold535662_11.0	1	p2	(TC)6	12	149	160	AACTGTGGCAAGATCCATCC
scaffold535695_12.8	1	p2	(CA)7	14	197	210	TTCGAATTTTATTATTGATTGAGCA
scaffold535748_16.0	1	p2	(TC)7	14	372	385	GCAATTTTAAGATCGAATCCCA
scaffold535940_14.8	1	p2	(TC)6	12	190	201	TTGCATGAACAACGTGGAAT
scaffold535998_10.8	1	p2	(TA)10	20	325	344	TGTCACCTTGAAAGAATTACAAACA
scaffold535999_15.2	1	p2	(TA)7	14	57	70	GAGTGACATCTTCGAGTGTTCG
scaffold536032_14.3	1	p2	(TA)7	14	564	577	TTAGCAGCTTATACGTGTGAAAA
scaffold536055_16.7	1	p2	(TA)11	22	2079	2100	TCCTTCTAATCGATTCTTCCACA
scaffold536076_17.0	1	p2	(TA)6	12	9802	9813	TGAAGAAGACGTGGTCGAGA
scaffold536227_15.2	1	p2	(TC)10	20	2582	2601	ATCCCTTCTGGTTCTCCCAC
scaffold536347_11.2	1	p2	(TA)7	14	103	116	GAAGCGACACGGGTTATTTT
scaffold536501_17.8	1	p2	(AG)9	18	148	165	GGGACTCGGATAAAAAGGGA
scaffold536690_15.6	1	p2	(TA)6	12	2905	2916	GCTGAGGCTTCTTCCATGTC
scaffold536760_16.8	1	p2	(TA)8	16	40	55	CACACTCACTCACAAACACACAA
scaffold536837_12.8	1	p2	(TA)9	18	1195	1212	CGTTGAACGTACCCTCGATT
scaffold536838_17.9	1	p2	(AT)7	14	152	165	TCCCTAGACTCCGTAATTGGAA
scaffold536849_19.9	1	p2	(CG)7	14	395	408	TGTCCCACATTGGAAAATGA
scaffold536949_12.3	1	p2	(AG)8	16	228	243	AAAACAAAACCTGGTGCTCG
scaffold536955_15.1	1	p2	(TA)9	18	1240	1257	ATCATGTAGTCGCGTGCTGT
scaffold537069_10.4	1	p2	(TC)6	12	222	233	TGAACAATTGAACACCGGAA
scaffold537092_13.8	1	p2	(CT)9	18	1010	1027	TGCTGTCCCTCTAGCAGGTT
scaffold537140_15.7	1	p2	(TA)9	18	45	62	TGCGTTTTGACAATATATAGGTGTTT
scaffold537198_18.7	1	p2	(GA)6	12	252	263	TATGCAAGAGCGAGCAGAGA
scaffold537238_12.9	1	p2	(GT)6	12	297	308	TGTCCAAGCCGATCCTTATC
scaffold537291_12.2	1	p2	(TG)6	12	1102	1113	AATTATGTGCAAGCTCATCCG
scaffold537352_15.0	1	p2	(TC)6	12	233	244	ACACCCCACAAAATCGAGAG
scaffold537491_16.4	1	p2	(TA)7	14	328	341	TCACGTGTCATTGTAACCGC
scaffold537565_15.6	1	p2	(AG)10	20	2369	2388	AATTGATTGCTGCTGCTGTG
scaffold537615_14.9	1	p2	(TG)6	12	561	572	TCTCCCCCTTCTGTGGTATG
scaffold537707_16.2	1	p2	(CT)6	12	4125	4136	CGGAGAATTGATGTTCCCAT
scaffold537803_12.5	1	p2	(AG)10	20	352	371	TGCAGCTAATTTTCTGCTGG
scaffold537808_15.6	1	p2	(CT)9	18	46	63	CGCCTTTCTCTTTCTAACCTCC
scaffold538092_13.7	1	p2	(TG)6	12	294	305	TCTCAACCAATGATGAGCCA
scaffold538481_12.6	1	p2	(AT)10	20	388	407	CGGATCGAAAATTCTCCTTATG
scaffold538790_12.7	1	p2	(GA)8	16	258	273	CCACGAGATGGGGACATTTA
scaffold538824_13.1	1	p2	(AG)11	22	383	404	TACCCTCTCCTCCTCCGATT
scaffold538862_15.7	1	p2	(TG)6	12	1393	1404	GAAATGCATCTCATTCGGGT
scaffold538950_12.7	1	p2	(AT)11	22	1303	1324	TACGGTTCGGTTCCAGTTTC
scaffold539112_12.7	1	p2	(GA)6	12	428	439	CAATGGAAAACACGCAACAC
scaffold539188_14.9	1	p2	(AC)6	12	770	781	CGCTGAGGGATCTGGTATGT
scaffold539258_13.3	1	p2	(TA)8	16	1777	1792	GCTGGATTTTTGGCATTCAT
scaffold539313_18.5	1	p2	(TC)9	18	1240	1257	TTTTTCGGTTCGGTTCAAAG
scaffold539453_12.5	1	p2	(TA)6	12	256	267	GTTTCACTTTGGTACCCGGA
scaffold539480_12.0	1	p2	(AG)6	12	432	443	AAGTCGGCCAAGAAGAGAAA
scaffold539543_11.6	1	p2	(AG)6	12	123	134	CTAAGCGATGCCTAAGGCTG
scaffold539547_17.6	1	p2	(CT)6	12	89	100	CTCACTAGCCGCAGCTCTCT
scaffold539558_12.6	1	p2	(TG)6	12	66	77	GGAAAATATCAAGTCAACACAAAA
scaffold539586_14.2	1	p2	(TA)7	14	716	729	TGATTAGCATCACAGCCCAA

scaffold539681_15.[illegible]	1	p2	(TA)6	12	144	155	CAACGCTAAAACATCTGAGAACA
scaffold539845_14.[illegible]	1	p2	(TA)7	14	338	351	GATATGGATGCCCCAGATTG
scaffold539847_15.[illegible]	1	p2	(AG)7	14	145	158	TGATTTTGGACGTTCTGCAC
scaffold539861_13.[illegible]	1	p2	(AG)8	16	159	174	GACGATGGAAGGAGGTGAGA
scaffold539911_11.[illegible]	1	p2	(TA)8	16	136	151	TTTTCCGAAGTACGAGCTGC
scaffold539915_11.[illegible]	1	p2	(AT)7	14	281	294	GGATTAGAATTCGGAACACTCG
scaffold539974_11.[illegible]	1	p2	(CA)6	12	131	142	TCAAATGCACAAATTCAATGG
scaffold539996_15.[illegible]	1	p2	(GT)7	14	472	485	AAGTAGGGGGCTTCAATCCA
scaffold540025_12.[illegible]	1	p2	(TA)8	16	680	695	ACATTTCGATTAGGTGTGACAA
scaffold540038_19.[illegible]	1	p2	(AC)6	12	750	761	TACAAATCGTGAGCAGCAGG
scaffold540104_15.[illegible]	1	p2	(AT)7	14	926	939	TTTTCTTTGTCTTCAATGCCA
scaffold540209_10.[illegible]	1	p2	(AT)8	16	68	83	CACAATAAGGGTTGGTATCATTTG
scaffold540216_15.[illegible]	1	p2	(AT)6	12	911	922	GGATAAAGTACGATGATTGACCA
scaffold540268_17.[illegible]	1	p2	(TC)6	12	1275	1286	TGAAGGAGGCAGGCTTTAGA
scaffold540326_14.[illegible]	1	p2	(GT)11	22	610	631	TCATTTTGCTTGCAGTGTCC
scaffold540344_14.[illegible]	1	p2	(TA)7	14	126	139	ATAGGTGTGGCCCAGTTTGA
scaffold540391_13.[illegible]	1	p2	(TC)6	12	100	111	ACGTTGGACCTGAAAGAGGA
scaffold540409_18.[illegible]	1	p2	(TA)9	18	48	65	CATGGGTTATCACTTGAATGAGAC
scaffold540461_15.[illegible]	1	p2	(AC)6	12	208	219	TTAGATGTGCAGCCGACAAG
scaffold540534_14.[illegible]	1	p2	(GA)6	12	544	555	TCCCGTAATATAGGTCCCAAAA
scaffold540681_12.[illegible]	1	p2	(AC)6	12	115	126	GGTTCTGAAACCGGGATAGG
scaffold540711_16.[illegible]	1	p2	(AT)8	16	464	479	CCTAAAAGGAACTGTGGAGGC
scaffold540728_12.[illegible]	1	p2	(TA)6	12	1138	1149	TTGACCTTTTACCTTGCCCA
scaffold540984_17.[illegible]	1	p2	(GA)6	12	285	296	GTCCGCGAAGAAACACTCAC
scaffold541174_10.[illegible]	1	p2	(AT)6	12	155	166	AGGAGCAATACTGCTTGTGGA
scaffold541255_16.[illegible]	1	p2	(TA)6	12	234	245	GAGAGGGCTTCTTCTGGGAT
scaffold541271_16.[illegible]	1	p2	(GA)7	14	184	197	TTGGGTAGAAGGCATTGAGAG
scaffold541320_13.[illegible]	1	p2	(AT)8	16	798	813	AACATCAATCATAGTTCAACTTTAGG
scaffold541387_16.[illegible]	1	p2	(CA)6	12	417	428	AGACTCCAAGCCCTGTTGTG
scaffold541395_14.[illegible]	1	p2	(TA)9	18	202	219	ATTATGTGGCCCGAATGACT
scaffold541485_11.[illegible]	1	p2	(TA)6	12	752	763	ATATTCGATCCGGAAGGGTC
scaffold541594_13.[illegible]	1	p2	(TA)11	22	869	890	GATCCAGTAATTTGGGAAGGC
scaffold541711_11.[illegible]	1	p2	(TC)6	12	73	84	CCTTTGGATATGGTGATTTGG
scaffold541780_14.[illegible]	1	p2	(TA)8	16	425	440	CCCGAGCTTCCATAAATCAA
scaffold541845_15.[illegible]	1	p2	(AT)6	12	1666	1677	ACCGCACAAATCATTTCCTC
scaffold541858_14.[illegible]	1	p2	(TA)13	26	515	540	AACCTCTATCCCGTGCAATG
scaffold541881_13.[illegible]	1	p2	(TC)7	14	54	67	CATGGCCCTTTCTTCACATT
scaffold541913_15.[illegible]	1	p2	(AG)7	14	102	115	TTCCATAAAAAGTTAGGCAAGTCT
scaffold541955_16.[illegible]	1	p2	(AT)7	14	331	344	ACAACATTCATCGTTCCCCT
scaffold542003_10.[illegible]	1	p2	(TA)6	12	889	900	TTGTTTCCACACCCATGTTG
scaffold542038_11.[illegible]	1	p2	(GA)8	16	723	738	GGCTGGCTCCAGTAGTTGTT
scaffold542086_16.[illegible]	1	p2	(GA)6	12	249	260	TCTGTGTGTTGAGAGATCAGTGAG
scaffold542128_16.[illegible]	1	p2	(AG)6	12	93	104	CAAAAACAGAGTGAGGGGGA
scaffold542231_15.[illegible]	1	p2	(TA)6	12	263	274	TGTCCTTTTGGATCCAGTCC
scaffold542295_14.[illegible]	1	p2	(TC)9	18	76	93	TTGCTGCCCTATTTTCTTTGA
scaffold542375_15.[illegible]	1	p2	(TC)6	12	455	466	ATTGGCAGCAGAAGGAATTG
scaffold542381_15.[illegible]	1	p2	(GA)12	24	830	853	CGCTCATTTTCTCACACCAA
scaffold542432_16.[illegible]	1	p2	(AT)9	18	1635	1652	TATGCAAGAAACATGGCTGC
scaffold542816_14.[illegible]	1	p2	(CT)6	12	333	344	TAAACACTTCGCGCAGACAA
scaffold542999_15.[illegible]	1	p2	(TG)8	16	151	166	AACTCACATCAGATTCCCCAA
scaffold543162_13.[illegible]	1	p2	(GA)9	18	227	244	GCTTGGTTCGAGACTTGAGG
scaffold543204_17.[illegible]	1	p2	(TA)6	12	875	886	TCAGCGGCTTAACATGTTTCT
scaffold543227_12.[illegible]	1	p2	(GA)9	18	865	882	TCCAAATGACCAACCAAACA
scaffold543234_15.[illegible]	1	p2	(TA)6	12	61	72	CGGCTGGATTTCATCCCTAT
scaffold543418_15.[illegible]	1	p2	(GT)7	14	970	983	GCCCTGTTCGTTCATGTCTT
scaffold543507_17.[illegible]	1	p2	(TA)10	20	68	87	TCCAATGGATGAGGATGGTT
scaffold543524_17.[illegible]	1	p2	(TA)6	12	194	205	TATTTTGGACGATTTTGCCC
scaffold543708_18.[illegible]	1	p2	(AG)6	12	399	410	GGCTTTTGAGGGATGGAGAT

scaffold543803_11.3	1	p2	(GA)6	12	364	375 GGCGAGTCTCGTCCTTGTAG
scaffold543910_19.2	1	p2	(TC)6	12	1278	1289 GTTCCTTGCTGCTGTCCATT
scaffold544054_16.2	1	p2	(AG)7	14	45	58 CCATGTTTGTAATTATTGTAGGGAA
scaffold544061_13.1	1	p2	(TA)6	12	169	180 GTGATCGACGGTTGGAAAGT
scaffold544076_22.1	1	p2	(CT)6	12	556	567 GCATGTGAGACCCATTCTCC
scaffold544082_11.3	1	p2	(TC)9	18	238	255 GATTCTGATCGACCAGTGGG
scaffold544117_17.7	1	p2	(TC)6	12	366	377 TCGATTGACCCCTCATCTTC
scaffold544424_11.8	1	p2	(TC)6	12	56	67 GGCTGGGAATTGCTGATATT
scaffold544487_13.2	1	p2	(GA)7	14	154	167 CCTCCCTTAGGGTTCCTCAC
scaffold544646_14.5	1	p2	(TC)9	18	413	430 AGCCGGTCAAGTAGAGGGAT
scaffold544675_14.1	1	p2	(GA)7	14	150	163 CGAGCAAGGAATAGACCGAG
scaffold544766_15.6	1	p2	(TA)6	12	136	147 AATTTAATTCCGTGCAACGC
scaffold544884_14.8	1	p2	(AT)7	14	99	112 ATGTTTCCTCTTGGGTGGGT
scaffold545085_15.1	1	p2	(AT)11	22	423	444 CGACCCTGAGAGATTCCTTG
scaffold545256_10.6	1	p2	(TC)8	16	735	750 TCTCAACATGCTGTCTTCGG
scaffold545380_12.6	1	p2	(TA)8	16	49	64 TCACCATGAAGAAACCCACA
scaffold545478_16.2	1	p2	(AG)6	12	1505	1516 GAGGGTCCGCAAATGTTAGA
scaffold545524_16.5	1	p2	(CT)9	18	2587	2604 TAGAGAAGCCGGATGCAGAT
scaffold545642_17.8	1	p2	(GA)6	12	875	886 ACCTGGGAGCATGTTTCTTG
scaffold545650_11.7	1	p2	(AT)6	12	641	652 TTCGGTTTTTACTCCCACTATG
scaffold545653_17.9	1	p2	(AG)7	14	185	198 ACCACAGCTACCCCGTCTTA
scaffold545663_14.5	1	p2	(CT)9	18	1903	1920 CCAGAATTTGCTGGGTTAGG
scaffold545676_18.3	1	p2	(CT)6	12	41	52 TCAAGCTTAATTAGAAGAAAAGAATG
scaffold545786_11.3	1	p2	(GA)6	12	440	451 TTGGAAAAAGTGGGTTGGAG
scaffold545832_10.1	1	p2	(TA)6	12	262	273 TTCGTCCGAAATACTCCCAC
scaffold545965_11.9	1	p2	(CA)6	12	1961	1972 CTCAAATCTCTGCCCTCTGC
scaffold546043_12.7	1	p2	(AT)6	12	221	232 TGCTGTGGTATGCTTTGATTG
scaffold546051_11.8	1	p2	(AT)8	16	50	65 TTCCCGTATACAAATTCCAACA
scaffold546338_10.5	1	p2	(AT)9	18	340	357 GGATAATCAGAATCCCCGGT
scaffold546431_12.5	1	p2	(TG)6	12	488	499 GTCGTTGTTGAATGTCGGTG
scaffold546483_10.8	1	p2	(TC)6	12	886	897 CCTCTCCTCTCATTCCACCA
scaffold546487_10.4	1	p2	(GA)8	16	214	229 ATTTTCTGTCGAATGGTCCG
scaffold546553_13.3	1	p2	(TC)7	14	67	80 GCTGCATGAACACCAAGAAA
scaffold546579_12.5	1	p2	(TA)7	14	802	815 AATAATAATTCCCTCCTCTCGATT
scaffold546668_10.7	1	p2	(AT)9	18	151	168 CAAATCCCCGTTCCATCATA
scaffold546887_12.3	1	p2	(TA)9	18	98	115 TTTGATATACATGGTATTTAATTTTCG
scaffold546894_16.1	1	p2	(TC)6	12	55	66 CCCTAGCTTTGCCCTCTTTC
scaffold546930_14.0	1	p2	(AT)9	18	51	68 TCGATTTGGATTCGAATTTGT
scaffold547078_16.6	1	p2	(GA)6	12	917	928 TTTGGTAGTTCTGCAAGGGG
scaffold547102_13.8	1	p2	(TA)6	12	592	603 TGGTGACGATTATAAAATCAAAAA
scaffold547135_13.7	1	p2	(GA)6	12	317	328 ACTGGACTTCGACCTGGATG
scaffold547231_16.1	1	p2	(CT)7	14	569	582 GCGGTCCTCTCTCTCAAATG
scaffold547309_12.1	1	p2	(TA)6	12	53	64 CGGACTGAGCTGAGCTAAAA
scaffold547466_18.6	1	p2	(CT)6	12	222	233 CCAGCTCCATCATCTCTTCC
scaffold547470_10.3	1	p2	(TA)6	12	223	234 TCCACCATTGATTTCCAAGA
scaffold547654_15.1	1	p2	(AT)7	14	91	104 GACCCACGTGCACTTCTTTT
scaffold547670_13.4	1	p2	(CA)6	12	300	311 TCGAAAGCATTTCCGAGTTC
scaffold547682_13.0	1	p2	(CT)7	14	425	438 CAGCCTGTGAATTGTTGCAT
scaffold547695_10.2	1	p2	(AT)6	12	94	105 GATGAACACCCGAACCACTT
scaffold547850_14.1	1	p2	(TC)6	12	89	100 GGCAACGGTATACCACTCGT
scaffold547944_10.1	1	p2	(TA)6	12	123	134 GACATCTTCGAGTGCTCGTT
scaffold547969_16.2	1	p2	(CT)7	14	2120	2133 CCCTCTTTCCCTCTCCAAAA
scaffold548030_10.5	1	p2	(CT)9	18	589	606 GCAGCAGAAATGAAGGGAAG
scaffold548080_11.8	1	p2	(TA)6	12	53	64 GATCTCCACCATTTACGGGA
scaffold548088_10.9	1	p2	(AT)7	14	182	195 TGCTAATCTACGAATTTGGGA
scaffold548101_12.4	1	p2	(TA)7	14	110	123 TAAGGGCGGTATCACCTGAA
scaffold548211_17.5	1	p2	(CA)6	12	31	42 TCCACACAGAGACACTGGAACT
scaffold548231_10.9	1	p2	(CT)9	18	280	297 GGAAGATAGGGATAAAAGGCAA

scaffold548317_10.	1	p2	(AC)6	12	35	46	TTCTTTATTCACAATGAACTCAGATT
scaffold548389_15.	1	p2	(AT)10	20	889	908	TCAATGATCACCGGATGAGA
scaffold548394_12.	1	p2	(TA)6	12	1197	1208	AGCTAGGGGTCTGCAAATCA
scaffold548420_15.	1	p2	(TA)11	22	3532	3553	GAAAGGCTCAGCCACTTCAG
scaffold548439_15.	1	p2	(TA)6	12	1277	1288	TCGAGCATTTGCAGACAATC
scaffold548492_15.	1	p2	(TA)7	14	2009	2022	TCAAGGGCTTTTGTTAATTGTG
scaffold548523_14.	1	p2	(CT)6	12	884	895	ATCACGTTGAGGATTCGGAG
scaffold548642_20.	1	p2	(CA)6	12	107	118	GCACTCACACACACGAAAGAA
scaffold548688_16.	1	p2	(TA)7	14	824	837	AGCTCTCTATCATCCACGCA
scaffold548721_14.	1	p2	(GA)7	14	608	621	TCTTTACCCTTGGTTGGTGG
scaffold548756_15.	1	p2	(TA)7	14	5093	5106	AACAAAAATGATGGTGAAGTGG
scaffold548781_11.	1	p2	(TC)6	12	224	235	TTGAACCTTGGGCTAAAGTATT
scaffold548869_16.	1	p2	(TC)6	12	576	587	TGACCACCACCTTGTTGAAA
scaffold548871_15.	1	p2	(AT)10	20	749	768	TGACAAACTCACATGGGTCAA
scaffold548934_14.	1	p2	(CA)8	16	1073	1088	CAAACATTTGAGGAAGGCGT
scaffold549006_11.	1	p2	(AT)6	12	218	229	CAAGCTTGGCGTTATGAATG
scaffold549065_16.	1	p2	(CT)8	16	357	372	ACCCCTTGTTTGCCTTTCTT
scaffold549189_15.	1	p2	(AT)9	18	568	585	GCATCGTCTCAACCCTTGAT
scaffold549201_15.	1	p2	(GA)6	12	1121	1132	AGGCATTTTTGAATTGGGTG
scaffold549206_15.	1	p2	(AG)6	12	4751	4762	GGGTACAGCTGCCACAAAAT
scaffold549313_14.	1	p2	(AT)7	14	245	258	ATTTTGCCCACGAATTAGCA
scaffold549324_15.	1	p2	(CT)6	12	381	392	ACCCATCTCCTAACACGCAT
scaffold549403_11.	1	p2	(TA)6	12	77	88	GGTCTGTTTTCCATGGCCTA
scaffold549483_10.	1	p2	(TA)6	12	182	193	ACGTGTCGCAATCAGAATGT
scaffold549522_20.	1	p2	(CA)6	12	136	147	CACAACACACACGGACATCA
scaffold549525_10.	1	p2	(GA)8	16	42	57	CACGAGTGAAGAAAGGGAGAG
scaffold549577_15.	1	p2	(TG)7	14	371	384	GAACACGACACGATACGCAC
scaffold549595_16.	1	p2	(CT)6	12	136	147	GCATCTCTCCCTTGCTCTCTT
scaffold549705_15.	1	p2	(AT)10	20	483	502	TGAAGACATGGTTGGACAAAA
scaffold549767_10.	1	p2	(GA)8	16	73	88	TGAAACGGAGGGAGTACACA
scaffold549803_13.	1	p2	(TA)7	14	155	168	GATGCTGACCTAACCCCAAA
scaffold549820_18.	1	p2	(CT)7	14	58	71	TGGTTATGCCTTCTTCTTCCA
scaffold549902_12.	1	p2	(TA)6	12	359	370	ATGCGTTCCCAATAGCATTC
scaffold549908_16.	1	p2	(CT)7	14	156	169	CTGCAAAAGCATCACCAGAA
scaffold549950_17.	1	p2	(AT)7	14	722	735	ATCAATTTTTGAGTTATGATGTTTGT
scaffold549977_13.	1	p2	(TA)10	20	103	122	GTGGACGCTCCAGATTGTTT
scaffold550135_10.	1	p2	(CT)7	14	116	129	GAGGGATTTTGGGACGATTT
scaffold550146_15.	1	p2	(AT)11	22	76	97	TTGTGTGGTTGTGATTTAGTTATTTG
scaffold550178_19.	1	p2	(CT)6	12	235	246	AGACTAAAGGCCAAGAGGGC
scaffold550185_17.	1	p2	(AG)10	20	690	709	ATGACAGTTGGGTTTCTGGG
scaffold550186_14.	1	p2	(CT)7	14	155	168	ACGTCTCTTCATGCACCAAT
scaffold550261_12.	1	p2	(TA)8	16	200	215	TTCAACATACAGGCACGAAAA
scaffold550296_12.	1	p2	(TA)8	16	198	213	GGAACCCATCCACCCTATTT
scaffold550523_15.	1	p2	(GA)7	14	4540	4553	ATTTTGGCGCGAAACATAAG
scaffold550548_18.	1	p2	(CT)6	12	59	70	GTCACACACACTCACGGCTT
scaffold550708_13.	1	p2	(TG)6	12	309	320	CCGAAATCTCAAAATCCGAG
scaffold550971_11.	1	p2	(AG)6	12	419	430	ATGGTCCCCTCAACACAAGA
scaffold551117_11.	1	p2	(TC)7	14	76	89	CCTAGCCTTTGCCCTCTTTT
scaffold551260_17.	1	p2	(AT)6	12	157	168	CAAACATTCATCAATATACACACGA
scaffold551306_10.	1	p2	(GT)8	16	76	91	CTGGAAGCACGTGACAGCTA
scaffold551489_12.	1	p2	(AG)6	12	393	404	CAGAGAAAAGTGAGCTCGGG
scaffold551538_15.	1	p2	(TA)11	22	2161	2182	CAGGTGGAGTTTCATCCACA
scaffold551597_18.	1	p2	(CT)6	12	1020	1031	GCTCCTCCCTTACTTGCCTT
scaffold551614_15.	1	p2	(AG)9	18	880	897	TGGTTCACGTAAAACAGCCA
scaffold551618_11.	1	p2	(CT)8	16	313	328	ATAATTCAACCCACACCCCA
scaffold551741_10.	1	p2	(TA)9	18	295	312	AAACCCATCACCCACGTTTA
scaffold551754_12.	1	p2	(TA)9	18	755	772	TTTGCGAGCAAATGAATAAGA
scaffold551800_10.	1	p2	(TA)8	16	219	234	CCTTACGAGGCCAAGAATGA

scaffold551848_13.[illegible]	1	p2	(TA)6	12	243	254 TTTGGGCTATTTCCCAACAG
scaffold551941_17.[illegible]	1	p2	(GA)7	14	941	954 TCATCCCATGCGTGTATGTT
scaffold551952_11.[illegible]	1	p2	(AT)7	14	54	67 GGCTTTATGATCCATTCTCAAA
scaffold551965_14.[illegible]	1	p2	(TC)6	12	462	473 GTTCCTCAAGCCGAAATCTG
scaffold552111_16.[illegible]	1	p2	(GA)10	20	922	941 CCAAACCAATTCAAAAGGGA
scaffold552132_11.[illegible]	1	p2	(TA)6	12	272	283 TCCCAGATGTTGAAATGGTG
scaffold552153_14.[illegible]	1	p2	(TA)9	18	294	311 ATTCCAAAAATCAAAGGCCC
scaffold552264_10.[illegible]	1	p2	(CT)8	16	654	669 TCCCTAATTCCAATTCCCCT
scaffold552288_11.[illegible]	1	p2	(TA)11	22	43	64 TGCGTTTTGACAATATTTAGTGTTT
scaffold552360_10.[illegible]	1	p2	(TA)8	16	48	63 ATCGGTTATTACCGGATCGC
scaffold552395_13.[illegible]	1	p2	(AT)11	22	236	257 CACATAAAGGATTCCCGTGG
scaffold552604_11.[illegible]	1	p2	(TA)6	12	139	150 TCAAAAACAGAGGAGAAGGGA
scaffold552690_16.[illegible]	1	p2	(CT)6	12	1449	1460 GCTTGATCGGCCTTGTAAGA
scaffold552898_13.[illegible]	1	p2	(AT)6	12	133	144 GGCCAAGTCTGGCCTATTTA
scaffold552919_12.[illegible]	1	p2	(AG)7	14	190	203 CACGGTTAGGCTCGAGAGAG
scaffold553085_28.[illegible]	1	p2	(TC)6	12	292	303 TTTGTGGCAACAAGTTTTCG
scaffold553295_16.[illegible]	1	p2	(TC)9	18	106	123 TCGATTTCAGCATCATTGGT
scaffold553331_14.[illegible]	1	p2	(CG)6	12	46	57 ACCAATGGTGGTATGGTGTG
scaffold553473_18.[illegible]	1	p2	(CT)6	12	210	221 TCGTATGATCCAACTTCGCA
scaffold553529_11.[illegible]	1	p2	(AG)9	18	255	272 GCTAATGGTGTTGCTGGGTT
scaffold553545_17.[illegible]	1	p2	(GA)8	16	381	396 TGGCAGAACAAAACTCATGC
scaffold553570_16.[illegible]	1	p2	(AG)7	14	157	170 TATGAGGCCAAACTGGGAAG
scaffold553589_17.[illegible]	1	p2	(AT)6	12	7942	7953 CCATGCCACCTTCTATTGGT
scaffold553785_16.[illegible]	1	p2	(GC)6	12	2420	2431 GGTGGTATGGTGTGGAGGAC
scaffold553822_16.[illegible]	1	p2	(AT)6	12	697	708 GCGAAAATTCCACCATAGTCA
scaffold553845_14.[illegible]	1	p2	(TC)13	26	994	1019 CAACAAATTAGCTGAATGGCA
scaffold554002_14.[illegible]	1	p2	(AT)6	12	440	451 AGGGAGATCGACGTGAGAGA
scaffold554140_10.[illegible]	1	p2	(GA)7	14	52	65 TGGGGCCTACGAGAAATATG
scaffold554161_20.[illegible]	1	p2	(CT)6	12	296	307 GTCACCTCACACACCACTCG
scaffold554228_11.[illegible]	1	p2	(AT)7	14	228	241 AAACAATGCCCGAAGATTGA
scaffold554469_18.[illegible]	1	p2	(GA)7	14	516	529 AGAAACGAAAGCACGACGAT
scaffold554497_15.[illegible]	1	p2	(TA)7	14	91	104 TCTTATAGCATCAACCGCCA
scaffold554694_11.[illegible]	1	p2	(TA)7	14	163	176 TGCAATCCAGTCGCATTTAG
scaffold554868_17.[illegible]	1	p2	(TA)7	14	52	65 GGGGTTATCATGGGATCAAG
scaffold555019_13.[illegible]	1	p2	(CT)13	26	1305	1330 AACTGCCCCTCTATTCCGAT
scaffold555038_15.[illegible]	1	p2	(AG)8	16	573	588 TCAGGATCAGAACACCATCAA
scaffold555065_12.[illegible]	1	p2	(TA)6	12	2168	2179 GAATCGTAACATGTAACACACAAAA[illegible]
scaffold555076_13.[illegible]	1	p2	(TA)7	14	676	689 ACCGTGCAATCCAATTTAGC
scaffold555353_14.[illegible]	1	p2	(TA)6	12	295	306 GGCGGCATTCTATGAGGTAA
scaffold555599_16.[illegible]	1	p2	(AC)8	16	47	62 GAAAAGCTTGGCCATTCAAC
scaffold555678_10.[illegible]	1	p2	(AT)6	12	52	63 TGATTGTAAAACATGTATGGGATTG
scaffold555837_13.[illegible]	1	p2	(AT)6	12	135	146 ACCTCGTGATCATTCTTGCC
scaffold555844_14.[illegible]	1	p2	(CA)6	12	81	92 TTAGATCCGAGATGGTTGGG
scaffold555903_11.[illegible]	1	p2	(TA)7	14	114	127 AATAAGTTCACTTTATGCGAAAGAA
scaffold555974_15.[illegible]	1	p2	(CT)6	12	265	276 AACCCCCAACCAACTCCTAC
scaffold555981_10.[illegible]	1	p2	(TC)6	12	82	93 CCGCCATTGACCTCATACTT
scaffold556006_17.[illegible]	1	p2	(CA)7	14	471	484 CGTGTCTGGTGTTTCAATGG
scaffold556037_15.[illegible]	1	p2	(GA)8	16	1058	1073 GAAGCCCACAAAGTTGCATT
scaffold556055_13.[illegible]	1	p2	(TA)10	20	46	65 TGACGGGATTTAGGTGTTTTG
scaffold556067_17.[illegible]	1	p2	(CT)7	14	410	423 CTCTCACCAACTCGCTTTCC
scaffold556089_12.[illegible]	1	p2	(TC)7	14	105	118 TGCTTCATTGGTGGTAACGA
scaffold556121_10.[illegible]	1	p2	(CT)6	12	420	431 AACCATGTCGAGGGTTTCAG
scaffold556174_16.[illegible]	1	p2	(AT)6	12	1325	1336 ACGGTTGAAACGTGGGTATG
scaffold556192_15.[illegible]	1	p2	(TA)8	16	197	212 TGAAATGAAATCATGCCCCT
scaffold556269_14.[illegible]	1	p2	(AT)6	12	603	614 CTGCTATTGAAACAACCCCG
scaffold556283_10.[illegible]	1	p2	(CT)6	12	215	226 ATTCACAATGGCAAGGAACC
scaffold556286_11.[illegible]	1	p2	(GA)6	12	542	553 CTCGAGTTTTCTTCGCAACC
scaffold556602_11.[illegible]	1	p2	(TA)7	14	478	491 TGGATGCACCAGATTGTTTC

scaffold556686_12.(	1	p2	(TC)10	20	51	70	CTGCTCCTTCTCACTCTTCCA
scaffold556812_19.4	1	p2	(TG)7	14	65	78	CGGTTATCACCGGATCGC
scaffold556875_15.5	1	p2	(AT)7	14	1219	1232	AGTTGGGAATTGGGATAGGG
scaffold556934_16.5	1	p2	(TA)11	22	60	81	CCTAAAACTTCAAACTGTGGGG
scaffold556952_14.1	1	p2	(CT)7	14	86	99	CTCTTCACTCTCACTAGCCGC
scaffold556985_15.1	1	p2	(CT)8	16	1738	1753	CTCATTTTCTCCGCATCACA
scaffold557004_14.(	1	p2	(GA)6	12	159	170	GCACTTAGGAGAGAGATTGTTGG
scaffold557047_10.2	1	p2	(TG)6	12	291	302	GCTCGAGGCATGTAAGAAGC
scaffold557147_16.3	1	p2	(TA)7	14	1118	1131	CAACATGTGCAGGTTGAGGT
scaffold557180_10.1	1	p2	(GC)7	14	268	281	TTCCGAACATTGCACCATTA
scaffold557232_20.1	1	p2	(GA)7	14	532	545	CTACCCTTTCCGAACATCCC
scaffold557276_13.(	1	p2	(AT)8	16	935	950	AGGACCGAAAGTAGCGAACA
scaffold557296_12.8	1	p2	(CT)7	14	392	405	TGCTTATCGAGTTTGGGACA
scaffold557306_11.9	1	p2	(CT)7	14	261	274	ATTCATTCTCCAACCATCGG
scaffold557313_14.4	1	p2	(TA)6	12	206	217	GTGGTATAGCGGTTGGCATC
scaffold557332_16.5	1	p2	(TC)6	12	211	222	CCATCATTTCCCTCACCTCA
scaffold557435_18.7	1	p2	(TC)7	14	409	422	TTCCATCAATCCACTCCACC
scaffold557470_17.7	1	p2	(AG)9	18	2321	2338	CGTACACCCATTTTGAAGGG
scaffold557475_12.5	1	p2	(AG)6	12	159	170	GGGTGAGTAACGGTGCAAGT
scaffold557476_13.7	1	p2	(AG)6	12	159	170	TTTGGGACTCAACGAAGACA
scaffold557488_11.1	1	p2	(CG)6	12	164	175	GTGTGGAAAAGGGAGTGCAT
scaffold557500_14.1	1	p2	(AG)7	14	1341	1354	AGAGGCTCGCCATTGATAGA
scaffold557574_10.(	1	p2	(GT)6	12	481	492	ATGCGACAGAGTGTGTGAGG
scaffold557614_11.5	1	p2	(AT)7	14	372	385	TGTTTTGGGCCTAAATGGAC
scaffold557697_14.7	1	p2	(AT)11	22	220	241	GGAGAGGAAAAATGGGGAAA
scaffold557765_16.(	1	p2	(TA)6	12	100	111	GCGTTGACGCTATTGAAATG
scaffold557828_11.(	1	p2	(TC)7	14	158	171	AATCGGAGTGGATTTGATGC
scaffold557920_11.3	1	p2	(TA)6	12	183	194	AGAAACGTCGGTGACCAATC
scaffold557982_13.5	1	p2	(TA)10	20	47	66	GGATTTAGCGGTTATTACCTGAA
scaffold557989_15.1	1	p2	(AC)6	12	63	74	CTCTCGAGCCTAACTGTGGC
scaffold558019_14.7	1	p2	(AT)6	12	93	104	TTTCCAATTTTGGGAAATACTATGA
scaffold558075_15.7	1	p2	(GT)6	12	89	100	GGAGAGTTGTAGAGTCAAGTTGTAAA
scaffold558294_15.3	1	p2	(TA)6	12	4394	4405	AAAGAGCATCAAACATCATTTCC
scaffold558310_11.(	1	p2	(AT)9	18	50	67	TTCCCGTATACAAATTCCAACA
scaffold558330_10.4	1	p2	(CT)6	12	543	554	AGAACCACCAAAGCAATACCA
scaffold558377_11.9	1	p2	(TA)10	20	395	414	CGGTACAGATTGCACACGAC
scaffold558501_12.7	1	p2	(TA)6	12	85	96	TCACTTAGCTCTTGATTTTCATCAC
scaffold558671_17.(	1	p2	(GA)6	12	272	283	TTGGAGTCTCTCTGAACTCGG
scaffold558710_12.3	1	p2	(TA)8	16	488	503	CGACCGAATACTCACCCCTA
scaffold558807_14.1	1	p2	(TA)6	12	160	171	CAAGGAAGGAACCTCCATTTT
scaffold558808_15.9	1	p2	(TA)7	14	158	171	TTTCGATATGGCTCGGTAGG
scaffold558810_15.7	1	p2	(AT)8	16	160	175	ACGGCTGGCATTTGTTAGAG
scaffold558855_14.(	1	p2	(AT)8	16	267	282	AAACTGCTGTAGCGAACCGT
scaffold558875_13.9	1	p2	(TC)6	12	571	582	CAATTCTCCATGCACACCAG
scaffold558941_10.3	1	p2	(CT)6	12	83	94	GCCCTAGAATCTGCCCTCTT
scaffold559054_10.(	1	p2	(AG)8	16	699	714	GCCAGTCCAATGGTCGTACT
scaffold559395_17.(	1	p2	(GT)6	12	1579	1590	ATCATCAGCTCGTGTGCTTG
scaffold559424_15.(	1	p2	(GT)6	12	193	204	CTCGTGAGAGTGCGTGAGAG
scaffold559628_16.1	1	p2	(TC)7	14	106	119	ATTCCCAACTGATTGGCTTG
scaffold559895_15.(	1	p2	(AG)10	20	153	172	TGAGCAAGGGACAAATGTGA
scaffold559935_15.7	1	p2	(AT)6	12	612	623	ATTGAAACCATGCCCATTTC
scaffold559963_15.4	1	p2	(TA)12	24	41	64	CCAAATTTAAGGGTTATCACCTG
scaffold559978_11.8	1	p2	(TC)6	12	319	330	TATCCCATGATCGAGTTCGG
scaffold559987_18.2	1	p2	(AG)9	18	154	171	TATGAAGCATAACCATGCCG
scaffold560056_10.1	1	p2	(GA)8	16	390	405	CTGAGTGCCTGTCACGAAAA
scaffold560209_13.8	1	p2	(TA)7	14	67	80	TTGAGCTGTTATGCGTTTTGA
scaffold560222_10.(	1	p2	(TA)9	18	498	515	GTTTTAGAGTTGGCATCGGG
scaffold560259_14.7	1	p2	(AC)7	14	2118	2131	GGCCAAGGGAAAGCTTTTAT

scaffold560291_18.9	1	p2	(TC)6	12	232	243	CGGCTTGAAAACGAGAAATC
scaffold560350_14.1	1	p2	(GA)7	14	90	103	GCAGGAAAATGAAAAGTGGG
scaffold560464_16.7	1	p2	(CT)6	12	743	754	CTTCTCCTTGTCGTGCCTTC
scaffold560569_11.7	1	p2	(AT)7	14	452	465	AGTAAGCAACTCCGGTGGGT
scaffold560607_16.3	1	p2	(AG)6	12	1332	1343	CTCTGGAAGAAGAGCCATCG
scaffold560639_13.9	1	p2	(TC)9	18	219	236	AATTTTGACGGCTCCAAGTG
scaffold560740_11.3	1	p2	(CT)6	12	146	157	GCATCTACTCTGGCTCTCATTG
scaffold560834_16.5	1	p2	(TC)6	12	2839	2850	AATGGACCGGACGTGAACTA
scaffold560851_13.5	1	p2	(TA)9	18	139	156	AACATGTTGATCTAAATAACGCAG
scaffold560857_11.9	1	p2	(TA)10	20	985	1004	CGTATTGCAGTGAAATTTTAGCA
scaffold560899_15.9	1	p2	(TC)10	20	1794	1813	CAGACATGTGTACCACCCCA
scaffold560969_13.9	1	p2	(AC)6	12	806	817	ACCGACCACCCTCTTTTTCT
scaffold561070_10.6	1	p2	(AT)7	14	98	111	CACCTGATCGCGACCCTAT
scaffold561218_17.9	1	p2	(TA)6	12	1787	1798	TCTACACGCGCATGTCATTT
scaffold561260_13.6	1	p2	(TA)7	14	160	173	TTACCACAATTTCCCTTGTTTT
scaffold561304_12.3	1	p2	(TG)6	12	439	450	GTTTCAGTTGGCAGTGCAGA
scaffold561453_12.6	1	p2	(TA)6	12	94	105	TTTGCCCTTATTTAGCACGG
scaffold561464_12.4	1	p2	(TA)6	12	535	546	GACAATTGAAAGAGCACTCGC
scaffold561612_15.1	1	p2	(GA)6	12	347	358	ACCTGCCCAGATCTCTCTGA
scaffold561613_15.8	1	p2	(AG)7	14	348	361	CGGTTTCGAGAGAGAAGTGG
scaffold561727_13.7	1	p2	(AT)7	14	67	80	CGTATATTTGCGGGGTTGTT
scaffold561754_19.2	1	p2	(AC)6	12	1016	1027	GTAAGGCCAGGCACATTCAT
scaffold561839_12.6	1	p2	(TA)6	12	161	172	TCGAATTTCTAGCGTGATGG
scaffold561840_15.8	1	p2	(TA)6	12	161	172	GCTTAATGCGTAGCTGGGAT
scaffold561841_10.4	1	p2	(AT)10	20	53	72	CAAACGTGAGTGGGAGATGT
scaffold561868_12.2	1	p2	(AT)10	20	654	673	ATGGCGTTAGAATGCGAAAC
scaffold561885_17.3	1	p2	(TG)8	16	2299	2314	AGTTTTCGTGGCCTCACATT
scaffold562217_14.0	1	p2	(CA)6	12	317	328	TGATAGCCAAAATTTACAACGTG
scaffold562222_12.0	1	p2	(TA)10	20	601	620	TGTGCTTATCCACCGACAAA
scaffold562231_14.4	1	p2	(AG)7	14	94	107	AAAACTACGATCTGCCACCG
scaffold562239_16.5	1	p2	(CT)8	16	158	173	TCAATTGCGGCTTAAACTCC
scaffold562316_15.5	1	p2	(GA)7	14	704	717	GAAATTCGCCGCAGTATAGC
scaffold562358_12.7	1	p2	(AT)9	18	579	596	AATTTTTGTTTGCTGCCGAT
scaffold562426_18.7	1	p2	(TA)6	12	258	269	ATTACGTCGATCCAACGGTC
scaffold562454_10.4	1	p2	(AG)6	12	380	391	GGATGGATGAAAAGGCTCAC
scaffold562505_14.2	1	p2	(TA)7	14	624	637	CACCAAATTTTATCATTTTTAATTCTC
scaffold562583_10.0	1	p2	(AT)7	14	49	62	TTGGAGCTTCAAAACTTAGGC
scaffold562623_12.7	1	p2	(CT)6	12	1092	1103	CACTTCACCATTCCACCATTT
scaffold562626_13.3	1	p2	(AG)6	12	572	583	ACTTCGTGGATTGGATTTCG
scaffold562635_17.7	1	p2	(GA)8	16	58	73	GCGACAATTCTGCGTGTTAT
scaffold562686_16.6	1	p2	(TC)12	24	2686	2709	TGAGCATAATCTTGGGGCTC
scaffold562688_12.7	1	p2	(TG)7	14	67	80	CGGTTATCACCGGATCGC
scaffold562697_11.3	1	p2	(AT)8	16	78	93	TTCGGAGCATGATTTAAGTGG
scaffold562808_11.0	1	p2	(CT)7	14	83	96	TTCCCCTTTGCCCTCTATTT
scaffold562856_11.4	1	p2	(TC)6	12	407	418	ATCAGCGACGAGTCCAAGAC
scaffold562890_14.3	1	p2	(TA)10	20	526	545	CCAACAAAAGGTGTGTGCAT
scaffold562958_12.1	1	p2	(AG)6	12	154	165	GCGACAGAAAAAGAACGAGG
scaffold562967_15.1	1	p2	(CT)9	18	829	846	GGGTTGAAAATGGTGTCAGC
scaffold562985_16.7	1	p2	(GT)6	12	3496	3507	TGCTGCTGCTGATCAAATTC
scaffold563044_17.6	1	p2	(TA)8	16	1544	1559	CGAGGCTTAATGGGGACATA
scaffold563053_15.7	1	p2	(TA)7	14	719	732	GCCACCTCACTCTTGCTACC
scaffold563133_16.8	1	p2	(GA)6	12	4354	4365	CGATTGAGTTTCAGCGCATA
scaffold563196_17.5	1	p2	(AT)7	14	159	172	AAACCGCTCGAATCCGTACT
scaffold563197_11.5	1	p2	(TA)7	14	160	173	CTTTAGGGCCCTTGATTTCC
scaffold563215_11.7	1	p2	(CT)6	12	95	106	TCTTGGTTGGTGGAAGAACA
scaffold563220_16.5	1	p2	(CA)6	12	286	297	AGGATTGTGGAGGATGGTGT
scaffold563255_13.2	1	p2	(TA)6	12	1151	1162	CAATTTTCAATCACAAGTTATAGTGG
scaffold563510_12.1	1	p2	(AT)9	18	139	156	AATTATTTCGGGCTAACGGG

scaffold563554_13.!	1	p2	(AT)7	14	168	181	AGTGATGGAACCAACCTTGC
scaffold563620_16.!	1	p2	(AT)6	12	97	108	TCCCTTGCTTTCTAACCCCT
scaffold563709_11.!	1	p2	(GA)6	12	242	253	TTCCTTTGTCATTCCAACCA
scaffold563752_15.4	1	p2	(TC)6	12	162	173	ATAAGTTTGTGCCTCCCGTG
scaffold563879_14.3	1	p2	(AG)10	20	832	851	TGGGAAAGTGTTGGGATTTC
scaffold563932_13.8	1	p2	(GA)6	12	2574	2585	TCCTAAGCTTTTCCTGCTGC
scaffold563953_17.2	1	p2	(CT)6	12	525	536	TGTTTGATGGCTCTTTGCAG
scaffold564036_11.!	1	p2	(AC)6	12	79	90	CGAAGTGTTGATTGAATTGCC
scaffold564148_16.2	1	p2	(AT)6	12	2412	2423	GTACAATCTTCCTGCCACCC
scaffold564242_20.4	1	p2	(TA)6	12	134	145	CTATTGAAACAACCCCGGAC
scaffold564251_12.!	1	p2	(AC)6	12	469	480	TGGCAGTTACCTATCCCCAC
scaffold564312_12.1	1	p2	(AG)6	12	231	242	GGAGGTGAATGGAGAGTGGA
scaffold564395_13.3	1	p2	(TA)7	14	221	234	ACACATTTTTCTTTTGGGCG
scaffold564462_18.1	1	p2	(AT)9	18	155	172	TCTTCGGTCCGACCTTGTAT
scaffold564465_17.6	1	p2	(GA)6	12	433	444	GGAACCCATGAATTTGTGCT
scaffold564515_13.0	1	p2	(TC)6	12	54	65	TCTCTCACCTCACCTCTCCA
scaffold564538_16.7	1	p2	(AG)8	16	37	52	TGAAGAACGATTACAAAGTTTATCCA
scaffold564579_11.7	1	p2	(TA)7	14	160	173	CACAGAACATAATTCCGCAAAA
scaffold564630_18.!	1	p2	(TA)8	16	158	173	GAGGGGTGGACTATTTCTTGC
scaffold564646_19.3	1	p2	(TA)8	16	357	372	CCAGGGCATATACCCATTTG
scaffold564706_12.3	1	p2	(TA)8	16	322	337	GTACCTGGCTGTAGGATGCC
scaffold564717_14.3	1	p2	(CT)6	12	1596	1607	ACCTACCGTCACAATCTCGG
scaffold564724_15.0	1	p2	(AT)6	12	1193	1204	ATTGGATGGAAATGCTCTGC
scaffold564772_10.8	1	p2	(TA)8	16	69	84	ACAAACTAATCAAGTTCCGCA
scaffold564776_13.4	1	p2	(AG)6	12	80	91	ACGGTGGGCCGAGTATATTA
scaffold564797_10.9	1	p2	(AG)9	18	484	501	AGTGAAGGTGACGAAATGGG
scaffold565051_13.3	1	p2	(TG)6	12	262	273	TGCTCTAGGTAAACCCACGG
scaffold565097_14.3	1	p2	(TC)7	14	1385	1398	CCATTCCTGCAAACCATCTT
scaffold565098_15.0	1	p2	(AG)9	18	157	174	TTCTTGGCGTGGTGTTGTAG
scaffold565111_14.!	1	p2	(GA)8	16	1156	1171	GAGTCGTTCGGGTACGTGTT
scaffold565137_21.4	1	p2	(TA)9	18	388	405	TTTCAAATGGAATTCATATAGCCA
scaffold565267_10.6	1	p2	(CT)7	14	182	195	GCCACTCAGCCTACATCCAC
scaffold565292_11.0	1	p2	(AT)11	22	245	266	ACGAGACTGTCGGTCAACAA
scaffold565336_10.4	1	p2	(AT)8	16	200	215	ATTGCAATGACTTGGCATGA
scaffold565357_17.1	1	p2	(CT)6	12	140	151	GGATCGAAAAGAGAGGGAGG
scaffold565407_11.9	1	p2	(AT)12	24	906	929	CGGTCGTTTTCCAATTTGTT
scaffold565538_15.3	1	p2	(TA)6	12	1133	1144	CATCAATTTTCAGGCCAACA
scaffold565619_15.7	1	p2	(AC)7	14	1696	1709	CCTGCGATATCCTCACCAAT
scaffold565636_18.2	1	p2	(AT)7	14	1849	1862	TGGTGAGAGAAAATGGAGGG
scaffold565677_16.9	1	p2	(TA)6	12	1347	1358	CCGCCGAAAAGACAAATAAC
scaffold565809_20.4	1	p2	(TC)6	12	420	431	CTCCAACTCCATCACCATCA
scaffold565819_16.4	1	p2	(GA)7	14	333	346	TTGCAAAGTCAGACGTCATTG
scaffold565878_10.9	1	p2	(AT)8	16	386	401	CCTCAGTTAGGATTCACTGATCATATT
scaffold565964_16.7	1	p2	(TC)6	12	192	203	TGACACCTAGCCCATAACCC
scaffold565978_14.3	1	p2	(AT)8	16	179	194	TCATTTGATCACAAACCCCC
scaffold565995_13.8	1	p2	(AT)6	12	266	277	TGTGCAGAAAACAGAGCTGG
scaffold566013_14.1	1	p2	(TC)6	12	708	719	CAGCTGACTGACAGAATGGG
scaffold566026_11.4	1	p2	(GA)7	14	329	342	TACCCCAATAATCAGCCCAA
scaffold566083_18.1	1	p2	(AT)9	18	464	481	CGGATTCAAGCTGTGAGAAA
scaffold566115_15.9	1	p2	(TC)8	16	522	537	GGATGTTGAGATCAGAACCACA
scaffold566295_17.9	1	p2	(CT)6	12	1854	1865	TTGGGAACTGGATACAAGGC
scaffold566366_13.7	1	p2	(AT)11	22	108	129	TGAGAAGGAAATTAATGTGTTTTAAG
scaffold566395_11.2	1	p2	(AT)6	12	89	100	TGTCATGTAATAGCCGTGCAA
scaffold566425_15.!	1	p2	(GA)6	12	2011	2022	CAGCACTCTGCATTTCCCTT
scaffold566539_13.0	1	p2	(TA)7	14	287	300	GCTTGAAGGATGAAGGTTGG
scaffold566575_12.2	1	p2	(TA)7	14	207	220	GCGAGTTGTTTCATGCCATA
scaffold566696_13.6	1	p2	(CA)8	16	314	329	GCTCAAAAGCACATCTGCAA
scaffold566713_18.2	1	p2	(AG)6	12	164	175	TGGCATTTTCCCACGTTAAT

scaffold566720_14.(	1	p2	(TG)7	14	135	148	CATTTGGTGAGGAGGGAGAA
scaffold566756_14.8	1	p2	(AC)7	14	722	735	CATGTTTTTGCGCAATTTCT
scaffold566772_12.9	1	p2	(AT)6	12	267	278	TTCTCAGCATATGGGAAGGG
scaffold566830_11.(	1	p2	(GA)6	12	323	334	GAGAGTGGAGAGAGAGAGGGAA
scaffold566861_14.3	1	p2	(TA)11	22	88	109	CCCTTTATTTGGTTGATCTAATGG
scaffold566885_20.(	1	p2	(TC)6	12	423	434	TGTGCGAGTCGGTAATTCTG
scaffold566929_17.1	1	p2	(TA)6	12	100	111	TAGCATGCAAAAGGAACACG
scaffold566974_13.9	1	p2	(TG)6	12	1684	1695	GAATTGGTATGCAAACGTGG
scaffold567063_16.(	1	p2	(TC)7	14	644	657	TTTGACGGCACAATTCTGTT
scaffold567128_10.8	1	p2	(TA)9	18	475	492	CACGTGGCATGATAATAGCG
scaffold567273_17.(	1	p2	(CA)6	12	76	87	CCTCACCCATTCACACACAC
scaffold567281_16.(	1	p2	(AT)7	14	262	275	CACATTTGCAGATGACAGCA
scaffold567287_12.1	1	p2	(TA)10	20	65	84	TCCAATGGATGAGGATGGTT
scaffold567478_14.1	1	p2	(AT)7	14	990	1003	GCCACTTGAGGGAGTTGAAA
scaffold567594_12.(	1	p2	(TA)13	26	39	64	TAAGAAATGCATGCACCTGA
scaffold567631_10.2	1	p2	(TA)8	16	304	319	CTGCTCGTTAATGCGAGTGA
scaffold567690_14.4	1	p2	(TA)12	24	86	109	CAAAAATCTAACGGCCCAGA
scaffold567693_10.9	1	p2	(TA)6	12	337	348	ATTGTGTGACGGGAAAAACC
scaffold567744_21.(	1	p2	(AG)6	12	42	53	CGCTCTATCTTCTTTCTCTTCAGG
scaffold567770_16.3	1	p2	(AT)6	12	180	191	AAATAGCAAATGTTTAGCATGCAC
scaffold567804_15.4	1	p2	(TA)6	12	2523	2534	TTTTTGGGAACTTGTCCTGG
scaffold567861_17.9	1	p2	(AT)8	16	111	126	CATTTTCAATGACCCGAACA
scaffold567942_15.5	1	p2	(TC)7	14	161	174	CGTGACCTTTTGGTCTCAGG
scaffold567943_16.9	1	p2	(TC)8	16	159	174	CCTGATCCTCCTCCATTTCA
scaffold567951_13.1	1	p2	(CT)6	12	70	81	GAGGACATTTGGACCTGAAAA
scaffold568052_14.8	1	p2	(GA)7	14	4573	4586	AAAATTGCAGTGCCATCACC
scaffold568086_12.(	1	p2	(AT)9	18	211	228	GTCGCAATTCAAGCGACATA
scaffold568170_16.9	1	p2	(CT)6	12	488	499	TGAGAAATGCAAAAAGCATGA
scaffold568177_12.2	1	p2	(GT)6	12	163	174	GATATACCGGAAGGGGGAGA
scaffold568178_13.7	1	p2	(CT)8	16	111	126	CACTACTAGGGTTTGGGAGGG
scaffold568252_12.9	1	p2	(AT)6	12	243	254	TGTCACCCAAGTCTCATGCT
scaffold568265_12.5	1	p2	(TA)8	16	365	380	AGGATTTGGGTTGTGAGGTG
scaffold568346_10.5	1	p2	(AT)9	18	263	280	CCGATTGTGAGCATGAAAAC
scaffold568371_15.2	1	p2	(CT)6	12	128	139	AAGAGGGGCGACGAAATTAG
scaffold568405_16.1	1	p2	(TC)6	12	1216	1227	GCCCCATCTCCATCTATTCA
scaffold568640_14.(	1	p2	(AT)6	12	408	419	TCGCTGAGAACACAAGCATT
scaffold568703_11.1	1	p2	(CT)6	12	348	359	GTCAACAGCTCGGTACAGCA
scaffold568707_13.1	1	p2	(TA)8	16	97	112	TCAATCTAACGAAATCTGCGG
scaffold568719_13.9	1	p2	(AT)10	20	411	430	GTTTTTGGTTGTCGTGGGTT
scaffold568758_10.(	1	p2	(GC)7	14	287	300	CACTATTATGGCCGACCGTT
scaffold568823_13.4	1	p2	(AT)6	12	89	100	GGATATCCGAAACATCCGATT
scaffold568828_14.8	1	p2	(TA)7	14	162	175	TGCACCCATACCTACACGAA
scaffold568897_10.2	1	p2	(TA)7	14	201	214	AAACCCTTCTGCCCCTTCTA
scaffold568922_10.(	1	p2	(AT)6	12	257	268	AGATGGAACGACCCTGTGAG
scaffold568930_11.2	1	p2	(AG)6	12	69	80	AACCACCACCATTGAAAGGA
scaffold569112_18.1	1	p2	(TC)6	12	400	411	TCCAACACATCCCTACAACTTTT
scaffold569140_15.1	1	p2	(GA)6	12	3243	3254	GGCTGATGTTGTTGTTGGTG
scaffold569145_16.5	1	p2	(AT)10	20	1070	1089	CCAAAAATATGGCCGTGAAC
scaffold569250_12.(	1	p2	(AC)6	12	65	76	GTTAGGAAACGAAAGGGGGA
scaffold569413_13.9	1	p2	(GA)6	12	252	263	TGGCTGAGGATGAATGAACA
scaffold569448_14.4	1	p2	(CT)8	16	2168	2183	TGCATGCATTTATTTGACAGC
scaffold569456_14.1	1	p2	(TA)8	16	1285	1300	TTTTGGAGATTCTGGGTTCG
scaffold569605_13.2	1	p2	(TA)9	18	1076	1093	TTAAGGTGGTGTTTGCATGG
scaffold569609_16.(	1	p2	(TA)7	14	1626	1639	AAGGTGATCTACGTCCACGG
scaffold569684_11.8	1	p2	(TA)8	16	50	65	TAAAAACAGGGGCAAAGGTG
scaffold569781_17.4	1	p2	(AT)7	14	162	175	AACTCGAATTGCAACGAAAA
scaffold569872_17.(	1	p2	(TG)6	12	1972	1983	TGGGAGGATCTTCAGATTGG
scaffold569911_16.(	1	p2	(AG)6	12	122	133	CCCAAAACATGCAAACACAG

scaffold569943_18.(	1	p2	(TA)8	16	46	61	TGCGTTTTGACGATATATAGTGTTT
scaffold570049_17.4	1	p2	(GA)6	12	2288	2299	AAACCTTCTGGCTGAACCAA
scaffold570085_14.9	1	p2	(GA)6	12	419	430	GAAGCACATTGCAGCAAAAA
scaffold570105_18.8	1	p2	(TA)12	24	306	329	TTGTGCTGAAAATCTCCTCAGA
scaffold570235_14.4	1	p2	(GA)6	12	3089	3100	AGCAAGTCAATTGGGGAATG
scaffold570245_19.1	1	p2	(AT)8	16	161	176	GAAGAAAGAGGGAAGTGGGG
scaffold570287_18.3	1	p2	(TA)8	16	67	82	CGGTTATCACCGGATCGC
scaffold570313_15.(	1	p2	(GA)11	22	471	492	TTGCACTCTGTGGAGCAATC
scaffold570441_16.(	1	p2	(AG)7	14	58	71	GATTTGATTCCTTTTGTCCCC
scaffold570591_13.5	1	p2	(TA)6	12	1015	1026	TGCATTACAGCTTGTTGCAG
scaffold570592_12.9	1	p2	(AT)9	18	601	618	GCAATTGATTGAAATGCCCT
scaffold570616_11.9	1	p2	(TC)6	12	211	222	AGAGGCGGCCTTTTTATTCT
scaffold570680_17.4	1	p2	(GT)7	14	88	101	TCCTCTTTCAGGTCCAAGTG
scaffold570721_12.(	1	p2	(GT)6	12	501	512	GATCAAGCTTCAGACCGGAG
scaffold570867_14.(	1	p2	(TC)6	12	264	275	GTGGGCTATGGCTCAAGTGT
scaffold571049_10.9	1	p2	(AT)10	20	83	102	CGTCCACGACCTCCACTATT
scaffold571050_15.(	1	p2	(GA)6	12	4839	4850	CAAGAAACCATGGAGAATGGA
scaffold571122_15.4	1	p2	(TA)7	14	452	465	CCATGAATCTTTCACATTTTTCA
scaffold571314_15.(	1	p2	(TC)6	12	55	66	TCTCTCTCTTTTTGGTTGGTGA
scaffold571363_16.2	1	p2	(CA)6	12	2140	2151	TTTCCCAAATTATGCTCACTCA
scaffold571404_13.3	1	p2	(GA)6	12	1331	1342	GTGATCTCGCCGCTTTAGAC
scaffold571712_13.2	1	p2	(AC)6	12	110	121	CTCTCTCGAGCCTAACCGTG
scaffold571731_12.8	1	p2	(TA)9	18	330	347	GGTGCATAGAAATTGCTGCC
scaffold571780_13.8	1	p2	(CT)8	16	270	285	TTTAAAGGGTGGTCCCATCA
scaffold571856_10.3	1	p2	(AT)6	12	280	291	ATTCACTTCGGCAAAATTGG
scaffold571857_15.(	1	p2	(CT)8	16	1060	1075	GACGTGTAAACCATCACCCC
scaffold571925_12.5	1	p2	(CT)7	14	53	66	AATCCCTTTCTCCTCTCCCA
scaffold571966_15.1	1	p2	(TA)6	12	489	500	TTTCAAAATTTGGCACTTAGCA
scaffold571992_14.3	1	p2	(AT)10	20	1311	1330	TCTTCCTCGCAATCACTTTCT
scaffold572265_15.7	1	p2	(CT)6	12	1426	1437	TTGCTGGCGACATTATCAAG
scaffold572289_14.3	1	p2	(TA)13	26	41	66	GAGGGTTATCATGGGATCAAG
scaffold572297_16.1	1	p2	(TA)12	24	430	453	TCATATGCGTTCGTCATTCC
scaffold572415_14.9	1	p2	(CT)6	12	395	406	TCTTCCCTCCCTCATTTCCT
scaffold572621_10.(	1	p2	(AT)9	18	243	260	ATGGCGTTAGAATGCGAAAC
scaffold572622_14.8	1	p2	(AG)7	14	2440	2453	TCATGCAAACAAACATGGTG
scaffold572672_18.(	1	p2	(CT)6	12	1235	1246	TGATCGGCCTTGTATCACAC
scaffold572762_12.9	1	p2	(AT)9	18	284	301	AATACCACCCGATCATTTGAA
scaffold572917_11.7	1	p2	(CT)6	12	332	343	TGGGATGAAAGGCTTCAAAC
scaffold572993_12.7	1	p2	(TA)9	18	955	972	TCCTCCGTTAAAGACTGTTCATC
scaffold573045_12.8	1	p2	(AT)8	16	162	177	TCCCAACTTTTTCTTCAAAATCA
scaffold573074_16.5	1	p2	(TA)9	18	41	58	GGAAATAAGGAGGTGTTATGATATG
scaffold573221_16.7	1	p2	(CT)6	12	1441	1452	GCTGGACTTGTGCAACAGAA
scaffold573223_11.3	1	p2	(TG)6	12	342	353	CCGAGCTGAAAATCCACAAT
scaffold573226_14.9	1	p2	(TA)8	16	113	128	GCACGCAAATAATGGTTTCTT
scaffold573229_10.7	1	p2	(CA)6	12	373	384	GGATAAATGTGGCGAATGCT
scaffold573319_11.5	1	p2	(TC)6	12	928	939	CAGTGCTGTATTGCAACGGT
scaffold573338_17.(	1	p2	(CT)6	12	54	65	CACGTTAGACCTTGAAGGGG
scaffold573439_15.2	1	p2	(AT)8	16	1210	1225	AATCCTGCAGATGGGATGAC
scaffold573497_11.8	1	p2	(TA)7	14	571	584	TGCGGATCTGTGAAAAAATGT
scaffold573586_17.(	1	p2	(TA)7	14	51	64	TGAAGTTAGGAAATAAGGAGGTGC
scaffold573637_15.5	1	p2	(TG)8	16	1526	1541	TTTGGGAGGTTCTTCAGGTG
scaffold573693_14.9	1	p2	(AG)6	12	47	58	CTCTGGATATTGCTCGTCGG
scaffold573732_23.2	1	p2	(TA)7	14	605	618	CAGGCTGGATGAACTGAGTG
scaffold573736_12.8	1	p2	(AT)7	14	389	402	GAATAAAACGGCGCCTACAA
scaffold573831_16.(	1	p2	(TA)11	22	94	115	TTTATTTTGGCGTTTCGTGC
scaffold573991_16.5	1	p2	(TA)6	12	1026	1037	CCACCGAGGAACATACAAGG
scaffold574001_16.(	1	p2	(GA)8	16	631	646	TATAAGCCAACCATCTCCCG
scaffold574175_13.4	1	p2	(AT)6	12	514	525	GGTCATTTCTGTTTATTTCGATTG

scaffold574192_17.5	1	p2	(AG)13	26	386	411	CTGGGGATGGCGTTATTAGA
scaffold574207_16.3	1	p2	(CT)8	16	906	921	ACCAGACCCACACGTTTCTC
scaffold574218_16.1	1	p2	(CT)6	12	1071	1082	ACTTTGCCACCTCATATGCC
scaffold574278_17.1	1	p2	(TA)7	14	164	177	GTGACCCGGTCTGATGAGTT
scaffold574293_14.3	1	p2	(TA)7	14	111	124	TCATCTACAGGTCAATGGTGACA
scaffold574294_11.4	1	p2	(TA)9	18	47	64	ATGGGTTATCACCGGATCG
scaffold574311_15.1	1	p2	(AT)9	18	145	162	GAAAAATCAAAGGCCCACAA
scaffold574553_14.3	1	p2	(GA)6	12	970	981	ATGGCATTCCCCATACACAC
scaffold574601_10.0	1	p2	(TA)8	16	209	224	AAATGCACACCCCTTTCAAT
scaffold574625_14.4	1	p2	(AG)6	12	135	146	ATGAACACCCCTAGTGACCG
scaffold574639_16.2	1	p2	(GA)6	12	181	192	TTATTCTTGATGGAACGGGG
scaffold574869_10.6	1	p2	(TC)9	18	85	102	TGGCCATGTTGGTAGATTCA
scaffold574923_11.7	1	p2	(TC)6	12	1128	1139	TTGCCTACATCATCCCACAA
scaffold574942_11.8	1	p2	(TA)10	20	852	871	TGGTTGCACCATAACACACC
scaffold574976_16.5	1	p2	(GC)6	12	95	106	GGTGGTATGGTGTGGAGGAC
scaffold575005_16.0	1	p2	(TC)7	14	781	794	CACCCCCAGATGAGCTAAAG
scaffold575075_11.6	1	p2	(TC)7	14	74	87	TCGCTCACCGAGATAGGTTC
scaffold575114_12.3	1	p2	(TG)6	12	658	669	CTCACCGCCTTAATATGCGT
scaffold575124_15.0	1	p2	(TA)6	12	65	76	TTCTAATCAAATCAAGCGACCA
scaffold575158_16.6	1	p2	(GA)6	12	145	156	ACCACCACATTACTGCCACA
scaffold575222_15.3	1	p2	(AG)6	12	1409	1420	CGTGCGACGACTTAACTGAG
scaffold575250_13.5	1	p2	(AT)6	12	916	927	ATTCGAATGTCTTGCCCTTG
scaffold575273_17.9	1	p2	(TC)6	12	341	352	CGTAGAGAATGTTGCCACGA
scaffold575296_19.0	1	p2	(CT)6	12	247	258	TTCCTGCTGTTCACAACTCG
scaffold575304_10.9	1	p2	(AT)6	12	192	203	TTTCATCTGGTTCCCTTTGG
scaffold575361_14.5	1	p2	(AT)8	16	1200	1215	CATCTGGGCCTCTCTGGATA
scaffold575414_17.5	1	p2	(TC)6	12	211	222	CAGGCAAATCAGGCAATTTT
scaffold575493_15.7	1	p2	(GA)6	12	510	521	ATTTGATGGAACGGGGATTT
scaffold575519_10.2	1	p2	(TA)6	12	88	99	TGTGTGATGTTGGAGTTGGG
scaffold575528_15.9	1	p2	(GT)6	12	1796	1807	TGATGGTTTATGCTCGTAAGGTT
scaffold575602_16.5	1	p2	(AT)6	12	634	645	TCCTTGTTGCACTGTGGTGT
scaffold575619_16.9	1	p2	(AG)7	14	129	142	TTCCACTTGAAACTATCAGTCCA
scaffold575674_13.4	1	p2	(AG)7	14	51	64	CGGCACGAATCCAGTTTTT
scaffold575683_17.9	1	p2	(CT)7	14	176	189	TCATCTTGCAAGCTCCATCA
scaffold575745_12.2	1	p2	(AT)7	14	209	222	TCTTATAGCATCAACCGCCA
scaffold575761_13.8	1	p2	(AT)6	12	271	282	CCCATCATCTCAATCCAACT
scaffold575764_13.3	1	p2	(AT)6	12	538	549	AAATGAGTGGCTGGATTTCG
scaffold575774_11.1	1	p2	(AG)7	14	644	657	GAGAGAGGAAGGGAGATGGG
scaffold575780_15.1	1	p2	(TC)6	12	736	747	CCGTGAATTCATGCCTGTC
scaffold575827_14.4	1	p2	(AT)11	22	228	249	TCATCCCCAAAATACCCTCA
scaffold575952_11.5	1	p2	(AT)8	16	243	258	AATTTCTCTGGCCGACACTG
scaffold576028_15.1	1	p2	(TA)6	12	195	206	TCAATAAACCCTCCGAATGC
scaffold576165_12.1	1	p2	(TA)6	12	321	332	AATCGATTCAACTCATGGGG
scaffold576196_13.6	1	p2	(TA)9	18	493	510	GAAGAAGGTGATGTCATGGGA
scaffold576378_11.3	1	p2	(AT)12	24	530	553	TGCGTGCATGTATATGCTGA
scaffold576439_17.3	1	p2	(TA)10	20	463	482	CCATAACCGTCGCATAACTC
scaffold576527_14.7	1	p2	(GA)6	12	67	78	GGGGAGGGTTTAGGTAGAGG
scaffold576538_11.2	1	p2	(CT)8	16	691	706	AAAAAGAAAAAGGGGTGGGA
scaffold576566_12.9	1	p2	(AG)6	12	251	262	CCGATGATGACGAACCTTCT
scaffold576606_13.8	1	p2	(AT)8	16	49	64	GGGGTGACAACTATCAACCAA
scaffold576687_10.9	1	p2	(AG)6	12	112	123	CGGCCATTCTCTTTTCCTTT
scaffold576693_15.5	1	p2	(GA)7	14	164	177	AAAGGTGAACTTGGGGCTTT
scaffold576740_14.9	1	p2	(CT)7	14	117	130	AAGCTCCACCATCTCTTCCA
scaffold576804_15.2	1	p2	(AT)6	12	2546	2557	TTTTTACAAACCTGCCACCC
scaffold576826_12.3	1	p2	(TA)8	16	366	381	ATTATATTGTACGTAAAAGCGAAAAA
scaffold576903_13.5	1	p2	(AC)6	12	114	125	CTTACCCAATCTTGGTGAACA
scaffold576978_14.4	1	p2	(AG)8	16	683	698	ATTTCTCTCAGTCGGTGCGT
scaffold577031_13.2	1	p2	(TA)6	12	94	105	CGGAGGGAATACAATGCTTTT

scaffold577068_15.(	1	p2	(AG)6	12	166	177	TTGGTTTGGTAGCCCTTTTG
scaffold577136_15.7	1	p2	(AT)6	12	552	563	GGAATATTAGCGGTGGCTATG
scaffold577194_11.1	1	p2	(AT)7	14	357	370	GGCACTTTGGCTACTTTTGC
scaffold577238_14.9	1	p2	(TA)6	12	100	111	GCGCAGATCTAATGGTCCAG
scaffold577306_12.5	1	p2	(AT)8	16	808	823	GACCTTTTTACTCCCAGCCC
scaffold577370_13.(	1	p2	(GA)7	14	867	880	TAGCAATTTGATTCCGTGCT
scaffold577424_13.(	1	p2	(TA)6	12	398	409	CGAGTCCATGGAAACAGATG
scaffold577511_16.7	1	p2	(TC)8	16	1130	1145	CCACAAGTTCTCCACAGCAA
scaffold577513_16.9	1	p2	(AG)8	16	45	60	GAAGAACAATTACAATGTTTATCCAA
scaffold577613_15.3	1	p2	(TC)6	12	1880	1891	ATTCGTTCAAAAACGGCAAG
scaffold577637_16.(	1	p2	(TA)6	12	228	239	ACGCAAATACGACTGGCTTT
scaffold577639_18.3	1	p2	(AG)6	12	2031	2042	GAAGATGAAGGCAAGTGGGA
scaffold577642_16.5	1	p2	(AG)10	20	1305	1324	GCATCCTTCCAACAAGTAGCA
scaffold577806_14.(	1	p2	(TC)6	12	186	197	TGCTTCGATTCTCAATGTTCC
scaffold577807_13.2	1	p2	(TC)7	14	190	203	GCTTCGATTCTCAGTGTTCCA
scaffold577811_13.7	1	p2	(GA)6	12	478	489	CGAGAAACTTTCAAGGACGC
scaffold578132_17.2	1	p2	(AT)8	16	455	470	CGATGAACTAGGGGCTGAAG
scaffold578155_16.4	1	p2	(TC)6	12	1159	1170	TGCTTACTTTCCGTTTTGGG
scaffold578337_11.2	1	p2	(TA)6	12	548	559	TATTCTGAAACATGCTGGCG
scaffold578340_17.(	1	p2	(TA)8	16	46	61	AAGGGTTATCACCTGATTTTCTC
scaffold578433_14.7	1	p2	(AT)9	18	331	348	TTCAATACTTCCAGCCACCA
scaffold578483_10.(	1	p2	(AT)8	16	113	128	TGTGTTCTTGAGTCGTTTGGA
scaffold578491_21.(	1	p2	(AT)6	12	467	478	ACTTCAATTTTGGGTTCCCC
scaffold578596_10.2	1	p2	(CT)6	12	476	487	AGTGAGGGGATGTTGGAGTG
scaffold578642_12.4	1	p2	(AG)6	12	254	265	GCCCATTTGAGCCAATCTAA
scaffold578667_15.(	1	p2	(TA)9	18	745	762	GAATACAGGCCGGCTAACAA
scaffold578727_15.8	1	p2	(AG)9	18	3622	3639	TGCGGTCATGTGATGATTTT
scaffold578810_13.7	1	p2	(AT)7	14	59	72	ACATGCAGGGCTGAGTACAA
scaffold579003_17.4	1	p2	(CT)7	14	36	49	AAAATTTTCCTCACTGCACG
scaffold579028_13.4	1	p2	(TC)8	16	164	179	TGGTTTTGTGCTTCTTGCTG
scaffold579038_17.2	1	p2	(TA)7	14	24	37	CAGTTATCACCGGATCGC
scaffold579075_14.7	1	p2	(CT)7	14	378	391	CCCCTTCCCATCTCTACTCC
scaffold579118_11.1	1	p2	(TA)7	14	52	65	TGGCCCATGTACACTGCTTA
scaffold579194_16.2	1	p2	(TA)6	12	816	827	ACCTCGACGACAGGAAAAGA
scaffold579231_13.1	1	p2	(TA)6	12	1158	1169	ACTTCATGGGGGAAAACAGA
scaffold579279_16.(	1	p2	(TG)8	16	463	478	CCCATGATTCCATGATCTCC
scaffold579315_11.(	1	p2	(GA)6	12	167	178	CCAAGAGACGTTTTTGTTGAAA
scaffold579406_15.4	1	p2	(AC)7	14	450	463	TTGCAGCACTTTCAACATCA
scaffold579468_16.(	1	p2	(AT)6	12	512	523	GAAGCACATGCAAGGATTGA
scaffold579555_18.1	1	p2	(CT)6	12	33	44	TCTCTCTCGTTCTTCCTCTCTCT
scaffold579586_14.4	1	p2	(AT)8	16	163	178	GGATCGTACCTTTTGCTTGC
scaffold579587_13.8	1	p2	(AT)8	16	163	178	GAAAAGCCTGTTGGCAGGTA
scaffold579588_16.4	1	p2	(TA)8	16	176	191	CCCAAAAATCTAACGGCCTA
scaffold579632_20.4	1	p2	(CA)6	12	726	737	TTATTCGGTTCCGCCATTAC
scaffold579794_10.1	1	p2	(TA)9	18	308	325	CATCATTCAAGGGGCATTTT
scaffold579841_15.1	1	p2	(AT)6	12	734	745	GCATATCGCTCGATCAAAAA
scaffold579980_12.8	1	p2	(TC)6	12	54	65	GGTTTCTGAATTTGATCCGC
scaffold580125_16.2	1	p2	(AG)7	14	1699	1712	AAAGGACCGAAGAGGTGGAT
scaffold580128_16.1	1	p2	(TA)6	12	73	84	GTTATCACCGGATCGCAAC
scaffold580132_17.(	1	p2	(TC)10	20	978	997	GTTGGTCTGGCAACCATCTT
scaffold580141_11.7	1	p2	(TG)6	12	157	168	CCGTTGCACAAGTCACAGAG
scaffold580243_16.7	1	p2	(AC)6	12	1088	1099	TATGATAGGCCACCGGTTTG
scaffold580313_20.4	1	p2	(TA)6	12	378	389	CCCATCGAAAAATTAATGCAA
scaffold580327_10.5	1	p2	(AT)9	18	233	250	ATGTGAGAGACCCAGGTTCG
scaffold580547_17.8	1	p2	(TA)9	18	862	879	TTTCAAATACGGTAGACTTCCCA
scaffold580560_16.8	1	p2	(GA)8	16	240	255	TGATTCACACGAGAGTGCAG
scaffold580709_11.2	1	p2	(TA)8	16	165	180	GGGGTTATCACTGGATCACG
scaffold580754_12.3	1	p2	(AT)8	16	603	618	TTCCCTTCATTTTCGTTTTATTTT

scaffold580788_12.(	1	p2	(TA)7	14	480	493	GGGTTATCCCCGCTATCATT
scaffold580828_13.:	1	p2	(AT)6	12	137	148	CATTACCCACACACACACTTCA
scaffold581004_16.(	1	p2	(TA)7	14	166	179	AGAAGTTAGCACGTCAAGGGA
scaffold581058_13.:	1	p2	(AT)8	16	99	114	GTTCCATGTTACCCGTGGAC
scaffold581122_13.(	1	p2	(CT)9	18	367	384	GGGCCCATTATTTCATCTCA
scaffold581188_12.:	1	p2	(TA)7	14	411	424	CATGTATTTTTCCTATGGACCGA
scaffold581193_12.(	1	p2	(AT)6	12	264	275	ATCCGATGACTGGATGCTCT
scaffold581236_13.(	1	p2	(AT)7	14	147	160	TTTTCTTGATAAGCTCGAAGCA
scaffold581369_17.(	1	p2	(CT)8	16	165	180	TCATCATCACACGCTGCTTT
scaffold581473_10.(	1	p2	(GA)6	12	219	230	TTCAGTATCGGGCCATTTTC
scaffold581479_19.!	1	p2	(TG)8	16	102	117	AATGCACTCATGGAAAAGGC
scaffold581722_16.:	1	p2	(AT)6	12	1385	1396	GCTCAAGGTCACACAAGCAA
scaffold581779_10.:	1	p2	(TC)6	12	40	51	TTTCTCTTGGTGGAAAACTCTT
scaffold581840_15.:	1	p2	(GA)6	12	361	372	AGATTGTTGTGAGGCGGAAG
scaffold581858_12.(	1	p2	(TG)6	12	77	88	TGGAGTTGAGATAATTTGCTGG
scaffold581910_11.:	1	p2	(TA)9	18	48	65	TGCGTTTTGACAATATATAGGTGTTT
scaffold581929_15.:	1	p2	(AG)8	16	39	54	GTGCACAAGCAGGGGAAA
scaffold581963_14.:	1	p2	(CT)6	12	994	1005	TCTACGTTCCTCAGGCTGCT
scaffold582130_11.(	1	p2	(GA)7	14	645	658	GCTTTCCTTCCTCCTTGCTT
scaffold582218_20.(	1	p2	(AT)7	14	116	129	TCTCTCGTTAACGTCCCATTT
scaffold582223_11.:	1	p2	(AT)7	14	54	67	CATAAACTAAGGTGGAGATTGTTATG
scaffold582253_10.:	1	p2	(TC)6	12	49	60	TTGTTATGTTTCCATGAGCAGA
scaffold582341_12.:	1	p2	(AT)8	16	199	214	GGATCTTATAGCATCAACCGC
scaffold582389_18.:	1	p2	(AC)6	12	509	520	CCGTTTTTACACCCCCTACA
scaffold582535_11.:	1	p2	(CT)6	12	282	293	AATTGCGAGTCGCTGATTTC
scaffold582548_19.(	1	p2	(TA)9	18	683	700	GGAATAAAACCGTTTCACCG
scaffold582646_15.!	1	p2	(CA)6	12	38	49	CTCTCACACTTTGCACACTGAA
scaffold582727_18.:	1	p2	(AT)7	14	605	618	TGTATGGCCACTTAGGCAAA
scaffold582883_10.(	1	p2	(TA)6	12	456	467	GTTCATCGTGAACTCGCGTA
scaffold582920_11.!	1	p2	(AT)6	12	658	669	AGCGTGCCATGTGTTATCAG
scaffold582934_17.(	1	p2	(TA)11	22	67	88	AAATCTTGTGGATGTTAGTGGTTAG
scaffold582977_18.(	1	p2	(TG)6	12	6939	6950	AAATCTGAAGAGCTCCACGC
scaffold583012_13.4	1	p2	(GA)6	12	97	108	TCGTTCGTCCTCTTCCTGTT
scaffold583100_16.(	1	p2	(TC)6	12	315	326	TGATCCACGGTCTTTACGAA
scaffold583106_15.(	1	p2	(AG)7	14	638	651	ACACACAACCACCACCAGAA
scaffold583165_13.4	1	p2	(AT)9	18	549	566	AGTTATGATGGGAAGATGGGG
scaffold583337_19.:	1	p2	(GA)6	12	87	98	GACGATTTGCAAGCCTATGG
scaffold583392_16.4	1	p2	(CA)6	12	531	542	GGGGATACACACCTACCGTC
scaffold583560_16.(	1	p2	(TC)8	16	4605	4620	TCCAAAATGGAAATGGGAAA
scaffold583566_22.(	1	p2	(TA)7	14	167	180	ACTCCCCAAAAATCCAAACC
scaffold583646_16.(	1	p2	(GA)8	16	706	721	CATGAGCAGGCTGTTTTTGA
scaffold583778_10.!	1	p2	(AT)8	16	69	84	AGTTATGATGGCAGGATGGG
scaffold583935_16.:	1	p2	(TA)8	16	853	868	GACCAGACCTGGACCTACCA
scaffold583988_11.(	1	p2	(CT)6	12	113	124	CGACAATCATCTAGTCCTCTAGCA
scaffold584103_14.(	1	p2	(TG)6	12	785	796	CACCAAAAGGAAGCCATCAT
scaffold584156_10.4	1	p2	(AT)6	12	53	64	ACGACTGTCGATCCACACAA
scaffold584194_15.(	1	p2	(CT)6	12	688	699	CCCTCCATCCAAAGTTGCT
scaffold584230_13.:	1	p2	(TC)7	14	117	130	AGCTTCTCTTGCTCTCTCATTG
scaffold584319_17.(	1	p2	(TC)6	12	383	394	AATTTCTGCATGTTTTCGGC
scaffold584324_12.4	1	p2	(TA)7	14	313	326	TGAGAAATAAGAAACAATCTGGGG
scaffold584328_14.:	1	p2	(GA)6	12	80	91	TGGTTGTGTGAACCAACTCTG
scaffold584341_10.4	1	p2	(AT)7	14	375	388	TCCATGACTCTCAATGCCTTC
scaffold584538_13.:	1	p2	(AC)6	12	289	300	CATCTTTCTTCTTCGACGCC
scaffold584646_15.:	1	p2	(TA)6	12	4341	4352	TGGATCAAACGTCGTCTCAC
scaffold584671_11.:	1	p2	(AT)8	16	437	452	TCTCCGAATTTCAAGAGGGA
scaffold584683_12.(	1	p2	(TG)6	12	191	202	TTTCGTCCATTGTAAGCAGTTC
scaffold584706_10.(	1	p2	(TA)11	22	251	272	TTCTCAGCATATGGGAAGGG
scaffold584993_12.(	1	p2	(TA)7	14	51	64	GCATATGTGCGTTTTATTCTTTC

scaffold585030_13.8	1	p2	(TA)8	16	358	373	CCCATCGGGAACAAGTCTAA
scaffold585161_15.8	1	p2	(AG)7	14	755	768	GCTCTTATGATGGCCGAAGT
scaffold585165_10.4	1	p2	(TA)7	14	324	337	AAAAGCAATTAATGCGTTCACA
scaffold585280_15.0	1	p2	(AT)6	12	193	204	CATTGCTACTGAATATTGTTCTTGTTC
scaffold585352_17.0	1	p2	(TA)6	12	348	359	CTTCAGTCCGTGAAAGGAGC
scaffold585398_14.4	1	p2	(TA)6	12	1381	1392	GTGGACCGCATAGGAATCAT
scaffold585400_13.4	1	p2	(TG)6	12	99	110	CCCATTTGTGTTTTCTTCGTG
scaffold585420_10.9	1	p2	(AG)6	12	292	303	CCTGCTTTGTGCAATCTTCA
scaffold585507_15.2	1	p2	(TA)6	12	566	577	TCTCACATCGCCATTTTCAA
scaffold585578_16.2	1	p2	(AT)10	20	77	96	AAGAGGAAAATCCATCGGCT
scaffold585592_10.2	1	p2	(AG)14	28	594	621	GGCATCACTGTCAGCAGGTA
scaffold585687_16.1	1	p2	(GA)6	12	172	183	TTGTTGAATTGGTCCTGCAA
scaffold585737_11.5	1	p2	(AT)7	14	168	181	TTCAAATCTTTTTCACCGCC
scaffold585774_19.2	1	p2	(AT)7	14	549	562	GAAAGGGCATGGTAGTCAGC
scaffold585816_17.0	1	p2	(TA)6	12	170	181	TGCATCACTACCTCCTTGTTG
scaffold585858_11.9	1	p2	(TA)6	12	221	232	TTGGTTGGATTGGATTTTGG
scaffold585961_16.2	1	p2	(AT)6	12	727	738	CCAAATCGGACTAATGCTGAA
scaffold585979_11.8	1	p2	(AT)6	12	225	236	AACTCGATAACGGATGCTCG
scaffold586020_15.2	1	p2	(TA)8	16	863	878	ATCCCAGCCACACATTTCTT
scaffold586021_16.3	1	p2	(AT)6	12	171	182	CGAGTCCAGGCTACATCACA
scaffold586022_12.5	1	p2	(AT)6	12	1295	1306	AAACCACCACCTTTCTCCCT
scaffold586034_10.3	1	p2	(AT)9	18	188	205	GGTGTTGGTGAGGCTGTTCT
scaffold586126_17.4	1	p2	(CT)6	12	118	129	GCGTGACAACAACAATTTCG
scaffold586152_17.7	1	p2	(GA)8	16	506	521	TCAGTGAGTTTCAAGCCGTG
scaffold586154_20.5	1	p2	(TC)6	12	1378	1389	ACTATCCAACCACACGCACA
scaffold586214_11.0	1	p2	(CT)6	12	158	169	GAGGATTCGACATTGGGAGA
scaffold586341_14.9	1	p2	(GA)6	12	256	267	TACACAGCCCGTGTTCATTC
scaffold586379_18.1	1	p2	(GA)6	12	698	709	TTCCTCAGATTTCTGCACACT
scaffold586400_12.7	1	p2	(AG)7	14	184	197	CTGCTTTGTGTTGCGTGTTT
scaffold586455_16.7	1	p2	(CT)6	12	704	715	TAGGATCTTTTCACGCGGTT
scaffold586466_11.9	1	p2	(GA)6	12	55	66	TTGAAAGATGGGTGTGTTCTTG
scaffold586522_13.3	1	p2	(TA)6	12	120	131	CCCTACCCATTTACCCGTTT
scaffold586645_17.9	1	p2	(AG)8	16	509	524	GGTTCCAACTCCAAGGTGAA
scaffold586750_10.2	1	p2	(AT)6	12	136	147	GTAGTTGGCTGCATTTGTGG
scaffold586752_15.3	1	p2	(TA)7	14	2246	2259	TGCCTTGAAAATGGGAAAAG
scaffold586821_13.3	1	p2	(AT)9	18	957	974	GGATGCGAAATTGGACACTA
scaffold586830_14.3	1	p2	(AT)9	18	249	266	AGGAGAGAGCGAGAGGAGAGA
scaffold586925_14.8	1	p2	(TC)12	24	3580	3603	TAACTGGCCCCAACTTATGC
scaffold586936_12.6	1	p2	(AT)8	16	334	349	GTGAAGCGCTGATCTTGTGA
scaffold587033_12.4	1	p2	(AT)7	14	423	436	TGAGGCAGAATGAATTGTCG
scaffold587102_15.4	1	p2	(AT)6	12	648	659	CGGGTAATGTCGAGAGTGGT
scaffold587105_13.1	1	p2	(TC)10	20	118	137	AAACCAAAGACGATGTTGCC
scaffold587202_15.9	1	p2	(AT)6	12	170	181	TCCCAATCGTTTCAAAATTCA
scaffold587246_14.4	1	p2	(TA)6	12	90	101	CGTAGACCTGGCATCTTGTG
scaffold587270_15.4	1	p2	(AT)6	12	316	327	TTGAAATGTAACACAAAAGGAGAA
scaffold587307_17.5	1	p2	(TC)7	14	60	73	TGATTTGTCATTGTTGGGGTT
scaffold587326_14.2	1	p2	(AT)7	14	230	243	AACGAGAGAACTTTCGACGC
scaffold587377_14.0	1	p2	(TC)6	12	171	182	AAGAGCTTCTGCGTCTTTCG
scaffold587406_14.8	1	p2	(GA)7	14	102	115	CATGCAAGGGTTTCGCTAGT
scaffold587423_12.0	1	p2	(GA)12	24	989	1012	TGCTGGATTAACGATTTCCC
scaffold587430_14.8	1	p2	(TA)6	12	705	716	CATGCTCCTATTTTAATACACCCC
scaffold587455_13.8	1	p2	(GT)7	14	720	733	AATCGAGCTTACACCCCAAA
scaffold587505_10.9	1	p2	(AG)6	12	66	77	GAAGACGACGAGAGGGTGAG
scaffold587521_13.6	1	p2	(TA)6	12	2093	2104	GCAAAAATGTCCCCTGCTAA
scaffold587526_14.8	1	p2	(AT)9	18	295	312	CACCAAATTTCATCATTTTTAATTC
scaffold587648_13.6	1	p2	(GA)6	12	375	386	TCGGTTTTTCTTGGGAGAGA
scaffold587662_10.2	1	p2	(AT)6	12	496	507	CATAACATGATTTTGCCCCC
scaffold587708_12.8	1	p2	(CA)6	12	388	399	CTTGCAAAATGATCCCCAAC

scaffold587721_17.5	1	p2	(TC)6	12	155	166	GGCAAACACAGGATCGATTT
scaffold587778_14.5	1	p2	(TG)6	12	937	948	CTCAAACTTAAGCACCCCCA
scaffold587788_15.9	1	p2	(AG)6	12	425	436	CCCCTCGGAAAATCCTAGTC
scaffold587811_16.7	1	p2	(GA)6	12	920	931	TTTCCCTTCCAATTTTGTCG
scaffold587905_15.7	1	p2	(TG)6	12	3619	3630	TAACGGCCCAGATCTTGTTT
scaffold588053_14.1	1	p2	(AT)6	12	66	77	GGGCCGAGATTTGTTAGAGG
scaffold588081_15.4	1	p2	(AC)6	12	1914	1925	CGTCACTGTTGCTCCGACTA
scaffold588142_15.1	1	p2	(AT)11	22	1072	1093	TTTTGATTTTCAAGTCCGGC
scaffold588186_13.6	1	p2	(TA)7	14	852	865	CGAAATTGAAATCGGACCAT
scaffold588235_14.2	1	p2	(AG)8	16	41	56	AGAAGGATGGCGGAGAGTTT
scaffold588239_11.7	1	p2	(AG)6	12	262	273	TGTGAAAAACAGTGGCAGGA
scaffold588247_14.8	1	p2	(TC)8	16	546	561	CAGCAATTGGTTTTGAGGGT
scaffold588272_16.4	1	p2	(TA)8	16	318	333	TGAGTTTGGTGTGTTGGCAT
scaffold588306_18.1	1	p2	(AC)7	14	1243	1256	CGCGGAGGAAATTATTTTTG
scaffold588492_17.8	1	p2	(TA)6	12	327	338	AGTTCGGGAGTGAGCTCGTA
scaffold588539_14.9	1	p2	(AT)13	26	65	90	GGATATGCTCAGTCCGGGTA
scaffold588543_14.8	1	p2	(CT)6	12	112	123	AGAAACACGTTTTGCTTTTGA
scaffold588780_10.2	1	p2	(GA)9	18	104	121	CACAATGCGTGTATTGACAGTG
scaffold588793_16.4	1	p2	(TC)6	12	505	516	GGCATGAATTCTTGGATTGC
scaffold588826_14.0	1	p2	(TC)6	12	546	557	GACCCGGCCTAACATATTCC
scaffold588894_17.3	1	p2	(CT)6	12	172	183	TGAATTAACAGCGAGCATGG
scaffold588902_15.0	1	p2	(AG)10	20	1930	1949	TTCCCGTAGTCCTGAGATGG
scaffold588980_13.6	1	p2	(TA)8	16	119	134	GCGTCCGACACCTCAATTAT
scaffold589079_14.9	1	p2	(GA)7	14	72	85	GACGCGAGATTTACGTGGTT
scaffold589104_17.1	1	p2	(TG)6	12	2574	2585	ATCAAAGCACTTGAGCCCAC
scaffold589147_13.7	1	p2	(TA)6	12	559	570	CCCCATCCATGTTGTTTTTC
scaffold589162_14.7	1	p2	(AG)6	12	56	67	TAACACCTTCATCGAGCGAG
scaffold589210_15.9	1	p2	(GA)7	14	402	415	GCTCTCTTTTTGGCTGGATG
scaffold589390_10.1	1	p2	(AT)8	16	284	299	GTCCCTTTTTACCCCTCAAA
scaffold589497_10.4	1	p2	(TA)7	14	47	60	GGAAATAAGGAGGTGTTATGATATG
scaffold589681_15.5	1	p2	(TA)8	16	168	183	GCAGTAACCCTCTCATCGGA
scaffold589682_12.8	1	p2	(TA)8	16	315	330	GGAACCAGCTCCGATTAACA
scaffold589683_12.6	1	p2	(TA)7	14	170	183	TGGACGAAAATGTCCTTCTTG
scaffold589879_13.2	1	p2	(TA)8	16	305	320	GACCGAATGTAATGAACCCG
scaffold589909_11.1	1	p2	(GA)12	24	349	372	AGAGCGGGAACAAAAGTGAA
scaffold589962_10.6	1	p2	(TA)11	22	307	328	TGCATGCACCTGATTACACA
scaffold590006_14.6	1	p2	(TA)6	12	1414	1425	GGCAGCTAGGGTTGAAAGAA
scaffold590039_11.5	1	p2	(TC)6	12	166	177	TACACGCACATCAACCCACT
scaffold590117_13.0	1	p2	(TG)6	12	786	797	TTCATTTGCCTGACCTACCC
scaffold590126_14.9	1	p2	(AT)9	18	2195	2212	GAATTGCCATGTCCATGTGT
scaffold590233_14.0	1	p2	(GT)7	14	2003	2016	TGGGGCCAATTTACTCTCTG
scaffold590366_19.0	1	p2	(TA)7	14	386	399	TCCGTAGGGAGCAAAGTGAT
scaffold590422_16.3	1	p2	(AT)9	18	454	471	GATTTTGAGTTCATGGGCGT
scaffold590531_11.0	1	p2	(AT)8	16	225	240	GAACTATCTACAGGCCAGGGG
scaffold590537_16.0	1	p2	(CT)7	14	126	139	TTGTATGGACGACACGCACT
scaffold590553_15.2	1	p2	(TA)8	16	500	515	GGGGTACGTGCCATAAAAAC
scaffold590606_17.0	1	p2	(CT)6	12	972	983	GCATTCTCCAGCGTCTTTTC
scaffold590709_12.4	1	p2	(CT)6	12	158	169	GCATCTCTCTCGCTCTCATTG
scaffold590746_11.6	1	p2	(TC)6	12	491	502	TCCCATTCAAAATCTTTCCA
scaffold590833_16.3	1	p2	(TG)7	14	358	371	CAATCCAGTTAGTCGGAGCC
scaffold590922_13.6	1	p2	(TA)7	14	483	496	CCGATCCCCATATAACCGAT
scaffold590940_16.4	1	p2	(GA)9	18	3699	3716	CTGAATGACCTGCCTCCATT
scaffold590965_10.4	1	p2	(CT)6	12	140	151	GCTCTCTTTGTAATCTCCCCC
scaffold590989_17.7	1	p2	(AT)6	12	351	362	TCCCTCTTCATCCTCCCTCT
scaffold591004_13.8	1	p2	(TC)6	12	346	357	CTGGGTTTCCTTCCTCTTCC
scaffold591038_16.4	1	p2	(CT)7	14	5638	5651	CACAAAACCCTCACACCCTT
scaffold591112_17.6	1	p2	(AG)6	12	803	814	GACCCAGCACTGCTTCTAGG
scaffold591131_13.4	1	p2	(TC)6	12	231	242	CAAGGGCTTGAGTAGCTTGG

scaffold591201_18.8	1	p2	(AT)6	12	241	252	AACGCGTCACACGTAATTCA
scaffold591300_16.6	1	p2	(AG)7	14	1675	1688	GTGCCATAGCCACCTGATCT
scaffold591418_13.8	1	p2	(CA)6	12	258	269	CTCTGTCTAGTGGCGAAGCC
scaffold591595_11.1	1	p2	(AG)8	16	114	129	GGGTGAGACAAGATGAGGGT
scaffold591630_13.7	1	p2	(TG)8	16	65	80	CCTTGACCAATCGATTTCTTG
scaffold591659_11.5	1	p2	(GC)6	12	370	381	CACTCGCTACGAGCCACAG
scaffold591722_10.4	1	p2	(GC)6	12	104	115	CCATCACCGCATTTTCTTCT
scaffold591745_10.5	1	p2	(CT)7	14	67	80	TCACCCACAAACTTTTCAACTTC
scaffold591839_11.5	1	p2	(AT)6	12	392	403	CGAGAAGCTTGCTTCAAACC
scaffold591864_15.9	1	p2	(TC)11	22	5223	5244	TGCAAACGGATGGTCTGATA
scaffold591925_15.6	1	p2	(TA)6	12	53	64	AAAATAATCCCAAAACCAATGA
scaffold592066_14.1	1	p2	(AT)8	16	50	65	AACGCACAACAAAATACAAAAGA
scaffold592112_12.7	1	p2	(TA)11	22	120	141	GCATCATTTTGGCCTTTGAT
scaffold592241_18.2	1	p2	(TC)6	12	370	381	ACCCTGCCCAAAACTCTCTT
scaffold592259_15.0	1	p2	(TC)7	14	744	757	CCCATTTCCATGCTTCAACT
scaffold592270_19.6	1	p2	(TA)7	14	120	133	GCGGGTAAATATCAATTTCGAG
scaffold592287_13.6	1	p2	(AT)11	22	1305	1326	AATTGTGGCCTCACCTTCAG
scaffold592305_13.4	1	p2	(TA)8	16	72	87	TGGGTTAGCACTTGATCGTG
scaffold592442_15.0	1	p2	(AT)12	24	1215	1238	TCAAACTACATTTGGGGGAG
scaffold592531_17.9	1	p2	(AT)6	12	342	353	TCGATTCGAATTTTCATACACA
scaffold592579_15.9	1	p2	(TA)8	16	286	301	TGGTATGACAATTGCAAGCAT
scaffold592611_16.3	1	p2	(AT)7	14	227	240	CAGCCAACTCGATCTCATCA
scaffold592631_17.6	1	p2	(TA)8	16	565	580	GCTTTGTGCATTCTTCCCTT
scaffold592632_15.3	1	p2	(TC)9	18	49	66	TGCTTCTCCAAATCCATCTTC
scaffold592652_12.9	1	p2	(AT)8	16	574	589	AAGTGTGCCCAACACCTTTC
scaffold592676_14.6	1	p2	(TC)6	12	430	441	TTGTTCGAAAAGGAGTTGCC
scaffold592854_12.2	1	p2	(CT)8	16	40	55	CACTCACTCTCACTAGCCGC
scaffold593027_15.8	1	p2	(TC)6	12	154	165	CAATACCGTGACATGCCTTG
scaffold593076_14.3	1	p2	(TA)13	26	1689	1714	ATGGCTGTTGATGTGGACAA
scaffold593214_15.1	1	p2	(TC)8	16	3492	3507	GCCAGAGCTTCTATGGTTGC
scaffold593224_10.8	1	p2	(GA)7	14	228	241	TTCGAACTCCAATAGCAGCA
scaffold593254_17.3	1	p2	(AG)9	18	439	456	ATTTGTGCAAAAGTTTGGGG
scaffold593363_13.0	1	p2	(AT)10	20	397	416	TTGGTGAACCTCTCCCAAAT
scaffold593364_13.4	1	p2	(AT)9	18	168	185	TGGACAAGACAAGGCTAGGG
scaffold593375_16.5	1	p2	(TA)7	14	5302	5315	ATGGCACACCATACCAGGAT
scaffold593389_17.3	1	p2	(AG)8	16	1010	1025	GCCCTTTTCATTGCTGTCAT
scaffold593479_10.0	1	p2	(TC)6	12	107	118	AACGGTGACGAATGGTTCTC
scaffold593504_15.0	1	p2	(AC)6	12	550	561	AATTGGAAACATGGCGAGAC
scaffold593542_13.5	1	p2	(TA)6	12	538	549	GGTCCAAACAACGAACCAAC
scaffold593579_10.6	1	p2	(TC)6	12	357	368	AAAACCCCACACTCTCCTCA
scaffold593613_11.4	1	p2	(TA)9	18	252	269	GAATGGTGGTGTTTGTTCCC
scaffold593649_19.2	1	p2	(AT)8	16	1537	1552	TTAAATGGGGTGACAGGCTC
scaffold593655_10.7	1	p2	(AT)7	14	308	321	CGTGGACAAGTCTCGCCTAT
scaffold593676_10.3	1	p2	(TC)10	20	358	377	TAGTGGTGACAAGTGTGGGG
scaffold593801_10.1	1	p2	(AT)10	20	165	184	CACCCACATTACATTAAACCGA
scaffold593943_13.6	1	p2	(TC)6	12	425	436	TGCCACATCAGCTTCTTCAC
scaffold593970_10.1	1	p2	(CT)6	12	159	170	TCGCTGCCATATTCTTGACA
scaffold593986_18.8	1	p2	(GA)6	12	710	721	AACAGCCATTAAAAGTCGGC
scaffold593987_15.2	1	p2	(AG)6	12	173	184	CCTATTCCCTCGATTCAGCA
scaffold593988_11.0	1	p2	(TG)8	16	66	81	CCTTGACCAATCGATTTCTTG
scaffold594002_10.8	1	p2	(AG)6	12	499	510	ATCCACGAGGATGATGAAGC
scaffold594094_15.4	1	p2	(CT)8	16	1068	1083	TTTTCATTTGATTAGAAGTTCTGGA
scaffold594177_15.3	1	p2	(CA)6	12	500	511	TCAGCAAACTGAAGAACAAGGA
scaffold594189_10.8	1	p2	(TC)7	14	725	738	TAGCCAACTCGCTTAGCCTC
scaffold594233_13.6	1	p2	(AG)7	14	146	159	GTCTGGTAATGGCCGGACTA
scaffold594246_12.8	1	p2	(TC)6	12	126	137	TTGGCAATGAGATACCGATG
scaffold594267_14.1	1	p2	(TC)8	16	893	908	TGAAATTTGAAGTCCATGCG
scaffold594277_11.6	1	p2	(AT)6	12	388	399	CACACGAACTCATTTGACGG

scaffold594438_16.5	1	p2	(TC)6	12	728	739	GAACAAGTTTGAGGGTGCGT
scaffold594451_14.5	1	p2	(CT)6	12	1162	1173	GCGTAACATTATGAAGGCGA
scaffold594463_14.7	1	p2	(TA)10	20	166	185	AAAAAGCGCTCGTACATTTTATG
scaffold594573_11.8	1	p2	(CT)6	12	290	301	AATAAATTCAGCGCAAACCG
scaffold594714_15.2	1	p2	(AT)6	12	721	732	TATGAATTCCGTGTTGGGGT
scaffold594764_15.0	1	p2	(AT)6	12	587	598	TCTTTTTGACATTTGGGTTCA
scaffold594769_15.1	1	p2	(AG)7	14	507	520	TGGGTTGTTCTGATCTGGGT
scaffold594816_15.6	1	p2	(TG)6	12	678	689	CGCATGGTGACATCAGTACA
scaffold594868_18.0	1	p2	(TA)6	12	405	416	TGCGTTAATGTACACAACTTCCA
scaffold594993_14.0	1	p2	(CT)6	12	414	425	GTTGGGAACGAATTGGAGAA
scaffold595018_14.1	1	p2	(TG)8	16	240	255	GCCATGATCTCCTGATTCAAA
scaffold595036_13.7	1	p2	(TA)6	12	1376	1387	ATCACGTTTGCAGCATGTTT
scaffold595161_14.8	1	p2	(TA)7	14	70	83	AGTGTTACGATAGAATCACGCC
scaffold595242_11.4	1	p2	(TC)6	12	198	209	TCTTGCCTCCACCATCTTCT
scaffold595285_10.7	1	p2	(TA)11	22	69	90	TGACGCTTAGAGGGTGTTTTG
scaffold595297_13.4	1	p2	(TA)9	18	40	57	TTTTGACAATATATAGGTGTTTTGACC
scaffold595344_14.8	1	p2	(CT)7	14	275	288	AAATGGCATTGCTCCTCAAC
scaffold595345_15.3	1	p2	(AT)7	14	235	248	CGCGGTGTTTTAACTTGCTT
scaffold595366_15.6	1	p2	(TA)7	14	552	565	CTCGATTAAATGGGACCGAA
scaffold595370_14.5	1	p2	(CT)7	14	548	561	CCTCACGACGACTCCAAAAC
scaffold595393_10.9	1	p2	(AG)8	16	373	388	CTCGTTTCGATCCATTTCGT
scaffold595399_11.8	1	p2	(TC)7	14	435	448	TTGTGTGGGTCGATCTGAAA
scaffold595618_12.1	1	p2	(TA)11	22	1010	1031	TGCGCGTTTTTAGGGTTAAT
scaffold595708_16.6	1	p2	(TA)8	16	170	185	TTTGAGTTGGGAGAGGGATG
scaffold595743_17.0	1	p2	(AG)9	18	566	583	TTCAGTGGCTACAAATCCCC
scaffold595784_16.1	1	p2	(CT)6	12	917	928	TTGTTCCCTGTGGGATTAGG
scaffold595787_12.8	1	p2	(TA)8	16	524	539	TGACGGTTGGATTTTGTCCT
scaffold595807_11.5	1	p2	(TA)6	12	171	182	AGGCTTGAGTTTCCTCACGA
scaffold595880_11.0	1	p2	(TA)9	18	169	186	ATTGGTGACGTGGCATCATA
scaffold595882_18.1	1	p2	(AC)6	12	309	320	TAAAGACATATCGCGACCCC
scaffold595883_14.2	1	p2	(CT)6	12	3462	3473	TCACATCTCGATCTTCGCAC
scaffold595886_16.7	1	p2	(TC)6	12	1417	1428	ACAGTCTGCAGGCCAAAACT
scaffold595926_10.6	1	p2	(CT)6	12	561	572	GCCCAATAGCCCGTATATCA
scaffold595941_14.1	1	p2	(TA)6	12	205	216	AAACGCGTCATGGATAAACC
scaffold595970_21.1	1	p2	(TA)8	16	50	65	ATGGGAAGAGGGGTATGAGG
scaffold596181_14.9	1	p2	(GA)7	14	574	587	GGGACGATTCTGAAAGGTGA
scaffold596222_14.5	1	p2	(AT)8	16	170	185	TGCAATGCATGGAGGAAATA
scaffold596228_10.4	1	p2	(TC)6	12	122	133	CAATTTGCAGCCCTCATTTT
scaffold596307_15.0	1	p2	(CT)8	16	835	850	ATACCCACCCACCCTCTCTC
scaffold596377_21.3	1	p2	(AG)8	16	456	471	CTCGAATTCGGTTCCAGAAA
scaffold596379_10.2	1	p2	(TC)6	12	38	49	ATCTCCTCCTCCACCGTGA
scaffold596380_12.3	1	p2	(TC)6	12	38	49	ATCTCCTCCTCCACCGTGA
scaffold596389_15.6	1	p2	(AC)9	18	3109	3126	CGAGTTGCCAATCATTTCCT
scaffold596409_18.1	1	p2	(CT)6	12	496	507	GTCCACCTTAGGCACGACAT
scaffold596443_22.9	1	p2	(TG)6	12	1242	1253	GACACGGGAAGCGTGATAAT
scaffold596452_13.9	1	p2	(AT)10	20	633	652	CGATCTTGCTCTACGTCTGG
scaffold596523_10.7	1	p2	(TC)8	16	61	76	TCTCGCTCTACACTTTCGGTC
scaffold596573_10.8	1	p2	(TA)10	20	485	504	CGAATCCAGGTAAGGCTTGA
scaffold596627_16.7	1	p2	(AG)8	16	1092	1107	TCTCGACTAGTTGGCGCATA
scaffold596723_13.7	1	p2	(TC)9	18	657	674	CGGCGGCTCTATAAAATTGA
scaffold596762_16.2	1	p2	(AG)8	16	1356	1371	TAGGTCATGGATCGACACGA
scaffold596793_16.1	1	p2	(AT)7	14	6368	6381	TCCAGGGCTACTGGAGGTAA
scaffold596900_16.6	1	p2	(TC)6	12	358	369	TGACTTGGGGTCCATCTCTC
scaffold596976_16.9	1	p2	(CA)7	14	2438	2451	CATGCATCAAGGAAAAACGA
scaffold596991_11.4	1	p2	(AT)7	14	521	534	CATTGCATTTTATGCAGAGCA
scaffold597012_17.1	1	p2	(AT)6	12	754	765	ACTCAGGTGGTTCAAATCGG
scaffold597124_16.0	1	p2	(AT)8	16	1620	1635	GTTGTGTTGCCTGCAGTTTG
scaffold597168_15.4	1	p2	(GA)7	14	407	420	ATCACCCAAAGCCATTTCAC

scaffold597246_13.[illegible]	1	p2	(AG)6	12	279	290	AGTTTGAAGAAGACCGCACG
scaffold597256_18.[illegible]	1	p2	(AG)6	12	249	260	GATGTGTGCCCAAAAGGAGT
scaffold597363_14.[illegible]	1	p2	(CT)10	20	52	71	AGTGCTCGAATATCTCCCCC
scaffold597440_17.[illegible]	1	p2	(AG)6	12	1685	1696	ATTGATGTCCGAATGCAACA
scaffold597663_14.[illegible]	1	p2	(AT)6	12	391	402	CACACCTTCAGAAATGCAATG
scaffold597844_13.[illegible]	1	p2	(TC)7	14	141	154	CCTAGCTTTGCCCTCCTTCT
scaffold597929_10.[illegible]	1	p2	(TC)6	12	287	298	GTCTTGCTCCTCCATTGCAT
scaffold597976_12.[illegible]	1	p2	(TG)7	14	606	619	ATTCAAACCACATCCTTCGC
scaffold598058_12.[illegible]	1	p2	(TC)6	12	34	45	TGTAATCTCTCTCGAAGTTATAGTGA[illegible]
scaffold598091_11.[illegible]	1	p2	(GA)6	12	417	428	CGAGATTTACGTGGTTCGGT
scaffold598230_11.[illegible]	1	p2	(AT)9	18	354	371	ATTAGAGCCACAGCGGCATA
scaffold598286_13.[illegible]	1	p2	(AT)7	14	604	617	AACTCATTAAATGTGCCGCC
scaffold598289_17.[illegible]	1	p2	(AC)7	14	2446	2459	ATTTCGAAGATCAAAGGCCC
scaffold598327_13.[illegible]	1	p2	(TC)8	16	796	811	CATGCCCACAAACACTCAAG
scaffold598350_14.[illegible]	1	p2	(AT)6	12	505	516	TGACATTTACGCAGTTTGTCG
scaffold598357_15.[illegible]	1	p2	(GA)7	14	4641	4654	ATTGTTTGAGCTTGGTTGGG
scaffold598366_14.[illegible]	1	p2	(TG)7	14	909	922	GGCTCCCACAATGAACAACT
scaffold598378_15.[illegible]	1	p2	(GA)8	16	117	132	CGAAGACACGATGTTGAACG
scaffold598476_17.[illegible]	1	p2	(TA)7	14	392	405	CCCAGAATTGTAAAGCGCAC
scaffold598568_17.[illegible]	1	p2	(AC)6	12	103	114	GCACCGAAACAGAGCTGATA
scaffold598605_14.[illegible]	1	p2	(AG)10	20	51	70	AGAATGAGGGAGGGAGGAGA
scaffold598702_19.[illegible]	1	p2	(AG)9	18	170	187	CTCGGAGACCCATTTCTCTG
scaffold598785_12.[illegible]	1	p2	(GA)7	14	389	402	TCTGCAGCTGAGGAAGTTCA
scaffold598802_16.[illegible]	1	p2	(TG)7	14	1593	1606	GTTGGGAAGGGAAGAAGGAG
scaffold598939_17.[illegible]	1	p2	(CT)6	12	722	733	GCTCTTGAATTTGTCCGAGC
scaffold599037_15.[illegible]	1	p2	(GT)6	12	1611	1622	GAGGCTTTTCGCTTTCCTTT
scaffold599049_17.[illegible]	1	p2	(TA)6	12	100	111	TTTTATCCCATGTTTGTGCAT
scaffold599175_17.[illegible]	1	p2	(AG)6	12	3324	3335	CAGCGAATGTAACCGGATCT
scaffold599322_15.[illegible]	1	p2	(CA)7	14	1790	1803	GTTAGGTGACCCACCTTCCA
scaffold599337_12.[illegible]	1	p2	(AT)7	14	491	504	TCTTTCGAGTCAGGGCATCT
scaffold599361_14.[illegible]	1	p2	(CT)6	12	807	818	CTTCAACACATCCATCACCG
scaffold599405_11.[illegible]	1	p2	(TA)8	16	49	64	TGCGTTTTGATGATATATAGGTGTTT
scaffold599512_17.[illegible]	1	p2	(AG)7	14	592	605	GTGGACCACGGTGATCTTTT
scaffold599525_11.[illegible]	1	p2	(TA)13	26	278	303	AAGGGGCACGTAAGCCTAAT
scaffold599559_17.[illegible]	1	p2	(TG)8	16	1073	1088	CTTGTTACGGGGAAAGTGGA
scaffold599563_13.[illegible]	1	p2	(TC)9	18	1004	1021	AACTCCGCGAGAGAAATTGA
scaffold599567_14.[illegible]	1	p2	(CT)7	14	282	295	TTTTTGCACCATTGGTATTG
scaffold599578_12.[illegible]	1	p2	(AT)9	18	527	544	TGACGAATGATACCATTTAAGCA
scaffold599600_11.[illegible]	1	p2	(TA)7	14	313	326	GAACTAAAAACGATGAAATTTGGTG
scaffold599602_13.[illegible]	1	p2	(TA)9	18	243	260	AGTCACAACATTCAAGGGGC
scaffold599635_16.[illegible]	1	p2	(AT)6	12	1036	1047	TGTGGTTTACCCGTCTGACA
scaffold599677_14.[illegible]	1	p2	(AT)8	16	1929	1944	TTTTCACGAATATACATGTTCCAAA
scaffold599755_14.[illegible]	1	p2	(TA)6	12	905	916	TGCCCTAACGCCTTATCTTG
scaffold599771_15.[illegible]	1	p2	(TG)7	14	2349	2362	TGGAAAATCTGTTGCAGGTG
scaffold599916_12.[illegible]	1	p2	(AT)8	16	51	66	GAGAGGAGTGAGATTGAGCGA
scaffold599919_14.[illegible]	1	p2	(TA)8	16	2546	2561	CCCTACCCATTTACCCGTTT
scaffold600080_10.[illegible]	1	p2	(CT)6	12	1011	1022	TTCTTCCCCACTGCTCATCT
scaffold600115_11.[illegible]	1	p2	(AG)7	14	52	65	CTGCTCCATTTTCACAACTGA
scaffold600146_12.[illegible]	1	p2	(AG)8	16	297	312	TTGGCCGTGAGTTCATTGTA
scaffold600193_11.[illegible]	1	p2	(AG)6	12	110	121	TGAGCCGTCAGTGCATTAAA
scaffold600276_11.[illegible]	1	p2	(TA)11	22	208	229	TGATGTAGCCCGCATGACTA
scaffold600294_14.[illegible]	1	p2	(GA)6	12	300	311	ATGCATACATGACTCGAGCG
scaffold600295_10.[illegible]	1	p2	(TC)6	12	617	628	CGGGCCGTGTACTTTACAAT
scaffold600308_18.[illegible]	1	p2	(AG)6	12	107	118	CGCGTTACGAATCCAAGTTT
scaffold600309_15.[illegible]	1	p2	(TA)9	18	205	222	TTTTTAGGGTTTAAAATCGCGT
scaffold600368_12.[illegible]	1	p2	(TA)6	12	542	553	AGATGATGGACAAGGCATCC
scaffold600426_13.[illegible]	1	p2	(TA)11	22	66	87	AAGGCCCACAATGGTTATTAG
scaffold600532_10.[illegible]	1	p2	(GA)6	12	69	80	GAGAGGGGCGAGAGGTAGAG

scaffold600567_12.	1	p2	(CT)8	16	102	117	TTGTGACGGTGACTAGAGCC
scaffold600707_10.	1	p2	(TA)6	12	51	62	ACCATATTTAAGGGTTATCACCTG
scaffold600784_17.	1	p2	(GA)7	14	336	349	AAGGGCTACTGTTCCCGACT
scaffold600812_14.	1	p2	(AG)6	12	115	126	CGTTTCCCATGTTGCTTGTA
scaffold600826_14.	1	p2	(TA)7	14	1000	1013	GATTCTCCCAATCTGACGGA
scaffold600901_11.	1	p2	(AT)6	12	530	541	TTTGGTTGTTTGAGATTGTGG
scaffold601142_14.	1	p2	(TA)7	14	300	313	TGCCTTTCTTTCCCCTCTTT
scaffold601244_15.	1	p2	(AT)7	14	460	473	ACTGTGAACGGCGCTAATCT
scaffold601248_11.	1	p2	(TA)10	20	51	70	TTTCTCACATAACTTAGCTGTTCTCA
scaffold601249_16.	1	p2	(TA)7	14	175	188	TTATGATGGGAATGGTGGGT
scaffold601315_13.	1	p2	(TC)6	12	86	97	TGGCTAATTTGGCAGCTTCT
scaffold601343_16.	1	p2	(AT)6	12	1289	1300	AACCGGTTAAAACCCAGACC
scaffold601417_16.	1	p2	(TA)8	16	457	472	GTTATCACCGGATCACGACC
scaffold601452_15.	1	p2	(AG)7	14	2063	2076	ATTTCAGAATTCGCTGGTGG
scaffold601464_14.	1	p2	(CT)6	12	194	205	GTGTGTGCATGCATTTGTTG
scaffold601503_16.	1	p2	(CT)14	28	1099	1126	GGTGGGAAGAAGTGTCTGGA
scaffold601628_15.	1	p2	(TC)6	12	339	350	CCATCCGAGCAAGAAAGGTA
scaffold601691_13.	1	p2	(TC)7	14	330	343	GCATGATCAATGTTGTAGAGGG
scaffold601750_13.	1	p2	(AT)9	18	814	831	GTCAACCAATGCAGATGCTC
scaffold601808_12.	1	p2	(AC)6	12	782	793	CTCACCAGCACCTTCTCTCC
scaffold601837_16.	1	p2	(GA)6	12	1709	1720	GAACACTCCCTTCTTCACGG
scaffold601969_12.	1	p2	(TA)9	18	266	283	TTAGAAACGGCATACCCAGG
scaffold601970_15.	1	p2	(GA)6	12	465	476	ATTTTCAAGGTTGTGCTGCC
scaffold602214_16.	1	p2	(AT)11	22	1028	1049	GGTTCCCTCTTCTCTAACGCT
scaffold602417_12.	1	p2	(AT)6	12	93	104	AACTCGAGATCGGTCCACCT
scaffold602422_15.	1	p2	(TA)7	14	175	188	CCCAAAGGTCTCATTTCCAC
scaffold602537_15.	1	p2	(GA)10	20	5660	5679	GTTGTCAAAAGCACCAGCAA
scaffold602562_20.	1	p2	(AG)7	14	1090	1103	CGGTCGCAGAAAAAGCTAAC
scaffold602610_15.	1	p2	(TC)9	18	442	459	TCCACAGACAACCTCACCAA
scaffold602630_15.	1	p2	(CT)7	14	146	159	CACGAGGAACTTTGGACGAT
scaffold602745_17.	1	p2	(GA)6	12	137	148	TGGGCGATTGAACACTTACA
scaffold602852_20.	1	p2	(TC)6	12	155	166	GGACTGCTGGATAATCCCCT
scaffold602878_14.	1	p2	(AT)6	12	379	390	ATCACTCCCTGTTACGGCAC
scaffold602884_12.	1	p2	(TA)7	14	80	93	GCTCGGTAAGTCGTTAATGGA
scaffold602948_10.	1	p2	(AT)6	12	167	178	CGATGCTGCTGTGAACAAAC
scaffold602989_15.	1	p2	(TG)6	12	2828	2839	ACGGGAATGGTGTGTTGATT
scaffold603047_13.	1	p2	(GA)6	12	1830	1841	TGTGGCAACACCAATCACTT
scaffold603104_16.	1	p2	(AG)6	12	1310	1321	AAACTCCAGCTACCGAAGCA
scaffold603136_15.	1	p2	(TC)8	16	845	860	TAGCGATGAAGACGACGATG
scaffold603193_15.	1	p2	(AG)6	12	1504	1515	CCATCGTTTGGTGGTTCTCT
scaffold603282_10.	1	p2	(GT)6	12	56	67	TTCGATTTGGGGTGTAACAA
scaffold603289_14.	1	p2	(GA)7	14	2607	2620	TTCCGGCGAGGATATATGAG
scaffold603386_16.	1	p2	(CT)7	14	53	66	ACAAAAAGGGAAGAGCGACA
scaffold603423_14.	1	p2	(TA)7	14	176	189	GGATCCGACGTCCAATATACA
scaffold603449_13.	1	p2	(TG)7	14	469	482	AGTTTCGCGATCTCAACCAG
scaffold603455_11.	1	p2	(TA)6	12	483	494	AAAAATGATGCCAATCCAGC
scaffold603469_14.	1	p2	(GA)6	12	55	66	TGATTCTTTTGTTTGGTTTGC
scaffold603471_16.	1	p2	(AT)6	12	1331	1342	GGTGTTTTTGGATTGGTTGG
scaffold603568_16.	1	p2	(TA)6	12	1118	1129	CCGCAATAATCCATACCTCAA
scaffold603841_16.	1	p2	(GA)7	14	495	508	GGTGATTCGAGATGATCCGT
scaffold603899_14.	1	p2	(CT)6	12	1081	1092	AAACTCACCATTTGCCAACC
scaffold603912_14.	1	p2	(CT)8	16	728	743	TGACGTGGTGTTGTGGTTTT
scaffold603920_13.	1	p2	(AG)6	12	112	123	GTGAATCACACTCCCCCATC
scaffold603931_15.	1	p2	(CA)6	12	1680	1691	CTGCCCAAGAAGGATTTGTC
scaffold603938_14.	1	p2	(TC)12	24	412	435	CGAACTGGGAAAGGAGAAAA
scaffold603947_15.	1	p2	(AT)14	28	420	447	GACACTCTTCTTCACCCCCA
scaffold604026_16.	1	p2	(CT)6	12	266	277	CCGTGGACGTAGGTTCATCT
scaffold604182_12.	1	p2	(TA)12	24	751	774	CTGCTGCCCAAAAACTTAGC

scaffold604267_15.[illegible]	1	p2	(CT)6	12	66	77	CGGGATGCCATTTGTAAGTC
scaffold604337_21.[illegible]	1	p2	(GA)6	12	307	318	GGATTGTTGACTGCCAGGTT
scaffold604360_15.[illegible]	1	p2	(GA)6	12	1872	1883	TCTGGATGTCAAGGCATACG
scaffold604442_16.[illegible]	1	p2	(GA)12	24	4925	4948	AGGTCACCTCCTGCAAGAAA
scaffold604450_12.[illegible]	1	p2	(TA)7	14	302	315	CTGAATGCTTACCGATGCGT
scaffold604503_13.[illegible]	1	p2	(CT)8	16	383	398	TCACCCTTTCTTTGATGGGT
scaffold604560_15.[illegible]	1	p2	(TA)8	16	425	440	GTCATGAATCGGTGCATTTG
scaffold604568_14.[illegible]	1	p2	(TA)11	22	812	833	TTTCGAGAACTCGGGATGAG
scaffold604572_11.[illegible]	1	p2	(TA)6	12	558	569	TCCTTTTCCATTATCCATGTTTTT
scaffold604576_16.[illegible]	1	p2	(TA)10	20	1128	1147	GGTGCATAGGTTAAAGGGCA
scaffold604580_10.[illegible]	1	p2	(AG)9	18	512	529	TGGATTGCATACCAACCAGA
scaffold604583_13.[illegible]	1	p2	(TC)6	12	613	624	TCTCCTTGGATCTTGATGGC
scaffold604677_13.[illegible]	1	p2	(AC)6	12	2373	2384	TGAAGGCTTTGATTCATCTCTC
scaffold604775_16.[illegible]	1	p2	(AT)7	14	749	762	CTTCCGCCCATTCTACAAAA
scaffold604778_14.[illegible]	1	p2	(AC)6	12	364	375	TGGAATTCGCAATATCCAAA
scaffold604910_15.[illegible]	1	p2	(AG)6	12	659	670	CAATGCTGGGATCGATTTTT
scaffold604993_11.[illegible]	1	p2	(TA)10	20	1356	1375	TGGCTTCATTTTGCAGTCAC
scaffold605049_13.[illegible]	1	p2	(TG)6	12	791	802	GGCGGACATCAAAGGAATTA
scaffold605309_17.[illegible]	1	p2	(TG)6	12	398	409	ATCACGGACCCCACATACAT
scaffold605417_15.[illegible]	1	p2	(CT)7	14	127	140	ATTTGCAATGGACTGCCTCT
scaffold605521_15.[illegible]	1	p2	(AT)6	12	178	189	GGAATGTTATTGTTCGTGCG
scaffold605566_10.[illegible]	1	p2	(AT)9	18	335	352	GGATTGTCCCGTCAAGAGAA
scaffold605578_10.[illegible]	1	p2	(AT)6	12	394	405	TGAGCAAGCGCATTGTTTAT
scaffold605583_18.[illegible]	1	p2	(TC)8	16	410	425	CTTCCCAACACCACCCTAAA
scaffold605696_12.[illegible]	1	p2	(TC)7	14	1451	1464	TCCTTTCTGCATTTGGGTTC
scaffold605702_11.[illegible]	1	p2	(CT)9	18	644	661	GGCTGGTGTGTGTTTTTGAA
scaffold605779_14.[illegible]	1	p2	(GA)7	14	172	185	AACGGGATGCTTTCAAAATG
scaffold605887_11.[illegible]	1	p2	(CA)7	14	1418	1431	TCCATAAACAGGGAAGATGTGA
scaffold605940_13.[illegible]	1	p2	(GA)8	16	898	913	AAAATTCTGAAACCCCCAGG
scaffold605955_17.[illegible]	1	p2	(TC)7	14	129	142	GGCAACGGAATACGATTTGT
scaffold606086_12.[illegible]	1	p2	(GA)6	12	605	616	ATTTGGGGTGATGGGTTTTT
scaffold606093_13.[illegible]	1	p2	(CA)6	12	571	582	GACCGAAATGCAACGATAAA
scaffold606111_12.[illegible]	1	p2	(TA)14	28	128	155	TGCTATGGATGTGGTTGGTG
scaffold606139_12.[illegible]	1	p2	(GA)7	14	410	423	CGAAGTAGCTCGAGCCGTAG
scaffold606208_14.[illegible]	1	p2	(CT)6	12	95	106	CCAAAATTGAACAGCCGATT
scaffold606262_19.[illegible]	1	p2	(TA)8	16	198	213	CAATCTCGAAAATGGATGGC
scaffold606291_11.[illegible]	1	p2	(TA)8	16	372	387	GGGGAAAAGGGTGATGTTTT
scaffold606433_14.[illegible]	1	p2	(GA)6	12	153	164	GCGGGGTTGTTGAAGAATTA
scaffold606469_10.[illegible]	1	p2	(GA)6	12	372	383	GGGGACAACGCCATTTATAG
scaffold606654_15.[illegible]	1	p2	(AT)8	16	2470	2485	TGAGGATATTCCAGTGTGTGAAA
scaffold606655_17.[illegible]	1	p2	(AT)6	12	673	684	TTCAACGTCGGACAAGACAA
scaffold606729_13.[illegible]	1	p2	(TA)7	14	1323	1336	TGAAAGCAGACAGGGGAATC
scaffold606741_10.[illegible]	1	p2	(CT)8	16	151	166	TCCTCTAGCATCTCTTGCTCTC
scaffold606877_12.[illegible]	1	p2	(TA)6	12	180	191	CCGATAGAAGCCCACATGTAA
scaffold607125_18.[illegible]	1	p2	(TA)8	16	684	699	TGCAGTGAGTTGCCCAAATA
scaffold607170_16.[illegible]	1	p2	(TA)8	16	1020	1035	TCCCCGCGTATAGAAATCAC
scaffold607201_16.[illegible]	1	p2	(TA)6	12	2960	2971	CAAGATGATGAAGGTCAAACGA
scaffold607266_13.[illegible]	1	p2	(TA)8	16	47	62	GAGAAGGGTTATCACTTGAATAGGA
scaffold607304_15.[illegible]	1	p2	(TG)7	14	286	299	ATAGGCACCCCTGGAAAAAG
scaffold607366_16.[illegible]	1	p2	(AG)7	14	178	191	GGTGAACCGCGTAAATCTGT
scaffold607414_14.[illegible]	1	p2	(AC)6	12	313	324	TGCGAATCAGTGCTCCATAG
scaffold607568_10.[illegible]	1	p2	(GA)9	18	343	360	GGCACCATACTGCCGATATT
scaffold607580_15.[illegible]	1	p2	(TA)9	18	174	191	CAACGGATAAACCGATTGCT
scaffold607598_15.[illegible]	1	p2	(TG)7	14	822	835	GCGATGGATTGATTCACGTA
scaffold607634_19.[illegible]	1	p2	(TC)7	14	178	191	TTCACCCATTGTTTGCGATA
scaffold607735_15.[illegible]	1	p2	(GT)7	14	229	242	TTTTCCGATTGACAAATCCC
scaffold607765_13.[illegible]	1	p2	(TA)9	18	1464	1481	ACGTCAGCATCCACGTGTTA
scaffold607774_10.[illegible]	1	p2	(AG)6	12	181	192	TTGTGCACGAGAATACATCCA

scaffold607777_14.[illegible]	1	p2	(AG)10	20	471	490	GAGAAGGTGAGAGGCAGACG
scaffold607854_10.[illegible]	1	p2	(AT)11	22	295	316	CATGCCTGATTACATCACCG
scaffold607952_17.[illegible]	1	p2	(GA)9	18	1968	1985	GACAATTGCTCGCAAAGGAT
scaffold608060_12.[illegible]	1	p2	(TA)7	14	1388	1401	TTCTCATTTGCAACCGATGT
scaffold608110_15.[illegible]	1	p2	(CT)7	14	1338	1351	GCTGTTTCTCAGATCCTCGG
scaffold608130_17.[illegible]	1	p2	(TC)7	14	514	527	CATGACCCACCAAACATGAC
scaffold608205_12.[illegible]	1	p2	(TC)7	14	29	42	TCTCTCTTGGTTGGTGAAGAAA
scaffold608255_12.[illegible]	1	p2	(AG)12	24	422	445	TTGTTGGCTATTTGTTGCTCA
scaffold608302_15.[illegible]	1	p2	(AG)13	26	1197	1222	TGGTACTTGCAATGTTGGGA
scaffold608523_18.[illegible]	1	p2	(GA)6	12	316	327	CAAAATTTGGCATCAAATTCAA
scaffold608615_15.[illegible]	1	p2	(GA)6	12	297	308	GGATGGGAGTCAATCCAGAA
scaffold608646_11.[illegible]	1	p2	(GA)6	12	320	331	CAACGAGATACGCTGCAAGA
scaffold608671_13.[illegible]	1	p2	(AT)8	16	51	66	TGTACATCTAGCTCTGTTGAATCTTG
scaffold608672_11.[illegible]	1	p2	(AT)10	20	173	192	TGGAGATGTGAAACATGCCT
scaffold608790_10.[illegible]	1	p2	(TG)8	16	126	141	TCGGTTTGGCCAATATTTCT
scaffold608952_11.[illegible]	1	p2	(AG)7	14	413	426	GATATTTGATTTTGCGGGGA
scaffold608958_13.[illegible]	1	p2	(TC)6	12	1359	1370	TGCTTTGGTGACTGAATGATG
scaffold609042_16.[illegible]	1	p2	(CG)6	12	100	111	ATGGTGGTATGGTATGGGGA
scaffold609074_12.[illegible]	1	p2	(TA)8	16	438	453	CAAATTCGGACTTAAGGGCA
scaffold609126_11.[illegible]	1	p2	(AT)9	18	338	355	AGGGGGTACCATTGTTCAAA
scaffold609371_15.[illegible]	1	p2	(TA)6	12	2056	2067	AGCAAGGACACTGGAACAGG
scaffold609482_12.[illegible]	1	p2	(TC)7	14	1120	1133	TTCGATCTACTGGGCCACAT
scaffold609516_10.[illegible]	1	p2	(AG)7	14	721	734	ATGGACTCGCTGGTCTCTGT
scaffold609568_17.[illegible]	1	p2	(TC)6	12	410	421	GGCAAAATTAAAAATGCCAC
scaffold609571_17.[illegible]	1	p2	(AC)6	12	988	999	TATCTGCTCCAAAAGCCTGC
scaffold609823_13.[illegible]	1	p2	(AT)7	14	240	253	TGCCTACCAAACACTAGGGG
scaffold609837_14.[illegible]	1	p2	(AT)6	12	908	919	CCGTACCGGAATTTAACGAA
scaffold609855_17.[illegible]	1	p2	(AG)7	14	2442	2455	GCCATGTCTAGCATTGGGAT
scaffold609896_14.[illegible]	1	p2	(AT)8	16	1202	1217	CTGCCATAACCCGAAAAAGA
scaffold609899_12.[illegible]	1	p2	(AT)6	12	263	274	TGCCGTGTTATTGTGAGTCC
scaffold609975_14.[illegible]	1	p2	(TC)6	12	2162	2173	TTTGCCCATCTTCAAACTCC
scaffold609985_16.[illegible]	1	p2	(TC)9	18	2733	2750	CATCATTGCCTGTCATTTCG
scaffold609992_16.[illegible]	1	p2	(AG)7	14	2600	2613	TGGAATCGCGATGAGATTTA
scaffold610007_12.[illegible]	1	p2	(TA)9	18	378	395	ACTTGCGAGCCACCAATTAC
scaffold610039_14.[illegible]	1	p2	(CT)6	12	229	240	AGTGTCGGCACAAACAAACA
scaffold610092_15.[illegible]	1	p2	(AG)6	12	463	474	GGTCCTTTCGAAACACGAGA
scaffold610125_16.[illegible]	1	p2	(AC)6	12	586	597	GTGCATTGGGTCAAAGAGGT
scaffold610175_15.[illegible]	1	p2	(AC)6	12	160	171	CCATGTCCTGGCTTACCTGT
scaffold610209_14.[illegible]	1	p2	(GA)6	12	2831	2842	TTTTGATCGAAAGGGAATCG
scaffold610267_17.[illegible]	1	p2	(CT)6	12	759	770	GCACACCATCTTCAAGCAAA
scaffold610390_15.[illegible]	1	p2	(CT)6	12	684	695	GAACGGTTTATTCGGGTTCA
scaffold610506_19.[illegible]	1	p2	(AT)6	12	182	193	CACGTGACATAAAATGCATCG
scaffold610560_10.[illegible]	1	p2	(TA)6	12	76	87	TGAAACCAGGGGAAAAAGG
scaffold610563_16.[illegible]	1	p2	(TC)7	14	210	223	CGGCACAAAATTTTCCACTT
scaffold610664_11.[illegible]	1	p2	(TA)6	12	890	901	TTGCTGCGGTACTTTCATTTT
scaffold610696_12.[illegible]	1	p2	(TA)9	18	387	404	ACCGAAATACCCCCAAACTC
scaffold610729_14.[illegible]	1	p2	(TA)9	18	106	123	TTCACAGTTTTCATTTGATCACC
scaffold610812_13.[illegible]	1	p2	(TG)6	12	129	140	GGCTATAGAGCCGTTGAATG
scaffold610851_19.[illegible]	1	p2	(TC)8	16	177	192	ACGGGACCCTCATCTTCTCT
scaffold610882_17.[illegible]	1	p2	(CT)6	12	523	534	TACAAACCACCTTAACTTCCGC
scaffold610938_16.[illegible]	1	p2	(TG)6	12	1052	1063	CATATATGGTTGCACAGGCG
scaffold610971_16.[illegible]	1	p2	(GT)6	12	130	141	CGAGCCACAATACTTCGGAC
scaffold611003_11.[illegible]	1	p2	(TA)7	14	52	65	CGGATTTAGCGGTTATTACCTGA
scaffold611010_17.[illegible]	1	p2	(GA)7	14	507	520	AACAAGAAGATGCCGAAGGA
scaffold611054_15.[illegible]	1	p2	(TA)11	22	43	64	AGGGAGTGCTCTCACCTGAA
scaffold611113_16.[illegible]	1	p2	(GT)6	12	2216	2227	TTCTGACATACCGTCCTCCC
scaffold611171_10.[illegible]	1	p2	(AT)8	16	326	341	GGGTTCTCGTGATAAGCTCG
scaffold611182_12.[illegible]	1	p2	(TA)9	18	47	64	GAGGGTTATCATGGGATCAAG

scaffold611282_17.4	1	p2	(CT)8	16	274	289	GTGGATCTCGGGTAGGGATT
scaffold611283_13.3	1	p2	(AG)7	14	180	193	GGTTGTGGCTCTGGTTCTGT
scaffold611386_10.5	1	p2	(TA)7	14	53	66	TGTGTGATGTTGGAGTTGGG
scaffold611417_16.6	1	p2	(GA)8	16	2446	2461	ATCGCGAGAAATTGAGAAGG
scaffold611462_18.6	1	p2	(TG)7	14	186	199	TTGATGTGGATGACGAGGTC
scaffold611494_21.8	1	p2	(TC)7	14	121	134	CGTTGATTTGAGGCAGGTTC
scaffold611506_14.4	1	p2	(CT)11	22	1194	1215	GGTGTCCGAGTTGTAGGCAT
scaffold611526_15.0	1	p2	(AC)6	12	444	455	TGCCAGTTTACTCCGAAAGG
scaffold611572_17.1	1	p2	(TC)6	12	68	79	GCTTCAAGTTTCCTCCTTTTG
scaffold611576_14.8	1	p2	(TC)13	26	2358	2383	GTTGGAGAGTTGACGGTGGT
scaffold611588_12.0	1	p2	(AT)8	16	466	481	TTTTCTCGATCTCACGCCTC
scaffold611598_13.1	1	p2	(AG)6	12	181	192	CATGGACCAATGGGACCTAC
scaffold611724_11.6	1	p2	(TA)9	18	422	439	TTTGTTTCTGGTCCAGTCCC
scaffold611943_14.5	1	p2	(CT)6	12	333	344	AACTCCATGCAAATCACACG
scaffold611952_16.7	1	p2	(TA)8	16	281	296	GCGGGTAGGGTAGGGACTAT
scaffold611973_14.0	1	p2	(TG)6	12	182	193	CGCTTCGAGTTGTTTTCACA
scaffold611995_14.3	1	p2	(GA)6	12	254	265	GGAAATGGGAATGGGTTCTT
scaffold612013_15.0	1	p2	(TA)7	14	2458	2471	TGCAAATCTTACAGAATCAGCAAC
scaffold612027_17.8	1	p2	(GA)6	12	1462	1473	CCACTCCCTGAGTATCCCG
scaffold612149_12.9	1	p2	(TA)6	12	603	614	CCATCCCAATTATACGGTCC
scaffold612161_12.6	1	p2	(TA)8	16	338	353	CATGATGTCAATCCAGTGGC
scaffold612194_15.9	1	p2	(TA)9	18	520	537	AGGAGGGGAAATGGATCAAC
scaffold612243_13.8	1	p2	(CT)10	20	1025	1044	CATTTATTCCCCGCAACAAC
scaffold612303_13.8	1	p2	(CT)6	12	106	117	AATCCCTTTCTCCTCTCCCA
scaffold612451_16.0	1	p2	(GA)10	20	2054	2073	CACAGCATGGACTTTGTTGG
scaffold612507_15.9	1	p2	(AG)10	20	2136	2155	CAAAGCAACCCTTAAGAGCG
scaffold612548_17.0	1	p2	(TG)9	18	177	194	GTTTTGAGATGGGTGGTTGG
scaffold612565_11.0	1	p2	(AT)6	12	128	139	CCCCAGGAATCTTGTTTTCA
scaffold612803_18.1	1	p2	(AC)6	12	470	481	TAGCCGGGTCGTGACATAAT
scaffold613039_14.5	1	p2	(CT)7	14	228	241	ACACATGCCTGGAACACAAA
scaffold613074_12.8	1	p2	(TA)11	22	431	452	CCACATGCTATGCCTCAAAC
scaffold613115_12.6	1	p2	(AT)10	20	592	611	TTATCTACAAAACGAGACCAATCTT
scaffold613129_13.4	1	p2	(AT)10	20	1420	1439	TCTATCCGCCAGTGCTCTTT
scaffold613180_20.8	1	p2	(TA)6	12	861	872	TGGCATTACAAACTTCAAACCA
scaffold613299_16.3	1	p2	(AC)6	12	2764	2775	CAACACTGCATCAGTCCCAT
scaffold613513_13.8	1	p2	(CA)7	14	116	129	CTTGAGCTCTGATGCCACAA
scaffold613543_13.7	1	p2	(GT)7	14	277	290	AAGACTTCGCATCTCGGAAA
scaffold613592_15.3	1	p2	(TC)6	12	75	86	GAGTTGGTCATTCTCAAGATGG
scaffold613604_10.8	1	p2	(AT)10	20	280	299	GCGGAACTTTCACTTCGCT
scaffold613675_15.0	1	p2	(AC)6	12	295	306	CTCTCCCTTCCTGAATGCTG
scaffold613857_10.7	1	p2	(CT)6	12	54	65	TTTGGACTAGGCCATTCACC
scaffold613976_12.6	1	p2	(AG)7	14	1896	1909	GGCCCATGTGTTAAATCAAGA
scaffold613984_12.0	1	p2	(TA)9	18	358	375	TTAAATGACCTTGGGATGCG
scaffold614031_12.5	1	p2	(TA)12	24	293	316	AGCGCCTAGCGATTTCTACA
scaffold614054_16.7	1	p2	(TA)10	20	3861	3880	CGCTCACCCATCGAAAGTAT
scaffold614067_15.2	1	p2	(CT)6	12	741	752	TCTCTCGCTCACCCTCTCTC
scaffold614099_13.1	1	p2	(AG)8	16	785	800	TAAGATACGGGCAGCGAAAC
scaffold614225_16.1	1	p2	(TA)8	16	258	273	CTTCCCCTGTTCTTGCATGT
scaffold614277_13.8	1	p2	(TA)8	16	620	635	CGCGGAGCATTGTTCATAC
scaffold614372_16.1	1	p2	(TC)6	12	184	195	ATTTCACCCCCAAGTCTCCT
scaffold614384_10.6	1	p2	(TA)6	12	53	64	TTTTTGTCTATATCGCTCAGTCAAT
scaffold614467_13.9	1	p2	(TA)6	12	170	181	TGGCGAAAAATGTTGACAGA
scaffold614511_10.6	1	p2	(TC)6	12	264	275	GAACCTCTGCACTCTCCAGG
scaffold614541_16.9	1	p2	(TG)6	12	1068	1079	GGAGTCCCTTCTTCCCAGTC
scaffold614549_16.4	1	p2	(TA)6	12	1079	1090	TCTGCAATGAACGAAAATGC
scaffold614562_12.7	1	p2	(AT)9	18	456	473	CATGTGTCCATGAATGAGGG
scaffold614678_10.5	1	p2	(AC)9	18	63	80	CTAAACGGGTCGGGCTAAC
scaffold614820_17.4	1	p2	(AG)6	12	2397	2408	TGAATCCACCATGGAAGAGA

scaffold614829_11.3	1	p2	(TA)6	12	324	335	TCCAAAGGTGGCCAAAATAC
scaffold614962_10.0	1	p2	(TA)8	16	279	294	TAAGGGCGGTATCACCTGAA
scaffold614988_18.7	1	p2	(AT)7	14	911	924	TGGGCATAAAAACGTGCATA
scaffold615030_17.5	1	p2	(CT)9	18	50	67	TCATCAACAGCGAAGCAAGT
scaffold615142_10.8	1	p2	(TA)12	24	290	313	GGCAGCACCAGAATTACTCC
scaffold615163_16.7	1	p2	(GA)7	14	1141	1154	GGCAAATTGTTGAAGTCGGT
scaffold615235_17.6	1	p2	(AG)7	14	182	195	GTAAAACAGCGCCCGTACAT
scaffold615294_10.5	1	p2	(CT)6	12	523	534	GATCGGAGCTCTGTTAAGCG
scaffold615323_12.1	1	p2	(AT)7	14	357	370	AGCGGAAGACTTGTAACGGA
scaffold615334_12.0	1	p2	(TA)6	12	39	50	GGGACCACTAGACATTCGCT
scaffold615340_15.7	1	p2	(AT)6	12	766	777	TCGCTTAAACTTCGAGGGTG
scaffold615374_14.7	1	p2	(CT)7	14	1282	1295	CATTGCGAGAAAAGCAGTCA
scaffold615379_15.1	1	p2	(AG)6	12	896	907	GGATGAGAGATTTGGGTTCG
scaffold615445_15.5	1	p2	(TA)9	18	48	65	TTAGTTAGCATGGGGGAAGG
scaffold615475_11.5	1	p2	(TC)6	12	350	361	CCGCTCAAATCACTCACTCA
scaffold615477_12.8	1	p2	(TA)8	16	48	63	ACCCCACATTTGTGGCATA
scaffold615562_10.1	1	p2	(TA)9	18	329	346	ATCTCAACGCATCAACCGTC
scaffold615703_15.6	1	p2	(CT)6	12	244	255	CTGCCCGAGGTCTGTTTCTA
scaffold615794_13.5	1	p2	(AC)9	18	178	195	CCAACGAAACCACTGACAAA
scaffold615841_15.9	1	p2	(TA)6	12	612	623	TTCGATTCGCAACAATTGAG
scaffold615895_15.5	1	p2	(AG)6	12	1678	1689	AGCACCCTTATTGTGGCTTG
scaffold615926_13.0	1	p2	(TA)8	16	58	73	GATGGGTTATTATTGGAACGAGA
scaffold615929_14.6	1	p2	(AT)7	14	1919	1932	TTGTACGTCCAATATGTAAATTATGTT
scaffold615938_15.1	1	p2	(AT)6	12	2961	2972	TTGCATGTGCATAACGGTTT
scaffold616050_10.2	1	p2	(GA)6	12	248	259	CTTCAATGGAGGTCGGAGAG
scaffold616097_17.2	1	p2	(TA)7	14	183	196	CAGGACGGTATTTTCCCCTT
scaffold616181_17.1	1	p2	(AC)8	16	1107	1122	CCAGAATGTACGAGTTGGGG
scaffold616202_16.2	1	p2	(TA)10	20	177	196	ACCTAAGGGGGTGTTTGCAT
scaffold616209_11.6	1	p2	(TA)6	12	67	78	AGGGTCAACAATGACGGAAC
scaffold616210_14.2	1	p2	(TA)7	14	65	78	AGGGTCAACAATGACGGAAC
scaffold616217_14.9	1	p2	(GA)6	12	1850	1861	TTCTGGTGGAAGGCCTAATG
scaffold616222_12.8	1	p2	(TA)6	12	381	392	GGTGATAGAAGGCTACGGCA
scaffold616223_15.3	1	p2	(CT)6	12	577	588	CTCAAATCCTTCCATTCCCA
scaffold616325_17.1	1	p2	(TG)6	12	1274	1285	TTTTTGCGATGGAACATGAA
scaffold616397_14.0	1	p2	(AT)8	16	213	228	AAAATAATCGGGTTGGGTCC
scaffold616440_10.2	1	p2	(CT)9	18	114	131	TCGACGACTTGTTGGAAATG
scaffold616467_16.6	1	p2	(TC)6	12	658	669	TATTCTTTTACGCATGCCCC
scaffold616479_16.8	1	p2	(TA)9	18	48	65	TCATCTATAGGGAAGTTATCACTGGA
scaffold616483_13.3	1	p2	(TA)9	18	96	113	CAACGGATTTGGTTTGTTCC
scaffold616484_15.1	1	p2	(AT)6	12	185	196	TTCAGAAACGAGGGCATTTT
scaffold616514_16.6	1	p2	(CT)6	12	184	195	TTGGTCATCCCTTTCTGACC
scaffold616516_15.0	1	p2	(GT)6	12	889	900	TTGAATTGATATTCAGTGAACGTG
scaffold616654_20.0	1	p2	(GA)6	12	1463	1474	CCCACAATTCCAGCATCTCT
scaffold616679_10.0	1	p2	(TA)9	18	203	220	TGGGGTGTCATGATAAGTGG
scaffold616817_13.9	1	p2	(AT)11	22	40	61	CGGATTTAAGGGTTATTACCTGATT
scaffold616830_16.5	1	p2	(TC)6	12	185	196	CCCAATCCCGTAGGAATTTT
scaffold616965_15.5	1	p2	(TA)8	16	293	308	AAATTACACCTTTGTCATTTTTCAG
scaffold616966_13.6	1	p2	(TA)9	18	805	822	TCGTTAAAGGGAATGCACAA
scaffold616993_18.6	1	p2	(AG)6	12	547	558	GGAGTGGGGGTTTTGAGTTT
scaffold617016_15.4	1	p2	(AT)6	12	643	654	TTGCAATCCGATATCAACCA
scaffold617133_14.7	1	p2	(CT)6	12	4366	4377	AACTTGGTTCCACGCAAAAC
scaffold617134_14.5	1	p2	(TA)6	12	375	386	TTGAAGCATGACCTGCAAAT
scaffold617135_16.7	1	p2	(TA)8	16	1053	1068	ATGCCGTTCACCTTCAACTC
scaffold617185_13.9	1	p2	(TA)6	12	130	141	GGGATAAGATTGGTGGTGATG
scaffold617208_11.1	1	p2	(TG)6	12	392	403	AATGCCTGCTTGCCTTAAAA
scaffold617275_16.2	1	p2	(AT)7	14	299	312	TTTTTCTCCCAAAATTGCC
scaffold617345_16.6	1	p2	(GA)9	18	473	490	CTGGATGAAGTTATGCGCTG
scaffold617346_15.6	1	p2	(CT)7	14	392	405	CACATCACCCCTTCGTTTTT

scaffold617463_10.(	1	p2	(AC)6	12	118	129	AGCTGGGCACTTGAAACCTA
scaffold617473_16.1	1	p2	(AG)8	16	180	195	CTCTTTCCAGTTGTGTCCTGG
scaffold617543_12.4	1	p2	(AG)7	14	165	178	GGCATACCGCAAATCAACTT
scaffold617739_10.1	1	p2	(TC)9	18	610	627	CTTCCTCCCACCTTCGTCTT
scaffold617830_14.5	1	p2	(GA)7	14	649	662	GCTGCAGCAGTATCAATCCA
scaffold617837_17.7	1	p2	(TA)6	12	985	996	CCGCTACACCTTTTGGATGT
scaffold617861_12.1	1	p2	(CT)8	16	82	97	ATCTTCCCGCCATTCTTTCT
scaffold618062_11.9	1	p2	(AT)12	24	298	321	CCATTTCCCTATCTATACCAATCAA
scaffold618076_13.4	1	p2	(GA)7	14	403	416	AAGGAGAGGAAGGTTGGCAT
scaffold618099_15.6	1	p2	(TC)7	14	602	615	AGAGCGAGTTGGAACTTGGA
scaffold618134_17.1	1	p2	(GT)7	14	1186	1199	CGTTCGCTATCCAAGTCAAT
scaffold618300_15.9	1	p2	(AT)11	22	580	601	TATAACATCAACCGCCAGCA
scaffold618374_16.4	1	p2	(AT)7	14	754	767	AATTCCCAAGATAATCTCAATCG
scaffold618449_14.3	1	p2	(AG)8	16	1647	1662	GCTCCCGTAGAACTTTCCCT
scaffold618490_12.4	1	p2	(TA)6	12	285	296	TGAGGAGCGTTGATCTTGTG
scaffold618502_10.3	1	p2	(TC)6	12	342	353	TGCAACAAGATGGTGCTCTC
scaffold618541_10.1	1	p2	(TA)9	18	67	84	AAGGCCGAGATTTGTTAGAGG
scaffold618562_10.3	1	p2	(TA)6	12	92	103	TGAAGTTCGGAAATAAGGAGG
scaffold618568_16.2	1	p2	(TA)6	12	285	296	TCTTATCCATTTCGCCATCC
scaffold618714_15.8	1	p2	(TC)10	20	177	196	TGGAGGGCTGTTTCTTCATC
scaffold618726_13.1	1	p2	(AT)7	14	289	302	GGTGGGAAATCATCCCTTCT
scaffold618865_18.(	1	p2	(TC)6	12	235	246	GACCAAGTTGCTCTTCTCGG
scaffold618867_12.5	1	p2	(CA)6	12	349	360	CACTCCCCTCCCTTTTCTTC
scaffold618917_15.3	1	p2	(AG)6	12	115	126	TGAGCCGTCAGTGCATTAAA
scaffold618933_13.4	1	p2	(TA)6	12	2053	2064	ACATAAATTTGGCAAAGAGAAAA
scaffold618956_10.(	1	p2	(TA)10	20	118	137	CCGGGAATTTTGAATGAACA
scaffold619031_15.3	1	p2	(GA)6	12	756	767	ACCCGTCTAGCAGAGACGAA
scaffold619067_14.6	1	p2	(CT)6	12	82	93	TTGCCCCTTCACTCTCATCT
scaffold619130_12.1	1	p2	(TG)7	14	206	219	TGACGTGGGTGTTGGAATAA
scaffold619131_12.2	1	p2	(TG)7	14	566	579	CGAGATTTGCTCATGTTGGA
scaffold619154_14.1	1	p2	(AG)6	12	186	197	AAACAGCGCCCATAATCTTG
scaffold619162_17.(	1	p2	(TG)8	16	498	513	CAAGCCATCTGTGGTTGAGA
scaffold619428_16.8	1	p2	(GA)13	26	296	321	TGGTTGAAGGAGGCTTAGGA
scaffold619479_14.4	1	p2	(AG)9	18	1661	1678	TGATTTCATCCGAATGTCCA
scaffold619490_11.5	1	p2	(TA)11	22	869	890	TGTGGGTTCACTGGATACGA
scaffold619515_10.6	1	p2	(AT)7	14	446	459	GGACAAATTTGGAATTTTAGGG
scaffold619537_11.4	1	p2	(AG)6	12	260	271	ATCGATCCACGTTCGAAAAC
scaffold619688_15.1	1	p2	(CT)7	14	668	681	TAATGTCTTCCCATCCAGCC
scaffold619793_12.2	1	p2	(AT)9	18	185	202	TGGCCGGTTTTGAATCTATC
scaffold619874_16.1	1	p2	(TA)7	14	1703	1716	GGGTTTCATTTTGATTCCCA
scaffold619876_16.9	1	p2	(CT)14	28	370	397	CTTTCTTGCGAGAACCCTTG
scaffold619925_14.(	1	p2	(AT)7	14	297	310	TTTTTGACCAAATCAATCCCA
scaffold620078_14.3	1	p2	(GA)6	12	1831	1842	CTCGTCCTATGCAAGCAACA
scaffold620096_15.3	1	p2	(CT)6	12	164	175	GGATTCAGCATTGTAAGGGG
scaffold620098_16.(	1	p2	(AT)6	12	399	410	TGAGAAAGCAGCTGGTGAGA
scaffold620187_11.1	1	p2	(TA)6	12	115	126	AAAAGAGTTGGGAAAAGTTATGAGT(
scaffold620244_14.7	1	p2	(GC)6	12	140	151	CGCTATGAGTCACGGCTACA
scaffold620289_10.(	1	p2	(AT)7	14	214	227	CCATAACCGTCGCATAACTC
scaffold620540_13.(	1	p2	(CT)9	18	1528	1545	TTCAATTACAAGCCGCAGTG
scaffold620561_15.7	1	p2	(AT)7	14	67	80	TTTTTGCTAGAGGATTTTGTTAATATG
scaffold620656_14.(	1	p2	(GA)6	12	754	765	CGAAAGTTTTGGAATTTGAAGG
scaffold620665_10.1	1	p2	(AC)7	14	119	132	GCACCATCTTCTCAACACCA
scaffold620809_15.8	1	p2	(AG)6	12	131	142	GAAAAGGCACTCGAATTTCC
scaffold620813_15.6	1	p2	(TA)8	16	3485	3500	TTTTACGCTAACGAAACGGG
scaffold620885_16.1	1	p2	(TC)7	14	1278	1291	GGCTTAGGTGAGGTGGAGTG
scaffold620904_14.4	1	p2	(AT)8	16	960	975	CAATAGAGCAGGAAGAGCCG
scaffold620983_16.7	1	p2	(TA)7	14	68	81	CCACACTTTTAAATATTTTAGCTGGC
scaffold620992_13.7	1	p2	(AG)6	12	134	145	GCTAGAGCTCACTTCACGAGC

scaffold621049_16.8	1	p2	(TA)6	12	1665	1676 TTATTCCCCAAGCAGGAATG
scaffold621101_12.5	1	p2	(AG)7	14	279	292 TTTAGAATGCCAATGACGCA
scaffold621114_12.5	1	p2	(AT)8	16	1189	1204 TATCCGATCCGAAGTATCCG
scaffold621155_11.4	1	p2	(TA)6	12	200	211 AGATGCCAATCTTCCATTGT
scaffold621173_10.8	1	p2	(TC)8	16	995	1010 AACCCCAGCAACATCAACTC
scaffold621178_15.2	1	p2	(TA)10	20	591	610 ACTTGAAAATGCGATGAGCA
scaffold621342_13.0	1	p2	(GA)7	14	437	450 TGAGGGGTGAAGACTTTTGG
scaffold621443_15.2	1	p2	(TG)8	16	2489	2504 CCCTTGGTCCTTGAGCATTA
scaffold621451_10.6	1	p2	(AT)7	14	88	101 TTCTACTGGAACTGACGCCC
scaffold621454_12.7	1	p2	(TA)9	18	48	65 CACCCAAAAAGGAGTTATTATTTGA
scaffold621473_15.9	1	p2	(TA)7	14	1639	1652 GAGGAGAGAAGAGCGGACCT
scaffold621481_14.0	1	p2	(CT)6	12	1950	1961 TTTTGCTTTGTTTTTGCTGG
scaffold621542_17.2	1	p2	(AT)8	16	184	199 AAAATCAGCGAGGCAGAAAA
scaffold621594_16.4	1	p2	(AT)6	12	784	795 GGTTGTTTAAATGCCCCCTT
scaffold621684_15.5	1	p2	(TG)7	14	1543	1556 GCAAATTCTGTGTGGGTGTG
scaffold621819_17.3	1	p2	(TA)6	12	3239	3250 GAATGACACTCGACGAAGCA
scaffold621872_13.6	1	p2	(AG)8	16	1495	1510 AGAGAGGGGAGGAGATTGGA
scaffold621898_17.7	1	p2	(TC)6	12	36	47 TCTCTCCCTTTCCACACTCG
scaffold621903_12.7	1	p2	(GA)7	14	454	467 TGGACCATTCGACGGATAAT
scaffold621904_10.3	1	p2	(GA)6	12	464	475 GAAAGCCGTCGATGTAGCTT
scaffold621941_10.5	1	p2	(AT)6	12	135	146 GGATGAAATGGCACCCAATA
scaffold621958_10.2	1	p2	(AT)8	16	90	105 CTCTCAATGCCTTCTACCCAA
scaffold621969_10.0	1	p2	(AT)6	12	146	157 TGGCCCACCCATTTTTACTA
scaffold622355_13.7	1	p2	(TA)8	16	79	94 TGATTGCACCATAATGCACC
scaffold622418_11.4	1	p2	(AG)6	12	135	146 CCAGTTATCCATCCATTCCA
scaffold622445_13.8	1	p2	(TA)8	16	830	845 TTCAGATCCACTAGACATTTGAGC
scaffold622473_11.8	1	p2	(TA)6	12	187	198 CTGCGCAGATCTAATGGTCA
scaffold622576_12.2	1	p2	(CT)6	12	65	76 TCCCTCCAATCAATTTTCCA
scaffold622593_18.5	1	p2	(AG)8	16	363	378 TTGGGAGGAGGAGATTGTTG
scaffold622612_10.9	1	p2	(TA)8	16	1093	1108 CGAAACAAGTGGCTGCATAA
scaffold622627_14.2	1	p2	(AT)8	16	746	761 TTATGGGCCAATTATGCTCC
scaffold622748_13.7	1	p2	(AC)6	12	43	54 GGAGAGTTCGCGAAATAACAT
scaffold622754_15.9	1	p2	(TC)7	14	1093	1106 TGGACTGCCTTCATCTTCCT
scaffold622771_15.8	1	p2	(AT)8	16	183	198 CGTGCAGCAAGTAAACGAAA
scaffold622782_14.7	1	p2	(TA)6	12	4780	4791 ATTAATGCATCGATGGAGGG
scaffold622906_13.8	1	p2	(AT)8	16	279	294 CTTGCTGGCAACAAAAACAA
scaffold622952_18.4	1	p2	(AG)9	18	1176	1193 AGCTGTTGCAGAAGCCATTT
scaffold623107_17.7	1	p2	(TC)6	12	188	199 AACAAAGCTCTCTTCTGGCG
scaffold623118_15.3	1	p2	(GA)6	12	1420	1431 AGGGGATTGTAACATGTGGG
scaffold623147_16.6	1	p2	(TC)8	16	184	199 CTGCAACACCATTGCAGAGT
scaffold623210_12.4	1	p2	(TA)7	14	981	994 GGGTGTTGAGGATGCACATA
scaffold623236_15.9	1	p2	(GA)6	12	189	200 ACCCTTCACCCCCATAACTC
scaffold623271_15.3	1	p2	(GA)9	18	2559	2576 GGCTGGCTTTCACTTCTCAT
scaffold623324_16.3	1	p2	(AT)6	12	252	263 GAGAATTTTAATTCGTTCATCTTGC
scaffold623354_18.7	1	p2	(CG)6	12	1405	1416 TGGTGGTATGGTGTGGAGAA
scaffold623376_11.0	1	p2	(TA)6	12	245	256 GTAGGGGTGTACATGGTCCG
scaffold623431_11.9	1	p2	(GA)6	12	291	302 TGCCGTGGTTCGATATACCT
scaffold623438_18.1	1	p2	(AC)7	14	1149	1162 ACAAATGCGTGGGCTTATTC
scaffold623484_17.0	1	p2	(TA)6	12	628	639 CATCCAAAACTCGAACCCAT
scaffold623582_14.3	1	p2	(AG)6	12	920	931 CCCGTGCTTCAATATCCTGT
scaffold623651_15.3	1	p2	(AT)6	12	139	150 AATGAGTTTCCAGCGCAAAG
scaffold623689_10.5	1	p2	(AT)8	16	101	116 ATGATTGTGCTCAATGGCAG
scaffold623692_13.6	1	p2	(AC)6	12	788	799 CACTCCACGAATGGTCAACA
scaffold623798_15.5	1	p2	(TG)7	14	4567	4580 TGTTCAACTAAATAATGCAGCG
scaffold623813_12.5	1	p2	(TA)9	18	259	276 TTCGTCCGAAATACTCCCAC
scaffold623895_12.1	1	p2	(CT)6	12	135	146 TGCATGATCAATGTTGTAAGGA
scaffold623992_13.1	1	p2	(TA)11	22	44	65 GGGGTTATTAGTGGAGTAAGCC
scaffold624028_15.7	1	p2	(GA)9	18	990	1007 AGGTGGTTTCTTGCCTGAAG

scaffold624046_11.(	1	p2	(AT)7	14	105	118	TCGGGAGGAAGAAGAAGAAA
scaffold624130_18.!	1	p2	(CT)7	14	309	322	GGCCACTTTGTTGTGCTGTA
scaffold624179_10.(	1	p2	(AT)8	16	1433	1448	TGACTTGAGGATTGTGTGCC
scaffold624231_20.(	1	p2	(AT)6	12	398	409	GCAGGATCGCCATAAGGATA
scaffold624521_11.7	1	p2	(TA)6	12	51	62	TTTAGTGTTTTGACATGATTACTCG
scaffold624546_14.4	1	p2	(AC)10	20	205	224	CTCGGAGGCTCCAACATCTA
scaffold624576_16.7	1	p2	(CT)6	12	391	402	GCACCAGTTCGGTGACATA
scaffold624593_13.!	1	p2	(TC)9	18	499	516	TTGCTCCTTGCAACTTGATG
scaffold624763_11.!	1	p2	(CG)6	12	67	78	TATGGTGGTATGGTGTGGGG
scaffold624769_15.7	1	p2	(CA)7	14	186	199	TATCAGGCCTGTGCAGTCAC
scaffold624789_10.8	1	p2	(AT)9	18	303	320	TGCTTTTTGACTTGCTCGAA
scaffold624845_11.9	1	p2	(TA)8	16	103	118	GGCATCGTAAGGAACGTTGA
scaffold624860_17.(	1	p2	(TA)7	14	1493	1506	TGGCGGTGGAAATAGTAAGG
scaffold624902_10.7	1	p2	(GA)7	14	373	386	TCACATGCTGAATGACCAACT
scaffold624988_14.8	1	p2	(AT)9	18	46	63	ATCGGTTATTACCGGATCGC
scaffold625007_12.(	1	p2	(AT)8	16	1194	1209	GGCGAGGACTGAAATTGGTA
scaffold625011_15.1	1	p2	(TC)7	14	873	886	TTTTGCCCCTTCTTTCCTTT
scaffold625063_11.(	1	p2	(TA)9	18	47	64	ATCGGTTATTACCGGATCGC
scaffold625214_15.2	1	p2	(AG)7	14	1029	1042	CGTATCCATGGTTGAGGTCC
scaffold625237_15.7	1	p2	(CT)9	18	184	201	TACACTGAGAGCGACATCGG
scaffold625307_15.4	1	p2	(AT)6	12	584	595	GGTCCTGTATGCTGTGGGTT
scaffold625315_13.(	1	p2	(AC)7	14	55	68	ACACAGATCTTTCACTGCCG
scaffold625353_18.8	1	p2	(TC)6	12	376	387	TTCTGATAAGCCAGGGGATG
scaffold625381_13.(	1	p2	(TA)6	12	183	194	AAAGCAAAAGAGGCGATTGA
scaffold625424_16.8	1	p2	(AT)6	12	676	687	GGAGATCACTTGCGGAGAAG
scaffold625541_12.9	1	p2	(AT)8	16	485	500	AAACTGTAATTCAGGCACCTACG
scaffold625621_12.7	1	p2	(AG)6	12	268	279	TTTTCGATGAACCACCACTTC
scaffold625655_17.!	1	p2	(TA)6	12	1407	1418	TCAACCTCACAATTTGCTCG
scaffold625681_17.3	1	p2	(CT)8	16	186	201	CACAAACGCCACCATTTTTA
scaffold625731_14.!	1	p2	(AT)7	14	783	796	TGGTGCTCTTGTTTCGTCAC
scaffold625922_11.(	1	p2	(GA)7	14	146	159	TGTGGCAGATCTGAAAAACG
scaffold625937_11.9	1	p2	(AG)6	12	250	261	GCATTTTGCTACGTGGTTCA
scaffold625943_14.1	1	p2	(GA)8	16	2324	2339	TGCACACAGCAGTTGTCAAA
scaffold626002_16.(	1	p2	(AT)7	14	754	767	TGATCATGCTCAACATGCAA
scaffold626022_10.(	1	p2	(AT)6	12	324	335	ATTCGGGTAAAAATTGACGG
scaffold626050_13.!	1	p2	(CT)6	12	584	595	CGCTAAAACTGGAGTCAGCA
scaffold626097_14.7	1	p2	(AG)6	12	2899	2910	ATCAGCCATGTTTGATCAGC
scaffold626277_16.3	1	p2	(CT)7	14	187	200	ATTTCATCCCTTGTCATGCT
scaffold626305_11.(	1	p2	(TA)7	14	247	260	CTGTTTTCTCAAGGCTCCCA
scaffold626362_14.2	1	p2	(CA)10	20	1461	1480	GAAAGGCAAAGATGGGTTCA
scaffold626395_13.(	1	p2	(AG)7	14	131	144	CTCTCTCTCAATTGCCACCC
scaffold626399_14.8	1	p2	(AT)6	12	100	111	TGGTGTTTATGCTACTTTGAACG
scaffold626406_14.9	1	p2	(AT)8	16	748	763	TGGAACACGTACCTTCTGATTC
scaffold626426_12.7	1	p2	(AG)7	14	270	283	GATTCTTCGGCTCTTGTGCT
scaffold626565_15.7	1	p2	(CA)7	14	188	201	TTTGTACCTGAATGCACCCTT
scaffold626640_17.!	1	p2	(CT)8	16	94	109	ACGTTGGACCTGAAAGAGGA
scaffold626683_14.!	1	p2	(TC)8	16	871	886	GCCTGAAAATGAACACCGTA
scaffold626778_15.4	1	p2	(TA)6	12	113	124	GACATGGAGTTGTGGTGTCG
scaffold626811_13.8	1	p2	(AT)10	20	58	77	CTTTTCCGGTCAAAACCAAA
scaffold626844_12.7	1	p2	(TA)7	14	475	488	TCGAAAATTTAAAGGCCCAA
scaffold626927_15.1	1	p2	(TA)9	18	185	202	GGCTCCATGACCGATAAAAA
scaffold627081_14.8	1	p2	(AT)7	14	138	151	GGCAACGACTATCGATCCAC
scaffold627084_16.4	1	p2	(CT)7	14	252	265	ATCCAAACACTTCTCACGGG
scaffold627186_18.!	1	p2	(AT)9	18	185	202	TTGCAACCGAAGTTTCATTTC
scaffold627418_12.3	1	p2	(AG)7	14	367	380	CTCACCGTCGATCAAATCCT
scaffold627438_12.8	1	p2	(TC)8	16	51	66	CATGCAAGACCCGAAGAACT
scaffold627490_15.!	1	p2	(AT)11	22	774	795	CACACATTTCCTTCCTACCAAA
scaffold627496_16.!	1	p2	(AG)6	12	441	452	TTGGATATCGAGATCGAGGG

scaffold627538_10.3	1	p2	(AG)6	12	155	166	TTATGCGATCTGCACGTGAT
scaffold627594_14.0	1	p2	(AG)6	12	239	250	GCATGTGTGTGCTGAGTGTG
scaffold627629_15.8	1	p2	(CT)7	14	2561	2574	TGTATTCATGTGTCAGCGCA
scaffold627743_14.7	1	p2	(CT)9	18	6412	6429	GTGCACTCCTGGAGGTCAAT
scaffold627751_16.6	1	p2	(TC)9	18	1405	1422	GCTCAGCTACAAAACCGGAC
scaffold627796_18.6	1	p2	(TA)7	14	250	263	CCCTCGAATGCACTCAAACT
scaffold627899_10.5	1	p2	(TA)6	12	142	153	TTTCAATCCGGGTCTCTCAC
scaffold627916_12.8	1	p2	(TA)7	14	344	357	TTAATAAATTGTCGCGAACGG
scaffold627950_14.0	1	p2	(AT)6	12	79	90	AAATACTCAGTAAACCAGATGATGAA
scaffold627988_12.0	1	p2	(TA)6	12	69	80	AAGGGGAGTATCACTTGAATGC
scaffold628001_16.4	1	p2	(CA)7	14	43	56	CTAACCTTGGGCTCTGATGC
scaffold628013_10.3	1	p2	(AT)9	18	51	68	CACACTCAATACAATATGAACGACC
scaffold628023_15.0	1	p2	(AT)8	16	263	278	TTTTCGCCTCCGACTAAAAA
scaffold628121_10.4	1	p2	(AT)9	18	66	83	GCATCAACGGCGTGTATTATT
scaffold628174_16.9	1	p2	(TA)6	12	1971	1982	GCATGCAATAGGTTGTGCAG
scaffold628195_11.9	1	p2	(AC)6	12	258	269	GCATATGCAAGCTCAAATTCA
scaffold628210_11.1	1	p2	(AT)9	18	223	240	GATGAGTTCCTGAACCAGCAG
scaffold628230_13.7	1	p2	(TG)8	16	66	81	TGTCGCACAGAATGGTTGA
scaffold628276_12.0	1	p2	(GA)7	14	669	682	GTTCGGTGGTCATCTCACCT
scaffold628286_14.4	1	p2	(TC)6	12	3657	3668	TAACGGGCAGCCTAAGTACG
scaffold628383_15.3	1	p2	(AG)8	16	805	820	AATTTCCCGCATCACACTTC
scaffold628454_13.6	1	p2	(TA)7	14	331	344	CGTTCACGTTCTGCGATTTA
scaffold628509_14.2	1	p2	(CT)7	14	189	202	CACTTTTGGGACCCCTTTTT
scaffold628595_12.8	1	p2	(CT)6	12	283	294	GCAGGTGTGAATGGAGGAAT
scaffold628622_15.9	1	p2	(TA)6	12	166	177	TGTTGCATACGATGTGTAGCAG
scaffold628645_12.0	1	p2	(AT)11	22	2434	2455	TATGGGCCAACTAAAGCGAC
scaffold628726_13.6	1	p2	(AT)6	12	123	134	GGCGTGACATAGTGGATTGTT
scaffold628742_10.4	1	p2	(AC)6	12	324	335	ATAAGTTCGGCGGACAACTG
scaffold628828_16.9	1	p2	(TC)6	12	642	653	GGGCTTATTCCGTCATTTCA
scaffold628903_13.9	1	p2	(GA)7	14	210	223	AAAAATTCGATCTTGGGGTG
scaffold628989_16.8	1	p2	(AG)6	12	422	433	GGTGCTGTAAGGGTTGGAGA
scaffold629007_16.1	1	p2	(TA)6	12	319	330	GCTAGTGTGTTTGCTTGCAACT
scaffold629027_14.4	1	p2	(CT)9	18	587	604	TTTATTTCTTTCCCGCCTCC
scaffold629032_11.5	1	p2	(TA)7	14	621	634	AAAAATGAACTTTATCGGATCTATCA
scaffold629056_12.6	1	p2	(GA)6	12	130	141	CTCGTCTCGGGATCTTTGAG
scaffold629097_13.3	1	p2	(GT)7	14	455	468	GTGTGTGCGTCTGAGTTCGT
scaffold629147_17.8	1	p2	(CT)8	16	187	202	AAAGCCCATGTTTTGTCTGG
scaffold629165_15.9	1	p2	(TC)8	16	91	106	CCCAACTGAAAACTTCCCCT
scaffold629228_13.4	1	p2	(CT)7	14	241	254	CAATCAATCCATCGTCTCCC
scaffold629246_13.7	1	p2	(GT)6	12	124	135	GCACGTGACATCTAACGGAA
scaffold629268_16.8	1	p2	(AG)11	22	181	202	GTCACTAACCCCACCACACC
scaffold629287_10.7	1	p2	(AT)6	12	26	37	TCCGTCAAGGAGATCTTGAAA
scaffold629468_14.7	1	p2	(AT)6	12	460	471	GGGTGGCTTGATTTTGTTCA
scaffold629604_15.0	1	p2	(TG)10	20	49	68	CGGATCGCGACCCTATACAT
scaffold629624_15.2	1	p2	(CT)10	20	273	292	TTAAAGCCTGCCCTTTTTGA
scaffold629652_14.0	1	p2	(AT)6	12	507	518	TGGTAAGTGATAGGTAATGTCAATCC
scaffold629674_12.6	1	p2	(CT)6	12	547	558	CCTATCTTTGCCATACCCGA
scaffold629718_15.5	1	p2	(GA)6	12	192	203	CATGCCCCTAGGGTTTTACA
scaffold629719_18.0	1	p2	(GA)7	14	190	203	GGAGGAAGAAGATTGTGCCA
scaffold629745_16.7	1	p2	(AT)8	16	1047	1062	TGCCTACATCATTTACCCCA
scaffold629831_13.4	1	p2	(AT)8	16	995	1010	GCCAAATCCTAGCCACACAT
scaffold629832_15.5	1	p2	(AC)6	12	1384	1395	TTTGGTGACATGACCACGAC
scaffold629840_17.6	1	p2	(CA)7	14	389	402	CTCACGTTTTTAGCCCAAGC
scaffold629918_16.4	1	p2	(TG)7	14	899	912	TCTTTTATGTGGGGGTGACAG
scaffold630015_13.8	1	p2	(AT)12	24	647	670	TCTCCTTTGCGTGGACAAGT
scaffold630038_14.0	1	p2	(CT)11	22	2022	2043	TGGGGTGTGTGTTCTTGAAA
scaffold630176_15.0	1	p2	(TA)11	22	1945	1966	AGCAAAGAAATGCAGAAGGC
scaffold630205_14.3	1	p2	(TA)12	24	719	742	TGGTTCTCACTAAAACGCGA

scaffold630212_16.5	1	p2	(TA)6	12	1123	1134	TCGTTCTTTCTTGCTTCCGT
scaffold630302_14.5	1	p2	(TA)8	16	674	689	GGGTTTATGTTTGGTGTTTGC
scaffold630303_11.7	1	p2	(TA)6	12	312	323	AGCTCCATCGAGTCAAGCTC
scaffold630342_14.8	1	p2	(GA)6	12	622	633	GTGGGAAGATTTGAGGGGTT
scaffold630522_16.2	1	p2	(TA)7	14	190	203	ACATCCACTGGCCAAGAATC
scaffold630524_14.7	1	p2	(AT)7	14	191	204	CGAAAAACCAAAAACCCTCA
scaffold630545_14.8	1	p2	(AG)7	14	54	67	GGCCAGTAACACAAAGGCAT
scaffold630580_11.3	1	p2	(TC)10	20	896	915	ATTGTCCTGACGACTCCACC
scaffold630603_17.0	1	p2	(GA)7	14	54	67	TGAGAGATCAGTGAGCACGAG
scaffold630717_12.0	1	p2	(CT)8	16	52	67	AAGTAACCACCAATAACCGCC
scaffold630718_12.4	1	p2	(TA)9	18	757	774	TGTTGATCTTATGAGTGTGAAAACA
scaffold630842_19.3	1	p2	(GA)6	12	1845	1856	TGTTCGAAAATGGTGGTTGA
scaffold630856_18.5	1	p2	(AG)7	14	120	133	CTGATAACAATGGGCGATCA
scaffold630930_16.4	1	p2	(AT)9	18	470	487	CTGGAGAATTGGGAGGCATA
scaffold630957_11.8	1	p2	(TC)7	14	251	264	TGGGAATTTTGTGGAGAAGC
scaffold630999_13.1	1	p2	(TA)6	12	1265	1276	CATCTGAACAGTGTCAATTTTGC
scaffold631052_17.7	1	p2	(TA)6	12	600	611	CGAACCAAAGCAACACAAGA
scaffold631090_15.0	1	p2	(AT)6	12	301	312	GCCCTCAACTTTGTACGTGAA
scaffold631137_16.5	1	p2	(AG)7	14	191	204	TTGATTCGGAGGCTTGTTTT
scaffold631138_15.8	1	p2	(CT)9	18	2037	2054	AAAGAACTCGTCGCCAGTGT
scaffold631153_14.0	1	p2	(CT)6	12	240	251	CTCACCCCTCGAGTTTCGTA
scaffold631282_16.2	1	p2	(AG)10	20	281	300	GCAAATGCATCAGATTCAGG
scaffold631500_13.3	1	p2	(AC)6	12	2198	2209	TAAAGTGAAACGCAGATGCC
scaffold631510_14.6	1	p2	(AG)6	12	677	688	GAATTGGTCTTCCTTTGCCA
scaffold631732_15.4	1	p2	(AT)6	12	1803	1814	AGGGCAAACTCACCATTGTC
scaffold631774_13.3	1	p2	(CT)6	12	87	98	TCACCACGTTCTACGCTCAA
scaffold631778_12.6	1	p2	(TA)8	16	49	64	CTCACAGGATTTAGCTGTTCTCA
scaffold631815_10.5	1	p2	(CT)6	12	333	344	CCTCTCATGTTTTCCCCAAA
scaffold631818_18.1	1	p2	(CT)7	14	333	346	TTCTTTCACCCCTCCCATAA
scaffold631832_12.6	1	p2	(AT)11	22	1674	1695	CTCCCAAAATCTCAATCCCA
scaffold631855_16.3	1	p2	(TC)6	12	456	467	CCCGTGACATGGAAACTCTT
scaffold631932_17.2	1	p2	(CA)6	12	322	333	AGTAGCAGCTGTGGATTCCC
scaffold632014_18.0	1	p2	(TC)7	14	1887	1900	ATTTTTGTTTTGCTGCCCAC
scaffold632030_10.0	1	p2	(TA)6	12	429	440	AAGAAAAACAGGGGAAAGGG
scaffold632071_10.2	1	p2	(TA)7	14	94	107	GGGGGAGGAATGGTAGAGAG
scaffold632124_14.0	1	p2	(AG)6	12	415	426	ACTATTGTCGGACACTGGGA
scaffold632245_12.3	1	p2	(AT)8	16	590	605	TCGCCTTTAATTCTAGCAACA
scaffold632287_15.9	1	p2	(AG)9	18	4321	4338	TCGAGAACTTGCTTGGTGAC
scaffold632471_13.2	1	p2	(AG)7	14	650	663	TTTGATCGGTTGACGAATGA
scaffold632518_13.6	1	p2	(TA)6	12	765	776	GCAAACATATGGTGGCATTT
scaffold632520_10.1	1	p2	(TA)6	12	642	653	TCAACAATTGGAGACAACGC
scaffold632632_16.3	1	p2	(GT)7	14	2126	2139	CCCCCAAATTCTCTCCTCTC
scaffold632734_12.1	1	p2	(AT)6	12	98	109	CCAACACAAAAACAGGGAAAA
scaffold632824_18.0	1	p2	(TA)7	14	192	205	CCTTGTGGGGTACAAGTGCT
scaffold632825_14.9	1	p2	(AT)8	16	805	820	AACTTCAAGCACTGGAATGAGA
scaffold632844_10.7	1	p2	(AG)8	16	189	204	CTTCCCCAGAAATCGAACAA
scaffold632854_11.6	1	p2	(GA)8	16	321	336	CATGCGAGTAGAGCTCGAAA
scaffold632956_17.1	1	p2	(CT)7	14	562	575	GAACAAGGGCAACTCGAAAG
scaffold632982_12.5	1	p2	(TA)8	16	389	404	ATTTGTGTGGGCATGTTCAA
scaffold633054_14.7	1	p2	(CT)7	14	475	488	TTTTGGAGCATCTCCCATTT
scaffold633168_16.4	1	p2	(CT)7	14	192	205	TTCCGAAGCTCTTCAAGCTC
scaffold633195_17.7	1	p2	(AG)8	16	1788	1803	CCTTCCCAACAGAGGAAAGA
scaffold633227_14.3	1	p2	(GC)6	12	269	280	TACCCTAAGGGCATCCACAA
scaffold633239_13.4	1	p2	(TC)6	12	877	888	GGCCACTAAAACAGGGATCA
scaffold633263_13.8	1	p2	(AG)6	12	400	411	GGGTTTACCTATCCCGTGCT
scaffold633265_15.5	1	p2	(CT)6	12	2809	2820	CCTTTGCATAGAGTGCGACA
scaffold633412_17.9	1	p2	(CT)7	14	1976	1989	ACTTGTCGAAGGAGACGCAT
scaffold633416_14.7	1	p2	(TA)6	12	1506	1517	TTTGGCTCGTATGCAGTCAG

scaffold633455_13.9	1	p2	(AT)6	12	267	278	AAAAATGAACATGGCCGAAG
scaffold633490_13.7	1	p2	(AT)6	12	431	442	TCGTCACCCTTATCCTACCG
scaffold633514_13.2	1	p2	(AG)6	12	103	114	TTGCTTGGAACTTAGCCCAC
scaffold633628_13.5	1	p2	(TG)6	12	53	64	CTCATGTACGTTACGATGGCA
scaffold633689_14.6	1	p2	(GA)8	16	65	80	TGGTAGCTTGAATACACGTATGA
scaffold633697_15.5	1	p2	(AT)6	12	560	571	TTGATATCAGAGCCACTGCG
scaffold633709_17.6	1	p2	(GT)6	12	912	923	CAAGGAGTGGAGGTCGGTAA
scaffold633731_14.9	1	p2	(CA)6	12	2848	2859	GCTTTTCTTTCGACCCATCA
scaffold633746_11.5	1	p2	(TA)12	24	39	62	TTGACGATATATAGTGTTTTGACATGA
scaffold633785_14.9	1	p2	(TA)8	16	45	60	AAGGAGGTGTTATGAAGGGATT
scaffold633874_10.9	1	p2	(TA)8	16	140	155	TCAAGGTAAATGGGTCTAGCAAA
scaffold633962_12.0	1	p2	(AT)8	16	796	811	TTTTGGGTAATTGGTGAACCTT
scaffold633964_14.3	1	p2	(CA)6	12	6190	6201	AATCGGGTCCTCATCATCCT
scaffold634032_13.2	1	p2	(TA)8	16	289	304	AAACAACCCAAGAAAAATACGG
scaffold634113_15.0	1	p2	(GA)7	14	1875	1888	AGGGGACTAGGGTTGAAGGA
scaffold634122_15.4	1	p2	(GT)6	12	366	377	GTAGAATCCGGCGAGTGAAA
scaffold634208_12.9	1	p2	(TC)8	16	54	69	CCTAGAAAGAGAAGAAACAGAGGC
scaffold634212_13.2	1	p2	(GA)8	16	1008	1023	CACCATCAGCAGAGAAAGCA
scaffold634219_12.1	1	p2	(CT)7	14	120	133	GGTTGGTCAACCAAAACCTG
scaffold634244_10.1	1	p2	(TA)9	18	378	395	ATGGACGCTCCAGATTGTTT
scaffold634370_17.3	1	p2	(TA)7	14	637	650	TTCCCTCCAAAAATATGGGC
scaffold634375_12.2	1	p2	(AT)10	20	590	609	CGTGATAAGAACACTCATGTCCA
scaffold634431_12.5	1	p2	(CT)7	14	1153	1166	TACTTCTCACCATCCCCCAC
scaffold634568_11.5	1	p2	(AT)10	20	503	522	CCGATTTTTCGGGTATTGAA
scaffold634579_16.6	1	p2	(CT)8	16	1732	1747	GCCGGGTAAACTACACTGGA
scaffold634597_10.0	1	p2	(AT)9	18	461	478	TAAACGGCTCGGAAAAGACA
scaffold634642_13.9	1	p2	(AC)7	14	128	141	AACCATTGAGCAAAAACAATG
scaffold634697_11.0	1	p2	(AG)6	12	925	936	CCGGGTTTAAAGCAATCAAA
scaffold634759_20.2	1	p2	(CT)6	12	240	251	GGGAGGACAGTCCGTAGACA
scaffold634773_17.3	1	p2	(AG)6	12	1837	1848	AATATTCCACCACTGTGGGC
scaffold634784_17.2	1	p2	(AG)6	12	638	649	TTTGACTTGCAGCCACTCTG
scaffold634791_18.7	1	p2	(TC)6	12	630	641	TCCAACTCCCTCCATTCTTG
scaffold634799_12.4	1	p2	(AC)6	12	615	626	CGTGGCTTTGTTGAAACTGA
scaffold634801_13.3	1	p2	(TA)11	22	46	67	AAGGGAGTGCTCCCACATTA
scaffold634806_14.3	1	p2	(AG)6	12	394	405	TCTTTTTCCACATTTGAAGTTCTTT
scaffold634829_16.2	1	p2	(CT)7	14	621	634	TCGCAGCGAAACGTGAAC
scaffold634844_16.1	1	p2	(AC)10	20	747	766	CCATCAACAAATGCAATGGA
scaffold634850_18.0	1	p2	(TC)8	16	1111	1126	AAAGGAATCAGAGCGCAGAG
scaffold634855_12.6	1	p2	(TA)6	12	53	64	ATGGGAAGAGGGGGTATGAG
scaffold634881_10.0	1	p2	(AT)10	20	483	502	CCAAACAAACCGGCATACTT
scaffold634890_13.5	1	p2	(CT)7	14	292	305	GCACACGTATTAGGACCTCCC
scaffold634931_13.6	1	p2	(AT)6	12	251	262	CACCCTGTGACAGATACAGCA
scaffold634934_15.9	1	p2	(TA)8	16	563	578	GCGCATGCACCTAATTACAC
scaffold634957_20.2	1	p2	(AG)6	12	248	259	CAACAATCAGCAGTGGCAAT
scaffold634960_14.8	1	p2	(AT)8	16	2328	2343	TGATCAGCGCAGTCCAGTAG
scaffold635009_16.8	1	p2	(AT)6	12	446	457	AGAGCACTGCCAGATCAAAA
scaffold635012_14.4	1	p2	(AT)8	16	1165	1180	GGGAAAGTCGATGGTTTCAA
scaffold635134_12.4	1	p2	(TA)7	14	332	345	TCCTTTGAGGATGGATTGATG
scaffold635223_15.9	1	p2	(AT)8	16	192	207	TCGTTCCTTAAGTCTTCCGC
scaffold635224_14.5	1	p2	(AT)6	12	196	207	GCCTTGCAAGCCAGTTTATT
scaffold635267_13.6	1	p2	(TA)6	12	906	917	GCCCTCAACGTTGGTATCTT
scaffold635274_12.3	1	p2	(GA)6	12	195	206	ATTTGCATGCATAACCCACA
scaffold635290_12.6	1	p2	(TA)6	12	192	203	AGGCTTGAGTTTCCTCACGA
scaffold635357_13.5	1	p2	(TC)6	12	1473	1484	GCAGAGTTGTCGGGAAAGAG
scaffold635446_15.5	1	p2	(AG)11	22	2691	2712	CCGAAAAAGGGTATTGGGTT
scaffold635524_16.2	1	p2	(AG)8	16	3225	3240	AGGCTTAAACCAGGACCGAT
scaffold635558_15.1	1	p2	(TG)6	12	960	971	GCAACAGTTGTGGTTGATGG
scaffold635611_15.9	1	p2	(TG)10	20	1017	1036	TCGAAGCTTTGCTACTGGCT

scaffold635625_16.2	1	p2	(CT)6	12	54	65	CAAGATAACCCTTCGATTTAGGAA
scaffold635668_12.4	1	p2	(TA)7	14	789	802	ACTGGCCCAATAACCTTCCT
scaffold635685_14.5	1	p2	(AT)7	14	1779	1792	CGTAAGGCCTACTCGAGCC
scaffold635695_19.2	1	p2	(AT)9	18	499	516	TCTTGGTGGCGGTAGGTAAC
scaffold635724_13.9	1	p2	(GT)6	12	118	129	TGTACGACATTGTTTGCGCT
scaffold635771_15.0	1	p2	(TC)6	12	110	121	CTAGCCTTTGCCCCTTTTCT
scaffold635893_16.0	1	p2	(AG)9	18	907	924	CCCTTTTGATTCTTGCCTCA
scaffold635903_16.6	1	p2	(GA)11	22	321	342	TGAAATTCACTTGATCCGCA
scaffold635916_15.7	1	p2	(TA)8	16	209	224	TTCGAAATATCGGGGCATTA
scaffold635944_19.1	1	p2	(TA)6	12	196	207	TTCCTTGGACCAAAATAGCC
scaffold635963_14.3	1	p2	(TA)6	12	1590	1601	TTGTGGTAACCGATTGGGAT
scaffold636040_17.7	1	p2	(CT)6	12	312	323	TCAAAGCGATGGAACATAACA
scaffold636047_15.9	1	p2	(GA)6	12	141	152	GTGAGAGAGGACCGATTGGA
scaffold636268_13.9	1	p2	(TA)8	16	219	234	TGCTGATGTGGGAAAATCTG
scaffold636281_13.2	1	p2	(TC)7	14	141	154	GGATTCCCCCTCGAAGTTT
scaffold636287_13.9	1	p2	(TA)9	18	48	65	AGGGGGTTATCACTGGATCG
scaffold636510_16.2	1	p2	(AT)9	18	1465	1482	CCTTCAATTTTATAACCGAAACA
scaffold636563_13.4	1	p2	(TA)7	14	253	266	TCTAAGGAGGTGTTATGATGGGA
scaffold636568_15.8	1	p2	(GA)8	16	863	878	ACCTCTTTCCAATCTGGCCT
scaffold636614_14.3	1	p2	(AG)7	14	144	157	AGAGAGAGAGAAACGCGAGG
scaffold636619_15.4	1	p2	(AT)7	14	2421	2434	AGCGAACCAGAACCGAACTA
scaffold636631_15.4	1	p2	(GT)6	12	656	667	GCGTCGAGTACCACTTTTCC
scaffold636757_15.8	1	p2	(TA)6	12	1412	1423	TTTTCTCTGGATCACACCCC
scaffold636762_18.3	1	p2	(TA)6	12	908	919	GGCGTGGCTCAAGATATCAG
scaffold636789_13.5	1	p2	(AT)7	14	342	355	TGCAAAAGTTTTTGTTGGGT
scaffold636846_11.5	1	p2	(TC)6	12	129	140	TCTCTCTCCCACTCTTCCCA
scaffold636860_15.1	1	p2	(CT)8	16	324	339	AAGCCTTCCCACCGAGTATT
scaffold636892_13.3	1	p2	(TG)6	12	565	576	CTCTCCATTCTCTCGCTGCT
scaffold636960_12.1	1	p2	(TA)6	12	228	239	CCTCCTTTAACCAAAAATCCG
scaffold636980_15.6	1	p2	(CT)6	12	197	208	CCTTCTCTCTCCTTTCCCGT
scaffold636996_12.7	1	p2	(TC)6	12	401	412	GGCAACGAGACAGACAAACA
scaffold637101_16.2	1	p2	(CA)9	18	2876	2893	AGGCCAATTTTGAAGCAGAA
scaffold637124_11.1	1	p2	(AG)6	12	323	334	TTGGGAGGAGGAGATTGTTG
scaffold637128_15.6	1	p2	(AG)6	12	713	724	TGGACTGCTGTTCAGAGACG
scaffold637138_16.6	1	p2	(TA)12	24	5104	5127	TGTGCCCACTTAAAAATTTGG
scaffold637154_10.7	1	p2	(TA)10	20	1030	1049	CTGCAGATCTAAGGGCCAAG
scaffold637163_10.4	1	p2	(AT)8	16	1340	1355	GCGACCAAATCAATCACCTT
scaffold637184_13.6	1	p2	(AC)7	14	335	348	ATTTGGGTTTTAGGCTTGGG
scaffold637204_16.0	1	p2	(GA)9	18	2354	2371	TGCAAGTTCAGACAAACCCA
scaffold637235_14.6	1	p2	(CA)7	14	4406	4419	GGTTCAACCTCCCAGATTCA
scaffold637317_13.5	1	p2	(TC)6	12	75	86	TGATTTCCTCACCTTGTTTGTG
scaffold637323_13.3	1	p2	(TG)6	12	351	362	CCCCCAACTCTTCTTCTTCC
scaffold637388_12.8	1	p2	(TA)7	14	362	375	GCATCACCTCAACCCTTGAT
scaffold637390_16.6	1	p2	(AT)11	22	684	705	AAGTTGCACAATCCTAATGATTTA
scaffold637393_17.0	1	p2	(GA)7	14	1489	1502	TTCCCAATTGCGTAGTCCAT
scaffold637402_16.6	1	p2	(CT)6	12	805	816	CCAATTGAATGGAGTCAGGG
scaffold637457_16.7	1	p2	(AT)6	12	1467	1478	ATTGTGGGGAAATGATCCAA
scaffold637460_15.1	1	p2	(GA)7	14	38	51	CGTGGTTCGATCGTAAATGA
scaffold637591_12.0	1	p2	(AG)6	12	346	357	CCCGAAATAAGCTGCGTAGA
scaffold637715_17.1	1	p2	(CT)6	12	77	88	ATAACCATACCCACACCCCC
scaffold637807_15.8	1	p2	(GA)6	12	4488	4499	CGCTGCTGTAGTCGGAAGTT
scaffold637842_13.1	1	p2	(TA)6	12	563	574	GAAGAAAACTGCCGAGTTGC
scaffold637934_14.4	1	p2	(TA)8	16	417	432	TTGACCATAATGCCCCTATCA
scaffold637975_10.0	1	p2	(AT)11	22	401	422	ATCTCATGCCTTAGAGGGGG
scaffold638050_17.2	1	p2	(TC)9	18	3319	3336	CAAACCTAACTGAAATAGGAATGGA
scaffold638095_14.4	1	p2	(GA)6	12	456	467	GGAAATTGATGGATTTGGGA
scaffold638134_16.4	1	p2	(AG)8	16	1865	1880	TCAAACTTGCCAATCCAAAA
scaffold638179_17.8	1	p2	(AT)10	20	122	141	AAATAAGGATGTGTTATGAAGGGA

scaffold638220_16.[illegible]	1	p2	(CA)7	14	778	791	CCACCCGAATTGGATAATGT
scaffold638221_13.[illegible]	1	p2	(TA)8	16	193	208	ATGTGTCGGCCTGAATATGG
scaffold638240_16.[illegible]	1	p2	(AT)6	12	503	514	ATAGTTCCGTCGTCGTTGGT
scaffold638259_14.[illegible]	1	p2	(TA)8	16	246	261	TGGTGGGTTGAATGCTATGA
scaffold638550_12.[illegible]	1	p2	(AT)6	12	1084	1095	TTCACAAGCTTGAAAGCAACA
scaffold638568_10.[illegible]	1	p2	(AT)8	16	95	110	ACGCACTCTCAAGCACACAC
scaffold638709_10.[illegible]	1	p2	(AT)7	14	71	84	AAAATCAAAGGCCCACATTG
scaffold638816_16.[illegible]	1	p2	(CT)6	12	554	565	TGTGCATGCCCATATCATCT
scaffold638835_17.[illegible]	1	p2	(TC)6	12	87	98	CTAGCCTTTGCCCCTTTTCT
scaffold638877_13.[illegible]	1	p2	(TA)7	14	123	136	TGTATGGGGAGGGTTATCCA
scaffold638895_15.[illegible]	1	p2	(TG)9	18	223	240	TTGATTTAACTTGCATTCTTTTTCA
scaffold638911_19.[illegible]	1	p2	(GT)6	12	486	497	GAAGAAGGCGAAGTGGCTTA
scaffold638946_11.[illegible]	1	p2	(CT)6	12	53	64	CCTAGCTCCTTCCCCTTCAC
scaffold638951_14.[illegible]	1	p2	(TC)6	12	662	673	ACAAAGTGGTGAGCATTCCC
scaffold639028_17.[illegible]	1	p2	(TA)6	12	550	561	AGACCAAAAGGTCACGGGTT
scaffold639055_15.[illegible]	1	p2	(GT)6	12	3074	3085	CCCAAGATTTCCCATAAGCA
scaffold639078_13.[illegible]	1	p2	(TA)12	24	40	63	TTGACGATATATAGTGTTTTGACATG[illegible]
scaffold639236_11.[illegible]	1	p2	(CT)10	20	30	49	CAGATTTTAGGATTTCAGCTCACTG
scaffold639265_19.[illegible]	1	p2	(GA)6	12	199	210	CTCGGTCCGGATCTTTCAT
scaffold639291_15.[illegible]	1	p2	(AC)6	12	1222	1233	ATGGAAAACCACGAGTGGAG
scaffold639338_15.[illegible]	1	p2	(CT)6	12	199	210	GAACCCCTGTGACCATCTGT
scaffold639339_16.[illegible]	1	p2	(GA)8	16	131	146	TTGGAAATCAAGTAGAACTGAAAA
scaffold639352_14.[illegible]	1	p2	(TG)9	18	1250	1267	GCCAAACGCCAAAGAAAAAT
scaffold639404_16.[illegible]	1	p2	(AT)6	12	4321	4332	GCGAAATAGTTGGACATCGG
scaffold639455_11.[illegible]	1	p2	(AT)9	18	1221	1238	AAAATTTGTTTCCCACCAAATAA
scaffold639471_16.[illegible]	1	p2	(TA)6	12	195	206	GGATGTGGTCGAGTTTCGTT
scaffold639505_13.[illegible]	1	p2	(AT)7	14	1235	1248	TCGAGTATTGCTTCGCTTCA
scaffold639543_12.[illegible]	1	p2	(AC)6	12	216	227	TGCTCACTCCTGCACATTTC
scaffold639560_15.[illegible]	1	p2	(AG)7	14	1776	1789	GGATTTGACTGGTTGGGAGA
scaffold639606_10.[illegible]	1	p2	(AG)6	12	183	194	GTGTAGATCGCCATGGTGTG
scaffold639648_15.[illegible]	1	p2	(CT)13	26	2166	2191	CCCCAAAATTCCCTTTTCAT
scaffold639686_13.[illegible]	1	p2	(AT)9	18	552	569	CGTCACCCTCAACAAAAGGT
scaffold639697_20.[illegible]	1	p2	(AT)9	18	67	84	CAAAGGCCCACAATGGTTAT
scaffold639704_13.[illegible]	1	p2	(TA)6	12	88	99	AACGAATCTTTTCGTGGAGC
scaffold639737_14.[illegible]	1	p2	(TC)6	12	199	210	TCCGGTTCACCCATTTAGTC
scaffold639761_10.[illegible]	1	p2	(TA)8	16	242	257	AATTACACATACACGTGGGCT
scaffold639863_19.[illegible]	1	p2	(AT)12	24	40	63	GGCTAGGCTGGATAAGTTTCA
scaffold639875_13.[illegible]	1	p2	(CT)7	14	197	210	TGATGGGGTTTAATCATCTGG
scaffold639876_10.[illegible]	1	p2	(AG)7	14	41	54	CCCATGCATCCATATGAGAGA
scaffold639917_13.[illegible]	1	p2	(TA)6	12	146	157	AACATTGTTCTCCCCCACAA
scaffold639932_13.[illegible]	1	p2	(TA)9	18	269	286	CAAAGAGGAAATTCGAACGC
scaffold639967_13.[illegible]	1	p2	(TC)8	16	194	209	ACTGGTGTTTGTTCATGGCA
scaffold640047_11.[illegible]	1	p2	(TC)6	12	132	143	GTACTCTCATGCGCTCAGCA
scaffold640055_16.[illegible]	1	p2	(AG)10	20	144	163	TGCTTTTGACGGAGTGTGAG
scaffold640108_14.[illegible]	1	p2	(GA)6	12	2360	2371	AATTAAATGAATGGGTGGCG
scaffold640114_11.[illegible]	1	p2	(CT)6	12	316	327	AGAGATCACACAGGCAGTCG
scaffold640121_12.[illegible]	1	p2	(AG)6	12	327	338	TCACGGGTTCGAGTTTCAAT
scaffold640167_17.[illegible]	1	p2	(AT)8	16	307	322	CTAATGGCGAAGTGGATCGT
scaffold640193_11.[illegible]	1	p2	(TG)8	16	129	144	CATTTTACCGGCATGGTGTT
scaffold640221_13.[illegible]	1	p2	(AT)6	12	764	775	AACCCACATGCAAGTTTCGT
scaffold640264_14.[illegible]	1	p2	(AC)9	18	193	210	TCCATCTCCACGTACGACTG
scaffold640287_10.[illegible]	1	p2	(CT)7	14	433	446	CCCAAGATGAAACGGAGAAA
scaffold640293_11.[illegible]	1	p2	(GA)7	14	51	64	TCGATTCAGTTTCCCCACTC
scaffold640324_14.[illegible]	1	p2	(TA)9	18	307	324	TGAATTCGGTCAAGCATCTG
scaffold640329_15.[illegible]	1	p2	(CT)7	14	824	837	TGGTGTTGGGAAGAATCACA
scaffold640361_15.[illegible]	1	p2	(TA)7	14	197	210	GCCTTTCGTCACAAACATCA
scaffold640406_15.[illegible]	1	p2	(AC)6	12	2346	2357	AGAAATTCAAACCCGGAACC
scaffold640497_12.[illegible]	1	p2	(TA)8	16	773	788	TTCTTCAGTCCCTACCGTCG

scaffold640504_11.4	1	p2	(AT)13	26	410	435	GCGATATGCATCTACCGTGA
scaffold640536_15.4	1	p2	(TG)6	12	178	189	TTGAAGGGGTCTCTTGGATG
scaffold640550_15.6	1	p2	(TA)8	16	47	62	TGCGTTTTGACGATATATAGTGTTT
scaffold640558_15.2	1	p2	(CT)6	12	252	263	GGAAGATTCAAACCCCATCA
scaffold640581_14.0	1	p2	(CT)10	20	447	466	CCCAAGATGAAACGGAGAAA
scaffold640654_13.7	1	p2	(TC)9	18	193	210	TTCCATGTGCATGCAGAAAT
scaffold640691_13.5	1	p2	(AG)8	16	1990	2005	GACATGGGCACCTTATTGGT
scaffold640704_13.3	1	p2	(GA)6	12	992	1003	TCATGTCGCCGATCATAAAA
scaffold640717_13.8	1	p2	(TA)8	16	47	62	AAGGAGGTGTTATGAAGGGATT
scaffold640774_10.7	1	p2	(AT)6	12	66	77	ATGAATGGCCCATATTGGTT
scaffold640856_16.5	1	p2	(TA)11	22	649	670	TTTACGATTTCGATAGCCCG
scaffold640977_13.2	1	p2	(CA)6	12	227	238	GCCCAGTCATTTTGTCCCTA
scaffold640982_17.4	1	p2	(TG)6	12	679	690	GAGAGGGTGATTTTGCCACA
scaffold641023_13.9	1	p2	(TA)7	14	51	64	TGAGATAAGGGGGTGTTTGC
scaffold641057_18.1	1	p2	(GA)7	14	1627	1640	TGGTCTACTTCTATGGCGGG
scaffold641073_17.3	1	p2	(AT)10	20	383	402	ATTTGGTTTGCTTGGGATTG
scaffold641108_14.8	1	p2	(TA)6	12	394	405	TCCCTTTGATTCCCTTCTCA
scaffold641238_16.5	1	p2	(CT)8	16	358	373	GGCTACCCCTCTCGCTCTAC
scaffold641242_13.5	1	p2	(AT)6	12	199	210	GCTGATGTCGATTTTCCAGA
scaffold641328_14.4	1	p2	(AG)6	12	27	38	CAATTACAATGTTTTTCCAATCCC
scaffold641334_15.7	1	p2	(CT)8	16	321	336	CGCATCAAGTTCCCTCATCT
scaffold641399_15.5	1	p2	(TA)12	24	1500	1523	TCCCGTATACCTCCAATTTACAA
scaffold641421_11.2	1	p2	(TA)7	14	350	363	TCACATGTGGCGTCTTTTTC
scaffold641434_14.4	1	p2	(CT)9	18	1208	1225	CTGTGGTGGAAGGGTTGTCT
scaffold641451_18.1	1	p2	(AG)8	16	129	144	AACAACTATCTCCAGCCCCA
scaffold641523_12.4	1	p2	(TA)6	12	671	682	AGGAAAAGGAGGAGCCAGAG
scaffold641543_11.7	1	p2	(AT)9	18	66	83	CAAAGAAACTTGGGACGGAG
scaffold641555_15.5	1	p2	(CT)8	16	52	67	AATTCTTCCTCCGGAACACC
scaffold641799_12.2	1	p2	(AC)6	12	658	669	CTTCACGGGGTTGTCCTTTA
scaffold641807_16.8	1	p2	(TA)7	14	198	211	TGATTCTTGGTGGCAGATTG
scaffold641808_11.0	1	p2	(TA)6	12	79	90	CTAGGAGGTTCTCGCATTGG
scaffold641924_15.4	1	p2	(TC)7	14	198	211	CTTCAAAATCTAACCGGCCT
scaffold641988_16.2	1	p2	(TC)6	12	681	692	GCGAGAGCTATGTGGAGACC
scaffold642088_10.2	1	p2	(TA)10	20	261	280	AGGGAGCTGGCACTAGATCA
scaffold642140_13.1	1	p2	(AT)9	18	1204	1221	TTGAGATTGCGAATTCAACG
scaffold642208_19.5	1	p2	(AT)8	16	1526	1541	GGGTTGGATCAACCACATTT
scaffold642278_16.9	1	p2	(CA)6	12	1230	1241	ACACAAGCACACACATGCAA
scaffold642334_10.5	1	p2	(TA)10	20	598	617	TTAAACCATGCACGTAGGCA
scaffold642373_10.6	1	p2	(GA)7	14	52	65	CCAACTCAGTGTGTGAGTGGTT
scaffold642596_16.4	1	p2	(AC)6	12	106	117	CCACAGGATCGAATGAACAA
scaffold642656_15.0	1	p2	(TA)11	22	4093	4114	CCATTTGGTGCTGGAGTTCT
scaffold642663_12.6	1	p2	(AT)7	14	50	63	GGTTATTAGTTATTACGGATTTAACGC
scaffold642707_18.8	1	p2	(TC)6	12	172	183	CCCCTTTGTCATCTTAGGCA
scaffold642926_17.7	1	p2	(AG)6	12	201	212	CAATAAATTGGGGTCGGCTA
scaffold643009_18.7	1	p2	(AT)12	24	43	66	GACTAGAGTTGGTTTCTTAAATGACT(
scaffold643031_11.0	1	p2	(TA)6	12	526	537	AAGAAAATGGGCAGAAACGA
scaffold643073_10.1	1	p2	(AC)6	12	98	109	TCGGGATCTTCACATCTTCC
scaffold643200_12.6	1	p2	(TA)6	12	2002	2013	CGAAAACATTTACATACGATGCC
scaffold643221_13.1	1	p2	(AT)6	12	261	272	AAAAACGAGCATTTTAGGGTTA
scaffold643357_15.0	1	p2	(AG)11	22	2227	2248	CCAGACGACTGCAAAAACAA
scaffold643411_12.6	1	p2	(AT)14	28	355	382	TAGGTGTCGGATTTGGGAGA
scaffold643419_10.5	1	p2	(TC)7	14	112	125	ATTGTTGCTTTGCCATCTCC
scaffold643444_14.7	1	p2	(GA)7	14	407	420	AGAAACAAACGGAATGTCGG
scaffold643448_16.0	1	p2	(TA)6	12	1512	1523	GCATGGACCAGAAATTCCAC
scaffold643464_11.5	1	p2	(TG)8	16	169	184	CTTGTGGAATGAAAGGGTGG
scaffold643556_12.8	1	p2	(AT)9	18	954	971	GGAGGTGAAGATTGGGTGAA
scaffold643665_10.7	1	p2	(AT)7	14	573	586	TCCTTCCTTGCTCCTTTTCC
scaffold643730_15.3	1	p2	(TA)9	18	244	261	GCGCTTTTTGCCTGATATTT

scaffold643835_16.4	1	p2	(TC)9	18	840	857	ACCGCTTACATTCCGATTTG
scaffold643874_16.9	1	p2	(GA)9	18	1122	1139	GCAATACTGTTTCCACCGCT
scaffold643881_11.7	1	p2	(TC)9	18	330	347	TTCAGCACTCCATCAGCAAG
scaffold643901_15.5	1	p2	(CT)9	18	50	67	TGGCCAAAACATCAACTCAA
scaffold643902_15.5	1	p2	(AG)7	14	248	261	TAGAGCCGGGTTTAGAGCAA
scaffold643943_12.7	1	p2	(TA)10	20	52	71	GGTGACGATGGTGATTGATG
scaffold643944_21.5	1	p2	(AT)8	16	198	213	TAGTGAGTAGTTCCGCCCGT
scaffold644011_13.9	1	p2	(AT)9	18	301	318	GGCATTTTGGGAAAGACAAA
scaffold644044_16.9	1	p2	(TC)6	12	2014	2025	GGATCGGTGACAAAGGGAAT
scaffold644123_15.0	1	p2	(CT)8	16	198	213	GATGCAGCTTGGTCTCCTTC
scaffold644303_10.8	1	p2	(TA)9	18	217	234	AAAATGGTCCCCTCATGGAC
scaffold644328_15.5	1	p2	(TA)6	12	864	875	TGTCCAAATAATTCATCACCAAA
scaffold644391_17.0	1	p2	(AC)6	12	2744	2755	CTTCGCCATCTGGTTTTGAT
scaffold644404_15.2	1	p2	(CT)9	18	1250	1267	ATAAAATGGGCGATTCCCTT
scaffold644420_18.4	1	p2	(GA)10	20	193	212	TTGCTGCCACTACCTGTGAA
scaffold644432_17.2	1	p2	(TA)7	14	119	132	ATGGGCTGTCTCTTGGATCA
scaffold644442_14.5	1	p2	(AC)6	12	1867	1878	TCCATCCGAAATCCGAATAA
scaffold644469_14.0	1	p2	(AT)7	14	1787	1800	TATACAAATGGGCGCATCAA
scaffold644485_15.4	1	p2	(CA)6	12	737	748	ATTTTCGCAATCATATCGGG
scaffold644501_15.4	1	p2	(CA)6	12	530	541	TCCTCCTTTTCACAACTGGC
scaffold644645_17.1	1	p2	(GA)6	12	202	213	GTTGGTTACGGGAATTGCAT
scaffold644655_17.7	1	p2	(TA)7	14	1102	1115	AAAATTCAATGACAATTGGAGGA
scaffold644735_15.7	1	p2	(CT)8	16	1427	1442	TGGCATTAAATATTAGCCCCC
scaffold644755_15.1	1	p2	(AG)8	16	34	49	GCCAATAATTTTTAGCAAGCAA
scaffold644805_15.8	1	p2	(GA)6	12	446	457	GAGAAGTGGCGGACTTTCTG
scaffold644826_12.8	1	p2	(TC)6	12	747	758	CATGATCCCGTGAGATACCA
scaffold644897_16.2	1	p2	(TC)6	12	345	356	AGCGTACAATACAGCCCACC
scaffold644967_17.2	1	p2	(AT)9	18	917	934	GGTATGTCAATTTGCCGCTT
scaffold644988_16.9	1	p2	(TC)8	16	91	106	CGAATATGCAATCACCAAAGAA
scaffold645028_10.8	1	p2	(GA)6	12	81	92	CACGAGATTTACGTGGTTCG
scaffold645087_12.3	1	p2	(AT)9	18	88	105	CTGCATGCACCTGATTACAA
scaffold645097_11.2	1	p2	(CT)10	20	188	207	TCACCTTCACTTCCTGCTCA
scaffold645177_16.7	1	p2	(TG)8	16	946	961	CATGCATTTGTTGTCGTTTTC
scaffold645222_10.2	1	p2	(CT)6	12	255	266	ACACACACCGTGGACGTAGA
scaffold645261_16.9	1	p2	(CT)7	14	148	161	CCTGTGGAGGGAACTTTTCA
scaffold645374_12.4	1	p2	(TA)9	18	838	855	AAAGTGCACAGAAAAATGACTTTG
scaffold645450_15.1	1	p2	(AT)7	14	892	905	GGTTGGTAGCTCGGAACAAA
scaffold645454_16.0	1	p2	(CT)9	18	1958	1975	CTGCCAACTGAATGAAAGCA
scaffold645511_15.9	1	p2	(AT)7	14	2109	2122	CAAACACGGAGATTTCAACAA
scaffold645647_16.3	1	p2	(AT)11	22	746	767	GCGCTGTAGATCTAACGGATG
scaffold645652_16.4	1	p2	(GA)6	12	1622	1633	ATTTGGGTGTAGAGAGGGGG
scaffold645722_13.9	1	p2	(AT)8	16	243	258	ACACGAAACACCACTCCTCC
scaffold645734_15.8	1	p2	(GA)6	12	162	173	TTGTTGAAGGGTGGTTGTGA
scaffold645775_13.3	1	p2	(CT)9	18	539	556	GTTGCTTAGGGTCTGTTGGC
scaffold645776_13.4	1	p2	(CT)7	14	391	404	TTGCTTAGGGTCTGTTTGGC
scaffold645867_12.0	1	p2	(TA)9	18	553	570	CGGGGCTCACTAAAAACGTA
scaffold645878_14.0	1	p2	(TA)8	16	130	145	TACCCCGCTACCACCAATAG
scaffold645923_15.0	1	p2	(AT)7	14	1440	1453	CCTTGATTTCAACTGTCTCCG
scaffold645926_15.1	1	p2	(TC)6	12	2656	2667	TTAAAACCGACACCCACTCC
scaffold646078_12.6	1	p2	(AT)9	18	146	163	GTTTCGATAGTTTTCATTTCATTTT
scaffold646166_13.5	1	p2	(AG)6	12	299	310	GCAATACAAGCGATGTGTGG
scaffold646230_16.4	1	p2	(AG)6	12	2747	2758	GGGTAGATGCAACATAGGGG
scaffold646252_10.2	1	p2	(TC)7	14	304	317	GAAAGCATCGTTTTTGGCTC
scaffold646272_12.7	1	p2	(AT)6	12	341	352	ACAATATGGTGTTGGGTATTAAGAT
scaffold646333_10.0	1	p2	(TA)9	18	144	161	TCAGTTTTTGAGTTATGACGTTTG
scaffold646334_10.5	1	p2	(AT)7	14	251	264	GAAATATGCATTTGTCGTCACC
scaffold646383_11.6	1	p2	(AT)7	14	78	91	CAATTCAAAAATGAGTGAACCA
scaffold646412_12.1	1	p2	(AT)8	16	87	102	TTCTCTACACATAGAGACCAATTTTGA

scaffold646435_11.7	1	p2	(GA)6	12	203	214	CGGACGGGAAGAGATAAACA
scaffold646494_13.5	1	p2	(GT)7	14	202	215	CTGCCTCTTTCCACACCATT
scaffold646531_11.0	1	p2	(AG)6	12	291	302	GCGACAATAACTGTCTGCGA
scaffold646575_16.9	1	p2	(TG)7	14	1706	1719	GCCTCCATAACGAGCTCATAG
scaffold646577_11.4	1	p2	(GA)6	12	151	162	TTCTCGATTCCCGATTTTTG
scaffold646596_15.2	1	p2	(GC)6	12	158	169	GCACAACGGCTATCTGGTTT
scaffold646693_19.3	1	p2	(TA)7	14	202	215	TGGCTCATTCATGACCAAAA
scaffold646721_12.7	1	p2	(TC)6	12	156	167	CTACTTGCGTGCTGGAACTG
scaffold646882_10.0	1	p2	(AT)10	20	340	359	GGCCATTCAAATTTCAAATCA
scaffold646910_16.3	1	p2	(TA)6	12	1381	1392	TTGTCTATTCCTCCGATGGC
scaffold647051_13.8	1	p2	(CT)7	14	149	162	GGATCGAAAGGGTGAGTGAA
scaffold647156_11.6	1	p2	(TG)8	16	327	342	CCATCTTGATGCAGGTGACA
scaffold647184_17.1	1	p2	(AT)10	20	236	255	CTATTTGCCGCTGAAAAAGC
scaffold647209_15.7	1	p2	(GA)6	12	838	849	GAGCTGCGACATTTTCAACA
scaffold647349_11.4	1	p2	(AT)6	12	627	638	AATTCAAGCATTTTCCGCTG
scaffold647449_10.3	1	p2	(CA)6	12	270	281	GCTGTCCACCATCTCCTCTC
scaffold647565_16.4	1	p2	(CA)8	16	3764	3779	CCATAGTCCAAGATGTGGTGC
scaffold647873_14.9	1	p2	(GA)11	22	44	65	TGGATGGATGGATTAGCAGA
scaffold647905_14.2	1	p2	(CT)7	14	368	381	CATTCTTTCTCATCACCCCC
scaffold647910_10.3	1	p2	(TA)6	12	204	215	ATGGGAAGAGGGGGTATGAG
scaffold647913_12.9	1	p2	(AT)9	18	199	216	TTTTAAAAGGATTTATGGCATGAT
scaffold647914_12.8	1	p2	(AT)6	12	54	65	GGAAGAATGGACCCTAATTCAA
scaffold648038_15.9	1	p2	(AT)6	12	668	679	CGCTTTCACCAGAATCTTCC
scaffold648061_18.2	1	p2	(GA)6	12	189	200	AAGTGTTTTCCCACAGACGG
scaffold648103_13.4	1	p2	(TA)7	14	261	274	CGCCAATTAATCATAGCCGT
scaffold648167_16.8	1	p2	(CA)6	12	1084	1095	AGGCCATGTGTTCGAGTTTC
scaffold648208_15.9	1	p2	(TA)13	26	40	65	AAGGGTTATCACCTGATTTTCTC
scaffold648281_17.3	1	p2	(AG)6	12	205	216	GATGGTGGACAAGAGAGGGA
scaffold648306_14.9	1	p2	(TA)6	12	53	64	GAAGTGTTATGCGTTTTGACAAT
scaffold648350_12.9	1	p2	(AG)7	14	203	216	AGGGGAAAAGAGAGAGGGTG
scaffold648456_11.7	1	p2	(AT)7	14	445	458	AAGGGGGAAACAACTGAGAAA
scaffold648465_13.0	1	p2	(GT)6	12	127	138	AGTGTGTGTTTGTGCGTGTG
scaffold648480_14.5	1	p2	(AG)9	18	415	432	ATTTTTCCTTGTTGGGGTGG
scaffold648481_12.2	1	p2	(AT)6	12	357	368	TCCGACCGGTTTTTAAATCA
scaffold648494_16.3	1	p2	(TC)6	12	824	835	CGTTTGATTTTTCACTAGAACCA
scaffold648531_11.9	1	p2	(AG)6	12	35	46	AACAACACAAAACCCTACAGCA
scaffold648539_14.6	1	p2	(AT)8	16	1410	1425	TCCATGCTTGAGCAGTTGAG
scaffold648766_11.6	1	p2	(AT)6	12	390	401	TGCAGGGTATGCATGTTTGT
scaffold648790_15.1	1	p2	(AG)11	22	3970	3991	AACTCCCTATCCAACTGCGA
scaffold648830_14.0	1	p2	(TA)6	12	1444	1455	TGTGGCTTCTGCTGTTATGG
scaffold648834_15.0	1	p2	(GA)6	12	200	211	AAATGGGGGAGAAGAAATGG
scaffold648850_14.8	1	p2	(AT)6	12	912	923	ATCGCCTTTACTCCATTCCA
scaffold649020_14.8	1	p2	(TA)8	16	1172	1187	TTCGTGGAAGGGAGAGATTG
scaffold649132_15.9	1	p2	(AT)6	12	1305	1316	TGAGAAGGGTGGGTATGAGG
scaffold649147_13.3	1	p2	(AT)10	20	2606	2625	TTTGTCCGTCAATATTAGGGATTT
scaffold649212_16.7	1	p2	(TA)11	22	152	173	CGCCCCAAGATTTGATAATG
scaffold649229_16.0	1	p2	(TC)7	14	203	216	CAACTCTGCGTCTCCTTTCC
scaffold649262_12.8	1	p2	(AT)10	20	1038	1057	ACCAATGCACATTTCCTGCT
scaffold649276_13.9	1	p2	(AG)11	22	2020	2041	GATTTCCAGGCATGAGGTGT
scaffold649279_16.9	1	p2	(CT)6	12	163	174	TGCTTTCGGGAAAAGCATAA
scaffold649367_10.0	1	p2	(GA)6	12	253	264	CTCAAAGCTCCACAAGCTCC
scaffold649579_13.5	1	p2	(TC)10	20	250	269	TCCCACCTCCATCTCATCTC
scaffold649617_17.3	1	p2	(CT)6	12	69	80	TTTAGACCAATCCCAGATCCC
scaffold649627_11.8	1	p2	(TA)8	16	380	395	TGGGAATTGGGTTTTGAATG
scaffold649736_17.8	1	p2	(AT)6	12	801	812	TGTCGTAGCATTTTTGTATGTTTTT
scaffold649739_12.6	1	p2	(AT)6	12	191	202	TGACGCCTAGAATCATCGAA
scaffold649741_16.6	1	p2	(TG)6	12	1493	1504	ATGTCGTATTTGCATTGGCA
scaffold649744_16.5	1	p2	(TA)6	12	717	728	GTGCAACGCACGTGTAAAAT

scaffold649746_13.3	1	p2	(AT)7	14	442	455	GATTTCAATGGACAGGCACC
scaffold649775_11.3	1	p2	(AT)6	12	338	349	AGCCGTTTGTCCGATTTATG
scaffold649780_16.8	1	p2	(GA)6	12	3887	3898	CAGGAAAAAGAAGATTGCACC
scaffold649841_15.0	1	p2	(GT)6	12	596	607	TCCCTTAATTGGAAGCCAAA
scaffold649845_15.0	1	p2	(TA)6	12	878	889	TGCAAGGCATGTGCAATTAT
scaffold649856_13.5	1	p2	(AG)6	12	37	48	TGGGGATTTTAGAGAGTATTGTGA
scaffold649889_16.7	1	p2	(CT)6	12	332	343	CCCCCTTTCTCTCCCTACAC
scaffold649893_17.1	1	p2	(AG)6	12	314	325	GGCCCACTTCTTGGTAAACA
scaffold649972_16.4	1	p2	(CT)8	16	201	216	TCGACTTTAACCGTGGGTTC
scaffold649979_14.7	1	p2	(TA)8	16	736	751	CACATGGGCATATGTGTCAAA
scaffold650072_10.9	1	p2	(TA)6	12	394	405	GGGTTGACATGGCTTCGTAT
scaffold650114_10.6	1	p2	(TG)6	12	138	149	CTCCCATTTCTGTGTTCTTCG
scaffold650121_13.0	1	p2	(CT)7	14	414	427	GCCTCCTCCTCCCTCTCTT
scaffold650213_14.9	1	p2	(TA)9	18	201	218	TTTTTACCCCATTTTGCCCT
scaffold650254_12.0	1	p2	(GA)6	12	302	313	GTGAGTACAAGCGGGTGACA
scaffold650419_11.8	1	p2	(AT)6	12	154	165	AATTGAGAGTAGCCGACCCC
scaffold650500_17.5	1	p2	(CT)7	14	205	218	TTCTCATCTTCAACCCCTGG
scaffold650504_17.1	1	p2	(GA)8	16	84	99	GATGAAGTTAGGTGTTGGATGC
scaffold650545_10.8	1	p2	(AT)6	12	999	1010	GCATGTATGCATATCCCTGG
scaffold650576_16.9	1	p2	(TG)9	18	1313	1330	GGTCGGTAAAAAGTGGCAAA
scaffold650722_10.6	1	p2	(CT)6	12	197	208	TCAGGGACTCATTTCCCAAC
scaffold650771_16.0	1	p2	(TC)10	20	1385	1404	AGCAATCAGCTCCTGCAAAT
scaffold650789_16.3	1	p2	(CA)6	12	547	558	CAATCGGCAGAAGTTTGCTA
scaffold650837_17.2	1	p2	(TC)6	12	1440	1451	CGAATGAGCTCGATTTGGTT
scaffold650859_18.8	1	p2	(TA)7	14	2820	2833	ACGGGAGACATTGCCATAAT
scaffold650917_18.6	1	p2	(TA)11	22	219	240	TTTAACCGAACGAAAATGCC
scaffold650973_21.0	1	p2	(AT)10	20	601	620	ATATGGGTTGTCGATCTCGC
scaffold651067_15.5	1	p2	(GA)6	12	1592	1603	TATGAATTTTGGCCCTTGGA
scaffold651073_15.0	1	p2	(TG)6	12	792	803	AACTTTTTGGTTGGCAAGGA
scaffold651126_14.1	1	p2	(GT)7	14	247	260	TTGTGATTCGAAAATTGGCA
scaffold651215_14.9	1	p2	(AT)6	12	335	346	TGTTTCCCCAATCCACATTT
scaffold651226_15.1	1	p2	(AT)6	12	838	849	GACGACCTTGTCATCCAACA
scaffold651327_16.1	1	p2	(CT)6	12	119	130	AGCATCTCTTGCTCTCTCATTG
scaffold651456_18.5	1	p2	(CT)7	14	64	77	GGAGATGCACTTCACCCAAT
scaffold651487_16.7	1	p2	(AG)8	16	153	168	AGAGGCATTTTGTTTGGAGC
scaffold651544_10.0	1	p2	(AT)7	14	299	312	CAAAACCCGTTACATATCTTTGG
scaffold651567_16.3	1	p2	(GA)6	12	1676	1687	AGAAGTGGTGGCGGATTATG
scaffold651585_11.3	1	p2	(AT)9	18	568	585	ACTGCGATCACGAATTCACA
scaffold651605_14.1	1	p2	(AT)8	16	754	769	GGAGGAAGAAGAAGAAAAGGC
scaffold651702_23.6	1	p2	(AC)6	12	206	217	TTGGTGCTCCACTATATTGCC
scaffold651841_14.4	1	p2	(CT)7	14	152	165	AGATGCCACTAACCCTTGAA
scaffold651923_16.5	1	p2	(TC)7	14	991	1004	TACCAGTGAGAATCCTGGGC
scaffold651971_13.3	1	p2	(TG)9	18	148	165	GTAACCGTCATGTTCCGGTC
scaffold652029_14.6	1	p2	(CA)6	12	1217	1228	TATGAGATGTGGGGGAGAGG
scaffold652045_16.4	1	p2	(AC)6	12	2569	2580	TCTTTCCCTCCCAATTCTGTT
scaffold652111_14.1	1	p2	(CT)6	12	2134	2145	TCAACGGTGAGTTATGGCAA
scaffold652217_11.9	1	p2	(CT)6	12	297	308	TGTTAGAAACAAACGCGGCT
scaffold652248_16.1	1	p2	(AG)6	12	1675	1686	GCTTCAATTGAGCAGAAGCC
scaffold652291_10.3	1	p2	(GT)8	16	284	299	CGCCCACAATTTGATACCTT
scaffold652307_16.5	1	p2	(AG)7	14	515	528	ACAACGTGATACGCTGCAAG
scaffold652331_17.2	1	p2	(GA)6	12	402	413	CGTTGCATTCGTCTTTCTCA
scaffold652343_14.8	1	p2	(AT)13	26	45	70	ACCATATTTAAGGGTTATCACCTG
scaffold652370_10.0	1	p2	(AT)6	12	365	376	CTAAACCTTATCCCCCACCG
scaffold652373_10.1	1	p2	(AT)7	14	52	65	CAAAATTTGGAACCTGTCTTCA
scaffold652391_15.9	1	p2	(GA)11	22	1206	1227	CCAAGAAAATCCCAGAAGCA
scaffold652468_18.4	1	p2	(CT)6	12	311	322	ATATTCTCCCGAACCGACAC
scaffold652486_19.1	1	p2	(TC)6	12	330	341	GGAAAATTCCGTTTTTGCCT
scaffold652495_14.1	1	p2	(TC)6	12	125	136	CTCTTCATTCCCTCATCCCA

scaffold652583_12.[illegible]	1	p2	(AG)12	24	48	71	TTCGAAACTGCATGAAAATCA
scaffold652588_17.[illegible]	1	p2	(AT)8	16	102	117	CACGTGTAAGGGTTGAAGAAG
scaffold652651_15.[illegible]	1	p2	(GA)6	12	3389	3400	AGCTGAAGAGCTGATGGCTT
scaffold652697_15.[illegible]	1	p2	(TC)6	12	1215	1226	TGAACTGGCTACTGGCTGTC
scaffold652699_10.[illegible]	1	p2	(AG)6	12	205	216	GTGTAGATCGCCATGGTGTG
scaffold652723_12.[illegible]	1	p2	(AT)6	12	156	167	GCGTCGTGGATCAAGGTAAT
scaffold652760_13.[illegible]	1	p2	(AT)7	14	207	220	ACGGTTACGGGCTGGTATTT
scaffold652798_14.[illegible]	1	p2	(GA)8	16	1411	1426	GGACAGATCAGAAGCAAGGC
scaffold652882_15.[illegible]	1	p2	(AT)7	14	1269	1282	AGAGCCACAAATTTTTCTTCAT
scaffold652932_14.[illegible]	1	p2	(TA)6	12	54	65	TGTGTGTGTGGTTCTATGTTGTG
scaffold652944_16.[illegible]	1	p2	(AT)7	14	620	633	TTTCAGGCCATAACTCGGTC
scaffold652958_17.[illegible]	1	p2	(GA)7	14	3164	3177	TGCAGATCAATAAAATGGCG
scaffold653091_12.[illegible]	1	p2	(AT)6	12	825	836	CGAAATCAAGCAACCAGTCA
scaffold653094_13.[illegible]	1	p2	(CT)7	14	96	109	CACTCTGCTAGGGTTGGGAG
scaffold653108_15.[illegible]	1	p2	(TA)7	14	154	167	TCAAGTTCAAATGCCAAGCTC
scaffold653141_13.[illegible]	1	p2	(AG)7	14	556	569	TATTGCCCATCACTCCCATT
scaffold653167_16.[illegible]	1	p2	(TC)10	20	322	341	CCCGATTTCTACACCCCTCT
scaffold653179_13.[illegible]	1	p2	(TG)6	12	486	497	CGCTGCACCTTCAAGTAACA
scaffold653222_11.[illegible]	1	p2	(GT)7	14	127	140	GACCACCCTAACGGATTTGA
scaffold653233_13.[illegible]	1	p2	(CA)7	14	677	690	TACAGCAGATGCCTCGTTTG
scaffold653275_11.[illegible]	1	p2	(AC)6	12	371	382	TTCTTATACCAAAACCTCCACTCA
scaffold653349_16.[illegible]	1	p2	(TA)6	12	1292	1303	TGTTGGACCCCCTAACTCAA
scaffold653358_12.[illegible]	1	p2	(GA)6	12	406	417	ATTTGCAAGCCTGTGGTAGG
scaffold653365_10.[illegible]	1	p2	(TA)9	18	320	337	TCGACAAGAAGCAACACAGG
scaffold653389_16.[illegible]	1	p2	(TC)12	24	2263	2286	AGAGGAAACCACCACCGTTA
scaffold653405_18.[illegible]	1	p2	(AT)10	20	1468	1487	TGTGGCATATTGATGATGGTG
scaffold653457_16.[illegible]	1	p2	(TA)8	16	301	316	TAGTTCCTCGCGGATCAATG
scaffold653581_10.[illegible]	1	p2	(AT)12	24	583	606	TTCCCCCAGAGAAGTTGTTG
scaffold653582_20.[illegible]	1	p2	(AT)7	14	208	221	CGCCCCAAAGTGCTTTAATA
scaffold653583_17.[illegible]	1	p2	(CT)6	12	1396	1407	GTGACAAGCATTCCTCCACC
scaffold653589_14.[illegible]	1	p2	(AT)6	12	675	686	GCCTTCCTTGGTAGTGGACA
scaffold653603_14.[illegible]	1	p2	(CT)7	14	652	665	TGACACATAGCCCGATGAAA
scaffold653648_16.[illegible]	1	p2	(AG)7	14	500	513	AAGCTGAAAAGTCGAGAAAATG
scaffold653667_15.[illegible]	1	p2	(TC)6	12	557	568	TCGATGAAATAGGGTCGAGG
scaffold653677_10.[illegible]	1	p2	(AT)8	16	157	172	AAGGAGATTGGGAGGAATGG
scaffold653707_13.[illegible]	1	p2	(GA)6	12	210	221	ACAGAGCTGAGGAGAGCGAG
scaffold653777_20.[illegible]	1	p2	(AT)7	14	207	220	AAATGGACCCACGAGATTTG
scaffold653827_14.[illegible]	1	p2	(AG)6	12	529	540	TTTTTGAGGGAACAATTTGAGAA
scaffold653858_19.[illegible]	1	p2	(TC)6	12	145	156	TGCAATCACCAATGAACTGA
scaffold653864_14.[illegible]	1	p2	(AT)6	12	475	486	TTCAATCAGCCAATGGACAA
scaffold653886_17.[illegible]	1	p2	(CT)6	12	477	488	GAAAATCGGACGTCGAAGAG
scaffold653964_14.[illegible]	1	p2	(CT)7	14	278	291	CGACTCTCGTTGGCAGATTT
scaffold654011_14.[illegible]	1	p2	(TA)12	24	114	137	GTTATCACCGGATCGCTACC
scaffold654038_18.[illegible]	1	p2	(TA)7	14	917	930	CCTTGTCCATGATTTCTTTGG
scaffold654087_12.[illegible]	1	p2	(AG)7	14	507	520	AAGGGAGGCTCTGCATCATA
scaffold654099_14.[illegible]	1	p2	(AT)8	16	419	434	AATCCAAACCATCCATTCCA
scaffold654154_14.[illegible]	1	p2	(AG)6	12	133	144	GCCCACAACTCTGGTTTCTC
scaffold654189_15.[illegible]	1	p2	(TA)6	12	194	205	GAACTTATGGGAAGGAGAACCA
scaffold654212_14.[illegible]	1	p2	(AT)6	12	1489	1500	CATAGCGAGGAAGTCCCAAG
scaffold654264_16.[illegible]	1	p2	(CT)6	12	210	221	GCCTCAAGAATGATCCCAAG
scaffold654315_15.[illegible]	1	p2	(TC)6	12	160	171	ATCATTACTGACCGCGAACC
scaffold654424_10.[illegible]	1	p2	(GC)6	12	362	373	TCCAATACGAAGCCCAAATC
scaffold654430_16.[illegible]	1	p2	(TC)6	12	2110	2121	AAAAAGGGATGAAAAGGGGA
scaffold654437_12.[illegible]	1	p2	(TA)8	16	206	221	CTGTAGATCAGAGGGCCGAG
scaffold654461_16.[illegible]	1	p2	(CA)10	20	409	428	GTGTCCGGAGCTATTTCTGC
scaffold654482_17.[illegible]	1	p2	(AT)6	12	99	110	AGCACACAGGTGAAGACGATT
scaffold654560_16.[illegible]	1	p2	(GA)6	12	2283	2294	ACTCCCTCCGTCCCATAAAC
scaffold654583_12.[illegible]	1	p2	(AT)8	16	999	1014	GCATTTTCTCCATGTGGGAT

scaffold654586_17.3	1	p2	(CA)8	16	206	221 GCCACTCCAAATTCTTTCCA
scaffold654602_15.(	1	p2	(CT)6	12	3906	3917 TCCTCCCGAGGATAGTCTCA
scaffold654608_19.3	1	p2	(TC)8	16	317	332 CTGCACTCTCCAGGAATCGT
scaffold654729_12.2	1	p2	(AT)9	18	687	704 TGGGGCGCGTATTTAGATTA
scaffold654741_16.1	1	p2	(CT)6	12	91	102 CCCACACTCTGCTTTCCATT
scaffold654749_13.8	1	p2	(AT)6	12	675	686 TATGCATGGCATGAGTGTGA
scaffold654765_17.2	1	p2	(CA)6	12	911	922 ACCGGAGTGTGAAGACGAAC
scaffold654787_15.4	1	p2	(AT)6	12	973	984 AATCTGACACGTCGGGAACT
scaffold654839_14.6	1	p2	(AT)7	14	1495	1508 TCAGCAATGAGTTGCTCCAT
scaffold654908_17.4	1	p2	(CT)6	12	354	365 GGTTGTCGTCTCTTGCCACT
scaffold654938_10.2	1	p2	(TA)7	14	160	173 ATGTTCGGATTTTGAAACGG
scaffold654952_17.3	1	p2	(GA)8	16	2995	3010 CCTTACTCGAACAGCCCAAC
scaffold654973_11.2	1	p2	(TA)11	22	42	63 GCGTTTTGACAATATTTAGTGTTTTG
scaffold655030_18.3	1	p2	(TC)6	12	469	480 AATTTCTCACTCTCCGCTGC
scaffold655058_14.6	1	p2	(AT)6	12	1404	1415 GAGAGAGAGGTTGTGGCCTG
scaffold655065_10.9	1	p2	(GT)9	18	43	60 AAGAGTGTGTGTGTGGGTGTG
scaffold655080_16.5	1	p2	(TA)6	12	210	221 CTACGTGAGGCGGTGATTTT
scaffold655084_15.3	1	p2	(AG)6	12	55	66 TCACAATACAACTGCCGAGC
scaffold655203_14.(	1	p2	(TA)7	14	315	328 TTGACAACCAGGTCACGAAA
scaffold655228_14.2	1	p2	(AG)11	22	1099	1120 GTTTTGTTTTCGTTCCCTCG
scaffold655241_14.4	1	p2	(TA)10	20	1459	1478 GGACGGCAACAGGAGTTACA
scaffold655260_10.6	1	p2	(TA)7	14	240	253 AGGTGGCAACATAATCAGCC
scaffold655303_15.2	1	p2	(GA)9	18	3025	3042 GCTCTGCATTAGCAGCTTCC
scaffold655334_17.1	1	p2	(TG)8	16	1681	1696 GATTTGGTTGGTCAAGTGGG
scaffold655336_16.(	1	p2	(TG)7	14	209	222 TCACATTCACACCTAACGCAA
scaffold655406_16.7	1	p2	(AT)6	12	211	222 CTTACCCACTCCACCACCTG
scaffold655500_10.9	1	p2	(GA)7	14	208	221 TAAAGGCCGCATCCTATCAC
scaffold655618_13.7	1	p2	(TA)10	20	42	61 CGGTTATCACCGGATCGC
scaffold655723_20.2	1	p2	(TA)7	14	209	222 CCAAGAATCAGTTAAGCCCG
scaffold655804_13.(	1	p2	(TA)10	20	2607	2626 AGGCCAAAAATGAACCACAC
scaffold655946_15.3	1	p2	(GA)8	16	1398	1413 TGCTAGGAAGCTGAGGAAGC
scaffold655992_14.1	1	p2	(GC)7	14	547	560 CGAGTCGGCAGACCTAAGAC
scaffold656032_14.7	1	p2	(CT)7	14	646	659 GCCAGTGTCCATCCATTTCT
scaffold656049_16.1	1	p2	(GA)6	12	752	763 GGAGGCATGGAAGAGAATGA
scaffold656095_14.8	1	p2	(TA)6	12	304	315 AGTAGAAGCATTTCGTCGGG
scaffold656176_13.8	1	p2	(TA)6	12	1325	1336 TTGACATGGTGTAGTCATCAAGG
scaffold656344_14.2	1	p2	(CA)6	12	443	454 GTGATTGGCTGGAAGAGCAC
scaffold656386_15.(	1	p2	(CA)6	12	2195	2206 TGACGGAACACGTCAGATTT
scaffold656448_18.8	1	p2	(TA)6	12	596	607 CATACTTCCACCGCCTTCAT
scaffold656456_16.6	1	p2	(CA)6	12	478	489 CAAGCACACCTACACGCACT
scaffold656509_12.9	1	p2	(TA)7	14	1033	1046 TTCAGGGAATCTTCGAATAAAAA
scaffold656749_11.6	1	p2	(AG)7	14	394	407 TGAGGCTTGTGGTTCAGGTT
scaffold656932_15.8	1	p2	(AT)7	14	396	409 GGTTCGGGCATAATGACATC
scaffold656980_13.8	1	p2	(AT)8	16	586	601 ATTTGGCCCTTGCAAGTATG
scaffold656991_12.7	1	p2	(GA)6	12	657	668 AAAACCAAGGATATCGGCCT
scaffold657127_15.8	1	p2	(TC)7	14	211	224 TCTCCCCCGGTAAACAGAAT
scaffold657140_16.8	1	p2	(TA)7	14	79	92 ATGTATGGCCCAGATTGGTT
scaffold657167_16.(	1	p2	(GC)6	12	180	191 CCCAATATCAGGTAAGGGCA
scaffold657174_10.6	1	p2	(AT)7	14	210	223 TGTTGGGATGAAATGTGAGG
scaffold657226_15.5	1	p2	(TG)6	12	357	368 GGATATGAAGTGGGAGCCAA
scaffold657239_12.4	1	p2	(AT)6	12	587	598 TGCCACCAAAATCAAATCCT
scaffold657300_13.1	1	p2	(AT)6	12	265	276 TCAAACCCTGTCATTCACCA
scaffold657457_15.8	1	p2	(TA)7	14	123	136 CCCAATTTGCCCTTATATGC
scaffold657464_17.1	1	p2	(TA)8	16	381	396 AAAATCAATTCGCGATCACA
scaffold657480_14.1	1	p2	(TC)7	14	655	668 TGCTGCAAGCTTAAGAAACAA
scaffold657509_20.6	1	p2	(AC)6	12	185	196 TCTTTTTCGAACCGAACCAC
scaffold657551_13.3	1	p2	(TC)6	12	97	108 GGGACTGCTGGATAATCCCT
scaffold657567_15.2	1	p2	(AT)8	16	176	191 ACTAAACTAGACCGGGCCGT

scaffold657618_15.6	1	p2	(TA)6	12	1078	1089	TGATTCTGGGTTTCCAGTCC
scaffold657644_15.7	1	p2	(TA)8	16	2350	2365	AACACCAACATGCTGACGAA
scaffold657865_13.9	1	p2	(AG)7	14	111	124	AACCTCTCGTGCTCCAACAT
scaffold657937_10.9	1	p2	(AT)6	12	303	314	GAATCGTCCCAAATTCCCTT
scaffold657960_15.1	1	p2	(TA)13	26	652	677	GTTTGGCCTCACTGGTGATT
scaffold657986_10.4	1	p2	(TC)8	16	684	699	TCAAAATTGGCACGATACGA
scaffold658006_15.4	1	p2	(AT)7	14	431	444	TCACAACCAAAATCTTGCTCA
scaffold658023_12.2	1	p2	(AT)6	12	1149	1160	ACCATTCAACGCAACCATTT
scaffold658056_12.5	1	p2	(AG)9	18	1155	1172	ATGCTGCTGTTTTCCATTCC
scaffold658065_10.6	1	p2	(TA)8	16	94	109	AAATCATTGCTTGAGCTTGAA
scaffold658104_14.8	1	p2	(TA)8	16	393	408	TCAATCCAAACTCTATTTTATCCGA
scaffold658132_19.7	1	p2	(TG)9	18	68	85	AGACCTGTCACCGTTTTGCT
scaffold658174_11.6	1	p2	(TA)8	16	67	82	TTTTAGTTATCACAGGATCACCC
scaffold658195_16.9	1	p2	(GA)10	20	3944	3963	ACTTGGCCCACAAGAACAAG
scaffold658213_16.1	1	p2	(AT)6	12	213	224	ACGGCCAATATTTGCTCCTA
scaffold658298_15.9	1	p2	(AG)6	12	1011	1022	GTGCTAACGATTTTTCCCCA
scaffold658309_17.9	1	p2	(CT)6	12	241	252	CACCGATGTTGAGGCCTACT
scaffold658334_14.5	1	p2	(TA)9	18	325	342	CATGGACGAAAATAGCCTCC
scaffold658337_15.2	1	p2	(CT)6	12	213	224	GTTTGCTGCTGCTGTTGTGT
scaffold658430_15.2	1	p2	(TA)6	12	2110	2121	CCATGCGCGGTCTAAATAAT
scaffold658441_17.4	1	p2	(TA)9	18	39	56	TTTAGTGTTTTGACATGATTACTCG
scaffold658444_13.8	1	p2	(TC)6	12	96	107	GGACCTCTGGACAATCCCTT
scaffold658485_10.2	1	p2	(TG)8	16	153	168	CAGTGTACAACCCCGGAATC
scaffold658489_14.6	1	p2	(TA)10	20	63	82	CTACAACATCATGCAAATCAATAGT
scaffold658580_16.0	1	p2	(AG)8	16	1536	1551	TATGGATTTAACTCCCCGCA
scaffold658666_18.0	1	p2	(AT)8	16	320	335	CAAGAACGAGAGGGTCAAGG
scaffold658747_14.6	1	p2	(AT)6	12	154	165	CAGCTCCAAGACATTCCACA
scaffold658755_11.1	1	p2	(TA)9	18	384	401	GACAAATGTGGCGTATGCTG
scaffold659005_13.2	1	p2	(AT)6	12	430	441	GCATTCCTTTCCCATCTTCA
scaffold659011_13.5	1	p2	(AT)6	12	293	304	TGCTCTAATATTCGGCCGTT
scaffold659016_17.0	1	p2	(TA)8	16	243	258	TTCGGAGCATGAATTAAGTGG
scaffold659045_14.6	1	p2	(AC)6	12	49	60	ATTGGCATATGTCCCCAAGA
scaffold659106_11.1	1	p2	(AT)9	18	465	482	TGACCCAGCCTTTTTAGGATT
scaffold659244_13.1	1	p2	(CT)7	14	1422	1435	ATCCAACGGATGAAAAGCTG
scaffold659245_11.5	1	p2	(TA)10	20	577	596	TTGCAAAAGAAATTGCTTAGCTT
scaffold659261_15.9	1	p2	(TC)6	12	882	893	TTTGTGCCTCTGATCCCAAT
scaffold659327_14.9	1	p2	(AG)8	16	160	175	TTGCAGCTGGAAATGAAGAA
scaffold659357_15.1	1	p2	(TC)7	14	2678	2691	CAACCTTTGAAGACTCCGTCA
scaffold659374_10.9	1	p2	(TA)8	16	58	73	ATGGATGGAGTCAAAGCCAA
scaffold659386_13.7	1	p2	(TA)7	14	1104	1117	CGAAAATTCGGATCGGCTA
scaffold659439_17.5	1	p2	(TG)7	14	212	225	GGTTAGGAATGCATTAAATACCAA
scaffold659440_14.2	1	p2	(AT)7	14	1510	1523	ACCATACGTAACGCCCAAAT
scaffold659527_13.1	1	p2	(AT)6	12	215	226	TGCATTCATCAATTTTTGGAG
scaffold659558_17.6	1	p2	(TC)8	16	210	225	CACTCTCTCACCTTCCATCCA
scaffold659629_16.5	1	p2	(CT)6	12	207	218	AGCCTCCCTCTCTTGCTCTC
scaffold659662_17.1	1	p2	(TA)6	12	193	204	CGAAGAGCACACACTTTTGG
scaffold659736_14.7	1	p2	(TA)7	14	416	429	GATCTCGAGCTCAAATCCCA
scaffold659753_12.8	1	p2	(TC)6	12	203	214	AAAGTTTTCCGCATGAATGG
scaffold659805_15.6	1	p2	(GT)8	16	1118	1133	AAGGCACTCTGGGCTTGTAA
scaffold659937_10.3	1	p2	(TG)8	16	762	777	TCTGTTTCCCCACACAAACA
scaffold660036_15.7	1	p2	(CT)7	14	168	181	GTGGGTCGTAGCCTGACTGT
scaffold660056_18.0	1	p2	(AG)6	12	2900	2911	TTAGTGCCGAAATCGTAGGG
scaffold660074_12.4	1	p2	(AT)8	16	467	482	AAATTAATCAACGATGGGCG
scaffold660140_19.0	1	p2	(TA)6	12	496	507	AGATCGACGGTCCGAAATTA
scaffold660174_16.8	1	p2	(TA)7	14	335	348	TCAGAGTGGCCATAGCAGAA
scaffold660202_12.7	1	p2	(AT)10	20	494	513	CCGAAAATCTAAAGCCCACA
scaffold660241_14.5	1	p2	(AC)6	12	2974	2985	GTTCGATGGACCTCGATCAT
scaffold660258_15.7	1	p2	(TC)6	12	1240	1251	AGCAGAATGCAGTTGTTGGA

scaffold660285_15.2	1	p2	(TA)6	12	80	91	GGAAATAAGGAGGTGTTATGATGTG
scaffold660330_14.5	1	p2	(AT)12	24	1748	1771	ATGAACTGCTAGAACGTGCG
scaffold660380_11.5	1	p2	(TA)6	12	59	70	TAGGCCGGTCTTTTAGCCTT
scaffold660509_14.6	1	p2	(CT)6	12	172	183	TTCAGCATTGTAAGGGGGAC
scaffold660520_17.5	1	p2	(TA)6	12	89	100	TTGCACCAATAAAATTTGGC
scaffold660551_15.9	1	p2	(TC)9	18	5572	5589	ACAGGGGATTGGTAATGCAA
scaffold660572_14.8	1	p2	(TA)6	12	341	352	ATTCCGAATATCAAAGGCCC
scaffold660599_15.8	1	p2	(CT)8	16	772	787	GTCACACAAGCTTGACTCGG
scaffold660640_10.5	1	p2	(TA)6	12	530	541	CGTAAATTGTTGCAACCGAG
scaffold660778_13.6	1	p2	(TG)6	12	644	655	GGTTTTGGATTTTGAGGGGT
scaffold660792_10.9	1	p2	(TA)8	16	141	156	AGAGCCCTTCAATGGACAAT
scaffold660953_19.2	1	p2	(AT)8	16	163	178	AACGGTACGTTCGGATTTTG
scaffold660956_11.4	1	p2	(CA)6	12	692	703	GCCCAACTTATTTGGATATTTGA
scaffold660967_11.0	1	p2	(AT)10	20	315	334	CCCAGTTAAGAGCGTTGGAG
scaffold660979_10.4	1	p2	(AT)10	20	513	532	CACTTCTCGGAACGAACAAA
scaffold661005_11.7	1	p2	(TA)7	14	312	325	TGCTCGTTTTTAACCCGATT
scaffold661093_10.2	1	p2	(AC)8	16	525	540	GGGCTCTATGGATGTGGCTA
scaffold661135_14.4	1	p2	(AT)9	18	1035	1052	CGTGTCATCCAAACAAATGC
scaffold661140_14.6	1	p2	(TC)6	12	521	532	ACCAGAGTCCACCACCACTC
scaffold661177_12.1	1	p2	(TA)7	14	338	351	TTGGTCTGATCTGTCCTACCC
scaffold661193_10.5	1	p2	(TA)7	14	421	434	ACCTTCCTTTCCGTTCCTGT
scaffold661212_18.5	1	p2	(AT)7	14	371	384	CTCCCAAATTTAGCCGATTG
scaffold661257_13.6	1	p2	(TA)10	20	417	436	GCCACACATGTCAGAAATGAA
scaffold661277_15.6	1	p2	(TC)8	16	1647	1662	ATTTCTGCTCACTGCCTCGT
scaffold661349_15.1	1	p2	(CT)6	12	2951	2962	CCAAATTAAGGTCATATTGCACA
scaffold661367_14.4	1	p2	(CT)8	16	1011	1026	GCGAAGTCAAACCACCAGAT
scaffold661368_13.9	1	p2	(GA)7	14	942	955	CTTCGCCCTTGGATGAAATA
scaffold661496_19.7	1	p2	(AT)9	18	640	657	CGAATAGTAGCTTCTGCGGG
scaffold661542_12.5	1	p2	(TC)8	16	429	444	ACTTTTGCGCACACAGTCAC
scaffold661555_16.9	1	p2	(TC)9	18	317	334	TTCAAATCCCACAAACGTCA
scaffold661572_12.2	1	p2	(TA)10	20	142	161	ACGCCCCAGATTGTTTCTTA
scaffold661591_15.0	1	p2	(GT)9	18	2562	2579	CACTGGTCCGAGTTCTCCAT
scaffold661625_15.2	1	p2	(TA)6	12	217	228	TGAGGAAGTTGATTTGTTGGG
scaffold661702_11.5	1	p2	(CT)7	14	215	228	CGCCGCTTTTTACGATACTT
scaffold661748_17.8	1	p2	(AT)6	12	164	175	CGAACTAAAGAAATAGGGACGC
scaffold661831_17.2	1	p2	(TC)9	18	3161	3178	CTGATAGGCCAACTGTGCAA
scaffold661849_18.4	1	p2	(AG)6	12	427	438	TCATGGGGAATTCAAAGAGG
scaffold661862_13.1	1	p2	(GC)8	16	333	348	ACGTGTGCTGACTTGTCCAC
scaffold661917_17.5	1	p2	(AG)8	16	213	228	TCGACAAAGAAATCATCGGA
scaffold661993_10.4	1	p2	(TA)6	12	65	76	GGTTATCACCTGATTTTCTCCC
scaffold662039_16.0	1	p2	(AC)8	16	212	227	GCAAATATGATATGCCTGCAA
scaffold662040_15.2	1	p2	(AT)7	14	214	227	CAAGGGTAATCCCAAACCTT
scaffold662046_17.4	1	p2	(TA)7	14	188	201	AACCACAAAAAGGATAAGTGGG
scaffold662102_10.4	1	p2	(TA)11	22	406	427	TGAAACGGACAACAAACGAA
scaffold662152_15.9	1	p2	(GA)7	14	5827	5840	TTGCTCTTGCTCCAACACAC
scaffold662159_16.1	1	p2	(TG)8	16	1100	1115	CGTTTTCAGACAAGCCCATT
scaffold662316_11.3	1	p2	(AG)11	22	594	615	GCTGGCAGCTTCAACAGATA
scaffold662321_18.0	1	p2	(AT)6	12	66	77	GGTTTTGATGGTTGTGATTTGTT
scaffold662371_11.3	1	p2	(AG)8	16	51	66	AACCTTGGAGTCCCTTCTCC
scaffold662396_13.7	1	p2	(TA)11	22	817	838	TGGACCCCCGATTTATAGGT
scaffold662403_14.2	1	p2	(TA)7	14	535	548	CCGAAACGTGAATACCCCTA
scaffold662426_14.2	1	p2	(TC)8	16	845	860	ATGGCTGTTGGCTTAATTGG
scaffold662496_17.9	1	p2	(AG)7	14	994	1007	GCTTTACACTCCCCTCCTCC
scaffold662571_14.5	1	p2	(GT)6	12	217	228	CGCGTTTAGAAAAGGGAAGA
scaffold662579_14.5	1	p2	(TA)6	12	76	87	TTTATCGTGGTGCATGTTTTT
scaffold662648_16.9	1	p2	(AC)6	12	3585	3596	AACTCCCAAGTTTGCGTTTG
scaffold662656_14.6	1	p2	(AT)9	18	525	542	TGTGAATTTTCAGATTGTGGTTTT
scaffold662674_14.9	1	p2	(AG)6	12	454	465	CCTTCTCAAAGACCGTCTGC

scaffold662838_14.(	1 p2	(GT)6	12	282	293	ACATTAAATGCAGCCGCTCT
scaffold662895_16.4	1 p2	(TC)6	12	330	341	GCAAATGGTTCACATGGTTG
scaffold662904_11.(	1 p2	(AT)10	20	259	278	ACCTCGGCACATTTACCAAA
scaffold662955_14.4	1 p2	(AT)8	16	580	595	CAGTTTTGCTATCGTTTATCTTCG
scaffold662973_16.3	1 p2	(CT)7	14	283	296	ATTATTTTGCGGTTCCAACG
scaffold662997_17.4	1 p2	(AT)7	14	310	323	TCCAGCCCTAATATTGCACA
scaffold663009_14.1	1 p2	(GT)7	14	1382	1395	AAACAGGGGTACAAGGGTGA
scaffold663218_17.2	1 p2	(AG)6	12	2039	2050	TTTTAACAATGCTGCACTCCC
scaffold663350_18.1	1 p2	(TG)10	20	38	57	TGGTTTTGAAAGTGTGATCTGG
scaffold663359_11.4	1 p2	(TA)7	14	351	364	AACGGGAGAATCGTGTCATC
scaffold663399_12.9	1 p2	(AT)9	18	64	81	TGGATTATCCTGCGGTCAGT
scaffold663403_13.9	1 p2	(TG)6	12	484	495	TCCTCAAGATTGTGCAACCA
scaffold663406_17.1	1 p2	(TG)7	14	3357	3370	GGCCATCTATCCCTATCCGT
scaffold663423_10.8	1 p2	(TC)6	12	347	358	GAGACGCGCAAAAGTAGGAC
scaffold663438_15.2	1 p2	(TC)7	14	3286	3299	TGTCCTCCTTTCCTCTGCAT
scaffold663450_18.(	1 p2	(GA)7	14	162	175	AATGATAAATCGCGGGTTTG
scaffold663537_13.6	1 p2	(CA)6	12	1026	1037	CGGTGTAAAGGAGGGGTGTA
scaffold663564_13.3	1 p2	(AG)8	16	91	106	ACTTCTTCCCAAGGCACTCA
scaffold663582_10.6	1 p2	(AT)6	12	265	276	TGAATCAATTTAACATCATGCAAT
scaffold663786_15.2	1 p2	(AG)11	22	114	135	TGTGAGAGAGTGCGTGTGTG
scaffold663797_18.3	1 p2	(AG)8	16	983	998	TTCCTTGCACTGGGAAAAAG
scaffold663905_10.5	1 p2	(AG)8	16	218	233	ACATCAACCGTTCACCACAA
scaffold663921_10.9	1 p2	(AG)7	14	286	299	GTAGAGGGAGTTGGGGGTGT
scaffold663933_15.8	1 p2	(TA)6	12	219	230	TTATTGAGCGCCATTGATGA
scaffold663948_12.7	1 p2	(TA)7	14	54	67	ACTTTTAAGGATCAAGGATATTCG
scaffold664014_15.7	1 p2	(TA)7	14	217	230	TTGGTCTAAATTGCCCCGTA
scaffold664105_11.9	1 p2	(AG)7	14	345	358	AGGGGTGGAGGTAAGAGGAA
scaffold664154_13.5	1 p2	(CT)13	26	809	834	ATCCACGCTGCAGAGAAACT
scaffold664159_12.8	1 p2	(TC)6	12	531	542	TGACCAATAGGATGTTGCCA
scaffold664160_14.2	1 p2	(CA)10	20	6773	6792	CCATGAATGGAAAATGGAGG
scaffold664189_13.1	1 p2	(TA)7	14	889	902	AAAAGTCACGCTTGAACCAT
scaffold664247_13.3	1 p2	(AG)7	14	1170	1183	CCAATGGGTGACCAGTCTTT
scaffold664315_13.1	1 p2	(AT)6	12	169	180	GTTCGAACCGAACCATGAAC
scaffold664326_11.4	1 p2	(TC)7	14	1407	1420	ATTGCCATTACCATTCCCAA
scaffold664330_16.9	1 p2	(TG)6	12	734	745	GATTTTTGGAGGGTTCTTGC
scaffold664383_17.4	1 p2	(TG)8	16	35	50	CAGGAGACGCTCATTCATCA
scaffold664386_14.7	1 p2	(AT)9	18	198	215	CATCCACTCTGAAGTTTGAACAG
scaffold664387_15.8	1 p2	(AT)6	12	1567	1578	GGACCAGGATTAGCCCAACT
scaffold664457_13.4	1 p2	(AT)8	16	1576	1591	TCATAAGGCCCACTTCAACC
scaffold664496_16.5	1 p2	(GA)7	14	725	738	GGGGTTTCCCTTACCTTTTG
scaffold664502_15.9	1 p2	(AG)8	16	5903	5918	TTTTCGGGAATCTTTCATGC
scaffold664615_11.7	1 p2	(AG)8	16	280	295	CAAAAAGGTCCCAGGTTCAA
scaffold664631_16.8	1 p2	(AT)9	18	214	231	GGGATTGAACGCACAAAACT
scaffold664766_20.5	1 p2	(AT)6	12	1492	1503	AAATGAACGTAGCAAAGGGG
scaffold664869_14.1	1 p2	(TC)6	12	46	57	TCTCTCTATCTTTTTGGTTGGTGA
scaffold664897_18.6	1 p2	(GA)6	12	154	165	CGGACCGTGAAAGCTCTAAG
scaffold664907_17.(	1 p2	(AG)6	12	359	370	ACTAATGGATATGCGGCGAG
scaffold664910_16.3	1 p2	(GA)6	12	1241	1252	CAGATTAGGAGATTTGGGCG
scaffold664920_18.6	1 p2	(TA)8	16	94	109	GCCGGATTTTGTCCCTATTT
scaffold664926_14.2	1 p2	(CT)6	12	219	230	TTTGTGTCCCTGAAAGAGGTG
scaffold664948_14.8	1 p2	(AT)8	16	215	230	CACGACAAGAATCCAGCTCA
scaffold665028_13.(	1 p2	(AT)8	16	1230	1245	GCCGAGAAGAGGAAGTGTTG
scaffold665075_14.5	1 p2	(TA)7	14	1305	1318	AGATTACGAGAAGGAGGCGG
scaffold665117_15.2	1 p2	(TA)7	14	366	379	TGACACGTGGCGTAATCTTC
scaffold665132_10.5	1 p2	(TC)8	16	167	182	AGAAGACACTTGGGCCTGAA
scaffold665149_15.7	1 p2	(CT)8	16	2361	2376	TGGTTCGCATTTCAATCTCA
scaffold665165_10.7	1 p2	(TA)8	16	278	293	AAAGGCTGCATGAGAGAGGA
scaffold665219_10.7	1 p2	(AC)6	12	262	273	AATGATCCGTTTTTGCCAAG

scaffold665336_18.[illegible]	1	p2	(GA)8	16	981	996	TGCTCGAAAGTTGCAACAGA
scaffold665356_15.[illegible]	1	p2	(AC)7	14	392	405	TCCAGCCCATTCTTATTTGG
scaffold665372_14.[illegible]	1	p2	(CT)6	12	1058	1069	GACGTTGAAAGGAGAGCGAC
scaffold665397_11.[illegible]	1	p2	(TA)9	18	331	348	TATCCAGCAGCAACATCTCG
scaffold665399_11.[illegible]	1	p2	(AT)7	14	116	129	AAGGAGGTGTTATGAAGGGAG
scaffold665474_15.[illegible]	1	p2	(CT)8	16	588	603	TGAAATCCTTTTCGATTCCG
scaffold665507_14.[illegible]	1	p2	(TC)9	18	819	836	CCAAACCCAGTACCAGCATT
scaffold665527_17.[illegible]	1	p2	(GA)6	12	2165	2176	CGTCAATCCAGAAGCACTGA
scaffold665568_16.[illegible]	1	p2	(AG)7	14	73	86	ACGTGGGTTTACTCAGAGCG
scaffold665596_16.[illegible]	1	p2	(AC)7	14	715	728	CGCAGTAATAGCATGCCAAA
scaffold665636_14.[illegible]	1	p2	(TC)6	12	293	304	CCTTTCTTCCGACTGCAAAA
scaffold665696_16.[illegible]	1	p2	(AG)9	18	341	358	CCACCACTCGAAATATGGCT
scaffold665728_15.[illegible]	1	p2	(GA)6	12	1929	1940	ACCGTGGCATCGAAGATTAG
scaffold665858_15.[illegible]	1	p2	(AT)6	12	220	231	AAACCAATGCGAGGTAGGTG
scaffold665866_12.[illegible]	1	p2	(TA)8	16	49	64	TTTTTAAGTTATCGGTGATGACACA
scaffold665915_18.[illegible]	1	p2	(GA)9	18	4226	4243	GGTCATGGACGATAGCTGGT
scaffold665930_15.[illegible]	1	p2	(TA)7	14	178	191	CACCCCGACGTAGATTGAGT
scaffold665969_11.[illegible]	1	p2	(TA)10	20	44	63	GCGTTTTGATGATATATATGTGTTTTG
scaffold665982_15.[illegible]	1	p2	(CT)8	16	211	226	GTATCGCTGCAAAAAGCACA
scaffold666110_12.[illegible]	1	p2	(AT)7	14	707	720	TCCACCCAAAAACCGAATAA
scaffold666116_11.[illegible]	1	p2	(AT)6	12	157	168	CTTTGTGAATGAAATGGTTGCT
scaffold666151_12.[illegible]	1	p2	(AG)6	12	221	232	CACCCTCCCACACATCAAAT
scaffold666222_12.[illegible]	1	p2	(TC)6	12	304	315	TTTCCTACGACAACCCTTCG
scaffold666249_11.[illegible]	1	p2	(GT)6	12	459	470	ACTCCTCGGGACGGTTTACT
scaffold666250_15.[illegible]	1	p2	(CA)6	12	2556	2567	TGCTCATGCAGGATTAGTCG
scaffold666291_15.[illegible]	1	p2	(TC)6	12	924	935	AGCTCCGACTTTGTTGTGGT
scaffold666402_15.[illegible]	1	p2	(GA)9	18	440	457	TGCGACACCGATTAATACCA
scaffold666450_10.[illegible]	1	p2	(AT)6	12	106	117	TGGGTTTTGTAATTTGACCCA
scaffold666501_10.[illegible]	1	p2	(TA)11	22	317	338	TAACCCCGAATGCTGTTAGC
scaffold666646_13.[illegible]	1	p2	(AG)6	12	722	733	CTCAGCTCAGGGTGGAGAAA
scaffold666658_15.[illegible]	1	p2	(AT)6	12	307	318	CATAAACCCTCACTCCGAGC
scaffold666672_11.[illegible]	1	p2	(TA)8	16	573	588	GGCCAACTGAAGTTGATCGT
scaffold666700_15.[illegible]	1	p2	(TA)7	14	241	254	AAAATGCCCTTGTATGCTGC
scaffold666709_17.[illegible]	1	p2	(AC)6	12	1685	1696	AGCGAAAGACAGAGCAAAGC
scaffold666755_12.[illegible]	1	p2	(AT)9	18	370	387	AGTTTGGGGGAGGAGAAAGA
scaffold666761_13.[illegible]	1	p2	(CT)8	16	378	393	ATAAAAATGACACTCGGCGG
scaffold666816_16.[illegible]	1	p2	(TA)6	12	257	268	GGCAACCAATTTTCTCTCTGC
scaffold666849_19.[illegible]	1	p2	(AG)6	12	221	232	GGCAAACACAAAGACACGAA
scaffold666869_12.[illegible]	1	p2	(TA)7	14	495	508	CCCTTATAAATGCGCTTGAA
scaffold666887_13.[illegible]	1	p2	(AT)10	20	3071	3090	TCTTTTGCGCTTTCGAGAAT
scaffold666913_14.[illegible]	1	p2	(CA)6	12	257	268	AATGCCTTTTGTTTGCATCC
scaffold667017_13.[illegible]	1	p2	(AG)7	14	764	777	GTCCTGCCCTCTCATAGCTG
scaffold667029_17.[illegible]	1	p2	(CT)7	14	2632	2645	TTCTAGTCCACCCGACCAAG
scaffold667096_11.[illegible]	1	p2	(AG)7	14	110	123	CGTCCACAGTGGTTTGAGAG
scaffold667118_10.[illegible]	1	p2	(AT)8	16	582	597	GTCGATACGGCATGACACAC
scaffold667119_17.[illegible]	1	p2	(TC)6	12	3364	3375	AAATCTGGTGGCGATCAAAG
scaffold667137_16.[illegible]	1	p2	(AT)7	14	581	594	TCGGTCGTGGGATTTTTATC
scaffold667278_14.[illegible]	1	p2	(TA)9	18	217	234	CGGGTCGAGAGAGGAGAAAT
scaffold667292_14.[illegible]	1	p2	(TA)8	16	49	64	TATGGGTTATCACCGGATCG
scaffold667335_14.[illegible]	1	p2	(CA)7	14	1122	1135	CAACGAAAAGAGAGCCTTGC
scaffold667425_16.[illegible]	1	p2	(AT)7	14	928	941	AAATCAATGGCATCCCACAT
scaffold667434_14.[illegible]	1	p2	(AT)7	14	894	907	TGGTGCGTGAAGGAGATATG
scaffold667614_13.[illegible]	1	p2	(CT)6	12	30	41	CTCTATAGCAAGATCTCACGCAG
scaffold667616_20.[illegible]	1	p2	(GA)6	12	203	214	TGGAGGTGAAGAGAGGGAGA
scaffold667687_12.[illegible]	1	p2	(TA)6	12	160	171	ATCATCCAACACCCATCCAT
scaffold667728_10.[illegible]	1	p2	(AC)7	14	103	116	CGCACACACTCTCTGATTCAC
scaffold667853_14.[illegible]	1	p2	(AG)8	16	395	410	GCGTGGAATTCACCTCAAAT
scaffold667872_12.[illegible]	1	p2	(AG)6	12	407	418	TCCGGATTCGAATACTCACC

scaffold667876_13.4	1	p2	(GA)7	14	508	521	AGTACGGATTGATGGAACGG
scaffold667889_12.7	1	p2	(TC)7	14	782	795	TTACGCCCTCTCCATTTCAC
scaffold667971_14.4	1	p2	(TC)7	14	49	62	CGTAATGTTTTGGTGGGAAGA
scaffold668030_12.6	1	p2	(TA)6	12	69	80	ACACAACTTAGGTGTTCTCATTTG
scaffold668036_14.4	1	p2	(TA)6	12	377	388	CTTTCGTCAAACCCTGCTTC
scaffold668050_15.9	1	p2	(GT)6	12	690	701	TGAGCGCTATCATGGAGTTG
scaffold668104_13.3	1	p2	(TA)8	16	298	313	TGATTGCACCTGAATAAGCC
scaffold668212_15.4	1	p2	(AG)7	14	1115	1128	TGGATTATAGATGCACGAGGAA
scaffold668367_12.9	1	p2	(TC)6	12	88	99	AAACTTAGGGACCGCTGGAT
scaffold668371_15.6	1	p2	(TA)6	12	884	895	GAAATTTGTCGACCACACCA
scaffold668397_11.7	1	p2	(AT)9	18	719	736	CATGGACGAAAATAGCCTCA
scaffold668449_11.3	1	p2	(AC)9	18	218	235	TCAATCATGACTGCTTTGGTTC
scaffold668465_16.3	1	p2	(AC)7	14	3748	3761	CCCCCAAGATATTGCAACTG
scaffold668495_19.9	1	p2	(AG)7	14	661	674	AGCACTCGTGTGGGATTTTC
scaffold668537_16.0	1	p2	(CT)7	14	417	430	AGGCAATCTAGGCGACAATC
scaffold668553_15.0	1	p2	(TG)6	12	171	182	TCGTGTTCTTCACTCATTCCA
scaffold668562_16.4	1	p2	(TC)7	14	2757	2770	AAAAAGTGAGGAGCGGTGAA
scaffold668623_13.1	1	p2	(AT)6	12	814	825	ACACCGATCCGTTCCTACAC
scaffold668765_17.2	1	p2	(TC)6	12	238	249	CCAGCCATTCAAATCAACCT
scaffold668779_12.5	1	p2	(TC)7	14	165	178	TGATTAGGCAAGCAGTGGTG
scaffold668788_16.8	1	p2	(TG)7	14	2506	2519	CCCTCCGCACCTATTATCAA
scaffold668878_11.6	1	p2	(AT)8	16	141	156	TAAGGCAAGCAGTAAACGCC
scaffold668909_13.3	1	p2	(AT)6	12	2135	2146	GCAAGGAATCTAACCTGCGA
scaffold669015_10.3	1	p2	(GT)6	12	526	537	GTTGGCATTCAAATCAACCA
scaffold669048_16.2	1	p2	(TA)6	12	200	211	TCGGGAAGAATATGAGTGCC
scaffold669060_16.1	1	p2	(TA)6	12	2048	2059	TCACAATGGCAACTCAAAGC
scaffold669075_17.0	1	p2	(CT)9	18	3521	3538	CAACGGTCTACTCAAACGGC
scaffold669255_14.2	1	p2	(AG)6	12	437	448	GCCTTTCTTCATGCTTTCCA
scaffold669313_13.8	1	p2	(TA)6	12	570	581	TTCGTGAATGACTACCCACG
scaffold669325_15.1	1	p2	(CA)8	16	1570	1585	GCACAACGTATATTCCCACTTTC
scaffold669364_10.4	1	p2	(TA)7	14	473	486	AAGGCCAACTCAAAAGGGAG
scaffold669386_15.8	1	p2	(AC)7	14	3581	3594	TCCATCCCCTGAAATAGCTG
scaffold669405_16.1	1	p2	(TG)6	12	509	520	TGCACATTTAGGCTCTGACG
scaffold669471_16.7	1	p2	(AC)6	12	214	225	CAACGTTGCTGCGTATGATT
scaffold669491_15.7	1	p2	(AT)8	16	220	235	TACCGTGATTTCCTTCCACC
scaffold669505_12.2	1	p2	(CT)6	12	923	934	GCATTCTATCTCCTCTCCGC
scaffold669507_13.4	1	p2	(TA)8	16	316	331	CATCAACAACACCATCACCC
scaffold669749_10.2	1	p2	(TA)7	14	301	314	GACAGCATACGCGCTCATTA
scaffold669837_11.6	1	p2	(AT)8	16	173	188	GTTCGAACTGTTGCATGTGG
scaffold669859_15.4	1	p2	(TA)6	12	225	236	ACAAAAATGCCCTCGTATGC
scaffold669901_16.0	1	p2	(AG)6	12	665	676	GGTGTGCTCGAATTTTTGGT
scaffold669911_16.9	1	p2	(TA)9	18	1646	1663	GACAGCCGATATTTGCTCCT
scaffold669928_16.0	1	p2	(AT)6	12	495	506	TGTTCTCTACTATATGTCATGTTTTGC
scaffold669944_12.9	1	p2	(AG)6	12	458	469	AAGAAAACGGCTGAGCAAGA
scaffold669946_15.1	1	p2	(AT)6	12	611	622	AAAGAGCTTCCTCCCACACA
scaffold669985_15.8	1	p2	(AT)11	22	344	365	ACCTTAACTTGCATGCACCC
scaffold670069_14.9	1	p2	(TA)6	12	91	102	GGACTTGATTCCATGATAACCC
scaffold670087_12.2	1	p2	(TA)10	20	218	237	TGCATGCAAGGAAAATGAAA
scaffold670100_16.3	1	p2	(AT)6	12	291	302	AAAACCCCATATCTGTGGCA
scaffold670133_13.8	1	p2	(AT)9	18	563	580	GAACCTCGAACCGTTGAGAC
scaffold670140_16.2	1	p2	(CA)11	22	549	570	AGTCGGGCAAGTCTCCTACA
scaffold670220_13.5	1	p2	(AT)8	16	2228	2243	CATCCCGCATCATAACAGAA
scaffold670223_12.9	1	p2	(CT)6	12	468	479	AGCATGTGGTCCCTAACTCAA
scaffold670348_14.5	1	p2	(AT)6	12	1424	1435	ACGTGTGCGTGTATCCATGA
scaffold670349_11.6	1	p2	(AT)8	16	428	443	CATCCCAGTTATGTCAAGCG
scaffold670379_14.5	1	p2	(TC)6	12	1890	1901	CCTTGGATTTTGACGCCTTA
scaffold670403_15.0	1	p2	(TA)9	18	47	64	TTGGTTATCGCTTGAATGAGA
scaffold670413_15.4	1	p2	(AT)11	22	2873	2894	AGCTCCTGCTGACTGAGAGC

scaffold670447_16.(	1	p2	(GA)7	14	2392	2405	GTTTTCCTGGAAGGGATGGT
scaffold670575_15.8	1	p2	(GT)7	14	1053	1066	TCCTTGTTTTCGGATGTTCA
scaffold670587_16.9	1	p2	(TG)6	12	1592	1603	TTCAGATGCTTGTTTTAGGTTCC
scaffold670618_11.(	1	p2	(AT)9	18	792	809	AATGGCCGACACTGCTTTAC
scaffold670620_14.2	1	p2	(AT)10	20	99	118	TTGTAAAAACTTCCTATACATAACGC/
scaffold670635_15.3	1	p2	(GT)12	24	188	211	GTCCCGGGAATGAAGAAGTT
scaffold670657_12.2	1	p2	(AC)6	12	227	238	TCAAATGTTGATCCAGCAGC
scaffold670659_15.6	1	p2	(CT)6	12	1499	1510	CCCTCCATTTCTCAAACCCT
scaffold670749_16.1	1	p2	(AT)8	16	2482	2497	ACAAATATCAGTAAAAATGCGCT
scaffold670903_14.3	1	p2	(TC)8	16	222	237	CCATTTGTTTCAGCTCGTGA
scaffold670924_11.2	1	p2	(AT)10	20	875	894	ACTTGGACCGTGAACACCTC
scaffold671009_17.4	1	p2	(CT)6	12	123	134	AACACAAGGAACTTGGGACG
scaffold671066_12.4	1	p2	(AT)6	12	122	133	CCCAATTCTCCATCATTATTATTC
scaffold671147_17.3	1	p2	(CT)6	12	78	89	CCACTCCATCACCATCACTG
scaffold671158_17.1	1	p2	(GA)6	12	733	744	CCGAATCTCGAGCCTTAACA
scaffold671163_13.2	1	p2	(TC)8	16	560	575	GTTGAGATTTGGACGGGAGA
scaffold671215_11.1	1	p2	(TA)6	12	443	454	CCCCTACTTCCTCGTTCTCC
scaffold671221_16.2	1	p2	(TA)6	12	1334	1345	TGCAAGATTTCAAAATCGCAT
scaffold671300_16.4	1	p2	(CT)9	18	1043	1060	TGAGGCAAGAAAAGGTTTGTG
scaffold671303_14.(	1	p2	(AG)6	12	304	315	TCGGCCAAGAAGAGAGAAAA
scaffold671367_16.1	1	p2	(CT)6	12	99	110	TCACATACTCATGCAAGCACA
scaffold671420_12.3	1	p2	(TA)7	14	376	389	TCCATCGTGAAAAACCTTCA
scaffold671445_19.1	1	p2	(TC)6	12	690	701	GAAGCATGCGAGACATTTCA
scaffold671534_17.2	1	p2	(AT)6	12	3132	3143	ACGCAATCGATCTTCCATTC
scaffold671580_10.3	1	p2	(GA)6	12	95	106	TCATGGTTACGAACCTCCAA
scaffold671623_13.5	1	p2	(TA)8	16	223	238	GTAATCTGTACCGTCCGCGT
scaffold671650_15.6	1	p2	(TA)8	16	1278	1293	CCACTTGACATTTTTGCCCT
scaffold671681_16.8	1	p2	(TC)6	12	502	513	CTCCTCAACACCACCACCTT
scaffold671694_11.6	1	p2	(AT)7	14	369	382	ACCCTAATTGTTTCGGGCTT
scaffold671706_18.1	1	p2	(TA)8	16	105	120	TCATTCCAACTTTGTAATCAAGAAA
scaffold671713_11.(	1	p2	(GA)7	14	56	69	GTGATGATAATAGGCGGCGT
scaffold671745_17.8	1	p2	(TA)9	18	84	101	CCCAAGACCCCAATTTGATT
scaffold671852_15.2	1	p2	(AT)8	16	2673	2688	GCAGTGCAACGCATAACAGT
scaffold671918_10.(	1	p2	(TG)7	14	152	165	TGAATTGGCGTGAGTGTGTT
scaffold671943_13.1	1	p2	(AG)6	12	2757	2768	ACGATGCCTATGATTCTCCG
scaffold671978_17.7	1	p2	(AT)8	16	556	571	ATCAGGGACCCAACAACAAA
scaffold672030_15.6	1	p2	(TG)6	12	644	655	TGTCATGGCAACAAACCCTA
scaffold672054_14.4	1	p2	(AT)6	12	795	806	ATACCGTACCCTTCGCACC
scaffold672080_17.3	1	p2	(CT)7	14	71	84	CTCCCTTGAGCAAAGTGGAC
scaffold672088_16.1	1	p2	(TC)7	14	1809	1822	GGAGAAGTGCTGAAGGCAAC
scaffold672101_14.4	1	p2	(TA)6	12	302	313	AAAATCCAACCATTCACACG
scaffold672112_13.7	1	p2	(AT)6	12	524	535	TAGAGGAGGAGAAGCACCGA
scaffold672213_14.8	1	p2	(TA)6	12	1458	1469	AGCTTCCTCCACATACACCG
scaffold672220_14.7	1	p2	(TG)7	14	52	65	TTGATTGAACTACATTACAACAAGTTT
scaffold672287_15.1	1	p2	(AG)6	12	228	239	TTGGATTCCCAAGCTTTTTG
scaffold672300_15.9	1	p2	(TA)6	12	4800	4811	TGATCCATTTTGTTGGCAGA
scaffold672315_13.5	1	p2	(TA)9	18	103	120	GTCTTCCGGAGTACGAGCTG
scaffold672403_16.3	1	p2	(TA)6	12	915	926	AATCCTCGTAGCTCCCGTTT
scaffold672414_13.5	1	p2	(TA)6	12	53	64	TTCGAATGCGGTTTATCACC
scaffold672512_10.(	1	p2	(GT)8	16	320	335	CCGAATACTTTCTTTTGTAGGCTT
scaffold672515_15.(	1	p2	(AT)10	20	842	861	CAATGCAGAGATGGACGAAA
scaffold672575_18.7	1	p2	(TA)7	14	176	189	TAATGTTGCGTGGGCTTTTT
scaffold672601_16.3	1	p2	(AT)8	16	1661	1676	TGATCATCCTGGAGTAAGATTGA
scaffold672615_15.7	1	p2	(AG)8	16	149	164	AAATCAGTCTCCATCCGTGC
scaffold672721_16.6	1	p2	(AT)9	18	763	780	ACCCTTGGTCGAATTCCATA
scaffold672728_13.6	1	p2	(TG)8	16	224	239	GCGCAAAACTTCCCTTTATTC
scaffold672750_13.9	1	p2	(TC)10	20	2252	2271	GATGATGATCGGTGGAGGTC
scaffold672758_13.(	1	p2	(AG)6	12	229	240	TCCCAATCACCATTCAAGTG

scaffold672763_16.!	1	p2	(AC)6	12	43	54	CCAGGATCGAATGGAGGATA
scaffold672764_17.!	1	p2	(TA)7	14	95	108	ATTTTCGTTGGAGGAGGGTT
scaffold672768_10.!	1	p2	(TA)6	12	436	447	GGCAAGCAAGGGAGTCTATG
scaffold672816_16.!	1	p2	(GA)7	14	227	240	GATGGTAGAAGAAGCAGCGG
scaffold672890_13.!	1	p2	(AT)6	12	3678	3689	AATTCAGCAAGAATGATAAATCG
scaffold672894_17.!	1	p2	(TC)6	12	229	240	CCAAAAATTAAAGGCCCACA
scaffold672967_12.!	1	p2	(GA)6	12	3298	3309	CAAACTGTTGAAGATTGTTTACGG
scaffold673055_15.!	1	p2	(AG)8	16	372	387	AAAAGGGCACAAACAGCAAT
scaffold673080_12.!	1	p2	(AT)9	18	467	484	GTTCGCGCAAACAATGTTAC
scaffold673085_15.!	1	p2	(CT)7	14	1074	1087	TAATGGGTGTGGTGCTTTGA
scaffold673093_16.!	1	p2	(GA)7	14	401	414	CAAATTGATCCAACGACACG
scaffold673201_15.!	1	p2	(GA)6	12	492	503	GTTCATCTCCGCCTTTGTGT
scaffold673210_18.!	1	p2	(TC)6	12	353	364	TTTTCAGACACGGGAACACA
scaffold673213_16.!	1	p2	(AC)7	14	1455	1468	ATCCATCAAGTTTGCACGGT
scaffold673223_17.!	1	p2	(CT)6	12	225	236	TGGACCCAAGTATGCACAAA
scaffold673245_11.!	1	p2	(TA)8	16	518	533	ACCTAATCATGTTTTGGGCG
scaffold673261_17.!	1	p2	(TC)8	16	599	614	CACCCACCCTTCCCTTTATT
scaffold673289_15.!	1	p2	(AG)8	16	1978	1993	GCCAGGGAAATGTTATGGAA
scaffold673306_14.!	1	p2	(GA)9	18	1102	1119	AATGATGGTGGAGCTGACCT
scaffold673308_19.!	1	p2	(AT)6	12	92	103	TGGGAAGATCTCTCCGTCAT
scaffold673363_13.!	1	p2	(TC)6	12	2552	2563	ATTGGACAAGTGGCAATGTG
scaffold673388_13.!	1	p2	(AT)9	18	641	658	TCAACCCCCACGTTATCTCT
scaffold673399_13.!	1	p2	(AG)6	12	200	211	GTTTTCTGCCGAAGCAAGAG
scaffold673558_15.!	1	p2	(CT)7	14	1748	1761	GTGGAAGTGTTTTGTGGGGT
scaffold673583_12.!	1	p2	(CA)6	12	686	697	TTTTTGCCGCCTTTGATTAC
scaffold673622_20.!	1	p2	(GA)6	12	230	241	TCAAGCCAAGACAAGCAATG
scaffold673792_17.!	1	p2	(AG)8	16	742	757	TTAGGGAGGGGAGAATTTGG
scaffold673803_22.!	1	p2	(TC)9	18	225	242	TTGAAGCATGTATCACTCTGCAT
scaffold673833_15.!	1	p2	(TA)6	12	76	87	TATGGGTTATCACCGGATCG
scaffold673938_12.!	1	p2	(AG)6	12	618	629	TCTCACGACAAGAACGTGGA
scaffold674004_14.!	1	p2	(AT)8	16	227	242	TCATGATGATTACATTTGTGTTGA
scaffold674108_15.!	1	p2	(TG)6	12	832	843	GAAAGGAAACGTCGGAAACA
scaffold674199_13.!	1	p2	(AT)10	20	27	46	GAATGACACACGCACACACA
scaffold674318_15.!	1	p2	(GA)6	12	1266	1277	AAAAGCCATCAAAACCAACG
scaffold674382_15.!	1	p2	(TC)7	14	2094	2107	CTCTTCCTTCTGCGGTGTTC
scaffold674407_14.!	1	p2	(AT)7	14	454	467	CCAAAATCAACGTCCCAGAT
scaffold674410_15.!	1	p2	(CT)14	28	526	553	CCCGACAACTATGTTCCCTG
scaffold674435_15.!	1	p2	(CT)6	12	230	241	TTCATGGCTTTCTGGGTCTC
scaffold674450_10.!	1	p2	(AT)7	14	125	138	TGTATGCTGTAAATGAAACTGCTG
scaffold674464_13.!	1	p2	(CA)6	12	1126	1137	CACTGGCAAAAATGAATCTCTC
scaffold674517_12.!	1	p2	(AG)10	20	600	619	GAAGGGTGAGGCTAGGGAAG
scaffold674598_14.!	1	p2	(AG)12	24	4580	4603	GGGCCTTAGTCCATGTCCTT
scaffold674645_14.!	1	p2	(AT)6	12	1709	1720	TGACCAACTCCATGTTTCCA
scaffold674703_14.!	1	p2	(AT)6	12	774	785	TCAATTAAAAACTTGCTCTCAATCA
scaffold674882_14.!	1	p2	(TG)7	14	326	339	GTTTCTGCATTTTGGGAAGC
scaffold674895_10.!	1	p2	(TA)8	16	332	347	TTCAGTAGCACAACCACCCT
scaffold674957_14.!	1	p2	(AT)8	16	933	948	TGCTATTTTCAGCCCAAACC
scaffold675038_10.!	1	p2	(AT)13	26	542	567	GTTGCTATTGCGGGCTTTAC
scaffold675057_10.!	1	p2	(TA)8	16	196	211	ATGGGATATGAGGGGAATGC
scaffold675064_15.!	1	p2	(AT)6	12	444	455	TTTGAAGTCAAGGGAGCAAA
scaffold675076_15.!	1	p2	(TA)8	16	228	243	GCGGATGCTCTTAGTTACGC
scaffold675153_14.!	1	p2	(GA)8	16	755	770	TACCTCCCCAATCAATCCAG
scaffold675158_10.!	1	p2	(CT)6	12	289	300	AATGCCCCATCTTCAGTTTG
scaffold675207_21.!	1	p2	(AG)7	14	104	117	TTATCATGCAAATGAACGGG
scaffold675235_13.!	1	p2	(AG)7	14	54	67	AAAGATGGCCAAAACTGTGC
scaffold675279_15.!	1	p2	(GA)6	12	974	985	ACGTTGCAAGGTCTACCACC
scaffold675328_10.!	1	p2	(TA)9	18	44	61	TGCGTTTTGACGATATATAGTGTTT
scaffold675375_19.!	1	p2	(TA)6	12	232	243	AGGTTGCGTACATCTGACCC

scaffold675385_13.8	1	p2	(CA)7	14	297	310	TTGCATTGACACAATCAAGTG
scaffold675419_15.9	1	p2	(TG)9	18	65	82	GGGCCGAGATTTGTTAGAGG
scaffold675490_15.8	1	p2	(CT)8	16	444	459	TATGACTTCGGCATTACCGC
scaffold675502_14.7	1	p2	(TA)7	14	230	243	CGTGTCAAAAATCTGTTCATTTG
scaffold675518_13.7	1	p2	(TG)8	16	64	79	TCAACTGGATATCAAACGCAA
scaffold675643_16.2	1	p2	(GT)6	12	2165	2176	CATCCCTCCACATTTGCTCT
scaffold675648_14.5	1	p2	(AT)8	16	178	193	TTTGAATACAATGCTTCATCCA
scaffold675862_10.0	1	p2	(AT)7	14	419	432	GAAATGCTGATGTGGCTGAA
scaffold675910_15.9	1	p2	(CT)7	14	123	136	CTTGAACATGGCCATCACAC
scaffold675919_14.7	1	p2	(GA)11	22	302	323	GTTGTAAGTGGGGGTTGGTG
scaffold675940_15.5	1	p2	(TC)7	14	198	211	ATTATTTGCATTGCTTGCCC
scaffold675984_15.3	1	p2	(GT)10	20	1011	1030	ATGTGTTGTGATGGGGGATT
scaffold675995_17.8	1	p2	(TC)6	12	2779	2790	ATGGATTGCAGAAAGATGGC
scaffold676028_13.0	1	p2	(TA)10	20	1194	1213	GAATTCTCCCAAACTGACGG
scaffold676073_14.4	1	p2	(TA)7	14	581	594	GTGTTATGCCTATCCCTATCAAGT
scaffold676155_16.0	1	p2	(AG)8	16	3993	4008	TTTCTCCTTGAGCCTCTTCAA
scaffold676194_17.4	1	p2	(AT)7	14	747	760	ATATCGCGGGCTAAGAACCT
scaffold676221_10.6	1	p2	(TA)8	16	798	813	TCCGGAGAAAATGAAGAAGAA
scaffold676277_15.7	1	p2	(GA)6	12	405	416	TGGGAACTCGAGAGGAAAGA
scaffold676368_17.9	1	p2	(GA)6	12	234	245	TGATTTTGATCCATTGCCCT
scaffold676437_11.7	1	p2	(TA)7	14	430	443	GTCGGACGATTAATCACGCT
scaffold676447_20.9	1	p2	(TA)7	14	167	180	TGCATTTGATCCCTAGAGTGG
scaffold676523_11.5	1	p2	(TG)6	12	101	112	ATTACGTCGATCCAACGGTC
scaffold676551_10.5	1	p2	(AT)9	18	48	65	TGAGTGCTAGACTATTGTAAAAACG
scaffold676572_10.0	1	p2	(CT)7	14	181	194	TTTATTCCCCACCCTTCACA
scaffold676582_13.7	1	p2	(TA)7	14	498	511	ATGATGGCGTCATGTTTTCA
scaffold676686_13.8	1	p2	(AT)6	12	847	858	CATTGATTTCATTGCATAATCTGA
scaffold676697_14.9	1	p2	(AG)6	12	1426	1437	AATCCACGAAACGGTTTGAA
scaffold676729_12.2	1	p2	(CT)6	12	1207	1218	AAACAGCCCATGTGAGAACC
scaffold676731_14.0	1	p2	(GA)6	12	288	299	AAACCAATTGGCAACGAGAG
scaffold676751_14.9	1	p2	(TA)7	14	317	330	CCGAATTTGGCTCTTTTCCT
scaffold676780_18.8	1	p2	(AT)7	14	232	245	ATCTGCTCCTCTCCTAGCCC
scaffold676820_18.2	1	p2	(AG)6	12	591	602	GAGGATTTCTTCCCCCAAAA
scaffold676823_13.7	1	p2	(TC)7	14	447	460	CTTGAATCCATGACGAGCCT
scaffold676828_16.5	1	p2	(TC)6	12	105	116	TCGGTCTCTGGGACCTTTAG
scaffold676963_16.6	1	p2	(AT)7	14	15032	15045	GTTGGGCGATTTGAAGCTAA
scaffold676972_16.7	1	p2	(CT)6	12	235	246	AGGCAATGGTTTGGTTTCAG
scaffold677015_13.4	1	p2	(TA)6	12	435	446	CTTGAGGCCCACACAAGAAG
scaffold677066_15.6	1	p2	(GA)6	12	107	118	TATGCACGTGACTTGCTTCC
scaffold677132_17.2	1	p2	(AC)7	14	1631	1644	TCCCGGAAATAGTTCCAAAAT
scaffold677153_13.3	1	p2	(AT)6	12	146	157	GTTGCATAAACCCTAGGCCA
scaffold677187_13.9	1	p2	(AC)6	12	425	436	AGGTCAGTTCCAACCCACAG
scaffold677317_16.5	1	p2	(AG)6	12	1230	1241	GGTTTCAAGTGGCAAACGAT
scaffold677320_16.6	1	p2	(AT)9	18	1493	1510	CCCTAACCCTAAAGTAAGGACCA
scaffold677391_14.9	1	p2	(AT)6	12	1638	1649	TGCACCAGAGTAAGTTGGAATG
scaffold677394_17.0	1	p2	(TG)9	18	230	247	GATGAAGATCCTGGACGGTG
scaffold677422_18.0	1	p2	(AC)6	12	346	357	TGAAATATCGAGCACCACCA
scaffold677484_11.6	1	p2	(AG)6	12	425	436	TGATCCGAGCTTGGAATAGC
scaffold677557_12.9	1	p2	(TC)6	12	1191	1202	AAACCTCAAAACCAGGCTCA
scaffold677558_12.1	1	p2	(AC)9	18	369	386	CTCCAATCATCGACCGAGTT
scaffold677562_16.3	1	p2	(AT)6	12	236	247	TCGAACACCAAACCAATTCA
scaffold677655_15.6	1	p2	(TA)6	12	222	233	TGATGGCAACCAAGAACTCA
scaffold677741_16.4	1	p2	(CT)6	12	1464	1475	AATTGCATTTGGATGCATGA
scaffold677766_17.8	1	p2	(AT)6	12	804	815	GGCTTTTCTGGAATATAAAGCG
scaffold677768_16.2	1	p2	(AG)9	18	1539	1556	TTTCTTCCATTGGCTTCACC
scaffold677773_11.7	1	p2	(AT)8	16	360	375	GGACATGGGATTGGAGAAGA
scaffold677825_17.9	1	p2	(AT)8	16	249	264	TCGAAAACCGGTTAAAGACC
scaffold677854_13.7	1	p2	(TA)8	16	536	551	TGAGAAACGAATGCACATGA

scaffold677886_12.2	1	p2	(TA)6	12	152	163	GGGCCAAAGAACAACAAAA
scaffold677929_15.9	1	p2	(AT)6	12	2360	2371	TTGTTGTACCATTGTGGAAAGA
scaffold677953_10.0	1	p2	(TA)10	20	411	430	AATGATGCTCAAACCCAAGC
scaffold677965_17.4	1	p2	(AG)6	12	1109	1120	TCCTGCATGCCCATATACAA
scaffold677968_10.9	1	p2	(CT)6	12	398	409	AGAGAGAAAATTGCATCGGG
scaffold678079_13.4	1	p2	(TA)7	14	899	912	AATTCGGAACAGACGTCATTG
scaffold678088_15.6	1	p2	(AG)6	12	383	394	AAGATCAGCATTCGGTTTGG
scaffold678114_15.3	1	p2	(CT)9	18	1347	1364	TGGGGTAATATAAAGGTTCATCC
scaffold678132_17.7	1	p2	(CT)6	12	1318	1329	CCGATTCAGATACTCCCCAA
scaffold678179_16.7	1	p2	(AT)7	14	333	346	CTCGTGCGCAAAAATTATGA
scaffold678180_15.7	1	p2	(TA)6	12	208	219	ACAAAACAGGGGACAAGGGT
scaffold678181_13.4	1	p2	(AG)6	12	125	136	TGGAGAGAGAGAAGATCGGC
scaffold678189_10.1	1	p2	(AT)8	16	119	134	CAATTGGATTAGTGACATGGCT
scaffold678309_12.8	1	p2	(TC)11	22	226	247	CACCAAAGGAAAATTGAATGG
scaffold678316_10.0	1	p2	(TG)6	12	347	358	TTCCCTCCTCATTTCCAACA
scaffold678317_10.9	1	p2	(TG)6	12	347	358	AAGGTTTTGGTGTTGGGGTT
scaffold678346_13.0	1	p2	(AG)6	12	53	64	TCATTGTTTGAAGAGTGGGAAA
scaffold678403_11.5	1	p2	(AT)7	14	1099	1112	ATTTTCTTGGCCCAACCTTT
scaffold678444_15.6	1	p2	(TA)7	14	1353	1366	CCAACCTGTATCGCTCTTTCT
scaffold678467_16.7	1	p2	(AG)8	16	746	761	GGGTCACCTTTGTGATGCTT
scaffold678511_14.6	1	p2	(CT)6	12	1240	1251	TGCAAAGATGACAGAAACAAGAA
scaffold678517_11.7	1	p2	(AT)9	18	1103	1120	TCGTTGTTCGTGTGAGCAAT
scaffold678565_13.8	1	p2	(CT)8	16	1071	1086	AAACCCTCTCAAGAACTCCAAA
scaffold678577_11.9	1	p2	(TA)10	20	384	403	CATGTTAAGGAGGGGATCCAT
scaffold678582_13.8	1	p2	(TG)6	12	53	64	TGGCTGGCGGTTTTAAATAAT
scaffold678624_10.6	1	p2	(TA)8	16	144	159	TTCGGGTCTCAACACATTCA
scaffold678701_14.6	1	p2	(TC)6	12	1009	1020	AGGGGGAAACTCGCTAATGT
scaffold678771_12.7	1	p2	(TA)6	12	499	510	TCGAAGTCCCACTTCAGCTT
scaffold678936_18.5	1	p2	(CT)6	12	730	741	AAATGTGACCGGATTCATCG
scaffold678975_15.6	1	p2	(AT)13	26	5282	5307	AGTCTGACCTTCATGGGCAC
scaffold679001_14.4	1	p2	(AT)10	20	186	205	TGTTGTGTATAAGGAAGTTTTTCG
scaffold679022_12.9	1	p2	(TA)7	14	102	115	TGAAACCTAAGTGGCCTGTCA
scaffold679105_16.3	1	p2	(GT)7	14	1691	1704	GAGGTTGATGTTGTCGGGTT
scaffold679179_15.7	1	p2	(AT)6	12	238	249	TCGATCAAAGTTATGAATTGGAAA
scaffold679241_16.8	1	p2	(GA)6	12	4194	4205	ACCGCTACTTCTCCACGAAA
scaffold679267_17.0	1	p2	(CT)6	12	184	195	GCATCTCTTGCTCTCTCGTTG
scaffold679268_13.2	1	p2	(TG)7	14	51	64	AGGTCGAGGATTGTCGTCTG
scaffold679319_14.1	1	p2	(TA)7	14	485	498	GGTGCCACGTGTCATACTCA
scaffold679377_14.8	1	p2	(AT)6	12	333	344	AAATCCTTGCACCTCCTCCT
scaffold679396_15.6	1	p2	(TC)7	14	1215	1228	GGATTGGACCTATAATATTGACCG
scaffold679420_16.5	1	p2	(TA)6	12	1055	1066	TTGCAGAATCATAAAGTGCAAAC
scaffold679436_16.6	1	p2	(TC)7	14	144	157	GGAGAACGCCATTGTTGATT
scaffold679537_17.0	1	p2	(TC)7	14	279	292	CATGGCGGATTTACAACTGA
scaffold679720_13.9	1	p2	(AT)8	16	1170	1185	TTTGCCCTCTTGTGATACCC
scaffold679746_14.3	1	p2	(GA)6	12	3884	3895	TGTAATGAAAGCAGCCGTTG
scaffold679880_15.7	1	p2	(CT)6	12	478	489	CTGTTAGCAAGTTCCACGCA
scaffold679898_11.3	1	p2	(TA)8	16	235	250	AGCGAAATTGATCTCTGCGT
scaffold679974_16.5	1	p2	(CA)12	24	1125	1148	CTGGGAAACCAATCTTGTCG
scaffold679975_14.7	1	p2	(AT)9	18	911	928	CAAAGGCCCACAGTGGTTAT
scaffold680003_11.4	1	p2	(AT)6	12	67	78	GATGATTGCACCATAACGCA
scaffold680004_14.2	1	p2	(TC)6	12	746	757	AGACCCTCAACCTCACCCTC
scaffold680063_13.1	1	p2	(TA)6	12	339	350	CAAGAGGAATTGAACCCAGG
scaffold680099_15.2	1	p2	(TC)8	16	681	696	GGCAGGAATGAGACACCAAT
scaffold680207_14.4	1	p2	(AT)6	12	1173	1184	TGGACAAACAAGCCTCACTCT
scaffold680229_18.3	1	p2	(CT)11	22	230	251	CTCGTAGAAGGGCAGTGGAG
scaffold680315_14.7	1	p2	(AG)6	12	132	143	ACCTGTCCTGGGTCTGTGAG
scaffold680338_13.5	1	p2	(GA)7	14	237	250	TGAATTCAATTGTGTTTTGGGA
scaffold680384_13.4	1	p2	(TA)6	12	74	85	ATCCAAGGGTCAATAATGGTT

scaffold680387_14.7	1	p2	(AT)8	16	170	185	TGGAGGAGTTCTTCCAGGAG
scaffold680390_20.6	1	p2	(CT)6	12	1213	1224	CGAGTATCCTCCCACTCCAA
scaffold680427_12.7	1	p2	(AT)10	20	590	609	TACCCCCAGCATCCAATTTA
scaffold680432_15.8	1	p2	(TG)6	12	748	759	AGGAGAGATTGTAACGACCCA
scaffold680492_16.7	1	p2	(TA)8	16	81	96	CATTATTTAATGTAGGGTGGGTGA
scaffold680548_13.2	1	p2	(CT)7	14	534	547	TGCTTCCCTCAATGGTTTTC
scaffold680570_17.8	1	p2	(CA)6	12	2675	2686	CTGGCTGCTCTACGCTCTCT
scaffold680574_13.2	1	p2	(AT)13	26	461	486	TTGAAGATCCCGGACAGTTC
scaffold680578_11.4	1	p2	(GA)8	16	534	549	ATGGACGGCTGTGATTTAGG
scaffold680579_16.3	1	p2	(TA)6	12	1549	1560	TTTGGATCACTAGGGCTTGG
scaffold680601_15.5	1	p2	(TA)6	12	138	149	CACGTGAGATGCTGAGTAGGA
scaffold680634_13.6	1	p2	(TC)6	12	55	66	ACAAGCCCTAGCTCTTTCCC
scaffold680635_11.1	1	p2	(TA)8	16	92	107	AGCGGTTATTACCGGATCG
scaffold680683_11.4	1	p2	(TC)6	12	950	961	CGCTACTCTTCGAGTGCGTT
scaffold680726_18.2	1	p2	(AT)8	16	733	748	ACACCGGTTCCAAGTGATCT
scaffold680734_14.2	1	p2	(TA)12	24	290	313	GGAAGGGGAGTTAGCGTATG
scaffold680885_10.4	1	p2	(CT)6	12	256	267	ATCCGATGGATGAAAAGCTG
scaffold680905_14.9	1	p2	(AC)7	14	378	391	CAATGGCTTGTGTTCCTCAA
scaffold680985_16.3	1	p2	(CT)12	24	504	527	AGCCTTTAAGACACGCCAAA
scaffold681070_19.5	1	p2	(TA)6	12	240	251	CGCGTGGGTGATATGCTAGT
scaffold681073_15.2	1	p2	(CT)12	24	1198	1221	TCCAAAGTCACACTTGCTGC
scaffold681076_14.4	1	p2	(CT)8	16	5591	5606	GAATGAGTGATGGCGGATCT
scaffold681093_15.3	1	p2	(CA)6	12	171	182	CATTAAAGCCTCACCCCTCA
scaffold681104_11.3	1	p2	(CT)8	16	54	69	ATCCTTTGCACAACCCACTC
scaffold681110_13.0	1	p2	(TC)6	12	419	430	CAAGAATTCCAAGCTTGCCT
scaffold681119_17.5	1	p2	(AT)8	16	1433	1448	GCTACATCACTTCCCCACAAA
scaffold681177_10.6	1	p2	(GA)6	12	345	356	CGAAGAAAACACCCGGACTA
scaffold681197_15.3	1	p2	(CT)8	16	2095	2110	AAGGGAGCTCCTCAGCCTAC
scaffold681208_16.5	1	p2	(TA)8	16	2474	2489	GAGTGAGTTGGCGAGAATCTG
scaffold681216_11.2	1	p2	(TA)7	14	1054	1067	TATCCCTACACCGCCTCATC
scaffold681219_16.8	1	p2	(GA)6	12	1954	1965	ACGGGAGGAAGACGAAGAGT
scaffold681242_16.3	1	p2	(TA)6	12	146	157	TTGGGATTAAGGCTTTGGTG
scaffold681244_10.3	1	p2	(TA)8	16	124	139	TCAATTCCTCCCTTAGACACAA
scaffold681257_15.3	1	p2	(CT)6	12	197	208	GCATCTCTTGCTCTCTCGTTG
scaffold681304_10.0	1	p2	(CT)8	16	183	198	TTTTGACACGTAGCGACACC
scaffold681324_14.1	1	p2	(TA)6	12	216	227	TGTGCGTTTTTGGCATAGTG
scaffold681334_18.0	1	p2	(CT)7	14	909	922	TTGCAGAGTGATTGTAGGCG
scaffold681341_13.9	1	p2	(TG)6	12	241	252	CAATAATGCCCCCTTCATGT
scaffold681470_14.9	1	p2	(GA)7	14	240	253	GCGTCTCCAAACAGGAGAGA
scaffold681503_14.8	1	p2	(GA)6	12	976	987	CGACGTCGTTCTTGATTCTTC
scaffold681544_16.0	1	p2	(AT)6	12	905	916	TACCGTTTCAAGGTGCAATG
scaffold681611_15.8	1	p2	(CT)6	12	1497	1508	GCGCTACAAAAACGAGTTCA
scaffold681640_12.7	1	p2	(TA)6	12	315	326	AGTAGTTAGCATGGAAGGGGA
scaffold681682_10.0	1	p2	(TA)7	14	320	333	TTCGAATTGATGTTTGATCCAT
scaffold681738_13.9	1	p2	(GA)8	16	1188	1203	ATCGCATTGCCTCACTTTTT
scaffold681852_15.6	1	p2	(TG)6	12	2530	2541	GATTTGGTACATGGATGGGG
scaffold681897_11.6	1	p2	(AT)9	18	528	545	TTGAGCCCTGCCAAAATATC
scaffold681915_15.0	1	p2	(AG)8	16	330	345	CGAATTTTAGTGGGACATCCA
scaffold681939_13.8	1	p2	(TC)8	16	797	812	ATCAATAGGTCACGGGTTCG
scaffold681940_21.2	1	p2	(AT)6	12	415	426	GCTATCACGCGAATGGGTAT
scaffold681966_17.6	1	p2	(TA)9	18	481	498	AGTCCTAGCCCCACATCTCA
scaffold682001_20.6	1	p2	(AT)6	12	243	254	TGCGGGCATAAGACCATATT
scaffold682014_17.3	1	p2	(CT)8	16	1064	1079	AGTGGGGAGCCTTACCTTGT
scaffold682217_12.0	1	p2	(AT)8	16	2326	2341	AGGCTGTTAAGGTTCGGGTT
scaffold682228_14.5	1	p2	(CT)7	14	240	253	AGTCTCCCCATTTGATGCAC
scaffold682235_10.2	1	p2	(GA)6	12	144	155	CTTTTGGTCGAGCCAGCTAC
scaffold682271_17.8	1	p2	(AG)6	12	242	253	GTTGCTCATTCCCCTTTCAA
scaffold682273_15.1	1	p2	(CT)10	20	594	613	CCGAGTTGGAGAGAGAATGC

scaffold682276_17.8	1	p2	(CA)8	16	432	447 CCCTGCCTGTAAATCCAGAA
scaffold682319_16.9	1	p2	(TC)6	12	2040	2051 TATTTTGATGCCGGGAATGT
scaffold682348_11.1	1	p2	(TA)6	12	1004	1015 CACGCTGATATCGCACAACT
scaffold682357_14.4	1	p2	(AG)10	20	511	530 ATCCCAGAATGCCTACACCA
scaffold682366_18.5	1	p2	(AG)6	12	97	108 TTAAATGCACAGCACAGCTC
scaffold682386_15.6	1	p2	(AT)6	12	742	753 TCAACTTATATTTCAGCGACGA
scaffold682390_16.3	1	p2	(TC)6	12	243	254 TCAACAACAGCAGAAATCGG
scaffold682445_13.6	1	p2	(TG)6	12	693	704 GAATGCTCGTTGTCCCAAAT
scaffold682519_15.0	1	p2	(AG)6	12	931	942 CTTTCACGGGTTTGAAGCTC
scaffold682541_14.9	1	p2	(TA)9	18	47	64 TTTCATATATCACGTTAAGACATGGAT
scaffold682542_17.7	1	p2	(AT)10	20	191	210 TAACCCTTGTTAGCCCGTTG
scaffold682556_15.2	1	p2	(GA)8	16	239	254 TCAGCCAAATGCAGAGATGA
scaffold682563_11.3	1	p2	(AT)6	12	191	202 GCTATAAGGTTGCACCCACG
scaffold682564_12.8	1	p2	(AT)12	24	33	56 TCAATTTCTCTAACCTGTAAAATGC
scaffold682565_10.6	1	p2	(AT)6	12	405	416 CCCATTCTCTTCATCCCTCA
scaffold682717_12.2	1	p2	(AT)6	12	420	431 AATACACCCCAAAGTGGAACC
scaffold682721_14.3	1	p2	(TA)10	20	46	65 CCAAATGTTAGGGTTATCACAGG
scaffold682783_19.7	1	p2	(CT)6	12	1126	1137 CCTTCATCCCCTGTCTTGAA
scaffold682842_15.2	1	p2	(AG)7	14	2487	2500 CTTTGAAATGACATGGGCCT
scaffold683010_12.4	1	p2	(AT)7	14	307	320 AAATGCATCATACGGCATACA
scaffold683017_13.1	1	p2	(TA)7	14	794	807 TTGATCCTTTTTATGATAACCCAA
scaffold683021_13.9	1	p2	(TA)6	12	60	71 GAAAGAGAGTTTAGATCAAGTGTGTT
scaffold683033_14.0	1	p2	(TG)7	14	227	240 TTCTTTTTACATTTGGTAGACCATC
scaffold683042_12.4	1	p2	(GC)8	16	190	205 CAATTCGAAGCCCAAATCAT
scaffold683083_15.8	1	p2	(GA)8	16	329	344 AATGGGTGGGTTATAATGGGA
scaffold683130_14.3	1	p2	(TA)9	18	344	361 CACGGGAGGTGAAAATTGAG
scaffold683136_16.5	1	p2	(AG)6	12	1377	1388 CGAGCAATATCTTGGGCACT
scaffold683191_13.5	1	p2	(AG)9	18	544	561 TCCAACCGCTATTTGAATCC
scaffold683196_13.4	1	p2	(TA)9	18	36	53 TTTAGTGTTTTGACATGATTACTCG
scaffold683201_15.2	1	p2	(TC)6	12	244	255 TGCTGCTTTAGGTCTTTGCC
scaffold683208_16.0	1	p2	(AG)7	14	5578	5591 CACGAACACATGAGGGAATG
scaffold683240_19.5	1	p2	(TA)6	12	1081	1092 GATAATGGCAAACCTGCTGC
scaffold683518_12.3	1	p2	(AG)12	24	498	521 GGGAGAGAGAAGGGAAGAGG
scaffold683521_14.5	1	p2	(AG)7	14	1192	1205 AAGAAAAGGGGGAAAGGTGA
scaffold683598_10.6	1	p2	(GA)7	14	678	691 ATTCTAGGTGCACCCCTCCT
scaffold683641_12.0	1	p2	(TG)6	12	54	65 TCCCCTTTTACTTTATTCTTTCAA
scaffold683665_12.1	1	p2	(AT)9	18	319	336 TAACGATGGACTAGGGGCTG
scaffold683682_15.1	1	p2	(TA)10	20	309	328 TCAAGGGGTATTTTTGTCCG
scaffold683718_12.9	1	p2	(TC)7	14	405	418 TGTGACTGTCGATGAAGTAAAGG
scaffold683792_14.5	1	p2	(TA)9	18	70	87 CGAACGATGACATGCTCCTA
scaffold683848_16.4	1	p2	(GA)7	14	243	256 ACTTGCAAAAGACTCCAGGC
scaffold683896_15.4	1	p2	(AG)6	12	245	256 ATTCACACCATTTGCCATCC
scaffold683909_15.1	1	p2	(AG)7	14	562	575 AATCCCCGCCTATCTAAGGA
scaffold683914_14.2	1	p2	(TA)7	14	180	193 TGGATACATGGGTGGATAATATG
scaffold684054_18.5	1	p2	(TC)8	16	515	530 ATATTCGGCTCCATCCCTTC
scaffold684061_13.8	1	p2	(GA)9	18	2304	2321 ATCTCGCAGTGCAAAATCG
scaffold684070_14.4	1	p2	(TG)6	12	571	582 TGTACAGGCATGGGTTTCAA
scaffold684076_13.3	1	p2	(GT)7	14	438	451 CAAGACCACGAGCCGTTAAT
scaffold684146_21.6	1	p2	(TC)7	14	95	108 CAAAACTCTCATACAGCCAAAA
scaffold684154_15.0	1	p2	(TG)6	12	650	661 TGTAATCTGGGATAACGGGG
scaffold684214_15.5	1	p2	(TC)7	14	3108	3121 GTAAGCATCGCCCTCCATAA
scaffold684244_14.7	1	p2	(TC)6	12	778	789 GGGGCATGAATTGTTACAGG
scaffold684247_17.4	1	p2	(AT)10	20	495	514 GGAGCACCTGATTTTCGCTA
scaffold684254_17.0	1	p2	(CT)7	14	245	258 AGGGGTTCGAGTGGAAAATC
scaffold684257_16.6	1	p2	(CT)9	18	719	736 AGAATTGGAACCCCAAAACC
scaffold684271_14.3	1	p2	(TA)8	16	705	720 AGCCCCAAATCACAAACAAC
scaffold684300_11.9	1	p2	(TA)8	16	323	338 CCCCTCGTATGCAGTCATTT
scaffold684332_13.6	1	p2	(TA)8	16	424	439 TCAGACGAAACATCCCTTCA

scaffold684366_17.7	1	p2	(AG)6	12	247	258	TGCTCCTCCTCTTCCTTTCA
scaffold684369_12.0	1	p2	(AG)8	16	413	428	TCTCGGCCAGAGAGAAGAAA
scaffold684376_17.4	1	p2	(TA)6	12	645	656	TAACCCACCAAATGCTGTGA
scaffold684408_10.7	1	p2	(AT)7	14	244	257	GCGTGAGAGATAGCCGAGAG
scaffold684409_16.7	1	p2	(AG)6	12	243	254	TTCACCTACCAATTCCAGCA
scaffold684438_15.7	1	p2	(TC)9	18	241	258	CACGCGATACCATTCAGTTTT
scaffold684483_20.0	1	p2	(TC)6	12	67	78	TTAGCTGCTGGGATTTTGCT
scaffold684488_15.0	1	p2	(TC)6	12	1314	1325	CTAGCAATGGGGTGCTTGTT
scaffold684542_12.1	1	p2	(TA)9	18	436	453	TGTTTGCTGTCATCGCTAGG
scaffold684580_15.2	1	p2	(AT)9	18	761	778	ACAAGAATGGATTGGGTTGG
scaffold684591_17.6	1	p2	(AT)6	12	707	718	TTGGATAAATGGGAGGCTTG
scaffold684693_16.9	1	p2	(TC)6	12	661	672	ATTGAATACGCGAGACCCAC
scaffold684722_15.3	1	p2	(CT)6	12	710	721	GGTCAACCTACAGCACCGAT
scaffold684780_11.1	1	p2	(TA)8	16	302	317	TCATGGGGAATGGAAAAAGA
scaffold684848_13.3	1	p2	(AT)9	18	776	793	GCCTCCTACCCCACTTCACT
scaffold684864_16.5	1	p2	(AG)6	12	1077	1088	AACTTTCAGCCAAGAGCCAA
scaffold684904_11.0	1	p2	(TA)10	20	581	600	ACGCGCGTTTTTAGAGTTTA
scaffold684910_13.3	1	p2	(TA)12	24	208	231	GTGGCTGGAGGCATACAACT
scaffold685076_14.9	1	p2	(TA)8	16	1347	1362	TTTCCGAAACTTCCAATCAA
scaffold685105_15.1	1	p2	(AT)6	12	1323	1334	GGTCCCTAAACCCAAATCGT
scaffold685114_14.0	1	p2	(AG)7	14	396	409	CATTTGGAGGTGCACACAAC
scaffold685228_13.2	1	p2	(TA)9	18	1110	1127	GGAAACCTTATGCGATTGGA
scaffold685230_15.2	1	p2	(AT)6	12	3041	3052	TGCTTGAGAGTGGGAGGAGT
scaffold685260_15.5	1	p2	(TA)6	12	249	260	CGAATACGTACGGGTGTGCT
scaffold685263_12.8	1	p2	(TC)7	14	623	636	ATGCCTTTGCAATTTCTGCT
scaffold685364_18.0	1	p2	(TA)8	16	415	430	GGCCAAATCCTAGCCACATA
scaffold685403_12.1	1	p2	(TA)6	12	456	467	CTATCAGCGCTTCTTTCCGA
scaffold685431_16.8	1	p2	(CT)8	16	561	576	ACTCGTCCTCGTTTCTTTGC
scaffold685461_16.1	1	p2	(CT)6	12	1755	1766	TAAATAGTGGCGGATCCAGG
scaffold685484_18.9	1	p2	(AT)8	16	751	766	CGCCAAGCACTCACAAACTA
scaffold685559_13.3	1	p2	(TA)13	26	40	65	AAGGGGGTTATTAGTGGATCA
scaffold685568_14.1	1	p2	(AT)8	16	1449	1464	AGGCTGGTATTTTGGCCTTC
scaffold685656_17.8	1	p2	(AT)8	16	2682	2697	TGATAATGCACTTCAATACAAGCA
scaffold685668_11.7	1	p2	(CT)6	12	78	89	CATCTCTTCTCCACCAACCG
scaffold685737_15.4	1	p2	(AG)7	14	2066	2079	ATCAAGAAATTCACCAGGCG
scaffold685751_11.3	1	p2	(TA)9	18	403	420	TACGATTTTTCGGTTTTCGG
scaffold685768_12.7	1	p2	(GA)7	14	450	463	CCATCTTCAACCCGTCACTT
scaffold685770_14.3	1	p2	(AT)7	14	1070	1083	CGACCTTGGCAATCAACTTT
scaffold685787_13.4	1	p2	(CA)6	12	38	49	CCCCCTTCTACTTTGGTCAG
scaffold685796_16.4	1	p2	(AG)6	12	3788	3799	AACGAGAAAAGACTGCTGCC
scaffold685799_10.6	1	p2	(AT)10	20	71	90	ATCGGTTATTACCGGATCGC
scaffold685838_13.5	1	p2	(AT)8	16	1092	1107	CAGGTGCGCCTTATCTCTCT
scaffold685928_11.2	1	p2	(AC)6	12	348	359	TGCATGATTTCTTCAATCTTCG
scaffold685955_10.8	1	p2	(TA)8	16	831	846	GGGAAAGCCTCAAGTCCAAT
scaffold685983_13.1	1	p2	(TA)7	14	819	832	TGTTTTGTTCGACAACGACTCT
scaffold685994_10.3	1	p2	(TC)11	22	31	52	TTTTTGTCTCACTAGTGTCGTTTTG
scaffold686016_11.8	1	p2	(AG)6	12	107	118	AGTTTTAGCGTGTCAGGCGT
scaffold686045_16.5	1	p2	(TC)6	12	108	119	AGGACACGTTGGACCTGAAG
scaffold686111_12.2	1	p2	(AT)6	12	578	589	ATCACGATATCCACATTTAGCA
scaffold686144_17.0	1	p2	(GA)9	18	869	886	TGCACAGTCCTAATCAAGCG
scaffold686176_17.3	1	p2	(AG)6	12	1263	1274	TGAAAGAATTTTTCGGCTGG
scaffold686190_14.6	1	p2	(CT)6	12	1004	1015	TTGCGAACTAGTTGTCGTCG
scaffold686197_17.0	1	p2	(TA)8	16	247	262	TCCCACTGCCCAAACTTATT
scaffold686260_13.3	1	p2	(TA)6	12	530	541	GCAATAACGCTGCAAAATCA
scaffold686315_17.7	1	p2	(TA)7	14	1693	1706	CGATAAAAATGGCACCCAGT
scaffold686332_11.3	1	p2	(AT)7	14	249	262	CGCTGCTTTCGTGTTTAGTT
scaffold686341_14.9	1	p2	(TG)12	24	818	841	GGGGGAGAGTTGGAGATCAT
scaffold686436_14.4	1	p2	(CT)8	16	430	445	CAGCGGCTTACTAGCTTCGT

scaffold686476_18.:	1	p2	(TC)6	12	153	164	CCCCCAAATCATTCAGCATA
scaffold686478_15.(	1	p2	(TA)8	16	501	516	CCCAAGTGGGATATTGTTGG
scaffold686480_16.9	1	p2	(TA)11	22	43	64	TCACATAAATAATGGTTTCTTCCTAA
scaffold686487_14.(	1	p2	(AG)6	12	579	590	AGTGCGTCAGAAGGTGTCCT
scaffold686517_12.9	1	p2	(CT)7	14	124	137	CCCCCGTGCATTAATAAAAT
scaffold686569_13.5	1	p2	(AG)7	14	351	364	GGAGGAGAGGCTCTTGGTTT
scaffold686585_17.3	1	p2	(GA)6	12	961	972	GCAGCATCGTCTGAGAGTGA
scaffold686586_13.(	1	p2	(AT)11	22	876	897	ATTTGGAAACTTGAACCCCC
scaffold686612_15.(	1	p2	(AT)6	12	997	1008	TCCACCAACCAAACATTCCT
scaffold686619_17.(	1	p2	(CT)10	20	54	73	ACCCATTTCATGTCACCTCC
scaffold686679_23.(	1	p2	(AG)6	12	252	263	GGCCACTGCGTTTGTTAGAG
scaffold686682_17.(	1	p2	(AG)6	12	251	262	ACTTTTCCTCCCCCATCATC
scaffold686683_14.9	1	p2	(GA)7	14	2722	2735	ATCTCCGTCTTTGTTGTGGC
scaffold686695_16.3	1	p2	(TC)8	16	1226	1241	CCTCTGCAGCCTAAAACCAC
scaffold686730_10.(	1	p2	(CT)6	12	229	240	GACACCTCCACACCCAATCT
scaffold686753_16.:	1	p2	(TA)7	14	249	262	GCAAGAAAGCAGTGCACCTA
scaffold686787_13.7	1	p2	(AT)8	16	248	263	TTGGCGTAGATTCCACTTGA
scaffold686802_16.2	1	p2	(TA)7	14	2933	2946	CACTCATTTGAGGATGGATTGA
scaffold686846_10.(	1	p2	(TC)6	12	153	164	GGTGCTTTCTGGTTCATCGT
scaffold686896_12.(	1	p2	(AT)8	16	500	515	TGTGGCATGGGTCAAGATAA
scaffold686935_13.2	1	p2	(AT)6	12	1276	1287	CGTCATTTTTCAATCCCAGC
scaffold686962_11.5	1	p2	(TA)6	12	32	43	ACGTCCTCTAGGTGTCGTCC
scaffold686993_15.(	1	p2	(AT)6	12	878	889	GATTCGTTGCAGAAATCCGT
scaffold687046_12.9	1	p2	(TA)6	12	2939	2950	CTCACGTGAAAATGCAAGGA
scaffold687051_17.3	1	p2	(TC)6	12	88	99	TGATTTCGCTGATTTTGGTG
scaffold687122_15.:	1	p2	(TC)9	18	735	752	CTTTCCCATGGAGTTCAGGA
scaffold687124_14.2	1	p2	(AT)6	12	65	76	GCTTAATTGTAAACCGTTGTCAT
scaffold687140_19.4	1	p2	(GA)6	12	465	476	TGGCACTCAGTTAAGGGTCC
scaffold687162_11.8	1	p2	(AT)7	14	454	467	GCGTCGTGGATCAAGGTAAT
scaffold687177_15.7	1	p2	(CT)9	18	48	65	TGAATCCTTCCTTGCTAGTCAC
scaffold687185_15.3	1	p2	(TA)9	18	498	515	CAATGTATCCAAACATGATGACG
scaffold687190_11.9	1	p2	(AT)6	12	373	384	ATCAGGCAAAAACGTGCATT
scaffold687225_15.5	1	p2	(TC)7	14	469	482	CAAAGCGGGTAAAGGAACAA
scaffold687276_13.8	1	p2	(GA)6	12	1273	1284	TCAGTGCGTGTGATTCTGTG
scaffold687337_16.3	1	p2	(AT)9	18	1253	1270	CCACTCACATGGGAATTGTT
scaffold687391_13.(	1	p2	(CA)8	16	1130	1145	TCACTAGCCGGACACATCAG
scaffold687426_16.2	1	p2	(AT)7	14	3700	3713	AGGGATGTGAATTCGTGCAT
scaffold687429_12.(	1	p2	(AG)6	12	415	426	GGCATGTCCGAGTTCTTAGC
scaffold687430_14.(	1	p2	(CA)6	12	1026	1037	TTCCATACGACATTGCAAGC
scaffold687435_16.(	1	p2	(AG)6	12	899	910	CCAGAACCCAGCTTCTTCAG
scaffold687446_10.(	1	p2	(TA)7	14	52	65	TCAACGTACCTATTTTGTTCCTAGTTT
scaffold687461_10.8	1	p2	(GC)6	12	276	287	CACACACAAGATTTGGGAGG
scaffold687466_15.9	1	p2	(GA)7	14	103	116	GGAGTTCGAGCTTCAGGAGA
scaffold687547_18.(	1	p2	(TC)6	12	366	377	GGGGATAATGACGAGACGAA
scaffold687641_12.2	1	p2	(TA)9	18	230	247	ATCCTGTCCATCACCCCATA
scaffold687693_15.:	1	p2	(TG)7	14	321	334	CAGCATACGCGAAGGGTATT
scaffold687724_16.5	1	p2	(AG)8	16	85	100	TCAATAAAATGCTTCCAATCG
scaffold687730_16.8	1	p2	(TC)9	18	1122	1139	ATCCAAGGAGCACACCTGAG
scaffold687735_12.8	1	p2	(GA)6	12	1003	1014	TGTGTGTTGTGTGTTCACGG
scaffold687752_18.:	1	p2	(TA)9	18	46	63	CCAATTTGAATGGTTATCACCTG
scaffold687769_10.4	1	p2	(AT)10	20	450	469	ATTTGGCCCTATAAAATGCG
scaffold687786_10.7	1	p2	(AT)6	12	165	176	ACCTCGCAGTCATTCCAGAC
scaffold687822_13.2	1	p2	(TA)8	16	52	67	GGAGGGGGTATAATTTGAATAGC
scaffold687843_12.(	1	p2	(AG)8	16	36	51	TTGAAATTGGATATTTTCAGGAGA
scaffold687858_14.(	1	p2	(AG)12	24	242	265	AACCTAACACCAAGGCAACG
scaffold687882_10.(	1	p2	(AT)8	16	122	137	CCTCCACCGCCTAACAATAA
scaffold688039_14.(	1	p2	(TC)6	12	544	555	CTGCTCCGATGAACCTCTGT
scaffold688055_10.:	1	p2	(TC)8	16	715	730	TGACCTCTACGAGGCATACCA

scaffold688116_13.9	1	p2	(TA)9	18	171	188	ATAGGGATTTCGGATTTCGG
scaffold688117_13.8	1	p2	(TA)8	16	1967	1982	CAACGAATTCCACACCAAAA
scaffold688192_16.1	1	p2	(TA)6	12	459	470	AAACTGAGCTCCATAGCCGA
scaffold688218_15.9	1	p2	(GA)8	16	271	286	GAAGCCCTTTCTTCCCAATC
scaffold688219_17.1	1	p2	(GT)6	12	2934	2945	TGAAACAACACGACCGGATA
scaffold688244_15.8	1	p2	(AT)6	12	116	127	CGGATGAATTTCACTTCACG
scaffold688277_17.3	1	p2	(AC)6	12	914	925	GTGTCGATGCCTGAAATCCT
scaffold688303_15.4	1	p2	(TC)6	12	358	369	ATGGCGAAATCACCAGAAAC
scaffold688304_18.5	1	p2	(TG)7	14	31	44	CCGTCAGTTTCAAGACATTCC
scaffold688334_19.2	1	p2	(TC)9	18	249	266	AATCCCCCAAACCAATTTCT
scaffold688344_18.3	1	p2	(AT)6	12	255	266	CATTCAGTGGGCACATTGTC
scaffold688347_16.9	1	p2	(GA)7	14	2000	2013	TGCAGAGAAGGGAAGAGGAA
scaffold688373_11.5	1	p2	(TG)6	12	367	378	CGGTTCAACTTCCCATGTTT
scaffold688374_11.8	1	p2	(AC)9	18	447	464	TTGACATTGAATCCCCTCATT
scaffold688474_11.5	1	p2	(AG)6	12	1613	1624	CTTGCTTGCTGAGGTAAGGC
scaffold688520_15.7	1	p2	(AG)9	18	2153	2170	AAGTCCCAGTGAATGATGCC
scaffold688548_13.0	1	p2	(AT)7	14	252	265	TAATGGCGCCGCTAATGTA
scaffold688555_10.3	1	p2	(AT)11	22	576	597	ACCCCTACACGCAACTCAAC
scaffold688635_11.8	1	p2	(AT)7	14	765	778	CCACATAAGCGTCATCTCAAAA
scaffold688663_15.3	1	p2	(TA)14	28	35	62	GGGCTGCAGGAATTCAATAA
scaffold688700_13.6	1	p2	(AT)9	18	1061	1078	TTTTGCCATCATAAACTCTATTCG
scaffold688722_17.4	1	p2	(AT)6	12	136	147	CCATTTCAGCCAAGCTGTTT
scaffold688807_16.0	1	p2	(CA)6	12	1163	1174	TCGGAAGGTTTATCCTCAGTG
scaffold688810_16.9	1	p2	(TC)6	12	452	463	TAAGCAAAGCGTGCACAATC
scaffold688844_16.8	1	p2	(CT)10	20	792	811	CCACTTTCCACAATCCTCGT
scaffold688857_15.3	1	p2	(TG)6	12	45	56	TCCTCTCCTCTCTCTCTACTCTCTC
scaffold688943_16.4	1	p2	(TA)6	12	477	488	GGGTGGGTCTCCCTTACATT
scaffold688977_13.8	1	p2	(TA)6	12	2434	2445	TACCCTCCGGTACACGAAAA
scaffold689002_13.6	1	p2	(AT)8	16	422	437	TATTTGACCGTGAATTGGCA
scaffold689015_16.4	1	p2	(CT)8	16	1505	1520	TCGCAACTTCACCTTTACCC
scaffold689048_17.1	1	p2	(AC)6	12	743	754	ATGTGGAACCAAACGATCAA
scaffold689068_13.3	1	p2	(AT)10	20	216	235	TTGGAATAGGTCCTTGGCTG
scaffold689071_16.0	1	p2	(TA)7	14	49	62	TTTAGTGTTTTGACATGATTACTCG
scaffold689075_11.2	1	p2	(AT)9	18	193	210	GGGTTACCAAATTAGTTCGTCG
scaffold689115_16.1	1	p2	(CT)7	14	253	266	TTTTCTTTTCGCTGCATGTG
scaffold689128_11.2	1	p2	(TA)7	14	78	91	ATAAGCCACCCAAACAATGC
scaffold689143_15.5	1	p2	(TG)10	20	3236	3255	GGATCATCTTCCCTCTCCCT
scaffold689173_15.6	1	p2	(AG)7	14	1647	1660	CAAGGCAAGGACCAGATCAT
scaffold689281_16.5	1	p2	(AT)8	16	1953	1968	AAGGAGGTGTTATGAAGGGATT
scaffold689286_17.2	1	p2	(TA)10	20	248	267	TCCCAAACTGATGGAAGGTT
scaffold689290_19.4	1	p2	(AT)7	14	109	122	TGCAAAAGTTATGGTAACAAGATT
scaffold689296_11.9	1	p2	(TA)7	14	933	946	GAGTTAATCATGCGCGTCAA
scaffold689432_14.9	1	p2	(TA)11	22	48	69	TGGTGAATGATTGACGATGAA
scaffold689502_18.4	1	p2	(TA)6	12	74	85	ATCAATGGTCCACATCGGTT
scaffold689544_15.2	1	p2	(CT)6	12	56	67	TCTCTCTTGGTTGGTGAAGGA
scaffold689559_13.0	1	p2	(AG)6	12	508	519	CGTGGTTGGTGTGAGATTTG
scaffold689571_15.4	1	p2	(TC)6	12	1736	1747	TTACGATTCACCAATTCGCA
scaffold689606_15.5	1	p2	(GA)7	14	1406	1419	GACGACGTGAAGAGACGACA
scaffold689646_11.9	1	p2	(GA)6	12	551	562	GTAGTCGGCAGAACCCAGAA
scaffold689685_20.1	1	p2	(CT)7	14	1074	1087	ATCACTGCATTTCGTCCTCC
scaffold689694_10.0	1	p2	(TA)9	18	154	171	TGCCTTTATTTTAGCCTCCC
scaffold689704_16.7	1	p2	(TC)6	12	2235	2246	ATCCTCACATCGCCAAAAAC
scaffold689732_10.5	1	p2	(CT)8	16	52	67	ACCTCATTTTCAACACCCCA
scaffold689793_13.8	1	p2	(GC)7	14	214	227	GCTTCTTTGCTACGAGCCAC
scaffold689905_18.9	1	p2	(AT)9	18	864	881	AAACCGGGCCTACAAGATTT
scaffold689910_17.3	1	p2	(CT)7	14	503	516	ACAAACAGCCCAAGGAAATG
scaffold689943_13.8	1	p2	(CT)10	20	568	587	AGGAACAGAGCTTCAACGGA
scaffold690035_15.9	1	p2	(TC)8	16	855	870	AAACAATTGGGATGAGCAGG

scaffold690100_14.2	1	p2	(AT)10	20	956	975	CAATGGTGGCCGAAAATTAG
scaffold690112_15.6	1	p2	(CA)8	16	180	195	CTAACCCTAGGTGGTGCCAA
scaffold690133_14.5	1	p2	(TA)9	18	253	270	CCCAGGCATAGAAAGAAAAGAA
scaffold690165_14.7	1	p2	(AT)12	24	1977	2000	CGTCGGTGTATGACCTGTTG
scaffold690190_14.5	1	p2	(AG)7	14	449	462	AACTTGCACCCAAATTCCAC
scaffold690206_14.1	1	p2	(TA)8	16	1194	1209	CAGAACGCTCTTTTGCACAC
scaffold690233_12.7	1	p2	(TA)6	12	397	408	TATCAGTGACCACCCAACCA
scaffold690316_14.9	1	p2	(GA)7	14	257	270	CTAAACCCAGGTGGCAGTGT
scaffold690355_17.0	1	p2	(AG)6	12	532	543	CTGGGAGTGCTGGAGAAGAC
scaffold690369_11.6	1	p2	(TA)8	16	122	137	CGGATCGGAATTTCTCAAAG
scaffold690396_15.8	1	p2	(AT)6	12	576	587	GGGGAAATAAAAGCCCAAAA
scaffold690418_17.1	1	p2	(CT)6	12	256	267	TAGAGGATTCGGCATTGGAG
scaffold690458_18.4	1	p2	(AG)6	12	513	524	GTTGGAAAAAGCCAATCGAG
scaffold690482_14.0	1	p2	(GA)8	16	707	722	TTCCAGGAGCCAGATAGGAC
scaffold690506_15.5	1	p2	(TC)6	12	224	235	CGAAGCCAAACCTAGGAAGG
scaffold690519_15.6	1	p2	(GA)6	12	565	576	TCCATCCACCTCTGCTTTTC
scaffold690523_13.9	1	p2	(AT)9	18	465	482	ATTTGTCCAATGGTGCTTCG
scaffold690546_13.2	1	p2	(GA)6	12	756	767	GGAACCTGAGAATGGGGAAT
scaffold690549_17.0	1	p2	(TA)6	12	1207	1218	CATGTGGATCATACAACAGGG
scaffold690566_13.2	1	p2	(AC)9	18	204	221	TCTACCAAGATCCTGCCTGC
scaffold690576_12.6	1	p2	(AT)8	16	1240	1255	CGACTATTGTTTGTCATCGCA
scaffold690579_18.2	1	p2	(TC)7	14	180	193	GAGGTGCTGGGTCACTGAAT
scaffold690582_15.4	1	p2	(CT)6	12	716	727	GGCAACCCACTTGTGAAACT
scaffold690586_12.3	1	p2	(AT)10	20	85	104	CCGCTCCTCCTTTTCTTTTT
scaffold690632_15.6	1	p2	(TA)9	18	45	62	AAGGAGGTGTTATGAAGGGATT
scaffold690676_15.9	1	p2	(TA)8	16	255	270	GCAGCACATTGTGGTCTTCA
scaffold690704_17.0	1	p2	(AG)6	12	556	567	TTTGGGGGTGCATATTTCTC
scaffold690737_12.8	1	p2	(AT)8	16	466	481	GTGGATCTGGGCTGCATTAT
scaffold690750_13.1	1	p2	(AG)9	18	914	931	TCAACCACACCTCCAACTCA
scaffold690758_15.5	1	p2	(AT)6	12	277	288	TGCAGTTGAGATAAGGGAGGA
scaffold690803_11.5	1	p2	(TA)8	16	116	131	TGAACATGCTCATATTTTAATACACC
scaffold690930_13.7	1	p2	(TC)7	14	217	230	CATCTCCAATGCCAGGTACA
scaffold691001_17.5	1	p2	(CA)6	12	1035	1046	TCGCATTCTTATTCCGATGA
scaffold691018_12.0	1	p2	(GT)8	16	706	721	TTAACTGCTCCTCCACCCAC
scaffold691045_16.1	1	p2	(TA)7	14	911	924	GCCCACCAATTACAACACCT
scaffold691061_16.0	1	p2	(AT)8	16	1116	1131	CGCATGCACATGCATTATTT
scaffold691064_12.3	1	p2	(TA)9	18	48	65	CCCAAGAATGGGTTATTATTTGA
scaffold691091_15.2	1	p2	(AG)6	12	167	178	AGACCGCAATAGCCACTGTC
scaffold691099_12.7	1	p2	(AG)6	12	732	743	CATGATCTGATGAGGCAGGA
scaffold691146_15.0	1	p2	(TC)6	12	219	230	TTAATCGGGCTCTGAGCAGT
scaffold691192_14.8	1	p2	(AG)6	12	1642	1653	GCCAAAAATGACGTTTTGCT
scaffold691213_14.5	1	p2	(AT)9	18	1587	1604	GCTAGAAGCAGCCGTGACAT
scaffold691246_10.7	1	p2	(CT)7	14	316	329	ATTGAATCTACGTCCACGGG
scaffold691268_12.4	1	p2	(AT)8	16	681	696	CCCTTAGTATGCTGTCCGAAA
scaffold691280_10.6	1	p2	(TC)7	14	170	183	AACAATCAAGCCCACCTCAG
scaffold691333_15.5	1	p2	(TA)6	12	918	929	TGAATTTGGTCTCCTTTGGC
scaffold691363_16.7	1	p2	(CT)6	12	508	519	GGAGAAATAATGACGGCCAA
scaffold691407_12.2	1	p2	(CT)7	14	259	272	TCGACCTCCATTAGAGCACC
scaffold691474_16.4	1	p2	(TA)6	12	139	150	ATTGTCCATGCAACCTACGA
scaffold691593_15.6	1	p2	(AT)8	16	258	273	GCAGACAACTGACAATGCAAA
scaffold691646_13.4	1	p2	(AT)6	12	2611	2622	GTGGAAAGAAGCTAACCCCC
scaffold691649_16.3	1	p2	(CT)8	16	258	273	CTAGATTCTTCGTAGCGGCG
scaffold691728_13.9	1	p2	(GA)6	12	857	868	AAGCCAAGTTAGCTCCCAGA
scaffold691762_11.6	1	p2	(TA)6	12	262	273	TTGTTAGAGGTTTTGACGGGA
scaffold691779_11.3	1	p2	(AT)10	20	87	106	ATGAATGGCCCATATTGGTT
scaffold691833_14.8	1	p2	(AT)6	12	514	525	CGAACATGACACAAATTGCC
scaffold691858_16.3	1	p2	(AC)6	12	1739	1750	CCTCCACGAATGGTTTTGAT
scaffold691883_14.2	1	p2	(AT)6	12	1240	1251	CACGGTCATGAATAAAATCGTT

scaffold691914_15.6	1	p2	(GA)6	12	571	582	ACTGACCAAGCATCCTCGAC
scaffold691937_18.3	1	p2	(GA)6	12	230	241	TGTTTACATGAAAGCGGTGG
scaffold691994_13.8	1	p2	(CT)6	12	1111	1122	CTCACCACACATGCACACTG
scaffold691996_14.8	1	p2	(TA)7	14	686	699	GCCCATCGTAACATGAAGGT
scaffold692049_13.0	1	p2	(AG)7	14	602	615	CTACATGCTGAGATCGAGCG
scaffold692055_16.7	1	p2	(TA)6	12	514	525	AAATAAAGGCTGCATGGCAC
scaffold692075_14.3	1	p2	(GA)6	12	487	498	GTTGCAGGGCAATACCATTT
scaffold692083_17.6	1	p2	(CA)7	14	3530	3543	TCAAATCCACAATTGCCTTG
scaffold692105_12.2	1	p2	(AC)7	14	434	447	GTACGCAGAGCTTTCCCAGT
scaffold692141_11.3	1	p2	(TA)6	12	452	463	ATGCCCTTTTCTCGAACATC
scaffold692209_16.1	1	p2	(TC)8	16	631	646	TCGTGGCTGGTTAAGCTTCT
scaffold692214_11.3	1	p2	(AT)7	14	787	800	AAACACATCTCGAGGGATCG
scaffold692246_14.2	1	p2	(AT)10	20	46	65	ATGAAGTTAGGAAATAAGAAGGTGTT
scaffold692326_14.1	1	p2	(CT)6	12	302	313	CCTTGAAGATCAAGACATCTACTCC
scaffold692347_15.2	1	p2	(AT)7	14	668	681	AGGGCAATTGATCTTGCCTA
scaffold692365_13.7	1	p2	(GA)6	12	2939	2950	AAAAACAAGAACCCACGCAC
scaffold692395_14.5	1	p2	(CT)6	12	305	316	AATGAGACCGGAAATGGATG
scaffold692427_18.3	1	p2	(GA)6	12	421	432	TGGTGGAGGCTCGGTTATTA
scaffold692439_11.2	1	p2	(AG)6	12	520	531	AGCTTCACCAAGCTTTTCCA
scaffold692495_14.6	1	p2	(CT)8	16	192	207	CAACCTCCTGCTGCTTATCC
scaffold692496_13.4	1	p2	(CT)8	16	260	275	AAAACTCCAAATCCCCCAAC
scaffold692534_14.6	1	p2	(CA)7	14	1866	1879	GAAAATGACCAACCATTCGG
scaffold692561_15.7	1	p2	(AC)8	16	323	338	TCAGTGATAGAAATTGAGGGGAA
scaffold692606_11.8	1	p2	(AT)9	18	573	590	TTACGGGTTGGGCTGTAGAC
scaffold692616_13.3	1	p2	(TA)11	22	44	65	GAGGGTTATCATGGGATCAAG
scaffold692643_18.1	1	p2	(TA)7	14	209	222	ATGCCGACACCACACATATC
scaffold692655_16.2	1	p2	(CT)6	12	613	624	GGTGCACCCTCACTTTTGTT
scaffold692694_11.5	1	p2	(AG)8	16	758	773	TAGGGTTCGAATCCCCACTC
scaffold692704_16.8	1	p2	(CT)6	12	2480	2491	TTTCCAGCAAAACATTGACG
scaffold692718_15.6	1	p2	(TC)6	12	298	309	TGGGAGAATTGGGCTAAGTG
scaffold692734_16.6	1	p2	(TA)6	12	136	147	TCCCATTTTGGGATGAATTT
scaffold692766_17.2	1	p2	(TC)12	24	1692	1715	GAACTTCTCCATCAGGGCAG
scaffold692777_12.3	1	p2	(TA)9	18	399	416	TGATCTCAAAAGATCAACGGTTT
scaffold692785_17.6	1	p2	(CA)9	18	6982	6999	TCTACCTAAAGCACGCCAAAA
scaffold692816_13.3	1	p2	(AC)9	18	582	599	CGATGGTTGGAAACAAATGG
scaffold692841_16.7	1	p2	(AG)6	12	178	189	ATTCATGAACCACAAAGGGG
scaffold692844_16.0	1	p2	(CA)6	12	7067	7078	TCACACCAAAGATGCTGCTC
scaffold692873_14.5	1	p2	(AT)8	16	261	276	CAAGTATTGGGGCAGCAAGT
scaffold692938_11.3	1	p2	(TA)6	12	907	918	AGTGTTGTACGGCGCTAGGT
scaffold692971_12.6	1	p2	(AT)8	16	1081	1096	CCACATAATGTTGCGCATGT
scaffold693010_14.2	1	p2	(CT)8	16	621	636	CCTGCTCAAACACATCAGGA
scaffold693084_14.0	1	p2	(AT)6	12	353	364	TGGCTTCATGCAAATTCAAG
scaffold693116_17.4	1	p2	(AC)6	12	842	853	CACGTTAAATCCCTTGCACA
scaffold693176_13.6	1	p2	(AG)6	12	456	467	TTTGTGGTATCAGAGCCACG
scaffold693218_10.5	1	p2	(CT)8	16	320	335	TGCAACAACAGAAGGATCAGA
scaffold693236_15.8	1	p2	(AG)8	16	251	266	TGTACCGTCCTTTGCATTGA
scaffold693248_12.2	1	p2	(TC)7	14	2015	2028	AATGCAAAGGATTTGATCCG
scaffold693278_16.9	1	p2	(CT)6	12	3142	3153	CCTTCCTTCTTCCTGTTCCC
scaffold693286_15.5	1	p2	(AT)8	16	262	277	GGACGCACCAGATTGTTTCT
scaffold693376_16.9	1	p2	(CA)10	20	1203	1222	TGTAAGGTGCTCCACCATGA
scaffold693381_17.1	1	p2	(AG)8	16	238	253	AAAATCTTCTCCGCATTTCG
scaffold693433_14.8	1	p2	(AT)7	14	435	448	ACATCAAAATCGGCATTTCC
scaffold693436_20.0	1	p2	(AG)7	14	264	277	AAATGGCACTTCAATAATGGC
scaffold693497_11.0	1	p2	(CT)6	12	41	52	TTTCTCAACCGAGCTCACCT
scaffold693576_16.0	1	p2	(CT)7	14	3631	3644	TCATGCAGTTGGGAGATGAA
scaffold693603_11.5	1	p2	(AC)8	16	55	70	TGATGTGATTAATTCCCACCATT
scaffold693616_16.1	1	p2	(TA)7	14	3339	3352	AAGCGTCGTGGGCTTGTAT
scaffold693624_15.5	1	p2	(GA)6	12	1340	1351	TGAGCAATAAATCCAGGGAGA

scaffold693634_10.	1	p2	(AG)6	12	200	211	TGCTTATCCAATCTCCGAGG
scaffold693683_17.	1	p2	(AT)9	18	261	278	CGCCACTCCAAACTTCAAAT
scaffold693684_14.	1	p2	(AT)7	14	265	278	GGGCTGGATACAATCCATCTT
scaffold693686_10.	1	p2	(TG)10	20	416	435	GCTGTGCGTCGATTTCTTTA
scaffold693687_12.	1	p2	(TG)10	20	405	424	GCTGTGCGTCGATTTCTTTA
scaffold693737_15.	1	p2	(TC)13	26	456	481	CAGCAAATCACCTCATCCCT
scaffold693788_12.	1	p2	(GC)6	12	53	64	TGGTTAGGTAAATGGGCCAG
scaffold693825_11.	1	p2	(AT)8	16	160	175	GCAGGTTAGCAGGATAAGGG
scaffold693828_13.	1	p2	(AT)9	18	1464	1481	TCTTGCTAAGAATATTGCAGGAGA
scaffold693835_15.	1	p2	(GA)11	22	2494	2515	TCTGGTTTCCAAACAAAGCC
scaffold693839_10.	1	p2	(TA)11	22	639	660	TAACGCGAATATGCTGATGC
scaffold693852_16.	1	p2	(AT)6	12	1262	1273	AATGCCCCACTAATCCCTTC
scaffold693861_17.	1	p2	(AT)7	14	264	277	GGGAAACTCACTACTCTCATTTCG
scaffold693913_15.	1	p2	(CT)7	14	916	929	CTTCCAGGAAACTCCACTGC
scaffold693956_15.	1	p2	(AT)6	12	918	929	TTGTTCGACGTCACAGCTAA
scaffold693972_16.	1	p2	(AT)6	12	108	119	CCGAATCCCGATAACTAAACA
scaffold693987_15.	1	p2	(AG)8	16	271	286	TGCTGCCTTAATCCACACTG
scaffold693990_11.	1	p2	(AT)9	18	452	469	GCTTCAATCCGTCACTCCTC
scaffold694024_13.	1	p2	(AG)6	12	648	659	AACTAATCGATGCACACCCC
scaffold694026_14.	1	p2	(AG)6	12	428	439	AGAAGTGGCGGAGGAGTCTT
scaffold694035_15.	1	p2	(TA)10	20	69	88	GGTTATCACCTGATTTTCTCCC
scaffold694087_10.	1	p2	(TA)9	18	1064	1081	AGGCCTTACATGCCGATCTA
scaffold694088_13.	1	p2	(TG)8	16	1767	1782	ATGAAGGACAATGGAAGGCA
scaffold694106_11.	1	p2	(TA)8	16	263	278	GGCCGATATTTGCTCACATT
scaffold694107_14.	1	p2	(TA)6	12	267	278	GCACCTCTATGGTTGGGAGA
scaffold694124_15.	1	p2	(CT)6	12	268	279	CACTGCGAGTTCAAGGATCA
scaffold694190_14.	1	p2	(AT)6	12	2480	2491	TTTTGTCGCCAATTGTTTGT
scaffold694231_15.	1	p2	(GT)6	12	126	137	TGAGTGAGTGAGCGTGTGTG
scaffold694269_15.	1	p2	(AT)8	16	830	845	CAGCCACGATGATATGACAAA
scaffold694304_11.	1	p2	(CT)9	18	782	799	CTCAATCGCACCGTCACTAA
scaffold694343_18.	1	p2	(AT)12	24	1068	1091	AGAAGCCTTATTGGAAGCCG
scaffold694371_14.	1	p2	(AG)7	14	159	172	TTCTACCACCACCCATCTCG
scaffold694382_14.	1	p2	(TA)8	16	264	279	ACGCGCGCTTTTAGAGTTTA
scaffold694394_18.	1	p2	(AT)6	12	45	56	TTCATTTTTAAATCCTATATCAACGA
scaffold694408_15.	1	p2	(AT)10	20	375	394	CCCAACCAGGCTGAATAAGA
scaffold694438_11.	1	p2	(GA)6	12	319	330	GGTGCTCAAAAATCCGAAAA
scaffold694445_13.	1	p2	(TA)10	20	59	78	CAGATTTGTTATGCGTTTTGACA
scaffold694489_15.	1	p2	(AT)6	12	269	280	TTCGCATGGGAATATAATGGA
scaffold694508_18.	1	p2	(CA)6	12	720	731	TCCTCCACTACCCAAAACAAA
scaffold694509_14.	1	p2	(TA)6	12	492	503	TGGTGAACAGAAACCCTGAA
scaffold694511_15.	1	p2	(CT)8	16	4678	4693	TCCTCCTCCCTTCCTTCAAT
scaffold694527_17.	1	p2	(TG)6	12	469	480	CAACAAAAATGGCACTCTTTCA
scaffold694540_16.	1	p2	(CA)7	14	2304	2317	CTCAGGAGCAATTGGGTGAT
scaffold694572_12.	1	p2	(TC)10	20	877	896	ATGATTTTGTGGTGTGGGGT
scaffold694600_16.	1	p2	(GA)6	12	187	198	AGGGCTGTCTCTCACTCCTG
scaffold694613_12.	1	p2	(TA)7	14	501	514	TTCACACATGCACCTCACCT
scaffold694620_18.	1	p2	(AG)6	12	1213	1224	ACTGCACTCAACCACAACCA
scaffold694632_16.	1	p2	(AG)8	16	622	637	CCTACCCTCTGTTCCGACAA
scaffold694649_14.	1	p2	(CT)7	14	868	881	GGCCTTTCCATTTTCCTCTC
scaffold694670_17.	1	p2	(TA)11	22	46	67	GGGTGTTTTGACAATATCGC
scaffold694728_15.	1	p2	(GA)8	16	1319	1334	CCCTTTGGTATGAGAAGCCA
scaffold694789_15.	1	p2	(TC)8	16	330	345	GCTCTTCTCCCCCAACTACC
scaffold694792_14.	1	p2	(TG)7	14	725	738	TATGCAGACGAAAAAGCGTG
scaffold694882_15.	1	p2	(AT)8	16	266	281	GCAAGGATTGTAGCAAAGGG
scaffold694884_17.	1	p2	(AT)6	12	1137	1148	TAGCAATCAAGTCCCTTCGG
scaffold694907_13.	1	p2	(CT)6	12	402	413	CATTGCTTTCCGACTGTTCA
scaffold694909_10.	1	p2	(AT)8	16	403	418	TCCTTCCTTGCTCCTTTTCC
scaffold694936_10.	1	p2	(AT)8	16	156	171	TCGATGAATGATGAGAAGCA

scaffold694945_13.2	1	p2	(AT)11	22	1051	1072	AACCGAAAAAGAAACCTTTCAA
scaffold695017_13.6	1	p2	(TA)9	18	264	281	GGTTTGCGTTAGATATGCCC
scaffold695023_16.5	1	p2	(TA)6	12	627	638	ACTTTGATTTCTTGGTGGCG
scaffold695091_15.6	1	p2	(CT)6	12	442	453	CCGTAATTTCTCGACACCGT
scaffold695092_16.6	1	p2	(TA)6	12	267	278	GTGCTTCCTGAGTACCCTGC
scaffold695097_16.6	1	p2	(TC)7	14	311	324	AAACTGAGTGATGCCAGCCT
scaffold695143_10.0	1	p2	(TA)10	20	499	518	AAGGCGACATGTTTCAATCC
scaffold695259_18.0	1	p2	(TC)9	18	264	281	AGAACCCATGGACATGGAAA
scaffold695306_15.0	1	p2	(TA)6	12	1337	1348	TCGTGGAGCAAAAACATCAA
scaffold695328_12.8	1	p2	(AT)7	14	269	282	CTTGTGAGCCATGTTGTTGG
scaffold695340_11.9	1	p2	(AT)6	12	363	374	CTGTGATGCCACTGCTGTCT
scaffold695375_14.0	1	p2	(AT)8	16	768	783	TCAAACCTTGTGACTCTGCAA
scaffold695514_14.6	1	p2	(AT)6	12	555	566	CATCTAATGTCCATTTTTGCCA
scaffold695519_15.6	1	p2	(CT)6	12	39	50	TTTCTCAACCGAGCTCACCT
scaffold695577_16.3	1	p2	(GA)6	12	685	696	CCATCCTGCACCATCATACA
scaffold695582_15.1	1	p2	(GA)6	12	1297	1308	AAATTGCACCTCGTCGACTC
scaffold695583_14.7	1	p2	(AT)8	16	554	569	GGACTTGTTCCCACTTCCAA
scaffold695589_14.1	1	p2	(AT)9	18	988	1005	TTTGGATCCATAACATGAAAAA
scaffold695611_13.6	1	p2	(GA)7	14	887	900	GCCAACTTTCTTCATCCGTC
scaffold695629_15.8	1	p2	(TC)8	16	1766	1781	TCACCTTCAGGGGACAAAAC
scaffold695676_15.0	1	p2	(TA)8	16	49	64	ATGGGTTATTACCGGATCGC
scaffold695714_16.4	1	p2	(AT)7	14	285	298	TGTTGCGATTTTCATTTTCC
scaffold695758_14.1	1	p2	(TC)6	12	1433	1444	TTCGTCAGTGGATCTTGCTG
scaffold695765_15.3	1	p2	(GA)7	14	219	232	AATGAATGGAAACGAGGTGC
scaffold695857_18.6	1	p2	(TC)7	14	952	965	AACCACAACCCTTCTCCCTC
scaffold695895_18.2	1	p2	(TC)6	12	54	65	CAGAAATTGCCTTGGTTGTTC
scaffold695899_15.5	1	p2	(GA)7	14	813	826	CTCTTCAATTTCTCCGCTCG
scaffold695900_15.6	1	p2	(GA)7	14	271	284	CGCCAAAAACACAAACAAGA
scaffold695928_16.7	1	p2	(AT)6	12	92	103	GAAACCCCCAATTGTCTCAA
scaffold695933_14.2	1	p2	(TA)9	18	266	283	ATGTTTTCAATCTTGGCCGT
scaffold695939_12.8	1	p2	(TA)6	12	273	284	GCACCGGATTGTTTCTCATT
scaffold695952_13.2	1	p2	(TA)9	18	1836	1853	GATTTATCGATCAACCATTTCTTTT
scaffold695977_12.9	1	p2	(TA)7	14	51	64	CCAATTTGAATGGTTATCACCTG
scaffold695991_16.7	1	p2	(CT)9	18	267	284	GGGTTGCAAGAAACCTTTGA
scaffold696009_16.4	1	p2	(AG)6	12	940	951	TTCAGAGGCGAGAACCAACT
scaffold696017_14.6	1	p2	(AT)8	16	908	923	TGGTTTCACCAAAAACCGAT
scaffold696040_15.8	1	p2	(TA)8	16	649	664	TGATTTGCATGCGTACTTGA
scaffold696041_15.6	1	p2	(AC)6	12	605	616	AGGGAAACGTTTTGGTGTTG
scaffold696116_15.9	1	p2	(AG)7	14	184	197	ATACATGGCACAAAGCGACA
scaffold696118_13.9	1	p2	(TA)9	18	639	656	CGGATCACCTAGGTTTTGGA
scaffold696155_14.8	1	p2	(CT)7	14	136	149	ATTCTGCTTTCCACGTGCTT
scaffold696185_14.5	1	p2	(CA)7	14	494	507	CTTTGGAGTTAATCCGCTGC
scaffold696222_15.2	1	p2	(TC)8	16	807	822	CAGCTGCACCACAGAAGAAA
scaffold696259_21.4	1	p2	(TA)7	14	88	101	CACATGAATGGATGTGATTTGTT
scaffold696282_19.6	1	p2	(AT)10	20	107	126	CCATTTGCAATGGACAAAAA
scaffold696299_13.2	1	p2	(AT)9	18	488	505	CAACAGGGAGAGATTGAGATTG
scaffold696313_16.1	1	p2	(CT)7	14	271	284	TCATGTCTCTTGGCCTTGTG
scaffold696314_16.2	1	p2	(TC)6	12	359	370	GGCAAAACCATCCTTTACGA
scaffold696328_13.9	1	p2	(TA)9	18	267	284	AAAGGCTTGCTAGTGGTTGG
scaffold696364_14.2	1	p2	(TG)7	14	1178	1191	ATCGAATGCACACACTTCCA
scaffold696392_15.8	1	p2	(AG)9	18	705	722	GTGCTGTGTTGAAACCCCTT
scaffold696393_16.0	1	p2	(CT)6	12	274	285	CCCCAAATTGATGGTTTCAT
scaffold696405_20.0	1	p2	(GA)6	12	190	201	CCTTCCCTTGACGACTTACG
scaffold696407_12.5	1	p2	(TG)7	14	287	300	TTCCACTTCTTGTTGTGATTGC
scaffold696457_11.3	1	p2	(CT)9	18	672	689	GCATGCGGGGACATAATCTA
scaffold696458_14.0	1	p2	(TA)7	14	969	982	GCTTATGAGGGTGAGTTGGC
scaffold696459_16.4	1	p2	(TA)7	14	974	987	TGGCCGAAAATTAGAGATGG
scaffold696479_19.0	1	p2	(TC)6	12	888	899	GCCTTCTCTCAAATAAGGGTG

scaffold696512_17.(	1	p2	(TC)7	14	319	332	TCCACGTTTCCATCTTCCTC
scaffold696581_17.9	1	p2	(TA)6	12	370	381	AGTCCGAGGATAACCCTTCC
scaffold696598_16.(	1	p2	(GA)6	12	101	112	GCAGAAAGACATTGATAACCACC
scaffold696695_10.9	1	p2	(TC)6	12	412	423	AAATCGAGACACCGACGAAC
scaffold696710_14.4	1	p2	(AT)7	14	179	192	CCCCTCCTCTCGAACTCTTT
scaffold696750_11.9	1	p2	(TA)9	18	44	61	TTTAGTGTTTTGACATGATTACTCG
scaffold696759_12.6	1	p2	(AT)10	20	356	375	AAACTGGACTGGGTATCAACAA
scaffold696762_16.9	1	p2	(TC)8	16	270	285	CTTTTTGGCAATTCCCTTCA
scaffold696792_11.8	1	p2	(TA)8	16	252	267	CAACAACACATGCTTCGACA
scaffold696857_18.1	1	p2	(AC)8	16	270	285	ACGTGCCAAGACTCAAAACA
scaffold696911_15.6	1	p2	(AT)7	14	1055	1068	CTGAGCCCTTCTTATTCATGTT
scaffold696927_18.(	1	p2	(CA)7	14	2114	2127	TCTGGCTGTGTTCGTTCTTG
scaffold696941_11.(	1	p2	(CA)7	14	344	357	CGGCCAAACCAATAAATACG
scaffold696975_15.5	1	p2	(AG)6	12	519	530	ACACACCCCAAAAGAAGACG
scaffold696980_13.3	1	p2	(AG)10	20	1623	1642	CTTCAGCTTCATTTGCCCAT
scaffold697029_16.(	1	p2	(AG)6	12	1626	1637	GCACATCAACACTCGCAAAT
scaffold697073_15.5	1	p2	(AG)6	12	3398	3409	CCGTGACAGGTCAAAACCTT
scaffold697080_18.2	1	p2	(GA)8	16	762	777	TCAGCTGAAGGAGGAGGAAG
scaffold697094_15.5	1	p2	(TG)7	14	3606	3619	CTCCAACTCTCACCCAGAGC
scaffold697124_13.5	1	p2	(AT)6	12	104	115	TCATGCAATCACTATTAAGACTCCA
scaffold697131_15.4	1	p2	(TC)6	12	322	333	CAGCGTTCTGTCGAAATCAA
scaffold697132_12.2	1	p2	(AT)6	12	502	513	CCAAGGCCGTACCTACTCAA
scaffold697140_14.6	1	p2	(CT)8	16	777	792	AGAAAAATGCACACTCGCAA
scaffold697194_15.1	1	p2	(TG)6	12	868	879	GGTCTTGCCGGATTCATCTA
scaffold697224_12.9	1	p2	(AC)8	16	3087	3102	CTGCATTAACCCCGCTTAGA
scaffold697232_15.(	1	p2	(CT)6	12	395	406	TCCCTTCTCTCCCACTCATC
scaffold697267_13.9	1	p2	(TA)10	20	675	694	CCTTTTCTTAGGCGTTCTTGC
scaffold697295_15.(	1	p2	(TC)8	16	2837	2852	CCCTCGATTGTCTTAATCGG
scaffold697300_13.9	1	p2	(AT)7	14	239	252	GCGTGAGTCGGTGAGTGTAT
scaffold697323_16.8	1	p2	(GA)9	18	233	250	GCTGAGGGGAGACTACATGG
scaffold697413_12.1	1	p2	(TA)7	14	835	848	CGTTGGCAAGGTATCGATTT
scaffold697448_10.5	1	p2	(AT)10	20	1384	1403	GGATGGAATGGATGTTTGGA
scaffold697449_18.4	1	p2	(GA)6	12	593	604	TCCATTTAATATGTCGATGACCA
scaffold697462_17.8	1	p2	(CA)6	12	93	104	CTAACCTTGGGCTTTGATGC
scaffold697511_10.6	1	p2	(CT)7	14	488	501	GGGGGAGGAGAAGATAGCTG
scaffold697522_12.4	1	p2	(TA)6	12	404	415	CTAAGGCTGATGGGGTTCAG
scaffold697531_15.8	1	p2	(CA)6	12	169	180	CCTTATCCAACGCTCCGTTA
scaffold697547_14.(	1	p2	(GA)6	12	4832	4843	TTGAGATTCCGTGAGGACTG
scaffold697582_18.9	1	p2	(AT)6	12	772	783	AAAACTTGACCCGATCATTG
scaffold697623_18.5	1	p2	(CT)6	12	655	666	CAGCAAAATCCTTCACCTCAG
scaffold697638_13.1	1	p2	(AT)10	20	330	349	GCACCAAAAAGGAAAAATAATCC
scaffold697661_11.2	1	p2	(AT)8	16	498	513	TTACCTCTGTTGGTGCAACTT
scaffold697701_13.6	1	p2	(AG)8	16	186	201	TTTCTTTGTTACCATGGGGG
scaffold697715_16.3	1	p2	(TA)6	12	625	636	GGACAAAACGGGAAATAGCA
scaffold697751_16.7	1	p2	(TA)7	14	94	107	CCCGTATACACACCACGGAT
scaffold697798_15.(	1	p2	(TA)9	18	271	288	CGGCATGTTCCTCAACTACA
scaffold697842_13.2	1	p2	(GA)10	20	692	711	TAGACGAAGGTCGAAATGGG
scaffold697898_15.8	1	p2	(TC)6	12	268	279	GTGTTGGGCCGTGTTCTACT
scaffold697926_13.9	1	p2	(AT)6	12	144	155	GTAGCTTCAAACTCCCGCTG
scaffold697965_15.3	1	p2	(TA)9	18	2628	2645	TGAGTAAAAGCACGATTTTGGA
scaffold697991_13.5	1	p2	(TC)6	12	452	463	AATTTCGTCCAGCCATTCAC
scaffold698043_15.3	1	p2	(AT)7	14	276	289	TGCAAGCACACTCAGACACA
scaffold698057_14.3	1	p2	(CT)6	12	585	596	GAGGCAAAGCAAGGCATAAG
scaffold698059_14.3	1	p2	(GA)12	24	3524	3547	CGAGTTCTTGATGCTGGTCA
scaffold698107_12.3	1	p2	(AT)7	14	166	179	CGATTAAAAACCAGACCGGA
scaffold698124_13.3	1	p2	(AT)11	22	1711	1732	ACTTACCGTCATTTCCGTCG
scaffold698138_12.(	1	p2	(CA)7	14	1517	1530	CACACTAAATTGGTGATGGATGA
scaffold698179_10.5	1	p2	(AT)9	18	430	447	GGCCTAGGTTTCGAGGTAGG

scaffold698215_10.4	1	p2	(AT)8	16	891	906	TTTTCCCATGAAGCCAAAAC
scaffold698296_15.4	1	p2	(TG)6	12	690	701	GCGCTCAAACTTAAGCATCC
scaffold698324_12.6	1	p2	(TA)6	12	337	348	AAATATCGGCCGTCAGATTG
scaffold698332_16.1	1	p2	(AG)8	16	868	883	TGCACTCTGTGATGGGAAAA
scaffold698336_15.2	1	p2	(TC)8	16	715	730	GTGCAGCAAAATTGCTTTGA
scaffold698341_14.0	1	p2	(TA)9	18	576	593	GCCTTCCATTCCAAAAATCA
scaffold698395_15.9	1	p2	(CT)6	12	2276	2287	AGTGAGGGAAAGTGAAGCGA
scaffold698414_15.2	1	p2	(TA)8	16	46	61	TGAAGTTAGGAAATAAGGAGGTGTT
scaffold698467_15.9	1	p2	(AG)7	14	308	321	TTCGTATTTGTCTAGCGCCC
scaffold698479_17.6	1	p2	(AC)8	16	961	976	CTTGAAATTCGCAACAGCAA
scaffold698504_12.7	1	p2	(AT)7	14	278	291	AATCTGACGGCCGATATTTG
scaffold698513_11.3	1	p2	(CT)6	12	174	185	TGGTGCAACCATTGGAGATA
scaffold698532_17.5	1	p2	(GA)6	12	1267	1278	AGTCAGCGGAACCGTATCAC
scaffold698554_13.4	1	p2	(AT)9	18	75	92	TGAAGTTAGGAAATAAGGAGGTGTT
scaffold698565_14.9	1	p2	(TG)7	14	618	631	AATTTAAAGCCTTGTGCCCC
scaffold698587_14.5	1	p2	(TA)7	14	905	918	TTTGATGGCTTGAGTTTCCC
scaffold698596_12.3	1	p2	(AC)6	12	599	610	CGAAAAGACACGCACACACT
scaffold698639_16.7	1	p2	(TG)6	12	1399	1410	ATCACAACTAGGGCTGACCG
scaffold698705_18.1	1	p2	(AG)6	12	203	214	TTTCATTTGGATTTCCGAGC
scaffold698714_14.5	1	p2	(CT)6	12	427	438	TCTTCCCTCTCTCCCCATTT
scaffold698744_15.5	1	p2	(TG)6	12	907	918	AACTCAAGATTCTGCATCGGA
scaffold698748_17.5	1	p2	(TA)8	16	151	166	CTGTGTGATGTGGAGTTGGG
scaffold698749_16.7	1	p2	(AT)6	12	3614	3625	ACAGGAATTCGCTTTCTCCA
scaffold698759_14.1	1	p2	(TA)10	20	273	292	TCACCCGGACCTCTTCTTAG
scaffold698799_13.9	1	p2	(AT)11	22	1285	1306	ACTGTCAACCCCTCCCTTCT
scaffold698822_15.4	1	p2	(TA)6	12	280	291	CGAAATTGAGCTTTTTGGGA
scaffold698829_14.8	1	p2	(AG)7	14	390	403	ACCACCCCAACACACAAAAT
scaffold698845_16.3	1	p2	(AC)7	14	357	370	TATATGGGCCGTTAGATCGC
scaffold698906_17.3	1	p2	(TC)8	16	1052	1067	AAGCATTTCATTGGGCAGAG
scaffold698943_15.0	1	p2	(CT)8	16	520	535	TAGCGTGCCTATTGCACATT
scaffold698972_12.7	1	p2	(AT)10	20	2017	2036	AACGCCGTCTGGATTTTATG
scaffold698973_14.4	1	p2	(TA)10	20	645	664	AAACCCTGTCGTATCTCATATTACAC
scaffold698996_14.8	1	p2	(TC)6	12	340	351	ATCACGAGTTTTTCCCTTAAAA
scaffold699029_14.8	1	p2	(AT)9	18	211	228	CACCGGTGAATGGAAAAATC
scaffold699067_12.4	1	p2	(TA)6	12	208	219	TCACATTAAATTTGGCGCAG
scaffold699077_13.1	1	p2	(AT)8	16	741	756	GATTAGCAAATTTCGGGACG
scaffold699082_14.9	1	p2	(GA)7	14	492	505	ACAACAATCAGCACACGGAT
scaffold699096_12.4	1	p2	(TA)8	16	712	727	TCATGATTTGGCTTTAGGGC
scaffold699102_17.5	1	p2	(AT)7	14	164	177	ATGAACAGAGCATTCCGTCC
scaffold699164_15.9	1	p2	(AG)10	20	273	292	AGTGCAAGGACAAATGGGAG
scaffold699181_16.2	1	p2	(CT)8	16	138	153	AAGTTGACGCCTCACTTCGT
scaffold699189_14.4	1	p2	(TG)6	12	74	85	CAAGAGTTGAAAATCCTCCCA
scaffold699279_13.9	1	p2	(GA)6	12	418	429	TAGGGTCCCACCACCTAACA
scaffold699298_16.3	1	p2	(TC)6	12	281	292	TGCTCCGTTTTCGATTCTCT
scaffold699304_12.4	1	p2	(TA)10	20	45	64	ATCGGTTATTACCGGATCGC
scaffold699331_15.8	1	p2	(CT)6	12	968	979	AAAGTGCAGCCCACCATTAC
scaffold699389_13.4	1	p2	(GA)8	16	77	92	TCTCAGATGTTTGGTCGGAA
scaffold699394_14.4	1	p2	(CT)8	16	279	294	TGGGTTTCATCAAGATTGGA
scaffold699411_12.8	1	p2	(TA)9	18	1127	1144	AGTGGTAGGCCGAGTTCAAA
scaffold699458_12.9	1	p2	(CA)6	12	164	175	AGTTGGGTTGGAGCATTGAG
scaffold699486_15.0	1	p2	(AG)8	16	1039	1054	TCAAGAAAGAATGTGGAGCAAG
scaffold699491_14.9	1	p2	(AT)6	12	2703	2714	GCACATAGCGTCTTCGTCAA
scaffold699495_16.3	1	p2	(CT)7	14	68	81	TTCCTTTCTTGCTTGTTTCTTTC
scaffold699501_13.3	1	p2	(TA)6	12	776	787	GCACATTTTATTTTGGGGGA
scaffold699523_13.4	1	p2	(CA)6	12	434	445	TTTCCACCATGATATCGACAC
scaffold699527_10.8	1	p2	(TC)6	12	41	52	GGCAACGGAATACCATTTGT
scaffold699550_16.4	1	p2	(TG)6	12	5903	5914	CAAGAGCGCCTCAAAAATTC
scaffold699573_13.4	1	p2	(AT)10	20	110	129	AGGTTAGCACGATAAGGGCA

scaffold699579_15.5	1	p2	(GT)6	12	337	348	CGTGTCCATTGAGTACCTCG
scaffold699582_14.1	1	p2	(TA)11	22	1316	1337	CTATCCTTTGCTTCAACCGC
scaffold699608_14.0	1	p2	(TA)7	14	50	63	TGAAGTTAGGAAATAAGGAGGTGTT
scaffold699617_15.0	1	p2	(AT)8	16	631	646	TTAGGACCCATAATCTGCCG
scaffold699694_16.6	1	p2	(TA)6	12	1864	1875	GTGGTTTAGGTGAACCCCTG
scaffold699728_16.0	1	p2	(AT)6	12	365	376	GGTTCAAAACCATGATTCCA
scaffold699746_16.0	1	p2	(CA)6	12	4086	4097	GCGGTACAGTTGAACCACAC
scaffold699762_13.2	1	p2	(TA)8	16	128	143	AGCACTGAAAACCCAGCCTA
scaffold699765_17.8	1	p2	(TG)8	16	279	294	AACAGTTCGCTGCAACAGG
scaffold699767_14.1	1	p2	(TA)7	14	281	294	CGTAGGAACTTACACAAAGTTGG
scaffold699770_13.7	1	p2	(AT)6	12	53	64	AAGGGCATTGTTGTACATTGG
scaffold699781_12.7	1	p2	(GA)7	14	73	86	TTCAAATTTGTTATTGATACCGATG
scaffold699784_11.8	1	p2	(TC)6	12	574	585	TCCTTCCACATCTCTCCGTC
scaffold699796_16.3	1	p2	(AT)6	12	284	295	ATCAAGTTCCTGCCCCTTCT
scaffold699799_14.4	1	p2	(AT)6	12	864	875	CGTGAATACACCAAACGCAC
scaffold699836_17.5	1	p2	(CT)6	12	555	566	CTGTCTCCTCCACCTCTTCG
scaffold699854_15.3	1	p2	(AT)6	12	141	152	GGCTAGCACAAATCTCTGGC
scaffold699868_18.0	1	p2	(TC)8	16	1826	1841	ATCCTTTATGGCGTTTGCAG
scaffold699932_11.2	1	p2	(TA)7	14	215	228	TCACGCGGAAATAAATCCAT
scaffold699968_19.7	1	p2	(AT)6	12	760	771	GCACCTTTCCTGAGACCAAT
scaffold699989_14.0	1	p2	(CT)6	12	364	375	GTTGCTTTGCAGACCCTCTC
scaffold700021_18.5	1	p2	(GT)8	16	106	121	CGGAGATCCAATCTCGTTTC
scaffold700025_13.4	1	p2	(AT)6	12	83	94	CATCCCTATCAACTCTTTCGATG
scaffold700058_12.8	1	p2	(TA)8	16	328	343	TCAAATGCACTTCATAAGCCC
scaffold700123_12.1	1	p2	(TA)7	14	283	296	TTGAACACACAGTTCATGCCT
scaffold700129_13.0	1	p2	(AT)7	14	283	296	GAAATTCTAGGTCCGCCTCC
scaffold700130_16.1	1	p2	(CT)7	14	644	657	TCGAAGCAAAGTCCTCATCC
scaffold700153_12.3	1	p2	(TA)10	20	518	537	TGACGCCCCAGATTGTTTAT
scaffold700155_15.2	1	p2	(AT)8	16	562	577	GAGCAACACAACCACGTGAC
scaffold700161_13.6	1	p2	(TA)6	12	1033	1044	TGGGTTTTACACATTGGCATT
scaffold700191_16.3	1	p2	(GA)9	18	3351	3368	AATTGCGGACGTAGTTGACC
scaffold700269_15.5	1	p2	(TA)6	12	119	130	GATCATTTCCAAAAACAATGATG
scaffold700275_12.8	1	p2	(AT)6	12	284	295	GGAACTACCATTTTTGAATTGGA
scaffold700290_11.2	1	p2	(TA)7	14	458	471	AAATCATGAGCGTCCGTCTT
scaffold700300_10.8	1	p2	(GA)6	12	618	629	ACCCTTTCCATCGTGAGAGA
scaffold700303_11.9	1	p2	(TA)7	14	161	174	TCCATTCCGAAAATCAAAGG
scaffold700392_17.8	1	p2	(TG)9	18	980	997	ACCACCACAGTTCACTGCAA
scaffold700399_17.1	1	p2	(CA)6	12	1299	1310	GGTGTCAGGCATGTTTGTTG
scaffold700408_16.3	1	p2	(AT)6	12	337	348	CCAGTTCACTCGAATCTCACC
scaffold700435_15.0	1	p2	(AC)8	16	730	745	ATCGTGTCCTTCTGGCATTC
scaffold700446_13.2	1	p2	(GA)8	16	73	88	TTCAAATTTGTTATTGATACCGATG
scaffold700452_15.8	1	p2	(AG)7	14	284	297	CAGACCATTCGAAAACAGCA
scaffold700474_14.3	1	p2	(AT)9	18	116	133	GATGTCTTTTGATCAGTTGGTGA
scaffold700522_16.7	1	p2	(TG)6	12	2329	2340	AACCGAGAGCATAACCTGACA
scaffold700534_16.6	1	p2	(TG)7	14	1299	1312	TGCATTATCGCATCAGGAAA
scaffold700643_16.0	1	p2	(TA)10	20	43	62	TTTAGTGTTTTGACATGATTACTCG
scaffold700675_16.5	1	p2	(TG)7	14	519	532	AAAATAAAGCTGGGCTGCTG
scaffold700676_13.5	1	p2	(AT)8	16	564	579	AACTTCATTATTTGGGGGTGC
scaffold700709_14.0	1	p2	(AT)9	18	2037	2054	CCTCCTCAACAGAGGATATAGGA
scaffold700733_13.5	1	p2	(TA)10	20	540	559	CCACTCTTTGACTCCCCAAA
scaffold700829_15.4	1	p2	(TA)10	20	572	591	GTCGGACGAAACCATGAAAC
scaffold700973_15.2	1	p2	(CT)6	12	659	670	CCCTAACTCTTGCCCCTTTC
scaffold700996_16.1	1	p2	(AG)10	20	565	584	CGAGCCTCAGCCAAAATCTA
scaffold701008_13.9	1	p2	(TA)7	14	497	510	TCCACATCACATGACCTTCTG
scaffold701035_15.5	1	p2	(GA)8	16	590	605	TCTCATCTGCTTGTCGTTGC
scaffold701119_22.9	1	p2	(GT)6	12	489	500	GTAGGGGGCAGACATTAGCA
scaffold701120_15.5	1	p2	(AG)6	12	426	437	AGAATTGAGACCCATGCACC
scaffold701150_16.9	1	p2	(AT)6	12	1545	1556	GAAATTGCTAGTTCGGGCCT

scaffold701172_11.[illegible]	1	p2	(AT)6	12	74	85	TTACGCTAGTCCGTGGGTTT
scaffold701181_11.[illegible]	1	p2	(TA)8	16	49	64	TGAAGTTAGGAAATAAGGAGGTGTT
scaffold701205_11.[illegible]	1	p2	(TA)13	26	754	779	TGCATGCATTTTAGCTTTGG
scaffold701296_14.[illegible]	1	p2	(TA)9	18	282	299	GGATTAGGTGGACATTTGGG
scaffold701333_17.[illegible]	1	p2	(TA)8	16	177	192	AAAATGCCCTTGTATGCTGC
scaffold701353_18.[illegible]	1	p2	(AT)7	14	139	152	ATAAAAATGGCACCTGGCTG
scaffold701393_13.[illegible]	1	p2	(AC)7	14	1012	1025	TGATTCACCTCCCATGATCC
scaffold701399_12.[illegible]	1	p2	(AG)6	12	376	387	ATGGGTTCGAGTCTCAATGG
scaffold701445_11.[illegible]	1	p2	(AT)9	18	406	423	TCCCGAAATCCGAATAAAAA
scaffold701452_13.[illegible]	1	p2	(TA)6	12	1491	1502	TGGTGATTGATCCACCTACG
scaffold701459_18.[illegible]	1	p2	(TA)6	12	422	433	AAGAGACAAACAAGCATGGGTT
scaffold701464_13.[illegible]	1	p2	(AC)6	12	241	252	GCTTTAGAGATGGGAGCACG
scaffold701485_16.[illegible]	1	p2	(CT)14	28	680	707	CATTCATATGAGGATGGCCC
scaffold701490_10.[illegible]	1	p2	(TC)7	14	490	503	CGGTACGGTAACGGTAAGGA
scaffold701516_14.[illegible]	1	p2	(CT)6	12	94	105	TCTTCACCACCTGAAGAGGG
scaffold701541_15.[illegible]	1	p2	(TA)6	12	80	91	ATTTGCTTGATCCAACGGTC
scaffold701583_11.[illegible]	1	p2	(TA)6	12	53	64	CCAATTTGAATGGTTATCACCTG
scaffold701631_15.[illegible]	1	p2	(GA)8	16	551	566	CAGAGGAGCCTCAAGATTGC
scaffold701680_16.[illegible]	1	p2	(CA)6	12	638	649	TCAGAGTGCTCAAAACCAAGAA
scaffold701744_15.[illegible]	1	p2	(AT)7	14	1248	1261	CGAAACCCTCTCTAATTTGATTGA
scaffold701747_14.[illegible]	1	p2	(AT)8	16	1157	1172	GGGTTATTATTTGATCACCTCCC
scaffold701749_16.[illegible]	1	p2	(TA)10	20	738	757	ATAAAACGTGGAGCGAGCAT
scaffold701775_18.[illegible]	1	p2	(CT)6	12	1601	1612	CTGCGAGTCATGCTCGATTA
scaffold701814_16.[illegible]	1	p2	(TC)6	12	290	301	TTTGCATGAGACGCCAATAG
scaffold701909_16.[illegible]	1	p2	(GA)7	14	502	515	AACACCAAAATATCGTCCCG
scaffold701924_13.[illegible]	1	p2	(AT)7	14	534	547	TGAAGAAGATTCTCATCCCACA
scaffold701930_17.[illegible]	1	p2	(TG)7	14	528	541	ACATTGTATTGGGCTGCTCC
scaffold702003_16.[illegible]	1	p2	(TC)6	12	782	793	CCACTCGATTTTGCTGGTTT
scaffold702100_15.[illegible]	1	p2	(AC)7	14	1620	1633	TAAAAGTTGATGATCCCCGC
scaffold702171_15.[illegible]	1	p2	(AG)8	16	1031	1046	TGATTCAGAATTTCTCCAGATGC
scaffold702185_12.[illegible]	1	p2	(AG)8	16	119	134	TGTGAGGGACACAATTCTTGA
scaffold702196_16.[illegible]	1	p2	(AC)7	14	3659	3672	AAATTCCAATGGTGGCCTCT
scaffold702232_14.[illegible]	1	p2	(AT)8	16	1014	1029	ATGCAAGTTTTGCTTCCCAA
scaffold702260_18.[illegible]	1	p2	(AG)6	12	28	39	GAGCAGGGAGATAAGAGAGAGAA
scaffold702277_12.[illegible]	1	p2	(GA)6	12	2226	2237	TGCATTCTTGGAACCACTTG
scaffold702324_12.[illegible]	1	p2	(AT)6	12	490	501	GGAGTTTCCGGTGTTGAAGA
scaffold702329_18.[illegible]	1	p2	(CT)7	14	1354	1367	CCACAATTTCGATCTCTCCTC
scaffold702355_15.[illegible]	1	p2	(AT)8	16	942	957	GGTAAACATGCACAGGTATAGACG
scaffold702425_13.[illegible]	1	p2	(CT)12	24	3604	3627	AGACCCAAAAAGTAGGCCGT
scaffold702488_14.[illegible]	1	p2	(TA)6	12	292	303	CAACCATTGATTCCCGAGAT
scaffold702519_17.[illegible]	1	p2	(CT)7	14	2282	2295	TCAATCCTTGTCACCAACCA
scaffold702561_16.[illegible]	1	p2	(AT)7	14	291	304	GGACACGCGGATCAGTATCT
scaffold702579_12.[illegible]	1	p2	(AG)12	24	1220	1243	TGCATTTTAAAGGTCCCAGAA
scaffold702581_14.[illegible]	1	p2	(CT)6	12	1015	1026	GAAATGGGAGAGCCAAACAA
scaffold702602_16.[illegible]	1	p2	(AG)7	14	867	880	CCCAGAAGCAGATCCATGTT
scaffold702605_19.[illegible]	1	p2	(TC)7	14	291	304	TCCTTGACCGCGTAAAGATT
scaffold702683_14.[illegible]	1	p2	(AT)8	16	288	303	TTCATGCATTCACCATACGAA
scaffold702692_16.[illegible]	1	p2	(TC)9	18	2966	2983	AGCACCGTGTACTCTGCCTT
scaffold702733_16.[illegible]	1	p2	(TC)6	12	1338	1349	TCGCTACCACGAACTAACCC
scaffold702762_14.[illegible]	1	p2	(GA)10	20	2652	2671	ATTTGGTCCCGTACAAGCAG
scaffold702813_16.[illegible]	1	p2	(AC)6	12	2850	2861	CGTTTCTTCCCTCATTTCCA
scaffold702828_15.[illegible]	1	p2	(CA)6	12	293	304	GACTTGCTTGCAAGAACATACA
scaffold702846_14.[illegible]	1	p2	(AT)6	12	2332	2343	CCACTTCAAGCAGTGCAGTC
scaffold702865_10.[illegible]	1	p2	(TA)8	16	421	436	GAATCTCAGCCACAAAATTCG
scaffold702867_16.[illegible]	1	p2	(TA)8	16	49	64	GGGTTATCATGGGATCCATTC
scaffold702908_15.[illegible]	1	p2	(TA)7	14	291	304	AAGAAGCAATGGTCGCAAGT
scaffold702923_15.[illegible]	1	p2	(CA)7	14	397	410	TCCAACTGGTTTGGTGGTTT
scaffold702947_15.[illegible]	1	p2	(GA)11	22	51	72	GGAGGAAGCATTGGACTTTG

scaffold702958_17.3	1	p2	(TA)6	12	2745	2756	CCTTTTTGCTTTATTAGAAACCTG
scaffold702968_14.8	1	p2	(CT)6	12	690	701	TTTTCGGTTTGTCTCATCCC
scaffold702997_17.8	1	p2	(AT)7	14	108	121	GGCGTGACACCATGTAAAAG
scaffold703028_14.5	1	p2	(AT)7	14	808	821	TTTTTGGTGGCGGTAGGTAG
scaffold703079_12.6	1	p2	(TC)8	16	640	655	TTTTGTTTCCCTTTCGATGC
scaffold703095_13.8	1	p2	(AG)6	12	544	555	CATTGATGGACCTGCTCTCA
scaffold703124_13.8	1	p2	(AT)10	20	2027	2046	TCTACTATACCCTTGCAAATTATCAGA
scaffold703139_15.2	1	p2	(AC)8	16	164	179	TTTGCATCGAGACCAGTTTG
scaffold703140_14.8	1	p2	(TA)6	12	294	305	TTCACAAGATTGTATGGTCCTTG
scaffold703148_14.4	1	p2	(AT)6	12	1082	1093	TATACACCCCGCATCCATTT
scaffold703157_16.5	1	p2	(TA)9	18	205	222	TCCAATTTGCCCTTTTATGC
scaffold703180_17.1	1	p2	(AT)6	12	2353	2364	AAGTTAGGCCTGCCATTGTG
scaffold703225_18.3	1	p2	(TA)9	18	71	88	TTCCAAATGAAATGAAAACCG
scaffold703256_14.4	1	p2	(AG)7	14	292	305	AAAATGGCGGTCAACTCAAG
scaffold703292_14.7	1	p2	(GA)7	14	1159	1172	ACATGACTCGAGCCTGTCCT
scaffold703348_15.9	1	p2	(GA)7	14	1022	1035	CCTACGAAGAAATACTGCGCTT
scaffold703349_16.0	1	p2	(AT)6	12	1071	1082	TCTTTGTGGGGAAGAACAGC
scaffold703360_22.3	1	p2	(CT)6	12	540	551	CACCTCCATCTCTGCTCCTC
scaffold703362_10.5	1	p2	(AG)8	16	41	56	CATGCATGAGCAAGAGACAGA
scaffold703371_15.6	1	p2	(AG)6	12	295	306	TTCCATTTCGTGATTCCTCC
scaffold703402_16.4	1	p2	(GA)6	12	62	73	CAACCATTATTATTCCAATCGAAC
scaffold703406_13.3	1	p2	(GA)6	12	624	635	CATGCATTCGCAAAGAAGAA
scaffold703428_15.1	1	p2	(AT)8	16	2617	2632	CTTTTCCCCCAGATGTCTCA
scaffold703442_16.1	1	p2	(CA)7	14	3063	3076	GGTCGAACACTCAGCAGTCA
scaffold703448_10.0	1	p2	(TA)7	14	149	162	TTTATGCGGCATTCATGTGT
scaffold703463_15.6	1	p2	(TC)9	18	289	306	CTGCATTTTCTGCTGGTTCA
scaffold703489_15.5	1	p2	(TA)6	12	909	920	ACGAAAACAAAAGGTACGGA
scaffold703502_15.8	1	p2	(TA)7	14	153	166	TGCAATGCATGGAGGAAATA
scaffold703586_18.1	1	p2	(AT)7	14	2880	2893	GGCGTTTGAATTAGCCTTGA
scaffold703621_12.9	1	p2	(TA)6	12	717	728	CGGTTTGAGAACCAATGACC
scaffold703629_14.2	1	p2	(AC)6	12	4470	4481	ATCTTTCTTGGGGCCATCTT
scaffold703634_14.6	1	p2	(AT)9	18	559	576	TGGCACACTCTCATTGCTTC
scaffold703650_10.7	1	p2	(CT)6	12	310	321	TGGAACACATGCCCAGAATA
scaffold703673_13.5	1	p2	(AG)12	24	33	56	GTAGATGCGTGTGGTCGATG
scaffold703695_13.3	1	p2	(AT)7	14	1036	1049	TTTCCCTATGAAAACCGTGC
scaffold703697_13.0	1	p2	(AT)6	12	162	173	GGAATAAACGGCCAATTCAA
scaffold703698_18.8	1	p2	(CT)7	14	93	106	ACAAAGCCACCGTCAGAGTC
scaffold703701_17.1	1	p2	(AG)6	12	1771	1782	CTGCTTTGTCCAGCATTTCA
scaffold703706_19.7	1	p2	(TA)8	16	434	449	AATTGTGCATGCCTGTTCAT
scaffold703716_13.4	1	p2	(AT)8	16	189	204	GATTCCGGTTTCAAATCCAA
scaffold703717_15.9	1	p2	(TC)6	12	1646	1657	TTCATTATCATCCGCCTTCA
scaffold703743_13.8	1	p2	(TC)6	12	949	960	ATGGACCAATGCACCAAAAT
scaffold703754_14.2	1	p2	(AT)6	12	88	99	ACAATTTGCTGTGGCTGATG
scaffold703779_15.7	1	p2	(AG)6	12	204	215	AATAGGCGCTTCGAGAAGGT
scaffold703824_17.3	1	p2	(TG)7	14	1588	1601	CATGTTTGCCTGTTTTGTGG
scaffold703847_13.3	1	p2	(AC)6	12	296	307	TGCATCGATGATCTTGAAGG
scaffold703877_13.9	1	p2	(TA)9	18	69	86	TTGAGCGACGTATCTCCTTG
scaffold703881_14.3	1	p2	(AT)13	26	2014	2039	AGGAAAATGTGATAGCAGCG
scaffold703903_12.0	1	p2	(TA)6	12	340	351	TCATATCATTGTTTCCGCGA
scaffold703910_16.3	1	p2	(TA)8	16	469	484	ATCCGGATCTGATTCGATTG
scaffold703922_16.4	1	p2	(GA)6	12	403	414	TCCACAGAGATTTCCCACATC
scaffold703968_16.9	1	p2	(TA)7	14	52	65	GGTGACGATGGTGATTGATG
scaffold703979_17.2	1	p2	(CT)6	12	298	309	TCCAGAACATAAACACCGCA
scaffold703985_11.1	1	p2	(AT)10	20	322	341	CTGGTACGTGCGACGTATTG
scaffold704017_13.8	1	p2	(CT)11	22	588	609	TGACTTGGTGAGTGCAAGAGA
scaffold704024_13.5	1	p2	(TA)7	14	894	907	ACTCAAGTGGGGGATTGTTG
scaffold704079_14.4	1	p2	(AT)9	18	292	309	TGATGATGATGGATTTAGCGA
scaffold704103_13.3	1	p2	(TA)12	24	874	897	GAAGACTCAATCATGGGCGT

scaffold704106_19.6	1	p2	(CT)8	16	864	879	TTGGTGATGTGCGTTTTGAT
scaffold704125_11.4	1	p2	(AT)7	14	365	378	GCTGGTCGGTTAATTCCTCA
scaffold704226_14.7	1	p2	(TA)7	14	94	107	ATCCAAGCCATTAATTCCGA
scaffold704345_13.7	1	p2	(GA)7	14	968	981	GGAAGACTCATTGCCCAAAA
scaffold704372_15.5	1	p2	(AG)6	12	622	633	GCATCTCTCCCATCTTCTGC
scaffold704400_18.6	1	p2	(TC)6	12	460	471	AGTGTTTTGATCGAGTCGCC
scaffold704406_12.4	1	p2	(TG)7	14	108	121	TTATGATATTTCCATAAGTCACACCT
scaffold704428_15.8	1	p2	(AG)6	12	300	311	AATGCAGAGACTGAATGGGG
scaffold704466_15.3	1	p2	(GA)9	18	594	611	CACTGGGATTTCAGACAGCA
scaffold704492_20.6	1	p2	(TA)6	12	97	108	AATGGCAAGCGTCTTTGTTT
scaffold704496_15.6	1	p2	(TA)14	28	132	159	CTTTAGGGCCCTTGATTTCC
scaffold704517_16.8	1	p2	(AG)7	14	1598	1611	GGAACTTTCTCTGGGGCTCT
scaffold704541_10.8	1	p2	(AT)8	16	480	495	TAGGTAACCGGTTCGACCTC
scaffold704601_15.7	1	p2	(TA)8	16	296	311	CTCTTCATACGAGAAGGGCG
scaffold704625_16.1	1	p2	(AG)7	14	673	686	AGGTGGGCCTAAAGTTTGGT
scaffold704725_14.8	1	p2	(CT)7	14	1673	1686	ACATTGAACGCTCCAAAACC
scaffold704821_16.8	1	p2	(TC)6	12	1080	1091	CCCCTCAATCATCCTCCTTT
scaffold704875_12.0	1	p2	(AT)8	16	340	355	GTTTGGGCCTTTTTATTCCG
scaffold704878_13.1	1	p2	(TA)8	16	1023	1038	ATACTAGTGCCTTGCCCGAG
scaffold704880_11.9	1	p2	(TA)6	12	177	188	TTCTAAAGGGGAAACGACAAA
scaffold704923_14.0	1	p2	(GA)7	14	1601	1614	ATAGTACACGTGGCGGAACC
scaffold704962_16.5	1	p2	(GT)6	12	1124	1135	AACGCCTTTCACCCCTAAAT
scaffold704967_16.4	1	p2	(GA)8	16	328	343	GCTACCCATTTCCCAGCATA
scaffold704974_14.2	1	p2	(AC)6	12	1148	1159	TGCAAGGCAGAGTCACATTC
scaffold704977_15.6	1	p2	(AT)7	14	667	680	AAACCACGAAATCAAGCAGG
scaffold704987_16.2	1	p2	(CT)7	14	200	213	TCCCATCAACCCTCAGTTTC
scaffold704992_13.6	1	p2	(TA)6	12	301	312	CAATAGCTGGCCACCCTCTA
scaffold705010_17.1	1	p2	(AT)6	12	2755	2766	GGGAGATGGGTTGAAACAGA
scaffold705023_14.3	1	p2	(AC)7	14	1729	1742	ATCGAACCATTCCTGCTTTG
scaffold705066_19.6	1	p2	(TC)6	12	606	617	CACTTACTTAAAGTCAACTCCCCTG
scaffold705067_10.7	1	p2	(TA)9	18	754	771	AATTTTTGCTCTTTTGCATCA
scaffold705103_19.2	1	p2	(AG)7	14	300	313	AAAAGAGAAGCCCTCCAAGC
scaffold705167_16.3	1	p2	(AC)7	14	1458	1471	CGGCAAAGAGTTATTCGGAG
scaffold705175_11.5	1	p2	(TA)8	16	368	383	GCAAGCTGGAGACGAAGATT
scaffold705187_13.8	1	p2	(TG)9	18	4016	4033	TGATTTGGGACACTCACAACA
scaffold705189_13.3	1	p2	(TA)6	12	112	123	TGAATTGAATAATCCCAAATCAAA
scaffold705210_14.6	1	p2	(AT)7	14	100	113	TCACCGAAAAGATATGAAAATCAA
scaffold705259_11.0	1	p2	(AC)8	16	51	66	TGCCAATACAAAACCAACCA
scaffold705383_13.5	1	p2	(TA)11	22	43	64	GCGTTTTGACGATATATAGTGTTTTG
scaffold705436_14.8	1	p2	(GA)8	16	4127	4142	GACTTCTTTTCGAACGTCGC
scaffold705524_16.3	1	p2	(AT)6	12	2902	2913	GCAATCGGTGACCTTTTCTT
scaffold705526_16.2	1	p2	(AG)6	12	2658	2669	TATCAGTTGGCAGGGGAGAG
scaffold705546_11.4	1	p2	(AT)6	12	342	353	CAAGTCCAGGTCGTTTGGAT
scaffold705547_19.1	1	p2	(AG)9	18	1186	1203	TGATGCTGCATTTTGTGTGA
scaffold705563_13.6	1	p2	(TG)7	14	1275	1288	CTTCAATCCCACCACCAAGT
scaffold705564_11.0	1	p2	(AC)6	12	122	133	TGGATGTCATACCGGACTCA
scaffold705572_15.4	1	p2	(AT)8	16	5412	5427	GAGCAGGATTCTGTGGAACC
scaffold705655_13.9	1	p2	(TG)7	14	834	847	GGCAGCTCACAAATCTCTCC
scaffold705684_11.6	1	p2	(GA)11	22	415	436	TCCCCAGCACAGTTATTTCC
scaffold705699_15.9	1	p2	(TA)8	16	113	128	AAAAGTGGCATTTTGCCCTA
scaffold705700_11.5	1	p2	(AT)11	22	62	83	GGAAATAAGGGGGTGTTATGA
scaffold705822_11.9	1	p2	(CT)9	18	591	608	GGACCAACTATGGCACAACC
scaffold705861_13.9	1	p2	(TA)7	14	1760	1773	CACTTTTCATCTCCAACCCAA
scaffold705864_10.5	1	p2	(CT)6	12	76	87	TCGGCTCTTTTTCTTTCTCG
scaffold705867_16.0	1	p2	(AT)10	20	1024	1043	TTATGCAGCAAAAATCCGAG
scaffold705898_17.0	1	p2	(GT)7	14	1995	2008	AGGAGAACCACGCATTTACG
scaffold705906_16.0	1	p2	(CT)7	14	5092	5105	CCATTCCTGAGACCAAAAGG
scaffold705912_14.8	1	p2	(TA)7	14	1684	1697	GGAATGATTTCCCTGAGCAA

scaffold705915_14.5	1	p2	(CT)6	12	210	221	TGTCCATTGGCAGTTAGCAG
scaffold705937_13.6	1	p2	(TC)6	12	247	258	CCAATAGTTCTTCGGTCCCA
scaffold705997_15.3	1	p2	(GA)6	12	736	747	GCACTCGTTTCTTTCTTCGC
scaffold706009_11.6	1	p2	(AT)6	12	312	323	TGAAATTCAATGATCGGACAA
scaffold706021_15.0	1	p2	(AT)7	14	866	879	CTGCCTTAGGTGGCTTTGAG
scaffold706118_14.5	1	p2	(TG)6	12	275	286	TTTTACGTGTTTGGATTTGGG
scaffold706119_15.5	1	p2	(AT)6	12	113	124	CATTACCCTGGCCAGTCAGT
scaffold706151_14.6	1	p2	(TA)9	18	860	877	TCCGATTTCAAACGAAAACC
scaffold706156_16.5	1	p2	(GA)8	16	820	835	ATTTTCCCCTCAAAACGAGC
scaffold706174_15.0	1	p2	(AT)8	16	524	539	GTGGAGAATGAAAGTGTGCG
scaffold706175_17.1	1	p2	(CT)6	12	721	732	TCTTATTCTCCCTCTCGCCC
scaffold706218_10.1	1	p2	(GA)6	12	185	196	CCTTCTTGCTCCTTGCTACG
scaffold706267_16.4	1	p2	(GA)6	12	1061	1072	CTTCAGATGGTGTCAAAACCC
scaffold706282_13.4	1	p2	(TA)6	12	1095	1106	TGTAATAAATCTTTAAAATGGGAATGA
scaffold706298_13.0	1	p2	(TA)7	14	734	747	CTCAGAATCCACTCCTGCAA
scaffold706369_18.5	1	p2	(AT)6	12	411	422	GGTGTGGTACTTGGGACAGG
scaffold706386_16.4	1	p2	(CT)8	16	302	317	TTTTAGCCACTTTCTTGCCC
scaffold706409_14.2	1	p2	(TA)11	22	98	119	ATGGGTTATCACCGAATCGC
scaffold706451_13.4	1	p2	(AG)8	16	1724	1739	CCGACCTTACATAGCGGAAA
scaffold706470_15.0	1	p2	(TA)6	12	307	318	GAGAAATGGCCCATGACTGT
scaffold706471_12.7	1	p2	(AT)8	16	128	143	GCCTCCTTTCATGCAATGTT
scaffold706486_16.0	1	p2	(GA)8	16	398	413	GAGAAACCCGACATGCAAAT
scaffold706549_15.3	1	p2	(TC)6	12	53	64	CTCCTTCTGCGCACTTTTCT
scaffold706550_15.3	1	p2	(CT)9	18	251	268	GTAGGAACCGTGCAGCACTA
scaffold706560_16.8	1	p2	(TC)7	14	502	515	AACCGCGTCTCTTAGTTTTATGA
scaffold706612_10.0	1	p2	(AC)7	14	979	992	ATGTTGCACGAATGGTTTGA
scaffold706622_12.1	1	p2	(TA)8	16	1081	1096	TCATTTTGATTACTTTTACTATTCGTG
scaffold706642_14.2	1	p2	(TC)6	12	843	854	AGTCGATTTTCTGCAACCGT
scaffold706670_16.0	1	p2	(CA)7	14	776	789	TGCAGCGAAAACATGGAGTA
scaffold706671_14.6	1	p2	(CT)6	12	68	79	TTTCTCAACCGAGCTCACCT
scaffold706689_17.2	1	p2	(TA)7	14	1343	1356	AATGCAATCCTTTCACTGCC
scaffold706701_16.0	1	p2	(TA)10	20	256	275	TTGTTCGGGTTGATTTGACA
scaffold706728_15.9	1	p2	(TG)8	16	1932	1947	TTGGCAACTATCCCTAACGG
scaffold706744_17.5	1	p2	(AT)6	12	309	320	TCAAAACGGTCAAACGATGA
scaffold706772_15.0	1	p2	(CT)6	12	1514	1525	ATGATAGGGCATGGCATTTT
scaffold706832_15.4	1	p2	(TA)6	12	610	621	AAGCAGGAAGTCATGCCACT
scaffold706837_14.4	1	p2	(AG)9	18	1009	1026	AGCAATTTGTGAAAGTGGGG
scaffold706839_15.0	1	p2	(AT)8	16	117	132	TTCGAGTTTTGATGGGAACA
scaffold706841_16.3	1	p2	(TC)9	18	3580	3597	AGAGGACTGGTCCGAGGAAT
scaffold706862_14.6	1	p2	(AT)6	12	116	127	CCTCTTTTCGACCTTAATAAGCC
scaffold706873_12.5	1	p2	(TA)10	20	96	115	TGAAGGTAGGAAATAAGGAGGTG
scaffold706887_14.0	1	p2	(AC)6	12	485	496	CTTGCATTATCCGGTCGATT
scaffold706898_12.4	1	p2	(AT)7	14	307	320	CGGTTTTTCACCGATTTTTG
scaffold706901_14.3	1	p2	(CT)6	12	166	177	CGAAGAGAACGATGTCGTGA
scaffold706909_17.1	1	p2	(CT)8	16	139	154	AACAACCGGCCTAATCCTTT
scaffold706914_13.1	1	p2	(TA)10	20	45	64	TGCGTTTTGATGATATATAGGTGTTT
scaffold706982_16.7	1	p2	(GA)8	16	1518	1533	ATGTTGCATGAGTGAGCTGC
scaffold707083_14.5	1	p2	(TA)9	18	202	219	ACCACGAACAATGACGAACA
scaffold707092_12.5	1	p2	(TA)11	22	203	224	TATGGGTTATCACCGGATCG
scaffold707098_16.4	1	p2	(AT)8	16	91	106	GGAATTGTGATTAGTGACGGC
scaffold707099_16.6	1	p2	(TA)6	12	896	907	GTTACTCGTGGAGGTGCCAT
scaffold707130_13.5	1	p2	(AG)6	12	243	254	TGCAAGAATGGTGAGAGTGG
scaffold707177_17.0	1	p2	(CT)7	14	1209	1222	CCCACGCCATATTCTCTCTC
scaffold707198_15.9	1	p2	(AT)6	12	1072	1083	ACCACTCATCAAAGGAGGGC
scaffold707199_17.7	1	p2	(AG)6	12	472	483	TCGGATTGTTGATTGGTTGA
scaffold707207_13.0	1	p2	(AT)9	18	1112	1129	TGATGATGGTGGTGGTGAGT
scaffold707224_15.3	1	p2	(GA)7	14	3055	3068	TGAATACGGAGCAAGAACCC
scaffold707307_15.4	1	p2	(GA)6	12	1495	1506	ACCGGATGCTAATTTGTTCG

scaffold707338_13.(	1	p2	(AT)6	12	669	680	TCGAGGAGTTTACACGGAAGA
scaffold707351_14.2	1	p2	(GA)8	16	308	323	GGTTCAAAAATCGGACTGGA
scaffold707370_15.8	1	p2	(AT)7	14	782	795	CAAGATGCGATCTATTTTGGC
scaffold707385_12.2	1	p2	(TC)9	18	306	323	CTTGGAAACCTTTTACCCCA
scaffold707412_12.5	1	p2	(AT)9	18	597	614	TGCACGTAGGCATGTTCAAT
scaffold707413_12.7	1	p2	(TC)6	12	1208	1219	CGAGGTTGCCCAAACTCTTA
scaffold707428_13.9	1	p2	(AG)8	16	308	323	TGGTGGTCTGGTGACTGAAA
scaffold707451_19.(	1	p2	(AT)6	12	311	322	AGGTGCACTAAGCCAGCACT
scaffold707472_13.1	1	p2	(TA)8	16	85	100	GCTACATATTTTGATGCATACGG
scaffold707551_14.8	1	p2	(GA)7	14	743	756	CAGCACACGAACAGCTTCAT
scaffold707578_14.2	1	p2	(AT)9	18	591	608	CCTAACCCTTGACTGCAAGC
scaffold707586_15.2	1	p2	(CT)6	12	418	429	CAACTCCCAAATCCTCCAAA
scaffold707613_15.3	1	p2	(TC)6	12	312	323	ACAAATTGGTTTTGGCGTTC
scaffold707617_15.8	1	p2	(AT)6	12	1306	1317	ATGAATCCACACAAGTGGGC
scaffold707632_18.3	1	p2	(TC)6	12	2235	2246	CAAAATTGGAACGACTTGCAT
scaffold707641_11.6	1	p2	(TG)11	22	63	84	ATGGGAAGGGGAGTTAGCAT
scaffold707689_12.1	1	p2	(CT)7	14	51	64	CAAACAACAATTCAGCAAATACC
scaffold707720_12.3	1	p2	(TC)11	22	1573	1594	CTCCTTCTGTTGATCCCCAA
scaffold707737_14.9	1	p2	(CT)7	14	874	887	TGAAGCAATTGTGCAAGGAC
scaffold707745_13.6	1	p2	(AT)6	12	56	67	CGTTTCTCATCTGATTACCGC
scaffold707757_14.4	1	p2	(TA)9	18	48	65	AGAGGGTTATCATGGGATCAA
scaffold707767_12.7	1	p2	(AT)9	18	123	140	ATATATGGGTTATCGCCGGA
scaffold707776_15.7	1	p2	(CT)6	12	313	324	CTGACTCGTGCCCTTATGGT
scaffold707804_15.5	1	p2	(TA)8	16	117	132	TTCGAAATCTAACGATCCGC
scaffold707869_12.4	1	p2	(AT)8	16	471	486	GGATCGAGTCCATGCCATTA
scaffold707877_15.4	1	p2	(TG)7	14	193	206	TACGTGTATGATGGAGGCCA
scaffold707878_15.6	1	p2	(CT)6	12	338	349	ACCACTGCATCACACCAAAA
scaffold707905_16.5	1	p2	(TC)8	16	1360	1375	GCCACCCCTAGTTTTGATGA
scaffold707928_13.5	1	p2	(TA)9	18	303	320	ACCAAACCAAACCAAGCAAC
scaffold707940_10.4	1	p2	(GA)6	12	117	128	GGGGACCATCTCAAATGTTTT
scaffold707958_16.8	1	p2	(AG)8	16	30	45	GCAAAGTGCCACCAACACT
scaffold707963_15.3	1	p2	(GA)6	12	2952	2963	TGGTTAGGATTTCTCTTTGCC
scaffold707964_15.(	1	p2	(TC)9	18	2779	2796	AAATGGCTGCAACGAAGAGT
scaffold707995_16.8	1	p2	(TA)6	12	635	646	GAGCATTCGGGGTGCTATAA
scaffold708052_19.3	1	p2	(AC)6	12	360	371	TCTCCTCCCTTCGTCTCTCA
scaffold708079_15.8	1	p2	(AT)6	12	4207	4218	CATTCATGCATCCAATCTGC
scaffold708095_16.8	1	p2	(TA)8	16	2090	2105	GTTCTCGCGCGACATTATTT
scaffold708097_14.1	1	p2	(AT)7	14	469	482	TTCATGGATTCAAAAGGGGA
scaffold708126_14.8	1	p2	(GA)6	12	272	283	TTTGATCCTTGCTTTGCCTT
scaffold708143_16.5	1	p2	(AT)9	18	95	112	TTCAGTTGGCCGAATGATTT
scaffold708146_15.(	1	p2	(AC)6	12	2903	2914	TAAGGCAGCTCAAAAGGGAG
scaffold708156_14.3	1	p2	(TA)6	12	252	263	CGATCTAACGGCCCAGATT
scaffold708161_16.9	1	p2	(AG)9	18	569	586	CAACGGTTTATCTGCGGAAT
scaffold708185_16.(	1	p2	(AG)6	12	922	933	CCGGACATGCTAACGAGACT
scaffold708217_12.3	1	p2	(TA)7	14	1221	1234	TTTGCACTAGATCACTCCCCTT
scaffold708218_15.1	1	p2	(CT)14	28	3268	3295	GGCGTCGGTTGATAGATTGT
scaffold708219_15.1	1	p2	(GT)6	12	309	320	CGAACTCCAGACACCACCTT
scaffold708236_14.4	1	p2	(AT)6	12	411	422	TGCAGTCTTACAGCCCAACA
scaffold708270_14.5	1	p2	(TA)10	20	1042	1061	CAACCAAAATGATGGCGATA
scaffold708291_16.6	1	p2	(GA)6	12	147	158	TTAAGGAACACACTTGGCCC
scaffold708301_14.3	1	p2	(AT)10	20	530	549	GATCTCGCGGATCAGAAGAC
scaffold708344_14.1	1	p2	(AT)6	12	161	172	GCAAGTAATGCGGAGCTAGG
scaffold708351_10.3	1	p2	(AT)13	26	491	516	ACCACTTCTTTCGCTACGGA
scaffold708362_13.5	1	p2	(AG)7	14	314	327	AGTGTTGAGCAAAAGGGCAG
scaffold708367_16.2	1	p2	(TC)10	20	4311	4330	TCTTTGCCTTCTTCCATGCT
scaffold708409_16.3	1	p2	(AT)6	12	712	723	CTCGGCCTTGGAACTTGTAG
scaffold708420_11.9	1	p2	(GA)6	12	547	558	ACGTTTGAATGCATTGGGTT
scaffold708427_15.8	1	p2	(TA)11	22	3435	3456	CCCCAACTAGGTACCGTGAA

scaffold708443_15.5	1	p2	(TC)6	12	115	126	CATCCCTTAATCCCCATCCT
scaffold708457_17.7	1	p2	(GT)6	12	650	661	AAAGTGATTGTGAAACCGGAA
scaffold708476_16.5	1	p2	(CT)7	14	1133	1146	TGTTTTTCCACAACTCAACCC
scaffold708483_15.3	1	p2	(TA)8	16	1409	1424	TGTCAGCTACCTAACAGCTGGA
scaffold708537_13.3	1	p2	(AT)6	12	1989	2000	CTTTCCCCATGCATCATCTT
scaffold708559_15.2	1	p2	(AT)7	14	2305	2318	CCTTATGCGTTCCAAAATGG
scaffold708578_17.2	1	p2	(TC)6	12	405	416	TGAGGCGGTTAGGAGTTGTC
scaffold708587_18.0	1	p2	(TA)7	14	614	627	ACCGGAATGAAAATCGGATA
scaffold708720_20.5	1	p2	(TA)8	16	474	489	TCTTTGTTTTCAACGCCTCC
scaffold708724_13.1	1	p2	(TA)9	18	130	147	GGAAAAAGAAACCCACATGC
scaffold708731_11.3	1	p2	(AT)6	12	161	172	GCAAGTAATGCGGAGCTAGG
scaffold708736_15.7	1	p2	(GA)6	12	646	657	GGACCAGAAATCCCAGTTGA
scaffold708778_12.6	1	p2	(GA)6	12	177	188	TTGCGAGAACCAACTCTGTG
scaffold708779_13.1	1	p2	(TA)9	18	590	607	ATGACACGCATAAAGGGACC
scaffold708802_15.4	1	p2	(TA)6	12	471	482	TGATGATTGCCCCCAGTTAT
scaffold708809_18.4	1	p2	(TA)10	20	202	221	CGGGGTCGGATTATACCTTT
scaffold708833_11.3	1	p2	(AT)8	16	313	328	TAAATACCAAGCTCCGCCTG
scaffold708864_13.3	1	p2	(GA)6	12	683	694	CTGTATAGGCGTGGCCAACT
scaffold708898_15.5	1	p2	(AG)6	12	2545	2556	TTCTTTGGTGACCTGTTGGA
scaffold708907_12.0	1	p2	(TC)6	12	706	717	GCCCACCACCTGATAACAAA
scaffold708957_13.6	1	p2	(TA)7	14	316	329	TGCATTTTTAATTTATTTTCCTGA
scaffold708972_15.0	1	p2	(TC)7	14	316	329	AATTATGTGGCCGAGGACAG
scaffold708989_16.1	1	p2	(CT)7	14	3719	3732	TGAAAATGGACCCCTCTCTC
scaffold709008_11.6	1	p2	(TA)9	18	47	64	AGGTTTTGACCATATTTACTGTTTTG
scaffold709023_14.1	1	p2	(AC)9	18	312	329	TCATAATGGTCTCCATCGCA
scaffold709053_15.5	1	p2	(AT)8	16	704	719	GGATTCATGAGATCGACGGT
scaffold709065_13.3	1	p2	(TA)9	18	134	151	GCATATGCATTAAAATAACTTGAGAC
scaffold709114_13.2	1	p2	(CT)8	16	1372	1387	ACAAAACAAAATGTTGGCCC
scaffold709115_14.0	1	p2	(AT)8	16	2627	2642	TTTTATGCATCGTGCCAAAG
scaffold709119_17.0	1	p2	(TC)9	18	2208	2225	GGAAGCGCTCTTTTTACACG
scaffold709122_15.1	1	p2	(AG)6	12	319	330	GAACGTCCAGTGCCAAAGTT
scaffold709144_15.4	1	p2	(TA)8	16	50	65	GGTTATCACCGGATCGCTAC
scaffold709198_16.4	1	p2	(AG)11	22	1673	1694	CGTAGGTTCAGATCATGGCA
scaffold709208_13.9	1	p2	(AT)8	16	465	480	AAAATCAAAGGCCCACAATG
scaffold709211_12.5	1	p2	(AT)7	14	408	421	GCACCAATATTAGGCCATCG
scaffold709246_14.2	1	p2	(AT)6	12	6809	6820	CAGCGTTTGCTTTGAGACAA
scaffold709260_14.5	1	p2	(TA)6	12	104	115	AATCTCGAAAATGGATGACCC
scaffold709301_15.4	1	p2	(CA)6	12	1384	1395	ATGGCATCCCACAAGTATCC
scaffold709311_18.7	1	p2	(AG)6	12	141	152	GCGAAATTCGTTGGTTTGAC
scaffold709363_14.8	1	p2	(TA)7	14	1040	1053	TATGCATGGATTGATGTGGG
scaffold709380_12.7	1	p2	(TA)10	20	313	332	TTTTTGGATGGAAAACCACT
scaffold709381_16.1	1	p2	(AT)7	14	67	80	GAGTTATCATTGGAGTAAGCCCC
scaffold709397_15.3	1	p2	(TA)8	16	1473	1488	AACTTTTCCGGCCTCTCATT
scaffold709399_11.3	1	p2	(AT)6	12	1127	1138	CGCAAATTAGTGGCTTGCTT
scaffold709457_15.5	1	p2	(GA)11	22	616	637	ATTTGGGTCTCTTCGACACG
scaffold709476_14.6	1	p2	(GA)6	12	274	285	AAGCCAGCACATGCATACAC
scaffold709491_10.5	1	p2	(TC)9	18	315	332	CCTCCAGGACACAAGACGAT
scaffold709510_15.3	1	p2	(TA)9	18	169	186	GATTGAAGACCGCCTCGATA
scaffold709517_12.4	1	p2	(TA)8	16	621	636	TTCATATTCACTTGAAGACTTGAAAA
scaffold709534_15.8	1	p2	(TA)6	12	1304	1315	TCTCACCTTCGGTCAAAACC
scaffold709557_16.5	1	p2	(TA)10	20	107	126	CCGGGGTATGCTATAAACGA
scaffold709564_11.2	1	p2	(TA)7	14	319	332	TCGTGAAACACTTCAACCCA
scaffold709580_16.7	1	p2	(AC)11	22	311	332	TGGACCATGTTTTAACTGTATGC
scaffold709591_15.6	1	p2	(TC)6	12	321	332	TTAACTCAAAGCACGAGGGG
scaffold709622_14.7	1	p2	(TC)6	12	641	652	ATTTCTGATGGTCAGGCCAA
scaffold709636_12.2	1	p2	(TA)9	18	855	872	TTCCAATTTCTCTTGGCAATG
scaffold709638_12.6	1	p2	(AT)8	16	90	105	AAATAGTTCGGGTTTTCGGC
scaffold709674_16.9	1	p2	(TA)6	12	1064	1075	GACGGCAACGTTTTACGTCT

scaffold709678_17.(	1	p2	(GA)6	12	734	745	TAGAAATGGAGGTTGGGTGG
scaffold709748_15.3	1	p2	(GA)8	16	414	429	CTCCACTTCACCGGCTTTTA
scaffold709760_17.2	1	p2	(TA)6	12	493	504	AATACCCTTGTATGCTGCGG
scaffold709767_17.8	1	p2	(CT)6	12	630	641	CACAGCCCCCATAACCAGTA
scaffold709794_10.8	1	p2	(TC)7	14	651	664	TAAATACCGACAACCCCCAA
scaffold709811_15.(	1	p2	(AT)6	12	488	499	AAACAAGCAAGCAATCAGCA
scaffold709867_10.7	1	p2	(AG)6	12	819	830	TGTGATCCGTTGAGGAATGA
scaffold709873_11.5	1	p2	(AT)8	16	517	532	TCTACCCCTCAGAATCCTCG
scaffold709874_10.9	1	p2	(AG)6	12	189	200	AAGAAGCAGCACTAAACCCG
scaffold709878_17.3	1	p2	(GA)8	16	1466	1481	GAAAGTTGCAGCCCAGTGAG
scaffold709918_13.4	1	p2	(AC)6	12	973	984	AGTTTTGCATCCAGGGTACG
scaffold709928_11.5	1	p2	(GA)8	16	433	448	CTGTCCGTCACGTTGAACC
scaffold709929_14.1	1	p2	(GA)7	14	661	674	TTCAAATTGCGGATGGATTT
scaffold709956_16.(	1	p2	(TA)7	14	72	85	GAAAATCAAAGGCCCACAAA
scaffold709966_16.3	1	p2	(CT)6	12	2429	2440	TGCACTTGTTTTCCCAATATCA
scaffold709975_11.(	1	p2	(AT)6	12	323	334	TTTAAGTCGGCGAAGGAAAA
scaffold709991_14.1	1	p2	(AT)7	14	321	334	GGAAAAACCATCACAAAAATTG
scaffold709998_10.4	1	p2	(TC)6	12	171	182	CAATTCGACGACCAAATCCT
scaffold710033_12.8	1	p2	(CT)10	20	2855	2874	GCACAGAGCAACCAATCTGA
scaffold710043_15.4	1	p2	(AC)6	12	634	645	AAAATGCCGAACTTCCAAGA
scaffold710063_12.2	1	p2	(AC)6	12	376	387	TCAGATCGCTATCGTGTTCG
scaffold710074_11.1	1	p2	(TA)12	24	657	680	CTATGCAGCCTCCAGCTTCT
scaffold710093_13.6	1	p2	(AT)6	12	1221	1232	GAATAAATATGAACGAAAGCACAAA
scaffold710095_15.7	1	p2	(CT)6	12	920	931	TTCGTGGTGTAATGGCAAAA
scaffold710125_11.3	1	p2	(AG)8	16	488	503	GTATCCCAGGCCGAATTTTT
scaffold710135_17.6	1	p2	(AT)6	12	637	648	GCATTACTTTCTGTTTGAGAGGAA
scaffold710151_15.3	1	p2	(CT)9	18	2238	2255	CTGTTGCTGGGAGAGGACAT
scaffold710157_14.7	1	p2	(AG)6	12	649	660	GGCATTGTCAGACGACGTAA
scaffold710166_16.(	1	p2	(GC)6	12	1873	1884	ATATATTGTGCAGGCAGGGC
scaffold710182_17.9	1	p2	(AT)6	12	498	509	TTGGACTTGTGCTTGCGTTA
scaffold710192_19.7	1	p2	(CA)6	12	1042	1053	ACCTGGCAAGATTCATCGAC
scaffold710204_13.6	1	p2	(AT)10	20	66	85	TATGATGGGAAGGGTGGGTA
scaffold710224_15.5	1	p2	(GA)10	20	3056	3075	ATTTTCATCGGAAATGCTGC
scaffold710229_16.6	1	p2	(TG)10	20	832	851	GCCTTCATTCCAGCTGTCTC
scaffold710263_14.2	1	p2	(CA)12	24	1977	2000	TCCATGAAGGAGGAACAAGG
scaffold710266_13.5	1	p2	(TA)6	12	132	143	ACTCAATCTGCAAGCGTGAC
scaffold710281_15.4	1	p2	(AT)10	20	539	558	GGTCAAATCGTGGTCCATTC
scaffold710295_13.4	1	p2	(TG)7	14	2165	2178	ACCAGAACCAACAAGCAAGC
scaffold710305_13.(	1	p2	(AG)7	14	524	537	TTCATTACCCCCACCCACTA
scaffold710335_15.8	1	p2	(AT)6	12	1985	1996	GAAAGCAATGATGACGACGA
scaffold710363_17.9	1	p2	(AT)8	16	118	133	TCCGCCTTAATCCATCAAAC
scaffold710364_12.2	1	p2	(AT)7	14	722	735	GATAGTCCATGCCCCATGAT
scaffold710392_12.4	1	p2	(AT)6	12	685	696	TGATTCCTTTATGGCGTCTG
scaffold710402_12.4	1	p2	(CT)7	14	1212	1225	CTCGCCAACGTACCTCTCTC
scaffold710403_14.9	1	p2	(AG)6	12	1222	1233	AAGCAGGACAGTAACAGCCG
scaffold710404_11.4	1	p2	(AT)11	22	44	65	TTCATAACATTTCTATCGGTATTTGC
scaffold710418_18.1	1	p2	(TA)7	14	77	90	TTTGCACCTGAGGAGGTGTT
scaffold710431_14.8	1	p2	(TA)6	12	841	852	ATTGCGGTAAAGACCACGAC
scaffold710471_16.(	1	p2	(GA)7	14	1220	1233	CACGTTGTCCTGGAACATTG
scaffold710474_15.8	1	p2	(TG)6	12	2329	2340	TCTCACATATTCCCATTGCG
scaffold710517_10.5	1	p2	(GC)6	12	271	282	GGACCATGAGTCACGAGCTT
scaffold710570_14.9	1	p2	(GA)7	14	1123	1136	TCCGGTCTATTCTCACCTCAA
scaffold710574_14.8	1	p2	(CT)8	16	582	597	TGCTACTTTGTCCCGGATCT
scaffold710588_14.9	1	p2	(TA)8	16	2600	2615	GGAGACAATGCACAGCTCAA
scaffold710662_17.(	1	p2	(GA)7	14	324	337	GGCACCTAACACCAATGCTT
scaffold710673_16.5	1	p2	(CT)6	12	334	345	TCGAAGGTGGATGTCTTTCC
scaffold710695_13.6	1	p2	(AG)6	12	537	548	GACGAAGAAAAGGATGTCCG
scaffold710696_16.1	1	p2	(AC)6	12	2023	2034	GAAAGCAGTGACGAAGGGTC

scaffold710722_11.4	1	p2	(TA)7	14	476	489	TCATCCTTAATTGGCTGCAA
scaffold710773_15.1	1	p2	(AG)8	16	693	708	TGGTTGGTCACGAAAGATGA
scaffold710778_16.9	1	p2	(CT)11	22	1200	1221	CTGGAAAAGACAGCCATCGT
scaffold710825_14.3	1	p2	(TC)6	12	2276	2287	CTAGTGTGCTGGCTGCTTTG
scaffold710844_15.4	1	p2	(TA)6	12	162	173	TGGCATCATATATGCGGTTG
scaffold710853_13.1	1	p2	(TA)8	16	50	65	TCGTGTTAAAGCACGGTATCA
scaffold710861_15.7	1	p2	(AT)10	20	788	807	TAATGGACGTCATGACCGAA
scaffold710884_21.1	1	p2	(AG)8	16	537	552	TACCAATGGACCACAAGCAA
scaffold710896_14.2	1	p2	(CT)10	20	316	335	CCCTTCAATCCCAAGGTTTT
scaffold710964_15.2	1	p2	(GA)6	12	787	798	GCTGTTTTCCATTTGATCTGC
scaffold710965_14.4	1	p2	(AG)6	12	221	232	CAGTCTGAAGCTTGGGGAAG
scaffold710971_12.2	1	p2	(GA)8	16	742	757	CAAGGTGGCTTCACACACAA
scaffold710990_12.7	1	p2	(TA)7	14	321	334	ATTCAATGGCAATCTCCTGG
scaffold711003_16.4	1	p2	(AC)8	16	1176	1191	CCCCAATGGTTAAGGTGACA
scaffold711010_15.4	1	p2	(AT)9	18	81	98	AATTGAATGGCAAGGATTGG
scaffold711029_15.5	1	p2	(TA)10	20	159	178	TGTGGTATCAAATCCAGCCA
scaffold711053_16.3	1	p2	(TA)8	16	3090	3105	CCAAGGGCATTACACTCGAT
scaffold711073_13.9	1	p2	(TC)8	16	324	339	CCCCGACAATCATCACTCTT
scaffold711094_14.7	1	p2	(CT)7	14	904	917	TGTCCCAATTGAAACTCGAA
scaffold711106_10.9	1	p2	(TA)9	18	1634	1651	TGATGCTAATCCAAACGATCC
scaffold711112_13.2	1	p2	(GA)6	12	1757	1768	TGTATTTTTCCCGTGAAGCAC
scaffold711116_15.9	1	p2	(AG)12	24	196	219	GCATTTTCCAAGGCAGACAT
scaffold711137_13.5	1	p2	(CA)6	12	1138	1149	TCCAGGTCCAAATGAAAAGG
scaffold711191_14.3	1	p2	(AG)6	12	815	826	CGCACTCTGCAACACTGAAA
scaffold711224_14.7	1	p2	(AT)7	14	328	341	TTCTCTCCGCACAATTCCTT
scaffold711240_15.9	1	p2	(TG)7	14	1759	1772	CCCAATAGTTCAGCACCATC
scaffold711245_14.4	1	p2	(TA)6	12	1108	1119	TGTCAATCGAATTAAACCGTACA
scaffold711249_14.3	1	p2	(GT)6	12	316	327	TGCATCTGATTCTGACGAGG
scaffold711263_16.5	1	p2	(AT)9	18	2824	2841	AAGGGGAGAGCAGGAAAATC
scaffold711286_14.6	1	p2	(TG)6	12	393	404	TTTGTTCAATCGCTTTGCTG
scaffold711287_15.2	1	p2	(TC)6	12	675	686	CGTGATAAGCACCCTCCAAT
scaffold711342_11.6	1	p2	(AG)7	14	53	66	GAGAAAGGGAGGAGGAGGAG
scaffold711375_15.6	1	p2	(GA)9	18	179	196	AGAGCATGGAGCCAAAAGAA
scaffold711422_16.0	1	p2	(AT)7	14	2831	2844	GATTGAATCTCCCACTTCCG
scaffold711426_16.9	1	p2	(GA)8	16	2330	2345	GACTGCTCTCCTCATCTGGG
scaffold711439_13.9	1	p2	(AT)7	14	711	724	CCGGCTATCTCACTCCAGAC
scaffold711480_10.0	1	p2	(AT)6	12	776	787	ATCCCGTATTCTCACCCCTC
scaffold711534_17.7	1	p2	(TA)9	18	2717	2734	CAGTCACAAAAGGGGGAAAA
scaffold711556_15.8	1	p2	(CT)7	14	2103	2116	CCTCCGGTTCAAAATCAAGA
scaffold711559_15.0	1	p2	(AG)8	16	419	434	ATTTCAGTCCCATCAGCCAG
scaffold711569_14.9	1	p2	(TA)10	20	1021	1040	AATGCGCTTTCTGCAAATCT
scaffold711578_10.6	1	p2	(AT)9	18	401	418	TCAATTTGATAATTTGAAGCTCTATCC
scaffold711621_11.3	1	p2	(TA)10	20	604	623	ACGTCAGCTTCTTCCCACTG
scaffold711666_17.0	1	p2	(AG)9	18	198	215	AAACGTGCGAGGAAAACATC
scaffold711737_16.9	1	p2	(CG)6	12	46	57	TATGGTGGTATGGTGTGGGG
scaffold711758_13.8	1	p2	(TA)10	20	658	677	TGAGGGGAATGCACCACTAT
scaffold711771_15.8	1	p2	(AT)8	16	1273	1288	GCCTTTGACATCCAGGACAT
scaffold711775_17.2	1	p2	(GA)6	12	5545	5556	TCAGTTGGCACCAAAATTGA
scaffold711861_17.6	1	p2	(TC)6	12	332	343	GCCGTTCTTCCCTTCTCTCT
scaffold711865_19.0	1	p2	(CT)8	16	329	344	TTTACGCACATACCCCCATT
scaffold711866_14.6	1	p2	(CT)6	12	333	344	CAGGAGCAACCATTTTGTGA
scaffold711870_11.7	1	p2	(CT)9	18	972	989	TCACCCATTTCCACCAAGAT
scaffold711885_15.5	1	p2	(AG)6	12	2907	2918	CCTGAGCGGATAAATCCAGA
scaffold711922_15.0	1	p2	(TA)7	14	50	63	AAGGAGGTGTTATGAAGGGATT
scaffold711972_12.5	1	p2	(AT)9	18	1059	1076	CATCATGTAGGAAAATGTTTACAGA
scaffold712010_16.9	1	p2	(TA)11	22	2925	2946	TTGACACGCATGCTTACACA
scaffold712013_15.3	1	p2	(TA)12	24	91	114	TCAAACTCATTGTTGTTTCACAAA
scaffold712061_17.3	1	p2	(AC)7	14	376	389	TCACCTAACCCCTCTCTTGG

scaffold712090_15.[illegible]	1	p2	(AT)6	12	1034	1045	CTCTTCGGTGATCAAGCGTT
scaffold712111_15.[illegible]	1	p2	(AG)9	18	7876	7893	GATCAAAACCCCTCCATCAA
scaffold712121_16.[illegible]	1	p2	(AG)6	12	860	871	GCTGAAGCAATCGAGAAGGT
scaffold712150_11.[illegible]	1	p2	(TA)7	14	778	791	TACAGCGTGGACGGTACAGA
scaffold712186_10.[illegible]	1	p2	(TA)6	12	129	140	TAGTGGGGGATGATGTGGAT
scaffold712243_17.[illegible]	1	p2	(AT)7	14	114	127	AGGCCTAATTGCCGTCCTAT
scaffold712254_15.[illegible]	1	p2	(TC)7	14	2195	2208	ACAAGAAGGTGGCCATGAAC
scaffold712274_16.[illegible]	1	p2	(AT)8	16	652	667	GAAACACAAAAATTAGCATACAACG
scaffold712288_16.[illegible]	1	p2	(TA)11	22	453	474	CAACAAAAATCCTAGTCCATCCA
scaffold712312_16.[illegible]	1	p2	(GT)7	14	738	751	CCTTCCCACATTCCACATCT
scaffold712339_15.[illegible]	1	p2	(GA)10	20	1671	1690	ATCTCTGCGATGCCTTTCAT
scaffold712385_15.[illegible]	1	p2	(TC)6	12	549	560	ATGTTGCCACCTCAGCTTCT
scaffold712389_14.[illegible]	1	p2	(AT)12	24	2018	2041	TCATGACTCACGAGATGGGA
scaffold712395_15.[illegible]	1	p2	(AG)6	12	118	129	CCCAAGCATTAAAGCCCATA
scaffold712398_14.[illegible]	1	p2	(AC)8	16	510	525	TCCAAACTTGATCCGAGAGG
scaffold712411_15.[illegible]	1	p2	(CA)6	12	134	145	CCCGTTATGATTCGTTAAATCG
scaffold712423_16.[illegible]	1	p2	(CT)7	14	274	287	TCAGACCAAATCAGAAGGCA
scaffold712434_14.[illegible]	1	p2	(GT)6	12	1724	1735	TGAGGGATGATGCATACGAA
scaffold712452_18.[illegible]	1	p2	(AG)6	12	1096	1107	ATCACAAAATTCACACCCCG
scaffold712532_10.[illegible]	1	p2	(GA)6	12	872	883	GGGCTAAAATGGTGTTGGAA
scaffold712582_11.[illegible]	1	p2	(TG)8	16	1508	1523	TTCCTGGAGGATCTGCATTC
scaffold712584_13.[illegible]	1	p2	(GA)8	16	1647	1662	CTCTCTCATGCGTGGTGAAA
scaffold712639_15.[illegible]	1	p2	(AG)8	16	333	348	TCAAGCTAACCAGCCAACCT
scaffold712655_17.[illegible]	1	p2	(CT)8	16	295	310	ACACAGACAACCCATGGAGA
scaffold712692_17.[illegible]	1	p2	(AG)7	14	5486	5499	CCGTGGAAGACAAGGGATAA
scaffold712728_12.[illegible]	1	p2	(TC)7	14	1144	1157	AACGCAAACACATACGCAAA
scaffold712738_18.[illegible]	1	p2	(AT)8	16	1196	1211	ACGATGTCGTGTAGGGAAGG
scaffold712780_16.[illegible]	1	p2	(TA)6	12	2918	2929	CGAACATTGTCACCGTCAAC
scaffold712783_15.[illegible]	1	p2	(CT)7	14	1005	1018	TTGGCTCTGGTTTGTTACCC
scaffold712784_12.[illegible]	1	p2	(GA)9	18	863	880	ACGTAAGGGTGATGGGTGAA
scaffold712789_16.[illegible]	1	p2	(TC)8	16	907	922	AATCCAAGGTACCCCACTCC
scaffold712812_18.[illegible]	1	p2	(AT)6	12	872	883	TGAAATTGTGTTGCAGGCTC
scaffold712814_15.[illegible]	1	p2	(AG)6	12	152	163	CGAACGCAATGCAATAAATG
scaffold712841_14.[illegible]	1	p2	(AT)6	12	3034	3045	GCAAAAGAAACAAACGGGAA
scaffold712854_11.[illegible]	1	p2	(TA)6	12	100	111	GCAAAATTGGTCTCTATGTGTAGAA
scaffold712896_14.[illegible]	1	p2	(CT)7	14	1099	1112	AAAGGAGGTGGATCGGTTCT
scaffold712904_15.[illegible]	1	p2	(TA)8	16	545	560	GAGAAATCAATCTCAGCCGC
scaffold712911_16.[illegible]	1	p2	(TA)6	12	191	202	CCGGTTATCCAATCCATTTC
scaffold712920_13.[illegible]	1	p2	(TA)10	20	734	753	TCCTCCTAAAATAACGGCCA
scaffold712941_11.[illegible]	1	p2	(AG)7	14	675	688	CGTGGGTGTAGTGATGGATG
scaffold712968_14.[illegible]	1	p2	(AT)7	14	612	625	GTGGCATCGTGAGGAGTGTT
scaffold712993_10.[illegible]	1	p2	(AG)6	12	282	293	AAAGTCCTACATGCCGGTTG
scaffold713006_14.[illegible]	1	p2	(AC)7	14	2096	2109	ACCCCCACTTCCTCTGTTTC
scaffold713008_16.[illegible]	1	p2	(TA)6	12	2511	2522	TGCATGAGAAATGAGCGGTA
scaffold713053_12.[illegible]	1	p2	(TA)13	26	660	685	ATGAGTTATCACCGGATCGC
scaffold713061_16.[illegible]	1	p2	(CT)8	16	525	540	CGAGAATTAGCTGCCACTCC
scaffold713078_15.[illegible]	1	p2	(GA)7	14	337	350	CCGGCTCCGATCTATACAAA
scaffold713091_14.[illegible]	1	p2	(TC)6	12	507	518	AAAACCTCCAATCCCTCCAC
scaffold713129_13.[illegible]	1	p2	(GA)7	14	702	715	GCATTGGCGTTTGAAGAAAT
scaffold713147_16.[illegible]	1	p2	(CT)6	12	309	320	ATCCGCCAAACCTTCTTCTT
scaffold713157_12.[illegible]	1	p2	(TA)9	18	46	63	GAACCACCGAGTACCAAGTGA
scaffold713163_12.[illegible]	1	p2	(AG)6	12	640	651	AAACAATGTAGGGCAAACGG
scaffold713171_14.[illegible]	1	p2	(TA)9	18	47	64	GGTTATCACCGGATCGCTAC
scaffold713175_15.[illegible]	1	p2	(AC)6	12	692	703	TGGCTCTTTCAACTCAGCAA
scaffold713262_16.[illegible]	1	p2	(AT)6	12	603	614	TGTGGGTCAGCTATGCATGT
scaffold713304_16.[illegible]	1	p2	(CT)6	12	2752	2763	TAGCGTAGATATGCCAGGGG
scaffold713308_17.[illegible]	1	p2	(CA)7	14	627	640	AATGTTGCGAATGGAAGAAA
scaffold713321_11.[illegible]	1	p2	(AT)10	20	224	243	GTGCGTGGCTCAATCCTATC

scaffold713396_14.(	1	p2	(TA)10	20	631	650	AATTACATTGGGCCATCCTG
scaffold713405_14.7	1	p2	(AG)6	12	341	352	ATATTCGGATCAGGAATGCG
scaffold713440_19.9	1	p2	(TA)7	14	339	352	TCCAAATGCAACTGGTGAAA
scaffold713444_15.2	1	p2	(TA)7	14	431	444	TGAGTAACGCATCAAGCCAC
scaffold713454_13.4	1	p2	(TA)8	16	539	554	TTCTCGGGATTTAACGTCCA
scaffold713458_10.9	1	p2	(AT)12	24	217	240	AGCTTAAAAGGCCGGTCAAT
scaffold713469_15.(	1	p2	(AT)6	12	341	352	CCCAGATAATTTTATATGTGGTGAAA
scaffold713481_10.4	1	p2	(AT)9	18	536	553	GTGATTGGTGGTTTTGTGGA
scaffold713488_14.(	1	p2	(AT)6	12	917	928	GGCTTCTTCTCTACCCAATCG
scaffold713497_17.(	1	p2	(CT)7	14	362	375	CTCTCCAAAAACAGTCCCTCTC
scaffold713502_18.5	1	p2	(GA)6	12	410	421	GAACTTCACCGCTTGGACAT
scaffold713510_14.7	1	p2	(CT)7	14	892	905	AGGCGTCAAAGAGGAAGACA
scaffold713592_19.2	1	p2	(TA)6	12	2482	2493	TTCGTTCATTGCATGTGGTT
scaffold713638_14.3	1	p2	(AT)8	16	932	947	CCCATCTCGAACATTCCTGT
scaffold713670_16.9	1	p2	(CT)8	16	338	353	CATGCAGGTTTTGTCCAATG
scaffold713708_12.(	1	p2	(TA)9	18	755	772	GCCGCAATATACATGGCTTT
scaffold713710_14.(	1	p2	(TA)9	18	2439	2456	TTCTTTTTGCATTAGCCTTTGA
scaffold713862_14.6	1	p2	(TA)7	14	510	523	TAGCAAATTTCGGGATGAAA
scaffold713868_14.2	1	p2	(AT)14	28	33	60	GGAGTTTTGACCTAATTAAGACCC
scaffold713877_16.(	1	p2	(CA)6	12	1549	1560	CTGGAAAACGCTGATGGATT
scaffold713944_14.1	1	p2	(AC)8	16	1023	1038	GCAAATTGTCACTCCCTTTCA
scaffold713953_13.9	1	p2	(TG)8	16	725	740	TGTTGCCTTGCAAATTATTTCT
scaffold713965_13.2	1	p2	(TC)7	14	342	355	GCAAAGAAGAGTGGAGGCAC
scaffold713990_14.9	1	p2	(TA)8	16	86	101	TTTCGTGTTTCTTTTGATTTTTCA
scaffold714016_17.1	1	p2	(TA)8	16	341	356	ACTCAATGGACACATGCCAA
scaffold714042_19.1	1	p2	(CA)6	12	345	356	CGCAACCTCTAGAAACCCAC
scaffold714055_16.9	1	p2	(TC)9	18	818	835	CGTCTTAGATCCCTCCCACA
scaffold714065_11.(	1	p2	(AT)8	16	1040	1055	GGCCTGGGCCTACATAAGTT
scaffold714078_13.7	1	p2	(AT)8	16	934	949	CCAACATCACACTCGACCAC
scaffold714104_16.(	1	p2	(CT)7	14	465	478	CCATTCAGCAATAATAGGCCA
scaffold714107_14.7	1	p2	(TA)8	16	48	63	TGGTTATTAGGGGATGGTGA
scaffold714116_14.(	1	p2	(TA)10	20	1627	1646	TTTCAAACGAGATCTCAGGG
scaffold714119_10.4	1	p2	(TA)9	18	339	356	ATTCCAAAAATCAAAGGCCC
scaffold714124_14.6	1	p2	(TA)12	24	1294	1317	GGAATTCAATTGTTTATTTGTTGC
scaffold714162_16.6	1	p2	(TC)7	14	1891	1904	TGTGTCGTGTTCGTGTCAAA
scaffold714265_13.5	1	p2	(GA)10	20	1319	1338	CCTCACTGGAGGAAGGATCA
scaffold714282_14.9	1	p2	(CT)6	12	607	618	GGCAACCCACTTGTGAAACT
scaffold714290_12.4	1	p2	(AT)6	12	332	343	TTTGATATTCTCACCATCCTACTCA
scaffold714293_17.3	1	p2	(AT)10	20	1520	1539	TGGGACCCTAATACACCATCA
scaffold714306_13.5	1	p2	(CT)7	14	345	358	CCAAAATTGGCGGATAACTG
scaffold714357_17.3	1	p2	(AG)7	14	63	76	GAGCGCACTCGAAAAGTAGC
scaffold714369_11.2	1	p2	(TA)7	14	643	656	AATCCAACGGTAAAACAGCG
scaffold714431_14.2	1	p2	(CT)14	28	102	129	GGCCTCTTGACAAAAAGCTG
scaffold714445_14.1	1	p2	(AT)8	16	599	614	GTGTGCGTGTTGAATATCGG
scaffold714479_14.5	1	p2	(TA)9	18	128	145	AATCATAGCCATCCATTCCG
scaffold714482_13.8	1	p2	(AG)9	18	831	848	ACACACACCCAGAGAGAGGG
scaffold714483_11.2	1	p2	(AT)7	14	664	677	CCAGGCTTGTGCAAGCTATT
scaffold714491_18.7	1	p2	(TC)7	14	784	797	AATCACCAAACGTCACACGA
scaffold714495_14.7	1	p2	(CT)10	20	2133	2152	CAGAAAGAGCCCATAGCCAC
scaffold714497_15.9	1	p2	(TA)6	12	348	359	TCAGCATGTTGCCATTAGGA
scaffold714501_15.3	1	p2	(TA)10	20	46	65	AAGGAGGTGTTATGAAGGGATT
scaffold714513_15.9	1	p2	(TA)7	14	3013	3026	CTCTTCTTCTGCCCCACCTA
scaffold714530_10.6	1	p2	(AT)7	14	719	732	TCACGCACTCTCACTCATCC
scaffold714540_14.5	1	p2	(AT)11	22	646	667	ATGGGGTTCTTCTCCTTTCC
scaffold714544_11.2	1	p2	(TG)7	14	378	391	GAGGTGGCTTCTTTAGGGCT
scaffold714545_15.6	1	p2	(AG)6	12	292	303	TCAGCATGTTCCATGTTCAAG
scaffold714551_15.8	1	p2	(CT)6	12	121	132	TATCTCTCATTTCCTCCGCC
scaffold714553_17.2	1	p2	(AC)6	12	661	672	TTGGCCTCACGATAGCTCTAC

scaffold714558_14.3	1	p2	(TA)8	16	742	757	GTGCCTGAACCACCAACTTT
scaffold714562_17.4	1	p2	(TC)6	12	426	437	ACGGCTAGATTCGATCCTGA
scaffold714615_16.0	1	p2	(AG)7	14	479	492	CACCTTTTTCTTCCCCAGGT
scaffold714619_14.2	1	p2	(TA)6	12	1670	1681	CACAACGAATTCCTACGGGT
scaffold714626_13.1	1	p2	(CT)10	20	75	94	TTCAAACCTTTGCCACTTCC
scaffold714655_15.2	1	p2	(AC)6	12	2838	2849	TCAACGAGGAAATCGCTTCT
scaffold714676_17.2	1	p2	(AC)6	12	3529	3540	TGGAACCAACTCGTCAGACA
scaffold714683_11.1	1	p2	(CT)7	14	995	1008	AGAAGCAGTTGGGATGATGG
scaffold714692_16.0	1	p2	(TA)6	12	153	164	ATGCCCATCTCATTTCAAGG
scaffold714694_13.5	1	p2	(TA)9	18	1029	1046	AAGGGATGGTATCATTTGATCG
scaffold714699_16.8	1	p2	(CT)10	20	3692	3711	TCTTATTGGGTTGTCCGAGG
scaffold714740_17.2	1	p2	(AG)9	18	344	361	AAAGAGAGAGTGCATGCGTGT
scaffold714751_12.4	1	p2	(TG)8	16	35	50	TGAGTGTGTGTCTCGTGTGTG
scaffold714756_16.0	1	p2	(AG)6	12	349	360	TTTGAAGGAAAACTCGTCCC
scaffold714781_16.5	1	p2	(AG)7	14	348	361	GTGCCTGCTCCACTTTCTTC
scaffold714824_12.7	1	p2	(GA)6	12	827	838	CTTCCATACTAGCTTCGCCG
scaffold714835_12.7	1	p2	(AT)10	20	1037	1056	TGCAAGTTCCGAGTTCAAGTT
scaffold714878_15.7	1	p2	(TA)6	12	1875	1886	TCTCAGCTCGATAAGTGGCA
scaffold714887_13.9	1	p2	(TA)6	12	287	298	CATATCCTTTGAACCGGCAC
scaffold714908_17.6	1	p2	(CT)7	14	349	362	TCGATCTGGGCTCTCTTGTT
scaffold714948_18.4	1	p2	(AG)7	14	324	337	GTGGAGAGTGAGGAAGCAGG
scaffold714956_14.7	1	p2	(GA)8	16	3692	3707	TTGGAAGAGGAAAGTGGGTG
scaffold714965_14.5	1	p2	(TA)6	12	685	696	CCACGATGATTTCGATACACA
scaffold714981_16.7	1	p2	(TA)8	16	683	698	ATTCACAGCCCTTCATCTCG
scaffold714990_11.9	1	p2	(TA)8	16	67	82	GGTCCATAAGTGTTTTGAGTTATGA
scaffold715022_20.1	1	p2	(AT)6	12	966	977	TTGGTCTTGGTCACGATTCTC
scaffold715072_11.7	1	p2	(GA)6	12	460	471	CGGAGAGATTTGTGGGGTTA
scaffold715088_16.1	1	p2	(TC)6	12	661	672	TTGGTGAAACCAAGCATGTG
scaffold715100_14.6	1	p2	(AG)6	12	110	121	CTGGACGCATTAGTGGGTTT
scaffold715124_12.8	1	p2	(TA)10	20	885	904	TATAAGGATCAACCGCCTGC
scaffold715127_14.5	1	p2	(TG)9	18	1286	1303	GGGGCTTCAGATGAAATCAA
scaffold715128_14.7	1	p2	(GA)7	14	349	362	AGCATCAGACCAATGTGCAA
scaffold715180_14.3	1	p2	(TC)6	12	1116	1127	TGGTGCAGATGTCCTGGTTA
scaffold715184_16.8	1	p2	(AT)7	14	1330	1343	CTACGCAGGGGCGTATGTAT
scaffold715227_15.7	1	p2	(AG)7	14	3053	3066	GTGATCGCTGGTTCTTTCGT
scaffold715253_15.5	1	p2	(GA)6	12	3360	3371	AGGTTGCGTCAGATTGGAAG
scaffold715292_17.2	1	p2	(GT)6	12	621	632	AGTATGCTTTGGCCGATCC
scaffold715301_12.1	1	p2	(TA)10	20	717	736	AATGATGTTAATCCAGCGGC
scaffold715305_11.7	1	p2	(TC)6	12	697	708	GCCACTCCATCACCATAACC
scaffold715329_17.5	1	p2	(AG)7	14	2663	2676	GCAGCAACATCTCTCGTGAA
scaffold715360_14.3	1	p2	(AT)6	12	185	196	TGCGAGTATCAAAACCGAGC
scaffold715374_14.2	1	p2	(AG)8	16	216	231	GTGGGAACGTGTAGTCGGAG
scaffold715418_13.8	1	p2	(TA)12	24	252	275	TCATATCATTGTTTCCGCGA
scaffold715443_18.7	1	p2	(AG)7	14	1517	1530	TTCCACCTCAAAAACAAATGG
scaffold715478_15.7	1	p2	(TC)8	16	901	916	AATCACGCGAGGAAATCAAC
scaffold715479_15.6	1	p2	(TA)6	12	3963	3974	GGGTGGAATGGTGGTGTTAC
scaffold715575_14.2	1	p2	(CT)8	16	3504	3519	GCATTGGAGATGGAGCAAAT
scaffold715590_14.7	1	p2	(TC)9	18	999	1016	GGTGGTGTTTGCCTGTCTCT
scaffold715599_14.0	1	p2	(CT)7	14	352	365	CCCAACTGGAAAAGTCCAAA
scaffold715605_11.4	1	p2	(TA)10	20	333	352	CGCAGATCCCAGATTTGTTC
scaffold715640_14.3	1	p2	(TA)6	12	1113	1124	TGAAATTGTGTTGCTTGATCG
scaffold715650_17.1	1	p2	(TA)6	12	705	716	TGAAGTTAGCACCTGAGGATGA
scaffold715713_16.4	1	p2	(GT)8	16	135	150	CAATGAGGTGTCTGCCCTCT
scaffold715742_17.8	1	p2	(AT)7	14	1970	1983	CCCGAACTCCGAGTACAAAA
scaffold715746_15.9	1	p2	(GA)6	12	80	91	TGCTGATCTATTTACATGCTTTTCA
scaffold715755_16.5	1	p2	(AG)6	12	481	492	AGGAGAGTGCAGCTGGTCAT
scaffold715758_14.2	1	p2	(CT)9	18	687	704	AGATTGAAGCTCGGATGGTG
scaffold715820_15.7	1	p2	(TC)7	14	1431	1444	TCACGTGGTGGAGACAAAAA

scaffold715836_14.3	1	p2	(AT)6	12	357	368	TGGTTTGAGCAAATTGATATTTTA
scaffold715840_16.2	1	p2	(AT)9	18	1081	1098	TTGTTGTCCATACATTTTGGAAT
scaffold715876_14.8	1	p2	(AT)7	14	2339	2352	ACCCAACTGGGTAAACCCTT
scaffold715902_16.9	1	p2	(AC)7	14	580	593	CTGGGGATGGATTGATGAAG
scaffold715953_17.0	1	p2	(TC)7	14	603	616	AAAATCCTTTCCACAACCCC
scaffold715989_16.6	1	p2	(GA)9	18	828	845	GATGTGGGTGGTTTGCTTCT
scaffold716007_22.9	1	p2	(GA)9	18	50	67	AGGGGTCCAAAAGCAGAAGT
scaffold716031_10.7	1	p2	(AT)7	14	224	237	TTGACATCGCAATCAACCAT
scaffold716048_15.6	1	p2	(AT)10	20	329	348	TTTCTGCACAGATTTGATGGTC
scaffold716057_16.1	1	p2	(GA)7	14	1010	1023	AACCGGGCAAGAAGAAAACT
scaffold716124_16.8	1	p2	(AT)6	12	565	576	TGAATTTCCTGTCATGCGTT
scaffold716130_16.7	1	p2	(TC)6	12	1021	1032	GCTTCGATGTTGATGCTTGA
scaffold716133_15.0	1	p2	(TC)8	16	1252	1267	TTTTGCCATGTGTATCGTTGA
scaffold716153_10.8	1	p2	(TA)11	22	43	64	CCCAAAAATGGGTTATTATTTGA
scaffold716155_16.0	1	p2	(CT)8	16	827	842	CCCATACACACAAGGCAGTG
scaffold716180_15.9	1	p2	(CA)8	16	2317	2332	ATTGATGCCCTGGTTACAGC
scaffold716229_15.6	1	p2	(CT)6	12	1858	1869	TGCCAGAACTTACCGACCTAA
scaffold716249_10.8	1	p2	(AT)12	24	936	959	CCAAATTTTGATCAATTTCCAA
scaffold716250_12.3	1	p2	(TA)8	16	1001	1016	CTCTGTGTGTGATTTCCGCT
scaffold716251_14.2	1	p2	(AG)8	16	1152	1167	ACGAGGGGTTTCTCTCGTTT
scaffold716269_14.2	1	p2	(AT)10	20	549	568	ATCTTGCATCGGCAAGAGAC
scaffold716273_21.7	1	p2	(TA)8	16	83	98	AAAATCGATATTGAATTAAAGGCTC
scaffold716275_15.8	1	p2	(AT)6	12	1709	1720	TTCACAAAGGGCTTTTCAGAC
scaffold716279_14.5	1	p2	(AG)10	20	134	153	TTGGGTGGAATAGACTTGGG
scaffold716307_14.7	1	p2	(TA)7	14	32	45	TCAACATCATCATCAACATACCC
scaffold716341_16.0	1	p2	(AG)6	12	848	859	AGCACTGCAACGCAACATAC
scaffold716344_17.6	1	p2	(AG)6	12	1019	1030	GGGCCGTACTTTCTGTCAAA
scaffold716365_16.2	1	p2	(TA)10	20	45	64	TAAGGGCGGTATCACCTGAA
scaffold716403_16.0	1	p2	(AG)7	14	2575	2588	TGAACCGAAAATTGGGTAGG
scaffold716461_16.4	1	p2	(AG)8	16	835	850	TCAAAACCGGTGATTAACTGC
scaffold716462_16.3	1	p2	(GT)8	16	694	709	TCTGATCATTGAGGAAGGGG
scaffold716518_17.8	1	p2	(AG)7	14	452	465	TGTTTTCGTTCTGAGAGGGG
scaffold716564_13.9	1	p2	(TC)6	12	475	486	GGTTCCCAGTTGGCATGTAA
scaffold716609_14.7	1	p2	(GC)6	12	240	251	CCGAAGCCCAAAAACAAATA
scaffold716610_17.0	1	p2	(CT)6	12	724	735	AAGTCCATTTCCACCCCTTC
scaffold716635_15.9	1	p2	(CT)6	12	233	244	GTTTCACAGGAGGCGGTTTA
scaffold716657_14.2	1	p2	(AG)10	20	1253	1272	CAGATTCAAAGACGGCGTTC
scaffold716687_17.7	1	p2	(CT)7	14	2466	2479	AGATGCTGAAACCTGGCATT
scaffold716768_17.4	1	p2	(CA)7	14	173	186	CGTGGTTGTAGGTCGGAGTT
scaffold716774_17.2	1	p2	(AG)6	12	362	373	ATCGAACAATTCCTTCCACG
scaffold716781_12.9	1	p2	(AT)7	14	1698	1711	CGCGAGATTCGATGGTAGAG
scaffold716808_12.2	1	p2	(TA)9	18	177	194	ACTTCGATCACTTCGTTCCC
scaffold716883_20.0	1	p2	(AT)6	12	626	637	ATATGTTGCAGGCCGAGAAG
scaffold716924_15.5	1	p2	(AT)7	14	2042	2055	CCCACACACAATGCAAGAAT
scaffold716938_17.4	1	p2	(GA)6	12	1175	1186	ACCCCTTTTACCCTTTCCCT
scaffold716979_15.8	1	p2	(GA)8	16	836	851	AGTCAGCACGCAATTTCTCA
scaffold717043_12.9	1	p2	(TA)6	12	467	478	TCAGACATTTCGATCCCAGA
scaffold717124_15.3	1	p2	(CT)10	20	1653	1672	AAGTTCCACTTGTTCACGGC
scaffold717140_15.3	1	p2	(CT)6	12	1536	1547	CAAGCCCAAATCAAGGAAAA
scaffold717150_11.1	1	p2	(TC)6	12	54	65	CTCCTCTTTCTCCCTCTCCTC
scaffold717156_17.9	1	p2	(TA)6	12	626	637	GTTCCCTGGAAACCATTTCA
scaffold717164_13.5	1	p2	(GA)8	16	817	832	AGGGAAAGATCCCTGCTTGT
scaffold717169_12.7	1	p2	(GA)6	12	575	586	TCAATTCCCAAACCATATCCA
scaffold717207_15.5	1	p2	(AG)7	14	544	557	TCAGCTTGTTAGTGTTCAGTTTGA
scaffold717256_11.6	1	p2	(AT)11	22	81	102	CCTGCATGTGCAAATTGAAA
scaffold717265_16.9	1	p2	(GA)7	14	1964	1977	GCAGCATTGATCTAGAGGGC
scaffold717281_16.0	1	p2	(AT)10	20	663	682	TGTTGCGTCTTGTATTGGAGTT
scaffold717327_12.1	1	p2	(TG)6	12	342	353	TCAAAACATTTATTTATGCACACATTT

scaffold717353_17.7	1	p2	(TG)9	18	3424	3441	CCAATGATGGGTAACGGAAT
scaffold717361_14.3	1	p2	(TA)7	14	867	880	GTTTATGTTGCGAAATCGGC
scaffold717363_12.8	1	p2	(AT)12	24	1000	1023	TTCGCACAAATACAAATCGC
scaffold717381_16.9	1	p2	(AC)6	12	367	378	ACATGCAATGAAAATGCCAA
scaffold717500_14.2	1	p2	(CT)6	12	368	379	ATTCTATGGTGCCCCCTCTC
scaffold717502_13.6	1	p2	(CA)6	12	1529	1540	CACGAAAATTTTGCATATCGG
scaffold717528_16.1	1	p2	(CT)8	16	279	294	GAATGATGCATGCTCTCACC
scaffold717532_16.0	1	p2	(AT)7	14	447	460	GAAGGTGACGTGTTGCAGAA
scaffold717571_14.6	1	p2	(AT)6	12	205	216	GGATCTTCTCATTCCATCGAG
scaffold717637_18.6	1	p2	(AC)8	16	730	745	CAGCAGCACCATACCAGAAA
scaffold717700_15.4	1	p2	(TC)6	12	321	332	AATCTGCTGGTGTTCCTTGG
scaffold717733_11.6	1	p2	(AT)7	14	368	381	GAATGAGGGTTTTTGTGGGA
scaffold717737_15.5	1	p2	(AG)6	12	1744	1755	CTTTCCCTGCGTTGCATAAT
scaffold717739_15.0	1	p2	(TC)9	18	355	372	TCCCTGACCACCATCAATTT
scaffold717774_13.9	1	p2	(AT)9	18	891	908	TGAGTGGATATATTGGGAAGGG
scaffold717801_15.2	1	p2	(CT)7	14	1336	1349	ATCTACCGGTTTTCCCCATC
scaffold717845_14.6	1	p2	(AG)6	12	2152	2163	CGATTTTGTTTGCCGAAAAT
scaffold717847_13.6	1	p2	(GT)6	12	334	345	AAGCGATGCTCCAACTTGAT
scaffold717880_16.3	1	p2	(AT)7	14	1227	1240	ACTGTTGATGCAGATGGTGG
scaffold717884_16.6	1	p2	(TA)6	12	709	720	AGAGAGAACTGGGGTCAGCA
scaffold717932_13.4	1	p2	(CT)7	14	756	769	TCTCTCCCTTCTTCTGCTTCA
scaffold717936_16.3	1	p2	(GA)7	14	226	239	GAAAGAAAAGTGTTGGGGGC
scaffold718021_14.0	1	p2	(CA)6	12	570	581	CAAGATGGCATCATCCATTG
scaffold718114_16.3	1	p2	(TA)6	12	601	612	ATAAGCAGCATTTGGCCTTG
scaffold718123_16.1	1	p2	(TA)7	14	670	683	TGTTCTTGTCCGTCGGATTT
scaffold718173_15.5	1	p2	(TA)9	18	45	62	CCAATTTGAATGGTTATCACCTG
scaffold718188_16.4	1	p2	(AG)7	14	2607	2620	CGGGTAAATTTGAGGTGGAA
scaffold718304_15.4	1	p2	(CA)6	12	509	520	TATGCTCGCTGGATTACGTG
scaffold718336_15.4	1	p2	(TC)7	14	337	350	CTTGCAGTTTCCTCACCTCC
scaffold718348_18.8	1	p2	(AC)7	14	372	385	AAACAACGATGGCAACAACA
scaffold718362_18.4	1	p2	(CT)6	12	2439	2450	TCATAGATTTGGGCATGCAA
scaffold718389_17.9	1	p2	(CT)6	12	67	78	GCACAACCACTCCACAAAGA
scaffold718410_11.8	1	p2	(TA)10	20	963	982	TTTTGATAATGGTTTTGTACACATTTT
scaffold718424_10.0	1	p2	(TA)6	12	67	78	TCTAAGGGCCGAGATTTGTT
scaffold718446_16.9	1	p2	(TC)6	12	1427	1438	TGGATATTGAGAGGCTGGCT
scaffold718451_16.2	1	p2	(TA)8	16	586	601	GGAGCTCGAGAGATATTGCG
scaffold718482_15.6	1	p2	(TC)6	12	632	643	TGCTTTATGCCTTGCCTCTT
scaffold718486_13.7	1	p2	(AT)11	22	178	199	GGGGTGTTTTGACAATATTACGA
scaffold718500_14.0	1	p2	(GT)7	14	64	77	GGCAGTCGTTTTGAGACGTT
scaffold718511_13.0	1	p2	(TA)7	14	260	273	TCGCGGGAATCAACTTTATC
scaffold718570_17.3	1	p2	(AT)6	12	1277	1288	CGGCACGTGACTCACTTAAA
scaffold718622_16.4	1	p2	(TC)6	12	1819	1830	TTCCTGAGTTGGTGAAAACG
scaffold718625_14.4	1	p2	(GA)6	12	2462	2473	TGGGGAAGAAAAGGAGAGGT
scaffold718629_12.4	1	p2	(AC)8	16	492	507	TTGCTATTGAGAGGAAGCGA
scaffold718636_12.0	1	p2	(TC)6	12	989	1000	CTTTGGCAAATCAGGCGTAT
scaffold718639_15.2	1	p2	(TG)6	12	3020	3031	AGGCGCTTCTCTCAGGTGTA
scaffold718648_14.5	1	p2	(AT)10	20	101	120	CGGAAACCGCCATAAAGTT
scaffold718652_14.1	1	p2	(TC)7	14	792	805	CCAGACAAAATCCCCTCTCA
scaffold718662_16.4	1	p2	(CA)6	12	1620	1631	TTCACATGATCTTGGATGGG
scaffold718667_16.3	1	p2	(TA)13	26	1349	1374	TTTTCTGCATGATTTGGATGA
scaffold718678_22.5	1	p2	(CT)10	20	340	359	AAACGATTTTTGATCGTGGC
scaffold718682_16.3	1	p2	(AT)10	20	2035	2054	TTGAGCTGGTTGATTCATTTTG
scaffold718698_16.0	1	p2	(AT)6	12	87	98	GTGGGCGCAAGTAAAGAGAG
scaffold718715_17.2	1	p2	(TG)6	12	2252	2263	TTGAACTTGTTTCAAATCCATGA
scaffold718726_16.1	1	p2	(AG)6	12	970	981	TTTGCTCTCTCACACATGCC
scaffold718757_14.8	1	p2	(CT)6	12	378	389	CAGTGCGTGTGTGTGTCAAG
scaffold718777_15.3	1	p2	(CT)6	12	845	856	ATCACCCCCTCTCCAAGAAT
scaffold718782_14.5	1	p2	(GA)6	12	72	83	TTCGAACTTCCATCCTGCTT

scaffold718786_15.[illegible]	1	p2	(GA)7	14	1403	1416	ACCGCCGTCTGTAATAATGC
scaffold718804_16.[illegible]	1	p2	(CT)7	14	123	136	AATTGCCTTGTGTTTCTGGG
scaffold718817_14.[illegible]	1	p2	(CA)6	12	2597	2608	TGCTGTGGAATACCTGGGTT
scaffold718827_19.[illegible]	1	p2	(AG)6	12	854	865	GAGAGGAGGAGGAGAATGCC
scaffold718856_13.[illegible]	1	p2	(GT)6	12	263	274	GCTGAGATATACGCGGTGGT
scaffold718893_14.[illegible]	1	p2	(CT)8	16	33	48	TGATTCTATTCTGTGCTTCGGA
scaffold718902_14.[illegible]	1	p2	(AT)9	18	1327	1344	GTTCTGCCGAGATGAGGAAG
scaffold718905_10.[illegible]	1	p2	(AT)10	20	189	208	CTATGCTGCCAAGTGCTCAA
scaffold718933_11.[illegible]	1	p2	(CT)7	14	503	516	TGGGCCAAACCCTATATCAA
scaffold718943_12.[illegible]	1	p2	(GA)8	16	1385	1400	ATGTCCGAGTTGTTCCCAAG
scaffold718963_14.[illegible]	1	p2	(TG)6	12	1389	1400	AGATCTCGAGCAGCCAACAT
scaffold718990_16.[illegible]	1	p2	(AT)8	16	954	969	GGTTGATCGTAATGGGATGG
scaffold719002_14.[illegible]	1	p2	(GA)6	12	1622	1633	CTCCTTCCGTTTGTTGAGGA
scaffold719044_15.[illegible]	1	p2	(AG)6	12	529	540	AGTTTGGAGGCCTGTCAATG
scaffold719046_14.[illegible]	1	p2	(AT)6	12	1603	1614	AATTGTTCAACCTCTCCGCT
scaffold719064_12.[illegible]	1	p2	(AC)6	12	1327	1338	CATGTGGTTGAATTTCGTTCA
scaffold719079_17.[illegible]	1	p2	(GA)8	16	4814	4829	CGCAAAAGCAAGACACAAAC
scaffold719090_13.[illegible]	1	p2	(TA)7	14	378	391	GAATCAACATTTCATCGCCA
scaffold719091_16.[illegible]	1	p2	(TA)10	20	372	391	CATTGTGATAGGGTTCCGCT
scaffold719132_12.[illegible]	1	p2	(AT)9	18	294	311	CACATATAATCAGGGGCGGA
scaffold719152_12.[illegible]	1	p2	(TA)6	12	668	679	AGCGCAAACCAAACAAAAAC
scaffold719157_15.[illegible]	1	p2	(GA)7	14	924	937	TGGGTGGGAGATAAGAGGTG
scaffold719203_16.[illegible]	1	p2	(TC)9	18	2585	2602	AAAAACCAGCACCAATCCAG
scaffold719206_15.[illegible]	1	p2	(AT)9	18	276	293	CCCCTAGGCATGGATTATCA
scaffold719243_14.[illegible]	1	p2	(CT)6	12	1000	1011	TCATCCAAGTTCATGCAAGG
scaffold719277_15.[illegible]	1	p2	(TA)6	12	2461	2472	CAACTGGCTATGTTGCTCCA
scaffold719304_11.[illegible]	1	p2	(GA)11	22	373	394	AATAATGGCGGGTACGTTGA
scaffold719358_17.[illegible]	1	p2	(CA)6	12	3927	3938	TCAACTGCTGCAAATGATCC
scaffold719361_15.[illegible]	1	p2	(TC)8	16	221	236	GCGTCTCACCTAACCTCACC
scaffold719370_17.[illegible]	1	p2	(AT)6	12	382	393	TCCCCAGAGCTTGAGAGAGA
scaffold719390_12.[illegible]	1	p2	(TA)10	20	42	61	TTTCCATGTAATAAAAAGTTTCCCA
scaffold719407_15.[illegible]	1	p2	(CA)10	20	286	305	GGTCCAGATCATGGGTCACT
scaffold719443_13.[illegible]	1	p2	(AT)9	18	68	85	TCCGATGGTTGTAGTGGAGTT
scaffold719448_15.[illegible]	1	p2	(AT)7	14	125	138	GTCGGCGTTTTCAGCACT
scaffold719457_12.[illegible]	1	p2	(TC)6	12	262	273	CCCTTCACAAGTGGTGACAA
scaffold719460_15.[illegible]	1	p2	(TA)6	12	201	212	ATATGGGATTTTGCATGGGA
scaffold719497_15.[illegible]	1	p2	(AT)8	16	379	394	AATGACTCAGAATTTGGCCG
scaffold719498_17.[illegible]	1	p2	(TC)8	16	4573	4588	ATGGCATGACTCTTGAGCCT
scaffold719535_14.[illegible]	1	p2	(TA)9	18	2305	2322	TTCTGATATGGAAATGCCCC
scaffold719545_15.[illegible]	1	p2	(AG)8	16	1542	1557	GACGTGGTTTGCAGGCTATT
scaffold719565_13.[illegible]	1	p2	(TA)7	14	125	138	GGTTTCCCTCCCAAAAACAT
scaffold719586_13.[illegible]	1	p2	(AT)6	12	1018	1029	CCTGAGATTGGTCGAGGGTA
scaffold719616_14.[illegible]	1	p2	(CT)6	12	431	442	TTCAGACTCCAAAAACAAAGCA
scaffold719641_15.[illegible]	1	p2	(AT)8	16	380	395	CTAGCCATCCATTCCGAAAA
scaffold719648_15.[illegible]	1	p2	(TC)7	14	93	106	AGAAAGTTGCAGGCTGGCT
scaffold719703_16.[illegible]	1	p2	(TC)8	16	381	396	CCACCATTTTTATTCCCCCT
scaffold719733_15.[illegible]	1	p2	(AT)6	12	1800	1811	TCCAAACTCCAACCTCCATAA
scaffold719739_16.[illegible]	1	p2	(TA)6	12	1158	1169	GCATGATCAAAGAGGCCATT
scaffold719759_16.[illegible]	1	p2	(TC)6	12	849	860	TCCTTGGCATACATTCTCTCTC
scaffold719777_14.[illegible]	1	p2	(CT)6	12	519	530	AGCAACCAGCTGCATTAACC
scaffold719794_16.[illegible]	1	p2	(TC)8	16	846	861	TCCCACCACCTCTACCCATA
scaffold719805_12.[illegible]	1	p2	(CT)6	12	1093	1104	GGAATGTGATTGGGGAAGAA
scaffold719807_17.[illegible]	1	p2	(TA)7	14	52	65	CACAGCTGTCAATCAACAAGAA
scaffold719813_18.[illegible]	1	p2	(TC)8	16	382	397	CTGTCTCAGTCACCGTCGAA
scaffold719821_10.[illegible]	1	p2	(TA)11	22	358	379	TTACACTGCAGTACGTCGCC
scaffold719856_17.[illegible]	1	p2	(AT)6	12	1432	1443	GACTTCTGGTGGACGACGAT
scaffold719898_16.[illegible]	1	p2	(AG)6	12	526	537	AGTCGCGAGTCTCTCTCTCG
scaffold719933_14.[illegible]	1	p2	(TG)12	24	368	391	TTCGCCATATCCATGTTTCA

scaffold719943_13.9	1	p2	(CT)13	26	34	59	CCACCCTCCATTGCTCATAA
scaffold719956_18.0	1	p2	(CT)9	18	2164	2181	TTTGCCTTTGATCGACACTG
scaffold719988_15.1	1	p2	(CA)9	18	723	740	CCCGGTCTATCTATGGGGAT
scaffold719999_12.1	1	p2	(TA)10	20	2000	2019	ACGCGTGTGACATGTTCCTA
scaffold720024_15.1	1	p2	(AC)9	18	615	632	CGCGAGACATGAACGAATAA
scaffold720031_11.0	1	p2	(TC)6	12	556	567	TTTGGCCTTGTCCTCGTATAA
scaffold720053_15.8	1	p2	(AT)6	12	1975	1986	TCGATTGTATTTAAGCACGCA
scaffold720067_15.2	1	p2	(TA)6	12	280	291	TGTCACTGCACTACGAGATGAA
scaffold720078_11.0	1	p2	(GT)7	14	1113	1126	ACCCCTTTGTCGATTCTCCT
scaffold720087_17.9	1	p2	(TC)6	12	374	385	CCCATAAACCACACTAAAGATGG
scaffold720091_12.8	1	p2	(TA)8	16	899	914	CAAGGCAAGACTTGTGGTCA
scaffold720162_17.6	1	p2	(CT)6	12	361	372	TCCCGTGAGGTAAACATCCT
scaffold720163_15.7	1	p2	(TC)6	12	180	191	CCGACATTCAGCAGATGAGA
scaffold720170_14.9	1	p2	(CA)6	12	1717	1728	GAACCTTCGGACAGCTCTTG
scaffold720176_14.0	1	p2	(TC)7	14	388	401	AGTGATCACACCTGCCCATT
scaffold720209_13.7	1	p2	(TA)6	12	390	401	AAACATGGAATGAGTTAAGGGAA
scaffold720219_14.0	1	p2	(TC)7	14	388	401	GGTGTGCCTTGTCCAATTTT
scaffold720224_18.0	1	p2	(TC)8	16	494	509	AAGAGGGCCAGGAACAAAAT
scaffold720255_15.7	1	p2	(AG)7	14	1741	1754	CACGCTGGCCATATACCATA
scaffold720298_15.0	1	p2	(TA)6	12	786	797	TCAAACCATTATACCTACCGCA
scaffold720305_15.3	1	p2	(TG)9	18	984	1001	CTGCTTATCTTTTCGGGCTG
scaffold720313_12.3	1	p2	(TC)8	16	613	628	CTACGTAGCTCGGTTCTCCG
scaffold720358_13.2	1	p2	(AT)8	16	230	245	CTTTCTCCTCACGCTCTGCT
scaffold720386_16.0	1	p2	(GA)6	12	223	234	GAGTGGAGGATTCGGTGAAA
scaffold720477_16.3	1	p2	(AG)6	12	3070	3081	TGGAGCAGATCCCAGTCTCT
scaffold720499_17.9	1	p2	(GA)7	14	6856	6869	TGAGGCCAATTCCTCAGTGT
scaffold720502_11.6	1	p2	(CT)9	18	121	138	ACTACCACCAGCACCTCCAC
scaffold720507_11.9	1	p2	(TA)9	18	171	188	AACAAACGAAAGGGACCACA
scaffold720519_14.8	1	p2	(GA)8	16	855	870	TCAGCAGCCATTGAGACATC
scaffold720581_14.7	1	p2	(AT)6	12	689	700	ACAAAACAAAACAGGGGCAA
scaffold720585_14.6	1	p2	(TG)6	12	37	48	CGAACCACGTTAAATCTCTGTG
scaffold720594_17.6	1	p2	(TC)8	16	923	938	AGCTCAATGCACTTCCCAAT
scaffold720600_16.6	1	p2	(CT)7	14	159	172	TAATCGTGTTGCATTGCCTC
scaffold720617_12.6	1	p2	(GA)6	12	359	370	AGGAGTCGGAGTGAAAAGCA
scaffold720619_14.6	1	p2	(AT)9	18	724	741	TTTGGCGATATGCAATCAAA
scaffold720658_14.0	1	p2	(TA)8	16	1694	1709	AAATCCGTCCCAATATTCCC
scaffold720698_15.9	1	p2	(TC)8	16	820	835	TGCTGAGACAGTGGAAGGTG
scaffold720701_16.7	1	p2	(AT)7	14	1458	1471	GCGTGGTTGTGAGCGTATAA
scaffold720705_16.8	1	p2	(GA)7	14	191	204	CTGCTTTGTGTTGCGTGTTT
scaffold720706_17.9	1	p2	(CT)8	16	2734	2749	CATGGCAGTTTCACATTTGC
scaffold720716_17.4	1	p2	(TA)9	18	388	405	TTTACAAGATCAATGGTCCCAA
scaffold720794_15.2	1	p2	(TA)10	20	80	99	ACGGCTGCAAAAGTTTCCTA
scaffold720839_14.1	1	p2	(AT)6	12	1458	1469	TCGACGTCTACAAAACGCAC
scaffold720850_16.0	1	p2	(GT)6	12	768	779	AGGAGGACGAGTGTGGTGAC
scaffold720886_17.6	1	p2	(TA)8	16	1738	1753	CATTTTTAATTGCAAAATTAATCCA
scaffold720915_14.5	1	p2	(AT)7	14	1270	1283	TGCATGTTCAATTGGGAAAA
scaffold720924_15.0	1	p2	(TA)9	18	44	61	AAGGGTTATCACCTGATTTTCTC
scaffold720941_14.5	1	p2	(TC)6	12	2816	2827	TAGCAAAAACGAAGCCACCT
scaffold720958_17.0	1	p2	(TA)6	12	592	603	CCCAAAATCAACGTCCAGAT
scaffold720960_16.5	1	p2	(TG)8	16	3737	3752	ACGATCATCAAAGAATCGGC
scaffold720978_15.3	1	p2	(GA)12	24	737	760	TTGCCTCTGCAAGAATTGTG
scaffold721021_16.5	1	p2	(TA)10	20	3781	3800	ACAAACCACTGAATCGCACA
scaffold721023_19.8	1	p2	(TA)7	14	237	250	TCTGCGTTACTTGCTTTCCA
scaffold721025_12.5	1	p2	(TA)7	14	521	534	TGAATATTGATTGGTCCATGC
scaffold721030_16.4	1	p2	(GA)6	12	1696	1707	TCTGCTTTCACACCAAGTCG
scaffold721084_15.0	1	p2	(AG)7	14	3577	3590	ATGTGCACAGCGAGGTATTG
scaffold721100_18.3	1	p2	(AC)6	12	931	942	AGGCCAGAAACTGCTCAAAA
scaffold721103_15.1	1	p2	(GT)6	12	2092	2103	CCCCTATGAAGCCCAGAAA

scaffold721114_15.4	1	p2	(TC)7	14	143	156	ATCACTTCCGATCGAACCAC
scaffold721167_13.0	1	p2	(TC)7	14	647	660	ACTACGGCCACATGAGCTTT
scaffold721191_19.2	1	p2	(TC)6	12	1026	1037	ATTTGTTCACCCTGGACGAT
scaffold721203_17.0	1	p2	(AT)6	12	109	120	AGATTCTCACGCCATTCTCAA
scaffold721213_14.7	1	p2	(TC)8	16	627	642	GCGTGGCATATTTCTGATGA
scaffold721226_14.0	1	p2	(CT)6	12	722	733	TCTGGACAAGAAGCCATTGA
scaffold721287_14.6	1	p2	(AG)8	16	1473	1488	CTGTTTCCTGTCCAATTCCC
scaffold721307_13.2	1	p2	(TA)9	18	513	530	TTTTCATTATTTATTGCAAGTTTTCG
scaffold721352_16.2	1	p2	(GA)8	16	848	863	GTCGGCAATTTCTTGATGCT
scaffold721357_13.9	1	p2	(AT)6	12	585	596	TTGTTGAACGTCTCCAACTCC
scaffold721399_14.6	1	p2	(AG)10	20	1360	1379	GGGAGTTGAGGGATCACAAA
scaffold721428_14.2	1	p2	(TG)6	12	644	655	GAGCAGAAACTTGCGGAGTC
scaffold721463_15.2	1	p2	(CT)9	18	984	1001	CCGTGGTGATTCTTTGGACT
scaffold721466_12.9	1	p2	(TA)10	20	654	673	GTTAACCTTGCAAGCTGCCT
scaffold721496_11.9	1	p2	(GA)7	14	728	741	CTTGCTCCTAATCGTCGGTC
scaffold721498_14.1	1	p2	(CT)7	14	898	911	GCCATGAACTATATTGATTTTCCA
scaffold721576_15.3	1	p2	(GA)6	12	6102	6113	GCCTCAAAATATCTGCTCCAA
scaffold721578_11.4	1	p2	(TA)6	12	347	358	ATTGGTTTGGCAAAAGCCTC
scaffold721635_11.8	1	p2	(AT)9	18	1972	1989	TCCTTTCTCAAATGAAGAGAAACA
scaffold721671_13.0	1	p2	(AG)7	14	871	884	TGCAGAGATTTGTGGCAGAC
scaffold721673_10.5	1	p2	(AT)6	12	402	413	TTTTCAGTGGTGTTTGTTGTTT
scaffold721676_11.5	1	p2	(AT)7	14	1133	1146	TATAAAAATGCAGCCAGGGG
scaffold721688_15.8	1	p2	(AG)9	18	3466	3483	TGCATTTTTCAGCATTTCCA
scaffold721720_14.5	1	p2	(AT)12	24	1403	1426	AAAAACGCGGAAATCACATC
scaffold721725_16.4	1	p2	(AG)7	14	1123	1136	GTCGTCGTTTCCACCTCTGT
scaffold721728_14.5	1	p2	(CT)7	14	402	415	GCGGCTCTGCAACTTTTAGT
scaffold721781_16.2	1	p2	(TA)8	16	1424	1439	GCCTTGTCCGTTGTCAGAAT
scaffold721823_14.3	1	p2	(CT)12	24	1036	1059	GCCATTCTCCTTCAAAACCA
scaffold721828_13.0	1	p2	(AG)9	18	398	415	GCCCAAGATACTCCATCCAA
scaffold721837_16.5	1	p2	(CA)7	14	3259	3272	AGGCATTGGTTTGTTGGAAG
scaffold721852_17.5	1	p2	(TA)9	18	47	64	AAGGAGGTGTTATGAAGGGATT
scaffold721854_14.9	1	p2	(GA)9	18	3391	3408	CGCTTGTTCCTTGGTAGGAG
scaffold721863_18.9	1	p2	(GA)6	12	105	116	GGTTGATGTCATCGTCATGG
scaffold721872_13.3	1	p2	(AT)9	18	202	219	ATTTTGACGGGATTCAGGTG
scaffold721878_13.9	1	p2	(GA)10	20	3282	3301	CAAGCAAGAAAAAGCGAACC
scaffold721888_15.3	1	p2	(TG)6	12	925	936	TGAAAGGTAGGGTTTTCGCA
scaffold721889_15.4	1	p2	(TA)6	12	656	667	TGCAAGTAAAATCGTCGTGC
scaffold721919_16.1	1	p2	(AG)6	12	6480	6491	TGAAGTTGACGAAGAAGATGACA
scaffold721938_15.3	1	p2	(TA)6	12	627	638	CCTAAAATTGCCCCATCCTA
scaffold721941_10.7	1	p2	(TA)8	16	566	581	TTGAATCTCCGGCTGAAACT
scaffold721958_14.7	1	p2	(AG)7	14	404	417	TTGGCCACAAATTAAAAGGG
scaffold721962_16.1	1	p2	(AT)6	12	2942	2953	TGAAACTCTCAACAGATTCCCA
scaffold721978_16.6	1	p2	(TA)9	18	47	64	CACTTAAGGGGGTTATTAGTGGA
scaffold721979_16.6	1	p2	(GT)6	12	106	117	TGGGAACACTCTTCAGGACA
scaffold722010_14.4	1	p2	(AT)8	16	1086	1101	TTTTCATTGGAGTTGTGCCA
scaffold722011_14.7	1	p2	(TA)6	12	54	65	CAGATTTGTTATGTGTTTTGACAAT
scaffold722019_12.3	1	p2	(CT)12	24	36	59	TGGTGGCTGAAATGCTTGTA
scaffold722025_16.7	1	p2	(CT)6	12	407	418	TTCTGCCCTTAAGGCTGTTG
scaffold722040_16.0	1	p2	(AT)6	12	4010	4021	TTCTAAAGCTCCACCACACG
scaffold722078_15.1	1	p2	(TC)7	14	504	517	GAGTCATCAAGAAGGCCAGC
scaffold722135_14.1	1	p2	(TA)6	12	65	76	GACCAAAAATCCAATCCAGG
scaffold722149_17.5	1	p2	(GA)6	12	1223	1234	GGAGAAATCAAGAGGAGGGG
scaffold722186_14.2	1	p2	(TC)6	12	190	201	AGCCTGTACGACTCCGACAC
scaffold722203_14.6	1	p2	(GA)6	12	170	181	ATTTGAGGGGGAAAGGAGAG
scaffold722210_13.3	1	p2	(AT)8	16	972	987	GGCATAGGTTTGGGTTTCAA
scaffold722226_18.3	1	p2	(GA)6	12	294	305	GAGGATCTGTGGGGTGGTAA
scaffold722235_13.3	1	p2	(AT)7	14	286	299	GGAATTCCAGCAACCTCTTG
scaffold722249_16.9	1	p2	(TG)6	12	3548	3559	TTCGAGGCGTTTTCTAGGTG

scaffold722281_13.7	1	p2	(AT)9	18	351	368	GTGTCAAATTCGCAACGAAG
scaffold722292_14.9	1	p2	(TA)6	12	1388	1399	TTTGATGGGATGGTATGTTTATG
scaffold722294_12.0	1	p2	(AC)9	18	328	345	AACCAACATCCCATCTCCAT
scaffold722304_14.8	1	p2	(TA)6	12	77	88	AAAAATCAAAGGCCCACAAT
scaffold722334_18.7	1	p2	(CT)6	12	860	871	GCCATCAAAAGAGGAAACGA
scaffold722337_14.6	1	p2	(TG)6	12	156	167	CACGAATGAAACAAGTTGGG
scaffold722361_14.2	1	p2	(CT)6	12	633	644	TGATTTCAGTTGGTAGCAGGAA
scaffold722379_15.9	1	p2	(CA)7	14	1476	1489	ACATGGCGTGCTACAATCAA
scaffold722386_15.7	1	p2	(TA)7	14	388	401	CTTCTTCCCAGTTTCCCCTC
scaffold722407_20.0	1	p2	(AT)6	12	358	369	GGGTTATCATTTGAACGCGA
scaffold722415_15.4	1	p2	(CT)8	16	148	163	CCCAAGCAAAAGACGACACT
scaffold722421_11.6	1	p2	(GA)6	12	800	811	AGCTTCTGACATCCACGCTT
scaffold722433_13.7	1	p2	(AG)7	14	525	538	ATTTCGTGAATTGGCTACGG
scaffold722436_13.8	1	p2	(AG)7	14	507	520	TCTTTTGCAGCCCTCCTTTA
scaffold722446_16.1	1	p2	(TC)6	12	840	851	GAAAAATGTATGGGGTCGGA
scaffold722452_14.4	1	p2	(TA)7	14	1722	1735	AGGGACTTAACAAGCCATCG
scaffold722457_12.0	1	p2	(TA)6	12	53	64	TTTCTCATACAAAAAGACATTTCTCA
scaffold722459_18.0	1	p2	(CT)8	16	313	328	TGCTCACAAGCAACCAAAAC
scaffold722466_16.8	1	p2	(AG)7	14	3130	3143	ACCTTAGCAACGAAGGAGCA
scaffold722515_13.8	1	p2	(AT)6	12	1711	1722	GCAAGTCTGGTACTTGGGGA
scaffold722542_11.7	1	p2	(AT)7	14	746	759	TTGACTGTCATTTTACCTCTTTATGA
scaffold722581_14.7	1	p2	(CA)9	18	4301	4318	CATAGCTTGCTCACCAACCC
scaffold722606_12.8	1	p2	(TA)8	16	567	582	CGGTTCTGACCACTAAACGA
scaffold722629_15.0	1	p2	(AT)10	20	86	105	TTTGGATCTGTTTGTCGAGTTG
scaffold722630_17.1	1	p2	(TA)6	12	2291	2302	GATAATCACCGGAAAAGCGA
scaffold722640_19.6	1	p2	(TA)6	12	1614	1625	CCCACACCTCATCAACCTCT
scaffold722663_15.7	1	p2	(TA)10	20	44	63	AAGGAGGTGTTATGAAGGGATT
scaffold722664_15.4	1	p2	(CT)9	18	671	688	ACATGGGCAATAAGTCAGGC
scaffold722678_10.5	1	p2	(TA)9	18	233	250	GAAAATCTGCAGTGGGGTGT
scaffold722691_17.1	1	p2	(CT)8	16	714	729	ATGGGAGCTCATGAACGAAC
scaffold722702_14.7	1	p2	(CT)10	20	993	1012	ATACGGGGCATAAGCTGTCA
scaffold722716_16.4	1	p2	(TA)6	12	413	424	CCCAGCATCCGTTAGTTACAA
scaffold722717_14.0	1	p2	(TA)7	14	411	424	CAAATCGATTCGGAGTTTGAA
scaffold722730_16.2	1	p2	(GC)6	12	168	179	TATAGGCACTTCCACCAGGG
scaffold722757_16.7	1	p2	(TC)7	14	2450	2463	TCACCTTCTTTCCCCTTCCT
scaffold722758_13.4	1	p2	(TA)8	16	829	844	GCGAAATAGGATCGCCAATA
scaffold722765_14.5	1	p2	(GA)11	22	3864	3885	TAGCGCTGCAGAATTCAAGA
scaffold722777_16.0	1	p2	(TC)6	12	746	757	GTAAATCACGAGACCGCCAG
scaffold722792_15.7	1	p2	(AT)6	12	415	426	AGAAATCCAGCAACCAATCG
scaffold722872_10.5	1	p2	(AG)6	12	1119	1130	TGAGGAATGAGAGCGTAGGG
scaffold722877_14.3	1	p2	(TA)6	12	320	331	CGTGCAAAATAGCACCAAGA
scaffold722890_14.9	1	p2	(TA)9	18	215	232	TCCAGCGCCCTATAATGTTC
scaffold722916_11.8	1	p2	(AT)7	14	673	686	CGTTGTCTGCCTTGGAATTA
scaffold722938_14.7	1	p2	(AT)8	16	332	347	TAATGGCCCTGAATCTTTCG
scaffold722980_14.0	1	p2	(GA)6	12	1165	1176	ACAACAAGATCCCCTGCAAT
scaffold722985_14.2	1	p2	(CT)7	14	782	795	TGGGTGCTTTTCTTGCTCTT
scaffold722998_15.1	1	p2	(AG)7	14	985	998	GCCCATTGATGAAAGTTGGT
scaffold723014_13.9	1	p2	(TC)7	14	930	943	AAGAGGGAGAGAATGGGAGC
scaffold723021_11.0	1	p2	(AT)12	24	692	715	CAGAGCGCACTCGAAGAGTA
scaffold723059_15.0	1	p2	(AT)6	12	649	660	TTCCCCCTTTTATGGTCAAA
scaffold723080_15.3	1	p2	(TA)7	14	2396	2409	TATGCAGCCATCAGACCAAG
scaffold723082_17.7	1	p2	(AC)6	12	2012	2023	CAAAAATGCCACTGCTTGAA
scaffold723105_11.0	1	p2	(TA)9	18	275	292	TTTGAGGAAGGATTTGAGGC
scaffold723108_16.6	1	p2	(TA)8	16	48	63	GCGGTTATCACCGGATCG
scaffold723119_16.3	1	p2	(TC)7	14	3796	3809	TTGCTGCTGACACCAATCTC
scaffold723130_16.2	1	p2	(TA)7	14	1114	1127	TCAACAACGGTAAGTTGCTCTT
scaffold723149_15.9	1	p2	(AC)7	14	1456	1469	CATGTGCGACTTTGGAGGTA
scaffold723159_17.4	1	p2	(AG)7	14	3584	3597	TCAAGAATCCCTTTAGCCGA

scaffold723162_13.5	1	p2	(TC)9	18	97	114	CCCCATTACCATCTTTCTCG
scaffold723187_14.9	1	p2	(AG)6	12	275	286	AGGGAGTTGCTAGGGTGTGA
scaffold723218_17.2	1	p2	(AT)6	12	977	988	CATGCAGGGCTGAGTACAGA
scaffold723219_16.2	1	p2	(CT)6	12	947	958	CTTCCCCTTCGCTCCTACTC
scaffold723225_10.2	1	p2	(AT)9	18	173	190	TCAAAAACCGAAAACCGAAC
scaffold723245_14.9	1	p2	(GA)6	12	841	852	TATTGTAGCGGCCCAATACC
scaffold723249_11.8	1	p2	(TA)7	14	1108	1121	GCATCGTAAGGAGCGTTGAT
scaffold723263_15.6	1	p2	(AG)7	14	417	430	AAATCAGCACCCAACCAAAG
scaffold723290_19.4	1	p2	(TC)6	12	419	430	GAGTGCTTCGAAAAGGTTGC
scaffold723294_18.7	1	p2	(TC)7	14	1344	1357	GTTAGGGTTTTTGTCGGGGT
scaffold723295_14.7	1	p2	(TC)6	12	419	430	GATATTCACCGACGTGCCTT
scaffold723300_13.7	1	p2	(TC)6	12	62	73	TAAGAGTTGTCCGGAGTCGG
scaffold723305_13.5	1	p2	(CT)8	16	415	430	CTTGTTGAGAGGTGGCTGTG
scaffold723308_14.9	1	p2	(TA)9	18	322	339	TCAATCATCCAACACACCAA
scaffold723360_11.1	1	p2	(TC)7	14	829	842	ACGCGAGGGTTGTGATAAAC
scaffold723469_14.4	1	p2	(CT)7	14	2275	2288	TGGTATCAGGGTTTCAGCTTC
scaffold723480_14.9	1	p2	(AT)9	18	575	592	TCCACCACTGTCAGAAAGCA
scaffold723507_12.5	1	p2	(AC)10	20	248	267	GAACCAGCTGACCATGTGAA
scaffold723517_13.2	1	p2	(CT)7	14	62	75	AATTTCTTGCTCCAAACCTTTA
scaffold723546_16.0	1	p2	(TA)9	18	1117	1134	AATCAAAGAGCAATGAGCCG
scaffold723552_16.6	1	p2	(GA)6	12	174	185	GCAACAAATGCCAACGCTAT
scaffold723561_13.8	1	p2	(AT)6	12	514	525	TTGATGTGAGATGGGCTTGA
scaffold723566_16.1	1	p2	(GA)11	22	35	56	CCTACCTAAGGAGGGAGGGA
scaffold723603_16.2	1	p2	(AC)10	20	630	649	CCCTACGCACTCGTTTTTCT
scaffold723608_14.8	1	p2	(AT)6	12	423	434	CCTGCTGTTGATTTGATGGA
scaffold723613_15.5	1	p2	(AG)7	14	422	435	AAATGGAATGTGGCGAAGAC
scaffold723723_14.9	1	p2	(TG)9	18	203	220	GAAAAATTCGTAAGTGGCCG
scaffold723752_15.0	1	p2	(CA)6	12	165	176	GTAACTCAAGGCCCATTCCA
scaffold723759_12.3	1	p2	(CT)6	12	727	738	CCCAACAATTTCGGTTTGAC
scaffold723771_18.3	1	p2	(TA)6	12	426	437	ACCAAACCCATTCTTGTCCA
scaffold723772_14.3	1	p2	(AT)11	22	708	729	TTGATGGATTTTTCAAGCCC
scaffold723781_17.3	1	p2	(CT)8	16	897	912	TTTCTTCAAATTTGGGGTGG
scaffold723790_18.9	1	p2	(TA)6	12	425	436	TGCAAGCAAAAAGACGAAAA
scaffold723810_17.2	1	p2	(AG)6	12	1340	1351	GAGGGAGCTGCAACAAGAAA
scaffold723824_11.6	1	p2	(TA)9	18	602	619	TTATGGCCCTTGGATTTGAG
scaffold723831_17.3	1	p2	(AC)8	16	25	40	TGTGACCTCAATTGCAAAAC
scaffold723864_10.1	1	p2	(TA)8	16	595	610	ACTGGACCGGACCGTTTT
scaffold723882_10.2	1	p2	(AT)9	18	1099	1116	TTGGCAAAGTCCCCTATTTG
scaffold723935_19.8	1	p2	(CA)6	12	370	381	AGCTGCAGCATTTCTCACAA
scaffold723948_15.8	1	p2	(AG)6	12	278	289	GGTCAGTTTCTTGCTTGCAG
scaffold724018_11.4	1	p2	(AT)10	20	1415	1434	TCGTGCATTACTCGGTTACTTG
scaffold724043_14.1	1	p2	(CT)7	14	3112	3125	GGAACCAATCTGGTGCATTC
scaffold724057_10.1	1	p2	(TA)7	14	269	282	TCAGATCACCACCGGACATA
scaffold724073_15.4	1	p2	(CT)8	16	1601	1616	CGCACACACCATCTGTGAAT
scaffold724092_15.7	1	p2	(TA)6	12	219	230	TTCCTCTTGAATTTGGTGGG
scaffold724093_15.8	1	p2	(CT)6	12	428	439	GGGAACCAGATACGAGGACA
scaffold724126_16.3	1	p2	(TC)6	12	2129	2140	TGGAAATGGAATCCGAAGAC
scaffold724130_15.3	1	p2	(AT)6	12	660	671	CTTCAAGGATTTGAGCCGAG
scaffold724163_12.1	1	p2	(AG)7	14	57	70	GCTTCTTCTTGCTGTGAGTGG
scaffold724170_12.9	1	p2	(GA)13	26	495	520	TGAAATCATGCTGGTTGCAT
scaffold724171_13.8	1	p2	(AT)8	16	1510	1525	GCCTGTTTGCCACTCAAAAT
scaffold724173_12.2	1	p2	(AT)8	16	426	441	ATTGTCACATGGTCCGCATA
scaffold724175_17.0	1	p2	(AT)10	20	1849	1868	TGTGCCTTGATTCATTGAAAA
scaffold724197_15.1	1	p2	(CA)6	12	737	748	TCAAGATTTTGTGTGGCTGC
scaffold724202_17.1	1	p2	(AG)7	14	428	441	CACCCCTGAAACTCCATCAT
scaffold724206_15.0	1	p2	(AT)8	16	743	758	GACGACGATGAAAATCGTGA
scaffold724216_17.8	1	p2	(TA)9	18	267	284	AACGCGCATTTTAGGGTTTA
scaffold724238_17.5	1	p2	(TC)6	12	51	62	TGTCGGTGGACTGATTTGTG

scaffold724242_13.[illegible]	1	p2	(TA)7	14	126	139	CGTGAGAAGCGTTGATATGG
scaffold724282_13.[illegible]	1	p2	(AT)6	12	552	563	CCTCTCACATGTCTCCACGA
scaffold724302_17.[illegible]	1	p2	(TA)6	12	383	394	GGATCTGCGTCCATTTCACT
scaffold724320_12.[illegible]	1	p2	(AT)7	14	1697	1710	CGAGACATGGAATTAGGGCT
scaffold724339_15.[illegible]	1	p2	(GA)9	18	834	851	CACCAAATGTATGTGCATGAGA
scaffold724386_13.[illegible]	1	p2	(AT)6	12	197	208	GGGGATGGGGGTAAGTAATG
scaffold724392_15.[illegible]	1	p2	(TA)6	12	431	442	TGACCGAAAAATCCAACGAT
scaffold724424_13.[illegible]	1	p2	(AG)7	14	156	169	ATCAGCTGCCAAACCAAGAT
scaffold724457_13.[illegible]	1	p2	(AG)9	18	638	655	AAAGGTCCCTCACCAGTCAA
scaffold724460_15.[illegible]	1	p2	(AG)7	14	940	953	GCTGCTCCTGTCTCTTTGCT
scaffold724500_16.[illegible]	1	p2	(GA)6	12	723	734	GGTGCTGCAACTGAACTGAA
scaffold724512_14.[illegible]	1	p2	(CT)6	12	426	437	CTGTCACGTCTCTTCCACCA
scaffold724530_14.[illegible]	1	p2	(AG)10	20	553	572	TGGCTATAGGACATCGAGGG
scaffold724562_11.[illegible]	1	p2	(GA)7	14	432	445	TTTGCATGGAATAAGAATTTTGAA
scaffold724567_13.[illegible]	1	p2	(CT)7	14	430	443	TTAACCGCCAAACTCAAACC
scaffold724572_15.[illegible]	1	p2	(AG)8	16	1553	1568	GGGCTATTGTCATTTCGACC
scaffold724573_17.[illegible]	1	p2	(CA)6	12	4009	4020	CCTCTGGCTGTTCTGGATTT
scaffold724593_11.[illegible]	1	p2	(GA)8	16	736	751	CTCCACTGATGTGTGGATGC
scaffold724601_14.[illegible]	1	p2	(AG)8	16	242	257	CATGAATTTTTGGGAAGCTGA
scaffold724637_15.[illegible]	1	p2	(TC)8	16	3806	3821	TTCATGCAAACTGCTGGAAC
scaffold724645_14.[illegible]	1	p2	(AT)6	12	1104	1115	TCTGTTCAGAGAATGCTGGG
scaffold724779_16.[illegible]	1	p2	(AG)7	14	1348	1361	CATGACCCAATCAATGCAAC
scaffold724796_14.[illegible]	1	p2	(TG)6	12	518	529	TGAAAAAGAAAAACGCGTCA
scaffold724813_14.[illegible]	1	p2	(AG)6	12	51	62	TGTACGTGCGTACGTGTGTG
scaffold724814_14.[illegible]	1	p2	(GA)6	12	991	1002	TCCTGTTTGATGAAATGCCA
scaffold724824_14.[illegible]	1	p2	(GT)8	16	51	66	GTCTGAGCGAGAGTGTGTGC
scaffold724856_15.[illegible]	1	p2	(AT)7	14	378	391	CAAATGAGATTGGTTCGAGCTT
scaffold724867_15.[illegible]	1	p2	(AT)9	18	1173	1190	CATACATGTTGAATTGCGGC
scaffold724885_15.[illegible]	1	p2	(AT)6	12	758	769	AAGCTCGGGCCTGAACTATT
scaffold724891_18.[illegible]	1	p2	(TA)6	12	407	418	ACTCAGAAATCCATCCCACG
scaffold724903_17.[illegible]	1	p2	(CA)6	12	1705	1716	AGAAACCCATGTGCAACTCC
scaffold724972_16.[illegible]	1	p2	(CT)7	14	2949	2962	CGCCTTTGCATCTCCTGTAT
scaffold725058_13.[illegible]	1	p2	(AT)11	22	53	74	CATTAAAATGTGTATGCACCTGA
scaffold725061_18.[illegible]	1	p2	(TG)7	14	1887	1900	TGTGCATTTCAACTTGGGAA
scaffold725088_13.[illegible]	1	p2	(TC)6	12	1059	1070	CAAAGGGGGAAAAGAAAAGG
scaffold725140_14.[illegible]	1	p2	(AT)6	12	537	548	CGGTGGAGATGTGGAGTACA
scaffold725167_15.[illegible]	1	p2	(TA)12	24	81	104	CATTGTGGGCCTTTGATTTT
scaffold725236_13.[illegible]	1	p2	(CT)7	14	1713	1726	TAAAAATGGGAGATGAGGCG
scaffold725238_15.[illegible]	1	p2	(AT)7	14	617	630	GCGGCCAGTTCAATACAAAT
scaffold725254_12.[illegible]	1	p2	(AG)7	14	606	619	AGAGAGAAAAAGCTTCGGGG
scaffold725256_13.[illegible]	1	p2	(GA)6	12	750	761	AAAAACATCAACTGGGACCG
scaffold725257_14.[illegible]	1	p2	(TC)6	12	2289	2300	ACCGATCCATCTCTCGTGAC
scaffold725262_13.[illegible]	1	p2	(TA)8	16	49	64	AGACCGGACCGTTAGGTAAT
scaffold725264_17.[illegible]	1	p2	(AG)8	16	881	896	ACCTCCCTCCTCCTCAGTGT
scaffold725271_16.[illegible]	1	p2	(CT)7	14	439	452	CCAAGATGAATAAATGAAAGGGA
scaffold725283_11.[illegible]	1	p2	(CT)6	12	359	370	GCAAGAGAAGCCCTCACAAC
scaffold725285_15.[illegible]	1	p2	(GA)6	12	255	266	ACGCAGCTCCCTTTTTACTG
scaffold725298_12.[illegible]	1	p2	(CT)6	12	1385	1396	CGGATTTAACCCCCAAATCT
scaffold725309_15.[illegible]	1	p2	(TA)6	12	4479	4490	TCGAAAGTCCAATTTCCACC
scaffold725325_18.[illegible]	1	p2	(GA)7	14	440	453	GAACTGAGAACTGCTGCGTG
scaffold725353_17.[illegible]	1	p2	(CT)8	16	439	454	TGCTCCCCTCATCTTTTGAC
scaffold725361_14.[illegible]	1	p2	(AT)6	12	132	143	CTTTGATTCCCCACCTTCAA
scaffold725362_17.[illegible]	1	p2	(TA)6	12	835	846	TGTTCGTGTTCTCGAACGAT
scaffold725430_16.[illegible]	1	p2	(CT)6	12	670	681	ACCTGGTGCTGTCCAATAGC
scaffold725451_15.[illegible]	1	p2	(AT)6	12	720	731	AAACTTCCGATGGAATGCAG
scaffold725452_14.[illegible]	1	p2	(AT)8	16	115	130	CAACGCGTCTTTGAGAAGTG
scaffold725458_15.[illegible]	1	p2	(GA)6	12	2508	2519	ATTCATTGCAGGTTTGGAGC
scaffold725465_13.[illegible]	1	p2	(CT)8	16	544	559	GGGATTGGACCCTCATTTTT

scaffold725467_14.8	1	p2	(TC)6	12	841	852	AAACTCGGTTTGGGCTTTTT
scaffold725470_15.7	1	p2	(TC)6	12	874	885	CGCTTGTCCTGAGTTGTGAA
scaffold725474_15.4	1	p2	(TA)10	20	2012	2031	AGTTGCCGATTGGTAAGCAC
scaffold725500_13.2	1	p2	(TA)10	20	706	725	TATGAGAAGGGCATTTTCGG
scaffold725505_15.4	1	p2	(TC)6	12	1756	1767	AACACATGTCATGATAGCAGTTAGA
scaffold725522_16.6	1	p2	(CA)8	16	169	184	ATGTCAGTCTGCCAAAGGCT
scaffold725540_15.1	1	p2	(CT)9	18	634	651	TTTCACGAACCCACATTTTT
scaffold725551_11.1	1	p2	(TA)7	14	444	457	TCGTAAAACATGAACTTCTTTGGA
scaffold725602_12.3	1	p2	(GT)10	20	1213	1232	TACGCAAAATACGCCAATCA
scaffold725613_12.0	1	p2	(AT)6	12	411	422	GAGGCTATCGAGGCTTTTCC
scaffold725617_12.9	1	p2	(AG)11	22	109	130	TCCACCTTGAAGCTCTCGAT
scaffold725634_15.2	1	p2	(GT)7	14	717	730	CTCTGCTGATCCCCTTTTTG
scaffold725638_16.5	1	p2	(GA)6	12	731	742	TCCACAGACAGATTTCCCAA
scaffold725684_13.1	1	p2	(AT)7	14	1177	1190	TATACACGTTTCCCTCGCCT
scaffold725707_16.1	1	p2	(CT)6	12	7547	7558	CCTCCTATACCCCTTCTCGC
scaffold725715_16.6	1	p2	(CT)6	12	801	812	AAGAAACCCCCATTGAAACC
scaffold725716_16.8	1	p2	(AG)6	12	449	460	CCACAATGTAAGGAGCACAAA
scaffold725727_12.6	1	p2	(GT)6	12	534	545	TGCAAACAGAAACAAGGCAC
scaffold725734_17.7	1	p2	(TC)7	14	920	933	CCTGGTCATAAGTACAAGAACCG
scaffold725735_13.3	1	p2	(TA)6	12	1332	1343	CATTTTCGACCGTCATGAGA
scaffold725756_16.0	1	p2	(AC)7	14	1031	1044	AATTGCCTGCACCTCCAAT
scaffold725776_15.1	1	p2	(TC)7	14	447	460	GAGAAATGGAGCAGCCTACG
scaffold725783_15.0	1	p2	(AT)7	14	928	941	GACGTACGACTCATGCGACA
scaffold725784_16.1	1	p2	(CA)6	12	1158	1169	CCAAATTTCCAATACAAGGCA
scaffold725787_16.3	1	p2	(TC)6	12	643	654	TCGACATATTTTCTTCCGCC
scaffold725811_11.8	1	p2	(TA)7	14	156	169	GCGGGGTTGTTAAAGAAGAG
scaffold725845_14.7	1	p2	(CT)6	12	449	460	AAATTCAACCTTGGATGCTACA
scaffold725865_15.7	1	p2	(CT)7	14	437	450	CCGGTTTGCACCAATTCTAT
scaffold725895_14.3	1	p2	(TA)6	12	360	371	TGGGTCAATTCCAATTCAAAA
scaffold725896_16.6	1	p2	(AT)8	16	512	527	CAAATCTTGTTGCCATGCTG
scaffold725945_16.7	1	p2	(TA)9	18	1105	1122	ATTGCATGCCACAATTTCAA
scaffold725946_14.0	1	p2	(AG)9	18	1095	1112	TGATATTCATTGCCTTTGCG
scaffold725982_14.5	1	p2	(AT)9	18	232	249	TCAATTGGCCATATTAGCACA
scaffold725984_18.4	1	p2	(AG)6	12	835	846	GGCAAGAAATAACAGCATACCA
scaffold725997_14.5	1	p2	(TA)6	12	810	821	CCCCCACAGACATATTGACTC
scaffold726014_14.5	1	p2	(TC)8	16	409	424	TGCAAACATGGGACTAATATGAA
scaffold726020_19.8	1	p2	(AG)7	14	1728	1741	ACCGCTTCAAACAAAAGCAG
scaffold726027_17.8	1	p2	(AG)6	12	764	775	TGCAAAATGGAATGGACTGA
scaffold726060_15.5	1	p2	(CT)7	14	473	486	ATCTTGTTCCGTGCATTTCC
scaffold726083_14.8	1	p2	(CT)9	18	1516	1533	AAACCCAAACCTTAATGCCC
scaffold726094_14.4	1	p2	(CT)7	14	356	369	CGATTTACGAGCATCCCATT
scaffold726101_10.0	1	p2	(TC)6	12	148	159	CTCCACCCTTTAATGTCACCA
scaffold726107_17.5	1	p2	(CT)7	14	836	849	AATTGCCCATGTTGGTTAGC
scaffold726109_14.7	1	p2	(GA)7	14	909	922	AGCCAAGAAATGGAGGGAAT
scaffold726132_14.7	1	p2	(CT)6	12	103	114	GGCCTCCAACGTTTTCAATA
scaffold726150_13.4	1	p2	(AT)9	18	360	377	CGTCGTTGGTGCTATACCCT
scaffold726157_14.9	1	p2	(TA)9	18	3852	3869	GGAACGCAGAGGATCAATTC
scaffold726163_14.3	1	p2	(AT)6	12	206	217	ATCTGGGCTACCGATTTGTG
scaffold726176_15.5	1	p2	(TC)6	12	231	242	AAGTACATTCCCCCGAAAGG
scaffold726185_13.3	1	p2	(TA)6	12	454	465	TACCCCATGCCTCAAGAATC
scaffold726193_14.6	1	p2	(CT)8	16	253	268	CAACGAGAAGTCGAGAAGGG
scaffold726233_15.7	1	p2	(CT)8	16	1373	1388	CTCCATCCCTGCTCTCTCAC
scaffold726248_14.9	1	p2	(AG)8	16	1770	1785	TTGATGGGTGGAATGTAAAGG
scaffold726278_18.7	1	p2	(CT)6	12	87	98	ATTAACGCACACACGTGACA
scaffold726298_13.8	1	p2	(AT)6	12	403	414	GTGTGGCCTACCAAACACG
scaffold726309_14.7	1	p2	(TA)7	14	1059	1072	TTAAAATGAATGAAGGGCGG
scaffold726352_15.6	1	p2	(TA)7	14	1427	1440	GGTTCTCATTTGAACGCACC
scaffold726353_16.0	1	p2	(TC)7	14	710	723	TGGGCTGCTAAGAAAGGAAA

scaffold726415_14.[illegible]	1	p2	(AG)7	14	311	324	CAAATTTGCTGATGCCATTG
scaffold726436_16.[illegible]	1	p2	(CA)6	12	299	310	CCGAATTCCGACCTAACAAA
scaffold726437_17.[illegible]	1	p2	(GA)6	12	1322	1333	AAGGAGAGTGCAGCGACAGT
scaffold726458_13.[illegible]	1	p2	(TA)6	12	2329	2340	TCAATCGGCAAATTCAGGAT
scaffold726461_16.[illegible]	1	p2	(CT)6	12	1629	1640	TCGGAAAAAGATTCCCATTG
scaffold726471_17.[illegible]	1	p2	(AT)9	18	1217	1234	GGGGCTGAAAAATCAAAGGT
scaffold726472_18.[illegible]	1	p2	(AT)7	14	455	468	CATGGCAGTATTGGCATCAC
scaffold726482_16.[illegible]	1	p2	(TC)7	14	2785	2798	TTGATGGTCCAACACAATGG
scaffold726498_13.[illegible]	1	p2	(CA)6	12	130	141	TTGGTGCTGACTTCCAACAA
scaffold726526_12.[illegible]	1	p2	(TA)9	18	325	342	TAATAGCGGCCGATCTGAAG
scaffold726529_13.[illegible]	1	p2	(TA)9	18	935	952	ACGTCCTACTCGCCCACTT
scaffold726548_15.[illegible]	1	p2	(GT)8	16	3789	3804	GCAGGTTTCCATATGTCGCT
scaffold726559_15.[illegible]	1	p2	(AC)7	14	2481	2494	GTGAAAAGTGGCCAACATGA
scaffold726560_11.[illegible]	1	p2	(AG)10	20	483	502	TCCACCCACTGTCCCTAGTC
scaffold726617_13.[illegible]	1	p2	(AC)6	12	743	754	ATACATATGGACGCCCCAGA
scaffold726639_14.[illegible]	1	p2	(GA)9	18	107	124	TTGCCTTGCTTTCTCTCTCC
scaffold726664_17.[illegible]	1	p2	(TG)6	12	339	350	CGTTCGGGATCATAAAAACG
scaffold726675_10.[illegible]	1	p2	(TA)6	12	120	131	TCATGTCCATTGATCTGAAACA
scaffold726677_16.[illegible]	1	p2	(AG)6	12	122	133	CCAAACCAGGTGAAAAGGAA
scaffold726700_21.[illegible]	1	p2	(AG)7	14	878	891	TTCTGAAAGCATGAACGGTG
scaffold726707_15.[illegible]	1	p2	(GT)10	20	453	472	GTTGTGATGGGGGATTTGAT
scaffold726721_17.[illegible]	1	p2	(GA)6	12	797	808	GCATGACCAATGCAGTACCA
scaffold726731_13.[illegible]	1	p2	(TA)7	14	1024	1037	TTGTTGGAATACGTGGGAAA
scaffold726739_12.[illegible]	1	p2	(AT)7	14	1143	1156	CGAAATCTTGTCCCTTCACG
scaffold726746_16.[illegible]	1	p2	(TA)6	12	51	62	ATGGGTTATCACCGGATCG
scaffold726749_14.[illegible]	1	p2	(AG)11	22	2049	2070	CGAAACCAATTTTTCATCCG
scaffold726753_15.[illegible]	1	p2	(AT)6	12	879	890	TCTGCCCATAAATTGTGTATGA
scaffold726765_16.[illegible]	1	p2	(CA)9	18	64	81	TTTTCTTTTGGGCAGCAACT
scaffold726774_17.[illegible]	1	p2	(CT)8	16	458	473	TTTGCCAAACTGAAGAGCCT
scaffold726807_15.[illegible]	1	p2	(CG)6	12	1698	1709	ACAAACGGAGTGTTCAAGGG
scaffold726814_15.[illegible]	1	p2	(TA)7	14	111	124	ATGAGTTTGCACCTGTGGAG
scaffold726817_10.[illegible]	1	p2	(AT)7	14	130	143	TGGAATCCAAAGCTATTAGACCA
scaffold726837_10.[illegible]	1	p2	(TC)9	18	456	473	GGGCTGAAGCTCCTTTCTTT
scaffold726839_14.[illegible]	1	p2	(CT)6	12	233	244	AAAACGGGAATCGGAATCTT
scaffold726842_13.[illegible]	1	p2	(AT)7	14	1073	1086	GAACCAAATCCACAAATCGC
scaffold726860_13.[illegible]	1	p2	(TA)10	20	883	902	CATTCGGGCCTCTCTGAATA
scaffold726873_18.[illegible]	1	p2	(AG)6	12	329	340	GAGAATGGAACTTGCCTCCA
scaffold726887_13.[illegible]	1	p2	(AG)11	22	567	588	GAGGATTGCACACTCCCACT
scaffold726894_13.[illegible]	1	p2	(AC)6	12	464	475	AAACTAGAAACCCGTGGCAA
scaffold726981_13.[illegible]	1	p2	(CT)7	14	1058	1071	TGGTCCCAATACGTGTCTCA
scaffold727026_13.[illegible]	1	p2	(TA)9	18	43	60	GAAATAAGGAGGTGTTATGAAGGG
scaffold727049_15.[illegible]	1	p2	(TC)6	12	838	849	AATTTGATCAACACGTCCCC
scaffold727064_15.[illegible]	1	p2	(TG)6	12	363	374	AAATTGACACACGCATGCAC
scaffold727066_15.[illegible]	1	p2	(GT)10	20	458	477	GCACATGTTCAAACACGCA
scaffold727087_14.[illegible]	1	p2	(TA)8	16	747	762	AAAATGCCCTTGTATGCTGC
scaffold727100_16.[illegible]	1	p2	(TC)10	20	414	433	AGGCATGAGTTGTCACTCCC
scaffold727103_15.[illegible]	1	p2	(AG)6	12	4452	4463	CTTATGCAGGGATCGTGACC
scaffold727108_12.[illegible]	1	p2	(TC)6	12	1045	1056	AGCCAGTGGTCCAGAGAGAA
scaffold727117_13.[illegible]	1	p2	(TA)9	18	240	257	GCCACAAATGTCACAAATCAA
scaffold727122_16.[illegible]	1	p2	(AG)8	16	1513	1528	GAATTGAGTTCATCCCGGTG
scaffold727152_16.[illegible]	1	p2	(CT)6	12	669	680	TGCAAGAAAATGATCCACCA
scaffold727157_13.[illegible]	1	p2	(AT)9	18	169	186	TGTGCAATTTGTAAGAAACACG
scaffold727158_14.[illegible]	1	p2	(CT)6	12	466	477	TTATGAAGGAGGCGTGGAAC
scaffold727179_15.[illegible]	1	p2	(TC)7	14	684	697	TAGCACGTCTGCACTTGGAC
scaffold727200_16.[illegible]	1	p2	(CT)9	18	250	267	TTTTCCTGCATTTTGCATTTC
scaffold727201_13.[illegible]	1	p2	(TA)8	16	922	937	ACAACTCGCGAGCGTAAATC
scaffold727212_15.[illegible]	1	p2	(CT)12	24	6482	6505	TGTTAACGATGGGAGCATTG
scaffold727223_15.[illegible]	1	p2	(TA)9	18	47	64	CGGATTTAAGGGTTATTACCTGATT

scaffold727225_14.3	1	p2	(AG)9	18	86	103	GGTTGAGAACAAGGGGTTGA
scaffold727236_16.2	1	p2	(TC)7	14	1527	1540	AATCATACCGCTTCTCGCTC
scaffold727241_14.7	1	p2	(AG)6	12	2569	2580	GATTTGGGTTGTTTGATGGG
scaffold727262_16.5	1	p2	(AG)6	12	195	206	GCACGCCCTTTTCTCTACAC
scaffold727263_17.0	1	p2	(AG)6	12	710	721	CGGGAAAAAGTCTCACAGTTG
scaffold727326_15.7	1	p2	(AT)8	16	466	481	GCAACTGGCGTGATTATTGA
scaffold727331_16.0	1	p2	(AT)6	12	63	74	TATGCCCGGATTTTTGTTTT
scaffold727352_13.2	1	p2	(TA)8	16	1062	1077	GTGCCCACCAACTTGTTCA
scaffold727385_16.6	1	p2	(GA)6	12	154	165	AGTTTAAAGGGGCCTTGCAT
scaffold727386_17.8	1	p2	(AT)7	14	468	481	TTGATCATAATTTTCGCGCTT
scaffold727390_14.9	1	p2	(AC)7	14	1084	1097	AACAATAGGACCTTCAGGCAA
scaffold727409_13.6	1	p2	(TA)11	22	545	566	TCCGTCATATAAATGCCACAA
scaffold727423_13.7	1	p2	(AG)11	22	58	79	TCGTTGGTCAAGGATAGCAA
scaffold727426_16.9	1	p2	(CA)13	26	43	68	AACACTCACGCCTTCCAAAC
scaffold727474_12.6	1	p2	(AT)13	26	436	461	TTCACCAAATCAATGCTCCA
scaffold727477_17.2	1	p2	(TC)6	12	243	254	CTTTTGAGGGCGCAGTCTAC
scaffold727480_13.5	1	p2	(TA)6	12	1163	1174	CCATTTCCTAGGTCGCGATA
scaffold727497_14.3	1	p2	(TA)9	18	673	690	AAGGCCCTTGGATTTGAGAT
scaffold727528_15.3	1	p2	(AG)10	20	464	483	TTGGAGCTGCATTTCTTGTG
scaffold727581_14.3	1	p2	(TG)6	12	1556	1567	TCACAAAGAGCATGTTCACG
scaffold727593_15.6	1	p2	(AT)9	18	467	484	AAAAGCTATGGGGAAGAACACA
scaffold727599_18.2	1	p2	(TA)8	16	916	931	GAACCAGACTGCACGTTTCA
scaffold727612_16.6	1	p2	(AG)6	12	510	521	GTGGACTGGTTTCAGGTGGT
scaffold727615_17.8	1	p2	(TA)6	12	154	165	TCTCTACAAAACGAGACCAATCTTT
scaffold727618_17.2	1	p2	(AG)6	12	1754	1765	TCTCTCCCTCTCCAACTCCA
scaffold727646_12.0	1	p2	(AG)9	18	987	1004	GCTGTCGTACACGGTGCTAA
scaffold727659_11.5	1	p2	(TA)6	12	422	433	TCAAATGACTTGGGCATGAA
scaffold727670_13.1	1	p2	(AT)7	14	965	978	TCATCCAAAAGCAAAGGAAAA
scaffold727671_17.7	1	p2	(AT)8	16	1015	1030	TGCGTTTGTTAGCTTTGGTG
scaffold727672_16.3	1	p2	(TC)9	18	302	319	CGAAGATTTATGCGGGGTTA
scaffold727707_15.0	1	p2	(AT)7	14	1031	1044	TGGATAAACAAAATTGATGGTTTG
scaffold727714_15.9	1	p2	(GA)7	14	5143	5156	GCGGAACAGTAGTTGGGATG
scaffold727727_18.0	1	p2	(AT)8	16	2120	2135	GAGAAGCAGCGGTAGGTGAT
scaffold727746_16.5	1	p2	(AG)6	12	1063	1074	CGTCCAAAATACCTTTTATTCCA
scaffold727757_17.7	1	p2	(AG)10	20	1216	1235	ACCCAGAAACAGCACAATCC
scaffold727769_16.1	1	p2	(AC)6	12	1714	1725	GATACCGTGGGCAACCTAAA
scaffold727771_16.4	1	p2	(TA)6	12	1676	1687	GCCATGGATGCGATAAAAGT
scaffold727773_14.9	1	p2	(TA)7	14	187	200	ATCTAGGGGCCGAGATTTGT
scaffold727806_16.3	1	p2	(AC)7	14	9455	9468	CGATTGTTGCGTGCATAAAC
scaffold727809_11.4	1	p2	(AT)6	12	518	529	CCCAAGCCATGGAGTAGAAC
scaffold727817_14.8	1	p2	(AG)6	12	1120	1131	TGAAGTCTAAAGGGAGACGGG
scaffold727849_14.8	1	p2	(AT)6	12	110	121	CTTAAGCAAATTGCAAGCCA
scaffold727852_14.5	1	p2	(TA)9	18	305	322	AGTTGGCACCGTATCACTCC
scaffold727861_17.2	1	p2	(TC)6	12	1284	1295	TTTGAAAGGTATGATTTGGCA
scaffold727889_15.6	1	p2	(AG)11	22	2768	2789	AAGCAAACATTGTTCCAGGG
scaffold727890_16.1	1	p2	(TA)6	12	1320	1331	GGTACCCCACATGAGAATCAA
scaffold727923_16.2	1	p2	(TC)6	12	479	490	GGCTCAATTATTTAGGCCCAT
scaffold727929_15.0	1	p2	(TA)10	20	1865	1884	AATTGGACCGGACCATTAGTT
scaffold727932_15.7	1	p2	(TA)10	20	45	64	GGGGTTATCATGGGATCAAG
scaffold727940_13.3	1	p2	(CA)7	14	203	216	GTTCTCTTCTTCTGCCACCG
scaffold727944_15.5	1	p2	(TG)7	14	1735	1748	GTGTGGATCTAGGGCACGTT
scaffold727947_14.6	1	p2	(AT)9	18	2409	2426	GAGTTGATCGTCGGTGCATA
scaffold727966_11.8	1	p2	(GA)6	12	1018	1029	CCCTTGGTATCAAAAGGGGT
scaffold727970_13.1	1	p2	(TA)9	18	474	491	TTTTTACCATTTTGCCCCTG
scaffold727977_15.4	1	p2	(GA)7	14	850	863	CAAATGCAAATCTTCTGGCA
scaffold728007_11.5	1	p2	(AG)6	12	552	563	TGGAACTGCATGCTAAGTGG
scaffold728008_15.4	1	p2	(GA)9	18	592	609	GAAGAAATCCCTGCCTTTCC
scaffold728011_15.8	1	p2	(AT)8	16	731	746	ACACAAGGATTGTTTTCCCG

scaffold728031_14.7	1	p2	(TA)10	20	780	799	CCTATTGCTTAAGACGACATGC
scaffold728033_17.0	1	p2	(AG)7	14	479	492	CTCCATTGGTGGAACGAGAT
scaffold728047_15.4	1	p2	(TA)6	12	983	994	GGCCCAAGCAAAATAAGTCA
scaffold728064_16.0	1	p2	(AT)9	18	299	316	TGCAATTTATATGCTTTTGCAC
scaffold728083_17.3	1	p2	(AT)10	20	188	207	CATAACAAGTGGCGAAAGGG
scaffold728097_15.6	1	p2	(CA)9	18	2099	2116	TGCAAGCAATAATAAGCCCC
scaffold728098_15.6	1	p2	(GA)7	14	134	147	CTGGCAATTTGACGGGTAAT
scaffold728120_18.9	1	p2	(TA)9	18	625	642	TGCACAATCGATTTACGTGG
scaffold728132_17.5	1	p2	(AT)6	12	580	591	GTTTCGCAAGGCAAATAACC
scaffold728137_15.0	1	p2	(AG)7	14	481	494	TCATCAAGGCCTCACATTCA
scaffold728155_17.2	1	p2	(AC)7	14	2004	2017	AAACAGCAGAGAAGCCCAAA
scaffold728201_17.3	1	p2	(CT)7	14	1573	1586	GAATTCGGGGTGTTTTGAGA
scaffold728210_14.4	1	p2	(AT)7	14	263	276	TGCGTTGTTTTACCATTGGA
scaffold728212_18.0	1	p2	(TC)6	12	249	260	CTCGTCCAGGACAAATCCAT
scaffold728223_14.1	1	p2	(TC)7	14	2660	2673	CATTGCCGCAAGATCAAGTA
scaffold728226_12.7	1	p2	(TC)6	12	689	700	ATCTGCATGTCTACGTCCCC
scaffold728229_18.0	1	p2	(TA)7	14	143	156	TCCCAACCCCATTTACAAAA
scaffold728235_12.9	1	p2	(AT)10	20	93	112	GTGGGTTTACCTAGAGCGCA
scaffold728248_13.2	1	p2	(GA)7	14	640	653	GGAATGAAGTATGTGGAGGAAGA
scaffold728256_15.0	1	p2	(AT)6	12	927	938	CGCCCTTTTAGTTAAAACCTTG
scaffold728266_15.8	1	p2	(TG)9	18	354	371	ACAATGGGTTCATTTGCCAT
scaffold728281_18.4	1	p2	(AT)6	12	203	214	CTATGTTGACTTCGCCGTGA
scaffold728304_16.0	1	p2	(AT)6	12	3210	3221	TCCTCCAGCGAGTCGTAGTT
scaffold728309_13.7	1	p2	(TC)9	18	721	738	TGTGAGTTGCCACTCTCCAC
scaffold728317_15.9	1	p2	(TA)6	12	741	752	CCATGAATTTCACCAGCAAT
scaffold728320_17.0	1	p2	(GT)8	16	3415	3430	TTCTTGATGGAAAGGCAAGG
scaffold728324_15.5	1	p2	(CT)6	12	1845	1856	GGCCAGAAACTGCCATAAAA
scaffold728325_13.7	1	p2	(TA)8	16	481	496	AAGACTCGGGTCCATGACAG
scaffold728329_16.5	1	p2	(TA)7	14	983	996	GTAACAGTAGCTGCCTGCCC
scaffold728349_16.9	1	p2	(AG)7	14	953	966	CCTTGAGCAATGTGGAGCTT
scaffold728356_16.6	1	p2	(TC)6	12	257	268	GTTAGGCTTCCCATCCCACT
scaffold728360_16.8	1	p2	(TG)6	12	2767	2778	TTCGCTCTGATATCCCCATT
scaffold728369_14.8	1	p2	(AG)8	16	1453	1468	TATTCCCGGTATGTAGCCCA
scaffold728388_16.1	1	p2	(AC)6	12	1799	1810	TCTCTCTGCTTCACACACGC
scaffold728394_13.9	1	p2	(TG)7	14	1327	1340	CCACAAAGCTCTTGGAAATAGG
scaffold728405_16.2	1	p2	(AT)8	16	193	208	TAGTATTGGAGGTTGGGGGC
scaffold728408_16.2	1	p2	(AG)11	22	1397	1418	CTCACGCTGGTAAAAGAGCC
scaffold728433_13.0	1	p2	(AT)6	12	397	408	TCGTTCCTACTTTGTGGTGG
scaffold728434_16.3	1	p2	(AT)6	12	1203	1214	GCAGTATCGGGAGTGAGAGG
scaffold728436_15.5	1	p2	(AG)7	14	1742	1755	GATTTGGGGTTTCGGAGATT
scaffold728458_16.2	1	p2	(TA)7	14	56	69	CCCTACCCATTTACCCGTTT
scaffold728478_13.4	1	p2	(TA)13	26	614	639	ATGGTCGTGATTGTATGCGA
scaffold728487_15.7	1	p2	(TA)7	14	132	145	CCTAGCCACACACGGAATCT
scaffold728494_15.5	1	p2	(AT)6	12	1431	1442	CCAATTTAAGTTCGAATGAGTTTTC
scaffold728495_14.8	1	p2	(CT)6	12	599	610	AACCGGTTTGACCGATTTTC
scaffold728514_18.8	1	p2	(AC)6	12	633	644	CTGCACCGGATAATACAGCA
scaffold728518_16.1	1	p2	(AG)6	12	390	401	CCACAAGATCAGCTTTTCCC
scaffold728533_16.8	1	p2	(TA)10	20	37	56	AAGTTTATATCAATAGTTTTGTGTCGG
scaffold728574_15.9	1	p2	(TA)10	20	972	991	TGCAGGTTGAGCAGGTGTAG
scaffold728575_12.4	1	p2	(AT)8	16	486	501	GGAGTTTGAGAGGGGCAAGT
scaffold728595_15.6	1	p2	(AC)6	12	2528	2539	TTTCATTGCTGCTGCAGTTT
scaffold728628_13.8	1	p2	(TA)6	12	1089	1100	AGTGTTTTCAGGCGTTCACC
scaffold728640_13.6	1	p2	(AT)7	14	746	759	ATTCCTCACGGACCTCACAC
scaffold728687_12.3	1	p2	(GA)6	12	2022	2033	GGTTGAGGCGGATAAAGTCA
scaffold728722_16.4	1	p2	(AC)6	12	3361	3372	GTTCGTACCGAACAACACCC
scaffold728724_14.8	1	p2	(AT)6	12	1043	1054	CAAAGCTCAGCCGACCTAGT
scaffold728729_15.6	1	p2	(CT)9	18	41	58	TGCAAGAAAAACAAGCCCTC
scaffold728738_16.0	1	p2	(GT)7	14	781	794	TTGTCGCTGGACACGATAAG

scaffold728740_15.3	1	p2	(AT)7	14	286	299	TCGACTGAAAATTTTGGCTAATC
scaffold728767_16.0	1	p2	(TG)6	12	2242	2253	CTTATGCCACTCCCCACTGT
scaffold728785_16.7	1	p2	(GT)9	18	118	135	CACGAGCTGTTAATCACCACA
scaffold728809_13.4	1	p2	(TA)8	16	292	307	TGGTTAACTCAATCTCAGCCG
scaffold728836_12.7	1	p2	(TC)11	22	482	503	TTAATCAGATGAGGGCAGGG
scaffold728845_13.6	1	p2	(AT)6	12	369	380	TGGACCACACCTATCATAACCA
scaffold728874_15.1	1	p2	(GA)6	12	1609	1620	TGTGGGCGTCAGATCTCATA
scaffold728875_16.1	1	p2	(AT)12	24	2156	2179	TGTTGGATTGGACATTGGGT
scaffold728888_18.7	1	p2	(TG)7	14	493	506	CACTCCTGTATGTAGTGACACGAA
scaffold728902_15.9	1	p2	(TC)9	18	794	811	TTTGGTTTTTGGTTTGCTCC
scaffold728907_16.4	1	p2	(TA)9	18	46	63	CCAATTTGAATGGTTATCACCTG
scaffold728951_13.9	1	p2	(AT)6	12	1777	1788	CCTGGGCTTTGTTCACAGAT
scaffold728957_15.0	1	p2	(GA)6	12	1197	1208	TGAGAGTTTCTGCGTTGGTG
scaffold728958_16.1	1	p2	(TC)6	12	183	194	GTTGGCTTTTTGTTGCCACT
scaffold729004_15.7	1	p2	(AT)7	14	2507	2520	CATCTTGATGTCCCCATTGA
scaffold729039_20.0	1	p2	(TC)6	12	239	250	GCTTCAGTTCTGTGGCATCA
scaffold729050_16.7	1	p2	(TA)6	12	693	704	TCATCAGCAAACTTCCCACA
scaffold729056_14.5	1	p2	(TC)7	14	564	577	TGGATTTAGCCGATTCCAAG
scaffold729057_15.2	1	p2	(AT)8	16	495	510	CACTTGTCGTCATGGGAATG
scaffold729059_11.9	1	p2	(TA)13	26	169	194	GCACTCTCCAGCAATGTTGA
scaffold729063_13.7	1	p2	(TC)9	18	492	509	TGGTGAAGAAAGTGGGCTTC
scaffold729077_14.3	1	p2	(TA)9	18	1325	1342	AGCAGTGGAACGAGGAAGAA
scaffold729100_12.6	1	p2	(CT)12	24	183	206	CGCATTTCATCTGCTTTTCA
scaffold729103_16.3	1	p2	(AT)7	14	186	199	GGGGGTGCAACATCATATTC
scaffold729105_12.5	1	p2	(AT)7	14	1231	1244	TTTGTTGGGTCTTGGGCTAC
scaffold729118_14.3	1	p2	(AT)6	12	741	752	CACCGAATATTCCCAACTCAA
scaffold729126_15.9	1	p2	(TG)6	12	918	929	CCTTCCCTTAAGCCCATCTC
scaffold729136_10.0	1	p2	(AC)8	16	735	750	GATAGCTCTGAGGCTGGTGC
scaffold729138_13.0	1	p2	(AT)6	12	576	587	CCACTTCTCCCATTTGCATT
scaffold729141_15.6	1	p2	(TA)6	12	31	42	CCGTAGCTGTTATTGATGGAA
scaffold729154_16.8	1	p2	(TA)6	12	267	278	CACCAGAATTTAGGCGGAGA
scaffold729176_16.2	1	p2	(TG)8	16	496	511	CCCGAATATCCCCCAACTAA
scaffold729179_15.3	1	p2	(AG)7	14	592	605	GAAGCCGAAGGAATCATCAC
scaffold729200_13.8	1	p2	(AC)7	14	3490	3503	TCTGAGCATTTGCAAGGATG
scaffold729229_18.7	1	p2	(TC)6	12	1272	1283	TAAGCAATCCCCATTTTTGG
scaffold729235_11.3	1	p2	(TC)9	18	1056	1073	CACAAGCTCGGAAGGGTAAA
scaffold729303_17.4	1	p2	(GA)6	12	1795	1806	CTGCAAAACTGGAGGAGAGG
scaffold729309_13.8	1	p2	(AT)6	12	502	513	ATCGATTAGGTCGCACAAGG
scaffold729316_15.0	1	p2	(GT)6	12	1465	1476	GCGCTGTAACCAACAACAGA
scaffold729337_20.0	1	p2	(AT)9	18	1171	1188	CAAAGCCCAATGGGAAACTA
scaffold729384_16.6	1	p2	(AT)13	26	217	242	GCAACTCGACATCAACGTCA
scaffold729414_16.4	1	p2	(TC)7	14	2087	2100	CGTCCCAAGAAATGTGACCT
scaffold729431_13.4	1	p2	(AT)10	20	490	509	TGGTTGATTAGATTACCGCTACAA
scaffold729441_18.0	1	p2	(TA)7	14	503	516	TCTCACGAGGATTTTCGATGT
scaffold729462_14.6	1	p2	(AT)11	22	933	954	AAAGGGCCTATATCCAATATGAAA
scaffold729473_16.4	1	p2	(TC)8	16	3224	3239	TGCGAATTTGGAATTATCTGC
scaffold729479_13.3	1	p2	(TA)6	12	62	73	GGGGTTATCATGGGATCAAG
scaffold729500_12.7	1	p2	(AT)10	20	180	199	AACGCAGATCTGATGGCAC
scaffold729504_17.0	1	p2	(CT)7	14	4612	4625	ACGGACCGTGTACTCTACCG
scaffold729518_13.8	1	p2	(AT)6	12	506	517	AACAATGCAGTAGCTGGTTGG
scaffold729545_14.8	1	p2	(AT)9	18	1109	1126	GGTGATTTTTGATTATAGGAATATGGA
scaffold729628_21.7	1	p2	(AG)14	28	46	73	GGAACAAAGACACCATTGATAAAA
scaffold729648_15.5	1	p2	(TA)8	16	81	96	TTTGATAACGTCAAATCCAACAA
scaffold729654_15.2	1	p2	(AG)10	20	1294	1313	CATTCCAATCATTCCCCAAC
scaffold729657_15.0	1	p2	(TA)7	14	654	667	TGACCCATTTCTCATCATTGG
scaffold729658_16.3	1	p2	(AT)9	18	1340	1357	TTTGTGCCTAAAATGCCCTC
scaffold729671_17.5	1	p2	(CT)7	14	143	156	TCCCAACTCACAATCCATGA
scaffold729676_14.9	1	p2	(AT)10	20	157	176	TTCTGCATGCATTTATTGGG

scaffold729678_12.8	1	p2	(AT)7	14	1232	1245	ACAAATGAACACTCCGGCAT
scaffold729682_13.0	1	p2	(GA)6	12	1502	1513	AGGCAGAAAAGAGGAGGGAC
scaffold729688_15.4	1	p2	(TA)9	18	1012	1029	GGATCCACGTCCACTATGCT
scaffold729692_17.6	1	p2	(AC)7	14	2941	2954	CTCGTTAGCAAGCAATGCAC
scaffold729700_11.8	1	p2	(TA)9	18	295	312	GGCTCCACAACCATCTTCTT
scaffold729701_15.2	1	p2	(GT)7	14	3029	3042	GCTGAAGAGGTCCAGCAATC
scaffold729706_15.3	1	p2	(AG)7	14	672	685	TCATGTGAGGATATGGCGAA
scaffold729707_16.3	1	p2	(CA)6	12	827	838	CAATGTCAGAGTGGTGGTGG
scaffold729761_11.8	1	p2	(AT)6	12	1350	1361	TCTTCTCGCATACCAAAGGT
scaffold729782_15.7	1	p2	(GA)9	18	503	520	GCGTGGGGAATAATGAGAGA
scaffold729810_14.8	1	p2	(TC)7	14	1091	1104	CCCAAGAAGCCCTTCTCTCT
scaffold729813_14.3	1	p2	(AT)6	12	1607	1618	AAATGGATGCTGATAAATGGA
scaffold729818_17.7	1	p2	(GA)7	14	1328	1341	CGAGAGAGCTTTTCCTTGACA
scaffold729824_15.4	1	p2	(AT)9	18	1244	1261	GGAAAAGAACTGCCAGAACG
scaffold729825_14.3	1	p2	(CT)10	20	317	336	TTTCCCCTCCCTCATTTACC
scaffold729839_13.6	1	p2	(AT)9	18	787	804	CCATTCCGTCGTTGTAGGAA
scaffold729896_13.0	1	p2	(AG)9	18	1755	1772	TGGCCTCAGATAGTTGGTCC
scaffold729897_14.8	1	p2	(AT)11	22	4285	4306	TGCAACGTGAGTAGTGACTCG
scaffold729919_15.4	1	p2	(TA)7	14	1204	1217	AAGGTACAAGTGGGTTCCCC
scaffold729940_15.2	1	p2	(AC)6	12	1953	1964	ATACCCAGACCCAAAGCAAA
scaffold729959_16.0	1	p2	(GA)6	12	582	593	CAACAAAAGCGTGTTCATGG
scaffold729977_14.4	1	p2	(GA)8	16	931	946	GTGCCAGAGATCGGAGAAAG
scaffold729991_14.2	1	p2	(AT)10	20	1643	1662	CACCACAAAGCAATTGGAAA
scaffold730036_14.4	1	p2	(GT)6	12	1297	1308	TGGGTGCCATTTCTATCACA
scaffold730050_19.9	1	p2	(TC)7	14	514	527	CTTAATGAGGGGCCACAAAG
scaffold730061_17.7	1	p2	(TA)6	12	602	613	TGATCTGGAAGTAAGGAATCCTAGA
scaffold730064_15.6	1	p2	(TC)9	18	2331	2348	GCAAAGACCATTCTTGAGGC
scaffold730069_15.7	1	p2	(TC)7	14	822	835	TTGGGTTGCATCAGAAAACTC
scaffold730102_12.4	1	p2	(CT)12	24	505	528	GATGAGAGAGAGAGCGGTGG
scaffold730113_17.3	1	p2	(GA)6	12	518	529	ACTCTCACGCACCGGAATAC
scaffold730123_17.2	1	p2	(AG)6	12	518	529	AAGTTGCATGCCATTTACCA
scaffold730145_15.6	1	p2	(GA)6	12	1614	1625	AATGCCCTCCTATTATGCCC
scaffold730159_16.3	1	p2	(TA)11	22	820	841	TATGGGTTATCACCGGATCG
scaffold730170_17.5	1	p2	(GA)6	12	430	441	GGGCGAAGTGATCGAGAATA
scaffold730186_14.9	1	p2	(TA)7	14	517	530	GGATTTTGGATTGGTCGAGA
scaffold730190_15.4	1	p2	(TC)9	18	170	187	CAATGTTGGTGACGTCTTGC
scaffold730222_14.4	1	p2	(GA)8	16	2582	2597	CACAGCTGGCATAACCAAGA
scaffold730227_15.5	1	p2	(GA)6	12	132	143	TCACGGATTCACCAACTGAA
scaffold730229_16.9	1	p2	(AC)6	12	2395	2406	TTGCAAACTTAATTGGTGCG
scaffold730230_13.1	1	p2	(AT)8	16	1207	1222	GCCCCCGATTTTTCAAAGTA
scaffold730246_15.0	1	p2	(AT)6	12	1189	1200	AATGCCACCATTGTTGGTTC
scaffold730256_16.5	1	p2	(AG)6	12	926	937	CTGGGTTGGTAGTTGCCAGT
scaffold730274_11.6	1	p2	(AT)13	26	67	92	AGAATTTCAAGGTAATGGGGA
scaffold730279_15.9	1	p2	(AG)8	16	103	118	GCACGTCTCTCGGACATACA
scaffold730290_14.3	1	p2	(TA)8	16	1056	1071	TCGAATTTGTGAACTCCGAA
scaffold730292_15.1	1	p2	(TA)9	18	2814	2831	TTTGCACTTAAGAGGAGTTTTGAA
scaffold730306_14.2	1	p2	(AG)6	12	1178	1189	GCATTGCAAGCTTCGATACA
scaffold730314_18.0	1	p2	(CT)6	12	955	966	GACCAGCGAGTCACACTCAA
scaffold730329_11.3	1	p2	(TA)6	12	118	129	GCGAAGCTAATGCATTCTGTC
scaffold730343_15.8	1	p2	(AT)8	16	469	484	GTTAAAGCCAAAAGTCGGGC
scaffold730347_13.1	1	p2	(AG)10	20	3547	3566	CAGCAGCAAGCAATAACGAG
scaffold730361_18.2	1	p2	(CT)6	12	1181	1192	CGAATGCTTTTTCTTCCACC
scaffold730364_12.8	1	p2	(CT)9	18	1002	1019	TTGCACTTCCTGGAGAACCT
scaffold730371_17.2	1	p2	(TC)6	12	516	527	ATTTTGTCGCAGTTGATCCC
scaffold730372_14.6	1	p2	(TA)6	12	2931	2942	CACACATGGAAAAGATCTCCAA
scaffold730402_16.5	1	p2	(TA)6	12	1242	1253	CTCCCAATTATGGACAGGGA
scaffold730417_14.6	1	p2	(TA)6	12	2652	2663	TAGTGGGGCAAGAAACAAGC
scaffold730418_12.3	1	p2	(TA)7	14	522	535	AACACATTTCTTCAACATTCATCA

scaffold730423_13.5	1	p2	(TA)9	18	110	127	ATCAACGGTTCACATCGGTT
scaffold730425_15.4	1	p2	(GT)6	12	731	742	TGGAGAACTCATTCTTACTGCG
scaffold730435_11.3	1	p2	(AT)7	14	959	972	ACGAACACACACGCACTCTC
scaffold730479_11.6	1	p2	(TA)7	14	1155	1168	GGCCAGATTTCGTCCCTATT
scaffold730494_12.3	1	p2	(TA)7	14	81	94	TGTTTGCACGTGAATAAGCC
scaffold730556_17.9	1	p2	(AG)6	12	143	154	GGTTCAACCATTTTGTTGGG
scaffold730579_12.0	1	p2	(AT)10	20	1000	1019	CGCCGACCAATCATGTAGA
scaffold730599_15.4	1	p2	(AT)8	16	3333	3348	TTGTGGATGCCCTAACCAAT
scaffold730607_13.0	1	p2	(TC)8	16	1581	1596	CCAAGCTTAAGCAAGCAAGC
scaffold730631_15.9	1	p2	(CT)6	12	530	541	TCTTTGACGTTAATTTTCGGAGA
scaffold730633_15.6	1	p2	(TA)7	14	2579	2592	CCTGAAAGAATTTGGGGGAT
scaffold730660_14.9	1	p2	(AG)6	12	953	964	ATCTCTGCCCCCATCTCTCT
scaffold730663_15.3	1	p2	(AC)6	12	4254	4265	ACCCTCCACCCTTTCTCTGT
scaffold730667_15.2	1	p2	(GA)7	14	715	728	CATGTCGTGTCGTGTCAGAA
scaffold730668_16.2	1	p2	(AG)7	14	4517	4530	GTACCTCTGTCCTTGCAGCC
scaffold730672_13.4	1	p2	(TG)6	12	55	66	TTGTGTGATCGAACCACGTT
scaffold730678_12.6	1	p2	(CT)11	22	1385	1406	GTCTAGCCGGCATCGATAAT
scaffold730713_15.2	1	p2	(TA)6	12	2400	2411	AAAAGAGATGCGTGCAAGGT
scaffold730761_16.3	1	p2	(AC)8	16	1365	1380	ACGGTGTCTCCGCAATTAAC
scaffold730768_16.4	1	p2	(TC)6	12	146	157	GGCTATGGGTGGTAGGACAA
scaffold730806_14.2	1	p2	(CT)7	14	615	628	CAATAAAGCCCGTCACGTTT
scaffold730834_15.0	1	p2	(TC)6	12	533	544	GTGGTGCGATGATAACGATG
scaffold730836_11.0	1	p2	(AT)11	22	35	56	AGCCGTGAGATTTACTTTTAACTT
scaffold730862_15.4	1	p2	(TA)11	22	43	64	GCGTTTTGACAATATATAAGTGTTTTG
scaffold730881_16.8	1	p2	(GA)10	20	1189	1208	CCAAACCAAAACTCATCAACC
scaffold730884_17.7	1	p2	(AG)7	14	533	546	TCTCCCAACACACCTTTTCC
scaffold730888_16.6	1	p2	(AG)9	18	1153	1170	GAGGATAACTGGCCAACCAA
scaffold730906_13.8	1	p2	(AC)8	16	1969	1984	CAACTCCCCCGTAGTTTTCA
scaffold730910_15.9	1	p2	(GA)6	12	2403	2414	GTTGGTGTTTGCTGGGATTT
scaffold730911_16.5	1	p2	(TC)8	16	1259	1274	TCGAGCTGAACGAAAGAGGT
scaffold730916_18.0	1	p2	(CT)10	20	525	544	CCCCACCTTAGCGTTAAACA
scaffold730932_15.5	1	p2	(AG)9	18	530	547	ACCCAAAACAAAATGCATGA
scaffold730934_14.3	1	p2	(TC)6	12	506	517	TATGGCAAAAACACCAACGA
scaffold730942_17.5	1	p2	(TC)7	14	534	547	ACTCAACTCTGATCGGTGGG
scaffold730951_18.1	1	p2	(AT)6	12	660	671	GGAGATGGAGGGTTCAAGGT
scaffold730956_15.7	1	p2	(TA)6	12	318	329	GGTTTATTGTGGCGAATGCT
scaffold730964_14.7	1	p2	(AT)14	28	1099	1126	GGTGTTGGGAGGTGAACAAT
scaffold730982_17.0	1	p2	(CT)6	12	554	565	CCAATAGGCCCATTCAAAGA
scaffold730992_15.1	1	p2	(AG)6	12	1496	1507	CTCAATGCGTAGGCTTCTCC
scaffold731000_16.2	1	p2	(TA)8	16	1095	1110	AAACCGCACTTTAGCTCGAA
scaffold731007_11.7	1	p2	(TA)8	16	534	549	AAATTCCCTTGTATGCTGCG
scaffold731011_15.1	1	p2	(AC)7	14	831	844	AGTTTCGATTCGATTGACGG
scaffold731028_15.5	1	p2	(AG)8	16	2523	2538	GATTCTAGCATCGTGAGGGC
scaffold731029_13.4	1	p2	(TA)9	18	913	930	TTCCAACAATCTCAGCATCA
scaffold731034_14.1	1	p2	(TA)9	18	918	935	AAGTATTGATCGACGTGGGA
scaffold731049_15.9	1	p2	(CT)6	12	198	209	AACACAGGTCCGCAAATAGG
scaffold731054_15.6	1	p2	(GA)8	16	1529	1544	CTGCCTCCCTTGTTCTTACG
scaffold731082_14.9	1	p2	(CT)7	14	793	806	TCCCTAATCATCTCCCCTCC
scaffold731100_16.4	1	p2	(CT)8	16	561	576	CCTAGAGCACACTCGAAGGG
scaffold731114_15.6	1	p2	(CA)7	14	2804	2817	CCTCCTTCCATAGCAGCAAA
scaffold731121_16.7	1	p2	(AT)9	18	3464	3481	TGATCAAAATCCACACCTCG
scaffold731137_12.3	1	p2	(TG)7	14	537	550	CAGAGTTTGTTTCGCAACCC
scaffold731143_12.1	1	p2	(TC)6	12	77	88	ACTGGTGGAAACTGGATTGC
scaffold731172_16.0	1	p2	(AG)6	12	541	552	CCACCTCTCTGCTCCACTTC
scaffold731183_14.0	1	p2	(TA)10	20	255	274	TTTGAACCCAAACCTCAAGC
scaffold731192_14.3	1	p2	(AT)9	18	385	402	AGGAAGCGAGGTAGGCTTTC
scaffold731196_14.5	1	p2	(CA)8	16	235	250	GCCAGATTCTCTTGGTGGAG
scaffold731223_15.1	1	p2	(AT)6	12	54	65	CCTTTCTCCATGGGCATCT

scaffold731236_15.[illegible]	1	p2	(AG)8	16	1299	1314	TCGTACGTACTGTGATCCGC
scaffold731260_13.[illegible]	1	p2	(TC)8	16	1979	1994	GGACAAAACGACCAAAAGGA
scaffold731278_15.[illegible]	1	p2	(TA)6	12	1195	1206	TCTCTCACCTCCTCCCATGA
scaffold731299_17.[illegible]	1	p2	(TG)9	18	362	379	ATCCAAGCAACAAAAATGGC
scaffold731307_14.[illegible]	1	p2	(AT)7	14	253	266	GCATATGCATGGTTCTCACG
scaffold731352_16.[illegible]	1	p2	(CA)8	16	542	557	TGTTGGTCCACCATTCTTTT
scaffold731383_14.[illegible]	1	p2	(TA)7	14	52	65	GAAATTAGATTAAAGAAAAGATGGT[illegible]
scaffold731391_16.[illegible]	1	p2	(AG)8	16	2519	2534	TGGGGTGAATCAGTTCAACA
scaffold731405_14.[illegible]	1	p2	(TA)11	22	129	150	CGAGTTAGGAAATAAGGAGGTGC
scaffold731434_16.[illegible]	1	p2	(AC)8	16	544	559	GCAGCTCAAAAAGACAGTCCA
scaffold731439_14.[illegible]	1	p2	(TA)12	24	402	425	CACTTTTTGTGAAACCCCTCA
scaffold731450_12.[illegible]	1	p2	(AT)9	18	1165	1182	GGGGATTCCTGCAATTTTCT
scaffold731515_15.[illegible]	1	p2	(TC)7	14	316	329	ATTCAAACCGCTAGTGTGGC
scaffold731518_16.[illegible]	1	p2	(GA)6	12	549	560	TCCCCATTAATCCCATCAGA
scaffold731525_15.[illegible]	1	p2	(GA)8	16	1204	1219	CCGGTTTCTTTCCATTTTCA
scaffold731528_17.[illegible]	1	p2	(AT)8	16	147	162	TTCCGTGTGCGTATTCTTGA
scaffold731554_17.[illegible]	1	p2	(GA)9	18	545	562	AAATTATTCAACTTGGATCACTCAT
scaffold731567_15.[illegible]	1	p2	(TG)9	18	545	562	ACTTGTCAATGCCGGTAAGC
scaffold731570_16.[illegible]	1	p2	(TA)8	16	916	931	TCACAACGGGGTCAAATGTA
scaffold731618_13.[illegible]	1	p2	(CA)7	14	935	948	ATCAGGCTCGAGTCCATGTC
scaffold731620_10.[illegible]	1	p2	(GA)6	12	552	563	CGGCATGAGATGAGATGAGA
scaffold731632_16.[illegible]	1	p2	(AG)8	16	319	334	AACAGAGCAAGACGAGGCAT
scaffold731664_17.[illegible]	1	p2	(AG)6	12	1411	1422	CTGCTTGAGTTGCTCTGCTG
scaffold731683_15.[illegible]	1	p2	(TG)7	14	68	81	GCCAATCACTAACCAACACG
scaffold731705_16.[illegible]	1	p2	(TA)8	16	1496	1511	TCCGCCCTCGTGTTTTATTA
scaffold731706_15.[illegible]	1	p2	(TG)6	12	469	480	TTCATTCATGGTCGTAGGCA
scaffold731712_17.[illegible]	1	p2	(TG)6	12	2533	2544	CATTGCCATTGCAGAAGCTA
scaffold731717_14.[illegible]	1	p2	(AT)9	18	548	565	TTAAGGTGTCAGAGGCGGTT
scaffold731721_16.[illegible]	1	p2	(TG)6	12	2460	2471	TGTACATGATGCATTTTCCCA
scaffold731735_14.[illegible]	1	p2	(CA)6	12	2317	2328	ACCAACCAGCTTAGCGAAAA
scaffold731752_16.[illegible]	1	p2	(AG)7	14	1630	1643	CAAACCCAATATCACAGCCC
scaffold731757_15.[illegible]	1	p2	(TC)8	16	1864	1879	AAAAAGTCCCCAGCCAATCT
scaffold731767_16.[illegible]	1	p2	(TA)6	12	445	456	CCACTAACCTTGTCTCCCCC
scaffold731805_17.[illegible]	1	p2	(AG)6	12	847	858	GGTGTGCTCGGATCTTGATT
scaffold731809_17.[illegible]	1	p2	(TA)6	12	557	568	AAGATTCTCTCTTGTAGGTTGTCCA
scaffold731830_12.[illegible]	1	p2	(CT)6	12	753	764	GCCCTAGGGTTCCTGATTTC
scaffold731831_20.[illegible]	1	p2	(GA)6	12	1298	1309	CGCAATGATAGACCACAACG
scaffold731834_15.[illegible]	1	p2	(CT)7	14	127	140	AATCCCCCACCACTTTCTCT
scaffold731879_16.[illegible]	1	p2	(TC)11	22	5994	6015	AGTGCATTGACATCGAGACG
scaffold731896_15.[illegible]	1	p2	(AT)6	12	1370	1381	AATCGTGAAATCAAACCAGAA
scaffold731906_11.[illegible]	1	p2	(TA)8	16	555	570	CCACCCGTTTACCTTTTTCA
scaffold731933_16.[illegible]	1	p2	(TG)7	14	2089	2102	AGAAGCTTGTTGTCCGCTGT
scaffold731939_13.[illegible]	1	p2	(AT)9	18	201	218	GAATTGTTCCTGCTCGTGGT
scaffold731949_15.[illegible]	1	p2	(AC)7	14	152	165	CACTGGATCTGCCGATGTTA
scaffold731954_14.[illegible]	1	p2	(AC)8	16	295	310	CCATATGATGCTGATGCGAT
scaffold731998_14.[illegible]	1	p2	(AT)8	16	704	719	AGTCACGTCTATTCATCATTGACA
scaffold732005_16.[illegible]	1	p2	(AG)6	12	1075	1086	TCTCACAAATCCACACCCAA
scaffold732031_16.[illegible]	1	p2	(TA)13	26	92	117	TTTAAGATGTGCATGCATTTGA
scaffold732039_14.[illegible]	1	p2	(CA)7	14	3809	3822	CGCAAACGATGGCAATAAT
scaffold732054_14.[illegible]	1	p2	(TA)6	12	749	760	ATTTTGCACCGCCATATCAT
scaffold732066_16.[illegible]	1	p2	(CT)6	12	185	196	AACGCGAAGTAGCATGGTTT
scaffold732086_14.[illegible]	1	p2	(CT)7	14	250	263	ACGACGCAAAAATGGAAAAC
scaffold732089_14.[illegible]	1	p2	(GT)6	12	627	638	ATGCCACCACTTCCCTGATA
scaffold732130_13.[illegible]	1	p2	(AT)10	20	2229	2248	CCAGATGTGAGGAGCAAACA
scaffold732131_15.[illegible]	1	p2	(TA)6	12	309	320	TTCTAACGGCCCAGATTTTG
scaffold732154_13.[illegible]	1	p2	(TA)11	22	185	206	CAAAAATCTAACGGGCCAAA
scaffold732173_16.[illegible]	1	p2	(AT)9	18	399	416	CCACGCTTCATTATCAACCA
scaffold732187_13.[illegible]	1	p2	(AT)13	26	753	778	TGCTCTCAATTAAATACACCCG

scaffold732233_14.7	1	p2	(AG)9	18	561	578	GCCAGCAGTAAAGAGGGTGT
scaffold732238_13.7	1	p2	(TA)9	18	657	674	AGATCAACGGATGGTATCGG
scaffold732257_15.1	1	p2	(AC)7	14	1162	1175	TGCCATTATGGTGCATTGTT
scaffold732273_14.6	1	p2	(TC)6	12	1440	1451	TCCACTCTCTTGCGCTTTTT
scaffold732279_16.1	1	p2	(TA)8	16	2394	2409	CAAGATGATGAGGGCCAAAT
scaffold732288_16.4	1	p2	(TC)7	14	664	677	GGAGACCAATTCTTGGCTGA
scaffold732298_16.8	1	p2	(AT)7	14	2647	2660	CAATAAATTGGGGTCGGCTA
scaffold732299_12.6	1	p2	(TA)7	14	50	63	TGGTTATTACTGGAACGCGA
scaffold732309_16.1	1	p2	(CT)8	16	454	469	CGGCCTATCGTCTTCCTACC
scaffold732346_16.0	1	p2	(TC)7	14	5937	5950	TGCAATGCTAATGGCAACTC
scaffold732348_14.8	1	p2	(GA)8	16	566	581	TGCAACCACCACTGGAGATA
scaffold732355_14.6	1	p2	(TA)6	12	570	581	GCAAAAGTGCCTACTTCACCA
scaffold732357_14.9	1	p2	(AT)6	12	744	755	AAACCAACTATCTCGGTGCG
scaffold732360_13.5	1	p2	(TC)7	14	1008	1021	GACCTGGTGTCCCATCTCTC
scaffold732372_14.0	1	p2	(CT)6	12	608	619	GCAATCTATCCCATACAAGGC
scaffold732380_15.5	1	p2	(TC)6	12	198	209	TCTTCTCTCAGGTGGGGGAT
scaffold732386_16.9	1	p2	(TC)7	14	569	582	TGCATCAAGAGAACAACTACATCA
scaffold732408_13.5	1	p2	(AT)7	14	1297	1310	TCCGGATCATTGACATTTGTT
scaffold732420_15.2	1	p2	(TA)10	20	989	1008	ATCTGTCTCCTGCCCATGAC
scaffold732423_17.9	1	p2	(TA)6	12	783	794	ACAGTGGCTAAAATGCACCG
scaffold732452_16.2	1	p2	(AC)9	18	2435	2452	GGGCTTTGTTGCTGGAGTAT
scaffold732460_14.2	1	p2	(CA)7	14	572	585	CCTGCATCACAAAAGCAAAA
scaffold732478_16.5	1	p2	(TG)11	22	49	70	TTTCTTGACTGTGGATTTCCAAT
scaffold732503_13.1	1	p2	(CT)8	16	637	652	TGTTGGTTTGTGAGGTCAGC
scaffold732512_18.6	1	p2	(AG)8	16	1274	1289	CGTTGTCTCCGCTACTGTTG
scaffold732521_17.1	1	p2	(TA)6	12	5132	5143	ATCGGATTCTGGGGGATAAC
scaffold732547_15.4	1	p2	(AG)7	14	1831	1844	GCATGCAAAGTTGCAAACAA
scaffold732557_15.4	1	p2	(TA)9	18	938	955	GCAGCAACACCAAAACTCAA
scaffold732558_13.2	1	p2	(GA)6	12	679	690	TGAAGGCACAGAGCATCATC
scaffold732576_15.4	1	p2	(GA)6	12	147	158	GGTAGAGGAGCTGATGTGGC
scaffold732594_14.4	1	p2	(TA)7	14	628	641	CATATGGTGTCAGGTTTGGG
scaffold732613_17.4	1	p2	(TA)7	14	651	664	ACATCAGTTGTAGCTGGGGG
scaffold732627_14.5	1	p2	(AT)6	12	535	546	ACATGCCCTAAAAACCGATG
scaffold732630_14.0	1	p2	(CT)6	12	381	392	AGACCAAACCTCAAGCATGG
scaffold732637_15.3	1	p2	(TG)6	12	1250	1261	TTGAGCCCCTTTTATGGAAA
scaffold732645_15.8	1	p2	(TA)6	12	1617	1628	TCCAACAGCTCAAACAGCAT
scaffold732661_15.6	1	p2	(GA)6	12	755	766	GGTGGAACTGATTGGGAGAA
scaffold732695_16.2	1	p2	(AG)6	12	579	590	TGCCTTGTGTGTCACGAGTT
scaffold732738_19.3	1	p2	(AG)6	12	581	592	TCCCTTCTTACATATGGCCC
scaffold732748_16.0	1	p2	(AC)8	16	577	592	CTGAGTACCCCGTCTTCCAA
scaffold732749_15.1	1	p2	(AG)7	14	2771	2784	CCATTATGTGTGCGAGTGGT
scaffold732754_15.5	1	p2	(CT)9	18	568	585	AAAACCACGTCGTTCTTGCT
scaffold732764_15.9	1	p2	(AC)7	14	1066	1079	CCAATGTATCATCCCTCGCT
scaffold732767_12.0	1	p2	(TA)7	14	1690	1703	CTGCTCCTCTCATGGTCAAT
scaffold732782_16.4	1	p2	(GT)6	12	247	258	AAAATCCAATTCGAATCCCC
scaffold732792_18.0	1	p2	(TC)6	12	2321	2332	TTGATTCCATGAACACCCTTT
scaffold732810_13.8	1	p2	(TC)8	16	119	134	CAGGGGATCTCAAGCCTGTA
scaffold732833_12.7	1	p2	(CT)7	14	565	578	CCCGACTCCAAAATGAAGAA
scaffold732856_12.0	1	p2	(AG)7	14	381	394	CAGCTGAACCAACCATCCTT
scaffold732859_13.4	1	p2	(TA)8	16	349	364	CCACCATGATTTCGACACAC
scaffold732862_15.8	1	p2	(CT)7	14	254	267	GCGAGCAGACCAGAAGTCA
scaffold732869_15.7	1	p2	(AT)10	20	599	618	GGAAGGGAAGAATCCTCGAA
scaffold732903_11.4	1	p2	(AT)7	14	903	916	TCGTGAGAAATTAATGGAACAAAA
scaffold732909_15.0	1	p2	(TA)8	16	1474	1489	CACTTGCTTGGAAAAAGGGA
scaffold732928_15.7	1	p2	(CT)6	12	586	597	TTCGGGAAAATGAACTCACC
scaffold732938_14.3	1	p2	(TC)6	12	2551	2562	GCCAGTTCTTTTTCATGGGA
scaffold732954_15.3	1	p2	(CT)6	12	918	929	GATCAAACACGCATCACCAC
scaffold732962_14.0	1	p2	(GA)7	14	1415	1428	ACAACACGGAACCACTAGGC

scaffold732966_15.2	1	p2	(CA)8	16	90	105	GCCGGACTTTGATTGAGTTC
scaffold732975_15.8	1	p2	(AG)8	16	1312	1327	ACCTGATCACTTGTCCGAGG
scaffold733003_17.3	1	p2	(AT)7	14	2227	2240	AGTGAACCATGGGTCCGTTA
scaffold733011_15.9	1	p2	(AG)6	12	1344	1355	TTCAGCAACAGCTTGACCAC
scaffold733020_13.1	1	p2	(GA)8	16	2452	2467	TCTCCGATAGGCAAAACGAC
scaffold733058_12.6	1	p2	(AT)11	22	79	100	GAAAAACCGAAAACCGAACA
scaffold733065_10.2	1	p2	(AT)9	18	1179	1196	AGAGCAACACATGCCAACAT
scaffold733087_14.4	1	p2	(TA)6	12	53	64	TCATTTGTTAGAGGTTTTGACCA
scaffold733098_14.6	1	p2	(TA)10	20	565	584	ACATCGGCTCAAACTCCAAA
scaffold733100_12.5	1	p2	(TA)9	18	233	250	CATGAGCCATTATGTGCTGG
scaffold733128_14.8	1	p2	(TA)11	22	41	62	TTTGATGATATATAGGTGTTTTGACC
scaffold733148_14.7	1	p2	(TA)6	12	259	270	TTGGGGTATGGGAATAGCTT
scaffold733172_15.9	1	p2	(AG)9	18	6201	6218	GTCACCTGTCACCAGAGCAA
scaffold733187_15.2	1	p2	(TG)11	22	583	604	GAAAGCCCCCTTTTCTCAAC
scaffold733216_18.4	1	p2	(AT)7	14	2493	2506	TTGCACAGTACATGCGTTCA
scaffold733238_15.0	1	p2	(AT)9	18	2470	2487	CGGACAAACACGACAACATC
scaffold733247_16.8	1	p2	(AT)6	12	595	606	GATTCTGCTGAGGGTCGTTT
scaffold733264_16.8	1	p2	(TC)6	12	222	233	TACTGCTCCATCTTCAACCG
scaffold733267_14.6	1	p2	(GT)6	12	54	65	CAAGAGTAGAGAAAATCCTCCCA
scaffold733276_14.4	1	p2	(CT)13	26	270	295	GGAGTGGAGTGTGAGTGGGT
scaffold733302_13.6	1	p2	(CT)6	12	2219	2230	CAACTGACCAACAACAGCCA
scaffold733306_14.6	1	p2	(GA)8	16	186	201	TTTCTCTCTCCTCTCGCTGC
scaffold733348_16.0	1	p2	(CT)6	12	206	217	ATCTCGGCCTAGCTGGATTT
scaffold733360_11.3	1	p2	(TA)11	22	292	313	TAGACCCAGGCTTCGAGGTA
scaffold733374_12.6	1	p2	(CT)6	12	598	609	ATTCCATCACCCAACTCTCG
scaffold733396_12.8	1	p2	(TA)11	22	1967	1988	AGCAACCATGCAATTAAGGG
scaffold733402_14.5	1	p2	(GA)8	16	1668	1683	TCATCAACGAAGAAACGCTG
scaffold733450_17.0	1	p2	(AG)7	14	1959	1972	GATGCCCTCCACATCAGATT
scaffold733457_16.8	1	p2	(AT)6	12	741	752	GTAGTTTACCTGGCTGGCGT
scaffold733479_12.0	1	p2	(AT)10	20	1172	1191	TGTGAAGGCCTACGAACTGA
scaffold733507_14.9	1	p2	(TA)8	16	201	216	ATCTTGACCGCTGATTTTGG
scaffold733509_16.7	1	p2	(TG)6	12	9357	9368	TTCGAGGGGATTAATGATGG
scaffold733510_16.7	1	p2	(AT)6	12	1895	1906	ATTGGCAATTTTTGAAACGC
scaffold733540_11.3	1	p2	(AT)11	22	1131	1152	GAAGCAACCTTCCTCTGCAT
scaffold733556_17.0	1	p2	(AT)6	12	2655	2666	TTCCATCCCTCTCCAATGAG
scaffold733573_18.4	1	p2	(TA)6	12	603	614	AGCAATGAGCAACATTCAGC
scaffold733575_17.0	1	p2	(CT)6	12	34	45	TTTTAATTCACGCACAATTACATTT
scaffold733588_12.4	1	p2	(AT)11	22	429	450	TGTTGAATGTTGTGCCTCGT
scaffold733595_16.5	1	p2	(AG)8	16	203	218	TACAAGCACAACAACACGCA
scaffold733610_16.3	1	p2	(CA)6	12	2608	2619	TCAAACACTGCGCTTCAGTC
scaffold733613_16.4	1	p2	(GA)6	12	815	826	GGTTGCACCTCTGTTTTCGT
scaffold733617_13.7	1	p2	(AT)7	14	254	267	CCAACTACGTGGGTGGTTTT
scaffold733647_16.5	1	p2	(AG)6	12	384	395	CCATCTGGCATTAGTTGCCT
scaffold733669_16.6	1	p2	(AG)6	12	484	495	TGTTGCAAAATGCTGGTGAT
scaffold733671_17.6	1	p2	(TC)10	20	149	168	TGTGTTGGTCTGAGAGGCTG
scaffold733674_12.1	1	p2	(TC)8	16	971	986	AGGTTTTTCAAGGAGTGCCC
scaffold733679_15.4	1	p2	(TC)8	16	1324	1339	GAGGACAGGAATGTTTCTGGA
scaffold733719_14.7	1	p2	(CT)6	12	607	618	TAAAGAATGCCCACTCCCAG
scaffold733745_18.2	1	p2	(TC)7	14	607	620	AGCAGAAGGCACCGAACTTA
scaffold733751_14.2	1	p2	(TC)7	14	5101	5114	CATGTCTCCCCTACTTGCGT
scaffold733770_15.1	1	p2	(AT)8	16	1226	1241	TGAAAAGGCCATTCGAGACT
scaffold733778_17.0	1	p2	(GA)10	20	2343	2362	GCTGACGAAAGGAGTGGAAC
scaffold733802_14.4	1	p2	(GA)9	18	1493	1510	ATGGCTTCACCAACCTTTTG
scaffold733820_16.2	1	p2	(AT)8	16	66	81	CGGCTTGGATTATTTCCTATTTC
scaffold733843_14.1	1	p2	(TA)8	16	52	67	TTAGTTAGTACCGATTTCAGGGTT
scaffold733849_15.8	1	p2	(CT)6	12	45	56	CCGAAAATACAGGCGCATA
scaffold733858_15.0	1	p2	(TG)6	12	951	962	GGTTTCCTTAACTCTGCGAAA
scaffold733864_11.7	1	p2	(AT)9	18	70	87	CAAAAACAACTTCAATATGGATCTC

scaffold733882_15.4	1	p2	(TC)6	12	1828	1839	TGGCTTCTGCACTTTCCTTT
scaffold733884_17.7	1	p2	(AG)6	12	80	91	CCAATTTTCTCTTCGGGCTA
scaffold733888_16.7	1	p2	(CT)6	12	133	144	ACCCCGTGATTGTACGAAAA
scaffold733893_11.9	1	p2	(TC)7	14	610	623	GGCCAAATTGCAAGTTCATT
scaffold733896_13.7	1	p2	(TC)12	24	900	923	TCATTTTCGCCCGATACTTC
scaffold733907_16.2	1	p2	(AG)6	12	1206	1217	TCTGATAGGATTGGCTCGGT
scaffold733910_17.8	1	p2	(CT)10	20	606	625	GAAGGTTCTGCAACGGAAAG
scaffold733913_17.3	1	p2	(CT)6	12	1794	1805	AGCACGTGTTGAGGCTTCTT
scaffold733920_16.5	1	p2	(AC)7	14	1673	1686	ACTGTGGATGGAGTTCGGTC
scaffold733958_15.0	1	p2	(AG)6	12	1549	1560	AAATTGAGAGGCTCCAACGA
scaffold733976_14.8	1	p2	(AT)6	12	323	334	CCATGCACGTAAGCATGTTC
scaffold733977_14.5	1	p2	(CT)10	20	2187	2206	TATTAGGCCACCCCCTAACC
scaffold733986_14.8	1	p2	(TC)6	12	8369	8380	GCTTGCTCTTGCAACCTCTT
scaffold734001_14.3	1	p2	(AG)9	18	876	893	CCCAACAAACCCTTTCTCAA
scaffold734012_13.8	1	p2	(CT)7	14	1745	1758	AGTACGGCAGTGGTAGGTGG
scaffold734024_17.7	1	p2	(GA)6	12	1305	1316	AGATGCATAGGTGTCTGCCC
scaffold734026_19.5	1	p2	(AG)6	12	2732	2743	CTTCCATTCCATTCCACACC
scaffold734054_14.4	1	p2	(TA)8	16	1020	1035	CCCAAAAATGGTTTCACACA
scaffold734072_15.8	1	p2	(TA)7	14	377	390	ATCCGACCTAATGAAAGCGA
scaffold734086_12.2	1	p2	(GA)8	16	344	359	GTGAGGTGGGCAGAGTCAGT
scaffold734094_16.1	1	p2	(AG)6	12	4040	4051	TCCAACCAACATCAAATGGA
scaffold734103_16.2	1	p2	(AG)9	18	440	457	CAAATGGGAATCCATGAAGG
scaffold734105_16.5	1	p2	(AG)8	16	836	851	CCGACAATAAGAAGAGAGGCA
scaffold734148_14.5	1	p2	(TA)11	22	166	187	AGCCACTGAACCAAGTGACC
scaffold734151_16.0	1	p2	(TA)7	14	620	633	ACAATGATATTCGCGAAGGC
scaffold734170_15.1	1	p2	(CT)6	12	623	634	TGGCCTCTAATCAGTTCGCT
scaffold734173_15.5	1	p2	(GA)11	22	3527	3548	TCCAAAAAGAGGATCCCAGA
scaffold734193_16.2	1	p2	(TA)6	12	667	678	GGGTTTCTTACCCGAGCTTC
scaffold734200_14.6	1	p2	(GA)6	12	1650	1661	ATTCAAAAGCTGGGATGGTG
scaffold734202_12.4	1	p2	(AG)6	12	958	969	GAGATTTAGTGCGCGAAAGG
scaffold734203_13.3	1	p2	(GA)6	12	3018	3029	TGGAAAAGGCATATTGTGGG
scaffold734208_13.6	1	p2	(GA)7	14	248	261	ATGGGAAGATGTGATGAGGG
scaffold734228_15.4	1	p2	(TC)6	12	1184	1195	GCACCACCCTACCCTTCTTT
scaffold734235_16.1	1	p2	(TC)8	16	2817	2832	GACAAGCAAAGGGTTGGTGT
scaffold734240_13.5	1	p2	(AC)6	12	283	294	TTGTCAACCAACACCTGCAT
scaffold734253_17.7	1	p2	(AC)6	12	1160	1171	CCAATGTGCCCAGAGAAGTT
scaffold734269_12.0	1	p2	(AG)7	14	425	438	TTTCTCGCATTGGTCAATCA
scaffold734309_14.3	1	p2	(AT)12	24	116	139	GGTGCTGTGTGATGATGGAG
scaffold734349_12.6	1	p2	(TA)6	12	446	457	GCTCGCTTTCATTTCACACA
scaffold734350_16.6	1	p2	(TG)7	14	1316	1329	CTCTTTTGTTTTCCATGCCC
scaffold734352_15.5	1	p2	(GA)6	12	1114	1125	TTGTGTGAAGCGGAGAGATG
scaffold734354_16.1	1	p2	(AC)8	16	1286	1301	GAGCTCTTCAGTGGCGAAAC
scaffold734356_14.0	1	p2	(TA)7	14	1492	1505	TGTTAAACGGACTGGCCAAC
scaffold734360_17.4	1	p2	(GA)7	14	865	878	CGTGTAGAATCGCCTTCCTC
scaffold734380_15.0	1	p2	(AT)6	12	2005	2016	TTTGGGTTGGAGATGGTTTT
scaffold734392_14.8	1	p2	(AT)8	16	520	535	CAAACGAAAAATTATCAACTGTAGGA
scaffold734402_15.7	1	p2	(AT)6	12	4939	4950	ACCAAGTTGAGGGCATTGAA
scaffold734463_14.8	1	p2	(CT)8	16	2282	2297	TGACCGGATCTCTCACTCCT
scaffold734469_14.7	1	p2	(CT)6	12	3323	3334	ACAAAACCATCAGCCACCTC
scaffold734481_14.3	1	p2	(AT)6	12	1328	1339	GGGGACTTTTTAACCAACCAA
scaffold734484_17.1	1	p2	(AC)9	18	29	46	TGTGAGTGTGTGTGCAATGA
scaffold734507_16.6	1	p2	(AG)7	14	631	644	AGGGAATATCAGGGGAATGG
scaffold734517_16.3	1	p2	(AG)12	24	617	640	TTGAATCGAACTGGAGGAGG
scaffold734524_15.8	1	p2	(AG)7	14	402	415	GGAAATCCAAGGCTACACGA
scaffold734541_14.0	1	p2	(AT)6	12	2147	2158	ACAGGATAACGGATGCCTTG
scaffold734571_14.9	1	p2	(CT)6	12	1945	1956	GGGGTGGTCCTCATTCTAAA
scaffold734592_16.9	1	p2	(TA)8	16	1527	1542	TCGCGTGTCATTCTAGACCA
scaffold734597_15.3	1	p2	(TA)9	18	630	647	GGCCTTCAAGCAAAATCAAA

scaffold734629_15.[illegible]	1	p2	(AG)7	14	5793	5806	GTCATCGAGCCTAATCGAGC
scaffold734634_15.[illegible]	1	p2	(GA)11	22	55	76	GCGATTTTAACAAAAACGGG
scaffold734642_16.[illegible]	1	p2	(CT)6	12	1126	1137	CATTTTCCCAAACCCACAAC
scaffold734646_15.[illegible]	1	p2	(CT)7	14	468	481	GGTGACAAGTGGCATGTTTG
scaffold734648_15.[illegible]	1	p2	(GT)9	18	631	648	CATTCAAAAGCTTGCTTCCC
scaffold734651_16.[illegible]	1	p2	(GA)9	18	576	593	TCCCCACGGTCTAATGTAGC
scaffold734656_13.[illegible]	1	p2	(GT)7	14	633	646	CGCATTTGTCTCTCACCTCA
scaffold734657_15.[illegible]	1	p2	(CT)9	18	632	649	AGACTGCACCTGCGGTAACT
scaffold734677_16.[illegible]	1	p2	(TG)7	14	1785	1798	CCTGGTGTCCACTGTGTTTG
scaffold734684_17.[illegible]	1	p2	(GA)6	12	569	580	CGGTTGATATTTGTTTGGGG
scaffold734695_14.[illegible]	1	p2	(CT)11	22	1060	1081	TGATGTGGAGCTTCTTCACG
scaffold734734_16.[illegible]	1	p2	(CT)12	24	1965	1988	CAAGGTCTGACGAAGTCGC
scaffold734745_16.[illegible]	1	p2	(AC)7	14	1923	1936	CAGCCCTTGGCTCTTTTAGA
scaffold734760_16.[illegible]	1	p2	(AG)8	16	868	883	TTGCACTCTCCTTTGTGTGG
scaffold734771_15.[illegible]	1	p2	(TA)6	12	2903	2914	TTTGCAGTCATACTCCTCGG
scaffold734813_14.[illegible]	1	p2	(AT)6	12	341	352	CCAAGCAAACTACAAACCCA
scaffold734817_15.[illegible]	1	p2	(TA)9	18	88	105	TCTTCCAACGTCAAAGTTGCT
scaffold734834_17.[illegible]	1	p2	(TA)7	14	985	998	TTGTCTATGCCGGTTTTTGA
scaffold734870_14.[illegible]	1	p2	(GA)6	12	670	681	AATTTCGGTTACATGGCAGC
scaffold734895_15.[illegible]	1	p2	(AT)9	18	50	67	TTGTTAGAGGTCTTGACGGGA
scaffold734911_13.[illegible]	1	p2	(TA)10	20	439	458	TTTGGGGAGGAGAAAAGAGAA
scaffold734917_15.[illegible]	1	p2	(TA)7	14	49	62	CTTATCCAACTCAATTTATATGATCG
scaffold734921_11.[illegible]	1	p2	(CA)6	12	1076	1087	ATGCGATGTGTGGTCGAATA
scaffold734937_14.[illegible]	1	p2	(AT)7	14	70	83	GGGTTATCACCTGATTACTACCCT
scaffold734940_16.[illegible]	1	p2	(AT)10	20	1243	1262	TCGTGCGATGTATGATGTGT
scaffold734951_15.[illegible]	1	p2	(CT)7	14	647	660	GGGAGGAAGATGCAACTCAG
scaffold735003_14.[illegible]	1	p2	(GA)8	16	2237	2252	AAAGGCCACGTGTTAATTGC
scaffold735037_15.[illegible]	1	p2	(TG)7	14	927	940	TTTCCTTTATGATCCCCTGC
scaffold735043_15.[illegible]	1	p2	(GA)6	12	349	360	TTGCTAGGGTTTCGTTTTCG
scaffold735067_15.[illegible]	1	p2	(AC)9	18	647	664	ATGCATTGCGACCATTATCA
scaffold735091_15.[illegible]	1	p2	(AG)7	14	652	665	ACTAGGTTGTTGTGGCGGAG
scaffold735092_15.[illegible]	1	p2	(AG)7	14	2309	2322	TCATATATGTTCCTGGCGGC
scaffold735150_16.[illegible]	1	p2	(AT)7	14	2192	2205	TCGATCGGGAGTCAGAAATC
scaffold735156_14.[illegible]	1	p2	(AT)6	12	246	257	GATCGGTTGGGAAAAAGTCA
scaffold735168_15.[illegible]	1	p2	(CT)7	14	105	118	ATGTGGCTTGCTTGCTTCTT
scaffold735178_18.[illegible]	1	p2	(CT)6	12	367	378	CTACATCCCATCATCACCCC
scaffold735190_13.[illegible]	1	p2	(AT)8	16	591	606	TTTGATTTTTAATGGGGCAAA
scaffold735196_14.[illegible]	1	p2	(CT)11	22	1937	1958	GCTCAATTCTTGGACCTTGC
scaffold735207_16.[illegible]	1	p2	(AC)6	12	1261	1272	GGACTCTGCAGCATGTTCAA
scaffold735212_17.[illegible]	1	p2	(AT)6	12	661	672	GCTCATGATTTGGCCAGTTT
scaffold735235_17.[illegible]	1	p2	(TC)7	14	1109	1122	AATTTGTGTTGGCTTCAGGG
scaffold735254_14.[illegible]	1	p2	(AG)8	16	1656	1671	GCGATCATTCTCGTGAAGAAA
scaffold735260_16.[illegible]	1	p2	(TC)8	16	432	447	GCTTGTACCCGTAGCGTGTT
scaffold735276_14.[illegible]	1	p2	(GA)6	12	1242	1253	AGTAGAGGGCGAGAACGACA
scaffold735290_15.[illegible]	1	p2	(TC)6	12	1222	1233	ATCTCCCACGCTCACACTCT
scaffold735292_15.[illegible]	1	p2	(TA)7	14	568	581	TTCCCACCATGACTTCAACA
scaffold735313_16.[illegible]	1	p2	(GA)9	18	384	401	CCCCCAAATCTACAAAAGGG
scaffold735314_16.[illegible]	1	p2	(TC)6	12	632	643	CAATCGAGCAATCTGTAAGGTG
scaffold735318_14.[illegible]	1	p2	(GA)6	12	961	972	ATTCGTAACGTAACACGGGC
scaffold735320_13.[illegible]	1	p2	(TA)9	18	1530	1547	ACCCACCCTTCTTTTTGCTT
scaffold735345_15.[illegible]	1	p2	(AT)6	12	259	270	CTTCAACGGAGGGGAAGATT
scaffold735377_15.[illegible]	1	p2	(CT)8	16	1294	1309	GTGTGGTCTGCAAAGTGTGG
scaffold735386_10.[illegible]	1	p2	(AT)6	12	352	363	AATCGCTGAACTGTTTTCCG
scaffold735395_15.[illegible]	1	p2	(TA)9	18	1234	1251	CACACCTACTTTAATGACGCACA
scaffold735398_17.[illegible]	1	p2	(TG)6	12	2186	2197	GGCCAACATGATTAGCATCC
scaffold735404_14.[illegible]	1	p2	(TG)6	12	1613	1624	CTTGTCAGCGCGTTTAATGA
scaffold735409_15.[illegible]	1	p2	(TC)6	12	667	678	GATGCTGGAAATTCTGGGAA
scaffold735420_16.[illegible]	1	p2	(AG)6	12	5753	5764	AAATTTAACGGGACACCACG

scaffold735428_15.(	1	p2	(AT)8	16	681	696	GAGACATCAGCTGAGGGGAG
scaffold735430_15.8	1	p2	(CT)6	12	667	678	GCTAAACTCCAGCGCAAAAC
scaffold735438_16.9	1	p2	(CT)6	12	113	124	CTCGCTCGGAAAAGCAATAC
scaffold735496_16.6	1	p2	(TC)9	18	2309	2326	GATTGTTGGGTTTTCCGATG
scaffold735529_17.3	1	p2	(CT)6	12	1368	1379	TCTTTGGGATTCCTCTCGAA
scaffold735531_15.2	1	p2	(GA)7	14	669	682	ACCTCGGACGCCTAAAGAAT
scaffold735543_17.7	1	p2	(TG)8	16	1303	1318	TCCTTCGAGACTCCATCGTT
scaffold735555_15.1	1	p2	(AT)6	12	626	637	TTTGAACTCCGTAGCAACCA
scaffold735600_17.6	1	p2	(AG)6	12	675	686	CCATGTCTTCCTCTTCGAGC
scaffold735624_16.(	1	p2	(TC)6	12	906	917	CTCACCTGCATCGTCTTTCA
scaffold735629_15.3	1	p2	(CT)10	20	668	687	CTGCTCTCCAGAAGCAAACC
scaffold735630_14.3	1	p2	(GA)9	18	1437	1454	CGGAGGCTGAGGCATTATAG
scaffold735638_16.1	1	p2	(AG)9	18	670	687	CGCAGGATTGTACGGTTGTA
scaffold735704_17.2	1	p2	(TA)9	18	44	61	TGAAGTTAGGAAATAAGGAGGTGTT
scaffold735717_16.3	1	p2	(AG)8	16	1340	1355	TGTTGGCATGGTTGATGAGT
scaffold735720_12.8	1	p2	(TC)6	12	680	691	GGATTCGAGTTTTTGAAACCA
scaffold735741_15.1	1	p2	(GA)6	12	132	143	TGGTTGAAAGATGAGGGAGAA
scaffold735748_11.5	1	p2	(TA)7	14	678	691	TCGATTAACTTGACAAGGTTTGAA
scaffold735749_15.1	1	p2	(AG)6	12	3685	3696	AGGCGTGTTTTGTTTGGAAG
scaffold735750_16.7	1	p2	(CT)7	14	129	142	GAGAGACGAGCTCACGCATA
scaffold735755_12.9	1	p2	(AT)10	20	348	367	TGGTTATCATTTTGTCACAGTTTTT
scaffold735763_14.7	1	p2	(AG)12	24	841	864	GGCCCACCATACCACAATAC
scaffold735767_14.8	1	p2	(CT)6	12	120	131	TAGCCTTGGCCCCTAAGTTT
scaffold735775_16.1	1	p2	(CT)6	12	78	89	AGCAACAGCGGAAGAAGAAG
scaffold735778_15.8	1	p2	(TA)6	12	2311	2322	CATGATACCTCACTGGGGCT
scaffold735781_16.(	1	p2	(CT)6	12	68	79	ATTTGTTCAAAATCCGCGAC
scaffold735794_13.9	1	p2	(AT)8	16	679	694	AAACTTTGGAATCGACATTGC
scaffold735796_16.4	1	p2	(AG)7	14	1791	1804	GTCGAGCAACAGCAGGTGTA
scaffold735801_18.7	1	p2	(AT)9	18	63	80	GGTTTACCCATAGCGCACTC
scaffold735814_16.2	1	p2	(AT)7	14	681	694	TTCTCCTCACAATGCTTCCA
scaffold735819_18.2	1	p2	(TG)6	12	183	194	GCTTTGTGATGGGTGAAGGT
scaffold735826_14.6	1	p2	(TC)6	12	858	869	TCCCAAGAATGAAGCCACTC
scaffold735830_13.9	1	p2	(GA)8	16	848	863	AAGCTCAGTAGCGTTCTCGC
scaffold735834_16.4	1	p2	(TC)6	12	857	868	CTTGATGCCCCGTTAAACAT
scaffold735835_15.3	1	p2	(GA)7	14	1042	1055	ATGATTCCTCCCCTTCCATT
scaffold735880_15.2	1	p2	(TA)6	12	558	569	CCGGTACATTCGGTAGATGG
scaffold735886_15.1	1	p2	(GT)8	16	755	770	TTGAGCATTGATACAGTGGAGA
scaffold735917_16.9	1	p2	(AT)6	12	1692	1703	TCTCGATTCAACTCATCGGA
scaffold735926_17.9	1	p2	(CT)7	14	5030	5043	CTCATTTCGGAAGGAGCAAC
scaffold735953_14.4	1	p2	(AC)7	14	2378	2391	TGTTAATGAAATTTACGCACACA
scaffold735955_16.4	1	p2	(CT)8	16	1791	1806	GTGTAACCGTGGATCAGGCT
scaffold735956_15.1	1	p2	(AG)6	12	221	232	GGGCTGTACTTCAGAGGCTG
scaffold736006_14.9	1	p2	(AG)12	24	1980	2003	CGGGAAGTGGAGAGAGAGTG
scaffold736018_15.9	1	p2	(AC)8	16	3000	3015	GAATGAGGGATCGGGTGATA
scaffold736064_14.7	1	p2	(TG)6	12	203	214	AGCGATTTTAACCTCCCGTT
scaffold736071_14.4	1	p2	(TC)6	12	529	540	CGGGGAGTAGATGAATCGAA
scaffold736072_21.5	1	p2	(AT)7	14	348	361	TGTGTGCGAATTTGGAAAAG
scaffold736080_15.1	1	p2	(GA)8	16	460	475	GGGGAAAGATTGCAAATTCA
scaffold736083_16.9	1	p2	(CT)7	14	3936	3949	CATCTTCACCGGCTTCTCTC
scaffold736117_15.4	1	p2	(CT)8	16	1458	1473	CATTTCAGCCACCATCTCCT
scaffold736122_14.7	1	p2	(AT)6	12	2246	2257	TTTCCGACCATCTAAGTCGC
scaffold736131_14.2	1	p2	(TA)6	12	698	709	GTGGCTTGTGTTTCCTTGGT
scaffold736139_16.2	1	p2	(TG)7	14	3153	3166	GAATTTTTGAGATCGACGCC
scaffold736154_18.(	1	p2	(TA)6	12	285	296	TTGATTCTCGCCATGTACAAA
scaffold736159_11.6	1	p2	(GA)6	12	699	710	ATGTGCAGCCCCTGAGTAAG
scaffold736177_16.8	1	p2	(AC)6	12	74	85	GGCGTGCATGTTTTATCTGA
scaffold736179_14.(	1	p2	(AG)13	26	347	372	CCATGTGTCGTCAAGATGCT
scaffold736180_16.3	1	p2	(TG)6	12	564	575	GATTTTGGCAATTCAACGGT

scaffold736182_16.5	1	p2	(CT)6	12	4509	4520	CGCTTCGCCATTGTAATTTT
scaffold736200_17.4	1	p2	(CA)8	16	528	543	ATGCCAACCCACGCTAATAA
scaffold736209_21.6	1	p2	(AT)6	12	703	714	TGAGAGCGAGCAAGGGTTAT
scaffold736215_13.0	1	p2	(TG)6	12	684	695	TGATTAAAAACTGAACCGGACA
scaffold736223_16.0	1	p2	(CT)6	12	1491	1502	AAATTGGTGGGAATGCAGAG
scaffold736243_16.8	1	p2	(AG)6	12	921	932	CACCGCCTCGTATCTTCATT
scaffold736251_14.9	1	p2	(AC)8	16	700	715	GGGCTTGGTCAGAAAATTGA
scaffold736262_15.0	1	p2	(TA)9	18	2535	2552	GGCCTGATCTTACCTAGGGG
scaffold736264_15.8	1	p2	(AT)8	16	701	716	CGTGATTCATGCCATATTTGA
scaffold736278_16.3	1	p2	(AG)7	14	3352	3365	GGATCCCGGTCATATACGTG
scaffold736310_14.9	1	p2	(TC)7	14	704	717	GCTCCTCCTATCCACATCCA
scaffold736320_14.8	1	p2	(TA)8	16	1202	1217	TTGGATGCGTTGATTGAAAG
scaffold736342_13.4	1	p2	(CT)7	14	432	445	TTTCACCCAAATCACGAACA
scaffold736345_14.0	1	p2	(AG)14	28	1260	1287	AACCCTGATCACCAAACGAG
scaffold736350_16.4	1	p2	(AT)8	16	2785	2800	TCGGTTGGTGAAGTTGTTGA
scaffold736375_15.0	1	p2	(TA)7	14	683	696	ACACGGATCGGAAATCCATA
scaffold736414_16.6	1	p2	(AT)6	12	710	721	TCCAACGGAAATCCTTATACTCA
scaffold736426_15.9	1	p2	(CT)6	12	844	855	AACCCACTTCCACAGAATGC
scaffold736503_15.0	1	p2	(GT)7	14	714	727	TGCACATGCAAAGCTAAAGG
scaffold736522_17.4	1	p2	(TC)6	12	1323	1334	CGATCACTGTAAGAACCGCA
scaffold736538_15.2	1	p2	(CT)6	12	340	351	CACCCATGTTTGTTTTTCCC
scaffold736558_17.2	1	p2	(CA)9	18	1111	1128	TCGTTTTGTCGAACTGTTGC
scaffold736606_16.2	1	p2	(AG)6	12	717	728	CGAGCAGTTTGAATTGGTGA
scaffold736650_16.3	1	p2	(AG)6	12	644	655	GGCTTATTTCCATCCCGAAT
scaffold736653_14.3	1	p2	(AC)7	14	718	731	GTGTTGGTGCTTGATGGCTA
scaffold736682_19.9	1	p2	(AC)7	14	723	736	TGCCATTTGATGGAGAATCA
scaffold736701_10.6	1	p2	(AT)10	20	344	363	CGAATCCAGGTAAGGCTTGA
scaffold736712_15.5	1	p2	(GA)7	14	723	736	GGGCAGAAAAGGTGAAACAA
scaffold736713_14.3	1	p2	(GA)6	12	4841	4852	TCTCTTTCCCCCTCCTTTTG
scaffold736724_15.4	1	p2	(CT)10	20	71	90	CTCCCCACTTTCCTTCAACA
scaffold736726_15.0	1	p2	(TC)11	22	1256	1277	GCCATTGCACTTATGCTCCT
scaffold736750_14.0	1	p2	(AG)7	14	518	531	TAGTTTTCACCATTGCGACG
scaffold736774_15.6	1	p2	(AT)8	16	887	902	CGTTTTCCACCTTCAAGACA
scaffold736787_16.0	1	p2	(AT)6	12	250	261	AACTCAATGGGGACATCAGG
scaffold736819_14.7	1	p2	(TG)11	22	1558	1579	TGCAACTGCATGGGATAGTC
scaffold736826_16.0	1	p2	(TC)6	12	2415	2426	GGAGGAACTCCATCTCCCTC
scaffold736850_15.5	1	p2	(AT)6	12	3308	3319	GAGACAAAAAGCATTTGCCC
scaffold736876_14.2	1	p2	(TC)6	12	151	162	AATGGAGATCCGTGCTTCAC
scaffold736882_14.7	1	p2	(CT)7	14	733	746	GGTGAAAGGCCATGTGAACT
scaffold736884_15.0	1	p2	(TC)6	12	1211	1222	TACACCAAATCTTGGCTCCC
scaffold736888_14.0	1	p2	(AT)7	14	1433	1446	TTTCGACCAATGAGAAAGGC
scaffold736900_14.0	1	p2	(AC)6	12	218	229	TGGTCTGCCTAATTTACTGCAA
scaffold736907_15.1	1	p2	(AG)6	12	1645	1656	AGAGACTGTTCAAGGGCAGC
scaffold736936_15.2	1	p2	(TA)13	26	763	788	TTGTCTTGACGCTGACAACC
scaffold736945_14.9	1	p2	(GA)7	14	4078	4091	CGAAGAAAAGCAGGTTTTGG
scaffold736970_15.0	1	p2	(GT)6	12	1265	1276	CCAAGCACATCAGCTGAAAA
scaffold736996_16.6	1	p2	(CT)6	12	960	971	TTCCTGCTTTTCATCGCTCT
scaffold737011_14.6	1	p2	(AT)13	26	1843	1868	GAGAGGAGACGCACAGAAGG
scaffold737021_15.7	1	p2	(GA)11	22	2325	2346	TGCACTAAGTGGTGCTTTCG
scaffold737037_14.0	1	p2	(CT)8	16	4765	4780	CACGTGGGACTACTGACCCT
scaffold737056_15.0	1	p2	(AC)6	12	347	358	TTTTAAGTCGTGTTGCGTGC
scaffold737064_15.9	1	p2	(AG)9	18	1124	1141	TGTCAGCAGACATAGCAGGG
scaffold737100_15.1	1	p2	(AT)8	16	447	462	CCAGTCGAACCGACAAGATT
scaffold737106_14.0	1	p2	(CT)6	12	117	128	TCGAACCCAAACAAATTTCC
scaffold737118_14.2	1	p2	(TC)6	12	1282	1293	GGCTGTTGTAAAACCCTTGC
scaffold737130_16.0	1	p2	(AG)8	16	38	53	AAAGCAATCAGTCAAAGGGG
scaffold737138_18.0	1	p2	(CA)7	14	747	760	GAGAGCAAGCAACAATCCAA
scaffold737185_16.4	1	p2	(TA)6	12	1518	1529	GAGTGAGTTGGCGAGAATCTG

scaffold737211_16.8	1	p2	(AT)6	12	1335	1346	CTATATAGGTCCCCCGTCCC
scaffold737252_15.7	1	p2	(TA)8	16	2012	2027	CCTGCGTGGTTTTTCTTTTG
scaffold737275_14.5	1	p2	(TC)7	14	5085	5098	TATATCGTCCAGCTCCGACC
scaffold737280_15.4	1	p2	(TA)6	12	516	527	GGAGGTTGAGATTTTGGAGGA
scaffold737296_17.4	1	p2	(GA)7	14	3113	3126	TGAGAGGAAAATGGTTTGGG
scaffold737300_14.6	1	p2	(CA)9	18	753	770	GGTAGACATGCAAACCGTGA
scaffold737312_17.0	1	p2	(TC)10	20	4403	4422	TATGACAAGCAGCAGTGCCT
scaffold737326_14.4	1	p2	(TA)9	18	40	57	CGGATTTAAGGGTTATTACCTGATT
scaffold737327_15.2	1	p2	(AT)10	20	1491	1510	TTTGGCTTGAACAGCACTTG
scaffold737330_15.6	1	p2	(AT)8	16	757	772	TTTTGGAGCCGAGAAGAGAA
scaffold737338_13.4	1	p2	(AG)6	12	128	139	TTGATTAGAGGAAACTGCGGA
scaffold737345_14.6	1	p2	(AT)6	12	1499	1510	GAATGTGGGCGATCTTGACT
scaffold737369_17.5	1	p2	(CA)6	12	841	852	GCTGCATATAAACCCGGAAA
scaffold737392_18.6	1	p2	(AT)10	20	756	775	CACTTTCCATCCAAAATGCAC
scaffold737393_13.8	1	p2	(AC)10	20	1844	1863	GCACAAATTTTCAATTCACGTT
scaffold737452_15.2	1	p2	(TA)10	20	2132	2151	TGAGAGGATGATGGTGTTGC
scaffold737479_17.0	1	p2	(AT)6	12	597	608	CCATTTAATTTCCAAAGGGTT
scaffold737494_13.6	1	p2	(TC)8	16	562	577	GCTTACGTGTTTCAGGGCTC
scaffold737504_15.7	1	p2	(CT)7	14	121	134	CCTTGCATATGATGGTTAGCA
scaffold737506_14.0	1	p2	(TA)10	20	1466	1485	GCGCAAGAGCTTTTTCTGAG
scaffold737509_19.4	1	p2	(GA)9	18	765	782	CACATTCTTTAAGCCATGGGA
scaffold737526_15.1	1	p2	(TA)6	12	753	764	GATCCACGTTAGAACCCCCT
scaffold737531_18.1	1	p2	(TC)6	12	1260	1271	TCCGATCTCTGTTTCGATTTG
scaffold737532_15.6	1	p2	(AT)6	12	1495	1506	TGCAAATGGTACTAGGGACG
scaffold737543_14.6	1	p2	(AG)6	12	177	188	CATTTTGAGCCATTGGACCT
scaffold737550_15.7	1	p2	(AG)8	16	1976	1991	CTGAATGCAATCAATGGGTG
scaffold737556_15.1	1	p2	(TA)10	20	895	914	TGTAAAGCCCATTTGAACCC
scaffold737575_15.6	1	p2	(TA)11	22	1303	1324	CGAGTGCCTCAAGTATGCAA
scaffold737576_17.8	1	p2	(TG)6	12	697	708	CCTCAATTTCTGCAGCCTTC
scaffold737583_14.0	1	p2	(AG)6	12	1256	1267	TGGATGGGTTACTGGATCAAT
scaffold737585_16.5	1	p2	(TA)7	14	298	311	ATATTTTATGGGACGGGGGA
scaffold737600_15.6	1	p2	(CT)7	14	4885	4898	GTGCGTTTTCGTCCAGAAAT
scaffold737636_14.2	1	p2	(CT)10	20	2027	2046	ATTCGATGAATGTGCTGCAA
scaffold737650_17.4	1	p2	(CT)8	16	1527	1542	TTAGAGGTGTCCAACAGGGG
scaffold737678_15.4	1	p2	(TC)8	16	1614	1629	CCACGCGCTCCTTAACTTAC
scaffold737685_14.8	1	p2	(GA)8	16	777	792	TTGATGGCATCGATGAGGTA
scaffold737691_13.3	1	p2	(TC)6	12	1465	1476	AGGCACGGCCAAGATAATAA
scaffold737699_15.3	1	p2	(TC)7	14	1736	1749	TCTCCGAATTCCGAAAAGAA
scaffold737713_14.3	1	p2	(AT)10	20	743	762	GTAGGTCGGGGTAGGGTTGT
scaffold737716_10.8	1	p2	(TC)6	12	1119	1130	AATTCATCACCACCTCACCC
scaffold737727_16.7	1	p2	(TA)8	16	203	218	CGTCCTCTCCCTTTCCTTTC
scaffold737729_15.3	1	p2	(TA)6	12	405	416	GACACTCACGATTTACCGCA
scaffold737734_15.0	1	p2	(TG)6	12	3259	3270	TTCACGTCATGGTCGTTTGT
scaffold737753_14.8	1	p2	(CT)9	18	222	239	TGGCAGAGTGGACTTCTCAA
scaffold737773_14.4	1	p2	(TA)6	12	4558	4569	TTGTATTTGATCCGCAGCCT
scaffold737780_16.6	1	p2	(AG)8	16	562	577	TAGGTTTGGATACCCGTTCG
scaffold737787_15.0	1	p2	(CT)6	12	1204	1215	GGAAGAAATTAAAAACCCCCA
scaffold737807_15.9	1	p2	(TC)8	16	194	209	TATGGATTCTGGGATGGGAA
scaffold737808_14.9	1	p2	(AT)11	22	779	800	AATCCTCAAAGCAATAACGC
scaffold737811_15.6	1	p2	(AC)7	14	786	799	TGTTTCATAATTTGGGCATGA
scaffold737827_15.6	1	p2	(AT)6	12	790	801	TCAGGCTTACAGCCATCCTC
scaffold737833_15.3	1	p2	(GA)6	12	480	491	TCATCCATTCCCAGTTCTCC
scaffold737852_16.3	1	p2	(TA)6	12	1968	1979	CTCGCAGTAGGTAAAAGGCG
scaffold737854_14.9	1	p2	(TA)14	28	1876	1903	CACAAAAGGATGGGAATTGG
scaffold737862_15.9	1	p2	(CA)7	14	790	803	ACATTTTCAACGGCACGATT
scaffold737870_13.6	1	p2	(CT)6	12	2083	2094	AGAAAAAGGAACGCCTCCAT
scaffold737877_15.8	1	p2	(TA)8	16	2389	2404	CCTTGACAGATGCAAAGACG
scaffold737885_15.3	1	p2	(CT)6	12	793	804	ACCATTGATAGCATGCGTGA

scaffold737907_17.(	1	p2	(CA)6	12	1844	1855	AGGTGGGATTGTTTGCTTTG
scaffold737924_16.(	1	p2	(AG)13	26	32	57	TTGCTAATCTTCGATGTATCAACC
scaffold737926_16.:	1	p2	(TG)7	14	270	283	TACTGTCCAGCCTTGCATTG
scaffold737951_14.:	1	p2	(AG)12	24	25	48	TGACAGATGCACTGATCCAAA
scaffold737956_16.7	1	p2	(AT)6	12	1536	1547	TGCATGTGTGCATGTTTGTT
scaffold737977_17.(	1	p2	(TC)8	16	2089	2104	GGATGCTATGATATGGTGATGC
scaffold737988_15.:	1	p2	(TG)6	12	1797	1808	CGTCAAATTCTGGTTTATCATTCC
scaffold737993_15.(	1	p2	(AC)9	18	1238	1255	CCGCAGCCTGAGTTGATTTA
scaffold737995_15.7	1	p2	(GA)6	12	1996	2007	TGTGATGGAACGAGGATTTG
scaffold738001_16.(	1	p2	(AC)7	14	3296	3309	TGAAGATACGAATCCTGGGC
scaffold738016_14.(	1	p2	(CA)6	12	1379	1390	CGCATGAACCATGAAATCAG
scaffold738017_17.(	1	p2	(CT)8	16	962	977	AAACGTCATTTCCTGCTGCT
scaffold738057_17.:	1	p2	(AT)9	18	799	816	AATATGGGGTTTTGCCATCA
scaffold738072_15.:	1	p2	(CT)6	12	180	191	TTCTGCCCATTCTTCCACTC
scaffold738080_17.!	1	p2	(AT)7	14	611	624	TTTTATAGGACTGGGCCACG
scaffold738092_17.:	1	p2	(GA)7	14	416	429	TGAGAGAGAGAAAGATAATCCTAAA(
scaffold738095_16.:	1	p2	(TA)6	12	1178	1189	ACACCGAAATCGGAGTTAGC
scaffold738113_16.(	1	p2	(AC)8	16	312	327	GCATAGGGAGATTTGCGGTA
scaffold738129_16.!	1	p2	(TG)10	20	82	101	TTCGGTGTATTTAGCCCCTT
scaffold738148_13.4	1	p2	(TA)10	20	753	772	TCGGTCAATCCACAACTTCA
scaffold738164_15.!	1	p2	(TA)8	16	4419	4434	TTTGGCCGGACTAGTTATGG
scaffold738172_13.!	1	p2	(CA)10	20	934	953	TCTATTCTCTTTGCCACGCC
scaffold738186_15.(	1	p2	(GA)8	16	5354	5369	GATTGGAAAAGGGTGGGTTT
scaffold738204_17.!	1	p2	(AT)6	12	361	372	ACCCACAATCAACTTGCACA
scaffold738227_14.(	1	p2	(GA)7	14	42	55	AGAGATCACTGATTATCTTTTCAATTT
scaffold738260_17.:	1	p2	(TC)6	12	2145	2156	GGCTATTGTGGCATGGAGAT
scaffold738262_15.(	1	p2	(CT)7	14	2125	2138	GATAAAGGTCGGCGTCAAAG
scaffold738329_16.(	1	p2	(AT)6	12	1471	1482	TCGAATTGACATCTTCGTGC
scaffold738385_15.(	1	p2	(AT)6	12	1664	1675	TTTGTGCCAAACCACTCTTG
scaffold738426_15.!	1	p2	(CT)8	16	2033	2048	TTCTCATGCCCCTTCAAATC
scaffold738454_19.:	1	p2	(TA)6	12	115	126	ACATGGTGGTGGGTTCAGAT
scaffold738456_13.!	1	p2	(AT)7	14	787	800	GTCTCGATTGAATTTTCGCC
scaffold738461_14.(	1	p2	(TC)6	12	1887	1898	CAATGAGACCATGTTGGTGC
scaffold738492_15.(	1	p2	(CT)6	12	2326	2337	AACATTGGCTGGATTTCAGG
scaffold738494_16.:	1	p2	(GA)6	12	5115	5126	GCACCTTCACCAACCATCTT
scaffold738495_13.7	1	p2	(TC)10	20	2260	2279	CATCAGCCACCATTTTCCTT
scaffold738499_15.!	1	p2	(CT)7	14	814	827	TCCCCAGTCAATTTCACACA
scaffold738504_19.(	1	p2	(AC)7	14	484	497	CAGCGTTTTTGAGTCCATCA
scaffold738511_14.(	1	p2	(TA)6	12	833	844	CGGTGGTGGAAAAGATGACT
scaffold738516_16.7	1	p2	(TG)6	12	428	439	TGCATTGAAATTGACAGGGA
scaffold738525_12.:	1	p2	(TC)6	12	70	81	TTTCCTAGAAATGCTCTCTTTCTGA
scaffold738555_14.7	1	p2	(TC)8	16	324	339	TGCTAACATACGCATGAGGC
scaffold738572_14.7	1	p2	(CT)12	24	176	199	ACCTGCTTGATTTTGTTGGA
scaffold738579_15.8	1	p2	(GA)8	16	834	849	CGACTTGCGCTAGAATCGTT
scaffold738591_15.4	1	p2	(AT)9	18	2033	2050	CCCCCATGATTTTCTTTCCT
scaffold738595_17.!	1	p2	(TG)6	12	512	523	AAGTCCTGGACCTATGTTTGGA
scaffold738596_15.(	1	p2	(CA)6	12	1921	1932	CCGGCTCAAGTCATTTTCTC
scaffold738613_17.7	1	p2	(CT)6	12	337	348	CCTTCACCACCTCTTGCATT
scaffold738618_16.8	1	p2	(TG)6	12	4968	4979	CATCCTACGCATTAAACCCA
scaffold738621_16.4	1	p2	(CT)8	16	4122	4137	GCCCCTGGTTTTGGTCTATT
scaffold738634_14.(	1	p2	(AC)7	14	410	423	CATCTGCTGCTTTGTTTGGA
scaffold738639_15.:	1	p2	(GA)6	12	121	132	AATCGCTTCCGCATTTAAGA
scaffold738652_14.4	1	p2	(CT)6	12	1927	1938	ACCTGAGGGAGTTGATGTGG
scaffold738656_13.4	1	p2	(AG)6	12	1610	1621	TGACAGCAAGCCAGATTGTC
scaffold738658_16.!	1	p2	(TC)6	12	621	632	TATTTGAAGGGAGTGCCCAG
scaffold738679_16.8	1	p2	(GA)7	14	846	859	CCTGCAGATGACTGGCTACA
scaffold738694_14.:	1	p2	(AC)6	12	2967	2978	TCATTTCAAGCAAAATCGCA
scaffold738708_15.8	1	p2	(TG)6	12	914	925	TTGGCAATTTATGGTCACCT

scaffold738736_19.	1	p2	(AC)9	18	846	863	GCTCAGGCTTACATGGGTGT
scaffold738743_13.	1	p2	(AT)9	18	5646	5663	AGCTACACCTGAGAGTGCCC
scaffold738761_16.	1	p2	(CG)6	12	93	104	CTTATGGTGGTATGGTGTAGAGG
scaffold738763_16.	1	p2	(TA)9	18	199	216	GCGGGTCAAGTTGAGGAATA
scaffold738768_19.	1	p2	(CT)7	14	463	476	TCCCAGAATTTGCCTTCAAT
scaffold738769_15.	1	p2	(TA)6	12	3283	3294	TGATTTTTGGAACCGTAGGC
scaffold738792_16.	1	p2	(AC)6	12	1401	1412	TGGTGGTGAATAGGGGACAT
scaffold738810_14.	1	p2	(GA)6	12	1284	1295	TGGTATGAGTTGGCTTTTTGG
scaffold738811_17.	1	p2	(GA)6	12	240	251	GGCAGGTCTCAGCCAAATTA
scaffold738820_17.	1	p2	(CT)6	12	804	815	CGAAACGAGAATAACCGGAA
scaffold738823_16.	1	p2	(CA)6	12	1141	1152	GGTGGAAAACATGCACACAG
scaffold738845_15.	1	p2	(GA)6	12	7006	7017	GGAACCACAACCCATCACAT
scaffold738846_13.	1	p2	(AT)10	20	197	216	TAACGATGGACTAGGGGCTG
scaffold738861_17.	1	p2	(TC)7	14	10508	10521	AACGAAATAAACCCCCAACC
scaffold738872_12.	1	p2	(TC)6	12	185	196	CCAAGTCCCTTCGTTTTCAA
scaffold738886_16.	1	p2	(AT)6	12	923	934	TTGCTTTGGTCATACAAACATT
scaffold738895_14.	1	p2	(TA)7	14	864	877	TTGGGTATTGTTAGCTCGGG
scaffold738899_16.	1	p2	(CA)6	12	1813	1824	TGGAACCTATTTCCTCCACG
scaffold738915_14.	1	p2	(GA)6	12	171	182	TCACTGGGGACAATCTCACA
scaffold738920_15.	1	p2	(AG)10	20	3020	3039	GCTTTGTGCCTGCATTATGA
scaffold738955_17.	1	p2	(AT)6	12	615	626	GGGACGTGGTTAGCAGATGT
scaffold738956_14.	1	p2	(CT)6	12	2247	2258	ACAGCTGGCCTTGGATGATA
scaffold738970_14.	1	p2	(AG)9	18	2899	2916	CAATGGAGCAGAAAGAGGGA
scaffold738973_13.	1	p2	(AT)8	16	683	698	AGGTCGGGTTTTCAGCCTTA
scaffold738978_15.	1	p2	(AG)7	14	928	941	AGTCAGTGCTCACCGGATCT
scaffold738979_15.	1	p2	(TG)7	14	1227	1240	TCCATTTATCGGAGTTACGGTT
scaffold738980_12.	1	p2	(TG)11	22	1812	1833	GGTTTATTGGTGCTGGTGCT
scaffold738987_13.	1	p2	(AT)9	18	300	317	TTTCTTGTTGAGGAATCGGG
scaffold738995_15.	1	p2	(TC)6	12	4019	4030	AATAAGCCTCCACACCCCTC
scaffold739003_14.	1	p2	(AT)6	12	164	175	TGAACCAAACACCCCCTAAG
scaffold739020_12.	1	p2	(AT)11	22	361	382	GCCATGTGGAGGTTGATTTT
scaffold739037_15.	1	p2	(GA)6	12	675	686	TTGGTGAAGATTCGATTCCC
scaffold739043_16.	1	p2	(CT)6	12	176	187	TTTACTCCGCCACGGTTAAG
scaffold739048_17.	1	p2	(CA)6	12	3540	3551	CAAATCGTCAAATCCCCCTA
scaffold739066_15.	1	p2	(AT)11	22	872	893	GAAAATGTCGTGAATGCCAA
scaffold739073_14.	1	p2	(TC)8	16	1087	1102	TGTGTTGATGCAAGTTTGGAC
scaffold739077_15.	1	p2	(TG)11	22	2600	2621	CGATTTACGCCGAGTTAGGT
scaffold739086_15.	1	p2	(TC)8	16	103	118	AAGCCTCCCAAGTCTCCAAT
scaffold739089_17.	1	p2	(AG)6	12	90	101	AGCCCCGAAAAAGTAATGGT
scaffold739092_14.	1	p2	(TA)8	16	2386	2401	TTGCGCAAATAAACCAACAA
scaffold739095_17.	1	p2	(GA)6	12	200	211	TTGGGGTGGAGAGAAGTGAG
scaffold739106_16.	1	p2	(CT)7	14	2441	2454	AGTGGTGTTGGACTTGGGTC
scaffold739142_13.	1	p2	(AT)6	12	33	44	TCAGTCTGCACAGCTGACAA
scaffold739157_14.	1	p2	(AG)7	14	880	893	ACGATGGCTTTGCTGTTCTT
scaffold739167_18.	1	p2	(TC)6	12	1703	1714	GGCGTGCCTAATGGTATTTC
scaffold739182_17.	1	p2	(TC)10	20	270	289	TGAAGCCCATGTTGAATCTG
scaffold739195_15.	1	p2	(TC)6	12	2029	2040	TTTCTGGGGAGTTCATGTCC
scaffold739201_19.	1	p2	(TA)7	14	67	80	GGGGTTATCATGGGATCAAG
scaffold739205_15.	1	p2	(AG)11	22	135	156	GGCCAGGAGATGAGTCAGAA
scaffold739208_17.	1	p2	(TA)8	16	169	184	CCAAGATCAATGGTTCCTAAGTG
scaffold739213_15.	1	p2	(CT)6	12	188	199	GAATGGCTTCGAGCTTACCA
scaffold739237_16.	1	p2	(GA)8	16	4349	4364	AGATGAGCCGAGGAGCAATA
scaffold739254_16.	1	p2	(TC)7	14	1768	1781	TCCAATTCCGATACCTGACC
scaffold739261_18.	1	p2	(AT)8	16	482	497	GGCTGCAAACATTTGAGGAT
scaffold739270_15.	1	p2	(CT)10	20	364	383	TTTATGAAAAATGGCCCCAA
scaffold739320_16.	1	p2	(TA)9	18	897	914	GTTCCGATACGTCTGAAGGC
scaffold739321_22.	1	p2	(TA)6	12	166	177	GGTAGGAACTGCATGACCAAA
scaffold739323_12.	1	p2	(TA)13	26	78	103	CAGGATAATCCAGAATCAACACA

scaffold739345_17.	1	p2	(GA)7	14	2746	2759	GGCCATGACCTCTTCCTGTA
scaffold739346_15.	1	p2	(CT)9	18	1846	1863	TCAACATTCAGCCGATCAAA
scaffold739349_16.	1	p2	(AG)8	16	5503	5518	TTTCTTCCACGCCTTTCAAT
scaffold739352_14.	1	p2	(AT)9	18	1964	1981	GCAGGACAAAGCAGATAGGG
scaffold739374_13.	1	p2	(AG)7	14	907	920	AAGAGGGCAGAAAGAGGAGG
scaffold739380_14.	1	p2	(GA)6	12	1437	1448	TGACGCTCGCAGTATTTTTG
scaffold739386_16.	1	p2	(AT)8	16	120	135	TTGAGTTGTGATGTTTGCATTTT
scaffold739391_18.	1	p2	(AT)9	18	2487	2504	GCTAGAGCCAAGGACATGAAA
scaffold739398_15.	1	p2	(AT)7	14	3597	3610	TATGAGATCTCGCCATGACA
scaffold739406_15.	1	p2	(AG)7	14	2426	2439	AAAATCGCCCCTCAAAACTT
scaffold739410_14.	1	p2	(GA)10	20	943	962	AAATATGAGTTTCCTCTTCCATGA
scaffold739440_14.	1	p2	(CT)8	16	743	758	TCAATCAAATCAAGGGGAGC
scaffold739447_15.	1	p2	(CT)6	12	190	201	GCCATCTATCTTGTTCGTGACA
scaffold739449_14.	1	p2	(TC)10	20	2325	2344	TAATGCCCACATGAGGAATG
scaffold739470_15.	1	p2	(CT)11	22	906	927	TATCCGAGTTTTGCCAGGTC
scaffold739480_14.	1	p2	(GA)6	12	2792	2803	AGCTTTTTCCGATGATCCCT
scaffold739491_14.	1	p2	(TC)6	12	36	47	TCCAGTCTTCTGCCAATCAA
scaffold739505_15.	1	p2	(GA)7	14	654	667	TGCTTTCGTTCAGGACTGTG
scaffold739518_15.	1	p2	(AG)7	14	2630	2643	CCCCAGCAATCCTCTCTACA
scaffold739531_16.	1	p2	(TA)6	12	1733	1744	TCAAAGCAAAATCGGCTAAA
scaffold739533_16.	1	p2	(TG)6	12	300	311	GGTCAGAATCACGGCAGTTT
scaffold739535_15.	1	p2	(AT)7	14	2176	2189	GTTTGATGACGTGGTTCGTG
scaffold739564_15.	1	p2	(TG)6	12	3104	3115	TGTACGCGTTATTTGCCAAC
scaffold739569_16.	1	p2	(CT)6	12	1099	1110	TACCAGCAACCATGTGGAAG
scaffold739572_15.	1	p2	(TC)8	16	2173	2188	GAGAGAAGGGGTGGGGATAG
scaffold739573_14.	1	p2	(GA)7	14	292	305	CTGTCTCCAGTGTCTTGCCA
scaffold739602_16.	1	p2	(AG)11	22	1785	1806	GCGTCCTCATTTATGATCGG
scaffold739627_16.	1	p2	(AG)6	12	931	942	CCATACGTTGGTAATGCAACC
scaffold739645_15.	1	p2	(CA)6	12	410	421	TGCAGGCAGTATTTCGACTG
scaffold739647_15.	1	p2	(AG)7	14	1509	1522	CCACTGCATCACAACCATTC
scaffold739654_14.	1	p2	(GA)8	16	834	849	CAAGGTCGTACTAGCCAGCC
scaffold739670_14.	1	p2	(TC)7	14	757	770	ATGTGGGGTTGAGATCTTCG
scaffold739682_15.	1	p2	(CA)7	14	910	923	TGAGTCGTTTTCATGATGGC
scaffold739695_13.	1	p2	(AT)6	12	121	132	TCTCCTCTCCTCAAAAGCCA
scaffold739705_14.	1	p2	(TA)8	16	50	65	AAATATGTGCTCTCATTTGATTACACT
scaffold739718_17.	1	p2	(TC)8	16	2078	2093	TCATGCAATACGGCTAGCTT
scaffold739750_15.	1	p2	(CT)11	22	618	639	TGATGATTTTTGGCCTTTGTC
scaffold739795_16.	1	p2	(GA)7	14	3211	3224	ACAGATTGCATTCTCCCGTT
scaffold739807_18.	1	p2	(AG)11	22	1929	1950	TTACAATTACGAACGCGTGG
scaffold739812_15.	1	p2	(AT)8	16	1802	1817	CATTCAGTTCATCATTTTCATGG
scaffold739826_14.	1	p2	(TA)10	20	292	311	TTGACCGTCCAACAATTCAA
scaffold739833_16.	1	p2	(GA)14	28	565	592	TGAAACTGAAGCTTGCATGG
scaffold739834_16.	1	p2	(CT)6	12	1475	1486	CCACGACCAATTCTTACAGC
scaffold739841_15.	1	p2	(CT)8	16	357	372	ATTCTCTCCAGCCCCTTCAT
scaffold739842_16.	1	p2	(CT)10	20	398	417	CCGGTTTATGGATGAGGAGA
scaffold739868_15.	1	p2	(CT)7	14	455	468	TAATATCCAAAACGCGCACA
scaffold739879_15.	1	p2	(TA)6	12	1505	1516	TCATCATGTGATGCAGACCA
scaffold739880_13.	1	p2	(TA)9	18	2247	2264	TGCCTGTATGAAAAAGCATCA
scaffold739934_15.	1	p2	(AT)9	18	437	454	TGAATGGAACACACACACCA
scaffold739939_14.	1	p2	(TA)6	12	2102	2113	TTCGGTAGGCCTGAATTGAC
scaffold739946_17.	1	p2	(CT)13	26	954	979	AGCTGCTTCGAGATGAGAGC
scaffold739958_17.	1	p2	(TA)8	16	96	111	AGGCATTCGAAAAGCTCATT
scaffold739970_15.	1	p2	(TC)6	12	3875	3886	TCCTCTGGTTTCTGTTTGCC
scaffold739996_15.	1	p2	(AT)7	14	126	139	GGCTAATTGTTGGCCTTTGA
scaffold740004_15.	1	p2	(GT)6	12	2056	2067	GGGAATGATGGAATGGTGAG
scaffold740012_14.	1	p2	(TA)6	12	441	452	TTGCAAAACAAAGCACGATT
scaffold740016_15.	1	p2	(GT)10	20	617	636	AGTACAAGGTGGCGGTTGAC
scaffold740017_17.	1	p2	(AT)6	12	399	410	AAAAACAAGTCATCCATGTCCC

scaffold740023_17.5	1	p2	(AG)6	12	1189	1200	CTCTCTCCCCCTTTCTTGCT
scaffold740028_15.0	1	p2	(AT)9	18	1913	1930	TTACCTGCGTGTTTTACAACTG
scaffold740029_18.1	1	p2	(GA)7	14	293	306	GCCACAGTGGAAGAACCAAT
scaffold740032_13.7	1	p2	(AT)9	18	59	76	GGCCATTGGGCTTATCCTA
scaffold740059_14.4	1	p2	(GA)6	12	1186	1197	TCAGTCGTCAGTAAACCCGA
scaffold740062_17.9	1	p2	(AG)6	12	316	327	GCCTCGCATGCTATTGCTA
scaffold740072_15.7	1	p2	(AT)6	12	115	126	GCTTGAGTGTGGCACATTTAGT
scaffold740077_16.4	1	p2	(AG)8	16	2818	2833	CTGTATCAGATGTCCGGCCT
scaffold740089_16.7	1	p2	(TG)6	12	977	988	TGTCCTGAGAGGCAGGAAAT
scaffold740116_17.0	1	p2	(TC)6	12	991	1002	TGGAATCGTTTGTCCACTTTT
scaffold740121_17.4	1	p2	(AG)7	14	3023	3036	CTTTGCTGTTCGTGGTTCAA
scaffold740124_16.7	1	p2	(AC)7	14	988	1001	CCATAGAAAACGGCCAGAAA
scaffold740151_17.2	1	p2	(AG)8	16	2037	2052	AATTTTTAGCCATGGTCCCAC
scaffold740158_13.6	1	p2	(CT)6	12	815	826	CTTCGTCTTCTCCAACTCGC
scaffold740176_20.9	1	p2	(CA)7	14	302	315	TTCACTGAATTTGCCTGAAAAA
scaffold740180_15.4	1	p2	(TC)8	16	336	351	ACCACTAATCCAACCCCCTC
scaffold740192_13.6	1	p2	(AG)7	14	1023	1036	GGGAGTTCTTGTGCATGGTT
scaffold740200_16.0	1	p2	(CT)6	12	1892	1903	TCGCAAAAGTGATCTTCGTG
scaffold740202_13.9	1	p2	(TC)7	14	714	727	ATGCGTGGGCGATATTATGT
scaffold740209_16.1	1	p2	(TG)9	18	4240	4257	TCAACACCCATCAAGATGCT
scaffold740212_15.3	1	p2	(GA)6	12	277	288	CTACCTCAGCCTCGCCATAG
scaffold740216_15.3	1	p2	(GA)6	12	1087	1098	GACCCAAACGGAGCATCTAA
scaffold740219_13.6	1	p2	(AT)6	12	1252	1263	CCAGAAGCAGTGAAGCACAA
scaffold740223_15.5	1	p2	(AT)7	14	5027	5040	TTTTCCACAGAAATGTTGGCT
scaffold740230_17.4	1	p2	(CT)8	16	4219	4234	TCGTCTTCATGCTCACTTCG
scaffold740237_16.6	1	p2	(AT)8	16	1972	1987	ATCAATGATGAGCCATGCAA
scaffold740242_16.7	1	p2	(AT)8	16	2378	2393	CTGTCACGGTCAGCACTTGT
scaffold740265_16.2	1	p2	(GT)8	16	1006	1021	CGGGGGAATAATAAGGGAAG
scaffold740281_17.6	1	p2	(TG)9	18	3386	3403	TGGGTTTAGGGATTAGCTGC
scaffold740284_17.5	1	p2	(CT)6	12	2361	2372	GCACTGAAAAAGGGATCCAA
scaffold740288_16.5	1	p2	(CT)8	16	2607	2622	GTGACGAGTTTGCGTGAGAA
scaffold740294_13.8	1	p2	(CT)6	12	1088	1099	CAAACACCCATTCAAACCCT
scaffold740309_16.4	1	p2	(AT)8	16	1083	1098	TTCCTTGAATCAGCCACACA
scaffold740311_17.7	1	p2	(AG)6	12	2268	2279	TGTGGATTCGTGTCGGATTA
scaffold740321_13.0	1	p2	(TA)10	20	1825	1844	GAACCTCCTCGTTAGCTCCC
scaffold740335_13.2	1	p2	(CT)7	14	1565	1578	TTATGCCCTCCATTCTCTGC
scaffold740353_18.5	1	p2	(AT)6	12	336	347	TTCTTTTATGGGCCATGAGG
scaffold740355_16.2	1	p2	(GA)9	18	2270	2287	TTTGGAATTCAAGGACTGGG
scaffold740378_16.4	1	p2	(TA)8	16	1072	1087	TTCGGTCAGATTATGTGGGG
scaffold740382_13.6	1	p2	(TA)6	12	1410	1421	TGCTTCATGAACCGACAAAA
scaffold740424_15.3	1	p2	(GA)6	12	1906	1917	GGAACTGAGCTTTGAGGGTG
scaffold740448_15.5	1	p2	(CA)6	12	1226	1237	TGGTTTACACTTTCCGGGTC
scaffold740452_18.1	1	p2	(TC)7	14	665	678	CACAGGAAAAGGAAACGGAA
scaffold740457_16.5	1	p2	(AT)6	12	387	398	ATGCACACCAACTGCCATAA
scaffold740485_15.3	1	p2	(GT)8	16	3885	3900	TGTCAAGTAACCATTCAGGGC
scaffold740524_14.0	1	p2	(TC)7	14	1328	1341	ACCACCTGCCCAACTATCTG
scaffold740534_16.0	1	p2	(TA)6	12	91	102	CAACAAGGTACTCGAAGAACACA
scaffold740536_17.0	1	p2	(GA)8	16	1816	1831	GCCTGGTTCCAAATTTTCCT
scaffold740542_17.7	1	p2	(AG)9	18	259	276	CCTCATTGCTGTCGCTATCA
scaffold740573_14.9	1	p2	(TG)6	12	577	588	ACAATCGGAGTGCCATTTTC
scaffold740580_13.2	1	p2	(TC)11	22	1410	1431	ACAGGAACAAGCCCATCAAC
scaffold740581_13.2	1	p2	(TA)9	18	963	980	TCAGCCCTGAGTGGTTTTTC
scaffold740585_15.4	1	p2	(GA)6	12	384	395	GAGAGGGGTGTTTGGTGCTA
scaffold740594_14.3	1	p2	(AC)7	14	632	645	TAGACAGTTGCACCCAGCCT
scaffold740607_16.4	1	p2	(TC)6	12	8655	8666	TTGGCTTTAATCAGGGCATC
scaffold740610_14.5	1	p2	(AT)9	18	1053	1070	AGTCGGGAGAAATCTCGTTG
scaffold740614_14.7	1	p2	(AT)6	12	522	533	CGAAACAGCAATCAAAAGCA
scaffold740619_15.0	1	p2	(GA)7	14	3302	3315	AGAATGGGACTCACTGGTGG

scaffold740657_18.[illegible]	1	p2	(GA)6	12	413	424 GCAGTGGAAGAACGAATATGG
scaffold740675_16.[illegible]	1	p2	(AT)12	24	49	72 GGAGGTGTTATGAAGGGATTACC
scaffold740683_15.[illegible]	1	p2	(GA)6	12	1882	1893 GAGGTAAATGTGGAAGCCCA
scaffold740689_15.[illegible]	1	p2	(CA)10	20	2043	2062 GCCATGATGCCTGTGAAGTA
scaffold740690_15.[illegible]	1	p2	(AT)9	18	475	492 GGAATTTAGACGATTGATCCGA
scaffold740726_15.[illegible]	1	p2	(TA)6	12	1372	1383 TTCCACCAGATACGGAGACC
scaffold740736_15.[illegible]	1	p2	(TA)9	18	46	63 CGGATTTATTGGTTATTACCTGA
scaffold740747_13.[illegible]	1	p2	(CT)6	12	616	627 CGACACATACGAATTGCCAC
scaffold740759_16.[illegible]	1	p2	(TC)8	16	3536	3551 GATACCCTGGTGGTCCATTG
scaffold740767_15.[illegible]	1	p2	(TC)6	12	186	197 TGAAATTTGGCTTGCATTTG
scaffold740768_16.[illegible]	1	p2	(CT)6	12	1071	1082 TGCTTTATGCAATGTCAGTGTG
scaffold740789_18.[illegible]	1	p2	(GA)6	12	1016	1027 ATGCAAAAATTGGTCCAACG
scaffold740812_17.[illegible]	1	p2	(TG)8	16	2619	2634 AGAAGGGGGATTGTTGCTTT
scaffold740818_14.[illegible]	1	p2	(TC)7	14	664	677 AGGTCTTTCGAACCCCCTTA
scaffold740819_14.[illegible]	1	p2	(AG)6	12	337	348 TGGGAGCTTTATGATTTGGC
scaffold740821_14.[illegible]	1	p2	(TC)6	12	345	356 CCACACCATAGCTGGTCGAT
scaffold740844_16.[illegible]	1	p2	(CT)10	20	783	802 TCCCTTTTTCTCAAGGGAGG
scaffold740854_14.[illegible]	1	p2	(AG)7	14	220	233 AGCTTGGCGAATTCATCATC
scaffold740855_16.[illegible]	1	p2	(AG)6	12	1301	1312 GCATGTTCATGGCTATCATTTG
scaffold740862_15.[illegible]	1	p2	(GA)8	16	1094	1109 CAGGAGAATTTGGATTCGGA
scaffold740865_17.[illegible]	1	p2	(AG)8	16	256	271 GTGACCGGTGGTTGTCTTCT
scaffold740876_16.[illegible]	1	p2	(TA)8	16	1095	1110 CTAAACTCCGCCAACTGCTC
scaffold740880_14.[illegible]	1	p2	(CT)8	16	118	133 CTGACATGGAAAGCACCCTT
scaffold740902_18.[illegible]	1	p2	(AT)6	12	890	901 AAGAATACCACCGCCAACCT
scaffold740923_13.[illegible]	1	p2	(AT)6	12	2314	2325 GAGGGTATCACCAATCTGCAA
scaffold740949_16.[illegible]	1	p2	(AG)6	12	1604	1615 CCCCTAGGGTTTCCCATTTA
scaffold740958_14.[illegible]	1	p2	(AG)8	16	1111	1126 GTTTGCTTTGGTGTGGGATT
scaffold740971_14.[illegible]	1	p2	(AT)8	16	87	102 ATTACGTCGATCCAACGGTC
scaffold740986_16.[illegible]	1	p2	(TC)6	12	1598	1609 TCCTCAGGAGTAAGAGGGCA
scaffold740988_17.[illegible]	1	p2	(AC)6	12	1996	2007 CTTTTCCATTTCCGATCCAA
scaffold740991_14.[illegible]	1	p2	(TA)8	16	3268	3283 TTAAACCATGCACGTAGGCA
scaffold740996_12.[illegible]	1	p2	(CT)9	18	2450	2467 AGATGTGCATTCAGAGGGTG
scaffold740999_16.[illegible]	1	p2	(TA)8	16	476	491 CATCGTTAATGTTGATTTTTATTCG
scaffold741004_13.[illegible]	1	p2	(AC)7	14	1973	1986 AGCCCTCCACGATAACATTG
scaffold741019_15.[illegible]	1	p2	(TG)7	14	759	772 ATGACATGGCCATAACGAGG
scaffold741029_16.[illegible]	1	p2	(AG)14	28	487	514 GAGACTGCAGCACTCACAGC
scaffold741032_15.[illegible]	1	p2	(TG)6	12	1568	1579 GGTGGTGGTGTTGAATAGGG
scaffold741043_15.[illegible]	1	p2	(TC)13	26	75	100 CACGTATGGATAAGGCTAAAGATG
scaffold741061_14.[illegible]	1	p2	(AG)8	16	565	580 CCGAAATCCGGGTTTTATTT
scaffold741082_15.[illegible]	1	p2	(AT)10	20	2274	2293 CTGTGTGCCTCCTCCTTTGT
scaffold741093_13.[illegible]	1	p2	(TC)13	26	618	643 TTTTATGCCAAAGCAAAGGC
scaffold741100_14.[illegible]	1	p2	(AT)8	16	1146	1161 TCGATTCTTGTCATCCATGC
scaffold741101_17.[illegible]	1	p2	(AT)7	14	1147	1160 GCCTAGCCGGTCAGTTACAC
scaffold741103_14.[illegible]	1	p2	(AC)9	18	1641	1658 TTTCTGATGGGGTTGAGGAG
scaffold741105_18.[illegible]	1	p2	(GA)6	12	3879	3890 CTGAATGCCTGTTGGTTCCT
scaffold741110_16.[illegible]	1	p2	(TC)9	18	591	608 TCAGAAACCACTCCCTCACC
scaffold741121_12.[illegible]	1	p2	(AT)8	16	1676	1691 GGGCAGCAACACCTTAAAAA
scaffold741122_13.[illegible]	1	p2	(AC)7	14	1932	1945 CACATTTCCATTTCCATTGGT
scaffold741129_13.[illegible]	1	p2	(AC)10	20	369	388 CACGCTTGCTAGACAACGAC
scaffold741138_15.[illegible]	1	p2	(CT)8	16	419	434 AAGAGAGGTCAAAGCTGCCA
scaffold741154_15.[illegible]	1	p2	(GA)10	20	3712	3731 GAAGAAGGAGAGGAGGCCAT
scaffold741157_12.[illegible]	1	p2	(TA)12	24	2783	2806 ATGGCGGTAGGTAGTTGTCG
scaffold741167_15.[illegible]	1	p2	(AT)8	16	1263	1278 AACCGAAGTGATTCGGGTTA
scaffold741185_15.[illegible]	1	p2	(GT)7	14	2428	2441 TTTGCTGAGCATGTCATGTG
scaffold741196_17.[illegible]	1	p2	(CT)7	14	5709	5722 GAGCAAAGAAGCCTCGAATG
scaffold741201_14.[illegible]	1	p2	(AT)6	12	511	522 TGGATGATGTCTGGGACTTTC
scaffold741212_18.[illegible]	1	p2	(AT)7	14	4661	4674 AGCTTCATCTCTGCGTCCAT
scaffold741213_16.[illegible]	1	p2	(GA)7	14	339	352 CATCCAGGTCACCCTTTTGT

scaffold741223_14.	1	p2	(AG)6	12	2117	2128	AGGCCTCGATAAATCCGTCT
scaffold741235_17.	1	p2	(CA)11	22	1163	1184	CTCAGCAATTGCCAAAGACA
scaffold741258_13.	1	p2	(AT)9	18	1503	1520	TCCCATTCAGGCTTGAGGTA
scaffold741277_18.	1	p2	(CT)7	14	49	62	TTTAATCGGGATCCGGGC
scaffold741279_13.	1	p2	(TA)8	16	2305	2320	GGCTCCATGGAAATGACAGA
scaffold741294_16.	1	p2	(AG)9	18	2969	2986	AGCCTCCTCAAATGCAGAAA
scaffold741303_15.	1	p2	(CT)6	12	523	534	GTTGCTGTCACTGTGCCACT
scaffold741305_15.	1	p2	(AG)7	14	1621	1634	CATTGGTGTTCGGAATAGGG
scaffold741316_13.	1	p2	(AC)7	14	143	156	TCCGACCGGTTTTTAAATCA
scaffold741339_15.	1	p2	(GA)8	16	495	510	TCAACTAGTGCTGCGAATGC
scaffold741351_13.	1	p2	(AC)6	12	3829	3840	AACTTAGTGCCCCAGTCCCT
scaffold741354_14.	1	p2	(GA)10	20	708	727	AAGCAGCCTCACACAAGTCA
scaffold741356_17.	1	p2	(AG)6	12	189	200	CCAACGCATCCATATCATCA
scaffold741380_16.	1	p2	(TC)13	26	2454	2479	GAATCTGTTGCGTCGTCTGA
scaffold741383_16.	1	p2	(GC)6	12	270	281	AAGTGCACTCGCTACGACCT
scaffold741386_21.	1	p2	(TA)7	14	52	65	CACCACATTTAGTGGTTATCATTTG
scaffold741411_16.	1	p2	(CT)6	12	2345	2356	GGAGGAGAGATGGCATGGTA
scaffold741426_15.	1	p2	(TC)9	18	1220	1237	ATTGGTCTCTCAAGCACGGT
scaffold741443_15.	1	p2	(TC)6	12	632	643	AATGAAACGGAATCCAGTCG
scaffold741448_14.	1	p2	(AT)10	20	214	233	CTTTTGGCTATCTCGCTTCG
scaffold741459_17.	1	p2	(AT)9	18	464	481	TGCACTTGCAAATTTAAGCTG
scaffold741462_16.	1	p2	(AT)8	16	1229	1244	CAGAGAGAAAATCGGTGGGA
scaffold741472_16.	1	p2	(TG)6	12	134	145	ACGACTCCATGTTCCGACAC
scaffold741476_13.	1	p2	(GA)6	12	2346	2357	TGTTGGAGACCCTTCTTTGC
scaffold741477_14.	1	p2	(AT)7	14	71	84	CCAGACTTTTGGTCTCAATATATCC
scaffold741510_17.	1	p2	(CT)8	16	1239	1254	TGCGCTCTTGTTCTGATTTG
scaffold741512_16.	1	p2	(AG)6	12	959	970	TACCAACCGGTTCCCTTGTA
scaffold741518_16.	1	p2	(CT)6	12	1063	1074	GGAACTGCAAAATTCTGGGA
scaffold741532_13.	1	p2	(CT)8	16	2003	2018	TTGGGTCTTGAAGAAGACGG
scaffold741535_15.	1	p2	(CA)11	22	5578	5599	AAGCACACCACCTCTGCTCT
scaffold741555_15.	1	p2	(TC)11	22	2399	2420	GGATCTTCGAGGAGTGGTGA
scaffold741584_15.	1	p2	(AT)10	20	1249	1268	CTATGCAATGCCACCACAAC
scaffold741589_16.	1	p2	(TG)6	12	1258	1269	TGTCGGAGTTTTGTGAGCTG
scaffold741592_13.	1	p2	(CT)10	20	3778	3797	TCAATGATGTTGTGGGTGCT
scaffold741602_16.	1	p2	(GA)6	12	1875	1886	TGTGTTCAGACCGAGTCAGC
scaffold741671_15.	1	p2	(TC)11	22	631	652	TATGATGCTGGACAAGTCGG
scaffold741680_16.	1	p2	(GT)7	14	2974	2987	TTAAGCCTGTAATGGGGCAC
scaffold741683_15.	1	p2	(CA)11	22	9540	9561	TCTTTGATTTGGCTGCTTTG
scaffold741697_16.	1	p2	(AT)6	12	1445	1456	TCTGGGATTGGGCTTTATCA
scaffold741700_15.	1	p2	(CT)7	14	4196	4209	TGTTGGTGTGATGGTTTTGG
scaffold741701_14.	1	p2	(CA)7	14	660	673	AAGCAACAAATGGCAGCTTT
scaffold741706_16.	1	p2	(AG)7	14	3517	3530	TAAGCAACCGAAAAGATGCC
scaffold741716_16.	1	p2	(GA)7	14	1758	1771	TTGCAGCAAATGGATCTGTC
scaffold741720_14.	1	p2	(CT)8	16	2281	2296	TCTTCTGCCGTTGATGTCTG
scaffold741727_15.	1	p2	(CT)6	12	2677	2688	CAAAGAAACCGGAAGGTTGA
scaffold741733_15.	1	p2	(TC)6	12	1303	1314	AGCGAAAACTAGATGCCGAA
scaffold741738_13.	1	p2	(TC)8	16	887	902	CGATGTTTCTCGTCTCACCA
scaffold741759_11.	1	p2	(GA)6	12	2383	2394	TGCTTTGCCTAGGCCTATTAAG
scaffold741788_17.	1	p2	(CT)10	20	391	410	ATCTTGCCGCTGTTAAGGAA
scaffold741810_16.	1	p2	(AT)12	24	2652	2675	GTTTCCGGTCAATGATGTCC
scaffold741811_15.	1	p2	(CT)7	14	1322	1335	ACAATCTTGGTGGTTACGCC
scaffold741812_15.	1	p2	(CA)6	12	2882	2893	TCTGCTTGTCAAAGTCGCAC
scaffold741826_18.	1	p2	(TC)6	12	1330	1341	CAAACCCCGAGTCTGATGTT
scaffold741843_14.	1	p2	(TC)6	12	1491	1502	GACCCCTCAGCAGTGACAGT
scaffold741868_15.	1	p2	(GT)6	12	2298	2309	ATTGCTGAATGTTGATGGCA
scaffold741872_14.	1	p2	(TG)8	16	1118	1133	CCGATAAGAATGGATGTGGG
scaffold741875_17.	1	p2	(AT)7	14	4062	4075	AAACAAGGGCAAGCATATCG
scaffold741890_16.	1	p2	(TA)7	14	122	135	TCGTTAGAGGTTTTGACGGG

scaffold741899_22.3	1	p2	(CA)6	12	3451	3462	TGGTGAATATAGAAGGGCGG
scaffold741903_16.4	1	p2	(AG)6	12	4178	4189	AAGCGCCAAATGTAAAATCG
scaffold741910_14.9	1	p2	(TA)6	12	116	127	CACACGCTGGAACTGCTTTA
scaffold741912_15.5	1	p2	(AC)7	14	1873	1886	CCTCACAATTTCCCCCTTTT
scaffold741913_16.0	1	p2	(AG)6	12	3485	3496	ATCGTCTCAATAGTTGCCCC
scaffold741920_17.0	1	p2	(AT)6	12	2814	2825	ATAAAGTAGTAGGCCCGCCC
scaffold741926_14.8	1	p2	(AC)6	12	3793	3804	ACAAGAAACGGTGAGAACCC
scaffold741941_19.0	1	p2	(GT)8	16	1365	1380	CTGATGCAGATGCAGACAGT
scaffold741948_15.0	1	p2	(AG)7	14	5572	5585	TTCGTGCTCTTGAACGTGAC
scaffold741958_18.0	1	p2	(GA)8	16	719	734	AAATGAAGTTGGGCAGTTCG
scaffold741971_18.9	1	p2	(AG)6	12	2330	2341	GGCATGCAATTTGTCAAGAA
scaffold741984_14.2	1	p2	(AT)11	22	3995	4016	CCAAAGAGTGACGACCGAAT
scaffold742012_17.0	1	p2	(GT)6	12	3487	3498	TCGTGTTCTCAGGATGTTGC
scaffold742020_16.4	1	p2	(TC)6	12	63	74	GTGCAATGTGAGTGGACGTT
scaffold742021_15.0	1	p2	(TA)7	14	2840	2853	GGTTCGAGTTTCGATGAGGA
scaffold742032_15.1	1	p2	(GA)6	12	2682	2693	TCCGAACAAAATGCACAAAA
scaffold742035_17.6	1	p2	(CT)6	12	3224	3235	CCAAGAAATCAAGGATCGGA
scaffold742061_16.2	1	p2	(AC)9	18	4743	4760	GATGCCAAGGAACCTGTTGT
scaffold742070_16.3	1	p2	(TA)9	18	1416	1433	ACAGGATCTGCAGGTGGAAC
scaffold742080_16.5	1	p2	(AG)8	16	1429	1444	TGACTTTTGGGGTTTTCGAG
scaffold742085_15.0	1	p2	(TA)7	14	777	790	TGTTTGTGGTTGAGTACGCC
scaffold742090_14.8	1	p2	(TC)8	16	3293	3308	CTTCTGTTGAAACCCCGGTA
scaffold742133_15.0	1	p2	(TA)6	12	2241	2252	TGTTCTTCAAATGGGATGTCA
scaffold742154_17.7	1	p2	(TA)7	14	1465	1478	TGTGTTGCCAAAAACACGAA
scaffold742165_15.4	1	p2	(AC)10	20	4075	4094	TGAGCTGGACGGACATTGTA
scaffold742174_14.1	1	p2	(AG)7	14	2635	2648	CAAGGCGTTGGAAAAAATGAT
scaffold742179_15.3	1	p2	(TG)8	16	696	711	TTTTGCTTCCACTGCTTGAA
scaffold742192_14.8	1	p2	(CT)11	22	1672	1693	TAGTACCATCGCCACCCTCT
scaffold742197_15.4	1	p2	(GT)7	14	1513	1526	ATGGAGACTTATGGAGGGGG
scaffold742198_16.5	1	p2	(GA)13	26	2640	2665	AGAGCGTTAGCTCTTTCGCC
scaffold742211_16.4	1	p2	(AG)6	12	5152	5163	AACCTGTATTGGGACATCGC
scaffold742218_15.5	1	p2	(AG)7	14	3402	3415	GTTTATCTTCGGCTTGCTGC
scaffold742221_17.5	1	p2	(CT)7	14	2119	2132	TGCTCTTATTCATGGTCCCC
scaffold742223_13.4	1	p2	(TA)6	12	1682	1693	TCAGTTTTCGAGTTATGAATTTGC
scaffold742236_16.0	1	p2	(CT)6	12	2902	2913	AAGCAGCAATCTCTGCCATT
scaffold742248_16.1	1	p2	(AC)6	12	587	598	GTGTGGATGTCCATCACTGC
scaffold742253_16.4	1	p2	(TA)7	14	3250	3263	AAATCTGCATGCACCTGTTC
scaffold742279_15.1	1	p2	(TG)7	14	856	869	TGTTCAAACTTTTGTGGGCA
scaffold742280_15.5	1	p2	(GA)8	16	3022	3037	CACCACAGAAGAGGGAGAATG
scaffold742293_15.2	1	p2	(CA)9	18	83	100	CTCGAACGGGATCTTGATGT
scaffold742300_16.0	1	p2	(CA)7	14	1214	1227	AAGCAAACACCACAACACCA
scaffold742309_16.8	1	p2	(CT)6	12	270	281	ATATATTGTGAGCCCTGGCG
scaffold742329_15.0	1	p2	(AT)7	14	206	219	GCAGTGCTTCAAGAGGGAAC
scaffold742332_17.5	1	p2	(TC)9	18	997	1014	TTCAATTTGCCTGTGGAAGA
scaffold742343_16.7	1	p2	(AG)7	14	1193	1206	TCGCCTCTCAAGATTGTGAAT
scaffold742351_17.3	1	p2	(CT)7	14	155	168	AACACGCACTCTTTCACACG
scaffold742357_16.6	1	p2	(GA)6	12	819	830	GATGGTAATTGGGAACGGTG
scaffold742366_17.5	1	p2	(AG)9	18	1945	1962	GAATAAACGGCGGTAATGGA
scaffold742411_17.9	1	p2	(CT)6	12	211	222	CGTCATCGTATTGGCTTCCT
scaffold742422_15.4	1	p2	(AT)6	12	2737	2748	CTTTTGGACCTCCCTTCCTC
scaffold742423_14.8	1	p2	(AT)10	20	4316	4335	TCTCCTCCGTGGACGTAGAT
scaffold742424_17.8	1	p2	(TA)6	12	167	178	GCTCTGATGCCAAACTGTTG
scaffold742447_14.6	1	p2	(AG)7	14	1433	1446	GTGGCAAATGAGGAGATGGT
scaffold742451_15.0	1	p2	(TC)9	18	2661	2678	CAATGAATGACCAGCCGTAA
scaffold742452_13.8	1	p2	(TG)6	12	1347	1358	TGAGTTGTTCATTTCAGATGTTGTT
scaffold742455_15.0	1	p2	(AT)9	18	1436	1453	CAGTTTTTGAGTTATGATGCTTTCA
scaffold742465_19.8	1	p2	(GA)7	14	32	45	TCTCTCTCTAACACACAAACAGAGAA
scaffold742467_17.3	1	p2	(CT)6	12	813	824	AGCGACCAACAGGTAACGAT

scaffold742475_15.5	1	p2	(TC)6	12	3959	3970	CATTTTGCTTTGCAGGAACA
scaffold742477_15.4	1	p2	(TC)6	12	2147	2158	ACACTATTACGCCTGGCTGC
scaffold742494_14.3	1	p2	(TC)8	16	2622	2637	AAGCTGATGCCATATCCTCG
scaffold742495_14.1	1	p2	(AT)10	20	4171	4190	GGCTCGAACCCAAAATATCA
scaffold742517_15.6	1	p2	(TA)6	12	5860	5871	CCGAATCCTTTCATAGAGCG
scaffold742539_17.3	1	p2	(TC)6	12	6158	6169	CCGATCCCAATCTCTCCATA
scaffold742546_14.7	1	p2	(AG)7	14	104	117	GTCCGTTGGTTTACCCAGAG
scaffold742556_16.9	1	p2	(AT)6	12	624	635	CAAGGAAGGAGGAGTGGTTG
scaffold742557_14.0	1	p2	(AT)10	20	1150	1169	GAGTGGGTATGAGGGGAACA
scaffold742558_15.5	1	p2	(TC)12	24	2355	2378	AAAGGCAATCCCACCTTTCT
scaffold742560_15.6	1	p2	(AT)7	14	2082	2095	CAAAGGTTCCAAGCCGTTTA
scaffold742566_16.6	1	p2	(TA)10	20	46	65	GGGGTTATCATGGGATCAAG
scaffold742587_15.9	1	p2	(CA)6	12	6974	6985	ACTTGCGTAGCATCATCCAA
scaffold742595_18.2	1	p2	(CA)7	14	2364	2377	TCGATGCATACTTGTTGGGA
scaffold742597_15.7	1	p2	(GA)9	18	1982	1999	TAAACCTGGAGGAATTGCCA
scaffold742625_14.7	1	p2	(CT)8	16	1869	1884	TCGTCACGTGCGATTTTAAC
scaffold742629_14.6	1	p2	(TA)6	12	1333	1344	CCCAGTTCGTCGAGAAAGTC
scaffold742640_17.0	1	p2	(GA)6	12	542	553	CAGACCATCTGGGAACTAATCA
scaffold742651_16.0	1	p2	(CT)9	18	902	919	TGGAGCGCAGGCTATAAGAT
scaffold742666_15.8	1	p2	(CT)6	12	2880	2891	AACAGCACTGACTGGCTTCA
scaffold742690_16.5	1	p2	(AG)12	24	1122	1145	GCAAGAACGAAGATTCCAGC
scaffold742699_15.7	1	p2	(AT)8	16	759	774	TGCAATAAAAAGACCTTGCATTT
scaffold742709_16.6	1	p2	(GA)9	18	1367	1384	TCTCTTCCACAATCACGCTG
scaffold742713_15.9	1	p2	(AT)7	14	1855	1868	CCAGGAAGACTCATTCGATCC
scaffold742725_15.7	1	p2	(TA)8	16	197	212	CTCACACCCACATCCATCAG
scaffold742727_16.2	1	p2	(AT)7	14	1868	1881	ACCTATTGAAAGCCTCCCGT
scaffold742742_14.5	1	p2	(TA)7	14	793	806	GACGGCTTTCTCAACACCTT
scaffold742753_14.8	1	p2	(TC)10	20	2651	2670	AGCAGACTGGTTCCAAAAGC
scaffold742756_18.3	1	p2	(CT)8	16	423	438	GGGGCTGCTTTCTCTTCTCT
scaffold742761_16.3	1	p2	(AG)10	20	2999	3018	TACTGCAAATTCCGCCTCTT
scaffold742763_16.0	1	p2	(TG)6	12	5372	5383	TGGGTCTCAGATTCTTGGTCA
scaffold742766_16.4	1	p2	(AG)6	12	855	866	TATGCATCGCTCAGATTTCG
scaffold742776_16.7	1	p2	(AG)8	16	730	745	GGTACTGGTGGAACTGCCAT
scaffold742777_17.7	1	p2	(AT)9	18	9078	9095	AATGTTTCCTGCCACTCAAA
scaffold742780_15.0	1	p2	(AG)6	12	8006	8017	CCATACAAGCAGTGGCAGAA
scaffold742781_15.0	1	p2	(CT)7	14	1034	1047	GGAAACAAACTATCCCATTTGC
scaffold742797_15.6	1	p2	(TA)6	12	519	530	ACCTCAGAAACCAGTGGTCG
scaffold742816_14.9	1	p2	(TG)6	12	2611	2622	CTTCAGTACTTGGCCCACTTG
scaffold742848_15.2	1	p2	(CT)8	16	2119	2134	CCATTTTCTTATCTCGCCCA
scaffold742852_16.5	1	p2	(TG)6	12	1600	1611	TGCTGACAGGATTAATTGATGG
scaffold742877_16.3	1	p2	(CT)9	18	441	458	GTTGAATCGATTGTACCGCC
scaffold742908_17.8	1	p2	(CA)6	12	728	739	GGCCACATGCTCAATTACCT
scaffold742937_15.6	1	p2	(TA)6	12	51	62	TGAAGTTAGGAAATAAGGAGGTGTT
scaffold742941_15.5	1	p2	(TC)10	20	7420	7439	GATCCTTCCTCTCGTTATGGC
scaffold742946_14.0	1	p2	(TG)7	14	2203	2216	TCGCGTAAACAACACGATCT
scaffold742952_18.4	1	p2	(CT)6	12	319	330	TTATCGGCTGTACTCTCTCGC
scaffold742993_16.0	1	p2	(AT)7	14	2079	2092	TCCTACAACTCCGGTTCCTG
scaffold742996_16.7	1	p2	(AT)9	18	2273	2290	AAGATTTTTGTCCAGTCGGG
scaffold743002_16.5	1	p2	(TC)8	16	312	327	ATCCCTCGCGCTCACTACTA
scaffold743011_17.9	1	p2	(TC)9	18	297	314	TATGGGATTGGGTCTTTTGC
scaffold743032_16.0	1	p2	(AG)6	12	4618	4629	ATTAAAGCACCTTTCGCGTG
scaffold743041_13.5	1	p2	(AT)7	14	2380	2393	GGGGAAAAACCTTTCCAGAG
scaffold743047_16.3	1	p2	(AG)8	16	181	196	CTGGTCCATTCAAACCTGCT
scaffold743049_14.0	1	p2	(AT)6	12	4783	4794	CCGCATCTTCTAACGAGAGG
scaffold743051_16.6	1	p2	(TC)8	16	3302	3317	AAGATGTCGCCGTCTCTCAC
scaffold743055_15.9	1	p2	(TC)12	24	1462	1485	AGGCCCAAGACCTTACGAGT
scaffold743065_15.9	1	p2	(AG)6	12	5227	5238	CTCGAGGAAGTAATCGCAGG
scaffold743066_15.9	1	p2	(TA)11	22	93	114	TTTTCGAGTTATGAAGTTAGCACC

scaffold743076_15.[illegible]	1	p2	(TA)8	16	350	365	CCCTCCCTTAAAATCGGAAA
scaffold743078_23.[illegible]	1	p2	(TA)8	16	228	243	GCCAAGTTCACTAACCTTTTTCTC
scaffold743085_16.[illegible]	1	p2	(AT)7	14	1049	1062	AACCAACATGCACAACCAAA
scaffold743101_16.[illegible]	1	p2	(GA)6	12	2997	3008	GGGTTCCTGCGTCATAAAAA
scaffold743107_16.[illegible]	1	p2	(GA)6	12	47	58	GGAGAGAGAGTGTTGGGTAGAGA
scaffold743132_15.[illegible]	1	p2	(TA)7	14	2359	2372	ATATCAGAAAGCCACACGGC
scaffold743175_16.[illegible]	1	p2	(AT)10	20	48	67	CGCCGGTGCCTATCATATT
scaffold743185_14.[illegible]	1	p2	(AG)8	16	6900	6915	GAACACCTCTCCAGAGCCAG
scaffold743191_16.[illegible]	1	p2	(CT)6	12	3218	3229	CAGCAAATACTCTGCACCGA
scaffold743198_16.[illegible]	1	p2	(TC)6	12	1295	1306	TATTCCGAGTTTCATTCGGG
scaffold743205_16.[illegible]	1	p2	(AT)10	20	2391	2410	GGAAATTGAAGCTGTTGCCT
scaffold743207_15.[illegible]	1	p2	(TA)6	12	87	98	CAGATGCTCGAACAAGTCCA
scaffold743212_16.[illegible]	1	p2	(AC)7	14	1887	1900	TTACGGATATGGCCGGTTTA
scaffold743221_14.[illegible]	1	p2	(TC)7	14	1824	1837	TTTCATCGTCTGCAACAAGC
scaffold743224_14.[illegible]	1	p2	(AT)6	12	2443	2454	GGTTTGTGGGACAAAAGGAA
scaffold743226_16.[illegible]	1	p2	(GA)11	22	3727	3748	GATCAATGTCTTTGGGCGTT
scaffold743241_16.[illegible]	1	p2	(GA)6	12	328	339	TTTTCCTAGAGAGGGAGGGG
scaffold743246_15.[illegible]	1	p2	(GA)10	20	2160	2179	TCACTGTTTATCCCCCTTGC
scaffold743251_14.[illegible]	1	p2	(AT)6	12	674	685	GGAGAAAGTTGATTTGTTGGGA
scaffold743261_16.[illegible]	1	p2	(AT)6	12	1763	1774	TTGTTGCCTTCTGATGCTTG
scaffold812_14.6	1	p3	(ATT)5	15	903	917	GGCCAACCCATTTGACTTTA
scaffold1098_16.9	1	p3	(ATC)5	15	248	262	GGGTGGTATGGTGGAAGAAA
scaffold1208_14.5	1	p3	(CTC)5	15	689	703	CATTCACCACATGAGATGCC
scaffold1331_10.4	1	p3	(ACA)5	15	334	348	CCCTTTGAGAATAATGACATTGG
scaffold1455_15.6	1	p3	(ATT)5	15	316	330	CGAACCGGAATCGTAGTTGT
scaffold1511_11.7	1	p3	(ATG)5	15	193	207	GCGACATCCACATAACATGC
scaffold1599_16.9	1	p3	(ATT)7	21	721	741	CTATTTGGGAGTGGGTGGAA
scaffold1761_16.1	1	p3	(TCA)6	18	424	441	GCCTTCAAAAACCATGCAAC
scaffold1840_16.0	1	p3	(CCA)5	15	821	835	GGTGTTGATTCCAGCAGCTT
scaffold2512_10.9	1	p3	(TGA)8	24	235	258	CCCAAGACCCCAATTTGATT
scaffold2627_15.0	1	p3	(ATT)5	15	104	118	TCTTTCGTTGCCAGGGTAAA
scaffold2962_15.4	1	p3	(AAT)5	15	237	251	TTCCGTTACCCTCGACTTTG
scaffold3235_12.9	1	p3	(CGG)5	15	184	198	AGTTGCAGCAGCATTCCTCT
scaffold3590_15.1	1	p3	(TCT)8	24	1917	1940	TCTGATCAAAACTGCTTCGG
scaffold3649_15.8	1	p3	(CGG)6	18	622	639	AGACCCACAATTCCAACCAC
scaffold3663_17.9	1	p3	(ATG)5	15	103	117	TCGAGTAAGCGGAAATCAAGA
scaffold3668_15.3	1	p3	(CTA)5	15	1452	1466	TGCATCAATCTCATTTCCCA
scaffold4159_16.1	1	p3	(GAC)5	15	905	919	TAAAATGGAGAAAATGCGCC
scaffold4441_14.6	1	p3	(GAT)5	15	5068	5082	GCCATTACATGCAGGGTTCT
scaffold4575_14.6	1	p3	(TCA)6	18	726	743	GCTCATGTCAACGTTTTCTGG
scaffold5211_16.6	1	p3	(ATT)5	15	1169	1183	CTGATCCCATGTTTTCTGCAT
scaffold5479_13.8	1	p3	(TTA)5	15	486	500	TCCCTATAACCGATACACCG
scaffold5712_14.7	1	p3	(CCG)5	15	121	135	CTTTTTGTATGGGTCGCGTT
scaffold6097_16.9	1	p3	(ACA)5	15	591	605	TGGAAGTTATGGTCGCAACA
scaffold7149_14.2	1	p3	(TTC)5	15	428	442	ATCGGGTGGAGATTGTTACC
scaffold7235_13.8	1	p3	(CAC)5	15	135	149	ATCACCGGCAGCAGATTAAG
scaffold7350_12.3	1	p3	(ATT)5	15	178	192	ATAAAGTGGCGGAGCTAGGG
scaffold7927_17.0	1	p3	(GAT)5	15	3619	3633	AGAGGAAAATGGAAAGGCGT
scaffold7953_13.9	1	p3	(TTA)6	18	147	164	CCACAGCATTGACATTAGGG
scaffold8247_15.0	1	p3	(CCG)5	15	1548	1562	CTGAAATCCCATTCGCATCT
scaffold8307_13.8	1	p3	(TGT)5	15	46	60	ATGTAGCCGACCCCAATTTA
scaffold9587_14.7	1	p3	(TTA)5	15	525	539	CGTTTTATCTGCGGATTTCAA
scaffold9866_15.2	1	p3	(AAT)5	15	2561	2575	ACAAGTTCGAAACCTGGACC
scaffold9883_12.9	1	p3	(TGA)6	18	43	60	CCCAAGACCCCAATTTGATT
scaffold10562_15.0	1	p3	(AAC)8	24	1636	1659	AGTATGACGGCCATGGGTAA
scaffold10985_16.8	1	p3	(AAG)5	15	52	66	TCAATATCTTACAATATCACAATGGC
scaffold11170_10.4	1	p3	(AAT)7	21	211	231	TGTTCGGACTGAACTGGTGA
scaffold11593_14.0	1	p3	(ACA)5	15	138	152	CGAGGCTGAATTTATGGCAC

scaffold12446_16.1	1	p3	(CCA)5	15	4411	4425	AACATTCCGCCACAGATAGG
scaffold13512_13.6	1	p3	(AAT)5	15	131	145	GGATGATGGATTCGAGTGGA
scaffold14589_16.6	1	p3	(CCG)5	15	1974	1988	GATTTTGCGGCGTTTGTATT
scaffold14724_15.1	1	p3	(GAA)5	15	52	66	GCTTCTCACGATTCTTGGTGT
scaffold14863_12.8	1	p3	(ATC)7	21	223	243	TGCAACACTTGAGCTTATGGA
scaffold14883_16.8	1	p3	(ACA)7	21	1620	1640	TGTTCGGTGAATGTTTTGGA
scaffold14889_13.5	1	p3	(GGA)5	15	430	444	AGCAATGCGAGGATGAAGAT
scaffold14944_10.2	1	p3	(ATA)5	15	52	66	CTACCTCGCCTCGCTATTTG
scaffold15105_16.5	1	p3	(ATC)6	18	1106	1123	TTTATTAGAATGCCGGGGAA
scaffold15303_14.0	1	p3	(TTA)5	15	115	129	GAAAAAGATGGTAAAAACCTTCCA
scaffold15875_18.9	1	p3	(GCA)6	18	31	48	CAGCCAAACATTGAGCAGTT
scaffold15940_14.7	1	p3	(TTC)5	15	970	984	AATGCCTTATTTTCCCACCC
scaffold16359_14.2	1	p3	(AAT)6	18	315	332	CAGAGCGCACTCGAATAACA
scaffold16669_13.0	1	p3	(AAC)6	18	286	303	TTCGAACCTTCTGCAAAATATAA
scaffold16827_11.6	1	p3	(TAA)5	15	58	72	AAGAACATTTATGGCAAACAACA
scaffold16963_18.3	1	p3	(GTG)5	15	539	553	CTACCACTGCCGGAAAACAT
scaffold17092_16.0	1	p3	(ATA)5	15	5841	5855	TTGAAGAGAGGCAACGAGGT
scaffold17138_15.2	1	p3	(ACT)5	15	1688	1702	TCTAGCCCACAACTTGGCTT
scaffold17836_16.5	1	p3	(ATG)5	15	380	394	TCATTGAGCATGCTACGACC
scaffold18172_21.9	1	p3	(ATG)5	15	37	51	CCTGAAACGAGTGATGCTCC
scaffold19294_14.7	1	p3	(AAC)5	15	1911	1925	CCGAACCGGAAAAGTCACTA
scaffold19396_18.2	1	p3	(TTA)5	15	757	771	CCACCTACAGGTGCACAAGA
scaffold19406_16.1	1	p3	(CGG)5	15	1370	1384	TTGGCTATCGGTTACTTGGG
scaffold19923_16.4	1	p3	(ATA)5	15	12235	12249	ACCTTCTTGCAACCAACGTA
scaffold20611_15.9	1	p3	(ATA)5	15	2540	2554	ATCGAACCGTGAACACCCTA
scaffold20806_11.9	1	p3	(GAA)5	15	270	284	CTCCTGCTTCACCTCGATTC
scaffold21294_13.9	1	p3	(GAC)5	15	53	67	ACAAAAATCCAACAAACCCG
scaffold21378_21.3	1	p3	(TAA)6	18	751	768	TGTGATTTCATTCGCATGTTC
scaffold21743_14.8	1	p3	(TGT)7	21	1398	1418	TGTCTTCTATTCATCTGGCCG
scaffold21825_14.7	1	p3	(TAT)8	24	653	676	TTGTACTGTGTTGGCTTCATGTT
scaffold21902_15.6	1	p3	(GTT)5	15	53	67	TGGTTTTAATTGTGGGCTCTC
scaffold21940_12.3	1	p3	(AGA)5	15	53	67	TTTTTCACGGTTGGAAAGGA
scaffold22365_15.4	1	p3	(ATC)6	18	61	78	TCAATCACCAATCAACACTTGAA
scaffold22515_13.1	1	p3	(ATT)5	15	504	518	TGGTTGAAAACCAAGGTGATG
scaffold23725_14.0	1	p3	(TGT)6	18	41	58	CCAATTTATTGGGATTAAGGC
scaffold23989_13.8	1	p3	(TTA)5	15	526	540	CAGCAAAACAACTTCATGTACGA
scaffold24715_16.6	1	p3	(TCA)5	15	852	866	CAGGCACTCCAAAATCAGGT
scaffold24965_16.0	1	p3	(CTT)5	15	81	95	CGCCGTTAGCAAAGGAATTA
scaffold25050_11.6	1	p3	(TGT)5	15	47	61	TGTAGCCGACCCCAATTTAT
scaffold25213_12.5	1	p3	(GAT)5	15	118	132	TACAAATCAAGCACGCAAGC
scaffold25814_11.1	1	p3	(ATT)5	15	229	243	TGAATTGGGTTTGACAATCG
scaffold25849_12.3	1	p3	(TTC)5	15	114	128	GGCGATTGTCAACTTGGTTT
scaffold25865_17.8	1	p3	(ACC)7	21	1329	1349	TGTCTGAAATAGCTTGGCCC
scaffold25889_18.4	1	p3	(TTC)5	15	786	800	TCATCACTCCACCTGCTCAC
scaffold26004_13.0	1	p3	(CAT)6	18	868	885	CCACAAGTTATCACACCCCC
scaffold26245_16.2	1	p3	(TTA)6	18	327	344	TTTTACGTTGTACGAATCCTATCTTT
scaffold26323_10.2	1	p3	(AAT)5	15	70	84	ACATCGCATACGTGTATGTTTAAT
scaffold26655_15.0	1	p3	(ATG)6	18	1001	1018	ATCCCAAGATGACCATGAGC
scaffold26685_12.9	1	p3	(TAT)6	18	2097	2114	ACGTGGGACCTTTCAGTCAC
scaffold27208_17.2	1	p3	(ATA)5	15	227	241	AATGCTGGAAATTCGATTGG
scaffold27305_15.1	1	p3	(CAC)6	18	2093	2110	TCTCCATGATTCCATCGTCA
scaffold28279_12.8	1	p3	(TTC)5	15	189	203	ATTTCGGGTCAACCTTTGAA
scaffold28381_14.5	1	p3	(ATC)5	15	64	78	GTGATGCCATAACATCGTCG
scaffold28526_13.0	1	p3	(TTA)5	15	224	238	TTTAAAAATCGGACCGGAGA
scaffold28775_13.4	1	p3	(AAT)5	15	233	247	TCGTGCAATAAAACTCGTGC
scaffold29076_18.7	1	p3	(CAG)5	15	466	480	ACATGTTGAGTCCGAGTCCC
scaffold29092_12.0	1	p3	(ATT)5	15	150	164	TGAAGGACCCGTCATACAACT
scaffold30000_12.6	1	p3	(TAT)5	15	700	714	ATTTGGTGCTCAGTGGTTCC

scaffold30204_15.3	1	p3	(AGC)6	18	128	145	TACGGCCGGGTAAATTACAT
scaffold30252_15.9	1	p3	(GAG)5	15	2987	3001	ATGGCCAGAGGAAGTAAGCA
scaffold30258_11.0	1	p3	(AGA)5	15	435	449	GATTGTTGAAGGCCGACAGT
scaffold30697_15.9	1	p3	(TGT)5	15	75	89	CATGTAGCCGATCCCAATTT
scaffold30714_14.5	1	p3	(ATG)5	15	684	698	TGGCAACTCAAAAATGCTCA
scaffold30745_12.4	1	p3	(CCG)5	15	235	249	AATACCGATACCCACTCCCC
scaffold31031_15.0	1	p3	(CCA)5	15	1588	1602	CCAGCAGAACAAGAACACCA
scaffold31088_10.2	1	p3	(GTG)7	21	733	753	GGATTTGGGAATGGAACTTG
scaffold31311_10.3	1	p3	(GCT)5	15	329	343	CCCAGTTTTGGTCGTACTGG
scaffold31601_14.9	1	p3	(ATC)5	15	407	421	ACCGTCCACGTCAGTATTCC
scaffold31994_14.6	1	p3	(ATA)7	21	516	536	CTTGCTTCCTCGCTTGTCTC
scaffold32356_16.6	1	p3	(CAT)5	15	382	396	TGTTGAAAGAGCCTCCATCC
scaffold32738_15.2	1	p3	(ACA)6	18	431	448	TAGTGTAGGACCCCTCACGC
scaffold32880_15.8	1	p3	(TTA)5	15	1877	1891	TTACCAGGTGGCAACATTCA
scaffold32930_16.0	1	p3	(TTG)5	15	227	241	ACCCTACATAGCGGGGAAAG
scaffold33369_12.1	1	p3	(GGC)6	18	245	262	GAGGGGGAAAAGAGAGGATG
scaffold33812_16.0	1	p3	(GCG)5	15	711	725	CGTTACTTCCATTGACCGCT
scaffold33872_13.8	1	p3	(GGC)5	15	267	281	TCGACCCAAATAGAAGACCG
scaffold33961_10.0	1	p3	(TGA)5	15	450	464	TGTTGTGTATTGACCCCCAA
scaffold34135_16.6	1	p3	(ACA)6	18	1568	1585	GTGGTCCACTCTAATGCCGT
scaffold34209_16.6	1	p3	(TTC)5	15	330	344	GCTGAGTACCCCATCTTCCA
scaffold34421_12.0	1	p3	(AAT)5	15	701	715	TGGAGGAATAAAGGCTGGTG
scaffold34893_10.5	1	p3	(GGC)5	15	233	247	ATCTTCTTCTGGATGCGGTG
scaffold35049_10.4	1	p3	(TGG)6	18	160	177	AACATCCAAGTTATTCACGGA
scaffold35083_15.4	1	p3	(CAA)5	15	140	154	ATTCGCCGACAAGATAATGG
scaffold35166_18.5	1	p3	(GCA)5	15	1645	1659	GTAGACGCCAGCCAGTTAGG
scaffold35388_12.1	1	p3	(CAA)5	15	310	324	ATCAAACACGCCAATCATCA
scaffold35477_15.4	1	p3	(GAT)5	15	246	260	CGCAACCATTTCATTGTGAC
scaffold35574_12.5	1	p3	(AAT)6	18	398	415	AATGCGTAACCCTTGTTTGC
scaffold35645_15.1	1	p3	(TAT)5	15	2047	2061	GGGTTTGAATTTTGTGGCAT
scaffold35846_10.7	1	p3	(TTA)6	18	222	239	TTTGTGTCGATCAACTTGTGTG
scaffold36287_19.6	1	p3	(GCT)5	15	286	300	GCCAGAACCGAGAGCACTAC
scaffold36382_13.1	1	p3	(TAA)5	15	1342	1356	CATAAATGCCGAAATGGAGA
scaffold36497_17.3	1	p3	(GCT)5	15	433	447	CAATGACGAATAGCAGCCAA
scaffold36522_15.9	1	p3	(AAT)7	21	221	241	CCGGATATCCAGTTTCATCC
scaffold36721_15.0	1	p3	(ATC)5	15	928	942	CAGCGAATCAATACGTGCAT
scaffold36722_16.1	1	p3	(TCT)5	15	642	656	CGCCGAAGTCATTAGGTCTC
scaffold37851_14.3	1	p3	(GAT)5	15	115	129	ACGAGCAAGAGGATGAGGAA
scaffold37987_13.4	1	p3	(AAT)6	18	1384	1401	CTTTCGTTGATCGATTGGGT
scaffold38130_15.7	1	p3	(CAT)5	15	1739	1753	TAAGGTTGGCTGTAATGCCC
scaffold38136_13.2	1	p3	(TGA)5	15	111	125	CCCAAAACCCCAATTTGATT
scaffold38205_12.6	1	p3	(TAT)5	15	213	227	TTTTTCCACCACTTATTCTTTTGA
scaffold38441_21.3	1	p3	(TAA)5	15	159	173	TTCGAATGTTGCTGCTCAAG
scaffold38600_15.0	1	p3	(GGC)5	15	1119	1133	AGAAGAGGGAAAAGCCCAAG
scaffold39144_18.3	1	p3	(TGA)5	15	43	57	CCCAAGACCCCAATTTGATT
scaffold39186_10.0	1	p3	(ATC)6	18	89	106	TTTCATGGGTACACATTTGAGC
scaffold39431_14.6	1	p3	(GAA)5	15	195	209	GGGAAGGGGACAACATTTCT
scaffold39471_13.4	1	p3	(GTG)5	15	376	390	ATCCACTTTTCTGCTTTGGC
scaffold39673_14.5	1	p3	(ATT)5	15	190	204	TTGATCACACACGAGTCCATT
scaffold39682_16.5	1	p3	(TCA)5	15	495	509	CGTTGGTGAAGAACAACTTTGA
scaffold39754_13.6	1	p3	(TAT)5	15	710	724	GTTTGGAGGTCAGGTGTTCG
scaffold39984_17.0	1	p3	(AAT)6	18	471	488	ATGGCCTCCCACCTTCTATT
scaffold40059_13.1	1	p3	(TGT)7	21	50	70	TTTGGTTCATTTAGCCGACC
scaffold40407_12.7	1	p3	(ATA)5	15	386	400	TCTCTCTTGCCCAGCATCTT
scaffold40412_17.5	1	p3	(ATG)5	15	37	51	AGGAGGATGACGACGATGAT
scaffold40525_19.1	1	p3	(GAA)5	15	199	213	CCACCAATGACAGCAATCAG
scaffold40587_13.6	1	p3	(CGG)5	15	50	64	CCACCATCGTCGTCTCATC
scaffold40594_12.2	1	p3	(AAT)5	15	328	342	ACGTTTACGCTCTGCGATTT

scaffold40686_10.2	1	p3	(AGA)5	15	68	82	TCTACTTAGAGCAGGGCCGA
scaffold40769_15.6	1	p3	(TCG)5	15	563	577	TTTTGTTGTTTGCTGCTTCG
scaffold40883_17.9	1	p3	(CTT)5	15	2833	2847	TTCGAAGCTTGGAGAACGAT
scaffold41212_10.4	1	p3	(ATC)5	15	867	881	CAAAACCTTGGTGAACAGGC
scaffold41535_13.9	1	p3	(TGT)7	21	39	59	TGTAGCCGACCCCAATTTAT
scaffold41659_15.8	1	p3	(TCT)5	15	886	900	TGGGGGAGAAGTATGTGAGG
scaffold41863_14.8	1	p3	(GAA)5	15	605	619	CCGTCAAGCTCTATGCTTCC
scaffold43211_15.3	1	p3	(CCG)5	15	4494	4508	AATCACAAACAAATTCCGGC
scaffold43343_13.9	1	p3	(ATG)6	18	102	119	TGGCTCGAATTACAAAAACCA
scaffold43373_16.5	1	p3	(ATT)6	18	725	742	TGCACTGCTTATCAGATTCAAG
scaffold43915_17.7	1	p3	(GAT)5	15	329	343	AGTTGAAATGCTTGCATGGG
scaffold44026_10.4	1	p3	(CTC)5	15	452	466	TGCCTCAGACTCTTCCACCT
scaffold44112_12.7	1	p3	(CCT)5	15	303	317	GCTCCTCCACCTCTGTTCTG
scaffold44366_18.4	1	p3	(ACA)6	18	1943	1960	GGTTGTGTGCCATCATCAAG
scaffold44479_13.6	1	p3	(CTT)5	15	175	189	GGCACAAACTTGTGACTCCA
scaffold45283_16.6	1	p3	(TTA)5	15	188	202	TTCTCATCTCCAGTCTCCATGA
scaffold45418_17.1	1	p3	(CAA)7	21	3618	3638	AGAGGTTGGAGCAATGAGCA
scaffold45616_16.5	1	p3	(CCG)5	15	502	516	CAGTCGTCAGCCGTCACTTA
scaffold45710_12.2	1	p3	(CCA)5	15	204	218	CACCCCTATTCTTTTTGCCC
scaffold45763_16.7	1	p3	(CGC)5	15	295	309	AAGAAGATTTTTGCCACCCA
scaffold45971_10.9	1	p3	(AAT)5	15	107	121	TTCCATGACCCTCGACTTTG
scaffold46072_15.4	1	p3	(AAG)5	15	866	880	TGCTGTCTCTGGCTCTTTCTT
scaffold46129_10.0	1	p3	(TTA)6	18	49	66	TTGCCCACTCCACCACTT
scaffold46353_16.5	1	p3	(GCC)6	18	1115	1132	AATGCAGGTTGGAAAAGGTG
scaffold46716_15.8	1	p3	(CAA)6	18	2651	2668	GCTTTCGTTGCACATCTTGT
scaffold46869_13.9	1	p3	(ACA)6	18	64	81	CACATGTTGTCAAATAGATTCACTAG
scaffold46945_12.2	1	p3	(ATG)5	15	947	961	CAATCGGTCATGAGTTCGAG
scaffold47006_15.0	1	p3	(AGG)7	21	1884	1904	GCCCCAACTATTGGTGTTGT
scaffold47721_10.5	1	p3	(TGT)5	15	38	52	TGTAGCCGACCCCAATTTAT
scaffold47809_14.2	1	p3	(AAT)5	15	400	414	TCGGATTCTATTAGTGATTCAAGA
scaffold48162_11.6	1	p3	(CAT)6	18	368	385	TCAAAATCATCACTCTCCACTCA
scaffold48290_13.9	1	p3	(ATA)6	18	1993	2010	TGGCTCATTACACAAAAACG
scaffold48400_16.2	1	p3	(AAC)5	15	1566	1580	CCTTGCAGAAAAAGGCTTAGG
scaffold48497_12.7	1	p3	(TTA)5	15	265	279	TGGCAAGATTTGTTTTGACAGT
scaffold48597_10.9	1	p3	(CAA)5	15	98	112	ATTCAAGGCCAACAATCCAG
scaffold48673_14.0	1	p3	(CAT)5	15	1313	1327	TCACACATGGAGAACGAAGC
scaffold49007_14.1	1	p3	(AAT)8	24	100	123	TTGTGATGGAGTAGAGGGGC
scaffold49118_17.3	1	p3	(TAA)5	15	2258	2272	TCAGGCCTGTTTACACCCTT
scaffold49163_15.2	1	p3	(TGT)6	18	39	56	ATGTAGCCGACCCCAATTT
scaffold49196_10.8	1	p3	(CGC)6	18	336	353	TTTCCGATGTTCGATTTTCC
scaffold49741_14.8	1	p3	(CAT)5	15	361	375	CGTACATCCCCAAAGTCCTG
scaffold50181_15.0	1	p3	(CAG)5	15	2843	2857	TCAGCAGCAACTGATCCAAC
scaffold50200_13.6	1	p3	(TGA)5	15	140	154	GAGAATGTACCTTGGGTCGC
scaffold50278_12.6	1	p3	(TGA)5	15	45	59	CCCAAGACCCCAATTTGATT
scaffold50981_15.0	1	p3	(TGA)8	24	2952	2975	GACCCCAATTTGATTGGAAA
scaffold51188_16.8	1	p3	(TTA)5	15	884	898	TTGCTCGCAACGATGTTAAA
scaffold51276_12.6	1	p3	(CCG)5	15	287	301	ACGGCTGGCATTTGTTAGAG
scaffold51281_17.3	1	p3	(CAT)5	15	477	491	ACCATATGCAAACATGCGAA
scaffold51290_14.6	1	p3	(ATC)5	15	1537	1551	AAATTGGGGTTTTCCTCTGG
scaffold51751_17.2	1	p3	(ATA)5	15	2341	2355	TGCATGAAACTTTGTAAAACCA
scaffold52070_10.8	1	p3	(GAA)5	15	181	195	GATACCACGTCAAGCAACCC
scaffold52203_15.9	1	p3	(ATA)5	15	445	459	AGGATAAAAGAGGCAGGGGA
scaffold52548_14.9	1	p3	(AAC)6	18	895	912	TGTAGCACGAGTGATGAGGC
scaffold52992_10.3	1	p3	(CAG)5	15	175	189	CGATCATCCCTCATCTGACA
scaffold53144_11.2	1	p3	(ATA)5	15	86	100	CACTTTGACGCTGCTTGAAT
scaffold53154_11.3	1	p3	(TCT)6	18	142	159	CGGAGGTGATCTCAAATGGT
scaffold53238_14.3	1	p3	(CAT)5	15	90	104	CAAACCATGAATAAGCAATCCA
scaffold53460_11.3	1	p3	(TTA)5	15	73	87	TTTGCTTACGGAAACAGCAA

scaffold53553_14.5	1	p3	(CCA)7	21	350	370	TTTTCCCCCTCCTCTCATCT
scaffold53693_18.1	1	p3	(GGT)5	15	1617	1631	ATGTGTTTTCGGATGGAAGC
scaffold53828_18.4	1	p3	(GGA)6	18	1754	1771	TGCTGGTGATGCTCAAAGAC
scaffold54151_11.8	1	p3	(AGA)5	15	281	295	CCCAATTTTCCGCATTTTT
scaffold54459_15.7	1	p3	(ACA)5	15	63	77	ATGTCCTTGAAGATGCTGGC
scaffold55038_13.4	1	p3	(GCT)6	18	227	244	AAAGCATACGGGTGAAGTGG
scaffold55284_14.2	1	p3	(ACA)5	15	1537	1551	CATCTCGGGATTCAGTGGTT
scaffold56112_20.0	1	p3	(GGC)5	15	283	297	ATATTCTCGCTTCTTCGCCA
scaffold56324_11.3	1	p3	(CAT)5	15	135	149	ACGTCGCGACTCCTCTCTAA
scaffold56338_16.5	1	p3	(TGA)6	18	2306	2323	TGGAGATTCCGGAGTTCTTG
scaffold56544_15.7	1	p3	(TTG)6	18	1022	1039	TCTGAATGTGCTTTGATGGC
scaffold57252_11.8	1	p3	(GCA)5	15	45	59	TGGGCAGCCAAATATTGAGT
scaffold57542_17.4	1	p3	(AAT)7	21	776	796	TCGACTTTGTTTGGTTCGGT
scaffold57553_15.3	1	p3	(GAA)7	21	2973	2993	TGCTTGCATCCCATAAATCA
scaffold57632_17.5	1	p3	(AAT)9	27	8943	8969	TTTGTCTTTCCCTTTGATGCT
scaffold57850_15.4	1	p3	(TGT)6	18	39	56	TGTAGCCGACCCCAATTTAT
scaffold58069_16.9	1	p3	(AGC)5	15	1677	1691	CTTTCGGAGTCACAGAAGGC
scaffold58175_12.0	1	p3	(ATT)5	15	471	485	GAATGCGTCGGAAAAGTTGT
scaffold58274_12.4	1	p3	(TTG)8	24	387	410	TCCATATAGCCGACCTCACA
scaffold58279_12.6	1	p3	(AAC)8	24	577	600	CAAAATCGATCTGCAACCCT
scaffold58280_11.6	1	p3	(CAT)5	15	64	78	GCATTCGTATAAAGAAAATCGCA
scaffold58361_14.2	1	p3	(TGC)5	15	220	234	CGATCTCGTCGGGTATGAGT
scaffold58539_18.6	1	p3	(TTA)5	15	326	340	GCTTGTGATGTAGAGAAATGAAGG
scaffold58591_13.0	1	p3	(CGG)6	18	348	365	TGTCGAGGGTATAAGGGACG
scaffold58797_13.8	1	p3	(GAA)5	15	148	162	TTCGAAGGTGTTGATCCCAT
scaffold58906_16.6	1	p3	(ATG)5	15	940	954	GCACAAAATGTTGCTGCTCA
scaffold58938_14.2	1	p3	(CGG)5	15	190	204	CCAAGATTTCCGAAACCAAA
scaffold59137_11.2	1	p3	(AAG)5	15	242	256	GGTGAGTTTGAAGCATTGCATA
scaffold59182_16.1	1	p3	(GAT)6	18	2602	2619	GGCCCAAAGATCTCAAACAA
scaffold59514_16.8	1	p3	(CAT)5	15	1294	1308	ATTGTCTGCAAACCCGAAAG
scaffold59534_14.4	1	p3	(CGC)5	15	347	361	TCCTATCTATTCCGCGTGCT
scaffold60035_15.5	1	p3	(CAT)5	15	548	562	TCTCAAATATACGAAAAACGGTGA
scaffold60232_12.0	1	p3	(ATC)5	15	64	78	TCCAATTAATACAGTCAGCATTTCA
scaffold60240_11.2	1	p3	(TAT)7	21	281	301	AAGGAGCATCCTTGGTTGAA
scaffold60631_16.9	1	p3	(GTT)5	15	50	64	ACCCTTGAGCTTGCATCTGT
scaffold60646_12.1	1	p3	(GGC)6	18	53	70	GTCGGTGACGAGGTGGAGT
scaffold60981_14.0	1	p3	(AAT)5	15	466	480	TGGAACCATTGCAGTCAAGA
scaffold61087_15.5	1	p3	(TGT)5	15	50	64	TGTAGCCGACCCCAATTTAT
scaffold61124_16.7	1	p3	(TTA)5	15	506	520	TGTGTACGTAGTTGAGCATGTTG
scaffold61228_15.5	1	p3	(ATC)6	18	763	780	AATGTCACGAGTTGCAGCAG
scaffold61379_19.6	1	p3	(TAT)5	15	1347	1361	GGTGCAGGCTAAAAATGTTG
scaffold61507_14.5	1	p3	(TGT)6	18	43	60	ATGTAGCCGACCCCAATTTA
scaffold61600_13.1	1	p3	(TTA)5	15	365	379	CGATGGTTGATCTTGATTGTTG
scaffold61680_19.1	1	p3	(AAT)7	21	533	553	GATTAGCAAATTTCGGGACG
scaffold62820_11.9	1	p3	(TGG)5	15	77	91	TGATGGATCGAATATTCTCACG
scaffold63009_14.7	1	p3	(TTG)6	18	2035	2052	ACAATGTGGGAAAAATGGCT
scaffold63068_16.5	1	p3	(TTG)5	15	5738	5752	ACTTGAGCAGAATGGGCAAT
scaffold64028_10.5	1	p3	(GCT)5	15	452	466	TGGCAGTCCATTCATCTGTC
scaffold64138_10.5	1	p3	(TGA)5	15	134	148	AGCAGCCCCTCTTTTGAAAT
scaffold64143_10.8	1	p3	(AAT)5	15	369	383	GAATCTGATTTAAACATGGTGGC
scaffold64355_10.8	1	p3	(CAT)6	18	63	80	TCTTGTGAGACTGTTGTTCTGGA
scaffold64588_16.3	1	p3	(TGA)6	18	43	60	CCCAAGACCCCAATTTGATT
scaffold64589_19.3	1	p3	(TGA)6	18	43	60	CCCAAGACCCCAATTTGATT
scaffold64591_20.3	1	p3	(TGA)6	18	43	60	CCCAAGACCCCAATTTGATT
scaffold64626_10.4	1	p3	(TTA)5	15	508	522	GATGGATGAAAACCCACACC
scaffold65362_12.5	1	p3	(TGA)7	21	41	61	CCCAAGACCCCAATTTGATT
scaffold65612_11.4	1	p3	(TGT)6	18	268	285	ATGTAGCCGACCCCAATTTA
scaffold65679_14.7	1	p3	(ATC)7	21	64	84	ATGATCGGATTTTGTTGGAGA

scaffold65777_18.1	1	p3	(ATC)7	21	3098	3118	TTAAACTGGCACCCATCAAA
scaffold66407_13.4	1	p3	(ATT)5	15	1206	1220	TCTTCCCTCGTTTGTTCCAC
scaffold66509_18.6	1	p3	(CAT)5	15	63	77	TGCATAGAGGAAATTGCATAGC
scaffold66719_23.0	1	p3	(CGG)5	15	797	811	CCTATAATTGAAGCCGCCAA
scaffold66929_16.1	1	p3	(CGG)5	15	3451	3465	GGAGGAGATCGAGAGTGACG
scaffold67462_17.3	1	p3	(TCA)5	15	65	79	TTGCAAAAGTGATGTGACCG
scaffold67857_11.3	1	p3	(CAG)5	15	60	74	GGGCAGCCAAATATAGAGCA
scaffold67895_12.0	1	p3	(TGA)5	15	46	60	CCCAAGACCCCAATTTGATT
scaffold68015_14.7	1	p3	(CAT)8	24	1566	1589	CTCAACTTTCCAACTGATGGC
scaffold68071_10.9	1	p3	(GCT)5	15	101	115	AGCTGTGTCTCCAAACTCCC
scaffold68075_14.8	1	p3	(TTA)7	21	1683	1703	AGCTGAAGGAGAGGAGGAGG
scaffold68572_16.1	1	p3	(TGT)5	15	54	68	CAACGGTTTACAAGTGTTGTTG
scaffold68631_10.1	1	p3	(CGA)6	18	54	71	GCTTAAAGGTGGAGGATAGTGG
scaffold69004_15.2	1	p3	(TAT)6	18	288	305	CGTTTTTGTCAATGCGTCTG
scaffold69128_10.7	1	p3	(TGA)5	15	46	60	CCCAAGACCCCAATTTGATT
scaffold69200_12.3	1	p3	(GGT)6	18	192	209	CTCATCCCTCGCACATTTCT
scaffold69821_15.6	1	p3	(CAT)7	21	64	84	TGGGGTCTACAACAAACTGAA
scaffold69862_13.8	1	p3	(CAT)5	15	67	81	CATATCATAAAGATAAAGACAGCTTC
scaffold69961_17.0	1	p3	(TTC)5	15	66	80	CGAGCTGCATCGAGCTTT
scaffold71059_10.8	1	p3	(TGA)5	15	46	60	CCCAAGACCCCAATTTGATT
scaffold71060_10.8	1	p3	(ATC)5	15	64	78	TATCAACCCAGCAACCATCA
scaffold71063_15.5	1	p3	(TCA)5	15	65	79	CAATTAAGTTCTACGAATTCATTTTTG
scaffold71275_15.0	1	p3	(ACA)5	15	1996	2010	ATCTTTGCGGTGATTTCCTG
scaffold71474_17.2	1	p3	(GAG)5	15	344	358	ATTCGTTGATTTGGCTCCAG
scaffold72269_18.2	1	p3	(TAA)5	15	108	122	TGCAACACAATCATTATGGCT
scaffold72723_10.7	1	p3	(GCA)6	18	422	439	ATAACCCTCCCTCTGGTGCT
scaffold73439_14.3	1	p3	(ATC)6	18	270	287	GCTGAAATTTCGTCCCAAAA
scaffold74073_17.1	1	p3	(CTG)5	15	900	914	GAAATTTGGAAGTGCCTGGA
scaffold74105_15.8	1	p3	(TGG)5	15	1550	1564	TGATTTTGGATTTTAGGCGG
scaffold74279_12.2	1	p3	(AAT)6	18	64	81	AATGAATAAGTTTGCATTTAAGAGG
scaffold74361_15.0	1	p3	(AAG)5	15	471	485	GCTCGTGTCCAAATCAAACA
scaffold74425_15.8	1	p3	(CTT)6	18	5667	5684	GCAATGAACACTTAGCAGCG
scaffold74977_15.4	1	p3	(TAT)5	15	84	98	TCAGGTTTGACTTTCATTCTCG
scaffold75789_14.4	1	p3	(TTG)5	15	740	754	ACCGAAATTAACGTGGAGGA
scaffold76051_14.8	1	p3	(GTG)5	15	76	90	TCACAAACCGCTGTTGTTTC
scaffold76374_11.6	1	p3	(ATC)5	15	50	64	GCCCGAGGTTGAAGATGATA
scaffold76597_13.8	1	p3	(TCA)6	18	267	284	TGTGTTGTTTGATGTCTTTGTGA
scaffold76665_12.0	1	p3	(TTA)6	18	43	60	TTTCTTTTTCCTCAACAATTTCG
scaffold76679_16.1	1	p3	(CCG)5	15	1005	1019	TTTGGGAGTTCTCCTCCCTT
scaffold76719_18.3	1	p3	(CAT)5	15	668	682	TGTGTTTTCTTGCCCTTTCC
scaffold76983_10.2	1	p3	(TCA)5	15	65	79	TGAATATGCGGGAAGAAAGC
scaffold77149_15.6	1	p3	(GAG)5	15	1733	1747	CATGTCATGGCTGAGAGGAA
scaffold77206_15.4	1	p3	(GGC)5	15	1046	1060	TGCTGAGATGGAACTTGACG
scaffold77233_15.9	1	p3	(CCG)5	15	447	461	CACGATCACAATCAAGGTGG
scaffold77300_13.5	1	p3	(TAT)5	15	1123	1137	CCAGCCCAATAGAGGTGAAA
scaffold77510_13.1	1	p3	(TTG)8	24	1689	1712	TATGATCGTACACGATGGCG
scaffold77569_13.7	1	p3	(TCA)5	15	65	79	GCAACGAGGTGTGTGTTCAA
scaffold77653_17.0	1	p3	(CAT)5	15	859	873	TCAGAACCAGCACGAAATCA
scaffold77932_17.3	1	p3	(TGG)5	15	3344	3358	CGATTGTGGTGACGAAGATG
scaffold78015_15.9	1	p3	(TTG)8	24	526	549	ATCGTTGTGAATTTGGGGAA
scaffold78522_11.1	1	p3	(TCC)5	15	213	227	TTCGGTGCGGAGCTATTATC
scaffold78812_13.1	1	p3	(TCA)6	18	66	83	TTGGCATAACAAGCTAGGACAT
scaffold79180_11.5	1	p3	(TGT)6	18	39	56	TGTAGCCGACCCCAATTTAT
scaffold79857_16.9	1	p3	(TTC)7	21	2157	2177	CGGTGGTTTAATGGAGTTGC
scaffold79982_13.4	1	p3	(ATA)5	15	806	820	GGGCAAAAGCCAATGAAATA
scaffold80009_16.0	1	p3	(ATA)6	18	2316	2333	AACGAACGAAGCTGAGCAAT
scaffold80115_14.4	1	p3	(TGA)6	18	43	60	CCCAAGACCCCAATTTGATT
scaffold80122_12.0	1	p3	(TGT)6	18	43	60	ATGTAGCCGACCCCAATTTA

scaffold80398_11.9	1	p3	(ATA)5	15	789	803	ATGCAAAATTCGGCAGATTC
scaffold80612_12.6	1	p3	(ATC)5	15	63	77	AAATTTACTCGCTGGTCTATCTATG
scaffold80725_11.4	1	p3	(TCA)6	18	80	97	ACCTGAGGTTCAGGGTCCAT
scaffold81012_15.5	1	p3	(GCA)5	15	388	402	GATGAGATTCCCGAGGTTGA
scaffold81318_13.5	1	p3	(ATC)7	21	64	84	GAAATGCAAAGACCAAGAACAA
scaffold81500_14.8	1	p3	(ATA)6	18	694	711	TGTCAACCATAATATATGCATCGAG
scaffold81525_13.9	1	p3	(TGA)6	18	45	62	CCCAAGACCCCAATTTGATT
scaffold81673_17.5	1	p3	(TGA)5	15	810	824	CCCAAGACCCCAATTTGATT
scaffold82205_14.3	1	p3	(TCA)7	21	65	85	GCGAACACAGTTTTTCAGCC
scaffold82517_15.7	1	p3	(CAA)6	18	64	81	TCTTGAAATTTTCTTATTGTTCCC
scaffold83000_12.3	1	p3	(GAT)5	15	1632	1646	TTGTGTCAGACTTGTTCGGC
scaffold83214_11.7	1	p3	(CTT)7	21	716	736	CCCCCAAACTTATTGACAAATG
scaffold83533_12.6	1	p3	(AAG)5	15	504	518	GTCAGCGAATTGTTGCAGAA
scaffold84374_15.8	1	p3	(AAT)5	15	5001	5015	TTGCATTTTTCATAGCCCAA
scaffold84394_15.9	1	p3	(GCC)5	15	749	763	CCTCCTCAACCAGAGTCTCCT
scaffold84645_14.2	1	p3	(ATG)5	15	1977	1991	GTATTCCACCACGCTCCTTA
scaffold84849_15.6	1	p3	(TGA)6	18	46	63	CCCAAGACCCCAATTTGATT
scaffold85214_13.8	1	p3	(CTG)5	15	801	815	AAGAAGGGGGCAAAGTGAAT
scaffold85521_16.7	1	p3	(TGC)7	21	900	920	GAGAGCAAAGAGTTGGCACC
scaffold86132_14.8	1	p3	(TAT)6	18	32	49	GATGGATGGATCAGAACCGA
scaffold86415_16.5	1	p3	(TTA)5	15	2711	2725	CAATTCGTGTCATGGTCGAG
scaffold87418_11.4	1	p3	(TTA)6	18	149	166	CTTGCGGTCTTGAACATGAA
scaffold87509_10.0	1	p3	(TCT)5	15	653	667	GCAGATTCCCTTGATTTTGG
scaffold87966_14.5	1	p3	(ATT)5	15	45	59	CAGAATGATTGAAGCAAACATCA
scaffold88286_14.8	1	p3	(CAA)5	15	667	681	TGGGACGATAAGGGATAGTG
scaffold88293_17.4	1	p3	(ATA)5	15	178	192	TGAGATTTTTGGTGTCAGTGG
scaffold88414_12.5	1	p3	(AAT)6	18	239	256	CCCGGTTTGATCTGTGAACT
scaffold88416_10.0	1	p3	(AAG)5	15	134	148	CCGGTTAATGACCCATTTGT
scaffold88719_15.2	1	p3	(TAA)5	15	1512	1526	TGGAATTCAACATCAATGGTTC
scaffold88797_17.5	1	p3	(ACA)5	15	2315	2329	TCGAGATGTGAAGATTGAGGC
scaffold88946_12.6	1	p3	(CTT)5	15	223	237	ATACCTCGGCCTTTGATGTG
scaffold89061_15.8	1	p3	(TAA)5	15	1116	1130	TACGCAAAGGCACTTCTCAA
scaffold89296_12.3	1	p3	(CAG)5	15	44	58	GAAGTACCTGCCTACTCGTCTGA
scaffold89991_17.3	1	p3	(TAT)5	15	297	311	GAGGGAGGCACTAAGAAATGG
scaffold90266_13.4	1	p3	(AAG)6	18	259	276	TCAACAGTTACCCCGAAAGG
scaffold90337_18.2	1	p3	(GCC)7	21	1899	1919	CGCAATTCATCACAACTGCT
scaffold90348_15.7	1	p3	(GCT)5	15	2684	2698	CCCAGAAGCAGAAGAGGTTG
scaffold90729_12.5	1	p3	(TCT)5	15	415	429	CGAGATTGGGAAATTGGAGA
scaffold90829_16.4	1	p3	(TGT)5	15	45	59	ATGTAGCCGACCCCAATTTA
scaffold91432_12.8	1	p3	(CTT)5	15	237	251	CAGCCGAGAACATCATCAAA
scaffold91636_14.0	1	p3	(ATA)5	15	272	286	GGTGGATTAGTTGTCGTTTTGA
scaffold91781_19.5	1	p3	(GAT)6	18	35	52	TGTATATTCCTGAAGACGATGGC
scaffold91968_13.2	1	p3	(CAT)5	15	999	1013	GCATGCATAATGTGACACCC
scaffold92678_11.1	1	p3	(TAT)7	21	77	97	TGATGATTGAGTAAAAACGACAAAA
scaffold93016_16.3	1	p3	(CAT)5	15	3939	3953	ACACATGCACATTTCACGGT
scaffold93225_12.5	1	p3	(CAT)5	15	63	77	CCGTATCAAGCCACCAAGTA
scaffold93244_12.9	1	p3	(GAG)5	15	389	403	TCGAGGAAGGAGAGGTGTTG
scaffold93468_14.2	1	p3	(TGA)5	15	48	62	CCCAAGACCCCAATTTGATT
scaffold94449_14.9	1	p3	(AGA)8	24	734	757	TCTTTGCGATGAGCTCCTTT
scaffold94746_13.7	1	p3	(CCA)5	15	246	260	TTATCCCATCCCCCTTAACC
scaffold95065_16.0	1	p3	(CAT)7	21	64	84	ATAAACTGTTACTCCTGGGTGC
scaffold95204_17.6	1	p3	(ATT)6	18	2054	2071	CTGCCACCGATTTGAACATT
scaffold95447_11.1	1	p3	(TTA)5	15	411	425	GACGCTTGGTTGGATAGCTT
scaffold95703_14.2	1	p3	(AAG)5	15	3336	3350	CCGCCTCCGTAAATAAACAA
scaffold95841_15.2	1	p3	(TGT)5	15	203	217	GATTCGCTACCGTGTGGAGT
scaffold96180_15.5	1	p3	(TGA)5	15	56	70	CGGACAGAAGGATGGCTTTA
scaffold96335_17.1	1	p3	(TCA)5	15	65	79	TTGTTTTATTGTGTCTTTGTCATTGT
scaffold96336_14.1	1	p3	(TCA)5	15	65	79	CATAAACCACTTCGAACGGC

scaffold96721_13.6	1	p3	(TGT)7	21	42	62	ATGTAGCCGACCCCAATTTA
scaffold96772_13.9	1	p3	(TTA)5	15	483	497	GCTCGATGGAAAGCAGAAAA
scaffold96914_15.8	1	p3	(AAT)5	15	657	671	TGCCACAACTTAGAATCGCA
scaffold96984_13.8	1	p3	(TAA)5	15	398	412	TGATGTTAAGAAACATTAAAAAGGG
scaffold97121_10.4	1	p3	(AAT)7	21	367	387	GCCAAAAGATACTAAACTTCCAAA
scaffold98102_16.3	1	p3	(CAC)5	15	4818	4832	AATGGAAAAGCATGTCAGGG
scaffold98266_17.6	1	p3	(ATG)5	15	117	131	CCAATGTGCTGCTTATGAGG
scaffold98421_14.9	1	p3	(GGA)5	15	901	915	GAAAACGGAATCGAAGGTGA
scaffold98706_10.7	1	p3	(CGG)6	18	197	214	GAAGGATGAGCGAAATGAGG
scaffold98827_16.5	1	p3	(GCC)7	21	8105	8125	AGTGTGAAGGCCATGATTCC
scaffold98865_16.1	1	p3	(GGC)5	15	142	156	TTCAGCCCAGCCCAATAATA
scaffold99241_17.2	1	p3	(ATC)6	18	722	739	CTAGGGCTAGCAGCTCCAGA
scaffold99256_11.4	1	p3	(TAT)7	21	90	110	GGGCCGATCCTATCACTACA
scaffold99551_10.5	1	p3	(CAG)6	18	94	111	ATTCCCGAGACTGAGATGGA
scaffold99744_15.0	1	p3	(TTC)5	15	554	568	TCTGGTGGAGTTCATTGAGATG
scaffold99800_10.3	1	p3	(AGA)5	15	56	70	TTCTCTCTGTGCTGATCGAAA
scaffold100302_14.7	1	p3	(GAT)5	15	4695	4709	GAAATGTAACAGCCTCCCCA
scaffold100451_13.7	1	p3	(TGA)5	15	47	61	CCCAAGACCCCAATTTGATT
scaffold101371_19.6	1	p3	(CGC)5	15	1428	1442	CTATAGGCACGCCGAACAAT
scaffold101828_15.9	1	p3	(TGT)5	15	48	62	ATGTAGCCGACCCCAATTTA
scaffold102165_10.9	1	p3	(CAT)5	15	65	79	CCCTTTAAGATAAAAATGAATATTGG
scaffold102526_13.1	1	p3	(ATC)5	15	45	59	GCCCGAGGTTGAAGATGATA
scaffold102969_17.3	1	p3	(ATC)5	15	1337	1351	CAATCCCAATTGTATCCGCT
scaffold103057_14.9	1	p3	(ATC)5	15	60	74	CCAACTCATTTTGCCCACTAA
scaffold103106_16.4	1	p3	(TAA)5	15	646	660	GATTAGCAAATTTCGGGACG
scaffold103110_12.8	1	p3	(AAT)5	15	351	365	ATTTGGGAGTGTTCGGACTG
scaffold103198_13.3	1	p3	(AAT)5	15	101	115	AAAAATCAGACCAAAACTTTTCAA
scaffold103594_16.6	1	p3	(TAA)7	21	1150	1170	TGGAGCTAGTGAGGGTGGTT
scaffold103833_16.8	1	p3	(CGG)7	21	595	615	CTGACCTCGTGTAACTGGCA
scaffold104959_14.9	1	p3	(ATG)5	15	998	1012	TTCCATGGGTACAGAGGAGC
scaffold104971_16.9	1	p3	(TTA)5	15	233	247	GGAAGATGAAAAGACAAGCGG
scaffold105270_11.2	1	p3	(TCA)6	18	65	82	TGAAGAATTATGCATCAGGCAA
scaffold105354_13.1	1	p3	(ACA)6	18	1165	1182	AGCAGCTACAACGGTAAGGC
scaffold105850_14.4	1	p3	(CTG)6	18	1219	1236	CCAGCAGTGCACTCGTAAAA
scaffold106554_15.6	1	p3	(GCG)5	15	1004	1018	TGGTTATGGAGGAGACGGAG
scaffold106916_15.0	1	p3	(ATT)5	15	2700	2714	GAGTGGATAGGCTCCGTTTG
scaffold106951_12.7	1	p3	(AGA)5	15	314	328	TCCCAAGCATGGAATCAAAT
scaffold107049_10.3	1	p3	(ACC)6	18	178	195	ATTCACACCTGCTCCAGTCC
scaffold107966_14.1	1	p3	(TAA)8	24	1475	1498	TGGGATCTATTTGATGAGGGA
scaffold108012_13.0	1	p3	(CTT)5	15	577	591	TGGGTTGTTAAGCCTTGGTC
scaffold108125_14.9	1	p3	(AAG)5	15	392	406	TTATTTCCGCATCTTTTGGG
scaffold108237_16.1	1	p3	(ACA)5	15	3230	3244	ATTTGGCATCAACCTCCAGA
scaffold108252_16.0	1	p3	(CGC)5	15	774	788	CTTGATCGGGTCCGACTTTA
scaffold108350_14.6	1	p3	(GGA)5	15	2788	2802	CCTCCGGAACTCTCCTAACC
scaffold108594_15.1	1	p3	(CCG)5	15	204	218	AAATTAAATAACCTCGCCGGA
scaffold108742_14.5	1	p3	(AAT)5	15	1872	1886	GGCTCGCCAAGGTAGATAAA
scaffold108933_14.4	1	p3	(TGC)5	15	634	648	GAAATGAAGCCAAACCCTCA
scaffold108955_19.3	1	p3	(AAG)5	15	1052	1066	CGCACACTTGGAGTTGAAAA
scaffold109303_19.5	1	p3	(AGC)5	15	49	63	TTGTGGAGAAAGGAATGGGA
scaffold110026_10.2	1	p3	(ATC)5	15	82	96	CCATGTAAGAAAAGTGGAAGGG
scaffold110229_12.0	1	p3	(GGC)5	15	207	221	CAAAATTAAAAATGCCCCCA
scaffold110992_16.6	1	p3	(AAC)6	18	384	401	TGAAGGATGGGTAGAATCGG
scaffold111120_13.1	1	p3	(TAG)7	21	870	890	CTCATTGAACTTGGCAAGCA
scaffold111374_15.2	1	p3	(TCG)5	15	577	591	CATGAGGCTGCTGAACTCAC
scaffold112284_16.9	1	p3	(TGT)6	18	42	59	TGTAGCCGACCCCAATTTAT
scaffold112656_14.8	1	p3	(TAA)5	15	441	455	CGGGGTTCGAGTTCATATTG
scaffold113320_16.1	1	p3	(GGC)5	15	790	804	TGCTGCCACACTCTCTCTGT
scaffold113395_12.9	1	p3	(TCA)6	18	1028	1045	AACATCATCCCCATCACCAT

scaffold113495_13.[illegible]	1	p3	(AAT)5	15	69	83	TGGTTGAGCTAAAATCTTTGAGAA
scaffold113771_14.[illegible]	1	p3	(GAG)5	15	3007	3021	TGGTGGTTTTTGGATGATGA
scaffold114539_16.[illegible]	1	p3	(ATA)5	15	498	512	TGCTAAACGATGAGCTTGGA
scaffold115034_17.[illegible]	1	p3	(AAT)6	18	403	420	CGTCGTCGCTACATGAAGAA
scaffold115356_17.[illegible]	1	p3	(GAG)5	15	1275	1289	GCCGTAGAGTAGATCGGTGG
scaffold115775_15.[illegible]	1	p3	(TGA)5	15	47	61	CCCAAGACCCCAATTTGATT
scaffold116107_13.[illegible]	1	p3	(CAA)8	24	2140	2163	CAAATGCGAAACCGATCTTT
scaffold117130_14.[illegible]	1	p3	(ATT)5	15	1205	1219	CGTGGAGCCTATTTGGATGT
scaffold117230_15.[illegible]	1	p3	(GAC)5	15	82	96	CAAAAACCCCGAATTCATTG
scaffold117330_15.[illegible]	1	p3	(GAA)5	15	307	321	TATGCCCGTGAAAAAGAAGG
scaffold117477_15.[illegible]	1	p3	(GGC)7	21	306	326	AAATGGAGTCGAAGCAGCAT
scaffold117583_15.[illegible]	1	p3	(CAT)5	15	57	71	GGCGTCTCAAACGTATCTCC
scaffold117956_17.[illegible]	1	p3	(GCA)5	15	1272	1286	TTTGAAGCAAAAGTTTGGGC
scaffold117988_16.[illegible]	1	p3	(CAT)6	18	971	988	TGTGGCTGTTCATTCTTGGA
scaffold118044_10.[illegible]	1	p3	(TGT)6	18	41	58	TGTAGCCGACCCCAATTTAT
scaffold118564_16.[illegible]	1	p3	(GCG)5	15	399	413	GTGGTAGAGGCGGTTATGGA
scaffold118788_15.[illegible]	1	p3	(ATA)5	15	673	687	TTTTCAATCATATAGAATCTGCTCTTG
scaffold120841_11.[illegible]	1	p3	(ACA)5	15	434	448	AATATGAGAAGAGCAAACCCACA
scaffold121154_11.[illegible]	1	p3	(AAT)6	18	121	138	TCAACGTGATTTGTCATTTGTG
scaffold121498_12.[illegible]	1	p3	(GAG)6	18	265	282	TCATGGATTTAGACTGCCCC
scaffold122425_10.[illegible]	1	p3	(AAT)5	15	382	396	TAAAGTGACCAGTTTGCCCC
scaffold122659_16.[illegible]	1	p3	(GGC)5	15	2221	2235	TTTTGGGGGTTGAAATGGTA
scaffold122741_16.[illegible]	1	p3	(CAC)5	15	379	393	TGAAACTCCCCACAATCTCC
scaffold122902_13.[illegible]	1	p3	(TTA)7	21	895	915	ACACACGCATACACACACGA
scaffold122929_17.[illegible]	1	p3	(ATC)5	15	2314	2328	TGTGTGAAACCTCCTTCATGG
scaffold123274_10.[illegible]	1	p3	(AAT)6	18	561	578	TTGCCTCTAATTTTGGCTCC
scaffold123912_13.[illegible]	1	p3	(GTG)5	15	49	63	TCTGGTCAACATCAATCTGAGC
scaffold124017_16.[illegible]	1	p3	(TAA)5	15	615	629	CGTTGGCCAGTCGGTTAAT
scaffold124181_11.[illegible]	1	p3	(ACA)6	18	479	496	AATTGCAAAAGCGCAAAAAT
scaffold124338_14.[illegible]	1	p3	(CCG)5	15	301	315	ACCATTCATCCATAGCGGAG
scaffold124641_14.[illegible]	1	p3	(CAA)6	18	66	83	TCCAGCCTTTACCTCTTTTTG
scaffold125218_15.[illegible]	1	p3	(TGA)5	15	50	64	CCCAAGACCCCAATTTGATT
scaffold125899_14.[illegible]	1	p3	(ATC)7	21	470	490	CCCAAGAATGACTGGGGTAA
scaffold126032_12.[illegible]	1	p3	(ATT)7	21	224	244	CTCTATCACAACCCAAGGCG
scaffold126241_13.[illegible]	1	p3	(TGT)9	27	38	64	TGTAGCCGACCCCAATTTAT
scaffold126926_15.[illegible]	1	p3	(TAG)5	15	690	704	GGTTCGTCTTCAACCACGAT
scaffold126957_16.[illegible]	1	p3	(ATT)6	18	318	335	TAGCAGGGCATGAACAAACA
scaffold127101_14.[illegible]	1	p3	(ATA)7	21	1625	1645	GAATGGCATTTTAACTTTGATCATT
scaffold127442_12.[illegible]	1	p3	(TGT)7	21	39	59	TGTAGCCGACCCCAATTTAT
scaffold128061_13.[illegible]	1	p3	(TCT)5	15	113	127	CAGCCGAGAACATCATCAAA
scaffold128316_17.[illegible]	1	p3	(TAT)5	15	672	686	GCATGAAACTTGGAATGGAAA
scaffold129273_17.[illegible]	1	p3	(GGC)5	15	3353	3367	AGATTTGGCCGTGTACGAAG
scaffold129468_14.[illegible]	1	p3	(TAA)5	15	3946	3960	ATAAAATGGGAGCGGAGAGG
scaffold129614_13.[illegible]	1	p3	(AAG)5	15	174	188	ATGGCATGAAACGGAATAGC
scaffold129760_16.[illegible]	1	p3	(CGC)5	15	509	523	GCCGGACATCTATACCAGGA
scaffold129887_17.[illegible]	1	p3	(CAT)5	15	163	177	TCTTGGGTTCTACCTTGGACA
scaffold130032_17.[illegible]	1	p3	(AAT)6	18	1954	1971	TCTTGTTGTCGCGTTTAATGA
scaffold130071_12.[illegible]	1	p3	(CGG)5	15	533	547	TGCATGAAATCAAACTCCCA
scaffold130338_18.[illegible]	1	p3	(CAT)5	15	67	81	AAAAACCAACTGAATTGCAAAA
scaffold130503_15.[illegible]	1	p3	(AAT)6	18	189	206	CCGATTCGTGGAAACCTGTA
scaffold130545_13.[illegible]	1	p3	(GAA)6	18	436	453	TTAAGACGGAAATCCACACG
scaffold130677_13.[illegible]	1	p3	(TTA)6	18	203	220	GACAGGGGTCTCGCAAGATA
scaffold130768_10.[illegible]	1	p3	(TTA)5	15	409	423	ATCGTATTCCACCGGATCAA
scaffold130853_17.[illegible]	1	p3	(TTA)5	15	354	368	TGTTTGTTGTGTGTCTCCCA
scaffold130999_11.[illegible]	1	p3	(GAA)5	15	195	209	TCAAAACTCGGTCTTGCACA
scaffold131057_13.[illegible]	1	p3	(GAT)5	15	22	36	AGTCCGTGTTCCGATGAGT
scaffold131383_12.[illegible]	1	p3	(TCA)6	18	257	274	CTTGTACCACTGGCCTCCAT
scaffold131514_12.[illegible]	1	p3	(TAA)6	18	414	431	GCAACCTGAAAATTGAACATTG

scaffold131570_12.8	1	p3	(CTT)5	15	156	170	CAGGTCAACTGAAATCTGAAAGAA
scaffold132139_13.6	1	p3	(AAT)5	15	888	902	TCCATATTCCCTTCGTCGTC
scaffold132189_14.5	1	p3	(TTC)5	15	632	646	ACGAGAACCCTCACATCACC
scaffold132972_18.5	1	p3	(TGA)5	15	217	231	ATTGGTGCAAAATGTGTTGA
scaffold133008_14.7	1	p3	(GAT)5	15	39	53	TACAGTCCGTGTTCCGATGA
scaffold133080_14.1	1	p3	(CGC)5	15	347	361	TCCCACATAGGAAAAACAAAAA
scaffold134661_14.4	1	p3	(ATA)6	18	562	579	CCTTGATCATTCCCGAAAAA
scaffold134875_18.5	1	p3	(TTG)5	15	675	689	GTCTTGAATGCCACCCACTC
scaffold134996_16.0	1	p3	(TTG)6	18	530	547	TGGTTCATATAACCGACCCC
scaffold135665_17.2	1	p3	(ATC)5	15	2784	2798	TTCCCACTAATTTCACCTGGA
scaffold135855_15.6	1	p3	(CAT)5	15	1712	1726	TACCTTGGGCTCCACCTCTA
scaffold135861_17.6	1	p3	(TAA)5	15	2190	2204	TTGTGCATATTGTTGGGGAA
scaffold135922_14.2	1	p3	(GAC)5	15	1258	1272	CGAGTGGCTGCTACCTGAAT
scaffold136436_12.7	1	p3	(GCA)6	18	349	366	TTGATGAGCCATAACCCTCC
scaffold136732_16.7	1	p3	(ATT)5	15	586	600	TCCTTGTCACAATCTGCTGC
scaffold137401_11.3	1	p3	(ATT)8	24	1146	1169	TGGTTTGACCATCATTGCTC
scaffold137593_17.4	1	p3	(ATG)5	15	477	491	TGGATGGGTGCACATAATTG
scaffold138160_12.8	1	p3	(AAT)6	18	182	199	TTGGGCAACTTGAAGATTAAAA
scaffold138190_14.3	1	p3	(CTT)6	18	110	127	CCCCCAAACTTATTGACAAATG
scaffold138293_12.2	1	p3	(TTA)6	18	200	217	CGGGAGAGAAAATGCAGAAG
scaffold138380_17.3	1	p3	(GCC)5	15	1092	1106	TGCAGCAGCCAAGACTTAGA
scaffold138473_16.5	1	p3	(ATT)5	15	287	301	TCCAATCCATAGCAACCAAA
scaffold138946_12.5	1	p3	(TAT)5	15	1049	1063	GTAACCATGGGCCAAAAATG
scaffold139070_14.1	1	p3	(CAA)6	18	66	83	TCTGTCGACTGAAATGCAAA
scaffold139657_16.8	1	p3	(CTC)5	15	725	739	CCAAATGTGATTGCCCTTTT
scaffold139767_14.8	1	p3	(CTC)5	15	1633	1647	CAGTACGACCAGGGAAAAGC
scaffold140269_10.6	1	p3	(TTA)6	18	248	265	CGTACGGAAATCTGATAGGAAAA
scaffold140486_16.1	1	p3	(ATC)6	18	64	81	CCAAATTTGTAAATGCAAACAAAC
scaffold140839_13.9	1	p3	(TGT)6	18	2223	2240	CCAATGGGTGAAAGAATATGG
scaffold140896_14.4	1	p3	(TGT)6	18	47	64	TGTAGCCGACCCCAATTTAT
scaffold141213_10.3	1	p3	(ATA)5	15	177	191	TACTCCCTCCGTCTCACTCC
scaffold141449_17.1	1	p3	(CAG)5	15	1468	1482	CATTTGTGCCAGATCCATTG
scaffold142004_15.7	1	p3	(ATT)5	15	6987	7001	CCCTGTTGAGCATACACCCT
scaffold142275_11.5	1	p3	(AGA)5	15	103	117	TCAGTTCCGTCTAGGCTTCAA
scaffold142836_11.3	1	p3	(AAT)5	15	172	186	ATCAGAGCCAGCGTTTGAGT
scaffold142847_17.2	1	p3	(AAG)5	15	998	1012	AGGTCATGTGTTCGAGTTTCAA
scaffold142901_15.8	1	p3	(ACA)5	15	549	563	TGTATTGAATCCGGTGGGTT
scaffold143093_12.5	1	p3	(GCG)5	15	305	319	CCTTTTATACGGGTCGGGTT
scaffold143192_11.6	1	p3	(TTA)5	15	609	623	CGGAGATTCGCAAGTTCAGT
scaffold143399_17.4	1	p3	(CTT)5	15	110	124	CGTTGGCTCGACGTTATCTT
scaffold143454_13.4	1	p3	(GAT)5	15	213	227	GGTGGCGGTAGTTTCAAAGA
scaffold143463_17.4	1	p3	(GAT)7	21	1520	1540	GCAACGGAAACAACCTCTTT
scaffold143491_11.0	1	p3	(TTC)5	15	180	194	CTGGCCTGATCTACAGCCTC
scaffold143500_10.2	1	p3	(CCG)5	15	144	158	TCTGCTCAGCCCCTTCAG
scaffold143579_14.6	1	p3	(TTG)6	18	357	374	GCTGTAGCTGACCCCAATTT
scaffold143952_14.9	1	p3	(TTA)7	21	8338	8358	GATGCGTTTGAAGGGAAAAA
scaffold144015_15.0	1	p3	(TGA)6	18	1162	1179	CCGAAGAGCATAGTTCAGCC
scaffold144406_13.0	1	p3	(ACC)5	15	653	667	ATAAGGATTCCGGGTTTTCG
scaffold144410_11.6	1	p3	(CGC)5	15	146	160	GGTTATTTATTAAAGTTGGAGGTGGA
scaffold144522_12.9	1	p3	(ATT)5	15	37	51	AAAATTGCGTCTCCTATGGC
scaffold144670_17.5	1	p3	(CAT)5	15	2538	2552	ACCTATGCATGTTGCCGTC
scaffold145156_10.4	1	p3	(GGC)5	15	876	890	CACAACGGTCATCTGCTTTG
scaffold145660_15.8	1	p3	(TTA)5	15	1313	1327	TCAGTCACAAGAGCACCCAC
scaffold146323_14.3	1	p3	(TGA)5	15	91	105	TTTGATTGGGAATAATTGGG
scaffold146343_12.1	1	p3	(ATT)7	21	230	250	CCCAACCCTAACCCGTTTAT
scaffold146883_14.8	1	p3	(TCA)5	15	113	127	TCGCATCTGGGATAATGAAA
scaffold147220_16.5	1	p3	(TGA)5	15	138	152	CAATTTGATTGGGAATATATTGGG
scaffold147347_15.4	1	p3	(ATT)6	18	1108	1125	CCACACCCCTTTTGACTGAT

scaffold147453_17.[illegible]	1	p3	(TTA)5	15	500	514	GGCATGTATTTATTATTGGACCAT
scaffold148028_17.[illegible]	1	p3	(ACA)5	15	5993	6007	CCTCGTGCAAGCATTACAAA
scaffold148504_11.[illegible]	1	p3	(TAA)6	18	311	328	TTCAAGGTTCTCGCTCTAATCC
scaffold148855_15.[illegible]	1	p3	(TCT)5	15	148	162	GCATTATGTAAGCTCAAATAGGCT
scaffold148911_16.[illegible]	1	p3	(TAA)6	18	133	150	CTCAAGATGCGAAGCAAACA
scaffold149734_15.[illegible]	1	p3	(GTG)6	18	2399	2416	AAGTCACGAAGACGGCAAGT
scaffold150286_14.[illegible]	1	p3	(TAT)5	15	59	73	TCGCTTTGATTATTAAATCTTCTTGA
scaffold150918_15.[illegible]	1	p3	(AAG)5	15	526	540	GAAAGAAGCTCCCGAAGGAC
scaffold151048_15.[illegible]	1	p3	(TGA)5	15	202	216	CCAAGACCCCATTTTGATTG
scaffold151259_17.[illegible]	1	p3	(TCA)5	15	29	43	CACCGGTTGGACCGTCTT
scaffold152047_12.[illegible]	1	p3	(ATG)5	15	39	53	TCCTGATGGAGGTGATGATG
scaffold153681_10.[illegible]	1	p3	(CAA)5	15	224	238	ATGTGTTGGTTGCAGGTTCA
scaffold153738_14.[illegible]	1	p3	(AGT)5	15	667	681	CAGTTGACACTTGGGGGACT
scaffold153827_13.[illegible]	1	p3	(CGG)7	21	1343	1363	CCATGCTAATTTCAGGGGAA
scaffold154407_15.[illegible]	1	p3	(GCA)5	15	161	175	CAGAGGATGAAATTCCCGAG
scaffold154628_14.[illegible]	1	p3	(TCT)5	15	2125	2139	TGTGGAACCAACACGATTTT
scaffold154673_11.[illegible]	1	p3	(CCT)5	15	115	129	GGGCTTCTTGTTGGAATTGA
scaffold154678_14.[illegible]	1	p3	(AAG)5	15	678	692	CGGATCTTACGGAGTTGAGC
scaffold154855_11.[illegible]	1	p3	(TCA)5	15	65	79	ATAGTGCAAAGTTGGTGCCC
scaffold155754_12.[illegible]	1	p3	(CCG)5	15	450	464	CCCCAAGCTATTCTCCAACA
scaffold155810_11.[illegible]	1	p3	(TAT)5	15	475	489	GTTTGGAGGTCAGGTGTTCG
scaffold155880_10.[illegible]	1	p3	(AAT)5	15	263	277	CCTTGTGCAAATTAATCCCG
scaffold155986_16.[illegible]	1	p3	(CGG)5	15	724	738	TAGGGAAAAGTCATCACGGC
scaffold156571_10.[illegible]	1	p3	(GCT)5	15	191	205	TTGAATATCGGCGAAGTGCT
scaffold156646_16.[illegible]	1	p3	(TGT)6	18	43	60	ATGTAGCCGACCCCAATTTA
scaffold157088_14.[illegible]	1	p3	(ACA)6	18	2032	2049	ACATCCATCATCATCAGCCA
scaffold157281_14.[illegible]	1	p3	(ATC)6	18	206	223	CTTCACATCAACACCCGATG
scaffold157353_16.[illegible]	1	p3	(AAT)5	15	1340	1354	ATTATTGAATGGGCCCAAAA
scaffold157360_12.[illegible]	1	p3	(CGC)5	15	65	79	TGGCTATCCTCAGCTGCTCT
scaffold157595_13.[illegible]	1	p3	(GGC)5	15	6078	6092	TATATGTGGCCTTCTTCCCG
scaffold158421_16.[illegible]	1	p3	(CTT)5	15	619	633	CACTGGACACACCTCTCCCT
scaffold158649_10.[illegible]	1	p3	(GAT)5	15	435	449	CATTGGCAGCAGCAAAACTA
scaffold158758_18.[illegible]	1	p3	(TAT)7	21	727	747	TAGCTCTGTTGCAGCCGATA
scaffold158879_10.[illegible]	1	p3	(ATC)5	15	69	83	AAGCCAATTTTGGTGGGTTT
scaffold159071_14.[illegible]	1	p3	(ACA)6	18	2584	2601	TCCAATCACTTACCAAATGCAC
scaffold159673_11.[illegible]	1	p3	(CCG)6	18	45	62	AATTACCTTGCCGGAAATTG
scaffold160644_15.[illegible]	1	p3	(TCA)5	15	2340	2354	CTTTCCCCAGCATCAACATT
scaffold161086_13.[illegible]	1	p3	(CCA)5	15	961	975	AGAACTCGAGCTTGACCCAC
scaffold161700_15.[illegible]	1	p3	(TCT)5	15	107	121	TCAGAAGGGGTTATGTCCTCA
scaffold161708_13.[illegible]	1	p3	(ATC)6	18	65	82	CAAGTTCTTTCAAATTGGTGGA
scaffold161765_17.[illegible]	1	p3	(CAG)5	15	1295	1309	CACATGGCTCAGACGACACT
scaffold162708_16.[illegible]	1	p3	(TTG)6	18	135	152	GCCAACCCTCCTCCTTTATC
scaffold163455_20.[illegible]	1	p3	(CAA)5	15	311	325	GGTACAACATCATGCCACCA
scaffold163819_16.[illegible]	1	p3	(AGT)6	18	1929	1946	AACCTCTGCCGTGAAATCTG
scaffold163874_11.[illegible]	1	p3	(TTG)6	18	64	81	TGACCCTAGTGGTAACACGG
scaffold164227_10.[illegible]	1	p3	(CAA)5	15	39	53	CAGCCATGAATGAGAGTGGG
scaffold164578_11.[illegible]	1	p3	(CCG)6	18	147	164	CTGTTCTCGCTTACCAAGCC
scaffold164722_10.[illegible]	1	p3	(CCG)5	15	90	104	ATAAAACCTGCCGGAATCGT
scaffold165540_14.[illegible]	1	p3	(TCT)5	15	1489	1503	TGGAGATCGGAAAATTGGAG
scaffold165855_16.[illegible]	1	p3	(CTC)5	15	4658	4672	CCAAAATGGGGAGATGAGTG
scaffold165914_14.[illegible]	1	p3	(ACC)5	15	503	517	GGGGGCCAAACAAATATACA
scaffold166272_17.[illegible]	1	p3	(TCT)5	15	1435	1449	TGTACTTGTTCGTGTTGCCC
scaffold166432_16.[illegible]	1	p3	(ATA)5	15	273	287	ATGGGTTCGAGTCTCAATGG
scaffold167500_17.[illegible]	1	p3	(ATC)7	21	3474	3494	GCATTATGTCATGGTTCCCC
scaffold167676_16.[illegible]	1	p3	(AAT)5	15	773	787	TGCAAATGAAAAAGGGGTAGA
scaffold168439_14.[illegible]	1	p3	(CAA)5	15	370	384	TATGGTAGCTGGCTCGACCT
scaffold168792_15.[illegible]	1	p3	(AAT)5	15	276	290	TCTCGCCAACTTCCTCTGAT
scaffold169730_12.[illegible]	1	p3	(CCA)5	15	93	107	GCCCGAATTTTCCCTAACTC

scaffold169924_13.(	1	p3	(ACA)6	18	424	441	GTGGGCACCCAACAAAATTA
scaffold170018_19.!	1	p3	(ATC)5	15	136	150	CGTCGGGTAGACCAATGACT
scaffold170983_10.8	1	p3	(CAA)5	15	170	184	AGAAATACATGCAGGGCTGAG
scaffold171005_15.6	1	p3	(TGT)5	15	713	727	ACCAAGGGTGCGAGAATATG
scaffold171064_12.7	1	p3	(CCG)6	18	312	329	TCCTCTTGGGTTGTTTCACC
scaffold171088_16.3	1	p3	(TCA)6	18	174	191	AGAAAAAGAAGAGGGAGCGG
scaffold171367_19.2	1	p3	(TCA)6	18	261	278	CTTGCCAATGCTCCACACTA
scaffold171500_15.(	1	p3	(CAT)5	15	39	53	ATCATCATCATGTCCACCGA
scaffold171588_12.7	1	p3	(GCG)5	15	575	589	AGGAGGAGGAAGAGGAGTGC
scaffold171913_16.3	1	p3	(AGG)5	15	576	590	GCTTTGCAACACACATTTGG
scaffold172165_14.4	1	p3	(GCC)6	18	734	751	ATAAATCGAACCTCCCCACC
scaffold172672_18.9	1	p3	(CAA)5	15	657	671	TGCAGCAATTATGCAACCAT
scaffold173246_15.2	1	p3	(TTA)5	15	582	596	AGTCAGAAAAGCCCACCTCA
scaffold173742_17.2	1	p3	(TCA)5	15	169	183	TAAGGCCGTATTAACCGTCG
scaffold173758_14.6	1	p3	(TCT)5	15	36	50	GCTACATCCAATGCTGGTGA
scaffold174008_14.4	1	p3	(TAT)6	18	1008	1025	CCGATATTTTGGCCTTCGTA
scaffold174265_14.6	1	p3	(AAC)6	18	1414	1431	AATGTCCCCATTGAGACTCG
scaffold174627_17.3	1	p3	(CAA)6	18	634	651	CGATTTTGCCCAAGAATACC
scaffold174841_17.(	1	p3	(GGC)5	15	3859	3873	ACCAACTTCACCGATCCAAG
scaffold175543_16.3	1	p3	(AAC)7	21	7296	7316	GTTTGGTGGATCAAGCACCT
scaffold175822_13.3	1	p3	(TCA)5	15	249	263	GGTGGTCGAGTTGGTTGAGT
scaffold177700_13.4	1	p3	(CAT)6	18	778	795	TATTGTGGCACTCTCCCCTC
scaffold178448_16.8	1	p3	(TGC)6	18	1070	1087	TGGAAGATGGAAGAGGATGG
scaffold178466_16.(	1	p3	(TCA)6	18	367	384	CACCTCAGCCACCCTAGAAG
scaffold179974_16.3	1	p3	(TTC)6	18	243	260	GATCGTGTTCGTGTCGTGTC
scaffold180284_13.8	1	p3	(TGA)5	15	107	121	ATGAGTCGGTTTGTTGAGGC
scaffold180318_11.2	1	p3	(CCA)5	15	282	296	GAAAGACCACGGCGATTTTA
scaffold180842_13.6	1	p3	(ATA)5	15	306	320	ACAAATTCCCGTTCCATCAA
scaffold180990_11.!	1	p3	(ATC)6	18	139	156	ACCTGTATTAGTACGGCGGC
scaffold181055_10.8	1	p3	(ATT)6	18	590	607	ACATGCAAATCCATTACCCA
scaffold181231_14.9	1	p3	(TGT)6	18	358	375	AACGTGGAGGACAGTGGTCT
scaffold181269_14.(	1	p3	(ATT)5	15	229	243	ATTTCCCAACACTCACTCGC
scaffold181536_11.8	1	p3	(TTA)5	15	58	72	CAGAAGATTCATATTGATTTTGAGG
scaffold181738_15.!	1	p3	(ATT)6	18	834	851	AAGGCGGTGTATTATGTCGG
scaffold182831_16.1	1	p3	(AAC)5	15	367	381	TGATCTCTCCCGTGAAAACAT
scaffold183025_18.!	1	p3	(CAA)5	15	1014	1028	CCTTTTCGGGTGCTTTACAA
scaffold183197_18.9	1	p3	(AGG)6	18	246	263	ATGCAGGATGAGGAGGAATG
scaffold183546_13.9	1	p3	(TCA)5	15	116	130	TTTCCAACAATAACCCCGTG
scaffold183680_16.!	1	p3	(AAG)5	15	36	50	CTCGCTGGATCCCTAGAAGC
scaffold184885_11.3	1	p3	(ATT)6	18	38	55	TTGAAAACCGATTTCCCTT
scaffold184963_10.2	1	p3	(TTC)5	15	284	298	TTCCAAGCATTTGATGCAAG
scaffold185898_16.!	1	p3	(TTA)5	15	1209	1223	TGAGGTTCCAATTATATCCTTATGC
scaffold186381_11.9	1	p3	(TTA)7	21	166	186	TGTGATTTGGATGATGTGTAAGC
scaffold186842_16.(	1	p3	(AAC)6	18	3267	3284	TTTTTGAATGGCCGTTTACC
scaffold186906_10.!	1	p3	(TAT)5	15	96	110	TTGTCAAGATTGGCTTCGTG
scaffold187133_12.7	1	p3	(TAA)5	15	543	557	TCGGCTTTTGTTACATGCTTT
scaffold187166_15.!	1	p3	(CAT)5	15	35	49	AAACATGCCCAACATTCAGC
scaffold187551_14.4	1	p3	(AAT)6	18	932	949	CTTCCAAAATAGGGGGTTCC
scaffold187703_10.!	1	p3	(AAT)6	18	37	54	TTTTGAATTAAATGGTGAGAAATGA
scaffold187795_14.(	1	p3	(TTA)5	15	242	256	GGGTTTATGTGTTTATCACGTTG
scaffold188397_14.4	1	p3	(AAT)7	21	734	754	CCGGTATCTTACTTTTGCCG
scaffold188707_14.4	1	p3	(TAA)5	15	134	148	TCTCCCTACTCTTTCGTTGCTC
scaffold189167_15.4	1	p3	(CGC)5	15	1236	1250	GCCAACTTTGCTTGCATTTT
scaffold189187_10.!	1	p3	(TAA)5	15	343	357	AAATGTATCCGCCGTGATTG
scaffold189617_16.6	1	p3	(TAT)6	18	91	108	TTTGAAGATCCGGAAATTTGT
scaffold190073_14.(	1	p3	(GAA)5	15	34	48	GGATCGTAAAGCCTGGTGAA
scaffold190114_13.!	1	p3	(AAT)6	18	222	239	GACGCCCAATGATCAAAAAT
scaffold190771_14.2	1	p3	(AAG)5	15	50	64	ACAGTGGAGGAGCTCGCA

scaffold191178_12.[illegible]	1	p3	(TAT)6	18	439	456	CTCGAACACGAGACCTCCAG
scaffold191414_12.[illegible]	1	p3	(TAA)5	15	195	209	CGGACCAGACTCCAACTTTT
scaffold192041_13.[illegible]	1	p3	(TTG)6	18	600	617	TATAGCCGACCCCACGTAGT
scaffold192964_15.[illegible]	1	p3	(CAT)5	15	262	276	GGGCATCAAAACCTCCAGTA
scaffold193012_15.[illegible]	1	p3	(TAA)6	18	102	119	CTTCATAGTACGCCACCCGT
scaffold194068_11.[illegible]	1	p3	(ATC)5	15	67	81	TTCCTCAATTTTGGGACCAG
scaffold194374_13.[illegible]	1	p3	(CTC)5	15	209	223	GATGTACGCACTTGGTGGTG
scaffold194512_14.[illegible]	1	p3	(ATT)5	15	299	313	GGGGCGTGACAATATCAAAG
scaffold194558_12.[illegible]	1	p3	(GAG)6	18	453	470	GTCGCCACAAGAACGAAAAT
scaffold194772_14.[illegible]	1	p3	(ATT)5	15	1343	1357	GGATTCACGGATTGAGCCTA
scaffold195714_10.[illegible]	1	p3	(CTT)6	18	378	395	AGGCCAACTGACATCTGGAA
scaffold195808_16.[illegible]	1	p3	(TCT)5	15	3274	3288	TCGGAGGCGATTTACATAGG
scaffold196105_15.[illegible]	1	p3	(GCA)5	15	3922	3936	GGGGTTTTGCTGCTACACAT
scaffold196261_14.[illegible]	1	p3	(ATG)5	15	87	101	TTTCATGTGTGCTTATTGTTTTCA
scaffold196288_17.[illegible]	1	p3	(ATG)5	15	94	108	TGCAATGGCTTTTCATTGTT
scaffold196384_11.[illegible]	1	p3	(ATT)8	24	272	295	TCATTGTTGGAAATAGGGGC
scaffold196393_12.[illegible]	1	p3	(AAT)7	21	662	682	TCACCACGCTCCCTAAGTGT
scaffold197059_15.[illegible]	1	p3	(ATT)5	15	1201	1215	CGTGAAAATCCGATTAGGGA
scaffold197372_17.[illegible]	1	p3	(GCG)5	15	1337	1351	CCAGCGAGTCTTCTGAGCTT
scaffold197513_15.[illegible]	1	p3	(TGA)5	15	672	686	TTGTTCGCGATTTGCATTTA
scaffold197773_16.[illegible]	1	p3	(CAT)5	15	67	81	CCCTGCTCAGTAGATTCCTTTG
scaffold198329_16.[illegible]	1	p3	(TCA)6	18	2570	2587	GGCAAATTCGTCGTCAGACT
scaffold198789_10.[illegible]	1	p3	(TAA)5	15	1018	1032	TGCTGTTGAGAATAGCGAGC
scaffold199206_17.[illegible]	1	p3	(GGA)5	15	658	672	TTGGCCAAACTGATCAACAA
scaffold199703_10.[illegible]	1	p3	(ATG)5	15	298	312	CCCGCTATCAATGTTCCACT
scaffold200363_15.[illegible]	1	p3	(ATT)6	18	1396	1413	GTGTCTACGGCTGCCTCTTC
scaffold201027_11.[illegible]	1	p3	(ACA)5	15	360	374	GGAGAGGATTGGATGGACAA
scaffold201126_15.[illegible]	1	p3	(TCT)5	15	755	769	GACGTATGCCAAGCATTTCA
scaffold201387_15.[illegible]	1	p3	(ATC)6	18	525	542	ATTGTGGAGTTGCCCATCA
scaffold202062_18.[illegible]	1	p3	(ATG)5	15	1261	1275	GTGCATGCAGCTGAAGATGT
scaffold202174_12.[illegible]	1	p3	(CGG)5	15	75	89	GGAGGTATACTTTGCTGCAGGT
scaffold202779_10.[illegible]	1	p3	(CAT)5	15	46	60	TTCAGCAAAGAGTAGTTTCCCA
scaffold203553_11.[illegible]	1	p3	(GGC)5	15	90	104	GTTCGGAGTTGGGTAGTTGC
scaffold204264_15.[illegible]	1	p3	(TCA)8	24	2514	2537	AAGTTGTGGCCAGCAGAAAT
scaffold205423_15.[illegible]	1	p3	(TAA)5	15	1539	1553	CGGTAATATGGTTGGATGGG
scaffold205615_10.[illegible]	1	p3	(CGC)5	15	437	451	ACACCTCCTTCCATGCAAAC
scaffold205832_12.[illegible]	1	p3	(CTT)6	18	211	228	GCCTTTGGTCTTGTCCGTAA
scaffold206820_15.[illegible]	1	p3	(CCG)5	15	1728	1742	TTCTCCACCACCTCCTCCTA
scaffold207002_12.[illegible]	1	p3	(TAA)5	15	322	336	GAAGCTGAGCAACGAGAAGG
scaffold207037_17.[illegible]	1	p3	(ATT)5	15	1198	1212	AAGGTAGGTCTGCCCCTGTT
scaffold207385_12.[illegible]	1	p3	(TTC)6	18	77	94	CGTCTCTCCCTATTCCCTCTC
scaffold207395_15.[illegible]	1	p3	(ATC)5	15	419	433	TGGTTTTGGAGCATCATCAA
scaffold207641_10.[illegible]	1	p3	(CGC)5	15	148	162	CCTCGCCTCTTACATCTGCT
scaffold208184_20.[illegible]	1	p3	(ACA)5	15	1491	1505	TCTGTTGATCGACATCCCTG
scaffold209780_14.[illegible]	1	p3	(TAT)5	15	779	793	CGAAAATGATCAGTGCAACG
scaffold210867_16.[illegible]	1	p3	(AGA)5	15	2535	2549	AATCCCTTGGGAAATGAACC
scaffold211281_16.[illegible]	1	p3	(AAT)5	15	232	246	TGGGTCTAAAGATGTTGCCTC
scaffold211472_13.[illegible]	1	p3	(CAT)5	15	191	205	CACAAACCAAAGGTGCATGT
scaffold211884_15.[illegible]	1	p3	(ACA)6	18	2487	2504	TACTTTAGCTGGCAGGCACC
scaffold211964_11.[illegible]	1	p3	(GGC)5	15	58	72	ATTTTGAACGGTAACAGCCG
scaffold212278_16.[illegible]	1	p3	(TTC)5	15	1713	1727	CGCAAACACAGAACAGCATT
scaffold212860_10.[illegible]	1	p3	(TAT)8	24	275	298	TCAATGTTACGTTCAGGCCA
scaffold213189_14.[illegible]	1	p3	(CAG)5	15	2261	2275	AGCATTGGCTTCAGCTGTTT
scaffold213237_14.[illegible]	1	p3	(GAT)5	15	181	195	GAGAAAGTCAGTCCACGCCT
scaffold214027_10.[illegible]	1	p3	(TAT)7	21	683	703	TGATGGGCCTCTTCATTTTC
scaffold214139_14.[illegible]	1	p3	(TAT)6	18	510	527	CCCTACTCCCCCGTAAATTC
scaffold214306_12.[illegible]	1	p3	(CAG)5	15	74	88	GGGCAGCCAAATATAGAGCA
scaffold214402_11.[illegible]	1	p3	(ATT)5	15	855	869	CGGGAACAATAAATGCCAAG

scaffold214491_13.5	1	p3	(TTC)5	15	72	86	GGCCAAAACCGTGTAGTTTG
scaffold214746_11.4	1	p3	(ATA)5	15	436	450	ACTCCGTGTTCACCTTCTGC
scaffold215089_15.2	1	p3	(ATT)5	15	61	75	TGGAGTAATTGCATTACATTCGAG
scaffold215240_19.2	1	p3	(GCC)5	15	148	162	TGAACATTAAAGAATATCTACTCAGC
scaffold215520_11.4	1	p3	(TAA)5	15	183	197	GAATATTATGGCACCATGGGTA
scaffold215830_14.2	1	p3	(TTG)5	15	2314	2328	TAGCCGACCCCATTTTATTG
scaffold215960_12.5	1	p3	(GAT)5	15	206	220	CAAAACCATGGCATTTTTGA
scaffold215985_17.6	1	p3	(CGC)5	15	919	933	TATGGATTCACCTCCTTCGC
scaffold216198_17.0	1	p3	(ATT)5	15	1141	1155	CGTATCATTTGGTGAGCTTGAA
scaffold218216_15.0	1	p3	(ATT)6	18	872	889	TAGGCTTGATGGTTGTTGCC
scaffold218381_18.7	1	p3	(TTA)6	18	51	68	AATTGGGGTGTTGGAACTTG
scaffold218972_11.2	1	p3	(CCG)5	15	270	284	GAAAAAGGGAATAGGGCCAG
scaffold219240_12.7	1	p3	(AAT)5	15	51	65	GTACGCAATCATGCATCACC
scaffold219726_16.1	1	p3	(ACA)6	18	857	874	GGCGTGTGTTTGCCTTAGTT
scaffold220421_14.6	1	p3	(ATC)5	15	337	351	TTCTTGGTCTCCAAAGTCCG
scaffold221256_16.7	1	p3	(TAT)7	21	92	112	TTTGAAGATCCGGAAATTTGT
scaffold222115_10.9	1	p3	(TAT)6	18	230	247	TATTTTCCGGCGATTTTGAG
scaffold222377_10.8	1	p3	(TTA)5	15	321	335	GATTGAGATTGAGCCCGAAA
scaffold222464_11.2	1	p3	(CAA)5	15	358	372	TGGTCAATGAACCAAAGCAA
scaffold222611_10.2	1	p3	(TAT)6	18	180	197	GTAAGTAAGGCGGTGGCAGA
scaffold222940_12.0	1	p3	(CCT)5	15	103	117	CTTTCGATGCTCCTCTCGAC
scaffold222996_12.0	1	p3	(ATG)6	18	250	267	CTCCTGATGAACCCCACATC
scaffold223478_12.9	1	p3	(AAC)7	21	227	247	GCTCCAGAGCAAGATTCCAG
scaffold224095_10.5	1	p3	(TTC)7	21	37	57	CTTTGCCCTCTCTCCATCTG
scaffold224185_11.9	1	p3	(TGT)6	18	62	79	TTTGGTTCATGTAGCCGACC
scaffold224350_13.5	1	p3	(CCA)5	15	105	119	TGCAGAAGCATTATTCATCGTC
scaffold224356_15.3	1	p3	(TCT)5	15	258	272	CAGCCGAGAACATCATCAAA
scaffold224711_11.0	1	p3	(GCT)6	18	137	154	AGCAGCTAATCGCCTCTCTG
scaffold224875_16.2	1	p3	(TCT)5	15	377	391	TTTTCCAAATGCTCCAAAGG
scaffold224926_11.3	1	p3	(ACC)5	15	294	308	GGCTTAAACTCGATTGGCAC
scaffold227230_19.4	1	p3	(TCA)5	15	297	311	TTTCCCCAACCTCAACATTC
scaffold228260_13.1	1	p3	(GAA)5	15	150	164	GAGGATTCAGAGGAGGACCC
scaffold228855_17.2	1	p3	(GAA)5	15	335	349	TCAAATTGAATGGCTTGGTG
scaffold229187_14.0	1	p3	(TCT)5	15	101	115	TCAGAAGGGGTTATGTCCTCA
scaffold229472_14.5	1	p3	(CGT)5	15	223	237	GATCCCCTGCAAGTGTGACT
scaffold230308_15.9	1	p3	(TAT)6	18	861	878	GCATCTAAGGAACATGGATACTG
scaffold231319_11.7	1	p3	(CGG)5	15	116	130	CCGGAAGAAAGAGTGGGTTT
scaffold231826_17.3	1	p3	(TAA)6	18	41	58	CAATATAAGAACATTTATGGCAAACA
scaffold232245_16.8	1	p3	(ATT)5	15	246	260	TTGTGATTCGTTGTGTTTTGC
scaffold232891_16.7	1	p3	(CAT)5	15	661	675	TCCCAAACTTGAATCCTTCG
scaffold233919_13.7	1	p3	(AGC)6	18	767	784	ACATTCCCATTTGCAAGCTC
scaffold234028_13.0	1	p3	(CAA)5	15	429	443	TAACTGTGATCACGAAATGCG
scaffold234814_13.9	1	p3	(TTC)5	15	67	81	GATGCAGCAACTTCCTCTCC
scaffold235450_20.3	1	p3	(TTC)5	15	404	418	GGTTCTCCCTACTCCCCCTC
scaffold235518_10.4	1	p3	(AAG)5	15	199	213	GTGCCCACACTGCTCAACTA
scaffold235758_13.3	1	p3	(CAT)5	15	157	171	GGGAGCATCATCAACGTTCT
scaffold236079_10.7	1	p3	(TGT)6	18	47	64	ATGTAGCCGACCCCAATTTA
scaffold236162_16.5	1	p3	(CCG)5	15	659	673	AATCCGTACCAAATCCCTCC
scaffold236638_18.1	1	p3	(TTC)5	15	106	120	CTCGCTGACTTCAATCCACA
scaffold237160_12.0	1	p3	(TGT)5	15	134	148	TTGGCAATTTCTTGGAGGAG
scaffold237457_15.6	1	p3	(GGT)6	18	119	136	TTAACGTCAAATGCGAATGG
scaffold237867_13.3	1	p3	(CAC)7	21	103	123	CCGAAGTGCTAGACCCAAAG
scaffold238321_14.6	1	p3	(CTT)6	18	254	271	CCCCAAACTTATTGACAAACG
scaffold238467_11.6	1	p3	(TCA)5	15	172	186	GCAGTACTTGAACCTTCGCC
scaffold238544_16.6	1	p3	(ACC)5	15	93	107	CTAAAACTTGAAAACGCCGC
scaffold239061_10.7	1	p3	(TGA)5	15	281	295	AAATTTATTTTGAGCCGGGG
scaffold239487_16.3	1	p3	(ACA)5	15	317	331	ACGTTTTCTTGCCCACAAAC
scaffold240471_16.7	1	p3	(AAT)6	18	371	388	CAGAAAGGGGAACACTCGAA

scaffold240556_16.9	1	p3	(TTA)6	18	1737	1754	CACTATGTGGACCATCAATCTCA
scaffold241245_17.6	1	p3	(GAT)5	15	185	199	CTCGAGCGCACAAAACATT
scaffold241498_16.7	1	p3	(CAA)5	15	3575	3589	GCAAAAGACAATGAAGGGGA
scaffold241682_10.6	1	p3	(ATA)7	21	152	172	GTCCTTTGTGGAGCGATGTT
scaffold241953_16.1	1	p3	(TCT)5	15	533	547	CGAACTAGCAAACCGGCTAC
scaffold241989_16.6	1	p3	(TGC)5	15	544	558	ATACGGGTGAAGTGACCAGC
scaffold242060_14.5	1	p3	(CCG)5	15	94	108	TCCCCTTTCTCTCTCTCTTCG
scaffold242167_15.1	1	p3	(CAA)5	15	113	127	ACCACTCAAACCCACAAAGG
scaffold242495_15.0	1	p3	(ATT)5	15	825	839	AGCCGCGCCTAAATAGTTTT
scaffold242545_16.6	1	p3	(GCA)6	18	826	843	ACATTCCCAAGGCTGAGATG
scaffold244774_16.6	1	p3	(TCT)5	15	276	290	TAATTCGCTGAAGCAGCAAC
scaffold245132_13.2	1	p3	(AAT)5	15	455	469	TGCAGAAACCATGTATTTACTTTGA
scaffold245188_17.2	1	p3	(GTG)5	15	562	576	TCCACTTTTCTGCTTTGGCT
scaffold245530_12.5	1	p3	(CAG)5	15	50	64	CTGGGCAGCCAAACATAGAG
scaffold246144_14.9	1	p3	(TTA)6	18	571	588	GAAAGCATTTAACCGCCAAA
scaffold246368_13.8	1	p3	(TAA)5	15	1575	1589	GCACAGGTAGTGTTGCATGG
scaffold246939_15.1	1	p3	(AAC)5	15	899	913	TCAAAGGGTTTGAATGGCTC
scaffold247019_12.6	1	p3	(TTA)5	15	576	590	AGATCGAGCGATTTGTACGG
scaffold247375_12.0	1	p3	(GAC)5	15	80	94	CAGAGAGGCACTATACAGTTCGG
scaffold248400_12.1	1	p3	(ATC)7	21	47	67	TTTTAATTCCCCATATTACCAACA
scaffold249059_18.2	1	p3	(TAT)5	15	481	495	AATGGGACAACTCATCCTCG
scaffold249220_10.6	1	p3	(ATT)5	15	139	153	GATAGCCGTTGGTGGTTGAT
scaffold250518_14.4	1	p3	(ACC)7	21	635	655	CGTCTCTTTTCCGTCGACTC
scaffold250584_18.5	1	p3	(GTG)5	15	131	145	TCGGATCCACTTTTCTGCTT
scaffold250649_13.5	1	p3	(CTC)5	15	361	375	GGAACCCCTTCAGGATTCTC
scaffold251316_16.7	1	p3	(AAC)6	18	4158	4175	CGTTGGACAATCTGTAGCCA
scaffold252132_14.6	1	p3	(TTA)5	15	95	109	TGCGGCTTCCATTTAGATTT
scaffold252319_12.3	1	p3	(GCC)5	15	68	82	CTTCACTCGCCGGAAAAAT
scaffold253256_16.0	1	p3	(CCG)5	15	197	211	CCAGATCTGCCAACGTTTTT
scaffold253263_10.6	1	p3	(TAT)5	15	274	288	TTTTCTTTTCCTTTTATGCTCG
scaffold255159_16.6	1	p3	(AAC)6	18	2397	2414	TATAAGGCAATTTGCGAGGC
scaffold256100_10.9	1	p3	(AGA)5	15	110	124	TATCCTTCATCAGTTCCGCC
scaffold256515_14.2	1	p3	(TCA)5	15	446	460	TTTGGGTCCTCCACAAGAAC
scaffold256590_14.1	1	p3	(TCT)6	18	88	105	GTCTTCGGAAGCGATCTCAA
scaffold256830_12.8	1	p3	(CCA)5	15	119	133	GTCATACCAGAGGAGCAGGC
scaffold259633_12.9	1	p3	(TCA)6	18	1912	1929	CATCACGTCCCATTGTTCTG
scaffold259748_17.4	1	p3	(TAT)5	15	444	458	CTGCGACAATTGCTCAAAAC
scaffold259833_15.3	1	p3	(TTG)5	15	2497	2511	TGACCCACATAGTGGGAAAGA
scaffold260297_13.0	1	p3	(GAA)6	18	73	90	GATGCAGATGAGGACGTGAA
scaffold260857_14.4	1	p3	(GGC)5	15	477	491	TTCTTCGCTACGGCTACGAG
scaffold260910_16.0	1	p3	(TAT)7	21	480	500	AAAGGTTGAGAACCATGATGA
scaffold261491_12.9	1	p3	(TTA)6	18	942	959	ATGCCGTGATTGAAACACAA
scaffold262041_13.8	1	p3	(GAA)5	15	431	445	GGCTCTGCAAGAATTTGAGC
scaffold262207_16.6	1	p3	(CGC)5	15	49	63	CCCTCCTCCTTCTTGCTCTT
scaffold262507_17.0	1	p3	(CAT)5	15	46	60	GAATCAAGTATCTGATCTGAGCCA
scaffold262904_10.5	1	p3	(TCT)5	15	99	113	TGCTGCTGCTTTCTTCATTG
scaffold263023_17.8	1	p3	(GGC)5	15	46	60	GAAAACAAAAGATAGATGGAGCAA
scaffold263605_17.5	1	p3	(CTC)5	15	934	948	TTGCAGAATCCAACTGCAAG
scaffold263663_18.3	1	p3	(CCG)5	15	216	230	ACGGTTATTGGGAAATGCAA
scaffold263979_16.2	1	p3	(GAT)5	15	358	372	GATTGCGACCACCCTTACAT
scaffold264197_12.8	1	p3	(TGT)6	18	485	502	TGCGATTAAGGCTTTGGTGT
scaffold264535_10.0	1	p3	(GGC)5	15	418	432	GGCGAAAGTGTAGATCTGGC
scaffold265184_12.7	1	p3	(GGC)5	15	317	331	GCCAGTCGTATCTAACCCGA
scaffold265537_18.7	1	p3	(TTA)6	18	1112	1129	TGTTCATCGGTGAAGACCAA
scaffold266411_13.8	1	p3	(GGC)5	15	198	212	TGTGTGATCCCTCAACAGGA
scaffold266886_17.2	1	p3	(ATC)5	15	733	747	TTTCTGGAAATAGGGGGACC
scaffold266915_10.3	1	p3	(TGT)6	18	51	68	GGTTCATGTAGACGACCCCA
scaffold266976_17.4	1	p3	(GTG)6	18	837	854	CGATCTCGTTGTGTTTCGTG

scaffold266982_15.8	1	p3	(ATA)5	15	960	974	CCTCTTCCTCGTACTCGGTG
scaffold267446_16.6	1	p3	(TTG)5	15	1185	1199	TGGCTCCACAATTCATAGCA
scaffold267951_17.5	1	p3	(TAT)6	18	1442	1459	TCGTAGTTTGTTGGCTCAAGG
scaffold268532_16.0	1	p3	(ATC)5	15	150	164	GAACCTGTGGTAGTACGGCG
scaffold268972_16.2	1	p3	(TTG)5	15	3261	3275	GCCATGCCTTCAGTTTTGAT
scaffold269307_15.0	1	p3	(AAT)5	15	2142	2156	TTGCACGAACAAAATTCGAC
scaffold270782_11.7	1	p3	(TTC)6	18	296	313	TCGAATCAAATCCATCAGGA
scaffold271156_13.2	1	p3	(TAT)5	15	2199	2213	AGCATCAACTGCGGCTAACT
scaffold272205_15.2	1	p3	(ATG)6	18	38	55	GATGATGATGCCACTCTTGG
scaffold273462_17.2	1	p3	(TAA)5	15	460	474	GGATTGGGGCGTGATAAATA
scaffold273485_23.3	1	p3	(TAA)5	15	1011	1025	ATGTTTTCGCGCCTTATTTG
scaffold273576_17.5	1	p3	(GAA)5	15	327	341	AGCCGATTCTGAAGTTCCAA
scaffold273832_10.4	1	p3	(GGT)5	15	657	671	AAATCTGCCCCAACACTCAC
scaffold274250_10.1	1	p3	(GCA)5	15	168	182	CCGTGCTATGATGAGCCATA
scaffold275742_10.2	1	p3	(ATA)6	18	394	411	ACACCCCGTATTTTCGAATG
scaffold275832_16.8	1	p3	(TGA)5	15	3842	3856	GAGAGAGTTGTGCATTGGCA
scaffold276474_14.7	1	p3	(TCA)7	21	1163	1183	GGCTTAATGGATCGTGTCGT
scaffold277111_17.0	1	p3	(TAC)5	15	824	838	GATGTGGGCTTTTTCCGTTA
scaffold277585_14.5	1	p3	(ACC)6	18	75	92	GCGACGACTCTCCGTCTATG
scaffold277947_14.9	1	p3	(TCC)6	18	371	388	ATCTTGATCATGCAGCTCCC
scaffold278106_12.3	1	p3	(TCT)5	15	266	280	TGGGTGGGTACCGATTAAAA
scaffold278683_16.3	1	p3	(CCG)5	15	170	184	AAATTCAATTACCTGCCGGA
scaffold279140_13.3	1	p3	(GCT)5	15	51	65	ATGACGTCGGCGGTGATT
scaffold279692_14.6	1	p3	(ATT)5	15	386	400	CTAGTTTCATTGGAGGCGGA
scaffold279735_15.9	1	p3	(CCG)6	18	1083	1100	CATCCTTTCAATGGCAATCC
scaffold279736_11.3	1	p3	(TTA)7	21	859	879	GGTGGGAGTAGCCTCATCAT
scaffold280046_15.7	1	p3	(GGC)5	15	49	63	GAGAACAAACAAAGAAGAGAAAACA
scaffold280177_11.3	1	p3	(ATG)5	15	470	484	AACATGTTTTCAATCTTGACCG
scaffold280767_18.1	1	p3	(TAT)5	15	660	674	TTGCACAGACATTATCGCATC
scaffold280832_16.3	1	p3	(TAA)5	15	234	248	AAACCTCCACCAGAAATTAAAGA
scaffold281345_13.2	1	p3	(TCC)6	18	301	318	TGCGTTAACGTACACAACTTCA
scaffold282108_10.3	1	p3	(AAG)5	15	188	202	GGTCCCAAGTAAGGTCGGTT
scaffold282240_19.0	1	p3	(CAG)5	15	111	125	GAGGACGAATCCTTTCCAGC
scaffold282444_14.6	1	p3	(AAT)5	15	577	591	TTCCCTACCCCAAATTTTCC
scaffold282574_10.5	1	p3	(AAT)5	15	212	226	GGCAAATAATTGATCGCCTT
scaffold282915_12.3	1	p3	(AAT)5	15	113	127	GCATTTCAGGATATTAAAAACCAA
scaffold283481_15.7	1	p3	(AAC)5	15	714	728	TCGTTCATGGCATATTCAGG
scaffold283870_13.6	1	p3	(TGA)5	15	161	175	ACGCTATGTACCTTGGGTCG
scaffold283926_10.1	1	p3	(TCT)5	15	219	233	CAAGGATCATGTCGCAGAAA
scaffold284069_12.0	1	p3	(GAG)5	15	179	193	TCATGGATTTAGACAGCCCC
scaffold284440_17.2	1	p3	(TTC)6	18	418	435	CGGGGAATTATATAAGTATGTCTTGA
scaffold284611_14.3	1	p3	(CAC)5	15	82	96	ACGAGGGTGATGTTGGAGAG
scaffold285080_10.2	1	p3	(ATT)6	18	292	309	TCAATGCTTTGCCTATGTTCA
scaffold286131_11.5	1	p3	(ATC)6	18	37	54	TGTACCGGTATCATCCCCAC
scaffold286954_14.5	1	p3	(CAA)5	15	206	220	TGCTCCAGTTTGTCGTGAAG
scaffold288377_15.3	1	p3	(GAA)5	15	542	556	CAATTGGTTGACAAGACCCC
scaffold288502_17.0	1	p3	(AGG)6	18	484	501	ACGATCATCCATCCATCCAT
scaffold288737_11.6	1	p3	(TTC)5	15	673	687	TCAACGAATACCTGCTACCAA
scaffold289957_18.4	1	p3	(TGT)5	15	528	542	TGACGGTGTACCAGAAGCAG
scaffold291053_17.7	1	p3	(CGG)6	18	47	64	CCACCATCGTCGTCTCATC
scaffold292048_16.0	1	p3	(AAT)5	15	1774	1788	TAGAAATGTCAGGGTTGGGG
scaffold292215_14.5	1	p3	(TAT)5	15	1760	1774	ACGTGGGTTCCATTTTTGAG
scaffold292334_12.0	1	p3	(ATG)6	18	114	131	GATGATGATGCCACTCTTGG
scaffold292844_13.0	1	p3	(CAG)5	15	85	99	GTGGGGCAGCCAAATATAGA
scaffold293464_11.0	1	p3	(TCA)6	18	79	96	GCGTCCGACTCCTCAACTAT
scaffold293507_15.3	1	p3	(CTT)9	27	298	324	TTTGTTGGGAAGGTCGTAGC
scaffold293811_16.1	1	p3	(ATT)5	15	239	253	CGTAGGCCTAGTTGGCTCTG
scaffold294318_10.9	1	p3	(ATA)5	15	367	381	CATCGGTTAGTTCTCCCACC

scaffold294595_10.6	1	p3	(CTT)5	15	114	128	ATGGTTGCCTTTGTTTGGAG
scaffold295174_10.1	1	p3	(AAT)5	15	696	710	GCTCTAACGATGCCTTTTCG
scaffold296016_11.0	1	p3	(TCT)5	15	101	115	ACACTCCACCAAGCCAACTC
scaffold296860_17.7	1	p3	(TGA)8	24	431	454	GGGAAATCCAAGTCCCAACT
scaffold297356_17.6	1	p3	(GAA)5	15	591	605	GAAGGTGTGTCGGAATTCGT
scaffold298144_15.6	1	p3	(ATG)5	15	1133	1147	CCCCCAATACCCAAATTAGA
scaffold298858_13.4	1	p3	(ATT)5	15	370	384	CAGTGTGGGTTTCGGTGTTT
scaffold299091_13.7	1	p3	(CGG)7	21	1197	1217	ACACAAGTTATTGGCGAGGG
scaffold299353_16.2	1	p3	(AAC)7	21	351	371	TCTCGAACCAAATTTCAGCC
scaffold299863_17.6	1	p3	(CAT)5	15	747	761	AAGGAGGAAAAGAACCTGCC
scaffold300774_16.9	1	p3	(TCA)5	15	750	764	CATGAGGGGAACTGGTTCAT
scaffold301696_13.9	1	p3	(GAA)5	15	680	694	GGTTTTTGGTGGGAGGAAGT
scaffold301952_13.4	1	p3	(TTA)5	15	277	291	CTCTATAGCCGCCTGCATTT
scaffold302025_16.5	1	p3	(AAT)5	15	2557	2571	AACCCTCTTAGGGTGGCAAT
scaffold302455_13.5	1	p3	(TGG)5	15	890	904	TGGAGTTGCATAAATTGTGG
scaffold302573_17.1	1	p3	(GGC)5	15	123	137	ACAATGAATAAACGGGGCTG
scaffold302805_14.5	1	p3	(CCG)5	15	1085	1099	AATATGGCAAAGGCTGCTGT
scaffold303551_12.4	1	p3	(GAT)5	15	67	81	GCTAGGAAGGCTCTTGGGAT
scaffold303804_12.6	1	p3	(TCT)5	15	40	54	GCAGCAACTTCCTCTCCTTC
scaffold304525_14.0	1	p3	(CAT)5	15	268	282	ATCATCAATGTCTCCTGCCC
scaffold304604_14.1	1	p3	(AAC)6	18	980	997	ATGACTGCAGGAGATTCGCT
scaffold306052_15.9	1	p3	(TTC)5	15	2485	2499	CGAAAAATGGGATGCTGAGT
scaffold306230_19.1	1	p3	(TAA)5	15	160	174	GAACTAACTGCTGCAAGGAAAAA
scaffold306253_18.5	1	p3	(CTC)5	15	336	350	TGGGACAAGCTCTTGTGTTG
scaffold306528_11.2	1	p3	(CCT)6	18	80	97	CTTCTCAGCCTCAGCCTCTG
scaffold306586_15.0	1	p3	(TAA)5	15	415	429	TTGCTTTGGCTCACAGTACG
scaffold307222_10.8	1	p3	(CTT)7	21	1220	1240	ATTTGCAGGAATTGGTGAGC
scaffold307589_16.9	1	p3	(CGC)7	21	5006	5026	CGAGTTGCCTGACACTGAAA
scaffold307773_13.0	1	p3	(AAG)5	15	166	180	CCGGGTTGAGACCTTTGTAA
scaffold308185_15.5	1	p3	(TTA)5	15	71	85	GGGAGTGGGTTATCGGTTTT
scaffold308307_16.0	1	p3	(ATT)5	15	32	46	GGAACTTGATTATGTATCTGATGATG
scaffold308739_10.5	1	p3	(AAT)5	15	416	430	AGCCCACCTTATTGTTCGAG
scaffold309330_12.0	1	p3	(GAG)6	18	335	352	CGCGAAGGTGACTGACATAG
scaffold309954_10.5	1	p3	(TGA)5	15	86	100	CATCACGACGAGGAGCATTA
scaffold310146_11.4	1	p3	(GCC)5	15	67	81	CCTTCCCTCTATTTCCGACC
scaffold311109_14.5	1	p3	(AAG)5	15	72	86	TCCAAGGACTTGTCGGTTCT
scaffold313403_15.8	1	p3	(AAG)5	15	482	496	GCTCGTTGCAAGCATATGAA
scaffold313707_16.1	1	p3	(GGA)5	15	50	64	AGTTGGGAGTTGGGAGTCCT
scaffold314284_10.9	1	p3	(ACA)5	15	494	508	GATCCATGTGTCCACGTGTC
scaffold314514_11.0	1	p3	(ACC)6	18	180	197	CTCACTCTCTCCCTCCCTCC
scaffold314863_10.7	1	p3	(GCA)5	15	52	66	CTTCAGCTTGGTTCTGGAGG
scaffold314882_16.8	1	p3	(ACC)6	18	679	696	GGGTCCATCATTCCCTTTCT
scaffold315255_14.1	1	p3	(AAC)7	21	408	428	TTTGCCAAGCTATCCAAAGG
scaffold316231_12.8	1	p3	(AAT)5	15	379	393	GCTGGATCACTATTTGGTGG
scaffold317633_10.7	1	p3	(TAT)5	15	512	526	ACCCGAACCGGAATACAAAT
scaffold317793_12.5	1	p3	(TGA)5	15	245	259	ACGGCACCCACAATAAATTC
scaffold318316_15.5	1	p3	(CAA)5	15	105	119	TACAACCTTCGATCCATCCC
scaffold318417_18.8	1	p3	(GAA)6	18	29	46	TCCTTACTGAATTTGGGGTTG
scaffold318907_13.2	1	p3	(GAA)5	15	232	246	CTCCTGCTTCACCTCGATTC
scaffold319802_11.1	1	p3	(GGC)7	21	154	174	CAGAGGTTTTGGAGTTTCCG
scaffold321117_17.3	1	p3	(CTT)5	15	239	253	TTCTCCTCCCTTGTGATTGG
scaffold321268_12.3	1	p3	(GCG)5	15	181	195	GCGGAGGCAATAGACAGAAG
scaffold321664_11.5	1	p3	(CTC)7	21	37	57	TCATCCTCGGAAGCAATCTC
scaffold321826_15.8	1	p3	(TAA)5	15	932	946	AGTACCATTTCGGCCATTTG
scaffold322064_14.2	1	p3	(CGT)5	15	45	59	CGTCGACCACCATCTTAACAC
scaffold322137_17.5	1	p3	(GAG)5	15	156	170	CTTGACGGTGAAGAGGGTTG
scaffold323396_17.5	1	p3	(TTA)5	15	101	115	TTTTCGTTTCTGGTTTCGCT
scaffold324512_18.8	1	p3	(TCT)5	15	1196	1210	AGACCTCGAGTCGGAGATGA

scaffold324909_15.(	1	p3	(CAA)5	15	2544	2558	CTCGGGGGTCAACACTACAT
scaffold325026_14.(	1	p3	(AAT)5	15	2623	2637	AAACGAGGGCGAACTGTAGA
scaffold325899_16.(	1	p3	(TGT)5	15	46	60	ATGTAGCCGACCCCAATTTA
scaffold325900_16.(	1	p3	(ATC)5	15	5101	5115	TTTCCCTCCAAAGCCAGTAA
scaffold326079_12.8	1	p3	(AAT)6	18	435	452	TGTTGCAATTCGTGTTAAATCTT
scaffold326219_13.(	1	p3	(TGC)6	18	157	174	CGCCCAGTTCTGGTCATACT
scaffold326522_15.(	1	p3	(CAC)5	15	568	582	TCACCAACATGCCCGTATAG
scaffold326671_11.8	1	p3	(ATT)6	18	436	453	TTCCTCTCGATCCTCGTTACA
scaffold327243_14.(	1	p3	(ATC)5	15	357	371	GGCTTGCATTTGAAAGTACAGA
scaffold327621_16.2	1	p3	(GTT)5	15	366	380	CGATATGAACGAATGCGATG
scaffold327776_15.(	1	p3	(ATC)5	15	22	36	ATTGCCACCATCCGAACA
scaffold328875_18.4	1	p3	(CTT)5	15	1227	1241	CTTTGCTCGATCTATTCGGC
scaffold329574_14.2	1	p3	(AGT)5	15	1063	1077	AGCTTCTTTCAATCGCCTCA
scaffold329965_11.8	1	p3	(CGC)5	15	64	78	TTCAAAATTATACCTGCCGGA
scaffold330161_10.(	1	p3	(CTG)5	15	67	81	ATAATTCCCGGCGTCATCAT
scaffold330313_15.5	1	p3	(TAA)5	15	418	432	GTGTCCAGTCCGAGCAAAAT
scaffold330377_11.7	1	p3	(TAA)7	21	752	772	ATTTTCCCATTGCACCAAAA
scaffold330386_13.8	1	p3	(AGA)6	18	392	409	GGGGATCTTGGGAAGTGATT
scaffold330605_15.8	1	p3	(TTA)6	18	3737	3754	CCCGTGGAGACGAGGTATTA
scaffold331266_15.7	1	p3	(TTA)5	15	773	787	CGTGGTGCACTTGCATACTT
scaffold332699_15.7	1	p3	(TGA)6	18	163	180	AGCAGATATGGGAGTGGTGG
scaffold333040_14.4	1	p3	(GTT)5	15	269	283	GGTGGAGCAAGGTTTGTGTT
scaffold333453_12.5	1	p3	(ACA)5	15	954	968	CGAGACTCATCCACTTGCAG
scaffold333557_20.1	1	p3	(CAA)5	15	189	203	GTCAGACCAGCTCTCCTTCG
scaffold333877_12.7	1	p3	(GAT)5	15	30	44	GAACAAAATAAAAATTTGGGAGGA
scaffold334955_15.5	1	p3	(ATG)5	15	489	503	AAGGGGCACTGGTAAGGTCT
scaffold335564_13.(	1	p3	(ATA)5	15	71	85	TTGCTTCCGTGTTTTATCCA
scaffold335872_18.3	1	p3	(GTT)5	15	1313	1327	TCTAGACTGCGGGGTCAATC
scaffold336179_12.(	1	p3	(CGG)6	18	776	793	AAGAAGAAGCTCCTCCCACC
scaffold336211_11.2	1	p3	(GAG)5	15	93	107	GTTGGGGGTGACGTAGTCG
scaffold336425_15.4	1	p3	(ATA)5	15	620	634	ATGTGTGTGGCCATTTTCAA
scaffold336918_13.7	1	p3	(TAA)5	15	1272	1286	TGCGTCAGAAGATCCATTACA
scaffold337007_12.7	1	p3	(TCT)5	15	114	128	GATGCAGCAACTTCCTCTCC
scaffold337462_15.2	1	p3	(TAT)6	18	3218	3235	AAGACGTTCACCCACTGGAG
scaffold337500_11.2	1	p3	(GTT)5	15	175	189	GGGAGAGTTCTCTGGGGAAG
scaffold337517_10.2	1	p3	(ATA)5	15	314	328	CAGTTCAGCAGCGGAATTTT
scaffold337542_13.4	1	p3	(TTA)5	15	390	404	CATGAAGTCCTTCCCCTTGA
scaffold338225_15.(	1	p3	(CGT)5	15	3772	3786	GCGGAATAGCATTCACGTTT
scaffold339547_14.(	1	p3	(AGC)5	15	835	849	AAACCATGCAATGAACACGA
scaffold339616_15.(	1	p3	(AGA)5	15	2908	2922	GGCGACATCAAACACAACAA
scaffold340330_17.(	1	p3	(TGA)5	15	1659	1673	GTCGCCAATTATTGATGCCT
scaffold340842_15.4	1	p3	(TTA)5	15	853	867	ACAGGCCATTAGTTGGCAAT
scaffold340866_10.4	1	p3	(GTT)5	15	286	300	TGGTTCCCTTCTTGTCCTTG
scaffold341209_10.7	1	p3	(TGA)5	15	82	96	GCGGTTTCGATGGACATAAC
scaffold341636_15.8	1	p3	(ATA)5	15	491	505	TCCATTTTTCTTCGTGCATTT
scaffold342387_10.3	1	p3	(CTT)5	15	48	62	ATCGTTCTGCAGGAGCTTGA
scaffold343217_11.7	1	p3	(CAG)6	18	153	170	CGATCATCCCACATCTGACA
scaffold343336_18.3	1	p3	(AGC)5	15	263	277	AAACTGCTTCAAATGGACCG
scaffold344047_13.7	1	p3	(TAA)5	15	242	256	TCTTCAAACTTTGTTCCGCA
scaffold345357_16.(	1	p3	(TGA)5	15	109	123	GGTAAGGTTGCGTACATCCG
scaffold345620_15.4	1	p3	(ACA)5	15	2464	2478	CGTGTGAAAATGAAATCGCA
scaffold346340_16.(	1	p3	(ATC)5	15	1068	1082	TCCCCTTTCTCCACATTTCA
scaffold346586_18.1	1	p3	(TCG)5	15	911	925	CGATCGAATGATCCCTGAGT
scaffold346770_14.(	1	p3	(CAA)6	18	1327	1344	TATAGGTGCTGGGGCTAACG
scaffold346875_18.4	1	p3	(TGA)5	15	1389	1403	TTCCACGAACCAACCAAAAT
scaffold347121_14.8	1	p3	(AAC)5	15	450	464	TCCTCGCTCCTAACTTGCAT
scaffold347206_14.(	1	p3	(TGA)5	15	1834	1848	AAACCGAGTGTTTTTGCCTG
scaffold347630_13.1	1	p3	(TAT)5	15	258	272	TGTTTAAAGTCCATTCGGGC

scaffold348055_13.[illegible]	1	p3	(GGT)5	15	785	799	AGGCAAAAATGGAGGTGTTG
scaffold348394_10.[illegible]	1	p3	(CTT)5	15	33	47	AGGTGCACGTGACTCCAAC
scaffold348692_16.[illegible]	1	p3	(ATG)5	15	567	581	CCCTCCCCCTCTCATATTTT
scaffold349508_13.[illegible]	1	p3	(CTT)5	15	944	958	CATACCTCGGCCTTTGATGT
scaffold350182_20.[illegible]	1	p3	(GAA)5	15	51	65	CATTGAAGAGCTCGCTGGAT
scaffold351426_14.[illegible]	1	p3	(AAG)5	15	117	131	TGAGGTCGCTCACAGATGAC
scaffold352549_18.[illegible]	1	p3	(ATT)5	15	1952	1966	ATTTGGGAAGGCCTTTGTTT
scaffold352687_13.[illegible]	1	p3	(CCG)5	15	1944	1958	TAGTACTGCAGGCGAAGCG
scaffold352962_17.[illegible]	1	p3	(CTT)5	15	108	122	CCTTTAGAACTTGCTCCACCA
scaffold354674_15.[illegible]	1	p3	(ATT)6	18	281	298	GTTCGATTCTCCTTGGGGAT
scaffold354863_11.[illegible]	1	p3	(GGC)5	15	177	191	CGATACTGGGCTTCTTCGAC
scaffold355579_12.[illegible]	1	p3	(TTA)5	15	1307	1321	CCCCTTAGGGTTCCTCATGT
scaffold355825_18.[illegible]	1	p3	(GAG)5	15	200	214	CGAAGTATGTGTCCGTGGTG
scaffold356260_12.[illegible]	1	p3	(TTA)5	15	409	423	TGATGTCGAGCTGGATTGAG
scaffold357485_13.[illegible]	1	p3	(GAA)5	15	49	63	GAGGTATTGGGGGATTTGAG
scaffold357571_13.[illegible]	1	p3	(CGG)5	15	215	229	AAATCTCTCAATCTTGCGTCG
scaffold358333_13.[illegible]	1	p3	(GAG)6	18	329	346	TAACTGAGCTGGCGGACTCT
scaffold358846_13.[illegible]	1	p3	(TAT)5	15	198	212	GTCGTCGAGAGAAGCTCACC
scaffold359058_11.[illegible]	1	p3	(GAG)5	15	47	61	GTTTAGACGTCACGCACAGC
scaffold360691_10.[illegible]	1	p3	(GCT)5	15	112	126	CTGTGTCTCCAAGCTCCCAT
scaffold361262_15.[illegible]	1	p3	(TTG)6	18	1178	1195	CATATGCTCCATACAGCCGA
scaffold361783_17.[illegible]	1	p3	(CGG)5	15	28	42	GCGGCTCCTCCTATGTAAAA
scaffold361832_11.[illegible]	1	p3	(AAT)5	15	819	833	GGTCCGATTGAGGAGATGAA
scaffold362263_13.[illegible]	1	p3	(TGG)6	18	473	490	GGGGTTTTCTGAAATCGTGA
scaffold363199_13.[illegible]	1	p3	(GGC)5	15	615	629	ACATCTTGGCCGTCGTAAAC
scaffold363512_11.[illegible]	1	p3	(AAT)5	15	230	244	AAATTGTTCTTCGTGGACCG
scaffold363652_16.[illegible]	1	p3	(CAT)5	15	705	719	CCCGCTTCGATATCATTTGT
scaffold363706_10.[illegible]	1	p3	(TAT)6	18	734	751	GTGCGGGCTAAAAATGTTG
scaffold363984_15.[illegible]	1	p3	(CCG)5	15	207	221	CGCTGCCTCTGTCAACAAT
scaffold364254_16.[illegible]	1	p3	(ACG)5	15	736	750	CTGTATCGAACGAGCATCCA
scaffold364668_17.[illegible]	1	p3	(ATT)5	15	151	165	CCAAATATTACAATAGCTCACAACAA
scaffold364863_15.[illegible]	1	p3	(CAT)5	15	212	226	GTGAGAAAGTTTGCTTCGGG
scaffold365149_12.[illegible]	1	p3	(ATC)5	15	88	102	TTTTAATACCCCATTTTACCAACA
scaffold365741_11.[illegible]	1	p3	(CAC)5	15	117	131	CCGAAGTGCTAGACCCAAAG
scaffold365823_11.[illegible]	1	p3	(TTC)5	15	236	250	GGAGTCACTCACGCTCTTGC
scaffold366054_12.[illegible]	1	p3	(GAA)5	15	189	203	TCAGCATGCAATACCAAACAA
scaffold366156_11.[illegible]	1	p3	(TCA)6	18	85	102	AGCTCCCTTCCTCATCATCA
scaffold367038_10.[illegible]	1	p3	(TAT)5	15	209	223	TCCATACTTGGCATCGGTTT
scaffold367064_15.[illegible]	1	p3	(TGT)6	18	44	61	TGTAGCCGACCCCAATTTAT
scaffold367414_15.[illegible]	1	p3	(TTA)5	15	33	47	TTTTCTTAAGTTGTGATTTGTCCA
scaffold367531_14.[illegible]	1	p3	(TTA)7	21	1214	1234	TTTGTCATATGCGAACAGCA
scaffold367865_12.[illegible]	1	p3	(AGA)5	15	199	213	TTGAGCTGCACTTGTTTTGTC
scaffold368139_15.[illegible]	1	p3	(ATG)6	18	928	945	ACGTACCCCAGCACTACTCG
scaffold368384_13.[illegible]	1	p3	(ATT)6	18	718	735	GATGGGACCCACACAATTTC
scaffold368453_15.[illegible]	1	p3	(ATT)5	15	1541	1555	TCTCCTTTTCCTCCACAAATTC
scaffold369065_12.[illegible]	1	p3	(TCC)5	15	89	103	ATCAGGCACATCACCATCAA
scaffold369933_14.[illegible]	1	p3	(AAT)5	15	188	202	TTTATTCCGTGACCCTCGAC
scaffold370153_21.[illegible]	1	p3	(TAT)5	15	797	811	TGAAAGTAATGCCCATGGAA
scaffold370868_13.[illegible]	1	p3	(GTG)5	15	721	735	GCGATGGGAAGTGCATATTT
scaffold371046_14.[illegible]	1	p3	(CTT)5	15	311	325	GGTTAATACCGCTGATGGCA
scaffold371152_16.[illegible]	1	p3	(ATT)5	15	111	125	CACGTGCATTAATTATTGTTTTCA
scaffold371327_14.[illegible]	1	p3	(AGA)5	15	72	86	TAGCAGGCCAAAGAGGAAGA
scaffold371613_15.[illegible]	1	p3	(TTA)5	15	604	618	TCTCCCAAGTTCAATCCATGT
scaffold372311_16.[illegible]	1	p3	(TTG)5	15	1738	1752	TTTCCTCTCTGAGCCTTCCA
scaffold372498_14.[illegible]	1	p3	(TTA)5	15	884	898	ATGCCTTATATGCCGGACAC
scaffold373173_10.[illegible]	1	p3	(TCT)5	15	184	198	CATTCTCTAAATGGTTTCCATTCC
scaffold374074_16.[illegible]	1	p3	(GAT)7	21	1138	1158	GATCTCGATCGTGACCCAAT
scaffold374595_10.[illegible]	1	p3	(GGA)5	15	305	319	GGGAGTTTCGTTTGAGCAAG

scaffold374606_11.!	1	p3	(AAT)5	15	252	266	ATAAATTGGGGTCGGCTACA
scaffold375106_16.7	1	p3	(AAG)5	15	313	327	TAGTGTGCGTGATCGAGGAG
scaffold375993_16.!	1	p3	(TCT)5	15	178	192	CACTAATGAATCTCCTAGCGGG
scaffold376324_14.!	1	p3	(CGC)5	15	341	355	GTAGCTCCCTTCCCAACAGA
scaffold376579_14.:	1	p3	(TTA)8	24	2311	2334	TTTTCAATGTCCACACCCCT
scaffold377319_14.2	1	p3	(CAT)5	15	192	206	ATGTCCTCAATCGATCCCAG
scaffold378423_17.(	1	p3	(CCG)6	18	4114	4131	CTCACGGTTGGAAGAAGAGC
scaffold379011_13.7	1	p3	(AAC)5	15	2761	2775	TCCAACCGCTTTTGCTTAAA
scaffold379895_16.(	1	p3	(GAT)5	15	950	964	CCGGATTACTCCGATGATGA
scaffold380032_15.(	1	p3	(GTT)5	15	457	471	ATTTCCCTGATTGGCTTCCT
scaffold380162_15.(	1	p3	(TCT)5	15	1942	1956	CGAAGTTGTGCAAACCAATG
scaffold380508_18.:	1	p3	(AGA)5	15	79	93	TTGAAAGAGCATCGTGCAAC
scaffold381524_10.(	1	p3	(CAA)5	15	46	60	GTGGCTTCTATGGGCAATGT
scaffold381536_14.:	1	p3	(AAG)5	15	441	455	GGGGTCAGTCGTGACAGATT
scaffold381572_14.:	1	p3	(TTG)6	18	46	63	TGTAGCCGACCCCAATTTAT
scaffold382019_17.7	1	p3	(TGG)6	18	732	749	ATGGCCTGCTCATACAGCTC
scaffold382035_12.2	1	p3	(ATT)5	15	492	506	CGGATGAATTTTTGTTCGCT
scaffold382318_11.!	1	p3	(AGA)5	15	51	65	GAGCACGTTATTGGCTGGAG
scaffold382656_11.(	1	p3	(AAC)5	15	243	257	CCTTGGAGTTGTCCAGCATT
scaffold382837_15.4	1	p3	(ACA)5	15	684	698	GCTCAACACCTGGGAGGTTA
scaffold382894_12.7	1	p3	(GAA)5	15	99	113	AGGGGGAGAGTTCTCTTGGA
scaffold382918_11.(	1	p3	(AAG)5	15	137	151	GAGTCTTCCGACAAACCGAG
scaffold384020_13.8	1	p3	(TGT)5	15	30	44	ATTGGGATTAAGGCTTTGGT
scaffold384289_16.:	1	p3	(TTA)5	15	727	741	CCCACTCGAAATTAACCACG
scaffold384495_17.(	1	p3	(TGA)8	24	39	62	CCCAAGACCCCAATTTGATT
scaffold385073_16.8	1	p3	(GAT)5	15	2131	2145	GGTCCTATATTGTGGTTTCCG
scaffold385714_14.!	1	p3	(CTT)7	21	375	395	AAACCTTAACGCCCTCACCT
scaffold386196_14.8	1	p3	(GGA)7	21	1195	1215	ACTCCGACATGGAGAACACC
scaffold386951_16.1	1	p3	(CCG)7	21	945	965	CCCCCTTTCCTCCTTTCTTT
scaffold387017_13.2	1	p3	(TAT)5	15	606	620	CCATTTTCCAAGAAAGGTTGTT
scaffold388081_14.(	1	p3	(TTC)5	15	616	630	TACACACCCCAAATATCCCG
scaffold388171_18.7	1	p3	(AAT)5	15	305	319	CGATGATCACGTCAAAAACCT
scaffold388181_15.2	1	p3	(AAC)5	15	323	337	TCGGTTCCGATCTTCACTTC
scaffold388480_14.!	1	p3	(ATG)5	15	42	56	CCTGAAACGAGTGATGCTCC
scaffold388544_11.2	1	p3	(GGC)5	15	154	168	GAGGTGAAACTGCCCTGAAA
scaffold389074_18.(	1	p3	(GTG)5	15	460	474	GAAGTTGGCGTGGTGGTAGT
scaffold389353_15.(	1	p3	(TTG)5	15	2863	2877	GTTTGCTGTTGGATGGGTTC
scaffold390641_15.4	1	p3	(ATG)5	15	84	98	TCCTGAAATGAGTGATGCTCC
scaffold390837_15.1	1	p3	(TGA)5	15	4209	4223	GAAGAGGAATGTGTTGGCGT
scaffold391802_19.!	1	p3	(CGC)5	15	73	87	ATGGTAGTATGGTGTGGGGG
scaffold392126_11.7	1	p3	(AAT)6	18	183	200	CTCCATCAGGTTTGTCCTCC
scaffold393743_17.7	1	p3	(TCA)5	15	1073	1087	TGGACCTTCTGATTTCGAGC
scaffold394131_18.(	1	p3	(CCT)5	15	2334	2348	GGGCTTCATCTTACCACCAA
scaffold394748_18.!	1	p3	(ACA)7	21	260	280	AGACAACGGGACGGAAGATT
scaffold394799_13.2	1	p3	(TGA)7	21	973	993	GATCTGCAGCCATGTTGAAA
scaffold395217_16.!	1	p3	(CAC)7	21	104	124	CCGAAGTGCTAGACCCAAAG
scaffold395267_11.(	1	p3	(TGA)5	15	447	461	AATGGTGGTGATGTCTGTGC
scaffold395887_15.!	1	p3	(ATA)5	15	2237	2251	TTTGCTTTGACTTTCGGGTC
scaffold396402_16.7	1	p3	(TCT)6	18	2002	2019	ATCTCCTGCAACACTCCCAC
scaffold397498_16.!	1	p3	(GAT)5	15	1796	1810	GATACGCGTCGTTGTCATTG
scaffold397875_17.1	1	p3	(CGG)5	15	366	380	TCTCCGCACACTGAATAACG
scaffold398183_12.(	1	p3	(GGC)5	15	154	168	TGTCACCCAACTTATAAAAGAAGAA
scaffold398509_10.(	1	p3	(TTG)5	15	391	405	AATTTTCTTTTGCCGACACG
scaffold399013_16.1	1	p3	(ATG)6	18	353	370	TGTGATGTGATTGGGAGAAATC
scaffold399959_10.(	1	p3	(CCA)5	15	473	487	GCCACATGCTTTGTGTGAGT
scaffold400179_11.!	1	p3	(TTA)8	24	699	722	CCCATAATTTTATTGGGGGA
scaffold400195_16.:	1	p3	(CAA)5	15	302	316	AGTTTGTTGCTCATAGCGGG
scaffold400346_14.1	1	p3	(TTA)8	24	3220	3243	CAATACAAGGGTTCTCGCGT

scaffold400467_12.[illegible]	1	p3	(TGT)6	18	51	68	TAGCCGACCCCAATTTAATG
scaffold400994_13.[illegible]	1	p3	(ATA)5	15	231	245	CAAGATGTTTGGCGATTGAA
scaffold401163_12.[illegible]	1	p3	(ATC)5	15	2352	2366	GCTGCAGATGCAAAACTCAA
scaffold401196_15.[illegible]	1	p3	(CGC)5	15	1640	1654	ACCACAAATTCCTCAACCCA
scaffold401353_14.[illegible]	1	p3	(CGC)5	15	701	715	TAAAATTGCCCTATTGCGCT
scaffold401617_20.[illegible]	1	p3	(TCC)5	15	217	231	TATCATCCTCGGAGGCAATC
scaffold402051_14.[illegible]	1	p3	(TCA)7	21	63	83	CATGAAACATTTCAAAATATCATCC
scaffold402182_11.[illegible]	1	p3	(TAT)6	18	220	237	GTGGTCCCGTAACTCGAAAA
scaffold402417_14.[illegible]	1	p3	(TGT)6	18	143	160	AACGTAAAGAGGGGTAGGGG
scaffold402720_10.[illegible]	1	p3	(CAA)7	21	99	119	TGAATCAGAAAATCCCCTGC
scaffold402745_14.[illegible]	1	p3	(ATA)6	18	4819	4836	TTCCCATCACTTCGAGGAAC
scaffold402881_16.[illegible]	1	p3	(AGA)5	15	118	132	TCACACCTCACTAATTTCTTGTTT
scaffold402964_10.[illegible]	1	p3	(CAG)5	15	357	371	ATTTACAGCTCGGTTTCGGA
scaffold403092_10.[illegible]	1	p3	(GCT)5	15	197	211	TCTGAAAATTGGTGCTGCTG
scaffold403381_16.[illegible]	1	p3	(AGA)5	15	45	59	CAGATGTAGGAGGCGAGGAG
scaffold403562_11.[illegible]	1	p3	(GAA)5	15	533	547	CGTGGTTCAACTGTTGCTTG
scaffold403578_16.[illegible]	1	p3	(ACC)5	15	5471	5485	CAATCTCCTCAGACATCGCA
scaffold404047_15.[illegible]	1	p3	(TTG)5	15	2237	2251	CCCTCCTTTCACATGTTCGT
scaffold404086_14.[illegible]	1	p3	(TGA)5	15	607	621	TCAACAATTTACGGGCTTCC
scaffold405201_12.[illegible]	1	p3	(ATT)5	15	206	220	CTGATGAAGAAGACGAGGGC
scaffold405553_15.[illegible]	1	p3	(TTC)5	15	323	337	ACCCCAAAACCACATCAGAC
scaffold405946_11.[illegible]	1	p3	(GCA)5	15	186	200	AGTAATCCTGACCACCGTGC
scaffold406857_15.[illegible]	1	p3	(AAC)5	15	1807	1821	TTTAACGGATATGCTTGGGA
scaffold407079_15.[illegible]	1	p3	(TGT)7	21	695	715	TGTAGCCGACCCCAATTTAT
scaffold407082_14.[illegible]	1	p3	(TAT)5	15	162	176	TGGGACAAGGTGACATAAAATG
scaffold408521_10.[illegible]	1	p3	(ATA)7	21	231	251	TGGTGTCGAGCAGCCTATCT
scaffold408554_15.[illegible]	1	p3	(CTC)6	18	952	969	CTCTTTCGCAGCCTCCTCTA
scaffold408727_12.[illegible]	1	p3	(CAC)6	18	79	96	AGCCATGAGCAATAACAGCC
scaffold408752_17.[illegible]	1	p3	(TTA)5	15	79	93	CCGAAATCTTCAAGCCACAT
scaffold408779_15.[illegible]	1	p3	(TGT)6	18	42	59	TGTAGCCGACCCCAATTTAT
scaffold408957_16.[illegible]	1	p3	(ATA)5	15	1121	1135	TCCACCATCTATGTGCCGTA
scaffold409454_14.[illegible]	1	p3	(TGA)6	18	41	58	CCCAAGACCCCAATTTGATT
scaffold409460_11.[illegible]	1	p3	(ATT)5	15	119	133	GGGCATTACATTTACGTTGGA
scaffold409635_15.[illegible]	1	p3	(ATG)5	15	667	681	CAGCCTCAGGACTCCTCATC
scaffold409658_13.[illegible]	1	p3	(TTA)9	27	192	218	ATCAGAGCCATGGAAAAGGA
scaffold409673_13.[illegible]	1	p3	(ATC)5	15	641	655	TTATTTGGCGGGTTAATGGA
scaffold409800_10.[illegible]	1	p3	(CCG)6	18	276	293	TCCCGCCCTCTACCTTTATT
scaffold409930_16.[illegible]	1	p3	(TCA)5	15	881	895	CCTTCAAAAACCATGCCACT
scaffold409975_13.[illegible]	1	p3	(AAT)5	15	581	595	GACCCAAATTTTCCCCAACT
scaffold409986_11.[illegible]	1	p3	(ACA)7	21	957	977	ACAGCTTCGGCAGATCACTT
scaffold410299_14.[illegible]	1	p3	(GCT)5	15	910	924	GCTTTGAGGGTCAAACCAAG
scaffold410669_15.[illegible]	1	p3	(CCG)5	15	634	648	ACCCCCTTCTCCGTTTCTAA
scaffold410673_15.[illegible]	1	p3	(ACA)5	15	344	358	GTTTCTGCCACGTGCATTC
scaffold411399_15.[illegible]	1	p3	(TCA)5	15	305	319	CGCTTTGCTAGATTTTTGCC
scaffold411453_15.[illegible]	1	p3	(GCT)5	15	4650	4664	GGATGCATTCCAGAGTTGCT
scaffold411723_15.[illegible]	1	p3	(GTG)5	15	52	66	ACCGTAACCGCCAAACAC
scaffold411825_16.[illegible]	1	p3	(TCA)5	15	813	827	AAGAACTGCTCCCTCAACAAA
scaffold411854_13.[illegible]	1	p3	(GGT)5	15	1615	1629	TTTGGCAGAATTGGATTTGG
scaffold412060_16.[illegible]	1	p3	(CTT)7	21	193	213	TCACCGGAAACAAATACATCA
scaffold412118_11.[illegible]	1	p3	(GAT)7	21	45	65	TTCTTCTGTACAATTAATGTCCAAGTC
scaffold412129_14.[illegible]	1	p3	(ATA)6	18	378	395	GTCCGTTGGGTGTTTGACTT
scaffold412312_16.[illegible]	1	p3	(ATA)5	15	399	413	ATGGGTAGCCAGTTTCACCA
scaffold412628_15.[illegible]	1	p3	(ACA)5	15	967	981	CCCAGTTCGCCACTAACAAT
scaffold412640_13.[illegible]	1	p3	(CGG)6	18	755	772	ATGCTGTGTTTGAGCGACTG
scaffold412823_14.[illegible]	1	p3	(CGG)5	15	39	53	CCACCATCGTCGTCTCATC
scaffold412941_12.[illegible]	1	p3	(TTA)7	21	629	649	TGTGATGAGATCGTGAACCTG
scaffold413335_11.[illegible]	1	p3	(TTA)5	15	549	563	TCCATTTAATCCCCCTTCTTC
scaffold413370_12.[illegible]	1	p3	(TTA)7	21	936	956	CTTGGCATTCTCTTTCTCGG

scaffold413822_15.4	1	p3	(TGT)5	15	1717	1731	AGTTATGTAGCCGACCCCAA
scaffold414264_15.3	1	p3	(GGT)5	15	903	917	AAGTGATATTTTCGGCGGTG
scaffold414770_16.7	1	p3	(TCA)7	21	2582	2602	TTTTCAAGATAACCAGCGCC
scaffold414925_15.9	1	p3	(AAG)5	15	399	413	CAAGGCAGAGAAAAAGCCAG
scaffold414928_17.3	1	p3	(GGC)5	15	1237	1251	TCCGCTGCTAATTTGCTTTT
scaffold414970_14.8	1	p3	(GAT)5	15	1017	1031	TTCAGGTTGAAGTCTTGGGC
scaffold415281_11.3	1	p3	(CGG)5	15	192	206	GGTTTTTGCTTGCACTTTCC
scaffold415387_13.3	1	p3	(AAG)5	15	1891	1905	TTGTATGGCACGAGTTCTTGA
scaffold415496_16.6	1	p3	(TCC)7	21	55	75	CTCGTCCTCCGTGGATATGT
scaffold415606_15.2	1	p3	(TAA)5	15	937	951	GCGCAGTTGCCAAACTTATT
scaffold415671_17.2	1	p3	(ACA)5	15	466	480	GCAGGTTGCAATAAAGCACA
scaffold415740_15.9	1	p3	(GGC)6	18	4949	4966	GAATCCGGAGAGTGAGACGA
scaffold415975_13.9	1	p3	(AGA)7	21	224	244	CGGAAGAAGCACAGACAACA
scaffold416035_15.9	1	p3	(TGA)7	21	3954	3974	GCCGACTCTTTCTTCCTTCC
scaffold416239_14.0	1	p3	(AAT)5	15	609	623	TAAACGTTCGTCACGTCAGC
scaffold416585_15.5	1	p3	(TGT)7	21	40	60	TGTAGCCGACCCCAATTTAT
scaffold417083_13.9	1	p3	(TGG)5	15	64	78	TGATATTTTCAATGATGGATCG
scaffold417533_16.1	1	p3	(TGA)5	15	4263	4277	TCAAATGCAAATCTACCTGGG
scaffold418366_16.5	1	p3	(ACA)5	15	855	869	TTCGACGCGGAAGTAAATCT
scaffold418822_16.3	1	p3	(TTA)5	15	2291	2305	TTGCCCATGCATACACATTT
scaffold418937_14.6	1	p3	(ATT)5	15	1097	1111	TTTTCGGTTACCATGTCGTT
scaffold419570_13.8	1	p3	(AGA)6	18	196	213	GAGCGGAGGCTTAAGGATTT
scaffold419756_15.3	1	p3	(GGC)5	15	638	652	TCGAAATTCAAGCACACGAG
scaffold419868_17.0	1	p3	(AAT)5	15	1053	1067	GAGAAAGAATCAACTGCGGC
scaffold419934_14.8	1	p3	(CGG)5	15	832	846	ACAAAAATCCCTCAGCAACG
scaffold419945_15.4	1	p3	(AGA)5	15	837	851	TTGGTTACTCTGGGAATGGC
scaffold419978_14.4	1	p3	(AAG)6	18	1206	1223	TTCTTGGATTGGAAGGCATC
scaffold420332_19.6	1	p3	(AAG)5	15	2411	2425	CGAGAAGATCTTGAGGTCGC
scaffold420462_13.8	1	p3	(TGT)5	15	49	63	TTGGTTCATGTAGCCGACCT
scaffold420574_12.9	1	p3	(ATC)7	21	737	757	ACGGAGAAATTCGACTCAGG
scaffold421484_16.0	1	p3	(GCG)5	15	765	779	CCACCATCACCGTTATTTCC
scaffold421527_13.3	1	p3	(TGT)5	15	121	135	CGGAGAAATGGTTTCTGCAT
scaffold421531_13.9	1	p3	(ATG)6	18	601	618	ATGAGGATGATGACGAAGCC
scaffold421606_15.0	1	p3	(GTC)5	15	1496	1510	TTTTTGCACCAAAATGGACA
scaffold421806_11.9	1	p3	(CGG)6	18	215	232	AATCATGTACACCCGGTTCG
scaffold422137_16.2	1	p3	(GAT)7	21	495	515	GAAAGTGGGGGTTGATTGAA
scaffold422920_15.4	1	p3	(CGG)5	15	248	262	ATAACCTGGAAATCGGGGTC
scaffold423260_13.3	1	p3	(GGC)7	21	988	1008	TGAAAATCCAACAAGCCACA
scaffold423275_16.9	1	p3	(ACA)6	18	1683	1700	TTGCACAAGCTCTTGCACTC
scaffold423536_15.5	1	p3	(ATG)6	18	404	421	TTTCTTGATTCCGTCGTTCC
scaffold423772_10.8	1	p3	(ATC)7	21	74	94	GATTTTACACCTGCAAGGGC
scaffold423946_15.9	1	p3	(TGA)7	21	1113	1133	TATGACACAATGTCCCGTGG
scaffold424283_16.9	1	p3	(CTC)5	15	689	703	TATTGGCTCCAACCAGGAAC
scaffold425527_15.6	1	p3	(AAT)6	18	241	258	CACGCTTGGATGTGATACGA
scaffold425572_12.0	1	p3	(TAT)6	18	76	93	CCTTTCTCCAATTGCTTTTCG
scaffold425691_12.9	1	p3	(ACT)5	15	1078	1092	ATAGATCCGGAATGATCGGA
scaffold425760_13.1	1	p3	(ATT)6	18	656	673	ACATACCGACGTGACCGTTT
scaffold425780_16.8	1	p3	(GGC)5	15	381	395	GTTCATCAATTCCCACCACC
scaffold425787_15.6	1	p3	(TGG)5	15	5747	5761	TGATCTACTCCGTCCCCAAG
scaffold425896_16.2	1	p3	(CAT)6	18	2496	2513	TGCAAGAGAAAACACATCCG
scaffold425909_19.8	1	p3	(GGC)5	15	645	659	ATGGTGGAAACCAAGGCAT
scaffold426298_12.3	1	p3	(AAT)5	15	204	218	GCTGTAAATGAAACTGCTGTATGC
scaffold426331_15.3	1	p3	(AAT)5	15	61	75	CAGAGCGCACTCGAAGAGTA
scaffold426861_12.7	1	p3	(TAT)6	18	114	131	TTGTCATATGAATCATGAAATTTTG
scaffold426888_15.2	1	p3	(CTT)5	15	871	885	GTTCGCTGAGGCAACAACTT
scaffold427405_14.8	1	p3	(ATA)5	15	527	541	TGACTGCGCGACTTTGATAC
scaffold429243_15.3	1	p3	(AAG)5	15	1172	1186	ACTCTAACCCCTACTCCGGC
scaffold430246_15.3	1	p3	(AAG)6	18	123	140	ATGCAGCTCATGTCTGATGC

scaffold430283_16.[illegible]	1	p3	(ACA)5	15	400	414	AGCAAAAGGTCGTCCTGCTA
scaffold430401_12.[illegible]	1	p3	(TAA)5	15	403	417	TTTAGGCCAAGTTGTACCCA
scaffold430477_13.[illegible]	1	p3	(CAT)7	21	1046	1066	GATTCAGGGGAAATTCACGA
scaffold430483_17.[illegible]	1	p3	(TTG)5	15	181	195	CTTTATGATGGAGGCGGATT
scaffold430561_14.[illegible]	1	p3	(GGC)6	18	294	311	GATCCCAGATCCGAATCCTC
scaffold430942_13.[illegible]	1	p3	(ATT)6	18	63	80	TTCCAAAGTTGGACTTATGTAAAAA
scaffold431025_15.[illegible]	1	p3	(GAC)5	15	1013	1027	TGAGCAACTGCCAAAACGTA
scaffold431685_16.[illegible]	1	p3	(CAC)5	15	533	547	AGTTATCTTCCTCCGGCGTC
scaffold431814_16.[illegible]	1	p3	(TCA)5	15	77	91	CATTGCGTCATACGATGTATTAAA
scaffold432461_12.[illegible]	1	p3	(TAT)6	18	120	137	CTAGCCCAGCATATTCCCAC
scaffold432493_16.[illegible]	1	p3	(ATT)5	15	177	191	ATCCCAAGCCATTACCATCA
scaffold432842_17.[illegible]	1	p3	(ATC)6	18	1401	1418	CTTTGAGCACCGAGTGGTTT
scaffold433340_16.[illegible]	1	p3	(GTC)5	15	845	859	CCTTCGACTTTTGACTGGGA
scaffold433423_14.[illegible]	1	p3	(CAT)7	21	734	754	AGCGAATCGAGATAGCCAAA
scaffold433438_14.[illegible]	1	p3	(TTA)8	24	519	542	AGCCACTGACAGCCACAGTA
scaffold433805_17.[illegible]	1	p3	(CCG)5	15	394	408	TCACACTCTCGCTCCCTCTT
scaffold434033_13.[illegible]	1	p3	(GGC)6	18	309	326	GCAAAATTAAAAATGCCCCA
scaffold434149_14.[illegible]	1	p3	(CTT)7	21	50	70	CCCTAAGTTTGCATCTCCCA
scaffold434661_17.[illegible]	1	p3	(ATA)5	15	1088	1102	AGACGTAAATGGGCGTGAAT
scaffold435475_16.[illegible]	1	p3	(TTC)5	15	37	51	CCTATTACTTCCTCGACTTTAATGG
scaffold435553_16.[illegible]	1	p3	(ATG)5	15	751	765	GATATGCAAGCAGCCAGTGA
scaffold435580_20.[illegible]	1	p3	(GAG)5	15	206	220	TGGTACCGATCTGGTGCATA
scaffold435621_13.[illegible]	1	p3	(AAC)5	15	161	175	CTAGAGGGCGGCACATTTAC
scaffold436624_16.[illegible]	1	p3	(ATA)5	15	134	148	TCTTCCGCTTGCTTGTTCTT
scaffold437143_10.[illegible]	1	p3	(ATT)5	15	403	417	GCTCTGGGTAAACTCACGGA
scaffold437378_17.[illegible]	1	p3	(ATA)5	15	441	455	TTCCATGCAATAAGCATCTATGA
scaffold437400_17.[illegible]	1	p3	(AAT)6	18	513	530	TCACCAAAAATCGAACACACA
scaffold437468_16.[illegible]	1	p3	(CAT)5	15	2717	2731	GGCTACAAAGGGACATCCAA
scaffold438063_15.[illegible]	1	p3	(ATC)5	15	75	89	CCACATTTTCCACCTTAGCC
scaffold438124_11.[illegible]	1	p3	(CCG)8	24	376	399	CTCTCCTGCCTCCGACTATG
scaffold438443_14.[illegible]	1	p3	(TCA)5	15	191	205	AGGCGGGTACACCACACTAC
scaffold438464_13.[illegible]	1	p3	(TGA)5	15	485	499	AAGATTACGACGGCGATACG
scaffold438570_15.[illegible]	1	p3	(ATC)5	15	775	789	CTCCCATCATCCTTTTTCCA
scaffold438846_16.[illegible]	1	p3	(GGA)6	18	269	286	TATGCGACCACGATCAAGAG
scaffold438946_13.[illegible]	1	p3	(ATA)5	15	278	292	CTCTAAGGGATGAATGCACG
scaffold439137_16.[illegible]	1	p3	(ATC)6	18	50	67	CATGCGCTCAGCATAAAGAA
scaffold439238_17.[illegible]	1	p3	(TTC)5	15	30	44	GGATCAGCAGTGGCTTTCTC
scaffold439445_17.[illegible]	1	p3	(TGT)5	15	2634	2648	TTGGGATTAAGGCTTTGGTG
scaffold439623_11.[illegible]	1	p3	(ATT)6	18	444	461	AAACTGCCAAGTGCCAACAT
scaffold439781_11.[illegible]	1	p3	(ATG)5	15	485	499	AACCGTCTCAGTTGTTCGCT
scaffold440287_13.[illegible]	1	p3	(ATC)5	15	975	989	CAAGACCTTTTTGTTCGCTT
scaffold440641_17.[illegible]	1	p3	(AAG)5	15	2298	2312	AGGTTCGAGCTACCGAAACA
scaffold440740_11.[illegible]	1	p3	(CAA)8	24	382	405	TTTGCTGGATACCGCAAGTA
scaffold440859_14.[illegible]	1	p3	(GGT)5	15	515	529	GAGGGAGAGAGAGGGGAAGA
scaffold440912_11.[illegible]	1	p3	(AAT)5	15	1127	1141	GCACATATTCGTTCCCGACT
scaffold440998_15.[illegible]	1	p3	(TAT)6	18	4517	4534	TTACGCGGGAACCTACAGAC
scaffold441003_12.[illegible]	1	p3	(GCG)6	18	1062	1079	ATGGAGGAGGAGGAAAAGGA
scaffold441074_16.[illegible]	1	p3	(TGT)5	15	2694	2708	TCGTTCAATTCACACACGTTC
scaffold441075_17.[illegible]	1	p3	(GCG)5	15	412	426	TGAGGGTTAGAGATCGGTGG
scaffold441392_13.[illegible]	1	p3	(TTG)7	21	157	177	GGTGGCTGAAGATAGGAATGA
scaffold442095_16.[illegible]	1	p3	(AGG)5	15	1363	1377	AGCAGAGTTTGTTCGTCGGT
scaffold442546_20.[illegible]	1	p3	(ATC)5	15	703	717	TGCGGAAGTAGCTTTGATGA
scaffold442620_12.[illegible]	1	p3	(TAT)6	18	955	972	GAGCCATTGATGGCATTTTT
scaffold443169_17.[illegible]	1	p3	(ACA)5	15	1165	1179	CAGAAAGCGCTTCGAGAACT
scaffold443522_17.[illegible]	1	p3	(GCA)5	15	794	808	TCGGTTTCGGAGGTATTGAG
scaffold443807_13.[illegible]	1	p3	(TAA)7	21	72	92	TGTTTGGATTTCATGAATTAGTATTTT
scaffold444527_20.[illegible]	1	p3	(ATT)7	21	428	448	TCAAGCTTGGGTGTTGATGA
scaffold444570_15.[illegible]	1	p3	(GCA)6	18	1519	1536	CTGACCACCATCTTCTCCGT

scaffold444771_14.9	1	p3	(TCC)5	15	84	98	CGCTCCTCCAAGAACAACTC
scaffold444925_17.2	1	p3	(GGC)5	15	388	402	GCTGTTTATCACGACGAGCA
scaffold445355_18.5	1	p3	(ACG)5	15	467	481	ACTAGCGAAAAACGGACGAA
scaffold445537_12.0	1	p3	(TTG)5	15	510	524	AAGGTCAATATGCGGTGGAG
scaffold445576_21.9	1	p3	(TCC)5	15	126	140	CTCCTTCCAAATCCAATCCA
scaffold445580_11.2	1	p3	(CTA)5	15	67	81	CCAACCAAATTGGACTTGTG
scaffold445586_14.3	1	p3	(CCA)5	15	2638	2652	CCTTTCAGGCTTGAATCTCG
scaffold445852_17.8	1	p3	(TGA)6	18	45	62	CCCAAGACCCCAATTTGATT
scaffold445868_17.4	1	p3	(CAT)5	15	2146	2160	CGCCTCTTGATTAACCTCCA
scaffold446294_11.8	1	p3	(TCT)6	18	324	341	GTTTGGTCCCTTTCGAATCC
scaffold446309_10.7	1	p3	(AAC)5	15	81	95	GGGTTAGTTATGGAACGATGC
scaffold446925_17.1	1	p3	(ATT)5	15	384	398	CATGTCACAACATGGAATCTCA
scaffold446948_14.9	1	p3	(ATC)6	18	1968	1985	CAGGATGGTTTGAAATGAGG
scaffold447024_11.0	1	p3	(CGG)5	15	230	244	TAATTTATCGATGGCGGACG
scaffold447151_19.0	1	p3	(TAT)5	15	67	81	TTGATTGCTTTAAATCCGGC
scaffold447256_11.4	1	p3	(AAT)5	15	117	131	TTTAAAGTTGCCATTTCAGCC
scaffold447391_19.2	1	p3	(TTA)5	15	214	228	GCCAAAATCTGTATACCCCG
scaffold447611_12.1	1	p3	(ACA)5	15	379	393	AACCTTTTTGAGCTCCGCTT
scaffold447880_17.1	1	p3	(CTT)6	18	4038	4055	CATGCATGACTTGGTGCTCT
scaffold448140_14.2	1	p3	(TTG)8	24	970	993	TTTGGTTCATGTAGCCCACC
scaffold448185_10.9	1	p3	(GCG)5	15	76	90	CGCTGAACGAGCTAGAACTTG
scaffold448317_20.5	1	p3	(AGA)5	15	40	54	AAGGAGAGGAAGATACGATTTGAA
scaffold449108_14.7	1	p3	(AAT)5	15	1335	1349	CCTCTCCATTCTGAATCCCA
scaffold449245_14.0	1	p3	(GGC)5	15	500	514	TTCTTTGTCGTGGTTTCGTG
scaffold449422_15.7	1	p3	(ATC)6	18	3391	3408	ACAGGAATTGAAATGACGGG
scaffold449695_17.7	1	p3	(TAT)6	18	46	63	TGCACAAAAATGTTTTAAAATTGC
scaffold449707_15.4	1	p3	(GGA)5	15	420	434	TAAGGATGGGTTTCCGAATG
scaffold449988_15.5	1	p3	(AAT)5	15	6175	6189	TGGATTTGGAATCGGTTTTC
scaffold450110_10.7	1	p3	(CAA)5	15	39	53	CAGCCATGAATGAGAGTGGG
scaffold450155_17.2	1	p3	(TGT)8	24	39	62	TGTAGCCGACCCCAATTTAT
scaffold450191_10.7	1	p3	(ATA)5	15	412	426	TCATTTATTTCCTTTTTATATGTGCAA
scaffold450206_16.1	1	p3	(TAA)6	18	177	194	TTCAAGTGTGCTTCCACCAA
scaffold450450_15.4	1	p3	(TTG)6	18	2128	2145	TAGCATTCGTGTTTCCAGCA
scaffold450521_11.7	1	p3	(TGA)6	18	40	57	CCCAAGACCCCAATTTGATT
scaffold451740_13.0	1	p3	(TAT)6	18	311	328	CCGATCCATTTTAGGAACGA
scaffold451795_15.3	1	p3	(TCA)5	15	319	333	GGCCAGTGTTGAGGTCAAAT
scaffold452041_16.6	1	p3	(GTG)5	15	191	205	AGGTTTGCCAATCGTTCTTG
scaffold452151_17.2	1	p3	(GAT)6	18	708	725	TTGATAAGCCAGCCACTTGA
scaffold452177_17.0	1	p3	(TTC)5	15	842	856	TGGGTAATGGCTTCAACTCC
scaffold452335_17.3	1	p3	(ATC)7	21	1164	1184	GTGCCACCTTTTAGCACCAT
scaffold452593_13.2	1	p3	(ATT)5	15	212	226	ACTGCATGGGCTATTTCCAC
scaffold452598_16.3	1	p3	(GAG)5	15	255	269	AGTAGAGGTGCCTGTGGTGG
scaffold453322_15.2	1	p3	(TAT)5	15	660	674	AAAGCAGTTAATAACGAAAAACCA
scaffold453374_16.0	1	p3	(TTC)5	15	276	290	ATTGTTGTTGCTGCTGTTGC
scaffold453474_20.2	1	p3	(ATA)6	18	48	65	ATGTTCCACCTCCCAAGGTT
scaffold453495_10.4	1	p3	(ATT)5	15	393	407	GACGGGTGTGGGCTAAAAAT
scaffold453707_10.2	1	p3	(ATA)7	21	376	396	CGTGTGCATGTGAAATTTTTAT
scaffold453730_16.3	1	p3	(GGT)5	15	173	187	TCCAAATCGCTCAAACATTG
scaffold453796_11.8	1	p3	(TTA)5	15	678	692	CCTAGACTCCGACGAAAAGC
scaffold453864_19.4	1	p3	(CTC)5	15	26	40	CACTCTCCTCAATGGCGTC
scaffold454028_14.0	1	p3	(TCA)8	24	2864	2887	TTGAGACTCAGATGAAATGTAGCC
scaffold454119_13.3	1	p3	(TAC)5	15	225	239	TTTATGCATTGCCGTGAAAA
scaffold454125_15.7	1	p3	(ATG)5	15	36	50	ATCAGGATCGAGTAAGCGGA
scaffold454537_12.4	1	p3	(AAT)5	15	790	804	ACTTGTGAGACAAAGAGAACAAAA
scaffold454628_19.4	1	p3	(AAC)5	15	565	579	TCCCTCATCACTACCCAACC
scaffold454729_15.0	1	p3	(ATC)5	15	86	100	TTCACATTTACAACCTACATCGG
scaffold455048_14.7	1	p3	(ATA)6	18	1881	1898	TTTTCGATTTGATTTGGGTTT
scaffold455436_17.1	1	p3	(CTT)6	18	412	429	TTATATGCCACTCTTCCCGC

scaffold455903_15.8	1	p3	(TGT)5	15	2580	2594	CCATCTTTCGAATGGTCGTT
scaffold456021_14.3	1	p3	(ATT)7	21	3164	3184	CAACGGCCTTTCTTTAGCAG
scaffold456170_13.7	1	p3	(CAT)5	15	1702	1716	ACATTTCTCTTGGGTGCCAG
scaffold456201_10.2	1	p3	(TAT)5	15	189	203	GCTAGACCATTGTAGGGCGA
scaffold456544_11.2	1	p3	(TAA)8	24	249	272	GCATTGGAAAATATTACTGGGG
scaffold456844_18.8	1	p3	(ACA)6	18	576	593	TACTTGGGACGGGATGGTTA
scaffold457138_16.0	1	p3	(GAG)5	15	3359	3373	GGCGAAGAATGCACACTACA
scaffold457617_14.9	1	p3	(TTC)5	15	3736	3750	TGTCGTCGTGGACAAGAAAA
scaffold457630_12.9	1	p3	(TTA)5	15	433	447	TCTTCTATACATAGTGCTTCCGGT
scaffold457709_13.4	1	p3	(ATA)5	15	323	337	GTTCGGTTCCGGTTTCAAAT
scaffold457714_15.0	1	p3	(CAT)5	15	608	622	CTTCCACAGGCATTGGGTAT
scaffold457742_11.2	1	p3	(CCG)5	15	62	76	CAAATCACCAATCAACAAAACC
scaffold458651_17.4	1	p3	(TGA)5	15	44	58	CCCAAGACCCCAATTTGATT
scaffold458875_17.0	1	p3	(GAA)5	15	1646	1660	ATGGTGAAAGGCTCCCTGTA
scaffold459137_13.7	1	p3	(TTG)5	15	362	376	GGTTGAGTTGAGGGGAATCA
scaffold459290_15.5	1	p3	(ATA)5	15	384	398	AACCCTCAGCAATATGCCAA
scaffold459314_12.4	1	p3	(TAA)5	15	639	653	CGACAGAGTTGCAAAGAAGTTG
scaffold459341_14.6	1	p3	(TTC)5	15	118	132	AATCCCACTCTTTTTGCTGC
scaffold459397_14.1	1	p3	(TTA)5	15	201	215	TCACGAGTTGGCTCTTTGTG
scaffold459510_18.7	1	p3	(GGC)6	18	234	251	CACCCTTCGATACTCCGAAA
scaffold459758_16.7	1	p3	(GAA)5	15	4141	4155	AGAAGGCAGAGACCGTGAAA
scaffold459783_10.6	1	p3	(AAT)6	18	46	63	CCGACTAGGACTAAGGGTCCA
scaffold459935_16.0	1	p3	(CAA)5	15	1435	1449	AAAGGCCATGCTGACAAAAC
scaffold459940_10.6	1	p3	(AAT)5	15	256	270	CAGCAACCACCAAATACTCTTG
scaffold459978_16.4	1	p3	(TAT)5	15	602	616	AAGATCAGGGAAAAGGGAGC
scaffold460000_16.6	1	p3	(GCA)5	15	80	94	CAGCTTGGTTTTGGAGGTGT
scaffold460486_16.8	1	p3	(ATA)5	15	445	459	ACGACCTGTCAAGACCCAAT
scaffold460616_14.5	1	p3	(TTG)6	18	66	83	TCCAAAATTAGTACAAGCATCAAAA
scaffold460921_12.9	1	p3	(CAT)5	15	127	141	TCGCCTTCTTTTGCAGCTAT
scaffold461024_15.0	1	p3	(TAA)5	15	4187	4201	TGTAAGCAGCACGCCAACTA
scaffold461087_18.5	1	p3	(GTG)5	15	283	297	ATGATCTCGACCTCCCTCCT
scaffold461143_17.0	1	p3	(TTC)5	15	3749	3763	CTGCACACCTGTATGTTCGG
scaffold461587_14.1	1	p3	(ATT)5	15	667	681	TGCTTGAAATGAGTTGGAATG
scaffold461764_10.2	1	p3	(ATA)5	15	397	411	GTCAGCGAGGCGAACTCTAC
scaffold461785_15.2	1	p3	(GAT)6	18	256	273	TTTCAAGGAAACATCCCAAAA
scaffold461811_17.4	1	p3	(CAC)5	15	681	695	TGTGACGTCTAGCTCGATCC
scaffold461839_12.5	1	p3	(TAA)5	15	427	441	TTTCCACAAAGCGTCGTACA
scaffold462337_17.9	1	p3	(TTG)5	15	280	294	GGGTTGGGTTTTCTCCATTT
scaffold462507_15.9	1	p3	(TCC)6	18	4338	4355	CAGATGGTGAATGGAGGGAG
scaffold462817_14.9	1	p3	(GGC)6	18	10495	10512	GAGAGTAGGATTGGGGGAGG
scaffold462866_18.5	1	p3	(TTG)5	15	520	534	CACCGGTTTCAAGGGTAGAA
scaffold463049_17.1	1	p3	(ACA)6	18	181	198	TGCTTGCGGTATTGTTTTGA
scaffold463389_17.3	1	p3	(CAC)5	15	579	593	AAACCTCACCACAAACGGAG
scaffold463414_11.9	1	p3	(GTT)5	15	69	83	TGTTGTTGAAAAAGGCGACA
scaffold463990_16.5	1	p3	(CGG)5	15	4440	4454	TAGGGCTTTTGTGTGGTTCC
scaffold464123_10.1	1	p3	(AAT)6	18	378	395	GTCTCAATGGGGACATCTGG
scaffold464261_10.3	1	p3	(CGC)5	15	247	261	TTGAGCAAGAAGGGTGTTGA
scaffold464272_10.0	1	p3	(ATT)6	18	169	186	CCCAAATTTTCTTAAGCCCTG
scaffold464462_12.5	1	p3	(TGT)5	15	50	64	TGTAGCCGACCCCAATTTTA
scaffold464577_16.5	1	p3	(ATC)5	15	648	662	ATCCATCCATGCATTTTGGT
scaffold464717_15.2	1	p3	(CCG)5	15	2017	2031	ACCCATGGATCCAACTTCAA
scaffold465065_14.5	1	p3	(TGT)6	18	48	65	TGGTTCATGTAGCCTACCCC
scaffold465180_10.4	1	p3	(ATC)5	15	85	99	GAATGTCAAGATGAGAGATGGAAA
scaffold465220_20.0	1	p3	(ATT)6	18	911	928	CCGAACAACACTCAACTCCA
scaffold465291_13.0	1	p3	(CAA)6	18	68	85	GGGGTTGAGGCCACTATATC
scaffold465589_15.4	1	p3	(TTC)5	15	369	383	TTACTCCTCGAAAACAGCCG
scaffold465615_15.0	1	p3	(AAT)6	18	601	618	CGAAGCAATACACTTTTGAAGAA
scaffold466368_14.7	1	p3	(TCT)5	15	459	473	AGTTGGGATATTCGGGAAGG

scaffold466499_15.1	1	p3	(AAT)5	15	185	199	GCTCTTTCAATAAACAGAATCCA
scaffold467167_13.9	1	p3	(CGC)5	15	1360	1374	GCAGCGCATAACAAGTGTCT
scaffold467173_14.2	1	p3	(ATC)6	18	2783	2800	CTGGCAATTCAGCCAAAAGT
scaffold467192_15.4	1	p3	(GAA)5	15	1011	1025	TTCAAATTCCACAAACACGG
scaffold467223_15.4	1	p3	(AAG)5	15	1027	1041	ATACCGCAGATCCCTTTCCT
scaffold467260_14.3	1	p3	(AAT)5	15	81	95	TTGAATTCGGACATGAGCAA
scaffold467863_21.5	1	p3	(TTA)5	15	278	292	ACGTAGGCGGCTTTACTGTG
scaffold468075_13.0	1	p3	(TAT)6	18	281	298	CAGAAAGGTGTTGGTGGGAT
scaffold468474_13.1	1	p3	(TGA)5	15	50	64	CCCAAGACCCCAATTTGATT
scaffold468552_12.9	1	p3	(CAT)5	15	228	242	ATTTTATCCGGACGGGTTTC
scaffold468607_18.8	1	p3	(TTC)5	15	215	229	TCTGTTCCCCATAGGCACTC
scaffold468781_15.5	1	p3	(ATT)5	15	587	601	TTGTTACTAAGGGTTGCGGG
scaffold469198_15.7	1	p3	(AAT)5	15	132	146	TCATTTTGGGACGGAGGTAG
scaffold469397_19.4	1	p3	(GCG)5	15	805	819	AGAAAAACGAACCGCATTTG
scaffold469846_13.2	1	p3	(ATA)7	21	357	377	TGATATCCCACCCGATCACT
scaffold469870_14.2	1	p3	(ACA)6	18	89	106	GCACAAATTGAAAGCACACAA
scaffold469987_11.9	1	p3	(CTC)5	15	575	589	TTGGTATGATGGAATGGGGT
scaffold469988_15.2	1	p3	(TGA)6	18	40	57	CCCAAGACCCCAATTTGATT
scaffold470735_12.3	1	p3	(GTA)6	18	286	303	CAGCTGCTGTCATCATCGTT
scaffold470842_12.6	1	p3	(ATA)5	15	259	273	AGTTCCCCATTGCTGTTTTG
scaffold470965_17.6	1	p3	(CGG)5	15	492	506	TGAGCCTACATCTGTGCTGG
scaffold471329_15.9	1	p3	(GCT)5	15	315	329	GCCGGTTAATATGCATTTGG
scaffold471508_17.5	1	p3	(CGC)5	15	280	294	CGGAGAGCTCGGTAAATGAG
scaffold471609_16.2	1	p3	(TAT)5	15	126	140	AAGGAGCAGCTGTCTTTTGTG
scaffold471673_15.2	1	p3	(ACA)5	15	934	948	TCTGCGGGAAAGGTGTAATC
scaffold471755_18.1	1	p3	(AGG)5	15	1843	1857	GATTGGTTCAGCTATTCCGC
scaffold472169_18.6	1	p3	(CAT)5	15	3196	3210	GCTGGATTCTTGAGGTGAGC
scaffold472376_16.4	1	p3	(CTC)5	15	205	219	GAAGTAAGCCCGACCTCCTC
scaffold472958_16.5	1	p3	(TGC)5	15	131	145	TCGCAACTATCTGCACCATT
scaffold472961_13.0	1	p3	(TAT)5	15	87	101	ATGTTGAGCCCAACCCAAC
scaffold473050_13.1	1	p3	(TTA)6	18	761	778	AATCATTCGATTTGGCAAGC
scaffold473101_14.8	1	p3	(AGA)5	15	159	173	GGGAGGTTTCGTCACACAGT
scaffold473158_16.7	1	p3	(TGA)5	15	105	119	GCAACGGAAACAACCTCTTT
scaffold473194_15.7	1	p3	(AGT)5	15	1696	1710	AGCAGGTGAGGGTGAAAATG
scaffold473246_15.2	1	p3	(TAA)5	15	655	669	AAACAAGGCAATTGTGGGTT
scaffold473296_18.5	1	p3	(ACA)5	15	119	133	GCAGATAATCACGATTTAGTTGG
scaffold473333_11.8	1	p3	(CTT)5	15	368	382	CTAGCTCTGAGATCCCGGC
scaffold473869_14.7	1	p3	(TAT)6	18	80	97	CCATTGAGACTCGAACCCAT
scaffold474017_14.3	1	p3	(TAT)5	15	5132	5146	ACGTCGGCATTGCTAAAATC
scaffold474186_12.4	1	p3	(AAG)5	15	132	146	GAAGCCAGGGAAAGAATGAA
scaffold474266_13.0	1	p3	(AGG)5	15	520	534	AAGAAGTCCCTGGCTTGGAT
scaffold474294_12.7	1	p3	(ATT)7	21	378	398	TTGATGGCGTCGGTATGTTA
scaffold474449_16.2	1	p3	(AAT)6	18	328	345	ATTTTCGTCGCCCCTCTTAT
scaffold474608_14.5	1	p3	(TGA)5	15	67	81	GTTGCGTACATCTGACCCC
scaffold475012_15.1	1	p3	(TTA)5	15	1041	1055	GCGGGACCTAGAATGTTGAA
scaffold475614_16.4	1	p3	(TCT)5	15	172	186	CTGCCCTTAGCCGTTAGATG
scaffold475675_16.5	1	p3	(TCA)6	18	2766	2783	CCTTCTCACTGACCTGCACA
scaffold475690_15.6	1	p3	(CAG)6	18	287	304	TAATTTACGGCTCGGTTTCG
scaffold475886_17.7	1	p3	(TCA)6	18	2055	2072	AAATGGCCGAAGAGATTCCT
scaffold476073_19.7	1	p3	(GAA)6	18	257	274	GGTTTCAAATTGGCCAAACA
scaffold476219_16.6	1	p3	(AAG)5	15	536	550	ACGTGCCCTGTTCTCTCACT
scaffold476431_10.7	1	p3	(AAT)7	21	511	531	CCAAGTCCTCATCTCGTGGT
scaffold476465_10.4	1	p3	(ACA)8	24	1122	1145	TTCCTTGTGAAGCACAATCCT
scaffold476622_11.1	1	p3	(TTA)5	15	64	78	CCCATACATTTGTGGGGATAG
scaffold476894_13.4	1	p3	(ACA)5	15	855	869	ATCGTGACCAACCCAAACAT
scaffold477057_14.6	1	p3	(AGC)5	15	712	726	ACGATGTTTTGTTGCGATGA
scaffold477122_15.2	1	p3	(CGC)5	15	35	49	GTCTACCCCGCCAGATCC
scaffold477874_14.6	1	p3	(CCG)6	18	350	367	AACTCACCCACCTTCTCCCT

scaffold478116_16.[illegible]	1	p3	(CAG)5	15	233	247	AAAATGAACGAGCATCCAGC
scaffold478184_12.[illegible]	1	p3	(CTC)5	15	413	427	TTAGGTTTTGCCCAGGATTG
scaffold478253_12.[illegible]	1	p3	(AAT)5	15	339	353	CCAAAATCTCGGCTTCAATC
scaffold478539_12.[illegible]	1	p3	(AAG)5	15	257	271	AGTGTGCGCGATAGAGGAGT
scaffold478567_12.[illegible]	1	p3	(TCT)5	15	298	312	GGGTTATATCCCCGTCTACCC
scaffold478817_15.[illegible]	1	p3	(CAT)7	21	5804	5824	TGACTTATGCGTGCATGTGG
scaffold479031_10.[illegible]	1	p3	(TAT)5	15	199	213	CCCAGCGGAGAGCTATACTG
scaffold479032_15.[illegible]	1	p3	(TTA)6	18	43	60	TCACTCCGGATCAAATAGGC
scaffold479178_15.[illegible]	1	p3	(AAC)5	15	741	755	TCCATGACATTGCCTACGTT
scaffold479188_14.[illegible]	1	p3	(GAA)5	15	199	213	GATAAGCGGCTGAAGGTGAA
scaffold479586_10.[illegible]	1	p3	(TCT)5	15	119	133	TGCAGCAACTTCCTCTCCTT
scaffold479607_17.[illegible]	1	p3	(TAA)5	15	1877	1891	ATCCTGTGGTGGCATGTTTT
scaffold479648_14.[illegible]	1	p3	(AAT)6	18	835	852	TGAGCGCACTCGAAAAGTAA
scaffold479791_15.[illegible]	1	p3	(TAT)6	18	309	326	CAACTTTAGTCGTCGTCATCATC
scaffold479935_11.[illegible]	1	p3	(GAG)5	15	365	379	TTTCGCAGTATGCCTCAGTG
scaffold480413_18.[illegible]	1	p3	(CGG)5	15	447	461	TTCGATAACAAAAGGCCAGG
scaffold481031_13.[illegible]	1	p3	(ATC)8	24	712	735	CCATTCCGTTGAACATCAAA
scaffold481610_14.[illegible]	1	p3	(GGT)8	24	1645	1668	CAGCAGGAGTGGTGCAGATA
scaffold481762_11.[illegible]	1	p3	(ATT)6	18	201	218	TCTAACATGCCATGGAGCAG
scaffold481907_13.[illegible]	1	p3	(CAT)5	15	92	106	GGGTAACTGAGCCGAGTCAT
scaffold482065_10.[illegible]	1	p3	(ATA)5	15	341	355	CACTCCATCGGATCCAAGAT
scaffold482080_15.[illegible]	1	p3	(TTC)5	15	295	309	TGATGTTTAGGCTGCAGGAA
scaffold482589_18.[illegible]	1	p3	(AGC)5	15	134	148	CAAGAAGCTTGCCAACATCA
scaffold483159_16.[illegible]	1	p3	(GAA)7	21	444	464	TCAATTGATTTTCGTAATCCTGG
scaffold483495_15.[illegible]	1	p3	(CCG)5	15	458	472	TAGGACGATAATTCCCACGC
scaffold483745_15.[illegible]	1	p3	(CAG)5	15	1699	1713	GCATTAGTCCTGGTGGCATT
scaffold484141_11.[illegible]	1	p3	(AAT)6	18	130	147	ATGCAGTTGGAAAGCACCTT
scaffold484554_14.[illegible]	1	p3	(AAT)5	15	1298	1312	GTGGGGCCTTACACCAACTA
scaffold484643_13.[illegible]	1	p3	(CGG)5	15	57	71	CTCAATTTTTCCGGCGAAT
scaffold484733_17.[illegible]	1	p3	(CAA)6	18	247	264	GGACTGTGATGGGCTAGGAA
scaffold484775_18.[illegible]	1	p3	(GAG)5	15	685	699	TGCGGATTAGGGTAGCTTTG
scaffold484921_16.[illegible]	1	p3	(GGC)6	18	59	76	AAGAAACCAGCGACACATTG
scaffold484989_15.[illegible]	1	p3	(TAT)5	15	108	122	CACAAATGGGGTTGACTTCC
scaffold485281_14.[illegible]	1	p3	(CCA)5	15	166	180	ACCAAACGATGCAGATTTCC
scaffold485673_16.[illegible]	1	p3	(TCT)5	15	1877	1891	CAAATTTGATTGGCTCTGTGA
scaffold485799_19.[illegible]	1	p3	(GAA)5	15	2402	2416	GCCCTTACAAGACCATCCAC
scaffold485867_16.[illegible]	1	p3	(ATA)5	15	309	323	ATGCCGGATCGAATAATCAG
scaffold485980_17.[illegible]	1	p3	(CTC)5	15	297	311	CCAATATCTTCCTCCCCTCC
scaffold486634_12.[illegible]	1	p3	(TAA)5	15	741	755	TGTATGCCCTGCAAAACAAA
scaffold486696_16.[illegible]	1	p3	(AAG)6	18	3251	3268	TAGGCACAAATCTATCCGGG
scaffold486850_16.[illegible]	1	p3	(TGT)6	18	33	50	CCAATTTATTGGGATTAAGGCA
scaffold486856_11.[illegible]	1	p3	(AAT)6	18	390	407	CCTCATTTTGAACCCGAATG
scaffold487310_13.[illegible]	1	p3	(AAG)6	18	2433	2450	TCCCAATTCAGACATGCAAA
scaffold487539_15.[illegible]	1	p3	(ATA)5	15	1116	1130	GAAGCTGAGCAACGAGAAGG
scaffold487685_16.[illegible]	1	p3	(AAT)5	15	1529	1543	CGTTTCCAACACCGACCTAT
scaffold487987_12.[illegible]	1	p3	(GAT)5	15	113	127	CATTGGAGATGCCCTTAGGA
scaffold488314_16.[illegible]	1	p3	(ATT)6	18	3792	3809	ATTCCGAAACCTGCTGTTTG
scaffold488353_10.[illegible]	1	p3	(GCG)5	15	307	321	TGCAAGATTGAGCGAGATTG
scaffold488371_10.[illegible]	1	p3	(ACA)6	18	206	223	GCTAGGTTTGTCTTCTGGCG
scaffold488624_16.[illegible]	1	p3	(TAA)5	15	261	275	TCCTGAATGTCCCCACTGAT
scaffold488980_14.[illegible]	1	p3	(TTG)6	18	912	929	TCATCCAAACCTCAACCAGA
scaffold489047_15.[illegible]	1	p3	(ATA)5	15	344	358	TTCATGCCTGTATTTCAATCG
scaffold489629_15.[illegible]	1	p3	(TAA)5	15	3362	3376	AAGTGGGCCTTATCTTGCCT
scaffold490478_16.[illegible]	1	p3	(TTC)6	18	4033	4050	TAGGCCTCTTCTTCAGCAGC
scaffold490516_12.[illegible]	1	p3	(TAT)5	15	639	653	GAATATATCTTGCCGTTGCCA
scaffold490615_16.[illegible]	1	p3	(CTT)5	15	385	399	ATTTCACGTTTCACGCTTCC
scaffold490670_18.[illegible]	1	p3	(TAT)6	18	364	381	CGGACCAGCCTAGAATTTGA
scaffold491260_17.[illegible]	1	p3	(CAG)5	15	366	380	AGAATGGCGAAGAAGTGGAA

scaffold491581_13.(	1	p3	(TAT)6	18	35	52	CGAACCATAGTTGTTGGACG
scaffold492617_18.(	1	p3	(ATT)5	15	215	229	TGTCATGCATAATCGAACTGG
scaffold492647_14.8	1	p3	(TGG)6	18	272	289	CCCATGGACCAAAACTATGC
scaffold492671_13.4	1	p3	(AAT)5	15	190	204	GTGCCACCAAAAATCACACA
scaffold493036_11.5	1	p3	(CGG)5	15	314	328	CAACGAATTCCGCCATAACT
scaffold493080_14.2	1	p3	(TAA)5	15	444	458	CATGACAACCCTCTAGTTCGC
scaffold493265_12.9	1	p3	(TCT)5	15	68	82	TGTGGAACTCATCATCTAATCCA
scaffold493275_14.6	1	p3	(GCA)5	15	1000	1014	GCACAAATTGTTCCCATTCA
scaffold493357_14.3	1	p3	(TTA)5	15	632	646	TCGTACAAAGGAACTTGGGC
scaffold493381_14.2	1	p3	(CTT)5	15	227	241	ATCCCCCTACCCCAAACATA
scaffold493505_16.1	1	p3	(GCC)6	18	1046	1063	GGGTTTTACCGCCGTTATTT
scaffold493735_12.8	1	p3	(CTT)5	15	165	179	TGATTTCGCACGAGTGTTGT
scaffold493770_17.3	1	p3	(GAT)5	15	861	875	CCATTTCCGGTGGAAGAGTA
scaffold493776_10.1	1	p3	(GGT)7	21	368	388	TCTGGTGGGAGATCAAAAGC
scaffold493825_15.9	1	p3	(TAA)5	15	387	401	AGCAAGGCTTGGATCAATGT
scaffold494013_16.9	1	p3	(CCG)5	15	1152	1166	AGGCTTTGCATAGCTGTCGT
scaffold494083_13.5	1	p3	(AAC)6	18	305	322	TTCGATTCAAGGTCTCACGA
scaffold494386_15.3	1	p3	(AGA)5	15	1021	1035	GAAAATGGGTGTGACTTGGC
scaffold494669_12.8	1	p3	(CAA)6	18	398	415	GCGGTGGATTTAGGATTTCA
scaffold494685_16.3	1	p3	(TGA)5	15	74	88	TTACAAGGGAAAGGTTGCGT
scaffold494750_14.9	1	p3	(TTA)5	15	61	75	CAAATACAAATTCAAGGATTATACAC
scaffold495384_15.4	1	p3	(ATA)6	18	2154	2171	TTAAGTTGAAGGCCGCAATC
scaffold495568_10.9	1	p3	(ATT)6	18	67	84	AATTAAACCCAAACCAAACTCTT
scaffold495571_16.5	1	p3	(TTG)5	15	51	65	TTGGGATTAAGGCTTTGGTG
scaffold495606_15.2	1	p3	(AAG)5	15	4174	4188	GTTGCAAAACGACTGGGAAT
scaffold495626_17.3	1	p3	(ATT)6	18	204	221	ACCACAGCGACCGATAATTC
scaffold495979_15.9	1	p3	(ATC)6	18	4760	4777	TCCTCTTTCTTGGGTGATGG
scaffold496061_17.6	1	p3	(ATT)5	15	323	337	TTAGTCCGTTTAATCCGCCA
scaffold496109_15.7	1	p3	(TCT)5	15	1413	1427	GGCACACAATCATGGTGAAG
scaffold496403_15.(	1	p3	(AAT)5	15	3562	3576	GGGAGAGAGAGGGGAGTGTC
scaffold496428_12.7	1	p3	(ATT)5	15	531	545	ATCGCAATCTCTCGGACACT
scaffold496437_16.8	1	p3	(TAT)5	15	235	249	ATTGGGCCTGAGTTTTGATG
scaffold496453_15.(	1	p3	(AAT)5	15	188	202	CGCGGTTATTTATGTCCGTT
scaffold496818_11.8	1	p3	(TTC)5	15	968	982	GTCTAGCATCCTTTGGCAGC
scaffold496985_12.7	1	p3	(TGT)7	21	41	61	TGTAGCCGACCCCAATTTAT
scaffold497224_12.8	1	p3	(ACC)5	15	130	144	GGGTTTGTTCTAGTTCATCCCA
scaffold497349_15.8	1	p3	(GCA)5	15	99	113	GGCTGAGATGGACTACGAGG
scaffold497442_15.1	1	p3	(ATA)5	15	1809	1823	GGTGTTGAATGAAGGGAGGA
scaffold497624_17.(	1	p3	(ATA)5	15	265	279	TGATGGGTGAGTTTTATATGATGC
scaffold497651_14.4	1	p3	(ATC)5	15	1459	1473	AGCTGCAGATCACAGAGCAA
scaffold497753_14.(	1	p3	(ATT)5	15	360	374	TTGTAATCCTTCCCACCTCG
scaffold497814_14.2	1	p3	(TTG)5	15	345	359	ATGTAGCCGACCCCAATGTA
scaffold497819_13.8	1	p3	(CAA)6	18	195	212	CCCCCATTACTCACATCAGAA
scaffold497842_15.7	1	p3	(TAT)7	21	619	639	TGAAACCTTCTGCCCTCAAT
scaffold498002_14.2	1	p3	(AAT)5	15	173	187	AGCATGAGTACATACATTTCTAGCAT
scaffold498003_15.9	1	p3	(AAT)5	15	5763	5777	TTGGTGTAATTTTGGAAGGGA
scaffold498440_11.(	1	p3	(TCA)6	18	89	106	AAATCCCCAAAAGAACATGG
scaffold498448_13.1	1	p3	(GAG)5	15	138	152	AGCCTTTTGTGAATGATGGC
scaffold498544_15.2	1	p3	(TCT)7	21	2575	2595	ACTGCTTTCTCACAACGCCT
scaffold498821_12.(	1	p3	(GAA)5	15	183	197	GGATTCGATGTCTGCCAACT
scaffold498874_20.2	1	p3	(TAA)5	15	527	541	GGTTCATCGGGTCCTGAGTA
scaffold498926_10.4	1	p3	(ATT)6	18	398	415	ACTCAATATGCCCAAGGTGG
scaffold499225_11.7	1	p3	(GAG)5	15	67	81	GGGTCGTCGATTTGGACTC
scaffold499541_14.6	1	p3	(GAA)5	15	1046	1060	ATCCAAGGGCTCGATTCTCT
scaffold499777_14.4	1	p3	(GGC)5	15	942	956	GCAGAAAGCGTTTTAGGTCG
scaffold500183_17.2	1	p3	(TTC)5	15	213	227	TTGTCCAAGTGGGAGATTGTT
scaffold500383_17.1	1	p3	(TTC)5	15	478	492	TGGATGAAGGGGTTGTTGAT
scaffold500570_12.5	1	p3	(TTA)5	15	115	129	TTTCAAATACCTTGACCAACGA

scaffold500689_17.	1	p3	(GCA)5	15	481	495	CAAGGCATATAAGCTGGGGA
scaffold501061_13.	1	p3	(ATA)5	15	1172	1186	GCTTGTAGACGCTTTACGCC
scaffold501094_15.	1	p3	(ATT)5	15	166	180	TGTGTTGTATGCGTTCATGACT
scaffold501158_15.	1	p3	(TAA)6	18	310	327	CACCGCCTTAATTTCTCCAG
scaffold501233_12.	1	p3	(TTG)5	15	190	204	CTTTATGATGGAGGCGGATT
scaffold501294_15.	1	p3	(CTT)7	21	365	385	CTCGACACAGTTCATCGGAA
scaffold501366_17.	1	p3	(ATC)6	18	2102	2119	TGCCTCATGTAATGGTGGAG
scaffold501811_15.	1	p3	(CAT)5	15	4343	4357	TGCCTGTAACAAGGGTGGAT
scaffold502014_15.	1	p3	(ACA)5	15	1163	1177	GTCGGGGTAATCCGACCTAT
scaffold502577_15.	1	p3	(ATT)6	18	3775	3792	TTGACCGTACTGGTGATGTACC
scaffold502663_14.	1	p3	(TCT)6	18	833	850	TGAAAACTCCGGTTCCATTC
scaffold502895_14.	1	p3	(CTT)7	21	242	262	TCATCGAATCAAATCCATCAA
scaffold503301_14.	1	p3	(TCT)5	15	928	942	CCATTTTCCCATCAACATCC
scaffold503573_15.	1	p3	(ATT)5	15	183	197	TTACTATGCGGACTGGGACC
scaffold503629_13.	1	p3	(ATT)6	18	151	168	AAGGGAGCTACAACCTTTCATC
scaffold503774_16.	1	p3	(GGC)5	15	530	544	GGAGACAGCGGCTAGTAACG
scaffold503777_15.	1	p3	(TGT)5	15	212	226	GCTACGAGGCAGATAAACGG
scaffold503861_18.	1	p3	(GCA)5	15	718	732	TAGTACCGTGGGGCAGATTC
scaffold503994_13.	1	p3	(AAT)5	15	386	400	GTTTGTCGATCATTACGCCC
scaffold504271_12.	1	p3	(AAT)5	15	729	743	TTCATGTTTCGCGATTCAAC
scaffold504489_19.	1	p3	(ACA)6	18	1120	1137	CATAGGGTTGCTGATGCAGA
scaffold504850_14.	1	p3	(TCA)5	15	51	65	GAATGGCATGTGGATCTGTG
scaffold504868_15.	1	p3	(ATA)5	15	343	357	ATTTGTTCAAAATCCGCGAC
scaffold505305_11.	1	p3	(AGC)5	15	67	81	TTACAGCTTGGTTTCGGAGG
scaffold505480_14.	1	p3	(ATT)8	24	1007	1030	GCATAGGATATTTATTACACCACGG
scaffold505906_15.	1	p3	(TCC)6	18	4062	4079	GCCCTCAAATCTGACCAAAA
scaffold506257_10.	1	p3	(ATC)6	18	543	560	TCATCACCACCACCATCATC
scaffold506391_15.	1	p3	(CGG)5	15	926	940	CAATTTGATTGTGCGATGGT
scaffold506560_13.	1	p3	(GAA)5	15	205	219	CAATTCCCTTGGATCCTGAA
scaffold507037_18.	1	p3	(ACA)5	15	212	226	AATTGGCCCTCATTTCCATT
scaffold507173_19.	1	p3	(CAG)5	15	86	100	TGCTCATCAAAAAGTTCCCA
scaffold507338_15.	1	p3	(TGA)6	18	991	1008	ACCCCCTGTTTCTGGATCTT
scaffold507379_10.	1	p3	(TAT)5	15	316	330	TGTACGGTGTAATGCTTGTGC
scaffold507492_16.	1	p3	(ATA)5	15	475	489	CTTGTAATCCGCATCCTCGT
scaffold507582_14.	1	p3	(ACA)7	21	438	458	CACGAGATGGGGACTCTTGT
scaffold507627_19.	1	p3	(CGG)5	15	507	521	ACAAGTATCCACCACTCGCC
scaffold507780_10.	1	p3	(GAA)5	15	29	43	CCTCCTCTATTGCTGATTTAAGGA
scaffold507899_15.	1	p3	(GTT)6	18	545	562	GGCGTTTCTTTGTTGAGAGC
scaffold507988_16.	1	p3	(TCA)1	30	1005	1034	AATTGTGCTCCATTTCCCAG
scaffold508421_16.	1	p3	(CTT)5	15	405	419	TCATCGAATCAAATCCATCG
scaffold509085_13.	1	p3	(AAG)5	15	245	259	CTGGTCCACTTCCAGCTTTC
scaffold509152_14.	1	p3	(GAG)5	15	254	268	ATTCTACGGTGATTCGTCGC
scaffold509447_16.	1	p3	(ATC)6	18	1028	1045	AGGCTTGCTCAACCAAATCA
scaffold509521_12.	1	p3	(GAG)6	18	437	454	CTTTTCAGGCCCATTTCTCA
scaffold509722_15.	1	p3	(TGA)5	15	49	63	CCCAAGACCCCAATTTGATT
scaffold510321_12.	1	p3	(AAT)5	15	470	484	TTCTCAGTGCTATTTTGTTCCG
scaffold510335_14.	1	p3	(CGC)5	15	300	314	AGGAGAAGAAAGCCGAGGAG
scaffold510527_17.	1	p3	(GAA)6	18	352	369	AGGAGTTTTCCTCTTTCCGC
scaffold510692_14.	1	p3	(ATT)5	15	769	783	CATGCAAGACAACAAGGCAC
scaffold510895_15.	1	p3	(TAT)5	15	396	410	TCCATCTGTCATCTCATCCA
scaffold511001_16.	1	p3	(AGG)5	15	476	490	GCAGCCACGATTCTCAAGAT
scaffold511248_13.	1	p3	(ACC)5	15	636	650	CGTTCTAAGGCGTGCGTATT
scaffold511303_13.	1	p3	(TGG)5	15	58	72	AGCTTTGGTTCATGTAGCCG
scaffold511557_11.	1	p3	(GCG)6	18	489	506	GGCCAGCTATACAAATTCGC
scaffold511714_10.	1	p3	(ATT)6	18	343	360	ACCCTAATCAACATCTGCCG
scaffold511725_15.	1	p3	(GCT)5	15	52	66	CGTCATCGTGACGTCTGC
scaffold511745_19.	1	p3	(CCA)5	15	417	431	CTCGTGTCACCAGGTCAAAA
scaffold511889_17.	1	p3	(TTG)5	15	52	66	CGACCCACATAGTGGGAAAA

scaffold512814_11.4	1	p3	(TGC)5	15	36	50	TCTCTTCCATCCAATAATTCCTGT
scaffold512912_15.2	1	p3	(ACA)5	15	445	459	TGTGACTGCATAAAGGCCAA
scaffold513228_11.6	1	p3	(TAA)5	15	408	422	CCACCAAGAATTTGTGCCTT
scaffold513615_11.9	1	p3	(CCT)7	21	1189	1209	TGTTTCCCTTTCCCTTCCTT
scaffold514041_16.6	1	p3	(TCC)6	18	104	121	TTTCAAAAGATGAGCTACTAGCAA
scaffold514110_15.7	1	p3	(CGG)5	15	357	371	CCTTTAACCAAAAGTGCCCA
scaffold514195_16.0	1	p3	(CTG)5	15	1399	1413	GATTCTTGCTAACATCTCTGCG
scaffold514417_16.3	1	p3	(ATT)7	21	3253	3273	GCCACAATAACTTGTGAAACCA
scaffold514430_10.6	1	p3	(TAT)5	15	172	186	ACCACGTTGGGATCTTGGTA
scaffold514431_10.0	1	p3	(ATC)6	18	631	648	CCTTTTTCTGTTTCTTGCGG
scaffold514552_17.0	1	p3	(ATA)5	15	605	619	TGGGAAAAGAAAATGGGAAA
scaffold514739_14.0	1	p3	(TAA)5	15	376	390	TGTTGTATGGTGAAGCGTCC
scaffold514975_10.5	1	p3	(GAA)5	15	326	340	GCTGAAAGAACTGAGGACGG
scaffold515030_19.9	1	p3	(ATA)5	15	1129	1143	TGTTCACACACCCCACCTTA
scaffold515492_15.6	1	p3	(ATC)6	18	980	997	CATTTCAACACAAGGCAAGC
scaffold515548_12.4	1	p3	(AAT)6	18	477	494	CGTGGATATAACTCGGCCAT
scaffold515701_17.3	1	p3	(TCT)5	15	1658	1672	CCCCATATGAATCCTTTCCA
scaffold515886_12.1	1	p3	(TGT)6	18	43	60	ATGTAGCCGACCCCAATTTA
scaffold516263_16.3	1	p3	(ATC)6	18	2304	2321	TGTACAATGACCGTGCCTGT
scaffold516296_12.5	1	p3	(CCG)5	15	225	239	TGTTGAGGAGTAGGGTTGGG
scaffold516472_14.4	1	p3	(GTG)5	15	365	379	GGGGTCCTTGTCGTATAGCA
scaffold516483_19.1	1	p3	(TGC)6	18	509	526	TGCCTTCTGAATGATGGACA
scaffold516719_12.0	1	p3	(TTC)5	15	239	253	GTCGCTGTTGATGTTCCAGA
scaffold517207_15.2	1	p3	(TCT)5	15	99	113	TTGATGTAGATATGGAGCGATTTT
scaffold517279_16.7	1	p3	(ATG)5	15	1638	1652	ATCAAGGGCCAGTCTGCTTA
scaffold517431_17.0	1	p3	(CCG)8	24	595	618	ACAGTGGAGACGGAATCGAC
scaffold517761_16.2	1	p3	(AGA)5	15	1178	1192	TTGGGAAGTTTCGTCACACA
scaffold518022_16.2	1	p3	(AGC)5	15	2047	2061	GGTAGCCGAGCAGCTTCTAA
scaffold518273_16.0	1	p3	(CGG)6	18	6007	6024	TTCGGAGGGCAACTCATAAC
scaffold518294_15.6	1	p3	(TCA)6	18	65	82	CGATGAAGCTCGGAGAAGTT
scaffold518433_15.8	1	p3	(CAT)5	15	1547	1561	TCCTGCAGTTTTTGTGGTTG
scaffold518493_10.8	1	p3	(TGG)5	15	258	272	TTTGGAGGGGTTTAGGTTCA
scaffold518587_14.4	1	p3	(TTA)5	15	277	291	TCGGGCTTTGATTTCAAGAT
scaffold518679_17.0	1	p3	(CAG)5	15	2219	2233	TGCAATTTCAGGCACATCAT
scaffold518703_14.5	1	p3	(GGT)5	15	1275	1289	TCTGGTTCGGATTGACATGA
scaffold518821_15.6	1	p3	(AAT)5	15	71	85	TTCAGAATCATTTACTTCCAAGTTT
scaffold519098_11.5	1	p3	(GAA)6	18	181	198	CTCCTGCTTCACCTCGATTC
scaffold519185_12.1	1	p3	(CGG)5	15	282	296	AGAGAGAACAGATGCGAGGG
scaffold519345_17.2	1	p3	(CTT)5	15	1253	1267	GTTGCCACAATTTCCTGCTC
scaffold519913_14.4	1	p3	(GGC)6	18	64	81	GGTGTAGATCTGGCGGGTAG
scaffold520527_13.6	1	p3	(TTA)8	24	258	281	TCCGAGGTCAGCAATTCTTC
scaffold520626_11.1	1	p3	(AAT)5	15	269	283	CAAAAAGGAACGCAAAAACAA
scaffold520840_20.2	1	p3	(CGC)7	21	140	160	TATCAAAACTGGATTGGCCC
scaffold521749_15.5	1	p3	(TTC)5	15	342	356	GGCGATTGTCAACTTGGTTT
scaffold521811_12.6	1	p3	(AAG)5	15	1468	1482	ACATGCAAGGAGTGAAAGGC
scaffold522153_15.5	1	p3	(ATT)5	15	222	236	GGGGCGTGACATTAACACTA
scaffold522367_10.4	1	p3	(TTC)6	18	90	107	GCCGGAAGAAAAGACACAAG
scaffold522523_13.0	1	p3	(CAG)5	15	314	328	ATCCGCAGTCAAAACGTACC
scaffold523135_16.2	1	p3	(CCG)6	18	391	408	CCCAACCTTTTACTCCTCCC
scaffold523314_10.1	1	p3	(TGA)6	18	76	93	CCCAAGACCCCAATTTGATT
scaffold523389_17.4	1	p3	(TTA)5	15	1009	1023	CGCAAGATGTTTCAGGGATT
scaffold523450_15.7	1	p3	(CAC)5	15	709	723	AATGTTTCTACGCATTGGGG
scaffold523632_14.9	1	p3	(AAT)5	15	66	80	GCAAAATCTCAATTGTAGTGCC
scaffold523640_13.7	1	p3	(CAT)5	15	328	342	ACATGTGGTGGCAGGTTACA
scaffold523697_10.7	1	p3	(CAT)5	15	297	311	TAGCCTGCTCAGCCTCTCTC
scaffold523770_16.4	1	p3	(GGC)5	15	216	230	ATACTGGGCTTCTCCGACCT
scaffold523794_16.8	1	p3	(GGT)5	15	648	662	TGAATCGGGACAGCATGTTA
scaffold523975_15.4	1	p3	(GCG)6	18	2329	2346	TGTAGCCCGTGACTGTACCA

scaffold524247_16.[illegible]	1	p3	(TAT)5	15	44	58	ACCCTCACTAGTTTCGGTTAGA
scaffold524451_15.[illegible]	1	p3	(AAT)6	18	872	889	ACAACCAAGCAGGTCCAAAA
scaffold524470_19.[illegible]	1	p3	(TCA)5	15	112	126	CGATACCCATAACGGGTGAG
scaffold524568_13.[illegible]	1	p3	(TTC)5	15	168	182	CATGGTTTTGGGAGGAGAGA
scaffold524730_19.[illegible]	1	p3	(CCT)6	18	432	449	TGTCAATGCATCTGCCTCTC
scaffold525239_10.[illegible]	1	p3	(TAT)5	15	134	148	AGAAAATGCTGGAAATGAGGA
scaffold525242_17.[illegible]	1	p3	(TGG)5	15	1278	1292	ACGGCTAAAATCAGGAGCTG
scaffold525365_16.[illegible]	1	p3	(TCA)8	24	855	878	TAAATCCTTCGAGGTCACCG
scaffold525366_10.[illegible]	1	p3	(GGA)5	15	77	91	GAATCAGTGCTTGCCGATCT
scaffold525873_11.[illegible]	1	p3	(AAG)5	15	273	287	GTGGAAGCATCAAAGCAGGT
scaffold526130_10.[illegible]	1	p3	(GCT)5	15	52	66	GCGTTATCGTGACGTCGG
scaffold526931_18.[illegible]	1	p3	(TTA)5	15	2679	2693	CTTTTTGTTTTGGAGTGGGG
scaffold527008_17.[illegible]	1	p3	(TAT)7	21	2541	2561	AATGCGATAGCCTTGCCTTA
scaffold527011_11.[illegible]	1	p3	(CAA)5	15	135	149	CCCGGATCCACTACAAGTTC
scaffold527183_15.[illegible]	1	p3	(CCG)7	21	1230	1250	TGCATCTGAATACACGAGGTTC
scaffold527231_16.[illegible]	1	p3	(GAT)7	21	995	1015	AATGCCAACACAACCAAACA
scaffold527324_16.[illegible]	1	p3	(AAT)6	18	1894	1911	ATGTTCATCAATTGCAGCCA
scaffold527554_12.[illegible]	1	p3	(ATT)5	15	347	361	TTCGACCCCAACTCAATAGC
scaffold527633_14.[illegible]	1	p3	(TGT)5	15	519	533	TAGCCGACCCCAACTTATTG
scaffold527876_17.[illegible]	1	p3	(CAT)5	15	467	481	TGAAACCCCATGATTTCCAT
scaffold527938_15.[illegible]	1	p3	(CTC)5	15	1180	1194	TGCACATTTTCCATTGAAGG
scaffold527943_13.[illegible]	1	p3	(TTA)6	18	136	153	TGTAAAATTTCCAAGGCAGAGA
scaffold527991_15.[illegible]	1	p3	(TCT)5	15	474	488	AATTGGCATTTTGTAGGCTCA
scaffold528041_17.[illegible]	1	p3	(TTG)5	15	57	71	AGCTTTGGTTCATACAGCCG
scaffold528206_10.[illegible]	1	p3	(TAA)5	15	1509	1523	CACCCGGAACTCGAAAAAT
scaffold528233_14.[illegible]	1	p3	(ATC)5	15	2989	3003	ACAAAAGCAAACCGCTAACC
scaffold528814_16.[illegible]	1	p3	(TCA)5	15	610	624	ACCGGACACAATACTGCACA
scaffold528816_13.[illegible]	1	p3	(TGT)5	15	45	59	ATGTAGCCGACCCCAATTTA
scaffold528832_12.[illegible]	1	p3	(TGA)5	15	51	65	GACCCCAATTTGATTGGAAA
scaffold528844_16.[illegible]	1	p3	(CAC)5	15	309	323	GCGAACTGACAACGAGATCA
scaffold528912_19.[illegible]	1	p3	(CCA)6	18	271	288	GTTGGTTCAGGGTTAGCAGC
scaffold528994_12.[illegible]	1	p3	(TTG)6	18	49	66	TTGTATTGTCTTGATTGCAGGAA
scaffold529027_17.[illegible]	1	p3	(GAT)6	18	51	68	GGTACGTTACTGTAAAACCCTGA
scaffold529220_18.[illegible]	1	p3	(AAT)5	15	51	65	CAGAGCGCACTCGAAGAGTA
scaffold529222_15.[illegible]	1	p3	(TGA)5	15	4189	4203	CAGAGGCACAGATTAGCAGC
scaffold529239_18.[illegible]	1	p3	(TCC)5	15	686	700	TACTGCTGATTCAAGTGGCG
scaffold529245_11.[illegible]	1	p3	(ATT)5	15	53	67	GCTAACCATTTACTTGCACATTTT
scaffold529357_15.[illegible]	1	p3	(ACA)5	15	297	311	AAAGCATTCACGTAACGGGT
scaffold529509_13.[illegible]	1	p3	(TCA)5	15	2543	2557	AACACTATCCCACAAATCACCA
scaffold529541_11.[illegible]	1	p3	(TTA)6	18	98	115	TCGATGTATTGTTTGGATAATATGCT
scaffold529672_17.[illegible]	1	p3	(AAT)6	18	399	416	ATTCGGATTGCATCCAAGAA
scaffold529862_16.[illegible]	1	p3	(GGC)5	15	1815	1829	TCCGTCATTTTCCCACTCTC
scaffold529953_15.[illegible]	1	p3	(TTG)5	15	59	73	TGCATTCAACATCATTACAAAGTC
scaffold530361_13.[illegible]	1	p3	(AAT)6	18	47	64	GGACTTTGGTGTGAGAAAATTG
scaffold530424_12.[illegible]	1	p3	(AAG)5	15	96	110	TGAATTTCACCAATCGGGAG
scaffold530500_13.[illegible]	1	p3	(TGT)7	21	214	234	TGAGATTCCCGCATCCTATC
scaffold530557_16.[illegible]	1	p3	(CAT)6	18	1936	1953	CGCCCGACTGAATTATCATC
scaffold530719_16.[illegible]	1	p3	(GAT)6	18	749	766	TGAAGATTCGTGCGATGTGT
scaffold530838_15.[illegible]	1	p3	(GAA)5	15	531	545	AGGTTTTCGGCGGAGATTAT
scaffold530931_15.[illegible]	1	p3	(TGA)7	21	43	63	CCTCAAGACCCCAATTTGAT
scaffold531584_17.[illegible]	1	p3	(AAT)5	15	1172	1186	TCCCAAATGATTTCAAAATGG
scaffold531681_16.[illegible]	1	p3	(AAT)5	15	823	837	GCAGAAGAGAAGGTGGGAGA
scaffold531990_12.[illegible]	1	p3	(TAT)5	15	107	121	CGAGAGAGAGAATGAGTGGTGA
scaffold532019_14.[illegible]	1	p3	(TCA)5	15	104	118	CCCAGGCTCAAATTCCTACA
scaffold532201_10.[illegible]	1	p3	(TTC)5	15	399	413	TTGTTGATGGTTGAGAGGTGTC
scaffold532317_15.[illegible]	1	p3	(TTG)5	15	891	905	AGAATCACAATGCTCCTGGC
scaffold532581_13.[illegible]	1	p3	(ATT)6	18	413	430	AAAATGATGATTCCGGGTCC
scaffold532616_17.[illegible]	1	p3	(AAT)5	15	622	636	TCAATATGAATTGATCGCCG

scaffold532628_14.[illegible]	1	p3	(TAA)8	24	399	422	ATTGCTCGATCCACCTCTTG
scaffold532879_16.[illegible]	1	p3	(TAT)6	18	412	429	TCAAATTGTGTCGAGGCTAATG
scaffold532982_16.[illegible]	1	p3	(GTG)6	18	3291	3308	GTCTAAAATTCGGCAGCGAC
scaffold533052_16.[illegible]	1	p3	(CCG)5	15	1171	1185	GGACTCCCATCGTCTCCATA
scaffold533134_10.[illegible]	1	p3	(AAT)5	15	282	296	GAGCTGAGCAATGAGAAGGG
scaffold533461_15.[illegible]	1	p3	(TTA)7	21	43	63	TGAAGTTTCGAAGACTGGAAGA
scaffold533689_11.[illegible]	1	p3	(AAT)7	21	424	444	TCACCGTTGTCATAGCCAAA
scaffold533873_16.[illegible]	1	p3	(CGC)5	15	516	530	AATATTGGCTTCCCCAAACC
scaffold533989_15.[illegible]	1	p3	(TGT)5	15	46	60	ATGTAGCCGACCCCAATTTA
scaffold534026_12.[illegible]	1	p3	(AAT)5	15	335	349	TGAAAACGATTTAAACGTGGG
scaffold534232_15.[illegible]	1	p3	(TGT)5	15	4596	4610	CAAATCATGGAAACAGCACG
scaffold534588_20.[illegible]	1	p3	(TCT)6	18	890	907	AGGTCGGCCCTGTAGAATTT
scaffold534628_17.[illegible]	1	p3	(TTG)5	15	914	928	TGCATACCAAGTTATTGGCG
scaffold535278_12.[illegible]	1	p3	(TCT)7	21	1028	1048	CAAAACGCCTCCAATTTTGT
scaffold535391_16.[illegible]	1	p3	(ATG)5	15	842	856	CATCGACTTCCACGACAAGA
scaffold535480_10.[illegible]	1	p3	(ATC)6	18	66	83	CTGCCATCTGGTACAACTGC
scaffold535533_17.[illegible]	1	p3	(TTG)5	15	1159	1173	CACCGGTTAGGTGGGTAGAA
scaffold535844_16.[illegible]	1	p3	(AAT)5	15	341	355	TGTGTTGCTGAAGGAGTTCG
scaffold535895_15.[illegible]	1	p3	(TGT)5	15	47	61	TGTAGCCGACCCCAATTTAT
scaffold535965_17.[illegible]	1	p3	(TCA)6	18	107	124	TGATCACAAACTTAGCGGACA
scaffold535992_16.[illegible]	1	p3	(AAT)6	18	639	656	TGCTTATTTGGATTGGCCTC
scaffold537445_15.[illegible]	1	p3	(TAT)5	15	1046	1060	ATTGATCGAGACCGAACCAC
scaffold537507_20.[illegible]	1	p3	(GAT)5	15	78	92	AGTTCTAGTAGCCCGGAGCC
scaffold537543_17.[illegible]	1	p3	(TCA)5	15	247	261	CATTGCAAGACAGAACCGAA
scaffold537561_17.[illegible]	1	p3	(TTG)5	15	208	222	GCAGACCTAACATAGTGGAAAA
scaffold537678_13.[illegible]	1	p3	(CCA)5	15	70	84	ATCCTCCTCCACCCGTCTAC
scaffold537876_11.[illegible]	1	p3	(ATT)5	15	435	449	CGAACCCATTAAGCCTCAAA
scaffold538028_15.[illegible]	1	p3	(CAT)7	21	1287	1307	AGGCTGGCTCTTTCAATCAG
scaffold538060_10.[illegible]	1	p3	(ATT)5	15	234	248	CTGCAGAAATGAGAGGAGGG
scaffold538209_12.[illegible]	1	p3	(CAT)6	18	63	80	TTGGCCTAGACTTCACATTTTTC
scaffold538494_15.[illegible]	1	p3	(ATT)7	21	919	939	AATGCTTGCAAGGGTGTAAGA
scaffold538554_11.[illegible]	1	p3	(ATC)5	15	54	68	GTACCGATATCATCCCCACC
scaffold538858_13.[illegible]	1	p3	(TAA)5	15	529	543	AGGTCAGATCGAAGGTCTCG
scaffold538885_15.[illegible]	1	p3	(TCA)5	15	202	216	ACTGCATCTGGATCCTCCAC
scaffold538967_14.[illegible]	1	p3	(ATA)5	15	3343	3357	GGAGCTTGGGATTATAGGGC
scaffold539049_16.[illegible]	1	p3	(TAT)6	18	3048	3065	AGGGAAGTGTGGGGAAGAAG
scaffold539270_15.[illegible]	1	p3	(ACC)5	15	760	774	ATCCCAATTTGGCAACTCAT
scaffold539640_18.[illegible]	1	p3	(GAG)5	15	409	423	GTGATCTTGAAGGAGCTGGC
scaffold539738_13.[illegible]	1	p3	(TGA)7	21	545	565	CAGAGCGAGGGGTACTCAAG
scaffold539829_15.[illegible]	1	p3	(TTA)5	15	583	597	TTCAGTCATTGCGTGCACTT
scaffold539843_13.[illegible]	1	p3	(ACA)5	15	82	96	CAGAAATGACAAAATTCATGGC
scaffold540156_14.[illegible]	1	p3	(GCC)5	15	85	99	AGTCTCCCTCGCCAGAAAAT
scaffold540632_15.[illegible]	1	p3	(AAG)5	15	1110	1124	CAAACATTATGCACCAAGCG
scaffold540721_13.[illegible]	1	p3	(AAT)6	18	287	304	GAAAGGGGTGACCAAGTTGA
scaffold540883_10.[illegible]	1	p3	(GCC)5	15	439	453	TGAGAGAACCTCCCCCTTTT
scaffold541049_16.[illegible]	1	p3	(GTC)5	15	1405	1419	CTCATGCCGGAGTTAGGAAG
scaffold541502_13.[illegible]	1	p3	(GAA)5	15	199	213	TAGGAAGCCAACAATGGAGG
scaffold541693_11.[illegible]	1	p3	(TGT)5	15	46	60	TGTAGCCGACCCCAATTTAT
scaffold541823_13.[illegible]	1	p3	(TAA)5	15	518	532	GCCTTCTCGAATTTGTCTGC
scaffold541975_17.[illegible]	1	p3	(TGT)6	18	43	60	TGTAGCCGACCCCAATTTAT
scaffold542130_16.[illegible]	1	p3	(ACA)6	18	1089	1106	CCGCAATTTGATTGGGAATA
scaffold542271_13.[illegible]	1	p3	(TAA)5	15	244	258	TCACGGGTTTGGGAATTAAA
scaffold542322_15.[illegible]	1	p3	(AAC)5	15	4564	4578	GGTAGCCCCTAACCACACAA
scaffold542396_14.[illegible]	1	p3	(ATT)5	15	992	1006	CCCAGCTCACGTTTACCCTA
scaffold542423_12.[illegible]	1	p3	(TCC)5	15	204	218	TTTTATCTTCGGTGCGGAAC
scaffold542447_13.[illegible]	1	p3	(TCA)6	18	217	234	TTCATCTGTAACGGGTGGGT
scaffold542685_12.[illegible]	1	p3	(TAT)5	15	152	166	ATCAGGTTGCCCTCCTTTTT
scaffold542744_13.[illegible]	1	p3	(CGC)5	15	50	64	GGTGGTATGGTGTGGAGGAC

scaffold542992_17.(	1	p3	(CTC)6	18	208	225	TCTTCGTGTTTGTCCATTCG
scaffold543113_15.9	1	p3	(AAC)6	18	955	972	CGCCATTTCGTGTTTAATGA
scaffold543610_11.8	1	p3	(TTA)5	15	509	523	AATCTGAGGCATGTTACCGC
scaffold544059_10.5	1	p3	(ATA)5	15	100	114	CACAATTTGAGGTGTATGGCA
scaffold544152_11.3	1	p3	(ATT)6	18	453	470	TATTAGGGCATGCAAAAGCA
scaffold544633_14.3	1	p3	(ATA)5	15	575	589	ATCATCCCCAAAGCAAAAAT
scaffold544880_17.1	1	p3	(TGA)7	21	89	109	GTTCGTAAAGAATGGGCTGG
scaffold544988_15.(	1	p3	(ATT)5	15	448	462	TTTTTGCAACGTGGTCTTCA
scaffold545383_15.8	1	p3	(TCT)5	15	1024	1038	CAACTGTCCTTGGAAGAGGG
scaffold545439_13.9	1	p3	(CGC)5	15	531	545	GTGTGGAAAAGGGAGTGCAT
scaffold545557_12.2	1	p3	(GGT)5	15	656	670	CCACCTGCGAACCATAAAAT
scaffold545657_15.7	1	p3	(ACA)7	21	338	358	TTGGATTGCAACCTCCTTTT
scaffold545774_15.9	1	p3	(AAT)5	15	923	937	CAAAATGTACCCCGAAATGG
scaffold545872_15.6	1	p3	(ATA)6	18	957	974	GGCCTACAAACTGCCCATAA
scaffold545934_13.6	1	p3	(AAT)6	18	391	408	AAATCCAGAACGCAGGTTTG
scaffold546127_18.7	1	p3	(GGC)6	18	592	609	CATATCGATTTTGCCTGGCT
scaffold546421_10.4	1	p3	(ATC)7	21	333	353	TTTGTGCTCACACTTCTCTCG
scaffold546929_13.(	1	p3	(AAC)5	15	336	350	AAGCCCACCTTATTGCTTGA
scaffold547013_16.8	1	p3	(AAC)5	15	922	936	CCCCGCACATAGTCAAGAAT
scaffold547073_15.4	1	p3	(TAC)5	15	2902	2916	TGTCCGGTGTCGTTTTAATG
scaffold547631_15.(	1	p3	(TTA)5	15	615	629	GAAGGCATGGGTTTTCTTGA
scaffold547644_17.5	1	p3	(GAG)5	15	145	159	AGAATGGAGCGGAGGTTCTT
scaffold547685_13.1	1	p3	(CGG)5	15	46	60	GGGAGGGAAGAGAGGAGAAA
scaffold547858_11.4	1	p3	(AAT)6	18	98	115	TTGACCAAGATACACCTTGCAT
scaffold548207_16.3	1	p3	(ATA)5	15	2746	2760	TGGAATGATGAGGTGGGTAAA
scaffold548235_16.5	1	p3	(CTG)5	15	783	797	ATTTGCTCTAACCAATGGCG
scaffold548319_10.4	1	p3	(GCC)5	15	55	69	ATCCCCCTTTCCTAGGGTTT
scaffold548443_10.7	1	p3	(ATC)6	18	457	474	TACTGCTCTCTGGGGCATCT
scaffold548567_19.4	1	p3	(AGA)5	15	957	971	CTGTTGGGAGGTTTCGTCAT
scaffold548636_16.(	1	p3	(CCA)5	15	283	297	TTTTTGCCCTATTTTTCCCC
scaffold548772_18.(	1	p3	(ATC)5	15	97	111	TGGCCCAACCTCAATATTCT
scaffold548861_15.8	1	p3	(ATG)6	18	1271	1288	GCGTGATACCAATGGATGTG
scaffold549199_17.1	1	p3	(TTA)5	15	258	272	TTCTCAATCAAATCGGGGTC
scaffold549288_18.9	1	p3	(TTC)5	15	67	81	CGGTTTTCCCTACTCTCCCT
scaffold549328_15.6	1	p3	(ATT)5	15	5240	5254	TTTAGCTTCTGGTGGGCTGT
scaffold549506_16.4	1	p3	(TTA)7	21	245	265	TTTGCCGATCATCCACTACA
scaffold549669_19.6	1	p3	(CGC)5	15	41	55	TTCTGTCAATTTATGTGTTAACTTCTG
scaffold549719_17.8	1	p3	(CGG)5	15	103	117	AATTTTTCCAACTCATCCGC
scaffold549806_14.6	1	p3	(ATC)5	15	310	324	TAGCCGAATTCTCCTCGAAA
scaffold549808_12.9	1	p3	(TCT)5	15	916	930	GGGGGTTTTGGATGAACTTT
scaffold549921_18.7	1	p3	(TCC)5	15	2436	2450	CATCCACCTTCACACCATCA
scaffold549955_17.3	1	p3	(CGG)6	18	57	74	AATAATTTACCGGCAATGGTG
scaffold550847_16.2	1	p3	(CGG)6	18	152	169	GAAAACTGAGCCAACCCAAA
scaffold551119_15.4	1	p3	(TTA)5	15	217	231	TTCCGGTAATTCAACAAAACG
scaffold551203_15.(	1	p3	(AAT)5	15	1673	1687	CCACGAATATGACGACAACG
scaffold551651_15.6	1	p3	(AAT)5	15	1989	2003	CGATCCAAGGAGAAAATCCA
scaffold552101_15.6	1	p3	(CAC)5	15	1399	1413	TGCCTTCTTTAACAGCCCAT
scaffold552626_16.5	1	p3	(AAT)6	18	925	942	TATTTCCTTCTCGTGGGTGC
scaffold552656_18.6	1	p3	(ATT)5	15	2265	2279	TCCCACGAGGAATTTGAGTC
scaffold552657_12.1	1	p3	(CGG)5	15	505	519	GATGAGACCACCGAGAGGAA
scaffold552834_11.1	1	p3	(TCC)5	15	339	353	ACAACTCGTCTTCCGTGGAT
scaffold553078_10.(	1	p3	(GCC)7	21	100	120	CAGAGCCAGCCTATCTCCAG
scaffold553245_13.3	1	p3	(TTC)5	15	242	256	GCATGGGGTTATTCAAGAGG
scaffold553457_13.7	1	p3	(GAA)5	15	284	298	TTAGCATCCAAAAGGGTTGG
scaffold553531_13.8	1	p3	(ATA)5	15	330	344	AGTTTGGGTATCCGCTCCTC
scaffold553555_15.5	1	p3	(TTG)5	15	1746	1760	AAAGCCGAATTCGTTACAACA
scaffold554172_18.7	1	p3	(TTA)5	15	176	190	TTCCGGTAATTCAACAAAACG
scaffold554280_16.(	1	p3	(ATC)5	15	1057	1071	GACCGTCACAGTTAGTGCCA

scaffold554654_14.2	1	p3	(AAT)6	18	756	773	AACGCATACCACATAAGCCA
scaffold554843_15.5	1	p3	(AAC)7	21	521	541	AAATCCAGCAGCAAATCGAC
scaffold554858_19.7	1	p3	(ATC)5	15	241	255	CCGAGAGATGTCTCCCAGAA
scaffold554905_12.5	1	p3	(CAT)6	18	108	125	TTTCGGGAATTCAAAGCATC
scaffold555104_14.5	1	p3	(CTT)5	15	807	821	ACCTTGCTCGTGTACCCATC
scaffold555414_14.8	1	p3	(ATA)5	15	121	135	TGGGAGCAAATTTCAAGAGTC
scaffold555451_11.2	1	p3	(TTC)6	18	76	93	AAAATTGGTGCGGATGTCTC
scaffold555629_18.4	1	p3	(TAT)5	15	620	634	CAAACCGACAGGGAAACACT
scaffold555829_14.9	1	p3	(TCA)6	18	109	126	ATTCGGTTGCTTTTGTCACC
scaffold555887_11.7	1	p3	(TTC)5	15	237	251	CCACTGTTGTTGCTGCTGTT
scaffold555897_12.8	1	p3	(CTC)6	18	685	702	AAACAGTTTCCCTGCAATCG
scaffold555931_16.2	1	p3	(ATG)5	15	3819	3833	GACGACTCAGCTTGACCACA
scaffold555958_12.4	1	p3	(CAT)5	15	425	439	ATGCTTTTGCAGGATTCAGG
scaffold556179_12.8	1	p3	(TTA)5	15	434	448	TGCAGCAATGGAGGTAAGAA
scaffold556392_15.0	1	p3	(TCA)6	18	1951	1968	TGTGAACTTCCTCCAACAACA
scaffold556563_18.3	1	p3	(TCA)5	15	1726	1740	CCACCAGTACCAAAACCACC
scaffold556620_11.7	1	p3	(CTT)5	15	135	149	GAATTTGGCCAAGTCGATTC
scaffold557096_16.9	1	p3	(GTG)6	18	5557	5574	AGCTTGCAAGTAGGAGGCAA
scaffold557514_13.4	1	p3	(ATT)5	15	450	464	TGTTGAGGCATGGCTATGAA
scaffold557542_14.5	1	p3	(ACA)6	18	3229	3246	ACAGTTCAGCTCCACAACCC
scaffold558103_14.0	1	p3	(ACA)5	15	797	811	CATGTGCATCAAACGGAAAC
scaffold558176_17.3	1	p3	(TGA)5	15	1063	1077	TGCTTACCTCCGGAATTTTG
scaffold558197_12.7	1	p3	(CTT)7	21	109	129	TCAACGCTCACCTTCACATC
scaffold558378_14.9	1	p3	(CAA)5	15	2605	2619	ACTTCCCAGACCTTGTTCCA
scaffold558565_14.1	1	p3	(TAT)5	15	3395	3409	TCTTCTTGACCAATAATAAAGAAAAC
scaffold558863_13.6	1	p3	(TAT)5	15	126	140	CCAAACCAACCTCGCTCTTA
scaffold558921_11.9	1	p3	(GGC)6	18	90	107	AAGAGATAGGGGAAGGAGCG
scaffold558930_11.3	1	p3	(GGC)5	15	273	287	CAAACATGGGAGGGTTTTGT
scaffold558979_10.9	1	p3	(GAA)5	15	86	100	CCAGACATCGAAGAAGCTGTT
scaffold559187_16.7	1	p3	(TGA)6	18	152	169	AGGAGCCACCTGATGAACAC
scaffold560033_16.7	1	p3	(CAC)5	15	1682	1696	GCTTGTCCTCCACTTGAAGC
scaffold560655_12.2	1	p3	(AGA)5	15	411	425	GGGAGGTTTCGTCACACAGT
scaffold560980_10.5	1	p3	(TGT)7	21	43	63	TGTAGCCGACCCCAATTTAT
scaffold561081_11.7	1	p3	(ATA)5	15	292	306	ACCAGTTGGGTTCGAGAAGA
scaffold561251_12.0	1	p3	(TTA)5	15	90	104	CCTAAGCAATTGATAGTGCCAA
scaffold561708_13.4	1	p3	(AGA)5	15	307	321	GTTTTCCACCAAGGTTGCAT
scaffold561791_17.0	1	p3	(GGT)5	15	1657	1671	TTTTGGGTTTGGAGACGAAC
scaffold561809_11.4	1	p3	(TTA)7	21	244	264	TTCGGTTTATACAATAACCAATTTACT
scaffold561884_14.9	1	p3	(TGT)5	15	216	230	TGACATTCCTTTCCATTGGC
scaffold561954_15.1	1	p3	(TCA)5	15	112	126	AGTCACGGGTTCGAATTTCA
scaffold562123_14.2	1	p3	(GGC)5	15	159	173	GCGAGTTTCTTGAGGTCGTC
scaffold562240_14.4	1	p3	(TCT)5	15	532	546	GTTGTCGGTGGTCATGTTGA
scaffold562470_18.2	1	p3	(TAT)5	15	294	308	TCGTGCAAGTTTTCAAGATCA
scaffold562739_17.3	1	p3	(TGA)6	18	994	1011	AGGTTGCGTACATCTGACCC
scaffold562848_15.6	1	p3	(TGT)5	15	3391	3405	AGGTGCTAGCAGCATGGAAT
scaffold563062_12.3	1	p3	(TTA)5	15	354	368	TCACGATCCTTCTGTCCCTT
scaffold563311_16.3	1	p3	(TCA)5	15	399	413	CTTGCAATTGTCGTGCAAGT
scaffold563459_13.6	1	p3	(ACA)6	18	667	684	ACCGTTGTGGTTCGGATTAC
scaffold563860_15.5	1	p3	(ATC)6	18	514	531	TCAATTCAAAATCAAACCATGC
scaffold564042_14.7	1	p3	(ATA)5	15	590	604	TGTTCACGGCAGAAGCATAG
scaffold564084_12.0	1	p3	(TTG)5	15	158	172	TTGATACTACACGGTGGCGA
scaffold564090_12.9	1	p3	(TAT)5	15	372	386	TGGTTAATGCATTTTGCCTG
scaffold564132_15.1	1	p3	(TTG)5	15	2203	2217	ACTTGGCACGATCCAATTTT
scaffold564273_10.7	1	p3	(CTT)5	15	926	940	ATCCCATGGCTGTTGTTCTT
scaffold564423_16.5	1	p3	(TTA)5	15	487	501	TCTCCTGTACTTCGTTTAGACATCA
scaffold564426_11.5	1	p3	(TTG)5	15	439	453	ATTGATCGTACACGATGGCA
scaffold564466_15.7	1	p3	(ATT)5	15	236	250	ATGTTCATGTTGGGTGCTGA
scaffold564720_14.6	1	p3	(TTA)7	21	937	957	GGGATCCAAACCTCGACATA

scaffold564738_18.3	1	p3	(CCG)5	15	841	855	CCAAGGTGCAACTTACAGCA
scaffold564825_13.8	1	p3	(AGA)5	15	54	68	AGCTTATGTTGCATGGAGGG
scaffold564826_14.6	1	p3	(ATA)6	18	155	172	TTGGAAAATGGAAAAGGCTG
scaffold565954_16.7	1	p3	(CTT)5	15	1230	1244	CCATGCATTTCAGGTTTTCC
scaffold566056_16.3	1	p3	(AGA)5	15	2474	2488	CTCCAAAAGCTTGCACACAA
scaffold566411_14.1	1	p3	(ATG)5	15	608	622	GGGCTGTTTTTGAATCGAGA
scaffold566510_10.2	1	p3	(CAT)5	15	345	359	TGACATTGTGACCTTCAGGC
scaffold566582_13.8	1	p3	(ATC)5	15	116	130	AAGGCTACAACCACATACCGA
scaffold566718_14.6	1	p3	(TAT)6	18	3291	3308	AGATTGCACATATAGCCCCG
scaffold566731_16.7	1	p3	(GAT)6	18	385	402	AAAATCTTTTGGGGCGTTCT
scaffold567634_14.9	1	p3	(TAA)5	15	750	764	GGAATTCAATCCAAGGACCA
scaffold568056_17.0	1	p3	(CAT)7	21	2364	2384	TCAAACTGGAAACCGAAACC
scaffold568120_18.1	1	p3	(TAA)5	15	1266	1280	CTTCCCTAACCGTCCCTTGT
scaffold568134_10.6	1	p3	(TGT)5	15	334	348	TTCTGATTTGCAGCAGGTTG
scaffold568234_16.8	1	p3	(TGA)7	21	479	499	ACAAAACACAACAACCCCGT
scaffold568426_14.1	1	p3	(ATA)6	18	645	662	ATGCGGATGTTGTGGGTAAT
scaffold568459_15.0	1	p3	(TTG)7	21	716	736	TGTCAAGCTCTTGCATAGCAGT
scaffold568554_14.3	1	p3	(ATT)6	18	1673	1690	AATAAAAGGCGGATTGACCC
scaffold568577_14.4	1	p3	(TTG)5	15	1294	1308	TGGAGCTCTTGGATACCTGG
scaffold568934_18.4	1	p3	(GCA)5	15	1034	1048	AAGCGTTGAAGCAGCAAAGT
scaffold568981_15.1	1	p3	(AAT)5	15	398	412	CCAACCTCAGAATCATCTTTCA
scaffold569142_18.1	1	p3	(CAT)6	18	369	386	TTGATCTCAGTCAGCCATGC
scaffold569219_14.5	1	p3	(ACA)5	15	53	67	TCAAAACATTTAGTTGGCACAAT
scaffold569381_11.0	1	p3	(TGC)6	18	403	420	TCTCCATCCTCAACCGATTC
scaffold569388_10.6	1	p3	(ATT)5	15	108	122	ATATAGGCCCAAATCTGGCA
scaffold569451_12.3	1	p3	(TTG)5	15	73	87	CTATCGGTACTCGGATGGGA
scaffold569925_18.0	1	p3	(AAT)5	15	3147	3161	AGCATATGCCTTGCTTGCTT
scaffold570026_13.0	1	p3	(AAT)5	15	492	506	TGAAAAATCCGAATCCGAAC
scaffold570065_16.7	1	p3	(CAC)5	15	123	137	CAATGGACAACCAAGCCTTT
scaffold570249_16.0	1	p3	(TCA)8	24	2131	2154	TGCCCAGTATTGGTCATTCA
scaffold570796_16.7	1	p3	(GGC)5	15	436	450	GCGTCTTTATAGTCGCCGAG
scaffold570844_19.1	1	p3	(TTC)5	15	686	700	TCTCGCAGAGGAGGTCAAAT
scaffold571167_15.0	1	p3	(GAT)5	15	560	574	AAGAAAGACGAAGCATGGGA
scaffold571251_15.3	1	p3	(ATT)5	15	1235	1249	ATCAAAACTGGTAATGCGGG
scaffold571590_16.7	1	p3	(CGG)5	15	2042	2056	TAGACACATGCCAACTCCCA
scaffold571630_17.4	1	p3	(ACG)5	15	764	778	ACGACGACGGAAAGATTGAG
scaffold571631_13.6	1	p3	(TTA)5	15	1227	1241	GGGACAACGGGTTCTGATAG
scaffold572154_13.9	1	p3	(CCA)5	15	216	230	CCAGCAGACACGAAACAGAA
scaffold572157_14.4	1	p3	(TCA)5	15	198	212	GGTCTCAACTTCGCGATCAT
scaffold572424_10.8	1	p3	(TGA)6	18	96	113	TGGTTTGGGAATAATTGGGT
scaffold572654_18.0	1	p3	(CAC)5	15	953	967	CGTTAAGGACACAAACCCGT
scaffold572788_11.9	1	p3	(CAT)6	18	114	131	GGGTGGTTTATGGCCTTTTT
scaffold573085_10.2	1	p3	(TAT)5	15	68	82	CATTTGGGGGTATTACAATTTTT
scaffold573220_15.4	1	p3	(TCT)5	15	359	373	GCGTTTCTTAAACTGCCTGC
scaffold573256_12.0	1	p3	(AAT)7	21	82	102	GACAGCTGCACGACATGATT
scaffold573328_17.0	1	p3	(TTG)5	15	331	345	GAAGGGGGAGACCGAAATTA
scaffold573394_17.0	1	p3	(AAG)6	18	122	139	ATGTTGGAGTCGGGAAGATG
scaffold573538_12.6	1	p3	(CTT)6	18	736	753	CTCCCACGACCTTTTGTTGT
scaffold573938_10.6	1	p3	(GGT)5	15	322	336	ATTTGTACACCGGTGGTGGT
scaffold574103_11.1	1	p3	(ATA)6	18	186	203	CGTTTGAAGCCGAGTTGAAT
scaffold574201_10.7	1	p3	(ATA)5	15	572	586	AAAATATGGTGTGCATAACGAGA
scaffold574226_11.0	1	p3	(CGG)5	15	248	262	TGCGCCACAAAATTTCAGTA
scaffold574250_10.0	1	p3	(ATT)5	15	891	905	GTTTGGATTGACAAGTCGGG
scaffold574770_15.3	1	p3	(ATC)5	15	1993	2007	TTGTTCTTGGGATTGGTGGT
scaffold574853_10.3	1	p3	(GAG)6	18	989	1006	GTGCAGATGTGTCTGTCGCT
scaffold574922_14.4	1	p3	(AAT)5	15	1892	1906	AAGAGGCCACCGAAACTCTT
scaffold574931_14.4	1	p3	(ATA)6	18	221	238	AAAGAGGTTGTTTCCGTTGC
scaffold575088_15.8	1	p3	(AGG)6	18	3022	3039	TCGTAATGATGCCACAGGAA

scaffold575473_14.3	1	p3	(TTC)5	15	548	562	CGTTGACTGCTGGAGAATGA
scaffold575600_15.8	1	p3	(ATC)5	15	1555	1569	CTGACCCAAGGTCCCAAGTA
scaffold575839_17.7	1	p3	(ACC)5	15	749	763	ACCTGCACCACCACCAAGT
scaffold575991_11.8	1	p3	(ACA)5	15	426	440	TCTTGCAAAATGATTGGGTG
scaffold576448_14.8	1	p3	(TAC)5	15	3270	3284	TTGACGGCTCATGCTTACTG
scaffold576766_15.9	1	p3	(TTA)5	15	1175	1189	AAAAAGGTCCCCTTTCCAAG
scaffold576805_10.6	1	p3	(TTA)6	18	219	236	GAAACCTGACACCGTAGGGA
scaffold577334_18.0	1	p3	(CCA)7	21	158	178	CCCATCTCTTGTTTTCTTCATCA
scaffold577475_11.3	1	p3	(CTC)5	15	413	427	CCGGATCACATTTCGAGTCT
scaffold577618_16.2	1	p3	(CAT)5	15	902	916	AGGCTTGCGGGTCTTACTTA
scaffold577659_13.2	1	p3	(CAT)6	18	1029	1046	ATACTTCCGTATTGCCGCAC
scaffold579121_17.0	1	p3	(TGA)5	15	2049	2063	CTTGCAATGTGGCATTCATT
scaffold579269_12.5	1	p3	(TCT)5	15	136	150	TTTGCTGAATTTTCTTCGCA
scaffold580205_15.1	1	p3	(TTC)5	15	2456	2470	CAAGCACAAAATAGCGAATCA
scaffold580233_17.0	1	p3	(CTT)5	15	85	99	TAGTGGGGGTCAGAACATGC
scaffold580663_15.0	1	p3	(CAT)6	18	132	149	CCACAACTGGTCGGTATGGT
scaffold580920_10.3	1	p3	(ATG)6	18	51	68	GCAGCAGCAACATACAAACC
scaffold581086_18.3	1	p3	(CCG)5	15	2245	2259	TCTCCGCCATCAAATTCTTC
scaffold581284_17.3	1	p3	(CAA)5	15	1664	1678	TTTGTTGTGGAGTTTCAGGTG
scaffold581372_20.4	1	p3	(TTA)5	15	290	304	ATTGCCTCCACAAGAATTGG
scaffold582178_16.4	1	p3	(CCA)5	15	484	498	GCAAGACTGTCGTCCTCCTC
scaffold582190_12.7	1	p3	(TGA)5	15	45	59	CCCAAGACCCCAATTTGATT
scaffold582229_18.5	1	p3	(AGA)5	15	453	467	TGTTGTGCGTGCTTTTCTTC
scaffold582394_10.0	1	p3	(TGT)5	15	45	59	TGTAGCCGACCCCAATTTAT
scaffold582449_17.4	1	p3	(ATT)7	21	218	238	TCGAACTCCACTGTTCCATTT
scaffold582782_16.1	1	p3	(ACA)5	15	2113	2127	CATGTCACACTTCGCTCACC
scaffold582785_12.1	1	p3	(AAT)6	18	1185	1202	AATTATGGCGTTGGGTGAAC
scaffold582866_15.1	1	p3	(TGT)5	15	51	65	TTCATGTAGCCAACCCCAAT
scaffold582893_13.8	1	p3	(ATC)6	18	183	200	GCTACCTCTCCCTCTCTCCC
scaffold582985_13.9	1	p3	(ACA)5	15	1401	1415	CCATAGTTTAAGCAAGGGCA
scaffold583212_14.4	1	p3	(TGA)5	15	102	116	AGGTTGCGTACATCTGACCC
scaffold583580_15.2	1	p3	(GAA)5	15	1288	1302	GATGGGGTTGCAGAAGGTAA
scaffold583927_13.2	1	p3	(TAT)6	18	1197	1214	CCCCACAATGTCTCGTCTTT
scaffold584134_15.9	1	p3	(GTG)5	15	255	269	AAGTGGACCATTATCGGACG
scaffold584289_13.0	1	p3	(CGC)6	18	356	373	CGCCTTCTTTGCACCTACTC
scaffold584291_12.8	1	p3	(TGC)6	18	913	930	GGATTTGGCCCTGTGTGTAT
scaffold584633_12.1	1	p3	(GGC)6	18	112	129	GTTGGCAACTGGATGTAGGG
scaffold584733_14.7	1	p3	(ACC)6	18	1126	1143	GTTTCCAACGAAGTTCCCAA
scaffold584739_12.2	1	p3	(TGA)5	15	103	117	CCAAGACCCCAATTTGACTG
scaffold584751_11.0	1	p3	(ATA)7	21	608	628	TAGATGGCTGGGAAAATTCG
scaffold584786_10.7	1	p3	(CTT)6	18	284	301	ATTGTCAAACCAGCGAGAGC
scaffold584787_13.2	1	p3	(CTT)6	18	274	291	ATTGTCAAACCAGCGAGAGC
scaffold584892_13.5	1	p3	(GAA)5	15	165	179	CGACCGAATAATCCTGTGGA
scaffold585028_15.8	1	p3	(ATT)5	15	317	331	CAGTGCTGTCCAAACCTCAA
scaffold585298_15.8	1	p3	(TCT)5	15	1144	1158	AGAAGGAAATGGGAATGGCT
scaffold585565_15.3	1	p3	(TAT)6	18	164	181	AAAATGGTGGCTTTCTTCCC
scaffold585983_17.6	1	p3	(TGA)7	21	2812	2832	CTATGCCGTCGATTCTGACA
scaffold586096_14.2	1	p3	(GGA)5	15	101	115	CATGTACCCCACAGAGGGTC
scaffold586300_14.5	1	p3	(CTT)5	15	1307	1321	TTTCCTCCAACATTGCTTCC
scaffold586545_16.5	1	p3	(TAA)5	15	955	969	CCAAGTCACCCAAGAATTGC
scaffold586802_16.0	1	p3	(ATT)5	15	233	247	TTTGTTGTGGGTTACCCGAT
scaffold586870_15.7	1	p3	(TGT)6	18	44	61	ATGTAGCCGACCCCAATTTA
scaffold586888_13.9	1	p3	(GAA)5	15	321	335	CTTTTCACGTCGGGATTGTT
scaffold587106_12.3	1	p3	(CGG)5	15	2609	2623	CGGAGAAGGAAATAGGACCC
scaffold587823_16.2	1	p3	(ATC)7	21	2237	2257	ATCATGTCAAGGGCTCAAGG
scaffold587828_10.5	1	p3	(AAG)5	15	318	332	GGATTTGTGTGACTGCATGG
scaffold587892_10.0	1	p3	(AAG)5	15	609	623	ATGAGCGAAACAAAATTGGG
scaffold587969_10.5	1	p3	(TAA)7	21	129	149	GTTTACCTGGCTGACGTGGT

scaffold588057_16.9"	1	p3	(AAT)5	15	711	725	TATCCGTTCGGCTGTACCTT
scaffold588111_12.2"	1	p3	(AAG)5	15	211	225	GTCAATGGGAACAACCGTCT
scaffold588157_16.2"	1	p3	(TAT)6	18	2732	2749	ATTGCAAGCAAAATCATCCC
scaffold588355_12.0"	1	p3	(ATT)6	18	320	337	ACCCCATCTCAACCAAATGA
scaffold588723_16.1"	1	p3	(TTA)5	15	815	829	CCAATCATACATGCAAAGTGC
scaffold588991_15.5"	1	p3	(AAT)6	18	1756	1773	ACCATGACCGACATGCAGTA
scaffold589399_16.1"	1	p3	(GGA)6	18	1119	1136	GCTGCTCCTCATTGTCATCA
scaffold589484_13.9"	1	p3	(AAC)6	18	1815	1832	CCGTAAATGCCTGTCAACCT
scaffold589549_13.8"	1	p3	(CGG)6	18	80	97	AATCATGTACACCCGCTTCG
scaffold589822_12.6"	1	p3	(CGG)5	15	49	63	CAGCACCACCAGCGAGAA
scaffold589885_10.6"	1	p3	(GTT)5	15	226	240	TTGCGGATAATACAATGGAGC
scaffold590321_16.4"	1	p3	(GCT)5	15	338	352	TGGCAAATATGGATGTGGAA
scaffold590370_13.7"	1	p3	(TCT)5	15	103	117	GGAAAACTTTATCATTTCCCTTGA
scaffold590530_16.3"	1	p3	(ATT)5	15	170	184	TGGCCTTTTAATAGGATGCG
scaffold590710_16.0"	1	p3	(ATG)5	15	4215	4229	TTGGGAATTTCTTTCCATGC
scaffold590887_17.4"	1	p3	(CAA)6	18	273	290	TTTGACCAAACGGAACAACA
scaffold590909_13.8"	1	p3	(AAT)7	21	84	104	GTCCAAAAGCTTATCGGCTG
scaffold590966_10.6"	1	p3	(ATT)8	24	501	524	TGAAATCCCTCAAATCTTACCAA
scaffold591125_12.7"	1	p3	(CGC)5	15	244	258	GAATCTTAGGACGCGACGAG
scaffold591654_10.1"	1	p3	(ATC)5	15	247	261	ACGAGTGAGGCTGGAAAGAA
scaffold591698_14.2"	1	p3	(TGG)6	18	1363	1380	CCCATCATAGGGAAAGGGAT
scaffold592654_16.7"	1	p3	(GTT)7	21	4824	4844	CCGACCCCACTTAGTGAAAA
scaffold592719_17.1"	1	p3	(AAT)5	15	1622	1636	ATGATGAGCCGTCAATCTCC
scaffold593051_12.8"	1	p3	(TTG)5	15	2869	2883	TGGTTTATGTAGCCAACACAATTT
scaffold593064_18.3"	1	p3	(TTC)5	15	977	991	GGTTCCCCATTCTCCTCTTC
scaffold593654_20.0"	1	p3	(TTG)5	15	232	246	AATTAGCGCACCCACAAATC
scaffold593761_13.3"	1	p3	(GGT)5	15	1250	1264	GATCCTTTGCTGGAGGTTCA
scaffold594136_13.6"	1	p3	(ATT)5	15	974	988	TCGTCTTGATTTTGTTCTGCAT
scaffold594378_18.1"	1	p3	(CCG)6	18	168	185	CCCGACTTCGTCTCTACGAC
scaffold594466_13.5"	1	p3	(GGA)5	15	620	634	TCACAGACTCACAGTGTTGGC
scaffold594517_17.2"	1	p3	(TAT)5	15	2359	2373	TTGGATGATGGAAGAAAATCG
scaffold594557_15.3"	1	p3	(ATT)5	15	1014	1028	AAAGGGAAAAGCCCCTATTTT
scaffold594591_12.6"	1	p3	(ATG)5	15	257	271	TCATTTCTAAACCGGAACGG
scaffold594592_10.0"	1	p3	(ATG)5	15	256	270	TCATTTCTAAACCGGAACGG
scaffold595066_12.5"	1	p3	(AAT)5	15	76	90	TCCCTTTAAAAACACAAACCTTTT
scaffold595279_17.3"	1	p3	(CGG)5	15	1470	1484	CGTCAGATGAGGGTCAGGAT
scaffold595313_13.0"	1	p3	(ATA)6	18	1079	1096	ACAGTTTGAGATCCATCGCC
scaffold595453_17.1"	1	p3	(TCA)5	15	2053	2067	GCTTCAACCATAGCACCCAT
scaffold595480_16.9"	1	p3	(TTG)5	15	48	62	TGTAGCCGACCCCAATTTAT
scaffold595499_16.6"	1	p3	(TGT)5	15	172	186	TGTAGCCGACCCCAATTTAT
scaffold595523_18.7"	1	p3	(TAG)5	15	2434	2448	AATGGTACCCCAACTTCACG
scaffold595590_10.5"	1	p3	(CAT)5	15	247	261	CGAGATTTATGAGAGCCCCA
scaffold595658_17.4"	1	p3	(TCT)6	18	684	701	AGGTTGGTGGATTTTGGGA
scaffold595808_14.0"	1	p3	(ATA)5	15	2715	2729	TCTCGTGTAACGAACAAGCAA
scaffold596212_22.3"	1	p3	(AGG)6	18	163	180	CGTTGCAGAAGTTTGCTTGA
scaffold596462_11.7"	1	p3	(TTC)5	15	163	177	TCTTTGCCTTTGTCTGGAGC
scaffold596958_17.9"	1	p3	(AAC)5	15	474	488	AACACCACAAGCGACAAGACT
scaffold597084_16.0"	1	p3	(CCT)7	21	578	598	TAGCAGTTGGTGTTGAAGCG
scaffold597091_17.5"	1	p3	(AGA)5	15	912	926	TGGAGCTCTGAAGCTCAACA
scaffold597322_15.3"	1	p3	(AGA)5	15	1240	1254	GGATACCCATGAACTGGTCG
scaffold597404_10.5"	1	p3	(CTG)5	15	86	100	GTCCCTTTTTCCTGAGGACC
scaffold597727_14.5"	1	p3	(CAA)5	15	231	245	GGATGATGATGTTCAGATGGG
scaffold597864_15.6"	1	p3	(CCA)5	15	538	552	CATCCTTGCCAATTTCCATT
scaffold597990_16.2"	1	p3	(ATA)5	15	24	38	ACCCCTTGCGGGGTAATA
scaffold598690_14.1"	1	p3	(TTG)5	15	1749	1763	CACGAACACCCCTGCATATC
scaffold598725_15.6"	1	p3	(GCC)6	18	206	223	GCTTATCTGGACAACCCCAG
scaffold598813_17.4"	1	p3	(CGA)5	15	906	920	GGCAACCTCAATGTCCAAAA
scaffold598951_16.2	1	p3	(AAC)5	15	386	400	TATGGAGAAAATCCCTCCCC

scaffold598988_17.	1	p3	(GCT)5	15	2196	2210	AATCCGAGGTGTGTTGCATT
scaffold599052_15.	1	p3	(AAT)5	15	592	606	TGCAATTATTGGGTTCCAAAG
scaffold599197_14.	1	p3	(GCA)5	15	1006	1020	GGGAGAATAAACAGACGGCA
scaffold599292_13.	1	p3	(ATT)6	18	1581	1598	TGCATTTTCACGTGCATTTT
scaffold599293_14.	1	p3	(AAT)5	15	134	148	TTCGAAGCTGGAGATGTGACT
scaffold599323_15.	1	p3	(TTA)6	18	383	400	GGCAACGTGATTCAGTTCAA
scaffold599519_13.	1	p3	(CGG)7	21	199	219	GCCGAGGGTGAAATAGATGA
scaffold599762_18.	1	p3	(AAT)5	15	65	79	TTCCACGTTTCCTTAAAATATGG
scaffold599913_17.	1	p3	(TTA)5	15	862	876	GGCCGTTTGGAATAGGATTT
scaffold599957_17.	1	p3	(TCA)6	18	188	205	TAATCCATGGAAGGCTCCTG
scaffold600030_11.	1	p3	(TGA)6	18	107	124	TTGGTACTCACAAAACCCCC
scaffold600150_16.	1	p3	(ATC)5	15	490	504	ATATGCGACGTGAATCCCAT
scaffold600283_16.	1	p3	(TCA)5	15	741	755	TCGCAATTTTCACTGATGGA
scaffold600495_15.	1	p3	(GAA)5	15	615	629	GGTTTCTTGCAGGAGAATGC
scaffold600778_13.	1	p3	(AAT)5	15	371	385	TGGAATGTCTTCCATTAAGTGC
scaffold601102_15.	1	p3	(ATA)6	18	3597	3614	GGACGGTTCACCAAAAATGA
scaffold601406_15.	1	p3	(TTC)5	15	515	529	TAATGGGGAAGTCCTAGGGG
scaffold601870_13.	1	p3	(AAT)7	21	502	522	ATACTGGCGGACAATATGGG
scaffold602035_10.	1	p3	(GGT)5	15	317	331	GCTACGAGGGCGAGATGTAG
scaffold602147_17.	1	p3	(GAA)5	15	1429	1443	GCCCTGCCCTAAGACTATCC
scaffold602169_10.	1	p3	(TAT)6	18	60	77	TTGCAAGAAAATTGTCCCGT
scaffold602364_16.	1	p3	(CGC)5	15	593	607	ACCACCACCACCGTACAAAT
scaffold602868_13.	1	p3	(TTG)6	18	382	399	ATCCATACAGCCGACCTCAC
scaffold602978_16.	1	p3	(CCA)5	15	545	559	TAATTTCCCCCAATTTTCCC
scaffold603033_16.	1	p3	(ATC)5	15	1788	1802	CTCACTCGAACTCGTCTCCC
scaffold603152_15.	1	p3	(CGC)5	15	756	770	GCCTAAGAAGCCCAAATTCC
scaffold603326_15.	1	p3	(ATA)5	15	72	86	TGAAGTCCGGATCAACTTACG
scaffold603510_15.	1	p3	(TCT)5	15	458	472	CAGCCATGCTTCTTATGCAA
scaffold603581_11.	1	p3	(ATC)5	15	106	120	CAGCGAAGTGACAACCTTGA
scaffold603611_11.	1	p3	(ATT)5	15	371	385	TGCAAAATCAAGTATACGTTTGG
scaffold603777_11.	1	p3	(CCG)5	15	318	332	AAACACTGTGGCTCCAGCTT
scaffold603892_13.	1	p3	(AAT)5	15	972	986	ACGTGGAAGAGGGACATTTG
scaffold603894_17.	1	p3	(TTG)5	15	1913	1927	ACTGGCGACCTAGCAAAATG
scaffold604078_15.	1	p3	(TTA)5	15	301	315	ACATTAACCACAATGGAGGC
scaffold604197_13.	1	p3	(TTC)5	15	1486	1500	GCCCTGCATTTAGTTGAGGA
scaffold604210_13.	1	p3	(AAT)5	15	888	902	GGATGCCATTTCTTCTCCAA
scaffold604338_11.	1	p3	(TAT)5	15	576	590	CCGATTTTCTCGGATTCCAT
scaffold604354_11.	1	p3	(ATC)5	15	204	218	TGCTTTCAAAACCAGCACAC
scaffold604513_16.	1	p3	(CAT)5	15	761	775	GTTTCTTGGACAAAGCAGCC
scaffold604644_10.	1	p3	(CCG)5	15	57	71	ATCGGATGAAAGTTGCGATT
scaffold604717_16.	1	p3	(TGA)5	15	123	137	GCTTTTGAGCCCTCTGTTTG
scaffold604976_14.	1	p3	(CAA)6	18	536	553	GGCAGCGAAAAATCAGCTAC
scaffold605010_15.	1	p3	(AAT)7	21	1877	1897	AAGGCAATGAGGCAAGAAAA
scaffold605122_16.	1	p3	(TTC)6	18	960	977	TCAATACATGGCATTGGTGG
scaffold605778_16.	1	p3	(AAG)5	15	1443	1457	TCTGCAATCAATCAGCAAGC
scaffold605900_19.	1	p3	(CAT)5	15	134	148	CTTGTGGTTGTTGGTGTTGC
scaffold605995_18.	1	p3	(TAA)6	18	481	498	TAGCACCCGTAGTTGCTGTG
scaffold606069_15.	1	p3	(GAA)6	18	2233	2250	GCCACACCTGGAGTTTCATT
scaffold606126_14.	1	p3	(TGT)6	18	32	49	CCAATTTATTGGGATTAAGGC
scaffold607064_12.	1	p3	(AAT)5	15	651	665	TCGCCCATTTTGAATTAACAC
scaffold607093_12.	1	p3	(GAA)5	15	74	88	CGGAAGGCATGAAGAAGAAG
scaffold607264_13.	1	p3	(TAA)9	27	822	848	CACGCCACGTCAACTTCTAA
scaffold607314_11.	1	p3	(CGG)6	18	174	191	CCAATCATCGGCCTTTTCTA
scaffold607448_17.	1	p3	(AGC)6	18	290	307	TTTTGGGGACACATCACAGA
scaffold607488_15.	1	p3	(AAT)5	15	926	940	GAGGTGAGGTTCCATCTCCA
scaffold607727_15.	1	p3	(GAT)7	21	657	677	GTCAATTGCGGGGATACAAT
scaffold607758_18.	1	p3	(CCA)5	15	176	190	AGTTTATGACCATCAGCCGC
scaffold608052_16.	1	p3	(CAA)6	18	3576	3593	CTTGGCCATGAACCTCATTT

scaffold608081_20.[illegible]	1	p3	(ATT)6	18	1880	1897	AAAAATGTTTCTGTAGTGGTTGTTG
scaffold608155_16.[illegible]	1	p3	(CGC)6	18	600	617	GTAACTCCATGATGCCGACC
scaffold608360_15.[illegible]	1	p3	(AAG)5	15	49	63	AACATGGACTGGAAGTTGCC
scaffold608879_13.[illegible]	1	p3	(GTT)5	15	541	555	CGCATTAAACGGATACGACA
scaffold608974_13.[illegible]	1	p3	(AAT)6	18	328	345	TTTATTCCGTGACCCTCGAC
scaffold609203_15.[illegible]	1	p3	(CCG)5	15	176	190	GATTGGATTTAATTCCCCCG
scaffold609402_10.[illegible]	1	p3	(TGG)5	15	940	954	TGATGATGATAACGAAGGCG
scaffold609553_15.[illegible]	1	p3	(TTA)6	18	1641	1658	TGTTGCACCAATGTCTGGAT
scaffold609634_20.[illegible]	1	p3	(GCA)6	18	381	398	TTGGTCCCAAATCCCTACAA
scaffold609768_15.[illegible]	1	p3	(AAT)6	18	709	726	CCAAGTTACGTGGCGTAGAA
scaffold609931_12.[illegible]	1	p3	(TAA)5	15	1263	1277	TTTGAACTCGATTTGTCCCC
scaffold610032_14.[illegible]	1	p3	(CCT)5	15	2418	2432	TCCCACTCCCATTCAACTTC
scaffold610073_13.[illegible]	1	p3	(AAT)5	15	612	626	TATCGCTTTAGGCCCAGCTA
scaffold610287_18.[illegible]	1	p3	(AAG)5	15	480	494	ATTCCACAGACCAAAACCCA
scaffold610373_17.[illegible]	1	p3	(CAA)5	15	10010	10024	GGTCATTGCCTCGGATTAAA
scaffold610467_11.[illegible]	1	p3	(ACA)7	21	465	485	GCTTTTCCAGTGAAAATCCG
scaffold610492_16.[illegible]	1	p3	(CGC)5	15	895	909	TGGGACTTGGAACTAGGGTG
scaffold610547_11.[illegible]	1	p3	(CAT)5	15	63	77	TGATTCATCCTCCGTTGTGT
scaffold610913_15.[illegible]	1	p3	(GAT)5	15	64	78	TGATGATAATGAGGTTGCTGG
scaffold610961_16.[illegible]	1	p3	(TAT)5	15	3973	3987	TTTTGAAAAAGTGTTGCCCC
scaffold611117_15.[illegible]	1	p3	(GAG)5	15	305	319	TGAAGCAGTGGAGACAGGTG
scaffold611323_14.[illegible]	1	p3	(TAT)6	18	235	252	TTAGAGGTGTCAAAACGGGC
scaffold611553_10.[illegible]	1	p3	(TTA)6	18	432	449	GCAAGGGGACAACCTTTGTA
scaffold611568_17.[illegible]	1	p3	(CGG)5	15	39	53	GGACTGGAGCAGGTGTGAAT
scaffold611615_13.[illegible]	1	p3	(CCA)5	15	252	266	CAATCAACAAAACAAGCCCC
scaffold611796_15.[illegible]	1	p3	(ATT)6	18	1937	1954	AATCACCCTCGAATCAGTTTG
scaffold611986_16.[illegible]	1	p3	(TTG)5	15	585	599	AGCCGACTTCAATTTATTGGG
scaffold612222_12.[illegible]	1	p3	(ATC)8	24	101	124	GTTTGGGTGAGAACGAGGAG
scaffold612273_14.[illegible]	1	p3	(GGT)5	15	320	334	GCAAACATCTCAATGGGGAC
scaffold612278_17.[illegible]	1	p3	(ATT)6	18	850	867	AAAACGGAATCTACTTAGGGGA
scaffold612505_17.[illegible]	1	p3	(ATG)5	15	209	223	GATGTGGCTTCTGATGACGA
scaffold612637_13.[illegible]	1	p3	(AAG)5	15	1198	1212	ACTAGAAGCATTTGGCGCTC
scaffold612799_13.[illegible]	1	p3	(CGG)5	15	165	179	ATGTACACCCACTTCGGCTC
scaffold612954_10.[illegible]	1	p3	(TCA)6	18	77	94	CGTTGGATTGGATTCCTCAT
scaffold613146_15.[illegible]	1	p3	(ACA)5	15	179	193	TTGAAGATGATACGTTGAACCC
scaffold613201_13.[illegible]	1	p3	(ATT)5	15	2640	2654	TGTAGTTAAGGCGGTGGTGA
scaffold613263_15.[illegible]	1	p3	(ATA)5	15	803	817	CCAAAAGGAACAAGAGTTGAGG
scaffold613467_14.[illegible]	1	p3	(GAA)5	15	167	181	TGAAGAAGAAGGGGAGGACA
scaffold614029_13.[illegible]	1	p3	(AAT)6	18	168	185	GGGGTCGACTACATGAACCA
scaffold614204_15.[illegible]	1	p3	(CGG)6	18	274	291	ATCCTCAACTCCATCGCTTG
scaffold614207_12.[illegible]	1	p3	(CGG)6	18	291	308	CAAATTCCGTGACCAAATCC
scaffold614208_11.[illegible]	1	p3	(CGG)5	15	297	311	CAAATTCCGTGACCAAATCC
scaffold614232_10.[illegible]	1	p3	(AGG)5	15	210	224	ATTAACTCGGGGTGCAGTTG
scaffold614368_20.[illegible]	1	p3	(CCG)5	15	52	66	AGCAAACCCTAATCGGCAGT
scaffold614683_14.[illegible]	1	p3	(ATT)7	21	654	674	TAGAAGCAGCAGCAGTTTGC
scaffold614691_16.[illegible]	1	p3	(TGT)5	15	48	62	ATGTAGCCGACCCCAATTTA
scaffold614748_13.[illegible]	1	p3	(ATC)5	15	213	227	TGCTCAAGAAAATGAGAGCAAA
scaffold614838_14.[illegible]	1	p3	(TAA)5	15	465	479	TTCCATGCACTCTTTTGTGC
scaffold614906_11.[illegible]	1	p3	(ATA)5	15	456	470	GCGTAATCCCCTATTTGCCT
scaffold615182_18.[illegible]	1	p3	(TCA)5	15	1379	1393	TCACCCTCAAACATTCCACA
scaffold615246_10.[illegible]	1	p3	(GGC)6	18	250	267	TCTGTTTTGTGCGAGTCTGG
scaffold615292_11.[illegible]	1	p3	(ATC)5	15	135	149	TCATGTTCAAATCTGTGAAGGC
scaffold615412_13.[illegible]	1	p3	(AAT)5	15	68	82	TCCATGAAACATAAATCTTGCC
scaffold615492_16.[illegible]	1	p3	(GGT)5	15	1105	1119	TAGGTCACACTTGCAGTGCC
scaffold615500_17.[illegible]	1	p3	(TAT)5	15	1141	1155	TGTGAAAATCTCGGTTAATCCAT
scaffold615740_11.[illegible]	1	p3	(TTG)5	15	691	705	GGGTCAGATGTACGCAACCT
scaffold615785_20.[illegible]	1	p3	(GTT)5	15	27	41	TGGGTTCAATGCATTTACTTCTT
scaffold615818_19.[illegible]	1	p3	(TTG)5	15	962	976	ATATAGCCGACCGCACAAAG

scaffold615877_15.(	1	p3	(ATT)6	18	65	82	TGAAATTGAAAACCTTAAAAATGAA
scaffold615988_15.:	1	p3	(ATC)6	18	2954	2971	TTCCTTTTCAAGGTGCTTCA
scaffold616009_11.8	1	p3	(CCT)5	15	440	454	GAGGACCTTCAAGGGTGTCA
scaffold616057_17.:	1	p3	(GAA)5	15	965	979	AGATTGCTGTGATGTGGGTG
scaffold616160_13.7	1	p3	(CGG)5	15	957	971	TATTCGACGAGGATTCCGAC
scaffold616351_13.:	1	p3	(TTG)5	15	77	91	ATCCAGAGGAGCTTGCTGGT
scaffold616426_12.3	1	p3	(CTC)7	21	489	509	AATGATCATCCAACGGTTTCA
scaffold616617_13.2	1	p3	(ATC)5	15	837	851	GACCGGGACTGTAATCCTCA
scaffold616704_15.7	1	p3	(TCA)8	24	1255	1278	TGTGGATGGTCTGAATGGAA
scaffold616715_11.3	1	p3	(TTA)5	15	359	373	GCATCAAAACATTTCAGCCA
scaffold617145_10.5	1	p3	(GAA)5	15	216	230	AGGGTAGGAAGCCCAAAGTG
scaffold617365_13.6	1	p3	(AGA)5	15	172	186	CCATCAGTTCCTTCTAGGGC
scaffold617701_14.8	1	p3	(TTG)6	18	240	257	TGTAGCCGACCCCAATTTAT
scaffold617751_10.:	1	p3	(ATA)5	15	160	174	CTCGCACCAAATGGGTTAGT
scaffold617757_19.:	1	p3	(AGG)5	15	201	215	CGTTGCTGGTGATGAAGAGA
scaffold617941_15.:	1	p3	(TCA)5	15	295	309	TCTTCAACATTCCTGGGCTC
scaffold618157_15.3	1	p3	(ATT)5	15	611	625	CATCGGGTGCAATGATTAAG
scaffold618488_16.6	1	p3	(TGA)5	15	47	61	CCCAAGACCCCAATTTGATT
scaffold618503_16.(	1	p3	(AAT)5	15	862	876	TAACGGGAGAAATGGTCCAA
scaffold618510_18.:	1	p3	(CGC)5	15	290	304	TGATGGTATGGTGTGGAGGA
scaffold618779_11.9	1	p3	(GGC)5	15	239	253	GGTGTGGAGGAGCAATTCAT
scaffold618970_15.6	1	p3	(TTC)7	21	355	375	ATCCTCCACAGAAATGCCAC
scaffold619060_12.7	1	p3	(TCA)6	18	139	156	ACATGGGTTTAGGGTTTCCC
scaffold619065_12.:	1	p3	(AAT)7	21	64	84	GTTTCATACCCCCACCACAC
scaffold619194_14.2	1	p3	(TTC)5	15	489	503	GAACACATTGCTGAGAGCACA
scaffold619509_17.3	1	p3	(TTG)5	15	1842	1856	GTTTCTGATGTTGTTGCCGA
scaffold619566_12.8	1	p3	(AAT)6	18	272	289	CATCTCATGTCCTCGCCTCT
scaffold619655_15.7	1	p3	(TGT)5	15	46	60	TGTAGCCGACCCCAATTTAT
scaffold619664_15.7	1	p3	(TGA)5	15	93	107	TGTTTTTGAGAAGAAACAAGAAGC
scaffold619698_14.:	1	p3	(ATT)5	15	405	419	TCCCCAAGAACAACCCATTA
scaffold619810_13.8	1	p3	(ATT)5	15	626	640	GGCTATGGAAACTTGATGATGG
scaffold619919_10.(	1	p3	(TTG)5	15	259	273	AATGTTGATGCTGTTTGGAGC
scaffold620058_11.9	1	p3	(GAG)5	15	553	567	GATGTTGGAGCTGAAGAGCC
scaffold620484_11.(	1	p3	(ATT)9	27	222	248	CCTCCATCAGTTCTGTCTAGGG
scaffold620678_14.3	1	p3	(TGA)5	15	233	247	GTCCGGGTATTGTCTGCTGT
scaffold621022_11.:	1	p3	(TAA)5	15	250	264	AAATGAAATCGTGCGAAAATG
scaffold621396_17.3	1	p3	(TTA)5	15	1066	1080	GGTATTTTTGCATGCGGTTT
scaffold621669_17.4	1	p3	(TTA)6	18	40	57	TCATTTTCATTTTCTTTTGTTTTTG
scaffold621918_12.3	1	p3	(GCC)6	18	349	366	GGCACACTATTTTGGAGGGA
scaffold621921_10.(	1	p3	(CGG)5	15	87	101	TTTTTCGAATATTCCGGTGG
scaffold622045_14.6	1	p3	(TAT)5	15	334	348	AATAACATTAAACCCTAAACCCTAAA
scaffold622147_15.4	1	p3	(TGA)5	15	1770	1784	TTTGATTGGGAATAATTGGG
scaffold622452_15.9	1	p3	(TCC)6	18	555	572	ATCCTCCATTTCCCATTTCC
scaffold622493_18.4	1	p3	(ATC)5	15	1284	1298	TAACTGGGCTGGCTAGGTGT
scaffold623095_14.2	1	p3	(TGC)5	15	287	301	ATATGCGCAAGCTCACCTTT
scaffold623307_13.(	1	p3	(CAG)5	15	868	882	GAAATCAGCCCGACTTGAAA
scaffold623833_13.7	1	p3	(TGC)6	18	1435	1452	ACCTCATTCCTCCCAATTCC
scaffold624068_16.4	1	p3	(TAA)5	15	115	129	TCTTGATGCTCACTGTTGCC
scaffold624072_10.8	1	p3	(TTA)8	24	859	882	ATTTTTCGTCGGAGGGACTT
scaffold624105_15.2	1	p3	(AAT)5	15	485	499	TAAAGAGATGCTTGGTCGCC
scaffold624217_12.6	1	p3	(ATA)5	15	1302	1316	ATAGTGGGGCCTCGTCTTTC
scaffold624389_10.4	1	p3	(ATA)5	15	354	368	AAATTATCAAATTTCGGGACGA
scaffold624545_15.6	1	p3	(TTC)5	15	2587	2601	AATCAAATCCAATCCCACCA
scaffold624547_17.(	1	p3	(AGG)5	15	683	697	GGATCTCGGGGAGCTAGAAC
scaffold624557_11.6	1	p3	(TGG)5	15	301	315	CGGGCCAATAAAATCTCAAA
scaffold624793_20.:	1	p3	(TTA)6	18	1593	1610	CGATCAAGGAACACATGCAC
scaffold625006_13.5	1	p3	(CGG)5	15	1432	1446	CAATGAACAGTTCGTCGGTG
scaffold625317_16.9	1	p3	(ATC)6	18	1627	1644	ACGAAAGCTGTCGGAAAGAA

scaffold625411_10.	1	p3	(CGG)5	15	141	155	GATTATTCTTGCCGTTGGGA
scaffold625752_15.	1	p3	(GGC)6	18	3602	3619	ATGATGGACTTGCTTTTGGG
scaffold625786_23.	1	p3	(TTG)5	15	68	82	TTTTGAATTGCTTTAATTTGTTGC
scaffold625942_15.	1	p3	(ATC)5	15	397	411	TTGAACGCCGTTAAGTTTGA
scaffold625965_15.	1	p3	(CCG)5	15	3722	3736	CAAAGGGGCTGAGTTGAGAG
scaffold626100_15.	1	p3	(ATT)5	15	6849	6863	TCAAATTTTGGCACTGTTGC
scaffold626167_12.	1	p3	(ATA)5	15	579	593	TAATTTGGTTCGGTTTTCGG
scaffold626207_14.	1	p3	(TAT)6	18	1624	1641	ATCCGTGCAGTAAAATGAAAA
scaffold626724_12.	1	p3	(CTC)5	15	283	297	ACGTTCGATCCAAAACCAAG
scaffold627157_14.	1	p3	(ACC)5	15	538	552	CTCTCATCTTCACCGCCACT
scaffold627181_15.	1	p3	(CAT)6	18	2818	2835	CTCTGGTGCAACTCCATCAA
scaffold627204_14.	1	p3	(ACA)5	15	608	622	ACTCAGCGAAGTGGCAATCT
scaffold627229_10.	1	p3	(GAG)5	15	306	320	ATGAAGAAGGAGGAGGGGAA
scaffold627828_15.	1	p3	(TGG)5	15	1968	1982	CACTTTTGGCGTTTGATTCC
scaffold627880_16.	1	p3	(TAT)5	15	949	963	TTAATGGGATGATAAGCGGC
scaffold627993_19.	1	p3	(TAT)6	18	260	277	GCTAAGTTGATTATGGTGGGC
scaffold628120_11.	1	p3	(AAT)6	18	48	65	CAGTTGCCCTTGGGATTAGA
scaffold628394_19.	1	p3	(CCG)5	15	965	979	CCCATCCGGAAATCTCTACA
scaffold628504_15.	1	p3	(TCA)5	15	689	703	GCATCTTCCCACAAGCAAAT
scaffold628603_15.	1	p3	(TCT)6	18	84	101	TTTGCTGAATTTTCTTGGCA
scaffold628604_17.	1	p3	(CGC)5	15	1477	1491	GCAGCCACAAATGACAGAGA
scaffold628769_11.	1	p3	(TAT)5	15	330	344	AACATGAAAAATCCTCAATCCC
scaffold628848_13.	1	p3	(ACA)5	15	91	105	GGCCAAACGTTTAGTCTACTTGA
scaffold628856_11.	1	p3	(ATG)5	15	29	43	TGTTTCCATCTACACCTTGAGAA
scaffold628888_15.	1	p3	(TTC)5	15	336	350	TGAGGTAATGGAACCCGGTA
scaffold628956_16.	1	p3	(AAT)5	15	254	268	TTTCTACATTGATGCCGACG
scaffold629305_14.	1	p3	(TCA)7	21	77	97	ATTGAGATCCATGGGGAATG
scaffold629403_17.	1	p3	(AAT)5	15	139	153	TTGATTTTGCTTTTGCATCG
scaffold629425_15.	1	p3	(TAT)5	15	510	524	TTGCAATAAACATTGGGGGT
scaffold629495_17.	1	p3	(TCT)5	15	137	151	CGTTTTCTTATCTCTGCGCC
scaffold629570_20.	1	p3	(CGG)5	15	1412	1426	GGAAAACGCATAATTCACCC
scaffold629585_15.	1	p3	(AGA)6	18	116	133	AAGGATGAGGAAGGCAAGGT
scaffold629724_15.	1	p3	(ATC)5	15	1579	1593	GCATTGCATCCTATCCCATT
scaffold629878_16.	1	p3	(GGA)5	15	316	330	AGTTGGACTCGGCTTTTGAA
scaffold629924_16.	1	p3	(TGA)6	18	3900	3917	AGGTTGCGTACATCTGACCC
scaffold630380_14.	1	p3	(GCC)6	18	309	326	TCGGTTGCGTTGTTATTTGA
scaffold630710_10.	1	p3	(AAG)5	15	379	393	GCCATTCTTAAAAGCCTCATTT
scaffold631006_16.	1	p3	(AAT)5	15	195	209	TGTCTCCCTCACACCCTCTT
scaffold631007_14.	1	p3	(CAA)6	18	137	154	AACAGGTTCAAACCAGTGGC
scaffold631260_16.	1	p3	(CAA)6	18	1159	1176	ATCTTGAACCCCACCATCAA
scaffold631334_10.	1	p3	(AAT)5	15	316	330	TGGAAAATCGTTGTTGACCA
scaffold631549_11.	1	p3	(AAG)5	15	59	73	TGAGAAGGTTTTCTGGGGAA
scaffold631652_10.	1	p3	(GCG)6	18	449	466	ATTACGGTGCCAATTTCAGC
scaffold631718_16.	1	p3	(TTA)5	15	305	319	GGTTCGGTCGGTTTAGGACT
scaffold631851_14.	1	p3	(AGA)5	15	80	94	CCCAAGATGACTAACCGCTC
scaffold631867_15.	1	p3	(TTG)5	15	2635	2649	CCTTAAAATCAGTCCCGCAA
scaffold632060_14.	1	p3	(TCT)5	15	1077	1091	CGGCCCAATATTTAGGCATT
scaffold632138_15.	1	p3	(TGT)8	24	40	63	TGTAGCCGACCCCAATTTAT
scaffold632186_10.	1	p3	(GCG)5	15	457	471	AATTCTTCATTCAGCGACGG
scaffold632199_10.	1	p3	(TTC)6	18	106	123	CCAAGAGACTGAAAGCTCCA
scaffold632312_10.	1	p3	(ATT)6	18	141	158	GGTTTTAAATTCGTTGAAACCCT
scaffold632435_10.	1	p3	(AAC)6	18	279	296	ATCAACCTACACCGTCCACC
scaffold632455_17.	1	p3	(GAA)5	15	544	558	AGAAATCCAAAAGGCAACGA
scaffold632556_12.	1	p3	(GAA)5	15	541	555	CCGCATCCTTTGATATGCTA
scaffold632565_12.	1	p3	(ACA)6	18	106	123	TAATCCTTGAACTCGGTGGC
scaffold632584_15.	1	p3	(AAT)6	18	331	348	AACAAGTCGGCAAAAATTGG
scaffold632602_14.	1	p3	(GAA)6	18	392	409	GAGTAATCGTCTCCGCTTGC
scaffold632911_14.	1	p3	(AAG)6	18	187	204	CTTGCCAGGTTGCAATTATG

scaffold632969_15.9	1	p3	(AAT)5	15	1352	1366	GCATTTGAAACAATGCCAAA
scaffold633117_20.8	1	p3	(CGC)5	15	651	665	GCGAAAGCCCAGAACTTGTA
scaffold633225_16.5	1	p3	(CAT)6	18	1990	2007	TCTGTGGTTGTTGGCATAGC
scaffold633236_18.2	1	p3	(CCG)5	15	431	445	TTCAGCCACTGACCTAACCC
scaffold633334_13.6	1	p3	(ATT)8	24	45	68	CCATTGCACTCCGAATAGGT
scaffold633415_13.0	1	p3	(CTT)5	15	408	422	GCCAATTCTGTGGACCTCAT
scaffold633538_19.8	1	p3	(CCG)5	15	41	55	CACCAAAGGCAGGAAAGAAG
scaffold633765_14.1	1	p3	(TAT)7	21	1297	1317	TTAACATGGAACCCGTAGCC
scaffold634138_12.1	1	p3	(GCC)5	15	52	66	AGATGATGAATCCGCAGCAT
scaffold634290_14.5	1	p3	(TAA)5	15	383	397	CGTGAGAAGATTGGGAGGAA
scaffold634484_16.7	1	p3	(GAA)6	18	1946	1963	TCTGGCCTTCTTCGTGTTCT
scaffold634564_11.5	1	p3	(TTA)5	15	579	593	GAATTCCCAACCAAAGCAAA
scaffold634749_10.3	1	p3	(GAC)5	15	207	221	CGAATTTCAGAAGCGCTGTA
scaffold634787_18.4	1	p3	(ACC)5	15	479	493	ACCCAGCCTCGATTATGTTG
scaffold634798_15.9	1	p3	(TAT)5	15	262	276	AGGAATCCCAATCAGAGAAAA
scaffold635102_14.5	1	p3	(GCA)5	15	386	400	CTTCAGCTTGGTTTTGGAGG
scaffold635208_15.3	1	p3	(GAG)5	15	1619	1633	GTCGGATTTCTTCATCGGAA
scaffold635225_13.6	1	p3	(TGA)5	15	221	235	GCAATGTATATTGGGTCGCA
scaffold635282_17.0	1	p3	(CGG)5	15	32	46	ATCTACAACAGGCGGCGG
scaffold635292_16.1	1	p3	(CCA)8	24	878	901	TCTTCTCTAGCCTGGTGGGA
scaffold635533_18.7	1	p3	(CTC)6	18	189	206	AAAACCGCAAATTGAACGAG
scaffold635783_13.2	1	p3	(TCA)5	15	171	185	TCAAACCCATCATGAAATCCT
scaffold635850_15.4	1	p3	(ACA)6	18	361	378	CGACTAATGCTCAAACGCTG
scaffold635902_14.2	1	p3	(GCC)5	15	191	205	GGGGTATTCCCGATTTCAGT
scaffold635926_14.3	1	p3	(ATT)6	18	1973	1990	GTCACGCCTGTTTCTGTTGA
scaffold636079_11.4	1	p3	(ATT)5	15	77	91	GTCTCAATGGGGACATCTGG
scaffold636104_16.3	1	p3	(GAA)5	15	503	517	TGAAAGTCATGCGAGGAGTG
scaffold636166_16.0	1	p3	(TAA)5	15	540	554	GAAATTTCCTCCCTCGAGATG
scaffold636416_10.3	1	p3	(AAT)7	21	29	49	GGGTCCAACTACGGATAATGC
scaffold636556_15.5	1	p3	(TTG)5	15	425	439	TTTCAAGCAACCTCGCTTTC
scaffold636586_17.3	1	p3	(GAA)5	15	825	839	CGTGCCTGGAATTTCATTTT
scaffold636945_15.5	1	p3	(CTC)8	24	580	603	AACTGAAACGACACCTTGGG
scaffold637444_15.1	1	p3	(CAA)6	18	2480	2497	AAACGGACAACCACTTTTCG
scaffold637467_10.7	1	p3	(ATA)6	18	514	531	TCGCCACTGAAAGAAAGAGG
scaffold637816_16.8	1	p3	(CTC)6	18	879	896	AAGGGTCCCATCCCACTTAC
scaffold637823_11.7	1	p3	(AAT)6	18	51	68	CATTCAAAATAAAATGATGATGATAA
scaffold637945_10.0	1	p3	(ATT)5	15	400	414	ACTCGACCAAAAAGGTGTCG
scaffold637965_14.8	1	p3	(TGA)5	15	1144	1158	AAGTGATGCAGGGAATCTGG
scaffold637984_13.5	1	p3	(AGA)6	18	1718	1735	GCCAGGAGAAAGCAGTGTTC
scaffold638045_16.4	1	p3	(ATT)5	15	276	290	TTGACTCGCGTAAAAGAATATAAAAA
scaffold638060_10.2	1	p3	(TTA)7	21	245	265	CGAAATTAATTAAGGAAAAGATTCCA
scaffold638078_20.9	1	p3	(GAT)5	15	416	430	CACCCTTTTTGTGGAAGCAT
scaffold638085_16.2	1	p3	(ATA)5	15	3528	3542	CGTGAGAATCTTCGACCTCC
scaffold638190_15.4	1	p3	(AAC)5	15	896	910	ACGACATGAAGCTTCTGCAA
scaffold638229_16.7	1	p3	(ACA)6	18	1511	1528	TCACAATAAACCTCCAGTTCCC
scaffold638302_14.0	1	p3	(ATT)5	15	545	559	ATTTTGCTCACCCTGCTCAT
scaffold638303_12.7	1	p3	(AAT)5	15	646	660	ATGGGGACATCAAGTTGGAA
scaffold638358_14.9	1	p3	(GCC)5	15	386	400	GTCTAATGTTTCCGCCTCCA
scaffold638386_16.1	1	p3	(CGG)5	15	319	333	ATTTTTATGGATTGTGCCGC
scaffold638527_12.5	1	p3	(ATA)5	15	142	156	TTTTTGTTTTTGACGGGTCC
scaffold638702_15.8	1	p3	(ATC)5	15	561	575	CGCAGTTGATCTTCTGTCCA
scaffold638872_17.3	1	p3	(TGT)5	15	44	58	TGTAGCCGACCCCAATTTAT
scaffold638901_15.1	1	p3	(AAT)5	15	1716	1730	TCCAAAATAATTCGGCCAAC
scaffold639087_15.9	1	p3	(AGA)5	15	911	925	AGCATTAACGATGCCTTGCT
scaffold639313_12.9	1	p3	(ATA)5	15	924	938	CGCATCTTCCGAGCTATCAT
scaffold639585_10.3	1	p3	(CAA)5	15	92	106	TGGGCTAGTAATCTGGGTCCT
scaffold639603_18.0	1	p3	(GAA)5	15	828	842	CCTAAAAGGTTTTGGGGATCA
scaffold640294_11.6	1	p3	(TTG)5	15	292	306	AATGTTGATGCTGTTTGGAGC

scaffold640334_12.4	1	p3	(TAT)9	27	175	201	GCCACTTGTAACCTCCCAAA
scaffold640409_18.5	1	p3	(ACA)6	18	358	375	AATCTCGATGCCATCTCCTG
scaffold640570_15.4	1	p3	(ATT)8	24	99	122	TGCTAAAAACAAATGCCAAGG
scaffold640572_18.2	1	p3	(ATT)5	15	311	325	AAACGTGAAGTCAATTTCAATGAT
scaffold640708_16.6	1	p3	(TGA)6	18	704	721	GCATCATGCATGGTCTTACC
scaffold640727_21.1	1	p3	(CTT)5	15	23	37	AAAAATTGGTGCGGGTGC
scaffold640769_14.0	1	p3	(ATA)5	15	155	169	GGAAAATTGGGTCATATAGCTCC
scaffold640833_12.1	1	p3	(TTA)6	18	565	582	TTGTTGCAGTAGCCATGCTC
scaffold640845_13.0	1	p3	(TCT)6	18	182	199	CCCCCTACACCACTCTTTCA
scaffold640903_10.1	1	p3	(GGC)5	15	56	70	TGTCAACGTAAAAGGAGGGC
scaffold641098_18.8	1	p3	(GAA)6	18	208	225	CCATTCATCGTCGAAGACCT
scaffold641454_16.0	1	p3	(TTG)5	15	917	931	TGTTATTTGCACAATCATTTCCTT
scaffold641545_11.8	1	p3	(GAG)7	21	998	1018	ATGCTTTGCTTCTTATGCGG
scaffold641557_11.6	1	p3	(TTC)7	21	54	74	GATCAGCAGTGGCGTTGTC
scaffold641720_19.8	1	p3	(GCG)7	21	191	211	CATTCTTTCTCCGTCTTCCG
scaffold641800_12.4	1	p3	(TTC)5	15	147	161	TGCTTTTCAGTTGGCATTTTT
scaffold641821_15.4	1	p3	(AAT)5	15	271	285	AATTTCTCCCACTTGCCTTG
scaffold641897_15.8	1	p3	(AAT)6	18	415	432	TTGGCAGCTAGTGCTCATTTT
scaffold642166_16.0	1	p3	(TGA)6	18	43	60	CCCAAGACCCCAATTTGATT
scaffold642361_15.9	1	p3	(GCC)5	15	492	506	CACCAATCAATAATGCACGC
scaffold642479_10.5	1	p3	(ATT)5	15	91	105	GGATAGAATGAGGGTTTTCCC
scaffold642489_15.4	1	p3	(ATC)7	21	1776	1796	CGTGCGTGTTGAATTCATGT
scaffold642561_21.8	1	p3	(ACA)5	15	142	156	CGTGCCAATAATGACACAAAA
scaffold642568_10.4	1	p3	(AAC)5	15	685	699	CGTTGTGCTCTTGAAACTGG
scaffold642595_14.7	1	p3	(TGG)6	18	1568	1585	GTTTCCTCACTACACCCCGA
scaffold642917_18.9	1	p3	(TTC)5	15	33	47	GGATCAGCAGTGGCTTTTTC
scaffold642956_11.6	1	p3	(AAG)5	15	217	231	CCGGAATCTGAGAGAGATCG
scaffold643117_15.8	1	p3	(CCG)5	15	942	956	CATCAAAAGCAGAGCAGCAC
scaffold643125_14.0	1	p3	(AAT)6	18	179	196	CGCATACGATCTTGATTATGG
scaffold643174_16.8	1	p3	(TTG)5	15	376	390	TTGGGATTAAGGCTTTGGTG
scaffold643178_10.5	1	p3	(ATA)5	15	431	445	TGGTGAAAAATCAAATACTTGGAA
scaffold643179_12.7	1	p3	(ATA)5	15	462	476	TGGTGAAAAATCAAATACTTGGAA
scaffold643326_14.1	1	p3	(GTA)5	15	490	504	TGGAATAGCTTGATAAATGATTTGAA
scaffold643440_14.2	1	p3	(CCA)5	15	616	630	AATCATCACAAGCCCCAAAG
scaffold643544_12.2	1	p3	(TTG)7	21	63	83	TAGCCGACCCCAACTTATTG
scaffold643773_11.9	1	p3	(AAG)5	15	67	81	GGGGGAGAAAAATAAAGAAAAA
scaffold643804_16.9	1	p3	(CTT)5	15	968	982	TCGAGAACCAATAATGATGAGC
scaffold644040_17.6	1	p3	(TTA)6	18	1197	1214	TTTGGTTTTGCTCCTCTGCT
scaffold644060_12.8	1	p3	(ATA)6	18	1295	1312	TCTTCCTTGTTTCTTTCTTTGGA
scaffold644115_10.3	1	p3	(AAG)5	15	198	212	CGAACAGAGGCAGCATAAAA
scaffold644287_15.9	1	p3	(ACC)6	18	37	54	CCTCTCCTCCTTCTTCCCAC
scaffold644626_15.3	1	p3	(TGT)5	15	210	224	AACCTTGGTTCATGTAGCCG
scaffold644647_10.5	1	p3	(AGG)5	15	112	126	ATTGACAGCAGGGAGGTGAT
scaffold644724_12.1	1	p3	(TGT)5	15	64	78	TTGGTTCATGTAGCCGACCT
scaffold645046_15.2	1	p3	(GGA)5	15	962	976	TGGTAAAATTCCACTTCGGC
scaffold645069_15.6	1	p3	(AAT)7	21	43	63	TGTTCGTATTAAATTACCGGGAG
scaffold645106_16.2	1	p3	(TGT)5	15	721	735	AATGGCAAAGGTAAATGAGCA
scaffold645203_14.4	1	p3	(ATA)6	18	803	820	AACCATTTAGAATTTTTCGGGA
scaffold645264_12.5	1	p3	(AAT)5	15	724	738	GGTCAGTGAAGCCCACCTTA
scaffold645280_18.5	1	p3	(TAT)5	15	463	477	AAGTCCAGCACTCCCAATTT
scaffold645295_11.7	1	p3	(CAT)5	15	150	164	GCGAGACAACGAAATTCTCA
scaffold645321_10.2	1	p3	(TGA)8	24	43	66	CCCAAGACCCCAATTTGATT
scaffold645365_16.1	1	p3	(AAT)5	15	367	381	GGTTGATCGTGTTGAATTATGG
scaffold645433_16.9	1	p3	(TTG)5	15	612	626	GTAGCAGCTCGGTGAGGTTC
scaffold645464_14.8	1	p3	(CTT)5	15	165	179	GACGATGGTTGCCTTTGTTT
scaffold645504_16.6	1	p3	(GAG)5	15	307	321	TGTTGGTGGTGGTGTCTTGT
scaffold645844_12.2	1	p3	(TTA)5	15	349	363	GAGAGCAGTCCTTTTCCCG
scaffold645973_14.9	1	p3	(CAT)7	21	855	875	CGTTCCTTGCAACAACAAGA

scaffold646043_16.6	1	p3	(AAT)6	18	381	398	TCTTGCCTCGATTCCTTGAT
scaffold646215_18.0	1	p3	(TGC)5	15	329	343	TGACCAGATAACATCGGCAA
scaffold646236_15.1	1	p3	(AAT)5	15	2478	2492	TGTGGGGACGATTATTCCAT
scaffold646256_14.9	1	p3	(ACA)7	21	2474	2494	CAGGAAAACCCAGAAGTGAGA
scaffold646375_16.3	1	p3	(CAT)6	18	1184	1201	GAAGCAAAAACAAACCCACC
scaffold646382_15.1	1	p3	(TTA)5	15	477	491	AAAACCTCATAAAACTGTCATGTCT
scaffold646448_14.8	1	p3	(TTC)6	18	196	213	TCTTGCTGCTTTTTCCACCT
scaffold646570_18.0	1	p3	(TGA)6	18	884	901	AATTGGGGTGCAAAACAAAG
scaffold646599_14.0	1	p3	(ATA)5	15	2372	2386	GAACCCAAGAAGCAACATGTAA
scaffold646648_11.4	1	p3	(AAT)5	15	625	639	TAGGGTTCATGTCCGGTTGT
scaffold646769_17.7	1	p3	(ATA)9	27	185	211	CGTTCCAAACTTCGAAGAAAA
scaffold646867_13.8	1	p3	(TTA)6	18	2075	2092	TTGACAACATTGCCACATACC
scaffold646919_16.0	1	p3	(CAC)6	18	626	643	CAGCTGAGGGAGGAGTTTTG
scaffold647024_14.7	1	p3	(TCT)7	21	365	385	TCCAGCTATCCCGTTTTGTC
scaffold647069_15.2	1	p3	(GGT)6	18	730	747	GCAGTGGCGTTGAACTAACA
scaffold647092_16.9	1	p3	(TAA)5	15	2370	2384	CCACCTCACGCAAAAAGAGT
scaffold647177_19.1	1	p3	(TTC)5	15	784	798	AAATAAGGCATGGGTTGTGC
scaffold647326_10.3	1	p3	(TTG)5	15	472	486	GAGCACGTGGAGAAGAGACC
scaffold647372_12.1	1	p3	(ATA)5	15	545	559	CGAAGTCTCACGGTTGATTG
scaffold647492_10.0	1	p3	(GGC)5	15	116	130	ATTCCGGTGACGGTTGAATA
scaffold647532_17.0	1	p3	(GCT)5	15	978	992	AGGGGGAAAGCAAAGAACAT
scaffold647558_15.9	1	p3	(ACC)5	15	643	657	ACTTCTCCTTCCACCGCTCT
scaffold647656_16.4	1	p3	(TAA)6	18	849	866	GAACTGACTTCGGGGTGAAA
scaffold647758_14.0	1	p3	(ATA)5	15	167	181	CAGTAAAATGCTGCCCAGGT
scaffold647817_15.0	1	p3	(CTT)5	15	1608	1622	ACTCAGATCACTCAACCCGC
scaffold648009_14.8	1	p3	(GAG)5	15	104	118	GAACGTGGGAGGTGAAGAGA
scaffold648136_20.5	1	p3	(GAA)5	15	579	593	CACGTCGAGCTAGAACCTCC
scaffold648138_13.7	1	p3	(TAA)7	21	1323	1343	CAATGAGGACATCCGAGGAG
scaffold648152_16.2	1	p3	(GGA)5	15	3721	3735	TCCTCTGTTGTGTTGGATGC
scaffold648392_16.5	1	p3	(GGT)5	15	3512	3526	ACAGACCCTCACACCATTCC
scaffold648520_17.9	1	p3	(AAT)5	15	63	77	TGTGTGCGATTTAATCCTAACC
scaffold648567_11.2	1	p3	(TTC)5	15	104	118	TCCATAAGCCATAGCTAAAAGAGG
scaffold648595_16.7	1	p3	(TGT)6	18	2274	2291	GCTGACACAACCACAACACC
scaffold648864_12.8	1	p3	(TAT)5	15	1109	1123	TCATTTTATCATGAATCCCAACA
scaffold649189_16.3	1	p3	(TTG)8	24	1891	1914	AACCTTCTCTCTGGTGCCAA
scaffold649219_14.0	1	p3	(TTG)5	15	50	64	TGTAGCCGACCCCAATTTAT
scaffold649257_13.0	1	p3	(GCT)5	15	314	328	AAAGCATACGGGTGAAGTGG
scaffold649376_18.2	1	p3	(GCC)6	18	3377	3394	TTCACCACCAGATCCTCCTC
scaffold649470_11.5	1	p3	(TTA)7	21	115	135	CTTCGCCGCATATGAAACTT
scaffold649912_17.2	1	p3	(TTA)8	24	1309	1332	TGAACTCGCTCCTTCCATCT
scaffold649917_17.5	1	p3	(GGT)5	15	356	370	CAGAAATTATCCCACACCCG
scaffold650244_10.3	1	p3	(AAT)5	15	46	60	GAACCCTTTCACCGTCCTCT
scaffold650477_16.0	1	p3	(TAT)6	18	3505	3522	CAAGCAAAATGTGTTGATTGTT
scaffold650582_13.5	1	p3	(ATA)7	21	670	690	CCATTTCCCGAAACGATAGA
scaffold650652_13.7	1	p3	(GCT)5	15	514	528	AAATGCTGCTGTGTCGTGAG
scaffold650732_16.4	1	p3	(ATC)5	15	160	174	TCAGAGGTGCACTCGAAGTT
scaffold651127_19.4	1	p3	(CGG)5	15	1634	1648	GACCAGTTCACAAAAGTCCCA
scaffold651206_16.4	1	p3	(ACA)5	15	334	348	CCACCGGACACATTCAATTA
scaffold651265_13.0	1	p3	(AAT)5	15	499	513	ATATTTGGTTGGTTCAGCGG
scaffold651362_12.5	1	p3	(TAT)5	15	259	273	AACTCGGCAAGATGAACTGC
scaffold651688_13.8	1	p3	(CAT)6	18	157	174	AACATATTGACTCCCGTGCC
scaffold651713_10.0	1	p3	(GAA)6	18	344	361	TTCAGGGATTCGATTTGGAG
scaffold652005_16.5	1	p3	(GAA)5	15	527	541	TCAGAAAGGATCCTCCTCCA
scaffold652119_14.9	1	p3	(TCT)5	15	203	217	GGGTTCTTGGGTGTCAGAGA
scaffold652207_15.9	1	p3	(ATT)6	18	82	99	CGTGTAGGATGGGAAATTGAA
scaffold652222_15.6	1	p3	(TTG)6	18	286	303	CGATCTCACTTCGTGGGAAA
scaffold652326_11.6	1	p3	(CGG)6	18	72	89	ACATGAAGAGGACGAAGCGT
scaffold652402_16.9	1	p3	(AAT)5	15	230	244	GTTAACCGCATGTCACTTCG

scaffold652415_16.6	1	p3	(AAT)5	15	505	519	CCTATTTCCCAACCCAAACA
scaffold652416_13.2	1	p3	(ACA)5	15	1030	1044	GCATGGATCTTTGGGAGAAA
scaffold652452_15.8	1	p3	(ATC)5	15	943	957	ATCTCCACCACCACCATCAT
scaffold652622_16.6	1	p3	(CTC)6	18	160	177	TTCCGTTGGCTTATTTTTGC
scaffold652819_13.9	1	p3	(GGC)5	15	136	150	AGAGTTATTCAGGCCAGCCC
scaffold652827_19.1	1	p3	(GCC)5	15	780	794	GTTGCTCAAATTCTTCCCCA
scaffold653011_17.2	1	p3	(ATT)5	15	746	760	TTCTGATGCTCTACCAAAACG
scaffold653279_16.0	1	p3	(TTG)5	15	238	252	CACTGATCGTACACGATGGC
scaffold653323_12.3	1	p3	(TGG)5	15	351	365	GATAGGCGTTCGAAGGTGAC
scaffold653377_14.8	1	p3	(TAT)5	15	772	786	ACGATGCTCGAGGTTCAGAG
scaffold653469_18.1	1	p3	(TGG)5	15	1048	1062	TTCCATGTTCTGGAGAAGCC
scaffold653573_16.6	1	p3	(TAA)5	15	821	835	CATTCCACCGGAAGGTCTTA
scaffold653577_11.3	1	p3	(CCG)6	18	165	182	GAAAACCCCCACTCAACTCA
scaffold653804_10.3	1	p3	(CAA)5	15	117	131	TGCAAAAACATCTTTCACAATCA
scaffold653817_15.2	1	p3	(CAA)5	15	1106	1120	TTTGAATTTGGAAAAGTATGGAA
scaffold653978_10.4	1	p3	(CTT)5	15	159	173	TCCGGTGCTGTCTTTTCTTT
scaffold654081_12.9	1	p3	(AAT)5	15	287	301	AAAGTAGCGGTTGCGGATTA
scaffold654155_14.4	1	p3	(TTA)6	18	1084	1101	AACGTCAACGCTCGTACCTT
scaffold654157_15.2	1	p3	(GAC)5	15	1152	1166	TGATGAACTGCTGCCAGAAC
scaffold654174_11.0	1	p3	(AAT)5	15	64	78	TGCATAATCAACGGTCAATCA
scaffold654317_14.0	1	p3	(ATC)5	15	511	525	CACCTCTCATTCACATGCCA
scaffold654748_15.1	1	p3	(ATA)5	15	759	773	CCAAAAAGGTGAAGGAAACAA
scaffold654864_14.9	1	p3	(ATT)5	15	921	935	GTGAGCCACGCACTCTGTAG
scaffold654872_16.1	1	p3	(GCC)5	15	436	450	TTCTTCTCCTCTGCCTCCCT
scaffold655196_15.0	1	p3	(TTA)5	15	496	510	GGTGTTTTCGTTGGAGCTGT
scaffold655208_12.2	1	p3	(GCC)5	15	894	908	CCCAACTTCCAACATCGAAT
scaffold655253_15.3	1	p3	(CCT)5	15	1467	1481	AAATTCCCCACCATTTCCTC
scaffold655440_15.9	1	p3	(ATC)7	21	162	182	CGCCTTGAGTTTGGGTAAAA
scaffold655761_14.4	1	p3	(GAT)5	15	1541	1555	TTTGATTGTGCGATGTTTGG
scaffold655803_16.2	1	p3	(TTG)5	15	3841	3855	AGTTCCCAATTTTTGGCTCC
scaffold655841_12.8	1	p3	(TAT)8	24	644	667	TGTTCTGTTAAAACTTCACATCTCA
scaffold655903_10.2	1	p3	(CCG)5	15	314	328	TCGATCCGGGACTTGAAATA
scaffold656025_18.3	1	p3	(ATA)5	15	59	73	CTTCAAATGAATCTTCAACAGGA
scaffold656244_11.1	1	p3	(TAA)6	18	204	221	TGAAGCCCAACAGATTGATTT
scaffold656330_12.1	1	p3	(ATT)5	15	36	50	GAATTCTGAATGCATGGTTCTT
scaffold656543_10.7	1	p3	(CGG)5	15	269	283	ATGGCGAGGGAGAGAGGTAG
scaffold656926_16.6	1	p3	(GAG)5	15	670	684	CCATCTCTTTCCAGAGCGTC
scaffold657050_15.7	1	p3	(TTG)6	18	336	353	CGACCTCACATAGTGGGACA
scaffold657177_19.9	1	p3	(ACA)6	18	1293	1310	GTGCACATGTCAAGTTTGGG
scaffold657676_14.9	1	p3	(ATA)5	15	894	908	TTGACCCCAATCTCGACTTC
scaffold657791_15.7	1	p3	(CTT)5	15	448	462	CCGCTGATCTTTCTGCTCTT
scaffold657905_12.1	1	p3	(CAC)5	15	322	336	GATTGCCTTGTTTACCCGTG
scaffold658273_18.4	1	p3	(ATC)5	15	164	178	ATGGAATCTGGGGTTCAACA
scaffold658434_10.1	1	p3	(TCA)5	15	638	652	CCCTGCCAATAGAAGATCCA
scaffold658636_12.1	1	p3	(TCA)5	15	384	398	TGGGCGAAAATTCAACTAGG
scaffold658782_16.6	1	p3	(TGA)6	18	45	62	CCCAAGACCCCAATTTGATT
scaffold658976_13.1	1	p3	(ATA)5	15	462	476	TGAGCGAGGTGAAGAGTCAA
scaffold658992_11.8	1	p3	(TGT)5	15	36	50	CCAATTTATTGGGATTAAGGCT
scaffold659030_15.2	1	p3	(TTA)5	15	901	915	CACGCGGATCTGTGTCTATTT
scaffold659075_11.5	1	p3	(TTA)7	21	1139	1159	TGCAATTTGGTTCCATTCAA
scaffold659115_12.2	1	p3	(ATT)5	15	104	118	TTGCCTTTAATGCTGTGCTG
scaffold659221_13.0	1	p3	(CGC)5	15	322	336	TGGTCGTAAGTTGGAAAGGG
scaffold659240_16.6	1	p3	(TCA)10	30	2769	2798	TCCCAAAATGAAACTTGTAAATCA
scaffold659269_13.6	1	p3	(ATT)5	15	123	137	AAAGGGCCTTAACACCAACA
scaffold659415_16.4	1	p3	(GTG)5	15	638	652	TGATTCATCGCATTTACGGA
scaffold659462_12.7	1	p3	(ACC)5	15	2243	2257	CGAGGCATAACCTCACCAGT
scaffold659934_10.0	1	p3	(ACA)6	18	540	557	AAGCTAAGAGCTCCTTGGGG
scaffold659958_16.8	1	p3	(TCC)5	15	545	559	GGTCCCACTCGAGTCTCATT

scaffold659977_18.2	1	p3	(TCC)5	15	388	402	TTTGACATCTCTGCTGGTGG
scaffold660176_16.1	1	p3	(TGT)6	18	47	64	TTGGTTCATGTAGCCGACCT
scaffold660204_16.1	1	p3	(AGC)5	15	212	226	ATTCAGCTCTTGTTTCCCCA
scaffold660324_15.8	1	p3	(TCT)5	15	1433	1447	AATCTCACGCAAGGTGCTTT
scaffold660419_13.5	1	p3	(TTC)7	21	498	518	ACATTTCTTTGGCTTCCCCT
scaffold660463_16.8	1	p3	(TCA)6	18	5085	5102	ATTTTCCTCAACTTCGGCCT
scaffold660578_16.8	1	p3	(ATT)5	15	1291	1305	ATAATGCCGGTGCAGGTCT
scaffold660598_15.6	1	p3	(AAG)5	15	979	993	ATGCATGCAAACCAGGATCT
scaffold660696_15.4	1	p3	(ACA)6	18	404	421	CTACCGGAGGAAACCATGAA
scaffold660748_11.6	1	p3	(TTA)5	15	617	631	CTTCCACCCTTGTTTTCCAA
scaffold660899_10.7	1	p3	(CGC)5	15	289	303	ATGGTGGTAAGGTGTGGAGG
scaffold660949_17.2	1	p3	(CTT)5	15	206	220	AGATCCCTCCCTCGCTACAT
scaffold661306_16.8	1	p3	(ACA)6	18	4868	4885	GGGTGGTGAAGAATGAATGC
scaffold661352_13.8	1	p3	(TCC)5	15	438	452	CAGTCACGGGATATGCAGAA
scaffold661391_16.6	1	p3	(GGT)5	15	493	507	TTGGGGATTTCTGAATTTGG
scaffold661410_12.8	1	p3	(GAA)5	15	1037	1051	TTCTCTTGCAATGCTTCACG
scaffold661463_17.0	1	p3	(TAG)5	15	90	104	GAGGAGAGCAGCCACAAGAA
scaffold661529_13.2	1	p3	(TAT)5	15	985	999	GGTGCGGGATAAAAATGTTG
scaffold661560_14.7	1	p3	(GAT)5	15	404	418	ATATCAGCTCGCACAATCCC
scaffold661569_13.0	1	p3	(TGT)6	18	44	61	TGTAGCCGACCCCAATTTAT
scaffold661701_15.3	1	p3	(ATC)5	15	558	572	TCCATTAACTCATCGTTAAAATTCA
scaffold661753_12.0	1	p3	(GAT)5	15	106	120	GGATGGGGATAGTGATAGGC
scaffold661841_14.6	1	p3	(TTA)6	18	1447	1464	CATATTCAGAGTCGTTTGAGCTT
scaffold662014_15.5	1	p3	(TAA)7	21	371	391	CTAATAGGGGGATTTTGCCC
scaffold662112_14.4	1	p3	(TTC)5	15	1287	1301	TGAACTTTTTGTTCCTGCGA
scaffold662215_12.6	1	p3	(TAT)6	18	201	218	CGAACAAGAAACGGGGATAA
scaffold662342_11.2	1	p3	(TAA)7	21	493	513	TGAAGGCTTTTCTCCTGCAA
scaffold662671_10.1	1	p3	(ATA)6	18	57	74	GGGGACATCTGAGTGTGTGA
scaffold663048_14.7	1	p3	(ATC)6	18	1315	1332	CCAAGTGTTAGTGGCCAACC
scaffold663140_11.6	1	p3	(AGA)5	15	547	561	ACAAGCAAATTTCTCCCCCT
scaffold663155_10.2	1	p3	(TGT)6	18	42	59	TGTAGCCGACCCCAATTTAT
scaffold663292_13.7	1	p3	(TTA)5	15	766	780	CCAATGTGATTGGCTAGTTGC
scaffold663347_15.7	1	p3	(AAT)5	15	977	991	ATGACCGAGGAGCAAATTGT
scaffold663475_15.4	1	p3	(GGT)5	15	2414	2428	CGGTTGACCCTGTTAGCATT
scaffold663798_11.0	1	p3	(GAG)6	18	321	338	GACACAGAGGCTGACGATGA
scaffold663925_13.1	1	p3	(AAT)5	15	334	348	CCGAGTAGCTGGGTCTGAAG
scaffold663931_19.5	1	p3	(CAT)5	15	697	711	TGTCAGTCAGGGATTCTTTTGA
scaffold663942_18.8	1	p3	(ATA)5	15	1443	1457	TGAGCATATTTTGTGTTGCTTG
scaffold664273_16.6	1	p3	(ATA)5	15	1368	1382	TTTAGTGGGAATCACGGGAG
scaffold664419_11.1	1	p3	(TTC)5	15	866	880	ATAATCGTCTCCGTCGCTGT
scaffold664531_13.4	1	p3	(GAT)8	24	982	1005	AGCAGAGGATTGTTGACGCT
scaffold664554_15.3	1	p3	(AAT)5	15	927	941	CACCCAACCCGAGTATCCTA
scaffold664565_16.6	1	p3	(TGT)6	18	31	48	CCCAGTTTATTGGGATTAATGTTT
scaffold664957_15.3	1	p3	(ATC)6	18	1133	1150	GTCCTTGCATTAGGCAGAGC
scaffold665059_16.4	1	p3	(TTA)5	15	2522	2536	CCCAAAAGGGAGGAGTGAAT
scaffold665352_15.4	1	p3	(AAT)5	15	2468	2482	GGGACAATGCTGATCTCCTC
scaffold665414_12.8	1	p3	(AAT)6	18	881	898	TTTTGACAATTCTCAGCCCC
scaffold665430_15.7	1	p3	(AAG)5	15	2517	2531	TAATCCCACTGAATTTGGGC
scaffold665432_12.8	1	p3	(ATG)5	15	66	80	CGAGCTCGATCATGCAACTA
scaffold665472_12.7	1	p3	(AAT)5	15	623	637	TGGTGAAAAGTAAGCATGCAA
scaffold665564_16.3	1	p3	(CCG)5	15	496	510	TACTCCCACCGGAAATCAAG
scaffold665580_17.7	1	p3	(CTT)5	15	1899	1913	TCGAATGCAACACTGGAGAA
scaffold665604_11.8	1	p3	(ATT)7	21	382	402	TGACGAGCTTCCCTCTTGTT
scaffold665608_12.2	1	p3	(GGC)7	21	1940	1960	GATCGGACAACCCATCAAAC
scaffold665663_14.0	1	p3	(TAT)6	18	1190	1207	TTTTGCTCACCCCTAACCAC
scaffold665688_17.6	1	p3	(CCT)6	18	499	516	CTTCTCCTCCATTTCCACCA
scaffold666123_19.2	1	p3	(TGT)5	15	681	695	TCAAACGAAAACATGCATCC
scaffold666243_15.4	1	p3	(ATT)5	15	1900	1914	CCAAGGTTGAAAGGGATCTG

scaffold666446_11.4	1	p3	(CCA)6	18	372	389	CCCAAACTCATGCAAAACCT
scaffold666461_16.1	1	p3	(AAT)6	18	1602	1619	TTACCTAGCAAGGGTTTGGG
scaffold666496_12.9	1	p3	(AGA)5	15	1258	1272	AGAGAGACGGGGAAGAGGAG
scaffold666519_17.2	1	p3	(ATT)5	15	730	744	GCCCATAAGCCCATCAAATA
scaffold666525_18.4	1	p3	(CCT)5	15	217	231	AATGGAAAATAAGCAGGGGC
scaffold666536_14.6	1	p3	(ATC)5	15	2922	2936	AGCACACTTCTCCTGCAACC
scaffold666537_17.4	1	p3	(TAT)5	15	297	311	GGGAATCTTGTTTTAAGTATCCGA
scaffold666555_16.7	1	p3	(CGC)5	15	218	232	AAGATCGAGAAAACGGAGGC
scaffold666612_13.5	1	p3	(TAT)5	15	3045	3059	CAGCCGCTACTCTTCGAGTG
scaffold666635_13.6	1	p3	(CTT)5	15	169	183	TGAGTGCATGCTCCAAAGAC
scaffold666641_15.0	1	p3	(TGA)5	15	1000	1014	CATTGAAGAAGAAGGCGGAG
scaffold666643_16.3	1	p3	(CCA)6	18	526	543	GAGAATTCCCGCAGATCAAA
scaffold666724_15.4	1	p3	(CCA)5	15	1401	1415	GCAGCCAAGTTTGGTTTGTT
scaffold666901_10.3	1	p3	(TAT)7	21	84	104	CCACCCCCTTCTTTTTCATT
scaffold666919_10.6	1	p3	(GAG)6	18	530	547	GAGATCCCCGTAAACAGCAA
scaffold667001_16.2	1	p3	(CGG)7	21	954	974	TGCATCTTGGCCCAACTATT
scaffold667145_11.8	1	p3	(TAA)5	15	333	347	AGGGCATGCATAGGTGTAGG
scaffold667272_16.5	1	p3	(AAT)5	15	4512	4526	TGAGCTCTGTTGGTCTTTGG
scaffold667488_14.8	1	p3	(TTA)5	15	1212	1226	TGATTGAAAGCACCGTCTTG
scaffold667520_12.2	1	p3	(TCA)5	15	173	187	TGGAAGAGTGCCCAAATACA
scaffold667604_16.5	1	p3	(CAG)5	15	1900	1914	CTTCGGCTCTCCAACTTCAG
scaffold667637_15.1	1	p3	(GAA)5	15	1265	1279	CAGCTCCAATGCAGTAGCAA
scaffold667908_15.9	1	p3	(ACA)6	18	1381	1398	TTTCCAAATTTTCTCCTCCG
scaffold667921_14.4	1	p3	(CCT)6	18	216	233	AGATGGATGTTCGAGTTGGG
scaffold667974_14.6	1	p3	(ATT)5	15	557	571	CCATAGGCGAATTCTAAAGTGA
scaffold668086_10.9	1	p3	(TTA)7	21	145	165	AAATTTTATGGGACGGAGGG
scaffold668175_16.0	1	p3	(CTT)5	15	1079	1093	TCAGTTTGCACACTTTCCCA
scaffold668473_15.6	1	p3	(GAC)5	15	2158	2172	TCTTAACGGATTCTCCAAAGATG
scaffold668550_11.5	1	p3	(TTC)5	15	324	338	TCCATCGTACCGAGGCTAAC
scaffold668599_13.0	1	p3	(GAA)7	21	279	299	AAGAGGTGTATGCCGGATTG
scaffold668617_18.4	1	p3	(AGG)5	15	2817	2831	GCGTGGGAATTGTTTCAAGT
scaffold668669_15.2	1	p3	(TTC)5	15	290	304	CATCATCCGTAAGCTCCCAC
scaffold668784_10.6	1	p3	(GGC)5	15	51	65	GGGGTCCCTGTCTTATAGCG
scaffold668791_15.0	1	p3	(CTC)5	15	2446	2460	CTTTCTAGTCCCCATTGCCA
scaffold668822_13.5	1	p3	(AAT)8	24	1288	1311	CGTATAGTCCCGAGCAGAGG
scaffold668860_15.7	1	p3	(AAT)5	15	1980	1994	TGCCTTGCGAATTGTGATTA
scaffold668868_15.0	1	p3	(TCT)5	15	648	662	TCGGCGTTCCTGTATTTTTC
scaffold668869_15.4	1	p3	(GCC)5	15	1191	1205	AGCCCATAAGCAACACGAAT
scaffold668932_17.6	1	p3	(ATC)5	15	363	377	TGGGTGCGAACAACATTATTT
scaffold669042_10.7	1	p3	(CGG)6	18	165	182	GCAGGCGATAAGCAGAAGTT
scaffold669360_13.6	1	p3	(TGA)7	21	42	62	CCCAAGACCCCAATTTGATT
scaffold669370_15.9	1	p3	(CCG)5	15	1540	1554	AGATCCAATCCAAATGCTGC
scaffold669488_11.5	1	p3	(GAG)6	18	360	377	CCTTATCTGGGCCTTCACAA
scaffold669500_11.0	1	p3	(ATT)7	21	82	102	CGTGTAGGATGGGAAATTGAA
scaffold669562_11.2	1	p3	(ACA)6	18	460	477	ATAGGTTGTTCAAAGCCGGG
scaffold669594_18.5	1	p3	(TGT)5	15	330	344	TTTGGGAGTGAATGGGAGAG
scaffold669672_14.8	1	p3	(CTT)5	15	481	495	TGAATGTAATGGTCGTGCGT
scaffold670000_14.1	1	p3	(TGA)6	18	1444	1461	GCTATGCCTGTGCCTGATTT
scaffold670038_17.5	1	p3	(CCA)5	15	1220	1234	CATCGCCCCTCTACTTCTTG
scaffold670094_17.9	1	p3	(TCA)5	15	3252	3266	CCAATCACAGTCGAAGACGA
scaffold670241_14.2	1	p3	(CGC)5	15	1172	1186	GCGGCTACAACCAAATTCAT
scaffold670441_17.5	1	p3	(ACC)6	18	329	346	CATGCCTCGAGTATGTGGTG
scaffold670833_13.1	1	p3	(TGA)5	15	48	62	CCCAAGACCCCAATTTGATT
scaffold671043_15.7	1	p3	(ATC)5	15	182	196	CAATCAAATTGGGGTCTTGG
scaffold671287_13.9	1	p3	(TTA)5	15	197	211	AGCAGGCTCAGACCAAGAAG
scaffold671359_13.4	1	p3	(ATT)5	15	80	94	TACTTCAACCCACGTGACCA
scaffold671402_16.9	1	p3	(CAT)7	21	2467	2487	TGCAGGTTCTGAAACAGGAA
scaffold671471_17.8	1	p3	(GTG)6	18	1531	1548	GGCCATTCGAGTTATGGAGA

scaffold671593_15.5	1	p3	(AAT)8	24	37	60	AATTGTTGCATCTATTTTCTAAATCA
scaffold671644_17.7	1	p3	(ACA)6	18	373	390	CAAGACCGTGACAGGCACTA
scaffold671731_15.2	1	p3	(AAT)5	15	1352	1366	ATTGTCCCTCCACACCACTT
scaffold671739_10.3	1	p3	(TGC)5	15	500	514	AATCGCATTTTCTCGACGAC
scaffold671883_18.3	1	p3	(GGC)5	15	302	316	AGCCAAGAGCCCATAGTTCA
scaffold671894_16.0	1	p3	(GAA)5	15	637	651	TTCCGATCAGAGCTTCTCGT
scaffold672194_14.5	1	p3	(ATT)6	18	759	776	AAAATTTGTCCTTTCTACACGCA
scaffold672264_20.1	1	p3	(TAT)5	15	856	870	AAAACCTGCCTCAACCCAG
scaffold672445_15.5	1	p3	(TGA)5	15	48	62	CCCAAGACCCCAATTTGATT
scaffold672486_14.6	1	p3	(ACA)5	15	8646	8660	TGCAGCAACAGTTGGAGTTC
scaffold672602_17.3	1	p3	(TCA)7	21	1837	1857	AACGCAACCTAATGGTTTGG
scaffold672961_16.1	1	p3	(TTC)5	15	986	1000	GGCAGAATTTGAATGGGAAA
scaffold673001_12.0	1	p3	(TGA)5	15	77	91	TGATCCCACTCTCCTTCTCG
scaffold673009_13.7	1	p3	(ACA)6	18	2182	2199	CATCACTTCTCCCAAAACCG
scaffold673018_15.2	1	p3	(TCC)5	15	3955	3969	TACTGCCTCCAGATCCACCT
scaffold673047_13.7	1	p3	(GGT)5	15	932	946	GGATCATTGTTATCGCGGTT
scaffold673250_12.5	1	p3	(TGC)5	15	602	616	TGAACTGTCTTTGGCACGAG
scaffold673283_16.5	1	p3	(ATT)6	18	1450	1467	TCAAAAACCAATTGATAGCACA
scaffold673352_19.3	1	p3	(CGG)5	15	700	714	TGAATTCTTTCCTCCATGCC
scaffold673481_15.4	1	p3	(GGT)5	15	425	439	TCAGCAAAAATGATCCCACA
scaffold673588_10.1	1	p3	(GGC)5	15	307	321	GTCGAATTTTGCTGTGTGGA
scaffold673610_10.8	1	p3	(AAG)6	18	66	83	GCAGGCCAAAGAGGAAGAGT
scaffold673614_16.6	1	p3	(GGA)6	18	224	241	GCTTTGGACTCGGAGTTTGA
scaffold673783_15.4	1	p3	(ATT)6	18	213	230	GACATTATCCATTTTATTGGGCA
scaffold673791_14.8	1	p3	(GAA)5	15	1198	1212	GAACTCGAGCATTGCAACAA
scaffold673827_11.8	1	p3	(ATT)8	24	1718	1741	CAAGAAAGCGAAATGCTTCA
scaffold673877_17.2	1	p3	(GAT)5	15	180	194	GGAAGCACTCGCAAGAAAAC
scaffold673910_17.1	1	p3	(TCA)6	18	178	195	TCACCCCCATACCCACATAC
scaffold673937_12.7	1	p3	(TTG)5	15	174	188	ATGTAGCCGACCCCAATTTA
scaffold673943_10.4	1	p3	(ATT)5	15	51	65	GGAGTGGGTGTCGGTTTTTA
scaffold673950_16.1	1	p3	(GCA)5	15	228	242	AAAAATACGAGTGGCGATGG
scaffold674092_14.0	1	p3	(ATT)7	21	175	195	TTTGGGTTTTAAATTCGTTGAA
scaffold674235_15.8	1	p3	(AAT)5	15	208	222	AGAACCAGACCTGCTTGCAT
scaffold674289_16.0	1	p3	(TTG)5	15	917	931	CGACCCACATAGTGGGAAAG
scaffold674441_16.3	1	p3	(TTA)6	18	52	69	TTCGACGGTTTCAAAGAAAAA
scaffold674521_10.8	1	p3	(TGA)6	18	408	425	TTTTGATTGGGAATAATTGGG
scaffold674534_17.1	1	p3	(GCT)7	21	908	928	TGTTCGTCCAGAGACAGCAC
scaffold674546_14.5	1	p3	(GAA)5	15	587	601	ATGTCTATCGTTTGGGCTGG
scaffold674630_16.2	1	p3	(TTC)7	21	1590	1610	AAATTTGAGACCGTGGATCG
scaffold674732_14.6	1	p3	(TGG)5	15	578	592	GGCTGACTCATGGAGAGGAG
scaffold674834_14.7	1	p3	(CAG)5	15	645	659	ACTGCCTCATCCAAACTTGC
scaffold674841_11.9	1	p3	(TAA)6	18	282	299	CCCCTATCCCCCACTGTTAG
scaffold674852_16.4	1	p3	(ACA)6	18	546	563	TGACATTGTTTCGGTTGCAT
scaffold674914_14.8	1	p3	(TCA)5	15	448	462	TCATGGTGGGGTGAGTTTTT
scaffold674941_13.2	1	p3	(TGT)5	15	476	490	GATTCTGGTTCGGTGATGCT
scaffold674951_18.9	1	p3	(AGC)5	15	228	242	GCTCGTACATCGAGTGATGC
scaffold675005_14.0	1	p3	(TAA)6	18	368	385	GTGGAGCTGTGGTGGGTTAG
scaffold675080_15.3	1	p3	(CAA)5	15	2057	2071	ATCCTATGTGCACCACGACA
scaffold675286_15.3	1	p3	(TTA)5	15	3192	3206	AATGAGAAAATGAGGCGCAC
scaffold675311_17.1	1	p3	(ACA)5	15	884	898	TTATGGCCAAAGACCAACCT
scaffold675378_15.0	1	p3	(CGG)5	15	655	669	CGGATTGTTTTTGGAAAGGA
scaffold675537_19.7	1	p3	(GAA)5	15	429	443	CAATGGAGGAGTGCAAGGTT
scaffold675836_14.5	1	p3	(CGC)5	15	180	194	ACCTAAATCCTACCCCCACC
scaffold675890_15.3	1	p3	(ACT)5	15	1064	1078	AAGGCCCTCTCAAAACCAAT
scaffold675935_14.6	1	p3	(GGT)7	21	2756	2776	GAAGATGTATCAGGGCGAGG
scaffold676349_14.8	1	p3	(CCG)5	15	39	53	GCATGAGGAAAAGGGATGAA
scaffold676398_17.5	1	p3	(TTG)6	18	482	499	ACCGCAGAATTTAGGAGGCT
scaffold676541_13.6	1	p3	(ATC)6	18	354	371	TCTTATTAGGGGCTTGTGCG

scaffold676817_14.8	1	p3	(GTG)6	18	3813	3830	TTCAGTGTGAGGGGTGAACA
scaffold676849_15.6	1	p3	(TTA)5	15	618	632	TTTTGTTGTGGCGTCTCTTG
scaffold676914_19.3	1	p3	(TTC)5	15	573	587	GGAGATAGAGAGGGGCCAAC
scaffold677018_15.3	1	p3	(AGA)5	15	1601	1615	GATTGGGGAGAGGATTGGAT
scaffold677109_10.1	1	p3	(CCT)5	15	791	805	TGTCGGAGCAGAATTGTACG
scaffold677321_11.0	1	p3	(TCT)5	15	348	362	AACGTATTCCTTCCCCCACT
scaffold677341_16.0	1	p3	(TCT)6	18	6041	6058	TTCCCCACCATTTCATCATT
scaffold677483_13.0	1	p3	(CGG)5	15	351	365	CCATCTCAAGGGTCTACCGA
scaffold677549_16.0	1	p3	(ATC)5	15	186	200	TTGCTCCCCTCTATCAAAGTTC
scaffold677567_13.8	1	p3	(CGG)5	15	834	848	GCCCTCGACTCCTTAGCTTT
scaffold677723_16.6	1	p3	(TGA)5	15	101	115	CCCCAAGACCCCAATTTTAT
scaffold677848_11.2	1	p3	(CCG)5	15	145	159	TACTCCATTACAGACCCCGC
scaffold677863_15.6	1	p3	(TAT)5	15	1606	1620	GGCACCCTCTCATGGATACT
scaffold678042_14.3	1	p3	(GCG)5	15	1342	1356	GTGTTTGTGATGGGCAACTG
scaffold678382_18.0	1	p3	(CTT)5	15	899	913	AAGTCGTCGTCATCATCCTCT
scaffold678446_16.4	1	p3	(AAT)6	18	46	63	GAGCATGCCTAGATGTTGCTT
scaffold678599_13.4	1	p3	(AAT)6	18	100	117	AGTCTCAATGGGGACACCAG
scaffold678729_17.4	1	p3	(TCA)6	18	2000	2017	ATCCTCCTCAGATCCTTGGC
scaffold678762_16.9	1	p3	(CAA)6	18	1035	1052	TGTAGGCCAAATGTGTTGGA
scaffold678946_15.9	1	p3	(TAT)6	18	408	425	CTCCAAATGCTCCTCTACCG
scaffold678957_17.0	1	p3	(GCG)5	15	1240	1254	GGCTCGCAAAAATAACCAAA
scaffold679033_14.5	1	p3	(GCT)5	15	234	248	GTAGTCGGAAAGTGGGGTGA
scaffold679249_16.0	1	p3	(TTA)5	15	773	787	GTGCAAAAGCAGGCAGAAGT
scaffold679316_12.3	1	p3	(ACA)6	18	1352	1369	CACTTACGAAATGTCAAGACGC
scaffold679404_15.2	1	p3	(ATA)5	15	1070	1084	TTAGGACTACCGGTCCGATT
scaffold679449_16.0	1	p3	(ACA)8	24	3071	3094	CCGAATGATGAGAAGTGGGT
scaffold679512_16.9	1	p3	(ATC)5	15	794	808	TGAGGGAACTTTGTGAACCC
scaffold679688_11.1	1	p3	(GAT)7	21	598	618	AGCTTAGGGCAACACGAGAA
scaffold679718_15.8	1	p3	(TCG)5	15	494	508	CAATTCTTTTGTGCGTGAGC
scaffold679793_15.4	1	p3	(TTG)5	15	397	411	CATTGATCGTACACGATGGC
scaffold679977_11.8	1	p3	(TAT)5	15	236	250	TGGACCCAATATGTCATAACCA
scaffold680021_12.4	1	p3	(TTG)5	15	235	249	GAAGGGGGAGACCGAAATTA
scaffold680090_18.0	1	p3	(CTA)5	15	443	457	TTGCGTCTGATTTGTTGAGC
scaffold680154_15.9	1	p3	(TAT)5	15	684	698	CATCATTTGATTGTGGTCCG
scaffold680165_14.7	1	p3	(ATT)5	15	452	466	TCGAAATTGACACGATCCAA
scaffold680230_12.7	1	p3	(GGA)6	18	317	334	TGGTTAAACTCCACCTTGGC
scaffold680274_10.3	1	p3	(GAC)5	15	792	806	TTGTATCGAACGAGCATCCA
scaffold680332_21.1	1	p3	(TAT)5	15	172	186	CAGAGAGGCGGTGGTAAAAA
scaffold680398_13.2	1	p3	(CAC)5	15	235	249	GACCCGACTCCATTTTGATG
scaffold680628_13.0	1	p3	(TGT)7	21	52	72	ATGTAGCCGACCCCAATTTA
scaffold680670_16.4	1	p3	(TTC)6	18	476	493	GCATACCTGGGAGTGCATTT
scaffold680764_16.8	1	p3	(TGA)5	15	3137	3151	TTTCCCTCTCAAAAACATCACA
scaffold680952_14.1	1	p3	(AAG)6	18	1326	1343	AGTCGTCTCCTATCCCATCG
scaffold681155_13.2	1	p3	(TTA)5	15	257	271	CCGGTCAAAACCAAATTACG
scaffold681172_16.5	1	p3	(TAA)5	15	4535	4549	CATGAATGTGTGGTTTCATCA
scaffold681190_15.3	1	p3	(TCT)6	18	504	521	TCGTAGCTCCCTTTGCATTT
scaffold681235_14.1	1	p3	(AAT)7	21	436	456	AGGCGTTTCATTGTTCTGCT
scaffold681338_13.6	1	p3	(AAT)6	18	558	575	ATTCGGCATCAACCAACTTC
scaffold681410_17.5	1	p3	(GGC)6	18	610	627	TGCATGAACGCTTCTCACTC
scaffold681456_17.2	1	p3	(GGC)5	15	237	251	AGGGGTAAAGTGCAAATCCC
scaffold681497_16.3	1	p3	(TTA)5	15	1896	1910	GCAACCATGATCTCATCGAA
scaffold681582_17.6	1	p3	(CAT)6	18	411	428	GTACCCTTGGCCAGAATCAA
scaffold681649_14.3	1	p3	(AAT)5	15	1583	1597	TTCAGTTGGATGGTTGGACA
scaffold681659_16.5	1	p3	(TGT)5	15	1125	1139	TTGGTTTGACGGGTGAAAGT
scaffold681748_13.1	1	p3	(TTG)7	21	42	62	ATGTAGCCGACCCCAATTTA
scaffold681783_14.6	1	p3	(CCT)5	15	143	157	GTCGCCTCACCTTCATCTTC
scaffold681896_16.4	1	p3	(AAT)5	15	396	410	CCATTACCGATGCCTTACTTG
scaffold682032_13.1	1	p3	(GAG)5	15	60	74	GACAGCGGTTGGTTCAGC

scaffold682128_14.5	1	p3	(AAT)6	18	228	245	CTCGTAGGTTTGGGTTTCCA
scaffold682169_17.2	1	p3	(TCA)6	18	621	638	GGTGAGCGATCAAAATCCAT
scaffold682196_17.8	1	p3	(ACA)7	21	875	895	CCGCCTGAAGTTCTTTTCTG
scaffold682371_17.8	1	p3	(GGC)7	21	680	700	AGAGGGGTGTTGAAGGTGTG
scaffold682474_15.8	1	p3	(TCA)5	15	4330	4344	TCCGGCGATTACTACAAACC
scaffold682577_17.5	1	p3	(TCA)6	18	600	617	TGGCTTCAGAAGAAACACCA
scaffold682681_16.9	1	p3	(TCA)5	15	2034	2048	GGATCAAATGTTTTGGGTGG
scaffold682707_14.7	1	p3	(TGT)6	18	46	63	TGTAGCCGACCCCAATTTAT
scaffold682813_14.2	1	p3	(CGG)5	15	866	880	TAGGGAGTGGAGCAGCAACT
scaffold682847_13.9	1	p3	(ACC)5	15	163	177	CTGCAGATGAGGGTCCTAGC
scaffold683020_17.3	1	p3	(CAC)6	18	1054	1071	GGTGTGCTGGAGATACCGAT
scaffold683164_12.1	1	p3	(ATA)5	15	218	232	ACTGCTCGCTATTTGGATGC
scaffold683206_19.2	1	p3	(TGG)6	18	1262	1279	GGAAATGGTGGTAACAACGG
scaffold683244_17.3	1	p3	(TCA)5	15	1020	1034	GTGCAGTTTCTCCTGCATCA
scaffold683403_16.8	1	p3	(TGT)7	21	41	61	TGTAGCCGACCCCAATTTAT
scaffold683406_15.2	1	p3	(GTA)5	15	673	687	GCAGACGATGCATTTTAGCA
scaffold683457_14.7	1	p3	(ATT)5	15	1962	1976	TTCTGTTGCTTGGTCATGGA
scaffold683590_15.4	1	p3	(ATT)5	15	513	527	TTGGCTCAAGTCTTTGCTGA
scaffold683664_15.5	1	p3	(AAT)6	18	575	592	TCTCCGTTGCTCCTACATCAT
scaffold684049_16.5	1	p3	(TGG)5	15	797	811	GGCATAGCCACGATTTCAAC
scaffold684240_14.0	1	p3	(GGC)5	15	159	173	GCGAGTTTCTTGAGGTCGTC
scaffold684335_17.1	1	p3	(TTG)5	15	1635	1649	CTGAAAGGTTGCATCAACGA
scaffold684340_13.5	1	p3	(TGT)6	18	43	60	ATGTAGCCGACCCCAATTTA
scaffold684364_18.1	1	p3	(TAT)5	15	479	493	CATTTTGTTACCCGAGCGAG
scaffold684382_16.3	1	p3	(ATA)6	18	288	305	AGATGGGATGAATCTGGCTG
scaffold684431_10.5	1	p3	(TAA)5	15	516	530	TCAAATACATAATTAGCACGGAGTG
scaffold684487_15.4	1	p3	(CAA)5	15	1510	1524	TCTGGATGCCTTGAAAAACC
scaffold684587_14.3	1	p3	(CTT)5	15	532	546	AGAGGAAGGAGGTGACCGTT
scaffold684640_14.2	1	p3	(TTG)5	15	783	797	TGGAGCATCCAACATGAGAG
scaffold684647_15.8	1	p3	(ATC)5	15	2390	2404	GATGCTTGGGGATGTCTGTT
scaffold684707_17.7	1	p3	(TCA)5	15	1073	1087	TGGGATTGTCATCCTCATCA
scaffold684774_14.4	1	p3	(TGA)7	21	50	70	CCCAAGACCCCAATTTGATT
scaffold684937_10.1	1	p3	(TAA)5	15	294	308	AAATGAAATCGTGCGAAAATG
scaffold684955_13.6	1	p3	(GGA)5	15	479	493	CGGTATCGGAGACGGTAGAA
scaffold684962_15.7	1	p3	(TGT)6	18	41	58	TGTAGCCGACCCCAATTTAT
scaffold685045_18.2	1	p3	(ATT)7	21	300	320	AGCGTAGAATCGCGAGAGAG
scaffold685072_15.5	1	p3	(ATA)5	15	642	656	AATCCCAAATTCATCCCTCC
scaffold685090_13.4	1	p3	(ATC)7	21	1256	1276	TGGCCAAAATTGCCTCTATC
scaffold685221_14.8	1	p3	(ATC)5	15	1183	1197	CAAAGCACCTGACAAGACCA
scaffold685365_16.1	1	p3	(GGC)5	15	638	652	GAGTTCTCCGGTGATCTTGG
scaffold685382_19.7	1	p3	(AGA)7	21	238	258	CCAAAGCTTGAAGAAATGTTTG
scaffold685440_11.6	1	p3	(GAT)8	24	54	77	GCAGACGAATTTAGTCGGGA
scaffold685448_15.4	1	p3	(TAT)5	15	1236	1250	AAGTGATGAAGGCTGCCACT
scaffold685554_16.9	1	p3	(TAA)6	18	604	621	ATCTCAAAAGACGCGAGGAA
scaffold685648_17.6	1	p3	(TGA)5	15	1490	1504	TCATTAGATTGGGAATAATTGGG
scaffold685675_17.9	1	p3	(GCT)5	15	547	561	ATGACGAAGGCTGAGATGCT
scaffold685691_14.9	1	p3	(CCG)5	15	476	490	AATATCCACCACCTCCTCCC
scaffold685756_15.6	1	p3	(GAA)6	18	2572	2589	ACTGGCAGCAACTCATTTCC
scaffold685903_19.2	1	p3	(GTT)5	15	89	103	TCCTTTTGCGTTTTATGTGC
scaffold685934_15.9	1	p3	(CAC)5	15	1036	1050	GGACTCGGGTGAGTCTCAAG
scaffold686097_13.4	1	p3	(TAA)6	18	227	244	ATAGGTCACGGGTCGAGTTG
scaffold686162_16.6	1	p3	(TTA)6	18	722	739	CAACTTTGGGATAAAATCTCCG
scaffold686166_13.9	1	p3	(ATT)5	15	183	197	TTTATCCCAAAATTGAGGCG
scaffold686201_17.4	1	p3	(CGT)5	15	347	361	AAAAACCGTTCCCACCCTT
scaffold686229_12.2	1	p3	(TCT)5	15	540	554	AGGCTTTCCAACACCAAGAA
scaffold686232_14.4	1	p3	(TTC)5	15	1141	1155	TCGGAGAACGGAAAATTGAG
scaffold686296_14.2	1	p3	(ATT)5	15	744	758	GTCCATGTCCTCGTCCTCAT
scaffold686345_16.3	1	p3	(ATC)5	15	1298	1312	CTTGGAAATGCCTGTTGGAT

scaffold686347_15.7	1	p3	(GCG)6	18	1799	1816	CCTCCCCATTTTCGATTTTT
scaffold686489_12.3	1	p3	(ATC)5	15	112	126	AGCAGGCTGAGAGAATGAGC
scaffold686608_14.7	1	p3	(CTC)6	18	1798	1815	TGCACAGAAGATCCTGCAAC
scaffold686657_12.2	1	p3	(ATT)6	18	1121	1138	TTTAAATCCAACTCACACATATACAG
scaffold686860_15.2	1	p3	(CGC)5	15	2905	2919	ACGGTCTCGAACTCGCTTTA
scaffold686864_17.0	1	p3	(AAT)6	18	237	254	CGAATAGTAGCTTCTGCGGG
scaffold686878_15.8	1	p3	(GCT)5	15	1598	1612	ATCCATGGGAATTGGACAAA
scaffold686924_22.4	1	p3	(AAC)5	15	777	791	GTAATCAGGGCCATTCCCTT
scaffold686941_19.7	1	p3	(CCG)5	15	291	305	TCATGCACCATCAAATCCTC
scaffold687037_15.9	1	p3	(TTA)5	15	2179	2193	TTGTATCCCCCAGTTTCGTC
scaffold687043_16.0	1	p3	(CGC)5	15	571	585	ATTTCAACTACGACGGCCAC
scaffold687104_12.7	1	p3	(AAT)7	21	43	63	CACTCGAAGAGTAGCGGCTG
scaffold687132_14.9	1	p3	(ATC)5	15	246	260	TGTTTTTACCTGCGTATCTGTCA
scaffold687164_16.0	1	p3	(ATT)5	15	1524	1538	AGTTGACGGTTGGAAGATGG
scaffold687182_15.7	1	p3	(TTC)5	15	276	290	GTTTACCTGACTGGCGTGGT
scaffold687293_13.2	1	p3	(ACA)5	15	576	590	CCCTGGAACAACACCAGTTT
scaffold687325_15.6	1	p3	(CAC)6	18	361	378	GTGCTTCGGTCTGGATGACT
scaffold687505_18.7	1	p3	(TCC)5	15	395	409	ACAGAAAGGTCGGCATCATC
scaffold687607_14.4	1	p3	(TAA)7	21	58	78	ATCTCCACTTGTTGTCTCCAA
scaffold687632_15.7	1	p3	(ATT)6	18	295	312	ATTCCTCGGGTTCTTGGAGT
scaffold687709_13.0	1	p3	(CGC)5	15	335	349	TGATTTAGTAAAGTTGGAGGTGGA
scaffold687953_14.9	1	p3	(AAT)5	15	498	512	GACCCTTTGTGGGATTTGAA
scaffold688085_14.6	1	p3	(ATC)5	15	250	264	TTATGGTGCGTCGAGTCTTG
scaffold688211_15.1	1	p3	(TTA)6	18	167	184	TCAAATGTTCGTCTTGCAGC
scaffold688282_13.4	1	p3	(ACA)6	18	1204	1221	AATTGAATTCAGAGCACCCG
scaffold688443_18.1	1	p3	(TGA)5	15	72	86	CCGACTACGACTATCCTCGC
scaffold688514_15.4	1	p3	(ATG)5	15	66	80	AATCGTCGAATTCGGTTTCA
scaffold688556_10.3	1	p3	(AAC)7	21	689	709	AACATACCATCGTGGCCAAT
scaffold688632_16.7	1	p3	(TTG)5	15	49	63	TGTAGCCGACCCCAATTTAT
scaffold688801_15.0	1	p3	(TGG)6	18	661	678	GATTTCATTTCAGCGCTTCC
scaffold688909_16.6	1	p3	(TTG)9	27	39	65	ATGTAGCCGACCCCAATTTA
scaffold688975_12.3	1	p3	(AAT)7	21	246	266	TCAGAATATGCAAGGGGGAG
scaffold689012_16.9	1	p3	(TTA)5	15	790	804	ACGGATTCGAGTTTCAACGA
scaffold689169_12.4	1	p3	(AAT)6	18	2042	2059	AGAGGGAGCATGTGACCTTG
scaffold689187_15.2	1	p3	(CAT)6	18	554	571	TAGTCATCGAGCAAGCCAAA
scaffold689294_13.1	1	p3	(CGC)5	15	140	154	TGACCAATCACCAATCAACAA
scaffold689352_12.0	1	p3	(GCG)6	18	156	173	AGTGCAAATGTTGACGGGTT
scaffold689414_11.2	1	p3	(CAA)5	15	509	523	ACGTCCTTGCCAAAGTTCAC
scaffold689422_14.6	1	p3	(TGA)6	18	53	70	CCCAAGACCCCAATTTGATT
scaffold689592_13.7	1	p3	(ACA)5	15	52	66	CACGCCAACTTACAAACTATCG
scaffold689790_18.0	1	p3	(ATG)6	18	2076	2093	GTCTCAGGTTCGAATCCCAC
scaffold689962_16.5	1	p3	(TTC)5	15	1905	1919	CCTTGAGCTCGTCAAAGTCC
scaffold690167_20.2	1	p3	(GGA)5	15	378	392	ATTGTTGAGAGCCGAAATGG
scaffold690274_14.2	1	p3	(TGT)6	18	46	63	TTGGTTCATGTAGCCGACC
scaffold690336_17.1	1	p3	(GAT)5	15	2331	2345	GAACAAGGTGAAGGCTCCTG
scaffold690376_15.2	1	p3	(ACA)5	15	889	903	GTTGCGAAATATGCCTCCAT
scaffold690492_16.0	1	p3	(CTT)5	15	621	635	TCTGTTTTATTTCCCCGTGC
scaffold690559_17.6	1	p3	(AAG)5	15	714	728	GCTGAACAATGCCTCGAAAC
scaffold690654_13.9	1	p3	(ATT)5	15	481	495	TGCATTTCATCGCTTTATGC
scaffold690801_10.2	1	p3	(TCA)5	15	294	308	GCCGTCTGATTGGAGGATTA
scaffold690813_11.3	1	p3	(ATT)6	18	405	422	TGAAAATGAGTTGGGGGTGT
scaffold690951_15.2	1	p3	(ATA)5	15	564	578	TGAGCATGATGTGTCTTGAATTA
scaffold690959_10.6	1	p3	(TCA)5	15	294	308	GCCGTCTGATTGGAGGATTA
scaffold690980_12.1	1	p3	(AAT)6	18	1180	1197	AAATATTTTAGAAAGAACAGATTTTC
scaffold691071_14.2	1	p3	(CGG)5	15	112	126	AAGAGGGGGAGGAAAATTGA
scaffold691138_14.1	1	p3	(TGT)6	18	69	86	CCGACCTTCCAAATTGTTGT
scaffold691161_13.8	1	p3	(ATA)5	15	258	272	ATGGGTAGCCAGTTTCACCA
scaffold691204_17.1	1	p3	(AAG)5	15	49	63	CAGTTCCATGTCACAAACGC

scaffold691215_13.8	1	p3	(ATC)7	21	208	228	ACCAATCAAACCCAACGAAA
scaffold691235_15.6	1	p3	(ATT)5	15	222	236	TCATTTCCGACAACTCGATG
scaffold691417_11.3	1	p3	(CCG)5	15	174	188	CATTCCCTGCAATCCTCATC
scaffold691471_15.1	1	p3	(AAT)5	15	227	241	CCATTGTAAACCCCTGCACT
scaffold691557_13.3	1	p3	(ACA)5	15	427	441	CCAGAACCATGGACACACAG
scaffold691616_15.6	1	p3	(TCT)5	15	1023	1037	ACTTATTGGCACGAAGTCGG
scaffold691653_14.9	1	p3	(TCA)5	15	456	470	CTGCTAGACCCAGTGGTGGT
scaffold691756_14.6	1	p3	(CCT)5	15	768	782	ATGTTTCTTCCGAGTGTGGG
scaffold691829_16.5	1	p3	(ACG)5	15	350	364	GATGAGCAGCTCGACAATGA
scaffold691890_15.8	1	p3	(TCC)5	15	55	69	GGCCACCAAGAATCTCAGAA
scaffold691961_14.0	1	p3	(TTA)5	15	556	570	GGGTTGTTAAGATTTAATGATTATCC
scaffold692060_13.7	1	p3	(ACA)6	18	1038	1055	CCTGGCCTTCAATTCATGTT
scaffold692111_17.5	1	p3	(ATA)5	15	195	209	TCAAAGAAAGTGAGTAGAATCCAAA
scaffold692174_13.6	1	p3	(TTG)7	21	422	442	TTTGGTTCATGTAGCCGACTC
scaffold692177_17.0	1	p3	(ATC)5	15	54	68	TGTGTTTCATGTTTGTTGTGATAA
scaffold692229_15.1	1	p3	(TAT)7	21	67	87	TGAAGTTTCGAAGACTGGAAGA
scaffold692292_18.4	1	p3	(ATG)6	18	457	474	CCTCGATCCTGACCCAATTA
scaffold692325_14.2	1	p3	(TTA)8	24	734	757	TGAATATTGGGGTGGGAAAA
scaffold692402_18.2	1	p3	(ACA)5	15	166	180	AATCACGGGACCAAAATGAA
scaffold692833_16.3	1	p3	(GCC)6	18	2385	2402	ACTTTTGTGACGGTTTTGCC
scaffold692860_13.6	1	p3	(ATT)7	21	75	95	TGCACATGTTAGCCAGAACTTT
scaffold693109_11.7	1	p3	(GTC)5	15	212	226	TCGATTGTATTTGTGCTGCAA
scaffold693228_17.2	1	p3	(GCC)5	15	590	604	TCATTCTCTTCACCGGAAGC
scaffold693259_18.6	1	p3	(TGG)7	21	210	230	AAGGTAACGGAAGGGTCTGG
scaffold693500_15.5	1	p3	(TGT)7	21	38	58	TGTAGCCGACCCCAATTTAT
scaffold693522_13.0	1	p3	(ATT)5	15	530	544	TATTGTCCTCCATTTCCCCA
scaffold693562_15.9	1	p3	(ATC)5	15	409	423	ATTTCCTGTTCTCTTCCCCG
scaffold693671_13.0	1	p3	(TGA)6	18	44	61	CCCAAGACCCCAATTTGATT
scaffold693981_15.4	1	p3	(GAA)5	15	559	573	TCATGGATTCATGGTGCAGT
scaffold693999_12.9	1	p3	(TTC)5	15	1517	1531	CCCGTTCAGACCACCTTTTA
scaffold694081_14.6	1	p3	(CAC)5	15	148	162	TCAACCTGAGTCTCCATCCC
scaffold694082_12.8	1	p3	(TGA)8	24	44	67	CCCAAGACCCCAATTTGATT
scaffold694368_11.9	1	p3	(TAT)7	21	718	738	TGGGATGAGGAAAAACATGA
scaffold694444_14.3	1	p3	(CCG)5	15	617	631	TCGACGCATCAGAAGACAAC
scaffold694475_18.0	1	p3	(TGT)6	18	957	974	GAGCTTTTGGAGAGTGCTGC
scaffold694520_12.4	1	p3	(TTG)5	15	1311	1325	GAAGGGGATCACCAAACTGA
scaffold694583_17.1	1	p3	(TGA)5	15	1345	1359	TTCTATGAAGCCCTCAGCGT
scaffold694651_17.1	1	p3	(TCC)5	15	800	814	TGCAGAAGGTCTCCTCCAGT
scaffold694740_10.0	1	p3	(CTT)5	15	408	422	ATGGTTGCCTTTGTTTGGAG
scaffold694869_13.0	1	p3	(AAT)5	15	932	946	CAACATTGCAAAATGGGACA
scaffold695150_17.6	1	p3	(TGT)7	21	33	53	CCAATTTATTGGGATTAAGGC
scaffold695217_12.8	1	p3	(TTA)6	18	997	1014	TGTTTACACGTGTGGGATATGG
scaffold695307_13.5	1	p3	(TCC)5	15	266	280	TATCTGCAGCGCATCATCTT
scaffold695378_15.3	1	p3	(GAG)6	18	262	279	GAGTCAAAGCCATCCACGAT
scaffold695394_16.4	1	p3	(TTG)6	18	298	315	ATGGATGCACCGGTTAGAAG
scaffold695593_14.2	1	p3	(TCC)6	18	3738	3755	GGCAATTGACCACCGTAAAC
scaffold695753_17.9	1	p3	(AAT)5	15	335	349	TCGGGATTCAGTGATTAAAGC
scaffold696024_13.8	1	p3	(ATG)5	15	1011	1025	CGTTTAGTGGTGCGAGGAAT
scaffold696088_17.4	1	p3	(TCA)6	18	259	276	ACCAGTAGTTTCCGGTGGTG
scaffold696156_16.6	1	p3	(AAT)5	15	454	468	TGAGGCGTAAGCAAATTTCA
scaffold696236_13.9	1	p3	(TTA)5	15	159	173	TGTTGCACTATTTGCTTGGG
scaffold696391_12.0	1	p3	(TAT)6	18	475	492	GACTATGGCCCAAAACCGTA
scaffold696396_16.6	1	p3	(AAT)7	21	378	398	GAGGACATCGGTCTGAGAGG
scaffold696426_17.0	1	p3	(TTG)5	15	151	165	TGATCCATATAGCCGACCCT
scaffold696529_13.9	1	p3	(TTG)5	15	569	583	GGTTCATGTAGCCGACCCTA
scaffold696548_15.1	1	p3	(CAC)5	15	500	514	CCCCAACCAAAATACCCTTT
scaffold696565_13.3	1	p3	(ACA)8	24	1435	1458	CAAATCAAAATAATTGCAAAGTGA
scaffold696653_15.7	1	p3	(TAT)6	18	226	243	TGCGAGCTAGCGTCATAAAA

scaffold696666_13.3	1	p3	(ATT)6	18	555	572	TGCTTGATTAGCATTTTATGGG
scaffold696702_16.(	1	p3	(TTG)5	15	532	546	TGAGGAAGCCGTTGAAGATT
scaffold696774_14.!	1	p3	(TGA)5	15	1010	1024	CCGAAAAATTGGGAAATCCT
scaffold696783_11.9	1	p3	(TCC)5	15	490	504	CTTTGCCTTTTCTGCCTCTG
scaffold696877_16.7	1	p3	(TTG)6	18	2190	2207	ATACGATCCATTTTGCCGAC
scaffold696944_15.2	1	p3	(GAG)5	15	517	531	GGAGGACGAGGAGCATCATA
scaffold696952_15.7	1	p3	(TGC)5	15	1946	1960	CTTGCTGTGTCGATCCCTTT
scaffold697048_18.6	1	p3	(AAC)5	15	271	285	GGCCGTGTACTACCGTGTTT
scaffold697212_16.2	1	p3	(CAA)5	15	11072	11086	ATGGTCTCGCACAACACAGT
scaffold697382_11.8	1	p3	(CGG)5	15	387	401	AGCAAATAAATCGGCAGAGG
scaffold697556_12.3	1	p3	(GGA)5	15	538	552	GTCGAAGAACCTCGACAACC
scaffold697655_12.7	1	p3	(TGA)6	18	96	113	TGCTATTTTCATCTACCCACCA
scaffold697775_11.6	1	p3	(TAT)6	18	263	280	AAACGCCTAGCTCTCCACAA
scaffold697867_14.!	1	p3	(AAT)5	15	676	690	GAGAATTGGGTAACCAGCCA
scaffold697883_17.1	1	p3	(TCA)5	15	5875	5889	GGGGGAGAGGTTAGATTGGA
scaffold697891_15.1	1	p3	(TAT)6	18	272	289	AGTGATTGATGGGCTAGGCA
scaffold698024_16.4	1	p3	(ATA)7	21	719	739	TATTTTATCTCCTCCGCCCG
scaffold698055_15.7	1	p3	(AAT)5	15	1197	1211	TGAAGGAAAGGGATGGGTAG
scaffold698313_17.(	1	p3	(GCG)5	15	522	536	TGCAAGTTTGAGCGAGATTG
scaffold698454_17.4	1	p3	(ATT)6	18	247	264	ATCTCGTGGGGGAAGACTTT
scaffold698486_12.4	1	p3	(AAT)5	15	970	984	GCATTTCCACGTTCAGAGGT
scaffold698492_16.9	1	p3	(TTC)5	15	62	76	TTCAACACTATTCAACACGACTGA
scaffold698641_14.3	1	p3	(CAT)6	18	2477	2494	GCTGCATTGGGGAAAGTCTA
scaffold698716_16.6	1	p3	(CCG)5	15	2435	2449	TCTCACTCAGCTCGAAAGCA
scaffold698771_18.3	1	p3	(CAT)5	15	779	793	CGGCAGTTAAGGCTCAAAAG
scaffold698813_14.9	1	p3	(AAT)5	15	628	642	TGAAGGCCTTAAATCACCAAA
scaffold698854_13.1	1	p3	(GTT)5	15	1067	1081	ATCCATATACCGACCCCACA
scaffold698894_14.(	1	p3	(TTC)5	15	854	868	TGTGCAATCCCCTAAGGTTC
scaffold698993_18.6	1	p3	(CAT)6	18	230	247	AGCTTTTTCCTCTCCTTCCG
scaffold699014_16.2	1	p3	(CAA)5	15	2271	2285	CCTTCCATTTGAGGATTGACA
scaffold699040_16.6	1	p3	(TTA)5	15	73	87	CTGGGTAAACCCACGGACT
scaffold699182_14.1	1	p3	(TTG)5	15	324	338	GACAGAGGCCTTGCAAGATT
scaffold699332_18.(	1	p3	(TGA)5	15	2101	2115	ATTATCTCGCAGCATTGCCT
scaffold699400_14.(	1	p3	(TTA)6	18	56	73	TCAGGTGTGATCAATGACAATTT
scaffold699585_14.4	1	p3	(TTG)7	21	4487	4507	TTTCTGCAACATCTGAACGC
scaffold699642_16.1	1	p3	(TAT)5	15	563	577	TGTACACAGTGAGGTCATACTTAGG
scaffold699764_12.8	1	p3	(GAA)5	15	315	329	TGGTGACATCCACTACCTGC
scaffold700045_19.2	1	p3	(CCG)5	15	533	547	GATAATTCCCAGCCCCACTT
scaffold700160_14.9	1	p3	(TAA)5	15	832	846	GGCAATGCTATTGGGACAAG
scaffold700222_16.!	1	p3	(AAT)5	15	1147	1161	TTAAGATGAGATTTGTCCTTCAAA
scaffold700235_14.8	1	p3	(CCA)5	15	776	790	CAAATTCCTCAACCACCACC
scaffold700439_16.!	1	p3	(GCA)6	18	522	539	ACAAGCATCATCCTCCCAAC
scaffold700478_14.6	1	p3	(CTT)5	15	1816	1830	GTCTCCAGAAGACGATTCGC
scaffold700495_15.8	1	p3	(TGG)6	18	109	126	GCCATATCACGGTGGAAAAG
scaffold700655_17.3	1	p3	(TGT)5	15	45	59	ATGTAGCCGACCCCAATTTA
scaffold700700_15.3	1	p3	(CTC)5	15	1350	1364	AGCTATAGGTGGGGATGGGT
scaffold700764_20.(	1	p3	(TAA)5	15	99	113	TTTACCCGATCTTGCTTTGC
scaffold700869_18.6	1	p3	(GGC)6	18	390	407	CACCGGAACCAAAACCTAGA
scaffold701073_13.1	1	p3	(TTA)5	15	67	81	TGGTTCTCCAAATTCTTCCTC
scaffold701194_19.7	1	p3	(ATC)5	15	166	180	AGGAATGGACGCAAGATCAC
scaffold701268_10.(	1	p3	(ATG)5	15	439	453	GTACATCTCCCAAGACCCCA
scaffold701317_13.7	1	p3	(TGT)5	15	49	63	GGTTCATGTAGCCGACCCTA
scaffold701334_16.1	1	p3	(AGA)6	18	2139	2156	GAAAATGCTCAGGGGAGTCA
scaffold701366_17.3	1	p3	(TTG)5	15	744	758	CAATTGCTAGGGCGGACTAC
scaffold701562_15.7	1	p3	(CAA)5	15	999	1013	CCGTTTTGTTACCATTTTGC
scaffold701619_16.2	1	p3	(CTT)5	15	2076	2090	AGGATACATGACACCCGTCC
scaffold701774_18.8	1	p3	(TTC)5	15	378	392	ATCAAACCAACCCCAACAAA
scaffold701831_16.7	1	p3	(TGA)5	15	3026	3040	TCCCAAGTCTGAGCTTTTCTG

scaffold702004_18.4	1	p3	(TAT)5	15	29	43	CATCAAACCCAAACCAACCT
scaffold702086_17.4	1	p3	(TCA)5	15	183	197	GCGAAAATTTAACTAGGCCG
scaffold702123_17.8	1	p3	(TGT)6	18	67	84	TTGGTTCATGTAGCCGACCT
scaffold702169_15.0	1	p3	(ACA)5	15	161	175	AATTGAGAGGTGGGACGATG
scaffold702205_15.9	1	p3	(GCA)5	15	1686	1700	CTTCAACTTGGCGCACTGTA
scaffold702294_15.6	1	p3	(CCA)6	18	1269	1286	CAATTTCTTTTTCCCCCTCC
scaffold702386_16.4	1	p3	(ATT)5	15	274	288	TTGGTGCGAGAATGATTTGA
scaffold702446_16.7	1	p3	(TCT)5	15	52	66	TCGTCACTTTCCTCTTCCTCA
scaffold702478_15.1	1	p3	(TTA)5	15	340	354	CACGATAAAACAATTTTGGAATGA
scaffold702510_17.2	1	p3	(TTG)5	15	1106	1120	CAAGATAGGGGGACAGCGTA
scaffold702525_10.9	1	p3	(TTC)6	18	1148	1165	ACAAGCCTCCAATTCACACC
scaffold702639_20.5	1	p3	(TTC)5	15	972	986	AGATATGGAAGATGCGGTGG
scaffold702734_12.8	1	p3	(TGT)7	21	45	65	ATGTAGCCGACCCCAATTTA
scaffold702758_14.1	1	p3	(GCG)6	18	250	267	TGAACCCAAATCTCTCCTCG
scaffold702789_13.3	1	p3	(ATT)7	21	185	205	CAATCCGCCAATCCTCTTTA
scaffold702801_10.6	1	p3	(TAT)5	15	773	787	TGCCTGAGGTCTTACCCTTG
scaffold702810_14.4	1	p3	(AAT)5	15	780	794	CGTAATTTTCAACCCCAACC
scaffold702818_15.1	1	p3	(GCA)5	15	2352	2366	ACAATCGATGAAGAAAGCGG
scaffold702917_16.7	1	p3	(CCG)5	15	1543	1557	AGAAAACCTTCCCCATTGCT
scaffold703016_16.4	1	p3	(AGC)5	15	2043	2057	ACCATGCGATGAACACGTTA
scaffold703049_17.6	1	p3	(ACC)5	15	985	999	CTCTTCGCCTTCAACCTCAC
scaffold703059_16.2	1	p3	(TTC)7	21	141	161	AAAAATTGGTGTGGATGTCTCT
scaffold703078_14.0	1	p3	(TGT)5	15	51	65	TGTAGCCGACCCCAATTTAT
scaffold703150_17.1	1	p3	(AAG)5	15	83	97	GTCATGCACCTCTTGAAGCA
scaffold703248_16.1	1	p3	(CTT)5	15	2318	2332	GGCGTTCAATCCTATGGAGA
scaffold703250_17.6	1	p3	(TAT)5	15	928	942	CCAATCAAATTGGGGTCAATA
scaffold703287_18.0	1	p3	(GCC)5	15	2277	2291	ATGAATATCCTTCGTCGCCA
scaffold703354_13.2	1	p3	(TTA)5	15	518	532	ACTTGGGTTGTGCCAAAGTC
scaffold703385_15.9	1	p3	(CCG)5	15	118	132	CCAGGTCGCTCAAATCTCTC
scaffold703429_14.1	1	p3	(AGC)6	18	2868	2885	ACTTCCTCCAGCAAGAGCAG
scaffold703438_16.8	1	p3	(ACA)6	18	2613	2630	CGAAAATAAGCCAACCGAAA
scaffold703693_17.1	1	p3	(GGA)5	15	1522	1536	CTGTTGCAGGGAAGGCTAAG
scaffold703871_14.2	1	p3	(AAT)5	15	56	70	GTCTCAATGGGGACATCTGG
scaffold703900_18.0	1	p3	(AAT)6	18	474	491	CCCATCCTTGTGATATGCAAC
scaffold703916_16.1	1	p3	(GCT)5	15	265	279	TGTCACCACTCCCAAACTCA
scaffold704036_11.2	1	p3	(GAT)5	15	1150	1164	TGCCCTTCCCGATTATAGTG
scaffold704150_13.0	1	p3	(TTA)5	15	588	602	AAGGGAATTAAGCCTGCCTC
scaffold704249_13.9	1	p3	(AAT)6	18	152	169	GCGATGGATTTTTCCTTTCA
scaffold704297_14.6	1	p3	(ATA)5	15	111	125	AGGAGCTAGCACACCATCCC
scaffold704328_18.8	1	p3	(TGA)6	18	3360	3377	AGGTGAAGCCCAGTCTGCTA
scaffold704366_13.7	1	p3	(AAT)7	21	568	588	CCACCATTCTTTTGACAGCA
scaffold704375_13.7	1	p3	(GGC)7	21	45	65	GTGAAGATTAGCTCCGCCTG
scaffold704448_15.0	1	p3	(ATA)5	15	296	310	ATTCCACTTCATTGCACACG
scaffold704592_15.9	1	p3	(AAG)5	15	176	190	CTCTTCGAGCGAGTTCAAGC
scaffold704597_12.9	1	p3	(AAT)7	21	169	189	TGGATGAGTATTGCCTGACG
scaffold704865_13.0	1	p3	(TAA)7	21	742	762	ATGCTTTGTGTTTTCCTGCC
scaffold704896_14.2	1	p3	(TTG)5	15	66	80	TGATTTATGTAGCCGACCCC
scaffold704983_14.6	1	p3	(CCA)7	21	879	899	GCAACTGAAAAACGGTTGGT
scaffold705000_16.1	1	p3	(TCC)5	15	1339	1353	CTGCACTGTATCGGGAACCT
scaffold705082_13.6	1	p3	(TCA)7	21	221	241	ACAGTAAAGCCCGGTTGATG
scaffold705129_17.0	1	p3	(AAT)5	15	499	513	TGAGCACCAAATTTATTATCGAA
scaffold705181_16.4	1	p3	(GGC)5	15	1090	1104	GAGCAGAAAGGAGGGGAAAC
scaffold705233_15.4	1	p3	(AAT)6	18	1010	1027	GCTCGGTATGCCATTTGATT
scaffold705522_16.3	1	p3	(GAT)5	15	147	161	GACATGGTTGTGGTTTGTGC
scaffold705537_18.2	1	p3	(ATC)5	15	754	768	AATTGCTTGTGTAGGGGTGG
scaffold705642_14.4	1	p3	(AAT)5	15	724	738	GGCTTGATCTTTGAGATGGG
scaffold705765_10.0	1	p3	(TCA)5	15	959	973	GGGCGGATTAGAAGAAGAGG
scaffold705778_10.5	1	p3	(AGT)5	15	86	100	GGAACCAACACAAACACACG

scaffold705836_14.(	1	p3	(CCA)6	18	299	316	TCTTGGCGCCTCACTTTAAT
scaffold705910_10.:	1	p3	(TAT)9	27	354	380	TCCTACCCAACCAACTAGCG
scaffold705936_14.!	1	p3	(ATC)5	15	303	317	CCACAGCTTCCCAAACAAAT
scaffold706010_11.:	1	p3	(CAT)5	15	404	418	TGCAGAACCCATGTACATCAA
scaffold706049_15.2	1	p3	(TTG)7	21	861	881	TCCATTTCGCACAAAAGACA
scaffold706197_10.!	1	p3	(TAT)6	18	300	317	CATACAGTCGGCATCCAATG
scaffold706293_16.8	1	p3	(CAA)5	15	255	269	GACGCCATTTACGTCTTCGT
scaffold706297_17.(	1	p3	(TAT)5	15	801	815	CAATTTCGGGTGGTAAGAGC
scaffold706370_13.8	1	p3	(GAG)5	15	837	851	TTTGTTCATTCTCCCGAGTTG
scaffold706479_12.8	1	p3	(TTC)5	15	837	851	TTGTCCCGTCAAGAAAAAGG
scaffold706480_15.4	1	p3	(TTA)5	15	242	256	TGGATTTCGTCACCATCTCTC
scaffold706508_18.7	1	p3	(ATC)6	18	572	589	AAACGCACTACACGAATCCC
scaffold706516_16.4	1	p3	(GCG)6	18	175	192	CGTGATTTCTTTTTGGGCTC
scaffold706552_16.(	1	p3	(CAG)5	15	1833	1847	GGTCTTTCTGCATCTCCAGC
scaffold706588_18.:	1	p3	(CGG)7	21	2057	2077	TAGAATCGGATTTATCGCCG
scaffold706611_11.(	1	p3	(GCT)5	15	626	640	ACTTCCAGCATACCTCCCCT
scaffold706617_13.2	1	p3	(TGA)7	21	43	63	CCCAAGACCCCAATTTGATT
scaffold706646_17.(	1	p3	(TGA)6	18	43	60	CCCAAGACCCCAATTTGATT
scaffold706649_14.(	1	p3	(CGC)5	15	1729	1743	CCCCCACCCTACAAGTATGA
scaffold706718_13.(	1	p3	(TTC)5	15	283	297	AAAACCGAGAGCAGAGTCCA
scaffold706748_17.!	1	p3	(GGC)5	15	60	74	CTTGTTGCTCAGCACAGTGG
scaffold706817_15.(	1	p3	(AAT)7	21	1258	1278	GGGACCCACATAACCATGTC
scaffold706915_14.(	1	p3	(CAA)5	15	1397	1411	CAAGCCAACTTTGAGGAAACA
scaffold707025_17.(	1	p3	(TTC)5	15	497	511	CCTTGCTAGGGCAGTACGAC
scaffold707062_16.:	1	p3	(ATG)7	21	1094	1114	CTGGCATTGGAGGAGGACTA
scaffold707085_16.!	1	p3	(TTG)5	15	498	512	GTCACCAAATACAAAGCCGC
scaffold707088_14.:	1	p3	(GGC)5	15	307	321	ATCTTCAGCAAACCATTCCG
scaffold707089_12.(	1	p3	(GCG)5	15	483	497	TAAGAATTGAATCGCAGGGC
scaffold707094_13.2	1	p3	(TGG)5	15	88	102	CTAGGTGTATGGCGGAGGAG
scaffold707103_12.!	1	p3	(GAG)5	15	1324	1338	CTTCTTCCCCTCCCAAACTT
scaffold707149_15.7	1	p3	(TCC)5	15	498	512	ACCTCTAGCCCGGTGAACTT
scaffold707279_15.8	1	p3	(TGA)6	18	46	63	CCCAAGACCCCAATTTGATT
scaffold707348_15.7	1	p3	(TTA)7	21	499	519	TGATTTCCAAACATACGTTGTCA
scaffold707358_16.:	1	p3	(TGA)5	15	49	63	CCCAAGACCCCAATTTGATT
scaffold707558_14.!	1	p3	(ACA)5	15	462	476	AGCTCTCGACATCAAATCGG
scaffold707664_16.7	1	p3	(ATG)5	15	254	268	TCCGGAGAAAGCTGATGTTC
scaffold707697_17.:	1	p3	(GAG)6	18	1246	1263	CCGGTTATGAGGTCGGAGTA
scaffold707808_14.8	1	p3	(ATC)5	15	82	96	AAGCCGGATAAGCGATTTCT
scaffold707965_14.4	1	p3	(TAC)5	15	112	126	ACCCAACTTCCAAGGTTCAA
scaffold707969_11.4	1	p3	(TGG)5	15	530	544	GGGTACTCGTAGTCAACGCC
scaffold707991_11.4	1	p3	(ATT)5	15	361	375	GGGATGCATCAAAGTTGTCA
scaffold708142_12.(	1	p3	(TTA)7	21	693	713	TCCTTAGTCAAGGGGAAATGAA
scaffold708309_15.9	1	p3	(TCA)5	15	269	283	ATGTGGCCATTCTGGAAAAC
scaffold708336_14.!	1	p3	(AAT)5	15	586	600	ACTTTGAACCCATCTCACCG
scaffold708437_15.2	1	p3	(CCA)5	15	499	513	CTGGTTCGCAACACAGAAAA
scaffold708519_14.8	1	p3	(ACA)5	15	620	634	GGATCCTAAAATTTGAAGGGG
scaffold708563_12.7	1	p3	(AAT)6	18	664	681	CCCTAGCAAAAGAGAGAGGGA
scaffold708577_13.:	1	p3	(TCA)6	18	146	163	CCACCCTTACTCCCCAATTT
scaffold708702_16.(	1	p3	(AAC)8	24	3720	3743	GCTATTGAGCAATATCAGAAACCA
scaffold708760_12.8	1	p3	(ATT)5	15	1563	1577	GCAACGAACCCCAAGTCTAA
scaffold708868_13.(	1	p3	(TTC)6	18	947	964	AAGATTGCTCTCGCCTCAAC
scaffold708936_12.:	1	p3	(TGT)6	18	42	59	TGTAGCCGACCCCAATTTAT
scaffold709004_15.!	1	p3	(TTA)5	15	88	102	TGATTCTAAAATGAATCCAATCCA
scaffold709020_10.(	1	p3	(GCC)5	15	215	229	GCCGACATGACCTTAGCAGT
scaffold709026_18.:	1	p3	(CAG)5	15	316	330	TGCACATTTCAACAGCAACA
scaffold709092_15.:	1	p3	(AGC)5	15	593	607	ACAAAATGGGCATGAACACA
scaffold709109_16.(	1	p3	(ATT)8	24	698	721	TCGTTAATTGGTTCCATTTTGA
scaffold709187_15.:	1	p3	(AAT)5	15	700	714	CCATGTCTTGAGAGTGGGGT

scaffold709254_12.9	1	p3	(GCG)6	18	201	218	CGGTGGAGACTTAGGTGGG
scaffold709313_14.7	1	p3	(AAT)5	15	262	276	TGCAGCTTGCTGCTGTTACT
scaffold709427_18.8	1	p3	(CAA)5	15	913	927	TCATGCTTCTTGTTGGGAAA
scaffold709440_10.8	1	p3	(AGA)5	15	369	383	GAAATCCGAAACGCCATTAT
scaffold709455_16.4	1	p3	(TAA)6	18	315	332	TTTGTCAAAGAACACGTTCACAT
scaffold709458_15.3	1	p3	(GTG)5	15	3069	3083	GTGGGATTTTACGGAGTGGA
scaffold709505_15.6	1	p3	(AAC)6	18	555	572	CCTTATACGTTCACCCCAAGTT
scaffold709523_16.3	1	p3	(AAT)5	15	563	577	CAACCGAAATTTTTGGAAGG
scaffold709585_17.3	1	p3	(TCA)6	18	796	813	TGATCATATTCGGAGCCACA
scaffold709637_16.7	1	p3	(TGA)5	15	655	669	GCCCAGGGATTCAAAGTACA
scaffold709750_15.1	1	p3	(CGA)5	15	5053	5067	ACTCTGCCGCCAAAAGTCTA
scaffold709784_17.1	1	p3	(TAT)6	18	195	212	TTGAGCCACTTATCATCTTTCAA
scaffold709819_17.0	1	p3	(TGC)5	15	1231	1245	CGGAGGTAGTTGGCATTTGT
scaffold709851_15.1	1	p3	(GGC)5	15	544	558	TATCGCCCGAGAAATGAATC
scaffold709927_15.3	1	p3	(CAC)5	15	236	250	GACGTCTTGCAAGTGACGAA
scaffold709987_16.9	1	p3	(GCG)6	18	442	459	TCGTCACCCTACAAGCATCC
scaffold710160_15.2	1	p3	(TAA)6	18	105	122	TTGGGGTCGGCTACATTAAC
scaffold710179_14.6	1	p3	(TGA)5	15	48	62	CCCAAGACCCCAATTTGATT
scaffold710207_16.5	1	p3	(TCA)6	18	3889	3906	AATCTCAAACCCGACAATGC
scaffold710264_17.6	1	p3	(CAA)5	15	769	783	TTCCCCTGCATGTTTAGAGG
scaffold710276_15.6	1	p3	(ATA)5	15	3141	3155	ATAGTGCAGCAAGATTGGGG
scaffold710399_16.0	1	p3	(GCT)6	18	677	694	TTCATACCTGTCCCGACCAT
scaffold710425_14.7	1	p3	(GCC)5	15	394	408	TCCACATGCAGAATCGGATA
scaffold710454_14.8	1	p3	(TCA)7	21	276	296	CCAAAGGAAGCAAACAAGGA
scaffold710461_14.3	1	p3	(CGC)8	24	1618	1641	TCCACTACTCCAATGTCTGCC
scaffold710490_15.6	1	p3	(AAT)5	15	1401	1415	ATTGTCTACCAATCGGGGTG
scaffold710546_16.4	1	p3	(TAT)5	15	458	472	GTCAAGTTTGAGACGTTGCG
scaffold710625_11.5	1	p3	(TAT)5	15	681	695	TAGCAACGCAATAACATCGG
scaffold710663_15.8	1	p3	(TTG)5	15	44	58	GGTTGACTCACACGTGGAAA
scaffold710792_14.9	1	p3	(ATT)5	15	406	420	TTCCAGTAAATCAACGAAACGA
scaffold710801_15.9	1	p3	(TCA)5	15	3100	3114	CGTTGAAATCTGTTCCTCCC
scaffold710804_14.7	1	p3	(TTG)5	15	76	90	ATGTAGCCGACCCCAATTTA
scaffold710909_14.8	1	p3	(TCA)5	15	4995	5009	TATCAACCACCCATCAGCAA
scaffold710936_15.7	1	p3	(TGT)6	18	45	62	TGTAGCCGACCCCAATTTAT
scaffold710962_14.2	1	p3	(AAT)5	15	612	626	TGACAACCGCACGTAAATTG
scaffold710998_16.5	1	p3	(TCA)7	21	2084	2104	AGTGCAAATCCCAGTGCTTC
scaffold711086_12.0	1	p3	(TTA)5	15	738	752	GGGTTTGCGTTTGATTTGTT
scaffold711156_12.0	1	p3	(AAC)5	15	461	475	CTTTGGAATTGGAAGGACGA
scaffold711215_10.6	1	p3	(TAT)7	21	576	596	GGGAGAAGAGGGTATAGCGG
scaffold711225_13.4	1	p3	(AAT)5	15	979	993	AAATCCAAAATGGCCTACCA
scaffold711260_17.5	1	p3	(AAT)5	15	139	153	TGTATCTTCTCACGAGCATTGG
scaffold711304_14.7	1	p3	(AAT)6	18	720	737	AGAACTGACGGGCAAATGAT
scaffold711472_12.9	1	p3	(TAT)6	18	311	328	GTGCATTGACCGTGTTTGAC
scaffold711492_14.1	1	p3	(AAT)5	15	744	758	ATTGTTTTAATGCATCGGCG
scaffold711502_17.1	1	p3	(TGT)5	15	44	58	ATGTAGCCGACCCCAATTTA
scaffold711628_11.5	1	p3	(ACA)5	15	1092	1106	TTCAACTTGCTCGGAACCTT
scaffold711687_17.9	1	p3	(AAC)5	15	329	343	CGTTCCCAGACTTTCCAAAA
scaffold711743_15.9	1	p3	(CAC)6	18	4840	4857	TTACACCCGCACCATCTGTA
scaffold711807_17.3	1	p3	(CGG)5	15	85	99	AATCATGAAAACCGAAACCA
scaffold711853_12.7	1	p3	(AAC)5	15	500	514	ACTACCACGCCAATTATGCC
scaffold711910_19.4	1	p3	(ATC)5	15	164	178	AAAAGAAAGAACCGAACCCG
scaffold712078_20.3	1	p3	(GGT)5	15	707	721	TCCGAGAAGATTTCGTCGAT
scaffold712140_15.0	1	p3	(CCG)5	15	614	628	ATAACACTCGAACAAGCGGC
scaffold712171_14.5	1	p3	(TTA)6	18	327	344	TCGTTCATGCTCCTCAGTTG
scaffold712207_14.2	1	p3	(ATT)6	18	622	639	CACTCTGTGTACCACCAATCTCA
scaffold712261_13.8	1	p3	(TTA)5	15	56	70	CCAGCAGTTGGCTCTATTTTT
scaffold712262_14.9	1	p3	(ATA)5	15	145	159	CCGGAATTGAACACCCTTAAT
scaffold712318_17.7	1	p3	(CCG)6	18	155	172	CATTCGGGAAAAAGGATCAA

scaffold712345_15.2	1	p3	(ATA)6	18	1200	1217	ACGCGTTAGAAACGTACGAA
scaffold712403_11.5	1	p3	(GAC)5	15	614	628	ATCCGAATGGATCAAAGCTG
scaffold712420_17.0	1	p3	(ATT)5	15	582	596	AACATGACACAATTCGTTCTGC
scaffold712430_12.6	1	p3	(AAT)5	15	1001	1015	CAATAAATTGGGGTCGGCTA
scaffold712535_15.7	1	p3	(TGA)5	15	47	61	CCCAAGACCCCAATTTGATT
scaffold712565_14.4	1	p3	(ATT)5	15	223	237	GTCCGGCAGAATACAAGGTG
scaffold712567_14.4	1	p3	(TCA)8	24	3543	3566	TCTTTTTCAGACGCAGCAGA
scaffold712598_11.4	1	p3	(ATC)8	24	325	348	GGGTGAGGATTACTTGGGCT
scaffold712613_13.6	1	p3	(ATA)8	24	3493	3516	GCAAGAGGCATGTTTTATTTGA
scaffold712707_17.1	1	p3	(ATG)5	15	1899	1913	GTTTTGAATTTTGCGACCGT
scaffold712790_14.8	1	p3	(CAA)7	21	3627	3647	ACTTTCTGCGGCAGTTCTGT
scaffold712820_16.3	1	p3	(TGT)6	18	41	58	ATGTAGCCGACCCCAATTTA
scaffold712835_15.0	1	p3	(AAT)5	15	883	897	GTCGAAAACTCAATGGGGAA
scaffold712855_13.9	1	p3	(TTG)8	24	1684	1707	TGGTGCTCCAAAAATTGTCA
scaffold712944_19.2	1	p3	(CAG)6	18	562	579	CCGGAGCCATAATTAGCAAC
scaffold712970_12.3	1	p3	(TTA)5	15	669	683	TGAGATCAAGAATTATGGCAAGA
scaffold713137_15.9	1	p3	(TTC)5	15	8313	8327	TCGAAAGCTTCACAACATGC
scaffold713205_15.8	1	p3	(ATC)5	15	860	874	GCAAGTCTCGTAGCTTTGGC
scaffold713483_14.4	1	p3	(TCA)6	18	292	309	AAAGCATATGGAAGAGCGGA
scaffold713552_17.9	1	p3	(CTT)5	15	571	585	CTGAGGCAGCAACTTCAGTG
scaffold713598_15.4	1	p3	(CGC)6	18	6331	6348	GTGTGGAAAAGGGAGTGCAT
scaffold713660_14.0	1	p3	(AAC)9	27	628	654	GAGTTGAAAGGCATGCAAGA
scaffold713746_15.8	1	p3	(TGT)7	21	41	61	ATGTAGCCGACCCCAATTTA
scaffold713777_15.6	1	p3	(ACA)6	18	2533	2550	TCCAAGCAAGCACATTTTTG
scaffold713807_15.8	1	p3	(CCT)5	15	875	889	CACCAACGAAGCCACCTATT
scaffold713897_16.4	1	p3	(CCG)7	21	399	419	GGTTCACGGTTTTGGGAATA
scaffold713947_14.9	1	p3	(ACA)6	18	1050	1067	TTTAGTGGGAGTCAAACGGG
scaffold713988_16.4	1	p3	(CCA)5	15	386	400	GGCTTAAAGACAACGGTGGA
scaffold714152_13.2	1	p3	(TCA)5	15	476	490	CAGTCTCAGGTGCGTCAAAA
scaffold714165_14.7	1	p3	(TCT)5	15	686	700	GAAACATATGCACCTGCCCT
scaffold714183_16.0	1	p3	(AGA)5	15	803	817	CGAATGACCAGCCAAGAATC
scaffold714211_15.4	1	p3	(TCA)6	18	1449	1466	TGTAGAACTCGGGTCAAGGG
scaffold714270_17.0	1	p3	(CAT)5	15	1735	1749	GGTGAGGTCTGGTCAGGTGT
scaffold714287_15.3	1	p3	(CAG)5	15	576	590	ACAGATGAGATGAGGCTGGC
scaffold714370_17.3	1	p3	(GAA)5	15	3749	3763	TTGTACCAGATCCCCCAGTC
scaffold714403_15.9	1	p3	(ATT)6	18	2882	2899	CACGTGTACGTGCGTTCATA
scaffold714448_15.3	1	p3	(GAG)5	15	344	358	TTAAACCTCCACCAGTTGGC
scaffold714449_13.0	1	p3	(CAA)5	15	180	194	GGAGCTTTCAACACAACTCCA
scaffold714464_13.2	1	p3	(TGT)6	18	44	61	TGTAGCCGACCCCAATTTAT
scaffold714475_14.1	1	p3	(AAC)5	15	637	651	AACGAATGTCACATCAACCG
scaffold714529_15.6	1	p3	(TGC)5	15	4445	4459	TGCACCTTGGCTTTCTTTCT
scaffold714592_13.3	1	p3	(ACC)7	21	720	740	CTTCCGAGCAAGGAGCTAGA
scaffold714629_18.4	1	p3	(CCG)6	18	592	609	GATCCTCTGGGCCAAATGTA
scaffold714730_16.3	1	p3	(ATT)5	15	345	359	GATCCGAAATGAAAACCCAA
scaffold714732_10.8	1	p3	(GTC)5	15	505	519	ATCCACCAAAATCATCCACC
scaffold714949_18.5	1	p3	(GAT)5	15	1030	1044	GGTCCTGGGTTTGGGACTAT
scaffold715074_16.5	1	p3	(TTA)5	15	1349	1363	GTTCGATGTGCAGATGAGGA
scaffold715220_15.3	1	p3	(CCA)5	15	1074	1088	TTTCTGAGTTGGGATACCCG
scaffold715229_12.9	1	p3	(TAA)5	15	179	193	CGCCGCCTAAATAGTCAAAA
scaffold715252_10.8	1	p3	(TGA)8	24	565	588	ATGCAGCAATTGGACATTGA
scaffold715260_10.7	1	p3	(GAT)5	15	433	447	CTCAACTTTCTTTCGAGCTGC
scaffold715391_14.6	1	p3	(TTG)6	18	1331	1348	ATGATTCATGTAGCCGACCC
scaffold715410_16.7	1	p3	(ATT)6	18	205	222	TCGAGTCCGCACATTAGTCA
scaffold715435_16.7	1	p3	(CGC)7	21	343	363	GAAACCACTCAGGCCAGTTC
scaffold715467_14.9	1	p3	(TGA)6	18	1118	1135	TTGTAAAACACCCCAATTTGA
scaffold715492_16.2	1	p3	(TTG)5	15	1060	1074	CTCCTTCTTTATCCGGGCTT
scaffold715518_13.1	1	p3	(ATA)5	15	130	144	CACCTTAGGTACCGCTCGTC
scaffold715519_16.1	1	p3	(TCA)5	15	1312	1326	TCCGAAATTTGTTCCACCAT

scaffold715564_15.	1	p3	(AAT)5	15	2247	2261	TCCAGGAGCCAAGAAAGATG
scaffold715732_13.	1	p3	(CTC)5	15	345	359	ATGAAATGAAGCTTCCACCG
scaffold715995_12.	1	p3	(AAT)7	21	187	207	TGGGGAGAGAGAGTGGAGTG
scaffold716061_15.	1	p3	(GAA)5	15	1448	1462	TGTCCTCATGGATTCTTGAAA
scaffold716147_17.	1	p3	(TCA)5	15	1961	1975	ATACCAGGGTCGAATCCACA
scaffold716170_15.	1	p3	(TCA)5	15	828	842	CTGTCACGTGTTGCAGTCCT
scaffold716216_14.	1	p3	(ATG)6	18	139	156	CTCTTGGAGAGGCCAACAAG
scaffold716233_15.	1	p3	(CGC)5	15	715	729	AAACCTTAGGCTTCCTTGGC
scaffold716235_16.	1	p3	(AAG)5	15	545	559	TCATGGAGAGAGGAGAGGGA
scaffold716300_19.	1	p3	(TAT)5	15	202	216	GGCTTAATACGTGTGTTGCG
scaffold716311_13.	1	p3	(TTG)5	15	482	496	GGTTCATTTAACCGACCCAA
scaffold716318_14.	1	p3	(TCA)5	15	355	369	GTGGGCAATCTTGTTGTGTG
scaffold716321_15.	1	p3	(TTA)5	15	422	436	GGTAGCACTGCATCTGTCCA
scaffold716407_13.	1	p3	(AAT)7	21	567	587	AACGGTGAAAAACATAATGCAG
scaffold716604_16.	1	p3	(TGA)6	18	45	62	CCCAAGACCCCAATTTGATT
scaffold716692_15.	1	p3	(TCC)6	18	97	114	CGTTTCTTCATCATCATCCG
scaffold716725_15.	1	p3	(TGA)5	15	3427	3441	ACACCAAAATGCATGAGACG
scaffold716750_11.	1	p3	(GAG)6	18	762	779	CACTGCTCTCACCACGATGT
scaffold716769_16.	1	p3	(CGC)7	21	80	100	CTTCTTTTCAAGCACTGGGC
scaffold716806_14.	1	p3	(TTA)5	15	70	84	TAGATTTGAACGACACCCCC
scaffold716825_16.	1	p3	(ATG)5	15	352	366	TTGGCTGGTTGATCTTAGCA
scaffold716826_16.	1	p3	(TTC)5	15	875	889	TGAGGCTGGGTTAGAATTGG
scaffold716987_16.	1	p3	(CGG)5	15	181	195	CTGGAAGGAGAGACGGCTTA
scaffold717225_16.	1	p3	(CTT)5	15	3849	3863	AAACGCCCTCCTTCTCTAGC
scaffold717331_20.	1	p3	(AAT)6	18	84	101	TTCGGTCCGATTAATCAAGAAT
scaffold717375_15.	1	p3	(CTC)5	15	2128	2142	CAGGAGTTCAAGTCGCACCT
scaffold717383_17.	1	p3	(AGA)6	18	1009	1026	CTCGCATCTTCTACGCCAAT
scaffold717481_17.	1	p3	(GAG)6	18	3713	3730	TTTTCCGAGCTTGTGTTGTG
scaffold717493_23.	1	p3	(AAC)5	15	257	271	TGCAAAATGGATTCAGGTCA
scaffold717538_15.	1	p3	(TGT)6	18	42	59	ATGTAGCCGACCCCAATTTA
scaffold717557_15.	1	p3	(AAG)7	21	696	716	CAAATGCGATTCAAGTGACC
scaffold717576_10.	1	p3	(ACA)6	18	467	484	TTTCGGACCATTGATATCTTTTT
scaffold717646_17.	1	p3	(ATG)5	15	211	225	GCCAAGGGAGTTGTTACGAA
scaffold717720_14.	1	p3	(TGA)7	21	2527	2547	TGAACGTATGGCCTTGGATT
scaffold717743_16.	1	p3	(AGG)6	18	2272	2289	AGAGGAGGAGGCTAAGCGAC
scaffold717769_15.	1	p3	(TGA)7	21	868	888	CCACTGAAATATTGAAGGCCA
scaffold717782_14.	1	p3	(GAG)6	18	185	202	TGTGGATGAAGATGAAGATTTTG
scaffold717812_13.	1	p3	(GAG)7	21	102	122	TGATGGTGTGACTGAAAGCC
scaffold717882_16.	1	p3	(AAT)5	15	1455	1469	GTTGGCCCTCCAAACCTAAT
scaffold717894_16.	1	p3	(ATC)6	18	453	470	CACAACAAAAACAATAAAAGGCA
scaffold717970_16.	1	p3	(AAG)5	15	309	323	ACTCAACACGGCCTGGATAC
scaffold717991_13.	1	p3	(CTC)5	15	848	862	TCTCCTTCCAACACCCAAAC
scaffold718036_14.	1	p3	(CCG)5	15	1267	1281	CGTCGTCCTTTTCTTCAACC
scaffold718041_15.	1	p3	(CCA)5	15	180	194	GATCACTGCAACAAGGGGAT
scaffold718061_14.	1	p3	(TAA)5	15	1344	1358	CCCATCTGACGGACAAAAGT
scaffold718078_14.	1	p3	(AAT)5	15	487	501	CATCGACAAAATCATAGTCCGA
scaffold718125_12.	1	p3	(GGC)5	15	560	574	TCAGTTACAGTGGGCAGCAG
scaffold718133_14.	1	p3	(ATC)7	21	115	135	ACCACCATCGACTGATAGCC
scaffold718203_14.	1	p3	(CTC)5	15	1826	1840	GCTCCTCCTCTTACGCCTCT
scaffold718233_14.	1	p3	(TCC)5	15	5104	5118	TCAACACACCAAAACACCAAA
scaffold718331_15.	1	p3	(TAT)5	15	272	286	CTTTATTAAAATTTCTTTCACCAAATG
scaffold718347_15.	1	p3	(TTG)5	15	48	62	TGTAGCCGACCCCAATTTAT
scaffold718539_13.	1	p3	(AAC)5	15	78	92	TATTGGGCCGTACCTCTCCT
scaffold718616_18.	1	p3	(ATT)5	15	468	482	ACCATGCCAGACTTTGGTTC
scaffold718668_14.	1	p3	(TAA)6	18	566	583	TGCAAATACCTAAAGGTTGGG
scaffold718672_13.	1	p3	(CCG)5	15	97	111	CCCTACCCTCCTCAGGGTTAC
scaffold718676_16.	1	p3	(ATT)5	15	326	340	TTTTGCATATCTCCATTCGGT
scaffold718686_13.	1	p3	(CGG)8	24	833	856	TGCTTTTCAGGGAGTTGGTC

scaffold718739_15.3	1	p3	(ACA)5	15	348	362	ACCCGCAAAAACACAAAAAG
scaffold718826_16.1	1	p3	(AAG)5	15	943	957	TTTCAATCACTTTTTCTCGACG
scaffold718869_16.2	1	p3	(ATA)7	21	2739	2759	GCTCGAATATGCAACGACAA
scaffold718926_14.9	1	p3	(ACA)5	15	482	496	GAAGGGAAGCATTCCAAACA
scaffold718950_15.0	1	p3	(TTG)5	15	48	62	TGTAGCCGACCCCAATTTAT
scaffold719004_15.4	1	p3	(GAA)5	15	1125	1139	TTATGGGGGACCCTATCTCC
scaffold719096_14.1	1	p3	(GAT)5	15	1494	1508	AACCGTTGATATTTGGCCTG
scaffold719124_15.0	1	p3	(GGT)5	15	466	480	AGTATGGGCATATGGCAAGG
scaffold719153_14.8	1	p3	(AAT)5	15	588	602	ATTGGGACATTTGGGTGTGT
scaffold719159_10.3	1	p3	(ATA)5	15	215	229	TCAAATAGCCGATTGCATCA
scaffold719225_15.2	1	p3	(AAT)5	15	675	689	GTGAAGTTGTCAATTCCCCG
scaffold719275_11.6	1	p3	(CCA)5	15	614	628	CGCTTCGTACGCTTTTAACC
scaffold719285_14.7	1	p3	(CTT)5	15	1233	1247	CGATGGATAAGGCCACTTGT
scaffold719494_17.3	1	p3	(TGG)6	18	1048	1065	GAGAGGTTGAAGGCGAACAC
scaffold719496_14.7	1	p3	(CCA)5	15	146	160	CACCAGCATTTCCATCTCCT
scaffold719547_15.5	1	p3	(AAT)5	15	922	936	CATGCGATCTAGAGTGTGGTG
scaffold719556_17.3	1	p3	(CGT)6	18	2085	2102	CGGAAGACCAGAGGTGAGAG
scaffold719562_13.4	1	p3	(ATA)6	18	241	258	CGAGAATTTCCTCGGATTCA
scaffold719770_16.0	1	p3	(TGA)6	18	189	206	TTAGCGTACATCTGACCCCC
scaffold719776_18.0	1	p3	(AGC)6	18	515	532	GACAGCATTGCTTTGCCTTT
scaffold719812_15.4	1	p3	(GAA)5	15	1803	1817	TCAACTCATGCAAATGCAGC
scaffold719843_15.9	1	p3	(CAT)6	18	1272	1289	GGTTAAGCATCGGATGAGGA
scaffold719917_16.0	1	p3	(TTG)6	18	128	145	ACCTCACATGGTGGGAAAAA
scaffold719937_16.6	1	p3	(TGA)8	24	2560	2583	AGGTTGCGTACATCTGACCC
scaffold720034_15.7	1	p3	(ATA)5	15	2107	2121	CACGGCAGAGCAATTCAATA
scaffold720364_13.3	1	p3	(TAT)6	18	540	557	AATAATTAGGCGTGCAACGG
scaffold720382_17.6	1	p3	(AAT)6	18	1046	1063	GAATCATCTCACACCACCCA
scaffold720424_16.8	1	p3	(CGG)8	24	226	249	GAAGTGGAGGTGACGGATGT
scaffold720451_18.3	1	p3	(AAT)7	21	627	647	TTGAATCCTCAGCGTGCATA
scaffold720464_19.0	1	p3	(CCG)5	15	2396	2410	GCCTCACAGCTGAAATCCTC
scaffold720547_14.6	1	p3	(CAA)5	15	87	101	CACAACTTGATCAAAATTGCAGA
scaffold720557_13.6	1	p3	(TGT)6	18	46	63	ATGTAGCCGACCCCAATTTA
scaffold720717_12.1	1	p3	(TCT)5	15	1579	1593	CTCGGTTTCTTGAGTCAGGG
scaffold720718_15.0	1	p3	(GAC)7	21	1509	1529	TCAAAACCTGAAGCTGTCCC
scaffold720780_15.2	1	p3	(CCT)5	15	1688	1702	ATCCAAAGGGCTTGCTTCTT
scaffold720801_15.4	1	p3	(AAT)6	18	909	926	TAGATGCCTGTTGGGGTCTT
scaffold720837_16.8	1	p3	(CCT)7	21	984	1004	ATCCTGAGTTGCACACCCTT
scaffold720863_19.3	1	p3	(AAT)7	21	1286	1306	CGCGTCGCCTAAATAAAAAT
scaffold720866_15.6	1	p3	(GTT)6	18	62	79	TGTAGCCGACCCCAATTTAT
scaffold720923_19.3	1	p3	(TCA)8	24	1132	1155	CCTCTGGAATGGGAGTTTCA
scaffold720936_16.7	1	p3	(GCA)6	18	852	869	TTAAACCGACCTCGCTGTTT
scaffold720949_15.4	1	p3	(ACT)6	18	390	407	ACATACGCGTGCTTCACAAG
scaffold721101_18.0	1	p3	(TGT)6	18	40	57	TGTAGCCGACCCCAATTTAT
scaffold721102_15.3	1	p3	(ATC)5	15	384	398	TGGAATCCTGTCCTCTGGTT
scaffold721128_14.7	1	p3	(TTA)8	24	496	519	GTTCCGGCCCTTCAATAATC
scaffold721162_17.1	1	p3	(CTT)5	15	2197	2211	TGATGTTGGTATTTTGGCTACC
scaffold721195_15.8	1	p3	(ATA)5	15	74	88	TGCAGCTCTTAACTCTATCGGA
scaffold721206_14.9	1	p3	(TTA)5	15	62	76	GCAATATCAATGTAAGATCATATTCA
scaffold721214_15.3	1	p3	(AAT)5	15	496	510	TGTTCCCGTGGTATGTTTTT
scaffold721273_14.1	1	p3	(TGA)5	15	166	180	TAAGGAGGCGAGAGAATGGA
scaffold721319_15.3	1	p3	(TAT)5	15	2014	2028	CTAAGCGAGATACCAAGCCG
scaffold721329_13.3	1	p3	(TAA)5	15	384	398	GGTTGGTTGTACAAAGAAACGAG
scaffold721398_17.1	1	p3	(TTG)5	15	2008	2022	GAATTTGCTTTGTCATTGGGA
scaffold721554_15.2	1	p3	(TAT)5	15	501	515	TCCATGCATTTCCAACACAC
scaffold721567_18.0	1	p3	(TCT)5	15	397	411	GAGAACCCTAAACCGAAGGG
scaffold721579_15.3	1	p3	(ATA)5	15	359	373	ACTCCAATGCTTCCCTTCTT
scaffold721591_17.2	1	p3	(TGA)6	18	5732	5749	GCACCCCTACAGCATTGTTT
scaffold721605_16.6	1	p3	(CGG)6	18	591	608	CCACTCCCTCCACTTTCTCA

scaffold721630_15.2	1	p3	(ATG)7	21	1318	1338	GCATTGCTTGACTGAGTTGC
scaffold721722_13.4	1	p3	(TTA)5	15	727	741	TTTGTTACGGGAATAACCCAA
scaffold721812_17.5	1	p3	(TGA)6	18	43	60	CCCAAGACCCCAATTTGATT
scaffold721862_14.6	1	p3	(ATT)5	15	511	525	TGTCTCGACGGACTGACACT
scaffold721868_14.9	1	p3	(TTG)5	15	776	790	GGAGACCTAAGGATGCACCA
scaffold721905_18.5	1	p3	(CGG)5	15	325	339	GTTCTGAGGTTGGTTGGTCG
scaffold721917_17.1	1	p3	(AAT)5	15	519	533	TGGGTAGCTTACAAGTCCAGC
scaffold721922_10.3	1	p3	(ATT)7	21	1066	1086	TCATCATCTGGCTTTGTGGA
scaffold721955_14.9	1	p3	(CAT)5	15	6061	6075	CCGCTACAATCTTTGGTGCT
scaffold722006_16.1	1	p3	(GAA)5	15	1844	1858	TCAGAAGAGAGTTTGCCATTGA
scaffold722013_18.4	1	p3	(TGC)5	15	96	110	ATTCAAAGCATATGCCCTCC
scaffold722029_15.1	1	p3	(TAA)5	15	143	157	CGTGTTTGAGTTTCTTGTTTGG
scaffold722068_16.9	1	p3	(CGG)5	15	1972	1986	CCGTTTCTCCAGTTGACGTT
scaffold722076_10.0	1	p3	(ACA)6	18	759	776	AACCTAATCCATCCCAACTTTT
scaffold722091_17.1	1	p3	(TAA)5	15	701	715	TAGAAGGAGATGGGTCCTGC
scaffold722101_14.8	1	p3	(AAT)5	15	200	214	CAAACAGCGATTTTGAGTCG
scaffold722123_12.5	1	p3	(TGA)6	18	978	995	AGCTCATCGCAGGTTGGATA
scaffold722127_14.7	1	p3	(GGT)5	15	2934	2948	GTTAGATTGGACCCCATCCC
scaffold722201_18.2	1	p3	(CGC)6	18	655	672	TCAGCTCTAACCGGAGGAAA
scaffold722234_16.5	1	p3	(AAT)7	21	125	145	TCACATCACTAGTCCGTGGG
scaffold722308_15.1	1	p3	(AAG)5	15	618	632	ATAAACGCAACTCAAACCCG
scaffold722351_16.6	1	p3	(CTA)7	21	399	419	CTACTTCTGCACTGCCTCCC
scaffold722352_13.7	1	p3	(AAC)5	15	600	614	GCATTTCCGTGCAACAACTA
scaffold722465_14.9	1	p3	(TGT)6	18	42	59	TGTAGCCGACCCCAATTTAT
scaffold722522_17.2	1	p3	(ATC)5	15	274	288	CAAACCCACCAACAAACAAA
scaffold722539_15.6	1	p3	(AAT)5	15	158	172	GCGTGTTTTCCAGAATCCAT
scaffold722747_14.8	1	p3	(GAC)6	18	570	587	CTGATTCCATTTCCCCTTCA
scaffold722770_11.8	1	p3	(CTT)7	21	886	906	ACATGAGAGCCTTGCATCCT
scaffold722809_15.5	1	p3	(ATA)6	18	1032	1049	ACAAACGATTTCCCGTCTTG
scaffold722865_16.6	1	p3	(ACA)6	18	3430	3447	CACGACGGAAACATGTAGTGA
scaffold722866_16.6	1	p3	(CGC)5	15	1689	1703	AAGAGAAACTTGACGAGCGG
scaffold722871_15.8	1	p3	(TGC)5	15	585	599	CTTGATTGATCTTGGTGCGA
scaffold722878_13.4	1	p3	(ATT)8	24	63	86	GGGAGTGGGTTATCGGTTTT
scaffold722908_10.5	1	p3	(GGC)5	15	297	311	ATTTGTAGGGTGGTGGTGGA
scaffold722934_15.7	1	p3	(AAT)5	15	1062	1076	AGGGAAGTGGAAATAAGCCC
scaffold722953_13.1	1	p3	(CGA)5	15	2574	2588	TGCGAACAATCTCTCCTCCT
scaffold722989_17.4	1	p3	(TTG)7	21	71	91	TTGGTTCATGTAGCCAACCTC
scaffold723043_15.3	1	p3	(ATT)5	15	955	969	AGCAAACAGCTCCCATCATC
scaffold723081_15.1	1	p3	(ATA)5	15	985	999	GGGAAATGGCTTGCATAAAA
scaffold723112_17.7	1	p3	(TCT)6	18	410	427	GGGTACAGGTGGATGGTAAGAA
scaffold723121_15.4	1	p3	(TAA)5	15	363	377	TCTCCTGTGTGGTGTGTGGT
scaffold723125_17.5	1	p3	(AAT)5	15	612	626	GCCCACACAAATTATCGAGG
scaffold723170_16.3	1	p3	(GGC)6	18	44	61	GAATTGGAGGCGGAGGAG
scaffold723197_16.3	1	p3	(GTT)5	15	153	167	CCATTTTCGCCTCAATCTGT
scaffold723238_19.7	1	p3	(GCG)5	15	2014	2028	AGGTCCCCGGATTTGTTATC
scaffold723244_16.8	1	p3	(ATA)5	15	321	335	GCCGAGGGTGATGTATCAAT
scaffold723287_13.5	1	p3	(CCA)5	15	387	401	ACGCCTCATCGTCAAAACTC
scaffold723382_16.1	1	p3	(TAT)5	15	5234	5248	GCCAGGTTTTCTGGGTACAG
scaffold723441_15.4	1	p3	(AAG)7	21	635	655	TCGCGAGTAAAACAAAAACA
scaffold723709_16.9	1	p3	(GGC)5	15	865	879	CCCAAACTGATTTCGCAACT
scaffold723713_13.6	1	p3	(ATT)7	21	1186	1206	TGGAATCTTCCCTTCCCTCT
scaffold723742_12.4	1	p3	(TGA)7	21	1548	1568	AAGAAGAGGGTTCGCTGTCA
scaffold723899_15.5	1	p3	(ATC)5	15	981	995	TCGGCAAAACCCTTTTATCA
scaffold723925_16.7	1	p3	(AAT)6	18	1568	1585	CGAAGGGACCACAAAAGGTA
scaffold723994_16.3	1	p3	(GGA)5	15	647	661	ATTTTCTTGTGGGTGGAAGC
scaffold724039_17.7	1	p3	(AAG)5	15	855	869	TGCCAAGCTTATATTATTGGTGC
scaffold724101_11.8	1	p3	(TAT)6	18	216	233	GCTAACGACGTAAGGCAAATG
scaffold724217_14.9	1	p3	(CTT)5	15	2990	3004	AACACCGAGTGCAACTTCCT

scaffold724260_12.9	1	p3	(TGA)7	21	937	957	TTCTTCCACAGTTGGCTCCT
scaffold724274_16.1	1	p3	(TGA)5	15	1583	1597	TTCGCTCTGTGTTTTCCTCA
scaffold724278_16.5	1	p3	(CTC)5	15	1518	1532	CAATGAACTCCAAGGGGAGA
scaffold724281_13.8	1	p3	(CGG)5	15	179	193	AGGATTTGAACCGGTGACAG
scaffold724467_16.7	1	p3	(TGT)6	18	50	67	TGTAGCCGACCCCAATTTAT
scaffold724478_13.3	1	p3	(TTA)6	18	1330	1347	CCCAGACTGAGTTATTATTTGATCC
scaffold724483_14.2	1	p3	(AAG)5	15	361	375	ACTCTTTCAGCCCCTTGGAT
scaffold724487_14.2	1	p3	(GGC)5	15	237	251	ATAGTAGCGGCTGCAGGTTC
scaffold724494_15.4	1	p3	(ATA)7	21	684	704	TGATGTTCTTTGGGTGCTGA
scaffold724520_15.4	1	p3	(CCG)5	15	574	588	AACAATAATATCGTCCGCCG
scaffold724534_16.4	1	p3	(TGA)5	15	1252	1266	AGCCACTGCTTTTGTTGCTT
scaffold724536_17.1	1	p3	(ACA)5	15	960	974	TGAATGTCCAACTCTTCCACC
scaffold724570_16.4	1	p3	(TCT)5	15	1407	1421	TTGCTTCAGCACCTTGAAGA
scaffold724663_24.0	1	p3	(ACA)7	21	924	944	GGTATTTTCATCCCGCTCAA
scaffold724670_15.3	1	p3	(CAG)5	15	61	75	ATGGCCCTAAACACAGCAAG
scaffold724747_14.6	1	p3	(TTA)7	21	1156	1176	GCCACAAATTCTGAAACCGA
scaffold724764_18.8	1	p3	(CTC)5	15	433	447	AGTCTCTGCTGCTCAGCCTC
scaffold724777_15.5	1	p3	(CCG)6	18	604	621	AATATGGCATGAGACGAGGC
scaffold724786_14.0	1	p3	(ATT)5	15	326	340	GGATCGTGTGTGGGATCAAT
scaffold724850_16.9	1	p3	(CAA)5	15	139	153	CTCAACCAGCATCGCTATCA
scaffold724896_14.1	1	p3	(CTG)5	15	1021	1035	TCTGAATCCCTTTTCATCCG
scaffold724953_11.5	1	p3	(AAT)6	18	773	790	CAGCCTTCCAGTTGGGATAA
scaffold724987_17.7	1	p3	(GGC)7	21	466	486	GATCCACCTCTCCATCTCCA
scaffold725053_14.2	1	p3	(TGA)5	15	48	62	CCCAAGACCCCAATTTGATT
scaffold725062_14.0	1	p3	(GAA)5	15	909	923	CCAGGTGTTGTTTTTCAGCA
scaffold725080_13.6	1	p3	(TTA)5	15	1827	1841	ATGTATATTTCGTTCTTTCTTGTTTTT
scaffold725108_19.2	1	p3	(CTG)6	18	127	144	GATCTGTTGAAGGTAGCGGG
scaffold725127_17.6	1	p3	(CGC)5	15	640	654	GATGCAGGGATACTGATTCCA
scaffold725135_17.0	1	p3	(TTG)7	21	1313	1333	TGGCAAACAAAGGATGTCAG
scaffold725138_14.1	1	p3	(AAT)5	15	800	814	TCACATGCTTTGCTGAAAGG
scaffold725219_14.4	1	p3	(TGT)5	15	805	819	TGCTCACCCACATTCACATT
scaffold725227_15.7	1	p3	(CCA)5	15	2413	2427	CCCCATATCTACCCCCTCAT
scaffold725252_15.8	1	p3	(ACA)5	15	1181	1195	ATGTGGTCACCTGGGGAATA
scaffold725280_16.0	1	p3	(AGT)5	15	1128	1142	CCTCGGATGAGTCGCATATT
scaffold725300_14.6	1	p3	(CCG)5	15	810	824	TCTTGCCGCCTTGTTAATTT
scaffold725307_17.7	1	p3	(AAT)5	15	221	235	TCCAAGTTACTGGCCAAACC
scaffold725363_15.8	1	p3	(ATA)5	15	324	338	CTCCACGTTTTAACAACCGA
scaffold725377_16.1	1	p3	(ATC)6	18	208	225	TGAACAAGGTTTCGATCTATGC
scaffold725384_17.6	1	p3	(GGT)6	18	865	882	GAGGATTGGGGGTTTTCATT
scaffold725394_15.9	1	p3	(AAT)5	15	879	893	GGCATTATCAGAATTTGGCG
scaffold725446_15.9	1	p3	(ATG)5	15	702	716	GCTGCATTGCTTGAAATGTCT
scaffold725543_15.9	1	p3	(TGA)5	15	45	59	CCCAAGACCCCAATTTGATT
scaffold725593_15.4	1	p3	(AAT)5	15	2588	2602	TTCCTTGGAACTCTCAATCCA
scaffold725603_19.8	1	p3	(TTC)6	18	440	457	CGTTGGTGGAATATCGTTGA
scaffold725607_14.4	1	p3	(CTT)5	15	1076	1090	CACCTCATCTTGCTTCGTCA
scaffold725924_17.9	1	p3	(ATC)7	21	404	424	GCAACGAAAAATACAAGCCAA
scaffold725951_18.4	1	p3	(GGC)5	15	2039	2053	CACAACACCACTCATCCTCG
scaffold725954_16.3	1	p3	(CAG)5	15	3479	3493	GATGCTTTGTGCAGTTGAGC
scaffold725965_16.4	1	p3	(TGA)6	18	39	56	CCCAAGACCCCAATTTGATT
scaffold726097_16.0	1	p3	(TCT)5	15	177	191	ATCTCGGGGTGCATTGTAAC
scaffold726100_17.2	1	p3	(CCT)6	18	4726	4743	CGCCTCTTGAATAACCTCCA
scaffold726146_10.5	1	p3	(AGG)7	21	328	348	AGTGGTGAAGTCCCACCTTG
scaffold726172_14.7	1	p3	(GGA)5	15	1727	1741	ACTCTGGAAAACCGATGACG
scaffold726195_15.0	1	p3	(TGT)7	21	45	65	TAGCCGACCCCAACTTATTG
scaffold726234_16.3	1	p3	(TAA)6	18	1877	1894	TTTTAAAACGCCTGAAAAAGTAA
scaffold726362_16.4	1	p3	(ATT)5	15	599	613	GCACTCGAAGAGTAGCGGTT
scaffold726404_14.0	1	p3	(ATT)5	15	1032	1046	GGCAAATTTGAATCGGTGAT
scaffold726438_15.4	1	p3	(TAT)5	15	770	784	ACCGTCATGGCCCTACATTA

scaffold726541_13.[illegible]	1	p3	(TGT)7	21	113	133	GATTCATGTAGCTGACCCCAA
scaffold726553_15.[illegible]	1	p3	(ATT)5	15	885	899	CGAAGGATCAACTTCATAGTCG
scaffold726670_14.[illegible]	1	p3	(CTA)5	15	456	470	CCCCACAACGTTATTCATCC
scaffold726704_14.[illegible]	1	p3	(TTA)6	18	393	410	TGGCAGCAACTACGACCATA
scaffold726717_18.[illegible]	1	p3	(TTA)5	15	289	303	TTGCTGCTGGTATCAAAACG
scaffold726718_15.[illegible]	1	p3	(CAT)5	15	1413	1427	GGTCATCAATTCAGGAAGCC
scaffold726723_17.[illegible]	1	p3	(TCA)5	15	528	542	CGAACCGATAAAGCCAAAAC
scaffold726769_14.[illegible]	1	p3	(CAT)5	15	1071	1085	GAACCATGATCTGCCTGGAT
scaffold726858_17.[illegible]	1	p3	(TAT)5	15	267	281	GTTGAAGCCAAGCGTGCTAT
scaffold726926_13.[illegible]	1	p3	(ATA)5	15	464	478	TGACAAATAAAAACCTACAAAATGG
scaffold726957_16.[illegible]	1	p3	(GAG)5	15	770	784	AAGAGGAGGACGAGCATGAA
scaffold727127_14.[illegible]	1	p3	(AAT)7	21	444	464	CATAGGGCAAGTACGGGAAA
scaffold727132_14.[illegible]	1	p3	(TTA)5	15	662	676	CACCGGTTTTGGATTTGACT
scaffold727149_15.[illegible]	1	p3	(ACA)8	24	896	919	CAAATTTAAGTGAGCTTTGCTTGA
scaffold727177_14.[illegible]	1	p3	(TTA)7	21	1035	1055	ATGGCATCCATCTCTTCGTC
scaffold727182_16.[illegible]	1	p3	(AAC)5	15	2194	2208	CTGCACACTTTGGTCCATTG
scaffold727192_13.[illegible]	1	p3	(TGA)5	15	127	141	CCGGAACTCAATAACCAAAGA
scaffold727270_15.[illegible]	1	p3	(TTA)5	15	2526	2540	ATGAGAGGGAGAGGGAGAGC
scaffold727297_13.[illegible]	1	p3	(CAA)6	18	464	481	CATTCATTTCCAGGCCACTT
scaffold727315_14.[illegible]	1	p3	(AAT)7	21	672	692	TGGAATCAAGAACCCCAAAG
scaffold727348_15.[illegible]	1	p3	(TTG)5	15	129	143	CACATAGTGGGAAACGGCTT
scaffold727374_17.[illegible]	1	p3	(GCG)6	18	812	829	AACAGCAAGGAGAGGCAAAA
scaffold727429_15.[illegible]	1	p3	(CGC)5	15	4889	4903	GGTTTTGGATGAAGCGGTAA
scaffold727464_14.[illegible]	1	p3	(ATT)5	15	298	312	AGTTCAGCGAGAACAAGGGA
scaffold727532_15.[illegible]	1	p3	(CAT)5	15	573	587	CATCAACATTCCTGGGCTCT
scaffold727585_16.[illegible]	1	p3	(CCG)5	15	1507	1521	TAAACTCCGGCATGGAGAAC
scaffold727637_16.[illegible]	1	p3	(TGT)8	24	67	90	TTTGGTTCATGTAGTCGACCC
scaffold727698_16.[illegible]	1	p3	(ATA)5	15	1959	1973	AACGAGCCAAGTGAAAGCAT
scaffold727816_15.[illegible]	1	p3	(GCT)8	24	596	619	ACATCCTCAACCATGGCAGT
scaffold727891_15.[illegible]	1	p3	(ACA)5	15	1509	1523	ATGTCCTGTTGATTCCGAGG
scaffold727900_15.[illegible]	1	p3	(TCA)6	18	2059	2076	CGTACGTTGTACGGGTTCAT
scaffold727962_18.[illegible]	1	p3	(CGG)5	15	2596	2610	AGAGTGGTAAAACTCCGCCA
scaffold728058_17.[illegible]	1	p3	(GGC)5	15	329	343	TCATCGGGTCTAGGTGTTCC
scaffold728166_17.[illegible]	1	p3	(AGG)5	15	1370	1384	GATTCCGACTCCAATCCTGA
scaffold728202_15.[illegible]	1	p3	(ACA)5	15	127	141	TATTTCCGACAATTCGGTCC
scaffold728260_14.[illegible]	1	p3	(TTG)5	15	34	48	CATTTCAGTTTACTGGGATTAAGG
scaffold728267_17.[illegible]	1	p3	(AAG)5	15	2417	2431	AGTTGGAACCTCGAGCATTG
scaffold728315_14.[illegible]	1	p3	(ATA)7	21	845	865	ATGTGGCGACATGAATTTCC
scaffold728354_15.[illegible]	1	p3	(AAC)5	15	1053	1067	CCTGGCCTTAAGTAGCCACA
scaffold728364_17.[illegible]	1	p3	(ACC)5	15	254	268	CAAACGACGAGCATCAAGAA
scaffold728386_10.[illegible]	1	p3	(TAA)5	15	383	397	TCATACACGTGGACACCTCA
scaffold728538_18.[illegible]	1	p3	(GCT)5	15	2579	2593	GAAGCAGAGGAGTTCCAACG
scaffold728563_15.[illegible]	1	p3	(CTC)6	18	1587	1604	TTTAATTACCAACCTGCCGC
scaffold728618_13.[illegible]	1	p3	(CTT)5	15	700	714	AGGCGATCAACTTCCACAAC
scaffold728710_16.[illegible]	1	p3	(TAT)5	15	739	753	GATGAGGAATGTGATTGCGA
scaffold728755_17.[illegible]	1	p3	(GGA)5	15	743	757	AATCATGGCATTGGCCTTAG
scaffold728756_16.[illegible]	1	p3	(GAG)6	18	2306	2323	TGTCTGAATTTGGTGGAGGA
scaffold728840_15.[illegible]	1	p3	(TTG)5	15	1092	1106	TGCCTCTCCTTCTTGTTGCT
scaffold728924_14.[illegible]	1	p3	(TAT)6	18	2623	2640	CATCAAACCCAAACCAACCT
scaffold729016_16.[illegible]	1	p3	(GCT)5	15	134	148	ATCTCGTTCGTCTCGCAGTT
scaffold729033_10.[illegible]	1	p3	(ATT)5	15	158	172	TGCAAATTGAATCAACCAAAA
scaffold729107_15.[illegible]	1	p3	(CAT)7	21	2248	2268	TCTGCCAGTAACTTTCGCCT
scaffold729119_14.[illegible]	1	p3	(TTC)5	15	723	737	TTTCCTTTTCTTTTGCTACCCA
scaffold729153_13.[illegible]	1	p3	(ATC)5	15	214	228	TATGGTTTGGTCCGATGGAT
scaffold729155_20.[illegible]	1	p3	(TCA)6	18	1159	1176	TGGGTGATTGAGTTTCCTCC
scaffold729160_15.[illegible]	1	p3	(TTA)5	15	2427	2441	TGGCCTAATCGTTCCTCAAC
scaffold729202_15.[illegible]	1	p3	(TGG)7	21	1915	1935	TCTAAAGCCTCCCACGAAGA
scaffold729346_18.[illegible]	1	p3	(AGA)5	15	1023	1037	TGAAACCCTAGCGAACCAAC

scaffold729349_15.9	1	p3	(TGA)5	15	1180	1194	TTCGTTGTTCCACACTTCCA
scaffold729374_13.9	1	p3	(CCT)5	15	176	190	CATCAACCTCCTTCTCCGAC
scaffold729407_15.4	1	p3	(GCC)5	15	1530	1544	TCTTGGTGGTTCTGGTAGGG
scaffold729450_15.9	1	p3	(ATA)6	18	7598	7615	TCAGCAACTACGTGCATCCT
scaffold729477_18.0	1	p3	(TTA)6	18	277	294	CGGGCTCTTAAATTCATTCG
scaffold729534_15.3	1	p3	(GCG)5	15	493	507	TAGGACTAGGGAAAACGGCA
scaffold729599_16.3	1	p3	(ATT)6	18	52	69	CAACAACTCGTCACCCCTTT
scaffold729611_16.0	1	p3	(AAG)5	15	143	157	TGGAGCAACTTCTTCGATCA
scaffold729643_14.7	1	p3	(TTG)5	15	128	142	GCCAACCTCACATAGTGAGAAA
scaffold729668_16.5	1	p3	(ATC)5	15	6189	6203	CAATCAAATTGGGGTCTTGG
scaffold729670_16.4	1	p3	(TGT)5	15	38	52	TGTAGCCGACCCCAATTTAT
scaffold729673_14.6	1	p3	(TGT)5	15	82	96	ATGTAGCCGACCCCAATTTA
scaffold729776_15.4	1	p3	(ATC)6	18	1071	1088	TGCAAGAGATTGGGTTGAAA
scaffold729843_17.8	1	p3	(ATA)8	24	46	69	GAAGCAGAAATCGGGATTGA
scaffold729874_19.0	1	p3	(CAC)6	18	181	198	AACCCAAATCGCAAACAGAC
scaffold729926_15.6	1	p3	(TGA)7	21	123	143	TTTTTGATTGGGAATAATTGGG
scaffold729957_14.1	1	p3	(TAA)7	21	1571	1591	ATCCACCCCACACATCATTC
scaffold730055_15.3	1	p3	(AAC)6	18	1402	1419	CGAAAAGATGTATGGCGAAAA
scaffold730086_18.0	1	p3	(GGT)5	15	741	755	GGATTCGTGACTTTCAGGGA
scaffold730115_14.2	1	p3	(TGT)5	15	46	60	TGTAGCCGACCCCAATTTAT
scaffold730173_14.1	1	p3	(CCG)6	18	667	684	CCAAAGCAAAAACATCCCAC
scaffold730238_16.0	1	p3	(AAC)5	15	120	134	AATCGACAATTTCGGTTTCG
scaffold730322_12.5	1	p3	(ATA)5	15	286	300	GCCATGAAAAACAAGGAGGA
scaffold730399_18.8	1	p3	(CGG)5	15	637	651	CACTGACCTTGGACTTCGCT
scaffold730429_15.6	1	p3	(GCT)5	15	3063	3077	GTGAAAATCCTCCCTCGTGA
scaffold730463_16.8	1	p3	(CTC)6	18	2158	2175	GCTGCCTCTGGACAACTCTC
scaffold730475_13.8	1	p3	(TGG)7	21	2101	2121	GAGCCGATGAGGCTGTTTAG
scaffold730581_12.1	1	p3	(GCT)5	15	600	614	TCAGAAAGAGGAGACGGAGG
scaffold730589_15.1	1	p3	(TCT)6	18	201	218	TTCGATCACAAGAAACGACG
scaffold730624_15.8	1	p3	(CCT)5	15	1793	1807	GGGAAGGGGGTACATGCTAT
scaffold730640_15.3	1	p3	(CAG)5	15	434	448	TCAGCAGCAGCAAATACAGC
scaffold730730_14.8	1	p3	(ATA)5	15	2699	2713	AGACAACGAGGGATGACGAC
scaffold730758_17.2	1	p3	(AAT)5	15	2883	2897	TAACTTGAGCCGACATCCCT
scaffold730759_15.6	1	p3	(CCA)6	18	250	267	TGGCAGCCCTAATTTACCAG
scaffold730779_15.6	1	p3	(AAT)8	24	967	990	CGGGATGTAGTAGGAAAGCG
scaffold730784_18.0	1	p3	(TGT)6	18	41	58	TGTAGCCGACCCCAATTTAT
scaffold730874_15.5	1	p3	(CCG)5	15	185	199	ACCTCATGAGCACAATTCCC
scaffold730879_14.3	1	p3	(TTG)6	18	154	171	GTTCGGAAAATTTTTGGCCT
scaffold730880_17.0	1	p3	(TCA)7	21	485	505	GGGCTTAAAACTTGCCACAA
scaffold730902_11.7	1	p3	(ATT)8	24	1576	1599	TCCCTTTTCAGAAGTCGCAT
scaffold730908_16.3	1	p3	(AAT)5	15	483	497	TGAATGAATGGTTCGACTCAA
scaffold731027_17.4	1	p3	(TTA)5	15	1687	1701	AACTGGACCATATTCGTCGC
scaffold731062_20.7	1	p3	(TTG)5	15	507	521	GCATCGGCCTATTTACCAAA
scaffold731067_15.6	1	p3	(TTA)5	15	907	921	ATTCTGGCAAATCATCGGAC
scaffold731099_13.7	1	p3	(CCT)5	15	531	545	CACCACCCTCTCATGTCCTT
scaffold731128_14.0	1	p3	(ATA)6	18	858	875	AGGAAATCACGACGGTGTTC
scaffold731151_14.3	1	p3	(ATC)5	15	1542	1556	CCCACACGTTTTTCCTCATT
scaffold731165_14.0	1	p3	(TCA)5	15	489	503	GCAACTTTCAAGGAAGCCAA
scaffold731171_15.6	1	p3	(GAG)5	15	636	650	ACAGGAATATCCCGTTGCAG
scaffold731239_14.3	1	p3	(AAT)5	15	48	62	GGTTCCCACGTCATCAAAAA
scaffold731259_17.2	1	p3	(ATC)5	15	3394	3408	TTGGCATCATTATGGAAGCA
scaffold731335_13.7	1	p3	(TTA)5	15	1025	1039	GGTGTTGATGCATGGAGTTG
scaffold731396_14.3	1	p3	(CAC)5	15	1158	1172	ATGGAGCTATTGGATGCTGG
scaffold731510_17.6	1	p3	(TTG)5	15	2152	2166	TTTTGGATTCGATATGGATTGA
scaffold731602_14.8	1	p3	(ATT)6	18	111	128	CCTCCAAACAAATACCCCCT
scaffold731623_16.6	1	p3	(ATT)5	15	1430	1444	CCATGAGATGGGGACAGTCT
scaffold731857_15.5	1	p3	(GAA)5	15	680	694	CAAGCAAACGCAAAAATCAA
scaffold731873_16.5	1	p3	(AAT)5	15	366	380	ATCCCACCAACACCTTCTTG

scaffold731918_16.[illegible]	1	p3	(TGT)5	15	867	881	GATTGGATTTTTGGTGGTGG
scaffold731958_14.[illegible]	1	p3	(TCC)5	15	557	571	AACTCCCACCTTCCATCACA
scaffold731967_14.[illegible]	1	p3	(TCT)7	21	304	324	TTCTCTCCCAACAATGACATCT
scaffold731979_16.[illegible]	1	p3	(TGT)7	21	32	52	CCAATTTATTGGGATTAAGGCT
scaffold731981_13.[illegible]	1	p3	(TAT)6	18	1393	1410	CCTCCATGTAAAGAAAAAGAGTTCA
scaffold731989_15.[illegible]	1	p3	(ATA)5	15	134	148	TGCCGAATAAAGCTAAATCCT
scaffold732030_16.[illegible]	1	p3	(GGC)5	15	71	85	ACCAGCTTTGCTCTGCAACT
scaffold732053_14.[illegible]	1	p3	(GAG)5	15	717	731	CGTCGAATTTCGCTGATCTT
scaffold732085_17.[illegible]	1	p3	(ATG)5	15	546	560	AGTCAAGTGCCGCTCATTTT
scaffold732102_15.[illegible]	1	p3	(TAT)5	15	164	178	TGACAATTCGAGCTTGATCCT
scaffold732116_16.[illegible]	1	p3	(ATG)5	15	942	956	TAGGCGATGTACCTTGGGTC
scaffold732126_16.[illegible]	1	p3	(GGA)5	15	930	944	TTGGAATGCGACAGAGACAG
scaffold732220_16.[illegible]	1	p3	(CTG)5	15	308	322	GACACCATTGACTGTGCTGG
scaffold732278_16.[illegible]	1	p3	(TGT)5	15	46	60	TGTAGCCGACCCCAATTTAT
scaffold732307_17.[illegible]	1	p3	(TAT)5	15	53	67	AGCAACAGGCACACTGGAAT
scaffold732308_10.[illegible]	1	p3	(TTA)5	15	811	825	ACCCAGAGCAAACTCGAAGA
scaffold732318_13.[illegible]	1	p3	(GCC)6	18	564	581	CAGTTGCATGCAGAGCATTT
scaffold732387_15.[illegible]	1	p3	(ATG)5	15	991	1005	CTCGAACGATGCAAACTTCA
scaffold732457_20.[illegible]	1	p3	(GGC)5	15	1082	1096	TGGAGGAGGAGGAATGTTTG
scaffold732523_16.[illegible]	1	p3	(AAT)5	15	348	362	GCCGGAAAACACAAATCAAT
scaffold732543_14.[illegible]	1	p3	(CTT)5	15	1006	1020	CTACCCTCGCTCTTCCTTCC
scaffold732548_16.[illegible]	1	p3	(GCC)5	15	151	165	AAAAATCTCCAGTCCGGCTT
scaffold732641_14.[illegible]	1	p3	(AGT)5	15	1277	1291	CAGCAAGCAATTCAAGGACA
scaffold732657_16.[illegible]	1	p3	(TGA)5	15	44	58	CCCAAGACCCCAATTTGATT
scaffold732659_12.[illegible]	1	p3	(CCG)6	18	571	588	TCTAAAATAGCCACGCCTCG
scaffold732672_15.[illegible]	1	p3	(GCT)5	15	902	916	GCAGGAGAAGAGGTGGTCTG
scaffold732679_14.[illegible]	1	p3	(ATG)5	15	641	655	GGACTCTTCCGGATGTTTGA
scaffold732703_17.[illegible]	1	p3	(ATT)5	15	115	129	AAGTGTGTCGAAATTGAGAAGAAA
scaffold732719_15.[illegible]	1	p3	(CAG)5	15	1194	1208	TGATTTGTGGCACCCTGTTA
scaffold732722_16.[illegible]	1	p3	(CTT)5	15	779	793	GACTGAGATGTCCCAGGTTCA
scaffold732727_15.[illegible]	1	p3	(TGT)8	24	39	62	TGTAGCCGACCCCAATTTAT
scaffold732740_14.[illegible]	1	p3	(ATC)5	15	1339	1353	CCCGACCCAACTCAGTAGAA
scaffold732789_16.[illegible]	1	p3	(TGT)5	15	47	61	TGTAGCCGACCCCAATTTAT
scaffold732863_14.[illegible]	1	p3	(ATT)6	18	4450	4467	GGAAACCCTAGTTTCGTGTTCTT
scaffold732871_15.[illegible]	1	p3	(CAC)5	15	688	702	TTCATGACGTGGAAGCAAAA
scaffold732934_15.[illegible]	1	p3	(GGA)5	15	177	191	CCATCTAGTTCCTCACCGGA
scaffold732952_16.[illegible]	1	p3	(ATC)5	15	1254	1268	TAAAACCCCCTTCAAATCCC
scaffold732960_16.[illegible]	1	p3	(AGA)5	15	1016	1030	TCATCTCAAGACCACCCTCC
scaffold732970_17.[illegible]	1	p3	(TTC)8	24	575	598	TGAAATGGGAAGGACTTCAT
scaffold733036_12.[illegible]	1	p3	(CGC)6	18	3869	3886	AAATCTCCCCCACCTCCATA
scaffold733040_14.[illegible]	1	p3	(GAA)5	15	1973	1987	AAAAGTGTCATGGCGAAACC
scaffold733053_14.[illegible]	1	p3	(AAG)6	18	659	676	CAAAGTGGTGGAGTGAGCAA
scaffold733085_16.[illegible]	1	p3	(AAT)6	18	109	126	TGCATATACACACATGTTGACCA
scaffold733132_16.[illegible]	1	p3	(ATT)5	15	580	594	GGCTGGGTAATAGCCTTTCC
scaffold733147_16.[illegible]	1	p3	(CTC)5	15	269	283	AGCTTAGCACAGCAGCATCA
scaffold733162_16.[illegible]	1	p3	(CCG)6	18	571	588	CCTCCTCGTCGTCTTCATCT
scaffold733222_14.[illegible]	1	p3	(ATA)5	15	299	313	AGATGGGCCGGCTAAATATC
scaffold733241_13.[illegible]	1	p3	(AAT)5	15	1139	1153	TCTTTGTGTAGCACCCCCTC
scaffold733292_16.[illegible]	1	p3	(CTT)5	15	1085	1099	AAGGAGGGTCAGTGAGCAAA
scaffold733318_13.[illegible]	1	p3	(GGT)6	18	383	400	AGGCATTGGATTCAGTGGTC
scaffold733322_17.[illegible]	1	p3	(TCA)6	18	3073	3090	CACGTCCTCAGGCCACTATT
scaffold733324_15.[illegible]	1	p3	(TTC)5	15	2030	2044	GAGGACTGGGCTGATCTTCA
scaffold733336_16.[illegible]	1	p3	(ATC)6	18	2479	2496	TTCTTCAAACTCCCACCGTT
scaffold733420_14.[illegible]	1	p3	(AAT)5	15	871	885	TGATGGAACTTGTGTGCATGT
scaffold733443_16.[illegible]	1	p3	(GTG)6	18	3044	3061	GCTCTCAGTGCATCCCAGAT
scaffold733452_17.[illegible]	1	p3	(ATC)5	15	596	610	TTCACACGCGAGAAGAGAGA
scaffold733476_16.[illegible]	1	p3	(TCT)5	15	837	851	AAGATAAAGGACGGCCGAGT
scaffold733535_16.[illegible]	1	p3	(ATG)5	15	598	612	GCTTCCAGCAGAAATCATCC

scaffold733551_15.8	1	p3	(CTT)5	15	2098	2112	AATGCAGTCCGGGTTCATAG
scaffold733688_16.2	1	p3	(TGT)5	15	46	60	ATGTAGCCGACCCCAATTTA
scaffold733737_15.0	1	p3	(TGA)6	18	43	60	CCCAAGACCCCAATTTGATT
scaffold733753_13.7	1	p3	(TGT)6	18	45	62	TGTAGCCGACCCCAATTTAT
scaffold733764_16.7	1	p3	(TTG)5	15	2331	2345	ACAGCTCGAGCATTCATTCC
scaffold733825_16.7	1	p3	(CGG)5	15	507	521	TTTCGTTGGGACTGGTTACG
scaffold733846_16.7	1	p3	(ACA)5	15	709	723	CATTCAATTCGAAATTATGCATGT
scaffold733892_13.9	1	p3	(ATT)7	21	817	837	TTGTGTGAACGAGGATTTGC
scaffold733993_16.7	1	p3	(CAG)5	15	316	330	TGCAACCTGCAAGTCACATT
scaffold734003_18.1	1	p3	(CGG)5	15	3663	3677	TAATGGGCCTTCACATCCTC
scaffold734029_18.9	1	p3	(GAG)5	15	697	711	GTTTTTGGACTAGCCCATCG
scaffold734045_12.3	1	p3	(ATA)7	21	415	435	TTCAAATCCGGGTCTCTCAC
scaffold734102_14.0	1	p3	(ACA)5	15	2347	2361	TTGTCGTGATGGGTGAATGT
scaffold734109_14.2	1	p3	(AAT)5	15	107	121	CGCATGACACTGATCTATCG
scaffold734128_16.0	1	p3	(TGA)6	18	44	61	CCCAAGACCCCAATTTGATT
scaffold734287_13.3	1	p3	(GAT)5	15	1606	1620	CCCCAACTCACATCAACACA
scaffold734312_17.7	1	p3	(ATA)5	15	5900	5914	TTCTTGTTTTCCCTCACTGGA
scaffold734377_19.2	1	p3	(AAC)5	15	1179	1193	TCCAGATTCAAGACCAAGCA
scaffold734421_15.0	1	p3	(GTC)5	15	1264	1278	ATTACCGATGGCCGATGTAG
scaffold734449_15.0	1	p3	(ATT)5	15	550	564	ACACCCCAGCATGAGGAATA
scaffold734496_16.6	1	p3	(ATA)6	18	1213	1230	TTGTATCGTGTTCGCCACAT
scaffold734506_15.6	1	p3	(ATC)7	21	3897	3917	TGGAACGATTGTGTGGAAAG
scaffold734522_12.0	1	p3	(TCA)5	15	631	645	GCCAGGATGCAGAACAATTT
scaffold734523_16.3	1	p3	(GGC)7	21	3258	3278	TTTTGGGTGGAGAAATGGAG
scaffold734552_15.1	1	p3	(TGT)5	15	51	65	ATGTAGCCGACCCCAATTTA
scaffold734557_16.9	1	p3	(ACA)5	15	583	597	CCCATGCCGAATGTTATTCT
scaffold734579_17.4	1	p3	(ATA)5	15	704	718	CATTATACCACGGAGGAAGGA
scaffold734599_14.8	1	p3	(CTC)5	15	354	368	CCCCACACACTCATCCTCTT
scaffold734607_16.3	1	p3	(CAT)5	15	453	467	GTTGGGTTGGATTTGAGCAT
scaffold734615_19.0	1	p3	(TTC)5	15	634	648	TGAAACATCCATTCTCCAAAGA
scaffold734647_15.2	1	p3	(AAC)6	18	3486	3503	TAGATAACAGGCAATGCCGC
scaffold734701_15.8	1	p3	(ACC)5	15	838	852	ACTCTTCAGCTCCCACTCCA
scaffold734715_12.1	1	p3	(TTA)6	18	696	713	GTTCCTAGCTGTCGGCACTC
scaffold734770_15.4	1	p3	(ATA)5	15	320	334	AAAAGAGGGGGAAAAATTCG
scaffold734792_15.0	1	p3	(TAC)5	15	1395	1409	CATATAAAGAGGGCAGCCGA
scaffold734798_12.3	1	p3	(AGA)6	18	1890	1907	CCTGATGAGGCTAAGGATGG
scaffold734809_16.0	1	p3	(ATA)5	15	906	920	CCGAAAATGTCCTTCATTGC
scaffold734812_14.5	1	p3	(ATT)5	15	831	845	TTGAGGACCGGACTGACTTC
scaffold734994_18.3	1	p3	(ATC)6	18	645	662	CAGCATGAGGCAGATTGATG
scaffold735098_16.0	1	p3	(GCG)7	21	363	383	CATGACTCCGCATTAGCAGA
scaffold735107_15.9	1	p3	(CAT)6	18	757	774	CCCCTTGGGATATGTGTTTG
scaffold735203_16.0	1	p3	(GGA)6	18	378	395	GAGATTTCGGAGCTTGTTGG
scaffold735237_16.5	1	p3	(TAA)5	15	3316	3330	TTGTCACTAAGCCAACACCAA
scaffold735266_18.8	1	p3	(ATT)5	15	1220	1234	GGAAATGATTGTTCTCCTAGGTC
scaffold735352_14.4	1	p3	(ATT)7	21	410	430	TGTTTGCTTTTCTTCATGCG
scaffold735359_17.6	1	p3	(CAT)7	21	3352	3372	GGCCTAAAGCTGATGGACCT
scaffold735371_14.0	1	p3	(GCG)8	24	2065	2088	CTTTGCTTCGTGCTTGAATG
scaffold735376_16.6	1	p3	(CTG)5	15	1471	1485	AGGGGACTCGTCAGGAAACT
scaffold735412_12.2	1	p3	(ATT)8	24	202	225	TCTTTTTCCATTGCTCACGA
scaffold735525_14.6	1	p3	(ACA)5	15	3192	3206	ACTGCATCGGTGTCTCACAT
scaffold735556_14.8	1	p3	(CGC)5	15	316	330	GAAGGCAGCTTGATCGTAGG
scaffold735591_19.3	1	p3	(AAT)5	15	827	841	GGAGGAAGCATCTACGCAAC
scaffold735606_16.8	1	p3	(TTC)5	15	670	684	ACGCTGTTTTTCCACTCCAG
scaffold735788_16.2	1	p3	(CTA)5	15	167	181	CAATAACGGCAACACAAACG
scaffold735797_16.4	1	p3	(CCG)5	15	1078	1092	TGCACGATCCATATCTGCTG
scaffold735864_16.3	1	p3	(TCA)6	18	3425	3442	TGATGGAATCTCTGGGAAGG
scaffold735875_15.3	1	p3	(AAG)5	15	1457	1471	AACAAAATCCCTCCCCATTC
scaffold735876_17.6	1	p3	(ATA)5	15	1382	1396	TGCCTTAACACAAAGGGGAA

scaffold735908_17.[illegible]	1	p3	(TTG)5	15	49	63	AGCTTTGGTTCATGTAGCCG
scaffold735952_16.[illegible]	1	p3	(TCC)5	15	2417	2431	CCGATGAGTCGTTTCCACTT
scaffold735980_13.[illegible]	1	p3	(CTT)7	21	427	447	TGAAACTGGACCTGGTATCAA
scaffold735983_16.[illegible]	1	p3	(TTA)7	21	1049	1069	TGTAAAGAAACGGACGGAGG
scaffold735996_14.[illegible]	1	p3	(TTC)5	15	623	637	TGGCACCCTTACATCTCCTT
scaffold736066_16.[illegible]	1	p3	(ATA)6	18	5779	5796	TTCGGAGGACAATGCTCTCT
scaffold736077_14.[illegible]	1	p3	(CAA)6	18	5519	5536	GGGACAAGCTATCACACAGGA
scaffold736097_18.[illegible]	1	p3	(TGA)5	15	359	373	GTTGCGTACATATGACCCCC
scaffold736191_14.[illegible]	1	p3	(CCT)6	18	1121	1138	GTTCCACCCCCTGAGGTATT
scaffold736194_17.[illegible]	1	p3	(TCA)5	15	652	666	CTCAAGGAGCTCAACTCCAA
scaffold736219_14.[illegible]	1	p3	(ATG)5	15	1855	1869	GGCTTATGTCCAACTGCTCC
scaffold736222_13.[illegible]	1	p3	(AAG)8	24	86	109	GCACTCGAAGAGTAGCGGTT
scaffold736230_17.[illegible]	1	p3	(TGG)5	15	1181	1195	TCTGGAAAAGAGAAGGCCAA
scaffold736335_15.[illegible]	1	p3	(GGT)5	15	102	116	TGTGGGAAAAACATCGATCA
scaffold736366_17.[illegible]	1	p3	(GAC)5	15	1467	1481	TCGGCAACTTTTGGAAGATT
scaffold736370_15.[illegible]	1	p3	(TTA)5	15	777	791	TTTCTCGCCTAATGCCATGT
scaffold736407_15.[illegible]	1	p3	(ATT)7	21	1446	1466	CGCGAACTAATCCGTCCTTA
scaffold736421_14.[illegible]	1	p3	(AAT)5	15	3134	3148	CCCTCAATTTTGTACCCGAA
scaffold736446_17.[illegible]	1	p3	(TGA)6	18	45	62	CCCAAGACCCCAATTTGATT
scaffold736448_14.[illegible]	1	p3	(TAA)6	18	403	420	GCCAAAAGTAAAGGAAGGGG
scaffold736470_18.[illegible]	1	p3	(AAC)6	18	2057	2074	TCAAAAGTCATCCCCTGGAG
scaffold736473_13.[illegible]	1	p3	(GGT)6	18	1201	1218	CTGATCACCCAGTTTTGCCT
scaffold736519_16.[illegible]	1	p3	(CCG)6	18	927	944	CTCCAATCTCTCTCCCACCA
scaffold736531_16.[illegible]	1	p3	(CAG)5	15	2314	2328	AGCATTGGCTTCAGCTGTTT
scaffold736549_14.[illegible]	1	p3	(TGA)5	15	41	55	CCCAAGACCCCAATTTGATT
scaffold736560_11.[illegible]	1	p3	(GCT)5	15	1150	1164	ATCCTCAGATTTACCGTGCC
scaffold736582_16.[illegible]	1	p3	(GGT)7	21	1219	1239	TCTGGAAAAGAGAAGGCCAA
scaffold736622_11.[illegible]	1	p3	(ATG)6	18	1139	1156	TTGCTTGACTGAGTTGCTGC
scaffold736630_16.[illegible]	1	p3	(CAC)5	15	1230	1244	TACATCACCCTCCCATCTGC
scaffold736676_13.[illegible]	1	p3	(CCG)5	15	720	734	TACTTCATCGTGCTCAACGG
scaffold736742_15.[illegible]	1	p3	(TCT)5	15	320	334	TCCTCCAGCTGTTCTTTCGT
scaffold736777_14.[illegible]	1	p3	(CGG)5	15	125	139	TGAACAGCATGGTCTGGATG
scaffold736799_15.[illegible]	1	p3	(TGA)5	15	2384	2398	GATAAGGGGATTGATTGGCA
scaffold736809_16.[illegible]	1	p3	(CAA)5	15	1213	1227	GAAGCCACGAGAGAATTTGC
scaffold736831_13.[illegible]	1	p3	(TAA)9	27	1231	1257	TGCCCTCACAAACAAATTCA
scaffold736875_16.[illegible]	1	p3	(GTA)6	18	2097	2114	CGAGACCTAAATTGGTTGTGG
scaffold736905_15.[illegible]	1	p3	(ACA)5	15	699	713	AAAAATCATTTATCCCCGGC
scaffold736926_16.[illegible]	1	p3	(CAT)5	15	1909	1923	TGCTGATCCACCCTTTGAAT
scaffold736960_12.[illegible]	1	p3	(ATA)7	21	590	610	GCAGCTGAGCTAGGTGCATT
scaffold736973_16.[illegible]	1	p3	(TAT)6	18	629	646	GCAATTTTCAAGGTTAGGCAA
scaffold737052_14.[illegible]	1	p3	(AGA)5	15	2620	2634	ATCTCACTCGGACATGGGAC
scaffold737068_14.[illegible]	1	p3	(CTC)7	21	2835	2855	TTGGTTCAATCTCAAAGCCC
scaffold737079_14.[illegible]	1	p3	(TCT)7	21	2378	2398	AAGGTAGTACCAGCCTGCCA
scaffold737111_15.[illegible]	1	p3	(CAT)6	18	697	714	CCACGCCATAACAAAGGACT
scaffold737119_12.[illegible]	1	p3	(TGT)5	15	45	59	TGTAGCCGACCCCAATTTAT
scaffold737133_14.[illegible]	1	p3	(CAG)6	18	443	460	TCGGCATGTTGCTCTTTATG
scaffold737227_13.[illegible]	1	p3	(AAT)5	15	1120	1134	GCGCTGAAAGAGGATGAGAT
scaffold737250_16.[illegible]	1	p3	(CGG)7	21	2605	2625	GTCCTCCTCGTCATCGTCAT
scaffold737290_16.[illegible]	1	p3	(TAT)5	15	637	651	CGACAGCACATGACTTTTCA
scaffold737303_17.[illegible]	1	p3	(ATA)6	18	318	335	TGCAAGATGTGCCCCTATTT
scaffold737344_15.[illegible]	1	p3	(CGA)7	21	513	533	GGACTATCCACCAACTCCGA
scaffold737357_14.[illegible]	1	p3	(TGA)5	15	2030	2044	GTGCAGGCTTGCTTTTGTTA
scaffold737372_16.[illegible]	1	p3	(AAT)5	15	256	270	AATAAGATTTGCACGCTGGG
scaffold737459_13.[illegible]	1	p3	(AAT)5	15	1049	1063	CGTTGCTTGGCAATTCTACA
scaffold737468_16.[illegible]	1	p3	(GGC)5	15	320	334	ACTCAGATCTGGCCTGGAGA
scaffold737477_15.[illegible]	1	p3	(ATA)5	15	40	54	TGGTAGATCTGCTCCTTGTAGTGA
scaffold737518_18.[illegible]	1	p3	(TTG)5	15	97	111	CTGAAAGGGAGTTGGAAGCA
scaffold737596_19.[illegible]	1	p3	(TGA)7	21	40	60	CCCAAGACCCCAATTTGATT

scaffold737616_18.5	1	p3	(GCG)5	15	1166	1180	ACGCCAATTTGGATTTTGAC
scaffold737617_16.8	1	p3	(TTA)5	15	51	65	GGTCCTTCGTCGCATATGAA
scaffold737621_16.5	1	p3	(ACA)5	15	5149	5163	AATGTCATATACCCGCCCAA
scaffold737755_14.0	1	p3	(CTG)5	15	149	163	GGTGGTGAGAACCACGACTT
scaffold737764_17.0	1	p3	(CTT)5	15	279	293	CCATCGGAAGTCTCAAGTCC
scaffold737782_15.9	1	p3	(TGT)5	15	50	64	ATGTAGCCGACCCCAATTTA
scaffold737783_16.7	1	p3	(GAA)5	15	1874	1888	GGGAGCCCTTGGATTTAGTT
scaffold737806_14.6	1	p3	(ATT)5	15	96	110	TTGGGATTAAGGCTTTGGTG
scaffold737841_15.8	1	p3	(TTG)5	15	533	547	GCACTTACAGCCAAGCAACA
scaffold737846_17.3	1	p3	(ATC)6	18	742	759	TTGTGGGATAAAATCCCCTT
scaffold737861_10.4	1	p3	(TCA)8	24	679	702	GAGTGTTCGCGCAGTTGATA
scaffold737876_16.4	1	p3	(AGC)5	15	2261	2275	CAGTGCATGCAAGCTGATTT
scaffold737895_16.4	1	p3	(TGA)5	15	1628	1642	GGATTCGAGCTCTTGTTTGC
scaffold737897_17.0	1	p3	(TGT)5	15	50	64	TTGGTTTATGTAGCCGACCC
scaffold737930_19.3	1	p3	(GAA)6	18	459	476	CCGATAAGCAGAGCTCCATC
scaffold738028_17.0	1	p3	(ATA)5	15	497	511	TGGTGAAAAATCAAACACTTGG
scaffold738078_16.6	1	p3	(GAA)5	15	1425	1439	GGGCTCCTCATGGTCTGATA
scaffold738083_15.3	1	p3	(ATT)5	15	1706	1720	TGCCTTTCAAATAGCATTCGT
scaffold738098_16.7	1	p3	(GCC)6	18	673	690	CTTCTTCCTCCGACGACAAC
scaffold738101_15.7	1	p3	(AGT)5	15	473	487	ATGCTTCTCCCCCTTGAAAT
scaffold738147_15.3	1	p3	(CTT)6	18	1784	1801	TCAGTTATAGCCGACCCCAC
scaffold738222_15.6	1	p3	(CAT)5	15	1923	1937	AAGTCGTGCATTGACGTGAC
scaffold738228_15.4	1	p3	(ATG)5	15	4927	4941	TCAACAGACATTGCAGCCTC
scaffold738278_13.5	1	p3	(TTA)5	15	1536	1550	TGGAACAAACAAACAAGCACA
scaffold738313_18.5	1	p3	(CAG)5	15	1130	1144	ATGCCGTTCTTTTGTTCTGG
scaffold738324_15.7	1	p3	(CAC)5	15	4550	4564	CCCATTTTGACGCTCTGATT
scaffold738341_15.5	1	p3	(TGG)5	15	615	629	GATTGATTCCGGTAGGCAAA
scaffold738352_15.6	1	p3	(TGT)5	15	4739	4753	CACCTATTCGACCCTGCAAT
scaffold738386_17.3	1	p3	(TCA)5	15	1658	1672	GCATAACCCGAACCCAATAA
scaffold738394_16.7	1	p3	(AGA)5	15	822	836	GGGAAGATTCGTGTTTCAGG
scaffold738407_14.6	1	p3	(TAA)6	18	5979	5996	TGACAGATGAATGGACCAACA
scaffold738410_13.8	1	p3	(TGA)6	18	43	60	CCCAAGACCCCAATTTGATT
scaffold738416_15.7	1	p3	(GCT)5	15	3801	3815	CATTAGCTCCGTGATGCAGA
scaffold738439_16.2	1	p3	(GGC)6	18	1826	1843	GACCCGGAAAGGAAATTTTG
scaffold738452_16.7	1	p3	(CGC)7	21	497	517	TATCCAATATAGGGCACGGC
scaffold738483_18.2	1	p3	(TAA)6	18	1621	1638	CGTTGATTCTTCAAAATCCCA
scaffold738486_15.3	1	p3	(CAC)5	15	4771	4785	GTATGTGGTAAGGCGAGGGA
scaffold738698_18.9	1	p3	(AAT)5	15	594	608	GCCCGTTTCTGAAAGACTGA
scaffold738712_15.0	1	p3	(GAT)6	18	844	861	ACGAAGATACAGTCCCACCG
scaffold738720_10.5	1	p3	(AAG)5	15	433	447	ACATTTCAACCGCAGGTAGC
scaffold738721_17.8	1	p3	(CGA)5	15	380	394	TACCTCCGAAGGAACGAGAA
scaffold738727_15.2	1	p3	(TGA)6	18	44	61	CCCAAGACCCCAATTTGATT
scaffold738754_16.0	1	p3	(CAA)5	15	1860	1874	CCATCACCATCACTTCCAGA
scaffold738837_17.0	1	p3	(AAC)5	15	429	443	TGAATGCTTTATCCGAGCCT
scaffold738838_17.1	1	p3	(TGA)5	15	48	62	CCCAAGACCCCAATTTGATT
scaffold738847_12.0	1	p3	(TCC)5	15	1499	1513	TCCCTCCTCCTCCTCTTCTC
scaffold738933_14.4	1	p3	(GAC)5	15	1337	1351	CCTCCCTCTAAAAACCCGAA
scaffold738943_15.1	1	p3	(AAC)6	18	65	82	TCAGCTTCATGATACTTTGGGA
scaffold739014_15.6	1	p3	(AAT)5	15	1764	1778	GCGTATTATTTTTCTCTCAATAGGG
scaffold739017_12.7	1	p3	(CTC)5	15	123	137	TGATCATCCTCCTCATTCTCTG
scaffold739030_18.3	1	p3	(TCT)6	18	963	980	CTCAGCTATGAAATCCGCGT
scaffold739057_15.3	1	p3	(GAT)5	15	2006	2020	TCAACACTGCAGATGAACAGAA
scaffold739072_15.6	1	p3	(CTT)5	15	1980	1994	GGAAGCTTCAAGTCCAATGC
scaffold739101_15.1	1	p3	(TTC)5	15	1975	1989	TTTCCGCCATTTTTAGCATC
scaffold739138_16.4	1	p3	(GGA)5	15	927	941	GCGGAGAGGAAGAGGAAGAA
scaffold739165_16.2	1	p3	(ACA)6	18	2136	2153	TTAGATTGGAGATGCCGAGG
scaffold739181_16.0	1	p3	(ATC)5	15	2712	2726	AAAGAAGCAACCATCAACCG
scaffold739190_15.9	1	p3	(ATA)5	15	999	1013	CTGCAGAATCAACTGCTTGG

scaffold739223_16.8	1	p3	(TGT)6	18	47	64	TTGGTTCATGTAGCCGACCT
scaffold739249_15.8	1	p3	(TTA)5	15	5158	5172	TCGTCCATATCAAGGCTCAA
scaffold739259_14.3	1	p3	(TAT)5	15	431	445	AAAATGGATGGGGTGATTGA
scaffold739268_17.7	1	p3	(ACA)7	21	3113	3133	ACACCCGGACACAAGAATGT
scaffold739299_16.2	1	p3	(TGG)5	15	5372	5386	GAGTATTCTTGCCGTTGGGA
scaffold739307_15.2	1	p3	(TGT)5	15	48	62	TGTAGCCGACCCCAATTTAT
scaffold739332_14.4	1	p3	(CGC)5	15	1580	1594	TGGTGAACCCAAACTCTTCC
scaffold739367_18.8	1	p3	(GCG)5	15	1772	1786	GGAGGAAATTGGCATCAGAA
scaffold739457_14.7	1	p3	(AGG)7	21	30	50	AAACAGCTTCTCTCTCCGGG
scaffold739460_14.3	1	p3	(CTC)5	15	878	892	AAAATAATGATAACGGCGGC
scaffold739464_18.0	1	p3	(AAG)6	18	695	712	TGGTGAGCTTGAGGAGGTCT
scaffold739514_17.6	1	p3	(TGT)6	18	44	61	ATGTAGCCGACCCCAATTTA
scaffold739534_15.2	1	p3	(TGA)7	21	45	65	CCGAGACCCCAATTTGATT
scaffold739542_13.5	1	p3	(TGT)5	15	68	82	ATGTAGCCGACCCCAATAAA
scaffold739596_15.5	1	p3	(TGT)6	18	42	59	TGTAGCCGACCCCAATTTAT
scaffold739600_16.3	1	p3	(TGT)7	21	41	61	TGTAGCCGACCCCAATTTAT
scaffold739613_17.2	1	p3	(ATC)7	21	3054	3074	AAACCATCTTTCCCCAAACC
scaffold739631_14.1	1	p3	(TTG)6	18	3284	3301	CCCAGTTTTTGTTGCACTGA
scaffold739678_15.7	1	p3	(TTA)5	15	734	748	AGTGAGTTGATTCGGGTTCG
scaffold739704_16.3	1	p3	(ATA)5	15	146	160	CCCACATCCAGCTTTCGTAT
scaffold739713_15.6	1	p3	(CAA)5	15	872	886	CAGTTTCAAGGGGAATCGAA
scaffold739714_17.0	1	p3	(TGA)10	30	3779	3808	GTGAGGATGATGGTGGAGGT
scaffold739733_16.6	1	p3	(TCC)5	15	2006	2020	AATTACCTGTTGGCCTGCAT
scaffold739753_15.4	1	p3	(CAT)7	21	5898	5918	GAAATCGGAATCCCCGTACT
scaffold739861_15.5	1	p3	(GGA)6	18	642	659	GTTCTCCTTCGGAGTTGCAG
scaffold739881_15.6	1	p3	(TTG)5	15	2151	2165	AGCCGACTTCACATAGTGGG
scaffold739896_17.2	1	p3	(GCC)5	15	1253	1267	ACGTTGTTGTTGATTGGGGT
scaffold739916_18.2	1	p3	(GAA)5	15	960	974	AAATACAAAGCCAGCCAACG
scaffold739960_15.6	1	p3	(TGT)5	15	47	61	ATGTAGCCGACCCCAATTTA
scaffold739977_14.5	1	p3	(TAA)6	18	80	97	ACGTGTATTGGGTGGTCCAT
scaffold740019_13.8	1	p3	(ATA)5	15	2284	2298	GCAGAAGGCTCCACTCTTGT
scaffold740035_13.9	1	p3	(CAT)5	15	1620	1634	AACTTTTGCCTGAAACGCAT
scaffold740153_17.1	1	p3	(CGG)5	15	758	772	ACCATGAAGATGAAGCTGCC
scaffold740170_15.7	1	p3	(CAC)5	15	703	717	TCGTTTTCTTTCTCGGTGCT
scaffold740175_15.2	1	p3	(TTG)5	15	51	65	TTGGGATTAAGGCTTTGGTG
scaffold740177_13.6	1	p3	(TTA)5	15	2268	2282	ATTTCTCATACGTGCCTGCC
scaffold740198_15.7	1	p3	(CTG)6	18	1117	1134	ATGTTGATAGGGTTGGACGC
scaffold740213_15.4	1	p3	(CAT)6	18	2858	2875	GATGCGGGGGATTTAAAGTT
scaffold740254_16.7	1	p3	(CAT)5	15	332	346	ATCATCACCGTGGATCATCA
scaffold740330_15.7	1	p3	(AGC)6	18	3834	3851	ACTCTTACGAGGGACGGGTT
scaffold740341_15.8	1	p3	(ATT)7	21	150	170	AATAAAGTATGTGGGAAAGTCGAT
scaffold740428_15.0	1	p3	(ATG)6	18	265	282	GTGAAACACCATCTTTCCCG
scaffold740432_16.1	1	p3	(TGT)5	15	45	59	TGTAGCCGACCCCAATTTAT
scaffold740445_15.1	1	p3	(TGA)6	18	291	308	CGATTAGGGCTGTTATCGGA
scaffold740478_12.6	1	p3	(TAT)5	15	262	276	GATTCCCTATCTTGCTCCCC
scaffold740500_15.6	1	p3	(GAT)5	15	3629	3643	ATGCAACTTGCCAACCTACC
scaffold740505_15.8	1	p3	(CCG)6	18	1836	1853	CAAGCCACTCAAAATCAGCA
scaffold740509_15.6	1	p3	(CGG)5	15	3265	3279	AAATGCCGTTGGTGAGGTAG
scaffold740549_16.3	1	p3	(TGA)5	15	3839	3853	TTGCGTACATTTGATTGGGA
scaffold740562_16.2	1	p3	(GGC)5	15	2685	2699	TTTTGCATGTTCAGAGGCAG
scaffold740671_16.1	1	p3	(AAG)5	15	1795	1809	TTAGGCTCTCCAAAGGACGA
scaffold740673_14.5	1	p3	(TCC)6	18	608	625	CATTTCGACCTCCACATCCT
scaffold740687_14.5	1	p3	(GAA)5	15	3788	3802	AGAGAAGTTGTGGGGTGGTG
scaffold740688_16.3	1	p3	(TTG)7	21	2787	2807	AAATCTGTTCCAATTCCGGG
scaffold740704_14.6	1	p3	(GAT)5	15	736	750	TGTGATCAATGAAGGGGACA
scaffold740712_15.7	1	p3	(ACA)6	18	4039	4056	CGGCTGTTACTCCTTCCAAA
scaffold740714_16.1	1	p3	(CAT)6	18	598	615	AGGCTGAAAAGGATGCTCAA
scaffold740744_14.4	1	p3	(CTC)5	15	47	61	GTGTAAGTCGCGGTCTCGAT

scaffold740786_14.[illegible]	1	p3	(CTT)5	15	1313	1327	TCCATCAACCTAACAGCAACA
scaffold740790_18.[illegible]	1	p3	(AGA)5	15	263	277	AGGTGGCAACGAAAATGAAC
scaffold740802_14.[illegible]	1	p3	(GGA)5	15	842	856	GACGGAGATCCCAATGAAGA
scaffold740811_15.[illegible]	1	p3	(TGT)7	21	41	61	TGTAGCCGACCCCAATTTAT
scaffold740824_14.[illegible]	1	p3	(AGA)5	15	2863	2877	CTCATTGACGACATCCAACG
scaffold740832_18.[illegible]	1	p3	(AAC)5	15	3422	3436	AGCGGGATAATGGAACAGTG
scaffold740833_15.[illegible]	1	p3	(TTA)5	15	753	767	CTGCCTGCAAAAATATCCGT
scaffold740873_16.[illegible]	1	p3	(GAT)5	15	571	585	TGCAACTTGAGGCTGAGAAA
scaffold740884_15.[illegible]	1	p3	(TTG)5	15	173	187	GTTTATGTAGCCGACCCCAA
scaffold740885_16.[illegible]	1	p3	(ACA)5	15	1600	1614	GATGCTTAGTTGCCCATTCG
scaffold740977_14.[illegible]	1	p3	(CTT)5	15	1365	1379	TCAGAGCTATTTGCTTCACCA
scaffold740985_11.[illegible]	1	p3	(GCT)8	24	868	891	GAAGGAGGGGATGGAGAGAG
scaffold741038_14.[illegible]	1	p3	(TGT)7	21	3070	3090	AGTGTTACGCACTCATTGCC
scaffold741071_16.[illegible]	1	p3	(AAT)5	15	607	621	CGATAACGTGAGTCGTGACC
scaffold741078_16.[illegible]	1	p3	(GCG)5	15	2381	2395	TACTTCGCCGTAGGTTTCGT
scaffold741092_13.[illegible]	1	p3	(ATT)7	21	342	362	CGCCACTTGTTGGGTATTTT
scaffold741094_16.[illegible]	1	p3	(TTA)6	18	1897	1914	CAACTGCACTTGGATCCTGA
scaffold741111_16.[illegible]	1	p3	(CGG)5	15	536	550	TCTGGGGAATTTTCCTTGAA
scaffold741114_15.[illegible]	1	p3	(CAT)6	18	1421	1438	TCTCCTACCCAAAACTCCCA
scaffold741117_17.[illegible]	1	p3	(GCA)6	18	373	390	TCTGAAGCAGCTCCTGTGAA
scaffold741208_19.[illegible]	1	p3	(ACA)5	15	2078	2092	ACAAATGCAGGGAGGTCAAC
scaffold741217_15.[illegible]	1	p3	(ATC)6	18	1200	1217	GGGGCCCTGTCTAAAAATTG
scaffold741243_17.[illegible]	1	p3	(ATG)6	18	1806	1823	TACATCTGACCCCCAAGACC
scaffold741246_15.[illegible]	1	p3	(GCG)5	15	645	659	GCACGTAAAACCACCGAGAT
scaffold741304_16.[illegible]	1	p3	(GAA)5	15	201	215	CTACCCACAACAGCATGACG
scaffold741311_16.[illegible]	1	p3	(TGT)6	18	43	60	ATGTAGCCGACCCCAATTTA
scaffold741320_16.[illegible]	1	p3	(TGA)5	15	49	63	CCCAAGACCCCAATTTGATT
scaffold741329_16.[illegible]	1	p3	(TGT)6	18	45	62	TGTAGCCGACCCCAATTTAT
scaffold741335_14.[illegible]	1	p3	(ATA)5	15	322	336	GAAAGGAAAGAGCCGTCCTC
scaffold741347_13.[illegible]	1	p3	(ATC)5	15	726	740	TCACCATCATTTTCTTGCCA
scaffold741381_16.[illegible]	1	p3	(CAC)6	18	1046	1063	CACCATTGTTGGCATCAGAC
scaffold741423_15.[illegible]	1	p3	(AAT)5	15	425	439	ATTGGTATGCGCAACAATGA
scaffold741536_16.[illegible]	1	p3	(CAT)5	15	5658	5672	TGTGGTCTCATAACAAACCTGA
scaffold741541_16.[illegible]	1	p3	(ACA)5	15	2475	2489	TTGGTTGAGCGAGGAATACA
scaffold741588_14.[illegible]	1	p3	(TTC)5	15	3267	3281	TTCATCTCCCGTGTCCTTTC
scaffold741594_16.[illegible]	1	p3	(ATT)6	18	95	112	AAAAGTGGCATTTTGCCCTA
scaffold741610_15.[illegible]	1	p3	(ATG)6	18	2685	2702	TGTGAATCCCAAATGCTTCA
scaffold741613_17.[illegible]	1	p3	(GTA)5	15	59	73	TTTTGGCATGAGAAACTCCC
scaffold741639_19.[illegible]	1	p3	(CAG)5	15	1022	1036	AAGGCGAATTCTTGGGAACT
scaffold741650_16.[illegible]	1	p3	(TCA)6	18	1722	1739	TGTTGACGACGGAACTGAAG
scaffold741675_16.[illegible]	1	p3	(CAA)5	15	3083	3097	GCAAGACAAACAAAGTGCCA
scaffold741677_14.[illegible]	1	p3	(TTA)8	24	43	66	TTATAAAACCGACCCACCCC
scaffold741702_17.[illegible]	1	p3	(GAG)5	15	847	861	TCTTCTTGCATGTCTCGTCG
scaffold741711_17.[illegible]	1	p3	(TCT)5	15	138	152	CGTTTTCTTATCTCTGCGCC
scaffold741748_13.[illegible]	1	p3	(TAA)6	18	542	559	GCATTTTCCTCAAAAAGGGA
scaffold741753_14.[illegible]	1	p3	(AAT)6	18	69	86	GGACGGAGTTGTGAAGGAAT
scaffold741763_15.[illegible]	1	p3	(CAT)5	15	49	63	CAATAGTCTAAGGGAGCACATGG
scaffold741790_16.[illegible]	1	p3	(TGT)6	18	44	61	TGTAGCCGACCCCAATTTAT
scaffold741791_17.[illegible]	1	p3	(TCT)6	18	195	212	TTCTTCCACCAATTACCCCA
scaffold741805_16.[illegible]	1	p3	(ATC)5	15	600	614	ACCGCGTTATTATTTGCAGG
scaffold741806_15.[illegible]	1	p3	(TGA)5	15	659	673	GCACACTCGAACAAAGCAAA
scaffold741820_12.[illegible]	1	p3	(TCC)6	18	2578	2595	TTTTTCACGCACAACACACA
scaffold741824_19.[illegible]	1	p3	(CTC)5	15	585	599	TTCCTCTCTCTCTCCCTCCC
scaffold741830_19.[illegible]	1	p3	(CTC)5	15	737	751	TGCAGTAAATCTGTGACCCG
scaffold741933_16.[illegible]	1	p3	(TGG)5	15	2678	2692	AATTCTCACCCCCGGTATTC
scaffold741955_18.[illegible]	1	p3	(TTA)6	18	1369	1386	TCCGTAAGAGCATTTTGGCT
scaffold741960_15.[illegible]	1	p3	(CCG)6	18	1825	1842	GAGGTGGTCGGTTGGTAAGA
scaffold741977_16.[illegible]	1	p3	(CAT)5	15	3275	3289	AGGCTCAGTGGTCTCTTGGA

scaffold741985_18.[illegible]	1	p3	(CCG)7	21	1270	1290	GTAAACGACGGTTAGCTGGC
scaffold742014_12.[illegible]	1	p3	(TAT)6	18	1719	1736	TTTCGATGTCGAATTACAACAAA
scaffold742051_15.[illegible]	1	p3	(TTA)5	15	1256	1270	TCGATTTTATCCCTCGCAAA
scaffold742055_16.[illegible]	1	p3	(ATT)6	18	470	487	GACATCATCCACCGAGAACC
scaffold742067_17.[illegible]	1	p3	(TGT)6	18	1476	1493	AAGGGAGGGAATGAAGTGGT
scaffold742069_16.[illegible]	1	p3	(GAG)5	15	2125	2139	CAAACGAAGGAGGAGGATGA
scaffold742072_15.[illegible]	1	p3	(AGA)5	15	1427	1441	CCCAAAATAACGGACCTGAA
scaffold742074_17.[illegible]	1	p3	(AAC)5	15	2043	2057	AACCAAACAAGCCATTCCAG
scaffold742081_16.[illegible]	1	p3	(AAC)6	18	2233	2250	GGGCATTACCTTTGCATCAC
scaffold742084_15.[illegible]	1	p3	(TTG)5	15	48	62	TGTAGCCGACCCCAATTTAT
scaffold742106_18.[illegible]	1	p3	(GGT)5	15	3533	3547	TGTAAGCTCACCAGCATTCG
scaffold742108_14.[illegible]	1	p3	(TTC)5	15	278	292	CCAGCTTCAGGTTCTCTGTTG
scaffold742114_15.[illegible]	1	p3	(GGA)5	15	2828	2842	ATTGACTCCCACCAGGAGTG
scaffold742125_17.[illegible]	1	p3	(CCG)5	15	1550	1564	CCACTTATTTACTTCCGCCG
scaffold742167_14.[illegible]	1	p3	(CTC)6	18	395	412	CCCGAAGCGAATTTATTTCA
scaffold742200_14.[illegible]	1	p3	(GCC)5	15	3772	3786	AGTTGGGAAGGTAATTGGGG
scaffold742219_16.[illegible]	1	p3	(TGC)5	15	2473	2487	TCGAACCCGTGACATGTTTA
scaffold742226_18.[illegible]	1	p3	(TAA)6	18	426	443	TACATCGCACAAAATCACGG
scaffold742264_16.[illegible]	1	p3	(ATA)5	15	2762	2776	TTATTTTGGGATTCACCCATT
scaffold742298_15.[illegible]	1	p3	(TTA)6	18	2818	2835	CGTTAAATGCCACTGTGTTGA
scaffold742314_17.[illegible]	1	p3	(TGA)5	15	49	63	CCCAAGACCCCAATTTGATT
scaffold742335_17.[illegible]	1	p3	(TGT)5	15	49	63	TTGGGATTAAGGCTTTGGTG
scaffold742347_14.[illegible]	1	p3	(ATT)5	15	267	281	AGCAAAAGAAGAGGTTGCCA
scaffold742362_15.[illegible]	1	p3	(AAT)7	21	1453	1473	AAGAGGTGCATTTCAATCGG
scaffold742372_13.[illegible]	1	p3	(ACA)5	15	4592	4606	GTTTACTTCCATTCCGCCAA
scaffold742403_16.[illegible]	1	p3	(AGA)6	18	3195	3212	AGCCAAGATATCCGATGCAC
scaffold742408_15.[illegible]	1	p3	(TGT)6	18	1252	1269	ATCAATGGGCATTGGATGAT
scaffold742430_15.[illegible]	1	p3	(AAT)5	15	2675	2689	GCCGTTAAAGCGTGAAGACT
scaffold742440_17.[illegible]	1	p3	(TAT)6	18	1246	1263	TGGATGACCGAACTCTGTTTC
scaffold742450_17.[illegible]	1	p3	(GCT)6	18	617	634	TTTTCGTCACCTTTTGAGGC
scaffold742462_16.[illegible]	1	p3	(TGT)5	15	45	59	TGTAGCCGACCCCAATTTAT
scaffold742463_14.[illegible]	1	p3	(TAA)7	21	1119	1139	CTTGAAATTTTTGTGTGGCAA
scaffold742466_13.[illegible]	1	p3	(CAA)7	21	3483	3503	TCACAGATCCACAGGTTCCA
scaffold742500_14.[illegible]	1	p3	(TGT)8	24	2896	2919	AAAAAGTCGTGACCAAACAAAA
scaffold742506_14.[illegible]	1	p3	(CAG)5	15	277	291	ACCAGCAATCTGGTGGATTC
scaffold742513_14.[illegible]	1	p3	(TTC)5	15	358	372	TACCTTCTCCGGAATCATGC
scaffold742522_14.[illegible]	1	p3	(ATT)5	15	918	932	TCAATGGTTGGGCTACACTTC
scaffold742525_15.[illegible]	1	p3	(TAA)5	15	694	708	GGCCTTAATTGACCCAACCT
scaffold742528_15.[illegible]	1	p3	(TGA)5	15	47	61	CCCAAGACCCCAATTTGATT
scaffold742563_16.[illegible]	1	p3	(TTG)6	18	5273	5290	AACGTAAAGAGGGGTAGGGG
scaffold742607_15.[illegible]	1	p3	(ATT)7	21	169	189	AGCCAGGTAAACACCAATGC
scaffold742609_14.[illegible]	1	p3	(TAA)5	15	2848	2862	TCTCAGAGATAGTGCCAAGGTG
scaffold742618_17.[illegible]	1	p3	(CAT)6	18	2600	2617	GAAAATGGGGTGGTTGATTG
scaffold742633_16.[illegible]	1	p3	(CAA)5	15	1714	1728	ATTCAGGAGGCACGCATATT
scaffold742659_15.[illegible]	1	p3	(ATT)5	15	255	269	TCCCTTTCAATTACATCGCA
scaffold742694_16.[illegible]	1	p3	(AAT)6	18	762	779	AGGTCGGTTGAAAAGATGGA
scaffold742735_14.[illegible]	1	p3	(AAT)6	18	979	996	ATGGGTTTTGCCATCAATTT
scaffold742746_15.[illegible]	1	p3	(ACA)5	15	1880	1894	AAAGATCCAGTGTTGGCAGG
scaffold742770_14.[illegible]	1	p3	(GAA)6	18	3794	3811	TAGTTCTCCGCCTGCTCAAT
scaffold742802_17.[illegible]	1	p3	(TCC)5	15	6123	6137	GAGGCTTTCCACATCATCGT
scaffold742812_15.[illegible]	1	p3	(TGA)6	18	31	48	TTTGATTGGGAATAATTGGG
scaffold742846_15.[illegible]	1	p3	(ATT)5	15	1856	1870	GAGAAAAACAGAGGAGCAACAAA
scaffold742872_15.[illegible]	1	p3	(TCA)5	15	2009	2023	AATGCTGCATCAACCAGTCA
scaffold742893_16.[illegible]	1	p3	(ACA)5	15	2050	2064	TCAAGGTGTCCTCGATTTGA
scaffold742903_15.[illegible]	1	p3	(TAA)5	15	1611	1625	TTCCAACATGCTGCCTAAAA
scaffold742913_16.[illegible]	1	p3	(TAG)5	15	1130	1144	AGAGGCCAACAAAGAGACGA
scaffold742915_17.[illegible]	1	p3	(CAG)5	15	3995	4009	GTGCTGTTAGCTCCTCTGGG
scaffold742916_16.[illegible]	1	p3	(TGT)5	15	45	59	TGTAGCCGACCCCAATTTAT

scaffold742938_17.(	1	p3	(TCT)5	15	492	506	GGATTAAGAAAGACATTGGACGA
scaffold742955_13.2	1	p3	(TTC)5	15	271	285	TCCCATATTTGGAGTCCAGG
scaffold742965_15.7	1	p3	(CAT)5	15	2169	2183	AGTTGGGATTTGCCAGTTTG
scaffold742969_16.7	1	p3	(TGT)6	18	38	55	TGTAGCCGACCCCAATTTAT
scaffold742981_18.(	1	p3	(GAG)6	18	4494	4511	ATTTGGTGTCGCCTAAATCG
scaffold743005_14.(	1	p3	(TGT)5	15	51	65	ATGTAGCCGACCCCAATTTA
scaffold743014_17.4	1	p3	(GGT)5	15	3468	3482	AAGCCTTTCATGTCACCAGG
scaffold743018_16.4	1	p3	(GCT)5	15	5456	5470	CTACCTGATGGGCTATGGGA
scaffold743021_15.(	1	p3	(GTT)5	15	1623	1637	GATCCATATAGCCAACCCCA
scaffold743022_16.2	1	p3	(GCT)5	15	8663	8677	CGGAAAGAGTTTTGCTCAGG
scaffold743037_14.8	1	p3	(TGT)6	18	45	62	ATGTAGCCGACCCCAATTTA
scaffold743045_15.6	1	p3	(CCT)6	18	2085	2102	TCGAAAAAGTGCGTCATCAG
scaffold743048_15.3	1	p3	(TTC)7	21	2817	2837	GTGCGCGTTTAATGTCTTGA
scaffold743057_16.1	1	p3	(CCG)5	15	2221	2235	CACCGCCGTTCAGTAAAGAT
scaffold743093_15.5	1	p3	(GAA)5	15	54	68	GGAAGATGCTCCAACTCCTG
scaffold743094_15.(	1	p3	(GCG)9	27	1100	1126	TCTAACCCCACCATTTCTGC
scaffold743098_16.(	1	p3	(TTA)5	15	243	257	TCCGTTGAAATAAGATCGGG
scaffold743126_16.6	1	p3	(TTC)6	18	1118	1135	TGCTATGTGATGTTGGCTTG
scaffold743127_17.6	1	p3	(ACC)7	21	2595	2615	AATCCGAAATGCAAAACTCG
scaffold743155_15.5	1	p3	(TGT)5	15	47	61	TGTAGCCGACCCCAATTTAT
scaffold743156_18.(	1	p3	(TAT)5	15	3209	3223	CACCCAGTTTGAATTTTAGCAA
scaffold743165_16.2	1	p3	(CAT)6	18	2948	2965	GGTCCAAAAACTCAGGCAAG
scaffold743167_16.9	1	p3	(CCA)5	15	4457	4471	ACTGGGCAACATAATCCAGC
scaffold743199_15.5	1	p3	(TGT)8	24	39	62	TGTAGCCGACCCCAATTTAT
scaffold743213_14.7	1	p3	(CGG)5	15	66	80	AAAATTATGTAATAGTTTGCCGACG
scaffold743219_15.9	1	p3	(TTG)6	18	3327	3344	TTTGGTTCTTGTAGCCGACC
scaffold743231_16.(	1	p3	(CAC)5	15	244	258	ATATGTTTCGCCTCTCCCCT
scaffold743239_16.(	1	p3	(ATG)5	15	490	504	CAACGTGATGTCGATGGAAG
scaffold743249_16.2	1	p3	(GCG)5	15	592	606	GCCGGTCAGTTAATCTGCAT
scaffold743250_15.8	1	p3	(CCG)6	18	925	942	TAATCTACCAATGGCCCGAG
scaffold1350_16.1	1	p4	(AAGC)	20	675	694	TGAATTTGCCAAAAGCAGTG
scaffold1551_14.8	1	p4	(TCAA)!	20	1304	1323	TCACATACCTGCAACCATGAA
scaffold4205_14.5	1	p4	(TAGG)	20	1651	1670	AATGCTCCAACTTCGACTGG
scaffold4332_15.2	1	p4	(ATCG)!	20	825	844	GAGCACGGAAATCGAGAAAC
scaffold18302_15.4	1	p4	(GGGT)	20	1133	1152	GAGCCATCTTGTGACTGCAA
scaffold18303_15.4	1	p4	(CCCA)!	20	314	333	GGCCTTCAAAAGCATAACCA
scaffold18349_15.3	1	p4	(CGAT)!	20	104	123	TCGATCGATCTCTCCTGTGA
scaffold19199_11.2	1	p4	(AAAT)6	24	1209	1232	TTGTGGTCTCACTCCTCCAA
scaffold24576_14.6	1	p4	(ATTT)6	24	1325	1348	TGTGGTGTCTTGTGGTCTTGA
scaffold36007_12.4	1	p4	(GGGA)	20	45	64	GGTGTTTATTTCGGCTGGTG
scaffold37946_16.9	1	p4	(TCAT)5	20	728	747	GCACTTTCACGGGAGTTGAT
scaffold39016_15.7	1	p4	(AAAT)!	20	576	595	GAGGAAGAGTTTCGGATGGA
scaffold49950_16.0	1	p4	(TAAA)!	20	2805	2824	CGGCAAATTAAAACAGACACG
scaffold57330_16.1	1	p4	(TTAT)5	20	2378	2397	TGGTGCATAATGGCTCTGAC
scaffold58946_16.3	1	p4	(ATTT)5	20	1534	1553	TCCACACACGTCTTCACATTT
scaffold60885_12.5	1	p4	(CATC)6	24	1265	1288	TCAGCATTATGTGCTCTGCC
scaffold61097_16.7	1	p4	(AAAT)!	20	1154	1173	CAGCAGAGATCATCCACCAA
scaffold65330_12.7	1	p4	(TAAT)6	24	317	340	AGAAGCACAACCAACCAACC
scaffold66955_16.4	1	p4	(TTCT)5	20	983	1002	CGGTTTCATAAGGTTGTCGG
scaffold107651_15.5	1	p4	(CCAA)	20	1007	1026	TACATTGTGCCTCCTCCCTC
scaffold111948_14.5	1	p4	(TTAT)5	20	764	783	CCACTCATTTTTCCACACGA
scaffold120623_16.3	1	p4	(AAAT)!	20	3985	4004	GGCTCGAGTTGATGATCTTTG
scaffold124168_12.4	1	p4	(TATT)5	20	513	532	TTTCAGATCTGCCACAGACG
scaffold124671_15.1	1	p4	(TACA)!	20	276	295	CCTATCGTTCCACATGCCTT
scaffold124853_15.5	1	p4	(CCAC)!	20	422	441	CGAGATCCACGTGTCATTTG
scaffold125326_16.4	1	p4	(ATTA)5	20	1472	1491	TTGTAGTCCTTAAATTCCCGATT
scaffold126775_13.(	1	p4	(AATA)6	24	452	475	CATCTGCCATGCACCATTTA
scaffold137114_17.1	1	p4	(AGTC)!	20	931	950	TGTTCGGAGCTTGTGTTGAG

scaffold144284_14.8	1	p4	(GAAA)	20	663	682	TCTCTCATTGGTGGCCCTTA
scaffold144356_13.4	1	p4	(AAGA)	20	414	433	AAAGCAAACCAAACCAAACG
scaffold150875_13.5	1	p4	(CCAT)5	20	568	587	CCATGACACTGAATATCCCAGA
scaffold166093_15.4	1	p4	(AAGA)	24	54	77	TCCCGTGCGAGAGTAAAAGT
scaffold167894_10.1	1	p4	(GATT)5	20	248	267	CAGTGACTGCGATCGGTCTA
scaffold170575_16.9	1	p4	(ATTT)5	20	765	784	TTTCTGTTAGTCAAAATCGAGAGA
scaffold171052_12.6	1	p4	(ATAA)7	28	1459	1486	TGACAAAAATTGAGGCGTTG
scaffold180305_14.8	1	p4	(ATTG)5	20	636	655	GGATTGTGTGGCTACTGGCT
scaffold180742_17.8	1	p4	(TTGG)5	20	1030	1049	TGTCATGTCATCGTGTGCTTT
scaffold181162_10.2	1	p4	(TTAA)5	20	245	264	TCTCAGATATGGGCGTCACA
scaffold212608_15.7	1	p4	(AAAG)	20	904	923	GGTGAAGCAGAAAAACCAGC
scaffold224867_13.5	1	p4	(AATA)5	20	2194	2213	TGTGGTTTGCCTCACTCTTCT
scaffold228171_13.3	1	p4	(TAAA)5	20	201	220	TTATTTTTGGTACCCGGTGG
scaffold238727_15.8	1	p4	(TCCC)5	20	669	688	TTGATTTGCAACTCCCTTTT
scaffold241894_11.1	1	p4	(TTTA)5	20	325	344	TCACTGCTCTTTTCAACCCTT
scaffold259208_13.3	1	p4	(TATT)5	20	850	869	GACGTGGAATGTGGATGATG
scaffold262258_12.8	1	p4	(ATAA)5	20	59	78	TTTTCCGACTTTGGCAAATACT
scaffold268278_19.0	1	p4	(ATTT)5	20	293	312	TGGAATTGACGACTCCATTG
scaffold280650_16.7	1	p4	(AAAT)5	20	91	110	CAAATTAAAATGCGCCACG
scaffold280939_15.6	1	p4	(GAGG)	20	558	577	TGGCTTATGAGTAGGTCCGC
scaffold283553_12.0	1	p4	(GATC)5	20	280	299	TGGCGAGAAAACTCAGATCC
scaffold294280_13.3	1	p4	(ATTT)5	20	417	436	ACACACGCAGGCAGAATATG
scaffold309554_16.7	1	p4	(ATTT)5	20	39	58	TGAAGATTGCTTCCTTATGAATTG
scaffold314358_14.9	1	p4	(TTGC)7	28	1703	1730	TGCAGAGCAAATTCATGTCC
scaffold315660_13.1	1	p4	(TATT)5	20	162	181	TGTTGGAAGAAACAAAACTGGA
scaffold322738_16.0	1	p4	(TTGT)6	24	4485	4508	GCTCCTCTTCACTGTCGGTC
scaffold357424_11.7	1	p4	(ATTT)5	20	512	531	AGTCTTGGGAAGCGAAATGA
scaffold359091_10.1	1	p4	(AAAG)	20	376	395	TTTTCCGTGGTATCAGAGCC
scaffold362989_10.1	1	p4	(CTCC)5	20	948	967	TCTTGTTTTCTCCCCAAACC
scaffold379093_12.8	1	p4	(TATT)5	20	252	271	TGGGCTTCTTCAGCTTCTTT
scaffold401117_17.5	1	p4	(AATA)5	20	525	544	GGTGTGGTTTGCAGGCTATT
scaffold408858_16.9	1	p4	(ATTT)5	20	2286	2305	TTGCAATAATCAAGTTCAAGCA
scaffold421065_16.9	1	p4	(TAAA)6	24	5969	5992	CTCTCGTTTGGACTCTTGGG
scaffold424451_15.4	1	p4	(TAAA)5	20	1408	1427	CCCCTATAACTCTCGGGGTG
scaffold424746_14.9	1	p4	(TTTA)5	20	1082	1101	GTCTTGGTTGCCATCTCCAT
scaffold432935_14.5	1	p4	(AAAT)5	20	811	830	AATCGCAGAACATGAAACCC
scaffold433361_17.2	1	p4	(AAAT)5	20	3083	3102	TCCCCTCATCTCTGCCTCTA
scaffold434282_14.6	1	p4	(TCTT)5	20	6551	6570	GAAGCAAGAGAGACGTTGGG
scaffold448298_14.4	1	p4	(TGTA)6	24	800	823	TTGTGTTGGCGGCAGTAATA
scaffold452632_15.4	1	p4	(AATT)5	20	1570	1589	AGGAGGGTAGGTGGTCTGGT
scaffold457263_16.0	1	p4	(TCAC)5	20	52	71	GCCTTCTTTTCACGCATTTC
scaffold469276_12.7	1	p4	(AATA)5	20	820	839	ATATATGGGGGAGAGGGACG
scaffold471287_15.4	1	p4	(ATTT)5	20	428	447	TCATTACACCCGGAAATGGT
scaffold475041_13.7	1	p4	(AAAT)5	20	106	125	TACATGGCTGGCGTAGTTTG
scaffold475749_11.9	1	p4	(ATTA)5	20	237	256	GGATTGGGAGCACAAACCTA
scaffold476383_11.4	1	p4	(CGAT)5	20	312	331	ATCCCAATGAAGATCGAACG
scaffold476605_14.9	1	p4	(ATTT)5	20	61	80	ACTTGGGCCCGATCTATTTT
scaffold479255_12.6	1	p4	(TTAA)6	24	2004	2027	GGCATAAGAAAGGATGAATGTAA
scaffold492229_16.8	1	p4	(CCGC)	24	745	768	TGGTATGGTGTGGAGAACCA
scaffold495441_16.9	1	p4	(CCTT)5	20	427	446	TCGCTAACAACCAAACACCA
scaffold501321_16.3	1	p4	(ACAT)5	20	2245	2264	AAATAAAACCAAAACCGGGG
scaffold505269_13.3	1	p4	(AAAT)5	20	222	241	CAGGTTCAAATCCCACCAAC
scaffold512811_10.8	1	p4	(AATA)5	20	737	756	GCCCGATATTTATCAAGGCA
scaffold516141_15.2	1	p4	(CCTC)5	20	742	761	ACCCTAAGCAGAGGCTCACA
scaffold523703_12.7	1	p4	(AATA)6	24	2122	2145	CACATGTCCCAAGTGATTTCAA
scaffold523978_14.0	1	p4	(TAAT)5	20	319	338	GCAACAAATGAATTGGGCTT
scaffold529630_12.6	1	p4	(ATAG)6	24	545	568	TTTGGGAGGGAAAACGACTA
scaffold530555_14.9	1	p4	(ATAA)5	20	2003	2022	TTGGGAAGAACAATGAAGGG

scaffold530624_13.4	1	p4	(AAAG)	20	258	277	GGCTTGTCGTGGTTCAACTT
scaffold532563_13.0	1	p4	(TCCA)5	20	64	83	TTTGCCTATTTAAAGCCCCC
scaffold534298_14.9	1	p4	(TCGA)5	20	954	973	GGACGAAATTGATCGAAGGA
scaffold535680_16.1	1	p4	(CATT)5	20	2589	2608	GCAAGGGTGGAGGAATGATA
scaffold536005_15.1	1	p4	(AAAT)6	24	230	253	TGCAGAGATTTCAGCCATTG
scaffold536011_16.8	1	p4	(CTGA)5	20	100	119	AAGAAAAGGCGTGAATGTGG
scaffold537975_15.1	1	p4	(AAAT)6	24	2736	2759	TTAGGAAAGCAGGCAACCTT
scaffold539125_13.8	1	p4	(TTAT)6	24	654	677	CACTAGCACCTCATGGCTCA
scaffold552987_14.6	1	p4	(TATT)5	20	1778	1797	GCCATTCACCATCTCTCGTT
scaffold554669_13.1	1	p4	(ATTT)5	20	442	461	ATTTGGGGGAAACCATTAGG
scaffold555082_15.3	1	p4	(AAAT)6	24	1202	1225	CAAATAATTGCCGGACCAAA
scaffold559721_10.4	1	p4	(TCAA)5	20	242	261	CCCCCATGAAAAGTCAAAAA
scaffold559844_15.6	1	p4	(TAAT)5	20	216	235	CAAGTCAAATACCCGTGTCCA
scaffold567931_15.2	1	p4	(CACT)5	20	289	308	ATAGCTCACCACCGACCAAC
scaffold569595_12.6	1	p4	(AAAT)5	20	349	368	CGTGTCATGTCGACTCGTG
scaffold571451_14.7	1	p4	(AATA)6	24	720	743	GTCCACCTTTCTCGCAGATG
scaffold574092_10.5	1	p4	(CAAG)	20	109	128	GACGCAGGTACGGATATTTGA
scaffold580301_11.9	1	p4	(GAGG)	20	318	337	TCTTTTGTTTCGGAAATCGG
scaffold588183_17.3	1	p4	(AGTT)5	20	519	538	CCCTCGGATCTCATCAGAAA
scaffold588818_15.6	1	p4	(AGTT)5	20	2823	2842	TGTCCAAGATGGGATTGGTT
scaffold590508_13.9	1	p4	(TAAA)5	20	1622	1641	GCATTTTCGGAATTAAAGCG
scaffold591015_16.1	1	p4	(AATT)5	20	6907	6926	CAGATGAGGAGCACCAGTCA
scaffold591306_12.1	1	p4	(GAGG)	20	501	520	TCCATTATCTGGCCCTTTTG
scaffold592220_10.6	1	p4	(TACA)5	20	275	294	AAAAAGCCAGGGCCTACCTA
scaffold597113_16.0	1	p4	(ATAG)5	20	558	577	GAGTTTCCTTGGCTGCACAT
scaffold597874_16.2	1	p4	(TATT)5	20	1148	1167	AGCTAGTAGGGCAAAGTTGATTAG
scaffold598154_14.5	1	p4	(ATTT)5	20	839	858	AAATTGATTGGAGCATTGCC
scaffold600096_16.8	1	p4	(TTTA)5	20	1002	1021	TGCAATTCCACCAACTTTGA
scaffold600130_20.2	1	p4	(TGTA)5	20	298	317	CCGGAATCTGAGAGAGATCG
scaffold608297_15.5	1	p4	(ATTT)5	20	1844	1863	CAAGCAATGGGGAATGAAAT
scaffold609679_10.9	1	p4	(TTGG)5	20	44	63	TTGTCCCTAAACCCCCTCTT
scaffold609955_12.8	1	p4	(GCCT)5	20	380	399	ACACATTTGGCTGGAAGGAC
scaffold618292_14.6	1	p4	(TTAA)5	20	1634	1653	GGGAAAAGGACAATTCCTCG
scaffold621922_10.3	1	p4	(TTGA)5	20	43	62	GAAGCATTTCACATGTAGAGTATATG
scaffold627019_14.5	1	p4	(CTTC)5	20	381	400	GCGAGGGTTTTCTTGGTACA
scaffold627354_16.6	1	p4	(GGGA)	20	105	124	TAGCACTTGGGATGAAGCGT
scaffold630379_17.8	1	p4	(AATA)5	20	613	632	GCAATTTCATTTAACAATCACGA
scaffold633212_11.7	1	p4	(TTTA)5	20	423	442	CTCTCCATGGACGTAGGTGT
scaffold637193_14.9	1	p4	(TATT)6	24	279	302	ACGGCAAATTAAAATGCGAC
scaffold637968_12.5	1	p4	(TATT)5	20	112	131	TGGGTTTGGTCCATCTTCAT
scaffold640410_12.1	1	p4	(GGTT)5	20	142	161	TGTTTCTGCAGGTTGCAAAG
scaffold642968_11.6	1	p4	(TATT)5	20	493	512	GAAATATCCCCTGGAGCCAT
scaffold644448_16.1	1	p4	(ATTA)5	20	1395	1414	TGAGTCGGACTTGCATTTGA
scaffold647194_11.4	1	p4	(AATA)5	20	685	704	CAATTCCTAAACCAGCCCAA
scaffold647811_14.9	1	p4	(TATT)5	20	1516	1535	TCAATGTGCCATGATTTAGCA
scaffold653587_13.4	1	p4	(AATA)5	20	770	789	GTCAATCCGCACTTGGATTT
scaffold655002_17.4	1	p4	(ACCT)5	20	79	98	ACCCATTCCCAAGAGATGCT
scaffold655146_14.9	1	p4	(GAAA)	20	698	717	TAATTTGTTGAGGGGAGGCA
scaffold655622_16.8	1	p4	(TTTA)5	20	339	358	TCCTGTGCCAAGTTGAATAAGA
scaffold656850_18.3	1	p4	(AAAT)5	20	163	182	GTGGTATACCCAAAGCGCAC
scaffold657451_12.6	1	p4	(TATT)5	20	1012	1031	CAACTCATGGTTGTCGGTTG
scaffold658766_19.6	1	p4	(ACAA)	20	1414	1433	AAGAGCGTCCAAATCTCCAA
scaffold659505_13.3	1	p4	(AATA)5	20	505	524	TACATGGCAACCTCGTCGTA
scaffold659643_17.5	1	p4	(AAAT)6	24	1425	1448	TCTGTTTATAGTCCCCCGGA
scaffold661552_12.7	1	p4	(TTTA)5	20	202	221	GAAGTTTTGGGGATCCGAC
scaffold661558_16.3	1	p4	(AATA)6	24	50	73	TGAAGAAATCAAATCCCACAAA
scaffold662814_16.2	1	p4	(TTAA)5	20	1866	1885	CCTGATTAGCCACAAGGGAA
scaffold663928_16.9	1	p4	(ACAT)5	20	383	402	TGTGCTCGTACACTCGACAA

scaffold666666_15.3	1	p4	(TTTC)5	20	2541	2560	TTCTCATCATCATCAGCTCCA
scaffold667242_17.1	1	p4	(CAAA)	20	1398	1417	GCACAAGAGGAGGCAACTTC
scaffold670842_10.7	1	p4	(TTCT)6	24	925	948	GTGAAGGAATCTCTGGCTCG
scaffold672696_17.3	1	p4	(AAAT)5	20	1141	1160	GCAGGCCATATGCTAGTGGT
scaffold675764_14.6	1	p4	(ATTT)5	20	50	69	GAACGAGTTCGTTGACTAATTAAAA
scaffold676142_11.7	1	p4	(AAAT)6	24	47	70	CAGAGCGCACTCGAAGAGTA
scaffold677282_12.4	1	p4	(TTAT)5	20	766	785	ACAATCCTGTAATTCGCGTC
scaffold677401_21.2	1	p4	(AAAT)5	20	227	246	TTTTGCACCCGAATGGTTAT
scaffold677644_16.8	1	p4	(ATTT)5	20	376	395	TGGAATTTGTCCACCATAACA
scaffold677954_12.2	1	p4	(TTTA)5	20	629	648	TGAGATATTTCCCGCTGTCC
scaffold678923_14.9	1	p4	(CATG)5	20	1259	1278	GCAATGCATATTTAGGAAGTGG
scaffold681418_11.3	1	p4	(TAAA)5	20	563	582	GCGAAACGAAATTTGGGAA
scaffold683701_14.8	1	p4	(AATA)5	20	749	768	AAGTGAGTTAAGTTTTGACCCAA
scaffold684812_15.2	1	p4	(TTTA)5	20	296	315	GTCATTTCTCGTTTCGCGTT
scaffold687881_10.7	1	p4	(AATA)5	20	759	778	CCATTCATCATGATTCCTTCC
scaffold688114_14.9	1	p4	(TTAA)5	20	854	873	TGGTGTCCTGTGAAAAACCA
scaffold688827_14.6	1	p4	(AATA)5	20	597	616	ATGATGGCGTCATGTTTTCA
scaffold691984_12.1	1	p4	(AAAT)6	24	96	119	GCAAATTAAAATGTGCCACG
scaffold694377_14.2	1	p4	(TTAT)5	20	261	280	ACTCGAACGAACACCCAATC
scaffold695096_15.3	1	p4	(ATTA)5	20	694	713	CACCCCGTATGGAAATTCAG
scaffold695443_18.7	1	p4	(AAAT)5	20	165	184	CCAATTTTTCAATCACCGGA
scaffold695906_14.8	1	p4	(GGCA)	20	261	280	GCCACGGATAAGTAACACCG
scaffold697713_15.2	1	p4	(ATCC)6	24	1945	1968	AAGCTGACTCGCACATTCCT
scaffold698805_15.3	1	p4	(AAAC)	20	333	352	AACAAACGAGGGCAAGTGTC
scaffold699053_12.9	1	p4	(CATA)5	20	1330	1349	ACGCTCAAGCCATTCAAGTC
scaffold700333_14.3	1	p4	(ATTT)6	24	43	66	TGACGTACGATCGAGTCCAG
scaffold700635_12.0	1	p4	(GGGT)	20	25	44	TGGGAATTTAGGAATCGAGAGA
scaffold701183_13.7	1	p4	(AAAG)	20	278	297	TGGAAAGCCCTTTTTCTTCA
scaffold704554_16.4	1	p4	(AAAT)5	20	1745	1764	TATTTGTTGGTGGGTCGGTT
scaffold705454_17.5	1	p4	(AAAT)5	20	531	550	TTGATGGGAGGCAACTCATT
scaffold707915_12.1	1	p4	(GCAG)	20	539	558	AAGCACGTCAGCACAGACAC
scaffold708416_16.1	1	p4	(TCTT)5	20	708	727	AACTGCCCCCTTTCAATTCT
scaffold708632_12.3	1	p4	(TACA)5	20	175	194	GACTATAATGGCCAGCACCG
scaffold709642_15.0	1	p4	(GCCT)5	20	762	781	GCCTGATTAATTGTGTGGGG
scaffold710333_15.4	1	p4	(ATTT)5	20	672	691	AAGGCTCAAGAACAATGCAAA
scaffold710652_15.5	1	p4	(CTAG)5	20	1429	1448	CAGGCGGAACTAGAGAGGAA
scaffold711218_12.3	1	p4	(AATA)5	20	264	283	TGGTGGATTTTTATTTATGCAA
scaffold714710_14.4	1	p4	(CTCC)5	20	1062	1081	GCCCATTCATCGATTTGTTT
scaffold715985_14.9	1	p4	(AAAT)5	20	2199	2218	AAACGGATCTCTTGCGTTTG
scaffold716555_15.0	1	p4	(TCAT)5	20	2539	2558	CGTTCTTCCTCAGCTTCTGG
scaffold716659_13.7	1	p4	(ATGT)5	20	1249	1268	GGAAAGATGAAATGCTGGGA
scaffold717898_15.0	1	p4	(CTGC)5	20	850	869	GGCAGCTCCCACATACATTT
scaffold717962_15.3	1	p4	(AAAT)6	24	460	483	AATCAAGAAGCCTCTGCACG
scaffold718240_13.7	1	p4	(CATC)5	20	2512	2531	TATCTGCCTGCATATGCCCT
scaffold721219_16.7	1	p4	(AAAT)5	20	45	64	GGTCGGCTACATGAACCAAA
scaffold722963_14.3	1	p4	(ATTT)7	28	368	395	TCCGTCCACCTCTCAATCTC
scaffold723761_16.4	1	p4	(TATT)5	20	315	334	CCTGGAAAAATGTGGGTGAA
scaffold724599_19.0	1	p4	(AAAT)5	20	656	675	AGTTATTGGCCGAAAATCCA
scaffold725295_15.1	1	p4	(CCTC)5	20	1893	1912	ATTGGCTTTCCCGAGAACTT
scaffold726004_15.0	1	p4	(TTTC)5	20	869	888	AGTCTTGTGGCCCAGAGAAA
scaffold726425_17.4	1	p4	(TATT)5	20	771	790	AGCTAGTAGGGCAAAGTTGATTAG
scaffold727253_14.1	1	p4	(TTTA)5	20	241	260	TTGTTTGCCACTTGCTTCAC
scaffold729308_14.3	1	p4	(ATAA)5	20	4621	4640	AGTGGGGAGTTTAGTTCCCG
scaffold729539_11.4	1	p4	(TTAA)5	20	279	298	GCTGCAGATGGACTCACAAA
scaffold730205_13.4	1	p4	(TAAA)5	20	374	393	TTGTGTTATTTCATATCTTTGTGCTG
scaffold730642_15.6	1	p4	(TTAA)5	20	849	868	GACTTCGTGGGCTTGGACT
scaffold731835_14.0	1	p4	(AAAT)5	20	222	241	AAGTTTCTTTGGGACACCCC
scaffold732041_16.8	1	p4	(TATT)5	20	93	112	CGTGTGCTGGGCTATTTTCT

scaffold732762_14.6	1	p4	(TTCT)6	24	2925	2948	TGGGTTTGAACTGAAACTGC
scaffold733823_15.1	1	p4	(GAGT)	20	320	339	AACTGCGAGATTCAAAACGG
scaffold733994_16.5	1	p4	(CATT)5	20	296	315	GTATGCAAAGGGAAAAGCGA
scaffold734089_16.0	1	p4	(GATC)5	20	3325	3344	AGATTGAAGAAAAAGGGGGC
scaffold734118_14.6	1	p4	(AAAT)5	20	1167	1186	AGACCAGACATCCACTTGCC
scaffold734464_13.3	1	p4	(AAAT)6	24	3231	3254	GGGCTTCATGTATGCTTGGT
scaffold734483_13.8	1	p4	(TTCA)6	24	557	580	CAAAAAGCCCATCAGTCCAT
scaffold734892_16.5	1	p4	(TATG)5	20	1168	1187	CAATTTCCAGATGGAACGGT
scaffold734928_13.6	1	p4	(TCTT)5	20	272	291	TGGTTTGACATGTGATGGGA
scaffold735282_14.4	1	p4	(AGTT)5	20	190	209	GCTGCACCATCCATATTCAA
scaffold736148_16.3	1	p4	(ATTT)6	24	1740	1763	CAAAACCCCCACCTAAGACA
scaffold736332_16.7	1	p4	(GATT)5	20	181	200	GGGATGTGTGTACCTGAGGC
scaffold736671_15.7	1	p4	(GTAT)5	20	283	302	TGGATTTTCTTCCCATGTCC
scaffold737466_14.8	1	p4	(TTAT)5	20	850	869	TCCAAAATCTGGTTGCTTGA
scaffold737840_14.7	1	p4	(ATTT)5	20	770	789	TGCTTCCATTTGTGAAGATATGA
scaffold738175_13.8	1	p4	(CTTT)5	20	801	820	GGGGGAGATTGAAAGGGATA
scaffold738509_14.8	1	p4	(ATAA)5	20	137	156	GCAGAATTCCGAATGACGAT
scaffold739168_17.5	1	p4	(TCTA)5	20	1497	1516	AGTCTCCGGGGACAAATTCT
scaffold739390_14.0	1	p4	(TTTC)5	20	110	129	CGTGCTTGAACAAAAGCAAA
scaffold739734_15.3	1	p4	(TGCC)5	20	5458	5477	GGCAGGAAATATGGCGAGTA
scaffold740449_15.2	1	p4	(TTAT)5	20	941	960	TCCACCATGGAACACAGAAA
scaffold740496_16.0	1	p4	(ATAA)5	20	255	274	TCTTCCACGAGAATTGGACC
scaffold740667_16.0	1	p4	(GCAT)5	20	2213	2232	CGAGCTGAAAAAGACCAAGG
scaffold740879_14.0	1	p4	(AAAT)7	28	1280	1307	TTGAACGAAAATTGATGGTCC
scaffold741336_13.2	1	p4	(TATT)5	20	485	504	GCACAAAATGAAGCAGACGA
scaffold741447_15.2	1	p4	(GTAT)6	24	644	667	CAATTATTGGCCGCAAAAGT
scaffold741558_14.5	1	p4	(ATTT)5	20	1876	1895	CGGATTGCCAAGCCTATTT
scaffold741876_15.8	1	p4	(TTTC)5	20	3089	3108	ATGGTGTCAAAGTTCACGCA
scaffold742457_15.7	1	p4	(TTTA)5	20	1592	1611	GAAGGCAACAAGAGATGGGA
scaffold742712_16.0	1	p4	(TATT)5	20	1039	1058	AATCATGCAACCACAACAATTA
scaffold742933_15.3	1	p4	(GATT)5	20	1198	1217	AAACAAACCTGCAAAATGCC
scaffold743044_16.1	1	p4	(ATAA)6	24	6543	6566	GCATCGACCTTGGACAAAAT
scaffold743097_16.5	1	p4	(TTAT)5	20	3265	3284	AATGGCTCAGTAAACAATGGG
scaffold78862_14.0	1	p5	(GCGG(	25	1209	1233	ACAAACCTCGATTCCTGTGG
scaffold129996_14.2	1	p5	(ATAAA	25	2190	2214	TTGCATCTTCATGTGCAATTC
scaffold209057_14.9	1	p5	(ATTTT)	25	28	52	TGCTCCCTCCAATTAAGACG
scaffold281552_10.3	1	p5	(CAGG(	25	240	264	GCGCCCATAAAAATGAAATG
scaffold346597_11.5	1	p5	(CTTTT)	25	41	65	CCGACTTTTATGGCTGCTGT
scaffold404725_15.9	1	p5	(AATAC	25	527	551	TGCGAATTGAATAAGGCAAA
scaffold419361_15.9	1	p5	(AATAA	25	767	791	GGCAACAAACAGAATCCCAA
scaffold436901_11.6	1	p5	(CCAAC	25	228	252	GCCGGGTCTAGTGAATGAAA
scaffold437080_10.1	1	p5	(ACACC	25	85	109	ATTTGGTTCGGTTCGGTTTT
scaffold450612_11.1	1	p5	(TTAGG	25	74	98	GAGTATTCATTGTACACAATAAGGAG
scaffold498369_13.7	1	p5	(AATCT	30	470	499	TTTGTCATGACTTCGGCTTG
scaffold502759_17.3	1	p5	(TAAAA	25	49	73	TCTAAAAACATGGGTAAAAATGATAA
scaffold549972_10.6	1	p5	(GATGC	25	81	105	TTTGAGGAGCTTTGCGTTTT
scaffold567385_10.3	1	p5	(ACAAA	25	64	88	AAAGAGCAATGTGGCATCAA
scaffold574523_12.8	1	p5	(CCTAA	25	186	210	TCAAACCAGGTTCTTCAATGG
scaffold588661_14.1	1	p5	(AGATA	25	2021	2045	CAATGACAACCAAACATGCC
scaffold634405_15.1	1	p5	(TCGGC	35	1527	1561	TTGGCGGAAGCCATAATATC
scaffold637883_16.6	1	p5	(GGGTT	25	22	46	AAACGGGGTTGGGTTGTG
scaffold652062_16.1	1	p5	(ATAAT	25	407	431	AACGATAACGGTAACGATAACAA
scaffold679227_16.8	1	p5	(AAATG	30	45	74	GGAAGGAAAGCTAAAAAGGATT
scaffold708599_14.5	1	p5	(AAAAT	25	254	278	TGCAAAAGCACTCTTCCTGA
scaffold720454_13.9	1	p5	(ACCTA	25	1226	1250	TGGCATGCGAGTGCTATTAC
scaffold732293_13.2	1	p5	(TTTTG)	25	1908	1932	GCAGACCATAATGCTTTTGTGA
scaffold733459_14.0	1	p5	(TATCT	30	1080	1109	GAAGAAGATGTCAAGCCGGA
scaffold733662_13.5	1	p5	(TGCTT	25	144	168	CCAAGAGAGAGAAAGGGGCT

scaffold737970_18.:	1	p5	(ATCTA	25	227	251	TTCTCTTGGAATGGGATTGG
scaffold740060_14.4	1	p5	(CTTTT)	25	123	147	GTTCGAACCGAACCATGAAC
scaffold741982_15.:	1	p5	(TTTAT)	30	1285	1314	TGCAACAATTTTTGGTATATATCTTTC
scaffold741994_15.2	1	p5	(ATATT	25	227	251	TTATTGCACAGCTTCATGCC
scaffold97925_17.0	1	p6	(TTGGA	30	420	449	AAAGCTTTGTCCCACATAGGAA
scaffold156626_11.2	1	p6	(CCAAC	30	252	281	CACTGCTGCTGAGGAGTTTG
scaffold339935_15.:	1	p6	(TCCAC	30	903	932	AAACATACCAACTCACCACTCC
scaffold502162_13.7	1	p6	(TAGTA	30	215	244	GAGAAACTGCAGCAAAAGGC
scaffold540849_12.9	1	p6	(ACAAT	36	367	402	AATTTCGTATCGAGTTGGCG
scaffold554235_20.7	1	p6	(ATCGG	30	204	233	GGATTTGGATGGGTTTTTCA
scaffold576716_13.:	1	p6	(GATTT	30	908	937	TTGGTCAGATCAAACCGAATC
scaffold652042_14.8	1	p6	(TTCTG	30	72	101	AGACAATCCATCTCCATCTGGT
scaffold722193_18.0	1	p6	(GATTG	30	1163	1192	TGCATCTGCTTCTGGTTTTG
scaffold736788_12.7	1	p6	(CAAAA	30	540	569	TCCGATCGAATGGACCTTAC
scaffold740076_14.2	1	p6	(TGTGG	30	1359	1388	CGCCTCGATTGAATCTTCTC

Tm(C°)	size	REVERSE PRIMER1 (5'-3')	Tm(C°)	size	PRODUCT 1 size (bp)	start (bp)	end (bp)
57.426	23	TACCCCTACCCTTTGGGAAC	60.046	20	207	1433	1639
59.728	21	TCTCACGATCCCCTTACCAC	59.927	20	178	21	198
59.955	20	AAACACGACCATTTTCAGGC	59.978	20	277	82	358
59.745	20	TTCTCATACTAAAAGGTCTTTCTC/	59.662	27	165	23	187
60.533	23	ATTTCCCGGCTTCTTGCTAT	60.061	20	132	599	730
59.844	20	GGCATTATGCTTCTGCCATT	60.067	20	148	362	509
60.397	20	CTATGCTTGCATTCACCCAA	59.688	20	279	14936	15214
60.217	25	CGTGCCAAAAACCGACTAAT	59.996	20	181	19	199
59.989	20	ATTTTGGACCACCCCTTTTC	60.032	20	158	667	824
60.119	20	CAAAACAATTCCTTCTACCTTTTTC	59.519	25	238	11	248
60.025	20	GCGTCTACGGTGCAGAAAAT	60.278	20	221	89	309
59.577	20	TGCGTGAAAAACAGATGGTG	60.699	20	173	20	192
60.073	20	ACCGTGTCTCCAAAAGATGG	59.966	20	179	162	340
59.842	20	CAATCTCGAAAATGGATGGC	60.414	20	193	272	464
58.973	20	CGTGAGAAGCAACCAAATGA	59.84	20	240	253	492
59.241	21	TTTATCCTTACGCAATCCCAA	59.442	21	197	414	610
60.626	20	CCCACACCCTCATTCTCTCA	61.071	20	232	369	600
60.299	20	CAACACAAGAACTCACCTCCC	59.607	21	242	383	624
60.232	20	AATTTCCACCGGTGTTCTTG	59.83	20	231	6	236
59.903	20	TTGGGAGGGAGAGATTGTTG	60.042	20	261	16	276
59.52	20	TGGGCTAAGTGGAGTGAGTG	58.879	20	150	13	162
59.756	20	CCGATTGTTAAAATTCCGGTT	60.062	21	210	16	225
60.198	20	TGTTGACTTGCTTTCTTGGC	59.05	20	113	661	773
58.254	21	GCTTAATCTTTCAACGCCGA	60.343	20	212	30	241
60.144	20	CAAATATGCTTTCTTGATCATCATT	58.646	25	240	622	861
60.066	20	CACCATTGTGTCCAGGTCAG	59.997	20	160	200	359
59.679	20	AATGATCTCATGGTGGAGGG	59.737	20	154	3	156
60.938	20	TCCACTCACCTAAACCCGAC	59.966	20	229	8	236
59.429	20	CCCCTTTTTCAGACGTTTTG	59.581	20	273	14	286
60.227	20	CGCGAACTTTACAGGGCTAC	59.904	20	239	85	323
60.13	20	TGGGCTCGAATCTCTTGTTT	59.813	20	183	121	303
59.772	21	TCAGGGGCATATGTGTCAAA	59.924	20	195	13	207
59.368	22	CCAGCCAATTTTATCCCAAG	59.406	20	208	1	208
59.927	20	GACTCGTAATGATCCCCCAA	59.75	20	209	252	460
59.694	20	GCCGTTTTAACGGTTTTGG	60.342	19	265	214	478
60.043	20	TAAGTACAGGAGCCATCGGG	60.088	20	116	66	181
59.99	21	ATTGGGATTTTTGGTTTGGA	59.111	20	115	132	246
59.989	20	CAAAACCAACAGCCGAGATT	60.11	20	277	219	495
59.182	20	CCGATATGCTATGCTTTTGC	58.421	20	163	76	238
60.005	20	CCATGTTGTAATTAATGTAGGGAA	57.155	24	125	2152	2276
59.997	20	GGTCACGAGAACACGCATAC	59.169	20	231	2250	2480
57.03	27	TAAGCATCCACTCGCATCAC	59.83	20	177	24	200
60.117	20	TGATGGATTGCATTTGAGGA	60.009	20	241	198	438
57.667	22	CGGAAACAAGCAACTACAGC	58.58	20	146	29	174
59.859	20	ATAATGCTTGTCGGTTTCGC	60.103	20	210	38	247
60.02	20	TCCCAAATTTTGTCCAAAGC	59.916	20	190	1023	1212
60.049	20	ACCCCACTAAACCCAAACTT	57.435	20	153	35	187
57.932	23	GCCATTTTCCAAGAGCTTAAA	58.508	21	273	28	300
60.052	20	ACACGAACGGTTTAGGATCG	59.993	20	161	132	292
59.95	20	CTTGACTTCAAACCCCAACG	60.52	20	196	336	531
59.985	20	GAGTCGCTGCAACAAATGAA	59.995	20	228	30	257
60.301	24	TCAGTGGTTGTAACATTGGCA	60.019	21	241	381	621
58.731	21	CACCCGAAAACCGAATAAGA	59.931	20	101	782	882
59.389	20	AATGCAGGGTTTGCAAGAGT	59.74	20	150	16	165
59.938	20	TGAGCGGCTACGCTAATTCT	60.138	20	229	16	244

60.153	20	CTCAACGGTATGCTCGGATT	60.096	20	231	406	636
58.76	22	AACAATCTGGATCGTTTGATACC	59.259	23	165	0	164
59.969	20	TCCAAAAATCAAAGCTTGAAGA	58.987	22	220	65	284
58.57	20	CGTCAGCATTTACCCCAACT	59.993	20	271	951	1221
60.065	20	CATTTTACGAACGAACCACG	59.083	20	268	143	410
59.67	20	TGGTTGCACATGAATCATCC	60.339	20	172	1333	1504
59.839	20	AACAACAACCCCACCCATTA	59.948	20	198	3118	3315
61.606	20	ATCCCTCGTGTTCTTCCTCC	60.456	20	172	8	179
59.64	20	ACCGTCCATCTTAATGCAGC	60.103	20	239	874	1112
60.103	20	CATCAGAGCCGTGATTGAGA	59.942	20	143	0	142
60.978	20	GGTTGTATGAAACCGTTAGTTGC	59.826	23	273	426	698
61.018	20	TCACTCCGCCAACTAACAAA	59.317	20	177	169	345
59.623	20	GTTTGTGGTGTTCATGTGGC	59.862	20	241	1849	2089
58.188	23	CGCTTCTTTTGTATCCCAGC	59.845	20	101	0	100
59.288	23	AGCATAAGACGATGCGAGGT	59.866	20	161	12	172
59.847	20	GCCCCCTTCTGAATCTCCTA	60.537	20	272	2564	2835
59.697	21	ATTCAAGCATGGAATCGCTC	60.185	20	202	12	213
59.081	23	CCCATTCCCAAATCCCTTAT	59.846	20	158	925	1082
59.399	20	GTCTTGCACGATTCTCCACA	59.837	20	158	654	811
60.162	20	TTATTGCCTACCAACCAGCC	59.96	20	183	4195	4377
59.442	21	CACATAAATGGGTTATCAATGGA	59.851	24	210	302	511
59.127	20	TCCAACAATCTCCCACTTGA	59.059	20	129	158	286
60.154	20	TGCGTTTGATGTTCGTCTTC	59.847	20	220	59	278
60.377	20	TTCGCTGGTTCTGAGTTCCT	59.989	20	278	61	338
59.808	23	TCCCTCTCTCTCTCTCTCGG	58.792	20	165	25	189
59.587	21	GCTAATTGCGCAACAAACTG	59.514	20	224	315	538
59.322	20	CGGTTTTGGTTTGTTCCTACA	59.884	21	255	1160	1414
59.773	20	CCCACTCTGGTGACACATTG	59.997	20	190	33	222
59.856	20	GTCGGTACTGGGGCAGATAA	59.955	20	228	422	649
59.315	21	TCTTGTCATCACCTTTCCCC	59.903	20	277	481	757
60.317	20	CCATACCTAACCTGTACCGTCG	60.652	22	177	504	680
59.795	20	TCTGCTATCGAAACAACCCC	60.074	20	267	189	455
59.907	20	CCTTTTTCTTGCAAGGATTGA	59.325	21	250	266	515
59.333	20	CGACCTGAAGGAGGCTAGAG	59.183	20	167	1217	1383
60.812	20	ACCGGCAAGAAGAAAACTGA	59.853	20	267	1525	1791
60.025	20	TCATTAGCCATGTGTTCCCA	59.924	20	125	981	1105
60.117	20	CATGAGCCGCTAGATTGACA	59.972	20	251	50	300
59.048	25	TGCGTGCCATTTATAAAACA	57.749	20	112	1	112
60.135	20	CCGAAAGAAGCAGGATAGCA	60.481	20	269	1394	1662
60.008	20	TACGGGATCTCTTTGGTTCG	60.066	20	243	884	1126
59.721	20	AAACTCCTCTCCCAGCCACT	60.252	20	226	2	227
60.19	20	ATTCCTCCTCAACCAAACCC	60.169	20	144	225	368
60.361	20	ATGTGCAAAGCTGCTGAAGA	59.746	20	260	293	552
59.166	20	TGGACGTAAATACCCTTTTACCA	59.667	23	125	236	360
59.9	20	GGGAAACCCACAATCATCAC	60.034	20	220	31	250
58.711	23	ATGCGCGCTGCTTTAATAAT	59.873	20	142	6	147
59.813	20	TGGGCAGTAGAATACCTCCG	60.088	20	118	84	201
59.034	20	TTTTGTTTGGGACAAAAATGA	58.032	21	280	90	369
59.691	20	TTCGAGTGACACAAGGCAAG	60.025	20	242	18	259
59.767	22	ATTAGGCGACAACCAACTCG	60.132	20	175	714	888
59.975	20	AGAGACGGCAGAAGGTGAGA	60.135	20	212	1263	1474
58.5	20	AATGGCCGAGAGGGTTTTAC	60.32	20	103	10	112
60.296	20	TCTCAGCCAACACATTTCCA	60.24	20	100	5017	5116
60.119	20	TGAAGTTAGGAAATAAGGAGGTG	58.743	25	137	315	451
59.194	24	GGCCCAAATCAATACGAAAA	59.773	20	152	26	177
60.656	20	TTGGCCTTGCCATGTATATC	58.457	20	278	249	526
59.894	20	GGGAAGCTATGATGGGGAAT	60.117	20	249	2139	2387
59.948	20	GCTTTCATTTTTCAGCCCAA	60.188	20	198	510	707

59.843	20	CAACAGTTTGGCATCAGAGC	59.445	20	163	70	232
60.021	20	GCTGGGACGATCACGTATTT	59.962	20	236	2098	2333
60.288	20	TTTGGTTATGGACATACGGTCA	60.11	22	171	650	820
60.218	20	ATGCATCTCGGATCACATCA	60.039	20	193	8	200
60.066	20	CTCTCTCAATGTGGCAACGA	59.984	20	188	581	768
59.925	20	TTCCACCGTTGAAAGATCAAT	59.42	21	151	48	198
58.212	23	ATTTATGCTGACCGGAGTGG	59.955	20	225	621	845
59.138	21	ATACGTGTGCGAAACAACCC	60.817	20	124	315	438
59.086	20	ATTCCCTCCTCAACACCCTT	59.795	20	216	101	316
60.111	20	CCCCACTCCCTACATTTCAC	59.259	20	218	902	1119
58.866	20	AAGGAGATGACACCTAAACCCA	59.864	22	156	12	167
59.883	21	TGGCTGTCATTATTTCTCATTTC	58.262	23	150	368	517
59.434	20	GGAAATAATAAACACAAACACCG	59.233	24	253	882	1134
59.564	20	TCCTTGAACCATTCCATCATT	59.234	21	116	149	264
59.929	20	GAGGGCCAAAATCTTTAGTGG	59.952	21	207	69	275
60.239	20	TCTACGTGGCATGTGGACTC	59.707	20	216	4	219
59.333	22	TTGAATTGAATTGGAGCAAAA	58.265	21	233	20	252
59.853	20	TATAACCGTCGGCTTGCTTC	60.23	20	254	17	270
60.074	20	TCATATCCACGAGAATGGCA	60.033	20	195	1264	1458
59.95	20	TGGTGGAGGTATGGTGTGAA	59.806	20	119	23	141
59.82	20	CCCAAGACCCCAATTTGATT	60.91	20	267	57	323
59.761	20	CATCGACACCACTCATCGTC	60.121	20	240	9	248
60.18	20	TGCTGCAGTCTGTCATGGAT	60.436	20	243	242	484
60.067	20	CGTTGCATGGTGTATTGGAC	59.847	20	200	29	228
60.124	20	GATGTTGGGTCTGCAGAGGT	60.12	20	237	566	802
61.023	20	TTCATCTCCCTAATCGACCG	60.029	20	269	411	679
59.706	20	TTAGCTTCACACCAGCAACG	60.05	20	276	18	293
60.037	20	TTGCCTAAAAAGTTGCTCCC	59.338	20	257	978	1234
59.93	22	TTTACCGACGTCTCACCATCT	59.607	21	155	18	172
59.17	20	GAAGGTGTCTTCAATGGAGGTT	59.484	22	151	163	313
59.244	20	TGTGAGCCATAACTCGACCA	60.263	20	251	6	256
59.831	20	AAAAATTTATCAGATTTACGAGCA	57.706	25	133	312	444
59.257	20	CTTAGCCCCCAAGTTTTCAA	59.198	20	280	303	582
60.162	20	AAAGGCGACAAAACCATCAC	59.978	20	195	572	766
60.129	20	GACAGCCTTTTTGTTTTGCC	59.73	20	171	8	178
59.801	20	CAGGATGTGGTCATCCTCACT	59.977	21	275	1875	2149
59.951	20	GTATGGGGGAGCTGAACAAA	59.933	20	231	1248	1478
59.867	20	GGACCGTTTTAACGGATTTG	59.315	20	271	166	436
60.492	20	GGATCAGTAAAATAATAGGCAGA	59.934	26	244	537	780
60.132	20	ATCCGTGCCCTATCAGGAAT	60.682	20	234	526	759
60.413	20	CCACATCCTCAGCTCAGACA	59.98	20	158	212	369
60.088	20	CGATCCTCCTCCTTCACATT	59.09	20	244	1542	1785
59.026	23	AAGGACTCAAGCCCCTCAAT	60.074	20	156	20	175
59.13	21	CAGACAGCAGAGGTGTGCAT	60.055	20	203	96	298
59.997	20	TGAAACGGAACAAGATTTGC	58.747	20	135	15	149
60.175	20	ACCCAGATTTGTTCCTACGC	59.056	20	214	796	1009
59.993	20	CGTGTCAACTGTGACGAGAAA	59.93	21	214	1837	2050
59.911	20	GAAACCCTAACCTGATGCCA	59.933	20	252	447	698
59.72	22	GCTATGCATCAACGCACAAT	59.724	20	253	409	661
60.301	20	TGCCTACTCCACCAACATGA	60.112	20	230	938	1167
59.988	20	ATGGCTAACCATCAATTGCC	59.791	20	268	501	768
59.676	20	TATTGTTGCGAAGGGCCTAA	60.58	20	249	221	469
60.257	20	CTACTCCAAGTCCGACCCAA	60.103	20	192	1185	1376
59.259	20	CACAATATCAAGAACCCCCAA	59.671	21	266	20	285
60.051	20	TTGCATCATCCAGGCATTTA	60.035	20	157	2174	2330
60.006	20	ATTTCAGCACCCCCAACA	59.893	18	130	1002	1131
59.505	20	TAGCCCACTAATCCCAAACC	58.903	20	113	17	129
59.859	21	GAACCTTCCATCTTCCACCA	59.903	20	156	2193	2348

60.001	20	ACCCCCAATTCTTGATAGCC	60.152	20	101	270	370
58.969	21	GAGCTCCACATCAACACCAA	59.682	20	169	12	180
59.981	20	TGCGTTTTGATGATATATAGGTGT	60.054	26	214	561	774
60.144	21	GGCCGTATTAGCAGCGTAAA	60.246	20	279	480	758
59.799	20	AGCCACGTGTGTTCATTGAG	59.751	20	241	515	755
59.761	20	ATCTGCCATAGCACCAAACC	59.962	20	269	362	630
60.047	20	CCTCGAAGTCGAAACCTGAG	59.982	20	192	604	795
59.483	21	TGAAAGCCTACCTTGCTTCC	59.452	20	246	3	248
59.152	20	CTCGGTTGAGCTCGGATAAG	59.971	20	161	81	241
59.379	23	CCTGACTTCGCTGTGGGTAT	60.134	20	169	2287	2455
59.865	23	AACCCACTCCTATTTCCTCTCA	59.101	22	145	175	319
59.933	20	TCAATGCTCTTTTCAACCCTTT	60.109	22	164	19	182
59.989	20	GGTGGTATGGTGTGGAGGAC	60.096	20	185	61	245
59.939	20	GGCATCAGAGCCAAGTTAGG	59.836	20	192	8	199
59.726	20	GGCACGATTCAAAAGTCACA	59.697	20	274	122	395
59.975	20	TGACAAAGGGTGTGTTGGAA	59.976	20	153	75	227
60.488	20	AACGAGGGGAGTGACACTTG	60.151	20	274	11	284
59.657	20	GTTGGCTTGTGAGCGTGTAG	59.515	20	155	52	206
60.047	19	CCATTGAGACTCGAACCCAT	59.927	20	183	75	257
59.272	20	GCCTCTCACATGCAACAAAA	59.847	20	253	308	560
58.774	26	CACGCAATCCACATCAAGTC	60.12	20	247	76	322
59.962	20	GAGAATGTTCGAGCGTGTGA	59.992	20	235	369	603
60.095	23	TACGACTCTTTCAAGGGGGA	59.665	20	233	1964	2196
59.815	21	GGGATGATGTTGAATTAGTGGG	60.431	22	280	20	299
59.23	20	ATATGTGGGATTCTGCCTGC	59.923	20	253	1421	1673
59.944	23	TCAAAACAAATACTAACATGCAC	57.428	24	192	1073	1264
59.576	20	TTGAGAGGATTTGAGTTGGCT	58.923	21	251	157	407
60.674	20	GCGACATGACACAATCAACC	59.975	20	165	430	594
59.926	20	TGATTTCCTAGATACGGGGC	58.998	20	131	46	176
60.08	19	GCGAGAACTTAGTCAACGGG	59.875	20	184	15	198
60.349	20	AGAAGACACTTGGGCCTGAA	59.844	20	203	222	424
60.903	20	CTCCTCTCTGCTGAGCCAGT	59.883	20	192	265	456
60.16	20	GCCTAGCCTATTTGAACCCA	59.182	20	163	233	395
60.052	20	TTTTTAAGGTGGGGAGAATGT	57.596	21	248	1093	1340
60.833	20	CAACCCAAGTTTACATGTGACG	60.318	22	280	47	326
58.144	25	GGGGCTGTATAATGCGAAAA	59.928	20	196	165	360
58.629	22	ACCATCCCGTTTCAAACTCA	60.353	20	178	7	184
60.262	20	TGAAATCCTTGAACCATTCCAT	60.542	22	200	15	214
59.056	20	AACAACCCTCCCCTCATTTC	60.169	20	121	92	212
59.89	20	GGACGGTTAATGATTGTCCC	59.105	20	264	909	1172
60.188	20	TATTCCGCCATAGAACCAGG	59.916	20	219	547	765
60.22	20	CCGAAAGCTGCAAGTCCTAC	60.015	20	176	256	431
59.369	20	AACCCTCCAATTCCAAGAGG	60.298	20	207	356	562
60.692	20	TCTTCGACGGAGGAGAATGT	59.803	20	194	1128	1321
60.21	20	TGGGCTACCAAACACAGCTT	60.689	20	189	765	953
60.031	20	TGGTTCAACCTAGCATTCCC	59.933	20	178	68	245
57.16	27	GAGAGGTTAAGGGGGCAGAC	60.074	20	258	561	818
60.018	21	TCCACCTAGTCTTTCAGCAGC	59.63	21	102	19	120
60.323	22	GAATGAAGAATGGTTTGTCCG	59.42	21	254	690	943
59.624	21	TTCCCTTTTCGTACGATTGC	60.074	20	234	1843	2076
59.756	20	TCAAACACCAATCGCTCAAA	60.234	20	131	3643	3773
59.778	20	CGGTTAAGATTCAGCAAGCG	60.895	20	221	73	293
59.988	20	TATACGCACTCACACACGCA	59.926	20	211	2608	2818
58.824	20	TCATTCACCATATCCACCCC	60.404	20	280	4	283
58.978	20	ATGGTTCCGATGAGATCCTG	59.886	20	212	10	221
59.894	20	GATAAAAATGGCACACGGCT	59.967	20	261	212	472
59.5	21	GAAATCCCACTGCCAACTTT	59.031	20	279	80	358
60.624	20	GGGAATGAAATGCACATTGA	59.33	20	272	438	709

59.685	20	TGTCCCGTTCTCTCTCTGCT	60.135	20	276	3	278
59.337	20	TGCCGGTCTTCTTCTGACTT	59.989	20	253	16	268
60.467	21	TGAAGTTAGGAAATAAGGAGGTG	58.743	25	279	828	1106
60.018	22	GGTTGAGCTCGGATAAGCTG	59.978	20	197	0	196
59.496	20	GCTTTTATTGACTCAAGATCCGA	59.75	23	268	6816	7083
60.199	20	AACCCCACACATTCAACACA	59.701	20	165	167	331
60.23	20	TCTGTTGTGCTGCTGCTTCT	59.929	20	183	389	571
60.517	19	ACAATGGTCCAAGACCGAAG	59.966	20	137	25	161
59.612	20	ACCACCGAGCACCATGTATT	60.263	20	207	2	208
60.268	20	TGGTTGAATTTTGTCCCACA	59.792	20	186	1433	1618
59.634	21	TCTCACGATATCCTGCATCG	59.781	20	187	26	212
61.271	20	CTCAAACCATACGCTTGGC	59.287	19	113	114	226
57.739	20	AGTGGATAAACCCTGCGAAA	59.569	20	147	0	146
59.184	21	TTGTCCTCATCATCGAACCA	60.048	20	211	15	225
59.747	21	CAAATTCCCACCCCTGTCTA	59.784	20	214	23	236
59.717	22	AGGAGATCGAGAGGGAGAGG	59.906	20	226	552	777
59.668	20	ATAATGGGGTTTCAGTCCGA	59.244	20	278	136	413
59.966	20	TGAATTCGGATATGCGATGA	59.997	20	261	3492	3752
60.24	20	CTTTCTCCATCCCATCTCCA	60.003	20	272	576	847
59.995	20	GAAGCCCGAGTAAGGAAACC	60.074	20	113	1574	1686
59.873	20	AGTGCCAGATAGGCCGTTTA	59.73	20	244	172	415
60.184	20	AGCCGTTTTGAGACGTTGTT	59.78	20	262	45	306
59.784	20	AAACCATTTGGGGACGATTT	60.411	20	168	28	195
59.839	20	CCCCGGTTTCATTTATAGCA	59.789	20	200	0	199
60.363	20	GGAGAGTACGAGAGTGAGGAGG	59.505	22	136	174	309
60.088	20	CTACCGTCAAATCCTGGCTC	59.694	20	271	678	948
58.78	20	CCGGGTTTAAAGCAATCAAA	59.937	20	143	24	166
57.573	20	TCCCCGTTTTTACTCGTCTG	60.103	20	168	25	192
60.074	20	CCTCCAGAAATTGGGAGTGA	60.042	20	224	3393	3616
59.759	21	AATTGAAACCCTGCAGCTTG	60.249	20	240	282	521
60.243	20	CACGTGTGTAAGTTTTGTGCG	60.26	21	262	90	351
59.943	20	TCTCCACTCTCTCCCACGAT	59.792	20	230	2993	3222
59.764	20	TAACCACCTCTTGGGTCCAC	59.82	20	170	1474	1643
57.265	20	TAGGCATTATTGGATTCCCG	59.751	20	159	30	188
59.902	19	TAATCCCGCTGAAAATGAGG	60.031	20	198	2	199
59.923	20	CCTTTGTCTTCGAACCCGTA	60.103	20	221	96	316
60.245	23	ACCTCTCAGGGTTCACCGTA	59.574	20	266	55	320
60.035	22	CTTTAGGGCCCTTGATTTCC	59.903	20	170	196	365
60.051	20	TCGACCTTAACCACCTCGAC	60.111	20	138	23	160
60.568	21	CGTCGGTTCAACTGGAGAAT	60.111	20	223	267	489
59.556	20	TCGAAAATGTCCGACTCTCC	60.195	20	224	26	249
60.039	20	GGGCTTTGAGTAACTGAGGTG	58.851	21	274	315	588
59.162	22	ACATAACTCTCCGGCCAATG	59.955	20	196	45	240
60.836	20	TCGAAATGCAGTGGTTGAAG	59.84	20	245	4	248
59.903	20	CGGAATATCGAGAAGGTTATTATT	59.689	26	266	69	334
59.885	24	CAACGGTGATGACGTCGTAG	60.175	20	230	151	380
59.693	21	ATCTTCCATCCATCGAGCTG	60.181	20	112	497	608
59.882	20	AGGGTATGTCCGGTATGCAG	59.836	20	264	2953	3216
59.247	20	CCACCACCTAATTCTCTGGC	59.55	20	163	1146	1308
60.249	20	TCAAGACTAAAATTGGTAAAGAA(	58.241	27	133	415	547
58.743	22	CGAGTGTGTACATGGGCATT	59.445	20	278	6002	6279
59.615	20	GCAACGGAATACCACTCGTT	60	20	241	97	337
58.327	23	CCATTTTCAATCCTGTGCCT	59.933	20	100	7	106
59.934	20	CAAGTTGATGCTAGTCCTCCG	59.883	21	225	3216	3440
59.814	26	CCGGATTCTACTGTCGGAAA	60.066	20	279	429	707
60.488	20	AGGCTTGATCTTTGCTTCCC	60.713	20	212	186	397
60.766	20	TGGGAATGAAAAAGCCATGT	60.309	20	237	731	967
59.993	20	GAACTCGCTTAGTTGCTCCG	60.154	20	276	453	728

59.801	20	CATTTTTCAATCCCAGCCAT	59.762	20	274	616	889
60.005	20	CCGGGTCAACAGATTGAACT	59.966	20	200	409	608
59.831	21	TTACGCTTTCAAGGAATGGG	60.067	20	171	19	189
59.454	26	CCCACTTTTGGGTTATTACCTG	59.641	22	275	80	354
59.907	20	TTGTGTGATCGAACTACGTTAAA	57.469	23	107	277	383
59.813	20	AGTTACAAGCCAACCCAACG	60.03	20	269	252	520
59.515	20	CGCACTGCTGTTGGAATTT	59.856	19	236	203	438
59.989	20	CTTTGCCCCCTTTCTCTCTT	59.821	20	206	375	580
59.797	20	TGAATCAAAGCATGTCATTTCAA	60.482	23	195	919	1113
57.764	22	TAGCCCCAATTTTTGTACCC	58.807	20	237	239	475
59.325	20	TCGGGTACGACAAAACAACA	60.004	20	280	658	937
57.193	27	GGCTTCCATTACGAACCTCA	60.074	20	129	23	151
59.799	22	ACCTCCTCCTCTTCTCTCCG	59.945	20	188	12	199
59.259	20	CATGCAACCATCAAAACATCA	60.373	21	189	1	189
60.014	20	TGCGACGGAATTTAAAAAGG	60.068	20	172	165	336
60.008	20	TGACGGTGTAAATATGGGTTTTC	59.997	23	159	62	220
57.125	21	GAGTTTGTTCCATCATTCGGA	59.925	21	273	1	273
60.307	20	TTTTTATCCCATGTTTGTGCAT	59.234	22	255	232	486
57.208	27	TGCACACAAGACTTTGCCAT	60.31	20	196	938	1133
58.315	21	CAAACAAGAAAAGGAGTTAGTGG	60.529	24	225	56	280
57.85	22	GGATGATCACCATTCGAACC	60.143	20	143	1	143
60.027	20	ATCGAGTTCCCTTCCGTTTT	59.94	20	280	611	890
57.483	25	ACTGACAAAATTTCGGGACG	59.971	20	249	25	273
59.96	20	GCTAATTGGGCAGCTTCTTG	59.982	20	167	22	188
59.499	22	GGACACCAGGGTGAGAATTG	60.363	20	278	280	557
59.299	25	AACATGATACGGCCGAAAAC	59.829	20	167	0	166
59.247	20	CGCCGGTATTCGTGTAAGTT	60.018	20	180	426	605
59.726	20	GTCGACTTTTGATCGGGAAA	60.051	20	196	20	215
60.335	20	AAAAACATGCCCCACTTCAC	59.836	20	182	49	230
60.173	20	CCATGGTCGTCCAAATCTCT	59.927	20	126	228	353
59.379	20	GCGTGTATCCGCCAAAAATA	60.831	20	244	56	299
59.372	24	TACCCCAACCCACTAACACC	59.569	20	104	1	104
60.434	25	GCATGACACATGGCGTAATC	59.963	20	152	18	169
59.656	20	AACTCTCTCCCTCCTCTGCC	59.952	20	223	14	236
59.534	20	TGAGGAACCAATTATCCCGA	60.266	20	265	18	282
60.031	20	GGCCCAATACCGTGACATAC	60.081	20	239	130	368
60.071	25	AAACATGATTAACAATGATGGAA(	59.553	25	201	1821	2021
60.732	20	GGAATGAAAGGCTTAATTCTTGTG	60.344	24	272	410	681
58.175	22	TCAGATAGCCCTCTCAGACCA	59.958	21	230	24	253
61.069	20	TTCCATCTTTCAACCTCATCG	60.059	21	136	538	673
59.973	20	GGGAATTCGAAGTGGGATTT	60.131	20	242	1365	1606
59.351	20	CCGAAATGAAGAAATGAGTTCC	59.945	22	230	1412	1641
60.096	20	GAGGTTTTGACGGGATTTAGG	59.822	21	103	307	409
60.149	20	TTGATGATGGAATGAAAATGAAA	59.291	23	276	45	320
59.832	20	CAAGCATAAGGATTTTCGGA	57.845	20	268	3388	3655
60.088	19	CCAGAAGGTGCAGTGGTATG	59.161	20	213	3539	3751
59.808	20	TTCGAATCGACGGTGTATGA	60.073	20	236	5779	6014
59.83	20	TCACAAATAAGGAGGTGCCC	59.933	20	275	42	316
59.635	20	AATTGTGTGAATTCTAAATTTTCCT	57.862	26	272	318	589
59.942	20	ATTTTTCCATGTGGCATCGT	60.199	20	275	1995	2269
59.943	20	TGAGGGGCCTCACTAACAAG	60.246	20	196	810	1005
59.576	27	TGATGCTCCTCAGAAAGCAC	59.116	20	124	18	141
60.135	20	TCCCACAAAATCAACCCATT	60.029	20	246	147	392
60.073	20	CTGCTGTTCTTGGTGCAAAA	60.027	20	194	32	225
59.871	23	TTATTTGTTGCGTAGGGGCT	59.609	20	277	176	452
59.962	20	GGAGGTATATGCCACCTGGA	59.773	20	198	395	592
60.225	19	ACGTTTACGGTCCTTACCGA	59.494	20	238	375	612
60.029	20	CTTGCAACTGGTCGAAATCA	59.84	20	225	1898	2122

60.755	20	GCCTTCTTGCCTGTTTCTTG	59.993	20	252	305	556
60.019	20	GATTACATGATGCAGTGCGG	60.104	20	194	432	625
60.074	20	GGGGATGGTTATGCATGG	59.534	18	124	409	532
57.761	20	AATGGCCACAAATGTTTCCT	59.292	20	109	740	848
59.697	21	TACTGTTCTTCAGCTCGGGG	60.388	20	136	23	158
60.045	20	TTTATATCGCCGAAAATGCC	59.898	20	229	22	250
59.002	20	AATGCACCCACAAATGGAAT	60.059	20	182	695	876
59.989	20	TGTGGATTAATATAGTGGTTCAAC	57.953	26	242	136	377
57.373	24	TGAGGAGCGTTGATCTTGTG	59.984	20	133	20	152
59.923	20	CCGCCCCAATAGAGACAATA	59.916	20	254	1598	1851
60.257	20	CTCGGATTCGTCACCTTTGT	60.111	20	272	72	343
59.88	20	TTTGTGGTGACCTTGAGGAA	59.109	20	131	41	171
59.948	20	TGTTTGATTCATTCCATTTCCA	60.167	22	262	1136	1397
59.16	22	CACGACTTGCAATCCGTTTA	59.729	20	238	0	237
60.929	20	CTCCCAATCCGGTTCTATCA	59.887	20	274	3165	3438
60.264	20	ACATGATTACCAACCGGCAT	60.081	20	226	2742	2967
59.67	20	TAATGCCCCGTTTTTCTTGT	59.451	20	280	47	326
57.425	21	TGAGGAGCGTTGATCTTGTG	59.984	20	126	24	149
57.298	21	TTTGGAAACCCATGGGATATAG	59.909	22	259	843	1101
60.49	20	GACCTTAGCTTGCGACTTGC	60.162	20	192	2	193
59.988	20	GCATCAGTAAATCCTGGCCT	59.154	20	196	92	287
58.935	20	TGAAATCCATCTCCCCTGAG	60.003	20	273	487	759
58.488	25	CAGCGTAAGAAAATAATTGATGG/	59.557	24	232	10	241
60.134	20	CTCTTTCCGCACCTTCACTT	59.473	20	280	94	373
59.787	20	GTGGTGTGGTGTGAAGTTGG	59.89	20	181	96	276
60.257	20	CGTTTGGATCCGATTTGGTA	60.692	20	220	578	797
60.431	20	GACCGGGTACAGAGCAAAAA	60.11	20	179	19	197
59.899	19	CGATCATTGTGCGAATCAGT	59.679	20	176	8	183
58.743	25	TCGATTGGTTTGTGAATTGTG	59.43	21	273	2821	3093
60.269	20	TCCCTTTCGGTCTCCTAACA	59.665	20	278	59	336
59.72	20	CTCGCACCACTCCTCTTTCT	59.598	20	270	0	269
60.192	20	TCGGTTTTACCGGTTTTGAC	59.839	20	181	13	193
59.016	25	CTGTTCGGTTCGGAGAGAAG	59.982	20	166	18	183
59.992	20	GGATGAAGTTGAATTGTTGGG	59.278	21	253	62	314
59.948	20	TTTGGTGTTTGCTGTTGGAG	59.734	20	120	464	583
60.155	20	CGTGGTTCGATCGTAATTGA	59.542	20	260	0	259
60.08	20	CCCAACCCTTGAGAACTTGA	60.081	20	278	222	499
60.69	20	ATTCTAAACGCCGGTGATGT	59.457	20	104	44	147
59.992	20	GCAGGTGGATGGTGAGAAAT	59.934	20	212	245	456
59.934	20	GTGTAAAGTTGGTGGGGGTG	60.126	20	241	2644	2884
59.903	20	CCGGTGAAGTTGGATTATCG	60.323	20	247	3008	3254
58.255	20	GCAAATTTGTGGAAAAACCG	60.335	20	257	245	501
59.964	20	GTCAACTTTGGCAATGGGAT	59.797	20	223	61	283
59.572	24	GCTAAACTTTAAAGGTGATGCCA	59.692	23	201	106	306
58.81	24	ATGGGAAGGGCATTTTCAG	59.879	19	166	7	172
59.997	20	TCCTTCAAGGCCAGATTGAG	60.331	20	169	150	318
59.528	20	TTTCTCTCCAACCCACAACTC	59.173	21	278	190	467
59.562	20	TTCATCCTCTTGGCCCATTA	60.405	20	204	201	404
57.88	21	TCAAAAAACCGGTGAAAAACC	59.817	20	276	1501	1776
59.942	20	TGTTTCTCTCGTGGAAGTCCT	58.929	21	231	379	609
60.167	20	AAGATTTGATCATGGGCGTC	59.9	20	207	527	733
59.489	23	TGGACATCCTATGCAATGTG	57.907	20	274	1205	1478
59.815	21	CAGACAAATCTTCGTTATCCCA	59.087	22	153	1048	1200
59.579	20	ACGCTCGAACTCGATGATCT	59.981	20	200	609	808
59.803	20	TCAAATGGTAACCAATCACTAAGC	59.328	24	273	984	1256
59.853	20	CACCTGAATCCACTCACCCT	59.962	20	190	1424	1613
59.708	20	CAGCATACCAAGGGCATTTT	59.96	20	251	1684	1934
59.933	20	CTGAGTCATCTACAGGTCAATGG	58.732	23	229	483	711

58.149	22	CCTTTCTGAATGTGGCTGCT	60.397	20	231	17	247
59.96	20	GGTAGTGGGGAGTGGGATTT	60.052	20	157	451	607
59.756	20	TCATGTACGTTGCTTATGGCA	60.148	21	234	25	258
59.038	21	CATATGGGAAGGGCATTTTC	59.23	20	211	1135	1345
60.69	20	GTTTGGATATCGAGATCGGG	59.344	20	188	293	480
59.07	23	CCAACCCAAACTCACACACTT	59.915	21	269	36	304
59.938	20	TCTTTACCGCCTCCATCTTG	60.206	20	202	29	230
58.075	20	CAGATCTGATGGCCCAGATT	60.034	20	133	0	132
59.717	20	ACTGACATTCACAGGCATTG	57.067	20	151	21	171
59.725	20	AAAAAGTTGATGCCAAAATTAGA(	59.605	25	147	251	397
58.858	20	TTTCTGCATTCCGAATTTCC	60.016	20	132	14	145
59.272	20	CCGCTGATTTCCGTTTTCTA	60.202	20	199	475	673
59.415	20	CCATCACCAGCTTTGTTGTG	60.152	20	160	34	193
60.103	20	CATGCATGAACCTGCAATCT	59.679	20	267	834	1100
58.884	20	CATTATCGCCACAAATCTGC	59.14	20	277	858	1134
58.532	22	GAGGGTCAACCAGGAAGTTG	59.549	20	136	25	160
60.481	20	GCATGCATGTGAAGTTGCTT	59.878	20	278	74	351
59.833	20	AGGTCACGCTCGAGAAAGAG	59.745	20	236	51	286
60.103	20	AATTTGGCCCCGTCTAAAAA	60.645	20	237	88	324
59.569	20	TGTTGCTTGCCAAAATTTCA	60.229	20	185	99	283
60.221	20	TCTAAGAGATGATTGCACCTGAA	58.986	23	243	374	616
59.556	20	GGAATGTCCACTCGGTCAAA	60.903	20	280	1553	1832
59.436	20	GGCCAAGAAATATTGCTCCA	60.038	20	262	248	509
59.188	21	CCCCCATAACTCCTTTCCTG	60.674	20	142	548	689
59.805	20	TTGAATCAACCCTTATCGCC	59.901	20	192	479	670
59.95	20	CTGAAAGCCATGTTTCAGCA	59.988	20	105	606	710
59.833	20	CATCGACAAGCACAAGCAAT	59.871	20	256	1842	2097
59.966	20	TGAATGATGAGTTTTGTGGGTC	59.836	22	223	7	229
59.363	25	CACTTGGCAAAAGCAGCATA	60.014	20	260	8	267
60.855	20	GGGTATATGCCACAGGATCG	60.177	20	260	99	358
58.242	26	CGTTCGATTTGCCGTTTTAT	59.964	20	278	27	304
58.612	24	CCAGCCCAATAACTCAACCT	59.052	20	159	12	170
59.886	20	CTGATCAGCGTGTGTAAACGA	59.92	21	240	5	244
60.384	20	CCCTCGTATGCTGTGGTTTT	59.993	20	268	649	916
60.142	20	GGGCCGAGATTTGTTAGAGG	60.949	20	180	751	930
59.871	19	GCACGCTCTCCTCTCACTTT	59.751	20	186	549	734
59.679	20	CCCCCTTTTGAGAAGAAAGC	60.18	20	250	14	263
59.245	22	ACCCAGTACCACCACCATTG	60.545	20	119	3	121
60.049	20	GCATCAGAGCCACGGTTAG	59.41	19	260	56	315
57.062	27	GAGGACGATGAGGGTGAAGA	60.199	20	232	28	259
59.677	22	CATGGACCCCACATGTCATA	60.048	20	174	20	193
60.155	20	CAGATCCAACGGCCATAAGT	59.955	20	257	877	1133
59.481	20	AAAATCAAAGGCCCACAATG	59.801	20	176	35	210
60.255	20	CTCAGCATATGGGAGGGGTA	59.91	20	253	311	563
59.786	20	ACATGGTTGGTTGCTTGGTT	60.277	20	163	285	447
59.592	20	CCAAGCTTTGTGAATTTTTGAA	59.272	22	267	1271	1537
59.752	20	TCAACTCTATCATATAAATTTTTGC	57.188	26	224	9	232
59.923	20	ACCTATGGTGGTATGGCGTG	60.657	20	193	716	908
60.116	20	CGAACCCTCACACTTCTGGT	60.151	20	206	11	216
60.081	20	TCCCGCTGATTTCTTCTCTC	59.508	20	277	3538	3814
60.449	20	TTGGGTATCCAAAAACCAAAA	59.195	21	269	1540	1808
59.789	20	CAAAACATACGTTGCGCTCT	58.992	20	147	173	319
60.029	20	GCGTGAAATTATTAAGCCGC	59.722	20	244	838	1081
60.532	20	TGTTTGGCGTAAGAGACGTG	59.904	20	240	973	1212
59.803	20	AGTCACACCCTCTCCCACAC	60.006	20	269	1349	1617
57.131	27	TTGAAGTCTGCTGTGCTGCT	59.929	20	228	20	247
60.144	21	ACTGTCACACACACCACCGT	59.96	20	204	12	215
59.82	20	ACACCAAATCAAGAACCCCC	60.979	20	277	10	286

59.315	20	TCACCTCCGTCTAAATTGCC	60.074	20	241	35	275
59.933	20	CGTAAAATTCCGGTGCAAAT	59.832	20	137	821	957
57.771	26	TAGAGGACACTTGGACCTGAAA	58.837	22	100	6	105
57.176	20	ACATGCCCTAAAAACCGATG	59.823	20	223	610	832
59.092	26	AGCGAGACCTTCTATCACGC	59.603	20	191	7	197
59.885	24	TTAATGAACGGTCCGGTTTC	59.801	20	220	21	240
59.697	20	GGTACCCCCACACAAACAAC	59.989	20	209	209	417
60.074	20	TGACTCTTCTAGCTCCATTTGC	58.745	22	125	2837	2961
60.149	20	CAAGGGGTCAAAGAAGGTTG	59.563	20	236	66	301
60.003	20	TTGGATGTAGCCCCATCATT	60.155	20	184	545	728
59.61	20	AGCCACTGAACCAAATGACC	59.973	20	265	2110	2374
60.005	20	GATGACGATGACGATGATGG	59.882	20	187	2049	2235
59.34	20	GCGATCGTTCACACACAATC	60.128	20	106	394	499
59.953	20	TCCGTCATCTATTAGCACCATC	59.07	22	137	106	242
59.598	20	CACTACTAGGGTTTGGGAGGG	59.866	21	215	97	311
58.909	20	CAACTGAGTTCCCTACCCGA	60.103	20	257	201	457
59.953	20	TCAATTCTTCATCGCTGCTG	60.096	20	280	1645	1924
60.258	20	TACAAGTGCAAGAAGCGGTG	60.05	20	209	909	1117
60.69	20	TGCTGGTACCTTCGGAACTC	60.255	20	188	68	255
60.02	20	CAAGCGAAAATGATCGGATT	60.038	20	200	2127	2326
59.165	23	AAAAGTTCACTAAAACTGCCTTCT	57.086	24	198	95	292
59.907	20	TTGTGTGATCGAACCACGTT	60.008	20	108	24	131
59.95	20	AATCGCCATAGCAAGAGAGC	59.579	20	127	1031	1157
58.816	23	CCACCGAGCTTCTACACGTT	60.312	20	188	6	193
57.467	22	ATGTTGGCTCCGATTGAAAC	59.939	20	276	29	304
60.249	20	ATATGTTGCCCAAGTGGGAG	59.813	20	214	149	362
59.189	23	TTGGCCTGTCTGCTATCATTT	59.722	21	192	23	214
60.039	20	TTGAAGCCTTGTTCCTTGCT	59.993	20	209	167	375
59.844	20	TAAGGTGGTGACGATAGGGC	59.955	20	201	398	598
60.053	20	GACAGCTGTGATGAAGGCAA	59.992	20	184	2712	2895
59.586	20	AGAGGAGAGGAGGTTGGAGC	59.952	20	210	48	257
60.104	20	GAAGATTGTGGGGCGAGTAA	60.074	20	225	1719	1943
59.934	20	CTCATGGATCAGGAATCTGGA	60.019	21	262	442	703
60.11	20	TTTGGTAAATACAACATAATTGGT	58.757	26	245	278	522
59.783	20	ATTGCATTGCATGTGTGGAC	60.409	20	278	396	673
60.054	20	AAGGAGGTGTTATGAAGGGATT	58.028	22	148	1416	1563
59.679	20	CGCGACCACGAAGTACATAA	59.752	20	244	69	312
60.331	20	TGAGCAAAAGTAATAAGAGCAAC	58.341	24	206	226	431
60.713	21	TGACTCTGCGTTTGTTGGAC	59.88	20	195	263	457
58.792	22	AATCCCAAAATCTATGGGGC	59.984	20	183	20	202
58.874	20	GCAGCCACTATCCTTTCACC	59.7	20	198	678	875
59.756	22	CAATTTCAAGAACCCCCAAA	59.772	20	240	5	244
59.455	22	TCATTATTCCGTCGATCGCT	60.574	20	181	2	182
59.875	20	CCAAAATCCCAGAACGAAAA	59.91	20	194	115	308
57.719	22	ATCCAATATGTGGGGCAAAA	60.016	20	263	2	264
59.872	20	AGAAGACGCTCACACAGGCT	60.205	20	185	33	217
60.003	20	TCCATTTTCCTCACCCCATA	60.126	20	219	334	552
59.435	21	TGGTAGGTGGGTTTAGGTGG	59.705	20	130	20	149
59.483	22	TGGTCAATTCACAATCCGAA	59.9	20	265	71	335
60.008	20	TTTTGAATCTTTGTCCCCAA	58.039	20	144	364	507
60.285	20	TCACCTAAACCACTTTTGAAGAA	57.979	23	238	68	305
59.42	22	CGTGGTTCGATCGTAATTGA	59.542	20	100	1	100
59.577	20	GGTTCGAGTCTCAATGGGAA	60.05	20	277	793	1069
59.978	20	TCAATTGTCACACGTGATTGAG	59.615	22	209	1028	1236
59.888	20	TCTGGACACATCCTTGTTTATCA	59.485	23	209	1076	1284
58.079	23	TCATCTTTTGGGAATGGTTTG	59.787	21	239	220	458
58.527	26	ACCTCATGGGGAAATTTTAGC	59.324	21	100	1	100
60.166	20	CTCTGTTCCACGCACACCT	59.881	19	224	19	242

60.495	20	GGTGGAGCATGGGAAAAATA	59.762	20	134	967	1100
60.203	20	CTTTCCTCAACCGAGCTCAC	59.989	20	252	16	267
59.966	20	GAGAAAGCCACAAATCTTTCC	57.916	21	262	3612	3873
59.948	20	AAATCAAAAGGCCCACAATG	59.801	20	153	281	433
59.973	20	TAGGGATCGCTCCAGAAAAA	59.778	20	146	2094	2239
58.221	25	AAATGCGCAATTTTAAACCC	58.982	20	246	150	395
59.984	20	CTTCCACTCCCACCAACCTA	59.959	20	245	257	501
58.045	24	TCTCATTTACACCGCCTCAA	59.272	20	274	699	972
60.303	22	AGTTTTGACATGAAAATTGAATGA	57.739	24	244	480	723
60.076	26	TTCCTTCATTCCTAAATGATTAATT	58.94	26	277	668	944
59.405	20	GCAGTACTTGCCGAATCCAT	60.103	20	277	43	319
59.894	20	GCCCCAATTATTTTAGTCCCA	60.029	21	253	318	570
59.978	20	CAGATCGACCGATCCACTTT	60.073	20	217	83	299
60.032	20	AAAGTGGAATTTGCTTCCCA	59.546	20	238	196	433
59.76	21	GCACATGCAACCTCAGAGAA	59.992	20	236	124	359
59.857	23	CCTGGGACACATAAGCCTCA	61.055	20	257	495	751
59.82	20	TGAAATGAAATCTTGGCCGT	60.448	20	275	171	445
60.181	20	ACATGTGATGTGATCCCCAA	59.611	20	116	1138	1253
60.008	20	TCGCATCATCAACCTTCTGA	60.353	20	223	594	816
60.132	20	GCTAGCTCGGGAGAGAGGAT	60.081	20	271	223	493
60.343	20	GACTAGCACGTGACCGGATT	60.142	20	262	252	513
60.393	20	CCAACCCAGAATTCGTCCTA	59.926	20	211	980	1190
59.918	19	TGTAGTTGATGGACGTGATTGTT	59.415	23	113	4	116
59.926	20	CACATCGACGACGCTACCTA	59.888	20	186	228	413
59.834	20	AAGGCCATTTCATCTTCAGC	59.273	20	200	813	1012
58.909	24	GGAAATAAGGAGGTGTTATGAAG	59.323	24	186	1222	1407
60.164	20	TGGACAAAATGAGTGGAGAGAA	59.711	22	203	1055	1257
59.993	20	AGCTCGAGGCTCAAAACGTA	60.154	20	271	870	1140
60.237	20	ATGGGTGTGGGTGTTAGTGG	60.545	20	127	271	397
60.497	20	TTTGTCGGGTCATTGCTACA	60.111	20	184	827	1010
59.903	20	TAATGGTGATGCCAGGGTTT	60.192	20	158	1220	1377
60.173	20	GGGAATGAGGGATGGAATTT	59.959	20	161	2290	2450
60.201	21	CCCGACTCGAGATTACCAAA	60.066	20	152	100	251
60.096	20	GTTGGTTGATGAGGTGGCTT	59.973	20	218	611	828
59.282	20	GGAGACGACCTTGCTCACTC	59.993	20	197	132	328
60.025	20	CCATCTCCAATTCGTCACCT	59.927	20	186	328	513
59.767	20	ATGGACAGGTGGCGTTAATC	59.82	20	254	592	845
59.2	20	CGTATACCCTCAAACATCCGA	59.831	21	176	790	965
60.073	20	TGCATGGTTTGAGTTTTGTGA	60.142	21	161	16	176
60.119	20	ACGGTACCACCCCAATACAA	59.971	20	112	2273	2384
59.801	20	CCCCCATCAATCATGAAAAC	59.991	20	219	535	753
59.806	20	CTCCTCTCCTATTCGCTCCC	60.306	20	261	150	410
59.641	22	AATCACAGCATTCAAAGGGC	60.081	20	184	18	201
59.572	20	TTCTCCATTTGTCTCCCTGC	60.195	20	193	2251	2443
60.149	20	GAACCACCACACCAAATTCC	60.073	20	221	397	617
60.234	20	CCATGCCCCAGTAATCATCT	59.773	20	219	118	336
59.849	21	TAAATAAGTTGCCAACCGCC	59.964	20	116	5	120
60.049	20	GCCAAGGATGAGAATGAGGA	60.158	20	202	60	261
59.532	20	CACAAACCATCATTGCAACC	59.823	20	270	971	1240
59.415	24	TGGACTGAACTGCAATGCTC	59.992	20	202	990	1191
60.223	20	CAGAGTGATGCGTGAAGGAA	59.984	20	195	334	528
59.805	20	GGTTACCGAACCGAACTGAA	59.971	20	264	154	417
59.048	23	AGTGGGCTTTGGCACAAGTA	60.689	20	114	1	114
59.072	20	TTACATCTGAAAATTTGACGCA	58.355	22	260	157	416
59.967	20	GGGGTTATCACTGGATCACG	60.195	20	187	509	695
60.991	20	AACCATCTTCGGAGAGAGCA	59.95	20	115	249	363
59.953	20	TCCATGACAGGAACCAAACA	59.935	20	212	1297	1508
59.95	20	AAGCTCATCCATTGGTTTGG	59.933	20	258	233	490

59.189	22	TGGCAGAAAGATTTTAGATATAGA	59.31	26	133	214	346
60.173	21	TTTCGAAGATCAACGAACGA	59.395	20	123	223	345
60.439	20	AGACCGAAACCGTTCAAAAA	59.587	20	258	633	890
59.792	20	CCACCAAGTCAGCACCTTTT	60.149	20	243	1073	1315
60.159	20	CGGCAAGGAGAAAGAGGATA	59.406	20	271	82	352
60.038	20	AACATGTAACCTGTAATCTGTAAA	57.047	27	226	182	407
60.02	20	CGGAAAACGACACCAGAAAT	59.971	20	164	49	212
59.96	20	CGGCCTAAATATCCCTTTCC	59.767	20	262	763	1024
59.574	20	ACGGGAGCATGAAATGAAAC	59.939	20	204	554	757
59.985	22	CCAATTTCGATTTCCTCCAC	59.363	20	272	0	271
59.575	20	TCAGCGTGAAATGTGGGTAA	60.111	20	224	1230	1453
59.931	20	GCGGCCACGTATTCTCTTTA	60.23	20	176	579	754
59.971	20	CATACATTCACACGGGGGAG	61.176	20	136	2377	2512
59.84	20	TCATCGACCCCATCTCTTTC	60.011	20	137	389	525
59.975	20	GACTTGAGCACCAAGAATCCA	60.248	21	143	82	224
59.7	20	GATTATGCAAGCCCAGCCTA	60.196	20	159	414	572
57.381	21	TCAAAATAGGCATAACCGATCC	60.168	22	150	46	195
60.009	20	ATGCAATCTAGTTGACGGGC	60.103	20	205	17	221
60.038	20	TGAATCGATTAGGCTTATCTTGA	58.055	23	146	87	232
57.658	22	GAAGTTGGGGGTGGAATACA	59.647	20	271	393	663
59.955	20	CGATGACCTTGAGGTTCGTT	60.111	20	227	3393	3619
60.096	20	GATGTGATACCCGGAGTCGT	59.81	20	167	31	197
58.86	26	AGGACACGTTGGACCTGAAG	60.151	20	105	24	128
59.88	20	TGGTTGTATGTCATTTAGCGG	58.57	21	247	303	549
59.829	20	GGCTTTCACATTAAGCCCTG	59.708	20	113	6530	6642
60.903	20	CAATGGTGGTATGGTGTGGG	61.898	20	105	18	122
60.02	22	ACCAAATAGGGTAAGCCGTG	58.973	20	241	1178	1418
59.823	20	ATCGGGCATTTCGACTACAG	60.096	20	160	830	989
59.536	20	GACAGCCCAAGATTGAGAGG	59.803	20	274	9	282
60.068	21	TCCAAGGCAAATTGAACTCC	60.051	20	258	229	486
60.24	20	AGCGACAAAGTGTGATGACG	59.905	20	169	120	288
59.569	20	GAATTGGTGGAAAGATTTGAGAA	59.472	23	244	15	258
59.487	22	CTATTTTGTGGCGCTGGAAT	60.096	20	256	22	277
59.982	20	CCCACCAACCCAGATCATAG	60.187	20	279	2448	2726
59.971	20	TGTGGTGTGAGGGCTGTAAA	60.152	20	220	2000	2219
59.248	20	CAGCACGCGCACTACATAAT	59.925	20	273	145	417
59.79	20	CCACACCTCTTGGACCAAAT	59.82	20	279	790	1068
59.835	22	TCTCCCTTTCCTCTCTCGTG	59.525	20	169	442	610
59.317	20	AACTCAGTTGGGTTGTTGCC	60.012	20	265	1110	1374
60.123	20	CCCACCAGTAGTCTGCCACT	60.174	20	230	3447	3676
60.545	20	TGCCATGATCATTGATTGGT	59.738	20	270	873	1142
59.992	20	CCCAACCAACAAGAACCCTA	59.824	20	117	455	571
59.775	20	CCCAGTTGCTCACAATGACA	60.722	20	200	374	573
58.792	22	AATCCCAAAATCTATGGGGC	59.984	20	199	20	218
59.993	20	TGGTAGGGTGGTAAACCAGG	59.705	20	102	210	311
59.942	20	CAATTCTCTCAACCAACCCC	59.381	20	191	30	220
59.226	20	CATCATGTAGTAGCGGGCTG	59.322	20	146	42	187
60.469	20	TCGTCGTCGTCAAACAAGAC	59.88	20	184	30	213
60.016	20	TGGGTTTGGTGTTGAATCCT	60.21	20	275	104	378
59.187	24	TGGACCGTTGAAATACAGAAAA	59.492	22	152	24	175
59.31	20	CGAAGTCGAAGCCTAACTCG	60.147	20	271	28	298
59.823	20	ATTGCCACAACTCCACCTTC	59.973	20	145	1832	1976
59.716	20	AGCCCACCTGATATGCTTCA	60.624	20	182	24	205
59.556	20	ATTCGAACCAAAATGCGATG	60.837	20	206	493	698
59.964	20	TGACAAAATATGGAACCACAAGA	59.37	23	156	23	178
60.134	20	AGAGCAGCAACAGTGCAGAA	59.929	20	164	204	367
60.014	20	GCGCGCAATCACTCTCTT	60.257	18	117	865	981
59.71	21	ATGCGGTTCAAAGGTAGGAA	59.569	20	214	36	249

59.627	20	CATCCTCCTTTTCCCAATCA	59.864	20	275	24	298
60.123	21	GGTTGACACACTCGAACCAA	59.571	20	225	5	229
59.445	20	GGTCCGAATGCTGGTATGTT	59.82	20	159	3143	3301
60.122	23	GGGGATTTGATTGATGATCG	60.096	20	280	97	376
58.074	21	TTAGACTGCGGATCACCACA	60.263	20	242	256	497
60.022	20	TTTGTCCCCACAAATGTCCT	60.21	20	244	2376	2619
59.904	20	GAAGCAGAGCATTGATGGGT	60.226	20	267	169	435
60.169	20	GACGCCACATCATTCTCTCA	59.794	20	280	4100	4379
59.933	20	TGACATGTCCTTTCACTCCAA	59.124	21	226	3126	3351
59.319	23	ATTTAGGCCACACAGGATCG	59.955	20	100	59	158
59.997	20	TCATTAAACCCGAATCCGTC	59.762	20	186	504	689
59.995	20	GGCACAGCTCCACTTTCTTC	59.997	20	198	117	314
59.67	21	CAGCCTTATCTCTGGCCGT	60.368	19	278	321	598
60.475	20	CAGAAAGTGCTTGTTCAGCG	59.781	20	163	1163	1325
59.745	20	CTCTGCGACGTAATTCCCAT	60.096	20	266	90	355
59.986	20	GACCAACTCTACTGGTGAAGGC	60.175	22	197	439	635
59.997	20	ACGCGATTATGGAAAAATGC	59.935	20	236	269	504
57.083	20	CGTGTTGTTTTGCTTGTTTCA	59.801	21	130	20	149
60.089	20	GGGTTCAATGTACGCAACCT	59.859	20	272	236	507
57.552	24	AGGAAGGATTAGGCGAAGGA	60.166	20	224	13	236
59.912	20	TGGCATTTCAGCTTTTGTAGC	60.391	21	145	96	240
60.038	20	TTGGTTCACACAACCACTCC	59.415	20	177	72	248
59.284	20	CGCTTTCGCCTCTTTATTTG	59.979	20	148	3	150
59.955	20	GGCTCACTCTCTGGCTCATC	60.104	20	242	1451	1692
60.051	20	CTTGGAGCTGTTTTCTTGGC	59.993	20	158	2641	2798
59.845	20	TGAAATACAAAAAGGGTGCG	58.674	20	253	687	939
60.587	20	CGGAGCAACCACCGTATAAT	59.845	20	279	67	345
60.209	20	GGGATTTGCTGCTGTTGTTC	60.646	20	233	3	235
60.257	20	CACATGTCATGAATCGGTGT	57.712	20	227	44	270
59.321	23	TGCGTCACACGTAATTCAGA	58.863	20	278	776	1053
59.604	20	GGGCCGAGATTTGTTAGAGG	60.949	20	279	926	1204
60.074	20	CGGTACGGTAAGTGCGGTAT	59.904	20	134	595	728
58.241	20	TCACACCATATTTTTCCCCC	59.481	20	219	755	973
60.08	20	TGCGTGTCTAAATGTGCCTC	59.871	20	142	379	520
59.569	20	AGCGTTGGATTTTTCAATGG	59.938	20	159	21	179
59.948	21	TGATCTTTTCGCCATTAGAGAG	58.605	22	107	209	315
59.829	20	TCCCAGTCTAAAAGTCCATGC	59.184	21	204	172	375
60.365	20	TCTCTCCCGACACACACGTA	60.309	20	116	100	215
60.11	20	TCAAGTCGCTACTCACCATCA	59.455	21	115	887	1001
59.945	20	ACAATGGAACGAGATCCAGG	59.927	20	198	323	520
60.073	20	TTTTTCTTGCTTTTGCTGTGAA	60.034	22	230	27	256
59.069	21	AAACAGGGGTAGCTGGGAAT	59.827	20	187	15	201
57.862	23	CAAGCTCAGTCTCAATGGGG	60.791	20	132	233	364
60.184	20	CTCTAGGGTTCAATTGGGCA	60.066	20	101	3000	3100
59.934	20	TTGCGATAGTTAACAGGCAGAA	59.911	22	162	359	520
59.978	20	ACGCTGCCTCAAAAGCTAAA	60.152	20	232	689	920
57.978	23	TTTTGGTGTCTAATCCATTTGTG	58.909	23	135	46	180
58.886	20	CCCAAGGTACATTGCGTCAT	60.776	20	233	575	807
58.374	21	TGAGAAACCGTAACCCAAGC	60.11	20	239	10	248
59.892	21	GCTGCAGCAATAAAATTCCC	59.688	20	229	2705	2933
60.067	20	TCGCTCTGAACCAAAAACCT	59.853	20	189	279	467
59.895	20	TGCACTCATCAGAGTTATCGG	58.888	21	161	3162	3322
59.992	20	ATTCAAACACGAGTCGCAAA	59.322	20	244	313	556
59.903	20	CAGGAGGGTTCACACAACCT	60.002	20	126	229	354
60.55	20	TTGGGGCTTGTAATAGCTGG	60.089	20	270	7154	7423
60.246	20	CATCTTCAACCCGTCACCTT	59.966	20	175	318	492
59.699	20	ATTGATATGGCACAGCAGCA	60.251	20	279	224	502
59.955	20	ATGCCAACACAAAAACACCA	59.864	20	213	189	401

59.562	21	TTGATTACGAATCCTCGCCT	59.668	20	270	83	352
59.943	20	TTAGTGTGGCCGGTTTAATTG	59.876	21	172	91	262
60.096	20	CCGATATAAGCGCCATCCTA	60.04	20	190	292	481
59.717	20	CCACGTACATCTTGTGTCCA	57.946	20	193	1050	1242
60.112	20	GCCAGCTTCCTCTCAATCAG	60.096	20	242	2204	2445
59.997	20	TGCAATTTGTTTCTCTCCCA	59.247	20	195	1739	1933
60.032	20	TGTGGATATGTAGATAATTTTTGG/	57.748	26	240	60	299
58.973	20	TGGTAAAGGGACGGCATTAG	59.953	20	129	770	898
59.665	24	TCCTTCTTCACTTTTGCTCACA	60.03	22	148	10	157
60.136	20	ATCCTTCAACTGCAACAGCC	60.263	20	195	1498	1692
60.051	20	GGCCCGAGCTAAAAACATTG	61.816	20	267	38	304
58.93	20	CCTCCCAACTTGTGTGTGTG	60.039	20	196	0	195
60.328	20	CCATGTCTCTGGACTGGGAT	59.92	20	247	3446	3692
59.677	21	TCTTTTATGTGCACAATGCG	58.354	20	170	359	528
57.223	20	CCCCAGAATGCAGTCAACTA	58.722	20	128	77	204
58.743	25	TCCTCCTCTTCCACTTCCAC	59.228	20	189	62	250
60.31	20	CCAGTAGATGCCCCTCCTAA	59.146	20	278	450	727
60.021	20	TCTCGCAGATCAACGTCAAC	59.992	20	276	6	281
59.797	20	TTAACTTCCTCCAGCTCCGA	59.948	20	128	103	230
60.762	20	TTCGCATAATTTTTGTGAGAGG	59.253	22	242	56	297
59.923	20	TCGTTGCTCTCCATATTCCC	60.036	20	131	324	454
59.939	20	GATTTGGAACTCCGGTGAGA	60.05	20	204	1443	1646
60.103	20	TGTGACTGCTCTGGTTGAGG	60.022	20	106	15	120
60.008	20	CGATTTTGGGTTTGTTTGCT	59.975	20	265	539	803
60.218	20	CCCTCTCTTTTGGCCTCTCT	59.95	20	122	1525	1646
60.04	21	CTGTATGGGCCTTCGTCCTA	60.088	20	269	2810	3078
60.096	20	CATGCGCCTAGATAACGACA	59.856	20	228	700	927
60.039	20	CGCAGATATGCGTTTCTCAT	58.897	20	166	20	185
59.505	20	ATATACCGGTGCAAACGAGC	59.988	20	210	1162	1371
60.143	20	TGGTTGTGGTCAATGGAGAA	59.935	20	196	432	627
59.679	20	TCAGCAGAGCAGCCATATCA	60.675	20	108	155	262
59.312	20	CCTCAAATTGTTGAAAAACCG	59.478	21	224	413	636
59.83	20	CCGTCCATCTCAAAAATGAA	58.546	20	214	898	1111
59.775	25	CAGCACCAGATTTGCACCTA	59.864	20	227	278	504
58.652	22	TGAATTTCAGTCATGGATTCTCTT	59.136	24	166	24	189
59.939	20	GCATACACACTCCGCATTTG	60.142	20	239	5	243
59.878	20	GGCCATTCAAATTTCAAATCA	59.765	21	235	307	541
61.278	20	GTGGACGCTCCAGATTGTTT	60.119	20	169	1	169
59.694	20	CGAATATTCAGACGAAGCCC	59.668	20	274	92	365
59.789	20	AACGGGATTTACTGTTTTGACA	58.539	22	262	177	438
59.966	20	AGCCCGTACCCGGTCTATAA	60.702	20	205	76	280
59.563	20	ATTTGGTGCATTGATTTCGC	60.847	20	173	658	830
60.008	20	ATCCCCGACAAATTTTCACA	60.17	20	106	61	166
60.022	20	GACAAATCCAAGTGGGAGGA	59.903	20	142	1888	2029
59.934	20	CCATTTTCATTTCGGGCTAA	59.901	20	238	353	590
59.375	20	GGGAAGAGGGGGTATGAGAG	59.89	20	244	143	386
59.957	20	CAGTCGGGGGTAACACTCTT	59.062	20	108	2155	2262
60.118	20	CCCTAGCTTTGCCCTCTTTC	60.332	20	270	595	864
59.939	20	TAAACCCTAAAAACGCGTGC	60.13	20	243	19	261
59.697	20	ACAAGCCCTAGCTCTTTCCC	59.848	20	102	103	204
59.562	20	TTTCTGTTTTTGACAGCCGA	59.439	20	182	5	186
59.647	20	AGCACTCTGCGAGTCTCCTT	59.355	20	278	69	346
59.691	20	TTCGAGTGACACAAGGCAAG	60.025	20	262	9	270
60.003	20	AATCGCTGTATCTCCATCGC	60.207	20	180	1485	1664
59.551	20	AAGGTGTCGCAATCTCCGTA	60.657	20	115	349	463
59.69	21	CTTTTTGCCTTTTGCTGGAC	59.861	20	268	344	611
60.066	20	GTGCTGATGGTTTTGCATTG	60.119	20	156	72	227
60.049	20	CCCGACAGTGAATCGTACCT	59.989	20	137	30	166

59.933	20	GCCGAAAACAAAGTCCAGTT	59.218	20	274	28	301
60.125	20	CCTTATTTCCTATTCCCGCC	59.767	20	136	598	733
59.184	21	TTCATATACACGTGCGTCTTTT	57.425	22	158	586	743
59.79	20	GGCTGTGCTTTCTGGTTCAT	60.263	20	266	53	318
60	20	GTTAGGCCTTGGAATCGTGA	60.074	20	280	1428	1707
59.713	20	GCTTGTTACCGGTGGAGAAA	60.11	20	189	34	222
60.022	20	CCCCCAACACACTCAACA	58.839	18	108	988	1095
58.672	20	GGACGAGATTTAACCCCACA	59.79	20	199	31	229
60.112	20	CGCAGGAGGACAAAAGAAAG	59.986	20	231	2496	2726
59.758	22	TCAAAGGAGGATTGTGTTAAGGA	59.987	23	132	60	191
59.874	20	GACAGCCTTTTTGTTTTGCC	59.73	20	194	22	215
60.665	20	TGTTTGAGATTCAGTGGGGA	59.059	20	248	2666	2913
60.209	26	AATACGGCTGCGTAAACCAC	60.025	20	273	12	284
59.983	20	CCCACGTAGCAATTGACAAA	59.585	20	259	688	946
60.074	20	GGGTTGACATGGCTTCGTAT	59.82	20	155	151	305
59.032	26	ATGCTTTCCCTCTCCATCAA	59.629	20	112	80	191
60.015	20	ATATTCGAGTCATCCGCAGC	60.207	20	263	2703	2965
60.144	20	GTAACACCAACCACACAGCG	60.072	20	192	169	360
59.844	20	GAAACACGAACGGATTCTTGA	60.103	21	209	91	299
60.006	20	ATCATAGTTGACGCCCTTCG	60.096	20	171	94	264
59.027	20	TCAAAACTCACCGGAATCCT	59.526	20	259	1455	1713
59.933	20	GATGCGATCACTGCCCTATT	60.066	20	207	667	873
57.575	24	CATGTGTTGCTTAGGTGGCA	60.72	20	246	1745	1990
59.992	20	GTCAACTTGGGCAGTGGAAT	59.973	20	132	3056	3187
59.831	20	AGCGAATTTCTCTTGACGTG	58.104	20	242	1145	1386
59.982	20	CCAGAAGGATTCTTCGGCTA	59.406	20	269	136	404
60.011	20	CCGGTCCTGAGTTTGTGTTT	60.005	20	272	597	868
60.142	20	CGCGTAGAGTTACCAAACCC	59.632	20	127	98	224
60.046	20	GATTTTGCCTTGCATGTTGA	59.67	20	230	1919	2148
60.125	20	ACACGAGTGTGGAAAGGGAG	60.151	20	221	70	290
59.673	20	ATGGGTATGGATCTATGGCG	59.628	20	274	275	548
58.388	25	CGGGATTTGGTCTTTTCCTT	60.291	20	229	765	993
60.453	20	CTTCTCCACGTCATTTGGCT	60.255	20	251	136	386
60.058	20	ATGCCTCCACATCCTCAAAC	59.934	20	256	312	567
58.627	20	TTTTGAAGTGTTTCGGGGAA	60.452	20	274	1281	1554
58.852	22	GAGGCCTTGAGGCAGACTTA	59.574	20	279	845	1123
59.955	20	GGCTGGTATCACCATAACGC	60.361	20	119	90	208
60.109	25	AGGCTGTGAAAGCTCCATCT	59.043	20	147	41	187
59.844	20	TGTTATATGGCTGGGGCTAAA	59.464	21	160	177	336
59.945	20	TGCAACACCAAGTAACCATCA	60.019	21	148	210	357
59.673	20	TAGGCACAAGTTCGAAACCC	60.11	20	268	23	290
60.262	20	ATTTCATTTCATGGGTTTCG	57.399	20	159	34	192
59.925	20	CACGAGTGAGAAAGGGAGAGA	59.583	21	181	84	264
59.971	20	GGTTCGGTTTTTCGGTTTTC	60.683	20	203	179	381
59.505	20	ATGCAGCAGAGCCAATTTTT	59.851	20	206	4	209
57.4	22	AATTTCCCTGCTTTCCAGTTT	59.123	21	276	449	724
60.203	20	TGCAGGTTGGAATCAGATCA	60.201	20	115	74	188
59.834	20	TTTGGGTTTCTGGGTGTGTT	60.246	20	267	796	1062
60.483	20	TGCTGAATCTTAACCGTCCC	60.074	20	133	761	893
59.467	20	CGTTTTGCTTTAGGTTACAAGGA	59.722	23	261	222	482
60.301	20	TCAACCACCAACATTGTTTCTC	59.875	22	151	233	383
59.982	20	TTTTCCCTCTTCTGGGATCA	59.6	20	253	407	659
59.663	21	GTTGAGGAAGAAGAGCGGTG	59.989	20	176	142	317
60.894	20	CATCATCCTCCTCTTCTCGG	59.755	20	221	321	541
59.764	20	TATTCCCTCAGACCACTCGC	60.218	20	280	819	1098
60.082	23	AGCTGGTTACCTTCGAGCAA	60.015	20	196	26	221
59.492	22	TCTTGTTTCAGGTTATCGTCTAAA	57.253	24	218	43	260
60.088	20	AATTTTTCGCTAAAGGGGGA	59.909	20	203	578	780

59.518	20	CCAGGAAGGCTCTTCACAAG	59.982	20	171	3573	3743
59.933	20	TCTTGGGTTGTCTTGATCCC	59.903	20	188	592	779
59.561	22	CAGCTTGCATGTAAGGTCCA	59.864	20	195	470	664
59.711	20	GAAAACCCAAAACACATGCC	60.214	20	244	3	246
60.073	20	GATGATGATGGCTTATGGGG	60.118	20	143	88	230
59.175	22	TCAAGTCAACATAAACATAAAAA(	58.978	26	136	93	228
57.552	24	GTTTTGGCTCCGTATGCTTG	60.637	20	144	18	161
60.067	20	TCGTGATTCGCAACTCTGAT	59.399	20	275	71	345
59.956	20	GTAATGTCAAATGCGCAACG	60.14	20	168	240	407
60.214	20	ATGGGTTAGCGTGTGATCGT	60.406	20	135	327	461
58.593	21	TTCAAAAATCAAAAGGTCGAA	57.419	21	272	3	274
60.068	21	GGGCCGAGATTTGTTAGAGG	60.949	20	169	1565	1733
59.836	20	CATATATTTCATTGGCCCCG	59.998	20	146	1452	1597
59.964	20	GCACTACATAAAATGAACATTCTC	58.234	25	269	1530	1798
59.91	20	CCACAAACCCTTCATCACCT	59.82	20	126	31	156
59.222	20	ATGTGGAGACGAGATGGGAC	59.928	20	174	1160	1333
60.153	20	TTTATCTTGCCCATGGAACG	60.827	20	239	5619	5857
60.298	21	TCCAGCTCTTCCAACCAAAC	60.232	20	168	306	473
60.234	20	GGGTTTTAAATTGGGGATTTTG	60.588	22	204	142	345
60.043	20	TTAGCCGATGAGAGGAGGAA	59.91	20	272	4676	4947
60.857	20	TCTTTTTCTTCGTCACGGGT	59.711	20	215	663	877
57.584	20	TACGAAGGAGGCTTGGAAAA	59.817	20	248	1020	1267
58.773	20	AAACAATCAACCAAGGATGACA	59.336	22	216	1765	1980
59.894	20	GATTCTCGCAGATCAACGGT	60.226	20	239	549	787
60.19	22	TGAATTCATCACCTCTACCGT	57.126	21	151	340	490
59.904	20	AGAGTGAAGCAAGGGAACCA	59.844	20	276	545	820
59.579	20	TGTGGCTATTTTAGCTCTTTGGA	60.257	23	156	8	163
59.847	20	CAATATTACAATGGGCAAGAGTC	57.725	23	253	1535	1787
60.194	26	CATGGCTGTCAGATCTCGTG	60.426	20	145	0	144
59.022	20	CACCTTGCTATGCAATACGG	59.206	20	280	96	375
60.27	20	CGAGAAGGAGAAACGTGGTC	59.844	20	250	194	443
60.264	20	TGCTTGTTGGCACCAGTAGA	60.449	20	139	262	400
60.343	20	CTGTGACGGTGCGTGTCTAT	59.776	20	157	138	294
60.126	20	AATTCTTTGAGGCGCAGAAA	59.96	20	264	2030	2293
60.17	20	CCATGCACTTCTTTCTTTGC	58.502	20	265	230	494
60.119	20	TTCCTGTTATCTGCCCAGAAG	59.322	21	179	719	897
60.051	20	GATCAGGAACCATTTGTGGG	60.173	20	183	1383	1565
58.161	20	GGCACCGACATCAAGTTGTA	59.572	20	258	93	350
60.065	20	GGGTGGGTTTGGAAAAGAGT	60.204	20	253	603	855
59.51	20	GCGTGTTCTTGGTGTTCTTG	59.339	20	218	89	306
59.359	20	ACAAATTCGAGATCGAACGG	60.074	20	269	563	831
58.799	20	AGCATCTGGAATGCAGGAAG	60.362	20	144	778	921
59.935	20	AAGGGCTTGGAACATGACAC	59.973	20	202	23	224
59.667	23	TGAAATAGGAGAATAGGGGCG	60.406	21	199	57	255
60.066	20	GAGAAAAGGGAAGCCTCAAGA	59.948	21	232	184	415
59.992	20	ATCCTCATCTTCACCATCGC	60.042	20	131	592	722
60.268	20	GCAAATCCGAGTAGCTTGGT	59.34	20	241	226	466
60.118	20	CGAAACAATAAGATGCTGTGTGA	60.18	23	159	111	269
59.458	21	ATGGAATTGGGTGTTGGATG	60.439	20	152	24	175
60.009	20	TTCACACAACCACTCCACAAA	60.033	21	121	274	394
60.102	22	CACCCGTATGGGACAAAATC	60.051	20	177	231	407
59.813	20	GGAGGATTTTCCTTTCCCAA	60.237	20	193	19	211
60.34	20	CGTGGACAGTTTCCCAAAGT	60.005	20	238	15	252
59.547	20	ATTACTCACACGCACACCCA	60.032	20	172	170	341
57.436	21	AAAGCCCCGATTTCGTATCT	59.93	20	184	0	183
60	20	TCTTGTGAGCCTGTGTTTGC	60.032	20	145	65	209
59.853	20	GCGGATAATCTGGAGATGGA	59.998	20	175	2658	2832
60.159	20	AACTTCTTGTCATCGCCACC	60.119	20	259	396	654

60.061	20	CACCAAATTTGAGGGTTATTATTT(	59.905	25	229	147	375
59.967	20	GGGTATTTCTGGAAATCATTGAA	59.22	23	197	68	264
59.259	21	GTGGTAATTTTCGCACCCAC	60.235	20	276	150	425
60.112	20	CGAAAAAGAAGAGGTGAGCG	60.125	20	277	1088	1364
60.535	20	AAAGGGCATAATCTATGATCGG	59.339	22	175	44	218
60.768	20	TGTGTTTTTCAGTGTGTGGGA	60.033	21	132	74	205
60.202	20	AATTGCACGGTAGTTTGCAC	58.703	20	269	1589	1857
59.964	19	CAGATGTGGACGGGTCACTA	59.545	20	280	19	298
60.285	20	TGGCATTTTCGATCTCTGTG	59.799	20	179	73	251
59.741	21	AGGTTCAGACCCAGGCTTTT	60.11	20	222	158	379
58.926	20	GCAGGAAGTGATGAAGAGAACA	59.478	22	268	107	374
57.626	21	TGGTGGTTTTCAGATGCTCA	60.24	20	211	52	262
59.215	20	TCTAATTGGGATCTCCACGC	60.036	20	254	157	410
59.82	20	CGAAGCATTTTGATTTGCGT	61.131	20	242	127	368
60.228	25	GCCATTGTGAGGAAGGTTGT	59.973	20	177	3	179
60.904	20	GGGCAGCAGCTTTACTTCAG	60.154	20	266	3368	3633
59.96	20	ATCAGAGCATCAGAGCGGTT	59.981	20	172	492	663
60.331	20	AGGCAGACAGATAAGGGCTG	59.454	20	245	429	673
59.751	20	AAAAGATGCATGTGTTCCCC	59.797	20	206	314	519
59.83	20	ATAAAACGACGTCGCAATCC	59.967	20	180	226	405
60.058	20	CTATTACCCCTTCCCCGTTT	59.197	20	275	429	703
57.427	22	CGGATTTAAGGGTTATTACCTGAT	58.81	24	207	682	888
60.067	20	TAAATTGCACCCATCAACCA	59.786	20	176	931	1106
59.83	20	CCAAGTACACTCCTCCTCCTG	58.804	21	252	3981	4232
60.088	20	TGATTTGAGTGTAAACACGTCCT	58.688	23	160	96	255
59.691	20	TCGATCCTGTGGCCTAAATC	60.036	20	184	7	190
60.179	20	AAAAATGGCACATGGGTTGT	60.096	20	262	1647	1908
59.697	21	CCAATTTCGACCGTTGATCT	59.933	20	272	2272	2543
60.18	20	AGCCGACTCCATTTATTGCT	58.814	20	279	1041	1319
59.508	21	AAACACCTGTGGCTCAAACC	60.012	20	240	53	292
59.325	20	GGCTCAAACATGTCAGCCTT	60.263	20	264	32	295
58.686	23	AGGGAGTGCTCTCACCTGAA	59.986	20	213	170	382
59.859	20	TTGCACACCCTCTCTCACAC	59.872	20	184	442	625
59.966	20	TTTGCTTGATCCAATGGTGT	58.976	20	264	380	643
59.624	21	GCTGGATAGTTGGGAAACGA	60.074	20	216	20	235
60.11	20	GAGAGGAAGGTGTGGAGCAA	60.386	20	268	324	591
59.967	20	TGAAAATATCATCGGCCACA	59.891	20	279	300	578
59.647	20	AGCACTCTGCGAGTCTCCTT	59.355	20	280	70	349
59.555	20	GGAATAAAGCCGTTTGAATTTG	59.851	22	226	2	227
59.703	21	TGCGTTTTGACAATATATAGGTGT	60.083	26	179	62	240
59.712	20	GGCCTGAATTCACATCCATT	59.756	20	271	1348	1618
60.481	20	CAAAATTACAAATGGCCAAGA	57.705	21	132	51	182
59.955	20	CCACTACATTCAGGGCCATT	59.813	20	235	749	983
59.952	20	TGCTCACCACACTCTTTTCG	60.025	20	105	325	429
60.692	20	GAACGAATCAAGTTCCGCAT	60.081	20	248	98	345
59.301	20	TCTGGCCACTTGTTTCTCCT	59.844	20	275	573	847
59.867	20	CAACTCGCATACCCAAAAGA	58.771	20	236	22	257
59.861	21	CAAATTTCGCCGTTCTTTTT	59.231	20	248	2008	2255
59.993	22	TCCGTCTTATTGGTCCGTTT	59.429	20	280	402	681
59.522	20	CTGCAGGTTCATGTTGCTTC	59.445	20	258	553	810
59.665	20	GCTGAGCATTTTTAGCACCC	59.851	20	280	6	285
59.927	20	CGCTGTATAATGGGGATCTCA	59.93	21	159	2342	2500
59.878	20	GGACGATGGTTGTGATGATG	59.769	20	276	40	315
59.864	20	TTCGGACCATTCAAACTCGT	60.495	20	261	4089	4349
59.803	20	ATGGGTTCGAGTCTCAATGG	59.927	20	227	289	515
60.557	20	GTCCTCAACCTCTCAATCGC	59.81	20	168	75	242
60.111	20	CATTAAATCCTGTCCACATACAAA	59.241	25	170	579	748
59.959	20	TGATGGTTGCAAGAGAATGG	59.648	20	218	1780	1997

60.147	20	TGCAATGCATGGAGGAAATA	60.035	20	119	38	156
59.933	20	GGCAAATGGCATAACAAACC	60.196	20	220	309	528
60.564	20	TCGGTACCATCATCTGTTTCTTT	59.885	23	258	138	395
59.729	20	TTTTCTTTTCCTTTTATGCTCG	57.789	22	160	22	181
59.973	20	AGATTCTGGGGATTCTGCCT	60.037	20	213	1031	1243
60.001	22	TCATAGTTGACGCCCTTCGT	60.657	20	169	10	178
59.555	20	AAGGATGTGGGTTGAAGGTG	59.82	20	131	43	173
59.339	20	CAAGCAAATTGTGGCTCTGA	59.988	20	252	213	464
59.931	20	TCGTTGTGAATTACCCAAGAAA	59.492	22	124	165	288
60.142	20	CGCGTAGAGTTACCAAACCC	59.632	20	134	99	232
59.836	20	ATGGGAGCTCATGAACGAAC	60.081	20	277	869	1145
59.258	20	ATCGTGTTCCTCAAGGTTGC	60.119	20	165	2532	2696
60.199	20	CCTCTCACTTCTCTCCCCCT	59.801	20	216	19	234
60.133	21	TCAATGTCCCAACAAATCCA	59.75	20	270	33	302
59.993	20	TGAGTGCTCAGGCACAGAAG	60.333	20	188	83	270
60.447	20	TTAGGTCGATCCAACGGTCT	59.55	20	240	398	637
57.172	27	CAATTTCTGCTCTAATAAATCCAC/	58.809	25	113	14	126
59.174	20	ACTTTGTTGGTGGAGCGAAC	60.156	20	264	3452	3715
59.072	21	CTTTATCCTTTGCGCGAGAC	59.982	20	190	2	191
59.61	20	ACCGACCGGAATTTTTATCC	60.02	20	249	882	1130
60.002	21	GATTCGATCCGAAAGGTCAA	60.014	20	243	1024	1266
60.02	25	GAAGCAGCCAATGGGAATAG	59.668	20	100	28	127
58.743	25	GTCCTCCGTTGGATTGTACG	60.375	20	199	3	201
59.93	20	TGCTCCCATTTAAACAACCC	59.801	20	259	722	980
58.255	24	TGGAAGAATCATAATCAAGAAGA/	59.663	26	104	183	286
60.458	20	TTTTATTGGGCACCAAGGAC	59.801	20	264	2225	2488
59.964	20	AACCGTCCGCTTACTTCTGA	59.875	20	251	213	463
59.962	20	TTCTCAACCTCGGCTCTGAT	59.95	20	199	45	243
60.596	20	TATCATGCCAATCCACCACA	60.757	20	183	334	516
59.971	20	TTTCCCATGTTGTAATTAATGTAG(	58.388	25	145	63	207
59.72	20	CTCGCACCACTCCTCTTTCT	59.598	20	278	5	282
57.108	26	TGGCCAAGTGTCAACAATTTA	59.073	21	274	109	382
60.318	20	CCCTAAGGAACCGGTCTAGC	60.089	20	186	155	340
59.956	21	TGAGTTGGTTGTTAGTCCGGT	59.506	21	166	35	200
59.772	20	GCCTTCTCGAATTTGTCTGC	59.962	20	215	180	394
60.777	20	CTCGTCTCCCTTCGCTTCTA	59.711	20	275	18	292
59.92	20	GAGCTGGAGTTGAGAAACGG	59.989	20	200	74	273
59.91	20	TTGCCCGGAATTCTTAACAG	60.067	20	160	268	427
59.989	20	GTGTTAATCGCTTCCGCATT	60.103	20	189	591	779
59.875	20	AACTCGCACCCCACAGTAAC	60.035	20	151	2350	2500
60.119	20	GCCTCAGAGGTCGAAATCAC	59.81	20	271	363	633
60.074	20	ACGTCATGTTTTCAATCGCA	60.119	20	254	18	271
58.901	20	ATCTTGGATCGACACTTGCC	60.081	20	179	16	194
59.807	20	ATGACTCAACGGCTGGTTTC	60.119	20	112	66	177
59.912	24	CGGACATGGTACATTTTCTGC	60.38	21	170	584	753
59.378	22	AAAGTTGATGGAAAAGGGGG	60.159	20	139	2043	2181
59.813	20	TTTTTCTGCATTGTCGAAGAGA	59.996	22	249	481	729
60.038	20	GGAAAAAGCCGATCAAATCA	60.016	20	268	105	372
60.24	20	GAGAGGGTTGTCAAAGAGCG	59.989	20	133	263	395
60.073	20	CGTTACCTTCATCCCCTTCA	59.926	20	183	15	197
60.053	20	TCTGTGTCAGCATGTGTGTGA	59.922	21	280	1848	2127
59.873	20	GAGAGAGAAAGGCTACGGGG	60.34	20	156	80	235
58.725	20	GCAACGGAATACCACTCGTT	60	20	100	8	107
59.285	20	TCAGGGATAAGTGAGCTCGG	60.353	20	226	433	658
60.278	20	CCGGGTTTGATACCTGAATG	60.184	20	248	1216	1463
59.297	20	AGACTTCAATGCCCCTCCTC	60.596	20	170	169	338
59.955	20	CTGTGCATGAAACATGGAGG	60.112	20	167	274	440
60.088	20	CTGAATGTCACGACCGACAC	60.162	20	238	606	843

59.937	20	CGAGGACATTTACTCATGGGA	59.94	21	251	447	697
60.603	20	CTCGTCGTGAACTCCCTGAT	60.261	20	276	400	675
59.468	23	CACCTCCTCCCTCACCAATA	59.92	20	248	114	361
60.046	20	TCAGAAACAACTTGCCCTCA	59.415	20	230	28	257
59.721	20	AATCGAGGAAGAGGGCAAAT	60.039	20	211	3087	3297
60.206	20	TTCTCTTTTGGTTGGTGAAGAAA	60.129	23	228	1723	1950
59.499	20	GTGGGTTGGGGAAAAAGAAT	60.032	20	219	227	445
59.682	20	GACAGGGGTTGGGGTTAAGT	60.088	20	233	62	294
57.122	26	AAAGCTTGCGAGTGCATTTT	60.025	20	148	27	174
59.332	23	TATCCCTTGCAGTCCCTTTG	60.066	20	254	0	253
59.693	20	AATGCATGCACCTGATTACAA	59.055	21	177	220	396
60.111	20	TGTACCATCCGGGATTTGTT	60.051	20	190	222	411
60.066	20	TTCGAAATTATGAGGCTCCAA	59.668	21	188	37	224
60.197	20	CTTCGGGTTAGCAGATCAGG	59.83	20	267	106	372
58.916	27	CCAAATAATTTTGTTTCCACCAA	59.974	23	124	97	220
59.701	20	TTTAACGGGCGGTTTTGTAG	59.993	20	190	105	294
59.644	20	CTTCCTTCCACCCAACAAAA	59.942	20	271	1205	1475
59.79	20	TACTGCATTGCGACCCTACA	60.28	20	237	333	569
60.246	18	CCCCTTTTGTAAGTCCCCAT	60.046	20	174	1	174
60.243	20	AAACATCGATCAGTCCCACC	59.786	20	115	196	310
60.656	20	AATGGCTGGATTTAGTCCCT	57.611	20	277	22	298
59.925	20	TTTGGCTACAAATTGCTAGGG	59.262	21	242	48	289
59.957	25	TTTTGGGAAATTCTTGCACC	59.916	20	101	0	100
60.131	20	ATTCCAAGATGGCAACTGCT	59.7	20	194	7	200
60.425	20	TATTCACAGCATTCAAGGGG	58.572	20	198	500	697
60.555	20	TGTCCATTTCCCCAAATTGT	60.029	20	280	3	282
59.749	26	ATACTGGTGTTGCCTGCTCA	59.318	20	178	26	203
60.226	20	ACCAACCAAGAAGTCGAACG	60.149	20	279	1840	2118
59.989	20	CATCCATCTCTCCCTCACTCA	60.207	21	117	170	286
60.307	20	AAAATCGGACTGGACCATTG	59.79	20	268	247	514
60.022	20	GGGGAATTTTGAGGTGGAAT	59.996	20	275	492	766
59.808	21	TGACTAGTGTGCATCGTGGC	60.907	20	208	89	296
59.924	20	TGTCTTGAGTTTGTATCTAAGAAG	59.365	27	139	221	359
59.877	20	CATCAACTCACCCTCACATTTC	59.454	22	183	390	572
59.927	20	AAATATTTCTAGCACATGAAATTG	57.859	25	246	45	290
60.088	20	TGAGAGCTTGAAGAAGAGCAAA	59.517	22	132	392	523
59.756	20	ATTAGGAACGGGGGTGAATC	60.017	20	210	662	871
59.973	20	TCCCGATCTTGGTTGTCTTC	60.05	20	216	17	232
59.724	20	GTCAACTTGGGCAGTGGAAT	59.973	20	274	763	1036
59.901	20	CCATTAACGCCCCTGATTTA	59.789	20	170	249	418
59.542	20	AGTCGGCATTCAAGTAGTCG	57.98	20	144	268	411
60.169	20	TGAAGTTGTAGCATGGGCTT	58.379	20	230	83	312
59.943	20	AATTGGAGCAAAATTGGCAG	60.074	20	253	335	587
60.096	20	AAGCAGAATGCAAGCAAGGT	60.022	20	168	526	693
59.79	20	CAGCCTGATAAATGGGACAAA	59.946	21	210	178	387
59.301	20	ATTTAAGCGCACGCCAATAC	60.123	20	216	207	422
58.035	22	CTGTCCTTCTCCCACTCCTCT	59.856	21	201	164	364
59.948	20	TCAAAACAACACCCAACCAA	59.834	20	261	84	344
59.381	20	CAACAGCCTTGTCTCTGCAC	59.622	20	240	338	577
58.379	20	TCATTGTACCGCAGCAAGAG	60.011	20	272	3744	4015
60.088	20	TGAAATTTCCCAACATTCGC	60.817	20	171	392	562
59.92	20	TGCAACTCGAGATGAGATGG	59.942	20	200	78	277
60.03	20	AATCACAATCTTGGGCTTGG	59.933	20	270	339	608
60.027	20	ATACCTTCGGTGCTCATCGT	59.579	20	280	512	791
59.43	20	TCCTCACCCTTTCCTTTCCT	60.045	20	101	50	150
59.623	20	TTGAAAAAGTCGGCTGAAAAA	59.855	21	234	953	1186
59.61	20	TGGATTTCTCACGAAAAGGG	60.044	20	252	646	897
58.646	22	CTTGACAAGGCAGTACCCGT	60.171	20	171	2	172

59.325	20	CAAAAGTCAAGGCTCGGAAG	59.986	20	163	3636	3798
59.713	19	TTGTCATAGCCAACCCAAAA	59.013	20	223	364	586
59.957	20	TCGTGCGACATAGTATGGGA	60.096	20	205	3	207
59.433	21	TCCTCCATTTCCAATCTGCT	59.629	20	258	87	344
59.585	20	TGGAAAGATTGAGATGGGATT	58.44	21	260	842	1101
58.882	22	TAGTGAACGTTGGGGACACA	60.001	20	182	104	285
59.267	20	GGCCCGAGTTGTTGAGATAG	59.694	20	140	521	660
60.039	20	TTTAAGCATTCTCTTGTAATCCCA	59.212	24	272	53	324
59.573	21	TCCAGAGACCTACATGGGCT	59.679	20	216	1291	1506
59.749	26	CCAGATATACCCCCACATGC	60.036	20	254	3	256
59.799	20	CTCAGGGCAAATAGGGACAG	59.688	20	279	4152	4430
60.208	20	AACAGGCGCCTTACCAGTTA	59.769	20	187	111	297
59.992	20	AATGAAATCCTTTGGGGTCC	59.996	20	186	2451	2636
60.873	20	CAATGGGGCTTGGTTTTCTA	59.931	20	271	1303	1573
59.934	20	TCGGTTCAGTTAGAGGCTGG	60.388	20	156	1628	1783
60.009	20	GTGGACGTCCCAGATTGTTT	59.827	20	250	1109	1358
60.031	20	CGAGTAGATCACAACGGCAA	59.864	20	159	377	535
60.112	20	AAGATCTTGGTTGTTGGGTTG	58.945	21	149	585	733
58.693	25	TCTCTTCGAAACTCGAATCGT	59.066	21	160	1	160
59.971	20	AGATCAAAGGGCCGAGATTT	60.039	20	209	52	260
60.016	20	TATATCAATGGCAGCCCACA	59.914	20	278	1790	2067
60.22	20	GTCCGATACGAGCTAAAGCG	60.003	20	190	14	203
60.005	20	TCGAAAGAGTTGACGAATGTT	57.468	21	249	87	335
59.325	20	CCAAAATGCCCTTGTATGCT	59.96	20	207	393	599
59.867	20	ACAAAACCCTAGACGGCAAC	59.101	20	238	145	382
59.971	20	ATAGGGATGGAAGGGTACGG	60.032	20	129	230	358
60.074	20	CGACGGGTGAAAATCCTAAA	59.931	20	257	95	351
59.871	20	CGTGTCCATGCCCTAATTCT	59.955	20	236	319	554
59.853	20	ATCGGGATTGGTGGAACATA	60.014	20	176	796	971
60.05	20	GTTCTCGCAAACAATGCTCA	59.995	20	275	149	423
59.702	20	CCCCGAAAATTAATCATTGC	59.259	20	230	2165	2394
60.17	20	TCAACATTGGGCATTTTCAA	59.907	20	247	983	1229
59.842	20	AATTCAAGCTTCAAACGGGA	59.685	20	236	418	653
60.008	20	AGTGAACCGAAGGTCCATTG	59.966	20	241	176	416
59.647	20	CGGGTTCGAATTTACCAGAA	59.931	20	257	1823	2079
59.758	20	TCGAGGTGTCGAACTTGTTG	59.873	20	262	1035	1296
60.065	20	AAAACATACTCAGCCACCGC	60.14	20	261	1266	1526
59.853	20	ATGTAGGGTTCCTGTGGCTG	59.989	20	253	340	592
60.242	22	AAAAGACCCTAAGCACTCACAA	58.061	22	227	4329	4555
60.517	21	TTCTTGTTGGTGTGTGGCAT	60.008	20	266	82	347
60.226	20	GCTTGTGGGCATGAATCTCT	60.226	20	266	625	890
59.986	20	ATTAAACGGGTAACTCGGGC	60.204	20	250	1903	2152
59.816	20	GACACGTGGTAGCACGAGAAT	60.194	21	188	235	422
59.725	21	CCTTCCTTCGTTTTTCTCCC	60.046	20	160	335	494
58.948	20	CCGAAGCAGCTATTCACTCC	59.978	20	111	1404	1514
59.212	20	GCTTTAAATGTGTTACAAAATCGA	58.332	25	277	247	523
60.352	20	GTAGTGGCTACGGTTTCCCA	59.993	20	134	3837	3970
60.197	20	CCCATTTTTCTAGGCACCTTT	59.489	21	259	201	459
59.878	20	TTACGGTTCCACTTTTTCGG	59.968	20	200	41	240
60.035	21	CGGAAGATCCGAGATCAGAA	60.295	20	189	35	223
59.668	20	TGTGGCATCAAATCTAGCCAT	60.484	21	236	333	568
59.968	20	CACGTCACACATTGGACTTTG	60.057	21	276	337	612
57.569	22	TTCACGCATTTCCACACATT	59.972	20	101	38	138
59.66	20	CACACAATACGCACACACCA	60.071	20	176	239	414
60.009	20	GTCGGACCAGCTCTCTATCG	59.973	20	266	249	514
59.499	23	AATCTGACGGCCGATATTTG	59.923	20	182	284	465
59.218	21	GCCATCTGAAACCCCATCTA	59.894	20	150	28	177
60.405	20	CAACCGATATCCCCTCCTCT	60.285	20	157	6	162

60.292	21	GGTGGGAATTGTGGAAGAAA	59.767	20	222	75	296
59.957	20	AAAAGCATTCCCCCATGATT	60.511	20	232	1544	1775
60.103	20	TGCTAAGAGAAAGCCCCTCA	60.088	20	213	534	746
60.118	20	GGGAGACAGCAAAAGCTCAC	59.997	20	189	409	597
59.95	20	CCCCTCGTATGCAATGAATC	60.296	20	279	105	383
59.311	20	GATGATAGAACTCTGGCGGG	59.653	20	180	232	411
60.578	20	TACCTTTTTGCCCTCGAATG	60.067	20	171	1	171
59.724	20	GCACAACCACTCCACAAAGA	59.726	20	264	83	346
58.994	20	CCGATTGGGCCTATCCTATC	60.623	20	194	101	294
59.688	20	CCAAATGTGAGACCCTTAACC	58.423	21	132	40	171
60.014	20	AGAGACTGACGCACGTTGTG	60.096	20	117	205	321
59.845	20	TCAATCTAAAACCCGTGCAA	59.157	20	139	237	375
59.601	21	GTTCAAGATTGTGCGAGCTG	59.596	20	211	18	228
59.5	21	ACCTGCACCAAAATGGAGAG	60.111	20	151	277	427
59.383	21	CGTGGTTCGATCGTAATTGA	59.542	20	105	102	206
59.241	20	AGGGTAGACATGGGTTTCCC	60.052	20	222	239	460
59.968	20	GAGGCGGTAGGAAACCCTAA	60.443	20	130	95	224
59.943	20	CAGTTGGTCAGAGATGGCAA	59.831	20	231	297	527
59.971	20	GGCGTTGGACCAGTGTAGAT	59.997	20	274	70	343
60.469	20	GATGACACGCATCAAAATGG	59.931	20	154	1298	1451
58.81	24	CTCTTCATATGGGAAGGGCA	60.029	20	197	10	206
59.783	20	GCATTTCATCCTCTTCCCAA	60.014	20	106	544	649
60.22	20	CACTGCGAACCAACATCACT	59.751	20	151	132	282
60.227	20	TTCTCAGCTCGGGTTCCTAA	59.948	20	243	147	389
59.518	20	ATTTGGCACATGAGATGTCG	59.527	20	246	615	860
59.911	20	CGGTCACATAAGCGTCCTCT	60.277	20	252	619	870
60.082	23	TGATGATGACAATGATATCGTGAA	60.217	24	175	71	245
59.836	20	TTAGGCCATGTTTGTCTGCTT	59.761	21	216	200	415
60.009	20	GGCCTTCATTCAAAAGCTCA	60.331	20	260	2897	3156
60.298	20	CAGCCGAAGTAACAAAGACG	58.579	20	213	2104	2316
59.526	20	ATTATCATACCTCGCCACGC	59.951	20	169	944	1112
59.894	20	CGTCCACTTCATTGAATTTCTTT	59.531	23	280	3919	4198
59.978	20	TTGCATTACATGCTCCCATT	58.994	20	247	225	471
59.9	20	GCACACCAAAGTGAAACCGT	60.996	20	234	103	336
57.864	20	CAACAAAGCATTGCAAAGTGA	59.903	21	224	0	223
58.048	21	TCAAATACTAGCATATCGATGAAC	59.623	27	174	88	261
59.83	20	ATGGACGTCGTGACTGCATA	60.144	20	168	25	192
60.971	20	CTTCACCTCCCAGATTTCCA	60.042	20	143	87	229
60.103	20	TGTGAGAACCATCTCGGTGA	60.246	20	134	6	139
60.262	20	CCCAGATAGCAATAAGGGCA	60.053	20	156	735	890
59.923	20	CATTGTCAGAAAGTGCCGAG	59.44	20	256	42	297
59.752	20	GGAAGCCAGGAAAAGAAACA	59.288	20	276	2118	2393
59.829	22	CGACAAAGACTTAACTACTTGTAA	60.049	27	278	853	1130
59.337	20	TCTTTTGAGACCCACCAACC	59.943	20	264	659	922
60.739	19	TTTGTTTGATATTCAACCACTTGT	57.681	24	102	268	369
59.982	21	TGTCGGCGGTTAAAAAGTTC	60.11	20	197	4467	4663
59.984	20	GCGTGGAAAGGCTGAATAAG	59.845	20	244	664	907
59.94	27	AGGGACACCTAACCCCAACT	59.717	20	115	1	115
60.173	20	AAAAACCTTTGTAGGCGCAA	59.758	20	265	365	629
59.172	21	CCAGCCCAATAACTTGACCT	59.052	20	235	70	304
60.461	20	TATACACGGCATACATGGGG	59.136	20	254	571	824
60.474	20	ATGGGTCCAAACAACCTTTT	58.266	20	168	257	424
60.162	20	CGTGTTAATTCCCCTTCCAA	59.795	20	191	1499	1689
60.081	20	CAAATTTGATAGCGGTCTAATGAA	59.557	24	170	1463	1632
58.161	22	CTCAGGGACTAGTTGAGCGG	60.005	20	188	65	252
59.682	20	CACTAATCATCACCGTGCCA	60.539	20	279	75	353
60.007	20	ATTTTCCCTCCAGTTGACCC	60.169	20	247	4485	4731
60.154	22	AGCGCAAGCACCTAGAAATG	60.543	20	209	722	930

59.146	20	TGCACAGAAAATGAAAAGCG	59.992	20	233	277	509
60.17	20	GCCATGTGAGAGGGAGAGAG	59.945	20	234	110	343
59.551	20	TCCAATGGATGTAATGAGTTATTA	57.297	26	167	374	540
59.844	20	AGCCGTCGTATTCTAAACGC	59.388	20	153	74	226
60.051	20	TGTTTGCTCTCTTACTCTCGGTC	60.068	23	195	66	260
60.765	20	AGTTGGGTGAAGATCATGGC	59.934	20	233	84	316
60.026	20	GGAGCAATAAAGGGCAATCA	60.038	20	156	2938	3093
59.93	20	GCTATGGTATGCTGTCTGCG	59.474	20	259	101	359
58.201	20	TGCAGGACAATATCCATCACA	59.94	21	174	12	185
57.769	27	ATCTCGTTTCAAGCCCAAAA	59.685	20	150	35	184
59.95	20	TGATGACGATCTTTTGAAACCA	60.481	22	234	17	250
60.195	20	CCTAAGCCTACTCCCCACCT	59.593	20	162	109	270
59.722	20	ATGCGCGTTTTTAGGGTTTA	59.624	20	198	2366	2563
59.711	20	CAGAGTCCTGGGAGCTCATT	59.405	20	230	1072	1301
59.992	20	TGTGCACAAGCTTGGAAAAAT	60.663	21	209	1575	1783
59.454	20	ACTTGTGCTCGCCTTTCATT	59.882	20	272	2202	2473
59.988	20	TGAAGACCATGAGCACAGGA	60.402	20	249	265	513
60.59	20	GGGACGGATCACTCTTTTGA	60.05	20	270	74	343
60.184	20	ATGGAGGTATGGTGTATGGG	57.047	20	247	2478	2724
59.402	20	AGTCCCCACGAGGAAGAACT	60.111	20	275	690	964
60.475	21	TTGACGATATATAGTGTTTTGACA	58.972	27	187	286	472
59.773	20	CATGGGTGGTATGGTGTGG	60.517	19	144	17	160
59.955	20	AGCTGTTAGGGTTGTATAGTGAGT	57.436	27	165	338	502
60	20	GCCTTTGCCCTTTTTCTCTC	60.32	20	129	298	426
59.91	20	TTCAAAAACGAATTTGGCGT	60.468	20	178	231	408
59.837	20	CTTGCCATGCATGTAAGGTG	60.134	20	164	1059	1222
60.074	20	TCCTCTCGTGTTCATCCTCA	59.34	20	156	59	214
59.712	20	CGTCGATCTCAGTATCTTCGC	59.992	21	239	80	318
60.536	20	GGTAAATGGTTACCGGTTTGG	60.33	21	214	49	262
60.227	20	TCACGTTGCAAAAATAGCGT	59.373	20	272	46	317
59.859	20	TGGAATCTTGTTGGGGAAAG	59.903	20	251	599	849
60.309	20	CATATACGCGAACCCGAGAT	59.945	20	258	215	472
60.053	20	AGCCACCTCCTAGCCATCTC	60.756	20	108	344	451
60.038	20	CCCATGTTTGTAATTATTGTAGGG	60.228	25	168	1909	2076
59.803	20	TGCCCATGATTCTGTAGTTTTG	59.996	22	255	180	434
59.679	20	TTTTGTGATTTCCCATGTTTG	58.374	21	136	94	229
59.645	20	AAGTGGTGTGTTCGAGGCTT	59.769	20	264	203	466
59.919	20	TTTGAAGGCCGGAATATGAG	60.031	20	225	0	224
59.76	21	GGGTGTGGTTATTCCAATCC	58.959	20	253	3	255
58.686	25	CACTCAGTGGACACATTGCC	60.162	20	251	145	395
60.555	20	ACTGACATTCACAGGCATTG	57.067	20	223	72	294
60.096	20	GGGACTTTCTCTCTCCTGGG	60.186	20	205	187	391
59.622	20	CATGCATGATCAATGTTGTAAGA	58.571	23	158	7	164
60.864	20	TCATGATAATGCATGTCTCTGAAA	59.614	24	100	430	529
59.161	22	AACAGTGGGTTTCGCGTATT	59.499	20	126	29	154
60.057	20	GACTAGTTTTCTGCGACGGC	60.022	20	200	866	1065
60.008	19	TTCTGCTCTCCCCTCCAAC	60.333	19	260	108	367
59.903	20	TCAACGCATCTACAGGTTGC	59.871	20	119	290	408
60.298	22	TCCAATTGTTGTCTTCCATCTG	59.971	22	157	284	440
58.57	21	GGTTTTGATGGTTGTGATTTGTT	60.014	23	185	98	282
60.163	19	TGTGGTTGATGAGTTGGCAT	59.967	20	279	4092	4370
60.16	20	GCCTGAACAGTCGAGGTGAT	60.269	20	149	24	172
59.931	20	AGACGCTTGACGTTTGTCGT	60.896	20	216	33	248
60.269	25	TGGTCGTGAGATAGAGTGCG	60.008	20	206	1	206
59.844	20	TGGCATCTTCTCTCAAATTCAA	59.823	22	222	329	550
59.733	20	TGTTTCGATGTGCATGGAGT	60.12	20	122	259	380
60.008	20	ACCTGTGAAAGCGATCGTG	59.846	19	126	2521	2646
60.143	20	AAAATCAAAGGCCCACAATG	59.801	20	106	1700	1805

59.837	20	TAAAAAGATGAGCCCACCCA	60.439	20	186	160	345
59.685	20	TGAGGAAGAAGTGGGATGATG	60.058	21	157	42	198
60.065	20	GAGCTCTGGTTCCCACAGAC	59.841	20	235	835	1069
59.988	20	CGAAAATGCCCTTTTATGCT	59.212	20	246	1179	1424
59.903	20	CATCAAGAGTCGGCTGTCAA	59.984	20	182	54	235
60.053	20	AGAGGAAGAAGGGGGTGAAA	60.045	20	217	467	683
58.809	23	TCATCCCGTTTCAAACTCAA	59.097	20	154	43	196
59.546	20	TGATGTGCAGCTCAAACTCC	59.992	20	267	831	1097
58.994	20	CCACCAAGTATCGGTCCAGT	59.844	20	266	107	372
60.103	21	TGATGAAAAACAATTGACCAAAA	59.372	23	223	1	223
58.869	20	CCTCAAAATCTTTGAAGGCTC	58.059	21	176	95	270
60.232	20	CAACTGAGTCCCCTATCCGA	60.065	20	260	100	359
59.569	20	TGTTGCGATTTTCATTTTCC	58.601	20	202	34	235
59.864	20	CCCTCATAACTCCTGACTGCTC	60.268	22	191	592	782
59.815	21	TTCAACCATCAACTCCACCA	59.935	20	258	236	493
59.859	20	ACCATCGATGCATTCACAAA	59.931	20	277	1570	1846
60.089	20	AAAATCATGTTCCGTCCCAA	60.17	20	244	45	288
60.375	20	TCAGTTTCGAGTTATGAAGTTAGG	59.749	26	100	163	262
59.813	20	TTAATCTTGACCGCCGATTT	59.547	20	280	39	318
58.94	21	TTAAATGGCCTCGGTATTCG	59.922	20	240	26	265
60.406	20	AAGCTCGGAGTTGCCAGATA	59.978	20	191	30	220
59.82	20	GGTGAGGGGGAAAAATAGGA	60.124	20	104	73	176
59.823	20	CATATGCATCCGCAATTCTG	60.058	20	280	4547	4826
59.937	20	TGTTGTGTTCGTGTCCGATT	60.008	20	241	189	429
59.592	20	GTTATCGTTGTTGGGGATGG	60.051	20	197	3585	3781
59.903	20	CGTCACGGTTATCCTTCGTT	59.993	20	259	77	335
59.862	20	TTTAGCAGAGTTGGTGAAATCAT	57.952	23	220	572	791
59.849	21	CACCCTAACACGCACAAATG	60.027	20	134	314	447
60.029	20	GAGATTGGGGACTTGGTGAA	59.903	20	120	548	667
59.682	20	AACACCCCTCCTCAATTTCC	60.169	20	105	20	124
60.063	23	ATGCACCAAAAATTGACACG	59.444	20	197	724	920
60.074	20	GGTGATTGTATCGCCACCTC	60.348	20	255	2320	2574
59.993	20	GACCGCTAAAAACCGTGAAA	60.11	20	261	459	719
59.928	20	TGATTGGGCTTTGTCTTTTTG	60.096	21	180	48	227
59.671	20	GCCGTACATGAAAGACAAAACA	60.039	22	217	142	358
59.873	21	GGGAGATTTGAAATGTGGGA	59.727	20	270	124	393
59.853	26	AGAACACGAGGAACTTGGGA	59.697	20	154	6	159
60.169	20	CTCAACTCAGAGCTCCATGCT	59.76	21	167	610	776
59.78	20	GCGAATGAAACCCAAGAAAT	59.022	20	178	214	391
59.968	20	TTAGGCCAAGGCAATCAATC	60.038	20	172	103	274
61.042	20	CCCCTCCCCACATTCTTTAT	60.011	20	105	9	113
59.45	21	GGGTGTTTTGACAATATCACGA	59.73	22	230	1943	2172
59.87	20	AAAGATCTCTGCAAGTCCGC	59.579	20	216	285	500
59.74	20	GCTCTCAACCATACCTGGTCA	60.126	21	263	275	537
60.088	20	AATTTTCGTTCATGCCCAAG	59.938	20	218	218	435
59.826	20	TTGAGCTGTTATGCGTTTTGA	59.5	21	148	83	230
59.664	20	CGTGCTCAAACTACATCCGA	59.864	20	268	215	482
58.25	22	TGGTGGGTTGAATGCTATGA	59.924	20	203	442	644
59.989	20	ATCCAAACGAACTTCCAACG	59.971	20	207	41	247
60.069	24	TAACCCAAACCCACCCAATA	59.91	20	106	17	122
59.074	24	ATATGCCCGGTAAAAGAGCA	59.569	20	245	14	258
60.151	20	ACCGACCCATCTCTTGTGAC	59.969	20	210	461	670
57.462	27	CCTGCCCACTTTGAAGAAGA	60.366	20	247	1	247
60.011	20	ATCAACATGCAAGCATCCAA	60.08	20	200	46	245
60.14	20	TTGCTCTCATTGTAATCTCTCCC	59.739	23	120	0	119
57.479	20	CAGCTCCAATCCATCTCCAT	60.034	20	227	309	535
59.96	20	TCTAGCACGGTTGCAATCAA	60.401	20	245	90	334
59.96	20	GCTAATTCGGCAGCTTCTTG	60.117	20	144	441	584

60.263	20	GTTCGACTGATGGGTGCTTT	60.119	20	212	1227	1438
60.14	20	TTCAAAAATGAGTGGACCAGA	58.187	21	128	1214	1341
59.95	21	CATGGCCTTCTTCTTCCAAC	59.67	20	191	999	1189
59.882	20	CCTAAATGTGGGAAGTGGGA	59.784	20	172	1488	1659
60.074	20	TGTGGAAGCAAATGGTTAGC	58.773	20	157	505	661
57.538	22	TGGGACCACAGGATAGGAGT	59.38	20	244	6	249
59.29	22	CACCTTTTCTCCCTCTTCCC	60.045	20	218	78	295
59.989	20	GATGATCCGTTCGCACTTCT	60.226	20	223	136	358
59.567	20	ACGAAATTGATGTGTTGGCA	59.972	20	226	228	453
60.323	22	GTTCGAACCCTGGTCTTCAC	59.555	20	247	968	1214
60.046	20	ATGACCTTGCCACCATCTTC	59.934	20	227	442	668
60.349	20	CCTCGATTCACTCATCGCTT	60.362	20	233	225	457
59.097	22	ATCAATCTTACGGACTGGCG	60.096	20	222	295	516
59.973	20	AAGATCGTGCAAAATTTGAAAAG	59.672	23	249	28	276
61.448	23	GGATAGGCGGTTGAGATTTG	59.528	20	115	12	126
59.674	20	TCAGTTTGGTTTATGAGTAGGACA	59.15	25	136	1073	1208
59.946	20	CGTGGCAGGCTAATATCCAT	59.945	20	152	97	248
59.95	20	TGACATCGGACCATTTTCTG	59.496	20	203	31	233
59.989	20	GCGCTTTTTGCCTGATATTT	59.349	20	210	561	770
60.035	20	GAATGTGTACTGTGCAGGGC	59.169	20	254	3928	4181
59.336	20	TAACTCCGTATTCTTGCGGG	60.089	20	224	140	363
57.128	26	AGGAGGAGCAAGAGAGGACC	59.952	20	257	37	293
58.783	23	TATGGGTCAGAAAAGCCCAC	59.933	20	241	189	429
60.28	20	GCGTTGCGTATGGAAGAATC	60.615	20	279	682	960
59.651	20	ATTTGGGGTGAGCTTCACAA	60.495	20	240	239	478
60.017	20	GATGTTGATTGCTGCGAAGA	59.955	20	251	2236	2486
59.48	20	CGTTAAATCTCTGTGTTGAATCTG	58.016	24	180	249	428
60.11	20	CTTGTCACTTTCTCCCTCGC	59.989	20	193	1	193
60.074	20	TTTCAGAGGGGATGAAATGC	60.014	20	229	51	279
59.972	20	ATGGACCACCCACCAAAATA	59.91	20	247	77	323
60.399	20	CAACCCACCATGATTTCACA	60.216	20	229	752	980
58.406	27	GGCCGAAACAAGAACATCAT	59.939	20	239	314	552
59.973	20	CCCGATTCCCTCTCTTTTTC	60.011	20	131	515	645
58.876	24	TTTGCTGCTGATGTTTGTCC	59.847	20	212	43	254
58.46	21	TTGACTAAAACATGGAAATAATGA	60.054	27	160	19	178
59.939	20	GCAGTTTTGCTCCCACTCTC	59.997	20	230	1238	1467
60.073	20	TTGTTCCAGAAAGGAGGTTGA	59.697	21	117	713	829
60.016	20	GGTCGATACATCAGGCAACA	59.527	20	196	1006	1201
60.029	20	TGCATGATGAGGTCTTCGAG	59.942	20	233	6018	6250
58.2	23	TTCGACTTAATCAATAAAGATAAC	57	27	264	94	357
59.931	20	CTCCAATCTCCAATCCATGC	60.426	20	243	500	742
60.206	20	CAATACAACTGCCGAGCTGA	60.011	20	119	88	206
60.495	20	CCGAAGGGATAGGATTGTTG	59.382	20	163	26	188
60.073	20	GAACTTGCTGTTTTGGATGGA	60.103	21	154	103	256
61.008	20	CACGAAATTGATGGAAGAGGA	60.059	21	198	344	541
57.914	21	TGAATAAGAGTTCAATGAATTTCA	59.912	26	223	48	270
59.924	20	AAGGAGGTGTTATGAAGGGATT	58.028	22	121	222	342
58.644	21	ACACGCTTGTCAAACATTTCT	57.402	21	242	656	897
60.029	20	TGTGCAGAGGGACTTTAATGC	60.264	21	112	385	496
60.008	20	TAAGTTGATGTGTGCAGGGG	59.566	20	254	764	1017
59.464	20	ACAAACTAATCAAGTTCCGCA	57.439	21	117	222	338
58.785	20	TCAATTATTATCTCGATCCAACCA	59.713	24	275	551	825
59.934	21	GACATGATTACTCGTCCCCC	59.218	20	279	1338	1616
59.927	20	TTGAACAATGGTACCTCCTCC	58.898	21	144	497	640
60.035	20	GAAACTCCCAATTTGGCTGA	60.051	20	263	701	963
59.874	21	TAGTTCACCAACTCCGGGAC	59.966	20	183	5647	5829
59.924	20	TTCTCTCTTCTATTTCTTTCCTTTTT	58.425	27	234	79	312
59.588	22	CGGACCGGGTGGTCTAAATA	61.972	20	100	2078	2177

60.178	20	CATTTGGATCCTCAAGTGGG	60.309	20	127	2618	2744
60.074	20	GGGCCTGGTAATCCATTCTT	60.152	20	259	219	477
58.348	21	CTGTTGCCGTCAGTTCATGT	59.751	20	161	0	160
57.987	21	TTGGGATATCCGTTTCTTGG	59.756	20	256	320	575
60.011	20	AAACTTCTTTCCAACCGGAAT	58.991	21	200	63	262
57.844	20	TTTGGATCATGATTTGAGTTTGA	59.442	23	280	491	770
57.192	20	AATGACCGATCTCTCTATCCCA	59.925	22	100	344	443
60.014	20	ATGAGAGCCGCACTTTGTCT	60.02	20	195	318	512
59.955	20	CAAAAGCTCGGTTTAATCTGC	59.023	21	248	149	396
60.198	20	ATTGGGGATGGAAAATTGGT	60.245	20	143	46	188
59.916	20	AGGCTGCTCTACCACAAGGA	60.012	20	260	104	363
59.522	20	GCATGATCAGGGTAAAATCCA	59.776	21	190	558	747
60.149	20	TCAGGGTTTTAGCCACAAGG	60.103	20	201	304	504
58.288	23	GGAATGAGAAACTTTGGAGCC	60.067	21	268	0	267
58.743	25	GGGACAACGACTACGGAGAA	60.111	20	266	1	266
59.311	20	CATTTCCCTTCCTTTCTCCC	59.875	20	228	2112	2339
59.982	20	ATCGAACCCACGAGAGCTAA	59.836	20	267	2096	2362
59.166	20	AAAATGCTGACATTGGAGGC	60.081	20	208	2464	2671
60.376	20	TGAAGCAAAAGAACCAGAATCA	59.861	22	190	230	419
60.096	20	TGTTATCATTCCACCCACGA	59.774	20	184	166	349
59.953	20	CTTGTAGGCAACGGGAAGAG	59.869	20	190	45	234
60.042	20	TTGCCTCAACTACCCAAACC	59.971	20	109	10	118
60.149	20	CCGAACCACGTACATCCTCT	59.989	20	215	475	689
59.401	20	ACAGGGTCGGCTCATTTTTA	59.569	20	254	5	258
60.251	20	AGGGGAGGAGGAAGAGGATA	59.091	20	244	390	633
59.623	20	TCCCATTTCGCTTTCCTTTA	59.655	20	279	5594	5872
60.019	20	GCGGGTCTAACCCAGATGTA	59.955	20	264	579	842
59.701	20	GTCTTCTCTCCAATGGCAGC	59.957	20	247	429	675
59.522	20	TCAGTTTTCGAGTTATGAAGTTAG[illegible]	59.749	26	102	384	485
59.44	20	ATACGAGCCCGAAGGCTTAT	60.082	20	172	657	828
59.619	21	TCCTAAGAGATTTGATTGAAAAGC[illegible]	59.651	25	168	39	206
59.971	20	AATGCGCCTCCCACTTTAAT	60.821	20	163	177	339
57.142	21	CCGAGCCCTCTCTCTTCTCT	60.23	20	259	455	713
57.459	27	GAATGCTTAATGGGAAGCCA	60.038	20	121	2	122
57.235	25	ATGAACCTATTTCGTTGCCG	59.96	20	242	1	242
59.967	20	CGGGGTGTAACTTGCTTGAT	59.993	20	272	1020	1291
61.066	20	TTTTGAGGTGAGAATTGCCC	60.051	20	246	107	352
59.993	20	GTTATGCGTTTTGACGGGAT	59.829	20	278	997	1274
59.67	20	ACACTGAAACCGAACCGAGA	60.69	20	254	35	288
60.059	20	GCGTGACTATCCACCGTTTT	60	20	113	43	155
59.823	20	CACATGTACGCGCTCTTGAT	59.895	20	172	6	177
59.853	22	CCCAATTAATTTCGCCATTG	60.144	20	104	456	559
59.797	20	CACCCTCAATCACTCACACG	60.154	20	171	361	531
60.27	24	GCCTAGTTGGCACAAACTCC	59.74	20	152	598	749
59.705	20	GGATGGTGTTTGTGGCTTGT	60.823	20	148	171	318
59.565	20	CAGCTGCCTGCTGTAACTGA	60.347	20	218	12	229
58.775	20	GACCCAGCAATGGGTATATGA	59.659	21	279	767	1045
59.905	20	TGTTCCCTGGCAGCTACTCT	60.012	20	243	397	639
57.794	22	GCCAGATGCATTTCAATGTG	60.08	20	243	104	346
60.119	20	CAAAGGCCCACAATGGTTAT	59.685	20	145	332	476
59.533	20	GGCAAGTGGGTTACCGATTA	59.823	20	172	13	184
59.96	20	GGGATTTGTGGCAAGAAAAA	59.916	20	149	343	491
57.631	27	TCCATTTATGGAATCCTTCCC	59.971	21	243	9	251
59.989	20	AGGAGTTTCATGCCCCTTTT	59.94	20	211	435	645
60.088	20	TACCTTTTTGCCCTCGAATG	60.067	20	202	688	889
58.407	20	CAATTTCTTGCTGGGTCCTT	59.17	20	280	184	463
60.639	20	AACAAGAGAGACAAGGCCGA	59.989	20	136	0	135
60.082	20	CGGAAATTATTTTTAACACCCG	59.633	22	255	588	842

57.369	24	CGTGCAATTAAATTCAAATCTTGT	59.476	24	250	217	466
59.763	20	TGCGTTTTGATGATATATAGGTGT	60.054	26	121	520	640
59.75	20	GAATAAACAACAATGAAGTGGAA	58.585	25	165	148	312
60.103	20	GAAAGCCAGCATTGAAACCT	59.316	20	259	79	337
59.667	22	TAACTTATTAATCGGGTTAAACAA	58.599	27	100	958	1057
59.966	20	CATCCATGCAATGGAGATGA	60.444	20	216	240	455
59.566	20	TGAAATTTGGCCTTGGAAAC	59.916	20	269	172	440
59.851	20	ATCATTTATTTCGGCAACGC	59.935	20	135	169	303
58.308	20	CGGAACCGGACCATATTAGA	59.778	20	251	86	336
59.964	20	GAAGAACAATTACAATGTTTATCC	57.771	26	204	75	278
58.821	20	GTACGTTGGAACACGTGGC	60.024	19	223	845	1067
59.996	20	TTTTTGCGAGGATTTTGGAC	60.053	20	203	102	304
60.212	20	CGTAAGATTTTTAAAAGAATTGAT	60.128	27	115	135	249
59.989	20	TTAAACCGAATGGCGGTTAC	59.832	20	215	13	227
59.997	20	TTAGCACTTCTGCCTGGGTT	59.875	20	226	613	838
60.118	20	ATGGTGGCTGCATGTCATTA	59.955	20	190	6250	6439
59.971	20	ATTAAATGCGCCTCCCTCTT	60.061	20	179	162	340
59.812	20	GAATTGAGTTTTTCAACTCTATGG	59.17	26	252	1546	1797
59.976	20	CCCAAAAAGGGATAACATGC	59.273	20	240	1003	1242
58.861	22	CCCGCATCACTTGAACTCTT	60.255	20	238	9	246
60.008	20	GTCCACGGGAGAGAATGAGA	60.199	20	279	160	438
59.685	20	CAATTTGACGTGATTTAACGTGT	58.978	23	240	369	608
58.2	21	TTCTGCGACCCTTCACTCTT	59.989	20	280	148	427
58.995	20	GGAGGAATTGGGATTTTTGG	60.482	20	101	192	292
59.607	21	GGTTGTGGCTCAGAGGGATA	60.073	20	232	45	276
59.276	23	TTTTCACCTTCAAATGTCGC	58.747	20	219	453	671
60.112	20	CCTCCATGTTCCTCATCGTC	60.475	20	161	548	708
60.096	20	CCGCCATGTTTCGATGATTA	61.747	20	271	2174	2444
59.95	20	TCCCCATTTTTGATCACCTC	59.727	20	207	73	279
60.223	20	AGGCCGAGTGAAAACCATTA	59.569	20	138	203	340
57.536	20	GGGGTTGTACTCGTGGGATA	59.67	20	262	88	349
59.691	20	TTTTCCGGAGTTCACCTCAC	60.088	20	273	156	428
59.694	20	CGATGTCAAGATGCACAAGG	60.263	20	277	2284	2560
59.987	18	TACCACACGTGTTGGCTAGG	59.634	20	280	1312	1591
60.219	21	AAACCAATATTTTGCGGTCG	59.832	20	270	32	301
59.899	21	ATCTTGCTGCTCCAAGGTGT	59.874	20	130	161	290
59.744	20	TGGGTAGGGGTGTTCAAAAA	60.198	20	232	210	441
60.103	21	AATCATGCTGCATATATAAATGTC	57.471	26	103	107	209
60.118	21	ATAGATTGTCCCTCGAACGC	59.154	20	218	37	254
60.285	20	TCGGAGCGATAAGGAATCTG	60.309	20	276	7	282
59.392	23	GGCGTCGCTGTAATAGTCCT	59.364	20	180	329	508
60.197	20	AAAGAAACAATCCCAACCCC	60.032	20	216	341	556
59.916	20	GACTCCCTTCCTCAACTCCC	60.05	20	236	92	327
59.875	20	CACTCACTTCCTCCGTCACC	60.713	20	166	584	749
58.297	20	AGGGCATCACTAGAAAGCCA	59.836	20	224	32	255
59.872	20	AATCATTTTCCGCACCCATA	60.153	20	171	92	262
58.397	23	CAAATTGATTATGAAGAAATTGGA	58.222	25	269	578	846
59.931	20	GCGGTTCCATACAGTGACCT	59.997	20	219	460	678
59.801	20	AGGGTTTTTCTGGGGAGAGA	60.045	20	262	3	264
60.127	23	CACACACACTCTATAAAATGCACT	60.501	26	114	420	533
59.728	20	ATTTGTGGGTGGTTCGTGAT	60.096	20	252	840	1091
59.955	20	GAACGAGGAGAAGGACATGC	59.81	20	172	42	213
60.159	20	GTCCACTTTGTTTCTGCTGCT	59.534	21	100	311	410
60.07	20	ACCTCTCATCACCGTTCCAG	60.112	20	182	7	188
60.444	20	TGCATATCTTGGTGAGTGTATGG	59.897	23	148	333	480
60.088	20	TCACTGGCTTGAGTTTGACG	60.025	20	186	263	448
59.006	21	TCATCGAATCACATGCTTGC	60.79	20	199	1469	1667
59.864	20	GACGGTCAGCCACTCAATCT	60.269	20	196	103	298

60.034	20	TTGGGAGAATTTTCAACTCTTG	58.352	22	191	77	267
59.089	24	CACAAAGGCAGCACTTGTTC	59.49	20	246	7	252
59.894	20	AAATCAAATGGGAATGTCGG	59.622	20	134	18	151
59.992	20	TTCCTTTCCCTCCAAAACAA	59.518	20	218	846	1063
60.111	20	TTTCTCTCTTGGTTGGTGAAGA	58.955	22	105	158	262
57.424	20	CCACGGTTCCTTGGTAATTG	60.22	20	206	491	696
60.025	20	ACACGAGTGTGGAAAGGGAG	60.151	20	161	59	219
59.556	20	AAGGCCAAACGAGAGCTACA	60.015	20	160	187	346
59.924	20	TTCCGGTCCAAACCAAATTA	60.16	20	221	142	362
60.009	20	TCTCGTCCTCCTTCGACACT	59.986	20	225	1	225
59.741	20	CGATACCATCATCCCAATCC	59.975	20	203	545	747
60.266	20	AATTTACACCTGTCCCTGCG	59.993	20	130	247	376
60.309	20	TTGAGTCGAGGAAGATCGGT	59.803	20	112	705	816
60.252	20	ATTGGCAAATATGCGAGGAC	59.929	20	132	29	160
58.554	22	ATCGGTTATCACCGGATCG	60.697	19	160	310	469
59.799	21	CTCGAGTGAGACCGAAGTGA	59.125	20	239	76	314
58.994	20	ATTCAAAGGGCATTTTCGTC	59.022	20	158	340	497
59.96	24	TTTTTATACAAGGGGCGGAA	59.429	20	150	71	220
60.049	20	CTCTGCTGTGAGCATTCGAT	59.143	20	143	64	206
59.635	20	ATGTAAGGCCCATAATCGCA	60.31	20	131	699	829
59.802	24	TGATAACCTTGAGGAAACTTGC	58.378	22	278	9	286
61.391	20	GGGTGGGTTTGGAAAAGAGT	60.204	20	272	78	349
59.989	20	AAATGCCAAATAAAGCTAAATCC	57.912	23	252	2781	3032
60.096	20	CTATGCATGGGTTCCGTTCT	59.955	20	165	25	189
59.984	20	GGCAAATTCCCACAAAAGAA	59.916	20	262	80	341
59.656	20	TTTCGCCCCTTCTAGAGTCA	59.948	20	217	1	217
57.555	23	TCAAGGAGGGTGTATGAGGC	60.073	20	267	5	271
60.147	20	CCATGTTTGTAATTATTGTAGGGA	58.388	25	108	87	194
59.933	20	CCTAGAACTGGCCTCACGAC	59.867	20	156	74	229
60.081	20	GAGAGAGAGGCAGACATCGG	60.096	20	202	82	283
60.074	20	GGCCCTATGCATGTGAAGTT	59.962	20	268	432	699
58.674	20	TCACTCTCGAGCCTAACCGT	60.012	20	211	441	651
58.199	26	ATGTTGGAAACCCTTCCCAT	60.422	20	280	2976	3255
59.019	20	GGATTCCTCCTCCTTCCTTG	60.008	20	202	116	317
59.987	20	TGAGAACCCGGTTTGATTTC	59.91	20	263	6511	6773
58.093	27	AATCGAACCTGGGTCTCTCA	59.655	20	172	10	181
60.081	20	GAATCACACCAACGCCTTTT	59.978	20	158	4067	4224
58.124	25	GGTTTTGACATAACTTAGGTGTTT	59.358	26	119	0	118
59.834	20	TCTTTCGTCGTTCATTGCAG	59.988	20	165	233	397
60.029	20	TGCACCACGTCATCTTCTTC	59.837	20	230	1114	1343
59.933	20	CTTTCCTTTCCTTCCAACCC	59.912	20	256	10	265
60.162	20	AAATTGGCATTTGTGAAGCC	59.945	20	182	748	929
59.777	19	TGGGTGGAACCCTAGATGAC	59.779	20	164	64	227
60.103	20	TTCTCACCAGTCTGTCAACCA	59.294	21	252	1000	1251
59.902	20	GGGTATGATTCGAACTGCGT	59.962	20	246	0	245
60.538	20	ATGAAAAATGAGGGCTGCAA	60.585	20	273	972	1244
59.04	20	AACTGAGTCCCCTACCCGAT	59.817	20	275	134	408
59.724	22	TGGCTACAATACAACGCCAA	60.133	20	190	96	285
61.606	20	AAAATCGTCCCAAATCCCTC	60.131	20	174	0	173
58.844	21	CTGAGCAATCTGTTCGTCCA	59.984	20	224	85	308
59.541	20	TGGGTTTTGCTGTCTTTTCC	60.088	20	178	6	183
60.218	20	GGGGTTAAGATTTGGGGAAC	59.51	20	255	70	324
59.845	20	ATAGGCTTGGGAAAGCAGGT	60.096	20	276	408	683
59.811	20	CGATAAATGGTTGACGGCTT	59.96	20	162	300	461
60.081	20	CCGACCTAGCATAGTGGGAA	60.088	20	263	1403	1665
60.12	20	CCTCTGCAGCTTCCAAAGAC	60.134	20	136	1061	1196
60.969	20	CGCATCATGTTGTTGGAAGT	59.572	20	238	983	1220
59.967	20	TCGGAGTGTTACCGTCTTCC	60.111	20	262	668	929

60.431	18	GTCACTTGCGGGTATTGGAC	60.384	20	149	43	191
59.284	20	GCATAGTTCATTCAAAAATGTCG	58.689	23	211	689	899
57.497	27	TTTAAACTGACGGCCGAGAT	59.708	20	161	68	228
60.61	20	TCCTCTCCAGCCAAAAAGAA	59.926	20	203	278	480
60.12	20	CCCAAAAATTGAACATATCGAA	58.848	22	194	546	739
60.039	20	TCGAGATGGGGTGCTTTTAC	60.074	20	234	97	330
59.871	20	GCCTTCTAGCATCTCTTGCTCA	61.155	22	234	176	409
59.923	20	GATGATGATTGCCCCATTTT	59.587	20	132	300	431
59.228	21	GCAAACGATCAATCCCTGTT	59.939	20	270	741	1010
59.647	20	GGTTAAAGATCAAACCAAACACG	59.801	23	276	99	374
60.096	20	CACATGGATAGAAGGGGTGG	60.187	20	192	898	1089
60.051	20	TTTTGGATCCGAACATCCAT	60.133	20	278	1571	1848
59.951	20	ATGTAATTCATTCACGCGCA	60.103	20	112	42	153
59.813	20	ACGTTGATAAGCCATCCCAG	59.955	20	229	12	240
59.946	20	AACCTCATTGCTCTCCGCTA	59.978	20	144	323	466
60.853	20	CGCCTTTATGTGACGTGGTA	59.609	20	258	2450	2707
60.019	20	TAAGACTGGTGGGAGTTGGG	59.959	20	187	275	461
59.731	26	ACGGCGGAAACATTAGACAG	60.132	20	208	86	293
60.103	20	TAACCCCCAAAGCTATGCAC	59.96	20	274	496	769
60.249	20	TCCCAAAAGCAATAATGCAA	59.146	20	268	285	552
59.769	20	GTTGGACTTTTGGCCTTTGT	59.077	20	176	332	507
59.988	20	TCTCACGTCTCACACCCTTG	59.864	20	255	15	269
60.386	20	TCGTGTTCTATATTCCCGTATCG	60.226	23	102	957	1058
60.006	20	TTGACCACAGCAGTACGACC	59.751	20	217	324	540
59.156	23	ACCACGCCAGTCAGGTAAAC	60.035	20	220	318	537
59.405	20	AGACACATGCACTCGCTACG	60.08	20	154	211	364
59.996	20	GGCGAGCCATATGTCATTTT	59.929	20	114	3603	3716
59.805	20	TGTGGATCTTGGTTTGGTGT	58.827	20	254	205	458
58.903	20	GTGTTGGTGGACGAGGAACT	60.009	20	186	45	230
59.967	20	CCCAACTGTTTCAGCCATTT	59.971	20	121	545	665
59.596	20	ATCGTATTTTCATCGACGCC	59.929	20	151	475	625
60.733	20	TGGAGAGGGTTGTTGGTGAT	60.363	20	101	107	207
60.041	20	GGGTATTTATGATATTTCCAACAC	57.935	26	228	11	238
60.532	20	CCACTAGGGGTGGCAATCTA	59.948	20	229	1235	1463
58.743	20	GGGCGAGTTAAGCTCGAATA	59.456	20	239	27	265
60.015	20	GAACTCAAACTGGTTGGGGA	59.943	20	244	333	576
59.293	20	GCTGGATTGGCATCATTTTT	59.907	20	246	107	352
60.626	20	GCTTGATTTTCCTTCCTCCC	60.017	20	156	33	188
57.53	21	CGGTCCAGTCTAAACCCAAA	59.964	20	260	32	291
59.926	21	ATGGCGAAGAAAGCTACCAA	59.845	20	267	41	307
60.488	20	TGTTTTCTAAAAATAGGACACACC	59.056	25	224	155	378
57.837	20	GCCTATCAGGTCGTTGTGGT	59.997	20	148	34	181
59.978	20	CCCTTTTCGATCCTTTGTGA	60.044	20	157	73	229
58.941	21	AAGTTGGTTTCCCACAGACG	60.005	20	162	114	275
60.519	20	TCCACTTGCAGCATTTAACAA	59.359	21	216	446	661
59.316	20	TTGGACAAAAGTACCAGGGC	59.971	20	272	1493	1764
59.261	22	ATTTCGGTGAATGCAAGACC	59.939	20	280	1467	1746
58.558	27	GCACCACACCGAACTCAATA	59.572	20	206	39	244
59.288	20	TCATATGTCACGTTAAGACATGGA	59.406	24	223	382	604
59.944	20	GACGACAACGTTGGTGTTTG	60.049	20	162	980	1141
59.734	20	TCCATGTGTATGCTGCTGGT	60.144	20	213	299	511
60.088	20	GGTGTGAAGAACTCCCTTGG	59.549	20	158	28	185
59.769	20	ACGCAAATCCTAACAGTGGG	59.993	20	254	1082	1335
59.872	20	GATGATGGGGAGGGTAGGTT	60.015	20	182	20	201
59.803	20	CCGAGGTGGAGTAGTATCCG	59.569	20	176	339	514
57.301	23	GGAATACGATTCGGAGCATC	59.491	20	270	1	270
59.861	24	TCCGGAATTAGGTGAAAGCA	60.576	20	160	213	372
60.441	23	CACGTTTCTTTAACCGCCTC	59.747	20	220	21	240

60.605	20	CTCTGTCACCCCAGAAAACC	59.549	20	127	1551	1677
60.042	20	GCCCTCATGACTCTCAGCAT	60.381	20	280	824	1103
59.691	20	AACACCTTAAAATGTCGCCG	59.996	20	221	78	298
58.93	20	CATGTGTCTTATGTTCGCAGG	59.209	21	236	2510	2745
58.079	23	GCTACCCTTTTTGACCTACGG	60.008	21	254	26	279
59.578	21	TGTTGTTCGGGTTGATTTGA	59.941	20	101	51	151
60.713	20	GAGTTTGGGAGTTGAGCTGG	59.844	20	228	570	797
59.826	20	AATTGCGGAGTTGCTGTTTC	60.257	20	278	5190	5467
60.001	20	TCCTGAAAGCAAGCAAAGGT	59.993	20	170	1102	1271
61.031	20	CAAAGGCCCACAATGGTTAT	59.685	20	110	17	126
58.51	27	TTCCCCAGTGTCACACAGAC	59.55	20	122	3	124
57.083	25	AGTGACCACCCAACCACAAT	60.135	20	216	66	281
60.626	20	CCCACAAAATAGGGGGAAAA	60.873	20	175	233	407
60.184	20	TCACTCTCATCTCTCTCACCTCA	59.19	23	258	142	399
59.916	20	TCTTATGCCAACAAGACGAAAA	59.758	22	178	25	202
58.264	25	TATATGCTCGCGTTTCATGC	59.832	20	146	272	417
59.062	20	AGCAATGTTGTTGGGTGGTT	60.277	20	135	1118	1252
58.273	23	AGATCTGCTCACCAATCGCT	59.981	20	272	15	286
59.864	20	AAATCGTGTTTCGTGCATCA	60.119	20	209	213	421
60.366	20	CTTGCCTAACTCCTCCTCCC	60.204	20	208	81	288
59.943	20	GATTTCTTTGGGCAGAACCA	60.051	20	107	757	863
57.552	24	CGAGAAAGGTTTCTTCGCAC	59.993	20	220	6	225
59.894	20	GGTCTTTTGTGTTAACATCATGC	58.553	23	223	292	514
59.072	20	ATCAGCTGTCCCTTTTCCCT	60.074	20	260	155	414
59.956	21	GACGGAGGTGATTCTTTGGA	60.05	20	190	256	445
57.734	23	AAAATGAAATTGATCATTTGAGGA	58.904	24	254	56	309
59.962	20	GAGGCAACTTGGGAATTGAA	60.051	20	273	2324	2596
60.615	20	GACTCTGCATTTGAGTCGCC	60.956	20	265	387	651
60.061	23	CCTTCCTTTTGCTTGTTGGA	60.22	20	133	142	274
57.606	22	TCACCATAGGATTCTCTCAATCTG	59.615	24	275	12	286
57.588	23	GGGTTCCCACGTCATAAAAA	59.662	20	104	1412	1515
59.518	20	TCTGCATTCTTGCATATCGG	59.792	20	163	1285	1447
58.842	22	TCAGCCTTATCTCTTGCCGT	59.978	20	196	16	211
59.996	20	AATGGCTGTGGAGAATCCAG	60.073	20	189	201	389
60.017	20	CGGTTCCAAATTGCTCCTAC	59.569	20	244	357	600
58.885	22	AAGCCTCCAGATTTAAGCCC	59.688	20	267	76	342
60.334	21	ACGCCAGCCTCACTCAAA	60.557	18	131	3	133
59.803	20	TGTTCTTGTGTGTGTGCGTG	60.412	20	272	627	898
59.664	20	ACGGAAAAAGGACTCCGACT	60.11	20	277	246	522
60.006	20	CGTTCCCATGGCCAGATA	60.435	18	174	36	209
60.039	21	AAACCCGGAATGGGTAAAAA	60.393	20	279	8	286
59.485	21	TCGACCTCGTGCTAATGATG	59.823	20	217	192	408
58.909	20	ACGGATCGGAAATCCATAAA	59.23	20	193	994	1186
60.292	20	GAGATCGGAGTGACTCTGGC	59.952	20	151	60	210
60.177	20	ACATACGTTCCTCGGACAGC	60.142	20	158	328	485
60.134	20	GCACAATTAAATGCGCGATA	59.705	20	171	131	301
60.088	20	GCAGCAGCTGAATCACAACT	59.195	20	145	1595	1739
59.691	20	ATCAGAGCCGAGGTTGAGAA	59.95	20	144	7	150
60.255	20	CTACAAAATGCGCGAGATCA	59.976	20	210	3425	3634
59.293	20	GGGGAACTTTAATTTTGTGCC	59.708	21	246	448	693
60.539	20	CCGTTGAGCCGAATGTTTTA	60.989	20	280	44	323
60.214	23	CCTGTGTGTGCTCCAATCTG	60.309	20	214	790	1003
60.408	21	ACGAAATTGCCCTTGTATGC	59.967	20	203	84	286
60.088	20	ATCACATCATCCTCCCCTTG	59.737	20	266	1	266
59.883	20	TTTCGTGTGTGTTCCTCTCG	59.873	20	229	108	336
61.021	20	AGAATTCTGCCTCCTGCAAA	59.955	20	196	1857	2052
60.593	20	ATCGATGTGTTGGCATTCAA	59.931	20	193	246	438
60.155	20	ACGTTCCGGTCTGTATTTCG	59.993	20	259	2347	2605

60.096	20	TCTCAACCATCACGAAATGC	59.654	20	269	1280	1548
59.107	21	TGAAATTTGGAAATAAGGAGGTG	59.382	23	102	686	787
60.04	20	TCTTTCAGCACAAATGCGAG	60.134	20	246	3328	3573
59.64	26	CTGAAGGCCGATATTTGCTC	59.807	20	264	907	1170
59.923	20	CATGTGGACCATCGATCTTG	59.918	20	173	13	185
60.096	20	GTCAGACCAGCTCCCCTTC	59.783	19	192	1002	1193
59.995	20	TTGACCAAGGTGAGAATTTGAA	59.587	22	216	27	242
61.066	20	TGGATTTCATCATCAAGTCTCC	58.984	22	208	51	258
60.229	20	ACGGCAGCTTCTCTCATGTT	60.02	20	213	816	1028
59.998	20	ATTGATTGTTAGCGGATGGC	59.929	20	224	830	1053
59.212	20	TTGTGAATGTGGTTTATTTGGC	59.742	22	136	12	147
59.801	20	TCTCAAACCGTGAGATGAGATT	58.792	22	183	962	1144
59.931	23	TTCCGTTTGAACTTTTCATTTTT	59.098	23	277	194	470
59.395	20	CGGATTTAAAGGTTATTACCTGAT	58.157	25	223	889	1111
60.151	20	GCCTTTGCCCCTTTCTCTAT	59.688	20	263	85	347
60.723	20	TTTCCGAATATGAAAACCGC	59.907	20	257	104	360
60.102	23	TGGATATGGTTTTTGCAGGG	60.692	20	168	89	256
59.273	20	TCGTCCAACCAAATTGTTGAT	60.22	21	268	607	874
60.656	18	TGACAATGTTATCGATGGCG	60.488	20	169	5	173
59.973	20	CGTACGCATGTTCAATTTGG	59.992	20	168	2171	2338
59.789	20	TGTCGAGACAATCGTGGAGA	60.402	20	244	467	710
58.991	21	GGGTTTAAAATAGCGCGTTG	59.624	20	156	326	481
59.823	20	AATTAAGATTTCGGGAAATTAGTC	57.21	25	243	167	409
57.308	27	ATCAAAATTCCAATTGGCCC	60.865	20	179	11	189
59.61	18	TGCAACTCCAATAAAGAATTTGG	60.342	23	254	26	279
60.227	20	CGAACAAGGGAAAACAATGG	60.336	20	264	276	539
59.705	20	CAATCAACATGCAAGCATCA	59.232	20	194	4	197
60.012	20	AAACAGCCGTTATCAGCCAC	60.14	20	254	80	333
60.029	20	CCTTGGCTATAAAAAGGGGC	59.93	20	161	532	692
60.188	20	GCGGGTAAATATCAGTTTTTGA	58.2	22	237	191	427
59.901	20	TTTGTGATGCTCCATGCTTC	59.805	20	168	378	545
59.846	20	TCAAGGGTTAGGGCTTGCTA	59.839	20	186	195	380
59.653	20	AATTGAATGGCAAGGATTGG	59.762	20	184	2116	2299
59.717	20	CGCCATTTTAGTGGTGGAAC	60.365	20	222	104	325
59.989	21	GTCCTCCGAACTCATGGTGT	59.969	20	256	54	309
60.134	20	AACCTCAGTGTTGCAGCAGA	59.622	20	275	179	453
59.903	20	TGCGATTCTGGAAGTGAATG	59.799	20	175	583	757
59.91	20	GTACGGTTGCTCATCAGCCT	60.285	20	264	1891	2154
59.957	20	TTTCCCACCGAAAGCTACAG	60.241	20	255	2060	2314
60.786	20	CTCTGAGCTTGGGAAACCAG	59.982	20	100	373	472
60.659	20	CCCAAGACCCCAATTTGATT	60.91	20	260	381	640
60.22	20	GGAGTGTAATCAAATGAGAGCAA	59.672	24	178	67	244
60.361	20	GTGCTTTGTGTTGGTGGATG	60.008	20	243	487	729
59.953	20	TGTTTCATGTGTCCATGTTTCA	59.864	22	235	2567	2801
59.803	20	ATGAAACTGCGTTCCCACTC	60.119	20	246	530	775
60.32	20	GGTTCTCACAGGAGGGTTCA	60.088	20	177	53	229
59.157	20	TGCATAGGTTGTTTCATCATCTT	58.642	23	265	243	507
59.894	20	ATGTTTTTCGGATCCCTTCC	60.131	20	228	10	237
60.035	20	AGTGGGGAGCCTTACCTTGT	59.994	20	265	180	444
59.928	20	GCGAAGAAACATTCAGGGAA	60.192	20	171	9	179
60.088	20	AGGCCTAAAATGCAAGCTCA	59.982	20	252	2030	2281
59.982	20	CCCCAACAAAAACACCAAAC	60.103	20	262	2634	2895
59.52	20	ACCTTCACCAATCACTGCCT	59.579	20	222	632	853
58.923	20	ACCTGGACTCATGTGGCTTC	60.12	20	264	1366	1629
60.277	20	TTTGTCTCCCACATTCATGG	59.343	20	180	632	811
58.544	21	AAAGCCTACCTTGCTTCCGT	60.262	20	233	35	267
59.778	20	TGCCATTAATGACGGTGAAA	59.93	20	176	15	190
59.047	23	CCGGAAAATGTGGATGAAA	59.31	19	280	10	289

60.168	20	TCACCAAAATCTATAGTTATCACA	59.412	27	170	342	511
59.917	20	AGCAGCAAGAACAAGGTGGT	59.914	20	252	770	1021
59.731	23	AGAACATCAGCGGGCATATC	60.066	20	173	236	408
59.919	20	ATTTCTCACTGACTTGGGCG	60.255	20	126	172	297
61.003	20	TGTGTGTGTCCAGTAGGGCA	61.223	20	280	784	1063
59.811	20	TTAAACCCTAAAAACGCGCA	60.593	20	279	323	601
59.36	24	GCAGTGGATTTTTGGTGATG	58.976	20	212	1637	1848
59.784	20	CGTGTGGATTGTAGCAGGTG	60.175	20	170	24	193
60.195	20	AAACCCAGTAATCTCAGCCG	59.195	20	119	7	125
60.016	20	TATTGACGCGTTTTGGCATA	60.096	20	223	134	356
58.943	20	TGACGTGGCATTTTCAGAGA	60.39	20	263	18	280
59.872	20	TTCGTTTGGCATGTTTGTGT	60.011	20	267	296	562
60.315	23	CATTGTACTACATGGATGAATGAT	60.03	26	223	388	610
58.995	25	TTGACGTTTCACCTAACCCA	59.021	20	208	258	465
59.779	20	GCAAGACCTCCACACACAAA	59.726	20	274	234	507
60.448	20	CAAAAGGCCCTCAATGGTTA	59.931	20	135	3020	3154
60.017	20	TTGCCCAACTCATGTAGCAG	59.864	20	276	466	741
60.186	20	TATTTCGAGAAATGGCACCC	59.901	20	273	143	415
60.038	20	TGCGTTTTGACAATATATAGGTGT	60.083	26	194	2885	3078
59.91	20	CACCCTCTCCGAACAACATT	59.966	20	224	775	998
59.866	20	TCGGAGTAGATCTCAACACGC	60.412	21	239	43	281
59.844	20	TAAACGCCGGTGAGGTAATC	59.96	20	138	314	451
59.969	20	ATACCGGCCTAACCTATCCC	59.182	20	257	0	256
59.655	20	ATCGAAACACATCCCCAAAA	60.17	20	222	352	573
60.096	20	TCAACCTCGCAGATTGATTC	58.799	20	175	1148	1322
58.792	20	AAAACCAATGGTGGAGCAAG	59.971	20	236	896	1131
60.168	20	AATGCTCGAGGGCTAAAACA	59.845	20	208	138	345
59.071	21	TTGATCGATTGTTTTCTTGGC	60.067	21	147	668	814
60.209	26	GCAATAAGTGAGTGACTTCCCC	60.004	22	222	14	235
59.911	20	TGAAGTGTTATGCGTTTTGACA	59.274	22	169	94	262
59.476	20	TGAGTTATGAAGTGTTATGAAGGC	59.11	25	165	176	340
60.173	20	AGTCGCTGCAATTTCCAGTT	59.882	20	112	29	140
60.036	20	CACTCTCCTGCTCCAGCTCT	59.883	20	183	27	209
60.269	20	CGGTTAGTCCAGCCATCTCT	59.308	20	186	173	358
59.374	24	ACCAATGGTGGTATGGTGTG	58.984	20	102	17	118
60.262	20	ATTTCATTTCATGGGTTTCG	57.399	20	180	42	221
60.025	20	TGCGCTAACAAGGTCCCTAT	59.73	20	144	221	364
59.835	20	TTGAAGATCAACGGTCCACA	60.088	20	183	41	223
60.299	20	TTGGGTTGAAATGTTTACATGG	59.603	22	195	152	346
59.901	20	AAACGCCACTTCAAACCATC	59.978	20	120	307	426
60.104	20	CACCAAATTTGAGGGTTATTATTT(	59.905	25	260	187	446
60.202	20	CGGATTTAAGGGTTATTACGTG	57.682	22	212	36	247
58.956	21	TGAAATTAGATTCGAAATTGCAT	57.856	23	184	151	334
59.871	20	CAGTTGCACCCAACACAAAC	60.049	20	274	535	808
59.827	20	AGCCACTCCCTCCAAAAGAT	60.074	20	141	116	256
59.72	21	CCGGTTTCGTGAGCTTTCTA	60.378	20	240	30	269
59.534	21	CATCCAATTTTGAGGGTTGG	60.162	20	233	680	912
59.971	20	ACGGGAGAGGCATACAAATG	59.955	20	170	2183	2352
59.95	20	CGATCACACTCCAAATGCAC	60.12	20	237	773	1009
60.11	20	TCGAGGAGCGTGGTTTTAAG	60.378	20	181	37	217
60.423	20	TGGAACCATGGGTAAAACAA	58.719	20	246	123	368
59.532	21	CCACACGACATACGATACACG	59.92	21	128	244	371
59.921	23	TAATGCAAACGGGATGTCAA	59.93	20	249	2095	2343
59.972	24	TGTTACTTTTCATCGCACTCAA	58.489	22	243	1255	1497
59.883	20	TGCGTGATTTACACCCGATA	59.953	20	122	173	294
59.989	20	GGTTTTCTCCGGTGATCGTA	59.933	20	217	36	252
59.96	22	AGCAATTCTGATGGAATGGG	59.894	20	235	48	282
60.036	20	ACTTGTCCACCCACATGACA	59.846	20	232	1132	1363

60.081	22	CAATCCCCTCAAACAAAATCA	59.787	21	279	1097	1375
59.898	20	TTTGCAGTTGCACCAGTTTC	59.888	20	239	53	291
60.051	20	TCACTCGCGCTAGATTCTCA	59.847	20	125	276	400
58.189	27	TTGATTCTGGAATATCCCGC	59.862	20	242	2	243
59.81	20	TCGTTGTAAACCCGTAAGGC	59.996	20	220	81	300
60.008	20	CATCACACAAAACCAGTGGG	59.848	20	197	289	485
60.036	20	CCGGTATGCTGACGATAACA	59.566	20	241	1013	1253
59.465	20	ACAAATAGATCGGGGGAAGG	60.145	20	272	89	360
60.293	20	CACAACATGACAGCAACCCT	59.597	20	240	7	246
60.34	20	CATGCCTTAAAATCCACGGT	59.823	20	228	9	236
60.278	20	GAGATGGTTGCACCATAACG	59.002	20	212	31	242
59.685	20	GACGAGATCATGGCTCGATT	60.189	20	164	99	262
57.898	26	AAATATTTCTAGCACATGAAATTG	57.859	25	114	1	114
59.226	20	GGTTTTGACCATATTTACTGTTTTG	59.631	26	267	199	465
59.91	20	TCAACAACCAGTCCACTCCA	60.129	20	208	100	307
59.927	20	GCAACGGAATACCACTCGTT	60	20	163	87	249
59.621	22	TGAAAAGCTTTGAGTGTGCC	59.05	20	158	657	814
60.445	21	AGGGTTTAAAATCGCGCA	59.257	18	280	693	972
59.801	20	CAGCATATTCTTTGGACGGAA	60.081	21	247	124	370
59.668	20	ATCGCTCCACATGACACGTA	60.144	20	244	131	374
59.694	20	GTTGGTCCATTTGCCATTGT	60.624	20	216	48	263
59.549	20	TGAAATTGTCATCTCCCCCT	59.336	20	211	69	279
59.937	20	TTCTTTTACTTTGCATGCTACTTTT(	59.476	26	280	275	554
59.727	20	CTGGAAAGATGGTGGCAAAA	61.001	20	278	248	525
60.547	20	ACGGAGCAAATTCAATCCAC	59.939	20	278	244	521
60.034	20	GAGACCCGGGTTTACCTCTC	59.935	20	132	88	219
58.842	22	ACCATCGTTACCGATTTCCA	60.192	20	193	32	224
59.773	20	AAATTTAACATGGTCCGCCA	60.188	20	227	82	308
60.004	21	AAAAACCCTGATGTGGATGC	59.797	20	273	28	300
58.976	20	TTGGACCAAAATAGCCTCCA	60.439	20	166	10	175
59.259	20	CATTGATCATCACCCTGTGC	59.925	20	209	3	211
59.96	20	CCAAGTCTTGCATTTGGTTG	59.17	20	217	1568	1784
60.282	20	TCCCCAACTCAAGGACACTC	60.088	20	258	356	613
59.847	20	GAATCATGCTACTACACGTCGC	59.803	22	260	495	754
58.028	22	CCCATTAATAGCGATATATGTAAG	58.562	27	140	151	290
60.074	20	CACCTAACCCATTAACCCCA	59.541	20	243	49	291
59.787	20	TTACCGCTGGGATCATTTTC	59.901	20	181	98	278
59.783	20	GGATGGGTTATCACTTGAATAGG	58.769	23	102	153	254
59.989	22	TGAAGTTAGGAAATAAGGAGGTG	58.743	25	254	213	466
60.027	20	CCCTCCAAGGAATCTGATGA	60.003	20	239	14	252
60.333	20	CAAGAAGTCCTGCGTCAACA	60.025	20	173	1	173
59.635	24	GGGATAATTAATGCGATTGTGA	58.83	22	274	540	813
57.221	24	TCTGTTTTGAGCTGGCAATG	59.988	20	190	47	236
60.416	20	GCTCCTTGTGCATCACTCAA	59.992	20	268	226	493
59.61	20	GAAGTCTCAAGTCAATGTATGATG	57.445	25	115	570	684
59.706	20	GAACGGAGAAAATATCACGAAAA	59.512	23	237	576	812
60.088	20	TTTCTCAACCGAGCTCACCT	59.989	20	121	833	953
59.486	20	TTCCAGATGCCTCTCAGCTT	60.096	20	279	1775	2053
59.996	20	CACCCCGGATAGTAATCCCT	60.032	20	271	480	750
58.276	27	TTTCTCAAGGGTATGGGTGG	59.784	20	246	29	274
60.02	20	GCCTTGAGGGGGAGAATTAG	60.032	20	167	77	243
58.82	27	TGAAATAGGAAATGCAGGGG	59.894	20	272	2	273
60.111	20	TTTCACCACCTCCATTCCTC	59.903	20	273	65	337
60.029	20	CACTGTTGTCATCGGTCCAC	60.005	20	245	103	347
60.232	20	TGTGGAAGGGTGTGTGATGT	59.846	20	274	1388	1661
59.547	20	TGATCTTCACCTTAAATCTGCAT	57.9	23	259	70	328
59.123	23	AGTTTGGGGGAGGAGAAAAA	59.912	20	279	65	343
60.02	20	AAGGAGGTGTTATGAAGGGATT	58.028	22	268	40	307

59.81	20	AAGGTTGGAGAGGGGACAAC	60.349	20	224	10	233
59.967	20	GCTCGACGGAGGTTTGATAG	59.836	20	165	389	553
58.651	20	ACCTCAACAAACCACCCAAA	60.246	20	204	26	229
59.833	20	AAAATCACTAACCCACCCCA	59.146	20	191	34	224
60.199	20	ATGGCCATCTCTTTCCCTTT	59.903	20	196	402	597
58.745	20	CCCATTTTCTTCTGCTCCAA	60.184	20	183	1144	1326
59.923	20	TTGTCCCACATAGGAAAAATAAA	57.644	23	243	447	689
60.257	20	TTGTGAGTGAAAGTGGTGGG	59.565	20	118	716	833
59.476	20	GCCTCGGTGGAATTTATTGA	59.901	20	264	2392	2655
59.859	20	CATGAGAAATGGGGTTGCTT	59.933	20	180	32	211
59.751	20	TGCACAATACAAGCACCCAT	59.995	20	123	880	1002
59.726	20	CTCGTGTTTTATCCTCCCCC	60.683	20	107	4	110
59.919	20	ATGGTGGTTTGGTGGAAGAA	60.21	20	189	600	788
59.971	20	ATTATGGCGCCGCTATTGTA	60.452	20	152	253	404
59.844	20	AATTGTCTGGAGCCAAGGTG	60.111	20	235	243	477
60.065	20	TCCACGGTGATGCTAGAGTG	59.855	20	212	452	663
57.201	27	TGGGCTGAGTATGTGTTTGTG	59.624	21	145	3	147
60.049	20	TTCTGAAACATGATGGCGTC	59.654	20	239	1371	1609
59.485	20	CCAAGTCAATTGTCGATGGTAA	59.861	22	269	815	1083
59.928	20	AGCTTCCATGGAGGTTGATG	60.073	20	165	237	401
59.181	21	GGAAGCACTTTTTGAACGGT	59.218	20	254	395	648
60.582	20	TCAGCCGACTTTTTAATGGC	60.209	20	192	37	228
57.73	23	TTTTTAAATGGACGGTGATGA	58.005	21	117	14	130
59.962	20	GGTGTTTGTTGCTTCGCTTT	60.292	20	231	566	796
59.831	20	TCAGCAGAGCAAAAGGGTCT	60.134	20	265	1757	2021
58.909	21	GCCTAAGGAATAAACACCTAGCA	58.895	23	235	24	258
60.051	20	GGGATGAGTTATCACTTGAATAG[illegible]	59.765	25	215	291	505
59.904	20	CCTATGGTGGTATGGTGTGG	58.569	20	248	214	461
59.951	20	TACGTGATAGGATACGGCCC	59.807	20	276	44	319
59.973	20	TTCCGTTGGCAAGCTAACTC	60.386	20	268	751	1018
60.195	20	CATGAAAATCATGTTCCGCA	60.466	20	159	312	470
59.975	20	CTCCAAAATGGTGCAGTCCT	60.111	20	204	423	626
57.414	24	ACACGAATCAAATCAATGGC	58.424	20	277	27	303
59.894	20	CAAACAAGACCAAGTGGGGT	59.861	20	131	283	413
59.923	20	GACTTGTGTGGGGAATGGAT	59.636	20	272	364	635
60.172	20	ACCGACGTGGCCTAACATAG	60.015	20	261	240	500
59.656	20	CAAATGTTGCACAATCATTGAC	58.945	22	211	77	287
60.238	20	GTTTGGATATCGAGATCGGG	59.344	20	117	96	212
59.072	21	ATCATAGTTGACGCCCTTCG	60.096	20	103	19	121
59.993	20	TATTCCAAAACCATCGCACA	59.93	20	277	79	355
59.505	20	TGCTTTGGCTTGGTCTTTTT	59.861	20	224	1103	1326
58.516	21	TCGGAGTTCATCATGACACAA	60.104	21	186	346	531
60.053	20	TGTGGTTCGATATTTTGTTTGG	59.736	22	118	222	339
60.111	20	AGCCTGCAATTGATACCGAG	60.235	20	239	679	917
60.397	20	TCCTAACTTCATAACTCGAAGACT	59.858	26	279	0	278
59.991	20	ATTAACCGTGAAAATGGCGA	60.323	20	154	331	484
59.75	20	GCAAACCATACACGAACACG	60.035	20	204	108	311
59.53	26	CAGGATGGACTCGAACGAAC	60.656	20	144	9	152
59.573	21	TTTGTCACAGTATTTCATCATTCG	59.055	24	192	4755	4946
59.923	20	CGTCTGCGCTGTAGATTCAA	60.157	20	134	259	392
59.901	20	GCCGGAGATTATCCATCATT	58.812	20	270	85	354
60.356	20	AAGGCCGAGATTTGTTAGAGG	59.733	21	174	1789	1962
60.701	20	ATGGCTTGGAAAAGATGGTG	59.933	20	266	930	1195
60.339	27	AAAATCCTGTCAATGAAAAAGCA	59.997	23	273	89	361
59.303	21	TGATGGGCCATAATCAAAGG	60.66	20	149	368	516
60.088	20	TTGCAATGGACCTAAGCATTC	60.089	21	119	947	1065
60.074	20	AGGTTGATCATTTCCATCGC	59.9	20	164	1034	1197
59.679	20	GAGAATCCATGGCGGAAGTA	60.036	20	124	636	759

60.309	20	CCACCCATTCATTCCCTCAT	61.882	20	100	147	246
57.643	26	GCTACTCCCGTCGAAGATTG	59.836	20	234	13	246
59.83	20	GCCCAACAAATGAACCGATA	60.701	20	278	2118	2395
59.927	20	TCTTCCCCAAATTGGTTTCC	61.023	20	187	199	385
59.939	20	TTCTCTCATTTCCCCAAACG	60.044	20	228	484	711
58.756	24	CATGGAATGGCTGTCAGATG	60.072	20	106	20	125
60.278	20	TCATGAGGACGAATATGCCC	60.833	20	195	2	196
60.917	20	CAACGTTTGTTCGTGTTTGTG	60.096	21	212	18	229
60.153	20	GGCTACGAGAAATGGATGGA	60.036	20	144	705	848
59.522	20	CACACACACGTGGCATGATA	60.029	20	147	69	215
60.096	20	CCTCCCGTCTAATCTCCACA	60.065	20	239	20	258
60.562	20	CGGAAATGACCCCATAATGA	60.523	20	256	253	508
60.353	20	GCAGCTACGTTTGTGTGCAT	59.942	20	129	1229	1357
58.672	20	AGATCCATGTTCATCTCCCC	58.741	20	130	87	216
57.587	27	GATGATCACGATGAAGCCAA	59.61	20	172	77	248
60.134	20	ATTTAGCAAGCCCGATGATG	60.06	20	145	727	871
60.423	20	CCTCCAATGATTGCCAGAAT	59.894	20	166	364	529
60.202	20	TTCAATCTCAACCATCAATTTTT	57.674	23	204	478	681
60.216	20	CATATGGTGAATGCCCCTTT	59.645	20	163	254	416
60.103	20	CTGGTTTGCGTGGTTCTTTT	60.147	20	224	660	883
59.648	20	CTGTTGGCCGTTGTTTTACA	59.627	20	201	1138	1338
58.857	21	CTCTTCATTGACACGCGAAA	59.988	20	275	155	429
61.027	20	CCCTATTTAGCTTTCTTTGCGA	59.885	22	259	29	287
60.21	20	TATGGAATTCCGACGAGCAT	60.435	20	276	478	753
60.029	20	TGTGCAACTTGTCTGGTCTCA	60.494	21	275	566	840
60.031	20	CAGTTCTGGCCGTGCATT	60.84	18	209	656	864
59.402	22	TGGAATCCGAGAAAATCAGG	60.006	20	190	895	1084
59.761	21	CCTCCGTATTTTGGGTAGCA	59.953	20	200	1004	1203
60.035	20	ATGTGCGCGTGTACTTATCG	59.782	20	188	669	856
59.95	20	TATTGGGGGAAGAAGCACTG	60.066	20	231	2020	2250
59.664	20	TTGAATCCATTGGTGAAGCA	60.049	20	202	220	421
59.688	20	GAGAGAAACATTAATTCCTTCACC	59.9	25	179	49	227
59.888	21	TGGTGGAAAGACCAGACAGA	59.23	20	272	0	271
59.756	20	AAATCAAAATAGGGCAGGGG	60.143	20	215	31	245
58.914	20	CTTTTGCTGTGGCTTGTGAA	60.027	20	254	204	457
60.66	20	TGCAGGTTAGGTACCTTCGG	60.125	20	244	2796	3039
60.06	20	TACAGCCGCTCTTGGAACTC	60.538	20	124	75	198
59.364	20	ACGATTGAAACCCTCCCTCT	59.935	20	241	261	501
60.096	20	ATCCGCTTTCCCAAGTTTCT	60.074	20	264	3289	3552
60.565	20	ACCATATTTAAGGGTTATCACCTG	57.552	24	278	254	531
60.081	20	GTTTTGCAGGTGGTGGATCT	59.973	20	171	1179	1349
59.071	21	GCGGATGTAATTTGGGTGAA	60.701	20	231	249	479
60.055	20	AAACCGAGAAAGAAACATTTCAA	59.194	23	201	1102	1302
59.82	20	TTGTTAGTGCGTGTTTTGGG	59.627	20	179	29	207
59.928	20	CGAACTTAGAAGGGCCACTG	59.869	20	235	20	254
59.596	20	TCTCTTTTGGTTGGTGAAGGA	59.697	21	166	290	455
59.126	20	CCTTGTGTGATCAAGGGTCC	60.363	20	176	1771	1946
60.118	21	GCTAATGGTGCCACAGTTGA	59.722	20	264	37	300
59.702	20	ATCAAAGGCCACAAATGGTT	59.292	20	232	97	328
60.406	21	AACTTCTTGCTGGTTGAGGC	59.478	20	263	48	310
60.255	20	GGTTATCACCGGATCACGA	59.311	19	123	687	809
59.032	20	TCCCTTCCCCATCTCAATAA	59.315	20	274	1194	1467
59.934	20	TTATATCCCCAAAAAGCCCC	59.982	20	263	400	662
60.033	20	AGCTCACTAGGATGGCAAGC	59.603	20	201	218	418
59.577	24	AAAAATCATGCCACCGTACC	59.691	20	212	10	221
58.831	25	TTTTAACGAATAAACATGTTCCAA	57.778	24	107	103	209
59.021	26	TGTTTTCACGAATAAACATGTTAC/	59.448	26	276	131	406
60.576	20	ATCCATCATCTTCCGTGAGC	60.042	20	199	706	904

59.894	21	GGTTTTCAACCCATGTTTTTG	59.211	21	273	1565	1837
60.263	20	CAAATTTTCCGCTTTGTCGT	60.11	20	199	1032	1230
59.964	20	GGTTATTAGTTATTACGGATTTAAT	57.055	27	164	278	441
60.192	20	CGGACTTCCTTTCTGCAAAC	59.853	20	278	91	368
60.204	20	CTTCGAACATGCACACTCGT	59.905	20	139	449	587
60.096	20	TTGACCGAGTGAAGTAAACCC	59.087	21	241	3285	3525
59.691	20	TTGACATGAGCGCATAAACC	59.694	20	161	941	1101
60.324	19	GCCCTCAACTCTCCCTCTCT	59.952	20	104	9	112
58.522	23	CCGGATCAGCTCTCTTTCTC	59.116	20	180	1608	1787
60.384	20	TGTCGTCTATCACCTTCCGTC	60.126	21	191	63	253
59.927	20	TTGATTCCTTTTGTTCCCTATTT	58.142	23	269	226	494
58.272	27	GGGAGCTTCAGTCTTTCATCTC	59.466	22	118	14	131
60.051	20	TAGAGTTCCAAGAGGGGGCT	60.204	20	202	5	206
60.147	20	TCTCTCAACCTCACTCGCAA	59.701	20	264	418	681
59.104	22	TCTACGACCGAAATTCACTCC	59.184	21	104	23	126
58.811	21	CGAACAAGGGAAAAACAATG	58.121	20	126	90	215
59.848	20	CAAGGGGAGAGAGGTCAATG	59.649	20	266	425	690
57.955	21	TCAACTTTTGACGGTCCGA	60.224	19	280	140	419
59.89	20	AGGATCGGCATATCGAGAAA	59.628	20	276	146	421
59.13	25	TTCTGACTTTGTTTTTGTGCAG	58.135	22	184	39	222
59.991	23	TGACTTTGATCAAGTGATAACATC	60.287	26	174	386	559
60.192	20	TCGGATAATCTGAAAGCGAA	58.455	20	263	1083	1345
59.955	20	ATCCTCCCCTTCCTTGCTTA	60.032	20	150	478	627
59.549	20	CATGTCAAAAATGCCCCTTC	60.309	20	268	483	750
59.499	21	CCCTCCTCACCTCCTCTCTT	59.801	20	263	933	1195
57.551	24	ACCTGCACCAAAATGGAGAG	60.111	20	257	60	316
59.792	20	CCGAATCCATTCCCTACTCA	59.887	20	263	2825	3087
59.484	19	CGGAGTGTTTGGGTAGTCGT	60.028	20	241	150	390
60.214	20	CCACTCCACAAAGATCTCACA	58.71	21	104	212	315
58.826	22	ATGTCCGTCGTTGGTTCTTC	59.973	20	173	24	196
58.45	20	TGCAATGCTTCAATTCATCC	59.628	20	230	0	229
61.027	20	TCGATCACGATAATCACCCA	59.883	20	199	16	214
60.096	20	AGGAGGGGAGTTGTTGGGTA	60.736	20	278	620	897
60.142	20	AAGGTGTTGTTGGGTTTTGC	59.875	20	256	242	497
60.103	20	AAATCCCTTTTCCTCTCCCA	59.875	20	147	245	391
59.777	20	TTGAATGGTTATCACCTGATCG	59.823	22	129	106	234
60.659	21	GCGGAAATTACTTACAGCCATC	59.996	22	178	295	472
59.989	20	GAATCCATGCAAACAGCAAA	59.67	20	161	2331	2491
59.411	20	ATGCGCGTTTTTAGGGTTTA	59.624	20	151	394	544
59.855	20	CATGTTTGGTGACTTGGTCG	60.001	20	244	16	259
59.904	20	CATCCAGGAACCACTTTCGT	59.966	20	265	225	489
59.975	20	AGCCCCCATTTTTCATTTTC	60.13	20	146	253	398
59.751	20	TCTGCAGTCTCCGTTTTTCA	59.566	20	137	407	543
61.532	20	CATGCCAAAACTCCACTCCT	60.111	20	262	298	559
59.88	20	GGACCGGACCGTTAGGTAAT	60.074	20	277	219	495
59.945	20	CCCACATTGGTTGGAGATTA	58.27	20	218	148	365
59.94	20	GAGGTTTTGACGAGATTTAGGTG	59.187	23	142	391	532
60.323	20	ATGGTGGTATGGTGTGGAGG	60.51	20	139	218	356
59.967	20	GAGCGAGTGGTTGTGAGGTT	60.307	20	280	27	306
60.263	20	CGAAAATGAGCTTCCACACC	60.636	20	162	241	402
57.496	21	TTTGATTCTTGAGTACACCATCG	59.144	23	171	57	227
59.148	21	CCATTGACTTGGCATCAAAA	59.518	20	279	109	387
59.992	20	CCATTTCATCTTCGGCATTT	59.901	20	231	74	304
59.941	20	TGATTTTTCATGTTTTGGAGCA	60.468	22	215	302	516
60.578	20	CGTTCCTCTCTCTCTCGGTG	60.127	20	167	60	226
59.962	23	GGCTTGAAGAATGATGAAGATG	58.789	22	113	132	244
59.781	20	CATCATTGTTTCGCAAGCAG	60.401	20	209	1007	1215
60.096	20	TCAACCATCTTGTTCTGTGGA	59.124	21	198	698	895

57.104	23	TGGTACTTAAAATGAGAGAGATG	59.913	25	243	303	545
59.797	20	TGTATCGTCCGCGATGTAGA	60.243	20	152	209	360
59.474	20	CGGCCGGTTTGTAACCTT	60.883	18	256	2169	2424
59.884	23	TGCACCCTTTGACATCAGTT	59.139	20	180	50	229
59.685	20	TTTTTGTTTGTTCCCAGGTTG	59.862	21	105	21	125
58.456	21	TCGTGTTCCGATCCTAGCTT	59.836	20	217	17	233
60.255	20	TAAAACAGAAGCAGACGCGA	59.752	20	154	41	194
59.917	20	TGGTGTGTACTTTTGTAGCAATCA	59.618	24	259	106	364
60.34	20	CAAAAGAGCTGTGGGAGAGC	60.134	20	215	87	301
59.874	24	GATGATGCATGTATACTATCAAAA	57.305	26	245	1336	1580
60.305	20	GGGAGGGTGGTTTGGTTAGT	60.088	20	266	17	282
57.333	24	TCACACTCACGCACTCACAA	60.07	20	121	3	123
59.805	20	TTCGTAGCATGATTTAAATGGTTT	59.021	24	242	575	816
59.827	20	TCCCAAGTGCACAAGTGTTT	59.187	20	276	295	570
59.823	20	ATGATTGGGACACGTGTTTG	59.267	20	196	1618	1813
59.726	20	GTGCAGTAGGGGTTGTCGTT	60.035	20	243	92	334
59.988	22	GGAAATAAGGAGGTGTTATGAAG	59.323	24	137	809	945
60.333	20	TTACAAACACCGATTCGCAC	59.59	20	146	62	207
60.148	20	ACGAAGGGTATTTTGGGAAA	58.417	20	179	208	386
58.974	20	GTAAACCGTCACCCCAAAAC	59.195	20	125	53	177
59.624	22	CCGTCGACAAGCTCAGAGTA	59.187	20	190	4	193
59.992	20	TGTATCACACAACGGTTCATCA	59.888	22	241	246	486
59.94	21	CACCAACCCAACCCATTAAC	59.948	20	105	54	158
59.916	20	GGGTGGTTATGGTTTGTAGGTG	60.385	22	171	1337	1507
60.187	20	CTCCGCCTTGACTCCTTCTA	59.569	20	109	308	416
59.823	20	TCGACCAAAATTTAAAAGTCCT	57.036	22	273	834	1106
58.874	22	TGTGGATATGTAGATGATTTTTGG	59.637	25	184	11	194
60.139	21	GGAGGTCAGGATCTGGTCAA	60.048	20	162	797	958
60.265	20	CCATCACACTAACCTCCCTCA	59.974	21	259	150	408
58.436	26	AAATATCGGCCGTCAGATTG	59.923	20	189	1	189
59.03	22	CAAACCTTGGCACTTGCATA	59.729	20	100	94	193
60.36	21	TGCATAGGCTGTCTCTTGGA	59.547	20	227	108	334
60.362	20	TGGTGCATGAGACGAGAATTA	59.287	21	163	177	339
59.425	21	TTGGAATTGGAACCAGCTTT	59.546	20	272	88	359
59.96	20	GAGAAGGTAGGGGGCTAAGG	59.191	20	180	29	208
60.554	20	TACCATGTGCTTGGAGTTGG	59.566	20	277	1015	1291
59.024	20	CCATGAATCTTTCACATTTTTCA	58.976	23	207	219	425
60.382	20	TGGTATTGGTCCACCTGTGA	59.806	20	207	360	566
59.95	20	AAAGTCAGCCGAGAAATTCAA	58.962	21	143	752	894
59.894	20	TTGACAATATTTAGTGTTTTGACAT	58.99	27	187	63	249
60.131	20	TTGTCAAAATCTCATTCATTTGC	59.114	23	126	50	175
60.257	20	CACACGAGCCTTCTTCTTCC	59.989	20	274	70	343
59.875	20	CCTTGAGGACTTCTTGGGGT	60.48	20	118	67	184
58.695	20	AGTCCTGGCACTTTGCTTGA	60.975	20	274	540	813
59.797	20	GTCCTCCGAACTCATGGTGT	59.969	20	179	73	251
60.073	20	CGCATCAACACACCAATGAT	60.399	20	216	52	267
60.195	20	TGGGACTCAAGCTGTTGTTG	59.873	20	240	1078	1317
60.21	20	GTTTGACAGCCCGACAAGAT	60.119	20	247	200	446
60.14	20	GTCGTGCTTAAACGTGGGTT	60.037	20	233	534	766
60.924	20	GAAAGCCTACCTCGCTTCCT	59.983	20	178	642	819
59.801	20	AACCGGTTAAAAACCGAACC	60.089	20	121	391	511
57.845	21	GGAGATCTTGCAGGATAGGCT	59.825	21	255	6	260
59.971	20	TTCACCGGATGAAAAAGGAG	60.044	20	268	67	334
60.157	20	GACGACCGATATTTGCTCCT	59.154	20	155	922	1076
60.088	20	TTCATGAAATCAAGCAAACCA	59.147	21	101	13	113
59.934	20	GCAAGAGTGAGTGAGGAGGG	59.986	20	123	339	461
60.012	20	TCAAGACAACCGAGCAAGTG	60.025	20	187	163	349
59.875	20	CCAACCCATTCTTCACCAAC	60.21	20	212	184	395

59.929	20	ATGGGAATCGGGTAAAAACC	59.888	20	255	225	479
60.081	20	GGGTTCGTTGTTAGCTTTCG	59.747	20	268	284	551
57.001	22	TGTTACGCCCGTGATAATGA	59.953	20	205	1143	1347
60.288	19	TGGATCCCAATGAAAATGAAA	60.117	21	276	390	665
59.986	20	TGAAGAATGAAGGGAATGGG	59.864	20	174	760	933
60.029	20	AGAGCGAGAGATGGACGATT	58.995	20	211	75	285
59.457	20	CAGAACTTGTCGTGGTGGTG	60.194	20	250	109	358
60.196	22	CTCCGGCCATAGTTTCTCAC	59.694	20	126	95	220
58.466	23	CTGTTCTTGCGCAAAATTATC	57.614	21	168	706	873
60.168	20	AAAGAGGTGTTATGAAGGGACAA	59.056	23	220	18	237
60.269	20	AAGCACACAATAACCAAAGTATT(	59.08	25	153	64	216
60.073	20	TCCCACTTGCTTGTTGACAT	59.139	20	186	39	224
59.989	20	CCAGCTTCGAACGTTGTATGT	60.183	21	213	1919	2131
59.276	20	TCAAGGAGTTGTACCTGATAGCC	59.666	23	253	1478	1730
59.502	23	TTAGCAGCAGATGCAAAGGA	59.712	20	125	538	662
59.966	20	TGAAATCAGGCCATCTCCTC	60.158	20	221	7	227
59.971	20	CTGTGCTGCTGGAAAATTGA	59.988	20	235	340	574
59.68	23	CACCAGCACCATCGTACATC	59.992	20	211	23	233
59.547	20	GAGGGGGAAAAGAGAGGATG	60.008	20	185	183	367
59.553	22	TCAGCTGACATTGGGTTGAG	59.831	20	268	1372	1639
60.371	19	TCGTAACTGAAAATCATCGGC	60.089	21	246	355	600
60.008	20	TTGGGAAAGCACTATGGACC	59.933	20	241	53	293
60.053	20	TCACTCATTCAAGAACATAGCCA	59.761	23	177	375	551
59.933	20	TGACATGCTCTTAAGGATGGG	60.081	21	100	217	316
59.94	20	CTCATAGTCAAACACGATTGCTT	58.464	23	177	77	253
59.5	20	GGACATGAAGTAACTGCACATGA	60.052	23	272	200	471
59.583	21	GAAGTGGGTGTGTGCTGAAA	59.726	20	173	274	446
59.742	22	TTCCAGGAAATCGGGAGAT	59.425	19	259	401	659
59.801	20	TAGTACGAACACGCGCAGAG	60.217	20	280	512	791
60.595	24	CATTGCACGTACTTAACCAAAA	57.851	22	269	17	285
59.827	20	GGGGTGAATGATCAAGATGG	60.135	20	177	11	187
60.044	20	AGAAGGCGGATAAGGTTGGT	59.962	20	272	3617	3888
59.999	21	CTTCATCACCTTCCCCATTC	59.336	20	249	171	419
60.059	21	GAGGGAGTTGTTTAATTGTCGG	59.871	22	276	97	372
59.415	20	ATTTCCAAATCGGTACATGC	57.424	20	273	881	1153
60.08	20	AAACCCATTTATAACGGCGA	59.323	20	211	258	468
60.134	20	GACTTACGTTCCGCCAACTC	59.74	20	215	1963	2177
59.526	20	CTCGTCAACAGCTCGGTACA	60.049	20	259	135	393
60.119	20	TCCATCTTCGTCCACCTCTT	59.655	20	107	15	121
59.694	20	CTAGGGTTAGCATGCTTGGG	59.724	20	220	2	221
60.008	20	GTCCTCGCGAAACAGAGATT	59.434	20	135	317	451
59.325	20	GCTGGGTAAAACTGAATAATACC/	58.575	24	109	129	237
59.458	20	TTTGCCTCTCCATCTCCATC	60.158	20	277	5	281
60.048	20	GCAAAACTTAGGAACGAAGCA	59.527	21	218	24	241
59.923	20	TTGAAATCCTTGAACCGTGTC	59.963	21	153	150	302
60.466	20	ACCATCTCGGGTTGTAGTGC	59.997	20	163	630	792
60.066	20	GCTTTATGTTGTTGATCCGTCA	60.003	22	152	30	181
60.088	20	CAGCAGATGCTTTCATCCAA	59.948	20	132	922	1053
59.978	20	CCGTCGTCGTTATGAAGAAAA	60.117	21	149	106	254
57.815	24	GTGAAATTATTCGTTGATAAACTC'	57.681	27	233	305	537
60.012	20	TGGTGGGTTGAATGCTATGA	59.924	20	240	1448	1687
57.051	25	ACATGGACGTCCCAGATTGT	60.246	20	131	2	132
60.636	20	GGTAGGGTGGGGATGAGATT	60.015	20	259	123	381
59.986	20	ATGGAAGAAAAACCCAACCC	60.032	20	245	210	454
57.433	26	CCCTGCACCTCTTTCAAATC	59.67	20	194	16	209
59.564	20	CGTTTTCCCTATTTTGTGCAT	59	21	251	81	331
60.214	20	TAAAACAATAAGGTCGCCCG	59.958	20	241	871	1111
60.075	21	AGGTGGAATAGGCAGTGACG	60.134	20	274	99	372

60.365	20	GCGAGCGAGTAGGTTGTGAG	61.136	20	110	2	111
60.162	20	TATCATTCTATGGTGGGGCG	60.681	20	202	830	1031
60.166	20	ATGGTTCGCCATAAAAATGC	59.801	20	162	37	198
60	20	CTTCACCCCCTACTCCATCA	59.92	20	266	393	658
59.895	20	TCGCTATGCACACTTTCTGG	60.011	20	127	646	772
60.224	20	GGATGAAGGTGTTCGTTCACA	60.95	21	115	355	469
59.968	20	CGCGCCTCTACTTTTTAATTACC	60.473	23	190	797	986
60.903	20	CTCGACATTGTTTGAGTGCAA	59.896	21	175	211	385
58.411	20	TATGCCATTTTCATCTGGGG	60.66	20	246	1885	2130
58.931	21	GCCATTTAAAAAGTCGGCTG	59.72	20	226	1109	1334
59.75	20	TTAGGAATGGGATGGTGGTG	60.572	20	267	82	348
59.916	20	TAAACGGGCCAATTTAAACG	59.835	20	240	118	357
58.155	20	GCTTGCACCCCAAATTAAAA	59.943	20	167	0	166
60.117	20	TACAACACTGACCATCCCCC	60.634	20	127	3919	4045
59.939	20	CTTCGCCACTGTTACCCTTC	59.734	20	250	791	1040
60.234	20	ACGTGTCCGAGTGTGTTGAA	60.203	20	119	79	197
60.249	20	ATGCAAGCTTTGTTTGGGAG	60.249	20	244	698	941
60.103	20	GTGTGTTGTTCTGCACTGGG	60.203	20	257	27	283
59.049	20	CTGGGTATACCTTGCCTCCA	59.948	20	180	111	290
60.205	20	TCTATCGGATTAAATGGAATAAGC	60.184	25	102	9	110
60.214	20	TCGGTGTATGCTGTAAACGAA	59.24	21	134	112	245
59.859	20	CAGTCACGACTTGCCAAAAA	59.881	20	221	852	1072
59.975	20	TCGTATGAATTTTTGTATGGACG	58.927	23	194	605	798
59.894	20	GGTTTACCCATAGCGCACTC	59.597	20	156	809	964
59.086	20	CCAACACTATTCGGTTTGGG	60.22	20	271	434	704
59.17	20	TGAATCCTCACAATATAACCTATT(	59.283	27	232	86	317
59.982	20	AGCCAAATTTGCAACCTCAG	60.249	20	279	1215	1493
59.43	20	TCAATTATGTCATGAATGTTCAAA	57.148	24	224	76	299
60.245	20	TTGTCGTGGTTCGGTATTCA	59.964	20	272	53	324
59.861	20	ACTGACATCCACAGGCATTG	59.551	20	122	76	197
60.221	20	TTTAACGACCGAGTTTTGGC	60.11	20	265	521	785
58.22	27	TGCAAATCCAAAGGTCAACA	60.088	20	135	0	134
60.134	20	GTCGAGGGTTTGGAGCTGTA	60.255	20	245	8	252
60.002	20	TTGGTGAGTAGGATGAGGGC	60.073	20	270	1336	1605
59.547	20	TCATGAATGAAGCAGAAGAGGA	59.955	22	267	260	526
59.96	20	ACCCCAGGCTGAGTGAGTTA	59.721	20	252	320	571
60.21	20	TTCAAAACCACCTTCGAACC	59.948	20	214	483	696
60.073	20	TTATCATAGGATCACCCTCCC	57.807	21	154	59	212
60.472	20	TGTATGCAGTCCATTTCCGA	60.073	20	274	637	910
58.82	21	TCCAAATTACAATCGTCGCC	60.837	20	274	147	420
59.823	20	ACGTTCGGCTCGAGAGTAAA	60.015	20	169	79	247
59.969	20	GTGTCGACCTCGATGCATTT	61.078	20	275	818	1092
60.074	20	CCCAAAATTGCGAATGCTAT	59.928	20	222	608	829
59.934	20	TCATTATCCTAACTCGGATGTGG	60.21	23	200	398	597
58.568	20	CAACCTCTGCAAACCCAAAT	59.971	20	275	373	647
60.061	20	ATCTGGTATCAGAGCCACGG	60.096	20	275	71	345
59.957	20	GGCTTCCATGGTGGTTTAGA	59.933	20	105	14	118
60.038	20	CAACCATCCCAAACTTCACA	59.389	20	157	333	489
59.923	20	TTGTATGCTGAGGCTTGTGC	60.019	20	228	30	257
59.929	20	AGCCACTAAGCCACCTTCAA	59.875	20	275	592	866
57.635	20	CCAAATTTAAGGGTTATCACCTG	58.849	23	151	98	248
59.375	20	ACATAGAGAAGGGTTATCACTTG/	57.537	25	154	70	223
59.476	20	TCAAAGCCCAAAAGTATGGC	60.074	20	214	81	294
59.923	20	GCGGAATACGAAGAGTCACC	59.7	20	244	14	257
60.192	20	CGAAGTAATGCTCACACGGA	59.864	20	263	55	317
59.645	20	CAAACACCGATTCACATTGG	59.816	20	167	269	435
60.471	20	TTGCTGGTGGAAACTCCTTT	59.711	20	279	1698	1976
59.982	22	TTGGCATCCATCGTAACAGA	60.073	20	169	11	179

59.977	23	GGGCATACGCTTCGATACAT	59.951	20	209	23	231
60.7	20	CTTGTTCCTATTGCCAACGA	58.771	20	177	87	263
59.745	20	TTCACTCACCCTTTCGATCC	60.05	20	267	1409	1675
60.204	20	CTGAGATTTGTACTGCGCCA	60.011	20	208	18	225
60.195	20	GGAGTTGGGCTAAGTGGGAT	60.328	20	220	158	377
60.255	20	CGCTTTGTTTTCGTGTTTGA	59.888	20	255	394	648
59.88	20	CGTTCATTTTCTGTCCGGTT	59.971	20	155	441	595
60.096	22	ATGCGCGATTTTAAACCCTA	59.586	20	175	5005	5179
59.937	20	GTCGAGGTGGTCTCAACGAT	60.12	20	269	47	315
58.984	20	CAACACAAGGACATCCATCG	59.96	20	141	888	1028
60.399	20	AAAGGGCGATGACACAAAAC	59.978	20	124	144	267
59.973	20	ATCACGAGATGGGGACTCAG	60.072	20	278	1190	1467
57.536	27	CTGTGTTAAATTTTGGAAGAGAAA	57.213	25	280	96	375
59.996	20	GGATACCATGGGCAACCTAA	59.645	20	280	904	1183
59.673	20	CGCTCCGTAATTCTCACTCC	59.836	20	219	58	276
58.703	20	GAACGAGCATCCATGTCAAA	59.654	20	267	184	450
58.008	26	AATTGAGAAGGGGGAGAGGA	60.008	20	132	59	190
59.96	20	AATCCTTTCCTCTTTCCCCA	59.875	20	219	150	368
60.073	20	CACCTCTCCGATTAAACCCA	59.926	20	154	340	493
60.012	20	TTTCTTTGAGTGGGATGCAA	59.247	20	266	114	379
59.925	20	TTGCTTCTCAAACTTGATTAGGTA(	58.301	25	204	5	208
60.081	20	AAACGAAAAATTGGGGTTCA	59.292	20	230	26	255
59.425	20	AAAACCGTAGTGGGTTAAAATTC/	60.041	24	182	96	277
59.676	20	TTGGGTGATGTGCTTGAAGA	60.24	20	127	162	288
60.12	20	TTGAATTCTATGGCTTCCGC	60.175	20	208	352	559
60.031	20	TCTTCGTGTTCTTCGTCGTG	60.025	20	232	3483	3714
59.948	20	TGGAGGAATGAAGGTGAAGG	60.042	20	105	108	212
59.989	20	GAAGAAATTCCAATAGAGTTGAT(	57.932	25	254	18	271
59.763	20	TGCCTAATTTATAGATAATGGACC	59.68	25	274	1070	1343
60.144	21	AAAATATGATCTTACTCCCACTGA	60.035	26	205	611	815
60.096	20	GGGGTAACTTGTCATCCCCT	60.052	20	138	12	149
59.801	20	CTGAAATCCTGGGCGTAGAT	59.15	20	275	91	365
59.831	20	TTTGTCCAAACAAATGAAAACA	58.097	22	257	253	509
60.243	20	CTGTAATCCCGATTGCTGCT	60.235	20	118	805	922
59.813	20	ATTGTGACATGGCCGAAATC	60.733	20	183	149	331
59.823	20	AAAGAAGACCGAGAGACACACA	58.991	22	159	0	158
60.228	20	GAGAAAAGACAATTCGGCCA	60.192	20	180	67	246
59.581	20	GCACACGAGCTCTCACTCAC	59.771	20	247	400	646
59.96	20	TAGCCGATAAAAATGGCACC	59.928	20	224	107	330
59.759	20	GACTCTCGCACCATCCATTT	60.081	20	220	270	489
59.889	20	AATTTGTACACACGTCCACCA	58.813	21	250	81	330
59.304	20	GCGTTGATCCAACGGTCTAT	59.962	20	208	149	356
60.578	20	CATGATCCAACCTTATTCCTCC	59.666	22	241	30	270
60.08	20	AGTCATGCGATCTGAACGTG	59.862	20	223	48	270
60.376	23	GCATGCTCTGTTTTCTGCAA	60.142	20	278	3	280
60.873	20	TGCGAAAATTTCTGGAATCA	59.232	20	123	318	440
60.007	20	CAATTTTGTCTGCTGCATGG	60.257	20	138	467	604
59.94	20	TTTGCAATATTCAACTACGAGCA	59.802	23	188	56	243
59.406	20	TGCAGATCCTCGGTACCTTT	59.694	20	232	384	615
59.337	20	TTACGAAAATACACGTTCGGG	59.876	21	214	20	233
61.235	20	TCCCCAGTGTCACACAGACT	59.113	20	258	259	516
59.659	21	TCTGCACGCTGCAATAACTC	60.165	20	262	1149	1410
59.685	20	TTTAGGATCGGAATATCGAGAAA	59.122	23	280	5	284
60.035	20	ACTTTGGCAAATCACACAGAT	57.186	21	276	164	439
60.206	20	ATGTTTTGTGAAAGTCCCGC	59.978	20	117	523	639
60.299	20	GGTAAAGAAACGGTGGAAAGG	59.858	21	158	122	279
59.933	20	GATAATTAGTGGAGGCGGCA	60.06	20	128	1530	1657
60.004	21	TGAAGAGAAGAGGTCGGGAA	59.92	20	213	30	242

59.756	20	CGTTATGGATGTGCCATTTG	59.809	20	236	762	997
60.255	20	TACCGACGAATGGTTGTTCA	59.964	20	274	507	780
60.298	20	AGCTTCAGGCCTTCAGTTTG	59.615	20	223	1363	1585
60.903	20	TGTTCAATCTGCCACCAAAA	60.088	20	255	1079	1333
60.323	20	GTGTGGGGGATTGTTGAAAT	59.508	20	280	87	366
59.839	20	CACAATCCCAACAAAAAGAAAA	59.007	22	238	2	239
59.566	20	TGCAAGTAAAACCATGCTCG	59.872	20	251	153	403
59.933	20	AGTTGCTTAGCGACCCCATA	59.73	20	257	4027	4283
59.239	20	GGTTCGGGTATTCACGTTGT	59.717	20	259	685	943
59.68	23	GGAGTTGCAGTTTTGGATGAA	60.103	21	193	207	399
59.143	20	TGTTCTGTCCTTTGCACGAG	60.025	20	268	23	290
60.405	20	AGACACCTAAGCCCATTCCC	60.328	20	153	27	179
59.089	24	CAAATTTCGGTTTGTTGGCT	59.975	20	133	32	164
59.943	20	CAAAAACCGAAAAACCGAAA	59.951	20	266	425	690
59.803	20	TACCCAACCCACTAACCCAA	60.081	20	140	63	202
60.439	20	CCTAACCCCACTAATGACCC	58.24	20	112	688	799
60.015	20	TTCGTGATTATTGCTGCTGG	59.833	20	140	514	653
60.11	20	CACATGTTGAGGTACACGGC	60.032	20	216	138	353
60.103	20	TATTACAGGGGCCAGTCGTT	59.452	20	231	2912	3142
59.916	20	ACCACCATCTTCTACGACCG	59.989	20	217	307	523
59.729	20	GAAGCCTCAAATCACAACCC	59.532	20	218	352	569
59.962	20	CAGTTGAAAAATTGCCAAAACA	60.014	22	187	610	796
59.476	20	AGCCATTTAAAGTCGGCTG	59.361	20	154	30	183
60.015	20	CTGCTCTCTTGATTCATGGCT	59.588	21	252	1121	1372
59.928	20	ACCTTGAGGCATTTGAGGAG	59.284	20	257	216	472
60.06	20	TCGATTTGGAAAACAGAGCA	59.395	20	242	360	601
59.95	20	TGATGCCTCAAATCACAAGC	59.805	20	198	1073	1270
60.504	19	CCACTCTAGACCCTAGCCCC	60.088	20	194	162	355
60.028	23	GCATTATAACCACCGCAACA	59.448	20	250	74	323
59.717	20	GAGTAGGAGTCCGGAGCAAA	59.43	20	240	240	479
60.19	20	TGACCGGACGAAAATACTCC	59.933	20	182	202	383
59.585	20	TTGGCCTGTTACGACTCAAG	58.923	20	147	159	305
59.928	20	TACAGTCGACCTCAGCTCCC	60.408	20	208	21	228
59.773	20	TAAAACCGACATCCCCACTC	59.79	20	110	2	111
59.847	20	CATTTGGAAAATCAATGGATGA	59.638	22	206	751	956
59.827	20	TTCAATACTTTGAGGATAGATTGA	59.474	27	267	20	286
60	20	GTGTGTCACTCCCCGATCTT	59.969	20	203	18	220
57.31	27	TTTATCAAAAGCCGAAACCG	60.068	20	169	10	178
60.291	20	TGTTGTCCGGTTCTCTCTCC	60.238	20	256	274	529
59.993	20	ATACCGCTTCCAAAATGTGC	59.967	20	143	2858	3000
60.357	20	AGCGGTTATTACCGGATCG	59.93	19	230	46	275
59.927	20	TCACGCACTCACGATCTCTC	60.146	20	151	999	1149
59.943	20	TTTTACGGGTTGGGCCAT	61.09	18	280	708	987
60.111	20	TGAGGGAGGGAAAGAGAATTAAG	60.076	23	129	176	304
59.303	21	TTGACGCAGCTTGAAGATTG	60.134	20	232	4	235
60.074	20	GGAGAAACTGACCGGATTGA	60.05	20	148	1091	1238
60.001	22	AGGCAATTAAGATTCGCCCT	60.061	20	185	6	190
58.462	21	CCGATCCCATCCCTTAATTT	59.978	20	256	249	504
58.78	20	ACGCTCCACCAAAAGGAGTA	59.734	20	227	26	252
59.809	20	GGAAATGTGATTTGATGGGC	60.141	20	162	31	192
59.083	20	CATTCCTTTTTATGTGTTTGTTTCA	59.38	25	242	1125	1366
59.315	20	TGTGGGTTAGCATTTGATCG	59.542	20	242	622	863
59.943	20	TGCTCTGAGAGGCCTGATTT	60.096	20	254	494	747
59.81	21	GTTAAAAACCGGACTGCACC	59.476	20	138	52	189
58.38	20	CCACAATTTTAGGCAGTTTCG	59.634	21	163	483	645
59.688	20	CCCATTTTCTCCATCCCTTT	60.124	20	141	224	364
60.54	19	CAAAATCTTACACCTGGAGCC	58.707	21	277	232	508
60.11	20	ATTTTTCGCCGAAGAGGATT	60.041	20	270	553	822

59.429	20	TCCGAGTTGAAGTAGTGGCTT	58.994	21	216	251	466
59.366	24	GCGTTTTGATGATATATAGATGTTT	59.327	27	189	306	494
60.066	20	ACCGTCCATCTTAATGCAGC	60.103	20	258	2789	3046
60.323	20	AGGAAAATGTGTCGAGAGGG	59.137	20	280	129	408
59.797	20	TTTCCTCCCAACATTCGTGT	60.353	20	224	88	311
60.096	20	TGAGGAGCGTTGATCTTGTG	59.984	20	200	271	470
60.507	20	CAGACGGTATTGCCCTTCAG	60.647	20	244	1175	1418
59.509	23	CCCGGTGTAAAGAAATCGAA	59.931	20	205	26	230
59.697	20	TCTGCAAAAATGAACAAACTATAC	59.639	25	178	12	189
60.038	20	CACGAGTGAAGAAAGGGAGAG	59.072	21	143	41	183
59.312	20	GCCGCGAAACAGACATAAAT	60.103	20	151	175	325
60.173	20	CGGTAACACAAAATTGCAGAA	58.736	21	240	2179	2418
60.142	20	AACATGCTTCACCATATCCAAG	58.976	22	280	928	1207
60.061	21	ATATGGAAGGCAGTGGTTCG	59.955	20	270	405	674
59.46	24	ATATGTTCACCAAGCCGCTC	60.103	20	226	6	231
60.059	20	GCAAGCAAAATGCTACGAGA	59.21	20	244	99	342
60.151	20	CGCGAAGACAATGTTGAAAA	59.849	20	265	25	289
60.217	20	TGTTTTGTAAAGGGGTCGAGA	59.595	21	246	845	1090
59.934	20	TGCAAGAAAGTGGACTGCAA	60.574	20	199	517	715
58.847	20	CTCTCAATGGACCAGCACCT	60.261	20	264	1466	1729
59.262	23	GACACAATTAACCAAAAACATTGC	59.705	24	242	332	573
60.155	20	TTTGTCTGCGTGGACAAGTT	59.339	20	240	217	456
60.044	20	TAATGCATTCGCCAACTGAG	59.833	20	213	125	337
59.861	20	TCGTCGTCGCTTTCATCATA	60.366	20	126	2473	2598
59.953	20	ATTTTTGTTGGCCGAAAATG	59.81	20	170	261	430
58.687	21	AGGTGCCCCTACTTCTTTGG	60.488	20	245	1	245
59.528	20	TGCTCTTGTATGCTGCGTTT	59.638	20	275	659	933
60.111	20	CGACGGATATTTGTTCTTGTG	58.161	21	196	157	352
60.352	20	TTCCCATCTAATCTCGTCCG	60.029	20	183	746	928
59.597	20	TTTGGGAGACAAACACACTCTC	59.232	22	233	92	324
59.588	20	CCACCCACACCTAAGGAAGA	59.959	20	118	3	120
59.175	20	CCCTAATACCCACTAACCCCC	60.643	21	110	1	110
59.941	20	TCATTGTGATGTGTGGGAAAA	59.808	21	219	874	1092
59.82	20	CGACTTGTCGTTGTTCGGTA	59.758	20	265	221	485
60.34	20	GAGGGACAGCCTAGAACTTTGA	59.886	22	134	294	427
59.986	22	GAAGTGCATCTCGATGACGA	59.95	20	165	38	202
59.82	20	CCACCTGAAAAAGGGTTGAA	59.942	20	265	218	482
59.612	21	CCCACACATTTGAGGGAAGA	60.892	20	234	253	486
59.622	23	GGCAACTTGGGAGTTGAAAA	60.088	20	275	353	627
59.434	20	AATGACACGCAAAAAGGGAC	59.978	20	101	173	273
59.75	20	AATCTGACGGCCGATATTTG	59.923	20	171	962	1132
59.966	20	TTGTCACACATTTACACACACG	58.016	22	220	137	356
59.667	23	TGGACTTAACTGGTAGCCCG	60.125	20	180	186	365
59.49	20	TGGATGTGTAGATGATTTTTGGA	59.329	23	169	43	211
59.627	20	AAGCGATCCTTACAGGCAGA	59.978	20	230	61	290
59.883	20	ACACACACGATGGATCGAAA	59.967	20	234	97	330
59.269	20	AGCATCTCTTGCTCTCTCATTG	58.842	22	161	1	161
59.973	20	CCATTTTTACATGCCGCAAT	60.7	20	203	805	1007
59.563	20	CCTCACCAACTCAACCCACT	60.002	20	212	26	237
59.101	20	TTCCTCCTTGCAACAAATCC	60.051	20	204	169	372
60.032	20	GGTGCTGTGTGATGTTGGAG	60.162	20	190	57	246
59.96	20	TTTCACCGTCCAAAACACAA	59.981	20	213	380	592
59.873	21	GCATGCAGAAGATGTGGAGA	59.95	20	215	1570	1784
59.967	20	ATGCAAGCTAACCGCTAGAA	57.835	20	266	52	317
60.157	20	CGTGTCAAAATTTGCTTGTCA	59.761	21	244	91	334
60.02	22	GGATGGGAGTACATTGGTGG	60.05	20	247	67	313
59.433	22	TTTGGGGGTGAGGTGTAAAG	59.824	20	215	522	736
59.595	21	GCGTTTTGATGATATATAGGTGTT	58.907	26	156	384	539

59.946	21	AGGAGCCTCACTGACCTTCA	59.986	20	136	342	477
60.23	20	CACTTAGCTCATTGATTTTCATCA(	59.24	25	153	602	754
59.888	20	AGAACTAGGGCACTCCATCG	59.308	20	278	78	355
59.926	20	CGAACCACCACACCAAATTC	61.196	20	154	487	640
60.059	26	CTCAACAGTCAACAAGCACACA	59.986	22	212	313	524
60.469	20	TTTAAGGGTTATCACCTGATTTTC	57.757	24	278	55	332
60.133	20	GAGGAAACCAGCTGTCCAAG	59.844	20	250	2029	2278
59.997	20	CATGCCTCAAACAACCTCAA	59.691	20	122	578	699
59.734	20	CCATTCACACACACACTCAGA	58.078	21	258	183	440
59.882	20	AATGGGGACACACACTCACA	59.846	20	126	355	480
59.931	20	GCCTCATCGTCGGATTTTAG	59.668	20	273	87	359
59.837	20	GTGGCTGCTGTGTGAGGTAA	59.905	20	222	171	392
59.224	23	ATTTCATTTCATGGGTTTCG	57.399	20	108	11	118
60.034	20	GACACAATGCACACCTCACC	60.013	20	272	1060	1331
59.614	20	TCGGTCGTCAGATTGAGAAA	59.369	20	222	2067	2288
59.67	20	CATCCAAATCAGGATCAGGTC	59.351	21	221	2405	2625
59.322	20	TGCATGTATTTGGGCTAAAGTG	60.02	22	150	18	167
60.241	20	TTTACCCGTTGCATGTGTGT	59.888	20	248	1416	1663
60.013	23	GAAGGAGACGATCGAACAGC	59.957	20	280	157	436
59.709	27	CAAATCCCATCAACCCAAAC	60.029	20	109	18	126
60.024	20	GGAGAAGAGATCGGAGACGA	59.486	20	190	36	225
59.982	20	CATTGCTGCTATGCCTGAAA	59.976	20	247	846	1092
59.916	20	GTGGCCTGAGATAGCCGTTA	60.235	20	202	9	210
60.019	20	CCCGTCTCTATCCCTCCTCT	59.653	20	246	2240	2485
60.049	20	GAGTGAGGAGCAGAGGGATG	59.945	20	205	476	680
59.523	21	TGCATTCAAAAGCCACATCT	59.276	20	228	418	645
60.142	20	GACCGCGAAGTTTACATGGT	60	20	238	53	290
59.973	20	TGTTTAGCAGTCCACCTCCC	60.111	20	253	3498	3750
60.386	20	TCCAATTAACGTTCCATTCACA	60.223	22	226	2381	2606
60.088	20	TTTCTACTCCCTTCCCCTGC	60.569	20	259	988	1246
60.096	20	GGTGGCTTCTCTCTTCGTTTT	59.878	21	221	10	230
59.93	22	TCTTCGACCCAACACCATATT	59.302	21	100	31	130
57.239	26	AAGGCGTTTTGTAAATTGGC	59.124	20	273	5	277
59.708	20	TCATCTTGTCAATTTCGCCA	60.197	20	188	204	391
59.761	21	AGCGGTTATTACCGGATCG	59.93	19	225	72	296
60.34	20	ACGGCTGGCATTTGTTAGAG	60.27	20	168	71	238
59.957	20	TGTTGTTGTTGTTGCTGCTG	59.504	20	222	99	320
60.081	20	TATGGGTTATCACCGGATCG	60.544	20	267	651	917
59.547	20	ATTAGGGTTCCTCCTTCCCC	60.497	20	112	3	114
58.302	27	CAGGATCTTTTGTTATCTTTGACG	59.224	24	174	4	177
59.962	20	AGCTTCAGTCACGCACACAC	60.104	20	250	96	345
60.016	20	TATGGGTTATCACCGGATCG	60.544	20	129	297	425
59.737	20	TTTCTTCCCATCATCACCGT	60.317	20	280	778	1057
60.166	20	AACACACACGCACTCACACA	59.818	20	147	262	408
60.015	20	TTGTCCAGTGTGTGTTTTTGG	59.495	21	224	59	282
60.128	20	GCCACATACTTTGGAGACGC	60.667	20	268	1242	1509
59.98	20	GATGGTTTCCGAAAGTGGAA	59.91	20	257	1689	1945
59.581	20	GGGCTCAGTAAACTGCTCTCA	59.63	21	243	95	337
60.886	20	CCCAAACATTTGAGGGTTGT	59.688	20	205	558	762
59.911	20	TTTTATGTAAATTGTCGTGCGT	57.373	22	130	190	319
59.755	20	GCTTGGTGAAGGAAGTGAGG	59.844	20	122	24	145
59.632	23	TCCTTTATTTTGATGTAGCAACCA	59.904	24	272	698	969
59.587	20	TGACCCTTTGATGTTATACACGA	59.393	23	209	534	742
59.993	20	CGAGAAATGAGAGCGTAGGG	59.971	20	260	86	345
59.853	20	TGGTGACTTGAAGAAATTTGGA	59.587	22	159	50	208
58.88	21	GTGGGTTTAAGTGGGGTGTG	60.126	20	104	77	180
60.317	20	CGACCCAGATTCAAACCCTA	59.926	20	275	73	347
60.434	20	AATGATTCCTTGGCACTTGG	59.933	20	160	490	649

59.962	20	GGATGCTGATGGAGGGAATA	59.854	20	246	446	691
60.008	20	GGACCAACTTGGAAGTACCG	59.449	20	103	23	125
59.74	20	ATAATGGTGGCAGCTGTGCT	60.688	20	201	484	684
57.155	20	TCCTTTTGGCGTAGTGAGGT	59.734	20	166	11	176
59.867	21	ATTGAATCTACGTCCACGGG	59.813	20	255	70	324
58.5	20	CGCTCGGGCTCATATCTATC	59.791	20	231	37	267
60.183	20	CGCTCCACAAAGATCTCACA	59.984	20	114	181	294
59.67	20	TGCATGCAAAACTGAGCTTC	60.142	20	174	375	548
59.758	22	AAGTGTTTTCCCACAGACGG	60.005	20	177	40	216
59.847	20	CTCTGCATGTACGCTACCTACC	58.954	22	219	42	260
60.285	20	TCGTGTAAGTTAGCCGGGAC	60.132	20	267	0	266
59.997	20	AATTTCTGTCCCGTTTTCCC	60.166	20	280	48	327
60.029	20	AAGGAATGGTTTGATGGGTG	59.647	20	216	145	360
60.088	20	TAAGTTGCTCGTTGGCATTG	59.872	20	189	3313	3501
59.95	20	AAATTGACAGCGATGGAAGC	60.221	20	171	109	279
58.161	20	AGGGGTTATCACCTGAATGC	58.864	20	156	484	639
59.73	20	CTGGAAAAACTTCCCACCAA	59.942	20	177	5835	6011
59.73	20	CGAGCGAGTGGTTGTGTAAG	59.51	20	100	12	111
59.823	20	GCTCATGGTTCAGGGTTCAT	59.934	20	242	223	464
60.033	20	ATCCGCCTACATTGGATGAC	59.78	20	188	1448	1635
60.118	20	GCCGGAATCGAGAATATGAG	59.628	20	202	390	591
60.066	20	GCGAGGTAGGCTTTCGAACT	60.894	20	249	71	319
59.809	20	CCTCTGCCTCGTCTGGTTAG	60.005	20	230	29	258
58.809	25	CTCCTAAGTTGCCCCATTGA	60.066	20	112	24	135
59.822	20	TGCATTCACCTTCTCTTTGC	59.002	20	200	157	356
60.456	20	TGGTGTAATAATTTGGTTTCATCG	60.006	24	254	245	498
59.296	25	GTCTTCCGCGTGCGTATAAT	60.124	20	222	23	244
59.997	20	GTTTACAACTCACAACGCGG	59.245	20	145	170	314
59.933	20	CCATATTTAAGGGTTATCACATGA	59.713	27	124	960	1083
59.988	20	TTCCTCCCTCGTTTTTCGAT	60.929	20	256	3303	3558
60.006	20	TTTGTCTTCCCGTTTGTTCC	59.948	20	278	146	423
59.751	20	ATAACCCGGTCAACAAGCTG	59.993	20	249	25	273
59.844	20	ACCAAAGACGGGCTAAAATG	59.087	20	214	399	612
59.988	20	TGTTGCAGTTGTGGGTGATT	60.008	20	168	366	533
57.299	25	CATCAACTCCATCCCCATTC	60.135	20	276	14	289
59.52	20	CGTAGGACAACGACGGTTTT	60.03	20	109	21	129
60.025	20	TGCGTAAGTTTTGGTGCAAG	59.911	20	260	168	427
60.011	20	CTTGATTGGTTGACGTCCCT	59.966	20	232	242	473
60.706	19	GAGCTGCGGTTTTGATCTGT	60.406	20	265	198	462
60.009	20	TGCTGTGGGGAGTAAACACA	60.152	20	276	2	277
60.612	20	TGCCTCACCACCAATGTAAA	59.964	20	217	537	753
57.229	25	CAGGCCCAATACCTTGACAT	59.813	20	134	21	154
59.734	20	CCTCCGAACTCATGGTGTTA	58.569	20	245	206	450
60.7	20	GATCATCCCGTGGTTTGTTT	59.653	20	268	29	296
59.982	20	GTCAACTTGGGCAGTGGAAT	59.973	20	249	102	350
59.454	22	TGCAAGCATCAAACAACACA	59.877	20	179	3	181
59.317	20	CGGGAAGCCTTTATCTATGC	58.809	20	222	613	834
58.899	22	TGACTAAGGTCCCATCACGA	59.086	20	234	51	284
57.557	25	TCTTATCACATCAACCGCCA	60.073	20	127	20	146
59.881	20	AAATCTACCTCTGCCACCCC	60.328	20	254	181	434
60.04	20	GTCACGAGGTTGAATTGTGC	59.143	20	177	1551	1727
59.882	20	CTTTTGGATGACGGAAGGAA	60.044	20	150	121	270
59.429	20	TTGGATGTTTCTGGAGCGAT	60.603	20	280	153	432
59.658	24	CATCGTGAATGTTGGGAGTG	59.96	20	172	3	174
59.799	20	TGCTGCTTCTCTTGCTGAAA	60.011	20	158	416	573
60.125	20	TACATGGGACGATTCCGATT	60.155	20	154	2864	3017
59.853	20	CCAGCTCAGTGGTGGAAAAT	60.111	20	204	100	303
59.304	20	GTGGAGAGGAGGGATGATGA	60.008	20	116	171	286

59.537	19	ACCTGGATCACGGGTTTAAT	58.24	20	280	2129	2408
59.779	20	GGGTGAAAAATGATTCAGAGGA	60.299	22	131	68	198
60.027	20	CATGATGCGAGGATCCTTTT	60.036	20	182	164	345
60.173	20	TGCGTTTTGACAATATATAGGTGT	60.083	26	251	50	300
58.93	20	CACCACTCTTCGATGGAACC	60.51	20	247	83	329
59.564	20	AGCGGTTATTACCGGATCGT	60.711	20	231	146	376
59.465	25	ATTTCTTACCCGTCCGTCG	59.94	19	214	21	234
59.505	23	TTGCAATATTCAACTACGAGCA	58.52	22	104	27	130
59.784	20	GTGGCGTTATTGGGTTCATT	59.691	20	268	4	271
61.21	20	TCAGCCTAGTCGCGATTTTT	59.982	20	276	195	470
60.074	20	GGGATGGGAGAAGATGTTGA	59.862	20	166	507	672
59.569	20	AGCCACTCCCTCCAAAAGAT	60.074	20	203	12	214
60.011	19	TATGCACGAAGCCAATTCAC	59.694	20	257	1987	2243
60.181	20	TCGTCCCATCTTTTACCCTC	58.981	20	256	337	592
58.608	20	TTTGAACTCTCGGCCTCTTG	60.508	20	249	79	327
60.184	20	CGGGCGTTTTTGTAGGTAAA	59.993	20	247	2252	2498
58.121	21	AATAAAATGTACGGGCGCTG	59.985	20	158	23	180
60.014	20	CGAGTGTGAGGCTGTGAGAG	59.757	20	127	64	190
60.065	20	AGTCACCACCGAACCAGAAC	60.009	20	249	21	269
59.934	20	TCTTACGGAATTCCACTGGG	59.926	20	129	177	305
59.572	22	AATCCTCAAAACCTTGCGTT	58.695	20	249	70	318
60.045	22	ATAAGGGCCTGGTCATTCCT	59.789	20	266	1982	2247
60.435	20	ACAAATTTGCCCTCGTATGC	59.967	20	125	126	250
59.053	25	CACTTGAGGAAGGATGATGAAG	58.789	22	100	425	524
59.984	20	CTTTTTCGACTTTGATCCGC	59.823	20	242	9	250
58.079	23	TAGGTGAGGTCGGGTCACTC	60.112	20	195	31	225
60.261	20	TCGTCCGAAAATCCTTTCAC	60.051	20	246	272	517
60.088	21	ATCGGTTATTACCGGATCGC	61.031	20	259	291	549
59.358	26	AAATGTTTGAAATATCGGGGC	60.035	21	100	10	109
59.997	20	TGGCACAATAGGAAAGCAAG	58.917	20	105	480	584
59.67	20	ACAAACTTATGGACCGCTGG	59.993	20	103	587	689
60.141	21	ACGTCACCCCACTTGGTAAA	60.269	20	199	1708	1906
59.083	20	AGTTGAGGTTACGCCAAACA	58.283	20	238	64	301
58.861	22	TATAACCGATCCCACCCCTT	60.391	20	126	94	219
60.11	20	AGTAATTGTCCAGGGGGAGG	60.183	20	139	386	524
57.85	20	ATATGAAGTGGGGATGGACG	59.629	20	258	1135	1392
59.556	20	CATCCACTTAAAGCAAGGCA	58.917	20	280	733	1012
60.128	19	CGACATTGCCCTATGAGAAAA	60.081	21	115	35	149
60.155	21	AAATTAAAATTATGGCAACTTTCC	57.89	25	258	3636	3893
60	20	CCATGGATGTCAGTCATTGC	59.925	20	264	1513	1776
59.907	22	CCGTCGATTTCTAGTGTTTGG	59.618	21	190	393	582
59.438	21	AGTTTTTCCGACTCAAGGCA	59.853	20	251	186	436
59.762	20	TCTTGCACATCTTGGTGAGTG	59.889	21	229	18	246
57.767	25	ACCTGTAGAATCTCTTACTCTATC	57.215	27	223	593	815
60.987	20	GATGGAGACACCTAAGCCCA	60.073	20	172	5	176
60.08	20	ATACGAGTAGCGATCGGCAT	59.719	20	263	24	286
60.052	20	GGCATCACAAAAAGCAGACA	59.847	20	271	64	334
60.081	20	TGAACCTTGAATCGAGTTTACAT	57.814	23	187	8	194
60.103	20	TTGTAGGCAACGAGCCTTCT	60.015	20	232	3460	3691
60.423	20	TTATGCATGCACTCCACCAC	60.549	20	170	320	489
60.155	20	CCACTGTTGGAATCACATGC	59.967	20	263	371	633
58.933	20	TTCATCAGGTCGTGGATTGA	60.048	20	122	445	566
57.617	23	GCCTCGGTTGCTTATTTGTT	59.229	20	279	378	656
59.988	20	GGTGGTTTGAGGGTGAATGT	59.679	20	172	44	215
59.903	20	TTGTCTCACTCACCGTGCTC	60.03	20	277	4176	4452
59.799	20	TCCCGTTTTACCCTTGAATG	59.795	20	196	89	284
60.791	21	CCTCACTCTTTTACGCCTTCC	60.248	21	102	16	117
59.708	20	CCTGTTCTCCGCTGTATTCC	59.694	20	149	2772	2920

59.978	20	CATGCAAACATGGAACATTG	58.389	20	268	297	564
60.015	20	TTGATCAAGACCTTTTGGGG	59.903	20	226	82	307
60.379	23	GCTGCTCCTTTGACTCCAAC	59.997	20	146	73	218
60.143	20	GATGTTCCACAAAACCACCC	60.073	20	175	538	712
60.153	20	GACCGCGAACTTAGAGGAGTT	59.899	21	266	110	375
59.939	20	ACCCTCCTTCAGCCAAAAAT	59.94	20	252	588	839
60.439	20	GATGATTGCACCATAACGCA	60.498	20	148	479	626
60.006	20	TGTTGTCATAACAAAAATGACACC	59.837	24	100	352	451
59.645	20	TCGCCATATTCGATCACAAA	60.035	20	235	180	414
59.238	22	GTTGTGCGCATGATTGAAAG	60.265	20	130	1	130
60.284	20	GGTGTTGGCCGAAAGTTTTA	59.975	20	103	71	173
58.178	25	TCGCATGTTGATTTTCCAAC	59.523	20	278	853	1130
59.848	20	TCGGCATGACGTAGTTTGAA	60.257	20	222	1767	1988
60.066	20	GGCCAAATATTTCCCTACCG	60.508	20	212	92	303
59.978	20	ACCATATTTAAGGGTTATCACCTG	57.552	24	274	304	577
59.954	20	CCCGTGACACAACAGATCAC	60.005	20	258	578	835
58.4	21	AAGTCCCCATTAAAGATGTAAGTT	58.935	26	280	229	508
60.807	20	TCATAAGTTAACATAAACCAAACT	59.079	27	109	79	187
59.969	20	GGATTGCTGAAAACTGGCTC	59.82	20	276	32	307
58.932	20	AATACGACGGCGAAGAAAGA	59.845	20	239	25	263
59.682	25	TCGGGTTCCAAATCTCGTAG	60.066	20	228	38	265
59.95	20	AAGGCCACACTCCAAGAAAA	59.711	20	176	673	848
59.639	20	CATCCCAGTTGTCTTGGGAG	60.501	20	181	18	198
59.677	20	ATGTCACACCCTCCCCAATA	60.05	20	171	233	403
60.483	20	ATCATGTAGTCGCGTGCTGA	60.445	20	161	829	989
59.195	20	TTTTCGAGTTTCTTGTTCTCCA	58.994	22	246	321	566
59.925	20	CGAGAGCTGAGAGACAAAGAGA	59.112	22	277	535	811
60.053	20	ACCCTCCTTCAGCCAAAAAT	59.94	20	244	1284	1527
59.956	21	CACTTTTAACTCCAAACCAGGA	58.288	22	220	105	324
59.799	20	CTTTCGTTGGCGATTTTCAT	60.074	20	236	40	275
60.255	20	GGGGAAGGAAAGAGAACGAG	60.183	20	221	37	257
60.089	20	AACGGTTATCACTGGATCGC	59.962	20	204	424	627
59.734	20	TCTCCCTTTCCTCACTCCTG	59.375	20	258	2	259
60.733	20	ATTTGCTTAAAATGAATCAATGG	57.325	23	191	275	465
60.908	21	TTTGTTTTCATTAACAAGACCATCA	60.047	26	217	1070	1286
60.066	20	TCCGTCATCGACCACTATCA	60.072	20	278	477	754
60.045	20	CGGCCGGTTTTTAATGTTTA	59.835	20	210	2187	2396
60.101	24	TGAGTGGAACGATCCATATAAATT	60.427	25	258	680	937
59.55	20	CCCACACTCACACGATTTCA	60.57	20	174	1163	1336
59.966	20	GCAGACATGTTTCGCTACCA	59.871	20	183	202	384
59.267	22	AATGCGCCTCCCACTTTAAT	60.821	20	120	4	123
60.263	20	TGCATTCGTCAGTAGTCCGA	60.413	20	178	480	657
59.033	21	CTACAAGATCAACCGCCTGC	60.796	20	201	104	304
59.346	20	CCCGTACCTCAATTAAAAGGG	59.719	21	263	716	978
59.801	20	CCGAATTCCTCTACCGTCAA	60.066	20	192	393	584
59.934	20	TGCACACTGTCACACTCCAA	59.905	20	201	2043	2243
59.75	20	CCAGGCTAGCCAGAACAATC	59.836	20	177	202	378
60.032	20	CACCCTCACATCTCTTGCATT	60.126	21	115	228	342
60.797	18	ATAAGAGGTATTTTGACAGTATCG	57.206	25	122	91	212
59.836	20	ATCGATGTGTTGGCATTCAA	59.931	20	250	225	474
59.816	20	CCACCCGTGTATGTCATGTG	60.722	20	226	397	622
59.778	20	CGAAATTGACGGAAGAGGAA	60.184	20	242	103	344
60.328	20	GCCGGATTTAGAGCAATCAA	60.175	20	212	184	395
59.339	20	GAACTTCGCCATCGTCTCTC	59.957	20	188	13	200
59.308	20	TGGATGAAAATCATTGTTCGC	60.832	21	275	583	857
60.111	20	TGGACTTCTCCAATCCATGC	61.016	20	239	234	472
60.452	20	TGCAATTCCTTCTGAACCCT	59.67	20	232	108	339
59.882	20	CTCGTTCTCTTCTTCATCTAAACA	57.05	24	123	281	403

61.225	20	CAGGCTAAAGATCGTCCGAG	59.971	20	249	213	461
60.39	20	TCATGGCTGGGTCTACATGA	60.072	20	177	3247	3423
57.497	26	CAACACTCAATTATAGGAAAATCA	59.318	27	250	210	459
58.133	20	GGGTAGTTGAAATGCAAGCC	59.574	20	168	416	583
59.523	20	GGGGAAGAGAATCAAGAGGG	60.008	20	103	369	471
60.508	20	AACACTGATTCGCACAAGGA	59.293	20	229	61	289
60.038	20	CATATTCCATGGTCCCCAAC	59.871	20	109	4452	4560
59.12	20	TCTTCACCTCAGTCTGCACG	60.178	20	215	1120	1334
60.038	20	TGCATCACATTTGTTCATATCG	59.448	22	195	88	282
60.309	20	ATCTCCATCTCCTTCCTCCG	60.553	20	276	947	1222
60.142	20	TGCCCTTGTATGCTCCCTAT	59.551	20	280	3139	3418
58.616	21	TGGGTTTTTACGAAAGGCAG	60.103	20	207	25	231
59.938	20	CGTTCATTTCGTACCTTTAGAAGTT	60.307	26	131	103	233
59.505	20	TGCTCGTTTCATATGTTGCC	59.694	20	139	147	285
60.331	20	GGAAGGCGTTGACATTTGAT	59.939	20	259	93	351
61.799	20	AATGTTGTCCCGGACTCCTC	61.291	20	100	59	158
61.248	20	TGGTAGTGAAATAAACATAAACTC	59.645	26	237	0	236
58.165	23	GGTTTTACGAACCACAATCCA	59.716	21	130	151	280
59.989	20	TACATTTCCCCAACCACTCC	59.647	20	173	12	184
60.423	20	CGTAAAAATGGTTATTATTGGATC	59.202	26	143	98	240
59.955	20	AATGGAGGCGATTTTGTGTC	59.939	20	246	1247	1492
60.549	20	TGCTTTGATTAATCCCTCCG	60.031	20	277	166	442
60.048	20	ATGGCATATGAGGCTTGGTC	59.923	20	175	181	355
61.11	20	GCCTATGCAGTCACAAATGC	59.306	20	266	734	999
60.648	20	ATCGCAATTCGATTAGGCAC	60.067	20	274	103	376
59.985	22	TGTTGCGTAATAGGCGAGAA	59.467	20	209	330	538
59.998	20	ACGATTACAACGCAACATCG	59.614	20	137	346	482
59.317	23	CCATTCAATTTAACTTCGGCA	59.95	21	217	97	313
60.028	20	GTTGGCAACCAGATATGCCT	59.962	20	127	304	430
60.14	20	TCCAATGGATGGAAATGGTT	59.991	20	279	288	566
59.439	20	GGCTAAAAGTTGTTGTCTGGCT	59.825	22	184	9	192
59.894	20	ATGTTGCAGCATAGGCATGA	60.251	20	279	608	886
60.037	20	CAATGCCGCTGAACATATTG	60.096	20	171	684	854
60.376	23	TTCTGTCCCGTCTTCTTCGT	59.844	20	170	13	182
60.723	20	GGACCCTAATACCACTAACCCC	59.847	22	102	513	614
59.955	20	CCTTCAACAAATGGTTCATGC	60.359	21	187	1721	1907
60.042	20	TTTCCAGGATCGAATTGAGAA	59.631	21	262	235	496
60.03	20	TTCAAACCTGGTTCTCGTCC	60.088	20	243	70	312
60.807	21	CAACATCACCAAACACTCCG	60.001	20	225	8	232
60.894	20	TTCATCATTTCATTTTATAAGCTTC	59.91	26	189	107	295
59.943	20	TGGTTGGTGGAAGAAATCAA	58.947	20	215	59	273
59.182	20	CAATGTTTCCATGTTCCGTG	59.816	20	262	1324	1585
59.979	20	ACAGAAATTGCCTTGGTTGC	60.118	20	134	249	382
59.049	20	GTCCGATTTGCCGTTCTATC	59.533	20	215	1028	1242
59.993	20	TCCAGCATTTGTATGTCCCA	59.924	20	213	1442	1654
60.294	20	CATGAACCTGAAACAGCGTG	60.301	20	229	2155	2383
59.983	20	ACAAGAGCTGAAATGCCCTT	58.936	20	215	30	244
60.005	20	TTGAATCATTTGCGTTGCAT	60.081	20	268	4312	4579
59.743	22	GCCAAAAAGTCAAGGCGTTA	60.244	20	262	3	264
61.061	20	GATGTCGAGATCCAACCCAT	59.744	20	230	364	593
59.762	20	TTAGGTGTTTTGACATGTTCACTC	58.225	24	165	236	400
59.534	20	CCCACTTCCTCCAACCAGTA	59.959	20	244	536	779
57.509	23	TGTTGTAATGAGGCTTTTGGAA	59.625	22	237	15	251
59.271	22	AAATATCAGGCCAAAAACGC	59.081	20	191	242	432
59.95	20	TTCCCCATCACCATCACTTT	60.173	20	166	111	276
60.074	20	CCGCCTGATTTGTTTCTCAT	60.074	20	127	159	285
59.665	20	TCCGATGACTAAATGGAAGACA	59.569	22	141	5522	5662
59.864	20	TTTCGTTTGCCTAAAATGCC	60.075	20	233	218	450

59.706	20	TACACAATGCCAAACATGGG	60.234	20	265	5399	5663
59.989	20	CAGTGGTCCTGGGAACCTAA	59.959	20	152	6	157
59.578	21	GTAATGGCGGTGAAGATGGT	59.82	20	276	100	375
59.926	20	CCTTGGCGGTTCATATCAGT	59.955	20	171	65	235
60.123	21	GAGAAGTTGTCGGTTTGGGA	60.088	20	213	639	851
60.147	20	GGACCTTGTCCAGAAACCAA	59.943	20	148	87	234
59.747	20	CATACTCTCACCAATCCCCG	60.331	20	141	4	144
59.978	20	TCCCAGCATTCTTGAACACA	60.24	20	138	71	208
60.162	20	TCATGTTGCTACACGTTGCT	57.926	20	149	175	323
59.274	21	TCGGGTAGTCAAGTTTGCTTT	58.893	21	255	20	274
59.864	20	TGTACAAAAGCTTGTGGTGAAGA	59.836	23	240	36	275
59.853	20	GCAACGGAATACCACTCGTT	60	20	167	1466	1632
59.481	20	GCACACTCGAAGAGTAGCGA	59.344	20	216	776	991
59.267	22	AAAATACCAAAGTCGATTATCCA	59.264	25	162	39	200
58.161	21	TATGGGTTATCACCGGATCG	60.544	20	119	104	222
60.152	20	AAAAAGCATTTCACACGTTCTG	59.312	22	179	294	472
59.727	21	CGCCACAATTACCAAAATGA	59.42	20	280	844	1123
59.844	20	CACACGTTATATTTATAGTTTGTG	59.19	27	124	223	346
59.955	20	GCGAATTCAGCTAGAACAACAA	59.541	22	180	1836	2015
60.211	21	AGCTTATCACTATCACCCTCTTCC	59.22	24	100	28	127
60.192	20	ACGGTTCACATACTCCTGCC	59.997	20	235	150	384
59.208	22	TGCAACCAACAAAAACAACA	58.626	20	190	25	214
59.969	20	TCCTTGGAACCTAGAAATGGTT	59.001	22	256	9	264
59.938	20	GGGAGAATGCCTTGTTTCAA	60.051	20	145	309	453
60.183	20	ATCCTTTTCGCCATCTTGAA	59.645	20	190	127	316
58.423	21	ATGGAATTGGGTGTTGGATG	60.439	20	115	132	246
57.315	21	AAGGAGCTCAACCTACGTGAA	58.994	21	271	201	471
59.824	20	AGGTTGCAAGGACAAGCCTA	59.875	20	274	700	973
60.175	21	TTGATTTCCCACCCTTCTTC	58.956	20	213	53	265
58.695	20	TGCCTCTAATGCACACAAGAA	59.478	21	279	67	345
60.344	20	GAGCCAGGATTTCACGGTAG	59.694	20	267	1022	1288
61.196	20	CAAGGGTGATTGTAGCCAGC	60.657	20	158	890	1047
59.206	24	CTCGCTAACGGCACTAAACC	59.904	20	106	148	253
59.967	20	GGTATTGTCGGAACTTCGCT	59.198	20	163	101	263
60.326	20	TGCAGAAAGCAGGCAGTAGA	59.888	20	190	4388	4577
60.255	20	AGACGATTGAGAGAAAGCGG	59.574	20	106	503	608
59.938	20	AAATTTATTGTAATCTTCCCTCCA	59.594	26	233	559	791
60.189	23	CACCAACCCAACCCACTAAC	60.126	20	120	11	130
58.984	20	ATCAATGGTCCACATCGGTT	60.057	20	217	25	241
59.708	20	TTCCTCTTGTCGCCATTTTT	59.685	20	280	319	598
59.964	20	GAACAATTACAATGTTTATGAAAT	59.51	27	105	25	129
60.286	20	TGGTTGTGTGAACCAACTCTG	59.626	21	141	272	412
60.044	20	GGCCATTAAAGCTTCACCAA	60.074	20	220	49	268
59.917	20	GTTTGAGGTCGGAGATTGGA	60.05	20	137	248	384
60.144	20	CAAGCTCATTTTGTGTCCCA	59.691	20	243	1056	1298
59.923	20	ATCGATGTGTTGGCATTCAA	59.931	20	145	79	223
59.945	20	AATGAAAGCGATCGTATGCC	60.067	20	190	887	1076
57.436	27	AAGGAGCAGGTGTGGTTGTC	60.159	20	240	18	257
59.784	20	TATTCACACAGCCCTTTCCC	59.933	20	211	107	317
60.081	20	AATTTGGAGAGGGAGGGAGA	60.008	20	102	27	128
60.195	20	AAGGGATTCACCTGCCTTCT	60.074	20	161	1158	1318
59.42	20	TTGATCCTGCAATCTCTCGTT	59.828	21	187	9	195
60.185	25	GACTCTTCACAACTTCTACACAAT	57.679	26	275	822	1096
59.933	20	GACACCCAATCCCATGAAAC	60.034	20	203	5	207
59.651	20	ATTTGCGTCGCCTAATGAAC	60.103	20	132	182	313
59.921	21	TGCTGAGAGTGTGTTGGCAT	60.473	20	108	1218	1325
59.964	20	CCCTCAATCGATCCATGTCT	59.886	20	177	498	674
60.312	20	GATCCCGTGTGTGAACTGTG	60.005	20	141	56	196

57.251	27 TTTGGTTCAATGGGTTATTCG	59.685	21	190	87	276
59.097	21 GCATGATCAGGGTAAAATCCA	59.776	21	191	46	236
60.188	20 ACAGCCACCATAATCGAAGG	59.955	20	262	285	546
60.218	20 GAACGGGCAGCAGTCTAAAG	60.015	20	157	58	214
59.6	21 CCTTCAGCATTTGAGATCGG	60.734	20	102	6	107
60.235	20 TGCTCACCTTCGATCTCTGA	59.656	20	214	47	260
59.888	20 GGTGGGAGGGAATTTGAGAT	60.133	20	235	1961	2195
59.971	20 TGTGTTTTATCCCTCGCACA	60.111	20	210	37	246
59.948	20 AGAACATGGTTTCCGAGCTG	60.255	20	101	163	263
59.988	20 TCTCCCCCTCTTCCCTCTTA	60.139	20	260	4096	4355
59.865	23 CACCTCACCTTCTCTCCACTCT	59.91	22	149	92	240
59.951	20 AGGCTAAACAAGGCCCAAAC	60.483	20	276	91	366
60.218	20 CAGAAATTGCCTCTGTTGTTC	57.468	21	170	111	280
60.008	20 TAATTTCGGCCAACACCTTC	59.938	20	180	1380	1559
59.027	21 TGCCTCAAGGCTAAACAAGG	60.378	20	259	87	345
60.088	20 CCTCTCGTGTTTCTTCGTCC	59.844	20	122	38	159
60.144	20 TACACCGAAGGATATCCCCA	60.148	20	266	285	550
59.697	20 AACAAGTGTGTTTGTGAGTGGG	59.969	22	146	385	530
60.068	20 CCCTCACCCCTTATGGTTTT	60.046	20	255	112	366
60.263	23 AACTCTGCCGGAGAATGTGT	59.727	20	232	1259	1490
59.472	25 TTCTGGATTATCCTGCGGTC	60.036	20	219	200	418
57.335	20 GGTTAAATTTTGTCCCTAGTTAGCA	59.109	25	103	141	243
60.074	20 TTTCTTGGCGTGGTGTTGTA	60.149	20	190	442	631
59.988	20 GAAGACACGGTTGGACAAAAA	60.001	21	264	233	496
59.78	20 TGTAGTGGTTCGTGGACTGC	59.751	20	219	32	250
59.928	20 AGCTTCCATGGAGGTTGATG	60.073	20	184	5	188
60.155	20 ATGGGCTCAAATATCGGACA	60.296	20	258	18	275
60.119	20 TATGAAGGCTAGGGGTGTGC	60.096	20	231	276	506
60.038	20 TCGTGGAGAAACTATCCAAGG	59.181	21	244	762	1005
58.987	22 CCCCTACGTTGAAATTGTCG	60.357	20	232	368	599
59.938	20 TTCGTTGATTTGAAACTCTAAAGC	59.028	24	159	341	499
59.864	20 TATGTCGCATTTGACATCGG	60.488	20	232	78	309
59.446	20 TACCTCATCACCATCCCCAT	60.011	20	150	40	189
60.073	20 TCCCGTTGTTGCTTTCTCTC	60.375	20	271	137	407
60.044	20 TAGGAAAATCATGCACGTCG	59.688	20	148	1360	1507
60.656	20 AGGGGTTATCACCTGAATGC	58.864	20	248	268	515
58.371	20 GTGGGCATAAGAGACGGAGA	60.218	20	153	2138	2290
59.945	20 GCGGAGATAGAGACAAGGCA	60.504	20	178	203	380
60.068	20 GAGCTGCTGTTGGAGGAAAC	59.997	20	279	69	347
58.697	20 GAGTTCTTATCGCCTCCGGT	60.599	20	255	9	263
59.91	20 TGGATTTGGGATTCTTCTGC	60.014	20	271	1807	2077
59.569	20 CATTTCCCCCAACTTCACAT	59.647	20	207	21	227
59.839	20 ACTTTCTCGCCTGGATTTTG	59.312	20	136	143	278
59.993	20 TAGAACATTAGCGGGATGGG	59.916	20	137	187	323
60.212	20 GGATGAATTGATGGTTTTTGG	59.125	21	169	145	313
59.671	21 TGGAATTTGAAAGTTTGGAACA	59.469	22	272	713	984
58.576	23 GGAGATTGAGCATCAGGAGC	59.917	20	226	32	257
60.059	20 GAGAGGGAAAAGTTGGGAGG	60.045	20	237	272	508
59.747	20 ATGATTATTTGCCTCGTCCG	59.923	20	259	1669	1927
59.945	20 GTGGAAGAGAAGATGCTCGG	59.95	20	164	47	210
60.067	20 CAGAGCGCTAGAGGTTTTCC	59.218	20	233	447	679
60.015	20 GTAAAACCCGACACCTGCAA	60.924	20	240	92	331
59.961	27 GGGAAAGATTATTGGAACCTTG	58.864	22	215	1	215
59.996	20 ATCGAAAATGCCAAAAATCG	59.907	20	241	3140	3380
60.027	20 TCTTGCAATGAAGAACGTCG	59.988	20	150	63	212
58.866	20 GCAAGGCCAATGGAAATATG	60.289	20	160	1711	1870
59.95	20 TCATCTTCAACCCCCAACTC	59.903	20	248	1539	1786
60.214	20 TGCTGGTGTTGAATGGAAAA	60.088	20	211	287	497

60.206	20	AAACGCTCGTAGTGTCCCAG	60.312	20	124	208	331
58.017	20	TGCGTGGTTCAGATTGAGTT	59.293	20	280	1131	1410
59.844	20	TCCTCATGTACGTTGTTTATGGA	59.393	23	221	7	227
60.187	20	TGATTGAGGGTGATTGGTTAGA	59.429	22	100	29	128
60.212	20	CCGCCAGCTTTTATTCAAAC	59.72	20	271	1	271
60.655	23	GATGCACTGCACAATCAACC	60.128	20	219	216	434
60.134	20	AACTCCCCCACGAACTCC	59.896	18	101	41	141
60.111	20	TTTTGATGATATATAGGTGTTTTGA	58.782	27	136	2035	2170
59.983	20	CGACTTGTCGTGGTTCGATA	59.716	20	242	233	474
59.844	20	TCCTCTCTCTTTCACCACCG	60.377	20	201	1	201
59.922	20	ATAGTGGTGGGAGGAGGCTT	59.957	20	229	34	262
60.138	21	GATGAGGGATGCCATAAGGA	59.854	20	240	667	906
60.833	20	GGGATGGCTTGTTCACTCTC	59.661	20	215	18	232
59.997	20	TTTCAGCCGTTTGGTTTTGT	60.514	20	274	312	585
58.922	24	CGTTTCCGACTACCCTTGAA	60.103	20	259	4	262
60.081	20	AGCACTGCAGTCTCTGGCTT	60.352	20	174	393	566
59.993	20	TTATCCCAGCTCCGCTAAAA	59.81	20	161	1887	2047
59.992	20	TTTGCGTGAACAATGGTGTA	58.601	20	272	178	449
60.025	20	AATGCATGTCTCCGAGAAAAA	59.699	21	225	1122	1346
59.891	25	TCTGTTGGCAAACACAGGTC	59.726	20	108	63	170
59.565	21	CACCCACACACTCACACACA	60.088	20	126	17	142
59.993	20	CGGTACGAGCACAAACACAC	60.222	20	183	419	601
60	20	CGTGTTTATCCTCACCCGTT	59.853	20	166	70	235
60.096	20	TGGCAGAAGTGGGAGTCTTT	59.844	20	137	74	210
60.142	20	TGTGAAAATTTGGTTGCAGAA	59.192	21	194	191	384
59.801	20	AAAATCAAAGGCCCACAATG	59.801	20	216	106	321
60.192	20	GGGATGGGAGAATTGGAAAT	59.959	20	201	375	575
59.973	21	CCAACTATCCATGAGAAATGAGG	59.843	23	100	3	102
59.91	20	GGAATCATCACCATGCAACA	60.339	20	224	53	276
59.982	20	AAACCTTCGGCTTGAAAACA	59.724	20	277	60	336
60.35	23	CCAATCTCAACAACACACGC	60.16	20	170	55	224
60.029	20	GGACGCCACAAATTGTTTCT	59.978	20	192	103	294
59.009	20	CCATTCAAATAAAAATCAAGGGA	59.243	23	279	309	587
60.499	20	ATAACCCACCTCCCCAAAAG	60.046	20	125	43	167
59.623	21	AGTCGCTGCAATTTCCAGTT	59.882	20	215	3	217
57.862	23	GCAGGCAGCTGATATGTTCA	59.979	20	200	13	212
59.43	20	CCCTAGCCTTTGCCTCTTTC	60.332	20	234	35	268
60.054	20	TGATATAGTTTTTCGTGGCATCA	59.507	23	215	53	267
59.819	22	ATGATCGACCAACCCTGTTC	59.786	20	155	25	179
59.788	24	CCATTGAGACTCGAACCCAT	59.927	20	143	839	981
60.617	20	GCGACGTTACCCACTTTCAC	60.559	20	129	82	210
59.805	20	TGGCAACAAGATACCCACAA	59.964	20	124	571	694
59.989	20	TTTCAATGGGAATCGGAAAC	59.739	20	203	1191	1393
60.014	20	CAGTTGAACCAAACACCCCT	59.861	20	280	278	557
59.853	20	TGCGGGGATATATGTGGTTT	60.038	20	275	403	677
61.072	20	CAACGCCATGCATTTATCCT	60.857	20	153	16	168
59.942	20	TCTGTTGCTCCAAATCATTCA	59.262	21	221	1496	1716
60.096	20	CTAACCTTGGGCTCTGATGC	59.836	20	136	76	211
58.944	24	TGTCGCGTTGCAGATCTAGT	59.621	20	209	43	251
60.134	20	GGGATGGGAAGAGCATTTAAG	59.917	21	274	1052	1325
59.797	20	GCTTGGGCGAAACATGTAGT	60.14	20	280	132	411
59.097	20	GCTCGCATAACACCCCTTTA	60.096	20	225	684	908
60.012	20	CTGGTTTGGAGAAAGCGAAG	59.986	20	269	340	608
59.434	20	CGGTATCACCCTCCTTTTCA	59.926	20	127	88	214
59.569	20	CTCTATTTGGAGTTGGGGCA	60.066	20	229	3663	3891
60.067	20	TCTCTTTCTCACACCCTTTTCA	58.955	22	212	355	566
59.691	20	CCGACATGGGGAAATTGTAG	60.184	20	246	339	584
60.088	20	GGTTCGTTTTCGTCGTGATT	59.978	20	114	483	596

60.277	20	CAGCAATTTCGATTTCAACG	59.301	20	180	77	256
60.444	20	TAAAGGAGCCCAGAAAAGCA	59.953	20	186	2075	2260
59.664	20	CTGGTCCCCCTTTCTCTCTC	60.186	20	217	59	275
59.859	20	CATGAATCCAACGTACGCAT	59.425	20	228	1428	1655
60.545	20	CCTTATGCGGTTCTTGGAAA	60.067	20	258	0	257
59.721	20	TCGGAGTGTCTCTGTGCATC	59.988	20	256	795	1050
60.067	20	TGAAGTTAGGAAATAAGGAGGTG	58.743	25	105	659	763
60.212	20	ATATTAGCCCGCTCTGATGC	59.305	20	265	2938	3202
60.929	20	CAAGCAAAGGCCAATATCGT	60.096	20	114	63	176
58.956	21	TCAATGCATGATACGCCCTA	60.058	20	237	2147	2383
59.934	20	TCAAATGCTCGACTTATGCG	59.976	20	279	414	692
59.961	21	ACAAACCAAAGTTGCGATCC	59.978	20	193	694	886
59.536	20	GATCGCTCATGCATCAAGAA	59.914	20	123	368	490
60.315	20	ACCATATTTAAGGGTTATCACCTG	57.552	24	236	262	497
60.169	20	TGAAACATGAGGCCTTGCTA	59.42	20	158	562	719
59.945	20	TAGTGGTATGGAGTGGGGGA	60.187	20	140	45	184
59.996	20	CGTGGATGCCCTAGATTGTT	59.955	20	250	379	628
59.702	23	GGACCGGTTAGACCGAATTT	60.187	20	123	268	390
58.982	20	CTGTCCTAACCCGCTGTCAT	60.134	20	277	4	280
59.945	20	CGACACGGATTGAATCACAC	59.967	20	140	366	505
60.068	21	CAAGGCATGCAATCAAATGT	59.548	20	255	504	758
59.605	20	TGAAAAGGCCGGCATAAATA	60.405	20	189	224	412
59.661	20	CCACAAATCATGATGAGCAAA	59.533	21	191	2674	2864
59.878	20	TTCATCCCTAGCCATCGAAC	60.036	20	196	10	205
59.682	20	CCGTACGGTCCCAACATATC	60.074	20	253	1393	1645
60.365	20	CGAAAATCAATGGATGGGAT	59.581	20	227	1370	1596
60.142	20	CACGAGAGAGGAAAGGGAGA	59.525	20	103	28	130
59.558	20	GCAGTTGGGAGAGGAAAACA	60.232	20	178	189	366
60.142	20	GAAAAGCTTGGCCATTCAAC	59.691	20	114	189	302
59.089	24	AGGGGCAAATGTTGATGAAA	60.309	20	221	26	246
59.807	20	CTTCCCCCTTTTCTCTCGTC	60.183	20	258	779	1036
59.822	19	AGTTGAAGGTGACGCTCAAAA	59.906	21	279	27	305
59.935	20	CGTAAAATTCCGGTGCAAAT	59.832	20	161	67	227
59.844	20	ACTGTGGAAAGGGAGCTGAA	59.844	20	115	657	771
60.066	20	AAAGCAAGTTCGATATATTAAAAC	59.345	26	205	31	235
59.021	24	GCGATTCTCATTCCAATTCAA	60.031	21	117	697	813
60.195	20	CCCTAAGATAATGCCACCCA	59.778	20	150	88	237
60.088	20	AGGTCTTGACAGGTTGTGGG	60.002	20	206	903	1108
59.14	20	AGGATACAAGGCAAAAGGCA	59.708	20	213	180	392
58.926	24	GCGTTCACACCCTCATTTTT	59.978	20	191	27	217
60.399	20	CCAAGAACACTCTCCACTCTCC	60.288	22	187	1	187
59.823	20	ACAAACTAATCAAGTTCCGCA	57.439	21	219	344	562
59.838	21	GTTATATCTATAGGGAGGGTTATC	57.293	26	227	1193	1419
60.008	21	TTCGATGATAACTATGGGTTTGT	57.589	23	276	375	650
60.111	20	TGGAAAGAAGGGAAAGTCAA	57.367	20	257	1356	1612
59.813	20	CCTTCCTAGGACATTTGCCA	60.066	20	228	26	253
59.415	20	GCTATGACCCAAGAACCGAA	60.074	20	209	5	213
58.467	25	TTCAAACCGAGCTTGAGATG	58.999	20	255	42	296
60.027	20	CACTTACACACGCACACACG	59.848	20	263	212	474
59.123	20	AGCCCAACGGCTGATTATAC	59.07	20	137	50	186
60.307	20	TCAGGATCTCACCTTCACCC	60.048	20	275	19	293
59.542	20	CCTCCTCCTCACACTGCTTC	59.986	20	201	696	896
59.872	20	CTCTGACCTTGAGGGCTTTG	59.982	20	276	142	417
59.978	20	ATTTGCGATTTATGATGGCG	60.802	20	164	1133	1296
59.864	20	CGTGAGGAAAAACCGTGATT	59.971	20	255	370	624
58.78	20	GCTCAGCCCAACTTCCTAAA	59.452	20	242	30	271
59.809	20	GCACGCGAGACAACTATCTG	59.621	20	123	239	361
59.224	23	CTGTCCCGTCTTCTTCGTTC	59.844	20	173	19	191

59.978	20	GGCCGATTGGAGTATAACGA	59.923	20	279	789	1067
58.83	21	AAGCTTTCTGAATTTGTTTGCAG	59.946	23	235	66	300
59.933	20	TTTGCATGTGTTGCAAATGA	59.691	20	252	35	286
59.899	21	TGGCTCCAACATCACAAAAA	60.088	20	194	261	454
59.707	21	AAGGAGGTGTTATGAAGGGATT	58.028	22	143	70	212
59.88	20	CTCCGATTTCTTGGTGTGGT	59.966	20	225	74	298
59.766	20	TCCAGTCTCAACAAACGTGG	59.72	20	251	1919	2169
60.102	23	TACAGCTCCTACCCGCAACT	59.898	20	229	296	524
59.794	20	CGAAAAATTTAAAATCAAACGATC	60.521	25	219	4	222
59.915	20	GGGAATGAAGAAGTTTCGCA	60.192	20	253	332	584
60.034	20	TTGGTGAGGTGGCATCATAA	59.924	20	185	232	416
59.762	20	TGCAGATTGACGAAGGAAGA	59.522	20	139	970	1108
59.009	26	TGGGAGGAGGCTTAGGATCT	60.169	20	113	2	114
59.901	20	CCAGCAACTACAGGACAGCA	60.049	20	157	836	992
60.067	20	CACTTATAGATGGGTTAGCGTGTG	59.972	24	164	238	401
60.016	20	TTTCTGAGCCTAACCTTGGGT	60.116	21	252	150	401
57.717	22	AATTTTCGACTTTGCCGTGT	59.615	20	238	17	254
58.236	23	TCCTGACCCTTTTTGAGATTG	59.157	21	221	23	243
59.712	23	GGGTACATGTTACGTGCCCT	59.74	20	280	37	316
59.52	20	ACAGGTATCCGAAGGGATGA	59.361	20	108	7	114
60.018	20	CAATTAACGGTAGCCATCACAA	59.89	22	271	436	706
59.439	20	GGTTCATCGGAAGAGTTCCA	60.05	20	237	193	429
60.075	20	GCCGATCATGTCCTCAAAGA	61.159	20	255	101	355
58.984	20	ACCACGCCAGTCAGGTAAAC	60.035	20	144	67	210
59.245	20	CCAATGACACGACACAAACA	58.974	20	186	569	754
59.694	20	AAAATAAGTGGGGTGCTGGA	59.429	20	130	713	842
59.999	20	GAAATGGTGTAAAAGGGGGTT	59.101	21	230	145	374
61.128	20	TGGACACCCCAGATTGTTTT	60.21	20	177	261	437
60.124	21	TGCTTACTGTTGTATGGGGTG	58.566	21	238	166	403
59.823	20	TCTAACGGCCCAGATTTGTT	59.569	20	275	664	938
60.352	20	GAGTTCGTCAAACCTCCCAA	60.088	20	265	838	1102
60.096	20	TACGAGACCGAAAGGAAAAA	57.49	20	208	2	209
60.014	20	GCGCAGCAGCATGTTAAATA	60.009	20	147	3078	3224
60.053	20	ATCCACTGCATGAACAACCA	59.967	20	175	75	249
60.045	20	GTGGCCTTTGTGGGCTACTA	60.132	20	194	2564	2757
59.464	20	AGATTCAGACGCATTTTGGC	60.221	20	251	2112	2362
60.214	20	TGAATTGGTGACACACACGA	59.541	20	209	1113	1321
59.864	20	TCCATCTGCTGTCTAACCCA	59.241	20	225	23	247
59.716	20	TTGAGGACATTTATTCGTCGG	59.946	21	153	413	565
59.935	20	TCACTCATACCCTCCCTCCA	60.465	20	273	304	576
59.933	20	TGCAACGCCAACTACAAAAG	59.911	20	145	9052	9196
60.372	21	TGCCTAACGTTAAATGGGCT	59.609	20	249	0	248
60.152	20	ATTTGGCTTCCAAAGCTTCC	60.562	20	209	335	543
59.697	21	CCACTAACCCAAACCCATCA	60.605	20	119	12	130
59.96	20	TGCTGTGAATTGTCCAGCTC	59.992	20	246	917	1162
60.14	20	AACATGGTTGGGCTAAGTGG	59.853	20	175	5	179
59.569	20	TCATTTAGAATTGGTGGAAAGATT	58.153	24	273	7	279
59.952	20	TTCCTTCAATCCATGCAACA	60.049	20	207	511	717
60.12	20	TGCCAGCGTATTGTGAAGAG	60.011	20	187	150	336
59.705	20	ACAACAAAAGGCAAAGGCTG	60.284	20	252	781	1032
59.923	20	GGCTTGCCCTTAACCCTATC	59.93	20	222	408	629
59.75	20	CCCCAAGAACACTCAAGAACA	60.133	21	233	358	590
60.149	20	CCACCTTGTAGCTTTTCCCA	60.103	20	221	1128	1348
59.637	20	CACTTTTCCAGTCTGCTCCC	59.844	20	130	415	544
60.055	25	AATCGGACCGGATCGTTAAT	60.531	20	210	134	343
60.081	20	TTAAACAAAGCGTGCAGACG	60.052	20	169	2611	2779
60.089	20	TATCAAACGAAGATGTGGCG	59.688	20	200	112	311
59.799	20	AGATTTCGATTTCCCCGAAC	60.265	20	189	213	401

59.923	20	CCCAGTAACCAACGCATACC	60.249	20	162	399	560
60.073	20	GGGGTACCCAATATTCTCCG	60.391	20	264	43	306
59.931	20	CCATATCAGAGAAGGGCAGC	59.797	20	242	42	283
59.96	20	GGAGTGCTCATGATCCGTAGA	60.233	21	207	154	360
60.044	20	ACGAACAGAATCCTCATCCG	60.073	20	268	87	354
59.026	20	TTTGACCGAACCGTTGTGTA	60.004	20	189	2312	2500
59.887	21	TGATGCAAATCACCTTCGTAA	59.172	21	271	105	375
60.125	20	GTGGACGGCCATAAGAAGTC	59.556	20	262	191	452
59.803	20	TCGGGGTATCAGAAATCGAG	60.029	20	167	79	245
60.745	20	GAAAAAGTGCTGGGTTGAGG	59.711	20	225	48	272
59.867	20	TGTAGGCGCCATCTTTTTCT	59.845	20	162	625	786
59.916	20	AATTTTTCGATCATGTGGGC	59.768	20	156	13	168
60.045	20	TCGTGTGTGTGAATTAAAATGAA	58.136	23	261	101	361
60.448	20	TATATATGGGTTATCACCTGATCG	57.071	24	149	463	611
59.997	20	CATTCTGTGTTCTCTGTTTTCGAT	59.696	24	260	916	1175
59.598	20	CATGGCGTTGTATTTGTGCT	59.614	20	199	1999	2197
59.216	21	TGGAAGGCTGTTCCGATAAC	60.074	20	188	1323	1510
60.776	20	CACCCTCTCTTCTGGTCAGC	59.986	20	230	537	766
60.01	22	ACCTGCACCAAAATGGAGAG	60.111	20	135	51	185
58.718	26	CTCCCAGCTCACGTTCTACC	59.867	20	270	4	273
60.011	20	TTTCTTCCATTCCATGGTCC	59.727	20	220	74	293
59.586	20	TGCCGTATATGGTGCTCATT	59.019	20	280	202	481
60.251	20	ACATGGAACTGCACCAGATG	59.551	20	231	100	330
59.343	21	CCGGTCGAATATTTCCTTCA	59.894	20	271	252	522
60.218	20	CATCAGAGCCACGGTTAGGT	60.134	20	240	87	326
60.169	20	TGTGTGTGAGTGTGTGGGTG	60.088	20	196	57	252
60.01	20	TCACGTCAGGTTCACCATGT	60.005	20	205	238	442
61.021	20	GAGAAGGGGAGAGGGACTGT	59.661	20	190	53	242
60.116	21	TCAACAGAGAATGCAAGCAAA	59.6	21	162	82	243
59.072	20	CGGATTAGCCACGGTCATAG	60.481	20	257	174	430
60.324	20	CTCCACTCCAAACCACCATT	59.82	20	225	356	580
59.791	20	TGTCCGTATTCATTCCGTGA	59.924	20	262	511	772
61.204	20	CGGGTAACGGGTAGGGTATT	59.96	20	280	84	363
60.357	20	CCTTAACATGTCGGGTTCGT	59.853	20	199	7	205
59.916	20	TCCATTCCGAAAATCAAAGG	59.872	20	153	184	336
59.15	20	GACTTCCGCCTTGTTCTTTG	59.853	20	219	225	443
59.342	20	GATTAACCTCGCATCCCTCA	60.036	20	160	439	598
59.225	22	CCAACCAATACGGGACACTT	59.711	20	262	717	978
59.528	20	AAAATCAAAGGCCCACAATG	59.801	20	226	975	1200
60.035	20	GACATCTCCCTCTCCGACC	59.583	19	194	328	521
60.301	20	CTGTCTCTCGCTCTCGCTCT	60.182	20	274	428	701
59.942	20	GACTGTGAGGAAACGTGCAA	59.88	20	223	500	722
59.111	23	GGGGAGAATGTAATATCCCGA	59.992	21	222	24	245
60.182	20	CCCTAACTCGACCCAGATCA	60.065	20	221	41	261
60.578	20	GATGCAGAATGTCGGTGAGA	59.794	20	237	1109	1345
57.268	23	TGGAGGTGAAGAGAGGGAGA	59.913	20	253	167	419
60.103	20	ACTATCAGCCGCCAAATGAT	59.556	20	140	210	349
59.648	20	TCAAGGATTCAAGCCCACAT	60.461	20	100	19	118
59.757	20	GCCCAAAAAGAACACTCACC	59.574	20	277	50	326
60.179	20	CATGATAATGCACGTCTCCG	60.096	20	104	91	194
60.008	20	TGAAAGTGATTCCCCTTGATTT	59.814	22	177	6	182
60.036	20	GGAAATAAGGAGGCGTTATGA	58.213	21	127	184	310
59.813	20	GTTGGACTCAAGTCATGCGA	59.837	20	156	298	453
57.33	26	CATCCCCTTCATTCAACACC	60.173	20	105	9	113
59.75	20	ATGTCTCAGCCGATCGATTT	59.658	20	162	1965	2126
60.088	20	CGTTGCATGGTGTATTGGAC	59.847	20	253	2217	2469
59.787	20	TTGCCTTTAATCGATCCTGG	60.031	20	176	4	179
59.948	20	TTCCACCTTTCACCCTGTTC	59.943	20	200	513	712

60.103	21	GAAGGAATTGCAAGGGACAA	60.051	20	181	285	465
59.878	24	TGGTTTTGGATGATGGATGA	59.707	20	159	22	180
60.699	20	CACCTAAAGCCCATGCATTT	59.96	20	279	47	325
59.822	19	CCCCTTGAACTCCAGCAATA	60.066	20	203	201	403
60.251	20	CAGTGGCTACCGAGCATTTT	60.27	20	120	2922	3041
60.035	20	TCCCCAAATGAAAGAATGGA	60.244	20	190	243	432
59.872	20	GAAAACGCCAAAAATCGTGT	59.982	20	114	20	133
59.903	20	CGAATTCTCTCTCTGCAAACC	59.066	21	103	289	391
60.074	20	GGCATGAGCGTGTAAGATGA	59.83	20	193	162	354
60.042	20	TGTCCGAAAAGGCAAAAATC	60.053	20	169	29	197
60.074	20	AGTAGTCGTCGAGGTGGTCG	60.321	20	228	298	525
59.052	20	CCCCGAACAGAGTCAAATGT	59.966	20	275	599	873
59.668	20	CTGGAGGAAAGAGCTTGAATC	58.134	21	168	1563	1730
59.943	20	GGGCACTCTTGTGTTTAGCC	59.74	20	260	1211	1470
59.953	20	GTTAGCCACCAATGAGGCAT	59.962	20	274	440	713
59.051	25	GTCTAGTGATGACGGGGAGG	59.53	20	198	1	198
60.22	20	TTGTTTTCCAAGCCCCATAA	60.294	20	134	320	453
60.022	20	TGGGAACTTGAACTTGGAAGA	59.697	21	247	351	597
60.291	20	ACGTCTTTGTGTCGAAACCC	60.012	20	173	178	350
60.02	20	TCGATAAAACTTTACCAACACCAA	59.817	24	250	15	264
59.82	20	TAAGTTCTCCACAGCGAGCA	59.74	20	177	143	319
59.318	27	GGACGTCTTTCTTGAGCAGC	60.142	20	203	4	206
58.286	21	GAAAACAAAATGGGGAGCAA	59.916	20	241	19	259
57.416	20	AATGCAGCGGAAGAACAAAT	59.713	20	201	4	204
59.966	20	GGTAGCGAGAGAGGGGAAAG	60.34	20	255	555	809
59.836	20	CTTTGTGAAGTAGTCGCCCC	59.734	20	177	10	186
59.836	20	GGCAACGGAATACCATTTGT	59.691	20	147	42	188
59.215	20	TTGACAATATTTAGTGTTTTGACAT	58.99	27	269	189	457
60.034	20	ATTTGGCCGGTTCTCTTTTT	59.945	20	198	4527	4724
60.173	20	GAGGACGTTGTGGTGGTCTT	60.009	20	188	199	386
60.542	20	CTGCACTAACGCCATTCCTT	60.27	20	271	223	493
57.318	22	AAGGAGGTGTTATGAAGGGATT	58.028	22	129	95	223
60.048	20	GGGAGGGATAAATGGGAAGA	60.089	20	244	1358	1601
57.885	21	ATATGTGGCTTGAGATCGTGT	57.152	21	248	136	383
59.933	20	TCGACCGAAGAGCTATCCAT	59.797	20	228	159	386
59.911	20	TTTTATGTAAATTGTCGTGCGT	57.373	22	253	182	434
60.401	20	ATCTGGGTTACACTGAGCGG	60.134	20	216	1	216
59.616	20	AGACTGCGGTGCAAGACTTT	60.058	20	263	1453	1715
60.24	20	TTCAACCAGGCAAAGACCAC	61.075	20	189	136	324
59.923	20	ACCGGAATAGCATCAGTTGG	59.955	20	221	7	227
60.665	20	TGGCTGTCAGATGATGTAAGTTC	59.256	23	175	354	528
59.744	20	GCATTCGGTTGATTATGCAG	59.14	20	243	953	1195
59.988	20	GATTGGCTCCATCCTCTTCA	60.158	20	254	385	638
60.045	20	GAACTCCCCTCTCTCTCGGT	59.807	20	120	89	208
60.149	20	CTTTCTTCTTCATTTCGCCG	59.953	20	128	3	130
60.036	20	TAGGTGTAGGTAGCCAGGGG	59.073	20	171	466	636
60.013	20	GCACTATCTCTCCCTCACCG	59.827	20	123	319	441
60.048	20	TATGCAGTGGATTTGGTGGA	59.924	20	195	179	373
60.463	20	GAAGCCTCAAATCACAAGCC	59.82	20	142	106	247
59.232	22	TACCGTTGGTTAATCCTGCC	59.823	20	117	0	116
59.992	20	GTCAACTTTGGCAACGGAAT	59.978	20	176	42	217
59.989	20	CAAGAGCTGAAGATGTGATTGC	60.022	22	198	259	456
60.619	20	TTCGAATGGATTTGTTGGGT	60.17	20	156	394	549
60.03	20	GTTGTCTGAAACCCTTCCCA	59.943	20	122	238	359
59.312	20	CGACCCTGTAAGGTTCTTTGA	59.225	21	272	130	401
59.929	20	GCGAAGAAGAGGGGAAGAAT	59.789	20	232	61	292
59.982	20	TTCTACAGCGAATGATGCCC	61.141	20	229	844	1072
58.982	20	AATCATTCCATTCCCCGATT	60.345	20	246	37	282

59.145	22	AAGGAGGTGTTATGAAGGGATT	58.028	22	238	34	271
57.077	21	TGGCAAACACAGGTCACAAT	60.008	20	163	5	167
59.4	20	GGTCGTCGGATTTCATTTTG	60.309	20	261	2756	3016
60.322	19	GGATACGGTGTCGGAAACTC	59.41	20	269	1391	1659
59.972	20	TTGAAATTTCGGCTTCAACC	60.053	20	221	4	224
59.203	20	CGACAACAATGCAAGGAAGA	59.84	20	183	14	196
60.074	20	TGTAAATACGGTCTGTGCATGTT	59.46	23	149	323	471
59.276	21	TGCTTTGATTTGATGGTTATGG	59.832	22	173	4291	4463
60.073	20	GATGATGGGGAAGAGACCAA	59.862	20	142	254	395
59.801	20	TCTCAAAATAACCAAAATCAGCA	58.796	23	220	109	328
59.846	20	ATAACCCGTGTGTGGGTTTA	57.729	20	230	102	331
60.257	20	TGTCGAGAGGCAAGTGTTTG	60.025	20	177	32	208
59.267	20	TCGCAGTCCATCACTCCATA	60.225	20	139	268	406
57.589	23	TGGAACTTGTGTTTGTACCCA	58.934	21	199	18	216
60.608	20	CGCAAGTACAACAACATGCC	60.18	20	270	235	504
60.111	20	AACTTGTATCACTGGAAGAATTCA	58.329	25	275	102	376
60.001	20	CAATTTTCATTGCCCCTCAC	60.309	20	176	46	221
59.83	20	GACGAGAATGCCCCTGTATG	60.483	20	200	331	530
60.151	20	TGAGTGGAAGAGAGGGAGGA	59.913	20	128	80	207
59.931	20	AGAGCCACGTTAGGCTCAAG	59.639	20	279	79	357
60.074	20	TCAACAGAGAATGCAAGCAAA	59.6	21	186	11	196
59.915	20	GGTTCTGAGACCGGGATAGG	60.842	20	273	0	272
59.858	20	AGGAAGCGAGGTAGGCTTTC	59.983	20	190	199	388
59.836	20	TCTTAGTGATTTTTCTTCGTCCA	58.064	23	247	666	912
59.708	22	GACAACAGTCACGTGGCATC	60.171	20	278	590	867
59.934	20	CGTGTGTCAGCGTGTCTCTC	60.685	20	177	1617	1793
59.984	20	TGGTTCATGCCATTGTGAAT	59.781	20	124	39	162
59.591	20	GAAATTTCACGAAACAACGAATC	59.877	23	258	299	556
59.09	20	TCTTCCAAGATGGGAATGGA	60.395	20	187	112	298
59.827	20	CCGTCTGGTCATCCCTTTTA	59.926	20	260	558	817
59.867	20	TCTCTCTTGGTTGGTGAAGAAA	58.955	22	169	53	221
60.47	24	ACCTTGAAATGCAACCGAAG	60.11	20	264	1627	1890
58.381	25	GCTCGAGCTCAGACATCAAGA	60.839	21	276	454	729
60.633	20	TCCAAGCTTTAATTGCGGAC	60.209	20	133	441	573
59.992	20	TATCCCGTGGGTCAAATACG	60.576	20	234	962	1195
59.96	20	TGGATGCGAAACATGAAAAA	60.051	20	271	513	783
59.795	21	TTTCACCACCGCCTTAATTC	59.938	20	249	28	276
59.971	20	CTGCTCTCCCAAGCAGAATC	60.096	20	244	3682	3925
59.742	20	CCTTTCAGGCATTTAAGTGGA	59.211	21	275	1747	2021
59.836	20	GTACACGCACCAATCGTCAC	60.04	20	276	886	1161
59.472	22	TCAGTTTCTGAGTTTTGAGGTTTG	59.835	24	108	747	854
60.057	21	TCTAAGCTTGTTAACCCCCG	59.221	20	232	9	240
60.265	20	TTTTCGACCAATAGGAACGG	59.931	20	132	1333	1464
59.978	20	GAGATGTGGTGGTGGTGATG	59.801	20	136	388	523
59.751	20	AAAGTCCAAATGTTGGCGAG	60.11	20	121	1552	1672
59.827	20	TAAATCACAAAACCCACGCA	59.969	20	135	343	477
59.67	20	CCCAGCAACTGTTGGCTACT	60.312	20	239	305	543
60.11	20	TTTTTAGTAGGGTTTTCAAGTTCAC	59.564	26	265	356	620
59.966	20	CATGCTTGAATGAAAACCACA	59.576	21	122	272	393
59.841	20	TGTAGGGGCAATGATGTAAAAA	59.363	22	232	25	256
60.088	20	CAGCCCAATAACTCAACCTACC	59.892	22	101	44	144
60.501	20	GGAGCCTCCGAACTTTGAA	60.324	19	188	56	243
60.002	20	TCCACTGTCTTGTGCCTCAG	60.022	20	127	891	1017
59.764	19	GCCAAGAGGTGAGTTTCTGG	59.844	20	168	9	176
59.54	20	ACAGTTGAAAAAGGAGGGCA	59.711	20	280	765	1044
60.05	20	CTTTGAATTGCAGCTCGACA	60.134	20	267	1127	1393
58.06	20	TTTCTTGTGTGTGTGCATGTG	59.203	21	170	181	350
58.826	22	TACGAATCTCAAGGGGCAAC	60.074	20	212	99	310

59.855	26	ACGTAGTTTGTGGGGGAAAT	58.293	20	280	916	1195
60.49	20	TATGCAGATTGGTGTGTGGC	60.549	20	262	141	402
59.667	24	CGTGCTGAGCTTATTCACCA	60.011	20	191	2	192
59.26	20	TGCGCAACCAATGTAATAGC	59.731	20	234	785	1018
59.521	20	GATCGTGTCCGGTTTGTGTA	59.42	20	251	104	354
60.067	20	GGAACCTGTTTTCCCAGTCA	59.943	20	215	215	429
59.624	20	TCGAAGGTGACTATCCCGAC	60.073	20	241	108	348
59.872	20	GCAGCATCCTTCACCATTTT	60.081	20	162	72	233
57.874	25	GCAGTAGGGTTGGAGATGCT	59.313	20	263	7	269
59.928	20	GTGGACGTCCCAGATTGTTT	59.827	20	182	665	846
60.178	20	GCATAGAGGGCAAGAAGACG	59.978	20	192	427	618
59.248	20	CGTTTTGATGATATATAGGTGTTTT	59.637	27	237	378	614
60.264	20	CCCTTAATCTTTGTGGTTGTGA	58.994	22	273	1896	2168
59.985	21	TTTGAGTTTGCATCTAAGAAGGTG	59.828	24	117	835	951
59.609	20	TTTGGTTTTATCGAAGAGGCA	59.707	21	260	845	1104
60.111	20	GACTCTCCAACTAAGGGGGC	60.074	20	276	243	518
60.557	20	CAGGAGCACTACAGTTCACGA	59.097	21	264	2128	2391
60.068	20	CGGCCAAGATTTGTTAGAGG	59.702	20	195	380	574
60.024	21	CATTCGGTTGAACCCTCAGT	59.966	20	109	189	297
59.82	20	GGATTGATGAATTTCCATTCG	59.221	21	194	1757	1950
60.376	20	CCATCATTCCAATTCAAGGAA	59.748	21	264	1869	2132
59.443	21	TTGACCAAGATACACCTTGCAT	59.499	22	185	17	201
60.289	20	GCTCTTTTATTTACCTTTTATGCTC(	60.067	26	212	338	549
59.93	19	GAGGGTTGTTGTTGTGGGAC	60.261	20	200	0	199
58.753	20	CCGAGATCTTTTCATGAGTGC	59.828	21	249	834	1082
60.307	20	TATGTTTCGGCCCGGTATTA	60.165	20	154	45	198
59.964	20	GTTCAATTTGGACGTTTGGG	60.206	20	267	530	796
58.388	25	TGAATCTCATGTTATTGAGTGATG	57.699	25	206	671	876
59.937	20	TTTTTCAACGTTTGCCATCC	60.846	20	183	189	371
60.088	20	GCCGGAATCCATTACTTCCT	60.286	20	220	17	236
60.21	20	CTTGTGAACTCCGATGGACA	59.676	20	156	448	603
59.935	20	TCTCGAGCAACAGAGCTCAA	60.008	20	219	155	373
60.152	20	GAAATCCCGCTCAATCTCAA	60.155	20	218	396	613
58.792	20	TCGGCAGAAAAGAAGAAGGA	60.066	20	114	428	541
59.621	20	GATGGCCCAGATTTGTTTTG	60.309	20	102	106	207
59.238	23	GGCGAACATGATGAATGAACT	59.954	21	280	23	302
57.557	25	GAGAGAAGTAGCAAGCGCGA	60.955	20	181	10	190
59.939	20	AAATGCTCATGCATGCTGTC	59.836	20	182	44	225
58.707	23	ACACACAAGCCTCGCTCTTT	60.058	20	223	658	880
60.435	20	TCAATGACGAAACATATCCCA	58.832	21	192	1779	1970
59.673	20	CTATCGAGCGTGTGGCAATA	59.856	20	146	24	169
58.004	25	CTTGTGGCTTGCAAACATCT	58.926	20	162	73	234
59.981	20	GCAGTGGGTTTTGAATGGTT	59.836	20	225	210	434
59.993	20	GCATTTGAAAGGTTTTATATGATC/	60.438	27	160	491	650
59.64	20	CTTACGGAATTCCACTGGGT	58.909	20	247	14	260
59.976	20	CGTAACCTTGGTGTAGCCGT	60.052	20	252	563	814
59.993	20	ATGTATGGCAGGTGACCCAT	60.081	20	274	123	396
59.146	20	AGTTGTCAATGGAGGTTGGG	59.82	20	252	267	518
59.787	20	AGAGCGAAGAAAGCAGATGG	59.717	20	143	1357	1499
59.849	20	ATGAATGTGGAGCAACGTGA	60.12	20	268	2148	2415
60.132	20	TTTGAACGCGTGACTTCTTG	60.027	20	259	4833	5091
59.267	22	GCATTAAATGAGCCTCCCAC	59.533	20	125	5	129
60.042	20	AAATGCTGGTAGAGGCAACG	60.27	20	257	322	578
60.142	20	TGATACAGGTGTTCCAAACAATG	59.777	23	247	6310	6556
59.964	20	AGGGAGAGGCTTTGGATGAC	60.596	20	232	39	270
60.214	21	TGCATGTGTGGGGCTATTTA	59.953	20	218	1005	1222
60.255	20	AAATTCCGTTTTTGCCTTCC	60.292	20	183	193	375
60.564	20	AGAAAGAAGTCGGCAACCCT	60.247	20	163	1170	1332

59.173	21	TCGGGCAACTGGAGCTAATA	60.732	20	212	2	213
59.962	20	TGCCACCCTAGATCCGTTAC	59.955	20	175	517	691
59.052	20	TGAAACAAAAATATAGCTAATGC(	59.293	25	273	385	657
57.436	27	TGACGAGTCCATTGTGCATT	60.12	20	159	14	172
59.236	22	GGGAGAGGATCAAGAGAGGAA	59.763	21	201	37	237
59.844	20	GATGTATCGCCCAAAGGAGA	60.036	20	225	2484	2708
60.111	20	CTGCCTTCCAACCATACACA	59.566	20	267	453	719
59.688	20	AATGGGACTGGCGATTGTAG	59.955	20	267	95	361
60.012	20	GGTCACCGTTCCCTGAAATA	59.79	20	257	1395	1651
60.162	20	TGTGAATGGAGGACAAACCA	59.935	20	156	201	356
60.081	20	TGGCAACAAATATGACTGCAT	59.055	21	116	341	456
59.905	20	TCTCCGAGGAGACAACTTTCA	59.974	21	170	4000	4169
59.33	25	TCTTTGAATTCAGCACGCTA	57.257	20	140	35	174
60	25	TTTTGATGATATATAGGTGTTTTGA	58.782	27	150	616	765
57.548	22	GATGGTGGGTCTGAACGAAT	59.786	20	212	554	765
59.911	20	CTCCGAGATCAGAGCTCACC	60.096	20	227	1756	1982
59.516	20	CCCATTTCTAACTACCCAAATCC	59.962	23	212	231	442
57.926	20	GGTTTGATGACGTGGCTTCT	60.119	20	194	1107	1300
60.073	20	AATGCCTCTGCAAAAAGCTG	60.523	20	189	2874	3062
59.469	23	CCCACCCCACTACTCAAGAA	59.959	20	116	8	123
57.236	26	GCTTTCCCATTTTGCTTTTT	58.361	20	280	646	925
59.247	20	TTGTGATTTGGTGTGTGTATGG	59.22	22	249	247	495
59.817	20	AACGGCTTGGATTCTTTCCT	60.074	20	147	397	543
59.823	20	ATATCCCTTGCACGACGAAC	59.962	20	274	523	796
60.096	20	TAGCCCATTATCTCCCACCA	60.288	20	162	1575	1736
59.938	20	TGCCTCTGCTATTACATGCC	58.897	20	211	967	1177
59.271	24	TGGAGAAACGAACTGGAACC	60.088	20	252	205	456
60.173	20	TTGTGGCAGGTTATTCATGC	59.548	20	201	432	632
59.964	20	CACCATGACGGCAGATGTAT	59.399	20	251	636	886
60.119	20	CGGCTTCACCAGTTTCTTTT	59.355	20	138	246	383
60.203	20	ATTGTATGGCGAGAGGTTGC	60.103	20	278	27	304
60.483	20	CACACGTTCTTCCCCAGATT	59.966	20	143	45	187
59.646	20	TTTTGAGGAGTTTGCGCTCT	60.132	20	239	756	994
60.011	19	GATGATTGCACCATAATGCG	59.921	20	126	2678	2803
60.073	20	CCTGCTTTTGGATGTTGGAT	59.933	20	172	1036	1207
59.703	19	GTGCATCCATCTGAGCATTG	60.233	20	246	186	431
59.884	19	GTCGTTCTGAGCTGCCTTTC	60.142	20	237	2257	2493
59.96	20	GGGTCCCACCACCTAACATA	59.526	20	232	567	798
59.911	20	TCCCTTAGACCTTACATAATGAAT(	60.001	26	221	45	265
59.957	20	AGATGCTGGAATCTTCGTGG	60.218	20	138	151	288
59.82	20	TCCAAAACTGGGAACCATTC	59.767	20	140	325	464
59.746	20	TCCATTAAGAATTTCCCATGC	58.888	21	178	1623	1800
60.062	23	CAGTGCGGACGGTTAAGATT	60.132	20	208	19	226
60.136	20	GAAGCTGGTGCAATTGTTTG	59.322	20	247	96	342
59.518	20	TTCTTGGTGGCGGTAGGTAG	60.125	20	198	1	198
60.119	20	TGACGATGAGACGTGTAGGG	59.701	20	279	303	581
59.293	20	CTTTCTTCACTACTGGAACTTTCAT	57.353	25	222	54	275
60.035	20	GATAAGAACTCTGGCTCCAAGG	59.371	22	255	354	608
60.096	20	TCATGCACGTGTTTCACAAA	59.721	20	276	184	459
60.032	20	AAAAATCTCAGGGACGGGAC	60.306	20	173	3450	3622
60.021	20	GTTTGCTTCCACGGCTAAAC	59.752	20	268	1205	1472
60.02	20	GTGTCGATTGTGTTCATGCC	59.975	20	188	96	283
59.883	20	ATACTTTGGCCCAGTTCGTG	59.993	20	261	2980	3240
59.969	20	TCGAGTGTTCGTTAGTTAGTGAGG	59.876	24	106	141	246
59.717	20	GGTGGTTGCTGGGTTAGAGA	60.111	20	200	7	206
59.225	20	ATTTCGAAGATCAACGGACG	60.074	20	206	195	400
59.394	20	GTTGTTTGGTTCACGGGTTT	59.734	20	249	109	357
60.053	21	CGTTCTTAATTAGCGCCGAA	60.347	20	114	1020	1133

59.988	20	GAGCGTCGATCTGGTGAAAT	60.226	20	253	385	637
59.573	21	GCGATCGAGAGAGAGAGGTG	60.246	20	176	1068	1243
60.362	20	CAAACAACGATCGACAATGG	59.964	20	207	35	241
60.642	20	TGACGTTAAGAGAGTTTTCACTGG	59.855	24	179	1004	1182
59.948	20	TTTGGCAAGGGTTTGAAAAG	60.081	20	243	167	409
60.014	20	ATTGGAACGTTTGAAACCGA	60.344	20	251	221	471
59.15	20	GGGGATTTGTCAACTGGAAA	59.767	20	236	58	293
59.763	20	CCTCACACATTCAGCCCTCT	60.261	20	276	144	419
60.042	20	ACCAACGTCCCTCATCTACG	59.989	20	247	191	437
59.485	21	GCAAACGGTATAGGATCGGA	59.923	20	192	247	438
60.11	20	TGGCTCTCACACTCACTCTCA	59.747	21	187	106	292
59.632	20	TTCGGCTTAATGGTCGAATC	60.038	20	208	61	268
60.05	20	TGATCCATTTGCAGAAGTCG	59.799	20	264	415	678
60.133	20	AACACGTGGAGATGACACCA	60.005	20	181	551	731
60.173	20	GCTCCATTTGTAAATCACCCA	59.815	21	222	158	379
60.034	20	TCACACAGATTAAACACTGAAAC	60.128	26	253	567	819
60.051	20	TGGAAATTTCGACACGCTTT	60.617	20	252	566	817
60.218	20	CGCGAAGTACCTGCGTTAT	58.956	19	274	35	308
58.353	22	AACTGCTGGACCTCCACAAC	60.159	20	194	308	501
60.034	20	GGGGGAAAAAGAAAGGTTGA	60.271	20	196	15	210
60.073	20	TGAATCAATCACGTTTCGCA	61.204	20	218	14	231
59.202	20	TTTGGAGATGGTGATGGTGA	59.893	20	276	65	340
59.274	25	TAGTCTTTCTCCCGTGCGTT	59.875	20	274	19	292
60.11	20	CAGCAAGAAAAATACACCAGCA	60.298	22	134	95	228
60.43	20	CCTTGGCCTTCTGACATCAT	60.073	20	190	135	324
60.683	20	TGACACTCACGCACACACAC	60.437	20	265	599	863
59.493	20	TGTAGCGTGGTGAGACAAGG	59.897	20	276	84	359
60.241	20	AGCCCGAGCCTAAGCATATT	60.211	20	265	285	549
60.472	20	CCCCCTTAACTACCACTTATGAAA	59.692	24	213	368	580
59.716	22	AAATCCCGTTCTGGGAAACT	59.805	20	275	1633	1907
59.401	20	GGGATTTGGGACGATTTTCT	60.131	20	147	199	345
59.803	20	TTAGAAACGGAGGTTGGGTG	59.964	20	139	15	153
59.607	21	TGAGCCTATCTTGGAGCACA	59.547	20	190	410	599
59.587	22	TCCCTCTCATCTCTCTCGATTC	59.919	22	118	87	204
58.003	23	TATTGTTCGCGCATCATTGT	60.103	20	126	51	176
59.96	20	GTTTCATCCCTCGCACATTT	59.939	20	260	200	459
59.664	20	ACGTTAAGGAGGGGATCCAT	59.651	20	258	19	276
59.871	20	CGTCCACGGAGAGGTTAGAG	59.861	20	169	264	432
59.84	20	TACGAGCGCATAGCCTTTTT	60.006	20	120	1375	1494
60.066	20	ACCTTAGTTGCGAAGACGGA	59.875	20	164	47	210
59.682	20	GGTTTCTTACTCGGGCTCCT	59.713	20	215	213	427
59.948	20	GAAAAATCAAAGGCCCACAA	59.916	20	246	93	338
60.11	20	GAGTTTATCAGCGGTCTCGC	59.985	20	157	3475	3631
60.088	20	GGCTTTCTTAACTTGTTGGAACC	60.376	23	205	3	207
60.02	20	GTGATCAACATCCGTGATCG	59.925	20	148	638	785
61.266	20	ATTCGGGAGATGTTCCAGTG	59.927	20	170	190	359
59.871	22	ACATTGGGGACAATGTTGGT	59.95	20	139	71	209
60.08	20	ATCCATAATGTCCCCGTTGA	60.014	20	277	269	545
59.966	20	TTTTCCTGGAACGTGAAAATC	59.054	21	279	162	440
59.359	20	TGTACCTAAAAAGTTGTTCCCAC	57.314	23	280	442	721
59.998	20	CCACTCCCACCCATACAAAC	60.088	20	145	3416	3560
59.989	20	CGTCACAGCCTCTTCAACAA	60.025	20	123	644	766
59.845	20	TCCGGTAGGTAGCCATTGTC	59.955	20	265	428	692
60.07	20	TGAGCCCGTAAATCACACAA	60.111	20	183	293	475
59.962	20	TTCTTCCACGTCCTCTAGGTG	59.338	21	160	226	385
60.832	20	TCTCTCACGCACTCTCACAAA	59.76	21	166	66	231
61.136	20	TTCGTCGTTCATTGCAGTTT	59.322	20	123	7	129
60.292	20	AAGGAGGTGTTATGAAGGGATT	58.028	22	180	267	446

60.272	20	CTCGAAAATTGAATGACAAGGA	59.202	22	181	255	435
60.159	20	TTTGTCACATGCATTCCAGC	60.675	20	211	96	306
59.931	20	TTTCTCATTGGCAGTCACCA	60.24	20	171	45	215
60.06	21	TTGATTCCTCCTCACTTGCC	60.195	20	162	14	175
59.758	20	CATGAGCAATTGAGCAGCAT	59.979	20	266	67	332
59.931	20	TTTTCTTCCCCACACACACA	59.976	20	183	287	469
60.081	20	TGGAGCTTTGATCCTTGCTT	59.955	20	151	26	176
59.706	20	GGGGGCATTTTGCACTATTA	59.795	20	260	238	497
59.934	20	TGCCATCTCCTCATCACTTG	59.787	20	189	420	608
59.971	20	AATTCAAGAATGCTGCCCAC	60.081	20	267	247	513
59.078	22	TCACTGGAATGCTATGGTGC	59.679	20	208	2	209
59.648	20	CGGATTTAAGGGTTATTACCTGAT	58.81	24	116	136	251
59.499	21	TTTGCGCATAAATATTGGTTTC	59.039	22	264	0	263
59.571	23	CGAACATAGTGCACCCACAC	60.032	20	235	29	263
60.074	20	TTAGTTATATGTATGGGGATGGTT/	57.868	27	122	107	228
59.72	20	TCTTTTTCCTGAACGATGAGC	59.443	21	267	1480	1746
59.807	20	TTCTTTCCTTTGAGCATACGATT	59.302	23	187	11	197
59.903	20	ACACAAACGAACCGGAAGAG	60.149	20	216	379	594
59.95	20	CAGGCAAATAGCTACCGAGG	59.861	20	227	106	332
60.111	20	ATCAATCTTGGGCTCCTCCT	60.037	20	211	2968	3178
60.066	20	GACTCGGAGGTGAAGGTGAG	59.835	20	192	77	268
60.306	20	GGATCTTAATTGGTTCGATTTTG	58.914	23	169	650	818
59.653	25	TATTTATGGTGGCTGCGGTT	60.343	20	265	509	773
60.404	20	GCACATTGATATCGTGTCCAA	59.415	21	253	193	445
59.904	20	CCTATGGTTGTATGGTTTGGAG	58.377	22	171	38	208
57.272	25	CCATCCATTTGCAACCATAA	59.232	20	225	34	258
59.96	20	AACTGGACCGAAAAATGCAC	59.978	20	118	554	671
59.934	20	TTGTTCAAAATTCGTCCAAAA	58.175	21	277	140	416
59.055	21	CGGGGCGTGACATTAGTTAT	59.845	20	147	39	185
60.056	20	AAGAGGTTATCCCTCGTCCA	58.594	20	278	425	702
60.119	20	ATTGTCAGCCCACTTTGACC	59.973	20	259	429	687
59.274	22	CCGCCTGCATTGTTTCTAAT	60.096	20	138	5	142
60.088	20	CATTCGAACATCATCATCCG	59.883	20	275	338	612
59.449	20	TTCCTTGGACCAAAATAGCC	59.019	20	183	42	224
60.05	20	ATTGATCGCATTCAGGGAAA	60.414	20	260	380	639
60.232	20	AAACAATTTTCTGGCCATGC	59.945	20	249	146	394
59.903	20	ACCATCCCACCAGCATTTAG	59.813	20	127	154	280
60.164	20	GGCCATCAGGGATTCAAGTA	59.894	20	120	166	285
60.133	20	CGCTGATGTGATCTCAATGG	60.225	20	252	88	339
59.049	20	ACTGGTGAAGATGCCTGGAG	60.261	20	264	602	865
58.881	23	GGGTCAATCGGCAAAAATAA	59.773	20	218	492	709
60.067	20	TAAATTGCTCCGTCTCCAGC	60.352	20	161	83	243
59.966	20	ATGGTGTTCTTCCATCAGGC	59.934	20	216	1	216
60.342	21	GGGTTGCCAGGTTGAATAAA	59.801	20	210	262	471
59.813	20	TGTAAGCAGGTATCATCATAAGC/	58.854	24	126	94	219
60.689	20	TTTCTCCCAACGTAGCCAAC	60.11	20	253	10	262
59.803	20	TTACACTCCCACTCCCGTTC	59.966	20	275	279	553
59.903	20	GTGTAAAGCATGGGGAAGGA	59.933	20	250	298	547
59.541	20	GAAACATTTCCACTGAATCAACTG	59.913	24	138	149	286
60.51	20	ATGTTGGTCCAAGTCCAAGC	59.973	20	184	280	463
60.523	20	CCATTTTGTGCCCTTGATTT	59.801	20	190	91	280
60.359	21	GAGATGCGAGCTGATGTTGA	60.104	20	212	55	266
60.134	20	TTCCGAACTTCATCCTTTGC	60.192	20	249	581	829
59.13	23	TGACTCACTATGGGCACAGC	59.862	20	108	375	482
59.682	20	TGTATGGTGCTTGGTGGTTG	60.427	20	188	35	222
60.111	20	CGGTTTGTTTTCGTGTTTGA	59.603	20	216	451	666
59.194	23	TGCCTACGCATCAAGACAAC	59.871	20	204	1	204
59.834	20	CGAGGAGAGGAGAGACATCG	60.088	20	241	42	282

59.154	20	AACCGGAAAAGTGGAAGAAAA	59.967	21	192	348	539
59.973	20	AGGTGGGCTTAGTTGAGGTG	59.208	20	213	19	231
59.745	20	TCAATCAAACCAACGAAACAA	59.048	21	212	645	856
60.198	20	TCAATGTAAATGCCAATCTCTTTC	59.525	24	197	298	494
59.937	20	TTCTCAGCATATGGGAAGGG	60.029	20	239	759	997
59.499	20	TTCTGAGGATGGAAATCAACA	58.131	21	277	2117	2393
59.955	20	TGGCAGTAGATTCATTCTATCTTTT	58.871	26	250	7	256
59.971	20	AGTCACCACCACTTTCCTGG	60.002	20	274	56	329
60.096	20	AATCTCTGCCACCTTTCCCT	60.074	20	164	650	813
57.101	25	CACAACTCCACCTGACATAATCA	59.906	23	172	547	718
60.578	20	GATTATGGCATACGTGTGGG	58.723	20	151	389	539
58.71	25	CCATTATGTCGATATCCATCCA	59.507	22	233	105	337
59.756	20	CCTATGGTTGTATGGTGTGGAG	59.238	22	113	98	210
59.904	20	ATTCTGAACCAACTGGCACC	59.973	20	274	1695	1968
60.032	22	GAAAAGCAAGGAAAAAGCCA	59.451	20	260	2819	3078
60.177	21	AAGACAACAGGCCAACTTGC	60.299	20	252	5328	5579
60.263	20	ATTTGTGTGGCCATTTGGTT	60.096	20	183	96	278
60.037	20	AAGCAAATTCCAAGATGACGA	59.699	21	274	4639	4912
60.249	20	CAACCCTACCTCCTTCTCCC	59.928	20	117	953	1069
60.844	20	ACGTGGCCTTTGATGGTAAA	60.365	20	279	69	347
58.925	20	AACACTATCACAAGGTTCCGAGA	60.047	23	236	70	305
59.834	20	TTTACTTTTGAGGTGGCGCT	59.883	20	140	4182	4321
60.125	20	CCTTCTTGGGATGTAGGGGT	60.183	20	171	446	616
59.494	22	ATGTTGTGAAGCACATGGGA	59.967	20	220	1008	1227
58.891	20	CAAATCCCTCTTTTCTTATTCTCAA	59.236	25	250	174	423
59.975	20	CCACGGATTACTCTGGGAAA	59.926	20	277	599	875
59.989	20	TGCATTTATCAGCTTTATCAGCA	59.895	23	223	549	771
60.538	20	GCCAAATTAAAGACCATATTTTCA	59.68	25	215	2643	2857
59.41	20	TGCATCCATGATATGGCG	59.988	18	216	384	599
60.073	20	TGTAAACCGACCTCCCACTC	59.966	20	167	1832	1998
59.691	19	ATAGGCTCCCCCGATCATAA	60.623	20	231	1306	1536
59.45	20	GGGAGAAAAGGGGAAATAAGTG	60.173	22	220	50	269
60.02	20	GATGAACTCTCTTGGGTGGG	59.505	20	113	262	374
59.853	20	CTCTCATTCCGGTGTGGATT	59.927	20	246	107	352
59.397	27	TTCTCATTTCCCAAATGTCG	58.546	20	277	488	764
60.17	26	AAGGGTGTTTTCTCTGCATCA	59.726	21	251	31	281
60.502	20	ACGAGAGAGCGAGTTGGGTA	60.012	20	179	321	499
57.922	21	TCTTCGCATGCTTATTTGGA	59.401	20	196	41	236
59.867	20	ATAAAAATGGCAGACGGCAG	60.096	20	254	528	781
60.004	21	ACCGGGATAAAATAGGGCAA	60.508	20	235	1630	1864
60.359	21	ATTATCATATTTAAGGGTTATCAC(	57.31	27	135	330	464
57.94	23	TTTCACTCTTCACGTTTGCAC	58.963	21	182	10	191
60.059	20	AAAACCCTAAACAACCAGTGAA	57.717	22	252	49	300
59.856	20	TCAAAATTGCGTCATTGCAT	60.081	20	200	247	446
60.193	25	AACAATACAATCCCGTGCAA	58.895	20	246	3	248
58.114	23	TTGCAAAAGTCACACTCTGTCA	59.534	22	248	52	299
59.73	20	TCTCCTACAACAACCCTCGG	60.103	20	233	192	424
59.821	20	TAGTTTTGCATGCATTTGGG	59.562	20	148	8	155
60.935	20	TAAAGGATCACGTTGGGAGC	60.074	20	265	168	432
58.82	20	CCGTTTATTTTTCACACGTTCA	59.904	22	177	1118	1294
57.051	25	GCAACGGATCTGATTTGGTT	59.939	20	241	31	271
60.169	20	GAATCGCAGAACCGATGATT	60.043	20	270	1247	1516
60.348	20	TCCTTATTGTTGGGTGTGGG	60.605	20	150	987	1136
59.709	21	GATCTAAGGGCCGAGATTTG	58.74	20	101	171	271
59.938	20	ATTCGAATTCCTAAGCGCAA	59.816	20	109	251	359
60.088	20	GTGGTACTTGCACGCACATT	59.646	20	261	62	322
60.374	20	TAGTCATGCGGGCTACATCA	60.243	20	220	312	531
60.21	20	TGAGGTGAAGTGTGGAGTGTG	59.766	21	180	28	207

60.237	20	GAGCCACAGCTACAGGCAAG	61.136	20	267	29	295
59.691	20	TCTCGGTGAGCGAGTAGGTT	60.012	20	117	5	121
60.223	20	TCCATTTATCGGAGTTACGGTT	59.737	22	207	249	455
59.009	23	CCTCACGCACTCCACTAACA	59.897	20	233	8	240
59.824	21	AATTGATGGAACGGGGATTT	60.378	20	263	83	345
59.875	20	TTGTCACATTTTTGGTGTTTCA	58.964	22	249	1609	1857
59.923	20	GGGATTCTTGATGAATGGGA	59.685	20	133	40	172
59.966	20	CAACGCACGGGTAAAAGACT	60.168	20	144	1422	1565
59.806	27	AGGCCTATAAATGCGTTTGG	59.101	20	228	34	261
59.91	20	ACAGACATCGGGAGAAGGTG	60.112	20	198	2602	2799
60.111	20	CATGCAAGCAGCACTTCAAT	60.019	20	278	109	386
58.616	23	AGGTAGCTGGGAGGAGGAGA	60.349	20	186	16	201
60.21	20	ATTGGCCGTGAATTTTTCTG	59.938	20	261	137	397
59.364	20	TCATGAAATTGTTGGGGGTT	60.029	20	192	195	386
59.873	20	TCTTCACCTATTATTTCAACAAGA	57.674	26	156	38	193
60.473	20	TCTTCGAGTACGCTCTGGGT	60.012	20	178	48	225
59.83	20	GTGCGTGACGGTTGGTATAA	59.469	20	280	64	343
59.959	23	GTTTTTCTCCCCAGTGACCA	59.943	20	234	15	248
59.79	20	ATAAAAATGGCACCTGGCTG	59.96	20	238	19	256
61.021	20	ATTTAGGGCAAGAGCCCATT	59.93	20	186	343	528
59.308	20	CCGATGTTTTCGAGGATGAG	60.594	20	266	204	469
59.78	20	TCGGCTACTTTGAAGGGAAA	59.817	20	237	228	464
60.319	20	TCTCGTGTTTGACCGTAATCAT	59.499	22	144	1015	1158
59.823	20	CAGAAATGCCACAGCAGAAA	59.988	20	186	861	1046
59.458	22	GAGTTGGAGTTCGGGATCG	60.606	19	278	2987	3264
59.019	20	GCTTTCACGGGAGAAAGTTG	59.853	20	252	2642	2893
60.758	20	TGTGTGGATCAATAACTGTCAGAA	59.54	24	209	37	245
59.89	20	TTAAGGTTGGCTTGGGTTTG	59.968	20	185	2254	2438
59.249	25	TGTAGTGGCTTCAGCTCGTG	60.199	20	194	41	234
60.021	22	TTTAAGAGATAGTACCGGATCGC	58.853	23	215	42	256
59.232	20	GAGGTCGGGTTCGTGTAGAG	59.721	20	128	16	143
59.966	20	CCACTCTTTTGGGGCAATAC	59.429	20	232	31	262
60.11	22	CCACGCATGTTTCTGTCATC	60.12	20	128	920	1047
59.476	20	GGAAACCACTCACCGTGTCT	60.009	20	228	383	610
60.103	20	CGATAAAATGGCACACATCG	59.953	20	248	1046	1293
60.011	20	AGCTTTTCACGCATTTCCAC	60.257	20	101	25	125
60.331	20	TGCACTGCATAACAAGCGTA	59.086	20	216	50	265
59.633	20	CGTATGAGCTCTGAGAGTGAGTG	59.315	23	233	1002	1234
59.989	20	CGTGTGTGAGTCCCCATGTA	60.44	20	166	231	396
60.074	20	TCCAACATCCAACCTCCATT	60.173	20	220	624	843
59.533	20	TGGTGGGGCTTAATTAGGTG	59.817	20	277	1318	1594
60	20	CACATTGGTAGAAGGCCGAT	59.955	20	202	434	635
59.007	23	GCAGGATAAGGGGTGTTGTT	58.912	20	116	2	117
59.971	20	CTGAACCGAACCGAAATTGT	59.971	20	205	724	928
59.795	20	TCTTTGAGGTAGGGCTGGAA	59.807	20	248	466	713
60	20	GGAGCTCCCTAAAGCATTCC	60.173	20	241	448	688
60.46	20	GCCATGACCAACTTTCTTTTC	58.684	21	106	26	131
59.986	20	TCTTTGGGAAGGAGGTTGTG	60.081	20	137	928	1064
59.331	20	TGCTTCTTGAAAACTTTCTTCATTT	59.84	25	213	866	1078
59.637	22	GGTGACATGGCTGGATTTCT	59.934	20	146	294	439
60.05	23	AATTGCTTCAATGTCACCCC	59.797	20	272	117	388
60.009	20	TGGAATGTACTGCTGATGTGG	59.581	21	201	0	200
59.973	20	TGGAAAAGAATTCGTCCTCG	60.184	20	223	77	299
60.309	20	TGCATACACGAGTGGAAAGAG	58.937	21	215	449	663
59.996	20	ATGACGAGAAAATGGTTCGG	59.933	20	136	425	560
57.866	21	CCTTGTAGCCCCTTTGAGC	59.822	19	280	111	390
60.049	20	CTTGTTTAGGGAGAGCACGC	60.015	20	250	1868	2117
57.436	27	GCAAGTCGCTCTTCTCGTCT	59.898	20	196	18	213

60.152	20	CTTTCTCTCTCCCGCGTATG	59.971	20	278	203	480
59.734	20	CCGAATTTCTTTGCACAGGT	60.11	20	275	11	285
59.816	20	TGAAATGAAGAAAAAGAAATTGG	57.512	23	150	96	245
60.145	20	TGGAGAAGGGACAAATGGAG	60.042	20	182	191	372
60.326	20	ACCAATATCCAACGGCAAGA	60.331	20	183	1278	1460
60.096	20	TTTCAAAATTCCCCACAAGC	59.916	20	219	2716	2934
60.736	20	TGAAGGTTGGAGGAATGGAG	60.042	20	276	146	421
58.275	22	CAGCTTGAAGGGGAGTCTTG	59.982	20	250	0	249
60.282	22	ATGTAGTGTGTCACGGGCAA	60.032	20	258	590	847
59.901	20	TATGGGTTATCACCGGATCG	60.544	20	259	641	899
59.939	20	TGAAGTTAGGAAATAAGGAGGTG	58.743	25	169	352	520
59.955	20	AATTGTATTCTCCCGAGCCC	60.286	20	177	636	812
59.924	20	TTGTAGCCGTTATAGTGTGGGA	59.536	22	265	12	276
59.88	20	CCTGTTCAGCTATCTTTTGCG	60.025	21	278	55	332
60.382	20	ATGGGTTAGCGTGTTATCGC	59.988	20	120	177	296
58.892	20	ACACTACCCTCCTCGTCCCT	59.991	20	211	72	282
58.097	22	CAGGTAATGCTAGCCGGAAA	60.223	20	197	380	576
59.792	20	AATCTTGCGAACCAAACACC	59.978	20	118	20	137
59.899	21	TGCGAACAACAAGAAACAGG	59.881	20	207	2457	2663
60.074	20	GGAAATAAGGAGGTGTTATGATA	57.235	25	225	94	318
59.951	20	TGGGTTATTTTGGCCCATTA	60.012	20	262	17	278
59.934	20	CTGCAAAATTGGCTGAGTTG	59.464	20	101	709	809
60.34	20	ACGTGTTGGATGGAATGCTA	59.002	20	153	79	231
60.011	20	AGCTCTCGTCAGCAGTTGGT	60.205	20	218	770	987
58.293	22	AGATGAATGAAGTACGGCGA	58.326	20	162	0	161
60.307	20	GGTTATCCTGTGGAGCGTGT	59.997	20	226	429	654
59.763	20	TTGTTTTCTATTCTTTATAATTGCC	57.667	26	275	6	280
60.713	20	TTGCAGTTGCAGTTGGTGAT	60.31	20	185	821	1005
60.175	20	AAGGGCACCGCTAATGTAGA	59.73	20	145	80	224
59.787	20	GCTTTGTCGGTTTCTCCATC	59.676	20	186	52	237
58.116	24	CCAAGTTACTCCCAGCGAGA	60.388	20	116	1	116
59.967	20	GCAAGAAATGGCTCCCTACA	60.214	20	260	794	1053
59.626	22	TGAAGGCCCAACCTTAAGAA	59.679	20	224	4	227
59.638	20	TGAAGTCACAGCGAGGTTTG	60.025	20	110	2	111
59.586	25	TGACGGTCAAGATTTGCTCA	60.39	20	130	1	130
59.836	20	TCCATCCTTCTTTGCTGCTT	59.955	20	179	631	809
59.697	20	TTAACCTCCCGTGGACGTAG	59.986	20	181	372	552
60.467	20	TTTTTGGCAATTTGTGACGA	60.088	20	202	39	240
59.157	20	TCCAATGGATGTGAATGGTT	58.617	20	274	67	340
58.527	21	CCTGCTTTTGTGCATAGGTT	57.906	20	175	253	427
59.672	25	AAATGATAGGGCACACAGCC	59.962	20	182	83	264
60.111	20	AAGTCGCGCAAACTTGAGAT	60.022	20	178	3813	3990
60.173	20	AAACCTGGCTGGACTGATGT	59.579	20	213	1763	1975
60.169	20	AAGTGAAACTGCTGTATGCTGAG	58.737	23	248	472	719
60.011	20	ATTTGGGGATGAATCTGACG	59.75	20	212	11	222
59.152	20	CTCGGTTGAGCTCGGATAAG	59.971	20	183	71	253
59.982	20	TAGCCTGGTCGTTTTGGAAG	60.241	20	266	1002	1267
58.268	24	TCAACGGTTTTGATTTGTTCC	59.831	21	182	73	254
60.126	20	GTGCATGGTAGGGCAAACTT	60	20	270	1540	1809
60.059	19	TGCGTTTTGACAATATATAGGTGT	60.083	26	103	292	394
59.763	20	ATGGCTGGGCTCTGAAATTA	59.668	20	241	351	591
59.931	20	GCGAATTCAAACCCCTTGTA	59.938	20	227	8	234
60.177	20	TAACACCACCACCCACACAC	60.178	20	115	1762	1876
59.948	26	TTACAAATGGCCAAGAGAGTTT	57.94	22	102	8	109
60.066	20	TTTTTGACAGCGAAAGTCCC	60.227	20	242	4197	4438
59.959	20	TTTCCCCTTTGCTCTCTATTTC	58.887	22	272	138	409
60.035	21	CCGAAGATCCGAGATCAGAA	60.295	20	209	35	243
60.119	20	CCAACTGAAGAACACGAGTTTG	59.813	22	269	1208	1476

59.542	20	GGCAATCCATTCCGTACAAG	60.331	20	249	21	269
59.157	20	GATCGACGCCTCAGATTTGT	60.226	20	169	948	1116
59.95	20	CCTCTAGCATCTCTTGCTCTCA	58.983	22	270	255	524
59.01	21	AGCAAGATTGATCCTCCTCG	59.386	20	277	21	297
59.759	20	ACAGGGTACTCAGTGGACGC	60.182	20	209	17	225
59.411	22	ATTTCCACGAAGCATCATCA	59.079	20	269	239	507
60.236	20	TTTCCAACTATCCAAACGGC	59.938	20	250	634	883
57.771	26	CACACTATTAGGGTTTGGGAGG	59.757	22	116	4	119
60.294	20	CCCCGAAAAACCCTCTATCT	59.415	20	254	2290	2543
59.6	21	CGAGCTCGGACCTTCAGTAT	59.454	20	112	21	132
59.811	21	AGGGCAAAAATAACCAACCC	60.055	20	272	83	354
60.083	26	GACCAAAAAGAGACGACCGA	60.232	20	121	1	121
60.006	20	ATCACATGGTGATGCCTTCT	57.939	20	215	28	242
59.928	20	TGGTGGCTTCATTTCCTGTT	60.495	20	210	549	758
59.478	21	TCTCGTGTACAAAAGCCGTG	59.904	20	109	0	108
60.104	21	TAAAACCATGCACGTAGGCA	60.133	20	268	0	267
57.425	21	GGGGTCTTGGCGTACTAACA	59.993	20	106	17	122
59.621	20	AAGGGATGTTTGCACCTGAA	60.495	20	161	50	210
60.199	20	TGAAGTTAGGAAATAAGGAGGTG	58.743	25	156	953	1108
59.476	20	GAGTTTTCATCTTACCTTGAGGG	58.333	23	210	46	255
58.388	25	TTGTGGGGTTGTGGTGAATA	59.667	20	187	35	221
59.034	20	TGGCCGGAGTTTTAGGTATG	59.953	20	266	43	308
60.118	21	GTTTTTAAAGAGCTGGGGGC	60.075	20	186	981	1166
59.617	23	GCAAAACAGCCAGAGGATTC	59.82	20	203	0	202
60.012	20	TAATTGAATTCCTGGGTCCG	59.756	20	265	899	1163
59.395	21	TGGCTATTTTGGTTTCTACAACA	58.731	23	236	64	299
58.976	21	TATCAGGCTTGATGATGGCA	60.182	20	266	228	493
60.377	22	CGCAGCAGGTCAAGATTACA	60.011	20	267	535	801
58.4	21	TTGAAGCATCGTTTGTGTTTTC	60.152	22	272	6	277
59.608	22	TGGTTTATATTTCGTTGATTTGC	58.141	23	258	31	288
59.229	24	GGGTGTTCATGGTTCGATTC	60.181	20	274	31	304
59.763	20	TCGAGTCCAATGTTGAAAGAAA	59.724	22	279	439	717
59.962	20	TCGATGAAGGTGACGAAGTG	59.831	20	232	22	253
59.667	23	TCCTTTTTGTCATTCACCTTTG	59.108	22	232	488	719
59.532	20	TCTTGGACTGCGTTAGGGAC	60.255	20	199	188	386
59.368	20	AATGGACCGGTGAAAACAAG	59.83	20	264	565	828
59.822	19	ATTTGGAGGAGGATGGGTTT	59.629	20	187	2432	2618
60.033	20	CTTCCTTGGGCACAACATTT	59.971	20	232	716	947
60.192	20	ACGTGAAAAACGGGAATGAA	60.344	20	189	113	301
60.299	20	TCACACAATTTCACTCTTCACTCT	58.044	24	169	97	265
59.786	20	TGGACTTCGTCACAACCAAA	60.128	20	124	269	392
60.073	20	CCATGAGCCCCAGTAAAGAA	60.066	20	257	3075	3331
61.296	20	CCCCCTCGTGTTTCTTGTAA	59.964	20	197	23	219
60.032	20	AAAAACCGCCACATCAGAAG	60.11	20	268	1879	2146
58.624	27	ATTTGACCATGGTTTTGGGA	60.029	20	223	18	240
59.821	21	TCAAACACGAGTAGAAAGGATTT	57.147	23	161	66	226
59.956	21	TCTCCTTCACCCATTTGGAG	60.042	20	126	815	940
57.426	20	AAATTTGGGCCCTTAGATCG	60.272	20	191	56	246
59.951	20	TGAAGTTAGGAAATAAGGAGGTG	58.743	25	188	136	323
60.096	20	TCTGATTCTGGGATTTTGGC	60.014	20	266	573	838
58.677	20	GAACTCTCATGCCCTGGAAA	60.195	20	160	113	272
60.08	20	CATTATTTAAAGCACGCGCA	59.872	20	216	452	667
58.028	22	CAAAAATGGATGCAAATACGG	60.192	21	106	14	119
59.813	20	GCTGGAACGTGCCATTTACT	60.14	20	140	19	158
60.088	20	TGCTCTTCACCTTTGTGTGC	60.032	20	132	529	660
59.485	21	TTAATCGTCCATTTCGTCGG	60.827	20	270	241	510
60.831	21	AGGGCCTCTTTTTGTCTGAA	58.909	20	206	10	215
59.976	20	GTAGCTGCACATTGTCCGTC	59.323	20	270	75	344

60.111	20	GATCACCATCACCATCCTCA	59.269	20	153	1389	1541
60.537	21	CCCTTCAATGGTGTTTCCTG	60.345	20	209	357	565
59.555	20	TAGGGGTCAGTGTGGGTTGT	60.275	20	173	299	471
60.096	20	CAATGTACCCCGATGTGAGA	59.369	20	278	764	1041
59.239	20	TGAACACGTTGCCAAAACAT	60.011	20	120	76	195
60.039	20	TTAAAAACTCGACCCGATGC	60.074	20	146	143	288
58.815	22	TCAAATCAGGAAAACCCCTT	58.477	20	201	55	255
58.687	21	TGTTTTTGGAGGGAAAATGG	59.772	20	122	38	159
60.495	20	GGAAGGGCTCAACAACACAA	61.075	20	224	0	223
59.799	20	CATGTTTTGTTTGGCTGTGG	60.004	20	277	194	470
59.72	20	TCGGTTAGAAAATGGACCGT	59.429	20	280	11	290
59.24	21	GGGGTGGGAATGATGTGATA	60.404	20	214	69	282
60.088	20	TTTGATTTGTGTTGGCAAGG	59.561	20	107	360	466
60.203	23	ACCACGAGATGGAGACCAAC	59.969	20	219	410	628
60.11	20	GCTGAATCTTAACCGTTCGC	59.851	20	221	80	300
59.587	22	TTGATACAGATGGTACGCCG	59.566	20	275	47	321
59.964	20	TCAACAGAGAATGCAAGCAAA	59.6	21	217	89	305
60.046	20	ACGGATATGCCCTTGTATGC	59.813	20	227	43	269
60.206	20	AGATGGCGGTAAACATGGAG	59.955	20	232	116	347
57.615	23	CCCTAATTGAGTAGAAAACCACA	57.399	23	263	1341	1603
59.072	22	TGCTGACTTGATTTTCGAGC	59.152	20	187	0	186
58.703	20	TGATACGACCTTGGGGGTTA	60.184	20	224	2	225
60.144	20	CACCCAAAAAGGTTATTATTTGA	57.25	23	279	308	586
60.235	20	GACCGAGTTGGTGGAGTAGG	59.574	20	233	2591	2823
57.804	20	GGTCTTTCTTTTTCCACGCA	60.227	20	185	485	669
60.127	20	TTGGTTATCGCTTGAACGAG	58.917	20	247	6	252
59.154	22	GCGTTTTGACAGGATTTGCT	60.257	20	103	4	106
59.2	20	TCTCAAAAGTCTCATTTTCACAGG	59.799	24	257	2269	2525
60.309	20	ACACCTTGTCTCCCAAATGC	59.973	20	218	2314	2531
59.962	20	CTCGATCTCCATCACCACCT	60.072	20	235	1654	1888
59.969	20	CCCTCGGATGCTATGCTAAA	60.188	20	182	2434	2615
58.787	25	CTTACAAAATCCGCCGTGTT	59.996	20	257	14	270
60.082	20	GACCGAAGCCACCATATCAC	60.348	20	145	316	460
58.121	22	TCTCTCCAACTCATTTCGCA	59.522	20	188	165	352
60.088	20	CCCCTTAAGTGTGAACGCAT	59.993	20	253	499	751
59.341	21	GCTGGAATACAACTACCCCAA	58.962	21	272	1439	1710
60.263	20	CAAATTTGAGAGGCTTTCGAC	58.962	21	191	2077	2267
58.188	20	TTAAGTTTGGGGGTGCAGAG	60.103	20	104	212	315
59.438	21	TCAAACTTACCGTCTTTGTCCA	59.649	22	239	13	251
59.882	20	CGAGTAGAGAAGGGGTGGGT	60.502	20	181	3	183
61.031	20	TGGCGGACACTCATGTTTTA	60.111	20	116	18	133
59.823	20	TTTGTCACCCTCTCCCAGTT	59.549	20	264	711	974
60.111	20	TTTTCTGACAAGGGTGGGAC	59.943	20	274	1156	1429
59.853	20	CTCGTGCTTGTCTTCCACAA	60.025	20	216	1135	1350
58.447	26	AATTCGAACCTGGGACCTCT	59.935	20	137	622	758
59.992	20	TCCACAGACAGATGGAGCAG	59.98	20	248	335	582
60.154	20	GCAATGGAGTGGAGGAGAAG	59.803	20	140	156	295
59.676	20	CACACAGAAGTTGTGGGAGG	59.148	20	161	13	173
60.199	20	TCCTTTGAGGATGGATTGATG	59.879	21	204	91	294
60.192	20	AATTGGTTTGCTTGGTTTGC	59.982	20	271	101	371
59.805	20	TTTTTCCGAGCAAATGAAAA	58.394	20	239	1103	1341
59.184	21	ACCACCTAGCAATGGTTTCC	58.912	20	264	5	268
59.493	20	AATCCGTATTTTTGTCCCCC	59.888	20	176	690	865
60.142	20	TCGAAGTTTGGTGTTGGTGA	60.128	20	199	78	276
59.813	20	GCAATGTGCTTCCATCTTGA	59.805	20	187	954	1140
60.038	21	ACCTTGACGAGGTGGTTGAC	60.009	20	258	24	281
60.192	20	TCTCTCTTGGTTGGTGAAGAAA	58.955	22	195	153	347
60.003	20	CCATAAGAAAGTGAGCCCCA	60.066	20	260	554	813

60.614	20	CGTTAGGTCCGTCTCGATTT	59.195	20	126	44	169
58.278	20	ACCATGAAGCACATCACAGG	59.551	20	236	1825	2060
59.979	20	TGACAAGCAGGTAGACCACG	59.897	20	214	18	231
59.049	20	TTCCGTCTACCCCTTTTCCT	59.934	20	176	839	1014
57.628	22	TCACCTCCTCTATCGGAACG	60.21	20	219	0	218
60.177	20	AAAAATAGAGTTATCACAGGATC/	58.199	26	134	217	350
60.224	20	CACCCCGGGCTAGATATTTT	60.164	20	261	4162	4422
60.298	20	ACCCCTTAGGGCAAGGTAGA	59.955	20	268	28	295
59.997	20	TATGGGTTATCACCGGATCG	60.544	20	246	768	1013
60.362	20	CTAATGCCCTCCAAAACCAA	59.931	20	243	361	603
59.972	20	CGCTGTAGATCTAACGGCTGA	60.549	21	259	321	579
59.866	20	CCTTCCATGGCTTTGAAAAA	60.046	20	222	585	806
60.448	20	GAGGCAAATTATTCGGATTTTC	58.99	22	230	1196	1425
59.972	20	CCCCATCAACCTCCATACTG	60.187	20	241	1211	1451
60.422	24	CCTAGCCAGGAACATACCCA	59.948	20	146	41	186
57.858	27	GATGATGATGATGCAGGTGG	59.882	20	271	3	273
60.063	20	CAGCTGCTTTGTTTCCATCA	59.988	20	240	149	388
60.255	20	TCAGGTCCACGTATTTTCCC	59.79	20	117	441	557
57.73	23	GCTTCTTTGCCGATGAAGAG	60.096	20	269	18	286
60.096	20	GGGTTATTACCTGATTACCTCCC	59.052	23	154	338	491
58.78	20	CTTCTAACGAAGCCCAAACG	59.876	20	267	82	348
59.73	20	GCTTCCACTGCCTACTTTGC	60.022	20	245	176	420
59.967	20	AATTTGGCAGCTTCTTGTGC	60.395	20	112	77	188
60.665	20	CCAGATGCAGCATGAGAGAA	60.096	20	209	693	901
58.982	20	TCCACCGTGGTTAGTTGACA	60.001	20	238	109	346
59.42	20	CTCAGCTCAGGGTGGAGAAA	60.523	20	230	167	396
58.486	22	CATCTGAGGAAGGCACATGA	59.787	20	153	59	211
59.264	20	ATAGTTGGTGTGAGACGGGC	59.997	20	200	159	358
59.155	20	CACAATATTTGAGGGTTATCATTT(	58.787	25	195	466	660
59.886	20	TGTGCGTAGAACAGAGAGCG	60.347	20	268	9	276
59.92	20	TAGAGGACATCGCGGAGAGT	59.973	20	172	44	215
59.84	20	GGTAGGATTTCAACGGCGTA	59.96	20	219	1168	1386
59.853	20	TGAAAAGAAAAGAGGCACTCG	59.618	21	212	63	274
60.277	20	GGTGAAGACGGCTTGTTGAT	60.119	20	161	742	902
59.81	20	ATTTCGAAGATCAACGGACG	60.074	20	137	97	233
60.235	20	TTCAAAGGCCGAGATTTGTT	59.685	20	262	23	284
59.415	20	CAACAACCTAATCATCCAACCA	59.724	22	224	230	453
58.97	23	CCACCAACTTGTCCACTCCT	60.002	20	248	1982	2229
59.946	21	GGCTTCACATTTCCCATCAC	60.326	20	228	52	279
59.109	26	TTTTTGCGAGGGAGAATGTC	60.192	20	125	0	124
58.896	24	CGGACGATATCGGTTATTGG	60.167	20	123	0	122
59.946	20	ACTCCTGGTGAATGGCAAAG	60.111	20	199	22	220
59.545	20	TTCGGATCTTCACATGGTCA	60.048	20	236	488	723
59.997	20	TATGGTGGTATGGTGTGGGG	61.296	20	221	179	399
59.803	20	CTGGTGTCACAACGGATGTC	60.005	20	194	40	233
59.875	20	TGCAAGAGCAGCAGGATATG	60.119	20	216	275	490
60.029	20	CCGCCTACTCTCCCTCTTTT	59.842	20	240	37	276
59.107	24	GCGAGCATCATACACGAGAA	59.979	20	254	2436	2689
60.05	20	TTCTCAGTGCATCCATTTCG	59.799	20	205	750	954
58.279	21	GGAGTTTGCTGTTGAGACCC	59.703	20	229	332	560
60.173	20	AGTCGCTGCAATTTCCAGTT	59.882	20	112	3	114
59.222	20	AGCCTAAACACACACACCTCA	58.285	21	103	105	207
59.85	22	GCAAGGGGCAAGTGTTATTT	59.091	20	223	513	735
60.34	20	ATCACATGCCATTCAGTCCA	59.925	20	234	1794	2027
60.431	20	AGCCGTTGTGGGATATCTTG	59.955	20	125	1117	1241
60.559	20	GGCTAATATAATTTATCTCCATGC/	58.976	26	215	235	449
59.967	20	TGGCACTACTGTGAATTGGG	59.566	20	257	5728	5984
60.016	20	TTGAGAAGTCAGAAGCGGGT	59.989	20	109	778	886

60.111	20	GAAAGCCTACTTGGCGATCTT	59.87	21	142	413	554
60.154	21	GTTCGAAAGCCTACCTCGCT	60.894	20	186	5	190
58.992	20	ATGGGAAGAGGGGGTATGAG	60.147	20	110	2109	2218
59.912	20	GCAATTAAAACCCCCTCGAT	60.15	20	248	285	532
60.156	20	TCTCTCTATCTTTTTGGTTGGTGA	59.304	24	232	4	235
59.404	23	CTCAGCATACGGACGCATTA	59.856	20	192	12	203
59.679	19	ACTGTTGGACGGTTATCGCT	59.621	20	151	87	237
59.938	20	ATGCGCGATTTTAAACCCTA	59.586	20	214	31	244
59.566	20	CCGAAAATCTCCGTCAAGAA	60.184	20	192	258	449
60.215	22	GGTTTGGGTTTAATTTGGGC	60.4	20	229	8	236
60.137	21	TCACTTCGAACCTTCTGCAA	59.566	20	185	7	191
59.746	20	AATTCATTCAAGCAGCAGCA	59.572	20	269	98	366
59.823	20	CCAAAATTTATGGTTATCACAGGA	59.191	24	172	79	250
57.388	23	CCCATGTGGGGAGATATGAT	59.44	20	191	801	991
59.839	20	TTTTGTGGAAGATGCAGGTG	59.691	20	173	2903	3075
57.222	23	TCGCATGCCACTAAAAATGA	60.215	20	213	20	232
59.971	20	CTCAACGGTGAAGTCGGATT	60.111	20	194	204	397
59.934	20	AATAGAAAAACCACGCAGGG	59.087	20	179	159	337
60	20	GGCGTCTGGTTTTTGTTTGT	60.015	20	259	15	273
59.716	21	AAAATTTCACATATTTGATCGGAA	58.838	24	192	34	225
60.69	20	ACAATCTGGGGCGTACACAT	60.263	20	229	82	310
60.142	20	CGGGGGTAAGAAAGTCAACA	59.964	20	264	938	1201
60.537	20	GGCTGCCAGAATAAAAAGGA	59.297	20	278	497	774
60.103	20	TGGTGATGCCAGAACAGAAG	59.831	20	231	279	509
60.081	20	ATTTAGGGGCTTTTGAGCGT	60.095	20	268	772	1039
59.586	20	CAACGGTTTTGGTTTGTTCC	60.241	20	152	76	227
60.126	20	GTTTGGCTGGTGCAGAGATT	60.263	20	220	370	589
60.119	20	GACTCACGGTTTTTCGGAAG	59.711	20	262	475	736
59.701	20	TGTTCGGAAGATTGAACGTG	59.691	20	212	94	305
60.275	21	TCGGACCAAATTACCCTTCA	60.301	20	254	396	649
60.025	20	TGAACAATTGAACACCGGAA	59.941	20	237	7	243
58.903	20	CGCGACAGAGTGGTATCAGA	60.008	20	253	369	621
59.113	23	GGTGAATCTGTAAAAGGTCATCG	59.885	23	235	337	571
60.247	20	TTGTTTTGGTTGGCAATTCA	59.946	20	264	67	330
60.171	20	TTGTGGAGAACGAACTCGAA	59.415	20	207	262	468
59.637	25	ACATGTGCTGTGAGAGTGGG	59.738	20	263	500	762
60.061	20	AAAAACCGTTCCACATTCAAA	59.348	21	166	1271	1436
60.142	20	TCAAACAGAACACACCTTCCC	59.997	21	105	177	281
59.22	25	CAACCGCATAGGCACAGTAG	59.366	20	121	15	135
60.053	20	GCAAATTTCTTCGAGACCCA	60.192	20	111	174	284
60	20	CAGTTTGACATCAGAGCCGA	59.984	20	150	41	190
60.055	23	TCAATTTGAATGGTTATCACCTG	58.865	23	116	230	345
59.429	22	TTAACGACTTGAACCCATTTTT	57.74	22	279	305	583
58.444	20	CTAGCTCCTTCCCCTTCACC	60.204	20	111	21	131
59.966	20	CCAACATGAAAGGCCATGAT	60.723	20	202	408	609
59.939	20	TGTGATTTCTACCATTTATTCTTTG	59.708	26	243	5	247
60.066	20	AACATGTTCTTGACGCACCA	60.16	20	153	50	202
59.172	21	TTGTTTGCATCCCACACAAC	60.413	20	158	264	421
59.816	20	GAGAATGTGAGACATCATCATCA	59.059	24	152	228	379
59.601	23	TTTCACAGAACACAACGATCA	57.72	21	260	10	269
60.298	20	TGATGATGAAATGACATGGAAA	58.82	22	131	81	211
59.606	21	AATAGGAGAACCAAACCGCA	59.569	20	220	1781	2000
60.408	20	ACCACTCCCCACTGCAATAA	60.375	20	164	297	460
60.111	20	CAACATAAATGGGGCGTAGG	60.202	20	103	1099	1201
60.695	20	GTGTTCTCGTGAGAGTGCGT	59.051	20	204	6	209
59.083	20	TTCCAAACCTTTGTTCCGAG	60.081	20	230	821	1050
58.935	25	GGGCCATTGGTTCTGTATGT	59.676	20	115	4	118
59.964	20	TGATTAATATCAGGGGGCGA	60.247	20	207	10614	10820

59.971	20	CAACGACTCCGATCACTTCA	59.831	20	276	883	1158
61.84	20	GAGACGATCGAACAGCATGA	59.95	20	278	0	277
59.862	20	AGGCCTTGATGTCCCTTCTT	60.074	20	205	110	314
59.873	20	AGAGGAGTGACCTTTCCGGT	60.111	20	248	249	496
58.955	20	GATAGGGGCACACCAAGAGA	60.073	20	257	17	273
59.971	20	TGATACACGAGCGTGGAAAG	59.864	20	126	217	342
59.779	20	ACGACAGTGGACAAGGGAAT	59.43	20	231	88	318
59.162	22	TGGGCTGAAGTATGTGTGGA	60.112	20	273	526	798
59.315	20	TCTGTTTGCCTTTCGAATCC	60.192	20	271	418	688
59.82	20	GCAGACTTTAAGGCAGCCAA	60.523	20	268	5296	5563
59.168	21	CGTGGTTCGATCGTAATTGA	59.542	20	208	373	580
59.996	20	TGCTTCCTTTTGCTGCTACA	59.752	20	275	215	489
60.05	20	GGTTTTCTCGCAATCCACTT	59.174	20	244	2270	2513
59.962	20	TGCAGGTACCTTCGGAACTT	59.734	20	272	49	320
59.624	20	TAGGGGAAAAGGGGAAAGTG	60.284	20	152	242	393
57.694	20	ACGCAGTCTAGAGATATATGTTGA	57.442	26	122	420	541
57.088	20	GGTGCCCACCAAATTTATGA	60.564	20	203	659	861
60.859	20	CTGAGCTCACTCGCCTTTCT	59.891	20	199	1499	1697
59.872	22	TTGTCATTGGGCATCGATTA	59.891	20	241	47	287
59.17	24	TTGTGGGAAGCATTTTCTCC	60.051	20	267	87	353
59.903	20	ATTTAGCCGTTCCTCGGTTT	59.966	20	180	2865	3044
58.78	20	TCCTTCAACCAGTTGTTCCC	59.943	20	179	69	247
59.984	20	AACTGGCTAACCCATCACCA	60.375	20	246	81	326
59.974	20	TTCACAATTAACAAGAACAATTTC	59.077	26	145	591	735
59.883	20	CAAGAGCAACAACACCATTCA	59.749	21	122	724	845
60.049	20	TGCTAACTCAAATTTCGGTGC	60.258	21	207	541	747
60.292	21	GACACGAGATTTACATGGTTCG	59.499	22	109	1	109
59.154	20	GATTCCTCGTGGGATTGAAA	59.871	20	270	48	317
58.961	23	GATTGTAACGTTATTTGGCCG	59.376	21	153	1259	1411
60.154	21	TGAGGGAGAACTGCGGTATT	59.694	20	194	16	209
60.232	20	AACTGAGTCCCCTACCCGAT	59.817	20	205	321	525
59.929	20	TTTGAGGCGAATCGAAATCT	59.784	20	225	1023	1247
58.842	22	TAAAAGGTTCCCCACAGACG	59.964	20	129	74	202
59.942	20	TTGTTAGTTTCCATTCCGCC	59.938	20	100	212	311
59.962	20	ATGCACATGCTACTTCCACG	59.746	20	238	572	809
60.915	20	CGGAAATTTTACCCGGATGT	60.902	20	221	42	262
59.737	24	ATATCTCCCTTTTCGGCCAT	59.761	20	187	13	199
59.955	20	TCAAGTCAAACTTGCCAAACT	57.528	21	252	960	1211
59.823	20	GATCGCATCAGAGAGTCGGT	60.381	20	229	1112	1340
58.409	20	TCTTCCCCAACTACTTTCCAG	58.305	21	280	264	543
59.607	21	ACCCATTGGTGCTCAAACTC	59.973	20	242	450	691
59.133	20	TTGCTTCAGAGGTGAAATCACTA	59.031	23	280	130	409
60.011	20	CCGCCATTCTCCGTTAACTA	60.089	20	269	1105	1373
60.08	20	CCCGGATTTACGAGACAAAA	59.931	20	265	73	337
59.843	25	TCATCAGCCGTCAATCATTT	59.079	20	148	6	153
59.786	20	CCATCTCCACCTCCATACTCA	59.935	21	110	1902	2011
60.134	20	ACGCCCCAAGTTGTTTCTTA	59.609	20	265	147	411
59.917	20	CATTTTGGACGGTCCTGTTC	60.353	20	271	181	451
60.701	20	GCATATACGAAGGGTATTTTGGA	59.289	23	204	477	680
59.959	20	CTGAGTCCCCTAACCGACAA	60.103	20	172	178	349
59.989	20	AATTAATGTGGCATGGGGAA	60.016	20	246	54	299
60.103	20	TCACCCACGTAGAAGCTTGA	59.44	20	277	387	663
60.103	20	GAATCCGACAACGAAAGATGA	60.066	21	210	511	720
59.945	20	AGCAGTAGTGCCCCTGAAGA	60.012	20	275	995	1269
59.935	20	AACACTCTGTGTTGTTGGGAAA	59.538	22	270	72	341
59.864	21	CGGGGCTTCACCTAGTCTAA	59.331	20	251	113	363
57.69	24	TTTTGATTCCCCATTAAAAAGTT	58.076	23	223	257	479
60.096	20	CAAGAATCGCCTGCAAAGAT	60.352	20	251	1152	1402

60.036	20	AAGATTTGATCATGGGCGTT	59.391	20	158	35	192
57.546	20	AGCCGTGCCTTCAACATAAC	60.14	20	216	694	909
59.901	20	GCTCCCTTAAACACGAATGTG	59.623	21	239	106	344
59.934	20	GCCCCTTAAAACTCTCCTTGA	59.712	21	264	382	645
60.055	20	TCGAACTCATGCATTCATTTTT	59.592	22	277	2039	2315
60.067	20	CACCCGAAAACCGAATAAGA	59.931	20	125	160	284
59.966	20	TGCAAGCTCAGCCTAATGAA	59.712	20	163	26	188
60.112	20	CACCCGTGAAGCACAAAATA	59.585	20	252	140	391
60.023	20	ACCGAATTCCTTCCAAGGTT	59.805	20	226	2045	2270
59.75	20	CATGGAACAACATGAGCCAC	59.967	20	256	377	632
60.004	20	GTGATGTGCAACGCTATTGG	60.142	20	158	1163	1320
60.083	26	CCGAAAATCGGAGGATGATA	59.856	20	153	5	157
59.547	20	ACTTTCCCCGTGGACGTAG	59.974	19	259	219	477
59.928	20	ATCGGTTATTACCGGATCGC	61.031	20	109	276	384
60.237	20	CTGGAACTGCAATGCTTCAA	59.988	20	235	414	648
59.903	20	GCTTTCAATCCTCCAGCATC	59.78	20	213	16	228
60.119	20	CATTACACCACCCTTCCCAC	60.088	20	201	332	532
59.162	20	GAGGTTTCCAACTTGCTTCG	59.853	20	213	169	381
59.989	20	TTGTGAGAACCAACTCGGTG	59.72	20	274	232	505
59.091	22	GGTTGTTGCCGCTTCTACTT	59.383	20	264	0	263
60.031	20	GAGATGGTTCGAGAACGAGG	59.803	20	150	438	587
60.397	20	ATTCAATTGCTGGTGGAAGG	59.933	20	265	28	292
59.708	20	TTGGAGATAGAATGAGATAGAAT(	58.905	27	168	218	385
59.73	20	CAACCCTACCTCCTTCTCCC	59.928	20	211	163	373
60.214	20	CAAGAGCTCTCATTCTCTCCCT	59.23	22	251	618	868
60.162	20	GCATCAAAATTCAAAAGCGA	58.89	20	269	382	650
59.004	20	ATCCGGCCATGTTGCTATTA	60.31	20	243	1550	1792
59.984	20	AAACCTAGCATTGGGGAAGG	60.312	20	211	88	298
59.573	25	AATTGATGGAACGGGGATTT	60.378	20	157	8	164
59.387	20	GGACAAAACTTCATGGGAGC	59.532	20	277	316	592
59.28	23	TTCATGATTCAAAAGCATCAAAA	59.595	23	136	66	201
57.665	23	TTTTCACATGCCATACACCAA	59.841	21	183	25	207
60.074	20	TCTCACCTTCGGTCAGAACC	60.238	20	263	289	551
59.849	20	GGAAGAGAGAGAAGGGGTGG	60.186	20	270	195	464
59.72	20	AATGGAAGGGACGAGTTTGA	59.526	20	135	292	426
59.938	20	TTAAAAACCGAACTGGACCG	59.968	20	193	2813	3005
59.935	23	CCACTCTCCCTCTCAGTACTTTTC	59.823	24	280	93	372
59.82	20	GGAGATCGAAGGAGCTGATG	59.91	20	221	313	533
59.265	20	TGATTACATCGATCCAACGG	59.35	20	270	54	323
61.146	20	GGGTGGGTCGATTCAATATG	60.014	20	243	9	251
60.058	20	TGGAGCTCTTGTTCCTCACA	59.545	20	133	75	207
60.016	20	GAATACCCCCTACTTTGCCC	59.666	20	233	5	237
59.144	26	AACAACTTTGAACCTGCGCT	59.92	20	226	9	234
60.658	23	TCTGCCTCGTCTGGCTAGTT	60.156	20	241	1	241
59.953	20	CGAAAGCTGGAGCTGAGAAC	60.277	20	123	10	132
59.01	20	AAAACATGCCTTTCGACCAC	59.978	20	246	96	341
59.76	21	ACGGTGTACCTTCCCCTCTT	59.856	20	227	49	275
59.624	20	ATGCATGGCCATAACTCACA	59.955	20	270	166	435
60.045	21	GAGGCAATTAATCAGGGCAA	60.038	20	186	1861	2046
59.861	21	ATATTACCTGCACAAGGCCG	59.982	20	253	86	338
59.666	22	TGCAACCAAGTTCCAAATCA	60.088	20	258	189	446
59.778	22	TCCCAACTGAGATCGGAATC	60.011	20	277	5	281
60.162	20	GTTCGCATACAAACACGCAC	60.188	20	231	106	336
60.242	21	GGCCCACGTACATGACTACC	60.263	20	231	2263	2493
59.726	21	AAGATGGGCGAATAAGACCC	60.286	20	122	77	198
59.917	20	TGTTGGGGTTGTTGTAGGGT	60.126	20	202	1792	1993
60.102	23	ACAAAATTGTGCCCAAGGAG	59.971	20	194	13	206
59.955	20	CTGATTCGCACAAATGATCG	60.22	20	231	36	266

59.694	20	TGCAATGCATGGAGGAATTA	60.035	20	179	84	262
59.997	20	TTGTTGTTGTTGGGCATTGT	59.864	20	222	1884	2105
60.251	20	AAGTTATCTGTGGCCGATGC	60.103	20	259	2368	2626
59.542	20	CTGAACCACATGTTCCCCTT	59.82	20	134	2	135
60.133	20	AACGGACGAACAACGAAAAC	60.015	20	235	320	554
59.89	21	AAACGACATCCTGACGCAAC	61.107	20	274	225	498
59.957	20	TATCATTGCTCGTTTTGGGG	60.827	20	254	190	443
57.589	21	GAACAAGGTTCCATTTCACACA	59.875	22	173	39	211
60.24	20	CCCCACTTGGCTTGAGATAA	60.066	20	267	54	320
58.694	22	GGATTGCATCATCAAGTCTCC	59.503	21	203	0	202
59.141	22	AGTTGTCGAATCCGCAAGAG	60.397	20	198	552	749
57.197	25	TATCCTTGCACTCTTGCCCT	59.836	20	252	13	264
59.72	20	TGTTTGAATCCGAGTTTCCC	59.91	20	235	47	281
57.636	25	GAAGTTGAGCCATATTCATTTGC	59.989	23	181	440	620
59.96	20	TCTTGGGAGGGAAAATGACA	60.43	20	185	448	632
59.941	20	GGGTCAAGTCATGTTCCCAC	60.223	20	237	615	851
59.809	21	GAGAGAGAGGCAGACATCGG	60.096	20	116	55	170
60.155	20	TGAGGATGCAAGCCTCAATA	59.375	20	232	3038	3269
59.435	20	GGACCGCTGGATAATCCTCT	60.431	20	210	98	307
59.075	20	AAGCTGGTGGATTGAATGCT	59.7	20	172	175	346
59.233	20	CATACAATTCCCCACCCTTG	60.044	20	213	90	302
60.162	20	GGGTAGGGGTCCCTGTATGT	59.935	20	192	434	625
60.011	20	GAGCACGAGGGTGAAAGTGT	60.307	20	252	59	310
59.938	19	CCCAGCTGAATGCGAGTTA	59.956	19	280	265	544
61.191	20	TCTCGTGGTGCAGTGGTAGA	60.463	20	255	28	282
58.379	20	TTTGCATGAGCTAACATGCC	59.839	20	206	650	855
60.134	20	CATTCCCATCATTTCGGCTA	60.797	20	109	1929	2037
59.807	20	AACGAATGTAACAATACGTACAC/	57.736	25	233	382	614
60.132	20	AGCACTCGTGGATTCTGGAC	60.269	20	152	962	1113
60.375	20	TTACAAAAATGCCCTTTCGC	60.075	20	249	427	675
59.64	20	GGTACAGCCGAGTAGCGAAG	60.037	20	269	215	483
60.667	20	AAGAACACAACGAGGATGGC	60.119	20	260	254	513
60.529	20	TTATCCTCACCCGTTTTTCG	59.931	20	148	293	440
59.574	20	AACCCTTTGTCCCGATCTTT	59.805	20	245	3835	4079
58.52	25	CGATTTGGAATCGATAAGCA	58.721	20	269	560	828
60.177	22	ACCTCTTCCACGGCCTCTAT	60.096	20	130	85	214
59.725	20	TCTTATTGGCCCGTTTATTTT	57.725	21	276	251	526
58.741	20	ATCTCACCAAGATGCCCAAG	60.073	20	100	7	106
60.081	20	TGTAATGGAGGGGGAATCTG	59.744	20	212	186	397
59.839	20	ACAAACTTATGGACCGCTGG	59.993	20	159	502	660
60.149	20	GTGCACGAAATTGGAGGAAG	60.636	20	179	2074	2252
59.853	20	CCTATGGTGGTATGGTGTGG	58.569	20	238	231	468
60.117	20	GCGAAGGAACTGTGGAGAAC	59.851	20	156	328	483
60.082	20	AAAAATCGACAAATTATCAGTACC	58.166	25	275	433	707
59.957	20	CACCTTTGCAAAAGGGAGAA	60.22	20	254	10	263
59.963	21	AACACAGCTGAGTAACCGGG	60.171	20	102	1291	1392
59.882	20	TAACCAGGCCCTTTCTCCTT	60.068	20	265	1437	1701
60.134	20	GCGTGTTAAATTTGGATTCCC	60.541	21	280	2247	2526
59.964	20	TGGCGAATATTTTGTTTTTGA	58.213	21	145	281	425
58.278	20	CTACAAATGTTTTGGCCCCT	58.949	20	183	1275	1457
59.685	20	AATTCAATCCCCAAAAACCC	59.867	20	269	363	631
59.774	22	AAGCCACTTTTGTCAAGGTGA	59.766	21	234	1097	1330
59.651	20	GGCTCTTCGGCAGTGAATAG	59.978	20	253	252	504
59.917	26	GAAAACGTCGAGTTTCAGGC	59.859	20	193	28	220
59.813	20	ATTGCCTTGTTGTTTGGGAC	59.836	20	201	46	246
59.67	20	GGTCTATGGTCGATTGGGTG	60.195	20	203	4617	4819
60.042	20	AAACAGAAAAATTCCCCTTCATC	59.725	23	264	4454	4717
60.149	20	TCCGTTGTAAAAAGACCGTTG	60.018	21	201	38	238

59.722	20	GTAGAAAACCACGAGGGGGT	60.225	20	256	2162	2417
61.009	20	TCACGCTAAAGAGGTGTTTTGA	59.92	22	252	212	463
58.876	24	TCGTGCTCATTTGTGTTGGT	60.16	20	183	7	189
58.745	20	GCCAGCCAGGAAAACACTAA	60.249	20	127	346	472
60.586	20	AAATTCACGTGTTAGAGTTGATTC[illegible]	59.424	26	231	911	1141
59.962	20	CCGTGCTTACATCAAGCAGA	60.011	20	227	15	241
60.228	20	ATCCCCTTCATTCAGCACAC	59.934	20	122	10	131
59.91	20	TCAAGAGTTGCATGTGAGCC	59.992	20	251	1470	1720
60.074	20	ACTGACATTCACAGGCATTG	57.067	20	204	57	260
60.011	20	TTAAACCCTAAAAACGCGCA	60.593	20	239	106	344
60.124	20	GGGATGGAGGAGGAAAGAAG	60.008	20	251	339	589
59.948	20	CCGAAAAGCTGAAAGAAACC	58.949	20	115	819	933
60.326	20	CCTGTTGTGCCCATCAATTA	59.395	20	183	9128	9310
60.058	19	TAGGATGATTGGCAACACGA	60.073	20	196	0	195
60.156	20	TCCAATCAAGGCAAAGATCA	59.2	20	205	5464	5668
59.74	20	GGTGATGTGTGACGTTGGAG	60.005	20	214	105	318
60.112	20	AGATCAAGGTGGCTGCAAAT	59.7	20	251	700	950
59.839	20	TGTGGTTGATGAGTTGGCAT	59.967	20	112	1229	1340
58.804	22	TTCCAAGTCTTCCCCTTCCT	60.045	20	176	3	178
59.717	20	TTTGTTTTCCATCGTATTCCA	58.005	21	143	4091	4233
60.104	20	TTTGAGTCGTTCACCACGAG	59.873	20	230	98	327
59.691	20	TTCAAAATTGAAACCGGACC	59.778	20	234	24	257
57.59	21	CTAGGCATGGAATCCGAGAA	60.17	20	269	59	327
59.432	24	TGTTTGATATGGCAGGGGTT	60.192	20	216	634	849
60.022	20	TGCAAGTGCATATCAATGAACA	60.14	22	135	1314	1448
60.468	20	TGCTGAGACATAGCGGACTG	60.16	20	169	228	396
60.133	20	GGTGGGTTCGACGACAATAG	60.375	20	131	541	671
60.005	20	TCAACTTCTTCAACAGGCCA	59.415	20	244	2186	2429
59.853	20	CAACCTAGAATGAGCATTGGC	59.722	21	207	703	909
57.234	21	CACGGGTCTTTCTTCTTCCA	60.224	20	201	9	209
60.228	20	AAGCCAGTGCTGGTTGACTT	59.914	20	239	1156	1394
59.714	20	AATGTGGATGCCCCCTATTT	60.399	20	252	233	484
59.934	20	CAGACGCCGACATTTATCCT	60.096	20	144	176	319
60.167	20	GCGTGATCTTGGTGTTCTTG	59.293	20	168	1948	2115
58.972	21	AAAATTCGATGATTGGCTCG	60.038	20	212	333	544
60.014	20	CCTTCAAGCTGTCGAACTCC	59.989	20	278	444	721
59.801	20	CCAAATTAAGCGAACCGAAA	60.068	20	222	334	555
60.056	19	TGTTGGAGTCTTGAACAAGGG	60.133	21	261	309	569
59.832	22	CATTGACTGTCACAATCCCG	59.96	20	251	1013	1263
60.161	20	TTAGGGTTTAAAATCGCGCA	60.564	20	280	7557	7836
59.56	20	TATGGGTTAGCGTGTGATCG	59.566	20	244	119	362
60.026	24	GAGATTGGTGGTTGTGGGAT	59.636	20	195	0	194
59.769	20	TGGAATAGGAAAGGTGCAGG	60.066	20	216	650	865
60.073	20	TCTTAATCCTTTGGCATTCAA	57.388	21	212	1124	1335
59.838	20	GGAGGGTGCACATCTCTTTC	59.661	20	268	2	269
60.032	20	CGCGGATGGTTAAGATTCAG	60.597	20	191	1435	1625
59.978	20	CAAAATCAGGCACCAGCTAA	58.917	20	232	909	1140
59.833	20	TTGTCCCCAAACAACGAAAT	60.206	20	185	54	238
59.28	23	CAGCATACCAAGGGCATTTT	59.96	20	215	24	238
58.96	22	GCCTCAAATCCTATTCTATTTTGC	59.549	24	246	77	322
60.195	20	CAACCCTTCCAGTTCTGGTC	59.549	20	258	1280	1537
59.937	20	CTACCACCGATCCCTCTGAA	60.065	20	228	1073	1300
59.405	20	AGCAAAACGGTCATCTGCTT	59.882	20	273	21	293
59.933	20	CACCCCTGAGCTCACTTTTC	59.844	20	218	644	861
59.805	20	TGTCCACTGATGTGGCATTT	59.967	20	189	297	485
59.52	20	CAGCCCACAAATCCTTGACT	60.111	20	214	710	923
60.272	20	ACTTGTTGAAATTCACCGGC	59.978	20	278	528	805
59.933	20	TTGAGGCTCCCAACTTTCAC	60.232	20	260	987	1246

59.473	20	AAAAATGTCCCCGAAGATACA	58.43	21	245	485	729
60.012	20	CACTAAAGTGTGCTGTCATCG	57.006	21	184	19	202
59.835	20	ACTCCCCCTCGAAGTTTTGT	59.972	20	261	219	479
59.694	20	TCATCAATTCTTTCTGCGCTT	59.974	21	279	1240	1518
59.858	20	CATGCCGAAAGAGTTTACGA	58.917	20	248	44	291
60.029	20	GTTATGCGTTTTGACGGGAT	59.829	20	200	514	713
60.04	20	TCTCATCATCTCCCCTCACC	60.008	20	227	324	550
60.192	20	AGTAGAGAAGGGGAAGGGGG	60.797	20	168	469	636
60.529	20	AATATCCATGCCATTCCCAA	59.979	20	272	166	437
60.245	21	TGGTGTTGCAATGCTACTGA	58.863	20	276	676	951
58.81	24	TTGGATTGAGAAAGCGATCC	60.155	20	278	15	292
60.029	20	GGGTTATCAGCAGCTTTTGC	59.851	20	259	1081	1339
60.221	20	TCGCACTTGGACTTTCCTTT	59.853	20	272	11	282
57.757	20	TTCAAATGTTTTTGTAATTTCCCA	59.653	24	100	30	129
59.993	20	GGCCGCTGATCATTTAGTTG	60.606	20	276	1217	1492
60.457	23	GAATGTAGGTATAAGACTGTGAA[illegible]	57.394	26	280	5	284
60.118	20	GGTTTGCCCCTGTATCTCAA	59.933	20	230	219	448
57.978	23	GCGACATACATTGATCTGCG	60.251	20	249	32	280
59.95	20	TTCCTCTCGTGTTTCATCCC	60.05	20	198	3	200
59.955	20	TTAGCCCATCAAAGTCCAGG	60.066	20	185	196	380
60.038	20	GTTGCGTTCCACATAAGCCT	60.14	20	170	2736	2905
58.941	19	ACCTACCATAACCGTCGCAT	59.34	20	223	21	243
59.931	20	AACCTTATAGGATGTCCGGC	58.019	20	265	776	1040
60.058	20	CCGAGTGCCTATTTTGCCTA	60.223	20	224	6804	7027
59.663	21	CGCGTCTATCGATACTGATCC	59.705	21	256	3227	3482
59.505	20	AAAAGGTCTGCCCTTGCTTA	58.973	20	268	228	495
60.024	20	GGCTTCCGAGTTTTTCCTGT	60.61	20	225	900	1124
59.971	20	ACAGTCCACACATGAAGCCA	60.162	20	176	1078	1253
59.682	20	GGGGTTATCATGGGATCAAG	59.055	20	211	6	216
59.507	23	CGGTTAAAAACCGAATCGAA	59.937	20	226	380	605
60.564	20	CGAAGTTAGAAAATAAGGAGGTG	58.471	25	152	115	266
59.931	20	GAGGCCGTCGGAATTTATTT	60.28	20	250	1336	1585
57.795	21	TCGCGTCTGACTCCTCACTA	59.726	20	164	34	197
58.78	20	GTCCACGGTGGTGCTAGAGT	60.182	20	104	32	135
60.255	20	TTTGTGTCGTCGGTGTGATT	60.008	20	217	937	1153
60.005	20	AAAAGGGGAGAAAAGAACGG	59.566	20	241	32	272
59.988	20	TTACCGAGCTTGATGGGAAC	60.074	20	178	488	665
59.66	20	TTTCTCTCGTGCCATTCTCA	59.522	20	265	312	576
57.591	23	TGGTAAAACTCGAAGGATGGA	59.555	21	191	33	223
60.05	20	TGTTGCACTTTTGAACAATGG	59.618	21	176	69	244
59.945	22	TTACATGTGTGGGGATGGAA	59.623	20	139	224	362
60.096	20	CGTGTTTATCCTCACCCGTT	59.853	20	200	235	434
59.42	20	AAACATAAAAGCCACCTGCC	59.091	20	204	16	219
60.624	20	TAGCATCGTGAGTAGCGTCG	60.18	20	167	0	166
59.273	20	TGTGGCATCACATGGTCTCT	60.121	20	237	35	271
59.29	22	GCCGATCGAGTTTTGGATAA	60.038	20	152	215	366
60.005	20	GGTGTGTGAGGGAAGGAGAG	59.682	20	242	853	1094
59.962	20	GAACACTATTCCCATCCCCC	60.388	20	146	28	173
59.945	20	ATAGACCCAGACACGGGCTT	60.903	20	197	27	223
58.877	20	TGCTCAATGAACTCCTTTGG	58.847	20	138	11	148
60.37	20	ATCTTACTGGCATTGTCGGC	60.103	20	217	1213	1429
57.576	24	GGGTGGGCTCCATTTTTAGT	60.187	20	200	11	210
60.135	20	AAAGAAAAGGACGGGAGGAA	60.046	20	114	80	193
59.962	20	AACGGCCGAGATTTGTTATG	59.96	20	229	883	1111
59.668	20	TGTGCGTGTAAAGCTGGTTC	59.911	20	237	741	977
59.926	20	TACTTTTGGGCAATCCTTGG	59.931	20	167	3147	3313
59.565	20	TTGCCAAACACACATTTTCAA	59.999	21	268	1298	1565
59.801	20	GAAAGGTGCTTTGCCACTTC	59.859	20	204	12	215

60.194	20	AGTAGGATTCCGGTTCGCTT	60.096	20	247	221	467
59.302	21	TGCCTATTTTGAATTTTGGTG	57.705	21	145	368	512
60.243	20	TATGGGTTATCACCGGATCG	60.544	20	217	1096	1312
60.048	20	TCGATCATAAGAGCATCCCC	59.998	20	138	490	627
60.145	20	CGTCCCACAAAATATGTCCT	57.393	20	212	45	256
60.617	20	CAGTTGGGTCAGTGACATGG	59.997	20	223	111	333
60.336	20	CGCTTAAAAACCGGACCATA	59.958	20	147	894	1040
57.538	25	GCCGAGATTTGTCAGAGGTT	59.288	20	103	23	125
59.782	19	GGGGTGACCTTATCACTCCA	59.779	20	189	0	188
60.189	20	CAAAAAGGAGCTCAACGGAG	59.986	20	143	105	247
59.735	20	CAAAATTCCCACCCAATCAG	60.162	20	271	107	377
59.853	20	AAAATGCACCATTTCTTGCC	59.945	20	276	1243	1518
59.925	20	GCTTCATTATTTTTGCCCCA	59.907	20	141	24	164
59.901	20	ACGCTATGAGAGCGAGGGT	59.989	19	274	38	311
60.196	27	TTCCGGCTACTTCGAACTTT	58.973	20	262	565	826
59.903	20	AACATGGGCAAGAAACAACC	59.836	20	257	869	1125
59.2	20	CCGGATCCTATTCACCTCAA	59.887	20	276	1044	1319
59.789	20	TTCATTGGAAAGTTAGGGCG	60.067	20	133	365	497
59.697	20	GAACAACCAAGGCAATTTCAG	59.601	21	235	25	259
59.923	20	CTGAATCGCGAAACATGAAA	59.809	20	178	498	675
59.089	24	ACCATGATGAATGTGAGGCA	59.925	20	267	31	297
59.955	20	ATTGGAACAATACTATACAACCAC	57.118	26	135	87	221
58.968	20	TTCTTGATGTGTTGGCAAGC	59.847	20	138	170	307
59.209	20	GGTCCATGGCCTGTTATGTC	60.203	20	258	248	505
59.779	20	TTAGTGGGCTAAGTGGGGTG	59.986	20	172	205	376
58.585	22	TTGCATCAAGTTTTAGAGCAGC	59.674	22	227	35	261
59.938	20	ACTGAATCAGCGCTCCAGAT	59.981	20	243	115	357
59.935	20	CTTCCCTGCACAAAAGTCGT	60.291	20	278	57	334
60.03	20	AAGGTCAGGAAAACCCAAGG	60.332	20	255	2782	3036
59.943	20	TACAGGTTCACCCTTCTGGG	59.959	20	158	178	335
59.997	21	ATTTTCCCGGTGAAAGAAGG	60.291	20	123	252	374
59.657	20	TTTCTTTTCTTTTCTTTCCATTTCA	59.678	25	276	101	376
59.127	20	GCCATCCTCTCCAAGCTACA	60.362	20	233	2214	2446
58.188	24	CGGCTATTTGAGACGACATTT	59.238	21	279	2	280
59.094	20	CGACCCAGATTCAAACCCTA	59.926	20	204	184	387
59.402	23	TGGTGGTGGGAGAAGAGAAG	60.229	20	207	84	290
60.241	20	GCCACACACGAAATGAATTG	59.972	20	127	351	477
60.292	20	CGAACGGTATAGGATCGGAA	59.916	20	227	79	305
59.843	20	GCCTTCCATTCCAAAAATCA	59.878	20	151	113	263
59.178	20	GTTGGGGGATGAAGAAGGAT	60.133	20	265	449	713
60.255	20	TGAGGCAAACTGAAAAACCA	59.293	20	246	323	568
59.92	20	TCGATCCCACAGAGATTGGT	60.475	20	255	324	578
59.997	20	CTGTGCATTTGCAGACCACT	59.905	20	206	397	602
59.585	20	ACAAGTTTGGGATTGCTCCA	60.495	20	272	206	477
60.03	20	CACCCCTTATCTTCCACAGC	59.55	20	231	173	403
59.896	21	GTACCTGCACGTTGCGTAGA	59.935	20	253	4225	4477
60.035	20	AGGGGCAAGTTGGCTATTTT	59.966	20	183	2577	2759
60.111	20	ATCGTCGAACATCCTTTTGG	59.933	20	255	2315	2569
59.909	19	TGATTGCATTATCAAGAACCAAA	59.485	23	135	107	241
60.564	20	GACACGTGGCAGCACTAGAA	60.056	20	219	445	663
59.917	20	AAAACTTCCTAAGCCCCCAA	59.939	20	206	665	870
60.32	20	CCCAATTGTCGTGTGTGTTC	59.855	20	213	2	214
59.682	20	CCACCTATCTGCATCCACCT	59.95	20	108	1497	1604
59.973	20	GGCGGTGAAACGATAAACAT	59.829	20	230	227	456
59.729	25	TTGACCCCAATGGATCGTAG	60.713	20	278	166	443
57.236	20	TTGGTGTTGGTGTTGTTGCT	60.049	20	225	45	269
59.708	20	ATTCGTTTTTCCTTCCCAGG	60.291	20	140	51	190
59.654	21	AGGTTGAGTCGGACGAGTTG	60.298	20	256	590	845

59.972	20	AGCATCAATGGAGTATTGAAAAA	57.835	23	252	345	596
59.823	20	AATGTCGTGACACCTAGCCC	59.997	20	173	84	256
59.601	21	TCGGGAGTCTGTATTGAGCC	60.218	20	190	27	216
59.997	20	TGGTAAATCACTAGACCCAAGGA	59.879	23	277	10	286
59.213	22	TTGCGGATTCTAAGATTGGG	60.031	20	151	60	210
60.278	20	ATTGAGAGAATGCGAATGGC	60.185	20	268	19	286
59.518	20	ATGCCCATTCAAACTTCAGC	60.081	20	113	86	198
60.535	20	TGATGGATGGATGGTCTTGA	59.85	20	104	92	195
60.134	20	TATGTATCGTCCGCGCTGTA	60.259	20	255	633	887
60.042	20	TAGTGCCGTATTGTTTCCCC	59.823	20	257	77	333
59.978	20	TTGGACAAGCATCGGTTACA	60.111	20	215	575	789
59.971	20	TGAAGTAGGAAATAAGGAGGTGT	57.525	24	263	215	477
60.088	20	AGCGTAAACACGTCCCCATA	60.386	20	231	155	385
58.512	26	GCTTGCAGCCTTCTGTGTTA	59.224	20	227	1	227
59.243	21	ATAACCCCACCAACCTAGCC	60.074	20	102	29	130
59.94	19	GGAGTATCACTTGAATGCACCA	60	22	280	15	294
59.064	20	AGGGGAGAGATGGAGGTGAT	59.889	20	236	111	346
60.422	20	AGCTGATCCTGCGACATTG	59.959	19	256	366	621
60.207	19	CACTCCCCCGTAAAACAAGA	59.964	20	272	370	641
59.955	20	CCTCCGTGGACGTAGATCAT	59.95	20	191	21	211
60.323	20	CGGTTCATTCCCATCCTAAA	59.756	20	210	227	436
60.166	20	GCCGTGTTAGCCAACGTAAT	60.025	20	246	57	302
60.183	20	CGTAGTGGAAGTGACAGCGA	60.049	20	171	284	454
59.801	20	ACCTTTTGCATTGTCAGCAA	59.322	20	225	393	617
59.109	20	CTAAGCACCACAAGCAAGCA	60.195	20	209	179	387
59.55	22	GAGCTTCCGCATCGTTCTTA	60.49	20	239	3	241
59.648	20	TGCATACGGAATTTCAGCAA	60.215	20	239	512	750
59.797	20	ATTCGAAAATGCTCGTGAAA	58.361	20	237	1908	2144
60.038	20	TGATCATTATTGGAACGCGA	60.035	20	209	82	290
58.923	21	ATCTCCAAAACCCGACAACA	60.353	20	271	110	380
59.756	20	TGTTATGCATTTTGACGGGA	59.93	20	180	89	268
59.862	20	CACTTAAGGGGGTTATCATTGG	59.604	22	209	99	307
59.95	20	CATGAACGGTTTAGGATCGG	60.323	20	255	159	413
59.929	20	TGCTGTTAATGAAGGCATCG	59.833	20	129	297	425
59.955	20	TGGAGAGGACCTTGGAACAC	60.088	20	229	2570	2798
60.195	20	CGTCCATTTCTGCAAATCCT	60.074	20	171	393	563
59.853	20	AAAATTGAAGCGCCAGAAAG	59.474	20	269	101	369
59.904	22	TGTCCGTTAAGCAGTAAAGACG	59.464	22	196	435	630
59.867	20	CCTACTCCACCCCTTCATCA	59.92	20	140	2762	2901
59.875	20	CGCGTGTGAAACTCTAGCAA	60.195	20	137	194	330
59.182	20	TGAATGAATCACAAAAGGGGA	60.294	21	228	24	251
60.195	20	GTTTTGCCCATTTTACCCCT	60.055	20	198	23	220
59.996	20	AGCTCTTTCTCCACAGACGG	59.598	20	255	2154	2408
59.92	20	TGAATCCTCCCGTTTTCTTG	60.044	20	227	176	402
59.691	20	TCCTTGTGGTTCTACAAAATAGCA	60.056	24	131	168	298
59.786	20	CACACCCACACGCACTTTAC	60.072	20	187	386	572
59.878	20	AGGATATTGGCGATGTACGC	59.951	20	263	445	707
59.116	20	CCGATCTTAATCTTCGCTGC	59.945	20	201	165	365
59.996	20	GGAAATTGATGGATTTGGGA	59.558	20	187	674	860
59.971	21	TCTCAGATCCAAACGCCTAGA	59.963	21	169	199	367
60.011	20	TTCGGGGCATACATGTGTTA	59.809	20	222	3066	3287
59.851	20	GAGTTGGGGGATGAACTGTG	60.363	20	215	70	284
60.016	20	GCTCCTAAAATATTGGTTTCTATTT	59.441	27	146	406	551
59.205	24	CAGCATTGCTTGCACTTCTAA	59.264	21	193	1104	1296
58.212	23	TGTTTTGTAATTTCCCATGTTTG	58.816	23	102	23	124
59.373	21	CGAGTATTGGTTCAATAGAATTGA	60.143	26	275	310	584
59.978	20	TGTGTACCGCAGTTCAGAGG	59.897	20	244	94	337
60.12	20	TTTGATCTACCTCGGCCATC	60.036	20	125	2321	2445

59.542	20	ACCACCTCGGCGTAAATATG	59.845	20	205	158	362
59.62	21	TGAAAAGTCACACGAAAACGA	59.352	21	181	239	419
60.131	20	TGGTTGTTGTGAGGGTTTCA	59.976	20	245	21	265
59.989	20	AGAAACACCATTGCAGTCCC	59.973	20	142	2263	2404
60.103	20	GGGGTTATCATGGGATCAAG	59.055	20	262	275	536
60.048	20	GAACCAGAACTACCCCACCC	60.607	20	245	1282	1526
60.215	20	ACCGGAACCGGAATTAGAAC	60.187	20	221	322	542
58.382	21	TGTGTTTTGACAATATTTAGTGTTT	58.633	27	101	151	251
57.68	21	GATCAGCCATCAACCCCTTA	59.894	20	179	0	178
59.402	20	CAATCCGCACACCATATTCA	60.343	20	258	555	812
60.074	20	GCAAATATTGGCCGTCAGAT	59.929	20	226	399	624
59.948	20	ATGGTGGTATGGTGTGGAGG	60.51	20	170	93	262
59.293	20	TGATGGAACGGGGATTGTAT	60.014	20	188	619	806
60.501	20	TGCTTGGGTCTACCCTTTTG	60.103	20	202	3383	3584
59.903	20	GTCTGGAGTCCATGAGGCTG	60.829	20	121	77	197
59.928	20	TGACGGGATTTAGGTGTTTTG	59.847	21	104	367	470
59.962	20	AGAACGAGAGAAATCGACGG	59.43	20	184	93	276
59.995	20	CAGCCCCTTTTTCATTTTCA	60.046	20	213	228	440
59.439	20	GTGGACGTCCCAGATTGTTT	59.827	20	164	3	166
60.894	20	ACAAATAAGCCCACAGCCAC	60	20	174	74	247
59.873	20	CCCTAAGTTCCCCCACTGAT	60.183	20	192	1053	1244
60.03	20	TCGACCTACGAGAGTGAGGG	60.399	20	279	363	641
59.972	20	CAACTTTTCCGGTCCGTTTA	59.968	20	276	714	989
59.969	20	GGGATGAAATGAAGTGGGTG	60.173	20	267	397	663
59.547	20	TCTTCGATGAACTCTGAAATGC	59.457	22	191	279	469
60.425	21	CGAACCCTTTCATTCTCCAA	60.044	20	278	293	570
58.974	20	TTTTCTTCAAAATGCACAATAATTT	58.319	25	237	97	333
59.536	23	CCATCGTCGCTATCACATTG	60.096	20	165	79	243
60.872	21	TGCAACAAATGGATGGTTGT	59.823	20	118	18	135
59.245	22	GGCATCAAAGTTGTCGATCAT	59.954	21	111	3	113
59.945	20	TCCCACATTGGTTGGAGATT	60.173	20	247	26	272
60.212	20	AATTAACGAAGGTCCCCACC	60.053	20	127	3669	3795
59.791	20	TTCGGTCACATTCAATAAGGC	59.952	21	244	1047	1290
59.779	20	TGCAAACCCATGATTGACAC	60.376	20	151	695	845
59.542	20	CAGGCTTGCAGCAACTAACA	60.195	20	236	689	924
59.84	20	GGATAATTTGGACAGCCCAC	59.249	20	176	826	1001
57.062	26	AAGAGGGGTGAGGGAGATGT	59.929	20	197	0	196
60.461	20	CAACGAAATCCTGCCAATCT	60.074	20	179	65	243
59.985	20	AACCGATCATTTCATCAGCC	59.9	20	271	2715	2985
60.176	24	GAGGCCTCGATCTTCTGTCA	60.497	20	104	11	114
58.943	20	TACGGTTCGGTTTACGGTTT	59.379	20	257	150	406
58.94	26	TGGGCTAATCACTCTCAGCA	59.547	20	178	11	188
60.222	20	CAATTTGAGAATGGGATGGG	60.126	20	236	776	1011
60.636	20	GTTGACAAAACGCACGTGAA	60.742	20	228	583	810
60.953	20	AACATTGCGGACTTGGTACA	59.05	20	265	774	1038
60.053	20	TGAGATGATATTATGAACTGCATC	59.394	25	264	208	471
60.397	20	CGTGATGCAACGCATTCTTA	60.807	20	248	227	474
59.964	20	TCCGAGAATCTGTTAGAGGAGAA	59.491	23	255	103	357
59.603	20	CCTTTTGCAAGCACTGATGA	59.988	20	196	533	728
59.36	20	TGAAAAGGGGTGAGAGGATG	60.042	20	252	124	375
60.003	20	TTTTTATGCACAAGCCTCCC	60.074	20	275	51	325
59.843	20	AACCACAGCCCTACATCTCG	60.134	20	263	344	606
60.249	20	TCATTTGTTCCACTTCATTTGG	59.839	22	250	4339	4588
59.794	20	GCGTGGCCTAAGTTGTCCTA	60.27	20	257	645	901
60.124	20	TAGTGAAGCAGCTGACGTGG	60.199	20	144	65	208
59.982	20	GGTTGATGGATCTGCGTTTT	59.939	20	224	68	291
59.927	20	TCAAAAATATCCTCGCTGCAT	59.696	21	128	4	131
59.773	20	AGAGTTGGGGATCTGCAAAA	59.67	20	211	103	313

59.917	20	ACCGTCTCGACGAAAGCTAA	60.015	20	224	343	566
60.257	20	TGAGGCATGCAAAGATGAAG	59.948	20	234	199	432
60.088	20	TTGCGTATGATAGGGTTAGCA	58.342	21	240	26	265
60.081	20	CATGCTACATTCCGTGATGG	59.948	20	201	592	792
60.081	20	TTTCTTATTTCCCGGCTCCT	60.034	20	137	553	689
59.776	20	AAAGAGGAAGAGGGAGGCAG	59.95	20	238	644	881
59.767	20	AATCCTTGCTCTGGCTGCTA	60.118	20	259	3	261
59.902	20	GGGATGTTTGTACCTGAATGC	59.307	21	121	3	123
59.96	20	ATAAAAATGGCACCTGGCTG	59.96	20	220	158	377
59.577	20	CAAGAGGCAATCACGTTGAA	59.84	20	232	281	512
60.472	20	GTTCCTTGCGTGGCATTATT	59.967	20	185	1027	1211
59.318	27	TCAAGAGCAAGCAAAGATCTAAA	59.7	24	164	6	169
59.938	20	TACTGACTTGTTGTGCGGGA	60.301	20	271	381	651
60.165	23	ACGGTCTGGTTTCAGTTTCG	60.149	20	146	769	914
59.028	20	GGAACCGGCTAAGATGATGA	60.036	20	227	244	470
57.369	25	CCCGCATGTCATAACCACTA	59.42	20	274	268	541
60.096	20	CCAATGGCGATATTTCCTGT	59.784	20	213	168	380
60.049	20	TGGTGTCTATGTATGGGTGGA	58.709	21	210	651	860
60.668	20	TGGACATAAATTGTAGTGCCAAA	59.415	23	176	667	842
60	20	ATAAATGCCTACGTCCACGG	59.845	20	231	151	381
59.935	20	CACCCTCCTTCCTCAAACAA	60.081	20	277	404	680
59.576	21	AACAAAGGGGGTTGGAGAAT	59.668	20	232	3177	3408
60.183	22	CGCGTGCTCCTAACTCTTTC	60.154	20	118	29	146
60.386	20	AAATGGTTGTTTGTCCCCAA	60.066	20	203	146	348
59.923	20	TAGTCAGAACTTGCATGCCG	60.011	20	171	46	216
59.989	20	TCTCTCTCTTTTTGGTAGGTCAATG	60.165	25	262	144	405
57.139	20	TAGAATGTTCCGAAATGGGC	59.901	20	176	23	198
60.051	20	TGTGAAGTGTTGCTGATGTGA	58.855	21	220	1168	1387
59.95	20	GCGGATCCCCCTTAATTTT	60.102	19	280	302	581
59.666	20	TGAAAATTAGAAACAGCACATTTG	60.051	25	272	767	1038
60.088	20	TTTATCAGGGAAGGACGTGG	59.926	20	165	3064	3228
59.78	20	TCCGGACGGAAAGATTACAC	59.933	20	251	9	259
60.045	20	CTGTTATGTCGTGTCCGGTG	60.025	20	244	1066	1309
59.989	20	CGATTTGGCAAGGAAGAAAG	59.817	20	183	430	612
60.088	20	TGGCGCCTTCTATTCTCCTA	59.938	20	151	342	492
59.23	20	CCCGTACAATATCAACCACAA	58.28	21	180	61	240
61.072	20	GAAGTGTTATGCGTTTTGACAAT	58.249	23	235	1789	2023
60.103	20	ATCTCACGATATCCCGCATC	59.884	20	235	101	335
60.12	20	GAGGAATCTTCCCAATTCACA	58.97	21	277	14	290
59.766	20	TGAGACGAAACTCATGCGAC	59.992	20	246	48	293
60.029	21	TCCACATATGCAAGATTTCAGC	60.103	22	125	50	174
60.11	20	ATGAGCACCAAATCTTTGCC	60.081	20	153	203	355
59.829	20	ACGGATATGCCCTTGTATGC	59.813	20	249	23	271
60.812	20	AATCCACACCCGAAATATGC	59.651	20	157	375	531
60.008	19	CAAATGGTTTGGGGTTTTTG	60.06	20	271	116	386
59.245	20	TCGGCATAGATGACTTAAAAGC	58.544	22	205	1454	1658
60.134	20	CAACCGAAGCAATAAGAGGG	59.702	20	266	6	271
58.421	21	GCAACCAACATCAACGTGAC	60.016	20	251	553	803
59.576	27	AGAAAACTCACCCTGCCTCC	60.628	20	272	112	383
60.375	20	TTTAAGGGTTATCACCTGATTTTC	57.757	24	211	830	1040
59.972	20	GTTCGGTGAAGATTGGAGGA	60.05	20	225	31	255
59.964	20	AACCAAGATCAGGCTGCAAT	59.7	20	171	669	839
60.43	20	CTTAGGGGACACCCAAGTGA	59.959	20	276	382	657
60.46	20	GACTCGCAAAGACTTGAGGG	59.989	20	153	34	186
59.762	20	GTGACGTGCTGGTTGTCCTA	59.751	20	225	1027	1251
57.559	23	TGACAATTGCAAATATGGCG	60.474	20	217	3	219
59.96	20	GCTGCATCGATGTATTGTGG	60.104	20	130	1998	2127
59.813	20	GAATCTCTTGCCGGATCAAA	60.155	20	259	692	950

60.159	20	TCATTCTCGGGCAAAAATTC	60.016	20	271	52	322
59.749	26	GTCGCTACAAGCAGTCACCA	60.056	20	264	3	266
60.117	20	GCGACTTTTCAGTTTCCAGC	60	20	208	749	956
59.837	20	CATTTGCAGATGCTGATGCT	59.979	20	268	1014	1281
58.863	20	GCGTGTGTCTGAGGATGTTT	58.727	20	181	17	197
59.948	20	GCAAAATACCCTGAGTGGGA	59.933	20	153	307	459
59.429	20	TGGAGGTCGAACTCAATTATGA	59.569	22	157	209	365
60.246	20	ATTCATCAAACCCAGTTCGC	59.939	20	229	20	248
59.975	20	ATAGCCAAGGCAGCTCTCAA	60.118	20	231	1867	2097
59.82	20	ATGTGAACGAAGTACCCCGA	60.375	20	187	263	449
60.582	20	GAAAAACGCTAGGCGGATCT	60.711	20	214	23	236
58.931	20	TCACACTAGTGGCATCGCTC	60.016	20	203	212	414
59.269	21	AAAGCGCGATTTTAAACCCT	60.095	20	235	2183	2417
57.192	22	TGGGACATTTTTAATTTTGCCT	59.728	22	232	1480	1711
61.489	19	TTTTTCGTGGATATAATTGGGC	60.044	22	175	64	238
59.528	20	CACCTCGAGCTTCGCTTCTA	60.807	20	263	4779	5041
60.088	20	GAAGGGAAAGAACCTTTGGG	59.912	20	252	1288	1539
59.996	19	TGTTGAATCACATGTCCCGT	59.812	20	200	271	470
60.059	20	GCATGCATCTGTCGGATCTA	59.788	20	173	334	506
59.569	20	GGAAATAAGGAGTTGTTATGAAG(	60.434	25	134	2768	2901
59.031	20	CATGTTCTAGGCGTTTCGGT	60.132	20	268	81	348
59.847	20	TTTTACTTTGGCCGAATTGTC	59.111	21	118	290	407
59.915	27	TTAAATTTTCGAACGCCTGC	60.205	20	129	212	340
60.118	20	GAAAGTTCGGATTTTTGGCA	60.053	20	244	98	341
57.756	24	AGCTCCAATAGTTGCTTCGG	59.476	20	192	0	191
60.132	20	ACTGCGCACACTTTGTTTTG	59.951	20	191	2168	2358
60.035	20	AGCCGATTTCTTTCACTCCC	60.578	20	226	35	260
59.03	20	AAAATCAAAGGCCCACAATG	59.801	20	142	2168	2309
59.948	20	TGGCGTCTGGTAAATCAACA	60.111	20	247	1343	1589
59.688	20	GATTATGAATCGGGCGAGAA	60.001	20	242	1702	1943
59.844	20	TGCATTGCCATGTTACTTTCA	60.126	21	167	146	312
60.103	20	ATCTCATACGTTGCCAACCC	59.82	20	238	456	693
59.799	20	CAGCAACCAAGAAGGGATGT	60.111	20	277	2010	2286
60.388	20	AAAAGGGAGAAAGAGGCCAA	60.18	20	227	4090	4316
60.22	20	GGCCGGTTAAAGGAGAAAAG	60.068	20	195	21	215
59.9	25	GCGGAAATTAACCACAACCA	60.732	20	147	8	154
60.042	20	CGCGTTACGAATCCAAGTTT	60.131	20	160	639	798
59.74	20	CCTTCTCGAGCACTGATTCC	59.95	20	221	81	301
60.003	20	GGCGGAATCACATTAGCATT	59.929	20	230	268	497
59.982	20	TCTCTCTTTTGGTTGGTGAAGA	58.955	22	183	210	392
60.034	20	GGACGCATCAGGTATAAAAGGA	60.332	22	240	6	245
59.968	20	CGCATCTGATTTTTGCAATC	59.261	20	258	1598	1855
59.896	21	GAAGAAACCCCTTTTTCTTCTCTT	59.682	24	247	593	839
59.615	20	ATCATCACCACAACTCACCG	59.393	20	104	333	436
59.813	20	TCGGATGGTTTATGGATGGT	60.014	20	274	295	568
60.005	20	TGTTCATACGCATTGGAAAAA	59.063	21	156	188	343
59.752	20	GAACTCCCCTCTCTCTCGGT	59.807	20	203	76	278
59.449	20	ACGCCCCGTATTTTGAATTT	60.547	20	278	1431	1708
59.31	22	GTTTGGCCTTGCATTTGATT	59.945	20	161	1952	2112
59.476	20	CTCCACCAAGCTTACACAACA	58.837	21	177	608	784
59.978	20	GCGTTGTCGTTTCTTTGGAT	60.118	20	222	267	488
58.987	22	ATAGCCATTGGCCTAGCCTT	60.082	20	278	1638	1915
60.177	20	TTGCTTTTCCGAATCTGTGA	59.395	20	210	581	790
59.238	21	GCGTAGTCTTGTCTTTCGCC	60.022	20	191	249	439
60.683	20	CCCCTAAACCGTAAATCAAGC	59.851	21	207	2	208
60.088	20	TGAAGTTGATCAAGCAAGGG	58.847	20	201	1187	1387
59.853	20	AACATTAATGGCGTCGAAGG	59.96	20	264	4526	4789
58.941	21	CGATCGTAATTGATCTACGTCCA	61.213	23	266	91	356

59.847	21	GTTGGTGCCAAAATTGCTCT	60.118	20	226	2	227
59.647	20	ATTACGAGCCACGGCTACAG	60.292	20	228	72	299
59.984	20	ATTCACATTTTCCCAGTCGC	59.939	20	235	1081	1315
59.933	20	GAAAAGCCGAATGCAGAAAG	59.96	20	170	1118	1287
60.291	20	CGGTTATCACCGAATCGC	60.033	18	280	97	376
60.03	20	GGCAGTACGTGTGCGATAGA	59.895	20	105	80	184
59.305	20	TTTCCACAAGTGAAGCAACG	59.881	20	260	3659	3918
60	20	AAGGCTATCGTTGCTTTCCC	60.582	20	149	378	526
60.147	20	TACAAGGCCCGACTTGAGTT	59.734	20	246	10	255
60.005	20	CTTTTGCACCTTTTTGCCTC	59.861	20	230	474	703
59.789	20	ACCCTCACCTCCCCTCAC	59.888	18	248	13	260
59.875	20	GGAACTCCCCATAACTGCAA	59.933	20	181	388	568
60.492	20	TTACGTAAAAATGGGTTATTATTT(	57.411	26	164	491	654
60.791	20	AACGTGACATGTTGCTGAGG	59.751	20	200	838	1037
58.759	21	TCCGATGTGGGATCATAACA	59.732	20	119	358	476
59.669	27	ATTTCGAAGATCAACGGACG	60.074	20	140	9	148
60.163	20	AAGGAGGTGTTATGAAGGGATT	58.028	22	249	120	368
60.134	20	CCCCATGTAAATAGCCGTTG	60.202	20	254	24	277
59.903	22	TGATTTGTTGGGGCTTTAGG	59.931	20	251	471	721
59.948	20	AAAACAAGAATTCGGCATCG	60.074	20	182	283	464
60.009	20	CCACCATCTTGTAGGTCGCT	60.134	20	123	1808	1930
59.335	22	TGAGTTTGCATCTAAGGAGGTG	59.378	22	269	503	771
60.156	20	TGAGGATACTCGATTTCACGG	60.081	21	135	362	496
59.83	20	GAGAAGGGTTATCACTTGAATAG(	57.473	24	267	87	353
59.898	20	ATTTCAAGGTTGGGCATCTG	59.933	20	243	2703	2945
60.817	20	CGTTTAAGCCAATATCATTGTTCA	60.247	24	279	477	755
59.767	20	TTTCAACCTCGCTATCGCTT	59.982	20	182	269	450
60.005	20	CATGCGCATTTATGAGTCCA	60.632	20	212	120	331
59.405	20	GAGGAAAACAGAGGCTGTTCA	59.463	21	138	84	221
59.96	20	TCGAAGTTTGGTGTTGGTGA	60.128	20	197	49	245
60.255	20	CCACGTTAGGCTCAGGAGTG	60.843	20	103	4	106
57.853	21	TCGTATCGCTTCCATGTTGA	60.22	20	227	5	231
60.278	20	TTCAACATTTCTTGCCCAAA	59.133	20	201	339	539
59.837	20	TTCTTTTGGGGCAGTGAATC	60.051	20	195	577	771
59.685	20	TTGTTTAACTCCCACTAATGCAAA	59.937	24	208	25	232
60.257	20	TGTTTCCATTTCCATGGTACG	60.602	21	261	2248	2508
60.025	20	GATGATAGAAGGGGGCATGA	59.854	20	279	1905	2183
60.049	20	AATCCAGCAATTTGCCTTGTA	59.6	21	277	2502	2778
60.246	20	CAAAGTCCGGCGTAGAATGT	60.132	20	220	344	563
60.02	20	TTGAGTCCCAAAACATGCAA	60.088	20	278	773	1050
59.017	22	CATGGCATGCACATTTTAGC	60.103	20	224	1266	1489
58.547	20	GAACTCGGATCTTTCACCCA	60.05	20	198	754	951
58.277	20	TGGTGGGAGCCTATAGCAAC	60.096	20	254	18	271
59.813	20	GCATTGGGACACCTAAGCAT	59.962	20	225	529	753
60.006	20	CAATATTACAATGGCCAAGAGTC	57.725	23	185	62	246
59.401	20	TGAGAAACTCCCTTGGGTGA	60.624	20	128	314	441
59.566	20	TGGTAGGTTTGTTTTAGGCCC	60.223	21	202	4	205
58.497	21	CCATGTTAAAACGGGCACAT	60.617	20	106	37	142
59.871	20	TGAAACGGACAAACGATGAA	60.088	20	146	159	304
59.866	20	AAAAGGGAAAAACCCTCAGC	59.571	20	150	410	559
59.982	20	AAAATACCCTTCCCCGAATG	60.013	20	177	83	259
59.817	20	AAGGGTGATGTCGCACTTCT	59.727	20	279	43	321
59.971	20	GGATGATGCCTTCAAGGGTA	59.894	20	236	460	695
60.578	20	ACACCCAACTCAGTCAAGGC	60.159	20	237	587	823
59.956	24	CATGCTAGAGGTGACGTTTTG	58.445	21	231	1631	1861
59.55	20	TTTTGTCTTTTGGGATTCGG	59.91	20	207	2087	2293
59.81	20	ATCCAACCCATTTGTGGAAA	60.029	20	279	332	610
60.002	20	CTAACATCGGAACTTGCCGT	60.132	20	259	592	850

60.103	21	TGGTGTACAGAGTGCATGTGA	58.719	21	279	174	452
59.246	27	GCGAATATAACCCGCTCTGA	60.196	20	219	44	262
60.134	20	CCTTGGGCGTTGATCTTAAA	60.067	20	119	99	217
60.457	23	CAGGAGAGATAGGGGGAAGG	60.023	20	263	18	280
59.962	22	CATCATCACCTGATTGACGC	60.08	20	261	42	302
60.024	21	GCGCAGATCTAATGGTCCAG	60.765	20	179	204	382
58.81	24	AGGTGGCAACATAATCAGCC	59.962	20	149	9	157
59.962	20	TTTCGCACGCATTAAATCAG	59.842	20	167	335	501
60	20	TGTGTGATGTTGGAGTTGGG	60.416	20	229	52	280
59.813	20	TTCCACGTTCGTGTTCGATA	60.111	20	232	239	470
60.081	20	TTATATTTCAGGGGGAGGGG	59.971	20	180	250	429
59.874	20	TTCCTTCACTCCCCCTCTCT	60.186	20	221	1849	2069
59.619	27	TTCTTATTTCTCACTTTAAGACCAT	58.093	27	216	451	666
60.119	20	AAAAACCCTTCTGAAACCCG	60.324	20	171	414	584
59.83	20	AGGCGATTTTGGGTGTTATG	59.823	20	251	71	321
59.486	20	ATGGGATTCACCTGCCTTCT	60.853	20	138	8	145
60.088	20	TAATTGTTTCGGGCTAACGG	59.958	20	208	200	407
60.517	20	ACCCACCAACATCACCAAAT	59.95	20	219	57	275
59.559	20	GGGCGTTTTATTTCCCATCT	60.15	20	243	157	399
60.081	20	TCATTATACGGGCCCAAGAC	59.784	20	231	44	274
59.756	20	TCGCCTAACTTATGCAATGAAA	59.753	22	157	230	386
58.03	20	TACATCGTCCACCCCATTTT	60.051	20	273	147	419
59.55	20	TCGTGTGGTTTTGAAGCAAG	59.881	20	238	264	501
58.402	23	ACCACCGGCTAAACCAGTC	59.981	19	208	17	224
59.864	20	GATATGATTTTGCGCGGAGT	60.067	20	230	28	257
59.783	20	CAATTTTCGGCAGCATTACA	59.702	20	237	351	587
60.152	20	TTTTTCCCAAAACACCCCTA	59.288	20	202	1262	1463
60.212	20	TAGAGTTCCAAGAGGGGGCT	60.204	20	209	4	212
60.286	20	GGCAACAAGAAGAAAATCGC	59.829	20	253	390	642
59.837	20	CACTACTCATCATCCTCTCTTTCTC	57.758	25	144	904	1047
60.448	20	CTGCAGACGTTTCAAGGTGA	60.025	20	211	736	946
59.126	23	TCATTTATGAAGCAAGGGGG	59.894	20	105	33	137
60.386	20	AAGCTTGGGCTTTCTTCTCC	59.962	20	235	3881	4115
59.871	20	CAGCGAGATCTTCGTCCATT	60.362	20	131	126	256
59.963	21	TGGCGTCTGTTCCCTTAATC	60.074	20	198	23	220
59.923	20	CATGCCAACCCGTCTTATTT	59.823	20	238	2420	2657
59.079	20	GGGTGGAGCAAAATCTGAAA	60.051	20	217	40	256
59.845	20	CAAGCAAATTGTGGCTCTGA	59.988	20	227	500	726
59.682	20	GATCGGTGAAGATTGGAGGA	60.011	20	210	11	220
60.195	20	GCGAAAGACATACGGGGTTA	59.96	20	250	44	293
60.448	20	CAGAGCGCACTCGAAGAGTA	59.49	20	276	63	338
60.603	20	ATCACGTGCACCGTCATCTA	60.144	20	185	320	504
59.807	20	CCACGAGATCAAGAATGGAA	58.643	20	250	1	250
60.195	20	CCCGTTTCGGAGTTTGTCTA	60.103	20	233	1097	1329
59.174	20	TTCAGCCCACATATGTTCCA	59.924	20	104	36	139
61.031	20	ACCCAACCCACGCAATAAT	60.066	19	272	20	291
58.038	20	ACAATTCCACGGAGAACCTG	59.966	20	248	773	1020
59.411	23	TCCATGGATCTGGATCATCA	59.806	20	249	117	365
57.929	20	TTGGGTTGCTGAAAATAGGC	60.074	20	237	27	263
59.696	20	AGGCCCGAGCTAAAAACATT	60.095	20	146	62	207
59.848	23	TGCCTAGGTGGCTACTATGTGA	59.791	22	169	30	198
60.173	20	TTGTGATTCTGAAAAATAGATCGT	58.337	25	204	321	524
60.134	20	ATCGAAAGACGAACACGCTT	59.882	20	220	585	804
59.823	20	GAAGATACTTCATGCTTGTAAAAC	58.527	25	185	358	542
60.237	20	TGTGGGACAAGCAAATGAAA	60.088	20	280	466	745
59.9	20	AACATGCACTTTCATTCGTGTT	59.545	22	219	59	277
60.336	20	AAAGGTAGCGGGGTCTTTGT	59.998	20	117	334	450
60.162	20	GCAAGGTTCATGGAGTGGTT	59.973	20	233	202	434

59.64	23	CCTAACCCCACTAATGACCC	58.24	20	143	3	145
57.833	20	GGTTGACGTTGGATCTGCTT	60.119	20	214	146	359
59.933	20	ATTTGGTCTCTTCCCCTCGT	59.935	20	269	680	948
59.797	20	CATGTTCCGAATCTTCGTCC	60.461	20	171	4	174
59.938	20	TGTGGCCCGTTTCTTATTTC	59.938	20	177	28	204
60.015	20	CGCATATAGCTCGACTGCAA	60.14	20	136	269	404
60.117	20	AAACCCTTGAAACAAATAAAATCA	58.132	24	259	45	303
59.539	22	TGAGTTGGTGGAGGGTTTTC	59.943	20	218	102	319
59.671	21	TCGAATTGAAGTTAAAGCTTAGTT	58.353	25	132	275	406
59.161	20	GAAACGAGTTCCGAAATGGA	60.051	20	220	47	266
60.081	20	TGAAGCCTTTAAATGGGCAC	60.074	20	204	377	580
60.591	20	CACCCATTCATTCCCTCATT	59.605	20	136	198	333
59.803	20	GGACACAACAGAGGGGAGAA	60.088	20	237	352	588
59.962	20	GGCGAATGCTATGCATTTTT	60.068	20	260	683	942
59.934	20	TTCTTGGTGGCGGTAGGTAG	60.125	20	178	223	400
60.284	20	GGTGTTGGCCGAAAGTTTTA	59.975	20	107	0	106
59.91	20	TGCTCTCTCATTGTAATCTCTCTCA	59.688	25	153	836	988
60.017	20	ATTGTTGTGGTGCGTTTTCA	60.011	20	236	415	650
59.927	20	TCCGATTGACACCTACACCA	59.96	20	167	1163	1329
59.967	20	GAGGGGGTGACCACAATCTA	59.779	20	229	211	439
60.045	20	TTCACGCAACACAAACCCTA	60.149	20	240	131	370
58.359	20	TTGGGGGAGGGTTTAGGTAG	60.172	20	121	12	132
60.065	20	AAAGAGAGCTGCAAATGGGA	59.955	20	152	44	195
59.894	20	ATTGGGGAGGATAAAGGACG	60.145	20	140	37	176
60.032	20	CCTGGAAACAAAGAAGCACC	59.711	20	275	706	980
59.982	20	CAGAGCCCTATTCATGCGAT	60.199	20	158	12	169
60.163	20	CTCCAGCAATTTCAGCTTCC	59.955	20	174	73	246
59.668	20	CACTTAAGGGGGTTATTAGTGGAA	59.692	24	150	1203	1352
59.816	20	GCTGCTGTATTAAACCGAACG	59.793	21	258	510	767
60.13	21	TAAGGGTTATCACCGGATCG	59.778	20	231	8	238
59.78	23	TGCTTTAATCCATACAAAGACCC	59.407	23	128	14	141
60.454	25	ACGCCTGCTTGAGAGATTTT	59.08	20	220	8	227
59.947	20	TGAGGGCAGTCAAAAATTCG	61.137	20	131	86	216
60.062	22	TGGTTGAAACCAGATCGAAA	59.097	20	275	1348	1622
57.122	24	GTCCACGGGAGAGAATGAGA	60.199	20	178	5	182
59.267	21	CCCCAAACAACGAAATGAAT	59.662	20	193	221	413
59.738	21	CGAGGAAGAAAATGGAAGCA	60.323	20	178	194	371
59.154	23	TTGTGGCATTCTTGACCAAA	60.088	20	177	231	407
59.992	20	ACGGTTCCCATCAGCAATAC	59.82	20	262	732	993
60.05	20	TTCCCCTCCACCCTCTATTT	59.761	20	269	334	602
59.83	20	TTGTAATCGGACCGGTTTTC	59.801	20	217	380	596
57.782	25	CACCTTCTGGACTCAGACGA	58.964	20	267	250	516
57.515	20	GAGCGCATTCGAAGAGTAGC	60.265	20	256	69	324
58.404	20	ATATCGGCCGTCAGATTGAG	60.059	20	216	325	540
59.726	20	ATAGGGTCATGCGTGTGTGA	59.992	20	169	98	266
60.301	20	TGCGTTTTGACGATATATAGTGTTT	59.092	25	169	1279	1447
59.013	20	CCCAACTTTTCTCCCCAAAT	60.159	20	256	1027	1282
59.05	20	CAAACCAAAGCATGCGTAAA	59.742	20	183	69	251
59.563	23	AAGTAGTCCTTTCCTCGGGC	59.713	20	241	125	365
60.184	20	TAGCCGAGAAGAACCTTGGA	59.948	20	183	79	261
60.003	20	TCCTGCCACATGTAATGGAA	59.924	20	147	6040	6186
60.03	21	AGATTTCACTTTGCAACCGC	60.257	20	243	2850	3092
60.051	20	AACAAACAAGCCCCCTCTTC	60.476	20	177	554	730
60.074	20	GCCTACCTGGGCCATTTTTA	61.155	20	230	12	241
59.445	20	TTTGCATCTTAGCGAGTTGG	59.062	20	253	936	1188
59.279	20	CGTATTGAATGAGGGAGGGA	59.887	20	214	406	619
58.268	20	TGGAATTTGGTTTTTGTAACTTGA	59.797	24	250	267	516
57.687	25	TTAGTAGCTTCGGGGTGCAT	59.73	20	235	200	434

59.457	20	AATCTCCATCCAAGCACCAC	59.934	20	198	470	667
60.448	20	CGAGGAATACGAAGAGTCGC	59.978	20	200	42	241
59.365	22	CATCGAGCCCACTTAGTGCT	60.419	20	248	991	1238
60.278	20	ATTGAGAGAATGCGAATGGC	60.185	20	274	19	292
59.473	21	GGGACGAAAATAACCCTCGT	60.187	20	215	706	920
57.647	21	GGAAATAAGGAGGTGTTATGATA	57.235	25	129	1309	1437
60.119	20	GGTTTTCTTGCCAAAAATGG	59.429	20	205	433	637
60.812	20	CGATCCATATGGCAAGAGAAA	60.045	21	226	12	237
60.478	26	GCGGAAGAATTTGCTCTCAG	60.096	20	232	20	251
57.608	20	ATTTCATTAAAATTGATCAACGG	57.193	23	122	22	143
60.048	20	ATCGGCGCTAGACGACATAC	60.265	20	145	1335	1479
57.024	26	GGTGTGTTGGCATTGCTAGA	59.722	20	193	1015	1207
60.773	20	AAGGAGGTGTTATGAAGGGATT	58.028	22	198	236	433
59.83	20	TTTTCCTCTCCCCTCTTGGT	60.045	20	274	319	592
59.934	20	CGCTGGTCTTGGAGATCAAT	60.218	20	251	1140	1390
59.101	21	TCAATTACATTCCCGTGGGT	60.051	20	235	1252	1486
59.006	20	TCAACAGCAATGAATTTGTGG	59.576	21	244	133	376
60.04	20	TCAGCTGCGATGTCAATTTC	59.955	20	110	94	203
60.493	20	CGGTGACGGTTGAGAATGAC	61.527	20	152	51	202
60.61	20	AATTGTGTGGGCGATTGATT	60.199	20	278	3737	4014
59.95	20	AGCGTATTTCTTGATTGCCG	60.23	20	250	575	824
60.058	20	TCCCTATTCCCACTCCTTCC	60.264	20	191	657	847
59.887	20	GCGTCTCTCGACGTTACACA	60.056	20	225	64	288
58.866	20	TGGAGGAAGATATTGTGACGTG	59.993	22	269	2123	2391
60.096	20	TCAGTGTTATCGAGCCTCTCAA	60.015	22	278	17	294
60.366	20	TCGGGATATTAAATCCATCAGG	60.002	22	128	220	347
59.823	20	CGCTTTCACCACGAACACTA	59.904	20	246	64	309
59.481	20	AACACACACGGATGTGGAGA	60.005	20	231	5852	6082
60.032	20	AAAAGAGAAGCCCTCCAAGC	59.962	20	230	551	780
59.83	20	CGAAAAAGCAAATTCCATCA	58.744	20	273	9	281
58.658	23	CGAGTGGTTTGGGAAGAAGA	60.224	20	262	0	261
60.042	20	AGGGCCAGAAAAACCAAGAT	59.94	20	268	349	616
59.577	20	TGCTGGGATACACAGGGTTT	60.375	20	172	96	267
57.73	23	ATGCCCTCATGACTCTCAGC	60.381	20	232	16	247
58.988	26	TTTCATGATCAAATCTTATGAACC	59.741	25	156	30	185
60.047	24	TTGAAACGAAATAGGGGTGG	59.795	20	267	532	798
59.933	20	TGTTGGTGGATTGTGCATTT	59.823	20	176	123	298
59.797	20	TTTAAGGGTTATCACCTGATTTTC	57.757	24	212	569	780
60.134	20	CACCTCCCAAACACAAAGGT	59.861	20	142	3943	4084
59.622	20	AGGTCTTGACAGGTTGTGGG	60.002	20	262	27	288
59.691	20	AGATGAGATCAATGCCCCAG	60.034	20	138	3188	3325
59.975	20	CCAGTTCAAAAGCATCACCA	59.691	20	234	1253	1486
59.962	20	ACATCCAGCCCACAAAGAAC	59.973	20	161	3047	3207
59.94	20	TTGCCATGTTCTGCTTCTCTT	60.008	21	240	1053	1292
59.907	20	AAAGAAAAGAGACAATCCGGC	59.717	21	252	504	755
59.871	20	GAACGAATGCATGGGTGTAA	59.4	20	261	886	1146
59.879	20	CAGAACCCTCAAATCCCTCA	60.042	20	215	8166	8380
57.268	20	AGAAATGGGGTCGACATGAA	60.317	20	232	43	274
59.716	21	CAACGAAGGATTTGGTGTTG	59.021	20	274	3247	3520
59.989	20	GATTGGACTTGTTTCTCGGC	59.676	20	228	30	257
60.032	20	TCTTCCAAGAATGGAGCAGC	60.483	20	183	962	1144
61.706	20	TCATTTGCCCCCGTATTTTA	60.144	20	177	0	176
57.785	25	CCACGAATCATGATCGAAAA	59.474	20	229	2331	2559
59.982	22	AAGATATATAATTCATTGAGCTTT	57.982	27	105	175	279
57.749	22	TGATTTCGAGAAGCAGTAGGTG	59.517	22	114	237	350
60.103	20	ACCAGAACCGAAGTGATTGG	59.966	20	238	627	864
60.11	20	TGGAAGGATTGAAGGAGGTG	60.042	20	224	1929	2152
59.282	20	CACCTTAAGATCTGCTCCGC	59.978	20	240	125	364

60.461	20	GGCAGTCACTGGAAGGAAGA	60.386	20	275	1161	1435
60.008	20	CACCCGAAAATGAGTTATTATTTG	60.438	25	166	242	407
59.598	20	GGCATTGCAGAGTTGAGAAA	59.002	20	144	4778	4921
59.967	20	TGCGTTTTGACGATATATAGTGTT	59.092	25	253	266	518
59.967	21	AAAATAGCTTATTGAATTTGATTT	59.723	27	195	108	302
60.183	25	GCCAGATGTGAATGGGTCAC	61.369	20	184	130	313
59.942	20	TCCCATTTCCATTTCCAAAC	59.599	20	156	494	649
59.933	20	CACCACGGAAGAGAGGTAGG	59.715	20	168	65	232
58.148	20	TCCGATTAATGGGCTCTGAC	60.036	20	117	1744	1860
60.089	21	TTTTTGCCTGATATTTGTATAACGA	59.061	25	273	891	1163
59.605	21	AAAGTTGAAGGTTGGGGAGG	60.332	20	257	13	269
57.486	27	TTCTGTCGAGCTTAGCCTCA	58.9	20	173	9	181
60.11	20	TCTCTCCCTCTCTCTCTTCGG	60.215	21	150	122	271
59.31	26	GGACGCATAACCCGTAAAAA	59.832	20	109	2	110
59.874	20	TGGAAGATCCGAGAACCACT	59.655	20	265	1121	1385
59.919	20	TTGTTGAACCGTGTGCAAAT	60.011	20	225	23	247
59.937	20	GCTGACATGGCTTCGTATCA	59.83	20	269	1702	1970
59.711	20	CAAAACAACTGCACCACACC	60.049	20	219	837	1055
60.05	20	GTCAACTTGGGCAGTGGAAT	59.973	20	176	51	226
59.592	20	TGTGTGCTTTATTGGAGCGA	60.401	20	277	10	286
60.067	20	GGATTTGCACACCCCTAAGA	59.933	20	277	217	493
59.717	24	GAACCGCCGGAAAAGAAT	60.014	18	268	5	272
59.967	20	CGGGAGGAATATCGCACTTA	60.053	20	202	954	1155
59.968	20	AGCCTAAGTAATGGCGCAGA	60.003	20	145	228	372
59.131	24	GAAGCCGGGTGTCAAATAAA	59.938	20	263	521	783
60.008	20	CAATGCCTAGTTTACCCCCA	59.817	20	252	644	895
60.365	20	TCCGGCCAAATTAAAAGAAA	59.538	20	105	778	882
60.111	20	ACCAACCTCCATTGAAGAGC	59.141	20	199	91	289
58.652	22	TATGCTCGCTGGATTACGTG	59.856	20	251	506	756
59.247	20	TTCCGTCTTTAGGCATGAGC	60.352	20	266	6	271
60.133	20	GACCCACGACCGTGTACTTT	59.891	20	263	66	328
59.434	20	TGTTGCCGTAAACAATGGTC	59.444	20	194	203	396
60.066	20	CAGATGTGGACGGGTCACTA	59.545	20	220	29	248
59.21	20	TATCCATTTGCCCTTTTTGC	59.907	20	249	226	474
58.145	20	TGGTTCGATCGTAATTGATCT	57.168	21	129	20	148
60.073	20	TCTTGGTGGGATTTGGAGAG	60.042	20	179	162	340
59.711	20	TCACTTCTTGGAGTTTGGCA	59.415	20	133	73	205
59.92	20	CAACCTCAGCTTCTTCCCAG	59.982	20	192	147	338
60.012	20	TGCAACCATTTTGTTGGAAA	59.946	20	247	37	283
60.172	20	ACGGATCGGAAATCCATAAA	59.23	20	212	270	481
57.239	26	CTTCGATGGAGTTCCCTCCT	60.586	20	187	7	193
59.903	20	ATGGGGTGAGACAAAGCAAG	60.111	20	272	121	392
59.926	20	AAAAGAGCTTGGGCCTCTTC	59.962	20	248	772	1019
60.096	21	CCGACACAAGCTGAAGTGAG	59.616	20	152	42	193
59.991	20	ACCCACTTTTCAAATCAGCG	60.11	20	125	2854	2978
60.081	21	GTTTTGGCATGCTAGTGGGT	60	20	146	70	215
59.894	20	CCCAATCTTTTCCACCCTTT	60.159	20	145	86	230
59.774	23	AAATTCCCGAAGAGCCATCT	60.039	20	258	49	306
60.265	20	TTCTCTGTTCTGGCAGTGGA	59.545	20	204	109	312
59.779	20	GGGCATGCCAAAAATATCAC	60.161	20	275	967	1241
60.118	20	CTGTGAAATTGCACGGCTTA	59.872	20	116	102	217
60.014	20	AGTTCAAGTTTGGGGGTGTG	59.861	20	189	343	531
59.831	21	TCAAGATTTAGGGGTTATCATTTG	58.508	24	113	170	282
60.05	20	CTTTCCTTGGGGACTGTGAA	60.081	20	195	5224	5418
59.978	20	TTTTGGCGGGAAAAATAGC	60.026	19	159	322	480
60.331	20	CTGAATTTGGAAGGAACGGA	60.044	20	248	523	770
59.756	20	CCTATGGTGGTATGGTGTGG	58.569	20	207	93	299
59.173	22	GTGGACGCTCCAAATTGTTT	59.978	20	143	63	205

60.288	19	GGCAACAGCTCTTACCGTTG	60.827	20	279	131	409
59.724	20	CACATCCTCACACACTCACACA	60.697	22	241	2630	2870
60.954	20	CCAACACAAATGAACCTGGA	59.389	20	215	19	233
60.011	20	ATTGAAACCGTGAAAACCGA	60.344	20	232	865	1096
60.439	20	CACGAACGGTATAACATCGG	58.917	20	272	706	977
60.119	20	CCCTCGTATGCAATGAATCTG	60.475	21	266	357	622
60.255	20	GTGAGGGGGTGACAGAAATG	60.363	20	268	39	306
58.851	20	CTGATTCGCTAGCTTCCCAC	59.978	20	119	20	138
60.721	20	GGAACCAAGTCTTCAGATGC	57.288	20	260	44	303
58.884	22	ACCGACAAAAGGTAGGCTCA	59.734	20	229	267	495
59.682	20	GCATCGTGTGAAGCATTGAT	59.685	20	138	219	356
59.273	20	TTGAAGGGAACGAGACCAAG	60.224	20	252	51	302
60.517	20	GGATTCCATCAACACCAACC	60.034	20	160	60	219
58.057	21	CTCATTGGAATTTTCCGGGT	61.042	20	269	914	1182
60.017	25	GAGCATTTGATGGAGGATCG	60.57	20	239	30	268
59.84	20	CTGCCCATTTTACCCTTTTT	58.113	20	111	866	976
59.931	20	CATTTCCTTCAAGCACACCA	59.691	20	247	102	348
60.885	18	CCATTTCAGTATTTGCCGGT	59.823	20	278	357	634
60.327	20	ATTTTCTGGCGACGGTACAC	60	20	158	1833	1990
59.706	20	ATGTTATCGTGTGGATGGGC	60.613	20	234	358	591
59.013	20	TCCCAATGATTGTTTGGTCA	59.75	20	213	750	962
60.14	20	CACTCACAGCCAACAGGGTA	59.745	20	148	1268	1415
59.801	20	TTCCAATTGCGAAAAGATTTG	60.061	21	180	62	241
59.779	20	ACTCCCAATGGATCGCTATG	59.917	20	245	2	246
60.134	20	TGAAAGCAGCATAAGCATGG	59.976	20	143	1304	1446
59.993	20	ATCTGGAAAACACGCCACAC	60.966	20	209	168	376
59.96	20	ATCCAAACGGCGAAGTGATA	60.469	20	187	60	246
59.976	20	TCCGGCAATCTCCTTAATTG	60.031	20	160	290	449
59.23	20	CGACACGAATCCAAAACTGA	59.691	20	276	1444	1719
60.058	20	TTGCAAACATTTTGAAGGACA	59.192	21	190	275	464
59.752	20	ACTGGACGGAAATCGGAAG	60.058	19	256	275	530
59.95	20	ACCACTGCATTCACTTTCCC	59.973	20	264	949	1212
58.676	24	TTGCTCGCGAGATTTCTTTT	60.096	20	269	649	917
60.11	20	CATGCCAAGTGGTGTGATCT	59.551	20	216	390	605
57.197	24	GGATGAGGTTGTATTGAAACTGC	59.891	23	142	59	200
59.917	20	CCCTAGCCTTTGCCTCTTTC	60.332	20	164	190	353
58.9	20	AGCGTTATTTTGCAAGGTCG	60.264	20	117	6	122
58.37	22	GGTGAGGAGGAACACGAAAA	60.088	20	253	14	266
57.719	22	GGGTGGCCCTCAATTTTATT	60.02	20	270	32	301
60.021	20	AACGGGGAAACATACGTCAC	59.717	20	209	896	1104
60.029	20	GAGCCTGACTTGCTTTCACC	59.997	20	256	299	554
60.36	20	TTTCTCTCTTGGTTGGTGAAGA	58.955	22	211	22	232
60.05	20	GCACCTATATACGCACGCAC	59.238	20	258	11	268
59.536	23	TTGACCGATCTCATTTGAAAAA	59.564	22	253	61	313
59.923	20	CCAAAATCACAATCTATCAATCCA	60.076	24	222	6193	6414
59.883	20	CGTATACTTGCGGGGTTGTT	59.883	20	223	657	879
60.603	20	TGAAATAGTAACCGCCGACC	59.96	20	244	174	417
57.238	25	CAAAGGCTCGGAATATGCAC	60.606	20	149	0	148
58.978	21	GATCAATGTTTGACGTCTCGAA	60.118	22	226	94	319
59.348	21	AACCCACTACCCATTCCCTC	60.052	20	142	0	141
59.877	20	TATTTGCCACCCAGAACACA	59.964	20	243	548	790
60.125	20	TTGAGCGAAAAGCCAAAGAT	59.96	20	152	3441	3592
57.439	21	TCAACTTTCGGAAGCGTACA	59.464	20	113	8	120
59.873	20	TGTCCCGTTTTCCTTGTTTC	59.948	20	242	117	358
59.787	20	CGAACATCGTTGCTGCTCTA	60.157	20	216	450	665
59.653	22	GAGGACGCCCAATGATAAAA	59.901	20	219	925	1143
57.054	25	CCAAACATGCTTCTCATGCT	58.877	20	210	21	230
58.412	23	ACTGGAAGAATTGGAGGGCT	60.074	20	207	105	311

58.963	22	CTTAAGCATGCACGACTCCC	60.796	20	262	745	1006
60.066	20	CTTCTTCCCCGTTTTGTTCA	60.081	20	259	1002	1260
59.871	20	ATTCCTCCTTTTCAGCACCC	60.443	20	275	10	284
59.9	20	TCCGTCTGCTTGGAGAACTT	59.989	20	232	866	1097
59.999	20	CCACTCTCTCTCCTCTTTCTCTC	57.98	23	151	641	791
60.268	20	GCCTCTTGAAACTCTGCCAC	59.997	20	274	269	542
60.074	20	TTCGCACGCTATAAAACAAGAA	59.916	22	130	668	797
60.388	20	CCTCCAGACGTGAAGAAACC	59.697	20	266	1520	1785
59.957	20	TGCCTCATTTGTGTTAATTTGG	59.868	22	231	41	271
59.931	20	CCTTTCCAGGAAGATTGTGG	59.521	20	189	73	261
60.386	20	TTTAACCTGTAGGGCACCCA	60.357	20	200	1611	1810
60.895	20	TTGCGCCTAAGGAGGAGTTA	59.975	20	247	1938	2184
59.52	20	GGATAGGCTAGATGACGTAGATG	57.085	23	216	112	327
60.036	20	TGCATTGGAAAAAGTTGTGG	59.561	20	240	309	548
59.934	20	TGGAGTTTTTGCTTTCTGGG	60.22	20	274	1719	1992
59.88	20	GGGAGTCTTTCATGCCACAT	59.934	20	157	230	386
59.88	20	TGGAGTTGGTGGTGTCTTCA	60.129	20	145	1030	1174
59.319	22	TTGGAACGCCACTAGTTTCC	60.11	20	276	116	391
59.791	20	GGTGTGTTAAATGCGTGAGG	59.05	20	272	3166	3437
57.063	27	CACCGTGCATTACAAGATGA	58.129	20	182	8	189
59.835	20	CAACCCTACCTCCTTCTCCC	59.928	20	199	18	216
58.749	20	GAAATCTGAAGTTGTTCCCGA	59.16	21	268	57	324
60.156	20	GAGGCGCGATACACTACACA	59.895	20	194	100	293
59.873	20	TCTGAAAATCAAAGGCCCAC	60.051	20	190	2038	2227
59.239	20	AGTGGGACCAAAATGCAAGT	59.454	20	231	368	598
61.136	20	AAGCTGTTGACTTCAGCGGT	60.058	20	269	10	278
60.214	20	AAAGGGAAAAGACAGCAGCC	60.743	20	272	94	365
59.871	20	CCACATATTCATTTCCCCCA	60.384	20	110	2405	2514
59.829	22	TGGCATTTCAGCTCTTGTAGC	60.537	21	203	26	228
59.966	20	GGCTGCAGAAGTGTTGATGA	59.992	20	201	4485	4685
60.066	20	TGTTCAGCTATGTTTGCCGA	60.401	20	144	88	231
59.773	20	GTTGGGGGTTGGGTTATCTC	60.422	20	235	0	234
59.547	22	CCCAAACTTGGGGAAGAAAT	60.159	20	180	741	920
58.775	21	CGATCGTGAGAGGGAGAGAG	60.088	20	261	108	368
59.67	20	GGTGGGTGCCTAGGTTTTCT	60.361	20	185	83	267
60.051	20	ATACGAACACTGCACCACCA	60.032	20	119	529	647
60.114	24	CAAAATTAGTGTCAGCACCGAA	60.166	22	148	1493	1640
59.803	20	GCAATATCCTCTTCAACGCC	59.674	20	267	149	415
60.035	20	CCATTCCATGTCACAAACGA	60.367	20	149	789	937
60.155	20	CAATCTTAATTCCGTCCCCA	59.756	20	127	492	618
59.923	20	CTCGGGTCATTGGATTGAGT	59.927	20	232	43	274
59.859	20	AAGGGAAGGGGAAGTGAAGA	60.045	20	217	98	314
60.005	20	GAAAGGAAGAATGCGAGTGG	59.813	20	109	383	491
58.955	22	TAAGTTGGAGTGGAGTCGCC	60.255	20	224	21	244
59.006	20	GGTTCAAAAATCCGGGAAAT	59.999	20	217	467	683
59.774	20	AAAATGCAGCAAAGGGTCAC	60.118	20	278	231	508
59.34	23	CTTTGTGACAGGGTTCCGTT	60.005	20	146	43	188
59.946	20	CCTCCTTCTCCCAAAACCTC	60.045	20	105	5	109
59.985	20	TCCCTAGTTAGCTCCTAGATATTG(	59.225	26	204	192	395
59.966	20	ACAAAGTGGGAGGGCTTTCT	60.11	20	173	2759	2931
59.533	20	CAAGGATGAGATGAGGGCAT	60.034	20	161	70	230
60.323	20	ACCTATGGTGGTATGGTGTAGAG	57.076	23	256	22	277
59.433	24	CACATTTCCCTCTCCCTTCA	60.042	20	219	42	260
60.46	20	GGGAAAATAGCTACTCCCATTT	57.766	22	267	588	854
59.668	21	TCTTGAAAACTCCAAGCCCA	60.745	20	135	1	135
60.153	20	GGATTTCACCTTTGGATGGA	59.727	20	147	33	179
59.881	20	TTATTTGCACGCGCAGTAAT	59.379	20	264	25	288
58.816	23	CGGCTTTCTCTATCAGCCAG	60.11	20	240	8	247

58.068	23	CCTCACCACCTCAAGCTTTC	59.844	20	199	119	317
60.323	20	GAGTCCAATTGAACCTCCGA	60.05	20	224	1094	1317
58.992	21	CACCAACTGCTTTATTGCCA	59.729	20	235	469	703
59.577	24	TGATGAACTCGTCAATTGGAA	59.112	21	241	430	670
59.369	20	TGTTGGGTTGGTTTGACAGA	59.976	20	239	22	260
60.096	20	CACGTGTGGACATCCATATCA	60.256	21	271	87	357
57.092	21	ATGCCGTGTAAATCCATCAA	58.846	20	280	445	724
59.916	20	TCGGACCAAATTACCCTTCA	60.301	20	277	22	298
59.901	20	GCCAAGAACAGACCGAATGT	60.119	20	113	84	196
60.032	20	TTTGCAAACCCTACTTCACTCA	59.785	22	249	52	300
60.203	20	TCTCGAGATATTACTGGCGTGA	59.867	22	187	2	188
59.939	20	GCCCTATCACAACTCCGAAA	60.074	20	276	360	635
59.743	21	CGATATAAGCAGTCGCCACA	59.856	20	269	0	268
60.17	20	TCGAAATCAGCATATCAAAACAA	59.616	23	268	42	309
59.215	23	ATTGTGCCCCTATTCAACCA	60.192	20	209	1	209
57.643	26	TTTGTGTGCATGGAATTTTTG	59.453	21	244	3	246
58.961	23	CACAAATTTGGGTTGCCAG	59.949	19	161	135	295
60.203	20	GAATAAATGCAATCACGCCC	60.297	20	262	398	659
59.411	20	CAAAATTTGGACGGGAGAAA	59.91	20	267	14	280
59.494	22	AGGCTTGAGTTTCCTCACGA	59.989	20	125	5	129
59.702	22	GCAAGATAAGGGAGTTGGCA	60.214	20	145	597	741
60.375	20	TTACATCAACCCGTCAACCC	60.615	20	277	796	1072
59.593	20	CCAGCGCAGACAGAATACAA	60.011	20	279	41	319
59.715	20	TTATGATCGTCGTCGTCCAA	60.073	20	269	90	358
59.002	23	TTCGTGTATTAAGGTCACGGC	60.011	21	187	654	840
60.319	20	AGCCAGAGACTCGGATTCAA	59.95	20	190	905	1094
60.14	20	TATGCCACAGTCCCAATACG	59.42	20	253	1099	1351
59.962	20	CCAACCTCCATTAACAACCC	59.146	20	218	17	234
60.042	20	GACCGATCGTGAGGCTTTTA	60.214	20	255	397	651
60.06	20	GGAGCTGCTCTTTGAGATCG	60.24	20	274	1006	1279
59.609	20	CCCGTATATTCACGACTCGTTT	60.257	22	241	304	544
59.888	20	GCAAATTATTCACACCCGCT	59.967	20	144	5421	5564
59.182	20	TCGACACATTCAGGCAAATC	59.654	20	166	3	168
60.206	20	GCCGTCAGTAGCTTAATCGG	59.867	20	248	1948	2195
59.677	20	ATTTTGTTGAGGGGTCGATG	59.79	20	274	2458	2731
60.02	22	TTTGCTTAAAATGGATCAATGG	58.98	22	264	85	348
60.142	20	AACATCAAAACTGCCGAAGC	60.257	20	246	219	464
59.495	21	ATTTCAGTTTGGAATCGGCA	60.448	20	278	402	679
60.32	22	CCAAGCCACACATCTCTCAA	59.831	20	262	245	506
59.938	20	TTCCGCACCAAGATTTAACC	59.938	20	276	40	315
60.042	20	TGCGTTTTGACGATATATAGTGTT	59.092	25	151	608	758
59.654	23	CACCCCACCTTAACCTTTGA	59.824	20	219	12	230
58.974	23	TTGCAGTAGCTTGTGAACGG	60.05	20	222	34	255
60.261	20	AAGCTTGTCCGTCGATTTTG	60.249	20	201	115	315
58.311	23	AGAGGCAGCATACCCATCAC	60.104	20	143	1983	2125
60.11	20	CCATCTTTCTCAAATTCGCC	59.645	20	136	96	231
59.996	27	TTCTACCCATGCAAATCAAGTG	59.996	22	121	65	185
60.226	20	GATGGCTCATTTGTTCCGTT	59.939	20	225	85	309
58.697	20	TGGATATTGTTGCCGTTTGA	59.93	20	265	0	264
59.993	20	TCGCCTTCACTCTCACACAC	60.03	20	125	828	952
60.68	20	ATGCCAACTTTTCCTCATGC	60.081	20	279	1771	2049
59.767	20	CAGCATACAGCAGTTTCGTTT	58.121	21	136	1059	1194
58.741	20	AATTTCACCAAGATGTCCAAGA	58.554	22	102	20	121
60.11	20	AGTTTTGGTGAAGACCGCAC	60.156	20	192	52	243
60.044	20	GAATTGCGTAGCCTCCAAGA	60.352	20	137	185	321
60.081	20	CGTTGCCGGTTAAAGCTAGT	59.424	20	267	4	270
59.859	20	GTGAGCACCCCAGATTGTTT	59.973	20	240	1492	1731
59.92	22	AAATTTTATGGGACGGAGGG	60.013	20	251	368	618

59.866	21	GAATCCAGAAGGGCAGTTGA	60.195	20	182	3235	3416
60.015	20	ACGCGCGTTTTTAGGGTTTA	61.796	20	124	300	423
58.847	20	CCTCGACTTGACAACTTTGCT	59.529	21	280	105	384
57.397	22	AGGTTTTTCAAACGATTGCC	59.067	20	196	702	897
60.02	20	AGTGGTAGCACGACATTCCC	59.997	20	219	2194	2412
59.595	21	ACAGCAATCATCCAACCACA	59.967	20	211	523	733
59.12	20	TTTAAGGGTTATCACCTGATTTTC	57.757	24	144	72	215
59.886	21	CCAATCTTGTTCGGGTTCTT	59.028	20	200	11	210
59.756	20	TGAAAGCGATTGTAAGCGTG	60.014	20	220	102	321
60.177	20	TGCGTTTTGACGATATATAGTGTT	59.092	25	274	1010	1283
60.015	20	TTCCCTATCTCTCCCAGCAA	59.767	20	100	3204	3303
60.412	21	AAACTCCAAAGTCCAAACTCCA	60.012	22	128	0	127
60.202	20	TTTCCCTCTCCATCTCTAGCA	59.017	21	263	1095	1357
59.66	23	AGAATCTGCTTTGCCCAGAA	59.955	20	257	449	705
60.578	20	ACCATCACCATCGTCACCTT	60.246	20	160	48	207
60.033	21	CCCTGCACCCTTTTAAATCA	59.931	20	206	78	283
60.21	20	AGGTGGTGGAGTGGTGTGAC	60.915	20	227	111	337
60.243	20	GTCTGCAAGAGAGAGGCCAG	60.284	20	233	539	771
59.916	20	TTTTGATGATATATAGGTGTTTTGA	58.782	27	238	396	633
60.053	20	AACGGACGAACGATGAAAAC	59.978	20	175	3243	3417
59.706	20	GACATGTCGCTCATGTCACC	60.13	20	145	4254	4398
59.964	20	CCTTTCTCACCTAATTACAACCC	58.153	23	203	3329	3531
59.982	20	TGCACGATTTGACTTGCCTA	60.401	20	203	685	887
60.104	20	TCTACGACCACAATTTGTCCC	59.845	21	165	521	685
60.074	20	TCAGCCACCACCCTAGTAGC	60.277	20	265	343	607
59.816	20	CAAATATTAGGCCAAAAACGC	58.687	21	202	2456	2657
58.643	20	TGAATTTCCATCGTTGATTTGA	60.302	22	141	133	273
59.996	20	GGAGGTGGAGGAGAGAGAGG	60.336	20	111	469	579
60.263	20	TCATCATAATATTGATTTTTGCACT	59.703	27	116	896	1011
60.157	20	CAAACTAATAGCAATGGCCGA	60.102	21	134	3808	3941
60.12	20	CCAGGTTTTGGAACTTGGAA	59.942	20	261	1439	1699
60.088	20	GGCTCAAAAGACAAGTCCCA	60.232	20	238	136	373
59.967	20	GTGGGGATGGGAGGTTTATT	59.881	20	200	1076	1275
60.027	20	TAGAGGGGCCTAGTGGGTTT	59.955	20	254	17	270
60.006	20	GTTATTTCGGGGTCTGGGAT	60.017	20	262	2433	2694
58.861	22	ATTTTGTGAATTTGGCCGTT	59.32	20	140	356	495
60.243	20	GGGCCATAGATCTTGTGGAA	59.894	20	220	539	758
60.401	20	CCTTGAATCCAGGTTGAGGA	60.042	20	185	30	214
59.101	20	CCCAATTTCACCATCCTTCA	60.692	20	178	168	345
60.074	20	GAGTTGAGGGGTGGTTGAAA	59.943	20	178	70	247
60.137	20	CCAACGGGTAGATACCCAAG	59.308	20	280	685	964
59.803	20	GGATTAGGTGTTGGCTGGAA	59.933	20	206	501	706
59.955	20	CCAATTCCATCAAATCCAGG	60.126	20	184	86	269
60.545	20	GGTTCGGAAAAGGTTAGGCT	59.593	20	262	42	303
60.173	20	CGACTTGTCGTGGTTCGATA	59.716	20	239	87	325
59.933	20	CGAGAAAAACAAAATTATACGAG	59.565	26	229	610	838
58.019	20	CTTTTCGTTTTTGCGTCGAG	60.904	20	169	80	248
60.013	20	GCATGTGAGGATGTTTGTGG	59.967	20	139	1804	1942
58.874	20	CAAGAGACGCATTAAGACGC	58.677	20	217	1327	1543
59.856	20	TCACACCAAGAAGGGGACAT	60.363	20	204	108	311
60.027	20	ATTCCCCATCATCAAACCAA	59.991	20	188	83	270
60.291	20	TGCGTGAAATCGAAAGTGAG	59.988	20	185	466	650
59.504	25	GCTCGAGTTTTAGTGTGGGG	59.734	20	272	150	421
58.856	21	TTGCTCACCCCTACCTATCG	60.088	20	199	26	224
59.859	20	CACAAGAAAATTATTGTCGCTCA	59.307	23	270	584	853
60.008	21	GATCCTGGTATCCATCTCGC	59.469	20	190	630	819
60.003	20	AAGACCCCATTTCCCCTAAA	59.639	20	265	1382	1646
59.597	20	GGTCTCACGGGTGGTTTTTA	59.83	20	242	1560	1801

59.931	20	CACCGCGTCTTTTCACTACA	59.904	20	217	2311	2527
59.601	21	AATCATGGTGCACGAAATTG	59.4	20	183	411	593
59.962	20	GGTTGTGGTGGAGCAAAGTT	60.012	20	160	4427	4586
59.969	20	CTCTTGACTTCTGATCCGGC	59.95	20	161	88	248
60.074	20	CGAGATTCTCTCTCGCTCTCA	59.981	21	238	253	490
58.77	23	GGCAAGCTTGGGTATGTGTT	60	20	256	2	257
59.711	20	TATGTGTTGCAGGGGATTGA	59.924	20	159	585	743
58.823	20	GTTGGTGCTTGATGGTTGTG	60.008	20	133	2022	2154
60.298	21	CCAAACGACATTATGACGCA	60.523	20	268	173	440
59.938	20	TTCAATATCCGCCAGAGTCC	60.036	20	196	2273	2468
58.072	21	TGTGTCGCGAATTTTGAACA	61.233	20	212	8	219
59.839	20	GCTCTTCTCAACCCTTTTATGC	59.398	22	244	95	338
59.111	21	TTGTGCCGCTTGTAATATGC	59.731	20	236	43	278
59.859	20	GTCACCTGCTCATGTTGCAT	59.714	20	214	257	470
58.462	20	TTGGTAGGAATGGTTCGAGG	59.926	20	264	0	263
59.922	21	AATTCTTGGGGTTGATGACG	59.79	20	222	556	777
60.067	21	GAAACACATCGTTTCCTGGG	60.353	20	251	76	326
59.881	20	TTTCCATCGGACCAAGTCTC	60.05	20	238	954	1191
60.194	20	GTTCAAATACACGTCGCCCT	60	20	212	19	230
59.827	20	CAAAAGCACAAATTGGGAAA	58.649	20	242	45	286
60.064	21	TTAACCTGCAACGGTCAAAA	59.202	20	217	1470	1686
60.038	20	CTCTAGAGTGTTTGCGGTGC	58.677	20	230	69	298
59.647	23	ACGTGGAGTTCCAAATGCTC	60.119	20	270	3	272
59.459	22	AGTATGCCGTTTTTCCTCCA	59.569	20	280	1319	1598
60.556	20	ACCTCCTCTCCGTCGCTAGT	60.414	20	252	2	253
59.243	20	TCTCTCCCTTTTCTCACGGA	59.92	20	136	832	967
59.997	20	TGGTTATTACTGGAACGCGA	59.182	20	110	784	893
59.928	20	TCTTCAAAGGAACCGACTGG	60.224	20	280	75	354
59.343	20	CCCGGGTTTTCTTTTTGTTT	60.19	20	199	1399	1597
60.051	20	CCCCAAATGGAGACGATAAA	59.756	20	180	4639	4818
59.953	18	AGGTGACAATTGTGATAGCCA	58.09	21	280	123	402
59.859	20	ATTTGTACGGCGTTTCCTCA	60.502	20	154	661	814
60.734	20	GGTCAACTTTCTGCACACCA	59.726	20	212	606	817
59.615	20	GTGGTGGTATTTGGTCCGAT	59.532	20	260	31	290
59.883	20	AACGCTCCAGATTTCATGCT	59.843	20	270	435	704
60.031	20	CAAGCAGACTAAGCATGGCA	60.157	20	239	79	317
60.177	20	TAATGGCGAAAACAGGAAGG	60.067	20	197	149	345
57.557	25	CTTTAGAGCGCCTGCAAGAC	60.292	20	267	19	285
59.859	20	ATTTTCCCAAATGCAAGCTG	60.074	20	250	252	501
57.379	21	CGCTTATATCGTGTCGGGTT	59.982	20	100	36	135
59.948	20	CCACAGCTATGCCTAAGTGC	58.555	20	188	3269	3456
60.411	20	GGCATTCAAATCAACCATTG	58.82	20	233	81	313
60.096	20	GGTCGGCTACATGAACCAAA	60.894	20	116	32	147
60.112	20	GTAGGAGGAGTGATCGGGGT	60.336	20	278	737	1014
57.111	27	ACTTCAACAGCCGCAAAAAT	59.752	20	175	278	452
60.132	20	GAGCAGTTATCACCGGATCA	58.672	20	237	29	265
59.707	20	AAAACGGGGCTAAGGATTGT	59.835	20	246	277	522
58.743	25	GAACTCACAAAGTATGGGGCA	59.985	21	127	3	129
59.215	21	AGGGATTCGAGTTGCTTTGA	59.813	20	263	5193	5455
59.033	21	CCCCATGAAGCAGAGATGAT	60.034	20	117	757	873
59.871	20	ATGCACGTGAATGCTTTATTT	57.351	21	190	34	223
59.187	21	ACATTCTGGAATGGTTCCCA	60.173	20	185	1247	1431
59.905	20	TGGTGATGTGTACTTTTGGCA	60.019	21	188	1936	2123
60.324	20	AGGACTCACTGAAGGAGGCA	59.986	20	279	662	940
59.903	21	TCTTGCGTTTGATTCGTTGA	60.379	20	225	369	593
58.732	21	GGAGCAAGAACTTGAGGCAT	59.434	20	240	170	409
60.634	20	CCGATTTTGTTCCTTTTCAA	57.728	20	230	642	871
60.345	20	TGTCTTGAGGGGAGTGTTGA	59.23	20	278	352	629

60.201	20	GTGACATTTTTAGGCGGGAA	59.938	20	195	1183	1377
60.332	20	AATCTTGAAGTTGTTCGCGG	60.249	20	221	17	237
60.191	26	AAGCCTAACAAGTGGATATTAAG/	58.116	26	166	1299	1464
57.905	27	GTCGGGCAATGATTCTGTTT	59.939	20	260	9	268
60.089	20	CAGGTAAAACCTAAAATAGCGCC	60.36	23	258	4905	5162
60.324	20	TGGTTATTACTGGAACGCGA	59.182	20	188	1106	1293
59.502	24	AGGCATACTTGGTTGATCCG	59.955	20	258	10	267
59.989	20	CACTAGGGCTTTGCTGCTGT	60.592	20	161	2868	3028
59.366	20	AGCACTCCCTTTTACCACCA	59.592	20	246	68	313
60.395	20	TCTTCGATTGGAGTTCAGGC	60.34	20	241	552	792
59.803	20	AAAGTTAAGTAGGACTGGATCCA/	59.856	26	100	26	125
58.521	26	CAGATCTGATGGCCCAGATT	60.034	20	105	18	122
59.679	20	CCTTCTCTGGCTGTTGTTCA	59.007	20	236	1349	1584
59.919	20	TGAACTAATCAAGTTCCGCGT	59.761	21	170	53	222
61.129	20	AATTTCACCCTGATTCCAATG	58.751	21	279	43	321
59.175	20	TCTTCGCTGTCTCTCAGCAA	60.008	20	228	1226	1453
59.993	20	AAAGGGAACTTCCTGTTTTAGC	58.034	22	174	93	266
59.957	20	TGGCTTCAGTTGCAGAGAGA	59.855	20	162	84	245
59.916	20	ATCGGTTATTACCGGATCGC	61.031	20	206	24	229
59.931	20	CTGGGCCCACACCTAATAAA	59.817	20	145	159	303
60.84	20	CCTCTCTGCAACACCATTCA	59.831	20	197	505	701
58.695	20	ACCATCGAACCCCACAAATA	60.051	20	217	13	229
60.048	21	GGGGTGGGGGTATGTTAGTT	59.805	20	219	9	227
60.052	20	ATTTCTCCTCCATCATCCCC	60.096	20	127	261	387
60.257	20	CGAATCAGAAAGAACGTAGCG	60.025	21	180	31	210
59.837	20	GCAAATCAAACCCTTGTCAAA	59.967	21	213	717	929
59.707	20	ACTTCCTCTGGCATCGCTTA	59.978	20	152	1045	1196
59.924	21	GCAGTTGGAGAGGGTGAGAG	59.986	20	206	119	324
59.723	20	TGCACCTTGTGCAGATTTGT	60.31	20	175	598	772
59.878	20	CACGGTTTGGGCTTAATTTTT	60.219	21	279	467	745
60.051	20	AAATCAAAAGCCCACAATGG	59.801	20	128	64	191
59.968	20	CATGCACAGATTCCGTCACT	59.707	20	215	303	517
59.607	21	CATGGTGCATGAAATTGGAG	59.924	20	130	134	263
60.183	20	AATCATCCTTTCGCAACTGG	60.074	20	220	471	690
59.897	20	ATGTGCGTGTGAGTGTTCGT	60.236	20	199	105	303
57.252	22	TCACATGAGATGATTAATTGGACA	59.343	24	181	64	244
60.452	20	TGGAATAACCCAAGCCAAAG	59.931	20	179	2622	2800
59.986	20	TGATTCGGAGGAATACGAGG	60.029	20	199	421	619
59.05	20	AACAGCAAATCCGAGACACA	59.293	20	150	552	701
59.078	22	CCATTTTCAGGTCCTCACCA	60.892	20	125	31	155
59.679	20	GGAGATCAGCTACGGTCTGC	59.981	20	238	43	280
60.221	20	TGAAACTGCTGTATGCTGAGTTAT	59.424	25	170	312	481
59.231	23	TTGGCAAGAAAACAACAGCA	60.415	20	227	37	263
60.067	21	TTCACAAAGAAATCAGCACGA	59.454	21	186	70	255
60.249	20	TCAACGGTTTAAAATTGTTCCT	57.74	22	171	129	299
58.359	20	TCCATCCACAGCAATGAAAA	60.049	20	149	342	490
59.887	20	TTTCACTTCGATCCCACCTC	60.05	20	128	65	192
59.53	20	ACGACAACAGCAACACAAGC	59.953	20	240	216	455
60.104	21	GGAGACAGAGCCTGTTGGAG	59.986	20	196	15	210
60.315	20	CATTGGGTGGAAGTGTAAAGAA	58.994	22	172	540	711
57.989	27	GCAGCCTTATGTGGAGGATG	60.624	20	225	29	253
59.539	22	GGTCAGTTTGTCCGAATCTTG	59.583	21	105	256	360
59.162	20	CGCTGAAATTAGTTGAGGCA	59.062	20	272	133	404
60.24	20	TCATGTACCCCCACATGTCA	60.665	20	275	283	557
59.542	20	TTGGGTGTAAGACATAGAAGGAA	57.815	23	153	898	1050
59.202	21	GCGAAAAACTCGAAAAACCA	60.223	20	256	1554	1809
58.81	24	TGGATGATGTGATGTTTTTAGAGG	60.237	24	176	11	186
59.685	20	AAACTCAAATCCGAGATCTAATG(	59.874	24	280	32	311

60.074	20	CTCAACGGTATGCTCGGATT	60.096	20	235	490	724
60.136	20	GAAAACCTGGAGTCAGCTCG	59.989	20	265	427	691
59.803	20	GAGAGGAGAACTGCGTGAGG	60.135	20	161	74	234
60.31	20	CGCTTCTTCGCTATGGCTAC	60.138	20	218	393	610
58.9	20	GGAAGAAGAAGCTCGCAAGA	59.836	20	200	3301	3500
60.05	20	AAGCCGATCGAGTTTTTCCT	60.208	20	181	120	300
59.726	23	AGAAGACGGAGAGCCACAAA	59.989	20	165	1310	1474
60.164	23	CAATAGCGTGTGTTTTGCGT	59.799	20	273	587	859
59.976	20	CCCGGTTTTGACTCTTTGAC	59.569	20	151	450	600
59.891	20	ACCCTCGAATGATATGCTGC	60.066	20	169	26	194
59.732	22	AACCGGTTAAAAATCGGACC	60.055	20	248	208	455
59.803	20	GCATGGCATGAAATTGTTGA	60.476	20	273	118	390
60.129	20	TTTTCCGGTGATGGATGAGT	60.317	20	174	449	622
59.729	20	TTGTCTAGCAATCTTCGAGTTTT	57.422	23	280	135	414
59.272	20	TCGACAATGTTAAAAATCAAATAA	60.026	27	171	65	235
60.162	20	TCAAGGAGTCACAACGTGAAA	59.324	21	179	132	310
60.439	20	CTGATGAACAAGCCGAACAA	59.84	20	278	532	809
60.246	23	ACGATCGTAAGGCGAAATTG	60.096	20	104	4	107
59.955	20	TACCCAACCCACTAACCCAA	60.081	20	171	136	306
57.599	24	ACTTTCAGCATGGAAGAGGG	59.284	20	120	0	119
59.544	23	CAAATGCAATGACTGTTGGG	59.964	20	208	30	237
59.545	20	AGCGGTTATTACCGGATCG	59.93	19	249	2231	2479
59.926	20	GGATACCCGAGCAGTTGAAA	60.074	20	248	2251	2498
59.847	20	CCAAGATCAATGGTTCCTAAGTG	59.879	23	128	1272	1399
58.941	21	ACAATGCGAGTGCATTTTTG	59.735	20	186	111	296
59.571	22	TGTTGATGTCCGCAAAACAT	59.972	20	174	79	252
59.178	20	GACGAGATCATGGCTCGATT	60.189	20	265	14	278
61.434	20	CAGCTGAAAGGAGCCATCTC	60.096	20	275	44	318
59.883	20	TATATTTGCTCTGGCAGGGG	60.053	20	217	681	897
59.662	20	GGATGACATAACGTTCCGCT	59.962	20	186	28	213
60.11	20	CTGGAAGGAGTGGCTAGCTG	60.149	20	159	1248	1406
60.636	20	TCATAGGTAAAGCCGCAACC	60.096	20	211	23	233
59.792	20	CAATGCTTCCATTTGCACAC	60.119	20	264	91	354
60.288	20	GGCTCAACAACACAATTCCA	59.547	20	262	16	277
59.868	20	GCTTCATGAATGGCATGACT	58.674	20	247	44	290
59.989	20	TGCAGTTCATCTTTGCCTCA	60.539	20	122	1134	1255
59.955	20	AAGAAACAAACAGGGTACGAGG	59.56	22	262	264	525
59.972	20	TTGGTAGGGGTCAGCACAAT	60.375	20	225	115	339
60.096	20	TTCGTTATTTGATTGGGGGA	60.124	20	235	1199	1433
59.992	20	AGGTATCGTTCGCGCTGTAG	60.428	20	170	105	274
59.752	20	AAGCAAGAAGGTTGCAACTGA	60.045	21	150	353	502
59.496	20	TCATGTCAACTGTCGAAGGG	59.676	20	120	7	126
59.933	20	ACCTGTCTCAATCCGCTCAC	60.269	20	188	1434	1621
58.947	20	GTCCGGAACAAGGGAAAGTT	60.34	20	236	2	237
59.96	20	TAGCCTAAGTCGGGACAGGA	59.83	20	256	600	855
59.946	20	GCATGATGCAGCCCTAGATT	60.207	20	224	1919	2142
58.864	20	GGAGGGTGAGATCAATCGTG	60.475	20	184	61	244
59.933	20	CCTCCCCGATATTTTCAACA	59.756	20	222	8	229
59.997	20	TCGACTTGTGCAATCAGTCC	59.837	20	246	753	998
59.984	20	ACAAAAATGCCCTCGTATGC	59.967	20	241	22	262
57.857	24	TTTTTACCCTTTCCGCTTCA	59.693	20	210	48	257
59.526	20	ACGACACTCGAATGGAGGAA	60.656	20	244	241	484
59.763	20	GTGTGTGTGCGTGAGTGAAA	59.321	20	184	426	609
60.049	20	CTAGCCGTTGGTGGGAGTAA	60.125	20	148	463	610
59.442	23	CCACAACTATGTGGGAAAAA	59.71	21	208	405	612
60.323	20	CACTCGAAAAAGGCATCCAT	60.074	20	193	2588	2780
58.826	23	TCGTTTTAGTTGGCCATAATCT	57.848	22	202	286	487
59.91	20	TTTTTCAGCACCACTTCACA	57.867	20	181	114	294

59.592	20	GCAGACCAGCTTTTACTGGG	59.875	20	192	454	645
60.249	20	GGGATGACAGAATGAATGTGAA	59.797	22	203	507	709
59.881	20	AAAGAGAGCCACGTGTGTGA	59.467	20	202	134	335
60.089	22	ATCGTTTTCAAAATCAGGCG	60.074	20	238	207	444
58.694	22	ACAGCGCGGAAGTTACAGAT	59.904	20	240	26	265
59.369	21	AAATGCCCTGCTGTAGGAAT	58.672	20	209	372	580
59.964	20	GACATCGTCGTCCCTGCTAT	60.104	20	235	1109	1343
59.978	20	ATGCTTAATGTGCATGTGCG	60.697	20	280	1932	2211
59.988	20	CGCTCACAATGATGGATACG	60.096	20	121	5036	5156
59.245	20	GGGTCAGGGGGTTAATAAGG	59.527	20	129	468	596
59.845	20	TGAACAAATTACACAAACAAAAC(	60.54	25	129	521	649
59.335	21	GCAAACAACCAGCACAATCA	60.709	20	174	1587	1760
61.184	20	TCGTGGAGTGAGTGGAAGTG	59.864	20	273	112	384
60.194	20	ATACACGTTGCCCCTCAATC	59.82	20	196	847	1042
59.78	20	ACGCCTGAATATCGGTCATC	59.923	20	243	221	463
59.923	20	TCCATTCCGAAAATCAAAGG	59.872	20	116	24	139
60.378	20	GCCGACCCATTTAAATAACG	59.323	20	122	28	149
60.096	20	GTGGTGACAGTTCGATGTGG	60.005	20	163	2967	3129
59.78	20	GGGAAGATAAGACAGCCACG	59.694	20	217	20	236
59.735	20	TGAGCCAACTGAAAACACACA	60.331	21	119	2849	2967
60.238	20	CCATGGCCTAGCTTTGGATA	60.053	20	167	199	365
59.706	20	GCATCTTGGCTCATGTGATG	60.233	20	162	302	463
59.91	20	TCCCTGTCCTTTTTCCCTTT	59.912	20	144	584	727
58.294	22	TGCAAGCATCTTTTGTCTCG	60.134	20	215	11	225
57.487	25	GTTTGTTCACCGGAATCACC	60.218	20	249	19	267
59.767	25	ACGTGAGGTGAGGGATGAAG	60.112	20	271	46	316
57.085	26	TGGTTGTGGTTTGGTTTCTTC	59.861	21	222	1	222
60.073	20	ATCATCTCGGGGTGCAATAG	59.917	20	214	18	231
59.96	20	CGGGGTATTACATAAATGATGC	58.285	22	206	27	232
59.727	20	TGAAGGTGTTTGTGGGTTGA	59.976	20	136	5	140
59.862	20	GAGTCTGCCACGTGTACCAA	59.751	20	204	368	571
58.999	20	TTTTTGATGAGCTGGAGCCT	59.955	20	258	3989	4246
60.266	20	GGGCCGAGATTTGTAGAGGT	60.464	20	240	96	335
60.011	20	GGTCGATTCTTTCCCCTTTC	59.881	20	150	994	1143
59.926	20	GTGGGGGCTTAACAAAGTCA	59.971	20	187	397	583
60.24	20	CCAAAATCTAATGCGCGTCT	60.23	20	245	970	1214
59.072	22	AATGTGGGGTGCGATTTTTA	60.188	20	256	1949	2204
60.331	20	TCGAGGCGTTTTCTTGATTT	59.823	20	203	778	980
59.829	20	GCCAGCTTGTTTTGGGATAA	60.074	20	202	252	453
60.481	20	TGGTGTCTGAACAGCAGAGG	60.022	20	272	164	435
58.774	20	ATGACTTCGCCATTTTCCAC	59.939	20	280	216	495
60.051	20	TGTCCCAAAATCGTGTCTCA	60.088	20	264	15	278
60.281	23	GGATAGGTTGAAACTGCCCA	59.933	20	278	49	326
59.691	20	TGCGCATCTAGCTTGAGAGA	59.995	20	280	523	802
59.37	21	CCCCGATTCTTTTAGCCTTC	60.034	20	147	121	267
60.11	20	GTGACCGTTGGTGATGAGAA	59.525	20	189	1758	1946
58.451	21	CGACCTGGTGGGTTGTTCTA	60.943	20	268	1	268
59.971	22	GTGTGATTGGAGGGTTTGCT	59.973	20	122	14	135
59.059	20	CATCAATGGCCTGATTTGTTT	59.815	21	166	97	262
61.019	20	CCTAAAACACGACAGGGGAA	59.964	20	261	60	320
59.716	20	ATGACACGCATAAAGGGACC	59.82	20	240	249	488
59.517	22	AGCGGAAGAACAAAAACGAA	59.861	20	223	6	228
60.601	20	AACTGTGGCAAGATCCATCC	59.934	20	122	33	154
59.967	21	TTACAGATAACCGTTCCCGC	59.96	20	225	44	268
59.801	20	CATAAAATGGACACGCACCT	58.502	20	132	79	210
60.437	20	CACACACGCGCATATACAAA	59.211	20	107	1637	1743
60.187	20	AAGTTAAGCGTGTTTGCGCT	60.082	20	173	1313	1485
59.203	20	TCGGATTTTTATAGGCTTCTTCA	59.285	23	278	1344	1621

59.847	21	TCAATCATGAGCGTCCGTAA	60.22	20	138	14	151
59.766	24	CGAAAATAGCCAAGTCGGAA	60.202	20	268	1	268
59.801	20	GCTTTCAGGGTTTAAAATCGC	60.089	21	277	222	498
59.851	20	TCATGATAGGATGGGGCTTC	59.854	20	224	2507	2730
60.037	20	TGGAAGGAGAAACGATGACC	60.05	20	194	55	248
58.444	21	CCACCTTTTCATTCCGACTC	59.526	20	148	44	191
59.989	20	GCTCCCTCCATTTTTGTGTG	60.495	20	180	417	596
58.631	24	AACACAGCAGGTTTCGATCC	60.119	20	244	12	255
59.978	20	ACTTCCCTTCATCAGCCCTT	60.074	20	188	1267	1454
59.813	20	GGCCGGTTAAAGGAGAAAAG	60.068	20	280	233	512
59.569	20	TCAAAACAGAGAAGCCCACC	60.232	20	202	414	615
59.959	20	TCGATGTGGTGGAGAACGTA	60.112	20	123	82	204
60.048	20	AGGGTTTGAATGAGGTGTCG	59.966	20	146	334	479
59.571	20	AATTTGTCGTTTGACCAGCC	59.978	20	269	1004	1272
59.917	20	CTCCTTCGACACTTTTTCGG	59.846	20	259	808	1066
59.986	20	TGTTGGAAATCCTCGTTTGA	59.097	20	203	9	211
60.173	20	CAAGCTTACCCGAGCTGAAC	60.015	20	249	10	258
59.428	21	GACCGATCATTGCTATCCTCA	60.051	21	185	542	726
60.074	20	TTTTCTCGAGTTTCACGCCT	59.993	20	259	4678	4936
59.844	20	TTTTGGTTCAGGGGTGGATA	60.162	20	119	170	288
59.963	21	CTTGTCGTATCCCTCCTCCA	60.065	20	165	1271	1435
58.308	20	GAAAACAAGTGTTGCGGGAT	59.978	20	280	84	363
60.142	20	TAACTGCCTCTTTGCACCTG	59.072	20	227	7	233
60.134	20	AATACCAGGCAAAAGAAGCG	59.361	20	240	961	1200
61.031	20	AAACGGACGAACAATGAAGG	59.971	20	152	23	174
59.55	20	AGAACATTCTCATCCCGGTG	59.927	20	269	67	335
59.315	20	ACGCAAAAGCCCCTTCTTAT	60.095	20	233	127	359
59.284	20	ATGGAGGATCACACCACGA	59.901	19	240	558	797
60.21	20	TCTCGCTTCTCTCTTACCGC	59.859	20	140	977	1116
59.981	20	CTCTCGTACGCTGCCTCTCT	59.914	20	183	331	513
60.159	20	CAAAACCACAGCCGACAATA	59.585	20	264	1488	1751
59.74	22	CAACATGTGAGATTCCACGC	60.12	20	262	304	565
60.124	20	AAAAATGACGAATGGGAGGG	61.042	20	267	92	358
60.801	20	AAACTTGAGCAATGTTGGGC	60.118	20	267	429	695
58.876	20	TTCGTATCTTGCCCATGGAT	60.296	20	249	241	489
59.845	20	GGACAGTTTGTCCGAATCTTG	59.583	21	279	130	408
60.13	20	AAGGAGGTGTTATGAAGGGATT	58.028	22	262	1156	1417
59.875	20	CCAAACGGCCGATAAGAATA	59.922	20	196	4	199
60.558	20	TGGGTGAAAACCCATAGACG	60.745	20	280	504	783
59.82	20	AAATTTTGTTTCCCACCAAATAA	58.744	23	252	135	386
60.074	20	TTCCCACAGTTTGATCACCA	59.935	20	263	876	1138
59.056	22	GTTGGCAGAATTTGTCCGAT	59.939	20	144	0	143
59.952	25	GATCACAGGCCATCTTTTCC	59.488	20	279	4	282
58.355	22	CCGCATTTATGTGTGCAAAA	60.508	20	278	40	317
60.214	20	CAGCGAAGATGTGAGGATCA	59.942	20	127	513	639
59.968	21	CGACGTGCTTTTGGTAATTG	59.226	20	139	1307	1445
59.182	20	AGCAAAGTTGCTACCGAGGA	60.015	20	196	1200	1395
59.264	20	TCAGTTGATGCCTGATGAAGA	59.384	21	184	1471	1654
59.823	20	CTTCCAGAAACCTGAGCAGC	60.134	20	272	1883	2154
60.017	23	TGCCGTCTCACACATTAGAAA	59.332	21	164	2303	2466
60.045	24	CCCACCAATTTTGAGACGTT	59.83	20	265	67	331
60.353	20	TTTAACGAATGCTCCAGCG	59.961	19	280	111	390
61.096	20	CGCACTTGGCTCCTTTTTAG	60.011	20	275	1009	1283
60.243	20	AGTGTTTTGACATGATTACTCGTC	57.373	24	101	2147	2247
57.681	24	TCGATTTGGATTCGAATTTTG	59.893	21	170	58	227
59.942	20	GGTTTGCGAGCATAAAAATGA	60.089	21	272	512	783
60.081	22	AAGAGAAACCTTGGCCCCTA	60.068	20	121	11	131
59.787	21	AGCGCACTCAAACAATAGCC	60.416	20	279	686	964

60.483	20	CAATGCCTAGTTTACCCCCA	59.817	20	242	0	241
59.647	20	GACCGAAAATGGAGGTTCAA	59.91	20	174	247	420
59.597	20	CACACACTCACGCACACATT	59.183	20	160	408	567
60.457	23	ATTGTTGCCGGAAAATATCG	59.795	20	256	19	274
60.154	20	CCCTCGTATGCAGTCAAACC	60.517	20	274	693	966
59.929	20	CTAGGCATGGAATCCGAGAA	60.17	20	254	12	265
59.482	20	CAACCTTGGCCACATAACCT	59.853	20	233	3	235
60.003	20	CCCACAATTAGGTCCCCTTT	60.046	20	163	259	421
58.287	20	CGGTTATTACCGGATCGCT	59.93	19	266	1528	1793
59.93	21	GTGGCGACATCCGAATAACT	59.962	20	216	178	393
60	20	CATAGCCGATAAAAATGGCG	60.424	20	199	80	278
59.457	20	CGAAATCCCCTTTTTCAACA	59.91	20	224	1029	1252
59.758	20	CGGCTCAAGATACCGATGAT	60.059	20	234	572	805
60.02	20	AGAATATTCATGGGGTCCGA	59.2	20	269	1128	1396
59.83	20	AACCCGGATCGGAAAAATAC	60.02	20	274	835	1108
59.803	20	TGAGTTGCACGTGTGTGAGA	60.07	20	200	323	522
59.894	20	TGGGCACATTGCTCATTAAA	60.073	20	266	2805	3070
60.288	20	AATGTTTGTCGGGCCTAAAT	58.443	20	266	547	812
60.439	20	TGATGTGTGATGCCTCCCTA	60.072	20	260	710	969
60.154	20	GTGAAGGGCCAATTTCTTGA	60.051	20	245	1468	1712
58.583	26	ATAAGTTGGGTCACGGGTTG	59.711	20	264	210	473
59.966	20	CTACGTAGCCCCCAAATCAA	59.953	20	201	221	421
57.869	22	AGGGCAGTCAAGACCATCAT	59.535	20	250	0	249
59.679	20	ATCACCAGTGGCAAACACAA	60.008	20	174	69	242
59.885	20	CGAATATCCCCCAATTCAAA	59.593	20	276	2278	2553
60.074	20	GCCTGCCTATTAAACTCCCC	59.93	20	178	33	210
60.37	20	TTTTGTTATGATAGGATAAAACCT(	58.996	26	259	466	724
60.002	20	CACCATGCTCAGGAATGCTA	59.823	20	234	2201	2434
60.149	20	AACCCTAACCCGACTCGTTT	59.864	20	257	41	297
60.732	20	GCAGCTGTCCATTGTGAGAA	59.992	20	261	218	478
57.528	21	TCAATGATGAAACGGGGATT	60.133	20	134	80	213
60.088	20	AACTCACCCACACACACACG	60.541	20	274	4	277
59.01	20	ATGGAGAAAAGGTACGCACG	60.132	20	158	8	165
59.264	20	TGAATCGAGTGTTTCTTGCG	59.988	20	205	1332	1536
58.925	20	CATGAAAGGAAACAAAATCGAA	59.098	22	119	762	880
59.638	20	TTTGTTATGCGTTTTGACGA	57.846	20	277	95	371
60	20	CCTTCTTCCACCGTTCTTCA	60.224	20	114	4327	4440
59.123	20	ACAAGCCCGTAGGGAAAAAT	59.835	20	273	44	316
60.045	20	GTTGGTGGGATTTGAACCTG	60.21	20	237	873	1109
60.842	20	GGGCTGCACACGAATGATTA	61.98	20	150	673	822
59.986	20	GGGAGGATAAACCGAGGAAG	59.897	20	161	2263	2423
59.831	20	TTCTCATCCACAGACCCCTC	60.048	20	120	1927	2046
59.316	20	TCGATTCAGCAACCAAACAC	59.697	20	190	27	216
60.594	20	TTTTTGGTTGAATAGGGGTGA	59.302	21	280	98	377
60.361	20	TGCAGGGCTGAGTATAGGGT	59.717	20	271	83	353
60.034	20	CAGCAAGGAACTGATGGACA	59.831	20	176	1327	1502
59.67	20	CCCTAGGATGTGGGGGTAAA	60.919	20	252	1737	1988
59.996	20	GTGGGTTTACCCAGAGCGTA	59.993	20	116	524	639
57.118	26	TTTTCAATCATACTTCCGGTTTT	58.955	23	220	57	276
59.726	20	TTTTGACCAATCCTTTTGGG	59.772	20	273	259	531
60.074	20	CGGCCAAATCAAAAGAAAAA	60.047	20	100	486	585
59.962	20	CCTCCATTCAAGCAGAAAGC	59.955	20	189	926	1114
59.953	20	TTGCAATAGCATGCCAACAT	60.103	20	224	191	414
60.149	20	TTCGGCATTATAGGAGGACG	60.053	20	278	35	312
59.79	20	GCCGTAGATCTAACGACCGA	60.235	20	127	1314	1440
59.406	20	TTCTTGATGTGTTGGCAAGC	59.847	20	134	206	339
60.792	20	GACCCAAAAGACTAGGTGCG	59.734	20	197	278	474
59.979	20	CAATATGAGAATGGCCGAAA	58.576	20	159	428	586

59.901	20	GTGGACGTCCCAGATTGTTT	59.827	20	144	777	920
59.637	20	CACGTGGCATTTAACTGGTG	60.027	20	258	1217	1474
60.175	20	AAGGGGAAGTTATTATTTGATCAC	59.882	25	194	278	471
60.051	20	AACAGTGAGGGAACACCAGG	60.002	20	233	186	418
59.95	20	CGTTGATGAAGCACCGTATG	60.134	20	160	671	830
60.074	20	TTCCTACGCACTCACATTAAGA	57.601	22	184	229	412
61.091	20	TTGGAAGGGGCTTGAGTATG	60.066	20	102	1325	1426
60.07	20	CGATGACATTAACAATAACAACA/	60.095	26	169	381	549
59.129	20	CACCCTTCGATACTCCGAAA	60.066	20	259	54	312
59.967	20	CAGATCAATGGCCCAGATTT	59.894	20	230	504	733
59.907	20	TGTGTGGCTTTTCTGCAAAC	59.888	20	277	565	841
60.356	20	GCGGACACAGGATTTCAACT	60.119	20	280	80	359
60.201	20	ACGGTGATAGTTTGCGTTGA	59.199	20	279	6155	6433
60.053	20	TCTGTGGTTCCATTCCATCA	59.893	20	214	506	719
60.852	20	ATCACAGGAGATTGGCATTT	57.043	20	223	496	718
59.938	20	GCCTGAACCACCAACCTTGT	62.297	20	191	207	397
58.145	20	AGCAACTCCTCAAACGCAGT	60.058	20	176	67	242
60.088	20	GCCGCAGTTGAGATCTAAGG	59.978	20	219	1040	1258
59.883	20	AAGTGGAGGTTCCATGTTGG	59.82	20	160	59	218
60	20	TTGCCTTTAGCTTTAGCCCT	58.299	20	224	2706	2929
59.916	20	GATTGCGAGAGACCAAAGGA	60.34	20	205	258	462
59.903	20	CATGCAGTTTTTGCATGGTT	59.59	20	272	1428	1699
58.556	22	ATTTCGAAGATCAACGGACG	60.074	20	124	34	157
59.267	22	AGAGAGACGACGGATCGAAA	59.95	20	237	16	252
59.774	20	GCAACGGAATACCACTCGTT	60	20	176	84	259
59.93	19	AAACAAAAGGCAACTGATTCG	59.257	21	273	19	291
59.429	20	AATTTCACATCCTCCCTCCC	60.133	20	268	42	309
60.088	20	TTGGAGGGAGAAAATTGTGC	60.051	20	275	1075	1349
60.12	20	ATTCTCAACAAGGGGCTGAA	59.67	20	219	230	448
57.069	26	AATTGCCACGTTGAGGAATC	59.939	20	270	2	271
60.008	20	GCGTTGGGATCGTGTTAAGT	60	20	196	3369	3564
60.892	20	TGAGCTTCCAACGTAAGTTTCAT	60.176	23	147	3181	3327
57.542	20	GCATGATACCAATGCTGCAC	60.112	20	241	48	288
59.948	20	GGATTGGAACTCCAAAACGA	59.91	20	227	400	626
59.95	20	GTTGACGGCTGGAACCTTAG	59.734	20	162	1396	1557
58.381	25	ACATTCCCGTTGTGTGTTCA	59.855	20	246	57	302
60.088	20	TGCAAGTGGGAGGGAAGTAG	60.246	20	218	583	800
59.781	20	TAATTTGGCAAACTCTGGGC	60.074	20	128	785	912
58.829	23	CGGACCAACCCGTTTATATG	60.067	20	163	67	229
60.088	20	TCCACGTCTTGTTTTCCATCT	59.583	21	280	4276	4555
60.192	20	AACATTGTTTGGGAAAAACCTT	58.822	22	162	828	989
59.801	20	TCGAAGGTCAATGGTTCACA	60.088	20	211	122	332
59.964	20	GCGCGGAGTTTAAGAAATGT	59.365	20	239	290	528
59.995	20	GCCATCAGATCTGCACTGAA	59.95	20	203	1237	1439
60.096	20	CTCGTTTCTTGCTAGCTCGG	60.284	20	235	660	894
60.081	21	GGCAATATTACCATGACGGG	60.038	20	276	2013	2288
60.447	21	GCCGGCAACTCACTTAACTC	59.882	20	114	38	151
60.12	20	GTGTGTGTATGCGCATGTGT	59.038	20	238	92	329
59.935	20	TGAGTCTGATGGACTGCCAC	59.827	20	274	101	374
60.096	20	GATCTAACTAGGCCCGTCCC	59.925	20	162	242	403
59.973	20	GAAAAACCCTAATCCGCCTC	59.909	20	141	80	220
59.56	24	AACCCGTGTTCTTCAGTTGG	60.005	20	242	213	454
59.663	21	TCCATGAAAGATTGGGGTGT	60.173	20	240	1734	1973
59.942	20	GATCGCCGAAGGTTTTTGTA	60.074	20	271	815	1085
60.238	20	CACCCCGAGATAACTTTCCA	59.926	20	224	16	239
59.783	20	TGTTCACCAAGTTCGCAAGA	60.427	20	162	115	276
59.697	20	GATGCTCCACTTCCTACTCCA	59.298	21	233	104	336
60.001	20	CTACCATCTCGGGATGCAGT	60.096	20	253	1328	1580

60.074	20	TGTGTAATTGTGTTGTGTCATGTTT	59.278	25	210	309	518
60.014	20	AGGTTCGGACAAACAACAGC	60.156	20	212	1223	1434
58.575	24	GCTATGAGCATGTCATCAACA	57.291	21	125	0	124
57.312	24	CCAAAAACCATCTGCCCTTA	59.931	20	182	1	182
59.661	20	CATGGGATCTCAACGATTCA	59.451	20	259	661	919
59.526	20	CAGCACTAACACACACACGCT	60.026	21	260	59	318
60.199	20	TGCATAACACGGGTGGAGTA	59.988	20	252	1364	1615
59.907	20	GAGGTCACCCTTTGTCCGTA	59.966	20	257	222	478
60.44	20	AACCCGCGAGTTGCTACTAA	59.904	20	137	283	419
60.038	20	GTGTAGATCTGGCGGGTAGG	59.574	20	279	373	651
58.823	21	TTGACTTAACAAACCCCAAGG	58.98	21	247	98	344
60.119	20	AATTGAGTTCGGGATTGTGC	59.939	20	271	18	288
59.864	20	TTACAATGACGTCAGCACGC	60.876	20	208	3	210
59.901	20	TTCTACGAATGTTGACGGAAAA	59.625	22	113	536	648
60.118	20	GATACGTGATGCGTTGATCG	60.104	20	183	140	322
60.218	20	TTAAATCTCCCCACAAACGG	59.795	20	241	11	251
60.032	22	GTACAGAGCGAAAACGAGGC	60.022	20	243	193	435
60.495	24	AAGCATGAGTTGTGTGCTGG	59.905	20	244	0	243
59.72	20	CTCCCGAATTGGGTTTTCTT	60.291	20	213	11	223
59.864	20	GTGGATCATGAGCGACCTTC	60.633	20	232	629	860
60.11	20	AGTGGTTATTACCGGATCGC	58.93	20	210	2692	2901
59.264	20	GATGGTGAATACGATTGGCA	59.355	20	268	118	385
59.043	20	TAGGCCCGAATGAAATGAAG	60.031	20	202	203	404
58.949	22	CTTCCCAGCAGAGAATCGAG	60.088	20	149	5	153
60.299	20	CCGGGGTAATCTCAAAACCT	60.18	20	247	540	786
60.103	20	ATCGGCCATTCCTATTTCCT	59.761	20	148	409	556
59.173	22	AGCCCATTCGTGATTTATGG	59.784	20	260	16	275
60.133	20	GCGTCCATTCATCAAGATCC	60.435	20	233	23	255
60.34	20	TTGATGCAGCCCAATGTATG	60.488	20	237	205	441
60.157	20	GAGCGGGATTATCAGACGTG	60.624	20	210	3	212
59.971	20	GGGAGAAACAACCACCATTG	60.21	20	252	600	851
59.997	20	CATGAGCCTCCAAACCCTAA	60.066	20	151	1056	1206
59.797	20	AGCACGAGAAAAAGACCAGC	59.621	20	176	1618	1793
60.058	20	AGCAAGAACTCACGCCTTTC	59.621	20	265	108	372
59.614	20	TTTTGCTGTGGTCCCCTTAC	59.971	20	151	444	594
59.745	20	TTTGGGTAGGCCTGAGTCTG	60.246	20	276	3281	3556
59.693	20	AGCTTCAAACATTGATGGCA	59.276	20	247	243	489
60.457	20	GCTTCGCAACGAAGGAATAC	59.851	20	270	17	286
59.566	20	CTCCAACCTGCCACAAAAAT	59.971	20	135	2515	2649
59.905	20	GGGATGTGTGGGATTGATTC	59.995	20	245	930	1174
59.836	20	TCACGCAATCACACAGATCA	59.823	20	271	1049	1319
59.257	20	GGTGAACGTAGGGGTGAAAA	59.83	20	198	109	306
59.111	21	TCGCGTAGAAATCCTTATGC	58.017	20	191	16	206
59.988	20	TGAAAGAAAACAAGAAAGAATCA	60.499	26	240	4238	4477
59.48	19	AGCGAGGGAATTAGGAGGAG	59.805	20	257	2	258
60.138	20	TCCAAAGAAATTGAGGTGCC	60.051	20	255	250	504
59.996	20	AACGAAGCAGCCGTAGAAAA	60.018	20	264	974	1237
59.946	20	TTATCTGTCCTCTGGCCTTGA	59.822	21	237	6	242
58.165	25	TGCGTTTTGATGATATATAGGTGT	60.054	26	244	1913	2156
59.685	20	ATCCAATGGCCATGATTTGT	60.021	20	145	3	147
60.349	20	TGCACAAAATCCCACAAAGA	60.088	20	243	11	253
60.667	20	TTGCCTATTTTGGAGGATCG	60.031	20	224	47	270
60.074	20	GGTTGTGAGAACCAACTCTGTG	59.664	22	187	118	304
59.651	20	TTCACTGCTTATCGCCACTG	60.011	20	236	41	276
58.619	21	CAACCACGTACAACCTGCAC	60.072	20	236	739	974
59.742	20	AGAGGAGGAACCCATGGAGA	60.991	20	215	355	569
59.748	22	GGTTGAATGGATATGGCTCAA	59.776	21	269	140	408
59.364	21	GGGGCGTGACAAGGATACTA	59.955	20	267	62	328

59.939	20	TAGGGAGCTGTCAATTTCGG	60.206	20	210	1109	1318
59.16	21	CAGATGGGAGGAGAGAACGA	60.34	20	236	1	236
59.95	20	AGGCAATATCAATCTCGGTGA	59.542	21	158	120	277
60.042	20	GGGGAAGCACAGTCTAGGGT	60.511	20	277	77	353
60	20	GGTGTTGTGTGATGTCGGAG	60.005	20	203	75	277
59.967	22	GTTTAGTGATGGAACGGGGA	59.79	20	144	47	190
59.903	20	CACGTGGCGCTAATTGAGTA	59.895	20	198	112	309
59.673	20	CTCGTCGTGTTCAAGGCTC	59.561	19	233	1618	1850
60.119	20	CCTCCCAAATTGTCGAGTGT	59.966	20	232	31	262
60.162	20	AGCAAAAATGCCACGTATCC	59.967	20	213	6	218
60.067	20	ATCTCCAATCACGTTCCACC	59.786	20	170	871	1040
60.434	25	CAACTTGCAGGTGCTAACCA	59.904	20	128	19	146
59.996	22	ACCCTCCTTCAGCCAAAAAT	59.94	20	256	76	331
60.049	20	AGTGTCTCCAGTCTCCACCG	60.306	20	225	2950	3174
60.885	24	GGGCTGAAAAGCCAATTAAA	59.189	20	280	9	288
58.439	22	GGCATTGGACATCAACATGA	60.339	20	187	72	258
60.246	20	TCGGCTCCTCAGACACTTTT	59.989	20	223	105	327
59.95	20	TCGCCTTAACCACTTTGAGG	60.241	20	275	363	637
57.171	23	GTAGAGGCGGAGTGCTTTTT	58.604	20	233	0	232
60.547	20	ATGCTGCTCTAAGGCGTGTT	60.044	20	250	2776	3025
59.696	20	CAAACCGGACTGGACCATTA	60.745	20	271	9	279
59.696	20	CAAACCGGACTGGACCATTA	60.745	20	267	9	275
59.967	20	AATCTTTGAGGACCCCCTTT	58.896	20	120	18	137
60.227	20	CCTATGGTGGTATGGTGTGG	58.569	20	259	53	311
60.052	20	CTCTGCATGTTTGTTGGGTG	60.152	20	216	1864	2079
59.381	20	TGGCGTATGCTGAGAGAATG	59.972	20	136	222	357
58.888	23	AAAATCAAAGGCCCACAATG	59.801	20	221	212	432
60.244	20	GGGAGTTATCATTGGATCAAGC	59.805	22	179	537	715
59.945	20	GGTCGACATTAAATCTTGGCA	59.952	21	264	581	844
59.926	21	CCAAAGAACTGATATCCTCACCTT	59.901	24	145	525	669
60.229	20	TTATCAAGGGCCCTACCTCC	60.278	20	204	605	808
59.772	20	CGCCTACCCTCCTCTCTTCT	59.972	20	107	89	195
59.326	23	CTTTTTAATCCACCTGGCGA	60.067	20	203	15	217
59.971	21	TCATGTGCCTGGATTGGATA	59.883	20	237	34	270
59.463	22	GCTGAACGAGTAATTCTTGGTG	58.906	22	278	561	838
58.955	22	AAGATGCACGTAGGGAGGTG	60.134	20	207	6	212
59.434	21	AGAAAACCCGGCTCGTATCT	60.096	20	238	184	421
59.823	20	TCAAAACTGCATTTCTGGGTT	59.601	21	214	675	888
59.809	20	CATGATTGCCATCCTCCTCT	60.034	20	141	659	799
58.161	20	AGAGCAAAACCCATCAGTGG	60.111	20	100	1726	1825
59.842	25	CAAATTCAAATGCCTCAGCA	59.809	20	243	0	242
59.955	20	TGAGTGACAAGACCCTGCTG	60.022	20	202	635	836
60.287	19	CCCGATCCAAGGTTCAACTA	59.926	20	279	38	316
59.592	22	CGTGGTTCGATCGTAATTGA	59.542	20	128	18	145
59.579	20	TGCTAACAATGATGGGCAAA	60.073	20	111	2757	2867
60.214	20	GTGGGCTGTGGCTTTTGTAT	60	20	188	353	540
59.943	20	CCGAGACAGCACTCACGATA	60.008	20	239	4147	4385
59.707	25	AGCATTTGATCTGATGCACG	59.83	20	151	3	153
58.381	21	ATGGCCCCCAAGAAAATAAT	59.512	20	268	123	390
59.08	21	ATGCCTTCTCTTTCCTGCAA	59.955	20	246	4215	4460
60.011	20	ATTGACGCCTACATTGCCTC	60.103	20	232	107	338
60.149	20	CCTCTCATGTTTTCCCCAAA	59.903	20	201	267	467
59.997	20	ATCCTCATCTTTCCCCAACC	60.133	20	170	1400	1569
59.028	20	TTGTCCTCACCCTCTCCATC	60.048	20	119	1	119
57.458	27	ATGCACTACCGGATAGGCTC	59.178	20	197	49	245
60.306	20	ACACCCACCACACCTCATCT	60.291	20	197	118	314
60	22	GCCAAATTAAGGGCATGAAA	59.907	20	139	258	396
59.978	20	TCTCCTCCCCTTTTAATTCCT	58.212	21	218	51	268

59.583	21	CACTTGGACCTGAAAAGGGA	60.081	20	141	0	140
59.799	20	TGCTATCCCATTGCCCTAAG	60.053	20	198	263	460
59.823	20	CGTTAGGCTCGAGAGTGAAGA	59.766	21	161	3	163
60.051	20	TTGAATTTTTGGGGTTGGAA	60.138	20	251	125	375
60.363	20	CCCACCAGTGCCATACTTCT	59.989	20	116	657	772
59.933	20	TCTTCAATCCTTCTTCCCCA	59.6	20	159	32	190
59.923	20	TTCGAAAATGGAGGGATGAG	60.006	20	211	99	309
59.96	20	TGGGTGGATCTTGAAAGAGG	60.042	20	227	753	979
59.927	20	GCCCTCCCAAAAGTAACTCC	59.94	20	211	40	250
59.861	20	CAGTCCACTTTTGAATGGCA	59.691	20	207	25	231
59.934	20	CAGGCACATGCAAAAAGAAA	59.849	20	177	102	278
59.121	25	TCTTCATCCCATTGACCCAT	60.135	20	264	112	375
60.274	22	TTCCGATTGCAAGAAAAACA	59.277	20	269	288	556
59.972	20	TTCATCATCTTCCCTTTCTGC	59.259	21	176	64	239
59.959	25	AATCCATACTGCCAAAACGG	59.823	20	251	228	478
59.914	22	GTCTCTTCCTGGGAGGGTTC	60.05	20	153	2	154
57.01	23	GGTCATTTTAGCTTGATCAACAG	57.952	23	174	524	697
60.082	23	TGTTTCTCGATAGCCCGTTT	59.708	20	243	1913	2155
59.545	20	TTTGAGAAAGCAGTGGCAGA	59.716	20	252	9710	9961
60.314	20	GGCAATCAGTAGCCCAAAAA	60.074	20	273	2516	2788
59.091	20	GGTCGGCTACATGAACCAAA	60.894	20	159	0	158
60.258	20	AAGCGAAGGTGTTGATGAGC	60.406	20	233	84	316
59.957	20	GCGTGGTCTTGCCAATTTAT	59.967	20	272	2773	3044
59.744	23	CTCAAGCTCGTCACCCTTTT	59.473	20	147	7	153
59.993	20	AAGGAGGTGTTATGAAGGGATT	58.028	22	268	973	1240
59.96	22	TTGATTCTGGAATATCCCGC	59.862	20	151	92	242
59.75	20	GACCTAAGCCACTCGCTACG	60.037	20	240	294	533
60.147	20	GAGCCCCACTATCTTCACCA	60.073	20	221	48	268
59.349	20	TTCTTTCTCATTCTCTCGGCTC	60.096	22	146	1149	1294
59.941	20	TCCTCATTGTTTGTGTGGAAA	59.01	21	206	56	261
60.012	20	ATGCCTCAAACAACCTCACC	59.973	20	161	986	1146
60.083	26	TGGATGAGCTCCAATGTGTC	59.637	20	147	1	147
59.995	20	GACGCTCCACATGATGACAC	60.13	20	265	118	382
60.036	20	AAAACTGCGAAATGAGGAAGA	58.962	21	154	253	406
60.11	21	TTGCTACAAAACCAGTGGCA	60.293	20	205	1060	1264
59.966	20	CGATGTTTATTTGAGGCGCT	60.23	20	108	190	297
60.584	20	TTTGTGAACGTCGTCATTGT	57.571	20	200	243	442
60.019	20	TCCACGTGGCACTTCATCTA	60.263	20	219	2319	2537
59.92	20	CCAGCAGACAAAAAGCATCA	59.988	20	131	467	597
59.75	20	TTCCATTGGGTTTTGGGTTA	60.024	20	142	4045	4186
59.206	20	TGCCCCTTCTGACTCTGTCT	59.986	20	230	193	422
60.703	22	ACTGCCCAACAATGGCTTAC	60	20	189	6	194
60.201	20	CCATGATTTCCTTTAATTCGATG	59.7	23	115	217	331
59.93	22	TGTTTTGCCCGTACGTTGTA	60.029	20	245	194	438
60.713	20	TGAGCTGGCAGAGACCTTTT	60.134	20	134	209	342
60.03	20	TTAGGCCCCTCCCTACATCT	59.919	20	147	335	481
59.9	20	CTCACCAGGCCAGGGTAGTA	60.126	20	212	1216	1427
59.971	20	TCCCAACAAAACGAAAAAGT	57.25	20	201	1235	1435
60.011	20	CGCTCTCTCATTGTAATCTCTCTC	58.83	24	136	345	480
60.096	20	TTAATGCACTTCGACGATGG	59.688	20	248	736	983
59.907	20	GCCCAGCTTATTTGGTGAAA	60.074	20	238	1678	1915
60.081	20	GTCAAGCCTGGGACTGAGAG	59.986	20	261	1176	1436
59.83	20	GACTGGAGGAGAGCGAGCTT	61.212	20	213	118	330
59.052	20	TTCAAACATGGCAACAATGG	60.357	20	139	409	547
60.131	20	AAATCCTTTGCACAACCCAC	59.836	20	111	48	158
60.058	20	TTCTTGGCAATCATGTCCCT	60.461	20	183	66	248
57.333	24	TCACACTCACGCACTCACAA	60.07	20	121	15	135
60.22	20	TTTGTACCAAAAAGTATATAAATG	57.58	27	260	597	856

59.455	23	TCGTTCAAGTCGGTCACATT	59.139	20	195	46	240
60.118	20	CAGATCTGATGGCCCAGATT	60.034	20	143	276	418
59.697	20	GCCCGGCATACAAATAGAGA	60.06	20	270	22	291
60.199	20	AGTCACCGACAACTCCAACC	60.009	20	250	20	269
60.523	20	TAACCACCCAATCTTCGCTC	60.074	20	181	96	276
59.966	22	TCGTGAGAGATAGATATGGAGAG	57.233	24	261	88	348
59.41	21	GGGTCTGACATTGTAATAAAGGG	58.812	23	272	31	302
60.817	20	CACGTTCGGGTTTGATAAGG	60.357	20	250	446	695
57.154	22	GGGTGTATGTGAACCAACCA	59.112	20	203	628	830
60.011	20	AATTTGGCTTGTTACAGCGG	60.131	20	253	563	815
58.4	21	CCTCCATTATCCACTCTCGC	59.653	20	258	739	996
59.556	24	AAGAGCAGAAAGGGGGAAAA	60.18	20	248	32	279
58.013	23	TTTTCCGGTTAACAAATCGC	59.943	20	279	792	1070
60.088	20	TGTCGAAATGGGAGTCACAA	60.088	20	105	1226	1330
59.847	20	TCAGAACATGGGCAAATTCA	60.049	20	169	491	659
60.375	20	GGGACATCTTGGTGGGAATA	59.605	20	279	35	313
59.697	20	GGGAACATTCGAGCCATAAC	59.391	20	163	41	203
59.768	24	GAGAGAGGCAGACATCAGGC	60.104	20	280	21	300
60.011	20	CAGATGATGTCCCAGCAAAA	59.648	20	259	36	294
59.583	22	ATCGTCCCCTCTCTTGCAG	60.356	19	204	375	578
60.683	20	TTGTGTGATCGAACCACGTT	60.008	20	123	48	170
60.116	21	CTCGTCATCAACTTTTCCCC	59.526	20	209	411	619
60.472	20	GGATAGGAATCTCAAGAGTATGCA	60.007	25	185	1013	1197
60.844	20	TCTCTCTTTCTTGGTTGGTGAA	58.955	22	190	142	331
59.89	21	ATTCGGACATCGAGATTTGG	59.894	20	238	69	306
59.778	20	CGAATACTCCAAATGACACCAA	59.861	22	238	53	290
59.322	21	TTGAATATGCAATCACCAAAGAA	59.485	23	219	26	244
57.122	26	GGTGCACCACCCCTATCTT	59.794	19	251	590	840
60.298	20	AAGAAAACAGAGGTAGTGAATTG	59.658	26	231	287	517
59.273	20	TTAACAACGTTTCGGAATTTTT	57.813	22	280	122	401
60.117	20	TTCACCATGATTTCGATACACA	58.895	22	230	656	885
59.791	21	TTTCTCCTCGTATGCGTATTCA	59.742	22	277	644	920
59.145	21	AGAGGGAAAACAGGGGTAGC	59.576	20	124	7	130
60.031	20	ATAAGCAACGAAGGGAGTGC	59.34	20	212	279	490
59.939	20	AATCTTCGCCCTTTTGTCCT	60.074	20	274	1615	1888
59.955	20	ATTTTATCCGACTGGCGTTG	59.96	20	272	457	728
59.933	20	AACAAAATCACCCCCTCACA	60.21	20	108	4	111
57.727	24	TTTCACTTTTTGCACTCCCC	60.088	20	151	0	150
59.263	20	TATCACCATAACGCACCCCT	60.214	20	247	127	373
60.255	20	CCAGGTGGAGTTACACCCAT	59.697	20	208	732	939
59.357	20	ATGGCCATGTTTTTGGATGT	60.059	20	202	651	852
59.566	24	GAATCACTTTGCAAGCACCA	59.847	20	183	221	403
60.081	20	GTGGAATCAGTGAGGAGGGA	60.048	20	182	66	247
59.903	20	ATTGAATCGCAAGCACATGA	60.228	20	124	194	317
59.84	21	GATTTTGAGCCTTTTGCTGC	59.967	20	212	5	216
60.214	20	GGGACTGTGAAACAGGGAAA	59.943	20	251	271	521
59.84	20	TGCAAAGAAAGAATCTGGGG	60.184	20	259	805	1063
59.839	20	TGAGATTTTCCTGCCAAACC	60.051	20	187	1596	1782
60.578	20	CTCTTCAGCTGCTCTCCGTT	59.891	20	244	304	547
59.396	21	CATGTGTGAATATCCGCTCG	60.096	20	190	39	228
59.989	20	ACGGCATTGAAGAAATGAGG	60.074	20	268	56	323
59.897	21	TCGAATCCGAATCTTTCACC	60.014	20	276	836	1111
59.792	20	GCTTTTACCAAGGTGAGGCA	60.249	20	249	686	934
61.165	20	GCCTTTGGTGGACTTTTCAA	60.088	20	240	1	240
60.119	20	TCCCGTTTCACTCTTCTGCT	59.989	20	264	917	1180
60.135	20	ATGCCAACTCACCCATAAGC	59.962	20	276	3	278
59.773	20	GAACCGTTTAAACAACCCGA	59.839	20	271	107	377
60.41	20	ACATCTCTTTGTCTCCCCCA	59.505	20	256	199	454

60.012	20	CAAGTTTCATCTTCCTCGCC	59.813	20	199	229	427
60.263	20	TTACCGTGTGTGTGCATGTG	60.071	20	104	1210	1313
58.388	25	TCAAGTAAATCAGCCGTCCC	60.074	20	270	0	269
59.973	20	GTCGGGAGAGAGGGGTAAAT	59.397	20	279	20	298
60.484	20	GGGGTGGAAAGGGTTTAGAA	60.159	20	245	485	729
60.475	20	TGGCAATGTGGTGGTAAAGA	59.964	20	187	164	350
60.011	20	CCATCGTTGTCGAAATCCTT	59.933	20	207	274	480
59.001	20	ATGAATGGATTCCGGTCAAA	60.133	20	276	6	281
59.928	20	TGGAGCATACGCAACATCAT	60.104	20	248	28	275
60.096	20	TGGGGCCTACGAGAAATATG	59.916	20	170	311	480
59.971	20	GGATTTCCGTTGTTATTGCC	59.277	20	276	83	358
59.971	20	TGGTTAGCACCAAATAGCCA	59.182	20	194	50	243
60.607	20	GAAAGTTGGCTCAAGCTCGT	59.621	20	274	12	285
59.797	20	GAGAGCTATACCGACGGCAG	60	20	256	386	641
59.984	20	AACACACAGAAGGGAGTGGG	60.002	20	240	635	874
59.935	20	CGTCGGTCAGAACCTTGATT	60.111	20	166	26	191
60.074	20	ATTTCCTTGGCCTTCTTCGT	60.074	20	189	1432	1620
59.939	20	GTCTTGGGGGAGTAACCCAT	60.052	20	217	2444	2660
60.111	20	GAAGTGCAGTTTCCCCTTCA	60.232	20	204	816	1019
58.188	22	AAATGGAGCAAACATTACCGA	59.465	21	244	551	794
59.615	20	CCATGTCAAGGGTTCGAAGT	59.966	20	235	118	352
59.564	20	TCCTTTGCACCTATTCGTCC	60.074	20	102	1846	1947
58.427	27	TTGTTGGTGGGTTTGAGTGA	59.976	20	218	6	223
59.942	20	GGTTCGAGTCTCAATGGGAA	60.05	20	224	399	622
59.933	20	CTTAAACCCTTGAGCCATGC	59.708	20	202	137	338
60.096	20	GAAGAAATTCCCCCAAGACC	59.744	20	278	1814	2091
59.744	21	ATGTGTTTGGTTTTGGCTGC	60.934	20	220	94	313
59.231	22	TGTGGTTGAAATCTAGCCCTTT	60.001	22	149	10	158
59.981	20	GCACTGCTTTTTGGTGATGA	59.847	20	200	270	469
60.008	20	TGACACAGATTTGGTGCTTG	58.246	20	238	385	622
60.191	20	AGAGGTTAGGGTGTTGGGCT	59.994	20	255	690	944
59.933	20	ATGCCACCTCCAATTTTCAC	59.797	20	121	134	254
59.847	20	GTCATCATCGTCTTCCCCAC	60.335	20	123	28	150
57.781	24	TGTGTTTTGACAATATTTAGTGTTT	58.633	27	101	756	856
60.523	20	GTTAGGGATACCGCTAGGCA	59.205	20	273	72	344
57.034	27	GAAATTTCTTGCCCGAATCA	60.016	20	202	52	253
60.332	20	AAAAGAAGCAGCAGTCGGAA	60.132	20	116	5	120
59.402	21	CTCAAAAGAGCACCTCCTGC	60.134	20	200	9	208
60.103	20	CCTCCCGAATTTCTGTGTGT	59.966	20	211	819	1029
57.729	24	TAGTCAATGGAGGGTGGGAG	59.92	20	241	544	784
60.112	20	GTTGGATGTTAAGGATGCCG	60.331	20	275	137	411
59.95	20	CGTGAACACACCGGTTCAAT	61.814	20	270	336	605
58.405	20	GGGTTGTGGTGAAGGAGAGA	60.088	20	195	22	216
59.761	20	TGCTTAAAATGGGTTTTCCG	59.937	20	220	169	388
58.897	20	TCAATGACCACACAACCACA	59.376	20	260	135	394
60.156	20	CACTAAATAAATATTTTGGCATAT(	57.149	27	247	19	265
60.331	20	TCAGATGAAAAGCCTACAGGAA	58.973	22	126	224	349
59.722	20	AGCCGAGGACATTCTTCTGA	59.95	20	248	370	617
59.827	20	CAAGTGCAGCAACTTTTGGA	60.027	20	241	65	305
59.882	20	TCCTCCCAACATATTGTGCAT	60.207	21	225	57	281
58.028	20	AATCTCGCATCCCAATTCTG	60.036	20	121	72	192
60.54	20	GCGACTGGCACCTTATCTTC	59.843	20	179	2076	2254
59.955	20	TGGAACCCATCAACACAAAA	59.792	20	263	379	641
59.75	20	CGGGTTTCGCTATTCATCAT	59.923	20	153	12	164
57.416	21	AGTTTTTAATCGTGGCCGCT	60.976	20	156	135	290
60.46	20	ACCTTTTACGGGGAAGGAAA	59.808	20	191	81	271
59.799	22	GGAGTGTTGGAGGAGGAGTG	59.682	20	276	6	281
58.649	22	CGTGGTTCGATCGTAAATGA	59.542	20	217	114	330

57.6	26	AGTCAAGGAGGGCAGAGTGA	59.986	20	274	4	277
60.006	20	TGCATTTATCCGATTTATCAAAA	58.561	23	207	814	1020
59.836	20	GTCGTGGATATGGGCTGACT	59.957	20	196	1105	1300
60.134	20	TCATGTTAATTGGTGGTATCATTTC	59.898	25	188	3400	3587
59.955	20	TGAAACTGAAGATTTGGGGC	60.051	20	176	1234	1409
59.162	22	ACAGGGCTTGGGACAAACTA	59.592	20	245	1892	2136
60.073	20	CCAGTTTTACCACCGCCTTA	59.99	20	172	803	974
59.937	21	GGGTATGTGGACAGAGTGGG	60.238	20	125	81	205
58.572	20	ATTTGTGCGAGTCTCGTGTG	59.905	20	248	698	945
59.824	20	TATTATCACGGTTTTCCGGG	59.655	20	150	510	659
58.858	22	TCATGTGTCTTCCACCATGAA	59.951	21	188	5026	5213
57.063	22	TCTTTCTTTCCCTTGCTCCA	59.926	20	221	123	343
59.976	20	CCAATGCATTACGAGTTGGT	58.502	20	224	418	641
59.992	21	TCCTAGCCCTCAATCTCGAA	59.91	20	252	608	859
60.11	20	GCACTCTCCAGCTCGATTTC	60.104	20	181	977	1157
59.325	20	GTACTAGCAACGGAGATGCG	58.555	20	215	41	255
59.976	20	CACCCGACGTATTCGAGTTT	59.993	20	249	176	424
60.081	20	TCACTTCTTGCCGATGAAGA	59.522	20	274	477	750
59.801	20	TTCATCATCAGCTGCTGTCC	59.95	20	216	1039	1254
60	20	TGCCACAGTTTCATCTTCCA	60.24	20	190	4610	4799
60.456	20	CGGAGTTGAGGACTTAACGG	59.728	20	249	109	357
59.434	20	AGGTTTGGAGCCATTTTGTG	59.971	20	142	324	465
59.933	20	CAACACAATACTAATGCAATCCG	59.437	23	223	14	236
59.169	20	TTGGGGGAGAATTGTTTGAG	59.903	20	248	84	331
60.044	20	CTTGACGACTTCGAACCTGA	59.007	20	249	113	361
59.072	21	ATCATAATTGACGCCCTTCG	59.923	20	105	11	115
60.192	20	ATGGACCCTTGTTGGTTTGA	60.21	20	146	299	444
60.11	21	GATGGAAAATGGTGATTGGG	59.991	20	187	84	270
59.01	21	CCGCACACCCCTATCTTTTA	59.953	20	229	356	584
59.135	20	ATTGCTTTGACACCACCACA	60.008	20	279	47	325
59.933	20	GACGAAAATTGCCCGTAAAA	59.943	20	247	103	349
59.693	21	ATTTCAAATCCTTCCCACCC	59.996	20	217	25	241
59.929	20	GAACATCGACTCACTGTATTGTTT	57.373	24	166	233	398
59.988	20	AGCTTCTGCTTCACACAGCA	59.929	20	232	107	338
57.321	26	TCCCTCAAAACAGATTCCCA	60.43	20	207	663	869
60.119	20	TGTCCGAAATTCTCTCACCC	60.05	20	210	38	247
60.131	20	ACTGCAACCCATCCTTTGAG	60.111	20	213	24	236
59.328	26	AAATCCTTGCACCTCCTCCT	60.074	20	132	2	133
59.848	20	AAGTTGGAACGAGCGAAAAA	59.861	20	239	87	325
59.82	20	CTCCATGCCTCTGCTCTTTT	59.574	20	186	594	779
58.158	20	GGCCCAGTAGCATGAATATGA	59.937	21	267	108	374
59.216	21	CGTAGTTGCTACATGTTAAAGAAA	57.052	25	280	21	300
59.881	20	AAAATCAAAGGCCCACAATG	59.801	20	277	2	278
60.095	20	CCCCCAGCAAAATAAAAAGG	60.637	20	162	4425	4586
59.789	20	CCCCAAGAGAGGATTTCACA	60.042	20	262	15	276
59.64	20	TCGTTTCTTCACTCCAACCC	60.088	20	162	241	402
60.363	20	ATGAAAATGGGCGAGAGTTT	58.647	20	225	231	455
59.85	20	AATACTCACGGGAAGCATCG	60.096	20	147	13	159
58.864	25	CCAATGACACGTTATGCTCAA	59.6	21	198	92	289
60.199	20	GTTGTTTTTGTCGCGGATTT	59.982	20	280	1	280
60.126	20	CAGCAATTTCGATTTCAGTGAG	59.883	22	193	262	454
59.52	20	CGGGATTTAGGTGTTTTGACC	60.579	21	280	1935	2214
59.848	20	ATCGATAATTCGCACCCAAG	59.923	20	234	960	1193
60.149	20	TCTGGGCTCCCAAATTTTTA	59.52	20	277	824	1100
59.91	20	GGGAGATGAGGGGAAAAGAG	60.008	20	171	279	449
60.088	20	TGGCTTAAGGCAATTTGTTAAT	57.918	22	241	222	462
58.573	21	CTTTGAATTGCAGCTCGACA	60.134	20	271	652	922
60.206	20	TCGTCCGAAAATCCTTTCAC	60.051	20	272	77	348

59.931	20	CCAAATGATTTCCCTTTGATG	59.26	21	229	60	288
59.805	20	CACTTTCTGCACCCCAAAAT	59.971	20	261	888	1148
58.2	22	ATACCGCTGACGACAACCAT	60.406	20	134	4	137
59.813	20	ATGTAGGGAAAGATGGCACG	59.955	20	275	412	686
59.772	20	TCTGGTTTGCAAAACTTTCTGT	58.927	22	167	896	1062
59.343	20	ATTGCTCTCCAACCCACAAC	59.973	20	257	130	386
60.13	20	ATTTCGAAAATCAACGGACG	59.938	20	162	221	382
59.6	20	GCTGGTGTTGGGAGAGAGAT	59.258	20	212	507	718
59.109	25	ATTACGTCGATCCAACGGTC	59.82	20	135	2	136
61.031	20	CCCAGTCGTATCACTTTGGC	60.517	20	201	22	222
60.184	20	CACAGGAACAAGAGCGAATG	59.44	20	273	132	404
58.895	21	ACATTGAAAACCAGCCGTTC	59.978	20	145	114	258
60.352	20	ACGGATTTGTCGATCGGTTA	60.331	20	224	1336	1559
59.182	20	GTTCGTCCCACCCTACTTGA	59.966	20	196	53	248
60.149	20	CTTCGGCAACGAAATACCAT	59.96	20	147	92	238
59.746	20	TGTAGGGAGTGGGATGTGTG	59.388	20	206	186	391
59.079	20	GGGAAATTTCAGTTGCAAACA	59.967	21	249	3	251
58.984	20	ATGTGCAGGAATCTCAAGGG	60.073	20	224	17	240
60.22	20	ACGTAGTGGGAAAAAGGGCT	59.998	20	273	127	399
60	20	CTTATTGCCTCCATCGTCGT	60.096	20	223	83	305
59.847	20	TGGGAATCTTTGCATCTTCC	60.014	20	127	333	459
60.066	20	AATTGGGGTGCAGTTCAAAG	59.971	20	180	110	289
59.813	20	CACTTAGGGGGTTATTAGTGGAA	58.515	23	279	7706	7984
60.096	20	CATCCACGTGTGCTAACTCG	60.324	20	184	2394	2577
59.952	21	AAACAAATATGGCCTCCCAA	59.273	20	196	584	779
58.83	21	ATGACAACCCACCAGAAAGC	59.973	20	172	950	1121
59.945	20	AGACCGAGCGCAATGAATTA	60.742	20	225	265	489
59.916	20	GTTGTGTAGCCAAACATGCG	60.18	20	151	0	150
60.207	20	ATTAAATGCCGGTCAAAGCA	60.456	20	267	150	416
60.448	20	TAAGATCGAACGATCCAGCC	60.177	20	208	97	304
59.882	20	GAAACTGACCACCACGGAAT	59.827	20	193	462	654
59.281	20	ACAAAACCACGGCTGTCAAT	60.419	20	183	16	198
59.833	20	AAAAACACGAAACTTGATTGTGA[illegible]	59.952	24	270	102	371
59.055	20	CGGAATGAGCCACAGAATTT	60.074	20	240	27	266
59.925	20	TGTTTGGTTGCTTGCTTGTT	59.366	20	204	1152	1355
59.074	21	GGGGTCTTCTTTTGGGACTC	59.912	20	180	462	641
59.332	26	CCGGGACCGAAACATAGAT	59.759	19	232	2015	2246
59.967	20	CCAATTTGAATGGTTATTACCTGA	59.191	24	141	581	721
60.06	20	TGGATGACGAGGTCGAGATT	60.622	20	253	91	343
59.691	20	TCGGAAATAGGTGGTTTTCG	59.931	20	267	18	284
59.898	25	AGGAGCAATACTGCTTGTGGA	59.89	21	146	10	155
60.081	20	CAACTTGGGCTAAGGATTTTG	58.751	21	152	59	210
59.887	20	GCGGTATACTGCCTTTCCAA	60.096	20	197	19	215
57.806	25	CAATGGTAAAACAACGCTCG	59.226	20	265	1	265
60.088	20	AACATGGCCGGAGAATACAG	59.955	20	258	144	401
59.955	20	TCGAGACTTCGGTTGGTTTC	60.232	20	165	3	167
60.001	20	TTTGTCTCCTGGGAGAATGG	60.042	20	253	396	648
60.118	20	GAGATGTGTGTGTGCGTGTG	59.772	20	264	983	1246
59.847	21	ATCATTCCCAGCCACACATT	60.203	20	152	9	160
59.989	20	AACCGTCAGATTTCAAACCG	59.971	20	230	384	613
60.111	20	ATTCACTCACCACCGTCCAT	60.246	20	253	22	274
59.966	20	GGGAGAGAGAGCAGCAGAGA	59.969	20	247	235	481
60.668	20	CGGATGAACACCCCTACTTG	60.366	20	256	1263	1518
60.274	20	TCAGCCTACTTTCGGCCTTA	59.975	20	132	130	261
60.493	20	GAACGATGCTGCCATGATTT	61.014	20	215	456	670
59.797	20	AGGCACCAAAGAGATAGGCA	59.836	20	260	6	265
59.993	20	TTAACCGCCAAACTCAAACC	59.975	20	109	507	615
59.502	20	TTAGATCATACACGTGGCCG	59.566	20	152	416	567

59.73	21	CAAGTTGAAGTTATGCCCCC	59.429	20	275	0	274
62.397	18	TCATGGAAACATGACGTGCT	60.12	20	228	39	266
60.011	20	CTGGGTAAAAATTGTGCGCT	60.131	20	243	1087	1329
59.901	22	TATTTTGGGCCTGAATGGAC	59.762	20	141	18	158
59.391	21	GTCCACGGTGGTGCTAGAGT	60.182	20	105	62	166
59.799	20	CAGTTTTCGTTGCACCCTTT	60.147	20	263	1664	1926
59.424	23	TTTGTGGTGGTGAAGATGGA	59.935	20	263	0	262
60.125	20	GTGGGGGACTTATCTGTTAGAA	57.236	22	278	53	330
59.597	20	TGACCGAATTCAGACATCCA	60.048	20	174	1005	1178
59.93	20	GGTGGTATGGTGTAGAGGACC	58.242	21	264	42	305
60.683	20	CCATTGGTATTGACAGCGTG	59.988	20	115	487	601
59.875	20	GCACTCTCACGAGCTCACAC	59.771	20	140	882	1021
59.272	20	TCATGCCTAAATGTTTTGATGC	59.971	22	180	282	461
59.75	20	AGAGCTTGCCGAGAGAAAGA	59.454	20	159	180	338
60.361	20	TGTGCTTCTCCAAGGCTTTT	59.993	20	245	132	376
60.86	20	TGTGGCTTATGGAGGTTCAA	59.123	20	132	172	303
60.725	20	TTCAATTCTCTGCGTTGTGC	59.995	20	205	375	579
60.22	20	AAATTACGGCCACAACAAGC	60.003	20	175	2203	2377
60.035	20	ATCTCACTTCACCTCCCCCT	59.929	20	202	78	279
59.262	20	TGTCTGTGAGAATGGCCAAG	59.831	20	201	107	307
59.973	20	CAAGTCCAAGTCCGAAGACC	59.697	20	223	19	241
59.939	20	GCTAAAAATGTGGTTGGCGT	60.003	20	279	1310	1588
59.897	20	ACGCCACAAATTTTCTCACC	59.978	20	211	429	639
59.801	20	GACCGAAAACCGTTCAAAAA	59.953	20	224	221	444
60.237	20	TCGCCCTTTGGATTTATTTG	59.901	20	278	48	325
59.329	20	GGAATAAAGGGTGGTTGGGT	59.919	20	203	53	255
59.9	20	GTCAGAATGGATTGAGTGCG	59.245	20	156	71	226
59.973	20	TCCCTAACAAATCACACCGA	58.973	20	111	142	252
58.644	23	TGTCCAAACAAATAAAAACATTC	60.139	24	187	15	201
60.156	20	GCATGAGAAAATCCTCCCAA	60.014	20	110	18	127
59.97	25	TGGGTGGTGGAAAAGAAGTT	59.425	20	199	38	236
57.416	26	CACACACAATGTTGGGAGGA	60.416	20	130	28	157
59.498	23	ATATTGGTTTCGGATCGTGC	59.791	20	221	4327	4547
59.231	22	TGTGGTTGAAATCTAGCCCTTT	60.001	22	153	10	162
59.485	21	CGCATCACTCTTTTTAGCCA	59.062	20	263	466	728
60.183	20	GTTATGCGTTTTGACGGGAT	59.829	20	210	250	459
59.008	25	GATTCGGAAGGTCGACAACT	59.141	20	142	8	149
59.583	21	CAAATGTCGCGGATTTTGAT	60.837	20	251	150	400
59.948	20	CTTTTATCCCCAAAATGCCC	60.489	20	208	412	619
59.447	21	GCCATGATAAATCCACCACA	59.205	20	268	40	307
60.053	20	AAAGCAATAATATTTGTGCCATGA	59.783	24	201	74	274
60.27	20	CGCGGACACATCATGTAAAC	59.995	20	185	102	286
59.943	20	TCACACATAATTGAGTTATCATTG	59.685	26	228	90	317
60.112	20	AGGTGAGGGAGACAATGTGG	59.962	20	239	457	695
59.811	20	ACCGTGTCGGATACACATGA	59.837	20	213	31	243
59.997	20	AGAGAGCATTCCCACAAACA	58.287	20	107	643	749
60.016	20	GCAAGGACGAATTCCAAGAG	59.813	20	269	1423	1691
59.915	20	TCCCCTATCGGTATTTTCCC	59.978	20	207	90	296
59.933	20	TGGGCTAGCAGACCATCTCT	59.973	20	259	68	326
60.24	20	TTGCCACCCCTAGAGAGAGA	59.943	20	247	76	322
59.628	20	ACTTCACCCGTACGTTGCAC	61.016	20	169	526	694
58.849	23	ACGATTTTGCCCTTGTATGC	59.967	20	213	7	219
60.822	20	ATTAGGGGAGAGAGGGCTCA	60.169	20	240	218	457
59.543	20	ACCCGAAGCTAGGAAGAAGC	59.983	20	142	87	228
60.025	20	GTGCACATTTCCCTTTCACC	60.362	20	247	225	471
59.5	21	AACGTGCCCTATTCACATTTTT	59.778	22	141	12	152
59.569	20	GACCAACGTGGCCTAACCTA	59.993	20	254	439	692
59.572	20	CATTGCAGATTTTGATGAGCA	59.824	21	253	2067	2319

59.823	20	TAAAAAGGAAACAGCCGTCG	60.236	20	126	145	270
59.546	20	AACCTTTGATCACACACGCA	60.16	20	171	13	183
59.989	20	TCCAACTCATCTTTCGGACC	60.05	20	277	586	862
60.932	20	CCCTAATTTATGAAAGAGTGGGA	58.576	23	195	313	507
60.088	20	TTGTGTGGGTCGATTCTTGA	60.088	20	257	1169	1425
60.11	20	CAACACCAACCACGAACAAC	59.897	20	263	58	320
59.104	22	TCCATCCTCCATAATCCTGC	59.854	20	221	95	315
60.375	20	TGCCTGCATGATCAAATAGC	59.799	20	247	2644	2890
57.516	24	ATTGACCACCATCATTGCG	60.346	19	158	98	255
59.352	23	GGACGCACCAGACTGTTTCT	60.307	20	199	867	1065
60.285	20	GTGTGTTTAGCAGGGACCGT	60.035	20	198	1696	1893
59.972	20	GTGTGGATGTGCGTGTTTTC	60.016	20	223	620	842
61.041	19	GCTAGACCATTGTAAGGGGG	58.528	20	194	70	263
60.288	20	TCCACGGATGTAAATTGTTCC	59.677	21	124	1727	1850
57.605	22	GCCATGCGCAGTATAATTGA	59.691	20	138	91	228
60.032	20	ACATTTTTCCACACTCAGCG	58.775	20	280	261	540
60.089	20	ATGGGAAGGGGAGTTAGCAT	59.789	20	108	43	150
60.015	21	TGACTCATACCATGGAGCACA	60.128	21	237	399	635
59.945	20	ACCATAGAACCACACGGCTC	59.997	20	267	146	412
59.982	20	ACCATAGAACCACACGGCTC	59.997	20	183	232	414
59.72	20	TCGTCCGAAAGAAACCTTTG	60.22	20	264	28	291
59.962	20	TCACAACCAAGGTTTGGACA	59.976	20	215	909	1123
58.869	20	ACAAGAGGATGAAGGCCAGA	59.803	20	258	78	335
59.347	20	CCTTCGGATTGCATTACCAC	60.331	20	222	51	272
58.566	20	TTACAGATAACCGTTCCCGC	59.96	20	158	0	157
60.103	20	CGGGAGTATTTGGTCACACC	60.232	20	190	549	738
59.598	20	GGCAGGCACAGAAGAGAAGA	60.678	20	270	2186	2455
59.098	23	TCATTGACCAAGTCTTGACGA	59.278	21	252	228	479
60.111	20	TGCAACCTACTCAAAGACTTCGT	60.341	23	266	571	836
60.132	20	TGATGCCCCAAATCTGAAAT	60.274	20	245	33	277
60.209	20	GCTTACTGATGCAGAAGCCC	59.985	20	249	105	353
59.844	20	ACCCCTCACATGCTCTTCAC	60.12	20	275	630	904
59.592	20	TTCGTTGACCCCATATGTCA	59.774	20	208	444	651
59.82	20	CAGCACCAGAATTGCTCCTA	59.024	20	144	184	327
59.488	20	GATTGCCGACTGAACCTCAT	60.081	20	273	152	424
57.837	27	TCCACACCATCTCAATCCAA	59.893	20	234	543	776
58.64	21	CCCAACACTCCATTCTTCGT	59.966	20	117	9	125
59.703	21	AACCAGAACGGCTTGAAAAA	59.724	20	280	1057	1336
59.933	20	CGATCGGTCAATCGAATTTT	59.901	20	177	434	610
58.801	20	AACCCTAGTTTCTGCTGCGA	60.015	20	175	20	194
60.177	20	TGTCGTAGATCACACGGTGG	60.593	20	269	2654	2922
62.397	18	TCATGGAAACATGACGTGCT	60.12	20	230	39	268
60.081	21	AACTCGATAACGGATGCTCG	60.235	20	223	12	234
59.903	20	CCTTCAGAGGCATGGTTTGT	60.111	20	176	44	219
60.418	20	ACGGGAGGGAGAGAGGGTAT	61.214	20	255	212	466
59.465	20	CCATTCTTTTAGGGCCAACA	59.931	20	276	388	663
59.993	20	CGATCCCTCTCTCTCACGTC	59.945	20	170	101	270
60.362	20	AATGGATCGGAAGCAATGAG	60.036	20	179	789	967
60.104	20	AAATCCTTTCCCCAATAAACG	59.216	21	280	3265	3544
59.916	20	GACACCGTTACGCAGACTCA	59.905	20	128	1480	1607
59.874	20	TCCCCTAATTCACGTGTTGA	58.973	20	263	612	874
59.976	20	GCCGATTCCTCCTTTCTCTC	60.293	20	128	4302	4429
61.006	20	TCGTCGATGATCGTATAGCAA	59.295	21	279	33	311
59.903	20	AACACGCGTTTTTAGGGTTT	58.676	20	217	72	288
59.109	20	GAGTGGGAGATTGTTGGGAA	59.903	20	241	70	310
59.232	20	TGGGTTCTCTCTCCCCTTTT	60.045	20	210	136	345
59.752	27	ACGTGTGAGACTTCGTGTCG	59.94	20	276	1098	1373
60.161	20	AACTGCTGGACCTCCACAAC	60.159	20	143	101	243

59.973	20	CCCCACCCAAGTATTTTTGA	59.657	20	101	140	240
60.068	20	CATGACTCTCAGCATATGGGC	60.64	21	262	32	293
58.947	20	CCACCTTACTCACGCCTCTC	59.867	20	251	40	290
59.993	20	ATGGTGGATTTTCCTTTCCC	59.996	20	172	45	216
59.767	20	CACACAAGCAACTTCCCTCA	59.873	20	239	640	878
59.73	20	TATTTTGGGCTTTTGCTTGC	60.205	20	219	2416	2634
59.988	20	CAACGAGCCTTCCTTTTGTC	59.853	20	257	356	612
60.496	21	TGAAGAAGGAAGCTAAAATGCAG	60.03	23	155	17	171
59.41	20	GCATTTGTTAGAGGTTTTGACCA	60.389	23	280	2196	2475
59.288	20	GGAGATCGACCAATTTGGAA	59.871	20	232	80	311
59.813	20	CGTGGTTCGATCGTAAGTGA	59.716	20	210	307	516
60.048	20	CATCTCTCCTTCTCTTTCTCGG	59.593	22	252	16	267
59.975	20	AAAAATGAAAACGTTACTTCTGCC	59.949	24	178	129	306
59.55	20	GTGGACTCTTGGGGTTGAGA	60.088	20	227	20	246
59.797	20	CCCTTTTCTCCTTCTCGCTT	59.955	20	221	255	475
58.446	20	GTCTCGATGGTGTGATCGG	60.063	19	158	15	172
60.269	26	CATATATGGGGAGCGTACCG	60.188	20	273	5	277
60	22	GCCGAAAATACCCCCAACTA	61.029	20	172	94	265
59.954	21	GCTTGCCCAGACTTTCAATTT	60.607	21	280	54	333
60.031	20	GGTGGTGGGCGAGTAATAAA	59.823	20	123	289	411
59.579	20	AAGTCCACACAGGTGACTCG	58.725	20	148	214	361
59.989	20	ATTGGCGAGGGGAGAGTG	61.188	18	228	1405	1632
60.043	20	TCAGCTCCAGCTCCTTGATT	60.096	20	219	1082	1300
57.439	21	TCAACTTTCGGAAGCGTACA	59.464	20	117	37	153
59.32	20	CACGCATTCCCAGAAAATCT	60.074	20	118	10	127
59.966	20	CCCTACGGGATTCTCCCTAC	59.783	20	226	356	581
60.125	20	AACCGAAATCACCGCTACAC	60	20	160	144	303
59.933	20	AGAGCAAACTCCGATGGCTA	59.978	20	256	1308	1563
59.758	20	AAGACACCTAGTTGCCCCCT	59.994	20	183	68	250
60.035	20	GCCACTGATCCCCAGAGTAA	60.073	20	267	1065	1331
59.724	24	TGCAGGGTTCGATTCACATA	60.073	20	238	269	506
60.688	20	CTTGGCGGATCATATCACAC	58.953	20	228	4	231
59.31	20	TTCACGAACACACCCACCTA	60.001	20	140	205	344
60.08	20	AAAAAGTGCGCCAAAAACC	60.103	19	269	130	398
60.147	20	GTGCCAGTGAGGACAAAACA	59.726	20	234	25	258
59.839	20	CGGTTATTAGTTATCACCCGAAA	59.322	23	191	785	975
59.518	20	GCGTTTTAACTCGTCGTGGT	60.176	20	233	1096	1328
59.917	20	GAGCTGAAGCACTTTGACCC	59.997	20	148	1628	1775
60.042	20	AAAATATGCAGTGGCTTGGG	59.96	20	173	1812	1984
59.586	20	GAACCTGAAATGCAAGGAGC	59.82	20	129	1258	1386
59.473	20	GAACTAGGCGAGAACGATGC	59.985	20	178	390	567
59.896	21	GCTTAGGAGGGTTTCCTTGG	60.068	20	239	227	465
60.155	27	CGTAAAAGGAAATGCATGAAAA	59.151	22	187	264	450
59.813	20	CCTTGTTCTCCTGCCTTGAC	59.844	20	218	99	316
60.56	20	ATCGGTAATTCACACGCACA	59.995	20	181	66	246
60.175	20	GTTGGCCCTTGATTGTTGTT	59.836	20	183	142	324
59.415	20	AACTAAAGGGGAAGCTCTGC	57.695	20	219	567	785
60.146	20	CTGGGTACGGCTAGAAGGAA	59.331	20	152	238	389
58.999	20	AGGGTCGTTCATGGATTACA	57.864	20	174	347	520
59.973	22	GAAGTGGGAGGATTTGGACA	59.903	20	112	482	593
59.933	20	AGAAGTGTAGAGCGGTCGGA	60.012	20	135	1792	1926
60.157	27	GCCTGCAATTACTTGGGGTA	59.96	20	156	68	223
60.148	21	CGTCGAAACGGATATTTTGG	60.315	20	232	48	279
60.397	20	GATGCCTTTTTGCTTTGCTC	59.967	20	187	1919	2105
59.67	20	TGTAACTTGATTGCTAAGAAATTG	58.849	25	251	90	340
59.694	20	GCATATCCAGCACGCTAACA	59.862	20	263	146	408
60.142	20	TGTAGTGAACTCGACGCTGG	60.049	20	206	209	414
60.188	20	TTTGCATGGGTCAAAGTGAG	59.691	20	264	8	271

60.042	20	GTCCACATTTTGCAGCCTTT	60.118	20	117	65	181
58.819	20	TGGTATCAGAGCGGGATTTC	60.036	20	142	629	770
60.029	20	AAATGGACAGTTACGGGCTG	59.993	20	217	127	343
59.175	22	TTGGGATATGAAAATGACACCT	58.303	22	117	295	411
59.973	24	CTCAGCATACGGGAAGGGTA	60.088	20	248	8	255
59.864	20	ATCTGGCCCATCTGAAACAC	59.934	20	152	394	545
59.872	20	CCCCTCACCAATGTCTCACT	59.962	20	230	69	298
58.895	20	AAGAATTGATCATGCGCGTT	60.615	20	221	1572	1792
59.174	20	TATGGGAACTGCAACATGGA	59.924	20	143	600	742
60.118	20	ATTCCCACAACAAAATGCAA	58.869	20	271	376	646
59.846	20	CAAGCTGTCAACCCAAAACA	59.734	20	269	42	310
59.409	20	GGAAGAAACTCAGCTGAACCA	59.463	21	187	179	365
60.135	20	TGGAAACAAGGGAAAGTCAA	58.173	20	218	0	217
60.232	20	TTAAGAGGGAGGGAAAAGGC	59.661	20	176	851	1026
57.86	20	TCGACGTGTAATCACCCTCA	60.112	20	263	9	271
60.157	20	TTCGGGTCCAGAAAGTTGTC	60.088	20	279	273	551
60.067	20	GCCATAACCGTGTAAAAATGC	59.38	21	272	16	287
59.694	20	TGTGAATGCACCTGAATACG	58.129	20	244	128	371
60.159	24	CCTAGGCCTCTTCTTCCACA	59.425	20	166	6	171
59.621	24	TGATTCACGCTAAAGCACAA	58.075	20	226	8	233
59.942	20	TTCGTCGACTACTCATGCAA	57.997	20	189	2401	2589
59.371	20	CAAATCCCACCATTTAGCACT	58.962	21	280	38	317
60.68	20	AAAGACCCGCAACGTACAAC	60.037	20	252	12	263
60.003	20	TCAACAGAAACGCACACACA	59.912	20	154	115	268
59.017	21	ACGGTGGTGCAAGAGTGATT	60.577	20	146	1	146
60.905	20	GGGATCCCTCGTATCTTTCC	59.728	20	150	4533	4682
59.839	20	GAGGGTGAGTTGGCATCATT	59.934	20	212	118	329
59.434	21	TCGACTTTCAGTATTGCGATTG	60.264	22	264	357	620
60.11	20	ATTCGAACACCACCGACTTC	59.973	20	276	118	393
59.866	21	TGGCACATATTTCAACGAGTG	59.6	21	266	8	273
59.261	20	TTGCATGCATTGCTTAAGTCT	58.597	21	250	209	458
59.82	20	CATGGGGAGAGGAGTTAGCA	60.21	20	161	253	413
59.127	20	CCAATATGGCGAACCACTCT	59.955	20	251	190	440
60.573	20	TTGATGGGGTGAAAATTCTTG	59.787	21	280	79	358
59.854	20	CTTTGTTTGGGAGTCGAAGC	59.853	20	243	1167	1409
59.596	20	AAGCATTGATGTCTGAATTTGAA	58.755	23	240	273	512
60.056	20	CAACAGTTTGGCATCAGAGC	59.445	20	270	117	386
60.216	21	TGTTGCCAGAAAAAATTGTCG	59.706	20	250	20	269
59.734	20	CGTGTATGCTGCATGTTTGA	59.307	20	238	347	584
59.845	20	CCTATGATGGTATGGTGTGGAG	59.195	22	253	73	325
59.998	21	GGGTTTCTCTGTCACCCCTT	60.349	20	229	53	281
59.988	20	GAATGAGCAGACGCGTTTTT	60.395	20	277	24	300
60.068	20	AGCTGCCTGCTTCATGTCTT	60.164	20	230	115	344
60.112	20	CGATAAATCATGCTGATATCGTAC	60.247	25	174	134	307
60.21	20	ATCGGCTACTTACCGCAGAA	59.867	20	120	26	145
59.791	23	CTTCAAATCCCTCCCCAAGT	60.298	20	154	347	500
60.008	20	GCAAGAGTAACCGGAAACCA	60.11	20	190	3211	3400
60.188	20	CGTTTTGACAATATTTAGTGTTTTG	58.7	26	134	996	1129
60.291	20	CTCATATGACCACAACCCGA	59.369	20	257	24	280
60.201	20	GACCAAACCCCAAAGTGAAA	59.807	20	270	113	382
59.722	21	TGTGTGGCTTCTTACACCCA	60.152	20	271	2103	2373
60.044	20	GAGTTTTGACGTCCGAGTCC	59.703	20	181	1207	1387
59.439	20	TTTTTACCATTTTGCCCTCG	59.937	20	208	1044	1251
59.989	20	CCCATCTCCACGACGTTATC	60.34	20	271	1506	1776
59.968	20	ACTTACACACGCACGCACTC	59.977	20	118	27	144
58.413	20	CGATCCATATTGGGTCAAAC	57.735	20	211	9	219
60.003	20	TACCAAAGAGCAGGGAGGAA	59.807	20	160	1877	2036
60.004	20	GGATCAGTATGGCAGTTGGG	60.34	20	128	65	192

59.092	25	CTGATCTGGGTTGGGAATTG	60.309	20	115	3	117
59.711	20	ATATGTGCTGGAGCCTTGCT	59.866	20	260	2116	2375
59.999	20	GACCCACTGACCTCCGTAAA	59.966	20	229	376	604
59.993	22	ACGCAAGTGTACGTGGTCAA	60.222	20	222	251	472
59.933	20	TCCCAGCCTGTTTCAATAATTC	60.312	22	241	2912	3152
60.045	20	TTTTTCGAGGTCCATTTTCG	60.046	20	249	6	254
62.397	18	AAGTATTGGTCGATCGGTTTTT	58.931	22	136	39	174
59.992	20	TTCACAGAGGGTGGTTTTCC	59.943	20	224	394	617
59.636	21	TCGCGGATTTTTGGATAAAC	59.907	20	226	0	225
58.655	20	GCAGCAAATCGATCCATACC	60.444	20	209	970	1178
59.907	20	CGGATTTATTGGTTATTACCTGA	57.222	23	182	469	650
59.355	20	TGGGGAAGTGTTTATCAGGC	59.933	20	192	156	347
57.842	20	GGTAGTTGATGAAAGCCGGA	60.074	20	179	31	209
59.95	20	CACACTTTACACACACTGAAAATG	59.163	25	182	359	540
60.142	20	CAGAGCCCACGGTTAGTCTC	59.867	20	245	139	383
59.989	20	GGGTTGTGTGCCCTATCATT	59.676	20	254	8	261
59.918	21	GTTTCTTTGTGGGTTTGGGA	59.807	20	191	4815	5005
58.976	23	TCGCTCAACCAAGTAAGTCG	59.072	20	201	362	562
58.955	22	TTGGGAGGAGAGATTGTTGG	60.042	20	277	17	293
59.586	22	GGTTCCCACGTTACCAAAAA	59.702	20	254	2114	2367
59.985	20	GCTAACACCACGCCACTACA	59.788	20	215	1241	1455
60.149	20	AGCAGTTATTGTGATGTGGGTG	59.92	22	251	20	270
60.615	20	GTCGTTGCCTCATCTTGGTT	60.119	20	260	270	529
60.162	20	TGGGAGGGAGATAATAGGGC	60.245	20	274	160	433
59.938	20	TTTGTGCTTAATTTTGTTTCTAATCC	59.969	26	177	148	324
60.096	20	GAGAGGATTTTGGGACGACA	60.05	20	197	941	1137
60.008	20	ATCCCCAATTTACCAAACCC	59.756	20	199	3	201
59.769	22	CGATCTGGTATCAGCGGTTT	60.096	20	200	343	542
59.066	21	ATTTTTGGATGAGGCAGTGG	59.933	20	279	1229	1507
59.833	20	TTTTCTGACCAACACCACCA	59.976	20	279	1212	1490
58.32	21	AAGGAGGAAGTGAGGGGAGA	60.186	20	232	15	246
59.504	20	TTGACAATATTTAGTGTTTTGACAT	58.99	27	145	344	488
60.008	20	CCTTTCTCTTGGCCTCCTCT	59.95	20	191	338	528
60.103	20	CGGGAGTATTTGGTCACACC	60.232	20	189	137	325
58.966	20	ATGCAACTCCCAAACGAACT	59.598	20	267	2413	2679
59.527	20	AGATTTACGTGGTTCGGTCG	59.993	20	153	1137	1289
59.148	21	CCACATTTAAGGGTTATCACCTG	59.654	23	102	231	332
60.051	20	GTTTGGGGTGTTTGGAGTTG	60.246	20	151	217	367
59.645	23	GGTGGGCTTGGTGTACTAACAT	60.172	22	112	917	1028
59.971	23	TAATAGGCCCACACCACCTC	59.813	20	244	61	304
57.235	25	TGGGAAGACAATTGGTGTGA	59.935	20	159	5	163
60.032	20	TGTGAGGCCCATCATACTTG	59.522	20	257	1265	1521
60.074	20	CCGATTCTGCCTCACTCATT	60.218	20	190	205	394
59.137	21	TACGAGGAGAGACCGCAAGT	60.012	20	272	77	348
59.929	20	TGTTTTGACAATATCACGACCC	59.73	22	175	241	415
59.788	20	GCAGATGCCACAAAAGGATT	60.081	20	187	843	1029
59.587	20	GGCCGTGAGTCCATTGTAGT	59.997	20	277	19	295
59.893	20	CGGTGATCATGCAACTACCA	60.539	20	279	989	1267
59.127	20	TACTACATCAACCGCCAGCA	60.28	20	278	370	647
60.026	24	CCGTGAATTTACCCATAGCG	60.335	20	117	7	123
60.081	20	GGAGGGATCGGATAGAAAGG	59.859	20	254	1355	1608
60.755	20	CCAATACTGGTCGGGCATAC	60.214	20	114	1	114
59.415	20	AACAACGTTATCTGCGACAAGA	59.813	22	194	528	721
60.095	20	GGATCATTAGGGTTGCCAGA	59.894	20	124	303	426
60.969	20	TTTCTTGGCCTACACCGAAC	60.11	20	277	55	331
60.366	20	GTAGCATTTGCCGCATTTCT	60.238	20	242	963	1204
59.916	20	GAAAAGAGCTGCAAAGGGTG	59.993	20	201	552	752
58.967	24	CGATCCATCCCGTTAGTACG	60.344	20	270	466	735

59.916	20	AAACCCATTTCCACCTGTCA	60.21	20	184	281	464
60.009	20	CCATGAAAGCTTGGTTTGCT	60.249	20	115	845	959
59.962	20	TGATTCCGGAAAGAAGATGG	60.006	20	145	980	1124
59.969	20	GGCGCACTGAATTGTTGTTA	59.735	20	216	3	218
60.425	23	AGGGTCACGAATCCTTCCTT	59.935	20	265	74	338
60.554	19	AAAATGCCCTTGTATGCTGC	60.103	20	146	21	166
59.916	20	TAGGATTTGAACCTGCGACC	60.074	20	227	76	302
60.47	20	TCTGCTGCAAATTTGGAAGTTA	59.89	22	179	851	1029
59.292	20	CCGTCGATTTTCAGGTTTTG	60.472	20	186	105	290
59.844	20	CGGAGAGGACTTACCGGATT	60.455	20	242	29	270
59.756	20	TTCATTCAAGCTACCCACTCTTT	59.317	23	163	105	267
59.924	20	TGGCCATGTTGGTAGATTCA	59.924	20	150	27	176
59.924	20	TGCTTCACCACTCAATCTGC	59.992	20	189	1080	1268
60.287	20	CGAAACGTAAATCAGCAGTGA	58.978	21	228	829	1056
60.096	20	TGTCCACTTGTGTCACGGAT	60.005	20	221	67	287
59.688	20	CCCACTGCAGAAATGGAGAT	60.073	20	125	695	819
60.362	20	TCATCATTTCGATCCTGTGG	59.451	20	148	1	148
60.117	20	TGGGTTTTTCTCATCTTCGG	60.044	20	197	612	808
59.72	22	TGGCAATTTGTCTTTCAATCC	59.93	21	198	21	218
59.88	20	AGCATCTCCAATGCCAGGTA	60.624	20	201	14	214
59.658	20	CGAGTGGATCCTCTTCAAGC	59.95	20	224	1270	1493
60.074	20	CAGCACTTTTCATGTTGTTGC	59.381	21	187	854	1040
59.864	20	GATATGATTTTGCGCGGAGT	60.067	20	230	215	444
60.025	20	AACGATTGGAGCATTATGGG	59.784	20	193	142	334
59.903	20	TTTTGTCGATGATATGGTTGTG	58.413	22	192	38	229
60.173	20	TGTAACAAATAAATTAACGTACTG	57.577	27	200	1055	1254
60.074	20	GCGTGTTCTAGGTGTTCTTGG	59.788	21	225	126	350
60.378	20	CTCATTGTAATCTCTCCCAAAGTT	57.927	24	158	399	556
60.416	20	CATGCATGATGTGATGTCAGTC	59.999	22	166	41	206
59.912	23	CCATTTACTAAGAACGACATGCA/	60.404	24	277	1645	1921
60.203	20	TTTTCGGATCGAAATTAGGTT	57.759	21	205	466	670
58.754	23	TTTTTCTTTTGCACGACTGC	59.087	20	156	13	168
61.016	19	TCGTCTTCCCCAATCATCTC	60.011	20	273	4	276
60.504	20	GAGCACAACCATGGTGAGAA	59.682	20	266	115	380
59.281	20	TTTTTGAAATCCAACGACCC	59.778	20	145	115	259
57.357	20	CCATTGGCTCGTTTATTTTAGA	58.331	22	280	171	450
60.074	20	TACAGATCGGGTCTTGAGGG	60.065	20	268	443	710
60.149	20	AGTATGCTTCTCCGAGTCCG	59.454	20	219	507	725
60.064	19	GCATACAAAGGTAAGGAGGCA	59.238	21	141	636	776
60.126	20	GGGAGAAAGACACGTGGAAC	59.555	20	271	133	403
60.255	20	GCAACATAGTTTATGCATCCCA	59.859	22	232	62	293
59.901	20	ATTGTGAACCGGTGGTTCTT	59.308	20	252	120	371
59.75	20	GCATGATCAGGGTAAAATCCA	59.776	21	200	266	465
59.918	21	CCTTACACCTCACACCCCAC	60.275	20	168	456	623
59.85	20	TTTTCCTTCATTCCCATTATGTC	59.22	23	257	398	654
58.12	20	AACGGCCAAATCTAACGAAA	59.586	20	265	285	549
58.917	20	ATTTCAAAAAGTGGGGTCCA	59.264	20	265	0	264
59.787	20	GAAAGAAGAAGAGAGATAGATA(	58.05	27	130	607	736
60.073	20	CCATCGTCCTTATGGGCTAA	59.916	20	249	222	470
59.703	21	CCCTCCATCTCTCTTCTCCC	60.149	20	246	9	254
60.553	20	TCCCTGCTGCTCTCTCTCTC	59.969	20	150	3	152
59.976	20	CCAAGCCCATAAGCATCCTA	60.053	20	195	28	222
59.803	20	TTCTCCTTGTTTCCGTGGAC	60.088	20	280	65	344
59.839	20	TGATTTGATTCTTCGCGTTG	59.809	20	108	2489	2596
57.318	27	TTGGTCCTCGATTGTTATGAA	58.079	21	280	316	595
57.593	21	GGATGGACCTTCCCAAACTT	60.169	20	242	50	291
59.874	20	CGCTTCAACTTTTCCACCAT	60.11	20	161	611	771
60.318	21	TCCCAGTACGCAGACTTTTCT	58.994	21	280	34	313

59.968	20	TTAGGAATTTGTTCCCGTGC	59.938	20	267	64	330
58.62	21	CGGCTTCTCCCTATCAAATG	59.662	20	105	500	604
59.889	20	AAAAACCCCGACTCAAACTT	57.697	20	224	309	532
60.765	20	GTGGACACCTTGTCAAAGCA	59.726	20	116	29	144
59.94	20	TTGACAATATTTAGTGTTTTGACAT	58.99	27	258	602	859
58.388	20	TAAATTTGAACTTTGCCCCG	59.937	20	134	796	929
59.086	20	GTTGCCCACAAGTTCGAGTT	60.156	20	216	314	529
59.873	20	TGCAAAATGGGGATTTGATT	60.132	20	227	1055	1281
57.771	26	CACTTGGACCAGAAAAGGGA	60.081	20	109	14	122
60.11	20	AGACGTTGCATGTGCTTCTG	60.056	20	250	1697	1946
59.775	20	TGCTGAGTTTTGAGTCACGA	58.112	20	178	131	308
60.195	20	GGATCCGAACCAGAAAACCT	60.306	20	267	1851	2117
60.264	21	GAACCAAGGGATTGAGTGGA	59.903	20	275	1256	1530
60.207	21	GTTTGTTGAAGCAACTCGCA	60.035	20	230	99	328
60.39	21	GCTGATTCTGAAGGCATGGT	60.226	20	280	100	379
59.993	20	AACCAGAACCGTGAAATTCG	59.971	20	217	416	632
59.83	20	CCAACCATAATTGTTTCGGG	60.046	20	246	298	543
60.103	20	CGTCATAAACAGCCGGAAAT	59.96	20	224	1049	1272
59.833	20	CCCATAACCATGACAAACAATG	59.975	22	231	429	659
57.73	23	TGCTACAATGACGATGCCAC	60.697	20	130	20	149
59.123	20	GGTTGGGATGGAATGAAAAA	59.599	20	246	301	546
59.324	21	AAAATCACTAGCATGCACGG	58.799	20	193	14	206
60.032	20	TTCATAGTCGGCTCAATCCC	60.036	20	232	309	540
59.962	20	CCTTGCTAGATGGCCAAAAA	60.202	20	280	318	597
60.038	20	TTCCACCTCATATCATTCCCA	60.14	21	210	145	354
60.096	20	ATCCTTGTCCTCCTGTCCCT	59.929	20	179	706	884
59.931	20	CCATTGCATGCGTCAGTATC	60.104	20	225	3525	3749
59.318	20	TGCAGAGATGGTGATGGTGT	60.121	20	248	7	254
57.878	20	CGTCGCCTCTTATTGGACAT	60.096	20	252	13	264
60.027	20	ATGAGCTTTGTATGCCCCAC	59.962	20	270	89	358
57.543	18	ACAAACGCCATCAACATCAA	59.972	20	118	0	117
59.89	20	AATGATTGAAGCTTCCCCCT	59.903	20	146	319	464
60.134	20	GCTTGTTGGATGCCTTTGAT	60.081	20	153	10	162
59.844	20	CGAGTTGCTTCTATCGTCACC	59.89	21	223	676	898
59.381	20	TTGGCATGTAATGCGAGTTT	59.186	20	277	976	1252
60.096	20	CAAGACGGACAAGTTGGGTT	60.005	20	263	240	502
59.286	22	TTGGAAGCAATTGCATGATT	59.115	20	196	31	226
58.994	20	CACGTCAGCTTCATCTCCAA	59.984	20	238	325	562
59.805	20	CAATGCGCTGTGTTCTTCAT	59.871	20	146	415	560
58.123	23	AGCACCGACTGAAAATGAGG	60.255	20	178	1	178
59.713	20	TGTGCTGCTTCAATTCGTTT	59.469	20	279	49	327
60.249	20	AACCCATCCTCTCATCGAAA	59.483	20	131	108	238
59.075	20	CCATTTTGCCCTGATATGCT	59.923	20	193	104	296
59.795	20	AGCGATTTCATGGAGATTGG	60.036	20	253	662	914
59.762	20	GCACACAAATAATGGTTTCTTCC	59.771	23	215	145	359
58.867	20	TCCTCTCGCTGTATTTGCAC	59.028	20	258	711	968
59.51	20	TTTTTAAATGGATCGGCCTC	59.011	20	270	2	271
59.935	20	AAACGGGTGAGCAAGACAAC	60.156	20	256	1558	1813
58.941	19	TCCAGTTTGGGTGGTTTAGC	59.971	20	244	49	292
59.973	20	CATGTTTTTGGATTTTGGGG	60.024	20	127	948	1074
60.5	20	TCTCGAACCACTTTCAAGCC	60.375	20	275	130	404
60.712	20	GGCTTGGTTGTTGTAGATTCTG	58.767	22	280	989	1268
59.791	21	TGGGAAGATCTCTCCATTCTG	59.224	21	228	240	467
60.112	20	AGGGGTTATCACCTGAATGC	58.864	20	117	159	275
59.886	23	CGAATTAATCCGTCCAATGAA	59.781	21	247	764	1010
58.509	20	GAATCACTTTGCAAGCACCA	59.847	20	242	157	398
60.195	20	TGCAACCGAAAATTAGGGAA	60.427	20	179	140	318
59.348	24	TCAAATTTTTCTCATGCATATTTGT	59.012	25	230	516	745

60.006	20	TTTGTTATGCGTTTTGACGA	57.846	20	252	290	541
59.385	22	CACGTGCAACCAGATTCAAA	60.699	20	142	82	223
58.994	21	GCTTCCTCTGAATGCCAAAG	59.955	20	142	115	256
60.096	20	TCACGTTAAAGGGCGGTATC	59.96	20	131	21	151
59.722	20	GAAGCGAATCGTCGAGAAAC	59.962	20	198	311	508
60.082	23	TAGCCATGATCTCCCGATTC	59.998	20	228	299	526
59.642	20	GAGGGCTATGCTGATTTGTTC	58.803	21	259	47	305
59.275	22	CCTCCTTGTGTTGCCAAGTT	60.149	20	160	120	279
60.423	20	TCAAGGGACCAGAGGAATTG	60.042	20	261	67	327
59.901	20	TCGGGGTATCAGAAATCGAG	60.029	20	260	54	313
60.081	20	CCATATAACGATGCCATCCC	60.001	20	264	26	289
60.032	20	ATGGTGCAGAGGATAGACGG	60.096	20	253	1264	1516
57.152	22	ATAACCCCCACCAAAACTCC	59.919	20	250	15	264
60.255	20	CCATCCCCCTCTCTTTCTCT	59.63	20	255	144	398
59.225	22	TTCGCTTCCAGTTTTGTCCT	59.853	20	265	8	272
60.083	26	CAACAACAACGTCGTCCATC	60.008	20	198	4	201
60.818	18	CCGACAATTCATCTTTTTCTCA	59.202	22	278	11	288
60.156	20	GGCCGGATTGCTGATATAGA	60.023	20	140	935	1074
59.962	20	AAGAGCAATCTCTCTCCCCC	59.778	20	166	579	744
58.707	21	CTGCCACCATTGATGCTAGA	59.823	20	166	61	226
58.21	27	TCGCTTCAGTCAGTTATCCG	59.024	20	158	11	168
58.294	22	TACATTCATTGGGGTGTGGA	59.623	20	102	15	116
58.686	21	TGGCAGATTTAATATTTACGTTCA	59.061	25	212	167	378
59.719	20	TGGAAGGGAAAAGAACCTCA	59.641	20	189	434	622
60.361	20	GACCTGACACAACCCTCCAT	59.817	20	266	52	317
59.315	20	GTGGACGCTCCAGATTGTTT	60.119	20	268	496	763
59.134	22	GGATCATGCTGGAAGCAGTT	60.226	20	125	7	131
59.182	20	TGATACAAGTGTTGAAGCCAAA	58.346	22	203	444	646
59.871	20	GGCATGTTGACACCCCTACT	59.851	20	223	312	534
59.746	20	CGGTGATAACTAGGAACAAATCC	58.941	23	190	576	765
57.821	25	CCCGGTATGCTGACGATAAT	59.807	20	202	0	201
59.579	20	ATGGTTGCTTGATGGAGAGG	60.073	20	148	6837	6984
59.844	20	AGCAACTTGGTCTGCGATCT	60.02	20	146	1	146
59.123	20	TGGGTCCAAGAAAACCAAAG	59.942	20	118	275	392
59.89	20	ACACGGAGGTGATGCCTATC	59.957	20	218	543	760
60.025	21	TGCGTTTTGACGATATATAGTGTT	59.092	25	100	509	608
60.606	20	AAATGGTTGTTTGTCCCCAA	60.066	20	221	17	237
59.141	20	GAGGGAATCGGAGTTCATCA	60.011	20	272	320	591
60.103	20	CGTCTTCCTCCTTGGACTTG	59.837	20	267	4574	4840
60.032	20	TCCGTTGAGATTTTGCATCA	60.197	20	257	38	294
59.988	20	TCATGGATGGGGACTTGATT	60.135	20	137	664	800
59.773	20	TTGTTGGGAAAGAAGAATGGA	59.532	21	271	31	301
59.962	20	GCGAAATGAATGTACCGGAT	59.791	20	263	672	934
59.934	24	CCTGGTGGATTGTTCTGTCC	60.363	20	259	40	298
59.933	20	CCTTGACAACGACGACCAAT	60.954	20	270	570	839
60.162	20	TCACTCACACGCACTCACAA	60.07	20	103	22	124
60.058	19	TGAAGCAAGAAAAGGGGCT	59.94	19	168	591	758
58.109	22	AACATGAGACGCAAACACCA	60.16	20	256	67	322
60.081	20	CGCGTTACGAATCCAAGTTT	60.131	20	248	206	453
60.667	24	AACCGAAAAATTAATCATCACC	57.085	22	203	157	359
59.626	21	TACAAAGCTGCTCGGTCCTT	60.015	20	233	30	262
60.211	21	GAGATTGGTGGTTGCAGGAT	59.934	20	263	254	516
59.955	20	CGTGTATGTGTGTGAGTGTATATG	59.332	26	182	151	332
59.682	20	GAAAAAGTAGCGATGTCGGC	59.851	20	236	4300	4535
59.744	20	GCTTGCGATTAGGAATGGAG	59.807	20	234	314	547
59.271	22	CTCAAGAAAGTTGTGGGGTTG	59.619	21	279	11	289
60.029	20	GCAACATCTAATGGTTGTGGG	60.242	21	215	125	339
57.929	23	CCTTAGGATTCTCACAGCCG	59.83	20	263	4	266

59.926	20	TTGAGACATGGGTATTTGATGA	57.944	22	224	204	427
59.297	20	GCAAGACCAAATCACTCTTTCC	60.117	22	198	613	810
59.666	22	CATGTTTCGGAGCATGATTTT	59.952	21	268	130	397
59.566	27	ACTCACGTGAAGTATGCCCC	59.997	20	148	106	253
59.989	20	CGCAAGCATGTTAATTTTATTCA	59.213	23	175	238	412
59.78	20	GAATGCATTTCCGGTGACTT	59.939	20	280	1214	1493
60.384	21	AGTGAGAGAGAGGGCTGCTG	59.883	20	275	54	328
59.988	20	TCTTCATCGTTGTAACCCTCG	60.118	21	236	92	327
60.197	20	CCATTTCTGCCACAAAACCT	59.971	20	189	481	669
60.039	20	CCCTCCACCGCCTAACTATT	60.332	20	106	52	157
59.862	20	CAACACTTCACAGCTTCTCCC	59.899	21	221	425	645
60.088	20	ATGGATGGGGGTTAAGGAAG	60.011	20	220	69	288
60.053	20	AACAAAGGGGCAATATGCTG	59.96	20	152	112	263
59.7	20	AAGGAGGTGTTATGAAGGGATT	58.028	22	280	318	597
58.787	21	ATGTCGCAGGTTCAACGTC	59.698	19	263	12	274
60.535	20	AAAAGAGACAAAAGGCAGCAA	59.144	21	257	31	287
60.081	21	TTGGGATTGGTCGTTTCAAT	60.17	20	217	701	917
60.235	20	TGGCTTCTGTACGATCCCAT	60.483	20	218	119	336
59.41	20	TCATGAGGTGGCACAATGAT	59.925	20	215	766	980
59.855	20	CAATTCCTTCCAACACCGAA	60.863	20	171	111	281
59.834	20	CGTGTGTGTAACGTGTGTGTG	59.591	21	148	1185	1332
60.159	20	AAGAAAATAACCACGTGCCC	58.952	20	190	96	285
60.157	20	TTCTTCCTCATTGGTAGGCG	60.206	20	105	57	161
60.025	20	ATCGGAGTTGATTTGATGCC	59.9	20	230	324	553
60.032	20	TGCTTTTTATAACTCGCCCG	60.218	20	208	1323	1530
60.011	20	AGGTTGGCTCGACTTTGTTG	60.291	20	186	49	234
59.572	20	TTTCCATGGCTATGCTTCTCT	58.941	21	184	111	294
57.528	21	TCATGGGTTCTTTCCTCACA	59.059	20	279	534	812
59.951	20	TAGCAGCTGATATTCCCGGT	59.691	20	165	72	236
59.708	20	TGGATGTTGGTTTTGAGCTG	59.691	20	245	669	913
60.008	22	TGCCTAAAGAACGTTACACGG	60.172	21	266	2	267
59.939	20	GGAGAGACTCGAGATGCAATG	59.965	21	142	84	225
59.943	20	AACGAGGTAGCAAGTTGGACA	59.788	21	101	481	581
59.199	20	ATCTGCCAATGAATTCACGA	59.079	20	253	66	318
60.046	20	GGCCGGATCTCACTAAATCA	60.036	20	236	2052	2287
58.574	20	TTGATTTGTTTAATCTAATGGATG[illegible]	57.304	26	273	779	1051
59.983	21	GCGTTTTGATGATATATAGGTGTT[illegible]	58.907	26	183	126	308
59.96	20	ATCGAAGTGACGATGGAAGC	60.226	20	262	3376	3637
60.144	20	GCTTGCTGAGTTGGCATGTA	60.019	20	207	248	454
59.799	20	AAGGTGACAACCGATCAAAA	58.048	20	266	248	513
59.989	20	CCCGAACAGAGTCTCCTTGT	59.3	20	254	488	741
59.978	20	ACTGTTATGAGGCGGGATTG	59.955	20	275	2	276
60.288	21	TTCAGTCTTGAAAAGGAGCCA	59.978	21	278	54	331
59.314	20	TGTTCTTCACGGTTCAATGC	59.697	20	261	33	293
57.925	24	GGAGAGAGAGGGAGGGAGAG	59.489	20	229	251	479
60.081	21	TAACCCCAAGTCTGGGACTG	59.959	20	172	36	207
59.621	20	AGGATCGAGAGAGACGTGAGA	59.142	21	265	82	346
59.898	20	AGAAGAAGCAGAGACCGACG	59.745	20	192	86	277
60.27	20	AATCACTTCCCACACGTTCC	59.827	20	153	12	164
59.901	20	GGCCTCATGAACTCTTGAATG	59.685	21	208	881	1088
59.997	24	CCGTACCGGAAATTAACGAA	59.826	20	241	672	912
59.569	20	CACTTCCACACGAACACACA	59.15	20	236	527	762
59.986	20	CCAACTGAGTCCACTACCCG	60.557	20	167	41	207
60.074	20	GGTTATCACCGGATCGCT	58.462	18	191	1935	2125
58.035	25	CCCAAACATCCAACCAATTC	60.029	20	263	217	479
59.784	20	ACTCTATCTCTCTCACACAATCTTT	57.487	27	246	188	433
60.016	20	AAATAAGGAGGTGTTATGAAGGG[illegible]	59.323	24	184	352	535
60.309	20	AGCTCATGGATGTTTGACCC	59.934	20	242	306	547

59.939	20	TCCAGCCTTCCAATCTCAAC	60.195	20	280	24	303
60.103	20	TTCCTAAGTCGTGTTGCGTG	59.904	20	245	796	1040
59.897	20	GTAGGAGCCCACAGTCCTTG	59.721	20	157	351	507
59.91	20	CGCCGTTTTAAGACCCATTA	59.958	20	254	767	1020
59.569	20	GATATGCGAGATGGATGCCT	60.028	20	155	3547	3701
60.949	20	TTCTCAGCATATGGGAAGGG	60.029	20	235	8	242
60.049	20	TTTTTGTTATGATAGGATCAAACC	58.947	26	178	1786	1963
60.053	20	CGTGTGGGAAAGATATGCCT	59.955	20	183	995	1177
59.762	20	TCATCGATTACGTTGAGATGAA	58.241	22	280	756	1035
60.212	20	TCACCCACTCTCGTCACTTG	59.864	20	105	2	106
60.278	20	CATTCCTGTCGCCTTCAAAT	60.074	20	278	37	314
59.971	20	GATCAACCTCCTCCACCTGA	60.048	20	277	507	783
60.008	20	CCTTATTTTTAACCCTAAAAACGC	58.713	24	258	210	467
59.56	20	TGCAACGCTTGTTTCAAGAC	60.035	20	275	1067	1341
60.012	20	TTACGAGCTTGCGAGATTGA	59.712	20	276	118	393
59.917	20	TATCAGTGACCACCCAACCA	59.806	20	260	22	281
57.616	21	ACTGAAAGAGAAGCAGCCGA	60.277	20	253	66	318
60.096	22	GGTCTTGCAGGCTATTACGC	59.873	20	263	61	323
60.423	20	ATGGGATATTCGGGGAAAAG	59.978	20	206	421	626
60.531	20	TCTTTCATGCAATCGCTTTG	59.953	20	229	504	732
59.833	20	CATCCATATTTGGGAGGTGG	60.006	20	217	138	354
60.065	20	TCCTCAATCTGGTGCTTGTG	59.831	20	137	1860	1996
59.962	20	TGGTTTCCGTTTATATGACGA	58.031	21	126	81	206
60.14	20	TTTCGTGGAAGGATACCGAC	59.933	20	266	25	290
60.263	20	AATAGGGGCATCACGATGAG	59.917	20	218	2455	2672
60.029	20	AGCAGGATAAGGGAGTTAGCA	58.111	21	200	405	604
59.024	20	AGCTGTAGGCAGCGAACAAT	60.044	20	103	14	116
59.955	20	CTTGTGGGAGGAAGAAACCA	60.081	20	149	343	491
58.02	20	CAAAACACCTAAATCCCGTCA	59.847	21	255	113	367
57.235	25	ATCGAGAGGAAAGTGGAGCA	59.95	20	263	11	273
60.218	20	CGTATTTAATTCCAACTTTGGGG	60.752	23	247	45	291
60.074	20	TGCGACGATGATGATACGAT	60.065	20	139	251	389
60.096	21	TTTGGACTGTATCGGTCTACCA	59.494	22	244	23	266
60.192	20	TCATGAACTAATCAAGTTCCACA	57.291	23	256	93	348
59.853	20	ATCTTGGTGGGACTACGTGC	59.997	20	275	155	429
59.701	20	TCTCCCTAACGTGCTAACTTCA	59.041	22	244	284	527
59.452	20	CTTGGACCAGAGAGCCATTC	59.803	20	279	1259	1537
60.032	20	GGAGCCCATTGTTTTTCTGA	60.051	20	177	60	236
59.933	20	AAGCTGTGCATGAAGCAGTG	60.207	20	207	671	877
59.223	20	TTGGAATTTCTGGGAGTGGT	59.381	20	271	2038	2308
60.066	20	ATTTTGATGGGCTCCAGTTG	59.933	20	169	1968	2136
59.694	20	CCGAATGTAGTGGATACGGC	60.352	20	106	331	436
59.939	20	CATGAACAAATGATGGCTGG	59.924	20	234	282	515
59.969	21	ATAGGGCACACAACCCTTCA	60.375	20	278	135	412
60.183	20	GACGGCACCAGCTTCTTTAG	60.015	20	257	41	297
59.205	20	TCAATGTGGCGCAATTAAAA	60.074	20	278	289	566
59.962	20	CCTCTTCAAACAGAGAGCCG	60.126	20	161	849	1009
60.256	21	ATTTTATGGTCTTCCAACATTTTT	57.249	24	271	108	378
57.983	20	AGATTCATGGTGCTGCAGTT	58.316	20	189	349	537
59.694	20	GCCTTGCCTTGTCCATATTC	59.533	20	274	134	407
60.734	20	GTAAAGGGTGAGTGGTGGGA	59.82	20	273	259	531
60.073	20	GAGCGCGAAAATAAGACGAC	59.988	20	258	3632	3889
59.184	21	TGATGGTCCGGTTAATACTCG	59.831	21	190	101	290
60.149	20	GGACTTGAGCTTGTTCGGAG	59.989	20	163	245	407
60.045	20	AGCATTTGGAGATGGTTTCG	60.074	20	257	322	578
59.861	20	TTCCAAACAGTGCACGAGAG	60.025	20	193	5557	5749
60.012	20	GGGACGACAAAAAGTTCCAA	59.948	20	280	750	1029
60.008	20	TCGTCCATTGTAAACAGTTCCA	60.399	22	175	132	306

60.18	20	CTCAGCCGTACAGAAAAGGG	59.869	20	275	63	337
60.104	20	AAACTGGTGAGACTGTGGGG	60.002	20	185	1618	1802
60.156	20	GATGGATAAATGATTGCGGC	60.262	20	246	86	331
58.95	20	CTCTCAAACTCACGGCCTTC	59.989	20	130	30	159
59.555	21	TTTCTCTTAGACCGAGCCCA	59.948	20	263	3	265
60.773	19	ATGAGTTTGATGGAGCGAGG	60.218	20	270	308	577
60.074	20	TGTTTCATGTGGCATCAAGG	60.517	20	218	0	217
60.41	23	AGGCGATTCAGTCGAACAAT	59.7	20	100	36	135
60.132	20	TGGCAGCTCATAATCAGTGG	59.823	20	172	304	475
60.073	20	GCAGCAGTGCAACTGAAAAG	59.788	20	277	5001	5277
57.444	22	AAATTCATGGGGATGGACAA	59.991	20	157	2	158
58.777	23	TGCATTGAATGGACTTAGCC	58.723	20	244	24	267
59.907	20	ATGCACCCTCACCCTTTATG	59.813	20	221	28	248
60.11	20	GGCTTGGAGGGTTCTTCTTT	59.691	20	249	219	467
59.933	20	TGACCCACTCCAACTCTTCC	60.088	20	227	673	899
59.837	22	TTTGCACCTGAGGAGGTGTT	60.69	20	126	51	176
60.111	20	AATTGCCCTTTCATGCATTT	59.415	20	205	1251	1455
59.716	20	GAAACAGAGGGAGGGAAAGG	60.045	20	248	47	294
57.52	20	CCTCATTCAACAACTCATCCAC	59.454	22	280	1083	1362
58.161	22	AAGGTCGGCAGAAAAGGTTC	60.61	20	274	289	562
59.055	21	AAGAAAAAGTCGCTGCCAAA	59.996	20	274	57	330
59.942	20	TCCTGACAACGCAAGATCAG	59.984	20	240	194	433
59.316	20	GAGCAATTGTTGGACCGAGT	60.119	20	155	532	686
59.259	21	AAAAACATTGCACCCCAGAC	59.836	20	146	24	169
60.012	20	ACCGACCAAAAATCAAACCA	60.206	20	232	414	645
60.227	20	TGGATCGACAATTAGCTGGA	59.226	20	185	309	493
58.78	20	CTACCCCCGTTGGAAAAGAT	60.18	20	222	17	238
59.988	20	TGGCTGGTTCTCAAAAGTCC	60.232	20	225	21	245
59.967	20	TAATCTTGGCCGCTGATTTT	59.682	20	227	1581	1807
59.985	20	GCTGCACACCCTGTTTTGTA	59.764	20	226	3450	3675
59.566	20	TCCTGATTTCACTTGGGAGG	60.042	20	218	120	337
59.839	20	TCCTTCAATGGAGGTTCAGG	60.042	20	173	370	542
59.381	20	ACCGATTTTCCCCCATTTTA	60.361	20	277	292	568
60.246	20	GTGTGATTTGGTGAAAGCCC	60.362	20	112	104	215
60.081	20	GAGGCTTGATGATGCACAGA	59.95	20	160	5217	5376
60.081	20	TGAAGTGGCAACAATTCGAG	59.84	20	276	920	1195
59.973	20	CCGACATAATACACCGCCTT	59.845	20	136	30	165
59.939	20	CCCTTATGGTGGCTTGTTTG	60.357	20	177	432	608
60.255	20	ACTTCCCGGACAGCATTAGA	59.694	20	226	376	601
59.549	20	GCGGCAGAATGAAGAAGAAA	60.469	20	127	273	399
60.073	20	TCCATGCAAGTCCATTCTGT	59.09	20	258	89	346
59.933	20	ATCGGTTATCACCGGATCG	60.697	19	209	1369	1577
60.277	20	CCGTGTCCGTGTACATTTG	58.394	19	269	139	407
59.435	20	TCAACCATTTTTGTGCTCCTT	59.601	21	152	259	410
59.625	22	GCGCGCTGTATATCTAACGA	59.104	20	153	62	214
59.992	20	TTTTAGAGCATCCCCAGTGTG	60.118	21	278	214	491
60.366	20	TGGCTCTGGTACCATGTTGA	60.112	20	218	6	223
59.229	20	CCTTCACGAGTGGTGACAAA	59.72	20	106	677	782
60.17	20	TTTTGGTGTTTTGCGTGTGT	60.05	20	241	25	265
59.555	21	AAGAGAAAACAAGAGGGGGC	59.691	20	102	3	104
60.042	20	GTTTACTTTTGGGACGGCTG	59.609	20	182	426	607
58.439	25	TCTGGGATAAGGAGCATCAGA	59.782	21	252	919	1170
60.03	22	GGGTTTGGAATGTTAGCTGG	59.429	20	267	304	570
59.752	20	GCTACAGCAGCCATTGATGA	59.979	20	128	644	771
59.955	20	TTGCAGATCTTCATCCAACAA	59.262	21	244	32	275
59.499	20	CCTCAATTCGATCCTGGAAA	60.006	20	179	48	226
59.664	20	GAATTGCGGTCGTCTTCTTC	59.82	20	254	825	1078
60.152	20	CAATTTGTTTCGAATTTTGCTG	59.64	22	172	257	428

60.156	20	CCCTGCTTCTGAAACCCTAGT	59.759	21	227	598	824
58.796	20	TCTTGGGAACCCTTGAGATG	60.042	20	257	965	1221
59.724	23	GGGAGTTTGCACCAGATCAC	60.52	20	276	19	294
60.095	20	TGAATCTGCATAGCCTCTACGA	60.003	22	188	161	348
60.051	20	AGGAAGGGTCTCGTGTGTATG	59.076	21	274	486	759
58.107	21	GGATCTCAAGCCATTTCATGT	58.998	21	222	469	690
60.363	20	TTGATCGACAGCATCCTAGC	58.979	20	228	330	557
59.113	20	ATTTCTTGAGTCGGGGGAAT	59.766	20	278	580	857
60.455	23	GGGAGATCTAGCAGGATACGC	60.209	21	239	247	485
59.91	20	GCGCCTAGAATTCGTCAAAG	59.982	20	260	228	487
60.029	21	TGTGCAGATCTAATGGCACAG	59.879	21	174	154	327
59.203	20	ACGCACGCACTCAGTTACAC	59.977	20	266	1203	1468
58.289	22	GACGCCAATGGAACCTTTTA	59.938	20	106	45	150
59.803	20	AAAATGGGTTTCCTTACCCG	60.048	20	220	138	357
60.29	21	AAATTGAGCTCCATAGCCGA	59.807	20	121	32	152
58.82	27	ACAAAAATGCCCTTGAATGC	59.945	20	265	0	264
60.081	20	CAGACCAGACTAGGGGCAAA	60.246	20	178	242	419
60.298	20	TTGATTTTTAATGGGGCAGG	59.767	20	280	66	345
59.894	20	CCGAATGCACTCACACAAAC	60.16	20	143	516	658
60.69	20	GCACCGGAGAAGAACTACCA	60.255	20	257	508	764
60.074	20	TTTCTCAACCGAGCTCACC	58.945	19	106	305	410
60.088	20	ATCCGCCCCTTATCTCTGTT	59.925	20	214	264	477
59.624	20	TTTCCCTCGATTCTCGTGTC	60.195	20	270	903	1172
60.042	20	GTGCTGCTTTTCTTCGGAGT	59.621	20	256	84	339
59.933	20	TCTAACTTGACCTCACCGCC	60.255	20	260	507	766
59.784	20	GATATTAAACGAGCGGTGGC	59.574	20	277	704	980
60.353	20	AAAATGCCCTTGTATGCTGC	60.103	20	224	457	680
59.989	20	CAAATTCCAATGAAAGGGGA	59.733	20	205	143	347
59.805	20	ATTTACGTCGATCTGCGTCC	60.103	20	168	64	231
59.923	20	GCACCGTGTGCAGTTATTTG	60.18	20	156	284	439
59.95	20	ACCAGCTGCACCTGTAACAA	59.365	20	170	3368	3537
59.914	20	TTCAAAGAGATGGGTCAGGG	60.042	20	168	1356	1523
59.779	20	GGGGTTCGTGCTTGTATTTG	60.365	20	241	404	644
59.829	20	AAATCGGTTAAAACCGGACC	60.055	20	179	71	249
60.147	20	GGCGCATCTGATCTTAAAAA	57.99	20	142	17	158
60.05	20	ACGGCTGTGGTTTCAAATTC	59.978	20	254	376	629
60.035	20	ACCATCGTAGGGTGTATGCC	59.7	20	218	34	251
60.074	20	GGAGAAGAACATGGATCGGA	60.011	20	127	86	212
60.194	20	ATATCAAAGGCGTGGATTGC	59.929	20	154	721	874
60.184	20	GGTAGCCATTTGAGTCGCAT	60.103	20	167	360	526
60.627	19	ACTTCATCCCATTGAGTCGG	59.927	20	103	2	104
60.627	19	CATCAAGAGCCTCCTCGAAC	59.95	20	125	2	126
60.074	20	CCGGAGCCTGTCAACTAGAG	60.005	20	256	3040	3295
59.997	20	TTGCTCTAACAATTCTGAAACGA	59.065	23	183	400	582
59.962	20	CGCCAGGGGAATTGTATTTA	59.789	20	216	1170	1385
58.623	20	GCAGCCATGGTATGGACTCT	60.104	20	279	548	826
59.63	21	TTATGTGGTTTGGTCCGGTT	60.088	20	143	21	163
60.206	20	GTGGATTTGGGGATGATGAC	59.995	20	257	362	618
59.59	20	CGTGGAGTCGACGACAATTA	59.716	20	198	1003	1200
60.185	20	CGCACAGATTGCTTGTGATT	59.871	20	242	597	838
60.072	20	GCCAAATGCCTACAAATTGC	60.465	20	237	1191	1427
59.688	20	TTGGCTTGTCGAGCTCTTTT	60.132	20	261	6193	6453
60.048	20	CCGAATGCTATCTTGGGGTA	59.916	20	248	323	570
59.664	20	TTTTGGCTCGTTGAGCTTTT	59.996	20	243	2350	2592
59.856	21	TGCAAAGCATACTAAAGATGCAC	59.463	23	185	385	569
59.966	20	ATTGTCTCATGGCCCAACAT	60.203	20	154	651	804
60.347	20	TTTGACGACCCATTCAAGGT	60.353	20	258	1480	1737
59.797	20	CGGTCTCCTTTGTCCAACTC	59.697	20	158	293	450

60.431	20	GAATAGTTGCGGATGCACG	60.235	19	177	156	332
59.973	20	CAAGCCCTAGTTCTTTCCCC	60.068	20	239	73	311
60.431	20	CGGTGGAAGGAGTGAGAGAG	59.978	20	103	4	106
59.931	20	AATCCGACACGAACATGACA	59.967	20	256	1659	1914
59.184	21	CACTACCAAGGGGTCTGCAT	59.989	20	131	340	470
59.976	20	TGGGGTTGAGCCCAATAATA	60.146	20	267	81	347
60.226	20	CCGGATATACAAACTACAGGGC	59.758	22	224	102	325
59.939	20	GGGATGAATTTCCACCATGA	60.526	20	183	564	746
57.16	27	TACATGATGGAACGGGGATT	60.014	20	155	2	156
59.993	20	GCATCTCTCTCTTGCTCTCATTG	60.665	23	110	370	479
60.762	20	ATCGGTTATTACCGGATCGC	61.031	20	199	198	396
59.967	20	TTCTTGGTGGCGGTAGGTAG	60.125	20	180	543	722
60.399	20	CAGCCGTCGGATTTAACATT	59.96	20	167	2364	2530
60.152	20	AATGCTTGCCATGAGAATCC	60.043	20	280	767	1046
59.784	21	TTAACCGTCCACATTGCAGA	60.111	20	166	435	600
59.971	20	CAGTTGGTTTTGCATCATCG	60.111	20	141	4573	4713
59.973	20	ACTCGGGCAGGAAAAGAAAT	60.074	20	184	869	1052
60.301	20	TTGAGGGATGACGGGAGTAG	60.065	20	263	39	301
60.637	20	AGGATCAATCCCCACAAAAA	59.22	20	271	159	429
59.028	20	GCGTGTGAGTGTTCGTGAGT	59.948	20	273	48	320
60.149	20	CTTTCTTCTTCATTTCGCCG	59.953	20	134	27	160
59.797	20	TTATCGATCTCCTGCATCACC	60.051	21	246	114	359
59.855	20	CATCACAGAGAATCAAACCAACA	60.023	23	120	325	444
60.045	20	AATGGACCAGCCACTACAGG	59.989	20	279	1375	1653
59.962	20	AACATCATCCAAATCCCAGC	59.756	20	202	644	845
59.966	20	ACTGTCCCTACCTGCCCTTT	59.994	20	256	1424	1679
57.885	21	TGAACCACTCCTCGCTCTCT	60.135	20	241	37	277
60.096	20	CCTTTTTGGGTGCGTATCAT	59.823	20	238	3169	3406
59.82	20	TTTAACAGCCATAAGCACAAGC	59.45	22	160	1713	1872
59.95	20	AGAGTCAAACATGCGATCCTT	58.806	21	190	381	570
59.96	20	GCCAATGGATGAACAGTCCT	59.934	20	191	693	883
60.054	26	ATGCCAATGGCCCAATAAT	59.994	19	244	5	248
59.827	20	GCAAGTGGTCACGTGAAATG	60.16	20	235	406	640
59.987	20	CCCTAATTTGTGAAAATGGGTT	59.141	22	274	69	342
59.964	20	GGTTCTCTGGTTGCACCATT	59.973	20	276	914	1189
59.955	20	CAAAACCCGTAAAGCGAAAA	60.102	20	231	861	1091
57.023	20	GTTCGAATCCTCTCTTCCCC	60.015	20	279	94	372
59.507	23	GTAGCCTTTCATTCCCTCCC	59.903	20	124	475	598
60.149	25	TAAAGCAATTTTGCCCAAGG	60.068	20	171	269	439
59.973	20	CTTGTCTCCATTCCATTGGC	60.461	20	231	85	315
60.001	20	AGGCTATAAAGGAGCGAGAGTT	57.926	22	260	929	1188
59.685	25	ACATATTTATACGCATATAGCAAA	57.577	27	197	1885	2081
60.223	20	TGACCCTATGAGGCCAAGTC	60.073	20	259	838	1096
59.691	20	CCGTGATGCCTGTGAAGTAA	59.716	20	254	2236	2489
59.694	21	TGCGAGTGTTAACCGTTTCT	58.432	20	260	0	259
59.939	20	CGACAATTTTAGAGACAACGTGA	59.322	23	212	2508	2719
59.803	20	CGAGGGAAGGAAGGTTTAGG	60.061	20	125	937	1061
58.912	21	TGTAGCCCGGTGCTAGAGTT	59.898	20	145	21	165
60.111	20	ACGTTGGACCTGAAAGAGGA	59.697	20	275	100	374
60.401	20	AAGGATGCCCAAAGTGTACG	59.993	20	235	8	242
60.243	20	CTTGTGAAATCAATGCACCG	60.111	20	202	98	299
59.854	20	GAGTTCGTCAAACCTCCCAA	60.088	20	184	259	442
59.883	20	TAGCCCATGAACACACCAAA	59.964	20	241	513	753
60.131	20	AGGCACGAACCTCTTTCTCA	59.989	20	145	8	152
59.118	22	AGCGGTTAAAGGTGGTGCTA	59.769	20	249	103	351
59.893	20	TTGGTTGGGTAGCTTTGGAG	60.103	20	278	404	681
58.013	21	ACAATCGTCGGATTTTAGCG	60.096	20	179	0	178
60.489	20	CGGATGGACCGGATGTATTA	60.544	20	250	13	262

58.882	20	CTCGGTATGCGAGAGAGAGG	60.111	20	209	57	265
57.552	24	TACGGCTGCGTAAACTACCC	60.152	20	271	16	286
60.132	20	CCCTAGCCTTTGCCTCTTTC	60.332	20	212	190	401
59.585	20	GGCTTGTCTGGAGTTTGCTC	59.997	20	277	65	341
60.011	20	TTAATGCGCGTACCAAATCA	60.096	20	274	925	1198
58.514	21	CACATGGAATTGCCTGAAGA	59.648	20	277	418	694
60.18	20	CCATGAACACATTGATTGGC	59.781	20	252	151	402
59.904	20	CACCCGAAAACCGAATAAGA	59.931	20	125	394	518
58.762	26	ACGGCCTGAATGACAGAAAT	59.556	20	280	10	289
59.871	20	CGGAGTGGGCTCATAAATGT	59.955	20	275	136	410
59.982	20	CATCCCAAGCCATACGAACT	59.955	20	250	56	305
60.089	20	CCTTTGTTCCTTTGGCACAT	59.971	20	242	1250	1491
60.203	20	ACACGGTCTCATGGCATAAA	59.002	20	254	425	678
60.074	20	GTTTCCTCCACAACGCTCTC	59.851	20	201	1936	2136
59.595	20	GCAATAACAAAACTCGGGTGA	59.989	21	253	63	315
60.088	20	TTCCTACTGGCTGTCCTTCG	60.388	20	227	962	1188
60.206	20	ATCCATGCATTCCCAACAAT	60.021	20	181	273	453
59.457	22	CAATAAGAAACACGCAACACAAA	60.082	23	200	221	420
59.25	20	GGGTCCAGACTTGAGACCAA	60.088	20	197	700	896
59.986	20	TGTGCGCTTGAGTGTGTGTA	60.096	20	164	676	839
59.697	20	TGACGTTCTTGCTGCTATGG	60.011	20	139	1633	1771
59.953	20	CACATGTCACGAATCGGTGT	60.45	20	274	108	381
60.118	20	TAAACAACCATGTCGGCGTA	59.992	20	279	235	513
59.001	21	GATCGACCAATTGTGTGACG	59.967	20	171	1003	1173
61.052	20	ACGTCCATGGGAGAATGTGT	60.246	20	228	68	295
59.381	20	AGGGCCACATGATGATAACC	59.635	20	252	61	312
59.888	20	TGAGCCTAAGGGGTGAAGTG	60.246	20	229	5579	5807
60.018	20	ATTTCTCTCGTCACACGCCT	59.874	20	205	949	1153
60.129	20	TCTTCGAATAAACCCCCACA	60.301	20	275	341	615
60.111	20	TGGAGGAAGAAGACAAGATGC	59.42	21	185	53	237
60.111	20	GGAGTGGCTTGCGAACTTAG	60.015	20	214	21	234
60.293	20	GTGCGTGTTTGTCGTGATTT	59.621	20	271	119	389
59.997	20	TAGATCACATTTGGCACGGA	60.073	20	164	265	428
59.238	21	GTCTTTGCGGGTGAAAAGTT	59.218	20	245	25	269
60.459	20	TCCAAACCAACAACACCTGA	59.976	20	136	107	242
60.096	20	TCCGTTATGGGCCGTTAATA	60.165	20	202	2707	2908
60.008	20	AAGGGACATTTTCTGCTTGC	59.316	20	215	1785	1999
60.015	20	ACATCTCCCTCACCCTCTCC	60.469	20	248	1167	1414
59.972	20	CTGAAACCACACCTCCACCT	60.002	20	272	722	993
59.966	20	GATACTCCGGCCAACTGAAA	60.074	20	280	1301	1580
58.866	20	TTGAACCTAGCCGGAGAAGA	59.948	20	237	32	268
60.016	20	GCTTGCCACGTCAACATAAA	59.735	20	136	2535	2670
59.853	20	TGACCGTTTCACCACAGGTA	60.001	20	234	14	247
59.659	21	AGATGTAGCAACCTATTTCTAATT	57.349	27	170	107	276
60.397	20	CTTGGACCAGATTGTCCCTC	59.505	20	233	346	578
59.907	20	GCATAGAAAAATATGTAAAATTCA	58.929	27	178	356	533
57.795	21	ATCCATCCTACACAACCGGA	60.195	20	212	19	230
60.066	20	GGCACGATCACTTTAACCGT	60	20	170	1229	1398
59.803	21	TCCCAATTTAAGGGTTATTACCTG	59.555	24	145	1018	1162
59.893	20	TAATTGCATTCTCCCGAACC	59.901	20	271	268	538
59.836	20	AACCAGCTGTCAGAAGTGGG	60.298	20	266	1043	1308
59.897	20	CTTCTTGTCTGCAACGTGGA	60.025	20	140	668	807
60.184	20	GGCCTGTAGACCAATACCCA	59.813	20	107	42	148
59.67	20	TGACTGCCACAGTTTCCTCA	60.44	20	223	1545	1767
59.284	20	TGTGACCACTTGTCTACGGG	59.591	20	209	319	527
60.088	20	AAAGAGACTGAGCCACCGAA	59.989	20	251	346	596
59.989	20	AAGGACCAATGAGATGGGTG	59.779	20	248	66	313
60.018	20	TGAAGGTGACGAGAATGGTG	59.676	20	118	699	816

60.331	20	GGTGTGTGAGGTGTGTGAGG	60.045	20	103	40	142
59.973	20	TGCTCTCTCATTGTAATCTCTCTCA	59.688	25	251	109	359
59.673	20	CACACTGGACATCCCTTGAA	59.52	20	260	1698	1957
59.844	20	TGCTGCTGAACTTCTTGTGAA	59.773	21	203	4779	4981
61.188	20	TGCATATCTTCGTGTGTGCAT	60.158	21	242	133	374
59.381	20	CACCACTGCTTCTGCTTTGA	60.175	20	160	303	462
59.931	20	GCCGAGATTTGTTATGCGTT	60.103	20	169	329	497
60.331	20	CAAGATCCAAGGGCTGTGATA	60.081	21	190	705	894
59.96	24	CGATCCGGTCTGATCCAA	60.582	18	268	352	619
59.96	20	GCAGCCCATTCTATGCAAAT	60.067	20	209	1076	1284
59.924	20	ACGAAAAAGGGCATGAAAAA	59.564	20	280	381	660
60.158	20	TTCACACGAGAGTGCAGATTG	60.04	21	199	534	732
58.53	22	GCCAGAAATAGTGGATTTTGC	58.709	21	240	2170	2409
60.067	20	TTTAAGGGTTATCACCTGATTTTC	57.757	24	184	607	790
58.958	20	TTTTTAAAAGGAGACATTGCTGTT	58.494	24	227	246	472
59.901	20	TGTTGCAAGTAGTAAAGTAAAGG(	59.475	25	182	528	709
59.847	20	AGAAGGTTATCACCGGATCG	59.006	20	250	1159	1408
59.901	20	AACCGCTGTTATCCCTCATT	58.53	20	152	674	825
59.934	20	GATGATTGGTTTCCACCACC	60.034	20	171	360	530
59.7	20	AACGCATTCTCCATGCTCTC	60.37	20	131	56	186
59.041	20	TTTTGGACAAAAATGCCCTT	59.429	20	251	43	293
60.05	20	GGCTGGGATGTCATTCATTT	59.756	20	263	150	412
59.472	20	GGTGTGGAAAAGGAACAACC	59.284	20	185	360	544
59.824	20	TCTTCTGTCTGTCCCTGGCT	59.986	20	265	347	611
60.051	20	ACAGCTTCACCGGTTTGTTC	60.156	20	231	1408	1638
59.591	20	ATGGCTGTTTTCCAATGAGC	60.081	20	167	528	694
59.938	20	GAGATTCGTGGTGAAAACCG	60.495	20	247	109	355
59.429	22	CAAATCTTAACCGCCGATCT	59.182	20	264	1274	1537
60.159	20	TTCCGTTTTCGACATCACTG	59.691	20	142	813	954
59.829	20	CAACAGTTTGGCATCAGAGC	59.445	20	143	97	239
60.278	20	GCCAACCACACAGTGAACAA	60.616	20	218	582	799
58.627	20	GTACAGTGGTGGTGTGCTCG	60.227	20	200	406	605
60.39	20	GGCTAATTGTTGGCCTTTGA	60.074	20	238	10	247
60.305	20	GGAGAATTCCAATGCCAGAA	60.014	20	171	307	477
59.938	20	TTCGTGGATGCTATGCTCAC	59.83	20	164	18	181
60.414	20	GCACAACTAATGCTTCCTGCT	59.536	21	250	150	399
60.032	20	ATTACATTGTTTCTGCCGCC	59.967	20	246	346	591
59.938	20	AGGTGATTGTGGTGGGTCC	60.649	19	155	35	189
59.297	20	GCCAATGCCTCATCCTAAAG	59.668	20	163	336	498
59.485	23	AAAAGTTTGGCGTCATCACC	59.978	20	213	2420	2632
60.278	20	TCGAGCTGGCTGAAGAATTT	60.096	20	212	568	779
60.195	20	ACGTAGGATATCACTAAATTGATT	57.146	27	279	1167	1445
58.055	22	GTGCAGCCTTATCTCTTGCC	59.985	20	207	93	299
59.967	21	TGGGTAGCATATGTCGATGG	59.375	20	161	68	228
60.257	20	GCGTTTTGATGATATATAGGTGTT	58.907	26	195	546	740
59.923	20	CGGGAGGGATGACTTTGTAA	59.926	20	233	971	1203
60.11	22	CCCTTGAAGAACTCATCCCTT	59.562	21	225	2837	3061
59.113	25	GTGGCAGAGGTTGGGTAGAA	60.111	20	216	18	233
60.312	20	CAGGGCTATCCCTCATCAGA	60.173	20	271	215	485
60	20	CATCGCTGCAAGATGGAATA	59.792	20	223	69	291
59.972	20	AGTTTTGTGGTCGAAGGTGC	60.156	20	202	151	352
59.813	20	CTCATTGACAAACCATGTCCA	59.405	21	276	122	397
59.96	20	AAACCCGTGTTCTTCAGTCG	60.149	20	256	19	274
59.504	20	GAGTGGGCATTATGGAGCAT	59.923	20	131	744	874
59.93	20	GACGAATCAAAGGTGCCAAT	59.939	20	173	73	245
59.739	20	GGGAAAGCCGATCAATTTTT	60.26	20	111	197	307
60.183	20	ATCGGTTATTATCGGATCGC	58.88	20	248	1258	1505
60.127	21	AAGGGTTCTGCTGAGAACGA	59.989	20	238	53	290

60.135	20	GTTAGCATGGGAAGGGAAGG	60.817	20	262	367	628
59.948	20	TAGGAATCACACGTGGTGGG	61.777	20	253	157	409
60.221	20	CAAACCCCGAGTCTGATGTT	59.966	20	202	1832	2033
59.127	20	AAGCCTTGTACCGAGCTTGA	60.015	20	243	1275	1517
59.95	20	ACCAATCTCGCATAGAACCG	60.096	20	274	1297	1570
59.656	20	CACAAATGGAGGTGATGTGG	59.806	20	251	317	567
58.955	22	ACTCAGGATGCCCACTGTTC	60.12	20	162	0	161
59.359	21	CCATCGTGAAGAACAGACCA	59.676	20	224	276	499
59.964	20	TTGACATGCCATTTTCTTGC	59.67	20	280	1028	1307
59.82	22	CCCCACTTCACCACTTCACT	60.002	20	219	142	360
59.862	20	AACAACCCTCATTGCTCTCG	60.255	20	278	121	398
60.157	20	CCCGGTAAGAAACCCATTTT	60.048	20	119	259	377
59.079	26	TGGGAAGAAAGGACAGGATG	60.042	20	188	4	191
59.09	20	TCGAGATGAAAACATTGGCA	60.197	20	218	113	330
59.411	20	CTTCACCTCCCAGATTTCCA	60.042	20	171	13	183
59.735	20	ATCCATGGTGAGGTGACTGG	60.806	20	145	390	534
59.707	21	TGCAGCTGCTCTAATCATGG	60.119	20	258	1254	1511
59.75	20	GGGGAGTAAAACGACCGAAT	60.187	20	231	74	304
60.067	20	AAGAACCGTGGTTTTTGTCG	60.008	20	251	298	548
59.146	20	TCCAAAATGAGACCAATCTTGA	59.546	22	248	164	411
60.298	20	CCGCGGCCTATCTTCTTAG	59.946	19	261	1972	2232
60.483	20	CTGTGTCCGGTTAAGTCCGT	60.028	20	213	1086	1298
59.866	20	ATGATTTTTGCAGCGAAACC	60.081	20	242	517	758
57.204	20	CAACAATGCCTTTGGTCTCA	59.691	20	152	328	479
60.49	20	GCTTGGGAGTCGAGGTACAA	60.255	20	264	963	1226
59.986	20	AGAGCGAGGTGAGAAGAGGG	61.061	20	260	113	372
59.826	20	CATCCTGATCCACACAGTGC	60.121	20	223	727	949
59.923	20	CCAACTGCAGAGTAACGCAG	59.658	20	232	2267	2498
60.067	20	GAAGTTAAAATAACGTACGTGTTG	58.397	25	250	1080	1329
59.572	20	GGTTCGTGTCGTTTTCAGGT	60.012	20	189	165	353
60.051	20	ATGCCATCACAAGGAAGAGC	60.226	20	118	2103	2220
60.073	20	CCCGAATATTCTGTGCCAGT	59.955	20	138	2664	2801
59.212	20	TTGTCAGGAGAGGCGTTCTT	59.989	20	185	2496	2680
60.14	20	CCGCTGATGAAATGATCTGA	59.756	20	166	352	517
60.199	20	CTGGAGCCATTTTTGTGGAT	59.933	20	251	23	273
60.232	20	TTACCATTCGCGTGGACATA	59.953	20	104	421	524
59.973	20	GATTACTCCACCCTCACCGA	59.927	20	277	381	657
59.989	20	CCACCCTCAAACAACACTCA	59.565	20	211	16	226
60.009	20	TTACGTCCCGATCCCAATAA	60.146	20	202	2698	2899
59.847	20	ACCTTTTCACAGCAGAGGGA	59.844	20	233	726	958
59.801	20	TGAAGTATCGACGCATTCACA	60.272	21	238	545	782
60.006	21	AAACATTTTCAAAAACGCCAA	59.502	21	198	70	267
59.888	19	CACCACCACCGTTAAATTCC	60.088	20	224	49	272
59.975	20	AAATAGCTCCGATAACCGGG	60.292	20	198	165	362
59.772	21	GGGTTGATAGATCGGGTTGA	59.75	20	168	765	932
60.053	20	GCGTGCTGAATCTTATCCGT	60.243	20	251	204	454
58.986	23	GAGCATGATTTGGCTGGACT	60.226	20	261	78	338
57.484	20	GCAAATGCTTCTTACCCATCA	60.089	21	261	60	320
60.073	20	CCGGTAAAAACAATTCGCAC	60.356	20	135	117	251
60.395	22	AGAGAGAGAGTGTGGAGAGAGA(	59.473	25	100	464	563
59.976	20	TTATACCACCGCCACCCTTA	60.202	20	144	1012	1155
60.657	20	CACTTTACGACCATCCCCAG	60.366	20	270	81	350
61.448	23	TTCTTGATGTGTTGGCAAGC	59.847	20	142	19	160
59.813	20	AAATGTTCGTGCGAATGTGA	60.119	20	136	440	575
59.986	20	TCATTCAAAAATTGATCACCCA	60.167	22	147	14	160
59.927	20	TAGATGGGAAATCCGACACC	59.75	20	250	2026	2275
59.836	20	AAACGATGTATGGGATTGATGA	59.162	22	222	153	374
58.32	21	AATCGTGCTGAAACGTGTTG	59.764	20	276	21	296

60.154	20 AAGGCCAAATGAGTCTGAGC	59.434	20	278	176	453
60.159	20 TCCATCTGCAAAATCCATCA	60.009	20	280	119	398
60.416	20 ACCCTGTGTATCCCAGCAAA	60.375	20	221	6	226
59.41	20 AAAAACACCAACACTTCTTGCAT	59.961	23	273	2325	2597
59.478	20 AGTTCCGGGGTCCATTCTAC	60.19	20	270	33	302
60.636	20 AGCGGAGGAGGAGGAAGTAG	59.972	20	246	43	288
59.997	20 ACTGCATCACTCTCAAGCCC	60.418	20	121	1119	1239
60.241	20 AAAACTCCGGTGTTCCATTG	59.83	20	116	374	489
58.111	21 AGACTATCAACAAGCCCCGA	59.694	20	227	3	229
60.009	20 GCCACCTGATCTAGCGATGT	60.249	20	156	2262	2417
60.483	20 CCAGGTGTTATGACCCGAAC	60.232	20	213	296	508
60.05	20 CACCACCATCACCCTCTTCT	59.962	20	203	96	298
59.943	20 TGCCACTTAGAAAAGTAAAAACG[illegible]	60.101	25	243	315	557
59.572	20 AGGTGAATCGTCAAACGTCA	59.139	20	134	299	432
59.33	20 CCGTACCGGAAATTAACGAA	59.826	20	247	249	495
60.027	20 GAAATTCTGCAGTTCGGAGC	59.962	20	193	144	336
59.996	20 TTTGATTTTGGGGTCAGCTC	60.051	20	260	216	475
60.652	24 TAGTAGGGTGCAGGGGATTG	59.948	20	225	2391	2615
60.472	19 TAAGCCGCTTTGCCTAGAAA	60.109	20	280	1373	1652
58.974	20 GCTCCTCTTTCCCTTTTGCT	59.962	20	260	456	715
59.925	20 AGTCACAGGATCCAAGAGCC	59.258	20	219	232	450
60.133	20 CATTCCCAACTCCCAAAATTA	58.794	21	240	424	663
60.188	20 GGTGCTGCAACTCAACTGAA	60.032	20	216	869	1084
60.008	20 ATGAAGCATGCAATTCGACA	60.228	20	239	56	294
60.152	20 TCCACCCGAGCTTTAGTTTTT	60.116	21	178	1997	2174
60.011	20 CATCTGGTGGTCCAACGAC	59.945	19	178	2000	2177
60.21	20 TTTTGATGTTCGAAAATACCCC	60.056	22	152	102	253
59.903	20 ACATGCATGCTCGCAAATTA	60.245	20	130	34	163
60.352	20 GCAGCGTCAAATCAATAACCT	59.242	21	280	266	545
60.008	20 CGTGTGCAATGCATCTGGTA	61.713	20	226	69	294
59.152	20 ATGGTTCTGGCTTATGTGGC	59.962	20	273	288	560
57.613	25 ACCCAACCCAACCACAATAA	59.948	20	239	489	727
59.978	20 ATGGCCGAGATTTGTTATGC	59.929	20	108	1391	1498
60.389	22 TTGTTTGCTTGTAGATGTTGGG	60.032	22	183	799	981
59.551	20 CATTTGGCCGACTTTAGAGC	59.845	20	209	2649	2857
60.136	20 TGGCATGGGGAAGCTACTAT	59.551	20	230	80	309
59.955	20 TTCTCCCCAAAAGCTAATCG	59.292	20	277	148	424
58.646	22 GACGAAGAAAATCAACCGGA	60.051	20	159	4	162
60.532	19 AACTTTGAATCTGGCAACGTG	60.161	21	280	161	440
59.943	20 TTGTGTAATTGTGTTGTGTCGTG	59.468	23	237	101	337
59.933	20 AGGAGCTTTCTGCGTGGATA	59.978	20	118	2	119
59.815	21 TTGTTCTCTCGAAGCCCCTA	59.948	20	269	1823	2091
60.827	20 TCAGTTTTTGAGTTATGAATTTGC	57.598	24	188	244	431
60.138	20 GCTGGTGTGAGTTGTACGCA	60.939	20	247	233	479
60.096	20 CCAAATTTAAGGGTTATCACCTG	58.849	23	271	3642	3912
59.817	20 TTTCGACCGACAAAAAGGAG	60.22	20	107	717	823
60.23	20 CCAAAAGAAAATCTCCTGCG	59.817	20	200	702	901
60.111	20 ATAATTGATGGTTGTGCGGC	60.731	20	212	112	323
60.235	19 AAAAATGCAACCAGCATTAAAA	58.72	22	270	492	761
59.795	20 TACCCATTTGAAAATCCCCA	59.988	20	144	160	303
58.484	25 TCATGAGGAGCGTCAATCAG	59.942	20	141	9	149
60.234	20 AGTGCTGCCTCAAAGGGTAA	59.875	20	172	92	263
59.986	20 CGATCCTGTGTGGCCTAAAT	59.955	20	236	96	331
60.05	20 TTCCGTTAGGGGATACGATG	59.778	20	246	989	1234
59.816	20 GACACTTAGAGGGTGTTTTGACC	59.068	23	211	914	1124
59.762	20 TTCCAGATCCAAAATATCCACC	60.02	22	269	249	517
59.578	19 TGGATCATGTAAGGAACAATGTG	59.738	23	150	15	164
59.009	20 TTCAAGGCATTTGAGATCCAC	60.066	21	196	2356	2551

59.801	20	AAGAAGAAAAGACATTCCGGC	59.717	21	121	275	395
60.46	20	TAGGGTCAACCATCAGTCCC	59.779	20	186	250	435
59.958	20	GGTGGATTGTCCTTGATGCT	59.934	20	139	867	1005
59.596	20	GAAGATCCCGGTGAAATGAA	59.871	20	153	15	167
59.7	20	TTAAACCCTAAAAGCGCACG	60.251	20	257	228	484
59.978	20	CCCCCTAATCCCTAAATCCC	60.678	20	213	967	1179
60.025	20	CCATCTCCAACTTCATGCCT	60.073	20	252	0	251
60.118	20	ACGCAACCGTAAAGAACCTG	60.168	20	279	304	582
59.875	20	CCCGATCACCATCACTATCC	60.158	20	270	129	398
59.166	20	TGGTATCAGAGCCATGACGA	60.225	20	276	8	283
60.378	20	GCACGAAGAAGGTTAAAAGGG	60.116	21	252	651	902
60.134	20	TATCGTGCGCAATAGACGAG	59.999	20	178	1179	1356
59.483	20	CTCGATAGGAAAGGATGCAA	57.916	20	119	836	954
59.04	20	CAATCTTTGGGCGTGAGAGT	60.255	20	140	5	144
59.984	20	GTGCGTTGACCAGCATTTA	58.274	19	125	275	399
59.639	19	AAGGACGTAACGGCTCTCAA	59.875	20	253	8	260
61.078	20	TTTTGGAGATCTATGGCTGTCA	59.708	22	164	254	417
60.388	20	TAAGAATTCCAATGGGGCAG	59.894	20	280	127	406
59.585	20	AAAGTGGGAGTTAGGGCACA	59.592	20	219	62	280
59.809	20	TAGGTGTGTGCATCTTGGGA	60.112	20	217	513	729
60.132	20	GTGTTTGCATGGTGAACTGC	60.168	20	242	1485	1726
59.242	23	CGGATTTAATCGTGGCTGTT	59.96	20	140	31	170
57.296	27	CCAAATCAGACTGCGAACAA	59.84	20	246	1780	2025
59.999	20	CGGTTATTAGTTATCACCCGAAA	59.322	23	125	2896	3020
59.797	20	AGGAGGATTCACCACGTCAC	59.969	20	173	224	396
60.18	20	CAAACCATCCAACCACAACA	60.255	20	261	11	271
60.366	20	TCCTTCAATCTCAGCCTTTCA	59.94	21	250	963	1212
60.61	20	TTTCCCTACAATTTCGGCTG	60.067	20	195	145	339
59.827	20	CATATGGGCACAGACACTGG	59.984	20	153	21	173
59.827	20	CATATGGGCACAGACACTGG	59.984	20	153	21	173
60.066	20	AAAGGAGCTTCTTGCACTGG	59.615	20	261	1632	1892
60.235	20	TCCCAGAATATCGAAGGCAC	60.036	20	165	322	486
59.864	20	AGGGTTTTGAGGGTTTGCTT	59.976	20	253	438	690
60.051	20	AATCCCAACAAATGACCCAA	60.029	20	175	1155	1329
59.888	20	ATGCAGACGAATAAGCGAGG	60.374	20	218	119	336
59.691	20	CACCATCATCTTCATCCACG	59.918	20	258	85	342
59.928	20	ACCCACATAGTGCCGAGAGA	60.678	20	121	633	753
59.464	26	GAAAGCTAAAATTTGCAATCAAA(	58.619	24	117	12	128
60.206	20	TCCTTGCATGCACATCATTT	60.08	20	263	34	296
59.685	20	GACAAAGCAGGCACACAACT	58.935	20	201	150	350
59.903	20	TTGAACGGAATCATGGACAA	59.9	20	167	79	245
59.055	24	TTTGTCTACCAGTTGCCCCT	59.592	20	234	798	1031
60.073	20	GGCATGATTCTGCTGGTTTT	60.081	20	278	1281	1558
59.215	20	GATTTGACCATGAGCGTTGA	59.654	20	157	157	313
59.9	25	AAATGGTCCCCTCATGAACA	60.173	20	139	8	146
60.013	20	CAACCAGCAAACAAAAGCAA	59.888	20	136	122	257
57.864	25	ATTTTAGGAAGGATCGGGGC	61.454	20	231	171	401
59.157	20	AAGCCTTCCATTCCGAAAAT	59.909	20	161	742	902
60.204	20	TTTTACACGGGACCGTCAAT	60.227	20	267	486	752
59.891	20	AGATGTTGGATCTGCAGCCT	59.834	20	253	591	843
60.015	20	AATCCTCCTGCTGTGGTGAC	60.12	20	230	4235	4464
59.276	20	CCGCAAGTGTACGGGACTAT	60.015	20	218	286	503
60.119	20	TCTTCTTCCACTCGCATCGT	60.945	20	237	864	1100
59.115	21	AAGCGCACGTTTGAGTTTTT	59.926	20	153	73	225
59.853	20	CAAATTGTTTGGGCTCGATT	59.938	20	226	340	565
57.656	19	CCAATGATGCATGGTTATGG	59.622	20	254	177	430
59.444	20	TTCAGCCATGGCAAATACTG	59.688	20	276	364	639
59.83	20	GCAGGTAAATATCAATTTTCGAG	57.112	23	272	211	482

59.875	20	TGCCAGCAGAGAGGAGGTAT	59.973	20	164	17	180
59.748	21	TACGCACGATGTTGGTGAAT	59.995	20	174	117	290
59.967	20	AATTAACATCACGGCCATCC	59.651	20	243	105	347
60.619	20	CCGTTGGAATTTTAGAGTTACG	57.9	22	111	578	688
59.979	20	GTGGGCTTCGACTCTCAGAC	59.993	20	184	566	749
59.993	20	CCAAAATCAACGTCCCAGAT	59.79	20	279	786	1064
60.039	20	GTGCTGAGGCGTTTCTTGAC	60.986	20	251	37	287
59.533	25	TGTTCGGATTTGGCGATTAT	60.289	20	276	219	494
60.074	20	TCCCTTAATTTCCTCACCCC	60.124	20	172	292	463
59.989	20	TGGACTCCGTGTGTATGACTG	59.606	21	203	478	680
57.849	20	CGCACCTTTCATGTGCAATA	60.665	20	280	1121	1400
60.096	20	ACATGATTTGCAAGATGCCA	60.08	20	249	519	767
58.532	23	CCGGCTCTGATACCATTGTT	59.955	20	270	687	956
59.713	20	TGGTCTTACATCTCCCAGGC	60.073	20	192	1510	1701
59.984	20	AACACGGTAACAAAATATATTGA/	58.491	27	163	199	361
59.992	20	TCGGTTTCACCAATGGAAAT	60.17	20	110	304	413
59.733	21	GATCTCTTTTGTCAAGGGCG	59.813	20	119	4	122
58.315	21	CCCAAAATAGCTTTCCCACA	59.931	20	224	48	271
59.862	20	GGATTGTGCACTCAATGAATCT	59.059	22	256	198	453
60.195	20	TTCCTCCTTTCCTCTTATGGG	59.536	21	270	139	408
60.133	20	TCCCCGAATATTCCAACTCA	60.266	20	163	163	325
59.989	20	TTGGTGAAAGGAGACGAAGG	60.224	20	234	94	327
60.045	20	TGAGTGAACATGTGTGTGCG	60.373	20	159	250	408
60.401	20	ATCCAACTCCTGCATTTTGC	60.081	20	270	13	282
57.141	23	CGGTTAAAAATCGGACAGGA	59.931	20	260	1836	2095
60.671	20	TACGGCACCATATAATCGGG	60.548	20	109	86	194
60.012	20	AAAGTGGCCGACAAAAACC	59.963	19	275	559	833
59.803	20	GGATCGTTGATCGGGTTAGA	59.894	20	133	40	172
59.816	20	GATCTTTTCACTCGAGCCCA	60.34	20	252	93	344
59.799	20	CGACTGGCAAATACAAAAGTT	57.007	21	203	426	628
60.096	20	ATGGCTCCATTCTTCCTCCT	60.037	20	242	162	403
59.831	20	GGAATTGAAATCCCACCTGA	59.727	20	256	389	644
59.807	20	TATTTTCGGCCACCACTCTC	60.074	20	163	219	381
59.859	20	TCTCTTTCTCCCAATGAACCA	59.656	21	229	1614	1842
59.96	20	ACTCGTAACATCCCCATCCA	60.195	20	269	741	1009
58.775	22	AATTGTTGAAACCGGTCCAG	59.83	20	200	316	515
59.939	20	AACACCACAATTGGCCTCTC	59.973	20	130	230	359
59.894	20	AGCCTGCATTTGACTTTTGG	60.249	20	228	580	807
59.901	20	AGGAAGGGCGAAAAAGAGAG	59.955	20	273	31	303
59.599	20	GATGTGGAAAAGCCCAGAAA	60.051	20	272	1638	1909
59.986	20	TCCTTTCCGCTGAGCTTCTA	60.227	20	253	281	533
60.153	21	CCACCAAGCAGTGTTTATAGTAGC	59.308	24	217	155	371
60.011	20	TATCCCCTCATGCATTCCTT	59.344	20	200	1690	1889
59.387	20	TGGACCATTTGTCCTTCAAA	58.947	20	226	44	269
59.855	20	TGGTCAATACCAGAAAAATCCA	59.315	22	220	234	453
59.541	26	CCCCTTATTTGTGGTTCCAA	59.657	20	259	22	280
60.035	20	ATTACTGCGGCTGATGCTCT	60.007	20	254	56	309
58.12	20	AAGCCCTAAATGCACTCGAA	59.845	20	280	107	386
59.872	20	TCCACGAATTTGACACGAAA	60.088	20	162	1462	1623
58.471	27	TGAATTTTGTTTCCCACCAA	58.841	20	126	8	133
59.978	22	TTTGTTTCAAAGAATTTGTGGTAG/	59.157	25	169	698	866
59.682	20	TGCTCCAGTTTCCTTTTGCT	59.993	20	116	52	167
58.765	20	CGACTTTTAATGGCGGTTGT	59.996	20	110	66	175
60.123	20	CCTAAATCATTGGGGCAGAA	59.894	20	220	3385	3604
59.721	20	TTGGGAAGGATGAAGTTTGG	59.903	20	142	1178	1319
60.11	20	GGTGCCGGTCCATAAAATAA	59.66	20	265	803	1067
60.573	26	GTGCTTGCATGAGAGATGGA	59.95	20	201	32	232
59.54	21	GTTGAAAAGAGCGCTGAACG	61.08	20	108	62	169

59.894	20	CAGCAACATCGAGTCGAGAA	60.136	20	260	1642	1901
60.215	20	TTCTACAACCACCCAAAGGG	59.824	20	251	109	359
59.878	20	GGATATCCAAAATATCCGAAAA	57.071	22	216	1066	1281
57.043	20	CGATCATATCCAATTTCGTTTC	58.449	22	149	156	304
59.973	20	CTTCATTACACCGGCAAGGT	59.993	20	277	922	1198
59.425	20	GGATCTGCGAGATAAGCGAC	59.946	20	229	471	699
60.081	20	ACCCTTGCAAATTATCGCAC	59.967	20	120	391	510
60.066	20	TTGCAATATGTCCTTGCACC	59.548	20	236	2340	2575
60.255	20	AACATGAGCGCACCAACATA	60.142	20	110	23	132
59.696	25	AAATTCTCCACTTCCCCACC	60.169	20	100	13	112
60.096	20	GGCTAATGTGTGGCAGGTTT	60	20	208	1536	1743
58.979	20	AACATCTCAAACATTCAAAGAGA(	59.724	26	243	1757	1999
59.96	20	TACTGCCTTGCATTGGATCA	60.22	20	189	35	223
60.055	20	GGACTTATTACCAATCATCCCA	57.808	22	149	729	877
60.008	20	TCTCAGCTTTCTTGATGGGAA	59.94	21	197	1403	1599
59.992	20	CCCCCAAATTTTACAAGCCT	60.177	20	147	3190	3336
60.149	20	TTGCCTTGGCCCATTTAATA	60.275	20	119	1439	1557
60.377	20	TTGTCCATTCAATCCTGTGG	59.343	20	175	9	183
60.155	20	TCAATTAACAATAGACTGACGCC	58.335	23	179	320	498
59.481	20	GGAAAGAGTGTGTGTTTGCG	59.339	20	228	400	627
60.532	20	CCGTCGTATTTTCCCAACAC	60.227	20	266	7	272
59.322	21	TTGTAGCCCCTTTGAGCCTA	59.839	20	239	9	247
60.181	20	TTTTTCTTTCCACAACCCCC	61.05	20	275	68	342
60.352	20	CCATTGATTTGGTTGATTGAA	58.321	21	106	56	161
58.215	20	GACATTGCTAGCACCGAACA	59.871	20	177	93	269
59.789	24	AAAATGTAACAACTTTTGATCCCT(	59.723	25	258	624	881
59.972	20	TTGTGATGTGGCAGCTTTTC	59.847	20	137	114	250
60.244	20	ACGCCAGATTTATCACCCAG	59.955	20	229	31	259
60.042	20	ATCGGAGTTGATTTGATGCC	59.9	20	277	134	410
59.872	20	TGGTGGGAAGGAGAGGTATG	59.92	20	154	989	1142
59.757	20	ATGCGTTTTGATGATATATAGGTG	57.255	24	100	706	805
58.341	21	TGAAATTTGGAACAAACAATGA	58.045	22	174	3	176
59.803	20	GCTATCAGCAACAAAACCCC	59.574	20	277	1011	1287
60.052	20	CACCCTAAAAATACGCCAACA	59.876	21	264	99	362
59.745	20	TCAAATGTAAAGCACTAGAGCCA	59.088	23	269	4579	4847
59.888	20	TGACATTGCCTAGAAAGTGGAA	59.747	22	279	117	395
60.022	20	TCTTTCGTGAACTGCTGTGG	60.025	20	102	1144	1245
59.757	20	GGCTTTTGTCCTCATGCACT	60.263	20	170	131	300
59.526	20	ATTCCATCCCTTGGCTCTTT	59.903	20	149	1311	1459
59.905	20	ACAGATGAGTGGCAGTCGGT	60.746	20	160	77	236
59.374	20	ATGTAATAGGGTTTGGGAAGG	57.006	21	196	823	1018
60.052	20	CCCGAAGAAGCCATCAAGTA	60.206	20	224	84	307
59.434	20	CATGACCACTCTTTCCTCCA	58.641	20	254	2352	2605
59.576	25	ATGAACTTGATGGTGGAGCC	59.934	20	276	219	494
59.806	20	GGATACAAGCAAGGGCACAT	59.962	20	104	1381	1484
60.111	20	GCTGCAGCATAAACACGGTA	59.902	20	238	137	374
60.352	20	CCCTTCACAAGTGGTGACAA	59.565	20	231	132	362
59.967	20	CGGTGTTGGATTGGTGAGTA	59.415	20	119	1123	1241
59.79	20	AAGACGAAGACGACGACGAT	59.874	20	218	538	755
59.955	20	ACTCCTTCACTCCCAGCTCA	59.986	20	203	811	1013
60.386	20	GGCAGCTCATCAAGTTCACA	59.992	20	167	10	176
59.679	20	TGATCCATGTACCCATGCTG	60.353	20	245	6	250
60.57	20	TCTTCACCAACTGTACGCCA	60.301	20	280	596	875
58.075	22	CAATTTGTTTCAGAACCCAAGA	59.108	22	246	4378	4623
59.933	20	AAATGCATGCACCTGATTACA	59.055	21	196	105	300
59.041	22	TCTTTTGCCGTAGCTTGTGA	59.609	20	243	44	286
58.117	22	CATTTCCTTTTCCACCAACG	60.336	20	215	19	233
59.328	20	CCAAGATCATATTCCGCCAT	59.745	20	190	892	1081

58.978	20	TTTTGATTTTATTGGGATTTGTTT	58.024	24	274	19	292
59.764	20	CGAGCCAAATCTTCCTTTTG	59.817	20	254	177	430
59.682	20	CGGTTATCACCGGATCGC	62.397	18	127	1345	1471
60.023	20	GGACTTTGAATTTGCTTGCC	59.691	20	266	338	603
57.557	25	ATCCAATTTGGGCCATTTTA	59.124	20	266	23	288
60.353	20	GACTTTTCCGATGGTGTCGT	59.973	20	199	100	298
58.035	19	CGTGGTTCGATCGTAATTGA	59.542	20	270	166	435
59.988	20	CTGCCTCCATTTCTTGCTTC	59.955	20	260	365	624
61.296	20	GTCTTCCTCGTAGCAGCACC	60.02	20	280	41	320
59.862	20	AGACCTTCTTTGGGTGCCTT	60.11	20	149	127	275
59.729	20	AGGGGTTATCACCTGAATGC	58.864	20	176	170	345
61.032	20	GTGGACGTCCCAGATTGTTT	59.827	20	173	9	181
59.953	20	TGTAAGCCCATCAACCTTCA	59.123	20	277	1378	1654
59.154	21	TGAGCCATGAACACCAACTC	59.682	20	207	338	544
61.031	20	TTGGGTTATCTTGTGGCTCA	59.123	20	114	21	134
60.074	20	CCACGATTCAATTATGTTCCG	60.195	21	180	1088	1267
60.048	20	CGCATTATGTTGCCTTTGAA	59.702	20	152	786	937
61.031	20	AAAGCCAAATGAATGGGGAT	60.511	20	126	21	146
60.195	20	TCCCCGAGTTATCGATCTTG	60.029	20	189	906	1094
60.008	20	CGAGAGAAAGAGATGGAGCG	60.232	20	236	61	296
59.851	20	TGATAACCATGACCTCACGA	57.442	20	256	488	743
58.882	20	TTCTCGTGTTCCTCCTCACC	60.238	20	103	0	102
60.029	20	AACAGCCATTAAAAGTCGGC	59.229	20	129	290	418
59.96	20	TGTACCAGGGAGTCTCGGTC	60.112	20	236	86	321
59.95	20	TCGATATTGTTGTTGCCGTG	60.523	20	278	572	849
59.61	23	CCAACTTGGATATTGGTGGC	60.192	20	264	454	717
59.963	21	ATACCGTTGAGACCACCTCG	59.989	20	279	108	386
59.84	20	CGGCTTAGCTGTTCGTTGTT	60.44	20	238	1323	1560
59.462	20	CTCTTGGGCCACACTTTCTC	59.844	20	266	146	411
59.88	20	CGAGCTCGAGTTTGAATTTG	58.649	20	280	679	958
59.84	20	CGCCCTCTCCACCTATCTCT	60.746	20	169	17	185
59.735	20	ATGGCAAAGAGCAAGTCAAA	58.502	20	252	91	342
60.071	20	TTTGTGTGACCCATCTTCCA	59.935	20	199	2232	2430
59.789	20	TTGCCATCCACTACACCAAA	59.964	20	269	551	819
58.807	20	CCGGGGCATTACATTATTTG	60.033	20	183	181	363
59.22	20	TGGGAGAGAGATCGATATTGTG	59.15	22	279	364	642
58.674	20	AATAACACGGCACCTACCCA	60.249	20	235	2798	3032
57.043	20	CAGAAACTACATCGCGCAAG	59.632	20	132	164	295
60.366	20	GGGGTTATCATGGGATCAAA	59.439	20	220	64	283
60.051	20	GCATGCTGATAGGCAACAAA	59.839	20	209	1339	1547
59.803	20	AAGTTGGAGGTGATGATGGTG	59.835	21	112	66	177
59.238	23	CGGGTTATGGCAGATTTCAT	59.784	20	142	5	146
59.101	22	TGTTGTTTGGAAAGTTGGAGG	59.994	21	237	641	877
59.579	20	TTCTGGTTTGGAAGGGAACA	60.465	20	216	133	348
59.485	21	CTGTCAAACGTGGTGAATGAA	59.6	21	276	164	439
59.697	20	GTCCACGGTGGTTTGAGAGT	60.009	20	141	38	178
58.625	20	TTGGACCTGCCACCAATTAT	60.192	20	229	739	967
60.005	20	AGCAATATCGATCTCGCAAA	58.483	20	207	48	254
59.947	20	ATGGATCAGTTAATCGGGCA	60.296	20	178	18	195
59.538	20	AAAATCAAAGGCCCACAATG	59.801	20	151	403	553
59.901	20	ATCTCAACGATATGGCTCGG	60.059	20	222	37	258
60.492	20	GCACACACCCACAAACACTC	60.055	20	220	103	322
59.966	20	TGAAGAGAGAGCTTCTGTCGG	59.876	21	278	177	454
60.103	21	GTGTTCGATACCGGGTTCTT	58.912	20	246	96	341
60.073	20	CACCCCACCTCATTTTCAAC	60.21	20	235	180	414
60.255	20	TTTTGAGATGGAACCACTTGC	60.103	21	164	6	169
58.994	22	TGCAAATACGGAAAATTTGACA	60.347	22	280	681	960
59.991	20	TTCTTCCTCACACCTCAGCC	60.386	20	237	240	476

60.251	20	TGAACAATTGAAACACCGGA	59.941	20	255	64	318
59.939	20	TGATCCATTCCGACTTCCTC	60.011	20	227	140	366
59.859	20	CCTCCATCAAACCATTGTCA	59.343	20	273	2329	2601
60.12	20	TTGAGCTATAGTTGGAGGGTAGC	58.986	23	176	6284	6459
59.882	20	ACATCTGTGGAGCTGCTGTG	60.055	20	254	1240	1493
60.255	20	CTTTAGGTGCATCTCTTGACGA	59.517	22	210	142	351
60.05	20	GTGATTCATGGCCGAGGTA	59.466	19	227	71	297
59.975	21	ATTTAAGCCCCATCCCCTAA	59.637	20	232	168	399
57.497	26	CTCAAGACCCGATCGGTAAA	60.066	20	254	50	303
59.487	22	GAGAGAAAACCGAGAAGCGA	59.694	20	260	41	300
59.836	20	AACACCTGTAATCGGGATCG	59.813	20	157	2	158
59.721	25	CGCCAAGTGAGTTATTGCAG	59.488	20	280	23	302
59.826	20	CGCGTCAGATTTGTTATGTGTT	60.06	22	253	90	342
59.873	21	TAAGGGTTATCACCGGATCG	59.778	20	108	0	107
60.288	20	GCGTCAATTACGTTCCATGA	59.548	20	241	1831	2071
58.928	21	AGGGGTCAAACCTCCACTCT	59.968	20	209	130	338
59.859	21	GTGCCAAAAATTGTCATCCA	59.375	20	228	127	354
59.806	19	AACTGGGCCATGATAGGTTG	59.813	20	246	0	245
59.969	20	GACTCCCTTCCTCAACTCCC	60.05	20	107	640	746
60.277	20	TTGGGCTACTGGAGATTTGG	60.066	20	162	3631	3792
59.939	20	TTTTGGGAAATGGGGTATGA	59.988	20	235	707	941
59.872	20	TACCTAATCTCCCGCCCTTT	59.924	20	277	148	424
60.193	20	GATCGGAGGAGATGAAACGA	60.158	20	112	148	259
59.934	20	ACATCAGAAGCAGTGGAGGG	60.261	20	254	80	333
59.822	22	GGAGGATCTTCTTGAGACACAGA	59.87	23	273	107	379
60.096	20	CATCCCTCGGTTCAAGGATA	59.887	20	274	2338	2611
59.874	21	TGCATCTTGGAGGTGAGTAGG	60.262	21	231	25	255
60.132	20	TGGTACCTCCAACAAGAGGG	59.959	20	279	87	365
59.901	20	CTGCTGACTGAAGCGGGTAT	60.419	20	258	509	766
58.879	20	CACCCCTTTCCTTTACCCAT	60.046	20	248	164	411
60.111	20	GTTGTAGGCAAAAGGGCGTA	60.131	20	162	363	524
59.63	22	ATTGTCATCCACGCAAAAAT	57.943	20	265	104	368
60.381	20	AGGGCAACAATCATGAGTCC	59.934	20	213	477	689
58.637	26	TTGTCATGTCCACCTCATTCA	59.951	21	182	501	682
59.943	20	CATCCAAGACATGATCGCAC	60.08	20	262	25	286
59.948	20	TTTAAACCCCAACACCTTCG	59.833	20	249	404	652
59.971	20	CTGGAGTGTCAAGCTGGGAT	60.261	20	198	103	300
60.332	20	CGAGGAAGTCGAGGATTGAG	59.943	20	220	25	244
60.281	20	ATAGCGTCTTCCAGTTTCCG	59.336	20	276	204	479
59.722	20	GATTATGTCGTGGATTTTGAACA	58.867	23	250	55	304
60.135	20	CCCTTTGAATTTTGCCTCTG	59.679	20	135	151	285
59.799	21	GAGTCAGCGGAGCTTTTACG	60.154	20	189	1	189
60.874	20	TGATCTGAGAATTCAGCAACAA	58.502	22	279	320	598
62.112	20	CGAACCATGAAAAACATGGA	59.371	20	230	25	254
59.826	20	AGGCACAATAGCCACGAATC	60.103	20	261	244	504
59.603	26	ACGAACTTGACACGAACACG	59.794	20	270	357	626
59.916	20	TATTCGGAGGGAGGGAGTTT	59.897	20	203	389	591
59.817	20	TTTGGGGCTACGTGTTCTTC	60.11	20	235	58	292
60.195	20	AGAGAAACTGGAACCCCCAC	60.349	20	181	122	302
58.843	20	CGGTTATCACCGGATCGC	62.397	18	250	836	1085
59.962	20	AAATAGCGCATTTTTGCCTG	60.226	20	232	903	1134
60.426	20	TTGCATGCTCAATAAAATGGA	59.159	21	251	1266	1516
59.883	20	ATGGGAATCTTGGGGAAGAC	60.133	20	194	232	425
59.838	21	GAATTTCCCAGTTCCAACCA	59.767	20	136	866	1001
60.833	20	GCAAGATCCAATGGTTGTGA	59.502	20	279	462	740
59.976	20	TAACCCATTTCAGCCGAATC	59.901	20	273	1923	2195
59.597	20	CCATCCTCCTCCAATAGCAA	60.029	20	218	1782	1999
59.464	20	GCACAGTGTCACCACAATCC	60.013	20	277	695	971

59.993	20	GACGGTGGAGATTGAGGAAA	60.05	20	197	1048	1244
59.238	21	AGTTTTGAATGGCTTGGTGG	59.971	20	241	592	832
59.709	20	AGGGAGGTGGTTGATCAGAA	59.505	20	279	122	400
60.169	20	ACGGCTGTGTAGTGTGTTCG	59.819	20	163	578	740
59.934	20	GCAAAAGCTGAGGTGTCAAA	59.05	20	245	11	255
59.947	20	TAAAAATGACCCTCGTTCGG	59.931	20	249	3	251
60	20	CAAGCCCGCATTAAAACATT	59.964	20	208	19	226
59.969	20	TAGTCGGCCGATAAGTTTGG	60.089	20	155	870	1024
59.299	21	CTTCGAGCTCCTCACATTCC	59.95	20	160	27	186
60.122	21	CGTGGAATTTCATGGTTTGA	59.371	20	222	18	239
58.734	25	TCATGTCCGTCCATTTTGAA	59.9	20	226	660	885
59.941	20	CAAGCCTAACACCACAAGCA	59.904	20	207	1755	1961
59.499	20	CTACCCTGCTGATGAAACCC	59.55	20	128	51	178
60.029	20	AAATCCATCCCATCCACGTA	60.014	20	216	296	511
60.051	20	AGCCAGCTAGTCGGTTTGAG	59.639	20	164	161	324
59.668	23	ATCGACCCTAATTCGAACCC	60.152	20	276	1190	1465
59.881	20	TGCATGTGAGTTGCACTTGA	60.029	20	209	456	664
60.161	21	TTTTGATGCAGCAACCATAAG	58.83	21	280	57	336
59.685	20	CATTGATTGATTCACAGCCCT	59.947	21	224	152	375
59.914	20	GAAAGTATGGCTGCTCCTCG	59.978	20	165	1915	2079
60.246	20	TTTGGGTGTTCTGTACACGG	59.46	20	165	182	346
59.23	20	GGGTCTCGCATAAAGTGGAA	60.074	20	278	124	401
58.92	20	TGATCTTGCTTGGTTGCTTG	59.988	20	260	2044	2303
60.051	20	CACATGTGGAGAGCTTGTGC	60.473	20	276	490	765
59.973	20	TTGCAAGATTTGACGATCCA	60.197	20	241	1710	1950
60.449	20	CCAACTTTGTGTTCTTGGCA	59.734	20	103	27	129
58.663	23	TTGTTAAAGGTTTCGACGGG	59.968	20	101	11	111
59.903	20	GAGGCTGTGAAATTGAGGTTG	59.726	21	101	277	377
59.359	20	CCACTTACCGGAGCTATCCA	60.088	20	241	181	421
59.864	20	ATGCGCGATTTTAAACCCTA	59.586	20	270	1579	1848
59.966	20	AATGCTTGCAAATTTTCGGT	59.592	20	136	407	542
59.313	20	AAGATGGAGAGACGGTGGTG	60.112	20	274	220	493
59.982	20	TGCCAATGGAATAGTGATCG	59.499	20	269	1664	1932
59.433	20	CCCACACTCACATGTACAAACA	59.385	22	152	324	475
59.89	20	TCCTTCCGGAGTACGAGCTA	59.971	20	111	36	146
58	20	TCGAACATGTAGTGGTCAAACTG	60.081	23	195	292	486
57.757	21	TGAACAATGAGATCCACAATCA	58.976	22	264	439	702
59.01	20	TTTTTACCTTCTCCAGGGCA	59.679	20	264	4236	4499
60.049	20	ATGTCACTGCCTTGCCTCTT	59.874	20	216	617	832
58.362	20	ATTTCAGAACGACACCGAGG	60.111	20	176	742	917
59.697	20	TGCCTTAGATCCACCGGATA	60.427	20	216	588	803
60.008	20	ACCCGAATTACCACCCCTAC	59.94	20	270	1898	2167
59.862	21	ACACACGAACACTCACCCAA	60.047	20	198	69	266
60.028	20	GGAAATCCAAACGAAAGCAA	60.053	20	213	60	272
58.983	22	TTGCCTTTTGATTTTCGGTC	60.053	20	280	697	976
60.044	20	GCAACCAAGTTGTCAACCAGT	60.066	21	251	65	315
59.322	20	TTGACTGAACTTCACCCATTT	57.16	21	199	175	373
59.853	20	CCTCACGAGTTTGGACAGTG	59.305	20	237	379	615
59.823	20	AGACCGGAGTAGATGACCTTC	57.321	21	218	250	467
59.505	20	GATTTTGACATCAAGATAAGGCA/	59.525	24	220	408	627
59.836	20	AAAATCCAGCAGGCAAAGAA	59.823	20	231	103	333
59.255	20	TTGTCCTCAACTGAAAGAATGC	59.359	22	269	1679	1947
60.323	20	CCACAGCTTTCAACATCACG	60.301	20	261	77	337
59.933	20	ATCGGAGCTGAGAAAGCAGA	60.24	20	228	854	1081
60.208	20	CGCCATTCTCTCTCTAACCTG	59.087	21	171	279	449
60.011	20	GGCCAGATATCTCTCCATGC	59.616	20	253	2717	2969
59.874	20	CACCAACAGCTTGTCGAATG	60.301	20	159	1883	2041
60.011	20	CAAGGGCCGAGATTTGTTAG	59.702	20	202	1377	1578

59.938	20	TCCATTCCGAAAATCAAAGG	59.872	20	178	176	353
59.948	20	TGACGTTGGTGGATTGAAAA	59.941	20	154	322	475
60.249	20	CATCGGTTCATGTCACTTGG	59.96	20	185	5	189
59.623	21	TCTGAGATGTACACTGCGCC	60.016	20	160	17	176
57.44	23	GATAGGATACGGCCCATTCA	59.745	20	254	17	270
59.972	20	TCTAAATGGTGGCCGAAAAC	59.938	20	171	445	615
60.103	20	CAACGGCTATTTACATGGGG	60.202	20	266	681	946
60.192	20	TGCATGCTATACGGCACTGT	60.31	20	225	2771	2995
58.972	27	CTAATAGCAGGCAAAATGCG	58.609	20	241	2	242
58.028	22	TCAATATTTCCCAAACTCGGA	59.397	21	191	15	205
60.01	23	CGGATTATGTATGTTACTTTTGGG	58.797	24	173	14	186
59.625	22	CAAAATTTGGGGCAAAAA	57.057	18	277	742	1018
60.682	20	TCTTAGAAGGGGCCAAAGGT	60.068	20	101	6149	6249
59.307	22	CCAGAGGAAAATGGGTCAAA	59.903	20	254	130	383
59.928	20	CTCAGAGGCACCTCATCCTC	59.945	20	130	1788	1917
60.214	20	TCAAAATCAAGTCTTCCTCCG	59.298	21	200	339	538
59.255	24	ACCTCGCGAAAGAGACAATG	60.397	20	270	23	292
60.136	20	GCAAGACATGGTGTTGTTTGA	59.606	21	267	842	1108
60.246	20	TGTAGGGGACTCGCTTCAAC	60.255	20	166	76	241
59.556	20	GGGTTCCAGCGTCATAAAAA	59.938	20	277	317	593
60.984	20	TGGGTGATCTGAGAGGAAAAA	59.656	21	248	518	765
60.045	23	GGACCTCATCTGTTGTAACCACTA	59.726	25	273	390	662
59.779	20	CGGTGGAAGCTTTGTCTGTT	60.291	20	223	1065	1287
59.767	20	TCACGCTAAGGGGTGGTATC	59.955	20	187	371	557
59.993	20	TCCGTGCATGAGAGAGAGAA	59.656	20	280	1500	1779
60.742	20	AGTCTGCCATTCTTGCAACC	60.263	20	221	438	658
57.677	21	CGAGGATGTTGTCGAGGAAT	60.073	20	198	83	280
59.937	20	GAGGACACTTGGACCTGGAA	60.088	20	226	784	1009
60.112	20	GCTTCGTGTCTCTGTGGTCA	60.03	20	203	90	292
59.676	20	AATTGAGGGAAATGTCAGCG	60.074	20	199	1707	1905
60.175	20	CTTTTCTGTATTGGGGCTGG	59.564	20	238	465	702
60.042	20	GGTGGAGGAGGGTATTAAGATG	58.847	22	119	568	686
59.881	20	CCTCTGGACCGACCATTTTA	59.926	20	206	467	672
59.55	20	CGCAGAATCATGAGACACAAA	59.856	21	236	16	251
59.606	25	GCTGCTGTGTTTGTCTTGGA	60.032	20	216	292	507
62.18	18	TGGGTGAGAGAAAGATAGATGAG	59.371	24	238	447	684
60.321	20	CAGCAGGCAGATCACAGGTA	60.008	20	226	658	883
59.717	20	TTGGTCCACCAATCTTGTGA	59.935	20	260	894	1153
60.147	20	TCATTATTACTGCGCACCCA	60.096	20	138	19	156
59.861	20	TGAGGAGCGTTGATCTTGTG	59.984	20	234	354	587
60.758	21	AACCCGCTGTTTGTTTATCG	59.996	20	255	265	519
59.76	21	CATAATGCCTTCCCGTGACT	59.955	20	208	117	324
59.214	20	AACAATGATGGACACATGGC	59.223	20	143	539	681
59.722	20	GAAATGATTGGAGAAGGGCA	60.014	20	202	134	335
60.16	20	ATCCTTGCACCTCCTCTTGA	59.803	20	274	2185	2458
58.597	20	GCATCTAGCCACTGCTGTCA	60.168	20	245	274	518
59.91	20	GCACAACAATCTGCAATCTCA	59.862	21	253	1032	1284
59.879	21	CGCGTCCGACTCCTTAACTA	60.399	20	110	269	378
59.452	20	TGAAGTTTGGCAAGGAAACC	60.088	20	198	167	364
59.365	20	TGGTAGGCCAGAGCTTGATT	59.836	20	173	95	267
59.056	20	ATGCAATTTTTGTGGGCAAT	60.196	20	259	755	1013
59.816	20	TCAGCATGATATTGCGGAGA	60.329	20	217	26	242
59.989	20	CAACAAATTCCAATGAAAGGG	59.302	21	212	164	375
59.989	20	TCACATTCAACCTCAGCGAA	60.39	20	255	1278	1532
60.048	20	CTCTCTCTCCTGATGCAGCC	60.246	20	235	2656	2890
59.962	20	TATGTCGAGGAGGACGCTCT	59.973	20	240	3047	3286
60.008	20	TGTGGCTTGAGTGAAGTTGC	60.032	20	261	809	1069
60.292	20	TTCCAGCAATGGAACCAAAT	60.309	20	277	972	1248

59.875	24	TGCTGTGGACACGTTCAAAT	60.16	20	249	24	272
59.827	20	TGGGAACATTATGATGGGAAA	60.002	21	217	633	849
59.985	19	GGACCATGTACTGGACTGGC	60.395	20	244	1694	1937
59.993	20	CACCGGGTACTGAGGGATTA	59.807	20	251	325	575
60.324	20	CACGTGTAATTAATTCGGTTACCA	60.057	24	264	31	294
59.85	20	GGGAATTGGCCCAAATAAGT	60.02	20	262	51	312
60.184	20	CATCAGTCTACAGCCAGGCA	60.008	20	126	857	982
60.197	20	TTGTGCCGTGATGATGAGAT	60.08	20	263	261	523
60.242	20	ACTTTTGATGCCGAAGTACAA	57.439	21	254	103	356
59.019	20	ACACATGTCGCCTTCTGGTT	60.577	20	197	35	231
60.051	20	CGAGGAATTTGAAGAACACTTG	58.88	22	266	1390	1655
59.172	21	TCCATTAAGCCCTGAACCTG	60.066	20	272	163	434
60.199	20	TTGAACTCACGATCTCACGC	59.992	20	165	25	189
59.648	20	GCATAAATTGTGTTCCCCGA	60.701	20	268	183	450
60.257	19	GTTGGAGGAGAGCTGTAGCG	60.156	20	239	22	260
61.095	20	CTCGAAGGGAGGAAACAACA	60.224	20	212	21	232
57.38	23	ATGGGAAGAGGGGGTATGAG	60.147	20	239	1277	1515
59.843	23	GAGTGTGTCGGGAGTGAGGT	60.161	20	250	214	463
60.074	20	ACAGGGTCAAATCGAGATGC	60.081	20	206	778	983
58.917	20	AGGAAGAAAGAAGGGGCAAA	60.18	20	196	65	260
59.875	20	GAATGCTGCTGTTGTGCATT	59.878	20	268	2376	2643
59.74	20	TGACGGACCTTCATTTTTCC	59.91	20	188	526	713
59.903	20	ATGAAAGGGACACGTGGAAA	60.353	20	127	1381	1507
60.765	20	GGAATGGTGTGGGGAATATG	59.871	20	124	832	955
58.174	20	CGTAAAAGAAGTCGTTGCCC	59.747	20	222	183	404
59.913	20	GGGTAGTGGCGAGGTGAATA	59.955	20	113	71	183
59.962	20	GGCCCCTCTACTCCTGTGAT	60.477	20	272	114	385
59.851	20	GAACCACCACCACTGTTTCC	60.261	20	212	410	621
59.825	21	TGATTGATTAATTCGTTTGTTAGGA	59.009	25	215	125	339
59.811	20	TCCATGAAGGAAAAAGTCGG	60.044	20	176	145	320
59.88	20	CTCCATTTCTTCTTCCTGCG	59.948	20	233	262	494
59.823	20	AGCTGGCTCAACTACTCCCA	60.012	20	246	2693	2938
60.042	20	CCTAGCTCTTGCCCCTTTCT	59.976	20	278	107	384
60.178	20	ATCGGAGTTGATTTGATGCC	59.9	20	209	594	802
59.855	21	TGTAAAGTGAACAATGGAAGCAA	59.677	23	215	4964	5178
59.971	20	ATTTGCTTGATCCAACGGTC	59.939	20	163	959	1121
59.939	20	CCAATTTGAATGGTTATCACCTG	60.457	23	157	1232	1388
60.177	20	ACAGTGGCGAATGTATGCAA	60.142	20	164	241	404
60.278	20	TTTCGTTGTTTCCAGCCTGT	60.668	20	239	2201	2439
59.903	20	TCTGCTTAGCTTTTGGACGTT	59.167	21	173	4369	4541
60.008	22	GTGAAGTGGGGTAGAAGGCA	60.111	20	264	50	313
60.045	20	AACATGCACACAAGCACACA	59.785	20	255	174	428
59.934	20	ATATCGTGGCACCATTGCTA	59.019	20	159	270	428
57.046	24	GCAGTTGGACTTTTCGCTTC	60	20	259	526	784
60.331	20	GCTTCAGCAAGGAAGATGCT	59.723	20	125	1434	1558
60.309	20	GAAACCCGTGAACTAAACGC	59.615	20	146	701	846
59.991	20	TGTGAACCTTTTTCAGCCGT	60.668	20	189	1443	1631
59.542	20	TCCAACGTTGTAACACACACA	58.508	21	134	4	137
60.356	20	TGAAGCACAACACCAAGCTC	60.032	20	247	284	530
60.558	20	GGGGAGGAAAGAGAGAGGTG	60.186	20	158	45	202
60.592	20	GGCAACTTTTGATTCTTCTCG	58.962	21	256	4356	4611
60	20	CCCAATAACTCGCAATAGTTTG	58.698	22	133	482	614
60.16	21	CACAATATTTGAGGGTTATCATTT(	58.787	25	212	255	466
60.422	20	TTCCAATAACTCACAAAACTTTAA	58.364	26	185	270	454
59.802	25	TCAAAATCAGCAATCGGACA	60.197	20	233	3220	3452
59.558	20	ACGGCCGTGTACTCTGTTTT	59.662	20	131	416	546
59.133	20	ATCCCTCGACTTTCCCCTAA	59.897	20	187	1800	1986
58.049	24	CATGTTCCCAATTAAATAAATGTT(	60.219	26	272	89	360

59.505	20	AAAGAAGGACCTCCCGTGAA	60.988	20	162	714	875
60.744	20	AATGGACGAAGATGCTCTGG	60.218	20	226	46	271
59.478	20	ATCTTCGCTCCTTCAGGGTC	60.735	20	277	450	726
59.924	20	CAGTTTCGAGTTTTGAAGTTAGGA	59.033	24	167	150	316
59.641	21	GAACTGGTGGCAACTGGATT	59.973	20	133	1027	1159
60.104	20	TAGGGCCGAAAACAACAAAC	59.975	20	154	4	157
59.801	20	CGTCCACGTGCATAATGAAA	60.523	20	245	3	247
59.914	20	GTGGCACCAATCAACTCCTT	59.973	20	128	497	624
59.85	20	TTGGAGAACAGGTAGCGGTC	60.255	20	158	32	189
60.006	20	CGGGAATGCACCAAGAAATA	60.827	20	113	89	201
59.266	25	GCCCACCATTTATCTAAACCTG	59.737	22	211	94	304
59.592	20	GGTTTCATTTCCACCTTCCA	59.767	20	217	338	554
60.204	20	CGACATTAAACACACGGTCG	60.027	20	156	5	160
59.973	20	ACCCACACATGCAAGACAAA	60.008	20	212	627	838
60.776	20	CATGAACCTCCAGGGAAGAA	60.042	20	277	389	665
59.894	20	GCCAACACCTACACCAGGAC	60.431	20	182	2977	3158
58.972	27	CTACGATCCGATCCAACGAT	59.917	20	176	3	178
60.625	25	CCCAAATTCAGGCTGTTCAT	59.933	20	186	1	186
60.019	19	CCTCCCCGTATCGATTATCA	59.739	20	268	62	329
59.966	20	GACTTGATGCATGCTGCTGT	60.024	20	214	1102	1315
59.817	20	CAAAATGAAGGCAATGAGCA	59.809	20	139	99	237
57.228	24	GCTATCCGCTGAAAAGCATC	59.951	20	204	79	282
61.298	20	CTGTCCACGGCTCCTAAAAG	59.869	20	277	1164	1440
60.469	20	TGGAACAACACTTCAACAACG	59.643	21	277	4151	4427
58.744	23	TTGCTAGAGGGTTTTGTTAATATG[illegible]	59.12	26	115	1167	1281
59.973	20	TTGGTGGGAGATTGCTAGAG	57.894	20	278	18	295
59.712	20	TGTTTAATCAGGTAGAAAGTTGCA	58.854	25	233	1115	1347
59.992	20	ATGCGGCGCCTTTTTAAT	60.557	18	275	139	413
59.903	20	TCTTCTTCCCCAATCCTCCT	60.008	20	250	1574	1823
59.992	20	CATCCCATCACTACCCCATC	60.011	20	128	122	249
59.992	20	TTTCCCAAGGATCAGACGAC	60.05	20	224	2004	2227
60.005	20	CTTGCTCGTTTTATCCCTCG	59.839	20	236	416	651
59.685	20	GACGAGCAAGGATAGCGAGT	59.603	20	173	2	174
59.316	20	TTGGACACATAGTTCATGCCT	58.09	21	153	49	201
59.79	20	GCTCTCAAAGATGCGGAAAC	59.962	20	185	121	305
57.046	21	TCCCGAACTCTTTCAGCACT	59.989	20	239	104	342
58.48	21	CATCTTCTCATCTCAACTCCCA	59.284	22	277	2	278
59.631	21	AATGGAAATGAGTGGCGTCT	59.556	20	102	145	246
60.838	21	TGCCAAGTGTGTTGAACCAT	60.008	20	243	10	252
60.21	20	TTGAAGAAGCACAGGCAATG	59.988	20	244	101	344
59.823	20	TGCAAATTAAATCAACGGGG	60.671	20	271	227	497
60.008	20	CCGGCATCATCTGCTTTTAT	60.06	20	209	105	313
60.319	20	CCCGAATTCGATACAACAAA	58.48	20	133	65	197
60.025	20	TTTTTGAGGGAAAGTTGTGGA	59.573	21	280	40	319
59.66	20	GGTGAAATGACCATTTTGGG	60.029	20	239	2281	2519
58.995	20	GATCGTGAATGATCTACGTCCA	59.962	22	111	242	352
60.495	20	AATTCCCCATGACCCACAT	59.854	19	277	88	364
60.096	20	GGAAGAACACCCCATACGTG	60.232	20	276	132	407
60.617	20	TGTGCTAGGGTTTAGGTGGG	59.986	20	167	42	208
60.419	20	TTTGGCTGATTGAATTTATGGA	59.433	22	270	585	854
59.701	20	AGAATCGATTACTCCCGCCT	60.06	20	213	91	303
60.044	20	GCGGTCCAGATCTGCTACTCT	60.938	21	167	324	490
60.05	20	GAAGTGAGGATCGTGGAAGG	59.655	20	113	6	118
59.799	20	ATGCATTTTCTGCACACGAG	59.871	20	236	211	446
59.935	20	CACCAGCAGTCTGTCCTTCA	60.022	20	228	711	938
59.697	20	AAAGCAATGGCGGATACAAG	60.096	20	267	1	267
60.166	20	AAATTTGCCCTTGTGGATTG	59.801	20	252	2243	2494
60.246	20	GTTTGGGCTTTTAGCCTTCA	59.338	20	217	658	874

59.676	20	GGGATTTATTTTGGGGTACA	59.774	21	106	358	463
60.042	20	CCATCTTCCGTTGTCGATTT	59.933	20	251	114	364
59.092	25	GCAGCCGATTGAAGAACTTT	59.457	20	109	4	112
59.727	20	TGGAAGAAAAGGTTGCTGCT	59.993	20	221	188	408
60.044	20	GCGGTCCAGATCTGCTACTCT	60.938	21	173	338	510
60.08	20	TCTTCTTCCTCCCTGGGATT	60.008	20	170	112	281
59.676	20	TGCTGTTGATGGTGTTTGGT	60.008	20	267	1821	2087
60.035	20	ATTCTTCAATCAAGGCGTGG	60.074	20	235	854	1088
58.028	22	AGGCTCTGCATGCTCTTTTT	59.222	20	164	17	180
58.977	20	CAGTTGGGTCAGTGACATGG	59.997	20	203	3	205
60.054	20	AAAAATGGTTATTACTGGAACGC	58.564	23	258	440	697
59.933	20	TATGTGTGATTTTGGCTGGG	59.395	20	232	117	348
61.046	20	TATTGATGGAATTGGGGTGG	60.384	20	195	617	811
59.933	20	GGCCAATTCTTGGTAATTTCG	60.659	21	253	15	267
60.088	20	GTGAGGACTGAGGGCAGAAG	59.986	20	115	1588	1702
59.801	20	TGGGTTTGATTCCCAAATGT	60.029	20	176	309	484
59.6	20	AATTCATGGCTGCGACTTTC	60.221	20	268	265	532
60.37	20	ATTGAATTCGGATCTGACGC	60.043	20	264	321	584
58.799	20	CGCTTCCGGAGTGGATAATA	60.053	20	158	86	243
60.64	24	ATAGCGGACACTTGGACCTG	60.134	20	101	0	100
60.218	20	TTTCCGATAGGTGTTGGGAG	59.926	20	192	266	457
59.632	23	TCGGTCAAATTCAAACTCGT	58.201	20	239	1332	1570
59.697	20	TGTTGTTGGGTTGTTGGTGT	59.745	20	215	323	537
60.002	20	GGTCTAGGCCCCTCAAATCT	59.536	20	139	1127	1265
59.55	20	ATCGAACTCTTGCGTTGCTT	60.022	20	132	43	174
59.95	20	ACGTAATTTGGCGGTCTCAG	60.132	20	239	552	790
59.705	20	TCCACAAAAAGTGGGTGTGA	59.976	20	143	36	178
60.306	20	TCCAAGAGTCTGCAGTGTGG	60.022	20	100	22	121
59.964	20	GCTACCCCTCCCTACACCAT	60.212	20	208	595	802
59.648	20	CTTTTCACACGAGGTCACGA	59.873	20	199	82	280
59.83	20	TGCAATCCAGTGAAAACTCTG	58.912	21	190	48	237
58.328	20	TCATGTTGCATATTATTTGGCAT	59.256	23	241	60	300
59.981	20	ATTTCCCACCAAACAGCAAA	60.344	20	191	533	723
59.973	20	GGCACACATCTGTGACATCC	59.97	20	140	234	373
59.809	20	GGAGTTCAAATTCGGTTGGA	59.91	20	182	1159	1340
59.508	20	CACCAGATCACTCCCCTGAT	59.92	20	268	1303	1570
59.785	20	GCAGCTGCTGTGTGGATAAA	60.019	20	187	1147	1333
60.133	20	AGCGGTTATTACCGGATCG	59.93	19	163	479	641
60.111	22	CATTCAACTGGGCATTTGTG	59.964	20	206	27	232
59.496	20	CAAGGCTTGAAGCTGGTAGG	60.008	20	252	76	327
60.111	20	AACAACGGTATCACCTGAACG	59.906	21	151	3990	4140
58.826	27	CTTCTTTCCTTTCGCGTGTC	59.993	20	159	3	161
60.066	20	CACGGTTAGGCTCGAGAGAG	60.149	20	154	81	234
59.789	20	ATGGAGGAAGGAAGGAAGGA	60.008	20	276	15	290
57.439	27	GAGCAAGGCACCTCAATCAT	60.226	20	182	11	192
59.685	20	TACCGGCTGGGTACCAACTA	60.378	20	167	478	644
60.011	20	AAAAGGGCGGTGGTAAAACT	59.871	20	125	45	169
60.593	23	AATCGGTGAACAGTTCGGTC	59.973	20	224	1882	2105
57.155	22	CACCAAATTTCATCATTTTTAATTC	58.035	25	205	149	353
59.881	20	TTTCTGTTCCCGATCTGAGC	60.34	20	233	2095	2327
60.452	20	GGGAGAATCGAGATTTGTTTTG	59.945	22	274	271	544
60.081	20	AAATTTGAGGGGGTTCATCC	59.996	20	240	22	261
59.971	20	AGTCCCTTTTTGTTGCCTGA	59.711	20	128	369	496
60.326	20	CATGAAGCGCTGATTGTTGT	59.871	20	280	1301	1580
60.345	20	TAGCCGATAAAAATGGCACC	59.928	20	259	91	349
59.903	20	CGCTAACTTCCAAAATGCCT	59.361	20	262	799	1060
60.683	20	GCCCGTGTGCATCACTACTT	61.128	20	132	550	681
59.349	20	GCACCAAATAATTCGACTTGC	59.605	21	270	167	436

59.96	20	TCGCAGACTGATCTTAGCGA	59.847	20	203	772	974
60.14	20	GCATTGATGGGATTCCACTT	59.756	20	212	1085	1296
60.136	20	TCACTCCAAAATCCACTCCC	59.903	20	136	304	439
60.088	20	TGGGTGCAACTTTCTAGCCT	59.875	20	164	7	170
59.975	20	GGGTTTGGAGGGGTATCCTA	60.011	20	197	158	354
59.769	20	TCCATAGCCGGTAAAAATGG	59.789	20	188	8	195
59.757	20	GATTTGCGTGGTCCATTGAT	60.733	20	270	1	270
59.916	20	TCAGCCTCCATCAATTATCAAA	59.547	22	163	183	345
61.087	20	AGGGGTGACTTCATGGAATG	59.779	20	221	1922	2142
59.957	20	GGACCACAAGTTCGACAACA	59.571	20	194	169	362
60.95	20	CATGCTGCCGGAAGTAATTT	60.096	20	262	150	411
59.221	23	TGGACATATGGAGGGAAGGA	60.272	20	255	831	1085
60.074	20	TCCCTCTTGAGTGAGCTTTGA	60.119	21	263	2536	2798
59.642	20	GCATTTTGGGTACACCTGCT	60	20	250	1185	1434
60.449	20	CAACAAGAACCAGGCATCAG	59.288	20	271	146	416
60.622	20	GATGGACTCCGAGGATGATG	60.439	20	207	51	257
60.225	20	AGGGTTTCAAGAATCCACCC	60.169	20	126	1821	1946
59.92	20	TTTTTGACGATTTTGCCCTC	60.053	20	245	1642	1886
59.757	20	CCGACCTCGGTAAATGCTTA	60.089	20	206	626	831
60.232	20	GCATTGTGATGTTGTTTGCC	59.979	20	210	503	712
59.691	20	TCCAATCGAAGATTCGGAAC	60.014	20	202	98	299
59.708	23	TTTGTTAGAGATCTTGACGGGA	58.834	22	204	960	1163
60.018	21	GTGTGTATGAGAGCCGCAAA	59.871	20	189	1350	1538
58.513	22	CAACTCCTTTGGAGCCATTC	59.67	20	260	3	262
59.989	20	CTGGCACATCTGCAGTTCAT	59.862	20	228	287	514
59.322	20	AAGCTGTGGGAGATGGAGTG	60.261	20	237	646	882
60.022	20	GTGGATGGTCCTCAGCTGTT	60.12	20	183	288	470
59.967	20	TGTGGAAATTATGGTTCGCC	60.701	20	253	791	1043
59.964	22	TGCGTTTACATTCAAGCAGTG	59.926	21	100	43	142
59.195	20	GTCTTCTGGCTTCTCTTGCC	59.166	20	114	36	149
58.288	20	ATTTCGAAGATCAACGGACG	60.074	20	111	65	175
59.545	20	AAATCACAACTCACACGCCC	60.966	20	242	24	265
59.076	21	CCCCATTAGCTTCATCCTCA	60.029	20	236	780	1015
60.072	20	CGGGCATGAAAAGGTTAAAA	59.937	20	274	191	464
60.081	20	CAATACAACTGCCGAGCTGA	60.011	20	134	52	185
59.744	24	ATGATCGCTCTGTTCCGATT	59.658	20	275	681	955
60.11	20	GCAACCAGGTACTCATTGGC	60.526	20	160	781	940
59.988	20	AAACCCCAACGGACAGATTT	60.59	20	174	1832	2005
58.661	21	TTCCACCTGATCCTGGTTTC	59.903	20	166	2084	2249
59.881	21	TTTTCATTCATGTTTCGCCA	60.051	20	228	673	900
60.183	20	TCAAACCCACGGACTGTGTA	60.001	20	157	1596	1752
60.009	20	GATAAAACAGGGGAAAGGGC	59.777	20	223	96	318
59.976	20	TACAAAGCTGCTCGGTCCTT	60.015	20	227	101	327
59.74	20	CGTGCCTAGTCTTTGCTTCC	60.015	20	160	473	632
60.249	20	GAAAGTATGGCTGCTCCTCG	59.978	20	174	325	498
60.124	20	TCGACGATCACAATTTGTCC	59.502	20	193	439	631
59.839	20	CCCAAATTTTGACCATTTCC	59.111	20	176	0	175
59.72	21	AAAAACACATATGGTGACACGG	59.654	22	225	1285	1509
59.83	20	GTCGGTGGCTGGAGAAAGTA	60.255	20	155	2593	2747
57.118	25	CCCATAACTCCAAACCCAAA	59.657	20	269	52	320
60.142	20	TCACATGGAAACATGTGGCT	59.967	20	191	176	366
59.269	20	GGGAATTTAATTTCTGGCCC	59.621	20	147	2665	2811
59.829	20	CAAAAAGCTGGGTGAACTCC	59.711	20	223	247	469
57.055	25	TCTTAAAAGGGAGGGGCTAA	57.95	20	280	138	417
58.418	24	CGGTTTAGGTATTTTGATGTTTTG	58.937	24	136	83	218
59.347	22	CGACGAGAAAGTCGTGTCAA	60.025	20	232	117	348
57.664	22	TGGTTACAAGCAGTGGCGTA	60.316	20	162	4	165
59.644	27	ACATGGATTGTGCATTCGAC	59.379	20	280	31	310

60.066	20	AGGGGAGTGATCTCATGTGG	59.92	20	247	161	407
60.111	20	TGCTGCTGATTCACATAGCC	59.979	20	237	87	323
60.019	20	ACCATGGCAGATTCATCCAG	60.893	20	197	165	361
58.965	21	TTTAAACCTTCCCCCAATCC	59.992	20	277	1561	1837
60.009	20	GTATGTGTGGCTGTGGATGC	59.999	20	245	53	297
60.14	20	CCTATGGTGGTATGGTGTGG	58.569	20	190	5	194
60.049	20	TCTACCACTCCGAAACCTCG	60.246	20	238	82	319
59.658	20	GCGACATCCAGTCTGAGTGA	59.988	20	116	129	244
59.765	21	TGGTACCTCCTCCTAATTTCCTC	59.854	23	181	258	438
60.036	20	TATGATGGGAAGGGTGGGTA	60.006	20	263	1166	1428
60.05	20	AGCGAATGTTGTGAGAAGCA	59.596	20	242	25	266
60.688	20	CTCGACACAAATCTGCACAA	58.409	20	262	248	509
59.985	20	CGGTTTTCGGTTTTCACAAT	59.839	20	235	78	312
59.995	20	AGGGCTACAACTCTCAGCCA	60.012	20	265	627	891
60.209	20	CTGCATACGAGCATGGAAGA	59.972	20	273	588	860
59.799	20	TTGAGTGCGTGTGTGAGTGA	60.07	20	231	157	387
60.39	21	TTGGGAAGGGAATGTGTCAT	60.173	20	158	3705	3862
59.027	20	CAAACGCTAAACGACACCAG	59.386	20	279	2	280
59.336	20	AAGTTATGCTGGTGGTTGGG	59.853	20	263	344	606
60.147	20	CGCGTTCAATATTCTCAGCA	59.976	20	207	171	377
58.122	24	TGAGGAACTTGAGAGTGGGG	60.229	20	101	167	267
59.311	22	ACATTTTCGGTTCATTGGGA	60.17	20	145	25	169
59.813	20	CTCTCCATTTTCCCTCCTCC	60.008	20	280	470	749
60.005	20	TGCATGATCGATATTGTAAGGAA	59.468	23	184	56	239
59.95	20	GAGCGTTAATGCCGTTTCAT	60.103	20	172	192	363
60.119	20	TCCATTTCCTTTTTCGATTCC	60.246	21	195	998	1192
57.73	23	CGGACCCAGGATTTTAGGTT	60.18	20	262	14	275
60.048	20	TTGACGGCCCTAAACAAAAC	59.975	20	206	182	387
58.249	23	TCCGCCTTGACACCTTCTAC	60.255	20	162	0	161
59.671	20	TGTGAATCAAGTACCCCGAA	58.973	20	279	72	350
59.963	21	TCGAACCTGCCCTCATTATC	60.036	20	227	401	627
59.234	20	CTACACCCATCGGAATCAGC	60.483	20	271	91	361
60.938	20	TGTGGCCTTATTCATGCTCA	60.22	20	251	372	622
60.294	20	GGGCCACTTTGGTGACTAAC	59.454	20	252	326	577
58.358	23	CTCTCTGCTGGCCAAAAGTC	60.134	20	273	781	1053
59.58	22	CATTACAGCCGTGAGAAGCA	60.011	20	265	2	266
60.136	20	GGCAACGATCATTTCTGGTAA	59.952	21	271	1250	1520
59.995	20	ACCAAGGGTGAGAAGCATTG	60.111	20	227	288	514
59.694	20	CGTGTTTTTCCCCCTTTTTC	60.674	20	252	3826	4077
59.864	20	GGAGGACCTAGACAGCCCTC	60.216	20	254	1289	1542
60.124	20	CAGCCAAATGTTGGGTTAGC	60.502	20	221	145	365
59.528	20	ATTGCCCATATCAGCACTCC	59.923	20	279	795	1073
60.187	20	ACCCTCGACGTTTTCCTTCT	60.11	20	220	1106	1325
59.92	20	ATTTGTCTGCATACGGGCTC	60.103	20	184	1261	1444
59.978	24	TTGGATGTTCCAAATACAGGC	59.815	21	279	2551	2829
60.281	20	CTGGAAAAAGTCACCGGAAA	60.081	20	277	49	325
59.989	20	ATCCCATTTCCCTCCTTGAC	60.133	20	185	113	297
60.526	20	TGGATTTTCTGAATTGTGGG	58.395	20	254	846	1099
59.934	20	TTTCATGCAACAAAACCACC	59.42	20	261	1918	2178
60.691	20	TCAGCAACTGAACCTGGAAA	59.415	20	160	79	238
60.134	20	ATGCATCCGTTAGATCAGGG	59.917	20	233	104	336
60.008	20	CCAGCTGACGAGCATACTGA	60.16	20	247	205	451
60.138	21	TCTGAATCTGATTGCGTTGC	59.955	20	266	39	304
60.535	20	CCCGAGGACACATAAGCCT	60.081	19	280	165	444
59.109	25	TCCATTCCGAAAATCGAAAG	60.009	20	280	609	888
59.375	20	AAAAACACGCGTTTGTAGGG	60.032	20	232	104	335
59.96	20	TCCGGTGAAACATTGACAAA	59.941	20	249	1319	1567
59.662	20	TTAAAGTTCTTAAGTCCTGGCG	57.792	22	255	677	931

60.326	20	AGATGGTTGCACCATAACGC	60.925	20	183	298	480
59.96	20	GGCACCAATTCTCATAATACACA	58.891	23	142	266	407
58.823	21	TGGTCGCCGTGTCTGTAATA	60.134	20	195	3852	4046
59.52	20	CGACAGGTGGGAGAGTCATT	60.112	20	251	393	643
60.103	20	CACCGTTGTCTTTAAGCCGT	60.168	20	240	715	954
59.057	24	ATACGGATCTCTGCCTCTCG	59.41	20	155	3	157
59.928	20	TTGGTTGGATATTTTGGGGA	59.988	20	244	266	509
59.971	20	GAAATCCCATCCACAACCAC	60.034	20	165	203	367
59.971	20	AACACGAGCAAAACGACAAA	59.366	20	251	95	345
60.25	21	GGAAGAGGAGGTATGAGGGG	59.89	20	223	556	778
59.82	20	AAGGAGGTGTTATGAAGGGATT	58.028	22	157	291	447
59.72	21	GATGTTTTCCGGCTCAGTGT	60.119	20	269	91	359
59.898	19	CGGTGACGGTTGAGAATGAC	61.527	20	271	183	453
60.519	20	CAGGAACCACCTCAGGGTTA	59.959	20	250	39	288
59.751	20	GAGTTTTCATCTTACCTTGAGGG	58.333	23	271	70	340
60.464	20	TCAAAACGCATAAATAGTGCAAA	59.687	23	280	44	323
60.042	20	CATCAGATCCATACTCGGCA	59.629	20	241	18	258
58.58	22	GCGAACGGTATAGAATCGGA	60.06	20	278	56	333
58.823	20	TGGATGATTCACAACCAAACA	59.808	21	189	943	1131
59.975	20	TTGCTAAGTTGGGCCTATCG	60.223	20	272	1126	1397
59.903	20	CACTTCAGCGTCACCTCAAA	60.025	20	274	123	396
59.985	20	GTTTAGCAAGCACGCCTCTC	60.162	20	278	1222	1499
59.062	20	GGTTGTTTCATGACATTTGAGG	59.336	22	270	411	680
60.214	20	CTTCTACGCGAAGCACGTTT	60.575	20	264	1236	1499
59.273	20	CCGTTGCAAGAGTAATGGCT	60.27	20	185	2773	2957
59.943	20	AGCAGGCGAGGTGTTACAAT	59.763	20	242	57	298
59.923	20	TTGATTAGTTCGTGCAACGC	59.879	20	275	386	660
60.26	20	CCACCATCTCTCTCTCCCAC	59.638	20	179	1448	1626
59.587	20	GGGTGGCTGATGAAGAGAAA	60.195	20	262	705	966
60.051	20	ACACACAAATGGTTTGCCCT	60.277	20	218	69	286
60.029	20	TGGGTTGGTTTGCTTACCTT	59.471	20	260	150	409
59.525	20	TTTACGGGTCAAAAGAAACCA	59.478	21	250	678	927
58.842	22	TGGCTTGTGAACGTGTAACC	59.615	20	184	67	250
59.934	20	TGCATGCACATCAAATCAAA	59.648	20	253	40	292
59.316	20	GCAGTGGATATGCAATGTGG	59.955	20	217	88	304
59.667	23	TTACCATCATTCTTGGCCATT	59.287	21	244	169	412
59.955	20	CAAAATTGCGTCTCCAGTCA	59.84	20	238	1501	1738
60.272	20	TATCGGTTATCATCGGATCG	58.404	20	171	439	609
58.589	21	GGAATTTCCTTGATACACATACAT	59.918	26	277	619	895
59.974	21	GGGATCTACATGCTGGGAAA	59.894	20	135	134	268
57.268	20	CTTAGATGCCATCAACCCGT	59.955	20	184	22	205
60.073	20	CATCAAGAAGCTGCAGCAAG	59.888	20	269	769	1037
60.24	20	TCCCCTCAAAATCCCCTAAC	60.124	20	190	56	245
59.879	20	ATTTAAAGGCACGGGAACCT	59.835	20	275	1087	1361
59.926	21	CGTTAAGATCAACGCGGATA	58.794	20	210	2491	2700
60.111	20	CATGGAGATCGTACGTTTCGT	60.008	21	173	2001	2173
60.802	20	ATCTCCGAAGCTCAAAACGA	59.955	20	100	261	360
60.103	20	TCCACAGTTTTGTACCCACTTTT	59.827	23	233	1489	1721
59.823	20	TACACGCATATTCGCACACA	59.741	20	181	149	329
59.935	20	GGATCGGAAGGATAAGAGCC	60.003	20	135	442	576
59.988	20	CCACAAAACCACGTTCCTCT	60.005	20	190	268	457
57.552	24	GGGAAGTTGAGATAATGGCG	59.528	20	272	11	282
60.545	20	TGATGGCGGTAGGTAGCTCT	59.859	20	231	288	518
59.108	22	CCGACCCTCGTGTTTAATGT	59.853	20	129	13	141
60.184	20	TATGGGGACTCTCATCTGCC	60.034	20	248	1075	1322
58.864	20	ACCTCCCCTGCTCCATATTT	59.789	20	101	286	386
60.292	20	CGGACCGTGAAAGCTCTAAG	60.008	20	240	234	473
60.003	20	TCGTTTAGGAAGCCTCTGGA	59.948	20	261	88	348

59.291	21	TTGAATTTCGTAACGGGGAG	59.931	20	196	11	206
57.409	21	TCGGGACGAAATTTTTGTATG	59.818	21	274	2	275
59.337	20	GGCAACGGAACAACAACTTT	60.015	20	280	3145	3424
59.037	20	GGTTCAAATCCCACCAACAC	60.073	20	249	1192	1440
59.992	20	CATCCCATCACTACCCCATC	60.011	20	129	144	272
59.962	20	AACCGAATAACCGAACCAAA	59.315	20	235	25	259
60.612	20	ATTCTTGGCCAAAACGTGTC	59.978	20	241	148	388
59.957	20	GCTTTCCTCATCTGCACCTC	59.957	20	259	1291	1549
57.073	22	CTTCTGCTGGTAGAGCACCC	60.012	20	233	1228	1460
59.445	23	AGTTTATCATTTGGGCCGGT	60.562	20	140	21	160
60.074	20	TTTCTTCACCGTTTTCCTTGA	59.71	21	219	470	688
59.662	20	ATTCCAAGGTTTTGCTGTGG	59.971	20	177	3077	3253
59.84	20	CAACAGTTTGGTTTGGCTGA	59.734	20	255	708	962
59.861	20	AGGCATAAAATTCTCCCGCT	60.061	20	251	21	271
60.38	21	GCTTCAGGGGGTGTGAAGTA	60.111	20	254	90	343
60.155	20	CTCTCGCCCCAAACTTAGAA	59.448	20	255	401	655
60.319	20	TTACCTCCTCCACCATCGAC	59.927	20	272	273	544
60.05	20	GCCGAATATTTTGGGCCTAT	60.135	20	222	416	637
59.79	20	GAAAAGAAATGAACACCGGC	59.551	20	210	62	271
60.011	20	AACTTTGCTGAGTTCGGGTG	60.291	20	270	471	740
59.098	24	TACCCCACCGACATTTTTGT	60.088	20	126	343	468
60.345	20	TCCGTGAATATTTAACCCGC	59.795	20	254	1158	1411
60.132	20	TGCAATCACCAAAGAACTGA	57.806	20	118	340	457
60.025	20	TGTCCCCTATTTGGACGAAG	59.926	20	210	184	393
59.449	20	AGAAATAAAGGCCCAGCTCC	59.688	20	151	2176	2326
60.213	21	TGGCCCAGATTTGTTCCTAC	59.933	20	185	1360	1544
61.129	20	GGTGCGTGAATTGGCTTAAT	59.967	20	239	128	366
60.081	20	GAGGGTTATCATGGGATCAAG	58.32	21	162	470	631
60.089	20	CAAGCTTGTGGAGTACATGAGA	58.036	22	252	40	291
60.52	20	TGGTCGCATGTTACGTGTTT	60.035	20	179	1342	1520
60.111	20	AATTGCCAAGTTTGCGAGAT	59.713	20	259	622	880
60.073	20	ATTGGCTGAAATGGAGTTGG	59.933	20	236	497	732
57.828	22	GGGCCAACCACGTTAGTTTA	59.861	20	234	449	682
60.029	20	ATGCCCGGTTACCCTCTAAA	60.683	20	156	514	669
60.264	20	GTCTGGGACAGACAACCGAT	59.969	20	174	85	258
60.033	20	TCAATCCTTTGGAAACACTCC	59.017	21	178	155	332
59.79	20	TGCGTTCACACCTTCATATTTT	59.519	22	196	142	337
59.971	23	CAACTCGCCTACTCCTTTCG	60.008	20	154	445	598
58.62	20	TGCAAGCCTATGGTAGGTGA	59.297	20	115	103	217
60.049	20	CACTGATCGTACACGATGGC	60.144	20	182	403	584
59.813	20	CACCATCATCGACAATCACC	59.769	20	215	385	599
60.397	20	ATTTAAGCTGCACTTTTATTCAACT	57.118	25	240	140	379
59.415	20	TTCCACGTTTCATGTTTCCA	59.941	20	144	60	203
59.415	21	CTTGCGCCGCCTAAATAATA	60.202	20	189	809	997
59.797	20	AGGGGCAGGATGTGTAGTTG	59.989	20	273	437	709
59.991	20	CCCTCGGATGCTATGCTAAA	60.188	20	231	351	581
59.703	20	CTCTCGTCTCCTCCTTCCCT	59.945	20	170	28	197
59.46	22	AAGTTGGGGATCGTGCATAC	59.82	20	261	97	357
59.83	20	CCCCTATATCCCCATGACTCT	59.138	21	228	1301	1528
59.629	20	GTTGGATTCGAGCAGCTCAT	60.37	20	256	143	398
59.962	20	TCAGCCACCAAATCAAATCA	60.049	20	161	114	274
59.901	20	TAGTGGTATGATGTGGGGGA	58.643	20	203	196	398
59.75	20	TAGAAAGATGTGCGGCTGTG	60.011	20	153	2081	2233
59.966	20	AAACTCTTCCGCCCCTTCTA	60.2	20	151	135	285
59.843	20	AGGAATCCTGCCCCAAGTAA	60.817	20	146	324	469
59.784	21	TGATTGGTGTGGTGAATTGG	60.216	20	276	71	346
60.19	20	TCCGTCCCAAGAAATGTGAT	60.317	20	207	2175	2381
59.756	20	TTGGAATTTTTCGAGTTCCG	60.046	20	176	899	1074

60.051	20	CAATGTAAGGGGGAGCAGAA	60.066	20	254	124	377
59.755	20	AACATGTTTGCTGGTGCTTG	59.764	20	209	3746	3954
60.408	20	GGCAAGCGAGTAGGTTGTGT	60.321	20	161	242	402
60.424	20	GGGGTTGTTAAAATGTCCACC	60.326	21	156	607	762
60.111	20	AAAAACCTTGGCAACAGTCG	60.147	20	223	58	280
59.656	20	ATGTTTCATCAACGGACACG	59.42	20	280	477	756
60.159	20	GGAGAAGACGGAGCTTGGTA	59.43	20	245	722	966
59.579	20	AATGAACATGCTCCTATTTTAATA(	58.22	27	280	735	1014
59.399	20	CCCAGTGAGGTTGTGGAGAT	59.962	20	130	1407	1536
60.307	20	ATGTGGCGACAAATTTAGGG	59.823	20	195	318	512
59.801	20	TGCATGGAGGAAATATGCAA	60.035	20	214	38	251
59.734	20	CCCCCTCTCTACACATTGACA	59.974	21	227	2892	3118
60.205	26	ATTCTGCATCAGGCCTTCAC	60.226	20	116	0	115
59.579	20	GGTGAGGTGAGGTGTGTGTG	60.045	20	157	395	551
59.986	20	ACCGGTTGACCCCGAATTAT	62.535	20	199	1244	1442
59.527	21	TCCATCTTCATGTTCCGACA	60.048	20	188	17	204
60.132	20	CTTGAATTATCGGCGGTGTT	59.96	20	153	143	295
59.871	20	TTTCACGCATTTCCACACAT	59.972	20	102	24	125
60.128	20	ATCGGTTATCACCGGATCG	60.697	19	280	73	352
59.587	20	CCCCAAGAACAGAACACCAT	59.82	20	112	1044	1155
61.096	20	GCGATTTCAATTCCGACTTT	59.162	20	279	1255	1533
59.962	20	CGGCCATGACTATCGTTTTT	59.96	20	242	181	422
60.265	20	TGCGCAAGTGTATTGTACCAG	59.807	21	259	2942	3200
60.21	20	CAACGCCATAGGGAAGTGTT	59.993	20	266	1562	1827
60.165	21	GGCAAATTGTGGGATGAGAT	59.756	20	212	86	297
60.413	20	TTAATTGGTTCGGGCTCATC	59.901	20	273	30	302
60.06	20	GACGTCGTCATCGAGACTCA	59.988	20	271	74	344
62.397	18	TCCGAAAATCAAAGGTCCAC	59.91	20	113	18	130
59.702	20	AATCGGAGTCTGTTTGGTGG	59.966	20	129	145	273
59.836	20	TGTCGCATGCCAATCACTAT	60.104	20	232	2549	2780
59.859	20	TCATTGAACTCCCCATCACA	59.893	20	251	1343	1593
60.012	20	CCTATGGTGGTATGGTGTGG	58.569	20	132	454	585
59.934	20	CACGAAGCATTGCCACTAGA	60.011	20	208	595	802
60.158	20	CATATCGGGTCAAAGCAACC	60.331	20	124	716	839
59.336	20	AAATCTCCACTTGGGAAATTTAGA	59.424	24	130	265	394
59.906	23	AACATTTTGGGTCTTTGTCG	57.578	20	190	1245	1434
60.812	20	ACATTTAATGCCCCAGCAAA	60.323	20	202	420	621
59.139	20	TGAACAGGTTGCATTTTAGATGA	59.639	23	254	2017	2270
59.955	20	CAACGGTACGTTCGGATTTT	59.861	20	212	519	730
59.97	20	CGACAAGAGAAAGAGGGTCG	59.982	20	142	393	534
59.14	23	AGAGCGTGGGATTGAGAGAA	59.95	20	226	900	1125
60.69	20	TGGAGACGGCTGTTTTCTTT	59.853	20	205	312	516
60.163	20	ATTGGGTGGGATAGTGCTTG	59.813	20	209	291	499
59.96	20	TAAGGGGCATTACCACATGC	60.721	20	218	490	707
59.799	20	GAGATATTCTGTCGGATAGGTGC	59.13	23	229	466	694
60.683	20	CACCGTTAATCGCAACAAAA	59.603	20	273	180	452
59.129	20	TTTGATGGGAATGGGAGGTA	60.126	20	114	15	128
59.778	20	CCTATGGTGGTATGGTGTGG	58.569	20	164	53	216
59.343	20	ATCAAAGCACCAGTTACCCG	59.993	20	279	182	460
59.894	20	TCTTCCTTTTCTCGTTCACATATT	58.4	24	221	198	418
60.309	20	TATGGGTTATCACCGGATCG	60.544	20	188	437	624
59.935	20	ACGCAGTTGCTCGGAAGTAT	59.904	20	265	39	303
59.282	20	TTTGATCCAATGGCCCTAAG	59.894	20	194	1	194
59.115	20	CTCAATCGTCGCCGATATTT	60.06	20	266	200	465
58.901	21	CACACCATGTGACGAAGGTT	59.441	20	271	627	897
60.088	20	TTTTGGTCACCGTGATTTGT	58.874	20	249	36	284
60.293	20	CACGATGAATTGTCGTGGTT	59.42	20	243	60	302
60.019	20	ATGCCAAATTGTGTTGGGTT	60.096	20	185	129	313

59.903	20	GATTGAAGATGTTGGGCGTT	59.939	20	167	1007	1173
60.16	20	ATTTCGAAGATCAACGGACG	60.074	20	214	2224	2437
59.727	20	TGGATTTTTGAAACGTGGAA	58.987	20	208	47	254
60.131	20	AAGCTTGCATTTTATCGGGA	59.682	20	220	248	467
59.973	20	GCAACAAAACAAATATTGGATCA	58.906	23	276	522	797
60.073	20	GTGCTGTTTTGGTTGGTGTG	60.049	20	244	480	723
59.307	21	CTGCCAGGACGAACTTGTAA	58.923	20	276	227	502
60.235	20	ACGTTGTAGTTGAGGCCCTG	60.171	20	276	1068	1343
60.081	20	TTCACGAATTCTTCTTTCCTTTC	58.919	23	276	951	1226
57.647	21	TCAACTCAATAGGTTTTCGAGTCT	59.732	25	198	8	205
60.199	25	ATTTGGCACCATTCTTCACC	59.797	20	266	331	596
59.769	20	GTCAGACGTCCTCCTGGTGT	60.161	20	108	39	146
57.201	23	CCGCGCTGTAGATTCAAAGT	60.407	20	124	40	163
60.149	20	GTGCACAGCTGAGACAGCAT	60.22	20	166	3909	4074
59.569	20	GGTTGATGGATCTGCGTTTT	59.939	20	240	154	393
59.938	20	AGGCTGAGATCTTTCCAACG	59.43	20	226	837	1062
60.134	20	CCAAATCCTTCGTTTCTTGG	59.541	20	271	105	375
59.528	20	GCCACATATGCAAAATATCAGC	59.486	22	280	163	442
60.104	20	AGGCCGAGGAGTAGGAAGAA	60.34	20	171	174	344
59.95	20	CAGGAAAAGCATCATCACCA	59.648	20	205	2039	2243
57.557	25	GACGTGACGAAAGTGGGAAT	59.973	20	196	11	206
60.314	20	TAAAAAGCGGCATTCGATCT	59.816	20	146	60	205
60.232	20	GTGGGGTGGGATTGAACTTA	59.647	20	272	23	294
57.395	25	AAATAGATGCATGAAACATTTTGA	59.012	25	280	28	307
60.281	20	AAGGAACAACTGGTGGATGC	59.973	20	201	1397	1597
59.837	20	TGTTGCGCGTGCTTTAAATA	60.401	20	155	246	400
59.831	20	AAAATTGCAAAGCGGTGTCT	59.752	20	246	2	247
60.142	20	TCCTAGCCCTCAATCTCGAA	59.91	20	236	251	486
60.014	20	AGATCCACCACTGCTTGGAG	60.261	20	217	280	496
59.704	20	TTGATTGTCTTTGCGTTGGA	60.234	20	227	127	353
60.081	21	ACGCGGACGGTACAGATTAC	60.022	20	189	171	359
60.155	20	GTAAAACGCTAGAAGGCCCC	60.095	20	124	9	132
59.952	21	AGGAACAATCCCAACTTCCC	60.169	20	248	273	520
60.074	20	TGATCTGGAACAAGGCAGTG	59.831	20	278	1276	1553
59.604	23	GGGTGTGATCTTGACCCTTG	60.363	20	216	473	688
60.461	20	CAAGCAAATCCCCTTCTCTG	59.807	20	141	849	989
59.542	20	CATGTTGCTTTTGCATGTGA	59.28	20	183	41	223
60.275	21	CAGATGGGGATTTGCTTCAT	59.894	20	224	2636	2859
60.461	20	CGACAGCGTAATTGAAGCAA	60.014	20	262	7	268
61.051	19	TCCGAAATATCCGGTTTTTATT	58.795	22	259	889	1147
58.456	24	AAGGGAGGGTTTCATTCTCA	58.568	20	277	54	330
59.229	20	CTGGCTCCCTATAGTGCTGG	59.853	20	266	1365	1630
58.613	21	CACATCCAGCATCAACCAAC	59.967	20	254	105	358
60.245	21	TTGCTGTAGGCCTTTTGCTT	60.018	20	117	157	273
60.238	20	GGGCAGCTGTAAAACCTTCA	60.249	20	250	154	403
59.485	20	CCTCCAGATTTGACAGGGAA	60.042	20	261	80	340
60.303	20	GAACGATTAGTGTAGGTTGGGC	59.898	22	177	348	524
59.938	20	ATGGCCTCTCCAGTTACGTG	60.134	20	129	138	266
59.875	20	TGTTGATTTATGGCGGATTG	59.377	20	173	1025	1197
59.976	20	TTTCTCATTGGCAGTCACCA	60.24	20	263	548	810
60.182	20	ACGGAAATCTAGGGAGCTGG	60.59	20	267	131	397
60.089	20	GTGGGTCCCTCTCTCACAAA	60.088	20	270	2739	3008
59.795	20	ATGAGTTATCACCGGATCGC	59.923	20	253	253	505
59.528	20	TGTTGTCGCTTTAGCTCCCT	60.015	20	267	430	696
59.547	20	GGGTTAAAAGAAACCCCCAA	60.027	20	233	257	489
60.067	20	ACAAAAATGCCCTCGTATGC	59.967	20	246	425	670
59.893	20	GGAATGGAATTCACTCCTAAAGC	60.316	23	223	2847	3069
59.445	20	GCCAGGCAGAGAGAAAGAGA	59.827	20	206	1142	1347

60.02	25	GGGTGGGATACAGCAAGAGA	60.073	20	106	44	149
59.101	20	TTGAAGAGGCAATTATCGGG	60.031	20	267	1677	1943
60.217	20	CGGCCCAGTCTAATTGGTAA	59.953	20	254	4	257
59.933	20	GCATTACGTGTGTGCGTTCT	59.794	20	268	51	318
58.536	20	TCAATCGAGCTACAAACGTG	57.506	20	108	16	123
60.192	20	AGGAAAGAGAAGTGAGCCCC	59.817	20	251	5474	5724
60.116	20	TTGTTGCATGTCATTGGTCA	59.516	20	216	268	483
59.467	20	GCGCATTTCTGATATCGTCA	59.799	20	159	675	833
59.226	20	TCGTGAATTATCCAAACTTTTCAA	59.885	24	155	414	568
60.032	20	GCGTTGGAGATGGTCTTACAA	60.125	21	146	546	691
58.595	20	CCAATGCGGATGCTCTTAAT	60.06	20	279	37	315
59.861	20	ACCGCCCTCCTATTTCGTAG	60.463	20	181	35	215
59.268	23	GGAGAGGGAGGAAAGACTGG	60.186	20	189	608	796
59.869	20	TCATGATCCCATGAACGCTA	60.033	20	252	194	445
58.897	20	AGGGGGTTATCACCTGATCG	61.095	20	111	448	558
59.586	20	TTTGATTTCTCCCCTACCCC	60.124	20	278	87	364
60.059	20	CCACCCGTTGATTTTTCACT	59.83	20	235	393	627
59.972	20	GCGTGCTGAATCTTAACCGT	60.278	20	141	1005	1145
60.006	20	TGGTTTCCATCGTTTGTTGA	59.941	20	143	451	593
59.003	21	GGCCGATATTTGCTCCTAGTT	59.606	21	272	145	416
59.972	20	AACACCTGGGTGAAACCAAA	60.246	20	164	312	475
59.542	20	CACCTAAAATATTGGTTTCTATTTC	57.121	26	139	281	419
59.562	21	GTCACGTGGCATCATGAGAA	60.698	20	199	336	534
60.02	20	TTGCCAGAGCAAAAACACAG	60.027	20	105	1622	1726
58.927	23	ATCTCAAACAGTGGGCTTGC	60.263	20	278	2836	3113
60.119	20	TGGCCCTTAGCCAGAATATG	60.053	20	165	916	1080
60.031	20	TCATGTACCCCGGATCTACC	59.629	20	280	828	1107
59.996	20	AAAAAGAAAAGAAAAGAAAGAAT	57.177	26	190	505	694
59.953	20	GCAAGTTGAGGGAGAATGGA	60.195	20	153	371	523
59.941	20	GTCTTTGGCTATGACCCACG	60.517	20	259	163	421
59.569	20	TGATTTTCATGTTTCGCGATT	60.45	21	216	81	296
60.112	20	GGACTCATACGAGGAAGCCA	60.218	20	196	2438	2633
59.956	21	CGTCATTGGAAAATAATGCG	59.029	20	218	65	282
59.407	20	GGAAAGAACTCAATGTCGGC	59.676	20	262	132	393
59.311	22	TTCACACAGTTCATCTCCCG	59.676	20	259	11	269
59.864	20	ATCTCCATTGCTGTTGGGAA	60.461	20	237	3106	3342
59.864	20	GGAATCCCTTGACACGAAGA	60.05	20	253	380	632
59.789	20	CCTATAGTGGTATGGTGTGGGG	60.365	22	194	180	373
59.2	20	GCAACCAAGGAGACTTGAGC	59.997	20	144	171	314
58.826	22	TGGCTAGCCTTTGAAGCAAT	59.982	20	149	24	172
58.667	21	CGTTGCTTCCTTCCTACTCC	58.934	20	204	186	389
57.904	20	TTGATGCACTCTTTTTCCCC	60.051	20	212	162	373
59.282	22	GAAGAGGGGGTATGAGTGGA	58.94	20	102	126	227
60.127	20	TGCGTTTTGACAATATTTAGTGTTT	59.109	25	188	281	468
60.032	20	TGCATATTTCTCGATCCCGT	60.435	20	249	5674	5922
60.11	20	TGAGCAAAACTGGTTCCCTT	59.711	20	269	966	1234
59.178	20	AGCAAGGTGTGTGAACGATG	59.751	20	274	525	798
60.014	23	TGAACACCCCTACTTGAGCC	60.111	20	185	0	184
59.534	20	GCGGATTGGATGCTTTAGTC	59.674	20	110	0	109
60.391	20	TCCGCACTAGTTCCACGATA	59.297	20	280	761	1040
59.817	20	GCCATGTTTACGATGAAATGC	60.345	21	205	487	691
59.96	20	CAACAAGATTCGATAGAAGCCAT	59.643	23	143	770	912
60.074	20	GATCTGCTGGCGGTCTTTAT	59.297	20	243	925	1167
59.469	20	GGCTCCTTGGATTCATCTTG	59.629	20	275	69	343
58.084	21	ATTCTTGCTGGCTGCTCAGT	60.164	20	125	9	133
60.147	20	GAGACGCGAATGTTGAGACA	59.992	20	210	3496	3705
59.793	24	CTTTGAGCTGATCTTGAAATGC	59.114	22	232	354	585
59.989	20	CTCCCTCTCTAAAGGGACGC	60.34	20	173	326	498

59.873	20	GCCAGTCTCTTTGGATCCTG	59.803	20	209	177	385
59.823	20	TGGTATCCATAGCAAAGCGA	59.281	20	249	158	406
60.365	20	GATTGACGGGATCACAGGTT	59.786	20	176	157	332
59.392	24	TTCTTACTGCCACACAAAATCAA	59.677	23	247	457	703
59.832	20	TGGTAAAAGTGCTCAAGACGA	58.566	21	277	93	369
59.136	20	TATTCCGCCGTGGTCTAAAG	60.089	20	267	145	411
59.304	20	CACTCTCCCTCACACACACG	60.358	20	143	1295	1437
60.124	21	CAAACTCGCTCAAAAAGACACA	60.453	22	222	1973	2194
60.008	22	CAAATAATGCTGCGTGATGG	60.096	20	200	11	210
59.934	20	AGCCCGATAAGTTCGCAGTA	59.867	20	204	257	460
60.483	20	CGGAACCAGACCGAATATGA	60.853	20	184	35	218
60.24	20	AGTCCGAAAATCCCAACAAT	58.36	20	270	271	540
60.138	20	TTAAAAATCGCCCGTGAGAC	60.074	20	267	3256	3522
60.022	20	ATCTTCTAATCACCGCCCCT	59.925	20	237	186	422
59.803	20	TTGTTTGAGGGAGAAGTGGG	60.081	20	220	3205	3424
59.795	20	TTTGGAGGATCGACGTTACC	59.933	20	260	67	326
59.846	20	TTGGATCATTCAAGGAATTTCA	59.388	22	218	918	1135
59.844	20	CGAATTCAAACTCTCCCGAA	60.184	20	266	14	279
58.44	24	TCACAACAGGTTTCGCCATA	60.111	20	199	142	340
59.624	20	ATGACTGCAAGCAACCTGTG	59.905	20	104	87	190
60.22	20	CTCCACCCTGGTTCTAATGC	59.55	20	275	937	1211
59.855	20	AATACCGGGAAGAATTTGGC	60.15	20	231	164	394
60.23	20	GACACGTTGGTGAAGCATTG	60.16	20	145	251	395
60.179	20	CCGCTATCGAAAATATAATCCC	58.9	22	272	112	383
57.122	24	TCCACGGGAGAGAATGAGAG	60.34	20	188	25	212
60.315	20	AAAAAGAGGGAGGGATACACC	58.452	21	274	59	332
59.928	20	TCACCCCACTCCTCACTTTC	60.088	20	116	283	398
60.02	20	GGAAAAGCAATCAAGATCCG	59.645	20	143	734	876
59.924	20	CTAAAATGGAAAGGGCGTGA	60.067	20	145	440	584
60.126	20	CTACCCGCAATGGTTCAACT	59.993	20	239	6672	6910
57.328	20	ACGGGTTATTACCTGATCGC	58.93	20	101	826	926
59.82	20	TTCCCAACTCCAATTGATCC	59.727	20	104	1119	1222
60.362	20	TCAACCTTTTCACACCACCTT	59.482	21	196	88	283
60.016	20	TACCCTTCATCTTCAACGCC	60.074	20	184	1341	1524
58.622	20	ACAGAAGGAGCAAAGCTGGA	60.134	20	262	621	882
59.942	20	TGTGCAGGGAAGATCAAGTG	59.831	20	191	7	197
58.898	23	GGGTGGAGTTATGGCTGAAA	59.933	20	235	150	384
60.328	20	CCATTTCAAGAATGCAGGGT	59.933	20	196	1510	1705
59.933	20	CGGTTCAACCCTTTTGTAGG	59.466	20	279	1497	1775
59.677	20	CAACACAAGAACTCACCTCCC	59.607	21	272	492	763
60.016	20	GGCCTAGCTGGGTCTTCTTC	60.349	20	187	5760	5946
59.942	20	TCTCGATCACTACAATCTTTGAGA	59.445	25	269	163	431
59.978	20	TGCCGTAACCTGTCATACCA	59.988	20	164	94	257
59.087	20	ACAAAGGCACAGAATGGACC	59.973	20	274	1449	1722
59.304	24	GAAGGAACGCTTACACGGAG	59.875	20	127	9	135
60.008	20	AGATGCTGGAATCTTCGTGG	60.218	20	191	20	210
60.081	20	TCTGTCACGTCTTCGTGTCG	61.092	20	177	254	430
59.662	20	CCTGGAACTGTCATCACCCT	59.962	20	264	1121	1384
60.15	20	TGGCTCTTCACCCTTGATTT	59.67	20	175	36	210
60.133	21	AGAAAGCCGGCAAACTGTAA	59.883	20	222	138	359
59.984	20	GCAGATGGCAGATTGGTACA	59.679	20	217	84	300
59.989	20	AACTGCCCAATCTGGATGTC	59.934	20	271	1139	1409
60.723	20	ATTTTGCAGTCCCTCCAACT	58.646	20	227	1192	1418
59.722	20	ACTTTTGGGGGACAAATCCT	59.668	20	226	265	490
59.844	20	GTCCATTGTAGCGGTTGGAT	59.82	20	250	96	345
60.197	20	GAAATGGCATTGATTGGGTC	60.141	20	226	2176	2401
60.096	20	TAGTCGCGTGCGATAATTTG	59.864	20	238	156	393
59.938	20	ACGTCCAACGATCATTCTCC	59.934	20	270	71	340

60.72	20	CTAAGGTCGGCGATAGCTTG	59.996	20	203	836	1038
59.894	20	CAAATGGGTCCATCACTCCT	59.779	20	184	308	491
59.997	20	TGCACGTGTGTAGGGTTTGT	60.072	20	271	1006	1276
59.972	20	GGAGGGCTGTGTGATATTGG	60.34	20	276	141	416
57.273	21	TGTTGCGCGTGCTTTAAATA	60.401	20	128	87	214
60.009	20	GGAACGAACCAAAAGTCCAA	59.948	20	271	509	779
59.853	20	GCAGGAGAAATGGAGACAGC	59.957	20	246	619	864
59.984	20	AGCAAGGTCTGGGGTGTATG	59.989	20	247	2105	2351
60.312	20	GCATCATCTTGTTGTATTCGTTG	59.532	23	223	2	224
59.864	20	TTTTTATGTGACACGCCCC	59.385	19	262	574	835
60.357	20	CCCTTGCTACCATCATCGTT	59.955	20	172	205	376
59.955	20	CTGTATTGTGGAAGGACGGG	60.366	20	241	266	506
60.096	20	TTGCAAACAATCCCATTCAA	59.907	20	235	1806	2040
59.993	20	GGGATGACTCCAAAAGGACA	59.903	20	237	169	405
59.829	25	TCGAACCTTGAACCAAAAGG	60.081	20	175	8	182
59.957	20	GCCTACGATCCAAATCCTGA	60.036	20	188	4143	4330
59.989	20	AGCCCCACATAATCCCACTT	60.578	20	254	56	309
59.956	27	TGTACCGCACCAAGATTTGA	60.111	20	169	1	169
60.021	20	TTGCAAGAAAAGCCACACAC	59.888	20	276	174	449
60.16	20	AAAAATTTAGTATGAGGTCACATC	59.925	26	235	657	891
59.134	22	AAGGGAGTTCTCCCAAATCAA	59.926	21	164	36	199
60.634	20	CATAAAGCCACCCCAACAAC	60.227	20	206	189	394
60.103	20	AAGGGAGCCCAATTGAAAAT	59.777	20	250	196	445
59.994	20	GAAAAGCTTGGCCATTCAAC	59.691	20	156	342	497
59.972	20	TCCCATTTGTGTGTTCTTCG	59.541	20	154	2458	2611
59.769	20	GGTAGGTGCTCTGACTTGCC	59.874	20	242	889	1130
59.953	20	GGAGGAGAAGGAACAAACCC	59.912	20	132	398	529
60.074	21	GAGCTTCAAGTCCCCACTGA	60.386	20	232	57	288
60.096	20	TGAGATTTGGACGGTTATGATTT	59.729	23	200	207	406
60.523	20	GCGAACTCGGCTAGAACAAA	60.523	20	116	695	810
59.694	20	CCCAATGATCAAAGACGAGG	60.452	20	272	201	472
60.119	20	CTGCAAGGGACAGCAAAATA	58.917	20	149	529	677
60.103	20	AGTATTTGCAGAGGCGGAGA	59.978	20	271	170	440
59.904	20	TTTCTGGTTGCATTTTGCTG	59.849	20	165	1610	1774
60.045	20	TCATTCTACAACTCATCCACATCA	59.499	24	267	266	532
59.96	20	GGTGTGCTCGGATTTTTGAT	59.939	20	161	279	439
61.113	21	CTATTTTCGCTGGCTTGGTG	60.762	20	279	210	488
59.74	20	TCCCATTTTCAGTGGAAACA	58.947	20	166	129	294
57.947	20	AAGGCGGTTTTTGATACGTG	59.996	20	275	346	620
60.096	20	TGTCTCAGAAACTGTAACCCTGA	58.898	23	260	2887	3146
59.945	20	TGAAAGGCAGATTTCCCAAC	60.051	20	204	189	392
59.973	20	GATGAAGATGACCACGGCTT	60.081	20	233	624	856
60.103	20	GTACGGTTGCATAAACGGCT	60.025	20	264	2458	2721
59.305	20	GGACACGTTGGACCTGAAAG	60.545	20	152	38	189
59.999	20	ATTTCGAAGATCAACGGACG	60.074	20	247	423	669
60.074	20	ATAAAAGCAAAAGACGGGGG	60.305	20	224	3215	3438
59.762	20	TGCCAGTAATTCACAACCCA	59.964	20	217	448	664
60.725	20	CAAGGGCTATGCAAATAAAAGC	60.114	22	276	83	358
60.544	20	TGGCAATTCTATGACCCTTG	58.572	20	135	22	156
60.132	20	CCGTTTTCCCCGTGAATAG	60.308	19	170	999	1168
60.021	20	TCTCTGTGTGTGAACCTGCC	59.872	20	271	795	1065
59.673	20	ACCATATTTAAGGGTTATCACCTG	57.552	24	167	773	939
58.464	23	TTGTGCATCGTGTTCTTGTG	59.298	20	127	0	126
59.913	20	GGGAAGGATGAAAGGGGTTA	60.124	20	104	177	280
59.869	20	TTGATGGTGACGTAGAACATTTG	59.912	23	166	44	209
59.482	21	CGTGTGTGTGGTGTGGTACA	59.95	20	146	65	210
59.939	20	AAAGTGCATGTGAACCTCCC	59.973	20	226	334	559
59.894	20	ATCAACACGTACGGAGATGC	58.599	20	240	214	453

59.813	20	TCATCTAGCATCTCTTGCTTTCA	59.261	23	184	390	573
60.074	20	GTGGAAGATTTCTTGGCAGC	59.82	20	193	756	948
59.847	21	CGACCACAAAATGGGAGAAC	60.353	20	152	1	152
57.432	24	ACGGCGTTAACTAACCTGGA	59.632	20	255	30	284
59.853	20	ACAATCCCCAGCTAATTCCC	60.152	20	273	334	606
59.972	20	GTCCCACCCAATCACATTTC	60.034	20	277	651	927
58.723	20	TATGTCGTTAGACGATGGCG	59.712	20	244	275	518
59.569	22	TAATGGGAATGGCTTGGCTA	60.415	20	277	909	1185
59.962	20	CGACTTGTCGTGGTTCGATA	59.716	20	276	44	319
59.394	20	TTCCTTCCCCACCTCTTCTT	60.045	20	232	854	1085
58.721	20	TTTCCTATGGACCGAAATGC	59.901	20	252	651	902
60.118	22	CGATTTGACCTCGGATAATCA	59.908	21	254	161	414
60.323	20	CATGTTATTCGCGTGCATGT	60.554	20	141	3658	3798
60.119	20	CACACCTCCTCCAAAGCACT	60.298	20	235	498	732
59.297	20	ACGGGAGGGAGAGGGAGTAT	61.214	20	111	371	481
59.278	21	GTTCTTGGTTGCCTTACGGA	60.11	20	275	138	412
59.853	20	GGTGAAGACGGATCGTCAAT	59.934	20	252	2605	2856
59.851	20	GAAGTGCATATGCGAGGTGA	59.83	20	139	734	872
59.933	20	GAGCCGTTGAATCCTTGTGT	60.119	20	265	28	292
59.864	20	AACCCGTTTGAGGTTGAACA	60.388	20	274	4	277
59.916	20	TCCCCTTGGTCCTAAATGTG	59.784	20	216	2372	2587
60.397	20	GTAAGCCAAAATCCTGCGAA	60.209	20	264	40	303
60.352	20	AAATGAAGGGTTGAGATGGG	58.836	20	204	2009	2212
59.375	20	TCATTTTACCTTTCTGTTTTTGGA	59.107	24	250	416	665
60.036	20	GGGACCATGATACATTTGGG	59.871	20	257	168	424
59.847	20	TTGTCAACCCTGTTGAGCCT	60.69	20	280	1856	2135
60.689	20	TCATGCAGGAAAAACCATCA	60.049	20	224	3338	3561
60.331	20	TGCATTTTATTTACGTGGTTACTTT	58.285	25	279	380	658
59.566	20	AATTTCGAAAATTTAAAGGTCCA	58.2	23	105	543	647
59.791	23	CATCGCATTCTCGATTCAAA	59.769	20	239	1492	1730
60.601	20	CACTGGCCAAGAATCAATCA	59.648	20	249	415	663
60.029	20	CCGTTGGATTTATCTTCTCCA	59.03	21	243	3503	3745
60.011	20	TTTGCTCGAATTCATATTTTTATGT	58.297	25	276	428	703
59.759	20	GTAGCCCGAGTCAGATCGTC	59.834	20	269	187	455
59.79	20	CCACCACACACAGGGTTATC	58.697	20	103	169	271
58.987	20	GATGTGTTTGGACGGTGAGA	59.525	20	147	815	961
59.656	20	TCCATTCCATTGACCCATTT	59.991	20	275	113	387
60.006	20	GATGAAAACGTGAGGCTGAA	58.85	20	259	137	395
60.16	20	TGATTTGGTTTTGGGGGTTA	60.024	20	251	12	262
59.967	20	GTGGCAGTAAAATTGTGGGC	60.374	20	243	80	322
59.978	20	CTACCACCTCCCATCACCAC	60.238	20	165	618	782
59.297	20	CAAGAACCCGGAAAGTGAAA	60.081	20	254	1581	1834
58.033	27	TAGCAGTGTTATCGATGGCG	59.856	20	177	446	622
60.132	20	ACTCTCCATTCCATCAACGC	60.081	20	249	286	534
59.844	20	TCCGAAAAATCAAAGGTCCA	60.418	20	236	458	693
60	20	GCCTTGTACGGTTGATTGGA	60.894	20	273	196	468
59.537	22	CAACTTGGTCACCTCCTTGG	60.536	20	111	66	176
60.193	20	CCCTGGATTTTGATTGAGGA	59.864	20	259	92	350
60.192	20	AGTTGTGCAGCCAGGATCTC	60.418	20	213	151	363
59.703	20	TGACGGGATTTAGGTGTTTTG	59.847	21	249	366	614
59.867	20	TTGTTTCGGTGTTGCTTCAG	59.881	20	265	357	621
59.499	20	TTGGGACACATTCACGAAAA	59.941	20	276	1994	2269
59.607	21	TGAGTTGGCCAAGAGAGTGA	59.545	20	224	342	565
61.035	20	GGAAAAATCAAACAAAAACACGA	60.238	23	257	1243	1499
59.148	20	GGCCCAAACAAAAATCTTGA	59.916	20	241	382	622
60.067	20	AATGCGATGTGAGGACATGA	60.08	20	239	1782	2020
58.374	21	GAGGCAGAGTAGGGGGAATC	60.037	20	137	22	158
60.041	20	CCTTCTGACAAAGGCGAAAG	59.986	20	198	2831	3028

60.169	20	TGTGCCATTTCTTGCACTTC	59.847	20	180	2250	2429
59.097	20	TTCTTCAACCGATCAATGAAA	58.201	21	278	844	1121
59.659	23	CATGAGATCCTGGGTAGTGGA	59.935	21	274	1521	1794
60.14	20	TGCCACGTGTCAAAATCTTA	57.763	20	154	757	910
59.5	27	GGGATTGGGATGAGAAGAGA	59.016	20	272	49	320
60.306	20	AGACTCACGCCCTCTCACTC	59.585	20	268	41	308
59.805	20	AATTTAGCGCAAACTGGTCG	60.264	20	264	190	453
60.298	20	GGGGATTTCAGACAGCAATG	60.461	20	239	1408	1646
57.188	23	CTCCGGTGTTAAAAGATGCC	59.569	20	260	2314	2573
59.84	20	CCCTCTTCTCCATCATCACC	59.46	20	226	134	359
60.009	20	CGATCGATTTTAGGGTTTTCC	59.795	21	197	773	969
60.005	20	CTTCTTTCCGCCTCTTTCCT	59.955	20	257	33	289
57.89	24	CATCAAGATCCAAGGGCTGT	60.073	20	201	4	204
59.954	20	CCGAACCCGATTACAGAAAA	59.931	20	196	16	211
60.344	20	TTTCAACAAACACACACGCA	59.765	20	274	505	778
60.05	20	AAAACCCATGGATGCAAAAA	60.167	20	275	468	742
60.067	20	TCACCCTTCGAGCTTAAAACA	59.869	21	183	361	543
60.583	21	CTTGTCTCCGTTCTGCTTCC	59.989	20	277	1245	1521
60.269	21	ACTTGCACTCCCTGTCACCT	59.756	20	208	966	1173
60.066	20	TGGAAACAAATCAATTGACCA	58.855	21	146	253	398
58.878	21	CACAGCAGTAGGGTCCAAAAA	60.154	21	156	7	162
59.097	20	CCCACCCCCAATATCAAAA	60.369	19	164	323	486
59.955	20	GTGGAGTTTGAAGGTGCCAT	59.973	20	241	561	801
60.043	20	GACCTGCGTAAGGTTCTTGC	59.882	20	194	2977	3170
58.973	20	CTATCTCATCCCCTCACCGA	60.027	20	104	28	131
60.022	20	TTATGGTGCTACTCCCACCC	59.813	20	240	132	371
59.971	20	CCAATCAACTAAACCCCCAA	59.657	20	222	1114	1335
60.002	20	AAAAGAGGAGGAGAGGCAGC	60.096	20	209	359	567
59.835	20	TGCAGTGGGTTTGATTGTGT	60.008	20	172	317	488
59.119	25	TTTTCAGTTAATGAGGTAATGCAA	57.967	24	199	22	220
59.951	20	GAAATTTTGGAGGCTGGACA	60.051	20	185	21	205
60.91	20	AAATGCCGCAGACATATCCT	59.556	20	246	14	259
59.942	20	TTGATTTGTATATAAAGAGGCTGG	58.367	25	276	2507	2782
60.16	20	GCAGCCTCCTCCTCTCTCTT	60.238	20	199	8	206
60.059	20	TTCTTCTAAGGTGCCCATCTG	59.322	21	247	2687	2933
60.21	20	GCATTGGATTCCTTTTATCCG	60.63	21	191	503	693
59.964	20	TACGCAGCCGGAGTAGTTTT	59.904	20	184	604	787
59.828	19	TTATGCCTATGTGGCTGCTT	58.414	20	280	550	829
59.844	20	TGTTAGGTGGTGGGGTTGAT	60.088	20	219	2	220
59.997	20	AGATCACCAAACCAACCAGG	59.82	20	270	1662	1931
58.325	20	TTGGTTTAAAAGTCGTTGTTGA	57.43	22	277	210	486
60.088	20	AATCTAACTAATGGCGCGGA	59.704	20	231	374	604
60.134	20	TCCAGCAGTTTCAATCACCA	60.24	20	239	1301	1539
57.557	27	GAAAAGGATTGGGTGCTTCA	60.051	20	123	4	126
60.046	20	CCTGCTCAAAGGGAGACAAG	59.982	20	144	153	296
60.049	20	TGTAATGGGTGCCAAAATCA	59.786	20	240	4666	4905
60.012	20	GGTGCAAGCCACAGGTTAAT	60	20	272	62	333
60.096	20	AAACAGTGCTTTTGTGATTCCA	59.654	22	146	885	1030
60.837	20	TGCAGATTTGTTGCACTTTTG	59.903	21	183	20	202
59.304	24	TGGGATAAATCTTCAACACCG	59.809	21	234	198	431
59.799	20	TTGTATTCGCAGTAGCCGTG	59.895	20	259	731	989
60.488	20	TTCACAGTTTTCATTTGATCACC	58.986	23	208	8	215
58.659	23	AGTGTTTTGACATGATTACTCGTC	57.373	24	247	1460	1706
60.081	20	GAAGCTAGCAACGAAATCCG	59.982	20	220	121	340
59.244	20	TTTATTAATCAATATAAGGTTGCA(	59.344	27	271	613	883
60.089	21	TGGTTCGAATCCTCCACTACA	60.501	21	255	94	348
60.297	20	GGAACAAAAGGAGAACATCCC	59.797	21	272	2162	2433
59.343	20	CTCTCATGTGCGTGTTTGCT	60.056	20	223	127	349

59.848	20	TAGAGCCCTGCCAAACAACT	59.875	20	275	11	285
59.805	20	AGGATGGCATTCCAGTTTTG	59.933	20	276	9	284
59.836	20	TGCATTGTCAAAGTAGTTAACTCA	59.027	26	259	241	499
59.978	20	ATTCGTGCCAAGTTTATGCC	59.967	20	230	128	357
57.507	23	TGAGTGATTTGAAATAAAATTTGA	59.694	26	270	3473	3742
59.805	20	TGGTGTTACTGGCGGTGATA	59.988	20	222	93	314
59.966	24	CGAGTAAACCAAACACGTGAA	58.737	21	191	3146	3336
59.615	20	TGATGTTGGAGAGAGGACCC	60.048	20	258	339	596
59.234	20	AGTTAAAATCGAACCGAACCA	58.616	21	206	344	549
59.964	20	ATTTCAACCTTTGCCAACGA	60.481	20	278	880	1157
59.964	20	TCCCCTTTCTCTCGCTGTTA	59.948	20	205	302	506
60.119	20	TCCTCACCACTGTACCCCTC	59.962	20	243	434	676
60.128	20	CGAGATGGGGACAGTTGTTT	59.966	20	158	260	417
60.384	20	GGACAGCCAGCTGAAGAAAC	59.997	20	237	1310	1546
59.964	20	ATAGGCAAATTCGGAGGCTT	60.061	20	227	194	420
59.823	20	ACGAGACCGAAAGCAACAAC	60.299	20	192	385	576
60.046	20	GAGTGGAGGAAGATGGGGAT	60.278	20	265	525	789
59.762	20	TTATTTGGAAGTGCAACCCA	59.013	20	108	1909	2016
59.535	20	CACTCCAACTTTGCAAGAACA	58.96	21	230	1013	1242
59.612	20	CTTCTGTTTACTCACGCGCA	60.195	20	186	12	197
59.42	20	GGAGGGAGACCATGTGAGTG	60.527	20	206	2414	2619
59.405	20	TTACCACAGGCTTTTGGAGG	60.103	20	253	444	696
60.132	20	GGTGAGGTGGCTGCATTTAT	59.962	20	168	140	307
59.721	20	TTTAAAGGTCCCTCACCCGT	60.714	20	229	1562	1790
60.075	20	AAATACACAATTGAACCAACAAT(	59.589	25	120	618	737
59.988	20	ATGTCCGCTCATACTTTGGC	60.103	20	176	196	371
60.25	20	TCCCTCTCCTCACCACACTT	59.682	20	266	536	801
59.789	23	GGGCGAGCAATCTGTAAGTC	59.843	20	262	183	444
60.544	20	TCCATTCCGAAAATCAAAGG	59.872	20	271	49	319
60.44	20	ATTGCATGTGCGAAAAGGAT	60.478	20	232	487	718
57.953	24	TGTGCAGGGTTGAATCTGAA	60.24	20	251	197	447
60.088	20	AACAAAACCAGGCACACACA	60.049	20	272	595	866
60.218	20	TTCAGACGAAGCGATCACAC	59.992	20	165	2	166
59.975	20	GGGCAGGTGGCTAATCACTA	60.096	20	139	1192	1330
59.989	20	TGCATGCTCACGCAAAGTAT	60.432	20	273	1895	2167
59.79	20	TCAAAATTGGGGAAATGACC	59.599	20	250	385	634
60.366	20	CCACGTGCTCCATACTTCCT	60.134	20	247	481	727
60.195	20	AAAGCCGACAGACAACAAGG	60.291	20	241	143	383
59.381	24	TCCGAAACAAAAGGGACAAA	60.452	20	167	38	204
58.836	22	CTCCATCCTCTTGCTTCGAG	60.088	20	219	1101	1319
60.204	20	ATGTGAAGAAGCTGCTGGCT	60.164	20	233	470	702
60.328	20	CCTACCTGCAATTGGACACA	59.566	20	262	4399	4660
59.935	20	AAATAGGCCCTTTTGCGTTT	59.97	20	150	1671	1820
59.363	25	AAAAATGAGTGGTTCATATTGGTT	57.968	24	134	719	852
59.691	20	CTTTAGGATCCCTTGTCCCA	58.978	20	226	268	493
58.215	20	CCCTTTGAAAGTTGATGGTTTT	59.39	22	266	202	467
60.074	20	TCGAGCAACATCTTGTGGG	60.823	19	135	839	973
59.748	20	TGAGATCACGAGGTCACGAG	59.98	20	192	437	628
60.882	20	GTTTTCAATCACAGCCACCC	60.362	20	127	170	296
58.475	20	CCAATTGTCCATATTTCGCC	60.153	20	204	347	550
60.01	20	TTTCGAGCACATTTTCTTCTCA	59.996	22	262	10	271
59.744	20	AGAGCATATCCAATGCCAGG	60.059	20	241	678	918
59.933	20	TGGAAAACAATGCCTAACAGC	60.124	21	247	143	389
59.377	20	TCCTACATTATTTGGGGATTAACA	58.735	24	135	10	144
60.118	20	CCACTGCACCACGAGATATG	60.136	20	189	6	194
60.035	20	CATCACATTCTCCAGTCCCC	60.326	20	252	774	1025
59.092	25	TGCAGTCGATATTTGCTCCT	58.477	20	120	1	120
59.997	20	CCCTTCTTTCCACAGCAGTC	59.844	20	279	25	303

58.172	21	TCTCGCGGTTTTCAGTTTCT	59.993	20	208	212	419
60.949	20	TGGCCTTCCTCAATCTATGG	60.029	20	146	2	147
60.991	20	TTGCAATTGAGACCGCTAGA	59.566	20	249	365	613
59.544	23	GAATGTTCAAGGCCATGGTT	59.797	20	227	133	359
59.172	21	TCGATCACACAGAAAACACGA	60.294	21	101	14	114
60.073	20	CTTCACTTCAAATGCCCCAT	59.933	20	275	2123	2397
58.161	22	CGACCAATTACACAAATCAACC	59.248	22	280	95	374
59.805	20	TGCGTTTTGACAATATATAGGTGT	60.083	26	170	306	475
59.967	20	GGCATGTGTGGGAGAAGAAA	61.046	20	171	7	177
60.126	20	TGCCAGGGTTATCTTGATCC	59.894	20	279	90	368
59.935	20	GGACAAGAGGTTGGGACAAA	59.943	20	122	170	291
59.91	20	AACTTTGGCGTTCTTGTGCT	59.92	20	108	955	1062
60.043	20	TGTTTGGGGGTGTTGAGTTT	60.246	20	182	2717	2898
59.526	20	TTTGCACCTGAGGAGGTGTT	60.69	20	131	1120	1250
57.733	24	TCCATACTCAACACAAGTCGAA	58.283	22	280	367	646
59.181	21	GTCCAGCTGCTCTCACACAA	60.187	20	173	3890	4062
60.082	20	TTTTGATTGCCACATTTCCA	59.907	20	248	691	938
58.89	21	ATAATGATATTTTGGAACGCATC	57.16	23	225	615	839
59.92	20	TTCACGAGTGGTGACAAACG	60.757	20	191	277	467
60.274	20	AATAAGGGTTTGGGCAGTCA	59.429	20	214	197	410
60.103	20	CCATCAAGGAAAGACGCATT	60.074	20	208	386	593
60.081	21	TCTGCCCTAAGATGAAACGG	60.206	20	178	99	276
59.82	20	TGAATCAATCTTGTGCGTTGA	60.25	21	183	19	201
57.09	25	TCGCCATAATTGACTGCAAA	60.215	20	108	18	125
60.162	20	CTGCAGTGCCTTTGGTAGTG	59.51	20	225	141	365
59.931	20	CACGTGGCATGATAATAGCG	60.118	20	213	390	602
59.005	24	GATGATCGGAACGTGAAGCTA	60.228	21	210	686	895
60.344	20	TCATCTTCTCCACCAAAGGAA	59.656	21	278	1231	1508
59.973	20	GCACGCAGCGAATAACAGTA	60.044	20	177	1108	1284
60.11	20	TGTTGAGACCAGGCGTACAG	59.897	20	216	180	395
60.553	20	TATCATTGTTTCGCGAGCAG	59.976	20	242	206	447
59.942	20	TTGATTAGAAAAATGCGCGA	59.424	20	175	97	271
60.237	20	CAGCTGCAGAGATGGAATCA	60.096	20	254	489	742
60.218	20	CGCGTTACGAATCCAAGTTT	60.131	20	177	344	520
59.28	20	ACTCCATGGTGCATTTCACA	59.967	20	264	4	267
60.209	20	TGGGTCACTCCTGTTTAGTGAA	59.633	22	276	14872	15147
59.971	20	TTGGCCATGTGAAAATCAGA	60.049	20	205	175	379
60.822	20	GCTCGGATATGTGGTAATCAAA	58.969	22	270	226	495
59.871	20	CGAATTCAGCCAGAACAACA	59.84	20	171	20	190
59.666	21	GGCCTAGGCACCAAATCTTA	59.182	20	188	1590	1777
59.96	20	ACCCACACCCTGTACCCATA	59.966	20	172	13	184
60.002	20	CACCATACAAGCATTCAGCG	60.28	20	253	284	536
59.978	20	CTTTCAATCGTTGATGGGGT	59.79	20	255	1153	1407
59.788	23	TGATGCAACCTTGCTCACTC	59.992	20	265	1459	1723
60.168	22	TGGTTTTATGTTTCTCTTTGGGA	59.865	23	248	1531	1778
60.475	20	AGAGATGCCGGGGAAGTAAT	59.925	20	227	144	370
60.073	20	TCTGTGAATCAAGCTCAAGCA	59.731	21	252	135	386
60.318	20	TTTCTCTCTTTTGGTGGAAGAAA	59.398	23	257	213	469
59.711	20	ACTGTGAAAGAGACCTCCGC	59.455	20	250	1095	1344
60.073	20	TATGGATCTTTGGCAGGTCG	60.992	20	240	195	434
59.941	20	TGGGAGTCTGTTTTCCCATC	59.903	20	100	178	277
60.24	20	CCCTCATAATGTGTGGCTCA	59.522	20	271	63	333
59.898	20	GGAATCTAGAACAGACCAATCCC	60.196	23	179	1339	1517
59.297	22	TGAGTCCATGACCCAACTTTT	59.439	21	175	753	927
60.051	20	AGAGATCCGTGCTTCACTTG	58.029	20	133	1469	1601
59.862	20	AAGGCGGCTAAATCAATCAA	59.682	20	268	283	550
59.064	20	CCTTGCACATTATCTGGAACAA	59.996	22	263	211	473
58.782	20	TTTCCCCAAGCTTCCTACAC	59.17	20	275	465	739

59.953	20	ACGCACCCACACTGTCAATA	60.032	20	135	60	194
58.054	22	GTGCGTTGTGTTGACGTTG	59.768	19	271	2325	2595
60.081	20	TTAGCATTTGACTACACTCCTCAA	58.151	24	186	272	457
59.914	20	TGCTGGAGCAGTCATTTTTG	59.988	20	140	1043	1182
59.269	20	TCCCGAAAATAAAAAGAAAAAGA	59.552	24	111	339	449
59.985	21	GATAACATGCCATCACACGC	59.963	20	269	873	1141
60.074	20	GGCTACTTGGCCCCTATGA	60.044	19	272	199	470
58.223	23	TGAACAGCCATCGAGCATAG	59.972	20	259	1211	1469
59.887	20	TCGGGTTAGGAATTTCATCG	59.894	20	272	1284	1555
59.842	20	TGTGATTTCATCCATTCGGA	59.859	20	227	262	488
60.64	20	CACACAATTGAGCACACACG	59.778	20	103	182	284
59.636	20	CCATCCACCACCGTCTACTT	59.844	20	252	72	323
58.976	22	ATGCGCAGATCTAATGGTCC	60.066	20	198	9	206
59.302	21	GGGTTTGCCTTTAGCCTCAT	60.451	20	239	131	369
60.43	20	TGGCCCTTTTGTACACTTCA	59.17	20	132	316	447
60.488	20	GTGCAGCACTAGGTCCATTG	59.318	20	277	127	403
59.587	22	ACGGGAGGTTAAAAATCGCT	59.966	20	193	24	216
59.814	20	GCCGCATTTCTTCTAACCCT	60.582	20	235	950	1184
58.474	21	AATATTTGCATGGCTCTGGG	59.923	20	274	1266	1539
59.973	20	GGGGCGTATAGACGCAAATA	59.95	20	168	638	805
59.912	23	GGCATCTTGGAATCACCAGT	59.934	20	172	1201	1372
60.31	20	TGGGGTTATTAGTGGATCAAGC	60.204	22	274	872	1145
59.255	22	TGCACCTCCTCTTCAACAACT	59.899	21	257	1022	1278
60.025	21	TGTTGCGCGTGCTTTAAATA	60.401	20	139	349	487
60.659	21	GAGCAAAGTGTTGAACTCTCGAT	59.942	23	121	7	127
60.088	20	AGAGAGAAAGTGGCAGTGGG	59.45	20	275	13	287
59.962	20	TCGCCGTGTTTGTATTCTTG	59.729	20	256	816	1071
59.989	20	AAACGCGCATTTTTAGGATTT	59.986	21	274	356	629
60.723	20	TCAGCTCGGGAAGAAAACTC	59.55	20	164	609	772
60.12	20	CGCTATGTGACCATCTGTGG	60.136	20	191	5217	5407
57.546	24	GTGCGTTCCACGTTAGGTTT	60.037	20	260	54	313
60.674	21	CGATTGAATTGGTGGGTTTC	60.17	20	170	78	247
59.827	20	TTTCTTCTCCCTCCCTCCAT	60.008	20	239	1548	1786
59.851	24	GTTAGGGCAATCAAGCAAGG	59.708	20	138	163	300
59.875	20	GGGCAAAACTGTAATTGCTCA	60.124	21	256	4053	4308
60.292	21	CACGAGATTTACGTGGTTCG	59.195	20	108	132	239
60.261	20	CATGGAATTGAAACTTTCGGA	59.924	21	156	16	171
60.604	20	CCTAGTGGCTTCCCCACTAAA	60.48	21	252	351	602
60.074	20	CGAACCATCGTCTCGTATCA	59.673	20	169	289	457
59.848	24	GGGGAATGGAAGAATGAGGT	60.133	20	244	1032	1275
59.311	23	TTGGCAAATTTTCTTGCACA	60.229	20	158	1028	1185
59.939	20	TATTGAACTGCCTTTCGCCT	59.845	20	193	113	305
59.542	20	AAACAGACTCCCATGTTCGC	60.119	20	280	153	432
59.933	20	AAGGGCTTTGTTGGTTTGTG	60.008	20	272	1049	1320
59.872	20	TTCATTCTTTGCATCCCTCC	60.014	20	111	3811	3921
60.05	20	GTGGTCGGAGAGTTGAGAGC	59.993	20	214	382	595
59.985	20	ACTCGAGCTGCAAAAACCAT	59.882	20	268	168	435
60.486	20	GCTTCCGCATTTTCTTTTTG	59.832	20	270	992	1261
59.853	20	CAAAACCTGACACGAACACG	60.19	20	271	850	1120
60.498	20	CTGAGGATAAAGGTGGCGAA	60.206	20	171	43	213
60.504	20	CCGGAAAACACCATCAATCT	59.79	20	279	581	859
59.521	20	CTCTCAGGGGCAGATACAGG	59.82	20	136	252	387
59.934	20	TGCAGTTGGTGGTAACCTCA	60.152	20	154	602	755
59.899	21	TGTTCTCGTATGATTACGCTCC	59.252	22	269	1024	1292
60.005	20	AGCAAGAAACCAGAGGCAAA	59.993	20	183	156	338
60.153	20	TTGTTATTGAGAGGGCAGGC	60.214	20	168	61	228
60.202	22	GATTGATGAGGGTTCCAAGC	59.488	20	253	94	346
57.278	21	TTATGCTGCCGTGTGTTTGT	60.18	20	228	0	227

59.375	20	CACACCTAGTGTGCTATCATCAAA	59.223	24	209	75	283
60.065	20	ATTTCCCGGAGAGGAGAAAA	60.011	20	210	1061	1270
60.146	20	CCAAATTGCCCTTCTGTGTT	59.971	20	265	454	718
58.658	21	CCAAGATTTGATCATGGCGT	60.864	20	272	584	855
59.089	24	ATAACATAATGGTTGGCGGC	59.688	20	125	19	143
60.051	20	AACCGTAAGGTAGATCCGGC	60.34	20	211	503	713
60.058	20	GTATTCGCTCTGCCACAACA	59.871	20	249	2574	2822
60.05	20	TACTCCTGCTCAACCTGGTG	58.879	20	132	378	509
59.955	20	CCACACCCTCTTCCTCTGAA	60.229	20	167	416	582
60.066	20	ACCAGCCTAGGGTTGAGGTT	59.994	20	250	1437	1686
59.049	21	TGTAAATCTGCCCGAAAACC	59.938	20	167	36	202
59.848	20	GGTTAACATTTCAGCGAGGC	59.713	20	185	0	184
59.93	19	AGGTTAATGAACGCAACCCA	60.365	20	119	66	184
60.73	20	CGTCCCATAAAATGTGACCA	59.247	20	241	824	1064
59.43	20	TGGTGTACAGTAAGAGGCAAACA	59.724	23	239	664	902
58.671	20	AAATCCTGCAATCATCACCC	59.756	20	234	259	492
60.036	20	ACGAAATGAGGAAATCGACG	60.074	20	254	67	320
59.691	20	ACCCGGACAATGAGCAATAA	60.331	20	199	276	474
59.883	20	TAATCCTCGTGGAAAATCGG	59.894	20	205	411	615
61.071	20	TGAACCAAACACCCGTGATA	59.816	20	244	87	330
60.032	20	TGAAATAAACATTGCCACCG	59.42	20	274	995	1268
60.042	20	CAAATCATGTGCGCTGAAAT	59.694	20	266	5404	5669
60.066	20	AGTTTGGTTCTGGCTCATGG	60.111	20	197	138	334
59.973	20	GTCAAGTCGACCACCCTTGT	60.009	20	123	30	152
59.452	20	TTACGAGGAGCAAGGTCGAT	59.836	20	191	385	575
59.985	21	CCAATTTGAATGGTTATCACCTG	60.457	23	272	1210	1481
60.103	20	ACGAGATGTGGAAGCCATTG	61.067	20	158	270	427
59.979	20	TCCTTCCCTGCATTTCATTC	60.014	20	214	1958	2171
60.005	21	TCATTTCAAAAATCAATGGATGA	59.291	23	193	2372	2564
59.917	20	GTTTTGTAGCTCTCGTCGGC	60.022	20	249	995	1243
60.252	20	TTCTTGGTTAGCTGGTCTGGA	59.861	21	224	1899	2122
59.931	20	TTTTGAACAATGATACCTTCTCC	57.314	23	215	36	250
58.694	22	TCATGGTGCTTGAAATTGGA	60.049	20	169	13	181
60.292	21	AGTTATGCTTGGACCGGTTG	59.993	20	277	146	422
59.764	20	GCTACGAACTACGGGGTGAA	60.132	20	259	82	340
60.698	20	GATTGGGATGGGCTAGGAAG	60.787	20	280	7	286
60.011	20	CTTACCACCATTCACCACCC	60.088	20	240	718	957
59.645	20	TACCAATGCCACAAAAACGA	59.969	20	254	79	332
60.533	20	GCATCACAAATTCCTGCAAA	59.67	20	276	185	460
59.867	21	CAGCTCTCATTATTTCCGGC	59.807	20	203	914	1116
59.585	20	GAGTGGAACTCGTCTTCCTCAG	60.427	22	217	776	992
59.111	20	TCACGATCGAACCACGTAAA	60.111	20	165	1440	1604
57.827	21	ACCTCTTTTCGGTTTGATGG	59.028	20	164	232	395
59.277	22	CGAATGATCTGGTGATGGTG	59.918	20	202	178	379
59.713	20	CAATGGATTAGGCCGAAAGA	60.031	20	177	1115	1291
59.871	20	ACCTGGTCTCCAAGTGATGG	59.962	20	243	2463	2705
60.038	20	GGGAATCAAAACCATTCCCT	59.996	20	280	393	672
59.809	21	GCCTGCCTTGGAAACTATTG	59.708	20	274	117	390
59.813	20	AAACTTGCGGGAATGAGTTG	60.11	20	228	637	864
59.951	20	GCTGCAGATCTAACGACCAA	59.028	20	130	368	497
59.679	20	TGGTAAGCGATTAACGGTCC	59.96	20	273	384	656
60.31	20	TTAGACGTGAATTGCAAGCG	60.014	20	272	181	452
59.994	20	AGGCCCATCATACCACAGTC	59.81	20	201	921	1121
59.998	20	CATTTTGTGGCCTGTGTTTG	60.004	20	206	2212	2417
59.934	20	GAGCTCACCGGTTCTTTTGA	60.375	20	184	134	317
60.015	20	CAACGATGTGCTAGTTGTGGA	59.773	21	221	4	224
60.051	20	AACACAAAAGGGCTCATTCC	59.031	20	221	93	313
59.95	20	AGAAGAGAGAGGGAGGGTCG	59.945	20	251	480	730

60.066	20	TTACGGCAATTTGCATCAGA	60.215	20	139	356	494
60.153	20	ACTTTTCACGGACTGCCAAC	60.156	20	132	1996	2127
59.895	20	GCCGGACTTGAAAATTGAAA	60.053	20	266	974	1239
60.34	20	TTGTCCTTTTGGGAACCAAG	59.942	20	226	471	696
57.662	20	TCACACACACGCACAAAGAA	59.912	20	107	74	180
57.628	22	GTTCGCCAAGAAACTTACCG	59.747	20	258	691	948
59.84	20	TGAAAAATGACGCCAAAATG	59.538	20	154	134	287
59.939	20	TCGAGTGGGACCAAAAGAAC	60.088	20	219	624	842
59.853	20	TGCAAGCATCAAAAATGGAG	59.809	20	216	759	974
59.191	27	ACCAAGTCATACATCATGTTATCT[illegible]	58.868	27	222	2	223
59.99	20	TTCTCACAGGATTTAGGTGTTCC	59.513	23	246	2	247
60.504	20	CTCGATGAGGAATGGGAAGA	60.15	20	278	69	346
60.523	20	TTTGTGTGATTTTGACCCCA	59.792	20	280	122	401
58.065	25	ATGGCGATATTGAAATGGGA	60.117	20	171	4	174
60.003	20	CTATCGTTTTCTCCGCCTTG	59.839	20	199	326	524
59.969	21	AAAACCGAACCAGGTCGTTA	59.471	20	225	231	455
59.654	23	ACGATTGTTTTGAATTCGCC	59.945	20	216	12	227
60.042	20	GTTGCTTCTTTAACGCTGGC	60.025	20	268	1016	1283
59.933	20	TGCATACAACACGCAAATGA	59.716	20	144	2397	2540
58.928	21	TTCGGAATTTATAATATGGGCA	58.4	22	276	99	374
58.836	24	GCTCGTGTGTTACAGGGGAT	59.997	20	249	703	951
59.03	27	AAATATGCCACCTATTGCCG	59.816	20	263	15	277
57.817	25	GGGTGGTGATATCTCCCTGA	59.737	20	270	19	288
59.901	20	ACGGCCCTTTCTCTTGAGTT	60.247	20	279	68	346
60.137	21	ACCATGGCGTGCAAGAGTAT	60.547	20	220	169	388
60.486	20	AATTAGTTGAGGCACGTGGG	59.993	20	233	207	439
60.235	20	TTTGCATCAAACATGGCTTC	59.67	20	253	1226	1478
59.901	20	TTGCGACTCAACACCAGAAC	59.88	20	196	454	649
57.557	25	TTCGATCTTCTTTCCTTTCCA	58.89	21	104	8	111
60.523	20	GTACAGGGTTCTCCAACCGA	59.966	20	177	138	314
59.96	20	GGTGGCCATTGATAGGAGAA	59.894	20	204	5491	5694
60.615	20	AAAAAGGGATTATCACGGGG	60.013	20	245	892	1136
59.367	20	GCGTTTTGGTGGATGAATTT	59.807	20	279	471	749
59.912	20	GCCATGCTCTATGGCAAACT	60.243	20	111	1149	1259
59.957	20	GGATTTTGATGTATGAGTTTGCAT	59.297	24	235	492	726
57.604	24	ATCAATCACAACCCCGTCAT	60.057	20	188	0	187
60.088	20	GGGTCGGGCTAATAGGCTAC	59.951	20	167	212	378
59.795	20	TCATATGGGTTGGTGTTGTCA	59.682	21	178	183	360
59.295	23	TGTGTTTTCAAAAGCCTAGACA	57.63	22	227	217	443
59.823	20	AGATCGCGGGCAAGTAGTTA	59.867	20	106	9	114
59.478	20	CCTCAGCAAAATGCAGTTGA	59.988	20	100	197	296
60.591	20	CTCACTAACTCGCACCACGA	60.049	20	264	101	364
59.888	20	TTTTGGAGGAGGCAAAGAAA	59.795	20	208	512	719
58.461	23	TGTAATAACCCTTATTTTCGTGCTC	59.834	25	265	69	333
60.252	20	TACAAAGTCGCCGGAAAACT	59.747	20	143	482	624
59.964	19	TCAAACCTCTTCCATCACCC	59.903	20	272	2135	2406
59.964	20	TTGCCAAATGGTGTAATGTCA	59.841	21	207	429	635
60.132	20	TTGACTCACAAACACACGCA	59.912	20	213	374	586
57.14	22	TCAAGGTGTTGATGCTTGGA	60.24	20	201	5	205
59.64	20	TGTGAGAGACGTCGTGTGTG	59.454	20	226	485	710
60.06	20	GCAAGAGCTTAGAACGGGTG	60.015	20	159	3004	3162
60.192	20	GTACGGCTGAGCCTGATTG	59.41	19	191	676	866
60.352	20	TTGGACAGGCTCCTTTATTTG	59.211	21	279	409	687
60.306	20	CCAGACATTTGTGGTGGATG	59.806	20	187	185	371
60.032	20	GTTGGGGTTGAGTTGTGCTT	60.012	20	194	570	763
59.836	20	ATTAATCCGATGGCCCTCAT	60.499	20	208	618	825
59.955	20	TCTAGGAAAGGGCGAATCAA	59.778	20	252	214	465
59.215	20	TGTTTTCTTTTACCTGTATTCAAGA	58.442	27	250	225	474

60.065	20	CGCCATAGCTTCTTGGTCTC	59.978	20	143	197	339
60.21	20	GAAAAGGGGAGGATCGGTTA	60.258	20	156	355	510
59.964	20	CGGATCACCAGATCCGATAG	60.439	20	202	520	721
60.263	20	TGAAAGAATATGAGTTTGAACGG/	60.004	24	189	100	288
59.123	20	CCAACCAAATCCGGTTACTG	60.22	20	266	62	327
60.011	21	AGGCCTTGCTCTCTCTGTTG	59.745	20	278	63	340
59.982	20	TTTCTGGACCACTTTCTGGG	60.081	20	169	4	172
60.132	20	TTCGGGGTGAGAACTAGGAA	59.665	20	275	1291	1565
60.011	20	TGAGTTGGTGTGTTGGCATT	60.008	20	164	394	557
59.647	20	CGGTTGACAGCCGATTTTAT	59.96	20	280	557	836
59.894	20	TGGAGCATTAATTTGGAGGG	59.894	20	124	649	772
59.962	20	TGCTCAAGTTCTTGCCATTG	59.988	20	215	554	768
59.997	20	CCATCATCGTCCACATCTTG	59.918	20	270	659	928
60.244	20	GGTAGCCTGCTGATGCAAAT	60.243	20	250	121	370
60.511	20	TGTCAAAATATGGGCATCCA	59.745	20	276	611	886
59.993	20	GAAAGTTTGGGGTGGGTTTT	60.068	20	206	961	1166
57.824	20	TTTCATATATCACGTTAAGACATG(	57.297	25	234	410	643
60.142	20	CCAAACGTATATGCCCTGGT	59.708	20	159	159	317
58.187	20	GCGTCGTTTCTTGAATTAGGG	60.962	21	206	1271	1476
60.053	20	ACAATACACGCCACACAAGC	59.646	20	220	1279	1498
60.008	20	AGCATTAATGGTGCCCAAAT	59.301	20	245	228	472
59.901	20	GCGTTTTGATGATATATATGTGTTT	59.956	27	198	971	1168
59.986	20	TAAGATCAAGGGCAACCTCG	60.206	20	244	2961	3204
60.964	20	TGAGAAAAGGTTGTACATGGCA	60.534	22	221	72	292
59.851	20	ATGTCCAAAGCTGGCAGAGT	59.874	20	137	576	712
59.923	20	ATGCATGCACGTGAATATGG	60.375	20	140	321	460
60.617	20	TTTTCATGTTTTGGAGCATCA	59.147	21	241	301	541
59.478	20	TCCCACCATTCACATCTTGA	59.893	20	255	482	736
59.916	20	GCATTCAAGATTCTGCCCAT	60.043	20	255	1550	1804
60.05	20	GGTTATCACCTGATTTCCTCCC	60.901	22	210	595	804
57.425	21	AATCCTTATGCCCCCAATTC	59.984	20	234	12	245
60.451	20	ACCCGGGTTTCGAAGTAGAC	60.361	20	190	1396	1585
59.688	24	TCCTTGCCAACAAGATAGTGC	60.264	21	248	2581	2828
60.646	20	CATTCGGTTTTCACTGGACA	59.541	20	276	45	320
60.074	20	TCGAAGGATGTGTTCATTGTG	59.556	21	260	1898	2157
59.937	20	AGTTGGGACGGAGGGAGTAT	59.817	20	142	339	480
59.966	20	GCACCATAAAGTTCAACGAACA	60.039	22	211	318	528
60.11	20	CAACAGGTCTCACCAATCCA	59.52	20	272	836	1107
58.642	20	TGACGAGTTAGAATGGCAAAGA	59.883	22	107	13	119
59.621	20	TCCAAGCTGACCAGTGTCTG	60.022	20	173	3756	3928
61.031	20	GCTGATCTTGGCCATGCTAT	60.207	20	172	38	209
59.598	20	TGCATATATTAGGGGAAAAATAA/	58.877	27	277	896	1172
60.209	22	TCCTTTAACAAATTGGTGTAGTCA/	59.056	25	195	216	410
60.443	20	TTGTGAATCGCCCAAATACA	59.93	20	222	802	1023
59.819	22	TGAGAGGTCTAGGGTTCGGA	59.797	20	243	642	884
59.786	25	CATCCGCCTGAGATTTTCAT	60.036	20	119	3	121
59.943	20	AAATGTGGATAGCAGAGGCG	60.235	20	164	47	210
60.151	20	GTCCACGGTGGTTTGAGAGT	60.009	20	151	41	191
57.162	22	TGTATGACATTTGGCTCCGA	60.073	20	227	499	725
60.011	20	AATTCCAGCCATGTAGGTGC	59.962	20	241	756	996
60.181	20	ACCCGATAATACCATAAAAGCA	57.629	22	265	1163	1427
60.05	20	TGCCCACTAATCTGTAAAGGG	59.098	21	184	927	1110
59.429	20	GCGGTAGGTAGCTCTTGTCG	60.037	20	201	170	370
59.848	20	TCCGTGAACCGTTCTTCTTC	60.232	20	233	476	708
59.823	20	GATTGTAAATCGGGCAAGGA	59.901	20	273	1558	1830
58.253	20	TCCGTTCATTCAACGAGGTT	60.495	20	280	50	329
60.278	20	AATTCGGGTACCATCGACAG	59.813	20	221	719	939
59.813	20	TCGGAAAAACTCCTGAGTGAA	59.838	21	182	380	561

60.66	20	TGTGGTGTTGACGGTGATTC	60.426	20	172	130	301
60.044	20	CCTGTAGCCATGTGATTGTTG	59.061	21	258	413	670
57.477	26	GGGAACATGCTTCCTGAAAA	60.051	20	158	7	164
59.907	20	CGTTCTTTCTGATTCGCCTC	59.955	20	149	484	632
58.694	20	TGGGCAATACGTGGTTTAGG	60.742	20	277	97	373
59.817	20	GAAGAGCAGGGGTGTCTTTG	59.844	20	268	207	474
60.304	20	TGCTAAATCTGCTCTGGCAA	59.712	20	244	755	998
60.032	20	TCATTCCCGAGAATCCTTCA	60.538	20	169	792	960
60.21	20	GCAAAAGGTTTGAAGTCCTGA	59.345	21	232	900	1131
59.636	20	ATCCCAAGTGGGTTTTTGTG	59.688	20	156	16	171
60.827	20	CATTAAATCTCATACCCTCAGTTT(	57.435	25	249	224	472
60.133	20	ATCAAGAGCACGGTGGTTTC	60.119	20	224	210	433
60.119	20	TCACTCCATCTTCACACCCA	60.088	20	195	2614	2808
59.875	20	GCGCCGATAGACGATAAAAA	60.192	20	151	1180	1330
59.817	20	GCGGGGAAAAATAGGAAGAA	60.381	20	178	176	353
59.224	20	TTCCTCAATTTCTTCCACCC	58.956	20	236	164	399
59.272	20	GACTTTCCAAGCATCCAACC	59.532	20	280	59	338
59.933	22	TACTTTTCGCATCCGACTCC	60.214	20	114	2873	2986
60.119	20	TCTCGGTGAGCGAGTAGGTT	60.012	20	232	6	237
59.924	20	GGTTTTGACCATATTTACTGTTTTG	59.631	26	117	439	555
60.448	20	TTAGTTAGTACCGATTTCAGGGTT	57.499	24	153	1177	1329
59.179	20	GAGAACGTGAAAGCCAAAGC	60	20	187	9	195
60.081	20	TTTGACCTTTAAGGCCGATG	60.067	20	254	782	1035
59.84	20	TTGTTCTTTGGTTGCAGGAA	59.293	20	201	2802	3002
59.664	20	TGCCAGTGTCTCAATCCTCA	60.402	20	235	34	268
60.042	20	CCAAAACGTCCATTTTCCAA	60.702	20	273	572	844
57.369	23	TGGATTGAATGGATGTTTGG	59.175	20	102	0	101
60.111	20	TCACAAATACCCCTAAACCCA	59.188	21	148	356	503
59.962	20	TGTCATGGCATTTTTGTATGTTT	59.288	23	236	315	550
58.462	22	CCCTTTGGAATGTTCAAGGA	59.903	20	103	22	124
60.124	23	ACCAAGATTTGATCATGGGC	59.756	20	237	377	613
60.511	20	TTGTTAGAGGTTTTGACGGGA	59.595	21	192	241	432
60.103	20	GGGCTGCCTCCTAATCCTAA	60.542	20	254	348	601
59.431	20	TTATATGACCCCCTCCCCTC	59.974	20	154	1188	1341
58.258	20	ACTGCACCAAGGCAGAAACT	59.914	20	257	1191	1447
59.855	20	GTTTGGAGGAGCAAGTGGAG	59.844	20	148	1053	1200
60.348	20	CCATCATTTCATTCCCAACC	59.991	20	198	3610	3807
59.843	20	GCCCCAAACGACTTACGAT	59.947	19	203	326	528
59.694	20	ACACCCTCAAATGGCTCAAC	59.973	20	225	959	1183
59.982	20	TTAACAAAAGCAAACCGCCT	59.758	20	248	704	951
59.813	27	TTTCGAATGATTTTGCCCTC	60.016	20	218	0	217
58.98	20	TTGTTTACACACGGTGCGAT	60.035	20	253	85	337
59.679	20	ATTTTCCCCAAATTTGTCCC	59.867	20	150	13	162
59.894	20	CTTGAATTGCTCTCCCTCCA	60.331	20	229	183	411
60.011	20	ATTACGTCCAAATTGCTCGG	59.96	20	265	130	394
60.117	20	CCCTTGTATGCAGTCCGAGT	60.134	20	276	201	476
57.792	21	GCCACTCTTGTGCTTCCTTC	59.997	20	167	9	175
60.261	20	TTACATCCAAGCCACCTTCC	59.933	20	158	1059	1216
60.088	20	TCCTTTCTTCTTACGGCACC	59.312	20	251	790	1040
60.457	23	CGCGAACTCATAAATTGAACA	58.83	21	214	12	225
58.96	20	CGTCCGTTTTGAAGATCCAT	59.933	20	256	276	531
60.269	20	CCAAAAGTGTTATTTTTGTTTCG	57.875	23	146	64	209
59.165	23	TGTTAATCAGTTTATGCATCTTGG/	59.917	25	149	24	172
59.028	24	TGTCCTCCATTAAGCAAGCC	60.214	20	114	1	114
60.03	20	TGTGATGAGAGAGAGGGGCT	59.945	20	235	104	338
59.953	20	CGAAGGTACGGTATGCGATT	59.982	20	172	62	233
60.408	20	CGGCGATAAGAGAGAGAAGG	59.17	20	161	422	582
60.663	21	TGGCAACTGCAAGTTCAAAG	60.027	20	269	514	782

60.109	20	TTTTGGGAGAAGTATTGGGA	57.13	20	183	56	238
59.415	20	CCAATTTGAATGGTTATCACCTG	60.457	23	107	1908	2014
59.978	20	GTTATGCGTTTTGACGGGAT	59.829	20	230	286	515
60.017	20	TGCATGGATGCAAGTTGAAG	60.812	20	229	138	366
59.964	20	AATAGCTTTGGGTGCATTGG	59.96	20	194	2862	3055
59.123	20	GGTTTGGCCGTTTTTGAGTA	59.975	20	225	13	237
60.081	20	TTCCGAAAATATGCTCCACA	59.112	20	166	801	966
59.939	20	AGAGGAGCCCCATTCATTCT	60.037	20	203	235	437
59.583	21	AGGTTGCGTACATCTGACCC	59.997	20	256	3	258
59.51	20	GATGGCTTCTCCTTGCAGAC	59.957	20	141	154	294
59.967	20	AGGGTCCCACCCTTTTGTTA	60.581	20	124	220	343
60.065	20	TATGAACCCACCAGCATTCA	59.924	20	131	1957	2087
59.83	20	ATTCTAAGCTCGAGGGCACA	59.978	20	238	327	564
59.234	21	GCTAGAGGGTTTTGTTAATATGTC	58.796	27	174	324	497
60.154	20	GTTAAGGCCAACTTCCCTCC	59.94	20	249	1543	1791
59.934	20	AGGAATGATGGGATCTGCAA	60.426	20	172	2082	2253
60.204	19	GGCGGCGTGACTTATTCTAA	60.23	20	262	208	469
60.035	20	GGGGGAAAATTATAGTTAGGGA	57.532	22	108	524	631
60.131	22	CGATAGATTCGATGAACCCC	59.344	20	226	665	890
60.038	20	GCAATTGAAGGTTTGACCGT	59.978	20	121	5	125
59.557	24	GGCCTATTGAGCCAAGACTG	59.836	20	279	972	1250
60.249	20	GTGCATTTTTGGGAGCAAAT	59.945	20	238	96	333
59.181	21	CAGAAACATGTCAACGTGAGAA	58.825	22	278	1114	1391
60.021	20	TCTCCGAGTTCTTGCTCTCC	59.679	20	228	352	579
59.966	20	CCTGGCTTCAAAACCAATGA	61.001	20	255	669	923
58.237	25	AATTGACCTTTGACGTTGGC	59.978	20	242	3	244
60.052	20	TTTTTATTCAGGCAAACATTCTCA	60.006	24	206	311	516
60.357	20	CGCTGGTTTTGGAGATCAAT	60.074	20	228	2294	2521
59.93	20	TCCGGTCCGATTACAATTTC	59.762	20	165	319	483
60.11	20	AGGCGTCTGTTGGAATTTTG	60.11	20	252	1446	1697
58.824	20	CTTCCAGAGGGATACCGTCA	60.065	20	198	634	831
60.066	20	TGTCCGGATTGTCACACCTA	59.96	20	225	90	314
57.557	25	TTGTAGTTGCTGGCCATGTG	60.72	20	105	21	125
59.773	22	TTCACTTTTACAACTTCCACATGA	59.959	25	205	96	300
59.992	20	TGGAAAGAACTAAATGCCCG	60.067	20	185	117	301
59.829	20	TGTGCATGTCTCATTTCGTTT	59.188	21	140	19	158
59.449	20	AGCCATGGTTCAATCATCAA	58.926	20	254	3115	3368
60.073	20	TCACAAACCCATGTCCCTTT	60.21	20	261	1440	1700
58.028	22	GAAGGAAACATTCTTTTGGGG	59.801	21	249	1914	2162
59.381	20	GCATGGTGATTTGAGGGACT	59.934	20	228	169	396
57.101	24	AGATTGGGTACCCGACACTG	59.844	20	175	5	179
59.839	20	AGATTCAAGGGCCGAGATTT	60.039	20	173	840	1012
59.916	21	TCCATAGCCGATAAAATGGC	59.892	20	217	6	222
60.057	20	AAAATCAAAGGCCCACAATG	59.801	20	139	11	149
59.829	21	GCGACAAATGCAAGGCTACT	60.416	20	177	20	196
60.001	20	GATGAAGGGTGGAAGGTTGA	59.903	20	160	456	615
60.073	20	ATGTGAGTGGCACGTGATTC	59.557	20	211	1611	1821
60.03	20	TATCATTTTGCCCAACCTCC	59.762	20	188	1324	1511
60.255	20	GGATGGCAAGCTTTTAGCTG	59.982	20	188	525	712
60.081	20	CGCATTATCGCTGTGAGTGT	59.895	20	224	1042	1265
58.337	20	AGCCTTGATTGGAACATGGA	60.461	20	252	102	353
59.939	20	TGTAACCCAAACCAAGCACA	60.004	20	247	2095	2341
60.21	20	AGAGGAGAGGCGAAGGAAAG	60.088	20	189	5	193
60.162	20	CCTATGGTGGTATGGTGTGGA	60.501	21	172	81	252
59.835	20	ATCAAAACCGTTACTTGCGG	59.996	20	260	814	1073
59.971	20	TGAATGCAGGACAAGAATGC	59.805	20	222	372	593
59.989	20	CTTTGCCCCTTTACTTGCTG	59.876	20	161	468	628
59.933	20	ATGCCCACATTTCAGAGTCC	59.934	20	234	692	925

60.315	20	TCAACCATTTGTTTTTGCCA	59.946	20	246	907	1152
59.986	20	TTAACCTCCCGTGGACGTAG	59.986	20	262	4	265
59.732	22	TGGTGGAGCGTCTGTTTTAAT	59.623	21	280	31	310
60.025	20	GACCAAGAGGCGTTGGTAGA	60.255	20	249	1943	2191
59.836	20	TCTAGGAGCGAAGGATTTGC	59.551	20	235	390	624
59.64	20	GACCGCAGGTTAATCCAGAA	60.074	20	240	1051	1290
59.806	20	TGTCTCCATTCTATTGGCCC	59.894	20	230	236	465
60.028	20	GATCTGAACCGACCGAACAT	59.934	20	255	206	460
59.986	20	ATTAGCGAACATGGAGGTGG	59.955	20	123	505	627
59.64	20	TCCGAGACAAGTTTCGTGAAC	60.284	21	270	63	332
60.122	20	AAAATCAAAGGCCCACAATG	59.801	20	265	391	655
60.17	20	TTATTGTGTGGGGCAGAGGT	60.375	20	226	139	364
59.685	20	TTTTCCCTGAGCACGTTAGG	60.241	20	178	404	581
59.239	20	GATTCTGTATCCGCCATTGAA	59.915	21	202	666	867
61.096	20	TGTGAAAGTCTTTGGATGGTTC	59.077	22	258	101	358
60.195	20	ACAGCTTCGTTGATGATGGA	59.245	20	247	474	720
60.894	20	CCGCCGATGTGATTTTAACT	59.96	20	273	242	514
60.133	20	TTCACACACCAAAATTCCGA	59.941	20	192	637	828
58.709	21	CATTTTTCCTCATGGAATGGA	59.748	21	188	1123	1310
60.362	20	GATTACAAGGCCCACCTCAA	59.933	20	239	124	362
59.744	21	CGGAATACAATTCGACAGAAAA	59.12	22	276	1051	1326
60.12	20	GAGGGGTGTTTCCAGACAAA	59.943	20	276	27	302
60.012	20	GTTGTTGAGGTGGTCTCCGT	60.009	20	268	585	852
59.829	20	TCCCGGCTGAATGTTACAA	60.057	19	176	10	185
58.028	22	GCATTGCAGGTTTCGAATTT	60.081	20	142	15	156
60.885	20	ACGAAAATCTCTGCCCAATG	60.074	20	165	221	385
59.762	20	ATTGCCACCCCTCACTACAA	60.375	20	246	488	733
59.923	20	AATGCCCTCAGATGCTATGG	60.059	20	249	384	632
60.129	20	CTGCCGATCAATGCAGTCTA	59.972	20	274	842	1115
59.822	21	CGTGTCAGAAATTGACAGCC	59.293	20	273	173	445
58.521	26	CAAAATTATGTGGCCCCAAC	60.053	20	274	87	360
59.522	20	GGGTCCCACCACCTAACATA	59.526	20	195	71	265
59.212	20	TCACTCCAGTTTTTCACCCA	59.109	20	206	902	1107
60.111	20	GGAAGCAAAAATGGCAGAAG	59.823	20	246	567	812
59.717	20	TGACTAAGTTGTGTGAGCAGGAA	59.966	23	262	741	1002
60.505	20	CGTAAAAATGGTTATTATTGGATC	58.395	25	254	909	1162
59.14	23	ATGCTGATGTTAGTGCGCTG	60.042	20	188	16	203
60.285	20	GCCCCCAATTTCTCTCTTTC	60.017	20	197	34	230
59.743	20	GATCGCATTGATCCCAACTC	60.435	20	167	605	771
59.978	20	CCATCTACGCAAACCCAGTT	59.993	20	213	150	362
60.117	20	AATTTCCAGATTCCCGTCG	59.879	19	173	1518	1690
60.569	20	TGTTGCGCGTGCTTTAAATA	60.401	20	134	1557	1690
59.813	20	CCACGACGGTTAAAGGAGAA	60.103	20	253	213	465
59.234	21	AGGGGGTTATCACCTGATCG	61.095	20	189	534	722
60.111	20	ATTTCTGCAATTGGGGTTCT	58.504	20	180	27	206
60.051	20	TGTACCGTGACAAGACCTTTG	58.687	21	100	887	986
59.901	20	CCACAACCCTTTCATCACCT	59.82	20	254	336	589
60.218	20	CATTGATGGTTTCGGAGCTT	60.074	20	280	232	511
59.002	20	CAATTACACTGCATGGGCTG	60.134	20	167	76	242
59.903	21	CAGCATTGATGGTCACATCC	59.925	20	217	118	334
59.94	20	AACATGGCCAACCCAAATAA	60.053	20	275	2488	2762
60.131	20	TGGCAGAAGTCGTTGAACTG	60.025	20	248	113	360
59.077	20	TCGGAGCCATAATCATCACA	60.033	20	225	696	920
59.595	21	TGGATCTTCGTATTTGTTACTTCA	57.899	24	129	213	341
58.977	20	GTTTTCATTTGATCACGCCC	60.318	20	106	24	129
59.972	20	TCCACTTTTCAATGGTGCAA	60.088	20	275	477	751
59.79	20	GAGATTGGGACAGACCCTGA	60.048	20	196	1667	1862
58.878	22	CGTCCAGTTCACTTCATTGG	59.135	20	113	1166	1278

60.269	20	CCTGAATTCTCCCCAACTCA	60.042	20	270	501	770
59.585	20	GAGTTCGTCAAACCTCCCAA	60.088	20	261	133	393
59.764	20	GAGGGAAATGGTGGGGTATT	59.881	20	107	1077	1183
59.82	20	AATCTTTGAACAGCCGATGG	60.074	20	208	577	784
59.722	20	CGAACCCTTCATCATCGAAT	59.894	20	100	570	669
60.103	20	AACCTCTTCGAGTTCGTCCA	59.844	20	278	390	667
59.829	20	AGAGTGCGCGTTTTCATCTT	60.022	20	108	442	549
59.518	20	TCACGACATTATTTCCCTCAAA	59.451	22	133	3481	3613
59.501	20	GCGGAAGCTGTGGTTTGTAT	60.14	20	270	228	497
59.129	20	AGATCTTAATCGTGGCCGCT	61.11	20	182	388	569
60.015	20	TCTTCTCCGTTTCAGTCCGT	59.844	20	226	560	785
60.073	20	TGAAGTTAGGAAATAAGGAGGTG	58.743	25	151	693	843
57.239	26	TAAATCATGTTTCACCGCCA	59.93	20	134	2	135
59.685	25	GACAATCTTCCAATTGGCGT	59.939	20	255	144	398
59.668	20	TCTTCGAGACGGAACCACTT	59.844	20	255	582	836
60.015	20	TGCAACTCCTCTGACCTCCT	59.986	20	125	2857	2981
59.75	20	CCACCACCAGAAGCAAACTT	60.149	20	143	247	389
60.46	20	CTTTATTAAAATGCGGGGCA	59.927	20	277	385	661
59.993	20	GAACAAGGGTAAGATGAACCCA	60.221	22	187	394	580
59.836	20	GCGGCTTCATCTTTACTTGC	59.988	20	239	167	405
60.032	20	TCACTGCTGACCGTGTTCTT	59.467	20	172	167	338
60.17	20	GTAAAGGGGGCACGAAAGTT	60.352	20	263	1643	1905
59.952	23	ATCAGATGGGCGAGCTCTAA	59.939	20	247	251	497
59.993	20	AGGCTGGATTTCGTCCCTAT	59.925	20	274	376	649
58.32	21	AAACGGTTGAAAAGCCCATA	59.451	20	247	18	264
59.25	20	TTTGGATGGAGTTTTGGAGC	60.051	20	199	186	384
60.012	20	CGTCACGGTGGTTCTTCATT	60.954	20	277	555	831
61.211	20	ATCCACGTCCTTGTGTCTCC	59.969	20	191	721	911
59.706	20	CCACAGCTGAAAACGTAGCA	60.05	20	225	2448	2672
60.066	20	TTGATGCACGAATTTGGAAG	59.664	20	228	198	425
59.067	20	ACCAGACCGTTAGGGGTGTT	60.66	20	277	50	326
59.803	20	CCACTTATGTCGAAGATTTTTGTG	59.933	24	261	1589	1849
59.994	23	TCTAAGGGATGATTGCACCA	59.075	20	125	322	446
59.897	21	ATGGCGCTATCTGGTTGTTC	60.103	20	109	6947	7055
61.118	20	AATTTGGGCCTCCATTATCC	59.984	20	213	416	628
59.647	20	GTTGTCTGAAAGGCATCGGT	60.119	20	225	155	379
59.992	20	CTTTTTGGTTGCTGTTGGGT	60.008	20	218	6944	7161
60.132	20	CAACACACAAAGCCACCATC	60.008	20	214	183	396
59.825	20	AGTGGTTATTACCGGATCGC	58.93	20	111	832	942
60.41	20	CTTGGAATCGTGAGAAGCGT	60.397	20	252	936	1187
59.831	20	TACAGGGGGATTTGGATGAG	59.744	20	149	567	715
59.809	20	TGCAGGGTATGCATGTTTGT	59.995	20	159	306	464
59.585	20	GGTTGCATACTGTGGGGATT	59.676	20	253	780	1032
59.716	20	CAAACAATGGGACCACAAAA	59.268	20	256	248	503
59.43	21	GTCTGGATCAGCTCGGAAAG	59.95	20	279	251	529
60.111	20	GTGGCTGTGGTTTTCGTTTT	60.015	20	279	181	459
59.901	20	AAGGCAATGGACATGGAAGA	60.461	20	244	1835	2078
60.045	20	GACAAGCACTCATGTGATGGA	59.698	21	193	3083	3275
60.119	20	TGCATCTCTAATTGGACCCC	59.894	20	122	205	326
60.112	20	GAAATGCAAGATTGCGACAG	59.425	20	131	1155	1285
59.297	20	ACCACCACCACCATTTTCAT	59.95	20	263	107	369
59.768	20	CAGTCATTGGATTGCATTGTG	59.98	21	159	342	500
58.959	21	ATGAAAATACGCGGGGAAAT	60.517	20	265	92	356
59.989	20	CAAAGGAAGTCGGTTGGTTC	59.569	20	166	17	182
60.201	20	CCCCCACCACACCTCTACTA	59.837	20	217	3485	3701
60.306	23	CCGAATCTTTTCGTGAATCAA	60.06	21	240	25	264
60.672	19	TTGAGATGAGGGATTTTGGG	59.864	20	197	3273	3469
59.654	21	CCAAGTAAAGCTCGAACCCA	60.241	20	265	1297	1561

60.17	20	TTTCATGATCCAGATGGACG	59.451	20	249	127	375
60.11	20	CTTGTGGAGCTACTCAGCCC	60.012	20	275	158	432
60.167	21	TGCGAAAAATTTGGTTCTTG	58.792	20	247	198	444
59.066	20	GTGGCACGATCATTGTTGTC	59.975	20	195	327	521
59.066	20	GTGGCACGATCATTGTTGTC	59.975	20	195	316	510
60.073	20	TGCTCTTGCATCACCTTCAC	59.992	20	268	396	663
59.817	20	CGTGCTCATGTCAGCAAGTC	60.627	20	164	23	186
58.812	20	TCGAAGATCAATGGTCCACA	60.048	20	134	118	251
59.561	24	TGCGTTTTGACAATATATAGGTGT	60.083	26	149	1376	1524
60.088	20	AGCGATAACATGGATGGAGC	60.066	20	275	2377	2651
59.832	20	GTTGGCATAAAATCCAACCA	58.335	20	244	512	755
60.152	20	GCTATCAGGAACCTATGTTTGG	57.774	22	207	1217	1423
60.514	24	TTCAAACAAGGCATAAAACTTTG	58.412	23	132	227	358
59.844	20	AGAGGCTTCATCTTCTGCCC	60.873	20	244	749	992
58.057	20	GCAAAGAAGGGACGACTGAG	59.989	20	238	885	1122
58.953	21	ACTCACGTGTCAGCACACAA	58.876	20	189	29	217
59.864	20	TTACAGCCCAGATTCCCAAG	60.066	20	277	231	507
59.81	20	TGATAGCGTTTTGCTGTATGC	58.998	21	202	418	619
59.82	20	GAGGTTGGTGACAGTGGCTT	60.159	20	272	484	755
60.393	20	ATCGCTTCTTCTTTCGCAGA	60.235	20	279	216	494
58.826	22	CCCCTGGTTGTTATCCTTGA	59.784	20	186	37	222
59.691	20	GTGTGACATTGGCCAGACAT	59.399	20	263	1022	1284
60.461	20	TCGATAGGACGTGTATGACGA	59.163	21	216	1687	1902
59.929	20	GGAGAAATTTGATTTGTTGGGA	60.164	22	274	205	478
60.073	20	TTAACCCTTGGATCTGAAGCA	59.693	21	269	49	317
59.984	20	TACTCTTCGATCCCGCGTAT	59.691	20	232	72	303
59.062	20	AGATGCTTTTTGTCGCGAAT	59.851	20	275	2434	2708
59.624	20	CGCCGCCATTATCTCTACTC	59.829	20	254	22	275
59.557	21	CCCCACTTTTAAATTGCGAA	59.937	20	280	710	989
59.864	20	GATTGCGTAGTCGTGTGTGG	60.183	20	267	561	827
60.702	20	TTAAACTACACATGTAATCCATAA	58.07	27	152	970	1121
60.913	20	ACAAAAATCTTGGTTTGGCG	59.975	20	266	115	380
60.17	20	CGCGTTAACAGTGGAAAACA	59.769	20	240	96	335
57.503	26	AATATCCTTTCGGGACACCC	60.017	20	148	13	160
60.066	20	ACACAGAATTCGCCCAAAAC	59.978	20	221	233	453
60.053	20	TTCCTACTCCCCTAGGCCAT	59.919	20	219	251	469
60.048	23	AAGGAAAGATCCGTCGAGTTC	59.705	21	170	3	172
60.123	21	GCTTAAGAATCGTGGCTTCG	59.982	20	199	220	418
59.457	21	AGCAAAAATAAAGGGGCTGC	60.566	20	278	609	886
59.109	20	GAATAAATGGACCCCCGAGT	60.017	20	164	427	590
60.008	20	ACGGCACATCAAACACCATA	59.847	20	217	4572	4788
60.145	22	ATGGAACATCTGATCACGCA	60.08	20	193	347	539
60.073	20	GCAACAATGGAGCAGATCAA	59.805	20	161	2273	2433
59.95	20	GCATCAAGAATCAAGCCTCC	59.78	20	262	714	975
59.579	20	TTGTATTTTGTCTGGCAGCG	59.872	20	215	159	373
60.162	20	GAAAAGGGAAAAGGACCGAC	59.919	20	173	368	540
60.203	20	ACCTCCCTCTTCTTTCCCAA	60.045	20	114	1144	1257
60.103	20	GATCCCGACGACCTCTCATA	60.034	20	139	539	677
60.017	20	AAACAAGGCATGGTTCATCC	59.797	20	238	840	1077
57.943	20	TATCCCTAAAGTGCCCTCCA	59.523	20	190	20	209
60.066	20	GAGTGCACCTCCTCAATGCT	60.418	20	244	1146	1389
60.074	20	GGGGTGAATTTAAAGGGGAA	59.992	20	259	261	519
60.014	20	ATTGACTAGGAGCACGCGAA	60.934	20	280	580	859
59.708	20	ACTGTCTGGGGCTTTGTTTG	60.149	20	240	88	327
60.206	20	CACACTCTTTCTCAGTGGACG	58.504	21	240	1020	1259
59.84	20	TTCCAGTTCCCTTCACCAAG	60.081	20	246	354	599
60.683	20	GGCATCACATGGTCTCTTGA	59.637	20	131	380	510
57.844	20	GTGCATGAGCTGTCTCTTGG	59.577	20	110	128	237

59.181	22	TTGACAATATTTAGTGTTTTGACAT	58.99	27	166	943	1108
59.438	20	AAAACGCGCAATGGATAAAC	59.971	20	243	179	421
60.11	20	TCCCATTCTACTGCCCAAAC	59.933	20	266	452	717
59.993	20	CGTAAATCCGGCTTGAATTG	60.448	20	217	300	516
59.874	20	TGCAGACATGAAGAGGGTGA	60.402	20	263	152	414
59.874	20	TACATGGCCAACGGGTATTT	60.074	20	260	177	436
59.939	20	TTCTCATCCTTTGCCTTGGT	59.67	20	235	395	629
60.173	20	GAAAATTGGCAGGATCCGTA	59.901	20	231	139	369
60.234	20	TTCCCACCATGATTTCAACA	59.75	20	220	1307	1526
60.152	20	TGTTTATGCCGTTGATACAAAAA	59.437	23	221	185	405
60.055	20	CCTAAAATTGGGTCCGATCA	59.756	20	242	293	534
59.475	21	TTTCCTGCAACAATTTCAACC	59.967	21	219	648	866
59.832	22	GGTTTGAACCATGATTTATTTGG	59.491	23	236	401	636
59.989	20	TTGGTTCGAGCCATTGTGTA	60.111	20	152	15	166
60.353	20	GTCCACAGTTACCCGCAGTT	60.035	20	239	497	735
60.263	20	ATCTCAGAACTCCCTTCGCA	59.95	20	219	1218	1436
59.943	20	AACTCCAAATTTTGATGCATGT	58.493	22	259	366	624
57.065	22	AACCAAAAACTCTCGACCCA	59.569	20	229	848	1076
59.676	20	CTATCCCCGAATGTGCCTAA	59.916	20	220	844	1063
59.943	20	TGAAGTTTACACAACAAATCTAAC	59.348	26	204	1628	1831
60.903	20	CTCTCAGTCCCAGACTTGCC	59.986	20	216	23	238
58.601	20	ATAGCTCAAGGGTTTTCGCA	59.845	20	208	259	466
59.984	20	GGGGACTGTCAATTCATTTTG	59.278	21	111	1388	1498
59.939	20	TGCAATTGTTCGTTCCTTCA	60.234	20	176	90	265
60.349	20	TGCCTACACAAATCCACCAA	59.964	20	133	921	1053
59.601	21	AAGGCAGTTATGACCGTTGG	59.993	20	158	29	186
60.088	20	TCACGCCTTCTCTCTCCAAT	59.95	20	257	617	873
59.746	20	CGGTACCGTCACCGTTATCT	59.875	20	232	126	357
59.767	20	AACCCTCAACCCTCTCCAAT	59.795	20	224	21	244
59.434	20	ATTGTGGCCCTCAGATTTCA	60.461	20	153	168	320
59.939	20	GCTCGTTTCGGACCAATCTA	60.214	20	271	214	484
58.9	25	TGCTTCCCTCACAACAACAA	60.278	20	268	1783	2050
60.457	23	GCGTTTTTGGCCTGATATTT	59.081	20	188	17	204
60.088	20	TGCATCTGTGAATGGAAAGC	59.805	20	278	76	353
59.989	20	CTGGGAGCATGTCAGTGTTG	60.309	20	224	763	986
60.206	20	TCGTTTGAGATCACGAGCAG	60.136	20	250	881	1130
58.858	20	TTTTTGTAAACCGCCTCGAT	59.586	20	193	584	776
59.869	20	ACAGGCCAAAGAGAGCAAAA	59.993	20	142	511	652
60.142	20	CAGAGAAAAGTCGTCGGGAA	60.366	20	221	117	337
59.926	20	GAGTTCCCGCGTCATAAAAA	60.074	20	256	498	753
59.882	20	GGGGAGAGTCAGGTAGGAGG	60.066	20	144	103	246
59.845	20	TGATGAATGACGACACATGC	59.037	20	266	316	581
60.175	20	ATGTGGTGTTCAAGCGTCTG	59.751	20	272	603	874
59.606	23	GCACGATCTCATGATTCCAA	59.61	20	255	21	275
59.396	20	TCATTTTTGACCGACGGTTT	60.344	20	188	23	210
58.789	22	CAATAAACGCCCACATGAAA	59.42	20	267	370	636
59.831	20	AAATTCCAAACTCCGACCAA	59.406	20	232	174	405
59.938	20	CGAGTGCCGTACTTGAGAGA	59.187	20	209	216	424
59.378	20	GCGCTATTTTTAACCCCAAA	59.11	20	214	204	417
60.12	20	ATTTCGAATGTCGCCTTGTC	60.081	20	242	1100	1341
60.012	20	TTGTCTCTTGCATCACTCCCT	59.859	21	259	488	746
59.481	20	CATGTGGCTTTGCTTTGAGA	59.988	20	160	191	350
59.728	20	CTCTTCACATTTCCTCCCCA	60.042	20	127	113	239
60.154	22	CTCGCCTCCAATACCAAGTC	59.694	20	272	178	449
60.827	20	ATGGCATTTCTTTGGCTTTG	60.074	20	257	524	780
59.7	20	CGGTATCACGGACGATTTTT	59.823	20	248	863	1110
60.031	20	TCTTCGTGTTCTTCGTCGTG	60.025	20	158	934	1091
57.943	21	CAAATCATGTTGAAAGGAACCA	59.839	22	272	728	999

60.05	20	TTGAGTGGTTCGGGGATTAG	59.926	20	205	242	446
59.397	20	TCGATAAATAGCCACATGCC	58.603	20	251	180	430
59.891	23	GGGACACCTCAGTGGTAGGA	59.962	20	264	59	322
60.119	20	TGTGAGGGACACAATTCTTGA	59.124	21	248	204	451
59.811	20	GGGGTGATTGGTAATAGCTCC	59.68	21	174	140	313
57.557	25	TTCAATCATGAGCGTTCGTC	59.805	20	136	16	151
58.486	22	TAATTCTGGCGAATCGGAAC	60.038	20	128	287	414
60.046	20	TCTTAATGAGGGCCCCTACC	60.278	20	263	125	387
59.298	20	AATTCCTATGGGTTTTGGGG	59.882	20	225	74	298
59.339	20	TGGGAGAAATCGATAATGGG	59.717	20	255	150	404
57.52	22	AAAATCAAAGGCCCACAATG	59.801	20	139	996	1134
60.025	20	GCGCAATTGCATCTATTGAA	59.809	20	177	2011	2187
60.198	20	CAGAATTGGGTGTAGCAGCA	59.864	20	175	263	437
60.005	20	CTCAAACCCCAAGAATCGAA	60.044	20	252	455	706
60.214	20	AATTAGCTTCTTCGTCGCGT	59.138	20	277	1389	1665
59.728	20	GTTCGCGAGATCCAGAAGTC	59.957	20	265	1445	1709
60.005	20	TGTTAATCTGTTCCGGCCTC	60.074	20	233	3245	3477
59.673	20	CTGAGGCCACTACCCGATAA	60.088	20	271	719	989
59.986	20	CAAAAGGTTTATCCCAGTCTCTC	58.333	23	108	3539	3646
60.036	25	TGAATTTTTGGAAATGGTGTTC	58.831	22	227	25	251
59.988	20	TAAGACACCAGGCAGTTCCC	60.111	20	202	263	464
60.125	20	AGCTTGAAACACACACACGC	59.953	20	252	307	558
59.469	20	ATGAAGAAAATCTGGCGCTG	60.352	20	244	615	858
60.036	20	GAAGTCGTCGTAGGAAAGCA	58.104	20	223	735	957
60.223	20	CAAAGCTCAAGCTGATGCAA	60.28	20	211	2993	3203
59.18	20	GGGGATTTCTGTCGCTTTTT	60.431	20	223	330	552
60.369	21	GTGATGATTCGGCTCGTTTT	60.081	20	194	558	751
59.523	20	CCATCATTCAACGCATTACG	59.953	20	274	2782	3055
58.755	20	ATAGGCCTACATCGAAGCCA	59.691	20	225	118	342
59.679	20	TGACCCTTTGTACCGTTCCT	59.449	20	181	113	293
59.96	20	AAGGAACGAACAAGTTTTCCA	58.73	21	270	654	923
60.526	20	TGAATGTTGTCGTAGCCTGTC	58.79	21	108	1358	1465
59.202	23	GACGCTAACGCCATTACGAT	60.124	20	255	531	785
59.708	20	GTGGCATGGGGAAGCTACTA	60.096	20	234	52	285
60.169	20	GAGCACGCAGAGAAGCAATA	59.326	20	238	378	615
59.688	20	GACAATCCGGCCAAATTAAA	59.773	20	276	196	471
60.089	20	GTGGAAGCGCCATGTTTTAT	59.967	20	240	130	369
58.8	20	GTATCAGCAGCCACCCAAAT	59.962	20	262	4780	5041
57.517	20	GCAAAGTGGAGATCATGGGT	59.934	20	229	631	859
59.861	21	GTCTTTAACCGCCACCAAAA	59.975	20	209	524	732
60.071	23	GAGAGCAGTTTCGGACATAAA	57.108	21	280	139	418
57.406	21	GCCAAAACTACCCCTTTCAG	58.694	20	176	414	589
59.657	20	CTTCAGCAAACCAGCTCTCC	60.134	20	247	44	290
59.938	20	TGCGAAGTTTGTTCTTGTGC	60.035	20	157	560	716
60.517	20	AAGGAGTTCACAGGTGGTGG	60.002	20	259	3	261
59.716	20	TCAAATTTTGGCTTTGCAATC	60.068	21	274	80	353
60.066	20	TGGAAAACACCCTTTTCTTGA	59.573	21	232	512	743
60.035	20	TGTCTCATTGTGCTCAAGCC	59.992	20	116	222	337
60.015	20	AGCTCGGTCGGGTAGAAAGT	60.268	20	246	91	336
59.758	22	GGGGTTATCATGGGATCAAG	59.055	20	266	2403	2668
59.939	20	GAGAAAGTGAAGAATCGATTGGA	59.717	23	123	370	492
60.235	20	TTCACGCAAACAAATTCCAA	60.088	20	161	242	402
59.982	20	TCAAGTGTTCCCATCACCAA	59.935	20	222	498	719
59.984	20	CCCAACTTTCTCCCCTCTTC	60.045	20	170	3415	3584
59.931	20	TGAAGCACAATGAAAACGGA	60.234	20	237	130	366
59.993	20	CCCACAACTTTTCTCTGCCT	59.328	20	212	1564	1775
59.738	23	CTCAACAGCGTGTTTGGTTT	58.825	20	280	1316	1595
60.089	20	TTTAGGCGTTCAAAAGGCAT	59.72	20	178	311	488

59.916	20	TCGAAGATCAACGATCCACA	60.201	20	177	799	975
59.988	20	TGTTGTGTGCAAGTTCCCAT	60.008	20	253	548	800
59.923	20	TGCACAACAAATAGGCAAAA	57.846	20	224	225	448
60.24	20	TACATTCCAATGGCAAAGCC	60.837	20	165	800	964
59.999	20	AACTCATTGTCAACCGCACA	60.16	20	217	566	782
59.878	20	ATTTCCAAGATCAACGGACG	59.933	20	169	496	664
59.989	20	AGGCTGATTGTGAACTCCAGA	59.859	21	250	2091	2340
58.743	25	GTGGACGCTCCAGATTGTTT	60.119	20	124	2	125
60.23	20	TCTTCCCAGCAAGAAAGGAG	59.545	20	268	95	362
59.992	20	AATTGTTCATTTGCAACGGC	60.876	20	273	802	1074
59.923	20	TTAAATATTCATGGGCACGC	58.513	20	209	158	366
59.924	20	CTCAGGCCTATCGAACCAAA	60.206	20	276	102	377
60.142	20	TGACCCGCCTCATCTTCTAC	60.218	20	197	1158	1354
58.743	25	CAGGTTTATCAACAAGGTTTTGG	59.795	23	140	24	163
60.312	20	AAGGCAAGCTAGAACCCACC	60.63	20	161	551	711
60.051	20	ACCTCCTCCTTTTCCTTCCA	60.045	20	265	787	1051
59.946	20	CCAGAGCTGAAAATCGAAGG	59.948	20	274	525	798
60.134	20	CGAATTCTAGGCGATTCCTG	59.801	20	218	1266	1483
60.016	20	GGGAAGAACGACGACGATAA	60.074	20	254	50	303
60.008	20	GGTTTGGTTGGAGAAAGCAA	60.088	20	188	296	483
59.828	21	CTTGAAAAGGGTTGCAAGGA	60.22	20	101	856	956
59.997	20	ACCCTGTGTATCCCAGCAAA	60.375	20	220	104	323
59.813	20	CTTTCTTGGACCAGATTGCC	59.67	20	240	3551	3790
59.28	20	TCAATCATGTTGGGTGCAGT	59.967	20	201	248	448
59.968	20	TACAAGCCTTGGACTTTGGG	60.103	20	173	1216	1388
60.181	20	GGACGTTGATTTGTTGGGAT	59.653	20	200	170	369
59.989	20	AACCATGTGCTTACACCACG	59.49	20	247	244	490
59.913	20	TGGTCGGAGACTAGGGTTTG	60.103	20	272	228	499
60.214	20	CCGAAACGATGAACGAGTTT	60.11	20	137	1001	1137
59.353	20	CTGCATGCACATAGAGTATTGG	58.346	22	274	380	653
59.96	20	TTCCAATATTCTTCGGAGCG	60.167	20	254	1879	2132
58.877	26	GGTACGTGGGGTATGGTCAA	60.495	20	276	610	885
57.036	22	TGAAGATCGATTGGCCATAA	59.065	20	237	268	504
60.17	20	TCTTTCTGCATCCACCACAA	60.24	20	258	110	367
59.702	20	TAGGCCCATAAATGCTTCCA	60.415	20	249	39	287
59.547	20	AACCGATTCTCACCTCTCCC	60.456	20	136	676	811
59.014	20	AGTCGAATCTGTCACGACCC	60.12	20	176	431	606
60.038	20	AACTGGCAATTGCTGGAATC	60.081	20	206	645	850
60.081	20	TCCCTACGAGGAAGTCAGGA	59.797	20	277	103	379
60.111	20	TTGGTTTTGTTTGGGATTGA	58.841	20	280	117	396
59.914	20	ATTTGTTGGAGGTTTGGTCG	59.83	20	223	57	279
59.157	21	TTTTTATTGTTTGCTCCGGG	59.937	20	138	48	185
60.224	20	GCCATTGGGGAAGGTAAGAT	60.152	20	143	371	513
59.955	20	CAACTCAAATGCATGGGAAA	59.518	20	129	237	365
61.031	20	TGTGCTGGATGTCGTTGATT	60.12	20	138	19	156
60	20	TCATTCATTCAGACTCAGGGG	60.058	21	113	935	1047
59.215	20	TGTTCACCTTTTGGCCATTT	60.344	20	195	30	224
58.897	20	CTTTCTGGTCAACACTCACGA	58.917	21	222	213	434
59.734	20	TGGGATAAAATAAGGGGTTTCC	60.241	22	255	966	1220
60.111	20	TGGGCTGCAAATGATAAAAA	59.146	20	204	88	291
59.494	22	CCGTCTCCTCCCTCTACCTC	60.208	20	245	843	1087
60.019	20	CAAGAGCAACACATGCCAAC	60.31	20	232	2586	2817
59.088	23	GTGTGTCTGGTGGGGAAGAC	60.421	20	166	15	180
59.638	20	GGGGCCAATCCAAATTTAT	58.563	19	192	618	809
58.285	21	ACTGAATCCTTTCACGGAGC	59.288	20	175	409	583
59.691	20	GTCGATCCCCTGAGTGTGTT	59.969	20	279	7	285
59.96	20	GGTCTCTCAAACTGGCCAAA	60.232	20	128	5861	5988
59.73	20	CGCCCCACATTGTTTCTTAT	59.823	20	119	70	188

59.161	20	GGAGGAATGCAAGTTGTGGT	59.973	20	280	263	542
59.845	20	TTTTGTTGGACGAAAATGTCC	59.831	21	248	1253	1500
58.743	25	TCTTAATTGGGGTGAAAGCG	60.067	20	260	6	265
59.916	20	TACCGACCATTAGCAGCCTC	60.235	20	189	557	745
59.304	20	CTCGCTTTATCCTCCTTCCC	60.166	20	258	1685	1942
58.243	20	TCGTCCAACTTTAAGTGATTTTCA	60.037	24	209	239	447
59.064	20	TGGTTTGGAATATACCGAAGTTG	60.115	23	115	4062	4176
59.875	20	GCGTAAGGGTGTTCGTGAGT	60.179	20	143	77	219
60.04	19	ATGCCATGACACGAACAATG	60.399	20	171	199	369
57.944	23	GGCCGAATTATGCTTTTGAA	60.04	20	261	94	354
59.738	21	TGTGTCATCTTACACTTTCCACA	58.201	23	136	20	155
59.229	25	ACAATCTGCGAAACTGGGTC	60.119	20	107	17	123
60.199	20	CTCGACAACCTTGCCCTATC	59.694	20	212	547	758
60.074	20	GAGAGTGCCTCACTTTTGTGG	59.899	21	203	177	379
60.035	20	GTCGTGGAGGCATGCTTAAT	60.103	20	275	682	956
59.978	20	CTTCAATTTCTTGGCGTGGT	60.11	20	267	393	659
59.985	20	TTCATCGCCTCTCCTCTTGT	59.95	20	275	35	309
60.096	20	CATTTGCAATGGAAAAAGGG	60.294	20	180	1720	1899
60.289	20	CGGTTATTACCGGATCGCT	59.93	19	196	61	256
59.141	20	AAGAGGTTGGGGGTGCTACT	59.994	20	254	669	922
59.997	20	CAAAGTCAATAGCCCAACCAA	59.982	21	238	320	557
59.629	20	CGTTTGAGCTTTGTTTTCTTGA	59.553	22	156	29	184
59.973	23	CCCTTTTTACCCACCGATTT	60.048	20	115	16	130
60.089	21	AAAATGCATCCTCGTGTGTG	59.572	20	277	260	536
59.202	21	GGCAAGACTCGATCAGGAAG	59.95	20	158	179	336
60.039	20	CCACTGTTCAAGTGGACGAA	59.72	20	214	119	332
60.34	20	ACCAAGCATTGTGGATGCTC	61.078	20	109	600	708
60.331	20	CCAATTTGAATGGTTATCACCTG	60.457	23	115	455	569
60.211	20	TCTCGTTTGGAGGAATTTGG	60.044	20	211	488	698
60.103	21	CCTCGTTTGCAGCAGTTACA	60.05	20	234	992	1225
60	20	GGCGTTACCGCATATGATCT	59.951	20	192	3205	3396
58.349	23	TGTTTTTCCGATTTCACCAA	58.987	20	177	33	209
59.26	23	TCCGTAGAATTTTAAAAAGAAGTA	58.158	27	267	61	327
59.7	20	AAAATCAAAGGCCCACAATG	59.801	20	168	369	536
59.655	20	ATCCCAAATTCCCAACCTCT	59.629	20	107	585	691
59.872	20	CGTGGCAGGCTAATATCCAT	59.945	20	168	85	252
60.203	20	AAATGGCCAAGCCTCTGTTA	59.708	20	253	805	1057
60.008	20	TCTTGTGGTAAGCAGCTCCA	59.591	20	194	1200	1393
59.712	21	TATATCGGGGGCTGAGCATA	60.403	20	146	248	393
60.081	20	CAAACTCCACCATAACCGCT	59.993	20	233	552	784
59.229	25	ACAATCTGCGAAACTGGGTC	60.119	20	109	17	125
59.84	20	AAGGGGACTTGCACAAACAG	60.149	20	244	100	343
59.091	23	CGATATTGTGCCGGTTTGAT	60.721	20	271	90	360
59.749	21	CGGGTGAGAATAATTTAAAGCC	59.049	22	179	2224	2402
60.179	20	CATCAGGTTCACTGGCAAAA	59.691	20	275	1120	1394
57.557	25	GGTTGGCCAAGTAAGGTCAA	59.971	20	244	15	258
59.496	20	TCAATGCAGATGGGATTCAA	60.009	20	201	421	621
60.068	21	TTCACGAAAGTTGCAAAACG	59.888	20	185	418	602
58.747	23	TTCATAATTTTGTTGCTTTTGC	57.131	22	272	1845	2116
60.081	20	AAATCCCAGCCACACATTTC	59.797	20	143	494	636
60.362	20	CATGATCACTTAGGCACTTAGGAA	59.687	24	279	547	825
60.068	20	CAGAGGCATGGTCTCAGTCA	59.98	20	239	610	848
60.481	20	GGGAGGACCATAGCTCATCA	60.034	20	187	467	653
59.535	21	GGAGATCGGAATCGAACGTA	60.036	20	199	466	664
60.144	20	GCCCACTCTTTCTAACCGTC	58.792	20	126	511	636
60.096	20	GCAGAAAAACCATGGGAGAG	59.67	20	106	451	556
59.934	20	AAAGCAAGCCTGGACTACCA	59.875	20	130	331	460
60.582	20	ATTTTCGATTTAGGGGCGAT	59.772	20	225	1417	1641

59.632	20	GGTTTTCAGATACACGTGCC	58.076	20	191	5	195
58.743	25	ATGAGGAGGAGGAGTTGGGT	59.929	20	224	3	226
59.842	20	CAAAGGCCTAGAAGTGTTTTGA	58.562	22	154	688	841
59.101	20	AGGAAATGCAGTGTTTTTCAA	57.411	21	259	216	474
60.103	20	GACAGTTTTCATTTGATCAAGCC	60	23	196	27	222
59.96	20	TTGAAACGGACCAACAATGA	59.941	20	266	1	266
60.692	20	GACCAGATGGGTGCTTGAGT	60.12	20	239	869	1107
59.927	20	TTCAATTGTATTAGCCGATTTTT	57.072	23	217	242	458
59.367	20	GAGAACCAAGGGTCAAGAAAAA	59.61	22	202	336	537
59.369	20	CAAAAATCCAACACCTCTCAGAT	59.513	23	228	1310	1537
60.04	22	TCACAATCGTCCATTTTTCG	59.518	20	273	244	516
59.978	20	TTGTGTGATCGAACCACGTT	60.008	20	179	118	296
60.118	20	CCGGAGCTTCAATATCCAAA	60.031	20	234	591	824
59.876	20	TGACTCAAGTCTTCAACCAACA	58.397	22	273	269	541
60.229	20	CGGAGGGTTGGTGTTAGAGA	60.103	20	180	1	180
59.939	20	CGGTGGAATATGTATGGTTGTG	59.996	22	221	6	226
60.457	23	GTGAGCTCTTCCGTGTCCTC	59.993	20	202	19	220
60.096	20	ACTTGCACCACCACTTCCTC	60.159	20	154	485	638
60.03	22	TGCCACGTAAGATCTCCAAA	59.272	20	280	609	888
60.785	24	GAAAGAAATGCACACGCTACC	59.766	21	220	1136	1355
59.813	23	GTTGGCTGTCCCACTCATTT	59.973	20	213	1128	1340
59.736	20	TTTAAGGGTTATCACCTGATTTTC	57.757	24	164	621	784
60.119	20	TTAAACTTTCCATCGTCGCC	60.074	20	246	1461	1706
59.833	20	ACGTTCTTCTCCATCCCCTT	59.935	20	179	218	396
59.685	20	GTACTGGCGACAGCGATACA	59.895	20	241	462	702
59.67	22	TGAAATTAGGAAATAAGGAGGTG	58.625	25	246	345	590
59.962	20	TGCCACGTAATCACAGCCTA	60.28	20	154	419	572
60.11	20	TCTGAGATTTTCATTTTGTCGG	59.202	22	198	713	910
59.901	20	TCAAATGCTCAAGGATCAATTC	59.162	22	250	1428	1677
60.576	23	GGCTGTGTGATCATGTTTGG	59.967	20	216	987	1202
59.124	21	AACGAAATCGAGACACCGAC	60.119	20	235	90	324
60.693	20	GATTCTGCTCTCGTGAAGGC	60.104	20	227	3555	3781
60.617	20	TGATCAATGTGGGATCATGTTT	60.059	22	224	955	1178
58.388	23	TCATGCTTGAATCAAAACAACA	59.206	22	193	1	193
59.691	20	AGGTTGTCCTGATGGTGGAG	59.962	20	114	2186	2299
60.088	20	TCACACACTCAAACACACGC	59.321	20	237	361	597
58.729	21	TTTTGCCATTTTCGAAATCC	59.886	20	253	1274	1526
59.854	24	GGAAATAAGGAGGTATTATGAAG	59.555	25	156	837	992
59.998	20	ATTGCTTCCAATCGAACACG	61.032	20	102	3550	3651
59.75	20	CACGAGCCGCCTAAAATAAA	60.218	20	270	203	472
59.935	20	GTGATGCAGTGTTGCTGCTT	60.064	20	239	2253	2491
60.104	20	TGCGAATGATAATCAACATGAA	59.032	22	269	206	474
59.573	21	GCAATTTCTGTTTCAGTATTTCAA	57.598	24	280	1003	1282
60.051	20	AAATATTTCACGCCGCTGTC	60.103	20	246	843	1088
60.073	20	CACGTACACCGACAAACACC	59.922	20	207	783	989
59.708	20	TGATCAGCAGCTATGGAACCT	59.851	21	258	83	340
59.945	21	TGATGGAAGCAATGCAACTT	59.276	20	171	173	343
59.937	20	TTGGCAAAACCCTATTCAGG	59.931	20	222	2852	3073
60.132	20	TTTCGCAAGGTAGAAACCGT	59.747	20	132	1257	1388
59.993	20	ACTGAACTCAGCAGCCTCGT	60.205	20	193	2510	2702
60.044	20	GGCCAAAACATTGCTCCTAT	59.046	20	175	2715	2889
58.108	22	TGGACAAATATGGTGATGGC	59.205	20	246	186	431
59.622	20	TTGGCTCGTACCAAGAAAGG	60.241	20	265	2305	2569
59.699	21	ATATCACATGCATGACCGGA	59.763	20	188	332	519
60.225	21	GCCCTAAGGTTGACTCATGG	59.55	20	211	25	235
59.882	20	GTTCCGGGATATCATCATGC	60.126	20	271	251	521
60.246	20	CGTTTGGCGGATAGTTTTGT	59.996	20	261	195	455
59.67	20	CGAAGGTGGGTTCTTGTGAT	59.966	20	217	11	227

57.797	24	GGGATCCATTGAGGATGTGT	59.593	20	266	2685	2950
59.91	20	GGGAGGGAATCTATTGTGATTG	59.666	22	198	577	774
59.05	20	TTGGATGAGATGATGGCAAA	60.009	20	236	1	236
59.99	20	GTGCTGCCCAAGAACTTAGC	60.022	20	242	639	880
60.053	20	GGCTGCATTTTTATCCCAAA	59.907	20	256	510	765
59.787	20	AAGCGTAGCATTGAGGGAGA	59.978	20	214	474	687
58.82	27	CCCCGACTTCTCCTTTGATT	60.434	20	253	1984	2236
59.84	20	AGAGGCGATTGCTTAGGTGA	59.978	20	269	66	334
59.007	23	GTTTTCGTTGTCCCCACAAT	59.694	20	161	199	359
60.038	20	CCAAGCCTACCGAGTCAATG	60.647	20	209	952	1160
59.907	20	TATACAAGCTGTGGCGGTTG	59.752	20	273	50	322
60.132	20	CGTCCAGTGCCTGTGAATTT	61.096	20	258	2258	2515
59.797	21	GGGTCGACAAGCTTGAGAAA	60.375	20	184	10	193
60.11	20	GCAGAGAGGGAGGGAGAGTT	59.952	20	237	123	359
59.866	20	TGGCACCCCTTTGTAATCAT	60.192	20	270	1035	1304
59.939	22	TCAAGATTATGGTGGGGGTG	60.572	20	274	878	1151
60.232	20	CACGAATTCCCCGACTTTTA	59.931	20	171	990	1160
59.945	20	TCACCCGAATCTTTCGTTTC	60.051	20	207	496	702
60.152	21	CGATCGATCATCGGAAAAAT	59.862	20	114	16	129
59.871	20	ATGAGGCCCATCAACACAAT	60.203	20	241	116	356
58.736	24	TGGCTGATCAAGCTCCTTCT	60.096	20	131	14	144
59.953	20	TCCCAAAGTTTCACACGTCA	60.128	20	216	501	716
60.042	20	TGGGATTGATCCTACCCAAA	60.126	20	262	2459	2720
60.03	20	TTTTCATGAATTGCCTTCCC	59.878	20	263	2855	3117
59.96	20	TTTAATTAGGTCCACGCGCT	59.742	20	103	123	225
59.988	20	TTGATAGTCCGGTTTTTCGG	59.931	20	238	179	416
57.278	20	TTGGTAGGTGTCCGTGATGA	59.96	20	249	836	1084
60.035	20	AGTTAGCATGGGGAAGGGTT	59.827	20	199	16	214
60.209	20	TCATCCCAAGTCCAGAGCAT	60.622	20	259	2726	2984
60.353	20	TTTTTCAGCTCACTTTGTGATG	58.081	22	229	647	875
59.903	20	CCAGCAATAGCCCTCTGTGT	60.277	20	267	4389	4655
59.992	20	TGCAAATGGTGATTGCTCAT	60.08	20	265	365	629
59.924	20	GACCGTGTGCAATGAAACAG	60.16	20	211	166	376
60.144	20	GTGGCATGCAAACGACATTA	60.532	20	107	2	108
59.938	20	CTGCAGATCTAAGGCCGAG	58.709	19	146	974	1119
59.773	20	GCTCGAACTCGATCACAACA	59.992	20	236	66	301
60.307	20	TCGCCAAAACATGATGAGAG	59.799	20	280	33	312
59.988	20	ATCGGAGTTGATTTGATGCC	59.9	20	261	1600	1860
59.005	20	TGGCGATTAATTTCCCTTCA	60.395	20	154	406	559
59.739	20	TGTCCAACTCAAAGAGGAAAA	57.441	21	260	130	389
59.065	20	ACAAAAAGCACAAACCCACC	59.875	20	259	1619	1877
60.059	20	CCAAGTTGATGCACGAGAGA	59.984	20	222	911	1132
59.722	20	GCTACCTTGCGGAGTTCAAG	60.015	20	188	59	246
60.36	20	CTCTCTCCTCCCACAACCTG	59.828	20	144	160	303
60.004	20	TGTGCATCAAAATAAACCCAA	58.922	21	144	1559	1702
59.756	20	AAAGCAGGTTCCCCCTATTG	60.312	20	207	270	476
59.024	20	TTGGGATTTCACGAAAGATTG	59.924	21	231	14	244
57.999	20	TTGCCGTTTTGTTCATGTTT	59.062	20	235	1925	2159
60.035	20	GGCTAATTGTTGGCCTTTGA	60.074	20	146	268	413
59.854	20	CCGGATACGGATTATATCCAA	58.659	21	249	383	631
59.918	21	CCTTAGATTCCACTTGGGCA	60.066	20	125	371	495
59.769	20	ACCTCCAACCCTTATCCCAC	60.052	20	146	8	153
60.111	20	GTCGATTCGCCACCTTTAAC	59.574	20	202	226	427
60.195	20	AGTTGCAATTGCCCGAAATA	60.456	20	175	250	424
59.606	21	AAGCCTACATTCACATTCCCA	59.443	21	241	418	658
59.82	20	TCCAGCGACTCATGTTGTTC	59.837	20	274	727	1000
59.079	21	AATCAATGAAGGGAGGGAGG	60.264	20	250	251	500
60.081	20	CCCAAGAATGGGTTATTATTTGA	59.14	23	149	778	926

59.972	20	CGAAATGCATGATTGAGGTG	60.073	20	249	722	970
60.074	20	TAGAGGTGGACGAGGAGGTG	60.252	20	224	274	497
59.369	20	TGACATGGATTGTGCTTTCG	60.665	20	233	2	234
60.051	20	TTGAGTTTGGGGATATTCGG	59.756	20	198	922	1119
59.917	20	TTTCTGGAAGGACCACCAAC	59.943	20	236	502	737
60.263	20	GGTTTCGACTGGTAACGGAA	59.971	20	277	390	666
57.297	26	AAGCGAAAGAAAATGGTTTGAA	60.108	22	280	5	284
60.073	20	CATGAAGTGAAAGGGACGGT	59.966	20	205	182	386
59.831	20	TTGAAGTAGGGCATTGAGGC	60.214	20	261	435	695
59.752	20	GATAGTGGAGTTGTGAGCATCTG	58.878	23	280	56	335
59.903	20	TGACGACCGATAATTGCTCC	61.003	20	174	44	217
59.817	20	TACTGCAGTGGAGTTGCCTG	60.049	20	250	1413	1662
59.052	20	CCAAAAACTGATAAATGATGCTG	58.687	23	253	423	675
59.971	20	TTTTTGGCCTTTGATCTTCG	60.181	20	262	95	356
59.864	20	TTCGTGAATAGTGCTGCCTC	59.028	20	235	512	746
59.978	20	TACCCATTCTTCGCCACTTC	60.074	20	209	1569	1777
60.264	20	CGGGAGAAAATGAAAACCCT	60.291	20	140	1025	1164
60.645	20	CGGTCTCGGTTTGGAATCTA	60.066	20	253	150	402
59.359	20	GGGATAGTACCGGAACACGA	59.813	20	186	877	1062
58.727	21	CGAACCTGGGTCTCTCACAT	60.112	20	164	118	281
59.882	20	GAGGGAGGGAAGATATTCGG	59.859	20	266	1447	1712
59.835	20	ATCCACCAAAAACCTGTCCA	60.21	20	173	1066	1238
59.923	20	AGAGTGAGAGTTGGCCTCCA	59.986	20	280	230	509
59.992	20	CGCTGCAACAAATACACACC	60.18	20	256	978	1233
60.11	20	TCCAACCTTGTTTTTCCGAC	59.948	20	227	573	799
59.903	20	ATCACCGATTTGCAAACACA	59.972	20	222	162	383
60.227	20	ATAGGATTTCACTGCCTGCG	60.235	20	247	140	386
59.903	20	TTGTCAAAAACTGCCAGCAC	59.888	20	265	2699	2963
60.074	20	CACCTGATTTTCTCCCTCCC	60.822	20	224	1547	1770
59.66	25	TGCACGCAGCATATTATGAA	58.88	20	216	578	793
57.587	21	TAGGCCTAAATGGTCGCATC	60.06	20	278	519	796
59.962	20	CAAATCCAAACCACCATTCC	60.029	20	224	156	379
59.839	20	GATTCTGCTCGATTCCCAAG	59.773	20	154	1348	1501
59.579	20	TTTGGAAAAACAGAGCACCC	60.088	20	134	342	475
59.992	21	GGCTTAATGGCCAAAAACAA	59.943	20	272	3788	4059
59.583	24	TGACATTTGTCTAACGATGGTG	58.554	22	269	70	338
59.851	24	TGTGATGCTCAGCAGTGTTT	57.976	20	208	48	255
59.826	20	GAAGCATTGGTAAGCCCAAA	60.074	20	197	21	217
60.194	26	AAAACGCGCTATTTTAAACCC	59.549	21	255	1	255
60	20	TCACTGCATTAAACCTACTCCC	58.264	22	263	3910	4172
59.174	20	GGTCTGGCAGTCGTGTTTCT	60.307	20	271	2751	3021
60.21	20	CAGACAATCCCGGAGGACTA	60.065	20	221	2622	2842
59.966	20	TGACACGGTGATTTACGTGG	60.427	20	242	248	489
59.833	20	CCTAATGAATCCTCCCCCAT	59.974	20	278	1154	1431
59.82	20	ATCTAATGCCCCAACACAGC	59.962	20	239	1130	1368
59.918	20	CTGTGCGCTAAAGGTTTTCC	59.883	20	145	77	221
59.661	20	TCCACTCCAACCCTTCTTTG	60.081	20	261	5285	5545
59.957	20	TTGTCTTCATCAAACGCCAG	59.84	20	183	787	969
59.933	20	AGGGAGGTGGCTCCTGTTAT	59.957	20	183	383	565
59.586	20	TGATTGCGAAACAGGAAAAA	59.277	20	168	85	252
57.796	21	GCCTGCTACACAGGGTTTTC	59.74	20	198	27	224
60.24	20	GAATGATGTGATTCGGCCTT	59.9	20	229	546	774
59.956	21	TGAGAAGAACACATAAATTCGCA	59.772	23	280	1610	1889
59.702	20	GGTGGAGACAGGAGGGTGTA	59.962	20	231	39	269
58.912	20	TCCAGCTGTTAAAACCCACC	59.971	20	235	1001	1235
60.132	20	TCGGAGTCTTCAGACAAATCAA	59.853	22	274	1919	2192
59.521	20	TGTGCTTCAGTCCTTGCATC	59.992	20	277	4899	5175
60.014	20	ATGGAATACCGGTGCAAAAA	60.188	20	201	1558	1758

59.864	20	ATTCTCTCTCCCCCTCCAAA	60.008	20	226	51	276
59.926	20	ATGCTTTCGTTCCATTGGTC	59.939	20	263	213	475
60.14	20	AATCCTGGTTCCTCGGAGTT	59.935	20	130	674	803
58.954	21	TATTTGCAGAATTTGCCACG	59.702	20	185	246	430
60.008	20	ACACCTGGGTGAGACCAAAC	59.859	20	181	742	922
59.723	21	TCCGATGGGGTATTCCACTA	60.148	20	182	180	361
59.989	20	GGCTAGGCTTGTAGCAAAAGG	60.389	21	234	71	304
59.916	20	CGCATTTTTGGCCTGATATT	59.928	20	221	776	996
60.431	20	TTTGAAAACGTTTCGCTTCA	59.462	20	269	646	914
59.293	20	TCCTTAATTCATTAAATACATACC(	57.288	26	123	481	603
60.673	20	TGCAAACACAATGTCTGAAATG	59.632	22	269	698	966
60.147	20	CCCTCTCTCCCCTCACTCAT	60.605	20	240	58	297
59.439	21	GTGTGATTGGCAGTGACAGG	60.162	20	143	1036	1178
58.503	27	GCACGGCAGTGGAAGAATA	59.814	19	277	1015	1291
58.957	20	CTCAGTACAATGGAGTGATGGC	59.621	22	168	606	773
60.275	20	AGTCAGCCGAGGAAATTCAA	59.813	20	277	170	446
59.338	20	GCTGAGAAGATGGCCTTTTG	59.955	20	213	248	460
61.981	20	CGGCTGTCAACAGAAGTTCA	60.025	20	202	72	273
60.089	20	GGCATGTGGGGGTATAGTTG	60.074	20	256	1533	1788
59.934	20	GACACCCCAAGTCCAAAGAA	59.943	20	269	143	411
60.081	20	TATTTTCGGTTTATTGGGCG	59.799	20	171	76	246
59.939	20	TGGGACACTGTTTTCTTTGG	58.594	20	195	284	478
59.757	20	TCCCCTGCTTCTAAAACCCT	60.068	20	140	10	149
58.952	20	TGCCCAGATGTCAAGAATTG	59.648	20	271	223	493
59.362	23	GGATTTACGTGCAAGGGAGA	60.074	20	100	442	541
59.972	20	TGGCTTGGTCTTGATCTGTG	59.831	20	103	938	1040
57.299	27	TGGGAATAATGTATGAATGATCG	58.707	23	271	943	1213
59.74	20	CACGGTTGCCCTTTGTTACT	60.03	20	238	660	897
60.401	20	TGTTTGTGATCCCAATTGAA	57.35	20	234	608	841
59.989	20	TTGGTTCGAGCCATTGTGTA	60.111	20	155	44	198
60.081	20	TCCAGTGTTGCTTTGCCATA	60.257	20	254	1190	1443
59.941	20	AAGTCGAAATTTGGCAACAA	58.267	20	103	203	305
59.953	20	ATGAGCACATGCCAAAATGT	59.005	20	204	1838	2041
60.088	20	TGTGCTCCATGGCTGATAAA	60.22	20	226	189	414
59.258	20	GCTAATGGGAAAAGCATTGG	59.547	20	109	1461	1569
59.874	20	CATGATAATGCTGATAAAAATTGC	57.831	24	203	447	649
59.971	20	CTCCTTCTTTGGCTCAATGG	59.807	20	221	837	1057
59.097	20	ACTTGGAATCTGGACTCGGA	59.655	20	227	15	241
60.073	20	CAACCGGCAGCATTTTTACT	60.131	20	216	3487	3702
59.67	23	CGGGTGGGAATGTATTAGGTT	59.952	21	250	70	319
58.703	23	ATAAAAAGGACTGGCCTGGG	60.312	20	214	60	273
59.923	20	TTTGGTTGAAGGTGCATGTC	59.547	20	121	399	519
60.327	20	CTGCCCATTTGACTCCATCT	60.073	20	216	133	348
59.984	20	TGAGGCCACTGACTTGAATG	59.831	20	251	39	289
59.835	20	CAAATAATGTGGCAATCCCC	60.016	20	146	40	185
60.054	26	TTCATCTTGTAGCGTTTGAATGA	59.772	23	105	1	105
60.024	20	TGCATCATCTGCCATTTTGT	60.08	20	191	1438	1628
60.008	20	CACAAAGCTGCTCTCTTCTCC	59.367	21	280	27	306
60.544	20	CTACGCATGGTTTTCCCAGT	59.993	20	169	176	344
59.448	21	GGAATATGATCGGTCGCAAA	60.807	20	239	2	240
59.997	20	AAATCACAGGAGGACGATGC	60.081	20	182	850	1031
59.831	20	TTATCAACCCAAAACCCCAA	60.024	20	105	176	280
59.653	20	GCTAAAAGGGATTCTTGGGC	60.041	20	123	1186	1308
61.429	20	AATTTTGGAGATGGACCGTG	59.79	20	261	918	1178
59.9	20	ATCGCCTCCACCTCAAATTA	59.528	20	211	299	509
59.801	20	GCTGACTAACCCATTCTTGTTTG	60.048	23	240	917	1156
60.074	20	ATCTGAACAAATGGGCTTGG	59.933	20	205	2894	3098
59.96	20	ATACGACAGCGTGTTGGACA	60.183	20	255	1285	1539

59.861	21	CTACCCAAACGTCCCAAAAC	59.331	20	175	531	705
59.91	20	TTTTTGAATGGTGGGGGTAA	60.024	20	207	229	435
59.696	21	TCTTTCTTGGAACATCTCGTCA	59.853	22	255	595	849
58.922	20	CCTGGTTCATTTCAGGGAGA	60.042	20	155	211	365
60.142	20	AACACACAAACGAATGGCAA	60.011	20	275	404	678
60.241	20	AGGAAACCTTAAAGGGCCAA	59.939	20	256	1135	1390
60.129	20	GTGGTGGTGTGCGTTCATTA	60.436	20	176	185	360
60.081	20	TGAACATGCATCCAAAGGAA	60.049	20	214	214	427
58.605	23	ACCTGGAAACGCATATGGAG	59.955	20	260	2	261
60.056	20	CCTTTTCTGCATTTGGTGCT	60.249	20	251	584	834
59.875	20	GGGCTATCGACCTCTTTTCC	60.039	20	239	496	734
59.903	20	AACAGAGGCATTCCATGAGG	60.073	20	129	355	483
59.845	20	GCTTGAAGCGAAAAAGGAGA	59.708	20	218	228	445
60.79	20	CTCACCTGCACACACATTCA	59.265	20	280	1181	1460
59.989	21	AGATGGGGCTGTTGTGAAAG	60.111	20	244	2085	2328
59.789	20	TCAGTCAACCAACCAACTGC	59.726	20	212	21	232
58.601	23	ATTGCACCTCGTAAACACCC	59.859	20	111	22	132
60.042	20	AACTGGGAAAAGGAAACGGT	59.842	20	171	1532	1702
59.847	20	TTTACACCACAAAGTTGCCG	59.627	20	184	790	973
59.722	21	AAGGTCCTTCTCATTGGGCT	60.074	20	142	25	166
58.32	21	CCCATATTGGCAAGCATTTC	60.289	20	100	21	120
59.127	20	AACTCAACAACCCGTGAAGC	60.156	20	225	94	318
60.134	20	CGCGTGACAAATTCTTGAAA	59.849	20	154	275	428
60.175	20	AAGCCAGGCAGCATAAGAAA	59.982	20	212	47	258
60.833	20	TGCTTGACAAGTGACATTGAAA	59.366	22	146	446	591
59.948	20	ACTTGCAAATTACCAAGGGA	57.217	20	126	162	287
60.008	20	ACTGAAGCTCGAACCCCTCT	60.393	20	238	248	485
59.933	20	GTGGCAGGAAGCATTCACTT	60.263	20	260	1169	1428
59.875	20	ATGGGGAGGGGATATGGTAA	60.224	20	130	239	368
60.046	21	TGGTCCCTAATTCAACTTTTGC	60.344	22	208	68	275
59.742	19	GCTGGTTGCATACTTGCACA	60.876	20	205	8	212
58.315	21	TGATTCCTCCTACGGACCTG	60.065	20	159	2913	3071
59.882	20	TGTGCACTCGATGATGTTTG	59.248	20	235	2657	2891
60.06	20	CCAAACAACTCCCTACCGAA	59.964	20	233	481	713
60.064	20	TGTCACAAGCTCTTCATCGG	59.984	20	269	334	602
60.041	20	CTGATGCATGTGGTAGGAGC	59.271	20	220	4028	4247
60.238	20	CCATACAAAAGCGCCAAACT	60.131	20	188	1967	2154
60.244	20	CAATTGTAGCTCCTCCTAATTTCC	59.564	24	181	416	596
59.823	20	CCCAACATCATCCCTCACTC	60.326	20	174	150	323
60.448	20	GGTGGGCTTTTCTGACTCAA	60.232	20	271	19	289
59.587	20	AATTTTGGCAACCGATCTTG	59.938	20	212	2767	2978
60.044	19	CTACCCTTACCTGTCTGCCG	59.751	20	246	186	431
59.96	20	TCAAAAACCACAAACCACGA	59.981	20	222	445	666
60.277	20	GGCGGTATGCACTCAAAGTT	60.14	20	218	782	999
60.13	22	TTGGGTTTTGATGATTGCTTT	59.442	21	265	1016	1280
59.962	20	CCACCAAACCAGAAGCAGTT	60.149	20	161	3169	3329
60.151	20	TGTGGCCTTTTGTCACTTCA	60.278	20	280	131	410
60.449	20	ATGCATTTGGGAGGACAAAG	59.933	20	219	368	586
59.377	20	AGTGGAGTAGGCGGAAGGTT	60.132	20	277	832	1108
59.971	20	TTTAAAATGGCCTGTTCCCA	60.294	20	204	10	213
59.917	20	GGACGTCCAAAAACGGAAAT	61.08	20	231	455	685
60.003	20	GGGACTACCGTGATGGAAGA	59.927	20	138	76	213
59.875	20	CAAGTGAAATTGGATGGTGCT	59.985	21	219	355	573
60.431	20	GCTTGTTGTTCAAGCTGCTG	59.788	20	245	200	444
59.955	20	GGTGCGGGATCAATAGGTTA	59.784	20	203	4270	4472
59.869	20	AGGTGGGAGTGGAAACACAC	59.859	20	273	524	796
60.235	20	GTTGCTGTTCTGACCCGACT	60.307	20	140	465	604
59.846	20	CGGCATGATGCAGTAAGAGA	59.972	20	261	3250	3510

59.974	20	TCAAAATCCTGCCTTGTTCC	60.051	20	238	61	298
59.462	21	CCTTAAAACCCTCCTCGGAT	59.415	20	251	619	869
59.861	21	AAGGGCTTTGTGCATGAGAC	60.263	20	267	947	1213
60.074	22	TGCCTCAACTTTGTGTGCTT	59.49	20	264	1320	1583
59.894	20	TTGACATGTGGCATTGTGTG	60.003	20	155	1948	2102
60.315	20	TGTTGCACATTGCTTGTTGA	59.877	20	241	2179	2419
60.255	20	TCACAGGCAAATGACCAAGA	60.24	20	248	258	505
59.23	20	CTTCCACATGGATGAAGTGC	59.09	20	280	582	861
60.227	20	GAAGGCATGGCTCAGAGTTC	59.957	20	208	397	604
59.41	20	ACACCATGGTTTCCAATGCT	60.24	20	276	6	281
60.003	20	CCGTGATGGAACAACCTTCT	59.966	20	133	76	208
59.903	20	AATTCACAAGCTCACAAAACG	57.947	21	244	483	726
60.025	20	CCTAGCAAGGCTTCCATCTG	59.971	20	194	129	322
59.82	20	AAGGTGAAAGAGACATCAACCAA	60.026	23	105	539	643
60.155	20	CCGCAACTTGACAATGACAC	60.16	20	261	246	506
60.034	20	CATTATCTCAAACGAGCGGC	60.742	20	246	166	411
60.223	20	TGAATTGGCCTAAGCGAAAG	60.335	20	167	210	376
60.154	20	CGAACAAGAGTCTTCCGAGG	59.982	20	142	589	730
59.109	20	AAAGGCTCCTCTCTTCAGGG	59.95	20	163	2437	2599
60.755	20	GGAACTGCACAGGGTGATCT	60.12	20	133	617	749
57.15	24	GCAATGCATCAGAGACTCCA	59.95	20	228	161	388
59.955	20	TCCTAGGCGCTATTGAGGTG	60.365	20	276	92	367
59.065	20	CACATAAGCGAAAACGCAAA	59.88	20	237	3694	3930
58.927	26	TTAGTTTCATGAATGGAAGTGAAT	57.035	25	100	5	104
60.033	20	GGATCCTGATTCTCGACCAA	60.011	20	233	206	438
59.893	20	TGGAATTGCTACACCTGCAT	59.152	20	203	623	825
57.617	26	TGTTGACGAACGAGACAAGG	59.873	20	228	60	287
59.708	20	TTTCGACAAATCATTGCAACA	60.103	21	211	1310	1520
59.702	20	CGAGCACAAGTTCTCCACAA	60.025	20	131	2556	2686
60.018	20	AAGGCAGCATGCTTGAAACT	60.022	20	260	2003	2262
60.156	20	GGAAGTCCTGGCATTGTCAT	59.934	20	242	225	466
59.415	20	TAGGGATTTTGAAACGGTGC	59.938	20	264	27	290
59.673	20	TGGACCATTTGTGCACTTGT	60.008	20	262	1649	1910
59.801	20	AATTGCCCCTACATGCTGAC	59.962	20	242	387	628
60.31	20	TTTTGTGATTCAGGGTTCCG	60.863	20	280	221	500
60.172	20	TTGGGGATGAGATGGGAATA	60.088	20	227	6625	6851
60.145	21	AATGGCCCAGATTTGTTTTG	59.801	20	145	30	174
59.635	20	CGAGAAAAGACAAAGAAGGGAA	59.865	22	103	1325	1427
60.49	20	CATGAAGTGAAAGGGACGGT	59.966	20	259	9	267
60.159	20	CGTAGTTTAATTTTGCCGGG	59.489	20	209	871	1079
57.037	20	CTTGCGAGCAACTTGTCAAA	60.172	20	224	280	503
60.212	23	TTGGAGTCCATGAGCATCAG	59.787	20	268	36	303
60.074	20	GGGGTAAAATCGAGGGGTAA	60.013	20	221	1423	1643
60.397	20	TTGGGGAATGTTGAGAAGGA	60.43	20	269	966	1234
60.111	20	TGCTGGCATCAAAATTGGTA	60.073	20	184	560	743
59.753	20	AAGAGAGGACACCTCCGGTT	60.111	20	160	198	357
60.112	20	GAGTTGCATGATGGCATGAG	60.233	20	138	230	367
60.177	20	TTAGGGATTAAAAGCGGCAG	59.341	20	219	88	306
58.494	26	CTTGTGAAATGAATGCACCG	60.111	20	172	535	706
60.088	20	TATCGTTTTGCCGCCTTATC	60.061	20	256	1129	1384
59.81	20	GCACGCATTTGTGATTTTTG	60.118	20	217	1	217
60.128	20	GAGTTTGAGGCCTGACGAAG	59.989	20	126	262	387
58.935	23	CACAGTTCACCCCCATTTTT	59.688	20	224	198	421
60.241	20	TTGAGGTGTGGCTGGTATTG	59.566	20	238	214	451
60.461	20	GCAAACCACGCCAAATAGAT	59.967	20	252	567	818
60.06	21	GCCAGCAATAATCTCAGTGGA	60.228	21	264	700	963
60.312	20	CCTAGGCATACCAAGCCATC	59.551	20	154	1	154
60.176	20	TGCATGGCAAAGCTTCAATA	60.356	20	233	1032	1264

59.784	20	AGCCGATAAAAATGGCACAC	59.967	20	231	689	919
60.241	20	GATGAGGGTTGAAGTTGGGA	59.903	20	250	335	584
59.982	20	GATGATGAAGGCCAGACGAT	60.042	20	261	350	610
60.766	20	CAGAATGGTGGCAACAAAGA	59.691	20	133	586	718
60.046	20	TCGGAGACGAGAGAGGAGAG	59.81	20	190	574	763
59.614	20	CCAGGGCAGTTGTTTCAAAT	59.971	20	238	307	544
60.048	20	CCTCACTTCACCCTCACCAT	59.962	20	138	786	923
59.234	20	CGGGGTCGTATCATATTTGG	60.031	20	115	475	589
59.517	20	AATTTGATTGATGGTTTCGGA	59.264	21	105	130	234
60.975	20	GTACCAGCAGAAGACTGGGG	59.721	20	238	1407	1644
59.993	20	CGAGCATCAGTGAGACATCC	59.372	20	280	825	1104
60.146	19	CGACTCCCTTGTGGAGAAAC	59.697	20	275	251	525
60.268	20	TCCTTTTTGTATTCAAACTCAACAC	58.708	25	202	513	714
59.916	20	CAAAACCAACAGCCGAGATT	60.11	20	149	3	151
60.356	22	ACGTTTTGGTTGTTTGGAGC	60.015	20	162	2397	2558
59.826	20	TTTTTGACTTTTTATGGGGGT	57.539	21	264	198	461
58.385	22	AATAAGGGGGTGCATGTGAG	59.813	20	175	225	399
59.933	20	CGATGTCCAGAGGAACAACC	60.51	20	170	146	315
59.992	20	TTTGTGTCGCAAGGTGAGAG	60.025	20	267	2663	2929
59.685	20	TTTTCTTCACCCAAATTTAATCG	59.404	23	276	487	762
59.972	20	GACGAAGAAGGTGTTGGTGG	60.545	20	264	197	460
59.74	20	TTGACAATATTTAGTGTTTTGACAT	58.99	27	173	543	715
58.729	25	ACGCGTTAACTAACCGGGAT	60.741	20	261	1134	1394
59.969	20	GCAGGAAGAGAGGAGAGGGT	59.952	20	230	742	971
60.15	20	GGCATGTTTCGAGTTTGGTT	59.978	20	233	313	545
58.995	24	GATTCCGGGTTCAATACTCG	59.387	20	280	484	763
60.261	20	GAAGCTGCATTGGATGAACA	59.805	20	158	2124	2281
59.722	20	GGTTTCGTCATAAGGCGGTA	59.96	20	264	538	801
59.951	20	CAAATGTCTTCATCCCCACC	60.173	20	275	1652	1926
60.436	20	AAACCCCCTCTTAAGTCCTGA	59.088	21	254	292	545
60.081	20	CACCATCACCTCCTTCCCTA	59.92	20	241	960	1200
60.006	20	GTGGACGCTCCAGATTGTTT	60.119	20	113	29	141
60.045	20	GCCGATGATTTGCTACGATT	60.067	20	183	2942	3124
59.957	20	TGCTCACTCATCCTCCAGTG	59.98	20	219	699	917
60.042	20	ATTATCCAGTTTGGATTTTGTTCT	57.292	24	135	1896	2030
59.041	20	GTGTACCCTGAGGGACGGTA	59.844	20	146	93	238
60.181	20	AGTGGCATGATAATAGCGGC	60.088	20	255	419	673
60.299	20	TTTTAATGCTTTTCCGTGGG	59.937	20	190	2093	2282
60.044	20	TGGAAAGGTACGAGGTGGTC	59.966	20	187	402	588
59.805	20	TCAGCTTCAACATCACCAGC	59.992	20	264	1859	2122
59.901	20	CTATGTAGGGGCGTGCAAAT	59.982	20	203	29	231
59.592	20	TCTTTCATTCAACGGTATTGTCA	59.505	23	193	629	821
58.721	20	CCAATTTGAATGGTTATCACCTG	60.457	23	102	628	729
60.012	20	CAGCTCATCGTGGATGCTAA	59.972	20	170	1187	1356
60.453	20	ACCATAAGGCGCACAAAAAC	60.003	20	141	1193	1333
59.71	26	GTCAAAGAGACAGGGGTTCG	59.697	20	252	10	261
60.69	20	TGCATCCAACGCTACAAATC	59.694	20	143	38	180
60	20	TTTTAACTAACTCCGGCCCA	59.581	20	175	739	913
60.001	20	GTTTGGACAGATTGCCTGGT	59.973	20	272	1005	1276
59.499	20	ATGGCATCAGGAGGATCAAG	60.034	20	254	2279	2532
60.269	20	ACCAATGGTGGTATGGTGTG	58.984	20	132	181	312
59.679	21	TCTTGGAGCGACTCCTCACT	60.135	20	245	929	1173
59.694	20	GTTTGCTGACGCAGGGTATT	60.14	20	210	501	710
59.992	20	GATCGAACGGGTTCACCTT	59.918	19	175	2468	2642
60	20	GGCACACATTATGGTTTGGA	59.251	20	251	163	413
60.05	20	CCACATCGGTGAAGGTAACA	59.415	20	200	210	409
59.67	20	TTCACACCTCACCATTCGAG	59.676	20	161	443	603
59.851	20	ATTGTTCTTTCCCGTGCTTC	59.174	20	274	1937	2210

59.257	20	TGAGTCAGCACTGTGGTTCC	59.872	20	155	409	563
60.088	20	TTCCCTTTACTTCTTCCCCC	59.393	20	265	495	759
60.255	20	AGAAAAGAGTTTCAGCGCCA	60.132	20	214	1078	1291
59.805	20	AGCAGCTCGATGGAGGTAGA	60.119	20	246	2153	2398
60.321	20	GGGATAGTATCGGATCGCAA	59.885	20	129	69	197
59.24	21	AGAGTGATCAACCGATTGCC	60.081	20	200	14	213
59.924	20	TTGTGCCACATTTTTCCTGA	60.088	20	185	742	926
59.964	20	AACCCCACCTGTGCTAACTG	60.028	20	190	429	618
60.159	20	TTTGGGACGCTCTATTCTGG	60.206	20	108	258	365
59.705	21	CCGTCAACCAAACGTATTCTC	59.485	21	276	720	995
59.982	20	CCTGCACCAAATTTTCCATT	59.801	20	131	158	288
60.757	20	TCTATATGCCCAAGCCAACC	59.923	20	259	601	859
59.894	20	TGCTCATGAAATATCGTTGG	57.162	20	223	267	489
60.605	20	TGTCCCCGATAAAAGAAGGA	59.5	20	279	1149	1427
59.762	20	TTTTAAATGGACGTAAATGGATTG	59.218	24	126	15	140
59.924	20	TTTAAAGTGTTGAGCGCGAA	59.625	20	235	60	294
59.955	20	CGTTAGTCGTTCCTGGGCTA	60.262	20	189	2968	3156
59.927	20	TTTTGAGGTTCGGACCAGAC	60.088	20	181	265	445
59.097	20	CCTGATTAGAAGATGGTGATGAA/	59.502	24	207	740	946
59.915	21	CGCAATTATATTAACTTTGCGTCA	60.375	24	268	1478	1745
59.989	21	CCACGTGGAATTATGGGAGT	59.67	20	177	1708	1884
60.081	20	AGAGACTGTTGAAGCTCGGC	59.751	20	239	34	272
59.903	20	TCCTAGTGTCTTGTCTCCCACA	59.762	22	183	1017	1199
61.201	20	AATCGGCAAAAACCACAAAC	59.845	20	220	741	960
59.813	20	TGCTGAAGAATCTGACACCG	59.984	20	276	217	492
58.019	20	GTGATTCCTGAGGCCATGAT	59.893	20	229	1692	1920
59.415	23	GTTCCTGCTTTGTGGTGGAT	59.973	20	277	966	1242
59.942	20	GTCCCAACTGAAGTTCCCAA	59.943	20	166	283	448
59.651	20	TCCACAATGGAAAAATGCAA	59.907	20	209	2756	2964
59.992	20	TGCTGGTGATCAAGTGGAAA	60.24	20	254	367	620
59.955	20	CTGGAGCGGTAATCACAAGG	60.647	20	147	566	712
59.367	20	ACCCGAGACAAAAACCACAG	60.005	20	163	10	172
59.955	20	GTGAGGGAATGCTCTCCAAG	59.803	20	280	83	362
59.483	20	TTCCTCCAGAGGCTCAGTGT	59.986	20	203	2805	3007
59.945	20	ATACCTTTCTTTTTCCCGCC	59.442	20	182	2231	2412
59.827	20	GACCAGAACCTAATCTTGGGA	58.108	21	270	536	805
60.154	20	AAAAATCAAAGGCCCACAAT	58.418	20	264	591	854
59.942	20	CGTGTTATCCTACAGCCCGT	60.015	20	217	2625	2841
60.044	20	TACTCAATCAGCTCCAGCCC	60.362	20	202	1992	2193
60.073	20	CCCCTCATGTTGGTGGTAAT	59.526	20	271	255	525
59.988	20	AACATCATCGAGGTCGGTTC	59.934	20	192	919	1110
60.214	27	GAGACCAATTTTGCTATGTAATTTT	58.304	26	226	234	459
60.444	20	GAAATAAGGATGTGTTATGAAGG(	59.656	25	145	512	656
60.118	20	AAAGAAAGCTTCAAGCAGCG	59.904	20	135	122	256
61.296	20	CCAAGCCTGGAGACCTAAGA	59.425	20	220	20	239
60.34	20	TGAGAATGGTGGGTGTGAAA	59.935	20	231	622	852
59.934	20	AATGCAAACCAGTGAAAGGG	59.971	20	166	1158	1323
60.088	20	CAGATTCTGGCACTGAGCAA	60.136	20	275	5356	5630
59.957	20	CACTAGATTTCTGCACCGCA	60.011	20	275	237	511
60.074	20	AATCTTGGGTCCAAACCCTC	60.169	20	230	248	477
59.691	20	GCTTGCTTCCACCAGGTCT	60.402	19	279	155	433
60.173	20	CGTCCATGCTCTGTTCTTCA	59.984	20	134	887	1020
60.17	20	TTCAGCCTCCATTTACCTCA	58.298	20	255	2688	2942
58.028	22	ATCGAGATGCTCTGATGCAA	59.507	20	277	20	296
57.372	25	AGCTAGACACGTGGAGACGC	60.621	20	249	931	1179
59.902	20	AGGGGAACGAAAATCTCTCC	59.513	20	221	2789	3009
59.575	24	TGGGTGATCAGGAAAAATCA	58.897	20	171	60	230
59.133	20	TGCGAGTGTTGCATCTGAAT	60.423	20	236	314	549

60.397	20	CCACGAAGAACCTGGTTTGA	61.064	20	277	866	1142
59.727	20	GGAGGACACCATTTCTCTGC	59.661	20	182	7747	7928
59.579	20	ATCGAAATCTAAACCCGCAC	59.046	20	251	672	922
60.324	20	TGAAGTTAGGAAATAAGGAGGTG	58.743	25	276	558	833
60.011	20	ACACAAGTATGACCAGGGGC	59.851	20	182	19	200
59.951	20	AAGAAAGTATCAGGCGCATCTT	59.42	22	187	26	212
59.973	20	CCGAAGGCAACACCACTACT	60.171	20	266	2007	2272
59.109	25	TGTTGAACGTGTACGTTGGG	60.463	20	228	590	817
60.223	23	AGCACGTGTGAATATTGGGC	60.925	20	255	299	553
59.779	20	CCTTCACACGATTGGAGCTT	60.255	20	275	660	934
59.803	20	ATTGTACACATGGTGTGGGC	59.143	20	151	1611	1761
59.874	20	TTTAGTGGCTGGAACCGAAG	60.241	20	220	517	736
60.205	20	TGGCTCAAAAAGTTTCATATTTCT(	60.022	25	119	1980	2098
59.922	20	AAAAAGCCGCACTTACGAGA	60.018	20	251	11	261
60.187	20	TTGGGTTAATCCGTGTAATCG	59.707	21	230	361	590
60.543	22	AAGGAGGAGGTGAAGAAGCC	59.817	20	265	23	287
59.369	20	TTGGAGAGTTGGGTACAAGC	57.797	20	135	176	310
60.033	20	TGCATGCCGCATACTATTACT	58.368	21	241	1620	1860
60.615	20	TTTCCTCTTCCGATTTTTGG	59.131	20	253	918	1170
59.801	20	GATGTATGTGCGATGGGATG	59.763	20	179	825	1003
60.158	20	CCCCCAATTTGTTTTACTCG	59.311	20	223	1381	1603
59.984	20	CCTAATAAAATGGCCATGCG	60.295	20	147	1623	1769
59.875	20	CTTCTGGTTCCAGCAGCATT	60.397	20	124	252	375
58.94	20	CGGAGACAAAGGGTCATTTC	59.526	20	239	174	412
59.926	20	CCGCTGACAAACATCATCAC	60.12	20	230	5360	5589
60.177	20	CTCGATCAAGACCCTATCGC	59.797	20	183	1073	1255
59.989	20	TCGGATTAAAGCTAGGGCAA	59.81	20	279	1048	1326
60.008	20	AAACGAATCTGTCGGTGGTC	59.973	20	264	2696	2959
59.971	20	CAAACAAAAGGGCCTTCTCA	60.22	20	236	962	1197
60.232	20	TGGTAGGTTTTGGAGGGTTG	59.824	20	280	659	938
60.052	20	CACTCGAACCAGTCAAACGA	59.873	20	228	732	959
59.847	20	GACCACAACCAACCACATGA	60.27	20	246	742	987
60.096	20	CTCACGCGAGGTCTTTCTTC	60.134	20	153	34	186
60.089	20	AAATAGATCTTTTGTAAAGCCCG	58.02	23	112	2970	3081
58.813	25	ACAAATTTGCAGATGGAGGG	59.933	20	198	41	238
59.935	20	GATGAGGAAGAAAGGGAGGG	60.008	20	253	1010	1262
59.923	20	CGTGGCCTTGATTAAAATGG	60.315	20	201	442	642
59.091	20	GGAGGGAGAGAGGAGGAGAG	59.489	20	251	149	399
59.542	20	GCATCGTCTCAACCCTTGAT	60.081	20	184	660	843
59.831	20	CTCCCTCCAAATCGTTGCTA	60.206	20	101	634	734
61.146	20	TGATAGGGCACACAACCCTT	60.375	20	280	520	799
59.993	20	TGAGATATTTAAATCCTTCCGTTT	57.452	24	249	99	347
60.349	20	TCGTCCTTTCGACATTCTCC	60.195	20	169	2049	2217
60.366	20	GACTCCCATTCCCAAGCTCT	60.596	20	216	2437	2652
59.923	20	CGGATTTCGGAATTGATGA	59.415	19	215	634	848
59.978	20	GTGCCTTCTTCTAACTGGCG	60.015	20	220	502	721
60.053	20	TTCGATTCAACAAAGTCCCC	59.91	20	224	272	495
60.169	20	CTGAGACCCCTCATGTTGCT	60.261	20	197	425	621
60.081	20	TCCAAGTTGCCTTTCAACCT	59.711	20	173	623	795
60.074	20	AATTTGCATTGGGTTTCGAG	59.938	20	108	279	386
60.02	21	CTTCGAAGCGATGGAGTAGG	59.971	20	101	20	120
59.861	20	GTCTCCATGCTTGAGCCAAT	60.226	20	277	431	707
59.415	20	TAATTAAGTCATAGAGCAGGGGC	58.853	23	100	24	123
59.716	20	TCACGTCTTGTTGCATCACC	60.733	20	210	578	787
60.144	20	AGGTGGAACAAGTAACGCAA	58.283	20	207	449	655
60.074	20	TGAACCGGGTCTCTCGTAAC	60.111	20	174	2646	2819
58.214	20	CAGCCCTCAGATTTTAGCGA	60.481	20	277	410	686
60.634	20	TCGGTTGACCGAAAATAGGT	59.429	20	200	85	284

59.645	20	GGATGTTTCATTTTGCCTGG	60.309	20	249	559	807
59.885	20	TCCTCCATTTCCCACAACTT	59.381	20	230	293	522
60.088	20	GGCACATCGTGTGAATCAAG	60.12	20	162	242	403
59.871	20	CGGACGTTAAACGAAGGAAA	60.103	20	280	287	566
60.439	20	TTGTTGTAGTATGGCAAGATCCA	59.527	23	199	383	581
60.095	20	CCCAAGGCGAGCTCTAAGTT	60.884	20	201	139	339
59.48	26	CAGTGTAAAACCTTATTGGGGG	59.641	22	268	123	390
59.241	20	TCAATGCTCTTTTCAACCCTTT	60.109	22	209	403	611
60.214	21	TATCCTTGGATGGAAATCGC	59.862	20	183	805	987
59.365	22	ACTTCCCAGTCGTGGATGAG	60.112	20	210	337	546
60.119	20	TCCTGAAGAATTTCGGCATC	60.155	20	250	250	499
59.989	20	CCCCTAATCCGAACACAAAA	59.795	20	158	778	935
59.972	20	CCTCCCCAAATATCCCGAAT	62.062	20	278	2455	2732
59.927	20	AGTTCAATAGAGGCAGCGGA	59.978	20	217	797	1013
59.964	20	ATCGAAGATGAAAAAGGGCA	59.645	20	151	247	397
59.956	20	AACGGCCGAGATTTGTTATG	59.96	20	255	577	831
59.044	22	CGATTTCTACACCATTGTTCCA	59.861	22	211	2363	2573
58.227	20	GTTCGGGTGTGCAAAAATTC	60.352	20	275	361	635
58.571	24	AGGTGAATCCGATGATGAGC	60.042	20	181	3	183
60.074	20	CTCTTCCCTGCAGATGATGG	60.758	20	280	1442	1721
60.103	21	ATGACTTCCAGTGCCAGGAC	60.12	20	175	894	1068
58.808	22	TGCTCCAAAATGATTACGTGG	60.871	21	280	575	854
59.997	20	CCCGACAATTTCACCCATAC	60.051	20	234	239	472
60.019	24	CGTGGTACTCGAGCTGTCAA	60.049	20	219	8	226
59.967	20	CTGCTCCTGGAGTACATGACAA	60.312	22	228	309	536
59.734	20	TTGACACGAATATGACACAAACA	59.003	23	191	276	466
60.065	20	CTGCATCACCTTGCTTCTCA	60.136	20	177	733	909
60.34	20	GCTAACCCTCCGCTGTATGA	60.235	20	269	902	1170
60.005	20	AATGCTTCGGACTTGCTCAT	59.843	20	273	714	986
59.936	21	ATGCAAGAAAGACGTGGGAC	60.119	20	278	410	687
57.736	20	AAGACCCTTGTGAGTGGTGG	60.002	20	215	24	238
57.865	20	GGATAGGCGGTTGAGATTTG	59.528	20	221	1491	1711
60.13	20	TTGTTGTGATGTGAAGAATTGTAA	57.312	24	190	270	459
58.881	24	AAAATCAAAGGCCCACAATG	59.801	20	119	1264	1382
59.751	20	CACAACCAAACCAACCATCA	60.255	20	236	1730	1965
60.191	20	CTCGGATTTGGGTTTCTTCA	60.044	20	231	1278	1508
60.012	20	TTGAAACGGAGGTTTGGTGT	60.388	20	207	493	699
59.07	25	AAAACCGATGCAATTATGCC	59.801	20	280	101	380
60.059	21	CCGTTTGCAACAATTGAGAA	59.706	20	246	1465	1710
60.315	20	CAACATGTCGGCATGATAAA	57.988	20	246	286	531
60.3	20	GCATCGTTTATCCGATTGCT	60.067	20	248	16	263
59.996	20	TGTAAAATCAATGCACCGGA	59.93	20	252	473	724
59.993	20	ACAGGGGAGAGAGACAAGCA	59.986	20	192	36	227
59.995	20	CGCTGGTTTTGGAGATCAAT	60.074	20	157	526	682
59.745	20	TATAAACGCTCGTCTGCTGC	59.234	20	206	42	247
60.153	20	CAAGGGTAAGGGAGGTGACA	59.959	20	210	750	959
60.407	20	ACCGATCTCACCCCTTTTCT	59.935	20	134	585	718
60.008	20	TGAAAAGGCTTCAATTTGGG	60.046	20	184	679	862
59.836	20	GAAGAGTGTTTGGGGCTGAA	60.232	20	153	2110	2262
60.22	20	CCTTCATTGGGCAGTTTTGT	59.971	20	215	297	511
58.028	22	TTGCTTTGGGTTCAAATGGT	60.344	20	271	16	286
59.425	20	CACTTCAACACTCCCCACAA	59.565	20	267	2814	3080
59.988	20	ATCGGTTATTACCGGATCGC	61.031	20	157	601	757
59.373	20	CCAATTTGAATGGTTATCACCTG	60.457	23	253	450	702
59.848	20	TCGAGTTAGCGTGTCGTGTC	60.056	20	123	306	428
59.707	21	GTGGAGGGGGAGTATGGATT	60.015	20	265	140	404
59.224	20	CAAATGACAGAATCGCGGTA	59.688	20	246	95	340
59.49	21	GCAGTCAAAGGAGGAAGACG	59.989	20	269	573	841

60.012	20	AGTTCACAACCCTGAATGCC	59.973	20	168	690	857
59.797	20	GAAATGTTCACACACGTCGG	60.008	20	221	262	482
60.332	20	ATTGGCAATTAACCGTCAGC	59.967	20	124	394	517
59.853	20	GAATTTTGCCACCGATGTTT	59.807	20	109	1638	1746
60.088	20	CCTTGGGCAACTCACTCATT	60.111	20	262	42	303
59.955	20	TCCGGCAAAAACCAAATTAC	59.81	20	240	2671	2910
60.285	20	GTGGAGGGTTTTTGTCTCCA	59.943	20	278	3387	3664
60.073	20	CCATTGTCGTCTCCAACCTT	59.966	20	226	877	1102
59.894	20	TGCTGCTCTTTTCTGGGTTT	59.993	20	133	77	209
60.041	22	GCTTCAACGGTATCCAGTAACA	59.158	22	125	1000	1124
59.926	20	GATGCCAACGTCATATTTGC	58.997	20	252	3617	3868
60.073	21	TTGGCACGAAATTAGTTGTTTG	60.034	22	214	221	434
59.352	21	TCCCCCTCTCTCTCCTTCTC	59.883	20	127	10	136
59.146	20	TGTTGCAGCTCTTGCTCTTG	60.472	20	279	137	415
59.997	20	TTTACGTGTTTGCGGATTTG	59.603	20	258	276	533
59.996	20	GGCCGTTTTAAAATCGAGTG	59.586	20	206	703	908
59.906	21	AAATGCATGCACCTGATTACA	59.055	21	169	913	1081
59.697	20	GAGCTGTTATGCGTTTTGATGA	60.272	22	238	1700	1937
60.331	20	GGTTTAACTTTTCAAATGGAACAA	58.903	26	276	144	419
59.95	20	GGGCCTTGAAGTTCTCTGTG	59.844	20	134	271	404
59.986	20	ATCACCTCATCACCGCCTAC	59.957	20	222	191	412
60.081	20	ATGAAACACCCCCAAACAAA	60.066	20	208	3583	3790
59.41	21	ATCCGGAAGGTCCAAAACTT	59.805	20	221	564	784
60.218	20	TCCAACACAAAACTCTAAATTTTC	59.821	25	228	537	764
58.561	25	CGTCGAGAGATGCAACGTAA	60.011	20	106	8	113
60.104	21	CCTCACGTGATATTTTTAGCACA	59.197	23	188	914	1101
59.926	20	CGACGTTGCAAGAAAAGGTT	60.284	20	218	293	510
60.552	20	TGCAATGTACAGCAGAAGTGG	59.92	21	268	458	725
59.993	20	CCTTTCTCTCTCTTCCCCGT	59.811	20	158	35	192
60.06	20	CCAATTTGAATGGTTATCACCTG	60.457	23	130	807	936
60.014	20	ATCCCCTTCTCTTTCCCGTA	59.897	20	179	1209	1387
60.272	20	TAAAGGTCGAAAATGGCGAG	60.202	20	186	254	439
60.112	20	TCCCCTTTTAAGGTTGATGG	58.876	20	269	1063	1331
60.003	20	ATTAGGAAAAGGGGCAGACG	60.443	20	158	1269	1426
60.263	20	GCAGCACCCTTTCTAACAGC	60.022	20	167	2995	3161
60.255	20	TGCAACCATGAAAACAAACAA	59.999	21	275	3149	3423
60.051	19	TGCCGGAAAACACCAAAATA	61.214	20	280	390	669
59.929	20	ACGTGTCAGAATCTGGGCTT	59.727	20	223	611	833
60.203	20	CCGGTTCTCCTATCCGATTT	60.278	20	175	628	802
60.144	20	CCACTTTTGAACGTGTGTGG	60.041	20	249	2452	2700
61.304	20	GTTAAAAGTGGCAAGCGACC	59.752	20	247	145	391
60.567	20	GCTTGAGATGGACTACCGGA	60.218	20	279	45	323
60.035	20	TGGAATGAAGGGGTGAGATT	59.336	20	265	77	341
59.825	21	GCTGCTTTCTCGCTCATTTC	60.243	20	230	1377	1606
60.081	20	TCGCCCATTTTCTCAACTTC	60.192	20	272	685	956
59.95	20	ATCGGTTATCACCGGATCG	60.697	19	175	3832	4006
60.043	20	GGGATTCAGGAAACAGGACA	59.903	20	256	3394	3649
60.159	20	GCCGCAAAGACTTAATCTCG	59.982	20	150	972	1121
59.942	20	TTGCCACGACCAAAACCTAT	60.365	20	274	263	536
60.603	20	GCATCCTCCACGTTACCATT	59.82	20	157	269	425
59.72	21	TCCGACCTTGAAGAATGAAGA	59.799	21	279	951	1229
59.875	22	ACGACATACACAAGGGGCAT	60.263	20	233	661	893
60.261	20	GCATTGAATGAATGGAGGTTC	59.382	21	169	39	207
60.103	20	CGGATTTAAGGGTTATTACCTGAT	58.81	24	231	1785	2015
60.158	25	TCTGGAGGAAAGCACCAATC	60.195	20	114	19	132
60.017	20	TTTTCACCCGACAAATCACA	59.941	20	262	305	566
60.218	20	AACCTCATTGATTTGCAGGC	60.081	20	248	593	840
60.128	20	CTGCATCTTGTGTCCTGGAA	59.831	20	269	1370	1638

57.857	24	TGAGATTTGGGCAAGCTTTT	59.823	20	255	288	542
58.287	23	TGAAATCCAAATAGGTTAGAATTG	59.664	25	198	1012	1209
59.593	20	TGATATATGGCGTGTTGCGT	59.983	20	224	2209	2432
60.272	20	CGCTGGTTTTGGAGATCAAT	60.074	20	159	525	683
60.032	20	GGAAGGCAAGGAAACAACAC	59.574	20	272	578	849
59.973	20	AATTCTTGCACGCCATTTTC	60.081	20	237	653	889
60.11	20	CACGAAACGAGATCGCAATA	59.833	20	186	10	195
59.931	20	TGATTCATTTTCTGCACGGA	60.197	20	190	131	320
60.118	22	AATCGTGCTATTTTTGCCTGA	59.734	21	238	254	491
60.11	20	TCCTTGGCTGACTAGGTGCT	60.012	20	164	977	1140
59.127	20	AAAAATATGGCATCCCGGTT	60.389	20	233	539	771
59.955	20	GCTTTACGAGCTGTTGTCCC	59.882	20	212	931	1142
59.984	21	TGTAGCAGCGAAATGCAGTC	60.165	20	268	1119	1386
59.047	23	TCTTCCGGATTTTAGGAGGC	60.522	20	256	10	265
60.025	20	AGTGAACCCAGTGGGAGATG	59.962	20	226	725	950
59.962	20	GGGATTCTGGAAGATGCAAA	60.014	20	264	2265	2528
59.755	21	TCTAGGGGGTTGTTGCTTTG	60.103	20	230	1699	1928
58.825	22	TTCGGTTAATCCAATGGTCA	58.818	20	251	782	1032
58.882	20	GACAAAGTTGTAATTTGCGGC	59.644	21	270	907	1176
60.11	20	ACACAAAATGGCAATGCAAA	59.976	20	271	1116	1386
60.37	20	TTTATCGCACAAATATTTTGATTTT	58.231	25	272	403	674
58.326	25	TTGGAATGAATAAATGGAATGAA	58.357	23	255	17	271
59.345	21	GCTATATTTCAACCCCGCAA	59.928	20	152	1685	1836
59.784	20	TGCCGACTTTGTGGATGTAG	59.716	20	133	85	217
59.565	23	TTTGATAAAAATTTGGCGGC	59.913	20	276	5	280
59.942	20	TTCTCTCTCTCCCTCCACGA	60.064	20	278	752	1029
60.11	20	ACATGCAGAAAGAAGCCGTT	59.882	20	263	807	1069
60.46	20	CCACTTTCAGCTCAATCTTGC	60.008	21	201	16	216
59.795	20	CCATCTGTTCACACTCACGG	60.154	20	258	2476	2733
59.989	21	TTCTCAGCAATCATCGCAAC	59.955	20	276	616	891
60.003	20	TCCAAGTTTTGAAGATGACCTC	58.293	22	275	519	793
60.224	20	CATGAGCGAGTAAACCAGCA	60.011	20	222	428	649
60.755	20	GAGTGCGCTCTGGGTAAACT	59.501	20	159	432	590
59.937	20	TGTCCCACATTGGAAAATGA	59.75	20	240	112	351
60.169	20	ACGTGGATGTTATGCACGAA	59.995	20	254	510	763
60.11	20	AGTGCAATCCTTCCAATCCA	60.461	20	185	139	323
60.776	20	TGCATGACTGGATGAAGAGG	59.787	20	240	1153	1392
59.7	20	AACCAGAGACGCAGCATTTT	59.882	20	263	2353	2615
60.028	20	AGGAATGTCCACCGAGAATG	59.927	20	154	102	255
59.933	20	CCAACGGTCCCGTATTATTG	60.067	20	268	221	488
60.894	20	TCTCTTTTTGGGATCAACGG	60.044	20	258	1596	1853
59.141	20	TGGTACCTCCTCCTAATTTCCTC	59.854	23	270	102	371
60.235	20	GCCGGTAATTTCGTGTAGGA	59.96	20	211	473	683
59.42	20	TCATGATTCCAGCTTACCCC	59.894	20	227	1986	2212
60.048	20	CAATGCTCCCTTTTCTCAGC	59.955	20	213	1097	1309
59.596	20	GAGACGCTGATGGGATTGTT	60.081	20	276	693	968
59.166	20	TCCCGATTAAAGTTTCCATGA	59.397	21	230	433	662
60.156	20	TGGTGTGAGAGAGAAAAGGGA	59.829	21	159	1606	1764
60.046	20	GACCTCCGATTTGTCAAGGA	60.05	20	268	1469	1736
58.627	21	GTAGAGAGAAGTGGGCGGTG	59.867	20	146	24	169
59.767	20	TGATGATGGAGCATTTGGTG	60.481	20	109	585	693
60.074	20	AGCTTTGATCTTCGCCATGT	59.843	20	269	610	878
60.002	21	TCGCCTGTATAAATGCCTCG	61.113	20	274	535	808
59.13	24	ACCCCTCTTCCTCTATTGGC	59.536	20	256	445	700
60.644	20	CAGATCAAAGATCCAACGCA	59.799	20	209	17	225
59.946	20	TCCATGTCTTTTTCCCTTCA	58.117	20	187	1895	2081
59.677	22	TGAAGTTAGGAAATAAGGAGGTG	58.743	25	162	562	723
59.969	27	CGCTTATAAGAGCGAGCAAGA	59.903	21	274	226	499

59.505	20	GCCCTCGTGAACATAGGAAG	59.694	20	238	3240	3477
60.465	20	TTCGGTTTTACGGATTGAGC	60.074	20	212	817	1028
59.708	20	CTCATCGGGTTAATTGGGTG	60.184	20	277	884	1160
59.937	20	TCAAACAGAGTGGGGAGGAG	60.229	20	227	288	514
60.293	20	AGCACTGGTTTTGGAAGGTG	60.149	20	272	344	615
60.321	21	GGCTCCCATTGTTTCTCATT	58.984	20	276	1326	1601
59.201	20	CAACACCGAAGAGCTTCACA	60.025	20	223	206	428
59.88	20	TAAAGGGGGACCATTTTTCC	59.992	20	244	271	514
58.679	21	TGGTGAGGATTTAACGGGAA	60.301	20	210	124	333
59.864	20	TCGTGTGATGCCTTGGAATA	60.073	20	185	653	837
60.111	20	ACTTGCTTAGTTCATGGCCG	60.27	20	135	223	357
59.767	20	AAGTGTTGTCGTTTCTTTGGA	57.376	21	266	204	469
60.096	20	TGCCTTGCCTTTTAAGCACT	60.018	20	264	1554	1817
60.173	20	CGCAACTGTTATTCGGGACT	60.132	20	246	202	447
60.034	22	AAGGGGGTTATTAGTGGATCA	57.425	21	236	700	935
60.017	20	CCCACCCATGCCATTATTAG	60.031	20	277	1192	1468
59.943	20	TGTTGGGACAAAATGGTCAA	59.792	20	222	2019	2240
59.843	20	TCTCTGTTTGCGTGCTTGTC	60.183	20	203	279	481
59.551	20	TCGAGGATCAATCTCGGAGT	59.761	20	264	1106	1369
59.986	20	TAAGTTTCTATTCGCCCCCG	61.27	20	197	581	777
59.294	21	CCATGAATTCTTTTGGGGTG	60.162	20	129	732	860
60.836	20	AAATATTGAGGTGGGGGTCC	59.881	20	146	118	263
59.883	20	AGGCTTTGGCTCCTCAATTT	60.208	20	163	530	692
60.23	20	TATTGCCCCTATCTATGCGG	59.907	20	181	563	743
60.495	20	TTAGCATAAATTTAGCCACCG	57.12	21	128	583	710
60.457	23	TAACGATGGACTAGGGGCTG	60.088	20	219	11	229
59.795	20	CACCTCCCTGGGGATTTTAT	60.011	20	194	2492	2685
59.856	20	TCGCCCATGACTACACCATA	59.948	20	230	398	627
59.844	20	TAATGTAATTGGCCCCGAAA	60.144	20	234	270	503
60.011	20	TGGTTGTAATTGCACCAAATTTAC	60.045	24	104	344	447
60.035	20	AGCGTGAGCTCATTTTCGAT	59.985	20	206	2322	2527
59.726	20	ATGTGAATAGCCGTTGAGGC	60.103	20	256	30	285
59.155	27	ATGAGTTATCACCGGATCGC	59.923	20	213	794	1006
58.787	20	CAACGACCTTCATTCGATCA	59.648	20	102	2	103
59.797	20	GATGACGACTCCAACGACAA	59.682	20	203	1266	1468
60.081	20	TGGTGAAAACCCCCAATAGA	60.162	20	242	415	656
59.982	20	ATCGTACGTATCGCCGTCTC	60.125	20	253	477	729
59.997	23	GCACTCCATTCCAAAATTCG	60.448	20	211	150	360
60.299	20	ATCCTGCCAAACCAAGAATG	59.933	20	112	35	146
60.038	20	GTGCACTTGAAATTGCATCG	60.265	20	231	177	407
59.904	20	TCAGAAACAATCTTTGTCTGTTCT(	58.992	25	112	1239	1350
58.746	20	CACCAAGTGGGAAATAGTGCT	59.111	21	145	1727	1871
60.045	20	ACCCTCTCTCCACTCACCCT	60.112	20	154	2369	2522
58.747	20	CTGGCATATTTGTGTTGGCT	58.651	20	233	368	600
60.096	20	GGTAGAAGAATAGGTGCATGAGA	57.533	23	269	781	1049
60.156	20	GGACCGTGATGGACTGACTC	60.537	20	230	2853	3082
59.946	19	CGTATCATACAAGTATGCTGTCCT	59.985	25	206	50	255
60.042	20	CTGAATTGGCTTGTGGGTTT	59.971	20	225	680	904
59.296	20	GTTGAATTTTCACCCCGGTA	59.662	20	262	1519	1780
59.101	21	AAGGAGGTGTTATGAAGGGATT	58.028	22	167	1236	1402
59.945	20	TTGCAACACAAAGCACGAAT	60.302	20	183	252	434
60.11	22	TTTAGTCCATTCTTTCGGCG	60.202	20	192	1994	2185
60.015	20	GAGGCTTATGCATTGACCGT	60.103	20	193	48	240
59.483	23	CATCTTTAATGGTGGCCTGG	60.323	20	221	2165	2385
59.992	20	AAAGATCGGCACAAAAATCG	60.074	20	127	944	1070
59.976	20	TTTGGGGACAACACTGACAA	59.976	20	188	351	538
59.755	20	CAAATCCTGCGACAAGTCCT	60.255	20	232	805	1036
59.813	20	ATGTAATGCCTCGCCCTATG	59.945	20	252	9	260

59.988	20	GAAGAAGGAAAGTTTGGGGG	59.912	20	217	1294	1510
59.971	20	TTCGGTACGAAAAATCGGAG	60.067	20	267	47	313
60.375	20	GGTTCACGATTTGGGAAAAA	59.778	20	258	2487	2744
60.3	20	CACCTCCATCTCCGATCATAA	59.902	21	202	695	896
60.125	20	TGTGAGGAATCCACTCTCCC	60.048	20	270	36	305
59.845	22	CACCAGATATTTGATGCGTGA	59.557	21	154	6	159
59.95	20	ACCGTTAAAGAAACCGGACC	60.221	20	255	1152	1406
60.157	20	TTGATCGCGACATTTGATTT	59.115	20	242	42	283
60.146	20	TGAGGCATCTCTCTCTCACA	57.05	20	244	318	561
59.966	20	TGAGGCAGTGTTGTTCTTCG	60.025	20	245	1321	1565
59.981	20	CTGCCAAGGAACCACAAAAT	59.971	20	229	1265	1493
60.014	20	CGGATTTAAGGGTTATTACCTGAT	58.81	24	255	747	1001
60.224	20	TTGGCCCTACTAACTTGAGCA	59.89	21	216	1489	1704
60.111	20	TGAAGCGTCAAATCACAAGC	59.995	20	229	350	578
58.792	20	CGTTTTGCTTCTCACCCTTC	59.853	20	241	1466	1706
59.43	21	ATTGACAAGTGCAACCCTCC	59.973	20	191	1237	1427
59.512	20	TCGTGTCTAGTGCAGCATGA	59.141	20	112	4751	4862
59.48	20	TTCTCATCCAACGAGCTGAA	59.522	20	248	316	563
59.955	20	GATCCTGTAAGCTCCACCCA	60.073	20	205	228	432
60.681	20	ATATGCACTGGCGGACCTAC	59.985	20	148	267	414
60.152	20	GGATTCCGAGTTTTCGATCC	60.777	20	279	441	719
59.92	20	CTTGTCGGCAGAGGCATTAT	60.235	20	233	811	1043
59.971	20	CGACTTCGGAAGCAAAATTC	59.823	20	133	2516	2648
59.739	20	AAAATTTGGACTCACAACTTCAA	57.841	23	257	63	319
59.648	20	GTGTTGGAAGAGGGGCATTA	59.933	20	274	853	1126
59.864	20	AGCAATCGTGATAGGCAACC	60.103	20	139	2362	2500
60.209	20	ATTTAACAACGGACGAACGC	60.003	20	267	252	518
59.805	20	GATGGGTTATTGATCACGGG	60.014	20	176	3815	3990
59.727	20	TTTGACCTCCTGCAAAATCC	60.051	20	197	167	363
60.214	20	ATCGAATCGGAAAGTTCCCT	59.903	20	259	296	554
60	25	CCAAAAGTTCACATCACTCCAG	59.633	22	190	3	192
59.774	20	AAAAGAACTTTCTCCTCTGGCA	59.521	22	219	200	418
59.463	21	CTGGTCAAATCATCCGAACC	60.317	20	243	4	246
59.992	18	TATCAGGCAAATCCTTGGGA	60.405	20	228	15	242
59.565	20	GGAGGCATTCGATCTTTGTG	60.603	20	178	197	374
59.979	20	GGTTGGAGTGAGCGAAGAAG	59.989	20	226	128	353
60.074	20	TTTTTAGGCTCTCGGAGCTG	59.724	20	241	179	419
59.834	20	CCTTCGTGCAATCATCTTCA	59.799	20	194	4442	4635
59.722	20	CCCAAGAATCAGAGGTCCAA	60.042	20	186	2249	2434
60.14	20	TTGGAAAGGAAAGCTCGAAA	59.931	20	246	1454	1699
60.032	20	GAAGTGAATCAAGTGATGGCTC	58.794	22	168	31	198
60.065	20	TGGAATGTCAAGTCAACACAA	57.556	21	179	891	1069
59.898	22	CTTCGTCGGAGCAGGAATAG	59.971	20	276	218	493
60.031	20	GTCATTGTATGGTGCGTTCG	59.995	20	112	321	432
60.149	19	GTCCCATGTTGCTGCTTTTT	60.118	20	219	46	264
59.882	20	AGGCACTGGCTTGGATTATG	60.096	20	280	146	425
59.415	21	TGATTGAGGCTTGGAACAAA	59.247	20	279	1724	2002
60.043	20	TTCACATTCCTGTCCTGCAA	60.24	20	144	1112	1255
58.411	22	TTGAATGCTCTGAATGCTGC	60.104	20	260	824	1083
60.278	20	TTGAGCATGATTTTTGCTGG	59.809	20	266	493	758
60.187	20	CAACCTCAAAAATGCCCACT	59.971	20	184	796	979
59.727	20	TCGAACCACGTAAATCCTTTG	59.982	21	279	988	1266
58.975	22	CTCTCTAGCACCGACCCAAC	59.867	20	232	15	246
60.022	20	CGTTAGCCTTCTGGATCAGC	59.978	20	213	208	420
59.935	20	TGTGAAATCCGAAAAATGGA	58.94	20	245	224	468
60.12	20	CTTCCCAAAGCCAGAGTGAG	59.982	20	272	1338	1609
60.033	20	AAGGTCTCCTCTCCCACCAT	59.929	20	139	470	608
59.891	20	CACAACAAAAAGCAACCCAA	59.603	20	276	226	501

60.853	20	GCGTGGAGCATTTAGCTTTC	59.988	20	223	0	222
59.84	20	CCATAACGCACCCAAGAACT	59.993	20	188	2129	2316
59.996	20	TCTCGAGAAAACGGGATTTG	60.184	20	226	629	854
60.183	20	AAATGGTGTGATCTTGGACCTT	59.735	22	279	1834	2112
59.833	20	CAACGTAACTGGCAGCAGAA	60.05	20	235	466	700
59.595	21	GAGCCTGACTGGTGTGGTTT	60.159	20	273	401	673
59.365	21	TTTTGTCAGCAGATGTTCGC	59.995	20	274	1922	2195
59.515	22	CTTTTAGCCAGCCCAGTTTG	59.876	20	274	48	321
59.935	20	TGTGGAGCTGAAAACCACAC	59.726	20	269	1051	1319
59.654	23	TGAATGTGCATGGAGGATGT	59.925	20	187	241	427
59.873	20	TTGTTCCTACGCACTCACATT	58.296	21	259	704	962
59.405	20	TGCAAGTGTGCAACAGATCA	60.029	20	271	338	608
59.942	20	GCAAGTAACCTAGTGGCCAAG	58.917	21	174	144	317
59.989	20	CACCTTCGAGATTGCTAGGC	59.978	20	185	1681	1865
60.395	20	CCGTTTACGTTTATCCATACCC	59.52	22	164	362	525
58.919	23	AATTCGTTCGGGGATAGAGC	60.419	20	280	337	616
59.836	20	GAAGAGGAGGCAGTTCATGC	59.957	20	256	185	440
59.94	20	CTGCCTACCATTTCAGCACA	59.864	20	235	409	643
59.425	20	TCATTAAAGCTAAGGCCCCA	59.677	20	252	1571	1822
59.385	22	TTCCGTAGTTGGTTTAGGTTCTTT	59.515	24	244	691	934
59.975	20	ACCATCAGATTCATTGTGCG	59.527	20	271	896	1166
60.03	20	TCACCTTCCAATTCACCCTC	59.903	20	108	568	675
59.891	20	AACAGAAGTGATGGGCTTTCA	59.726	21	230	85	314
60.05	20	CGAACACCATTTTCTTCGGT	59.971	20	175	135	309
59.945	20	GGCATCTCCTCTTGTCTTGG	59.803	20	237	2993	3229
60.656	20	GTCCGCTATAAAGCCCAGC	59.821	19	254	6640	6893
60.033	20	CATTGCAAAATCTCGCTTCA	59.953	20	276	88	363
60.388	20	TTTCGGTTTCTGCAATTTTTG	60.095	21	272	58	329
59.95	20	CCTCAGACAGCTTCAAATCCC	61.138	21	216	728	943
60.378	20	CATCACCGTCATCGTCTCAC	60.121	20	169	656	824
59.671	22	GCTTGTGAAGATGTGCAGGA	59.992	20	276	1	276
59.7	20	CCAGATCATACCTCCCCTCA	59.879	20	229	890	1118
59.694	20	ATCGCCGCCTCTTACTGTTA	59.867	20	191	57	247
59.989	20	TAACACGCAGTCAGTCCGTC	59.905	20	119	280	398
60.037	20	AAGCAGCCATAACTTGCCTC	59.481	20	194	583	776
59.852	20	GGAATTCTATCCGAGCACCC	60.797	20	243	1556	1798
60.022	20	AGACTTGAGAGAGCCGCAAC	59.751	20	191	778	968
59.759	20	GCACCCTCTCTCACTATCGC	59.981	20	234	1423	1656
59.951	20	CAACTTCAGTGCCTCCCATT	60.111	20	243	8	250
60.119	20	GAAGGAGAGTTCCAGATGCG	59.95	20	129	2688	2816
59.315	22	ATGTTATGCCCTAAGGGGGA	60.522	20	206	309	514
59.883	20	CGACTTTTCACTATTCTCCGC	58.986	21	151	19	169
59.911	20	GGGTCCTCCGTCATAAACCT	60.19	20	196	1432	1627
60.161	20	AAATTCCAACGACAATAATTCCA	59.616	23	255	589	843
58.536	25	GAAATAAGGAGGTGTTATGAAGG	59.323	24	152	1635	1786
59.907	20	GAAAAGCTTGGGAATGGTGA	60.051	20	247	1074	1320
57.73	23	CTTGCCACTTTATCCCTCCA	60.066	20	102	18	119
59.883	20	GTTAGATGATGCCACTGCGA	59.83	20	280	2748	3027
59.79	20	GCCTAGATAGGGAGCAGCAA	59.574	20	131	522	652
60.043	20	CTTCCCCTGCAATCAAAGAA	60.184	20	189	3625	3813
59.988	20	TTGCTGAAAAGCAAGCCTCT	60.27	20	222	564	785
60.16	20	CCCTCCTATGCACTCCAAAA	60.066	20	238	3637	3874
59.609	20	TGAGTGCGCTATGCCTAACA	60.566	20	279	172	450
58.296	21	GATACTCGCCATTTTCCCCT	60.286	20	268	496	763
60.025	20	GAGTCTGCAAAAGCAAAGCC	60.14	20	147	1659	1805
59.746	20	AACACACGTCGGTTCATTCA	60.008	20	226	3546	3771
59.993	20	TGTGGCATGTTCTTCGAATC	59.654	20	241	747	987
60.014	19	TCAGATGAAGCCAAAGAGGG	60.331	20	276	2066	2341

59.934	20	TAGAGTAGCGGCTGCAGGTT	60.176	20	178	13	190
59.763	20	CGCTCCCATTTTTGGTAGAA	60.067	20	124	587	710
59.263	20	CCAAACACCCCCTTAGTGTT	58.812	20	249	865	1113
59.828	21	GGGATGTTTGGAGAGACGAA	60.05	20	138	39	176
59.652	20	ATCAGCAGCAACCAAGTTGA	59.445	20	199	540	738
59.369	20	CACGACGAACTTTTCACTTCA	58.96	21	214	559	772
59.381	20	CCCACAGATCTTGGACTGGT	59.962	20	110	1408	1517
59.837	26	GGCTGGATCCAATGGTCTTA	59.894	20	256	453	708
60.221	20	GCTGACAACATGGTCGCTTA	59.871	20	153	813	965
60.141	21	AAGTTTGGGGGAGGAGAAAA	59.912	20	256	381	636
59.903	20	TGGTTGGAATTTTCTCCAGG	59.903	20	278	1174	1451
60.142	20	CCTCCTGAACCAAATCCAAA	59.903	20	146	565	710
59.966	20	AATTGTACATCAAAGGGGCG	59.823	20	173	947	1119
59.522	20	CAACGGTTGCAATTTGATTC	59.016	20	226	508	733
59.836	20	ACTCCATCTCTCTCCCGCTT	60.358	20	239	565	803
59.28	24	CAGGTGGAAGGAAGAGGAAAG	60.225	21	221	732	952
59.31	21	GAATCTTCACCAGTGCCTGA	58.802	20	280	6061	6340
61.339	20	CAATGGTGAATATTATAAAGGCA/	59.607	26	216	192	407
59.411	24	TGCACTTGTGCCCCTAAAAT	60.502	20	270	1771	2040
59.992	20	AATTTCCCCGCGTTATTTCT	59.808	20	175	770	944
57.248	22	TTGTAAAAGGATTCCACGTATTTG	59.351	24	260	352	611
59.922	20	GACCAGCCTGACCTCAAATC	59.661	20	224	1059	1282
60.193	20	AGAGCTCAGGATCTTGCGAA	60.24	20	151	3401	3551
[truncated: 521,440 more chars]
